# A robust and tunable halogen bond organocatalyzed 2-deoxyglycosylation involving quantum tunneling

**DOI:** 10.1038/s41467-020-18595-2

**Published:** 2020-09-30

**Authors:** Chunfa Xu, V. U. Bhaskara Rao, Julia Weigen, Charles C. J. Loh

**Affiliations:** 1 Abteilung Chemische Biologie, Max Planck Institut für Molekulare Physiologie, Otto-Hahn-Straße 11, 44227 Dortmund, Germany; 2 Fakültät für Chemie und Chemische Biologie, Technische Universität Dortmund, Otto-Hahn-Straße 4a, 44227 Dortmund, Germany

**Keywords:** Organocatalysis, Reaction mechanisms

## Abstract

The development of noncovalent halogen bonding (XB) catalysis is rapidly gaining traction, as isolated reports documented better performance than the well-established hydrogen bonding thiourea catalysis. However, convincing cases allowing XB activation to be competitive in challenging bond formations are lacking. Herein, we report a robust XB catalyzed 2-deoxyglycosylation, featuring a biomimetic reaction network indicative of dynamic XB activation. Benchmarking studies uncovered an improved substrate tolerance compared to thiourea-catalyzed protocols. Kinetic investigations reveal an autoinductive sigmoidal kinetic profile, supporting an in situ amplification of a XB dependent active catalytic species. Kinetic isotopic effect measurements further support quantum tunneling in the rate determining step. Furthermore, we demonstrate XB catalysis tunability via a halogen swapping strategy, facilitating 2-deoxyribosylations of D-ribals. This protocol showcases the clear emergence of XB catalysis as a versatile activation mode in noncovalent organocatalysis, and as an important addition to the catalytic toolbox of chemical glycosylations.

## Abstract (web summary)

Halogen bonding (HB) catalysis is rapidly gaining momentum, however, cases of XB activation for challenging bonds formation are rare. Here, the authors show a robust XB catalyzed 2-deoxyglycosylation with broad scope and featuring a quantum tunneling phenomenon in the proton transfer rate determining step.

## Introduction

Noncovalent catalysis constitutes one of the major pillars of organocatalysis^1–4^, by capitalizing intermolecular interactions, such as hydrogen bonds (HB) to activate specific functional groups under mild conditions. Indisputably, HB catalysis^5,6^ dominates the majority of noncovalent organocatalyzed protocols, which is largely spearheaded by thiourea catalysis^7–14^. Furthermore, thiourea organocatalysis is widely dubbed as biomimetic^2^, as it derives inspiration from how enzymes capitalize noncovalent interactions to catalyze biochemical reactions^1–4^. Hence, biomimetic thiourea catalysis is well recognized as a powerful synthetic tool spanning from asymmetric catalysis to natural product synthesis^5–14^.

In contrast, while the utility of the more directional halogen bond (XB) is increasingly establishing its prominence in noncovalent organocatalysis^15–21^, mechanistic understanding and utility of unique XB activation modes is highly limited. While nature also exploits XBs in catalytic processes^22^, for instance in thyroid hormones, the development of biomimetic XB catalytic strategies in constructing biologically important molecules is surprisingly underexplored in the literature.

In recent years, despite the development of excellent proof-of-concept XB-catalyzed reactions by multiple research groups^23–42^, protocols whereby XB catalysis shows clear catalytic advantages in over conventional thiourea catalysis is lacking^21^. Moreover, a conceivable configurational advantage pertaining to XB catalysis would involve the ease of σ-hole tunability via halogen swapping to accommodate challenging substrates—an advantage not well adoptable in thiourea catalysis due to strict requirements of hydrogen atoms for dual HB activation. This property is however not yet successfully exploited. In addition, the majority of currently reported XB catalytic strategies are limited to monofunctional group activation, such as solely on carbonyls, imines, quinolines, iodonium ylides, or halides^15–21^. Biomimetic complex kinetic behavior involving in situ dynamic XB activation on multiple reaction species offers great promise in unraveling unknown XB mechanisms and reactivity, but little is currently known.

**Fig. 1:**
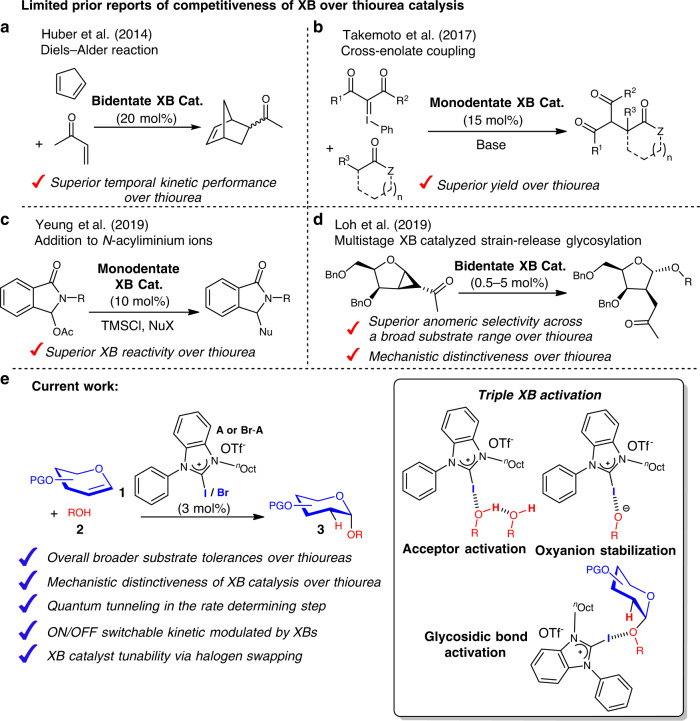
Prior reports in enhanced XB over thiourea based HB catalysis. **a** XB-catalyzed Diels–Alder reaction. **b** XB-catalyzed cross-enolate coupling reaction. **c** XB-catalyzed Friedel–Crafts-type bis-indolylation. **d** XB-catalyzed strain-release glycosylation. **e** Current work.

In an elegant seminal benchmarking case, Huber et al. demonstrated a XB-catalyzed Diels–Alder reactions between cyclopentadiene and methyl vinyl ketone (Fig. 1a) through XB-ketone activation^27^. A comparison of the kinetic profiles between XB and thiourea catalysts illuminated superior XB catalytic performance. An extension of the same activation concept was exemplified by Toy et al., wherein they demonstrated substantially improved effectiveness of a bidentate XB catalyst over thiourea catalysis in a bis-Friedel–Crafts-type reaction of indoles on ketones^41^.

Furthermore, Takemoto and coworkers showcased in 2017 a powerful cross-enolate coupling reaction catalyzed by a monodentate XB catalyst, which furnished superior yield over thiourea catalysis (Fig. 1b)^32^. Recent DFT calculations by Wong et al. offer theoretical basis into the potential competitiveness of XB catalysis with respect to thiourea catalysis^43^. Lately, Yeung and coworkers also reported that XB catalysis enables addition of silylated *C*-nucleophiles to *N*-acyliminium ions (Fig. 1c), which was unachievable using thiourea catalysis^35^. Our group also demonstrated in 2019 that a multistage XB catalysis gave broadly superior anomeric selectivity in strain-release glycosylations^44^, (Fig. 1d) superceding that of our earlier reported thiourea-catalyzed protocol^45^. These very limited prior examples provide pressing impetus for more experimental proof and deeper mechanistic understanding of XB catalysis, as a high-performance noncovalent catalytic tool in challenging reactions.

Specifically, we aim to harness and unravel noncovalent mechanisms unique to XB organocatalysis, so that enabling methodologies, which facilitates the preferential construction of challenging bonds over thiourea catalysis in biologically relevant molecules can be developed. In line with this aim, we have identified the biologically relevant 2-deoxyglycosylation as an ideal reaction model for the discovery for unknown XB catalytic mechanisms^46–59^. The unique poly-oxygenated nature of glycosyl substrates and products provides numerous XB acceptor moieties for the in situ catalytic establishment of dynamic noncovalent activations. Moreover, the synthetic importance of 2-deoxyglycosides as a privileged and biologically useful compound class is well exemplified by the continual intense interest by many different research groups^46–59^, due to its prevalence in glycosidic natural products, such as digitoxin and saccharomicin B (ref. ^46^).

On the organocatalytic front^60–64^, seminal studies by Galan and McGarrigle et al. demonstrated the utility of thiourea catalysis in accessing 2-deoxyglycosides via mild organocatalytic conditions^47,56^. However, significant limitations still exist in such protocols. For instance, the sole application of thiourea catalysis limits the donor scope to galactals^47^, and expansion to challenging glycosyl donors, such as glucals and rhamnals required harsher conditions, i.e., the usage of either a conventional strong Brønsted acid^48^, or a judiciously matched combination of thiourea and an appropriate enantiomer of a strong chiral Brønsted acid^49^. A caveat of directly employing strong Brønsted acid activators lies in the product decomposition due to greatly enhanced hydrolytic cleavage, of up to 2000-fold elevated acid labilities of 2-deoxyglycosides (Supplementary Note 14)^65^, which necessitates rigorous water exclusion procedures, such as extended pre-vacuum suctioning of substrates^47–49,56^. As the critical mechanistic influence of activators and catalysts in glycosylation pathways is well recognized but largely understudied^66^, the recent realization of unconventional XB catalytic mechanisms offers huge potential to expand the glycosylation activator toolbox^15–21^ and to accommodate improved substrate scopes, while retaining mild conditions required for broad substrate utility and circumventing decomposition.

Furthermore, the applicability of XB activation in advancing chemical glycosylations is still in its infancy. Apart from our above mentioned XB-catalyzed strain-release glycosylation^44^, Huber and Codée et al. demonstrated a seminal stoichiometric proof-of-principle XB activation in Koenigs–Knorr glycosylation^67^, and recently Takemoto et al. described a powerful XB-co-catalytic role to elevate the Brønsted acidity in thiourea for *N*-glycofunctionalizations and *N*-glycosylations^68,69^.

We herein report a remarkable XB-catalyzed 2-deoxyglycosylation of glycals **1** (Fig. 1e), featuring robustness, mildness, substrate broadness, and mechanistic complexity via a switchable intricate reaction network. Noncovalent catalytic benchmarking studies are conducted between our protocol and widely established thiourea-catalyzed protocols^47,56^, and our protocol furnishes overall superior donor and acceptor substrate generality, noteworthily in silylated galactals, glucals, rhamnals, and pentose-derived donors. We introduce a halogen swapping strategy for challenging D-ribal substrates, and this was found to be effective in tolerating challenging 2-deoxypyranoribosylations. In situ NMR monitoring reveal a sigmoidal kinetic profile characteristic of autoinduction, suggesting an amplificative formation of an in situ catalyst. Kinetic isotopic effect (KIE) experiments suggest that quantum tunneling is operative in the proton transfer rate-determining step of the mechanism.

## Results

### Establishment of an XB-catalyzed 2-deoxyglycosylation

**Fig. 2:**
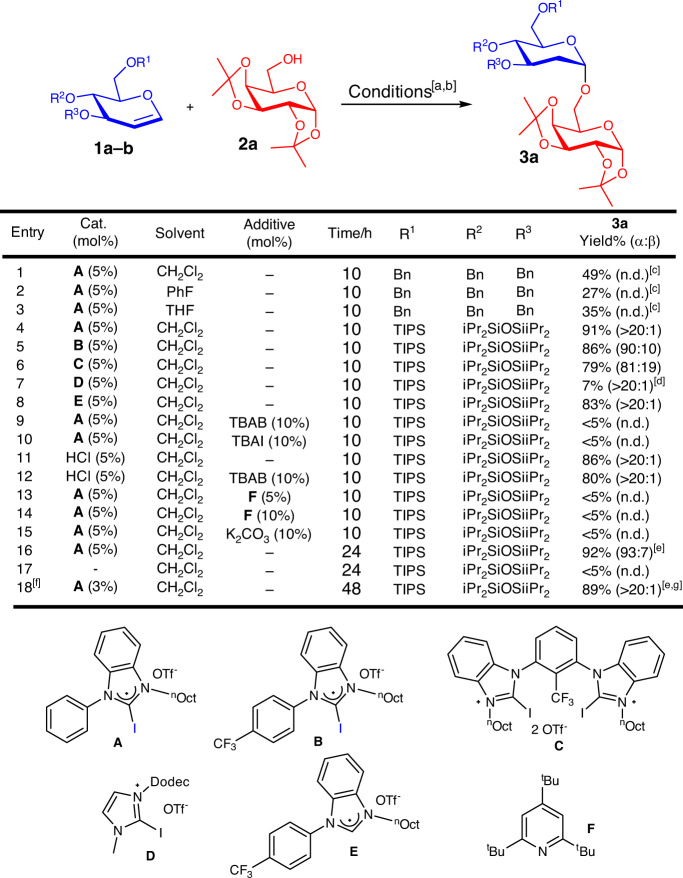
Optimization of XB-catalyzed 2-deoxyglycosylation. [a] **2a** (0.1 mmol), **1a–b** (0.15 mmol), catalyst, 50 °C, solvent (0.2 M), time, argon. [b] Yield and α:β ratio were determined by crude ^1^H NMR spectra analysis using 1,3,5-trimethoxybenzene as an internal standard. [c] 40–50% NMR yields of Ferrier rearrangement side-products detected. [d] 92% **1b** remaining. [e] **2a** (0.2 mmol), **1b** (0.30 mmol), catalyst, 50 °C, CH_2_Cl_2_ (0.2 M), time, argon. [f] Conducted at 40 °C. [g] Isolated yields. n.d. not determined, iPr isopropyl, TBAB tetrabutylammonium bromide, TBAI tetrabutylammonium iodide.

We initiated our investigation on a model glycosylation by selecting the D-glucal substrates **1a–1b** as our glycosyl donor, and **2a** as the acceptor. Utilizing the benzylated D-glucal **1a** (Fig. 2, entry 1–3) was deleterious in our reaction because of substantial formation of Ferrier side products. Encouragingly, when silylated D-glucal **1b** (Fig. 2, entry 4) was employed^48^, we observed a yield elevation of **3a** to 92% with excellent anomeric selectivity. A screen of various XB catalysts **A–D** revealed that **A** is optimal for our reaction^32^. We eventually arrived at our optimized protocol (Fig. 2, entry 18), whereby only 3 mol% of catalyst **A** is required for the glycosylation, furnishing **3a** with 89% yield and excellent anomeric selectivity (>20:1).

### Control experiments for confirming XB catalysis

To better understand the possible interference effects due to trace acidic HI generation, a series of control experiments were conducted^21^. While the non-iodinated control catalyst **E** gave good yields of **3a** (Fig. 2, entry 8), the benzimidazolium hydrogen is known to be acidic and could function as either a nonclassical HB or a Brønsted acid catalyst (Supplementary Note 15)^70,71^, and does not constitute exclusionary proof of XB catalysis by **A**. To better investigate the criticality of XB catalyst influences on our system, TBAB and TBAI are added into the reaction to inhibit the XB activation mechanism by competition for the σ-hole site, due to the extremely high affinity of halide ions to the XB catalyst (Fig. 2, entries 9 and 10)^25,29^. Both halide competition experiments gave negligible yield (<5%) of **3a**, further augmenting the presence of XB catalysis.

As parallel controls to ascertain TBAB poisoning effects on conventional Brønsted acids without XB influence, the glycosylation was carried out twice in catalytic amounts of HCl (5 mol%), a surrogate for HI, in the absence and presence of the competition reagent TBAB (Fig. 2, entries 11 and 12). The similarly good yields of **3a** in both control experiments (80–86% NMR yield) signify that TBAB competition has no effect on fortuitous acid catalysis, further solidifying the cruciality of XB catalysis in our protocol. In control experiments simply reacting **A** with isopropanol, the absence of *O*-acceptor substituted benzimidazolium side products in LC–MS and ^1^H NMR analysis of the reaction mixture further supports the absence of trace acid catalysis (Supplementary Figs. S5–S9 and Supplementary Note 16).

Furthermore, the addition of catalytic amounts of organic base **F** (Fig. 2, entries 13 and 14), or inorganic base K_2_CO_3_ (Fig. 2, entry 15) terminated the glycosylation (<5% yield). This observation is in contrast with our previously reported XB-catalyzed strain-release glycosylation^44^, whereby the reaction still proceeded with relatively good yields in the presence of base. A control experiment in the absence of catalyst **A** did not result in reaction (Fig. 2, entry 17). Evaluation of these control experiments revealed a strong dependency on XB catalysis in this protocol. The presence of base deactivation does not necessarily confirm the presence of trace acidity, if XB activation is crucial in initiating a proton transfer step, and the base serves as quencher of the proton transfer. These observations point us toward a possible complex interplay of mechanisms through dynamic XB interactions in our methodology.

### Substrate scope

**Fig. 3:**
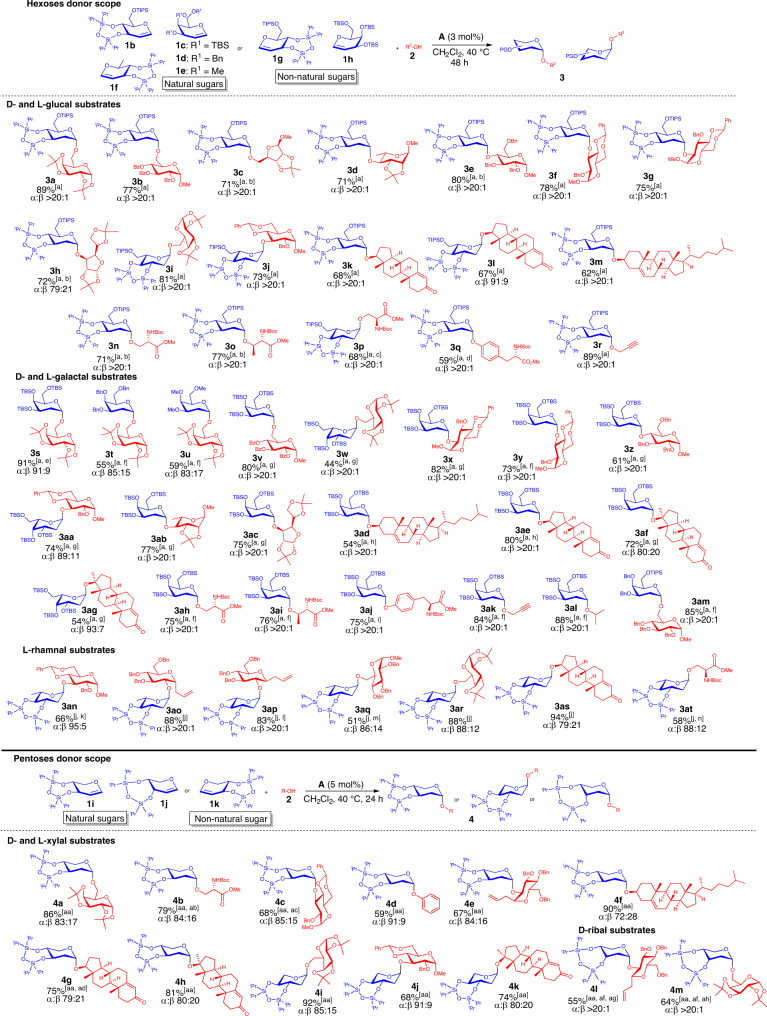
Substrate scope of the XB-catalyzed glycosylation. Hexoses donor scope: [a] **2** (0.2 mmol), **1b–1h** (0.3 mmol) and 3 mol% catalyst **A**, in CH_2_Cl_2_ (1 mL), argon, 40 °C, 48 h; α:β ratio was determined by crude ^1^H NMR analysis. [b] 5 mol% catalyst **A**, 50 °C. [c] 5 mol% catalyst **A**, 50 °C, 72 h. [d] 5 mol% catalyst **A**, 50 °C, 108 h. [e] RT, 24 h. [f] 30 °C. [g] 30 °C, 72 h. [h] 30 °C, 24 h. [i] 10 mol% catalyst **A**, 40 °C, 96 h. [j] **2** (0.2 mmol), **1** **f** (0.4 mmol) and 5 mol% catalyst **A**, in CH_2_Cl_2_ (1 mL), argon, 30 °C, 48 h. [k] 108 h. [l] 40 °C. [m] 96 h. [n] 50 °C, 72 h. Pentoses donor scope: [aa] **2** (0.2 mmol), **1i**–**k** (0.3 mmol) and 5 mol% catalyst **A**, in CH_2_Cl_2_ (1 mL), argon, 40 °C, 24 h; α:β ratio was determined by crude ^1^H NMR analysis. [ab] 96 h. [ac] 30 °C, 48 h. [ad] 48 h. [ae] 3 mol% catalyst **A**, 30 °C, 48 h. [af] **1j** (0.4 mmol). [ag] 3 mol% catalyst **A**, 35 °C, 48 h. [ah] 3 mol% catalyst **A**, 30 °C. RT = room temperature 23 °C, PG = Protecting Group.

With an optimized protocol in hand, we proceeded to establish the substrate scope of the protocol. We determined that this protocol tolerates a wide range of hexose-based donors **1b–1h**, furnishing glycosides **3a–at** with good to excellent yields and generally excellent anomeric selectivity (Fig. 3). For the hexoses-based donors **1**, natural and nonnatural D- and L-glucal substrates, D- and L-galactal, and L-rhamnal-based substrates are very well tolerated in this methodology. Generally, a range of different *O*-acceptors bearing the free hydroxyl moiety at multiple positions are very well tolerated to yield the target glycosides **3** with excellent anomeric selectivity. This include the protected monosaccharides acceptors to generate **3a–3j**, **3s–3ac**, and **3an–3ar**. Steroidal acceptors such as cholesterol, testosterone, and methyltestosterone are also well accommodated within the different hexoses donors (**3k–3m**, **3ad–3ag,** and **3as**). Interestingly, protected amino acid residues, including L-serine, L-threonine, and L-tyrosine work well in our protocol to generate glycopeptide-type derivatives (**3n–3q**, **3ah–3aj**, and **3at**). Simple primary and secondary alcohols, such as propargyl alcohol and isopropanol also gave the target glycosides with generally good to excellent yields, and very good to excellent selectivity (**3r** and **3ak–3al**).

Furthermore, we sought to investigate whether our protocol is robust enough to accommodate less facile pentoses-based donors **1i–1k**, which are challenging in thiourea catalysis^47,56^. Gratifyingly, natural (**1i**, **1j**) and nonnatural pentose-based donors (**1k**) work generally well in our XB catalysis protocol, to generate the target glycosides (**4a–4m**) with good to excellent yields and good anomeric selectivity. In the case of the D-xylal substrate **1i** (Fig. 3), while there is a clear anomeric preference toward the α-anomer, a diminishment of anomeric selectivity was observed compared to the hexoses, probably due to absence of steric hindrance on the β-face from the C5 substituent. The nonnatural L-xylal was tolerated in the XB catalytic protocol (**4i–4k**), furnishing anomeric selectivity in the similar range as the natural D-congeners.

We also investigated the use of D-ribal **1j** to access the 2-deoxyribose scaffold in **4l–4m**. Glycosidic linkages to 2-deoxypyranoribose can be successfully constructed using glycosyl acceptors bearing free primary or secondary alcohol groups, with generally moderate to good yields and excellent anomeric selectivity (Fig. 3).

The overall substrate scope indicated broader donor and acceptor tolerance over established thiourea catalysis, particularly in accessing 2-deoxyglycosides from challenging glucals, rhamnals, and pentose-derived donors. It must be mentioned, however, that thiourea catalysis offers better tolerance on D-galactal donors with ether protecting groups, while XB catalysis is superior when challenging secondary and phenolic acceptors are used on silylated D-galactal donors. Data on benchmarking studies comparing these substrates and protecting group influences against thiourea catalysis will be explored in a later section.

**Fig. 4:**
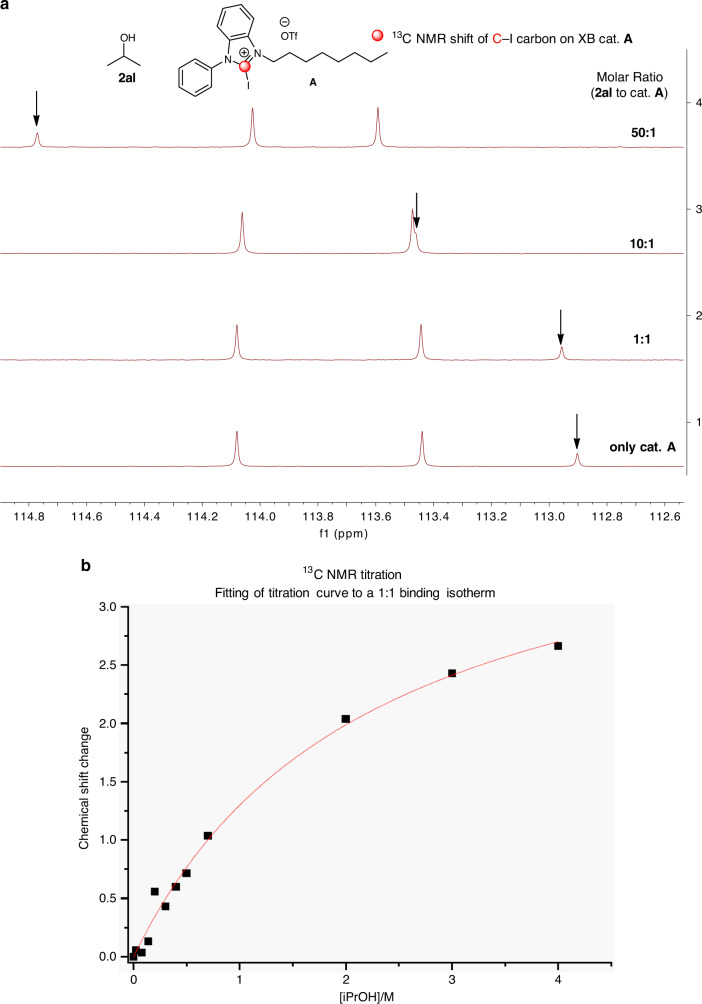
NMR titrations and isothermal titration calorimetry to ascertain XB activation. **a** ^13^C NMR titration (^13^C resonance of C–I bond denoted in arrows). **b** Fit of the ^13^C NMR titration to a 1:1 binding isotherm.

To further ascertain the presence of XB activation on a model glycosyl acceptor **2al**, a ^13^C NMR titration with the addition of increasing quantities of isopropanol **2al** to **A** was conducted (Fig. 4a). A downfield shift of the C–I carbon resonance in **A** of ~1.9 p.p.m. was observed. The direction of the NMR shift is in accordance with known literature reports of catalytic XB activation^27,29,44,68^. Fitting of the host–guest titration to a 1:1 isotherm curve yielded an excellent fit with a *K*_a_ value of 0.45 M^−1^ (Fig. 4b, Supplementary Fig. S2 and Supplementary Table S2). The TBAB and TBAI competition experiments were corroborated by ^13^C NMR resonance shifts of the C–I carbon of catalyst **A** when an equimolar amount of either TBAB or TBAI was added (Supplementary Figs. S24 and S30)^25^. In the case of a 1:1 molar ratio of **A**:TBAB, a downfield shift of 15.45 p.p.m. of the ^13^C resonance was observed. When a solution of a 1:1 molar ratio of **A**:TBAI was measured, an analogous downfield ^13^C resonance shift of 14.34 p.p.m. was observed. The distinctively larger downfield shifts arising from interaction with TBAB and TBAI, compared to the 1:1 adduct formed between catalyst **A** and isopropanol (Fig. 4a and Supplementary Table S2) further support that halides, such as bromide and iodide bind much tighter to catalyst **A** than isopropanol^25^. Isothermal titration calorimetry (ITC) experiments between TBAB or TBAI with **A** yielded *K*_a_ values of 7050 and 2210 M^−1^, respectively (Supplementary Figs. S3 and S4), confirming that halide ion binding to **A** is of significant orders of magnitude higher than an isopropanol to **A** interaction, reaffirming the value of halide competition experiments (Fig. 2) in ascertaining XB influences by poisoning the catalyst. A ^13^C NMR experiment obtained by mixing a 1:1 molar ratio of donor **1c** with catalyst **A** gave an almost negligible ^13^C NMR shift of the C–I resonance (Supplementary Fig. S36). This supports the hypothesis that noncovalent interactions between the glycosyl donor and **A** are insignificant in the reaction mechanism^38^.

### Mechanistic investigation by deuterated experiments

**Fig. 5:**
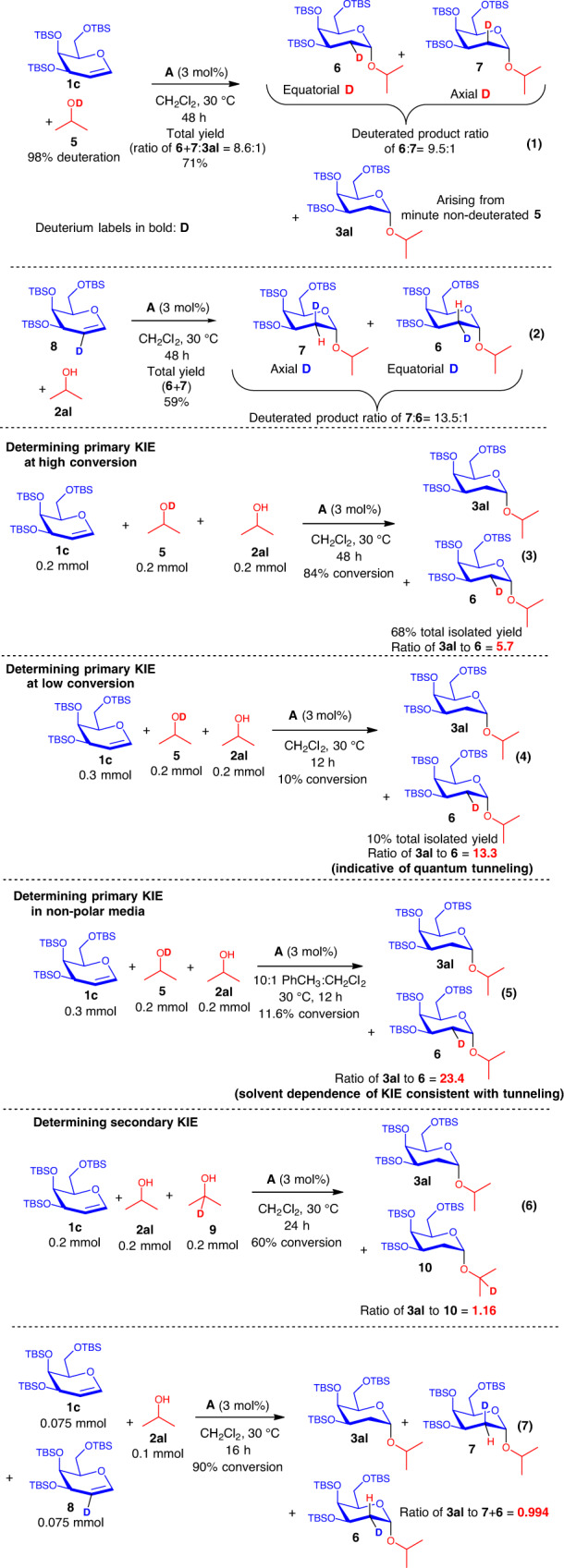
Deuterium-labeling studies. Deuterated experiments confirming step-wise mechanism through deuterium scrambling and ascertaining source of C2 proton originates from the glycosyl acceptor. Intermolecular competition experiments to understand primary and secondary kinetic isotopic effects (KIEs), and KIE elevation to support quantum tunneling.

For a deeper insight into the mechanism, deuterated experiments (Fig. 5 and Supplementary Fig. S44) were conducted. In a first control experiment (Fig. 5, Equation 1), the deuterated 2-propanol-OD **5** was instead used as the acceptor in the XB-catalyzed glycosylation. We observed the formation of both the *cis*-addition product **6** and the *trans*-addition product **7** in a ratio of 9.5:1 (**6**:**7**).

In a second confirmatory experiment, a galactal donor **8** with a deuterium label on C2 was synthesized and subjected to the standard XB catalytic conditions with isopropanol (Fig. 5, Equation 2). In this case, we detected the formation of both the *cis*-addition product **7** and the *trans*-addition product **6**, in a product ratio of 13.5:1 (**7**:**6**). The scrambling of the deuterium labels on C2 in both experiments and the appearance of *cis*- and *trans*-addition products, suggests that the reaction proceeds through a step-wise reaction, indicating that protonation of the C2 of the glycal from the acceptor –OH proton constitute the first step of the mechanism.

Intermolecular competition experiments using equimolar amounts of isopropanol and deuterated 2-propanol-OD (Fig. 5, Equations 3–4, and Supplementary Figs. S39 and S40) were conducted to determine the presence of primary KIEs. We first conducted two experiments, each terminated at high and low conversions, respectively. In the former, a KIE value of 5.7 is obtained and in the latter, a KIE value of 13.3 is obtained. We postulate that this difference in primary KIE attained under different conversions might be attributed to a mechanistic shift in the overall mechanism as time proceeded, i.e., the initial significance of tunneling in the proton transfer rate-determining step is less pronounced, when a more predominant dynamic acceptor exchange cycle sets in the later part of the reaction (see mechanistic explanation in the later section of the manuscript), which can involve further proton transfer elementary steps. This relatively larger KIE values beyond the semiclassical limit (>9) supports the involvement of quantum tunneling in the rate-determining step^72–78^, where the conversion from reactants to products tunnels directly through a kinetic barrier without traversing an energetic maxima.

While such quantum tunneling phenomena were previously known in proton transfer reactions^72–78^, as well as in enzymatic catalysis^77,79,80^, this is hitherto not well understood in the context of noncovalent organocatalysis. A further primary KIE value was determined using a nonpolar solvent mixture of 10:1 PhCH_3_:CH_2_Cl_2_, and a substantial elevation of primary KIE to 23.4 was observed (Fig. 5, Equation 5 and Supplementary Fig. S43). This solvent dependence is in line with previously reported proton transfer reactions involving quantum tunneling^81,82^, as polar solvents increase the effective mass of the proton via coupling of solvent dipoles, which reduces tunneling and hence decreases the measured KIE.

Furthermore, competition experiments to determine secondary KIE were carried out (Fig. 5, Equations 6–7, and Supplementary Figs. S45 and S46). When equimolar amounts of isopropanol and **9** were used, a secondary KIE value of 1.16 was obtained. In addition, when galactals **1c** and **8** were employed in an analogous experiment, a secondary KIE of 0.994 was obtained. The large primary KIEs and the close to unity values attained for secondary KIE experiments strongly support the possibility of OH bond breaking as the rate-determining step.

### Mechanistic experiments via in situ NMR spectroscopy

**Fig. 6:**
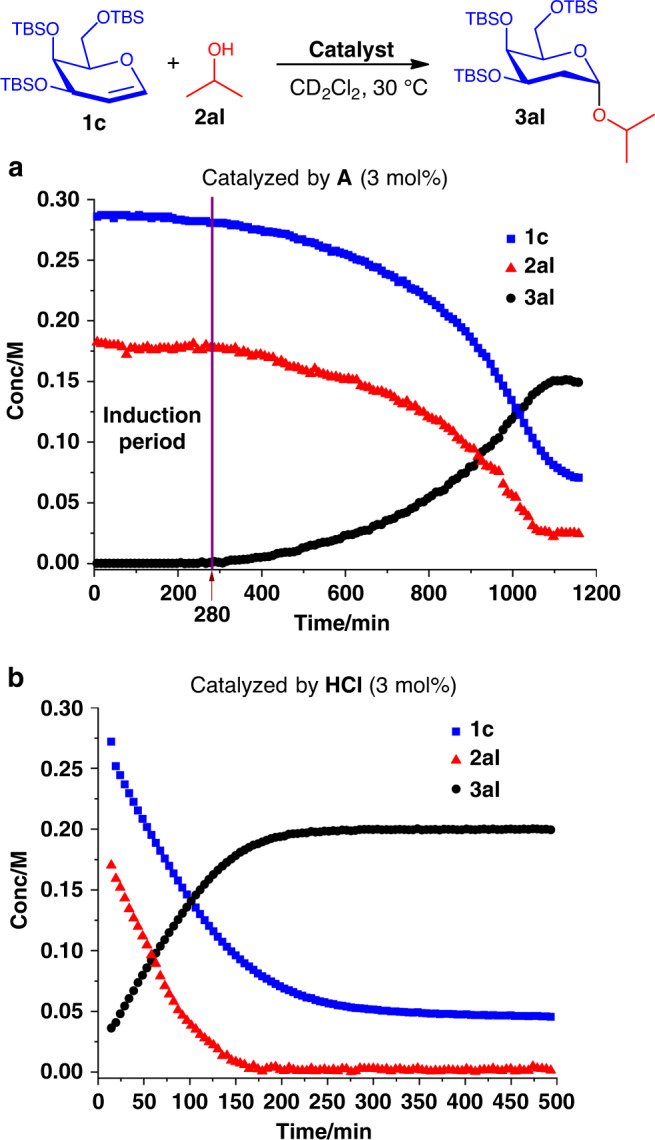
XB vs strong acid kinetic profile comparison. Comparison of **a** sigmoidal kinetics under standard conditions with **b** saturation kinetics with a strong Brønsted acid.

We then undertook an in situ NMR monitoring experiment (Fig. 6) under standard conditions. Surprisingly, the experiment displayed a counter-intuitive sigmoidal kinetic profile^83–85^, differing from saturation kinetics observed our previously reported XB-catalyzed strain-release glycosylation^44^. Superimposition of the product formation profile of **3al** with the depletion profiles of both reactants (Fig. 6a), isopropanol **2al** (limiting reagent) and **1c** uncovered the symmetrical nature of the product vs substrates profiles. Previous literature precedence corroborate that such a symmetrical profile is indicative of a slow in situ generation of a critical active catalytic intermediate^83^. Spurred by this finding, we wanted to delineate the mechanistic differences between an XB-catalyzed case with a true strong Brønsted acid catalyst.

**Fig. 7:**
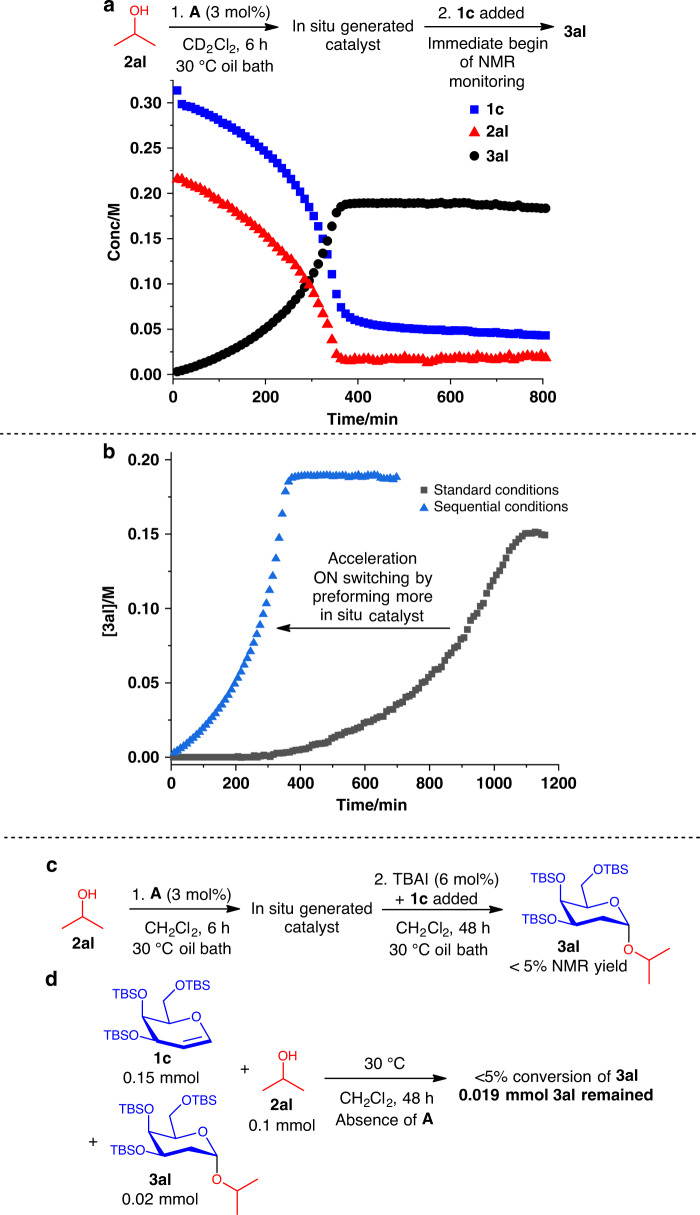
Sequential and control experiments. **a** Sequential kinetic experiment unraveled enhanced acceleration of product formation. **b** Comparison of **3al** formation kinetics of standard vs sequential conditions. **c** Poisoning using TBAI to ascertain XB influence on the in situ catalyst. **d** Control experiment to support the absence of autocatalysis.

Comparing the profile of catalyst **A** with 3 mol% HCl (Fig. 6b) showed clear divergences. True Brønsted acid catalysis adheres to saturation kinetics, and no induction time was observed. This further indicate that in our XB catalytic method, a mechanistically different route from a true Brønsted acid catalyst is operative. To ascertain the postulate of in situ generation of an active catalytic intermediate during the induction period, we conducted a sequential control experiment. **2al** is first reacted with catalyst **A** at 30 °C for 6 h in the absence of **1c** (Fig. 7a), to first generate more of the in situ catalyst in a time period analogous to the induction period. Subsequently, **1c** was added with the immediate commencement of NMR monitoring. We observed then the disappearance of the induction period and a significantly accelerated reaction time of ~400 min.

The acceleration arising by conducting the same experiment in sequential order supports the generation of an in situ generated active catalyst from **2al** and **A** (Fig. 7b depicts accelerated kinetic trace with respect to standard conditions)^83^. The observation of a concave shaped profile indicates continual amplification (active catalyst increasing at an increasing rate) of the in situ catalyst, as the catalytic cycle proceeds upon **1c** addition^86^. In addition, the persistently accelerating kinetic behavior also corroborates that the amount of in situ active catalyst present during the entire experiment is significantly lower than the 3 mol% of **A** added at the onset. This acceleration is analogous to an ON switching of the catalytic network.

In an orthogonal competition experiment to test if this in situ catalytic species involves XB activation, **2al** is first reacted with catalyst **A** at 30 °C for 6 h (Fig. 7c), followed by the sequential addition of TBAI as an in situ XB catalyst poison and **1c** resulted in negligible formation of **3al**. This is consistent with the hypothesis that the in situ active catalyst depends crucially on XB participation for catalytic activity.

**Fig. 8:**
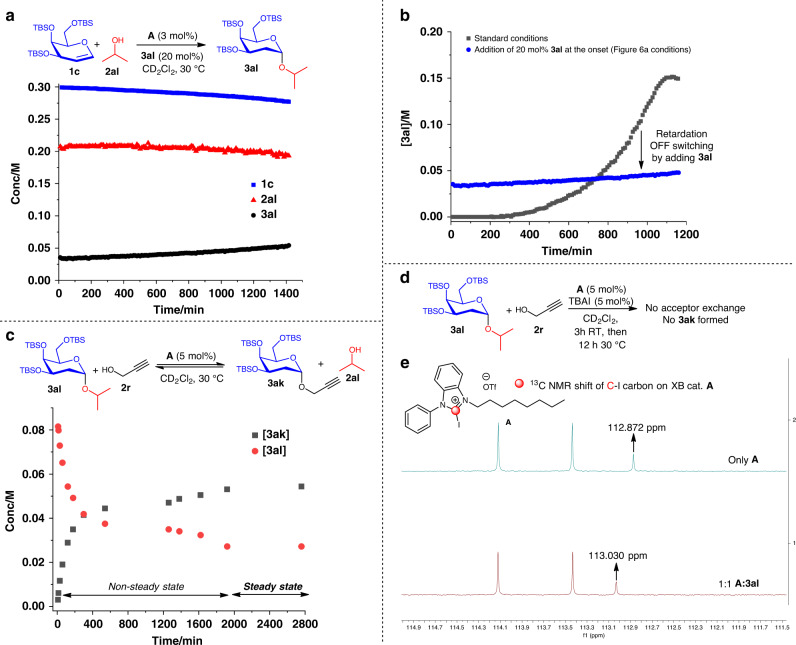
Dynamic acceptor exchange. **a** Addition of 20 mol% product at the onset retarded XB catalysis. **b** Comparison of **3al** formation kinetics of standard vs conditions in **a**. **c** Acceptor exchange experiment to ascertain dynamic acceptor exchange. **d** Competition experiment with TBAI showed XB catalyst poisoning halted the dynamic acceptor exchange. **e** 1:1 adduct of catalyst **A** to **3al** revealed a downfield ^13^C NMR shift.

To investigate if our sigmoidal kinetic at standard condition is representative of an autoinductive or an autocatalytic profile^86–88^, we conducted a control reaction between **1c**, **2al**, and product **3al** as a putative catalyst in the absence of **A**. This experiment resulted in negligible conversion (<5%, Fig. 7d), and further supports the hypothesis of an autoinductive mechanism over autocatalysis^83,86^. Unexpectedly, an in situ NMR monitoring experiment adding 20 mol% of product **3al** to the standard XB catalytic conditions revealed retardation of catalysis (Fig. 8a). While this observation supports the absence of autocatalytic behavior, it points toward a possible catalyst deactivation by **3al**. It is essential to note that addition of **3al** at the onset of the reaction enables the direct deactivation of **A** before the formation of any in situ catalyst in the reaction mixture, hence hampering reaction progression. This retardation effect is analogous to an OFF switching of the catalytic network (Fig. 8b, compared with standard conditions). For a confirmatory test to ascertain XB catalytic influences between **3al** and **A**, an acceptor exchange experiment mixing propargyl alcohol **2r** with **3al** in the presence of **A** was conducted (Fig. 8c, Supplementary Figs. S49 and 50, and Supplementary Table S10). The temporal **3ak** formation and **3al** depletion profiles indicated that a XB-catalyzed transacetalization-type reaction is operative, and displayed saturation kinetics. Moreover, this control experiment is indicative of a steady-state dynamic exchange equilibrium between unreacted acceptor in the reaction and the aglycone component of **3al**. Intriguingly, evaluation of all NMR spectra along the time coordinate revealed the detection of only α-glycosides **3al** and **3ak**. The absence of β-glycosides corroborate an S_N_1-type exchange mechanism, which involves the cleavage and reformation of the α-glycosidic linkage. A separate control experiment evaluating transacetalization on benzyl protected D-galactal revealed that this dynamic acceptor exchange process is not operative when ether protecting groups are used (Supplementary Fig. S55 and Supplementary Discussion 3).

To test the dependence of this dynamic exchange on XB activation, additional 5 mol% TBAI was added into the acceptor exchange experiment conditions to poison **A** (Fig. 8d). This experiment failed to generate **3ak**, supporting crucial XB catalytic influences in this dynamic equilibration. A ^13^C NMR experiment mixing a 1:1 ratio of **A:3al** (Fig. 8e) yielded a 0.158 p.p.m. downfield shift of the ^13^C C–I resonance of **A**, evidencing an XB catalytic interaction on the glycosidic bond.

### Concentration dependence of the reaction profile

In order to understand the mechanistic role of the glycosyl donor, the glycosyl acceptor and the XB catalyst on the reaction profile, as well as on the induction period, we varied the concentration of each substrate and conducted an in situ ^1^H NMR monitoring for the reaction between **1c** and **2al** catalyzed by **A**.

**Fig. 9:**
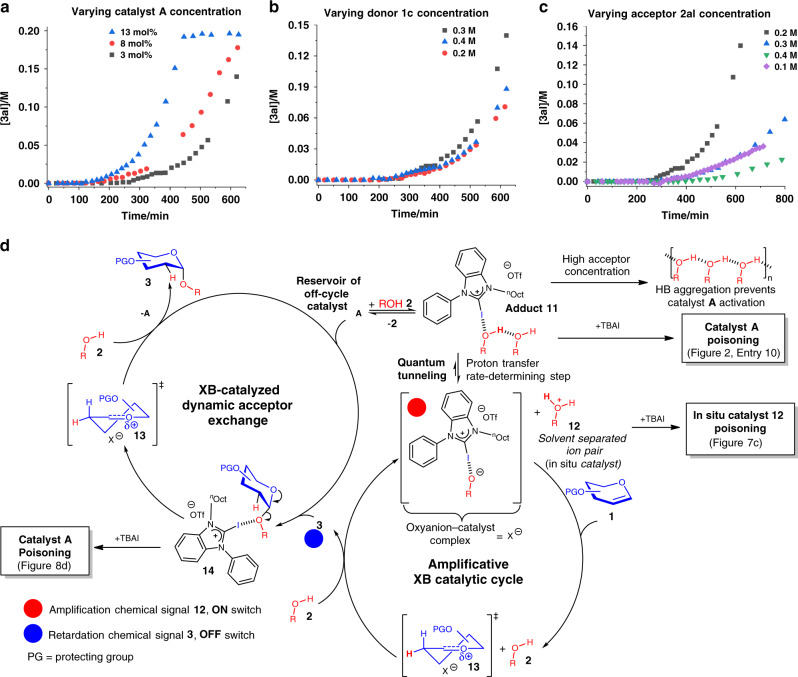
Reaction mechanism. **a** Varying catalyst **A** concentration on the kinetic profile, **b** varying donor **1c** concentration on the kinetic profile, **c** varying acceptor **2al** concentration on the kinetic profile, **d** proposed mechanism of XB-catalyzed 2-deoxyglycosylation through an XB-modulated intricate reaction network.

When the concentration of catalyst **A** was varied (Fig. 9a), we observed that increase in catalyst loadings resulted in a corresponding decrease in the induction period, and the left shifting of the sigmoidal profile, which is consistent with a positive order with respect to XB catalyst, and is indicative of the participation of the catalyst in the rate-determining step.

When the concentration of the glycosyl donor **1c** was varied (Fig. 9b), we observed that the reaction profiles were relatively invariant to the concentration changes and overlapped rather well with each other. The induction periods also remained relatively constant, suggesting that the glycal donor is not involved in the rate-determining step.

Interestingly, when the glycosyl acceptor’s concentration was permutated, a more complex kinetic behavior was observed (Fig. 9c). We observed a positive correlation at lower concentrations, i.e., in the concentration window between 0.1 to 0.2 M, concentration increases resulted in shortening of induction period. However, at larger concentrations beyond the 0.2 M threshold, we observed the onset of a negative correlation where retardation sets in at concentrations from 0.3 to 0.4 M. The observation of a positive order at the lower concentration window suggests the involvement of the glycosyl acceptor in the rate-determining step at standard conditions (0.2 M acceptor).

We speculate that the observed retardation at higher acceptor concentrations beyond the standard conditions could be due to the formation of a more extensive hydrogen-bonded isopropanol network (Fig. 9d), which might inhibit catalyst activity by stabilizing the OH bonds in isopropanol multimeric aggregates^89,90^, hence hindering the establishment of productive catalyst–glycosyl acceptor XB interactions for catalytic activation.

### Proposed intricate XB-modulated network mechanism

Based on the entirety of our mechanistic data, we propose that an intricate reaction network, comprising of multiple dynamic XB catalytic influences is operative in our methodology (Fig. 9d). This mechanistic explanation hinges upon the presence of an off-cycle reservoir of **A**, which shuttles between an amplificative cycle and a dynamic exchange cycle in response to ON/OFF chemical signals.

The mechanism starts with a slow formation of a critical amount of an in situ catalytic intermediate, which we postulate is the actual catalyst responsible for product amplification. While we made rigorous efforts to detect this intermediate in situ, however, due to minute amounts of this intermediate (presumably much less than the 3 mol% of **A** inferred from continuous amplification in standard and sequential kinetic studies) beyond the NMR detection limit (5%), we are unable to directly detect and characterize the intermediate.

Despite this, based on holistic analysis of numerous informative indirect experiments, such as in situ sequential experiments, base addition, and poisoning of the in situ generated species by TBAI, we deduced that this intermediate possesses Brønsted acidic characteristics, with concurrent XB catalytic dependency. Significantly, TBAI poisoning characteristics were not observed in control true Brønsted acid-catalyzed experiments, such as HOTf, which performed similarly regardless of the presence or absence of TBAI, hence excluding non-XB effects in our mechanism, such as trace acid catalysis^21^.

Hence, we propose based on these data that the catalytic intermediate in our protocol is a solvent-separated ion-pair **12**, comprising of a solvated XB-stabilized oxyanion–catalyst complex (X^−^) and a solvated protonated acceptor, arising from the rate-determining proton transfer step, which involves quantum tunneling (Fig. 9, and Supplementary Notes 17 and 18). X^−^ will further serve as an oxocarbenium neutralizing counteranion in subsequent steps. Our experiments ascertaining the presence of acceptor OH bond cleavage (Fig. 5), the crucial involvement of Brønsted acidity (Fig. 2, entries 13–15), and successful TBAI poisoning of the in situ catalyst (Fig. 7c) collectively corroborate the intermediacy of XB-dependent ion-pair **12**, as the true in situ catalyst through a proton transfer rate-determining step. This in situ solvent-separated ion-pair hypothesis is also further supported by primary KIE elevation due to increase in the tunneling effect^81,82^, when the reaction was conducted in a more nonpolar 10:1 PhCH_3_:CH_2_Cl_2_ solvent mixture. This proton transfer arising from the Lewis acidity of the XB donor on the alcoholic glycosyl acceptor is reminiscent of the Lewis acid-assisted Brønsted acid concept proposed by Yamamoto et al.^91^. In addition, this concept has also been applied by Schmidt et al. in glycosylations using gold complexes or boronates as Lewis acids^92,93^.

**12** can subsequently protonate C2 of glycal **1** to form oxocarbenium intermediate **13**. This step-wise mechanism is evidenced by scrambling of deuterium labels originating from the acceptor on C2 (Fig. 5, Equations 1 and 2). As **12** gets consumed in the initial catalytic cycle, the reversible **11** to **12** reaction will be increasingly shifted toward **12** as reaction time progresses, in situ amplifying the active catalyst **12**, resulting in an accelerating concave shaped kinetic in the initial phase of the sigmoidal profile (Fig. 7b). Subsequently, the alkoxide in **13** will attack the oxocarbenium species to form 2-deoxyglycoside **3** with the release of **A**, coupled with a sequential in-cycle intermolecular proton transfer between two molecules of acceptor **2** under the activation of the released catalyst **A** to regenerate the in situ catalyst **12**, analogous to an **11** to **12** conversion. On the other hand, the formation of more glycoside **3** through the amplification cycle (blue circle, Fig. 9d) activates the dynamic exchange mechanism, which siphons off-cycle **A**, arising from XB interactions with **A** and the glycosidic bond oxygen in **3** to form **14**. This lowering of the off-cycle concentration of **A** due to the dynamic acceptor exchange will accordingly deplete the concentration of **A** required to form increasing **12** in the amplificative cycle. This effect is registered by the rate deceleration after the inflection point in the standard sigmoidal kinetic profile (Fig. 6a), but attenuated in the sequential profile (Fig. 7b) due to pre-generation of more **12**. However, this effect is strengthened when we directly introduced the glycoside at the onset of the reaction, resulting in retardation (Fig. 8b).

Subsequently, the XB catalytic effect of **A** in **14** results in a dynamic acceptor exchange evidenced by our acceptor exchange experiment (Fig. 8c), TBAI poisoning, and ^13^C NMR shift (Fig. 8d, e), which involves firstly cleavage of the newly formed glycosidic linkage in **3** to reform oxocarbenium intermediate **13**. Next, a new molecule of **2** will first initiate a proton transfer with the original alkoxide ion in X^−^ to form a new alkoxide, which will attack the oxocarbenium ion to regenerate **3** coupled with the exit of the original protonated alkoxide as **2**, releasing catalyst **A** back into the off-cycle reservoir. Intriguingly, our proposed mechanism seems to constitute a simplified synthetic mimetic of complex noncovalent interactions modulated biochemical mechanisms catalyzed by enzymes, operating in tightly regulated mechanisms. Unexpectedly, we determined that dynamic XB-modulated mechanistic complexity^94^ could be constructed simply from a ternary chemical mixture comprising of a glycosyl donor, an acceptor, and a XB catalyst.

### Benchmarking noncovalent organocatalytic robustness between XB and thiourea catalysis

To evaluate the advances in noncovalent catalyst robustness of benzimidazolium XB catalyst **A**, and we sought to benchmark our XB catalytic protocol with conventional thioureas^47,56,95,96^.

In an effort to understand broadness and robustness across glycal donors beyond widely reported galactals, we observed a general superiority trend in using XB catalysis over thiourea on these non-galactal substrates. First, within the hexose family, donors such as silylated D-glucals and L-rhamnals work excellently with catalyst **A**, but not with **15** and **16**. Recognizing the possible caveat that could arise from protecting group influences, we performed the comparative glycosylation also with benzylated glucals and rhamnals. Significantly, literature known ether protecting group preference using thiourea catalysis on 2-deoxygalactosylation^47^ holds neither for glucosylation nor rhamnosylation.

**Fig. 10:**
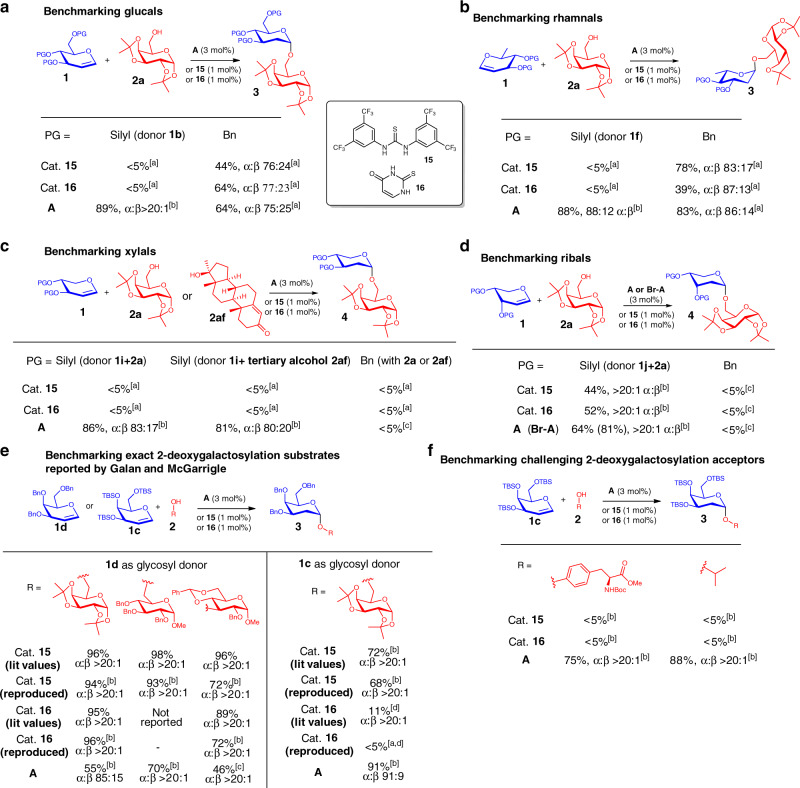
Benchmarking XB and thiourea organocatalysis. [a] ^1^H NMR yield determined with 1,3,5-trimethoxybenzene as an internal standard. [b] Isolated yields after flash column chromatography. [c] A mixture with inseparable Ferrier-type product impurity was obtained after flash chromatography purification, the yield was calculated by ^1^H NMR analysis. [d] Reaction time was 48 h, literature gave 68% yield with 2-methyl THF as solvent at 83 °C refluxing temperature. **a** Benchmarking glucals, **b** Benchmarking rhamnals, **c** Benchmarking xylals, **d** Benchmarking ribals, **e** Benchmarking reported 2-deoxygalactosylation substrates, **f** Benchmarking challenging 2-deoxygalactosylation acceptors.

Second, analogous trends favoring XB over thiourea catalysis can also be observed from the pentose family of glycal donors. Using silylated D-xylal **1i** (Fig. 10c), the benchmarking experiments revealed that catalyst **A** enabled smooth glycosylation, but no reaction was observed with **15** or **16**. Using a challenging sterically hindered tertiary alcohol, such as **2af**, we noticed that XB catalysis still furnished the desired glycosylation product smoothly, whereas catalysis via **15** or **16** do not generate any observable product. In the case of silylated D-ribal **1j** (Fig. 10d), both catalyst **A** and in particular the brominated version of catalyst **A** (**Br-A** in parenthesis) gave superior yields superceding the thiourea congeners. This halogen swapping advantage by XB catalysis will be further discussed in a later section. As a control experiment to clarify specificity of protecting group effects on noncovalent catalysis, we noticed that benzylated xylals and ribals remained lowly reactive, regardless whether thiourea or XB catalysis are employed.

To provide fairer comparison with catalyst **15** and **16**, and a more holistic understanding across these noncovalent catalysts, we reproduced the exact conditions reported by Galan and McGarrigle with catalyst **A** on benzylated galactals, a known strength of these prior methodologies^47,56^. Our comparative data confirmed that ether protected galactals are better suited for thiourea catalysis, although XB catalysis can also activate benzylated galactals reasonably (Fig. 10e). However, we have demonstrated that employing silyl protecting group in conjunction with XB catalysis gave consistently excellent performance in yields and anomeric selectivity on the galactal donor family (Fig. 3). In cases where more challenging acceptors, such as the phenolic group in protected tyrosine and isopropanol are used, XB catalysis gave clear superiority, while thiourea/thiouracil protocols generated negligible product (Fig. 10f).

In all, we note that XB catalysis offered distinct advantages over thiourea/thiouracil catalysis in activating the entire spectrum of different sugar donors beyond galactals. Pure thiourea catalysis is useful in 2-deoxygalactosylation with ether protecting groups, as previously demonstrated by Galan and McGarrigle et al.^47,56,95,96^, but reactivity do not scale well across donors, as well as challenging acceptors, and the limitations are evident when other non-galactal glycosyl donors are employed.

These benchmarking experiments reinforce immense value of exploiting XB catalytic protocols as an enhanced organocatalytic tool for accommodating a broader variety of non-galactal glycosyl donors, when conventional thiourea catalytic methods are not optimal.

### Applicability of switchable mechanism to elevate yields

**Fig. 11:**
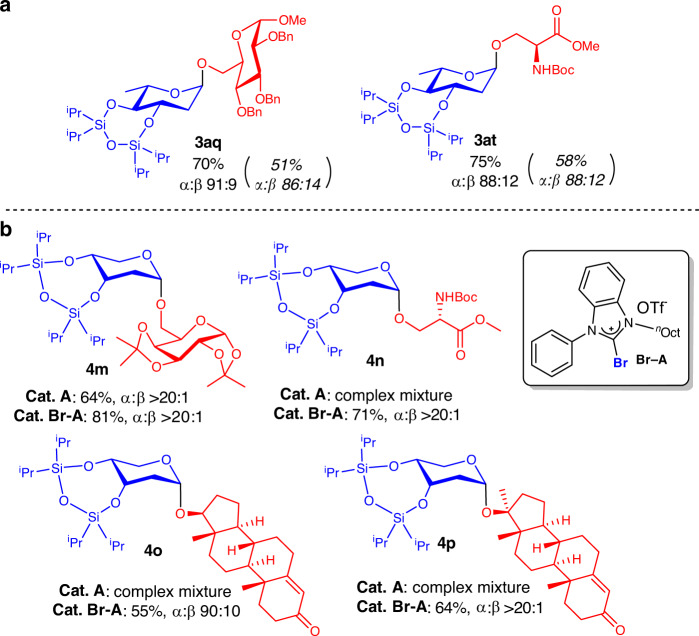
XB tunability. **a** Elevation of yields on rhamnosides using the sequential procedure, yields, and selectivity from standard conditions indicated in parenthesis. **b** Iodine to bromide halogen swapping on **A** resulted in significantly expanded 2-deoxyribosylation scope.

To demonstrate further applicability of the XB-dependent “switchable” characteristic, we performed sequential experiments analogous to Fig. 7 in an attempt to elevate yields of representative rhamnosides **3aq** and **3at** (Fig. 11a) that gave moderate yields in our reaction scope under standard conditions. To our delight, the pre-generation of in situ catalysts had a positive effect in our protocol to substantially improve chemical yields of sluggish substrates.

### Tunability of XB catalysis via halogen swapping

Despite the broadly improved performance of catalyst **A**, we noticed certain limitations of our protocol utilizing the iodide derivative **A**, particularly in 2-deoxyglycosylation reactions with D-ribals. Meticulous experiments revealed that **A** is very sensitive to the acceptors employed, furnishing complex mixtures when amino-acid-derived acceptors or more sterically hindered secondary and tertiary alcohol containing steroidal acceptors were employed (Fig. 11b, **4n–4p**).

To address this issue, we reasoned that the σ-hole on the iodine atom on **A** could be fine-tuned by employing an iodine to bromine halogen swapping strategy. Surprisingly, using the bromide derivative **Br-A** as catalyst showed marked improvement (Fig. 11b) to access such challenging glycosidic linkages in **4n–4p** with very good to excellent anomeric selectivity, which were not accessible via **A**.

Gratifyingly, even 2-deoxyribosylation to **4m** gave substantially improved yield (81%) using **Br-A**. These observations support that postulate that stronger iodide XB donors might be overly strong Lewis acids for sensitive ribal substrates, which resulted in undesired decomposition pathways. As such, tuning XB donor strength by employing the weaker **Br-A** donor could provide an attenuated, milder catalytic activation route which circumvents decomposition, but increase catalytic performance. This success of our halogen swapping method depicts the power of XB catalysis as a high-performance-tunable noncovalent organocatalytic tool, especially when sensitivity of substrates is pivotal.

## Discussion

In conclusion, we demonstrate a robust XB organocatalyzed 2-deoxyglycosylation, operating through an intricately XB catalyst controlled reaction network. This report showcases a marked advancement in noncovalent organocatalysis, where the use of lesser explored XB catalysis resulted in mechanistic divergences, and significantly elevated substrate scope compared to conventional thiourea catalysis, particularly in the formation of challenging glycosidic linkages. Detailed mechanistic studies were also illuminating and instructive of unique and hitherto unknown intricacies and dynamism of XB activation, such as the involvement of quantum tunneling.

In situ temporal kinetic experiments and comparison revealed a sigmoidal kinetic profile characteristic of autoinductive reactions. KIE measurements also revealed unusually high KIE values beyond the semiclassical limit, as well as KIE elevation in nonpolar solvents, which are indicative of quantum tunneling and the formation of an in situ ion-pair intermediate in the rate-determining proton transfer step. Intriguingly, deeper studies unraveled a biomimetic reaction network embedded with switchable ON/OFF mechanism, modulated by dynamic XB interactions between catalyst, substrates, and products. Furthermore, we capitalized on this switchable property, to elevate yields of 2-deoxyrhamnosides through a sequential protocol. We also demonstrate that tuning σ-hole properties via a halogen swap on the catalyst is beneficial in elevating XB catalyst robustness on glycosyl donors, such as D-ribals, particularly when more challenging glycosyl acceptors are employed.

We are optimistic that the development of this protocol has promising wider implications in noncovalent organocatalysis, in deconvoluting differences of mechanism and substrate tolerances between XB and thiourea catalysis, and will contribute to developing mild XB catalysis as a competitive new generational tool in challenging, but biologically relevant bond forming reactions.

## Methods

### General techniques

Unless otherwise stated, all reactions were set up under inert atmosphere (argon) utilizing glassware that were oven-dried and cooled under argon purging. Silica gel flash column chromatography was performed on deactivated silica gel Merck 60 (particle size 40–63 μm). Starting materials were procured from commercial sources and used without purifications. Solvents were dried according to reported procedures or procured from commercial suppliers. Monitoring of reactions was done using thin layer chromatography (TLC) on Merck silica gel aluminum plates with F254 indicator. TLC visualization of plates was performed under UV light (254 nm) or employing KMnO_4_ stain or H_2_SO_4_-EtOH (10% H_2_SO_4_ v/v).

NMR spectra were collected at 300 K on a Bruker DRX400 (400 MHz), Bruker DRX500 (500 MHz), INOVA500 (500 MHz), or Bruker DRX700 (700 MHz) spectrometers, using CDCl_3_, CD_2_Cl_2_, acetone-d6, CD_3_CN, or benzene-d6 as deuterated solvents. Data for ^1^H NMR are reported as follows: chemical shift (*δ* p.p.m.), multiplicity (s = singlet, d = doublet, t = triplet, q = quartet, m = multiplet, and br = broad), coupling constant (Hz), NMR spectra were internally referenced to the following residual solvent signals (CDCl_3_: *δ* = 7.26 p.p.m. for ^1^H, *δ* = 77.16 p.p.m. for ^13^C; CD_2_Cl_2_: *δ* = 5.32 p.p.m. for ^1^H, *δ* = 54.00 p.p.m. for ^13^C; acetone-d6: *δ* = 2.05 p.p.m. for ^1^H, *δ* = 29.92 p.p.m. for ^13^C; CD_3_CN: *δ* = 1.94 p.p.m. for ^1^H, *δ* = 1.32 p.p.m. for ^13^C of CD_3_, C_6_D_6_: *δ* = 7.16 p.p.m. for ^1^H, *δ* = 128.06 p.p.m. for ^13^C).

High-resolution mass spectra were recorded on an LTQ Orbitrap mass spectrometer coupled to an Accela HPLC-System (HPLC column: Hypersyl GOLD, 50 mm×1 mm, particle size 1.9 μm, ionization method: electron spray ionization) and Bruker ultrafleXtreme MALDI-TOF–TOF (three decimal accuracy). Optical rotations were measured in a Schmidt + Haensch Polartronic HH8 polarimeter equipped with a sodium lamp source (589 nm), and are reported as follows: [*α*]*D* *T* °C (*c* = g/100 mL, solvent). ITC experiments were performed on a MicroCal VP-ITC device.

The anomeric ratio was determined by ^1^H NMR spectroscopy on the crude reaction mixture and the anomeric configuration was determined by 2D NOESY. Chemical yields refer to combined isolated yields of all anomers after flash column chromatography. NMR yields were determined using appropriate internal standards.

### General procedure for XB-catalyzed 2-deoxyglycosylation

A mixture of catalyst **A** (3–10 mol%), glycosyl acceptor **2** (0.2 mmol), and glycal **1** (0.3–0.4 mmol) was dissolved in anhydrous CH_2_Cl_2_ (1 mL, 0.2 M for **2**), and sealed in a dry tube under argon. The mixture was stirred at a specific temperature (room temperature to 50 °C depending on the substrate). Subsequently, the solvent was removed under reduced pressure and the residue was first analyzed by crude ^1^H NMR to determine anomeric selectivity and then subjected to flash column chromatography (dry loading) to give the 2-deoxyglycoside **3** or **4**.

## Supplementary information

Supplementary Information

## Data Availability

The authors declare that the data supporting the findings of this study are available within the article and its Supplementary Information files. Additional data are available from the corresponding author upon reasonable request.
