# Supplementary material for: A robust and tunable halogen bond organocatalyzed 2-deoxyglycosylation involving quantum tunneling
Source: Nat Commun. 2020 Sep 30;11:4911. doi: 10.1038/s41467-020-18595-2 (PMC7527348; doi:10.1038/s41467-020-18595-2)
Supplement: Supplementary file 1 — Supplementary Information [file 41467_2020_18595_MOESM1_ESM.pdf]

Supplementary Information

A Robust and Tunable Halogen Bond Organocatalyzed 2-Deoxyglycosylation Involving Quantum Tunneling

Xu et al.

Table of Contents

Table of contents………………………………………………………………… S2
Supplementary methods………………………………………………………………S3
General information……………………………………………………………………S3
General procedure for optimization of XB catalyzed glycosylation of D-glucal…………S3
General procedure for XB catalyzed glycosylation of glycals……………………………S3
General procedure for XB catalyzed glycosylation of glycals in a sequential operation.…S4
Method for *in-situ* NMR monitoring for glycosylation of *tert*-butyldimethylsilylated galactal **1c** under standard conditions…..………………………………..……...........S4
Methods for *in-situ* NMR monitoring for glycosylation of *tert*-butyldimethylsilylated galactal **1c** catalyzed by HCl……………………………………..…………………..S4
Methods for *in-situ* sequential NMR monitoring for glycosylation of *tert*-butyldimethylsilylated galactal **1c** ………………………...…..…………………….……S4
Method for *in-situ* NMR monitoring for the reaction with initial addition of 20 mol% **3al** to the standard XB catalytic conditions……………………………………………………S4
Method for *in-situ* NMR monitoring for the sequential reaction with following addition of 20 mol% **3al**……………………………..……………………………………………S4
Method for *in-situ* NMR monitoring for the glycosylation of tribenzylated galactal **1d**.....S4
Method for concentration dependence experiments…………………………………….S5
Method for confirmatory control experiments to exclude pure autocatalysis….…………..S5
Method for sequential reaction terminated by TBAI…………………………………….S5
Method for determining the primary kinetic isotope effect…………………...………S5
Method for determining the secondary kinetic isotope effect…………………………..S5
Method for acceptor exchange experiment……………………………………………..S6
Procedure for the acceptor exchange experiment in the presence of TBAI……….………S6
Procedure for the *in situ* acceptor exchange experiment…………………………………..S6
Procedure for the determination of anomerization for the compound **3t**…………………S6
Method for NMR titration experiments and binding constant calculation……………......S6
Method for investigating the interaction between halogen bond catalyst **A** and TBAB or TBAI by NMR spectra analysis………………………………………………………S6
Method for determining the interaction between halogen bond catalyst **A** and TBAB or TBAI by isothermal titration calorimetry……………………………………………......S6
Method for detecting S_NAr attack product………………………………………………S7
Procedures for the benchmarking cases ……………………………..………………S7
Decomposition experiments……………………………………………………………S7
Synthetic methods and characterization data………………………………...……..S7
Supplementary notes..………………………………………………………………....S39
Supplementary discussions……………………………………………………………S42
Supplementary references…..……………………….....………………………..........S45
Supplementary tables and figures..……………………………………………………S46

Supplementary Methods

General Information: Unless otherwise stated, all reactions were set up under inert atmosphere (argon) utilizing glassware that were oven dried and cooled under argon purging. Silica Gel Flash Column Chromatography was performed on deactivated *Silica gel Merck 60* (*particle size 40-63 μm*). Basified silica gel was obtained by immersing silica in the 5% NEt_3/pentane overnight, and then the solvent was removed in vacuo. Starting materials were purchased directly from commercial suppliers (Sigma Aldrich, Acros, Alfa Aesar, VWR, TCI) and used without further purifications unless otherwise stated. All solvents were dried according to standard procedures or brought from commercial suppliers. Reactions were monitored using thin-layer chromatography (TLC) on *Merck silica gel aluminium plates with F254 indicator*. Visualization of the developed plates was performed under UV light (254 nm) or KMnO4 stain or H_2SO_4-EtOH (10% H_2SO_4 v/v).

NMR characterization data (^1H NMR, ^{13}C NMR and 2D spectra) were collected at 300 K on a *Bruker DRX400 (400 MHz)*, *Bruker DRX500 (500 MHz)*, *INOVA500 (500 MHz)* and *Bruker DRX700 (700 MHz)* using $CDCl_3$, CD_2Cl_2, acetone-d_6, CD_3CN or benzene-d_6 as solvent. Data for ^1H NMR are reported as follows: chemical shift (δ ppm), multiplicity (s = singlet, d = doublet, t = triplet, q = quartet, m = multiplet, br = broad), coupling constant (Hz), integration with the solvent resonance as internal standard ($CDCl_3$: δ = 7.26 ppm for ^1H, δ = 77.16 ppm for ^{13}C; CD_2Cl_2: δ = 5.32 ppm for ^1H, δ = 54.00 ppm for ^{13}C; acetone-d_6: δ = 2.05 ppm for ^1H, δ = 29.92 ppm for ^{13}C; CD_3CN: δ = 1.94 ppm for ^1H, δ = 1.32 ppm for ^{13}C of CD_3, C_6D_6: δ = 7.16 ppm for ^1H, δ = 128.06 ppm for ^{13}C).

High resolution mass spectra were recorded on an *LTQ Orbitrap* mass spectrometer coupled to an *Accela HPLC*-System (HPLC column: *Hypersyl GOLD*, 50 mm x 1 mm, particle size 1.9 μm, ionization method: electron spray ionization) and Bruker ultrafleXtreme MALDI-TOF-TOF (3 decimal accuracy). Optical rotations were measured in a *Schmidt + Haensch Polartronic HH8* polarimeter equipped with a sodium lamp source (589 nm), and are reported as follows: $[\alpha]_D^{T\,°C}$ (*c* = g/100 mL, solvent).

Isothermal titration calorimetry (ITC) experiments were performed on a *MicroCal VP-ITC* device.

The ratio of anomers was determined by ^1H-NMR of the crude reaction mixture *via* integration of characteristic signals of the anomeric proton in the ^1H NMR spectra, and the anomeric configuration was determined by NOESY. Chemical yields refer to isolated substances after flash column chromatography, combined yield of both anomers reported. NMR yields were determined using 1,3,5-trimethoxybenzene or mesitylene as internal standard.

General procedure for optimization of XB catalyzed glycosylation of D-glucal (see Supplementary table S1, Supplementary figure S1): A mixture of halogen bond catalyst, isopropylidine protected galactose **2a** (0.1 mmol, 1 equiv.) and D-glucal **1a-b** (0.15 mmol, 1.5 equiv.) was dissolved in anhydrous solvent (0.2 M) and sealed in a dry tube under argon. The mixture was stirred at a preheated temperature. Afterwards, the solvent was removed under reduced pressure and the reaction mixture was analyzed by crude ^1H NMR with 1,3,5-trimethoxybenzene as the internal standard.

General procedure for XB catalyzed glycosylation of glycals.

Procedure A: A mixture of halogen bond catalyst **A** (3-10 mol%), glycosyl acceptor **2** (0.2 mmol), glycal **1** (0.3-0.4 mmol) was dissolved in anhydrous CH_2Cl_2 (1 mL, 0.2 M for **2**) and sealed in a dry tube under argon. The mixture was stirred at a specific temperature (23 ˚C to 50 ˚C depending on the substrate). Afterwards, the solvent was removed under reduced pressure and the residue was analyzed by crude ^1H NMR and then subjected to flash column chromatography (dry loading) to give the 2-deoxyglycoside **3** or **4**.

Procedure B: A mixture of halogen bond catalyst **Br-A** (5-10 mol%), glycosyl acceptor **2** (0.2 mmol), glycal **1j** (0.4 mmol) or **1k** (0.3 mmol) was dissolved in anhydrous CH_2Cl_2 (1 mL, 0.2 M for **2**) and sealed in a dry tube under argon. The mixture was stirred at 40 ˚C for 24-48 h. Afterwards, the solvent was removed under reduced pressure and the residue was analyzed by crude ^1H NMR and then subjected to flash column chromatography (dry loading) to give the 2-deoxyglycoside.

General procedure for XB catalyzed glycosylation of glycals in a sequential operation (Figure 11a in the manuscript)

To a dry tube, halogen bond catalyst **A** (5.8 mg, 5 mol%) and acceptor (0.2 mmol, 1 equiv.) were added under argon and dissolved in CH_2Cl_2 (1 mL) and stirred at 30 ˚C or 50 ˚C for 6 h. Then glycosyl donor **1f** (0.4 mmol, 2 equiv.) was added subsequently and the newly mixture was stirred at 30 ˚C for another 48 h and 50 ˚C for another 36 h. Afterwards, the solvent was removed under reduced pressure and the residue was analyzed by crude ^1H NMR and then subjected to flash column chromatography (dry loading) to give the 2-deoxyglycoside.

Method for *in-situ* NMR monitoring for glycosylation of *tert*-butyldimethylsilylated galactal 1c under standard conditions. (See Supplementary figure 10-11, Supplementary table S4)

To a dry NMR tube, halogen bond catalyst **A** (1.7 mg, 0.003 mmol) was added, then isopropanol **2al** (6.0 mg, 0.10 mmol) was injected into the tube *via* microinjector. The *tert*-butyldimethylsilyated galactal **1c** (73.3 mg, 0.15 mmol) was dissolved in CD_2Cl_2 (0.5 mL) and then transferred to the NMR tube. Mesitylene (10 uL, 0.072 mmol) was also injected. Afterwards, the tube was sealed with parafilm and measured on the NMR spectrometer at 30 ˚C by recording ^1H spectra at different time.

Method for *in-situ* NMR monitoring for glycosylation of *tert*-butyldimethylsilylated galactal 1c catalyzed by HCl. (See Supplementary figure 12-13, Supplementary table S5)

To a dry NMR tube, isopropanol **2al** (6.0 mg, 0.10 mmol) was injected *via* microinjector and the *tert*-butyldimethylsilyated galactal **1c** (73.3 mg, 0.15 mmol) was dissolved in CD_2Cl_2 (0.5 mL) and then transferred to the NMR tube. Then mesitylene (10 uL, 0.072 mmol) and HCl (1 M diethyl solution, 3 uL) were injected respectively. Afterwards, the tube was sealed with parafilm and measured on the NMR spectrometer at 30 ˚C by recording ^1H spectra at different time.

Method for *in-situ* sequential NMR monitoring for glycosylation of *tert*-butyldimethylsilylated galactal 1c. (See Supplementary figure 14-15, Supplementary table S6)

To a dry NMR tube, halogen bond catalyst **A** (1.7 mg, 0.003 mmol), isopropanol **2al** (6.0 mg, 0.10 mmol) and CD_2Cl_2 (0.2 mL) were added, and the mixture was placed in the 30 ˚C oil bath for 6 h. Then *tert*-butyldimethylsilyated galactal **1c** (73.3 mg, 0.15 mmol) was dissolved in CD_2Cl_2 (0.3 mL) and transferred to the NMR tube. And mesitylene (10 uL, 0.072 mmol) was injected. Afterwards, the tube was sealed with parafilm and measured on the NMR spectrometer at 30 ˚C by recording ^1H spectra at different time.

Method for *in-situ* NMR monitoring for the reaction with initial addition of 20 mol% 3al to the standard XB catalytic conditions. (See Supplementary figure 16-17, Supplementary table S7)

To a dry NMR tube, halogen bond catalyst **A** (1.7 mg, 0.003 mmol), isopropanol **2al** (6.0 mg, 0.10 mmol), **3al** (11.0 mg, 0.02 mmol) were added, and **1c** (73.3 mg, 0.15 mmol) was dissolved in CD_2Cl_2 (0.5 mL) then transferred into the same NMR tube. Afterwards, mesitylene (10 uL, 0.072 mmol) was injected. Then the tube was sealed with parafilm and measured on the NMR spectrometer at 30 ˚C by recording ^1H spectra at different time.

Method for *in-situ* NMR monitoring for the sequential reaction with following addition of 20 mol% 3al after *in situ* generated catalyst 12. (See Supplementary figure 18-19, Supplementary table S8)

To a dry NMR tube, halogen bond catalyst **A** (1.7 mg, 0.003 mmol), isopropanol **2al** (6.0 mg, 0.10 mmol) were added. The mixture was dissolved in CD_2Cl_2 (0.2 mL) and immersed in the 30 ˚C oil bath for 6 h. Then **3al** (11.0 mg, 0.02 mmol) and **1c** (73.3 mg, 0.15 mmol) was dissolved in CD_2Cl_2 (0.3 mL) then transferred into the same NMR tube. Afterwards, mesitylene (10 uL, 0.072 mmol) was injected. Then the tube was sealed with parafilm and measured on the NMR spectrometer at 30 ˚C by recording ^1H spectra at different time.

Method for *in-situ* NMR monitoring for the glycosylation of tribenzylated galactal 1d. (See supplementary figure S22-23, supplementary table S9)

To a dry NMR tube, halogen bond catalyst **A** (1.7 mg, 0.003 mmol), isopropanol **2al** (6.0 mg, 0.10 mmol), tribenzylated galactal **1d** (62.5 mg, 0.15 mmol) were added. Then CD_2Cl_2 (0.5 mL) and mesitylene (10 uL, 0.072 mmol) were injected. Afterwards, the NMR tube was placed in the 35 ˚C oil bath, and measured on the NMR spectrometers at 27 ˚C at different time.

Method for concentration dependence experiments (See supplementary figure S61-71, supplementary table S17-20)
To a dry NMR tube, halogen bond catalyst **A** and mesitylene (10 uL, 0.072 mmol) were added. Tri-*tert*-butyl galactal **1c** was dissolved in CD_2Cl_2 and then transferred to the NMR tube before immersing in an oil bath (30 ˚C). Isopropanol was injected at the same time. Afterwards, the NMR tube was measured on the NMR spectrometer at different time. The accurate volume or mass used for the concentration dependence experiments, please see the Supplementary table S17 for details.

Method for confirmatory control experiment to exclude pure autocatalysis. (See Supplementary Figure S21)
To a dry tube, glycoside **3al** (11.0 mg, 0.02 mmol) and *tert*-butyldimethylsilyated galactal **1c** (73.3 mg, 0.15 mmol) were added, then isopropanol **2al** (6.0 mg, 0.10 mmol) was injected into the tube *via* microinjector. Afterwards, anhydrous CH_2Cl_2 (0.5 mL) was added and the tube was sealed with parafilm and stirred at preheated 30 ˚C oil bath for 48 h then the solvent was removed and the residue was analyzed by crude ^{1}H NMR spectroscopy.

Method for sequential reaction terminated by TBAI. (see Supplementary figure S38, Figure 7c in the manuscript) To a dry NMR tube, halogen bond catalyst **A** (3.5 mg, 0.006 mmol), isopropanol **2al** (12.0 mg, 0.20 mmol) and CH_2Cl_2 (0.5 mL) were added, and the mixture was placed in the 30 ˚C oil bath for 6 h. Then TBAI (4.4 mg, 0.012 mmol) and *tert*-butyldimethylsilyated galactal **1c** (146.7 mg, 0.30 mmol) dissolved in CH_2Cl_2 (0.5 mL) were added to the tube. After stirring at 30 ˚C for 24 h, the solvent was removed and the residue was measured by crude ^{1}H NMR spectrum analysis.

Method for determining the primary kinetic isotope effect. (See Supplementary Figure S39-43 and Figure 5, equation (3) (4) and (5) in the manuscript)
Method A: A dry tube with a stirrer bar was charged with *tert*-butyldimethylsilylated galactal **1c** (97.8 mg, 0.2 mmol), isopropanol **2al** (12.0 mg, 0.2 mmol), deuterated isopropanol ^{i}PrOD **5** (12.2 mg, 0.2 mmol) and halogen bond catalyst **A** (3.5 mg, 0.006 mmol) under argon. Then anhydrous CH_2Cl_2 (1 mL) was added to the tube and the mixture was stirred at 30 ˚C for 48 h. Afterwards, the solvent was removed in vacuo and the residue (dry loading) was purified by flash column chromatography (pentane/ CH_2Cl_2 as eluent). The ratio for normal product and deuterated product was determined by ^{1}H NMR spectroscopy.
Method B: A dry tube with a stirrer bar was charged with *tert*-butyldimethylsilylated galactal **1c** (146.7 mg, 0.3 mmol), isopropanol **2al** (12.0 mg, 0.2 mmol), deuterated isopropanol ^{i}PrOD **5** (12.2 mg, 0.2 mmol) and halogen bond catalyst **A** (3.5 mg, 0.006 mmol) under argon. Then anhydrous CH_2Cl_2 (1 mL) was added to the tube and the mixture was stirred at 30 ˚C for 12 h. Afterwards, the solvent was removed in vacuo and the residue (dry loading) was purified by flash column chromatography (pentane/ CH_2Cl_2 as eluent). The ratio for normal product and deuterated product was determined by ^{1}H NMR spectroscopy. This experiment was further performed twice, one reaction purified by normal silica gel and the other purified by basified silica gel.
Method C: A dry tube with a stirrer bar was charged with *tert*-butyldimethylsilylated galactal **1c** (146.7 mg, 0.3 mmol), isopropanol **2al** (12.0 mg, 0.2 mmol), deuterated isopropanol ^{i}PrOD **5** (12.2 mg, 0.2 mmol) and halogen bond catalyst **A** (3.5 mg, 0.006 mmol) under argon. Then an anhydrous mixed solvent (0.1 mL CH_2Cl_2 + 1 mL Toluene) was added to the tube and the mixture was stirred at 30 ˚C for 11 or 14 h. Afterwards, the solvent was removed in vacuo and the residue (dry loading) was purified by flash column chromatography (pentane/ CH_2Cl_2 as eluent). The ratio for normal product and deuterated product was determined by ^{1}H NMR spectroscopy.

Method for determining the secondary kinetic isotope effect. (See Supplementary figure S45-46 and Figure 5, equation (6) and (7) in manuscript)
Method A: A dry tube with a stirrer bar was charged with *tert*-butyldimethylsilylated galactal **1c** (97.8 mg, 0.2 mmol), isopropanol **2al** (12.0 mg, 0.2 mmol), deuterated 2-D-isopropanol **9** (12.2 mg, 0.2 mmol) and halogen bond catalyst **A** (3.5 mg, 0.006 mmol) under argon. Then anhydrous CH_2Cl_2 (1 mL) was added to the tube and the mixture was stirred at 30 ˚C for 24 h. Afterwards, the solvent was removed in vacuo and the residue (dry loading) was purified by flash column chromatography (pentane/ CH_2Cl_2 as eluent). The ratio for normal product and deuterated product was determined by ^{1}H NMR spectroscopy.
Method B: A dry tube with a stirrer bar was charged with *tert*-butyldimethylsilylated galactal **1c** (36.7 mg, 0.075 mmol), deuterated *tert*-butyldimethylsilylated galactal **8** (37.0 mg, 0.075 mmol), isopropanol **2al** (6.0 mg, 0.1 mmol), and halogen bond catalyst **A** (1.7 mg, 0.003 mmol) under argon. Then

anhydrous CH_2Cl_2 (1 mL) was added to the tube and the mixture was stirred at 30 ˚C for 16 h. Afterwards, the solvent was removed in vacuo and the residue (dry loading) was purified by flash column chromatography (pentane/ CH_2Cl_2 as eluent). The ratio for normal product and deuterated product was determined by ^1H NMR spectroscopy.

Method for acceptor exchange experiment. (See Supplementary figure S49-50, Supplementary table S10 and Figure 8c in manuscript)
To an NMR tube, compound **3al** (27.4 mg, 0.05 mmol), propargyl alcohol (5.6 mg, 0.1 mmol) and catalyst **A** (1.5 mg, 5 mol%) were added. Then CD_2Cl_2 (0.5 mL) and mesitylene (10 µL) were also added. Afterwards, the tube was measured and analyzed by ^1H NMR spectra.

Procedure for the acceptor exchange experiment in the presence of TBAI. (See supplementary figure S51, Figure 8d in the manuscript) To an NMR tube, **3al** (27.4 mg, 0.05 mmol, 1 equiv.), propargyl alcohol (5.6 mg, 0.1 mmol, 2 equiv.), TBAI (0.92 mg, 5 mol%) and catalyst **A** (1.5 mg, 5 mol%) were added. Then CD_2Cl_2 (0.5 mL) and mesitylene (10 µL) were also added. The mixture was left at room temperature for 3 h then 30 ˚C for 12 h. Afterwards, the tube was measured and analyzed by ^1H NMR spectra.

Procedure for the *in-situ* acceptor exchange experiment. (See supplementary table S11, supplementary figure S52-53) To an NMR tube, **1c** (73.3 mg, 0.15 mmol, 1.5 equiv.), isopropanol **2al** (6.0 mg, 0.1 mmol, 1 equiv.), and catalyst **A** (2.9 mg, 5 mol%) were added. Then CD_2Cl_2 (0.5 mL) and mesitylene (10 µL) were also added. The mixture was immersed in the oil bath (30 ˚C) and monitored by ^1H NMR spectra. At one point, **2r** (11.2 mg, 0.2 mmol, 2 equiv.) was added to the NMR tube and then the tube was measured by ^1H NMR spectra.

Procedure for the determination of anomerization for the compound 3t (See supplementary figure S54-56)
Method A: To an NMR tube, compound **3t** (67.7 mg, 0.1 mmol), acceptor **2a** (26.0 mg, 0.1 mmol), halogen bond catalyst **A** (1.7 mg, 0.003 mmol) were added. Then CD_2Cl_2 (0.5 mL) and mesitylene (10 µL) were injected. The tube was placed in the preheated 30 ˚C oil bath for 24 h.
Method B: To an NMR tube, compound **3t** (67.7 mg, 0.1 mmol), acceptor **2al** (0.2 mmol), halogen bond catalyst **A** (2.8 mg, 0.005 mmol) were added. Then CD_2Cl_2 (0.5 mL) and mesitylene (10 µL) were injected. The tube was placed in the preheated 30 ˚C oil bath for 24 h.
An experiment only with compound **3t** and halogen bond catalyst **A** in CD_2Cl_2 (0.5 mL) in the preheated 30 ˚C oil bath for 4 h was also performed. The ^1H NMR spectrum did not show any observable change in anomeric selectivity.

Method for NMR titration experiments and binding constant calculation. (see Supplementary table S2, Supplementary figure S2 for titration of **2al**, Figure 4a-b in the manuscript; Supplementary figure S36 for titration of **1c**; Supplementary figure S37 and Figure 8e in the manuscript for **3al**): To an NMR tube, halogen bond catalyst **A** (5.8 mg, 0.01 mmol) and varying amounts of isopropanol **2al** were added. Varying amounts of CD_2Cl_2 were added in order to reach a total volume of 500 µL. The detected ^{13}C shifts of *C*-I are shown.
The change of ^{13}C shift of *C*-I versus concentration of isopropanol **2al** is plotted and the titration curve is fitted to 1:1 stoichiometry model to determine a binding constant.

Method for investigating the interaction between halogen bond catalyst A and TBAB or TBAI by NMR spectra analysis. (see Supplementary figure S24-35) To an NMR tube, halogen bond catalyst **A** (5.8 mg, 0.01 mmol) and TBAB (tetrabutylammonium bromide) (3.2 mg, 0.01 mmol) or TBAI (tetrabutylammonium iodide) (3.7 mg, 0.01 mmol) were added. Then CD_2Cl_2 (0.5 mL) was injected into the same tube, after that, the mixture was recorded on the NMR spectrometer.

Method for determining the interaction between halogen bond catalyst A and TBAB or TBAI by isothermal titration calorimetry. (See Supplementary table S3, Supplementary figure S3-4)
ITC experiments were carried out at 30 ˚C in MeCN on a *Microcal VP-ITC* using a reference power of 20 µcal/s, a filter period of 1 s, a stirrer speed of 307 rpm, an injection volume of 9 µL, and a time spacing of 240 s between injections. Evaluation of the obtained data sets was performed with *Origin 7*. The halogen bond catalyst **A** was regarded as Host, and the TBAB or TBAI was regarded as Guest. The detail experiment information was listed in the Table S3.

Method for detecting S_NAr attack product. (See Supplementary figure S5-9, Supplementary Note 16) To an NMR tube, halogen bond catalyst **A** (5.8 mg, 0.01 mmol), *N*-(*tert*-Butoxycarbonyl)-L-cysteine methyl ester (0.04 mmol) or isopropanol **2al** (0.04 mmol) and anhydrous CD_2Cl_2 (0.5 mL) was added. Then the NMR tube was sealed and placed in the preheated 40 ˚C oil bath for 4 h (Experiment at 30 ˚C for 6 h for reaction **B** (Figure S5) was also performed). Afterwards, the mixture was measured by crude ^1H NMR. As well, the mixture was measured to detect the MS by LC-MS.

Procedures for the benchmarking cases (see supplementary table S12 and S16, Figure 10 in the manuscript)
Method A for thiourea **15** catalysis**:** The monosaccharide acceptor (0.500 mmol) was weighed into a tube and placed under vacuum for 1 h. Then the flask was filled with N_2, followed by the addition of the Glycal (0.600 mmol), thiourea and CH_2Cl_2 (0.6 mL). The solutions were then heated at reflux under N_2 until the reaction was stirred at reflux for 24 h.
Method B for thiouracil **16** catalysis**:** The glycal donor (0.6 mmol) and acceptor (0.5 mmol) were weighed into a round bottomed flask equipped with a stirring bar under N_2 and put under vacuum for 40 minutes. The flask was refilled with N_2 before anhydrous CH_2Cl_2 (0.6 ml) was added to make a 0.8 M solution with the acceptor. After everything was in solution 2-thiouracil (1 mol%) was added to the solution under N_2. The reaction was refluxed under N_2 for 18 h or until TLC or NMR analysis showed the reaction was complete.
Method C for palladium catalysis (According to the procedure in literature[1]). The glycal donor (0.12 mmol), acceptor (0.75 equiv.), $Pd(CH_3CN)_2Cl_2$ (0.25 equiv.) and ligand 2-(di-*tert*-butylphosphaneyl)-1-phenyl-1*H*-pyrrole (0.30 equiv.) were weighed into an oven dried dry tube, sealed and placed under vacuum for 1 h. The vial was then filled with argon and anhydrous CH_2Cl_2 (1.0 mL) was added. The mixture were stirred and heated at 50 ˚C until the reaction was determined to be complete by TLC.
Method D for gold catalysis (According to the procedure in literature[2]). The glycal donor (0.10 mmol) and acceptor (0.08 mmol) were weighed into a dry tube and placed under vacuum for 1 h, after which time the tube was filled with argon. Meanwhile, a suspension mixture was prepared by mixing gold catalyst (2.5 mg) and AgOTf (1.85 mg) in anhydrous CH_2Cl_2 (1 mL). This mixture was stirred for 10 mins in a sonicating bath and then added to the dry tube containing the donor and acceptor. The reaction mixture was stirred at room temperature until the reaction was determined to be complete by TLC of the crude material.
Method E for electron deficient pyridinium salt catalysis (According to the procedure in literature[3]). In a dry vial under argon, the glycal (0.26 mmol, 1.0 equiv.) was dissolved in 5 mL of dry CH_2Cl_2. To this solution, catalyst (0.01 equiv.) was added followed by the acceptor (0.31 mmol) and the vial was closed tightly. The reaction mixture was then stirred at room temperature for 24 h.

Decomposition Experiments (see supplementary table S13-14, supplementary figure S59-60)
Method A: Decomposition experiments performed in CD_2Cl_2: Compound **3al** (27.4 mg, 0.05 mmol) was dissolved in CD_2Cl_2 (0.5 mL) in a HPLC vial, then transferred to an NMR tube. Afterwards, catalyst **A** (0.87 mg, 3 mol%) or HCl (1 M in ether, 3 uL) and mesitylene (10 uL) was also added to the same tube. Then the tube was measured on the NMR spectrometer at different time.
Method B: Decomposition experiments performed in CD_3CN: Compound **3al** (27.4 mg, 0.05 mmol) was dissolved in a mixed solvent CD_3CN (0.4 mL) and CD_2Cl_2 (0.1 mL) in a HPLC vial, then transferred to an NMR tube. Afterwards, the CD_3CN (0.1 mL) solution of catalyst **A** (0.87 mg, 3 mol%) or TsOH•H_2O (0.28 mg, 3 mol%) and mesitylene (10 uL) was also added to the same tube. Then the tube was measured on the NMR spectrometer at different time.

Synthetic methods and characterization data

(5a*R*,6*R*)-2,2,4,4-Tetraisopropyl-6-(((triisopropylsilyl)oxy)methyl)-5a,9a-dihydro-6*H*-pyrano[3,4-*f*][1,3,5,2,4]trioxadisilepine

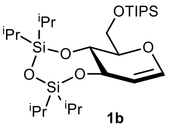

1b

The compound was synthesized according to the literature.[4] D-glucal (5.0 g, 34.2 mmol) and imidazole (4.66 g, 68.4 mmol) were dissolved in anhydrous DMF (40 mL) under an argon atmosphere and the solution was cooled to 0 ˚C. TIPSCl (9.4 mL, 43.8 mmol), was added dropwise, the reaction was allowed to warm to 23 ˚C and stirred for 18 h. The reaction was concentrated in vacuo and the residue was dissolved in CH_2Cl_2 (200 mL), washed with H_2O (100 mL), the aqueous phase was then washed with

CH_2Cl_2 (100 mL) twice and the combined organic phases were washed with brine (200 mL), dried over $MgSO_4$, filtered and concentrated in vacuo to give a crude product (5.3 g).
The above crude product (5.3 g, 17.5 mmol) and imidazole (2.26 g, 33.3 mmol) were dissolved in distilled pyridine (80 mL) under an argon atmosphere and the solution was cooled to 0 ˚C. 1,3-dichloro-1,1,3,3-tetraisopropyldisiloxane (8.4 mL, 26.3 mmol) was added dropwise and the solution was then allowed to warm to 23 ˚C and stirred for 18 h. The reaction was quenched with H_2O (150 mL), extracted with pentane (150 mL) three times. The organic layer was washed with brine (150 mL), dried over $MgSO_4$, filtered and concentrated in vacuo. Following by column chromatography (99:1 to 98:2 pentane/ethyl acetate), the title compound was obtained as a colorless oil (9.2 g, 50% yield for two-steps).
^{1}H NMR (400 MHz, $CDCl_3$) δ 6.34 (dd, *J* = 6.0, 1.8 Hz, 1 H), 4.62 (dd, *J* = 6.0, 2.0 Hz, 1 H), 4.41 (dt, *J* = 6.8, 1.9 Hz, 1 H), 4.12 (dd, *J* = 11.1, 1.9 Hz, 1 H), 3.92 (dd, *J* = 11.1, 5.5 Hz, 1 H), 3.86 (dd, *J* = 10.2, 6.8 Hz, 1 H), 3.79-3.74 (m, 1 H), 1.16-0.85 (m, 49 H) ppm. **^{13}C NMR** (126 MHz, $CDCl_3$) δ 144.12, 102.97, 79.53, 72.83, 72.32, 63.05, 18.12, 18.08, 17.80, 17.60, 17.55, 17.47, 17.46, 17.41, 17.35, 17.30, 13.22, 13.02, 12.66, 12.41, 12.18 ppm. $[\alpha]_D^{20}$ = +22.7 (c = 0.73, CH_2Cl_2). The data is in accordance to the literature.[4]

(((2*R*,3*S*)-2-(((*Tert*-Butyldimethylsilyl)oxy)methyl)-3,4-dihydro-2*H*-pyran-3,4-diyl)bis(oxy))bis(*tert*-butyldimethylsilane)

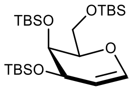

1c

The compound was synthesized according to the literature.[5] D-galactal (516.0 mg, 3.5 mmol), imidazole (1.39 g, 20.5 mmol), TBSCl (3.09 g, 20.5 mmol) and *N*,*N*-dimethylpyridine (418.0 mg, 3.4 mmol) were dissolved in anhydrous DMF (20 mL) under an argon atmosphere. The mixture was heated to 60 ˚C and was left stirring at this temperature for 36 hours. Pentane (50 mL) and crushed ice were added and the layers were separated. The aqueous layer was washed with pentane (30 mL) twice. The organic layers were combined and washed with water (10 mL) twice, brine (10 mL) twice, dried over Na_2SO_4, filtered and concentrated in vacuo. Purification by column chromatography (99:1, pentane/ethyl acetate) afforded the title product as a colorless oil (1.48 g, 86% yield).
^{1}H NMR (400 MHz, $CDCl_3$) δ 6.21 (dd, *J* = 6.1, 0.9 Hz, 1 H), 4.65 (t, *J* = 5.3 Hz, 1 H), 4.11 (s, br, 1 H), 4.03-4.01 (m, 3 H), 3.89-3.82 (m, 1 H), 0.91-0.89 (m, 27 H), 0.10-0.055 (m, 18 H) ppm. **^{13}C NMR** (126 MHz, $CDCl_3$) δ 142.85, 102.75, 79.72, 68.87, 65.06, 61.03, 26.20, 26.13, 26.05, 18.60, 18.41, 18.34, -4.06, -4.23, -4.61, -4.78, -4.98, -5.10 ppm. $[\alpha]_D^{20}$ = -33.1 (c = 0.76, CH_2Cl_2). The data is in accordance to the literature. [5]

(5a*S*,6*S*,9a*S*)-2,2,4,4-Tetraisopropyl-6-methyl-5a,9a-dihydro-6*H*-pyrano[3,4-f][1,3,5,2,4]trioxadisilepine

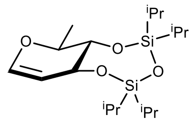

1f

The compound was synthesized according to the literature.[4] L-rhamnal (3.04 g, 23.3 mmol), imidazole (3.19 g, 46.9 mmol) were dissolved in anhydrous pyridine (100 mL) under an argon atmosphere. The solution was cooled to 0 ˚C, and 1,3-dichloro-1,1,3,3-tetraisopropyldisiloxane (11.6 mL, 36.2 mmol) was added dropwise at this temperature. Then the mixture was allowed to warm to 23 ˚C and stirred for 18 h. Afterwards, the solution was quenched with water, and extracted with ethyl acetate (100 mL) three times. The organic layer was washed with saturated $CuSO_4$ to remove pyridine, brine sequentially, dried over $MgSO_4$ and concentrated under vacuo. Following flash column chromatography (96:4 cyclohexane/ethyl acetate) afforded the title compound as a white solid (6.62 g, 76% yield).
^{1}H NMR (500 MHz, $CDCl_3$) δ 6.29 (dd, *J* = 6.0, 1.8 Hz, 1 H), 4.65 (dd, *J* = 6.0, 2.0 Hz, 1 H), 4.36 (dt, *J* = 6.9, 1.9 Hz, 1 H), 3.85-3.78 (m, 1 H), 3.58 (dd, *J* = 9.9, 7.0 Hz, 1 H), 1.37 (d, *J* = 6.3 Hz, 3 H), 1.14-0.91 (m, 28 H) ppm. **^{13}C NMR** (126 MHz, $CDCl_3$) δ 144.18, 103.85, 77.34, 75.10, 72.77, 17.79, 17.75, 17.56, 17.54, 17.46, 17.45, 17.41, 17.40, 17.39, 13.12, 13.03, 12.58, 12.42 ppm. $[\alpha]_D^{20}$ = -3.6 (c = 2.03, CH_2Cl_2). The data is in accordance to the literature. [4]

(5a*S*,6*S*,9a*S*)-2,2,4,4-Tetraisopropyl-6-(((triisopropylsilyl)oxy)methyl)-5a,9a-dihydro-6*H*-pyrano[3,4-*f*][1,3,5,2,4]trioxadisilepine

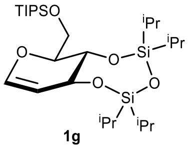

1g

The compound was synthesized according to the literature. [4] L-glucal (1.6 g, 10.9 mmol) and imidazole (1.49 g, 21.9 mmol) were dissolved in anhydrous DMF (25 mL) under an argon atmosphere and the solution was cooled to 0 ˚C. TIPSCl (3.1 mL, 15.3 mmol), was added dropwise, the reaction was allowed to warm to 23 ˚C and stirred for 18 h. The reaction was concentrated in vacuo and the residue was dissolved in CH_2Cl_2 (100 mL), washed with H_2O (100 mL), the aqueous phase was then washed with CH_2Cl_2 (500 mL) twice and the combined organic phases were washed with brine (100 mL), dried over $MgSO_4$, filtered and concentrated in vacuo to give a crude product (2.3 g).
The above crude product (2.3 g, 7.6 mmol) and imidazole (0.98 g, 14.5 mmol) were dissolved in distilled pyridine (30 mL) under an argon atmosphere and the solution was cooled to 0 ˚C. 1,3-dichrolo-1,1,3,3-tetraisopropyldisiloxane (3.7 mL, 11.4 mmol) was added dropwise and the solution was then allowed to warm to 23 ˚C and stirred for 18 h. The reaction was quenched with H_2O (50 mL), extracted with pentane (100 mL) three times. The organic layer was washed with brine (50 mL), dried over $MgSO_4$, filtered and concentrated in vacuo. Following by column chromatography (99:1 to 98:2 pentane/ethyl acetate), the title compound was obtained as a colorless oil (3.8 g, 64% yield for two-steps).
^{1}H NMR (500 MHz, $CDCl_3$) δ 6.34 (dd, *J* = 6.0, 1.9 Hz, 1 H), 4.62 (dd, *J* = 6.0, 2.0 Hz, 1 H), 4.41 (dt, *J* = 6.8, 1.9 Hz, 1 H), 4.12 (dd, *J* = 11.1, 1.9 Hz, 1 H), 3.91 (dd, *J* = 11.1, 5.6 Hz, 1 H), 3.86 (dd, *J* = 10.2, 6.8 Hz, 1 H), 3.77 (ddd, *J* = 10.2, 5.5, 2.0 Hz, 1 H), 1.15-0.94 (m, 49 H) ppm. **^{13}C NMR** (126 MHz, $CDCl_3$) δ 144.12, 102.97, 79.53, 72.83, 72.33, 63.06, 18.12, 18.08, 17.80, 17.60, 17.55, 17.47, 17.46, 17.41, 17.35, 17.30, 13.22, 13.01, 12.66, 12.40, 12.17 ppm. **ESI-pos HRMS**: Calculated for $C_{27}H_{57}O_5Si_3$ $(M+H)^+$: 545.35083, Found: 545.34141. $[\alpha]_D^{20}$ = -23.0 (c = 0.59, CH_2Cl_2).

(((2*S*,3*R*)-2-(((*Tert*-Butyldimethylsilyl)oxy)methyl)-3,4-dihydro-2*H*-pyran-3,4-diyl)bis(oxy))bis(*tert*-butyldimethylsilane)

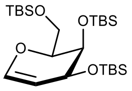

1h

The compound was synthesized according to the literature.[5] L-galactal (332.8 mg, 2.3 mmol), imidazole (899.2 mg, 13.21 mmol), TBSCl (2.0 g, 13.2 mmol) and *N*,*N*-dimethylpyridine (27.8 mg, 0.23 mmol) were dissolved in anhydrous DMF (15 mL) under an argon atmosphere. The mixture was heated to 60 ˚C and was left stirring at this temperature for 36 hours. Pentane (100 mL) and crushed ice were added and the layers were separated. The aqueous layer was washed with pentane (50 mL) twice. The organic layers were combined and washed with water (20 mL) twice, brine (20 mL) twice, dried over Na_2SO_4, filtered and concentrated in vacuo. Purification by column chromatography (99:1, pentane/ethyl acetate) afforded the title product as a colorless oil (640 mg, 57% yield).
^{1}H NMR (400 MHz, CD_2Cl_2) δ 6.21 (dd, *J* = 6.1, 0.9 Hz, 1 H), 4.69-4.66 (m, 1 H), 4.15 (s, br, 1 H), 4.07-3.97 (m, 3 H), 3.90-3.83 (m, 1 H), 0.93-0.90 (m, 27 H), 0.13-0.06 (m, 18 H) ppm. **^{13}C NMR** (101 MHz, CD_2Cl_2) δ 143.22, 103.41, 80.24, 69.33, 65.73, 61.61, 26.44, 26.40, 18.93, 18.83, 18.76, -3.74, -3.99, -4.33, -4.52, -4.79, -4.91 ppm. **ESI-pos HRMS**: Calculated for $C_{24}H_{54}O_5NaSi_3$ $(M+Na+H_2O)^+$: 529.31713, Found: 529.31838. $[\alpha]_D^{20}$ = +33.8 (c = 0.70, CH_2Cl_2).

(5a*R*,9a*R*)-2,2,4,4-Tetraisopropyl-5a,9a-dihydro-6*H*-pyrano[3,4-*f*][1,3,5,2,4]trioxadisilepine

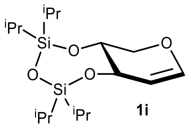

1i

D-xylal (2.0 g, 17.2 mmol), imidazole (9.38 g, 137.8 mmol) were dissolved in anhydrous DMF (150 mL) under an argon atmosphere. The solution was cooled to 0 ˚C and 1,3-dichloro-1,1,3,3-tetraisopropyldisiloxane (5.9 mL, 19.0 mmol) was added dropwise and the solution was then allowed to warm to 23 ˚C and stirred for 5 h. The reaction was quenched with H_2O (160 mL) and pentane (150 mL) was added. The layers were separated and the aqueous layer was washed with pentane (150 mL) twice.

The organic layers were combined and washed with water (100 mL) twice, brine (100 mL), dried over Na_2SO_4, filtered and concentrated in vacuo. Purification by column chromatography (98:2, pentane/ethyl acetate) afforded the title product as a colorless oil (5.4 g, 87% yield).
^{1}H NMR (400 MHz, $CDCl_3$) δ 6.29 (dd, *J* = 5.9, 1.6 Hz, 1 H), 4.65 (dd, *J* = 6.0, 2.0 Hz, 1 H), 4.37 (dt, *J* = 6.9, 1.9 Hz, 1 H), 4.01 (dd, *J* = 10.6, 4.8 Hz, 1 H), 3.94 (ddd, *J* = 11.1, 6.9, 4.8 Hz, 1 H), 3.62 (t, *J* = 10.8 Hz, 1 H), 1.10-0.94 (m, 28 H) ppm. **^{13}C NMR** (126 MHz, $CDCl_3$) δ 144.25, 103.49, 72.79, 71.89, 68.09, 17.76, 17.71, 17.44, 17.41, 17.40, 17.39, 17.32, 17.31, 13.19, 13.12, 12.56, 12.42 ppm. **ESI-pos HRMS**: Calculated for $C_{17}H_{35}O_4Si_2$ $(M+H)^+$: 359.20684, Found: 359.20779. $[\alpha]_D^{20}$ = -21.3 (c = 0.86, CH_2Cl_2).

(6a*R*,9a*R*)-2,2,4,4-Tetraisopropyl-6a,9a-dihydro-6*H*-furo[3,2-*f*][1,3,5,2,4]trioxadisilocine

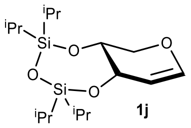

1j

D-ribal (2.38 g, 20.5 mmol) and imidazole (11.2 g, 160.0 mmol) were dissolved in anhydrous DMF (120 mL) under an argon atmosphere and the solution was cooled to 0 ˚C. 1,3-Dichloro-1,1,3,3-tetraisopropyldisiloxane (7.0 mL, 22.5 mmol) was added dropwise and the solution was allowed to warm to 23 ˚C and stirred for 13 h. The reaction was quenched with H_2O (10 mL), then pentane (160 mL) and H_2O (160 mL) was added. The mixture was separated and the aqueous phase was extracted with pentane (100 mL) twice. The organic phases were combined and washed with brine, dried over Na_2SO_4, filtered and concentrated in vacuo. Following purification by flash column chromatography (98:2 pentane/ethyl acetate) afforded the title compound as a colorless oil (5.4 g, 73% yield).
^{1}H NMR (400 MHz, $CDCl_3$) δ 6.39 (d, *J* = 5.9 Hz, 1 H), 4.84 (t, *J* = 5.8 Hz, 1 H), 4.46-4.26 (m, 1 H), 4.12 (dt, *J* = 10.9, 3.9 Hz, 1 H), 3.87 (ddd, *J* = 10.0, 4.1, 1.5 Hz, 1 H), 3.81-3.75 (m, 1 H), 1.12-0.92 (m, 28 H) ppm. **^{13}C NMR** (101 MHz, $CDCl_3$) δ 146.14, 100.84, 70.45, 64.59, 64.50, 17.83, 17.68, 17.63, 17.59, 17.52, 17.33, 17.26, 17.24, 14.29, 13.41, 13.34, 12.80 ppm. **ESI-pos HRMS**: Calculated for $C_{17}H_{35}O_4Si_2$ $(M+H)^+$: 359.20684, Found: 359.20781. $[\alpha]_D^{20}$ = +102.4 (c = 0.93, CH_2Cl_2).

(5a*S*,9a*S*)-2,2,4,4-tetraisopropyl-5a,9a-dihydro-6*H*-pyrano[3,4-*f*][1,3,5,2,4]trioxadisilepine

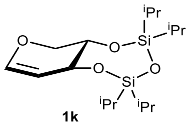

1k

L-xylal (1.74 g, 15.0 mmol), imidazole (8.16 g, 120.0 mmol) were dissolved in anhydrous DMF (60 mL) under an argon atmosphere. The solution was cooled to 0 ˚C and 1,3-dichloro-1,1,3,3-tetraisopropyldisiloxane (5.1 mL, 17.5 mmol) was added dropwise and the solution was then allowed to warm to 23 ˚C and stirred for 5 h. The reaction was quenched with H_2O (160 mL) and pentane (150 mL) was added. The layers were separated and the aqueous layer was washed with pentane (150 mL) twice. The organic layers were combined and washed with water (100 mL) twice, brine (100 mL), dried over Na_2SO_4, filtered and concentrated in vacuo. Purification by column chromatography (98:2, pentane/ethyl acetate) afforded the title product as a colorless oil (5.1 g, 94% yield).
^{1}H NMR (500 MHz, $CDCl_3$) δ 6.30 (dd, *J* = 6.0, 1.8 Hz, 1 H), 4.65 (dd, *J* = 6.0, 2.0 Hz, 1 H), 4.37 (dt, *J* = 7.0, 1.9 Hz, 1 H), 4.01 (dd, *J* = 10.8, 4.8 Hz, 1 H), 3.94 (ddd, *J* = 11.6, 6.9, 4.8 Hz, 1 H), 3.62 (t, *J* = 11.0 Hz, 1 H), 1.10-0.91 (m, 28 H) ppm. **^{13}C NMR** (126 MHz, $CDCl_3$) δ 144.25, 103.49, 72.79, 71.89, 68.09, 17.76, 17.71, 17.44, 17.41, 17.40, 17.39, 17.32, 17.31, 13.19, 13.12, 12.56, 12.42 ppm. **ESI-pos HRMS**: Calculated for $C_{17}H_{35}O_4Si_2$ $(M+H)^+$: 359.20684, Found: 359.20789. $[\alpha]_D^{20}$ = +21.6 (c = 1.35, CH_2Cl_2).

(((2*R*,3*S*)-2-(((*Tert*-Butyldimethylsilyl)oxy)methyl)-3,4-dihydro-2*H*-pyran-3,4-diyl-5-d)bis(oxy))bis(*tert*-butyldimethylsilane)

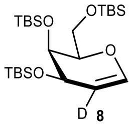

8

The compound was synthesized according to the literature.[4] [2-^{2}H]-D-Galactose (500.0 mg, 2.76 mmol) was dissolved in a solution of pyridine (6 mL), acetic anhydride (3 mL). DMAP (30.0 mg, 0.25 mmol) was added and the solution was stirred at 23 ˚C for 18 h. The mixture was then diluted with CH_2Cl_2 (50 mL), washed with 1 M HCl (20 mL) twice, saturated $NaHCO_3$ aqueous (20 mL) twice, brine (50 mL),

then dried over $MgSO_4$, filtered and concentrated in *vacuo* by azeotroping with toluene to yield the product 1,2,3,4,6-penta-*O*-acetyl-[2-^{2}H]-D-galactopyranoside (1.0 g) which was used for the next step without further purification.
The above product 1,2,3,4,6-penta-*O*-acetyl-[2-^{2}H]-D-galactopyranoside (1.0 g, 2.56 mmol) was dissolved in anhydrous CH_2Cl_2 (8 mL) under argon, the solution was cooled to 0 ˚C and 33% HBr/AcOH (3.6 mL) was added dropwise. After complete addition, the solution was allowed to warm to 23 ˚C and stirred for 1 h, after which the solution was added to ice water (20 mL) and extracted with EtOAc (80 mL) and washed with $NaHCO_3$ (30 mL) twice, water (60 mL), brine (60 mL) and dried over $MgSO_4$. The solution was filtered and concentrated in *vacuo* to 4 mL and added to a solution of zinc dust (1.0 g, 15.8 mL), NaOAc (1.0 g, 12.3 mmol), $CuSO_4$ (25 mg, 0.16 mmol) in 60% AcOH/H_2O (4 mL). After 3 h of stirring at 23 ˚C the reaction mixture was filtered through celite which was rinsed with EtOAc (20 mL). The filtrate was diluted EtOAc (60 mL), washed with $NaHCO_3$, water and brine, dried over $MgSO_4$, filtered and concentrated in *vacuo*. Purification by flash column chromatography (95:5 to 4:1 pentane/ethyl acetate) gives the 2-^{2}H-3,4,6-*tri*-acetyl galactal.
2-^{2}H-3,4,6-*tri*-acetyl galactal (320 mg, 1.17 mmol) was dissolved in MeOH (10 mL) and NaOMe (18 mg, 0.35 mmol) was added. The mixture was stirred at 23 ˚C for 3 h. The solution was concentrated in vacuo and the crude product was then further dried under vacuum. Afterwards, the product was used for next step without further purification.
The 2-^{2}H-galactal (204 mg, 1.39 mmol) was dissolved in anhydrous DMF (10 mL) under an argon atmosphere. Imidazole (547 mg, 8.0 mmol), DMAP (17 mg, 0.14 mmol) and TBSCl (1.21 g, 8.0 mmol) were added and the reaction mixture was heated to 60 ˚C, the solution was left stirring at this temperature for 36 h after which pentane (50 mL) and crushed ice were added and the layers were separated. The aqueous layer was washed with pentane (50 mL) twice. The organic layers were combined and washed with water (10 mL) twice, brine (10 mL) twice and dried over Na_2SO_4, filtered and concentrated in vacuo. Purification by column chromatography (99:1 pentane/ethyl acetate) afforded the product a colorless oil (470 mg, 34% yield for three steps).
^{1}H NMR (500 MHz, $CDCl_3$) δ 6.22 (s, 1 H), 4.11-3.99 (m, 4 H), 3.88-3.83 (m, 1 H), 0.97-0.82 (m, 27 H), 0.13-0.00 (m, 18 H) ppm. **^{13}C NMR** (126 MHz, $CDCl_3$) δ 142.76, 102.49 (m), 79.77, 68.84, 64.87, 61.03, 26.19, 26.11, 26.04, 18.61, 18.40, 18.33, -4.07, -4.24, -4.63, -4.79, -4.99, -5.10 ppm. **ESI-pos HRMS**: Calculated for $C_{24}H_{52}DO_4Si_3$ $(M+H)^+$: 490.33089, Found: 490.33071. $[\alpha]_D^{20}$ = -7.0 (c = 0.28, CH_2Cl_2).

Propan-2-*d*-2-ol
A sample of $NaBD_4$ (2.0 g, 47.6 mmol) was dissolved in 60 mL of 0.1 M NaOH in a 100 mL round-bottom flask in an ice bath. A 14 mL sample of acetone was added dropwise to the solution, at all times keeping the solution temperature below 20 ˚C. When the addition of acetone was complete, 5 M sulfuric acid was added until the pH of the solution had reached between 1 and 2. A crude product was distilled off at 80.5 ˚C. The product was redistilled and its purity checked by 1H NMR spectra (D% >98%, 6.0 g, 52% yield)

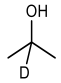

9

^{1}H NMR (400 MHz, $CDCl_3$) δ 4.68 (s, 1 H), 1.10 (s, 9 H) ppm. **^{13}C NMR** (100 MHz, $CDCl_3$) δ 63.70 (t, *J* = 22.2 Hz), 25.04 ppm. The data is accordance to the literature. [8]
Halogen bond catalyst A
To a flame-dried round flask (100 mL), 2-Iodo-1-phenyl-1*H*-benzo[*d*]imidazole (1.0 g, 3.1 mmol) and anhydrous CH_2Cl_2 (25 mL). Then octyl triflate (2.3 g, 8.7 mmol, 2.3 mL) was added to the solution, and the resulting mixture was stirred at 23 ˚C for 12 h. Afterwards, the solvent was removed under reduced pressure, and the residue was rinsed with pentane (10 mL) five times to remove excessive octyl triflate and then recrystallized in CH_3CN/Et_2O to give a white solid (1.3 g, 71%).

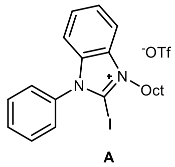

A

^{1}H NMR (700 MHz, CD_2Cl_2) δ 7.83 (d, *J* = 8.4 Hz, 1 H), 7.80-7.74 (m, 3 H), 7.69-7.67 (m, 1 H), 7.60-7.57 (m, 1 H), 7.53-7.52 (m, 2 H), 7.38 (d, *J* = 8.4 Hz, 1 H), 4.61 (t, *J* = 7.8 Hz, 2 H), 2.02 (p, *J* = 7.7 Hz, 2 H), 1.52 (p, *J* = 7.6 Hz, 2 H), 1.42 (p, *J* = 7.1 Hz, 2 H), 1.36-1.28 (m, 6 H), 0.89 (t, *J* = 6.9 Hz, 3 H) ppm. **^{13}C NMR** (176 MHz, CD_2Cl_2) δ 135.64, 134.68, 133.71, 132.57, 131.44, 128.36, 128.12, 128.10,

121.17 (q, J = 320.8 Hz), 114.09, 113.45, 112.91, 51.62, 32.24, 29.72, 29.62, 29.59, 27.27, 23.15, 14.37 ppm. **ESI-HRMS**: Calculated for $C_{21}H_{26}N_2I$ (M-OTf$^-$)$^+$: 433.11352, Found: 433.11349.

Halogen bond catalyst Br-A

To a flame-dried round flask (25 mL), 2-Bromo-1-phenyl-1*H*-benzo[*d*]imidazole (546.2 mg, 2.0 mmol) and anhydrous CH_2Cl_2 (10 mL). Then octyl triflate (786.9 mg, 3.0 mmol, 0.79 mL) was added to the solution, and the resulting mixture was stirred at 23 ˚C for 23 h. Afterwards, the solvent was removed under reduced pressure, and the residue was rinsed with pentane (10 mL) five times to remove excessive octyl triflate and then recrystallized in CH_3CN/Et_2O to give a white solid (676.7 mg, 63%).

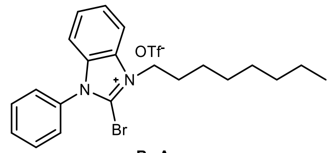

Br-A

^{1}H NMR (500 MHz, CD_2Cl_2) δ 7.87 (d, J = 8.4 Hz, 1 H), 7.79-7.72 (m, 4 H), 7.67-7.61 (m, 3 H), 7.41 (d, J = 8.4 Hz, 1 H), 4.64 (t, J = 8.0 Hz, 2 H), 2.03 (p, J = 7.7 Hz, 2H), 1.51 (p, J = 7.1 Hz, 2 H), 1.54-1.47 (m, 2 H), 1.44-1.38 (m, 2 H), 1.36-1.26 (m, 6 H), 0.89 (t, J = 6.9 Hz, 3 H) ppm. ^{13}C NMR (126 MHz, CD_2Cl_2) δ 134.24, 132.80, 132.59, 132.52, 131.88, 131.34, 128.61, 128.43, 127.83, 121.28 (q, J = 319.2 Hz), 113.84, 113.61, 49.79, 32.21, 29.55, 29.54, 29.28, 27.22, 23.12, 14.36 ppm. **ESI-HRMS**: Calculated for $C_{21}H_{26}N_2Br$ (M-OTf$^-$)$^+$: 385.1279, Found: 385.1275.

(5a*R*,6*R*,8*S*,9a*R*)-2,2,4,4-Tetraisopropyl-8-(((3a*R*,5*R*,5a*S*,8a*S*,8b*R*)-2,2,7,7-tetramethyltetrahydro-5*H*-bis([1,3]dioxolo)[4,5-*b*:4',5'-*d*]pyran-5-yl)methoxy)-6-(((triisopropylsilyl)oxy)methyl)tetrahydro-6*H*-pyrano[3,4-*f*][1,3,5,2,4]trioxadisilepine

The title product compound is prepared according to the general procedure with 3 mol% catalyst **A** and 0.3 mmol glycal at 40 ˚C for 48 h and isolated by flash column chromatography (20:1 Pentane: Ethyl Acetate) giving a pale yellow syrup (144 mg, 0.18 mmol, 89% yield, α/β ratio >20:1).

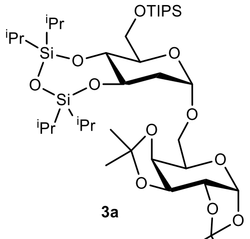

3a

^{1}H NMR (500 MHz, $CDCl_3$) δ 5.50 (d, J = 5.0 Hz, 1 H), 4.94 (d, J = 3.5 Hz, 1 H), 4.59 (dd, J = 8.0, 2.3 Hz, 1 H), 4.29 (dd, J = 5.0, 2.3 Hz, 1 H), 4.19 (dd, J = 7.9, 1.9 Hz, 1 H), 4.05-4.00 (m, 2 H), 3.98-3.95 (m, 1 H), 3.82-3.74 (m, 2 H), 3.64 (dd, J = 10.7, 6.2 Hz, 1 H), 3.59-3.55 (m, 1 H), 3.50-3.46 (m, 1 H), 2.09 (dd, J = 13.3, 5.4 Hz, 1 H), 1.68-1.62 (m, 1 H), 1.50 (s, 3 H), 1.42 (s, 3 H), 1.31 (s, 6 H), 1.12-0.85 (m, 49 H) ppm. **^{13}C NMR** (126 MHz, $CDCl_3$) δ 109.32, 108.54, 96.48, 96.08, 74.71, 73.50, 71.77, 71.20, 70.82, 70.79, 65.63, 64.60, 63.41, 38.13, 26.14, 26.06, 25.07, 24.52, 18.13, 18.10, 17.73, 17.54 (two carbons), 17.51, 17.44, 17.41, 17.39, 17.38, 13.12, 12.95, 12.45, 12.40, 12.12 ppm. **ESI-HRMS**: Calculated for $C_{39}H_{76}O_{11}NaSi_3$ (M+Na)$^+$: 827.45876, Found: 827.46142. $[\alpha]_D^{20}$ = +25.3 (c = 0.58, CH_2Cl_2).

(2*S*,3*R*,4*S*,5*R*,6*R*)-2-Methoxy-6-((((5a*R*,6*R*,8*S*,9a*R*)-2,2,4,4-tetraisopropyl-6-(((triisopropylsilyl)oxy)methyl)tetrahydro-6*H*-pyrano[3,4-*f*][1,3,5,2,4]trioxadisilepin-8-yl)oxy)methyl)tetrahydro-2*H*-pyran-3,4,5-triyl tribenzoate

The title product compound is prepared according to the general procedure with 3 mol% catalyst **A** and 0.3 mmol glycal at 40 ˚C for 48 h and isolated by flash column chromatography (20:1 Pentane: Ethyl Acetate) giving a pale yellow syrup (162 mg, 0.15 mmol, 77% yield, α/β ratio >20:1).

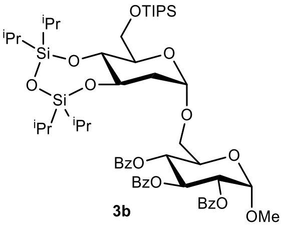

3b

^{1}H NMR (500 MHz, $CDCl_3$) δ 8.00 (d, *J* = 7.6 Hz, 2 H), 7.95 (d, *J* = 7.6 Hz, 2 H), 7.88 (d, *J* = 7.7 Hz, 2 H), 7.52-7.48 (m, 2 H), 7.43-7.34 (m, 5 H), 7.30-7.26 (m, 2 H), 6.15 (t, *J* = 9.7 Hz, 1 H), 5.54 (t, *J* = 9.9 Hz, 1 H), 5.25-5.21 (m, 2 H), 4.90 (d, *J* = 3.3 Hz, 1 H), 4.25-4.22 (m, 1 H), 4.09-4.04 (m, 1 H), 3.96-3.91 (m, 2 H), 3.74 (dd, *J* = 10.8, 6.2 Hz, 1 H), 3.62-3.58 (m, 1 H), 3.55 (dd, *J* = 10.9, 2.8 Hz, 1 H), 3.48-3.44 (m, 1 H), 3.46 (s, 3 H), 2.01 (dd, *J* = 13.3, 5.2 Hz, 1 H), 1.67-1.61 (m, 1 H), 1.12-0.90 (m, 49 H) ppm. **^{13}C NMR** (126 MHz, $CDCl_3$) δ 165.99, 165.93, 165.19, 133.45, 133.33, 133.15, 130.06, 129.93, 129.80, 129.43, 129.27, 129.23, 128.53, 128.49, 128.36, 96.92, 96.80, 74.70, 73.49, 72.39, 71.65, 70.76, 69.89, 68.59, 65.40, 63.44, 55.26, 38.03, 18.12, 18.07, 17.74, 17.54 (two carbons), 17.50, 17.46, 17.43, 17.42 (two carbons), 13.09, 13.00, 12.45, 12.33, 12.09 ppm. **ESI-HRMS**: Calculated for $C_{55}H_{82}O_{14}NaSi_3$ $(M+Na)^+$: 1073.49046, Found: 1073.49309. $[\alpha]_D^{20}$ = +60.0 (c = 0.55, CH_2Cl_2).

(5a*R*,6*R*,8*S*,9a*R*)-2,2,4,4-Tetraisopropyl-8-(((3a*R*,4*R*,6*R*,6a*R*)-6-methoxy-2,2-dimethyltetrahydrofuro[3,4-*d*][1,3]dioxol-4-yl)methoxy)-6-(((triisopropylsilyl)oxy)methyl)tetrahydro-6*H*-pyrano[3,4-*f*][1,3,5,2,4]trioxadisilepine

The title product compound is prepared according to the general procedure with 5 mol% catalyst **A** and 0.3 mmol glycal at 50 ˚C for 48 h and isolated by flash column chromatography (20:1 Pentane: Ethyl Acetate) giving a pale yellow syrup (106 mg, 0.14 mmol, 71% yield, α/β ratio >20:1).

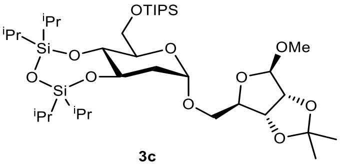

3c

^{1}H NMR (500 MHz, $CDCl_3$) δ 4.93 (s, 1 H), 4.87 (d, *J* = 3.2 Hz, 1 H), 4.66 (d, *J* = 6.0 Hz, 1 H), 4.56 (d, *J* = 6.0 Hz, 1 H), 4.33 (dd, *J* = 8.8, 5.6 Hz, 1 H), 4.08-4.06 (m, 1 H), 4.05-3.99 (m, 1 H), 3.77-3.71 (m, 2 H), 3.59-3.56 (m, 1 H), 3.41 (t, *J* = 8.9 Hz, 1 H), 3.34-3.30 (m, 1 H), 3.27 (s, 3 H), 2.11 (dd, *J* = 13.3, 5.5 Hz, 1 H), 1.69-1.63 (m, 1 H), 1.48 (s, 3 H), 1.31 (s, 3 H), 1.13-0.91(m, 49 H) ppm. **^{13}C NMR** (126 MHz, $CDCl_3$) δ 112.31, 109.54, 97.57, 85.49, 84.94, 82.45, 74.89, 73.65, 71.79, 67.88, 63.72, 54.88, 38.10, 26.54, 25.05, 18.12, 18.10, 17.76, 17.53, 17.51, 17.50, 17.46, 17.40, 17.38 (two carbons), 13.07, 12.97, 12.41, 12.34, 12.09 ppm. **ESI-HRMS**: Calculated for $C_{36}H_{72}O_{10}NaSi_3$ $(M+Na)^+$: 771.43255, Found: 771.43232. $[\alpha]_D^{20}$ = +23.7 (c = 0.31, CH_2Cl_2).

(5a*R*,6*R*,8*R*,9a*R*)-2,2,4,4-Tetraisopropyl-8-(((3a*R*,4*R*,6*S*,7*S*,7a*R*)-4-methoxy-2,2,6-trimethyltetrahydro-4*H*-[1,3]dioxolo[4,5-*c*]pyran-7-yl)oxy)-6-(((triisopropylsilyl)oxy)methyl)tetrahydro-6*H*-pyrano[3,4-*f*][1,3,5,2,4]trioxadisilepine

The title product compound is prepared according to the general procedure with 3 mol% catalyst **A** and 0.3 mmol at 40 ˚C for 48 h and isolated by flash column chromatography (20:1 Pentane: Ethyl Acetate) giving a pale yellow syrup (108 mg, 0.14 mmol, 71% yield, α/β ratio > 20:1).

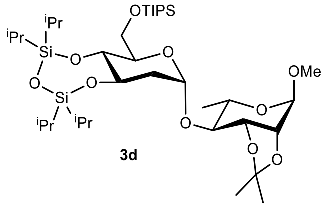

3d

^{1}H NMR (500 MHz, $CDCl_3$) δ 5.04 (d, *J* = 2.7 Hz, 1 H), 4.83 (s, 1 H), 4.18-4.16 (m, 1 H), 4.06 (d, *J* = 5.6 Hz, 1 H), 4.00-3.97 (m, 2 H), 3.89 (d, *J* = 10.5 Hz, 1 H), 3.75-3.69 (m, 2 H), 3.64-3.61 (m, 1 H), 3.36 (s, 3 H), 3.33 (dd, *J* = 9.8, 7.2 Hz, 1 H), 2.03 (dd, *J* = 13.0, 5.0 Hz, 1 H), 1.65 (td, *J* = 13.0, 3.5 Hz, 1 H), 1.51 (s, 3 H), 1.32 (s, 3 H), 1.26 (d, *J* = 6.4 Hz, 3 H), 1.10-0.93 (m, 49 H) ppm. **^{13}C NMR** (126 MHz, $CDCl_3$) δ 108.99, 98.24, 97.96, 80.36, 77.27, 75.89, 73.87, 73.50, 71.65, 64.91, 62.34, 54.93 (two carbons), 38.55, 28.28, 26.54, 18.20, 18.10, 17.84, 17.76, 17.71, 17.59, 17.52, 17.48 (three carbons), 13.24, 13.05, 12.64, 12.60, 12.22 ppm. **ESI-HRMS**: Calculated for $C_{37}H_{74}O_{10}NaSi_3$ $(M+Na)^+$: 785.44820, Found: 785.45009. $[\alpha]_D^{20}$ = +54.5 (c = 0.31, CH_2Cl_2).

(5a*R*,6*R*,8*R*,9a*R*)-8-(((2*R*,3*R*,4*S*,5*R*,6*S*)-4,5-Bis(benzyloxy)-2-((benzyloxy)methyl)-6-methoxytetrahydro-2*H*-pyran-3-yl)oxy)-2,2,4,4-tetraisopropyl-6-(((triisopropylsilyl)oxy)methyl)tetrahydro-6*H*-pyrano[3,4-*f*][1,3,5,2,4]trioxadisilepine

The title product compound is prepared according to the general procedure with 5 mol% catalyst **A** and 0.3 mmol at 50 ˚C for 48 h and isolated by flash column chromatography (20:1 Pentane: Ethyl Acetate) giving a pale yellow syrup (161 mg, 0.16 mmol, 80% yield, α/β ratio >20:1).

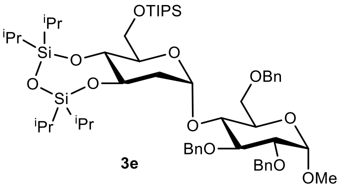

3e

^{1}H NMR (500 MHz, $CDCl_3$) δ 7.34-7.26 (m, 15 H), 5.35 (s, 1 H), 5.01-4.97 (m, 1 H), 4.74-4.70 (m, 1 H), 4.70-4.67 (m, 2 H), 4.64-4.58 (m, 2 H), 4.50-4.46 (m, 1 H), 4.05-4.02 (m, 1 H), 3.88-3.86 (m, 2 H), 3.82-3.77 (m, 4 H), 3.71-3.68 (m, 1 H), 3.59-3.54 (m, 3 H), 3.41-3.39 (m, 3 H), 2.06-2.02 (m, 1 H), 1.61-1.54 (m, 1 H), 1.13-0.96 (m, 49 H) ppm. **^{13}C NMR** (126 MHz, CD_2Cl_2) δ 139.37, 139.27, 138.94, 128.90, 128.82, 128.80, 128.45, 128.29, 128.20, 127.98, 127.90, 127.78, 99.68, 98.11, 82.43, 80.95, 76.58, 75.62, 74.81, 74.71, 73.61, 73.35, 72.09, 70.53 (two carbons), 63.56, 55.51, 39.23, 18.40, 18.35, 18.07, 17.88 (two carbons), 17.73 (four carbons), 17.65, 13.60, 13.50, 12.97, 12.88, 12.60 ppm. **ESI-HRMS**: Calculated for $C_{55}H_{88}O_{11}NaSi_3$ $(M+Na)^+$: 1031.55266, Found: 1031.55309. $[\alpha]_D^{20}$ = +61.1 (c = 0.38, CH_2Cl_2).

(5a*R*,6*R*,8*R*,9a*R*)-8-(((2*S*,6*S*,7*R*,8*S*)-7-(Benzyloxy)-6-methoxy-2-phenylhexahydropyrano[3,2-*d*][1,3]dioxin-8-yl)oxy)-2,2,4,4-tetraisopropyl-6-(((triisopropylsilyl)oxy)methyl)tetrahydro-6*H*-pyrano[3,4-*f*][1,3,5,2,4]trioxadisilepine

The title product compound is prepared according to the general procedure with 3 mol% catalyst **A** and 0.3 mmol at 40 ˚C for 48 h and isolated by flash column chromatography (20:1 Pentane: Ethyl Acetate) giving a pale yellow syrup (141 mg, 0.156 mmol, 78% yield, α/β ratio >20:1).

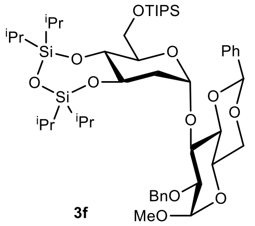

3f

^{1}H NMR (500 MHz, $CDCl_3$) δ 7.47-7.45 (m, 2 H), 7.38-7.37 (m, 8 H), 5.52-5.51 (m, 2 H), 4.91 (d, *J* = 12.5 Hz, 1 H), 4.60 (d, *J* = 12.4 Hz, 1 H), 4.45 (d, *J* = 3.7 Hz, 1 H), 4.34 (t, *J* = 9.3 Hz, 1 H), 4.25-4.17 (m, 2 H), 3.94 (d, *J* = 2.6 Hz, 2 H), 3.89-3.86 (m, 1 H), 3.84-3.76 (m, 2 H), 3.69 (t, *J* = 10.3 Hz, 1 H), 3.59 (t, *J* = 9.3 Hz, 1 H), 3.43 (dd, *J* = 9.6, 3.8 Hz, 1 H), 3.38 (s, 3 H), 2.13 (dd, *J* = 13.3, 5.1 Hz, 1 H), 1.70-1.64 (m, 1 H), 1.14-0.97 (m, 49 H) ppm. **^{13}C NMR** (126 MHz, $CDCl_3$) δ 138.39, 137.43, 129.08, 128.63, 128.37, 128.30, 128.11, 126.14, 101.25, 99.57, 96.68, 83.44, 77.64, 74.33, 73.74, 72.98, 71.93, 71.88, 69.22, 62.66, 61.87, 55.35, 38.21, 18.18, 18.08, 17.96, 17.64, 17.61, 17.58, 17.55, 17.46, 17.44, 17.43, 13.20, 13.17, 12.55, 12.27, 12.22 ppm. **ESI-HRMS**: Calculated for $C_{48}H_{80}O_{11}NaSi_3$ $(M+Na)^+$: 939.49006, Found: 939.49343. $[\alpha]_D^{20}$ = +44.3 (c = 0.23, CH_2Cl_2).

(5a*R*,6*R*,8*R*,9a*R*)-8-(((2*S*,6*S*,7*R*,8*S*)-8-(Benzyloxy)-6-methoxy-2-phenylhexahydropyrano[3,2-*d*][1,3]dioxin-7-yl)oxy)-2,2,4,4-tetraisopropyl-6-(((triisopropylsilyl)oxy)methyl)tetrahydro-6*H*-pyrano[3,4-*f*][1,3,5,2,4]trioxadisilepine

The title product compound is prepared according to the general procedure with 3 mol% catalyst **A** and 0.3 mmol glycal at 40 ˚C for 48 h and isolated by flash column chromatography (20:1 Pentane: Ethyl Acetate) giving a pale yellow syrup (135 mg, 0.15 mmol, 75% yield, α/β ratio >20:1).

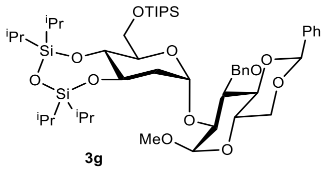

3g

^{1}H NMR (500 MHz, $CDCl_3$) δ 7.49-7.47 (m, 2 H), 7.39-7.34 (m, 5 H), 7.26-7.23 (m, 3 H), 5.54 (s, 1 H), 5.10 (d, *J* = 3.5 Hz, 1 H), 5.00 (d, *J* = 3.4 Hz, 1 H), 4.89 (d, *J* = 11.6 Hz, 1 H), 4.79 (d, *J* = 11.7 Hz, 1 H), 4.30 (dd, *J* = 10.1, 4.7 Hz, 1 H), 4.18-4.12 (m, 1 H), 4.05 (d, *J* = 9.5 Hz, 1 H), 3.99 (dd, *J* = 9.5, 3.5 Hz, 1 H), 3.93 (t, *J* = 9.2 Hz, 1 H), 3.86-3.77 (m, 3 H), 3.72 (t, *J* = 10.2 Hz, 1 H), 3.62 (t, *J* = 9.3 Hz, 1 H), 3.52 (t, *J* = 8.8 Hz, 1 H), 3.44 (s, 3 H), 2.24 (dd, *J* = 13.5, 5.4 Hz, 1 H), 1.77-1.72 (m, 1 H), 1.16-0.88 (m, 49 H) ppm. **^{13}C NMR** (126 MHz, $CDCl_3$) δ 138.92, 137.52, 128.98, 128.28, 128.25, 127.70, 127.39, 126.20, 101.42, 97.29, 92.88, 81.47, 77.27, 75.22, 74.59, 73.70, 73.44, 71.84, 69.20, 63.49, 62.61, 55.24, 37.94, 18.22, 18.18, 17.62, 17.58, 17.56, 17.51, 17.46, 17.44, 17.41 (two carbons), 13.02 (two

carbons), 12.39, 12.35, 12.12 ppm. **ESI-HRMS**: Calculated for $C_{48}H_{80}O_{11}NaSi_3$ $(M+Na)^+$: 939.49006, Found: 939.49148. $[\alpha]_D^{20}$ = +34.8 (c = 0.06, CH_2Cl_2).

(5a*R*,6*R*,8*R*,9a*R*)-8-(((3a*R*,5*R*,6*S*,6a*R*)-5-((*S*)-2,2-Dimethyl-1,3-dioxolan-4-yl)-2,2-dimethyltetrahydrofuro[2,3-*d*][1,3]dioxol-6-yl)oxy)-2,2,4,4-tetraisopropyl-6-(((triisopropylsilyl)oxy)methyl)tetrahydro-6*H*-pyrano[3,4-*f*][1,3,5,2,4]trioxadisilepine

The title product compound is prepared according to the general procedure with 5 mol% catalyst **A** and 0.3 mmol glycal at 50 ˚C for 48 h and isolated by flash column chromatography (20:1 Pentane: Ethyl Acetate) giving a pale yellow syrup (116 mg, 0.14 mmol, 72 % yield, α/β ratio 79:21).

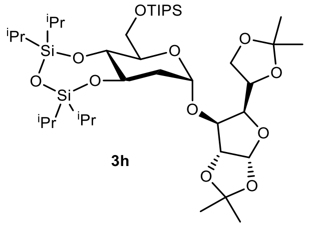

3h

^{1}H NMR (500 MHz, $CDCl_3$) δ 5.87 (d, *J* = 3.0 Hz, 1 H), 5.14 (d, *J* = 3.3 Hz, 1 H), 4.66 (d, *J* = 3.5 Hz, 1 H), 4.24 (d, *J* = 2.9 Hz, 1 H), 4.20-4.16 (m, 1 H), 4.12-4.07 (m, 3 H), 3.98-3.94 (m, 2 H), 3.78 (dd, *J* = 10.6, 6.9 Hz, 1 H), 3.63-3.60 (m, 1 H), 3.40 (t, *J* = 8.9 Hz, 1 H), 2.09 (dd, *J* = 13.3, 5.3 Hz, 1 H), 1.70-1.64 (m, 1 H), 1.47 (s, 3 H), 1.40 (s, 3 H), 1.31 (s, 3 H), 1.28 (s, 3 H), 1.09-0.90 (m, 49 H) ppm. **^{13}C NMR** (126 MHz, $CDCl_3$) δ 112.01, 109.21, 105.48, 98.80, 83.91, 81.63, 80.85, 74.98, 74.53, 72.69, 71.38, 67.83, 63.95, 38.20, 27.18, 26.91, 26.51, 25.42, 18.15, 18.14, 17.68, 17.55, 17.52, 17.49, 17.43 (two carbons), 17.39 (two carbons), 13.06, 13.00, 12.43, 12.40, 12.08 ppm. **ESI-HRMS**: Calculated for $C_{39}H_{77}O_{11}Si_3$ $(M+H)^+$: 805.47682, Found: 805.47765. $[\alpha]_D^{20}$ = +42.1 (c = 0.39, CH_2Cl_2).

(5a*S*,6*S*,8*R*,9a*S*)-2,2,4,4-Tetraisopropyl-8-(((3a*R*,5*R*,5a*S*,8a*S*,8b*R*)-2,2,7,7-tetramethyltetrahydro-5*H*-bis([1,3]dioxolo)[4,5-*b*:4',5'-*d*]pyran-5-yl)methoxy)-6-(((triisopropylsilyl)oxy)methyl)tetrahydro-6*H*-pyrano[3,4-*f*][1,3,5,2,4]trioxadisilepine

The title product compound is prepared according to the general procedure with 3 mol% catalyst **A** and 0.3 mmol at 40 ˚C for 48 h and isolated by flash column chromatography (20:1 Pentane: Ethyl Acetate) giving a pale yellow syrup (130 mg, 0.16 mmol, 81% yield, α/β ratio >20:1).

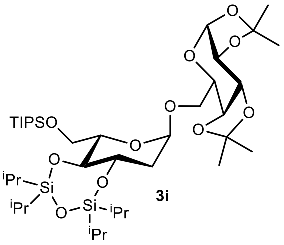

3i

^{1}H NMR (500 MHz, $CDCl_3$) δ 5.52 (d, *J* = 5.0 Hz, 1 H), 4.92 (d, *J* = 2.9 Hz, 1 H), 4.57 (dd, *J* = 7.9, 2.3 Hz, 1 H), 4.28 (dd, *J* = 4.9, 2.3 Hz, 1 H), 4.19 (dd, *J* = 7.9, 1.7 Hz, 1 H), 4.06-4.00 (m, 2 H), 3.98-3.95 (m, 1 H), 3.83-3.79 (m, 2 H), 3.59-3.56 (m, 1 H), 3.54-3.46 (m, 2 H), 2.12 (dd, *J* = 13.1, 5.2 Hz, 1 H), 1.67-1.61 (m, 1 H), 1.51 (s, 3 H), 1.41 (s, 3 H), 1.32 (s, 3 H), 1.29 (s, 3 H), 1.11-0.87 (m, 49 H) ppm. **^{13}C NMR** (126 MHz, $CDCl_3$) δ 109.30, 108.66, 96.99, 96.34, 74.64, 73.52, 71.91, 71.41, 70.83, 70.73, 67.54, 65.37, 63.43, 38.06, 26.16, 26.04, 25.17, 24.27, 18.14, 18.11, 17.78, 17.53 (two carbons), 17.50, 17.49, 17.42, 17.41, 17.38, 13.13, 12.98, 12.49, 12.32, 12.13 ppm. **ESI-pos HRMS**: Calculated for $C_{39}H_{76}O_{11}NaSi_3$ $(M+Na)^+$: 827.45976, Found: 827.46046. $[\alpha]_D^{20}$ = -66.9 (c = 0.46, CH_2Cl_2).

(5a*S*,6*S*,8*S*,9a*S*)-8-(((2*R*,4a*R*,6*S*,7*R*,8*S*,8a*R*)-7-(Benzyloxy)-6-methoxy-2-phenylhexahydropyrano[3,2-*d*][1,3]dioxin-8-yl)oxy)-2,2,4,4-tetraisopropyl-6-(((triisopropylsilyl)oxy)methyl)tetrahydro-6*H*-pyrano[3,4-*f*][1,3,5,2,4]trioxadisilepine

The title product compound is prepared according to the general procedure with 3 mol% catalyst **A** and 0.3 mmol at 40 ˚C for 48 h and isolated by flash column chromatography (20:1 Pentane: Ethyl Acetate) giving a pale yellow syrup (134 mg, 0.15 mmol, 73% yield, α/β ratio >20:1).

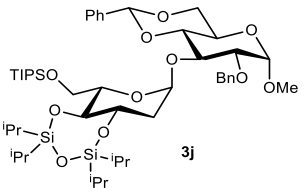

3j

^{1}H NMR (500 MHz, $CDCl_3$) δ 7.43-7.31 (m, 10 H), 5.47 (s, 1 H), 5.21 (s, 1 H), 4.73 (d, *J* = 12.2 Hz, 1 H), 4.64 (d, *J* = 12.2 Hz, 1 H), 4.59-4.58 (m, 1 H), 4.25 (dd, *J* = 10.1, 4.7 Hz, 1 H), 4.09-4.05 (m, 1 H), 4.00-3.95 (m, 1 H), 3.84-3.79 (m, 1 H), 3.71-3.64 (m, 3 H), 3.53-3.46 (m, 2 H), 3.42 (s, 3 H), 3.32 (d, *J* = 11.3 Hz, 1 H), 3.19 (d, *J* = 11.1 Hz, 1 H), 2.07-2.02 (m, 1 H), 1.67-1.62 (m, 1 H), 1.12-0.85 (m, 49 H) ppm. **^{13}C NMR** (126 MHz, $CDCl_3$) δ 137.90, 137.43, 129.14, 128.67, 128.33, 128.25, 128.24, 126.42, 102.19, 98.88, 98.24, 80.34, 80.25, 74.25, 73.43, 73.39, 72.11, 72.09, 69.19, 62.71, 61.36, 55.47, 38.41, 18.16, 18.08, 17.89, 17.62 (two carbons), 17.60, 17.52 (two carbons), 17.50, 17.43, 13.21, 13.09, 12.60, 12.40, 12.16 ppm. **ESI-HRMS**: Calculated for $C_{48}H_{81}O_{11}Si_3$ $(M+H)^+$: 917.50812, Found: 917.50923. $[\alpha]_D^{20}$ = -29.8 (c = 0.33, CH_2Cl_2).

(8*R*,9*S*,10*R*,13*S*,14*S*,17*S*)-10,13-Dimethyl-17-(((5a*R*,6*R*,8*S*,9a*R*)-2,2,4,4-tetraisopropyl-6-(((triisopropylsilyl)oxy)methyl)tetrahydro-6*H*-pyrano[3,4-*f*][1,3,5,2,4]trioxadisilepin-8-yl)oxy)-1,2,6,7,8,9,10,11,12,13,14,15,16,17-tetradecahydro-3*H*-cyclopenta[*a*]16henanthrene-3-one

The title product compound is prepared according to the general procedure with 3 mol% catalyst **A** and 0.3 mmol glycal at 40 ˚C for 48 h and isolated by flash column chromatography (20:1 Pentane: Ethyl Acetate) giving a white foam (113 mg, 0.14 mmol, 68% yield, α/β ratio >20:1).

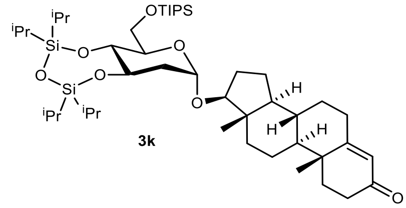

3k

^{1}H NMR (500 MHz, $CDCl_3$) δ 5.72 (s, 1 H), 4.88 (d, *J* = 2.6 Hz, 1 H), 4.10-4.02 (m, 2 H), 3.74 (dd, *J* = 10.6, 6.9 Hz, 1 H), 3.67 (t, *J* = 8.2 Hz, 1 H), 3.61-3.58 (m, 1 H), 3.37 (t, *J* = 8.9 Hz, 1 H), 2.45-2.31 (m, 3 H), 2.27-2.24 (m, 1 H), 2.03-1.98 (m, 3 H), 1.85-1.82 (m, 2 H), 1.69-1.61 (m, 3 H), 1.59-1.53 (m, 2 H), 1.46-1.38 (m, 2 H), 1.35-1.26 (m, 1 H), 1.18 (s, 3 H), 1.12-0.84 (m, 53 H), 0.78 (s, 3 H) ppm. **^{13}C NMR** (126 MHz, $CDCl_3$) δ 199.71, 171.48, 123.96, 94.44, 82.16, 75.12, 73.71, 71.69, 64.05, 54.33, 50.44, 42.35, 38.74, 38.55, 37.00, 35.86, 35.45, 34.09, 32.94, 31.73, 26.47, 23.54, 20.75, 18.15, 18.14, 17.67, 17.55, 17.49 (two carbons), 17.45, 17.42, 17.41 (two carbons), 17.36, 13.11, 12.99, 12.49, 12.45, 12.08, 11.74 ppm. **ESI-HRMS**: Calculated for $C_{46}H_{84}O_7NaSi_3$ $(M+Na)^+$: 855.54171, Found: 855.54171. $[\alpha]_D^{20}$ = +99.5 (c = 0.19, CH_2Cl_2).

(8*R*,9*S*,10*R*,13*S*,14*S*,17*S*)-10,13-Dimethyl-17-(((5a*S*,6*S*,8*R*,9a*S*)-2,2,4,4-tetraisopropyl-6-(((triisopropylsilyl)oxy)methyl)tetrahydro-6*H*-pyrano[3,4-*f*][1,3,5,2,4]trioxadisilepin-8-yl)oxy)-1,2,6,7,8,9,10,11,12,13,14,15,16,17-tetradecahydro-3*H*-cyclopenta[*a*]16henanthrene-3-one

The title product compound is prepared according to the general procedure with 3 mol% catalyst **A** and 0.3 mmol glycal at 40 ˚C for 48 h and isolated by flash column chromatography (20:1 Pentane: Ethyl Acetate) giving a white foam (112 mg, 0.13 mmol, 67% yield, α/β ratio 91:9).

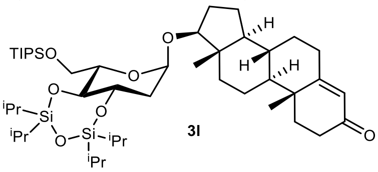

3l

^{1}H NMR (500 MHz, $CDCl_3$) δ 5.71 (s, 1 H), 4.93 (d, *J* = 3.1 Hz, 1 H), 4.05 (dd, *J* = 10.7, 1.7 Hz, 1 H), 4.03-3.99 (m, 1 H), 3.75 (dd, *J* = 10.7, 6.9 Hz, 1 H), 3.64-3.60 (m, 1 H), 3.57 (t, *J* = 8.5 Hz, 1 H), 3.40-3.36 (m, 1 H), 2.41-2.23 (m, 4 H), 2.08-1.98 (m, 3 H), 1.84-1.80 (m, 2 H), 1.72-1.49 (m, 7 H), 1.46-1.37 (m, 1 H), 1.27-1.23 (m, 1 H), 1.18 (s, 3 H), 1.18-1.16 (s, 1 H), 1.11-0.88 (m, 51 H), 0.78 (s, 3 H) ppm. **^{13}C NMR** (126 MHz, $CDCl_3$) δ 199.71, 171.50, 123.96, 97.59, 86.52, 75.14, 73.82, 71.73, 63.97, 54.04, 50.42, 42.90, 38.77, 38.66, 37.37, 35.83, 35.59, 34.08, 32.94, 31.66, 28.63, 23.40, 20.76, 18.13, 18.12, 17.65, 17.52 (two carbons), 17.51, 17.50, 17.42 (four carbons), 13.10, 13.01, 12.49, 12.44, 12.10, 11.71 ppm. **ESI-HRMS**: Calculated for $C_{46}H_{85}O_7Si_3$ $(M+H)^+$: 833.55976, Found: 833.56013. $[\alpha]_D^{20}$ = -20.0 (c = 0.33, CH_2Cl_2).

(5a*R*,6*R*,8*S*,9a*R*)-8-(((3*S*,8*S*,9*S*,10*R*,13*R*,14*S*,17*R*)-10,13-Dimethyl-17-(l-6-methylheptan-2-yl)-2,3,4,7,8,9,10,11,12,13,14,15,16,17-tetradecahydro-1*H*-cyclopenta[*a*] 17 henanthrene-3-yl)oxy)-2,2,4,4-tetraisopropyl-6-(((triisopropylsilyl)oxy)methyl)tetrahydro-6*H*-pyrano[3,4-*f*][1,3,5,2,4]trioxadisilepine

The title product compound is prepared according to the general procedure with 3 mol% catalyst **A** and 0.3 mmol glycal at 40 ˚C for 48 h and isolated by flash column chromatography (3:1 Pentane: CH_2Cl_2) giving a pale yellow syrup (119 mg, 0.12 mmol, 62% yield, α/β ratio >20:1).

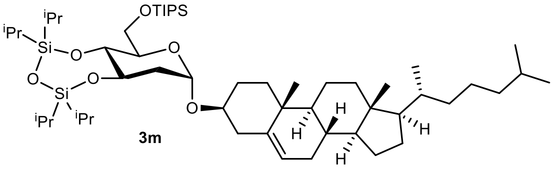

3m

¹H NMR (500 MHz, $CDCl_3$) δ 5.31 (d, *J* = 4.4 Hz, 1 H), 5.09 (d, *J* = 3.1 Hz, 1 H), 4.11 (d, *J* = 9.6 Hz, 1 H), 4.07-4.02 (m, 1 H), 3.73 (dd, *J* = 10.6, 7.6 Hz, 1 H), 3.67-3.64 (m, 1 H), 3.59-3.53 (m, 1 H), 3.37 (t, *J* = 8.9 Hz, 1 H), 2.31-2.29 (m, 2 H), 2.03-1.91 (m, 4 H), 1.84-1.81 (m, 2 H), 1.71-1.23 (m, 12 H), 1.19-0.91 (m, 62 H), 0.92 (d, *J* = 6.4 Hz, 3 H), 0.87 (d, *J* = 2.1 Hz, 3 H), 0.86 (d, *J* = 2.1 Hz, 3 H), 0.68 (s, 3 H) ppm. **¹³C NMR** (126 MHz, $CDCl_3$) δ 140.85, 121.64, 93.54, 75.39, 74.24, 73.50, 71.88, 64.09, 56.95, 56.27, 50.28, 42.45, 39.97, 39.94, 39.68, 38.69, 37.11, 36.90, 36.34, 35.94, 32.11, 32.02, 28.41, 28.17, 27.03, 24.45, 23.96, 22.99, 22.73, 21.22, 19.60, 18.87, 18.23 (two carbons), 17.78, 17.58, 17.54 (two carbons), 17.51, 17.45, 17.44 (two carbons), 13.09, 13.01, 12.45, 12.44, 12.13, 12.01 ppm. **MS (MALDI-TOF)**: Calculated for $C_{54}H_{102}O_6NaSi_3$ $(M+Na)^+$: 953.688, Found: 953.671. $[\alpha]_D^{20}$ = +51.1 (c = 0.18, CH_2Cl_2).

Methyl *N*-(*tert*-butoxycarbonyl)-*O*-((5a*R*,6*R*,8*S*,9a*R*)-2,2,4,4-tetraisopropyl-6-(((triisopropylsilyl)oxy)methyl)tetrahydro-6*H*-pyrano[3,4-*f*][1,3,5,2,4]trioxadisilepin-8-yl)-L-serinate

The title product compound is prepared according to the general procedure with 5 mol% catalyst **A** and 0.3 mmol glycal at 50 ˚C for 48 h and isolated by flash column chromatography (20:1 Pentane: Ethyl Acetate) giving a pale yellow syrup (108 mg, 0.14 mmol, 71% yield, α/β ratio >20:1).

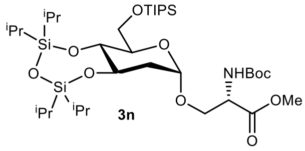

3n

¹H NMR (500 MHz, $CDCl_3$) δ 5.25 (d, *J* = 8.7 Hz, 1 H), 4.83 (d, *J* = 2.9 Hz, 1 H), 4.475-4.44 (m, 1 H), 4.02 (d, *J* = 10.7 Hz, 1 H), 3.99-3.96 (m, 1 H), 3.89-3.86 (dd, *J* = 10.1, 3.8 Hz, 1 H), 3.79-3.76 (m, 2 H), 3.73 (s, 3 H), 3.51-3.45 (m, 2 H), 2.02 (dd, *J* = 13.3, 5.2 Hz, 1 H), 1.67-1.61 (m, 1 H), 1.44 (s, 9 H), 1.11-0.90 (m, 49 H) ppm. **¹³C NMR** (126 MHz, $CDCl_3$) δ 171.39, 155.49, 97.58, 80.10, 74.48, 73.88, 71.62, 67.15, 63.36, 53.85, 52.51, 37.98, 28.40, 18.10, 18.07, 17.74, 17.53 (two carbons), 17.48, 17.43, 17.42, 17.40, 17.39, 13.11, 12.98, 12.48, 12.38, 12.09 ppm. **ESI-HRMS**: Calculated for $C_{36}H_{73}O_{10}NnaSi_3$ $(M+Na)^+$: 786.44345, Found: 786.44299. $[\alpha]_D^{20}$ = +53.3 (c = 0.57, CH_2Cl_2).

Methyl *N*-(*tert*-butoxycarbonyl)-*O*-((5a*R*,6*R*,8*S*,9a*R*)-2,2,4,4-tetraisopropyl-6-(((triisopropylsilyl)oxy)methyl)tetrahydro-6*H*-pyrano[3,4-*f*][1,3,5,2,4]trioxadisilepin-8-yl)-L-threoninate

The title product compound is prepared according to the general procedure with 5 mol% catalyst **A** and 0.3 mmol glycal at 50 ˚C for 48 h and isolated by flash column chromatography (20:1 Pentane: Ethyl Acetate) giving a white foam (119 mg, 0.15 mmol, 77% yield, α/β ratio >20:1).

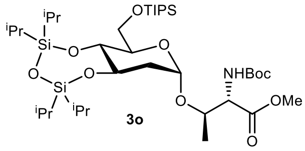

3o

¹H NMR (500 MHz, $CDCl_3$) δ 5.14 (d, *J* = 9.8 Hz, 1 H), 4.82 (d, *J* = 3.7 Hz, 1 H), 4.30-4.25 (m, 2 H), 4.06 (d, *J* = 10.5 Hz, 1 H), 3.95-3.91 (m, 1 H), 3.76-3.72 (m, 1 H), 3.71 (s, 3 H), 3.65-3.63 (m, 1 H), 3.36 (t, *J* = 8.9 Hz, 1 H), 1.92 (dd, *J* = 13.3, 5.3 Hz, 1 H), 1.62-1.56 (m, 1 H), 1.46 (s, 9 H), 1.31 (d, *J* = 6.4 Hz, 3 H), 1.12-0.88 (m, 49 H) ppm. **¹³C NMR** (126 MHz, $CDCl_3$) δ 171.80, 156.15, 98.89, 80.11, 75.70, 74.93, 74.04, 71.45, 63.91, 58.39, 52.36, 38.56, 28.43, 18.74, 18.09 (two carbons), 17.69, 17.53, 17.50, 17.47, 17.41 (three carbons), 17.39, 13.04, 13.02, 12.42 (two carbons), 12.05 ppm. **ESI-HRMS**:

Calculated for $C_{37}H_{75}O_{10}NnaSi_3$ $(M+Na)^+$: 800.45910, Found: 800.45910. $[\alpha]_D^{20}$ = +53.6 (c = 0.27, CH_2Cl_2).

Methyl *N*-(*tert*-butoxycarbonyl)-*O*-((5a*S*,6*S*,8*R*,9a*S*)-2,2,4,4-tetraisopropyl-6-(((triisopropylsilyl)oxy)methyl)tetrahydro-6*H*-pyrano[3,4-*f*][1,3,5,2,4]trioxadisilepin-8-yl)-L-serinate

The title product compound is prepared according to the general procedure with 5 mol% catalyst **A** and 0.3 mmol glycal at 50 ˚C for 72 h and isolated by flash column chromatography (20:1 Pentane: Ethyl Acetate) giving a pale yellow syrup (103 mg, 0.13 mmol, 68% yield, α/β ratio >20:1).

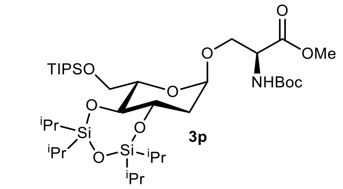

3p

^{1}H NMR (500 MHz, $CDCl_3$) δ 5.35 (d, *J* = 8.8 Hz, 1 H), 4.84 (s, 1 H), 4.50 (d, *J* = 8.4 Hz, 1 H), 4.09-4.07 (m, 1 H), 4.03 (d, *J* = 10.5 Hz, 1 H), 3.92-3.87 (m, 1 H), 3.76 (dd, *J* = 10.5, 5.1 Hz, 1 H), 3.70 (s, 3 H), 3.52-3.49 (m, 1 H), 3.43-3.38 (m, 2 H), 2.04 (dd, *J* = 13.4, 5.3 Hz, 1 H), 1.66-1.60 (m, 1 H), 1.44 (s, 9 H), 1.10-0.87 (m, 49 H) ppm. **^{13}C NMR** (126 MHz, $CDCl_3$) δ 170.83, 155.54, 96.84, 80.12, 74.49, 73.77, 71.74, 66.89, 63.57, 53.66, 52.24, 37.81, 28.43, 18.06 (two carbons), 17.72, 17.48 (two carbons), 17.45, 17.43, 17.38 (two carbons), 17.35, 13.06, 12.98, 12.45, 12.22, 12.09 ppm. **ESI-HRMS**: Calculated for $C_{36}H_{73}O_{10}NnaSi_3$ $(M+Na)^+$: 786.44345, Found: 786.44316. $[\alpha]_D^{20}$ = -28.9 (c = 0.32, CH_2Cl_2).

Methyl (*S*)-2-((*tert*-butoxycarbonyl)amino)-3-(4-(((5a*R*,6*R*,8*R*,9a*R*)-2,2,4,4-tetraisopropyl-6-(((triisopropylsilyl)oxy)methyl)tetrahydro-6*H*-pyrano[3,4-*f*][1,3,5,2,4]trioxadisilepin-8-yl)oxy)phenyl)propanoate

The title product compound is prepared according to the general procedure with 5 mol% catalyst **A** and 0.3 mmol glycal at 50 ˚C for 108 h and isolated by flash column chromatography (20:1 Pentane: Ethyl Acetate) giving a pale yellow syrup (98 mg, 0.12 mmol, 58% yield, α/β ratio >20:1).

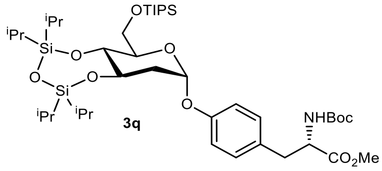

3q

^{1}H NMR (700 MHz, $CDCl_3$) δ 7.05-6.98 (m, 4 H), 5.52 (d, *J* = 2.8 Hz, 1 H), 4.95 (s, 1 H), 4.55-4.51 (m, 1 H), 4.23-4.20 (m, 1 H), 3.99 (d, *J* = 10.8 Hz, 1 H), 3.84 (dd, *J* = 10.9, 5.5 Hz, 1 H), 3.70 (s, 3 H), 3.69-3.67 (m, 1 H), 3.61 (t, *J* = 9.0 Hz, 1 H), 3.05-2.98 (m, 2 H), 2.26 (dd, *J* = 13.1, 5.3 Hz, 1 H), 1.84-1.80 (m, 1 H), 1.42 (s, 9 H), 1.15-0.92 (m, 49 H) ppm. **^{13}C NMR** (176 MHz, $CDCl_3$) δ 172.49, 156.38, 155.24, 130.25, 129.33, 117.17, 96.49, 79.96, 74.46, 74.04, 71.60, 63.23, 54.63, 52.25, 38.43, 37.54, 28.43, 18.08, 18.05, 17.76, 17.58, 17.56, 17.52, 17.45, 17.43, 17.40, 17.39, 13.16, 13.05, 12.50, 12.39, 12.10 ppm. **ESI-HRMS**: Calculated for $C_{42}H_{77}O_{10}NnaSi_3$ $(M+Na)^+$: 862.47475, Found: 862.47442. $[\alpha]_D^{20}$ = +78.8 (c = 0.20, CH_2Cl_2).

(5a*R*,6*R*,8*S*,9a*R*)-2,2,4,4-Tetraisopropyl-8-(prop-2-yn-1-yloxy)-6-(((triisopropylsilyl)oxy)methyl)tetrahydro-6*H*-pyrano[3,4-*f*][1,3,5,2,4]trioxadisilepine

The title product compound is prepared according to the general procedure with 3 mol% catalyst **A** and 0.3 mmol glycal at 40 ˚C for 48 h and isolated by flash column chromatography (2:1 Pentane: CH_2Cl_2) giving a pale yellow syrup (107 mg, 0.18 mmol, 89% yield, α/β ratio >20:1).

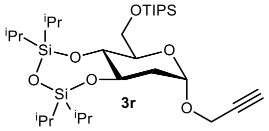

3r

^{1}H NMR (500 MHz, $CDCl_3$) δ 5.09 (d, *J* = 3.6 Hz, 1 H), 4.26-4.19 (m, 2 H), 4.09 (dd, *J* = 10.7, 1.9 Hz, 1 H), 4.04-3.99 (m, 1 H), 3.77 (dd, *J* = 10.7, 7.0 Hz, 1 H), 3.58-3.55 (m, 1 H), 3.43 (dd, *J* = 9.6, 8.2 Hz, 1 H), 2.39 (t, *J* = 2.4 Hz, 1 H), 2.12 (dd, *J* = 13.5, 5.5 Hz, 1 H), 1.73 (ddd, *J* = 13.6, 11.3, 3.9 Hz, 1 H), 1.12-0.91 (m, 49 H) ppm. **^{13}C NMR** (126 MHz, $CDCl_3$) δ 95.25, 79.53, 74.91, 74.27, 73.82, 71.73, 63.67, 53.39, 37.86, 18.10 (two carbons), 17.74, 17.53 (three carbons), 17.44, 17.42, 17.39 (two carbons), 13.07, 12.97, 12.37, 12.34, 12.12 ppm. **ESI-HRMS**: Calculated for $C_{30}H_{60}O_6NaSi_3$ $(M+Na)^+$: 623.35899, Found: 623.36006. $[\alpha]_D^{20}$ = +82.9 (c = 0.24, CH_2Cl_2).

(((2*R*,3*S*,4*R*,6*S*)-2-(((*Tert*-Butyldimethylsilyl)oxy)methyl)-6-(((3a*R*,5*R*,5a*S*,8a*S*,8b*R*)-2,2,7,7-tetramethyltetrahydro-5*H*-bis([1,3]dioxolo)[4,5-*b*:4',5'-*d*]pyran-5-yl)methoxy)tetrahydro-2*H*-pyran-3,4-diyl)bis(oxy))bis(*tert*-butyldimethylsilane)

The title product compound is prepared according to the general procedure with 3 mol% catalyst **A** at 23 ˚C for 24 h and isolated by flash column chromatography (20:1 Pentane: Ethyl Acetate) giving a pale yellow syrup (136 mg, 0.18 mmol, 91% yield, α/β ratio 91:9).

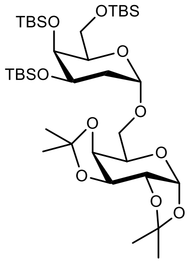

3s

¹H NMR (500 MHz, $CDCl_3$) δ 5.48 (d, *J* = 4.9 Hz, 1 H), 4.89 (d, *J* = 2.9 Hz, 1 H), 4.58 (dd, *J* = 7.9, 2.3 Hz, 1 H), 4.28 (dd, *J* = 5.0, 2.4 Hz, 1 H), 4.19 (dd, *J* = 8.0, 1.6 Hz, 1 H), 4.06-4.03 (m, 1 H), 3.95-3.92 (m, 1 H), 3.83 (s, 1 H), 3.74 (dd, *J* = 10.2, 6.9 Hz, 1 H), 3.70-3.65 (m, 2 H), 3.61-3.53 (m, 2 H), 2.07 (td, *J* = 12.1, 3.5 Hz, 1 H), 1.60 (dd, *J* = 12.4, 4.3 Hz, 1 H), 1.51 (s, 3 H), 1.41 (s, 3 H), 1.31 (s, 6 H), 0.89 (s, 9 H), 0.88 (s, 9 H), 0.87 (s, 9 H), 0.09 (s, 3 H), 0.07 (s, 3 H), 0.063 (s, 3 H), 0.056 (s, 3 H), 0.04 (s, 3 H), 0.03 (s, 3 H) ppm. **¹³C NMR** (126 MHz, $CDCl_3$) δ 109.35, 108.57, 97.54, 96.42, 72.49, 71.37, 70.81, 70.80, 70.07, 68.37, 66.67, 65.38, 62.15, 33.71, 26.36, 26.28, 26.24, 26.10, 26.01, 25.15, 24.56, 18.72, 18.64, 18.31, -3.80, -4.26, -4.60, -4.81, -5.12, -5.23 ppm. **ESI-HRMS**: Calculated for $C_{36}H_{72}O_{10}NaSi_3$ $(M+Na)^+$: 771.43255, Found: 771.43381. $[\alpha]_D^{20}$ = +16.7 (c = 0.83, CH_2Cl_2).

(3a*R*,5*R*,5a*S*,8a*S*,8b*R*)-5-((((2*S*,4*R*,5*R*,6*R*)-4,5-Bis(benzyloxy)-6-((benzyloxy)methyl)tetrahydro-2*H*-pyran-2-yl)oxy)methyl)-2,2,7,7-tetramethyltetrahydro-5*H*-bis([1,3]dioxolo)[4,5-*b*:4',5'-*d*]pyran

The title product compound is prepared according to the general procedure with 3 mol% catalyst **A** and 0.3 mmol glycal at 30 ˚C for 48 h and isolated by flash column chromatography (20:1 Pentane: Ethyl Acetate) giving a pale yellow syrup (75 mg, 0.11 mmol, 55% yield, α/β ratio 85:15).

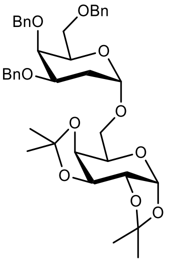

3t

¹H NMR (500 MHz, $CDCl_3$) δ 7.37-7.26 (m, 15 H), 5.55 (d, *J* = 5.0 Hz, 1 H), 5.07 (d, *J* = 2.9 Hz, 1 H), 4.95 (d, *J* = 11.6 Hz, 1 H), 4.65 (d, *J* = 11.7 Hz, 1 H), 4.63-4.61 (m, 3 H), 4.52 (d, *J* = 11.7 Hz, 1 H), 4.45 (d, *J* = 11.7 Hz, 1 H), 4.33 (dd, *J* = 5.0, 2.4 Hz, 1 H), 4.24 (dd, *J* = 7.9, 1.8 Hz, 1 H), 4.00-3.97 (m, 4 H), 3.78 (dd, *J* = 10.7, 6.9 Hz, 1 H), 3.70 (dd, *J* = 10.7, 6.4 Hz, 1 H), 3.65 (dd, *J* = 9.2, 7.5 Hz, 1 H), 3.57 (dd, *J* = 9.2, 5.6 Hz, 1 H), 2.25 (td, *J* = 12.5, 3.6 Hz, 1 H), 2.06 (dd, *J* = 12.7, 4.4 Hz, 1 H), 1.54 (s, 3 H), 1.45 (s, 3 H), 1.36 (s, 3 H), 1.35 (s, 3 H) ppm. **¹³C NMR** (126 MHz, $CDCl_3$) δ 139.00, 138.66, 138.19, 128.45 (three carbons), 128.33, 128.27, 127.92, 127.72, 127.56, 127.39, 109.37, 108.60, 97.59, 96.43, 74.77, 74.39, 73.45, 72.89, 71.14, 70.73, 70.67, 70.48, 69.88, 69.24, 65.88, 65.59, 31.21, 26.21, 26.06, 25.03, 24.62 ppm. **ESI-HRMS**: Calculated for $C_{39}H_{48}O_{10}Na$ $(M+Na)^+$: 699.31397, Found: 699.31420. $[\alpha]_D^{20}$ = +11.2 (c = 0.66, CH_2Cl_2).

(3a*R*,5*R*,5a*S*,8a*S*,8b*R*)-5-((((2*S*,4*R*,5*R*,6*R*)-4,5-Dimethoxy-6-(methoxymethyl)tetrahydro-2*H*-pyran-2-yl)oxy)methyl)-2,2,7,7-tetramethyltetrahydro-5*H*-bis([1,3]dioxolo)[4,5-*b*:4',5'-*d*]pyran

The title product compound is prepared according to the general procedure with 3 mol% catalyst **A** and 0.3 mmol at 30 ˚C for 48 h and isolated by flash column chromatography (20:1 Pentane: Ethyl Acetate) giving a pale yellow syrup (53 mg, 0.12 mmol, 59% yield, α/β ratio 83:17).

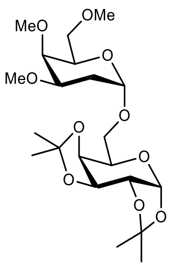

3u

^{1}H NMR (500 MHz, $CDCl_3$) δ 5.50 (d, *J* = 3.2 Hz, 1 H), 4.97 (s, 1 H), 4.58 (d, *J* = 7.9 Hz, 1 H), 4.29-4.28 (m, 1 H), 4.21 (d, *J* = 7.9 Hz, 1 H), 3.93 (t, *J* = 6.5 Hz, 1 H), 3.86 (t, *J* = 6.4 Hz, 1 H), 3.73-3.70 (m, 1 H), 3.64-3.58 (m, 3 H), 3.56-3.51 (m, 4 H), 3.46-3.42 (m, 1 H), 3.38-3.35 (m, 6 H), 2.00-1.89 (m, 2 H), 1.50 (s, 3 H), 1.41 (s, 3 H), 1.30 (s, 6 H) ppm. **^{13}C NMR** (126 MHz, $CDCl_3$) δ 109.39, 108.61, 97.61, 96.42, 75.87, 74.40, 71.45, 71.14, 70.73, 70.66, 69.45, 65.92, 65.68, 61.00, 59.20, 56.15, 30.78, 26.17, 26.05, 25.01, 24.63 ppm. **ESI-HRMS**: Calculated for $C_{21}H_{36}O_{10}Na$ $(M+Na)^+$: 471.22007, Found: 471.21919. $[\alpha]_D^{20}$ = +13.8 (c = 0.44, CH_2Cl_2).

(2*R*,3*R*,4*S*,5*R*,6*S*)-2-((((2*S*,4*R*,5*S*,6*R*)-4,5-Bis((*tert*-butyldimethylsilyl)oxy)-6-(((*tert*-butyldimethylsilyl)oxy)methyl)tetrahydro-2*H*-pyran-2-yl)oxy)methyl)-6-methoxytetrahydro-2*H*-pyran-3,4,5-triyl tribenzoate

The title product compound is prepared according to the general procedure with 3 mol% catalyst **A** and 0.3 mmol glycal at 30 ˚C for 72 h and isolated by flash column chromatography (20:1 Pentane: Ethyl Acetate) giving a white foam (158 mg, 0.16 mmol, 80% yield, α/β ratio >20:1).

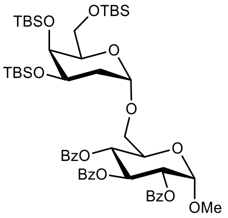

3v

^{1}H NMR (500 MHz, $CDCl_3$) δ 8.00 (d, *J* = 8.2 Hz, 2 H), 7.94 (d, *J* = 8.1 Hz, 2 H), 7.88 (d, *J* = 8.2 Hz, 2 H), 7.52-7.48 (m, 2 H), 7.43-7.35 (m, 5 H), 7.30-7.26 (m, 2 H), 6.16 (td, *J* = 9.6, 2.8 Hz, 1 H), 5.65-5.60 (m, 1 H), 5.25-5.21 (m, 2 H), 4.94 (s, 1 H), 4.24-4.21 (m, 1 H), 4.20-4.14 (m, 1 H), 3.88-3.85 (m, 1 H), 3.81 (s, 1 H), 3.65-3.53 (m, 4 H), 3.47 (s, 3 H), 2.14-2.07 (m, 1 H), 1.69-1.65 (m, 1 H), 0.96-0.86 (m, 27 H), 0.13-0.01 (m, 18 H) ppm. **^{13}C NMR** (126 MHz, $CDCl_3$) δ 165.95, 165.87, 165.21, 133.44, 133.36, 133.13, 130.04, 129.90, 129.77, 129.41, 129.24, 129.22, 128.51, 128.51, 128.33, 98.01, 96.85, 72.83, 72.36, 70.84, 70.48, 69.54, 68.74, 68.28, 65.33, 62.89, 55.31, 33.44, 26.37, 26.23, 25.93, 18.72, 18.69, 18.24, -3.73, -4.27, -4.75, -4.86, -5.21, -5.37 ppm. **ESI-HRMS**: Calculated for $C_{52}H_{78}O_{13}NaSi_3$ $(M+Na)^+$: 1017.46424, Found: 1017.46441. $[\alpha]_D^{20}$ = +54.6 (c = 0.07, CH_2Cl_2).

(((2*S*,3*R*,4*S*,6*R*)-2-(((*Tert*-Butyldimethylsilyl)oxy)methyl)-6-(((3a*R*,5*R*,5a*S*,8a*S*,8b*R*)-2,2,7,7-tetramethyltetrahydro-5*H*-bis([1,3]dioxolo)[4,5-*b*:4',5'-*d*]pyran-5-yl)methoxy)tetrahydro-2*H*-pyran-3,4-diyl)bis(oxy))bis(*tert*-butyldimethylsilane)

The title product compound is prepared according to the general procedure with 3 mol% catalyst **A** and 0.3 mmol glycal at 30 ˚C for 72 h and isolated by flash column chromatography (20:1 Pentane: Ethyl Acetate) giving a pale yellow syrup (66 mg, 0.09 mmol, 44% yield, α/β ratio >20:1). (83 mg glycal **1h** was recovered)

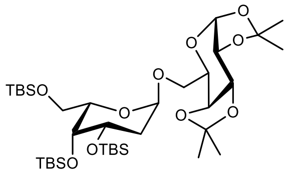

3w

^{1}H NMR (500 MHz, $CDCl_3$) δ 5.51 (d, *J* = 4.9 Hz, 1 H), 4.93 (d, *J* = 2.9 Hz, 1 H), 4.58 (dd, *J* = 7.9, 1.8 Hz, 1 H), 4.29-4.28 (m, 1 H), 4.19 (d, *J* = 8.1 Hz, 1 H), 4.06-4.03 (m, 1 H), 3.93 (t, *J* = 6.0 Hz, 1 H), 3.82 (s, 1 H), 3.75 (dd, *J* = 10.5, 5.1 Hz, 1 H), 3.67-3.61 (m, 3 H), 3.49 (dd, *J* = 10.4, 7.2 Hz, 1 H), 2.07 (td, *J* = 12.1, 3.4 Hz, 1 H), 1.65 (dd, *J* = 12.4, 4.0 Hz, 1 H), 1.52 (s, 3 H), 1.42 (s, 3 H), 1.32 (s, 3 H), 1.30 (s, 3 H), 0.90-0.88 (m, 27 H), 0.09-0.04 (m, 18 H) ppm. **^{13}C NMR** (126 MHz, $CDCl_3$) δ 109.35, 108.66,

98.09, 96.37, 72.84, 71.51, 70.84, 70.74, 70.31, 68.55, 67.54, 65.71, 62.53, 33.48, 26.38, 26.27, 26.25, 26.12, 25.99, 25.18, 24.47, 18.72 (two carbons), 18.27, -3.74, -4.28, -4.62, -4.83, -5.09, -5.20 ppm. **ESI-HRMS**: Calculated for $C_{36}H_{72}O_{10}NaSi_3$ $(M+Na)^+$: 771.43255, Found: 771.43256. $[\alpha]_D^{20}$ = -61.2 (c = 0.38, CH_2Cl_2).

(((2*R*,3*S*,4*R*,6*R*)-6-(((2*S*,6*S*,7*R*,8*S*)-8-(Benzyloxy)-6-methoxy-2-phenylhexahydropyrano[3,2-d][1,3]dioxin-7-yl)oxy)-2-(((*tert*-butyldimethylsilyl)oxy)methyl)tetrahydro-2*H*-pyran-3,4-diyl)bis(oxy))bis(*tert*-butyldimethylsilane)

The title product compound is prepared according to the general procedure with 3 mol% catalyst **A** and 0.3 mmol glycal at 30 ˚C for 72 h and isolated by flash column chromatography (20:1 Pentane: Ethyl Acetate) giving a pale yellow syrup (141 mg, 0.16 mmol, 82% yield, α/β ratio >20:1).

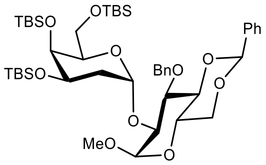

3x

^{1}H NMR (500 MHz, $CDCl_3$) δ 7.49-7.26 (m, 10 H), 5.57 (d, *J* = 6.3 Hz, 1 H), 5.15 (d, *J* = 7.3 Hz, 1 H), 5.05 (d, *J* = 7.0 Hz, 1 H), 4.89-4.87 (m, 2 H), 4.32-4.31 (m, 1 H), 4.12-4.08 (m, 1 H), 3.97-3.92 (m, 2 H), 3.90-3.60 (m, 7 H), 3.45-3.44 (m, 3 H), 2.18-2.12 (m, 1 H), 1.80-1.75 (s, 1 H), 0.96-0.88 (m, 27 H), 0.17-0.03 (m, 18 H) ppm. **^{13}C NMR** (126 MHz, $CDCl_3$) δ 139.23, 137.56, 128.96, 128.26 (two carbons), 127.34, 127.31, 126.22, 101.40, 97.40, 93.35, 81.61, 77.42, 74.95, 73.35, 73.30, 70.46, 69.19, 68.42, 63.39, 62.55, 55.18, 33.27, 26.27 (two carbons), 26.08, 18.71, 18.50, 18.32, -3.71, -4.14, -4.59, -4.73, -5.13, -5.30 ppm. **ESI-HRMS**: Calculated for $C_{45}H_{76}O_{10}NaSi_3$ $(M+Na)^+$: 883.46385, Found: 883.46597. $[\alpha]_D^{20}$ = +47.8 (c = 0.46, CH_2Cl_2).

(((2*R*,3*S*,4*R*,6*R*)-6-(((2*S*,6*S*,7*R*,8*S*)-7-(Benzyloxy)-6-methoxy-2-phenylhexahydropyrano[3,2-*d*][1,3]dioxin-8-yl)oxy)-2-(((*tert*-butyldimethylsilyl)oxy)methyl)tetrahydro-2*H*-pyran-3,4-diyl)bis(oxy))bis(*tert*-butyldimethylsilane)

The title product compound is prepared according to the general procedure with 3 mol% catalyst **A** and 0.3 mmol at 30 ˚C for 48 h and isolated by flash column chromatography (20:1 Pentane: Ethyl Acetate) giving a pale yellow syrup (125 mg, 0.15 mmol, 73% yield, α/β ratio >20:1).

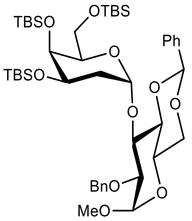

3y

^{1}H NMR (500 MHz, $CDCl_3$) δ 7.49-7.32 (m, 10 H), 5.52-5.51 (m, 1 H), 5.42-5.41 (m, 1 H), 4.87-4.84 (m, 1 H), 4.60-4.56 (m, 1 H), 4.44-4.43 (m, 1 H), 4.36-4.31 (m, 1 H), 4.24-4.22 (m, 1 H), 4.16-4.14 (m, 1 H), 4.07-4.05 (m, 1 H), 3.89 (s, 1 H), 3.84-3.78 (m, 1 H), 3.72-3.66 (m, 3 H), 3.57-3.53 (m, 1 H), 3.43-3.37 (m, 1 H), 3.35-3.34 (m, 3 H), 2.12-2.08 (m, 1 H), 1.69-1.67 (m, 1 H), 0.95-0.91 (m, 27 H), 0.14-0.08 (m, 18 H) ppm. **^{13}C NMR** (126 MHz, $CDCl_3$) δ 138.64, 137.48, 129.06, 128.58, 128.35, 128.04, 128.01, 126.30, 101.58, 99.53, 97.80, 83.55, 78.33, 73.67, 72.26, 71.80, 70.19, 69.27, 68.50, 62.15, 62.06, 55.42, 33.76, 26.37 (two carbons), 26.05, 18.76, 18.68, 18.38, -3.82, -4.17, -4.51, -4.75, -5.20, -5.27 ppm. **ESI-HRMS**: Calculated for $C_{45}H_{76}O_{10}NaSi_3$ $(M+Na)^+$: 883.46385, Found: 883.46581. $[\alpha]_D^{20}$ = +32.9 (c = 0.79, CH_2Cl_2).

(((2*R*,3*S*,4*R*,6*R*)-6-(((2*R*,3*R*,4*S*,5*R*,6*S*)-4,5-Bis(benzyloxy)-2-((benzyloxy)methyl)-6-methoxytetrahydro-2*H*-pyran-3-yl)oxy)-2-(((*tert*-butyldimethylsilyl)oxy)methyl)tetrahydro-2*H*-pyran-3,4-diyl)bis(oxy))bis(*tert*-butyldimethylsilane)

The title product compound is prepared according to the general procedure with 3 mol% catalyst **A** and 0.3 mmol glycal at 30 ˚C for 72 h and isolated by flash column chromatography (20:1 Pentane: Ethyl Acetate) giving a pale yellow syrup (115 mg, 0.12 mmol, 61% yield, α/β ratio >20:1).

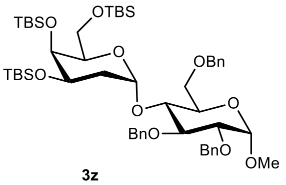

3z

^{1}H NMR (500 MHz, $CDCl_3$) δ 7.39-7.29 (m, 15 H), 5.36 (d, *J* = 3.4 Hz, 1 H), 5.09 (d, *J* = 11.3 Hz, 1 H), 4.75 (t, *J* = 11.1 Hz, 2 H), 4.69 (d, *J* = 11.7 Hz, 1 H), 4.66-4.62 (m, 2 H), 4.57 (d, *J* = 11.7 Hz, 1 H), 3.98-3.94 (m, 1 H), 3.92-3.81 (m, 4 H), 3.70-3.68 (m, 1 H), 3.67-3.63 (m, 3 H), 3.64-3.59 (m, 1 H), 3.57 (dd, *J* = 9.7, 3.6 Hz, 1 H), 3.45 (s, 3 H), 2.04 (td, *J* = 12.4, 4.0 Hz, 1 H), 1.58 (dd, *J* = 12.3, 4.2 Hz, 1 H), 0.94-0.93 (m, 27 H), 0.13-0.07 (m, 18 H) ppm. **^{13}C NMR** (126 MHz, $CDCl_3$) δ 138.76, 138.53, 138.10, 128.52, 128.42, 128.31, 128.21, 128.00, 127.63, 127.52, 127.51, 127.42, 100.28, 97.72, 82.37, 80.29, 76.62, 75.40, 73.49, 73.34, 73.24, 70.18, 70.16, 70.11, 68.14, 62.49, 55.32, 34.20, 26.27, 26.24, 26.02, 18.67, 18.54, 18.33, -3.77, -4.30, -4.58, -4.91, -5.08, -5.20 ppm. **ESI-HRMS**: Calculated for $C_{52}H_{84}O_{10}NaSi_3$ $(M+Na)^+$: 975.52645, Found: 975.52624. $[\alpha]_D^{20}$ = +49.0 (c = 0.35, CH_2Cl_2).

(((2*S*,3*R*,4*S*,6*S*)-6-(((2*R*,4a*R*,6*S*,7*R*,8*S*,8a*R*)-7-(Benzyloxy)-6-methoxy-2-phenylhexahydropyrano[3,2-*d*][1,3]dioxin-8-yl)oxy)-2-(((*tert*-butyldimethylsilyl)oxy)methyl)tetrahydro-2*H*-pyran-3,4-diyl)bis(oxy))bis(*tert*-butyldimethylsilane)

The title product compound is prepared according to the general procedure with 3 mol% catalyst **A** and 0.3 mmol glycal at 30 ˚C for 72 h and isolated by flash column chromatography (20:1 Pentane: Ethyl Acetate) giving a pale yellow syrup (129 mg, 0.15 mmol, 89% yield, α/β ratio 89:11).

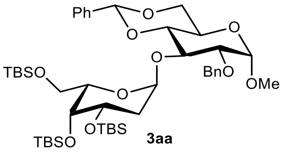

3aa

^{1}H NMR (500 MHz, $CDCl_3$) δ 7.54-7.33 (m, 10 H), 5.50-5.46 (m, 2 H), 4.73 (d, *J* = 12.3 Hz, 1 H), 4.63 (d, *J* = 12.3 Hz, 1 H), 4.59-4.57 (m, 1 H), 4.31-4.23 (m, 2 H), 4.10-4.08 (m, 1 H), 3.95-3.94 (m, 1 H), 3.81-3.77 (m, 1 H), 3.71-3.67 (m, 2 H), 3.65-3.61 (m, 1 H), 3.55-3.54 (m, 2 H), 3.50-3.46 (m, 1 H), 3.35 (s, 3 H), 2.12-2.07 (m, 1 H), 1.63-1.60 (m, 1 H), 0.91-0.84 (m, 27 H), 0.10-0.04 (m, 12 H), -0.08 (m, 3 H), -0.12 (m, 3 H) ppm. **^{13}C NMR** (126 MHz, $CDCl_3$) δ 138.10, 137.56, 128.81, 128.51, 128.15, 128.05, 127.97, 126.25, 101.60, 98.69, 97.42, 80.92, 80.65, 72.75, 72.37, 71.35, 70.58, 69.20, 68.18, 62.97, 62.55, 55.20, 33.68, 26.28, 26.19, 25.97, 18.66, 18.57, 18.20, -3.80, -4.28, -4.65, -4.96, -5.33, -5.64 ppm. **ESI-HRMS**: Calculated for $C_{45}H_{77}O_{10}Si_3$ $(M+H)^+$: 861.48190, Found: 861.48252. $[\alpha]_D^{20}$ = -18.4 (c = 0.48, CH_2Cl_2).

(((2*R*,3*S*,4*R*,6*R*)-2-(((*Tert*-Butyldimethylsilyl)oxy)methyl)-6-(((3a*R*,4*R*,6*S*,7*S*,7a*R*)-4-methoxy-2,2,6-trimethyltetrahydro-4*H*-[1,3]dioxolo[4,5-*c*]pyran-7-yl)oxy)tetrahydro-2*H*-pyran-3,4-diyl)bis(oxy))bis(*tert*-butyldimethylsilane)

The title product compound is prepared according to the general procedure with 3 mol% catalyst **A** and 0.3 mmol at 30 ˚C for 72 h and isolated by flash column chromatography (20:1 Pentane: Ethyl Acetate) giving a pale yellow syrup (110 mg, 0.16 mmol, 77% yield, α/β ratio >20:1).

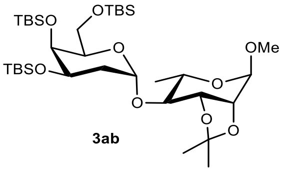

3ab

^{1}H NMR (500 MHz, $CDCl_3$) δ 4.96 (d, *J* = 3.2 Hz, 1 H), 4.83 (s, 1 H), 4.07-4.06 (m, 2 H), 4.02-4.01 (m, 1 H), 3.93 (s, 1 H), 3.82 (dd, *J* = 9.3, 5.5 Hz, 1 H), 3.68 (t, *J* = 9.3 Hz, 1 H), 3.60-3.52 (m, 2 H), 3.36 (s, 3 H), 3.31-3.30 (m, 1 H), 2.14 (td, *J* = 12.1, 3.7 Hz, 1 H), 1.58 (dd, *J* = 12.2, 4.3 Hz, 1 H), 1.52 (s, 3 H), 1.33 (s, 3 H), 1.23 (d, *J* = 6.3 Hz, 3 H), 0.89-0.88 (m, 27 H), 0.09-0.06 (m, 18 H) ppm. **^{13}C NMR** (126 MHz, $CDCl_3$) δ 109.03, 99.09, 98.20, 80.08, 77.35, 75.92, 72.11, 69.57, 68.39, 65.23, 60.63, 54.99, 33.97, 28.34, 26.60, 26.39, 26.34, 26.01, 18.78, 18.65, 18.21, 17.75, -4.02, -4.33, -4.58, -4.67, -4.85, -5.14 ppm. **ESI-HRMS**: Calculated for $C_{34}H_{70}O_9NaSi_3$ $(M+Na)^+$: 729.42198, Found: 729.42303. $[\alpha]_D^{20}$ = +38.3 (c = 0.41, CH_2Cl_2).

(((2*R*,3*S*,4*R*,6*R*)-2-(((*Tert*-Butyldimethylsilyl)oxy)methyl)-6-(((3a*R*,5*R*,6*S*,6a*R*)-5-((*S*)-2,2-dimethyl-1,3-dioxolan-4-yl)-2,2-dimethyltetrahydrofuro[2,3-*d*][1,3]dioxol-6-yl)oxy)tetrahydro-2*H*-pyran-3,4-diyl)bis(oxy))bis(*tert*-butyldimethylsilane)

The title product compound is prepared according to the general procedure with 3 mol% catalyst **A** at 30 ˚C for 72 h and isolated by flash column chromatography (20:1 Pentane: Ethyl Acetate) giving a pale yellow syrup (113 mg, 0.15 mmol, 75% yield, α/β ratio >20:1).

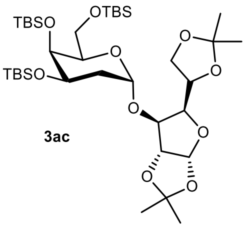

3ac

^{1}H NMR (500 MHz, $CDCl_3$) δ 5.83 (d, *J* = 3.5 Hz, 1 H), 5.08 (d, *J* = 2.9 Hz, 1 H), 4.63 (d, *J* = 3.5 Hz, 1 H), 4.19-4.16 (m, 2 H), 4.10-4.06 (m, 2 H), 3.98-3.94 (m, 2 H), 3.81 (s, 1 H), 3.69-3.64 (m, 3 H), 2.09 (td, *J* = 12.3, 3.7 Hz, 1 H), 1.63 (dd, *J* = 12.5, 4.3 Hz, 1 H), 1.47 (s, 3 H), 1.39 (s, 3 H), 1.31 (s, 3 H), 1.27 (s, 3 H), 0.89-0.88 (m, 27 H), 0.09-0.06 (m, 18 H) ppm. **^{13}C NMR** (126 MHz, $CDCl_3$) δ 111.97, 109.16, 105.48, 100.11, 83.80, 81.61, 81.31, 73.69, 72.78, 70.40, 68.15, 67.79, 63.23, 33.75, 27.14, 26.93, 26.49, 26.30, 26.22, 26.07, 25.43, 18.68, 18.61, 18.48, -3.75, -4.24, -4.54, -4.82, -5.15, -5.24 ppm. **ESI-HRMS**: Calculated for $C_{36}H_{72}O_{10}NaSi_3$ $(M+Na)^+$: 771.43255, Found: 771.43264. $[\alpha]_D^{20}$ = +38.3 (c = 0.41, CH_2Cl_2).

(((2*R*,3*S*,4*R*,6*S*)-2-(((*Tert*-Butyldimethylsilyl)oxy)methyl)-6-(((3*S*,8*S*,9*S*,10*R*,13*R*,14*S*,17*R*)-10,13-dimethyl-17-(l-6-methylheptan-2-yl)-2,3,4,7,8,9,10,11,12,13,14,15,16,17-tetradecahydro-1*H*-cyclopenta[*a*] 23 henanthrene-3-yl)oxy)tetrahydro-2*H*-pyran-3,4-diyl)bis(oxy))bis(*tert*-butyldimethylsilane)

The title product compound is prepared according to the general procedure with 3 mol% catalyst **A** and 0.3 mmol at 30 ˚C for 24 h and isolated by flash column chromatography (3:1 Pentane: CH_2Cl_2) giving a pale yellow syrup (95 mg, 0.11 mmol, 54% yield, α/β ratio >20:1).

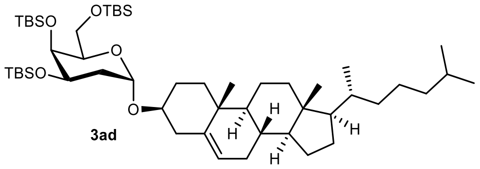

3ad

^{1}H NMR (500 MHz, $CDCl_3$) δ 5.30 (d, *J* = 4.3 Hz, 1 H), 5.04 (d, *J* = 2.6 Hz, 1 H), 4.05 (d, *J* = 11.4 Hz, 1 H), 3.82 (s, 1 H), 3.70-3.62 (m, 3 H), 3.47-3.41 (m, 1 H), 2.26 (d, *J* = 7.8 Hz, 2 H), 2.10-2.05 (m, 1 H), 2.02-1.94 (m, 2 H), 1.87-1.81 (m, 3 H), 1.60-1.21 (m, 12 H), 1.19-0.98 (m, 8 H), 0.91-0.86 (m, 41 H), 0.68 (s, 3 H), 0.10 (s, 3 H), 0.09 (s, 3 H), 0.08 (s, 6 H), 0.05 (s, 6 H) ppm. **^{13}C NMR** (126 MHz, $CDCl_3$) δ 141.15, 121.51, 95.52, 75.56, 72.75, 70.50, 68.55, 62.87, 56.91, 56.28, 50.28, 42.46, 40.32, 39.94, 39.68, 37.29, 36.90, 36.34, 35.96, 34.26, 32.11, 32.04, 28.41, 28.18, 27.82, 26.38, 26.29, 26.05, 24.45, 23.98, 23.00, 22.74, 21.22, 19.60, 18.88, 18.72, 18.65, 18.38, 12.01, -3.72, -4.16, -4.51, -4.77, -5.09, -5.19 ppm. **MS (MALDI-TOF)**: Calculated for $C_{51}H_{98}O_5NaSi_3$ $(M+Na)^+$: 897.662, Found: 897.662. $[\alpha]_D^{20}$ = +40.6 (c = 0.12, CH_2Cl_2).

(8*R*,9*S*,10*R*,13*S*,14*S*,17*S*)-17-(((2*S*,4*R*,5*S*,6*R*)-4,5-Bis((*tert*-butyldimethylsilyl)oxy)-6-(((tert-butyldimethylsilyl)oxy)methyl)tetrahydro-2*H*-pyran-2-yl)oxy)-10,13-dimethyl-1,2,6,7,8,9,10,11,12,13,14,15,16,17-tetradecahydro-3*H*-cyclopenta[*a*]23henanthrene-3-one

The title product compound is prepared according to the general procedure with 3 mol% catalyst **A** and 0.3 mmol glycal at 30 ˚C for 24 h and isolated by flash column chromatography (20:1 Pentane: Ethyl Acetate) giving a white foam (125 mg, 0.16 mmol, 80% yield, α/β ratio >20:1).

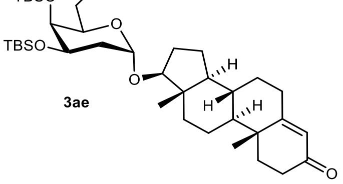

3ae

^{1}H NMR (500 MHz, $CDCl_3$) δ 5.72 (s, 1 H), 4.87 (d, *J* = 3.0 Hz, 1 H), 4.08-4.04 (m, 1 H), 3.81 (s, 1 H), 3.67-3.57 (m, 4 H), 2.42-2.31 (m, 3 H), 2.28-2.23 (m, 1 H), 2.09 (td, *J* = 12.0, 3.5 Hz, 1 H), 2.03-1.95

(m, 2 H), 1.86-1.78 (m, 2 H), 1.70 (dd, *J* = 13.9, 4.9 Hz, 1 H), 1.65-1.58 (m, 1 H), 1.56-1.51 (m, 3 H), 1.47-1.37 (m, 2 H), 1.34-1.25 (m, 1 H), 1.18 (s, 3 H), 1.16-1.09 (m, 1 H), 1.03-0.92 (m, 3 H), 0.90 (s, 9 H), 0.88 (s, 9 H), 0.87 (s, 9 H), 0.77 (s, 3 H), 0.09 (s, 3 H), 0.07 (s, 3 H), 0.064 (s, 3 H), 0.059 (s, 3 H), 0.029 (s, 3 H), 0.026 (s, 3 H) ppm. **^{13}C NMR** (126 MHz, $CDCl_3$) δ 199.67, 171.43, 123.98, 95.53, 82.66, 72.68, 70.54, 68.50, 62.86, 54.19, 50.52, 42.41, 38.76, 37.28, 35.83, 35.47, 34.08, 33.99, 32.94, 31.69, 26.74, 26.35, 26.24, 25.98, 23.60, 20.71, 18.72, 18.69, 18.33, 17.52, 11.73, -3.81, -4.29, -4.66, -4.85, -5.15, -5.23 ppm. **ESI-HRMS**: Calculated for $C_{43}H_{81}O_6Si_3$ $(M+H)^+$: 777.53355, Found: 777.53587. $[\alpha]_D^{20}$ = +86.3 (c = 0.13, CH_2Cl_2).

(8*R*,9*S*,10*R*,13*S*,14*S*,17*S*)-17-(((2*R*,4*R*,5*S*,6*R*)-4,5-Bis((*tert*-butyldimethylsilyl)oxy)-6-(((tert-butyldimethylsilyl)oxy)methyl)tetrahydro-2*H*-pyran-2-yl)oxy)-10,13,17-trimethyl-1,2,6,7,8,9,10,11,12,13,14,15,16,17-tetradecahydro-3*H*-cyclopenta[*a*]24henanthrene-3-one
The title product compound is prepared according to the general procedure with 3 mol% catalyst **A** and 0.3 mmol glycal at 30 ˚C for 72 h and isolated by flash column chromatography (20:1 Pentane: Ethyl Acetate) giving a pale yellow syrup (114 mg, 0.14 mmol, 72% yield, α/β ratio 80:20).

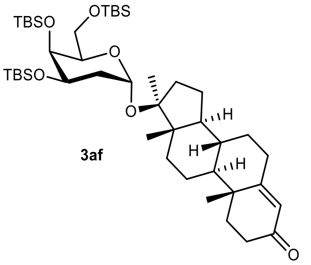

3af

^{1}H NMR (500 MHz, $CDCl_3$) δ 5.71 (s, 1 H), 5.10 (s, 1 H), 4.07-4.05 (m, 1 H), 3.81 (s, 1 H), 3.76-3.74 (m, 1 H), 3.65-3.61 (m, 1 H), 3.58-3.56 (m, 1 H), 2.44-2.30 (m, 3 H), 2.27-2.24 (m, 1 H), 2.12-2.07 (m, 1 H), 2.03-2.00 (m, 1 H), 1.87-1.82 (m, 2 H), 1.71-1.65 (m, 1 H), 1.61-1.39 (m, 7 H), 1.32-1.28 (m, 2 H), 1.22 (s, 3 H), 1.19-1.18 (m, 4 H), 1.02-0.97 (m, 1 H), 0.90-0.85 (m, 31 H), 0.09-0.01 (m, 18 H) ppm. **^{13}C NMR** (126 MHz, $CDCl_3$) δ 199.66, 171.49, 123.94, 94.33, 86.51, 72.40, 70.54, 68.54, 62.80, 53.96, 49.17, 46.33, 38.78, 36.28, 35.85, 35.39, 34.97, 34.09, 32.99, 32.95, 31.91, 31.80, 26.36, 26.27, 25.98, 23.51, 20.79, 18.68, 18.64, 18.32, 17.50, 14.55, -3.87, -4.28, -4.59, -4.77, -5.16, -5.31 ppm. **ESI-HRMS**: Calculated for $C_{44}H_{83}O_6Si_3$ $(M+H)^+$: 791.54920, Found: 791.54983. $[\alpha]_D^{20}$ = +42.7 (c = 0.18, CH_2Cl_2).

(8*R*,9*S*,10*R*,13*S*,14*S*,17*S*)-17-(((2*S*,4*S*,5*R*,6*S*)-4,5-Bis((*tert*-butyldimethylsilyl)oxy)-6-(((tert-butyldimethylsilyl)oxy)methyl)tetrahydro-2*H*-pyran-2-yl)oxy)-10,13,17-trimethyl-1,2,6,7,8,9,10,11,12,13,14,15,16,17-tetradecahydro-3*H*-cyclopenta[*a*]24henanthrene-3-one
The title product compound is prepared according to the general procedure with 3 mol% catalyst **A** and 0.3 mmol at 30 ˚C for 72 h and isolated by flash column chromatography (20:1 Pentane: Ethyl Acetate) giving a pale yellow syrup (86 mg, 0.11 mmol, 54% yield, α/β ratio 93:7).

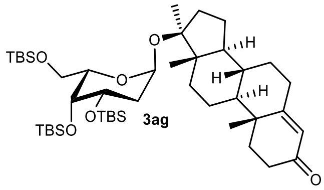

3ag

^{1}H NMR (500 MHz, $CDCl_3$) δ 5.71 (s, 1 H), 5.19 (s, 1 H), 4.11-4.07 (m, 1 H), 3.81-3.77 (m, 2 H), 3.67-3.58 (m, 2 H), 2.45-2.31 (m, 3 H), 2.27-2.18 (m, 2 H), 2.11 (td, *J* = 11.9, 3.3 Hz, 1 H), 2.03-2.00 (m, 1 H), 1.85-1.82 (m, 1 H), 1.68 (td, *J* = 14.0, 4.8 Hz, 1 H), 1.60-1.39 (m, 8 H), 1.34 (dd, *J* = 12.2, 4.1 Hz, 1 H), 1.30-1.22 (m, 2 H), 1.19 (s, 3 H), 1.16 (s, 3 H), 1.03-0.97 (m, 1 H), 0.90-0.87 (m, 30 H), 0.08-0.03 (m, 18 H) ppm. **^{13}C NMR** (126 MHz, $CDCl_3$) δ 199.69, 171.51, 123.95, 93.28, 86.02, 72.97, 70.71, 68.54, 63.06, 54.01, 49.27, 46.94, 38.79, 36.29, 35.86, 35.42, 35.12, 34.10, 32.99, 32.18, 31.92, 26.36, 26.25, 25.99, 23.70, 22.06, 20.84, 18.72, 18.68, 18.34, 17.53, 14.68, -3.87, -4.30, -4.65, -4.81, -5.16, -5.29 ppm. **ESI-HRMS**: Calculated for $C_{44}H_{83}O_6Si_3$ $(M+H)^+$: 791.54920, Found: 791.54952. $[\alpha]_D^{20}$ = -6.4 (c = 0.22, CH_2Cl_2).

Methyl *O*-((2*S*,4*R*,5*S*,6*R*)-4,5-bis((*tert*-butyldimethylsilyl)oxy)-6-(((*tert*-butyldimethylsilyl)oxy)methyl)tetrahydro-2*H*-pyran-2-yl)-*N*-(*tert*-butoxycarbonyl)-L-serinate
The title product compound is prepared according to the general procedure with 3 mol% catalyst **A** and 0.3 mmol at 30 ˚C for 48 h and isolated by flash column chromatography (20:1 Pentane: Ethyl Acetate) giving a pale yellow syrup (106 mg, 0.15 mmol, 75% yield, α/β ratio >20:1).

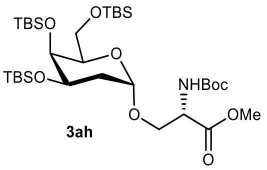

3ah

^{1}H NMR (500 MHz, $CDCl_3$) δ 5.29 (d, *J* = 9.1 Hz, 1 H), 4.81-4.80 (m, 1 H), 4.44-4.41 (m, 1 H), 3.97-3.94 (m, 1 H), 3.81-3.79 (m, 3 H), 3.72-3.71 (m, 3 H), 3.65-3.63 (m, 2 H), 3.58-3.56 (m, 1 H), 2.09-2.02 (m, 1 H), 1.56-1.53 (m, 1 H), 1.43 (s, 9 H), 0.89-0.87 (m, 27 H), 0.08-0.04 (m, 18 H) ppm. **^{13}C NMR** (126 MHz, $CDCl_3$) δ 171.29, 155.55, 98.73, 80.01, 73.28, 70.12, 68.22, 68.08, 62.45, 53.98, 52.42, 33.53, 28.42, 26.30 (two carbons), 26.20, 25.95, 18.66, 18.27, -3.82, -4.34, -4.67, -4.87, -5.14, -5.25 ppm. **ESI-pos HRMS**: Calculated for $C_{33}H_{69}O_9NnaSi_3$ $(M+Na)^+$: 730.41723, Found: 730.41939. $[\alpha]_D^{20}$ = +44.3 (c = 0.42, CH_2Cl_2).

Methyl *O*-((2*S*,4*R*,5*S*,6*R*)-4,5-bis((*tert*-butyldimethylsilyl)oxy)-6-(((*tert*-butyldimethylsilyl)oxy)methyl)tetrahydro-2*H*-pyran-2-yl)-*N*-(*tert*-butoxycarbonyl)-L-threoninate

The title product compound is prepared according to the general procedure with 3 mol% catalyst **A** and 0.3 mmol glycal at 30 ˚C for 48 h and isolated by flash column chromatography (20:1 Pentane: Ethyl Acetate) giving a white solid (110 mg, 0.15 mmol, 76% yield, α/β ratio >20:1).

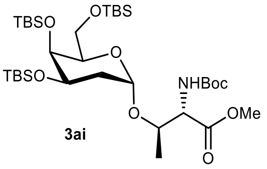

3ai

^{1}H NMR (500 MHz, $CDCl_3$) δ 5.04 (d, *J* = 9.5 Hz, 1 H), 4.81 (s, 1 H), 4.25-4.20 (m, 2 H), 3.94 (d, *J* = 10.7 Hz, 1 H), 3.79-3.77 (m, 1 H), 3.78 (s, 3 H), 3.70-3.62 (m, 3 H), 2.04-1.99 (m, 1 H), 1.47-1.44 (m, 1 H), 1.44 (s, 9 H), 1.26-1.25 (m, 3 H), 0.89-0.86 (m, 27 H), 0.07-0.02 (m, 18 H) ppm. **^{13}C NMR** (126 MHz, $CDCl_3$) δ 171.65, 156.14, 99.94, 79.97, 75.82, 73.27, 70.41, 68.18, 62.98, 58.51, 52.33, 34.00, 28.41, 26.29 (*two carbons*), 26.18, 25.96, 18.69, 18.64, 18.32, -3.82, -4.29, -4.63, -4.88, -5.23, -5.34 ppm. **ESI-HRMS**: Calculated for $C_{34}H_{72}O_9Nsi_3$ $(M+H)^+$: 722.45094, Found: 722.45157. $[\alpha]_D^{20}$ = +44.2 (c = 0.14, CH_2Cl_2).

Methyl (*S*)-3-(4-(((2*R*,4*R*,5*S*,6*R*)-4,5-bis((*tert*-butyldimethylsilyl)oxy)-6-(((*tert*-butyldimethylsilyl)oxy)methyl)tetrahydro-2*H*-pyran-2-yl)oxy)phenyl)-2-((*tert*-butoxycarbonyl)amino)propanoate

The title product compound is prepared according to the general procedure with 10 mol% catalyst **A** and 0.3 mmol glycal at 40 ˚C for 96 h and isolated by flash column chromatography (20:1 Pentane: Ethyl Acetate) giving a pale yellow syrup (117 mg, 0.15 mmol, 75% yield, α/β ratio >20:1).

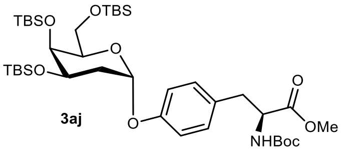

3aj

^{1}H NMR (500 MHz, $CDCl_3$) δ 7.01-6.97 (m, 4 H), 5.53 (d, *J* = 2.3 Hz, 1 H), 4.95 (d, *J* = 7.5 Hz, 1 H), 4.535-4.51 (m, 1 H), 4.24-4.20 (m, 1 H), 3.89-3.88 (m, 1 H), 3.78-3.76 (m, 1 H), 3.69 (s, 3 H), 3.67-3.62 (m, 2 H), 3.04-2.99 (m, 2 H), 2.25 (td, *J* = 12.2, 3.5 Hz, 1 H), 1.81 (dd, *J* = 12.5, 4.2 Hz, 1 H), 1.42 (s, 9 H), 0.94 (s, 9 H), 0.91 (s, 9 H), 0.84 (s, 9 H), 0.13 (s, 6 H), 0.12 (s, 3 H), 0.09 (s, 3 H), -0.00 (s, 3 H), -0.02 (s, 3 H) ppm. **^{13}C NMR** (126 MHz, $CDCl_3$) δ 172.47, 156.55, 155.22, 130.22, 129.18, 117.22, 97.44, 79.95, 73.71, 70.27, 68.27, 62.69, 54.61, 52.25, 37.55, 33.83, 28.43, 26.33, 26.25, 25.93, 18.70, 18.67, 18.26, -3.74, -4.24, -4.59, -4.80, -5.20, -5.29 ppm. **ESI-HRMS**: Calculated for $C_{39}H_{73}O_9NnaSi_3$ $(M+Na)^+$: 806.44853, Found: 806.44853. $[\alpha]_D^{20}$ = +74.2 (c = 0.40, CH_2Cl_2).

(((2*R*,3*S*,4*R*,6*S*)-2-(((*Tert*-Butyldimethylsilyl)oxy)methyl)-6-(prop-2-yn-1-yloxy)tetrahydro-2*H*-pyran-3,4-diyl)bis(oxy))bis(*tert*-butyldimethylsilane)

The title product compound is prepared according to the general procedure with 3 mol% catalyst **A** and 0.3 mmol at 30 ˚C for 48 h and isolated by flash column chromatography (3:1 Pentane: CH_2Cl_2) giving a pale yellow syrup (92 mg, 0.17 mmol, 84% yield, α/β ratio >20:1).

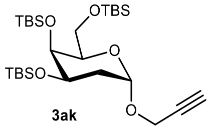

3ak

^{1}H NMR (500 MHz, $CDCl_3$) δ 5.09 (d, *J* = 3.4 Hz, 1 H), 4.22-4.15 (m, 2 H), 4.00 (ddd, *J* = 11.7, 4.5, 2.4 Hz, 1 H), 3.82 (s, 1 H), 3.67-3.66 (m, 2 H), 3.62-3.59 (m, 1 H), 2.37-2.36 (m, 1 H), 2.12 (td, *J* = 12.3, 3.8 Hz, 1 H), 1.66-1.62 (m, 1 H), 0.90-0.89 (m, 27 H), 0.11-0.05 (m, 18 H) ppm. **^{13}C NMR** (126 MHz, $CDCl_3$) δ 96.38, 79.89, 74.03, 73.19, 70.27, 68.32, 62.81, 53.72, 33.32, 26.36, 26.25, 25.99, 18.72, 18.64, 18.33, -3.70, -4.19, -4.52, -4.80, -5.14, -5.21 ppm. **ESI-pos HRMS**: Calculated for $C_{27}H_{57}O_5Si_3$ $(M+H)^+$: 545.35083, Found: 545.35219. $[\alpha]_D^{20}$ = +73.0 (c = 0.19, CH_2Cl_2).

(((2*R*,3*S*,4*R*,6*S*)-2-(((*Tert*-Butyldimethylsilyl)oxy)methyl)-6-isopropoxytetrahydro-2*H*-pyran-3,4-diyl)bis(oxy))bis(*tert*-butyldimethylsilane)

The title product compound is prepared according to the general procedure with 3 mol% catalyst **A** at 30 ˚C for 48 h and isolated by flash column chromatography (3:1 Pentane: CH_2Cl_2) giving a pale yellow syrup (96 mg, 0.17 mmol, 88% yield, α/β ratio >20:1).

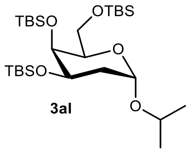

3al

^{1}H NMR (500 MHz, $CDCl_3$) δ 4.98 (d, *J* = 2.7 Hz, 1 H), 4.05-4.02 (m, 1 H), 3.86-3.81 (m, 2 H), 3.39-3.61 (m, 3 H), 2.07 (td, *J* = 12.1, 3.7 Hz, 1 H), 1.54 (dd, *J* = 12.3, 4.3 Hz, 1 H), 1.16 (d, *J* = 6.3 Hz, 3 H), 1.09 (d, *J* = 6.3 Hz, 3 H), 0.91-0.89 (m, 27 H), 0.10-0.05 (m, 18 H) ppm. **^{13}C NMR** (126 MHz, $CDCl_3$) δ 95.93, 72.71, 70.52, 68.58, 68.38, 62.90, 34.31, 26.37, 26.30, 25.99, 23.53, 21.66, 18.74, 18.66, 18.35, -3.73, -4.19, -4.53, -4.76, -5.16, -5.24 ppm. **ESI-pos HRMS**: Calculated for C27H60O5NaSi3 $(M+Na)^+$: 571.36408, Found: 571.36626. $[\alpha]_D^{20}$ = +60.6 (c = 0.22, CH_2Cl_2).

(((2R,3R,4R,6S)-3,4-bis(benzyloxy)-6-(((2R,3R,4S,5R,6S)-3,4,5-tris(benzyloxy)-6-methoxytetrahydro-2H-pyran-2-yl)methoxy)tetrahydro-2H-pyran-2-yl)methoxy)triisopropylsilane

The title product compound is prepared according to the general procedure with 3 mol% catalyst **A** at 30 ˚C for 48 h and isolated by flash column chromatography (20:1 Pentane: Ethyl acetate) giving a pale yellow syrup (160 mg, 0.17 mmol, 85% yield, α/β ratio >20:1).

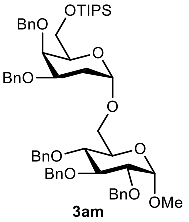

3am

^{1}H NMR (400 MHz, $CDCl_3$) δ 7.38-7.22 (m, 25 H), 5.00-4.92 (m, 3 H), 4.83-4.77 (m, 3 H), 4.69-4.66 (m, 2 H), 4.57-4.52 (m, 4 H), 3.97 (t, *J* = 9.2 Hz, 1 H), 3.91-3.90 (m, 1 H), 3.88-3.84 (m, 1 H), 3.76-3.69 (m, 5 H), 3.65-3.62 (m, 1 H), 3.48 (dd, *J* = 9.6, 3.5 Hz, 1 H), 3.42-3.37 (m, 1 H), 3.29 (s, 3 H), 2.19 (td, *J* = 12.2, 3.6 Hz, 1 H), 1.98 (dd, *J* = 12.6, 4.5 Hz, 1 H), 1.07-0.99 (m, 21 H) ppm; ^{13}C NMR (101 MHz, $CDCl_3$) δ 139.23, 138.86, 138.61, 138.61, 138.31, 128.58, 128.51 (three carbons), 128.26, 128.24, 128.16 (two carbons), 128.01, 127.84, 127.80, 127.75, 127.66, 127.50, 127.48, 98.09, 97.86, 82.23, 80.12, 78.42, 75.95, 75.13, 74.46 (two carbons), 73.40, 73.03, 72.06, 70.26, 70.09, 65.93, 62.81, 54.99, 31.23, 18.11, 12.00 ppm. $[\alpha]_D^{20}$ = +63.3 (c = 1.12, CH_2Cl_2). The data is in accordance to the literature[2].

(5a*S*,6*S*,8*S*,9a*S*)-8-(((2*R*,4a*R*,6*S*,7*R*,8*S*,8a*R*)-7-(Benzyloxy)-6-methoxy-2-phenylhexahydropyrano[3,2-*d*][1,3]dioxin-8-yl)oxy)-2,2,4,4-tetraisopropyl-6-methyltetrahydro-6*H*-pyrano[3,4-*f*][1,3,5,2,4]trioxadisilepine

The title product compound is prepared according to the general procedure with 5 mol% catalyst **A** and 0.4 mmol glycal at 30 ˚C for 108 h and isolated by flash column chromatography (20:1 Pentane: Ethyl Acetate) giving a pale yellow syrup (99 mg, 0.13 mmol, 66% yield, α/β ratio 95:5).

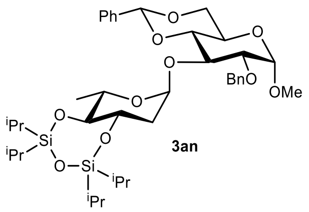

3an

^{1}H NMR (500 MHz, $CDCl_3$) δ 7.49-7.47 (m, 2 H), 7.36-7.31 (m, 8 H), 5.52 (s, 1 H), 5.20 (d, *J* = 3.3 Hz, 1 H), 4.72 (d, *J* = 12.1 Hz, 1 H), 4.63 (d, *J* = 12.1 Hz, 1 H), 4.60 (d, *J* = 2.4 Hz, 1 H), 4.27 (dd, *J* = 10.2, 4.8 Hz, 1H), 4.16-4.10 (m, 1 H), 3.99-3.92 (m, 1 H), 3.90-3.80 (m, 2 H), 3.69 (t, *J* = 10.3 Hz, 1 H), 3.53-3.47 (m, 2 H), 3.39 (s, 3 H), 3.17-3.13 (m, 1 H), 2.08-2.05 (m, 1 H), 1.71-1.65 (m, 1 H), 1.11-0.89 (m, 28 H), 0.78 (d, *J* = 6.3 Hz, 3 H) ppm. **^{13}C NMR** (126 MHz, $CDCl_3$) δ 137.94, 137.37, 129.06, 128.65, 128.21 (two carbons), 128.19, 126.44, 102.07, 98.82, 98.27, 80.36, 80.31, 80.04, 74.39, 73.32, 71.64, 69.20, 67.76, 62.78, 55.38, 38.70, 17.83, 17.54, 17.51 (three carbons), 17.48, 17.45, 17.43 (two carbons), 13.04, 13.02, 12.38, 12.32 ppm. **ESI-HRMS**: Calculated for $C_{39}H_{60}O_{10}NaSi_2$ $(M+Na)^+$: 767.36172, Found: 767.36194. $[\alpha]_D^{20}$ = -27.0 (c = 0.59, CH_2Cl_2).

(5a*S*,6*S*,8*S*,9a*S*)-8-(((2*R*,3*S*,4*S*,5*R*,6*R*)-2-Allyl-4,5-bis(benzyloxy)-6-((benzyloxy)methyl)tetrahydro-2*H*-pyran-3-yl)oxy)-2,2,4,4-tetraisopropyl-6-methyltetrahydro-6*H*-pyrano[3,4-*f*][1,3,5,2,4]trioxadisilepine

The title product compound is prepared according to the general procedure with 5 mol% catalyst **A** and 0.4 mmol glycal at 30 ˚C for 48 h and isolated by flash column chromatography (20:1 Pentane: Ethyl Acetate) giving a pale yellow syrup (150 mg, 0.18 mmol, 88% yield, α/β ratio >20:1).

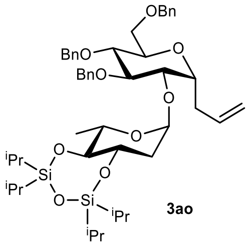

3ao

^{1}H NMR (500 MHz, CD_2Cl_2) δ 7.36-7.21 (m, 15 H), 5.93-5.88 (m, 1 H), 5.17-5.14 (m, 2 H), 5.11 (d, *J* = 10.2 Hz, 1 H), 4.84-4.81 (m, 3 H), 4.59-4.55 (m, 2 H), 4.50 (d, *J* = 11.9 Hz, 1 H), 4.06-3.98 (m, 2 H), 3.85 (dd, *J* = 9.6, 6.0 Hz, 1 H), 3.77 (t, *J* = 9.2 Hz, 1 H), 3.72 (dd, *J* = 10.4, 3.7 Hz, 1 H), 3.68-3.64 (m, 2 H), 3.63-3.57 (m, 2 H), 3.27 (t, *J* = 8.7 Hz, 1 H), 2.60-2.53 (m, 1 H), 2.47-2.43 (m, 1 H), 2.09 (dd, *J* = 13.4, 5.1 Hz, 1 H), 1.73-1.68 (m, 1 H), 1.28 (d, *J* = 6.3 Hz, 3 H), 1.12-0.93 (m, 28 H) ppm. **^{13}C NMR** (126 MHz, CD_2Cl_2) δ 139.34, 139.07, 138.98, 135.71, 128.91, 128.85, 128.83, 128.45, 128.34, 128.29, 128.16, 128.12, 128.09, 117.07, 100.01, 82.79, 80.50, 79.11, 78.79, 75.98, 75.74, 75.44, 73.83, 71.99, 71.90, 69.89, 69.52, 39.10, 30.38, 18.33, 18.01, 17.77 (two carbons), 17.72, 17.70 (two carbons), 17.60, 13.55, 13.48, 12.89, 12.88 ppm. **ESI-HRMS**: Calculated for $C_{48}H_{70}O_9NaSi_2$ $(M+Na)^+$: 869.44506, Found: 869.44511. $[\alpha]_D^{20}$ = -3.9 (c = 0.34, CH_2Cl_2).

(5a*S*,6*S*,8*S*,9a*S*)-8-(((2*S*,3*S*,4*S*,5*R*,6*R*)-2-Allyl-4,5-bis(benzyloxy)-6-((benzyloxy)methyl)tetrahydro-2*H*-pyran-3-yl)oxy)-2,2,4,4-tetraisopropyl-6-methyltetrahydro-6*H*-pyrano[3,4-*f*][1,3,5,2,4]trioxadisilepine

The title product compound is prepared according to the general procedure with 5 mol% catalyst **A** and 0.4 mmol glycal at 40 ˚C for 48 h and isolated by flash column chromatography (20:1 Pentane: Ethyl Acetate) giving a pale yellow syrup (142 mg, 0.17 mmol, 83% yield, α/β ratio >20:1).

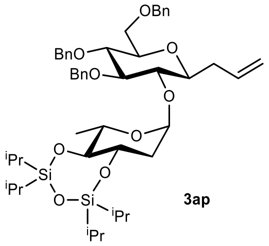

3ap

^{1}H NMR (500 MHz, CD_2Cl_2) δ 7.37-7.28 (m, 13 H), 7.23-7.22 (m, 2 H), 6.02-5.97 (m, 1 H), 5.32 (s, 1 H), 5.19-5.16 (m, 1 H), 5.11 (d, *J* = 10.2 Hz, 1 H), 4.97 (d, *J* = 11.3 Hz, 1 H), 4.79-4.74 (m, 2 H), 4.61 (d, *J* = 11.4 Hz, 2 H), 4.55 (d, *J* = 12.1 Hz, 1 H), 4.00-3.95 (m, 1 H), 3.76-3.70 (m, 3 H), 3.65-3.59 (m, 2 H), 3.48 (t, *J* = 8.9 Hz, 1 H), 3.45-3.42 (m, 1 H), 3.34 (td, *J* = 8.8, 2.7 Hz, 1 H), 3.24 (t, *J* = 8.8 Hz, 1 H), 2.64

(dd, J = 14.6, 6.9 Hz, 1 H), 2.34-2.29 (m, 1 H), 2.03 (dd, J = 13.3, 5.2 Hz, 1 H), 1.66-1.60 (m, 1 H), 1.28 (d, J = 6.3 Hz, 3 H), 1.11-0.91 (m, 28 H) ppm. **^{13}C NMR** (126 MHz, CD_2Cl_2) δ 139.17, 139.05, 138.95, 135.66, 128.92, 128.86 (two carbons), 128.38, 128.32, 128.18, 128.08, 128.07, 127.92, 117.04, 99.64, 88.01, 80.52, 79.95, 79.51, 79.43, 78.89, 75.69, 75.25, 73.86, 71.91, 69.65, 69.36, 39.49, 36.56, 18.24, 18.02, 17.79 (two carbons), 17.71 (four carbons), 17.62, 13.52, 13.50, 12.92, 12.87 ppm. **ESI-HRMS**: Calculated for $C_{48}H_{70}O_9NaSi_2$ $(M+Na)^+$: 869.44506, Found: 869.44480. $[\alpha]_D^{20}$ = -33.4 (c = 0.27, CH_2Cl_2).

(5a*S*,6*S*,8*R*,9a*S*)-2,2,4,4-Tetraisopropyl-6-methyl-8-(((2*R*,3*R*,4*S*,5*R*,6*S*)-3,4,5-tris(benzyloxy)-6-methoxytetrahydro-2*H*-pyran-2-yl)methoxy)tetrahydro-6*H*-pyrano[3,4-*f*][1,3,5,2,4]trioxadisilepine

The title product compound is prepared according to the general procedure with 5 mol% catalyst **A** and 0.4 mmol glycal at 30 ˚C for 96 h and isolated by flash column chromatography (20:1 Pentane: Ethyl Acetate) giving a pale yellow syrup (85 mg, 0.10 mmol, 51% yield, α/β ratio 86:14).

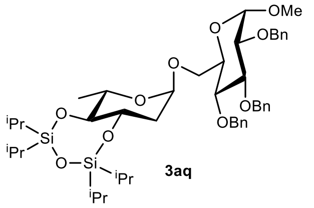

3aq

^{1}H NMR (500 MHz, CD_2Cl_2) δ 7.38-7.27 (m, 15 H), 4.95 (d, J = 11.0 Hz, 1 H), 4.87 (d, J = 11.1 Hz, 1 H), 4.80 (d, J = 10.9 Hz, 1 H), 4.77 (d, J = 3.1 Hz, 1 H), 4.75-4.71 (m, 2 H), 4.65 (d, J = 11.7 Hz, 1 H), 4.56 (d, J = 11.1 Hz, 1 H), 4.02-3.97 (m, 1 H), 3.92 (t, J = 9.2 Hz, 1 H), 3.85 (dd, J = 10.7, 1.8 Hz, 1 H), 3.77-3.74 (m, 1 H), 3.64-3.61 (m, 1 H), 3.52 (dd, J = 9.6, 3.5 Hz, 1 H), 3.45 (dd, J = 10.8, 6.3 Hz, 1 H), 3.41 (d, J = 8.7 Hz, 1 H), 3.37 (s, 3 H), 3.25-3.22 (m, 1 H), 2.07 (dd, J = 13.1, 5.2 Hz, 1 H), 1.71-1.64 (m, 1 H), 1.24 (d, J = 6.3 Hz, 3 H), 1.11-0.89 (m, 28 H) ppm. **^{13}C NMR** (126 MHz, CD_2Cl_2) δ 139.58, 139.09, 139.00, 128.90, 128.84, 128.79, 128.46, 128.42, 128.28 (two carbons), 128.16, 128.01, 98.11, 97.99, 82.44, 80.93, 80.63, 78.76, 75.95, 75.37, 73.44, 71.97, 70.80, 68.51, 66.59, 55.19, 38.94, 18.36, 17.91, 17.78 (two carbons), 17.72 (two carbons), 17.68, 17.63, 17.58, 13.51, 13.47, 12.88, 12.85 ppm. **ESI-HRMS**: Calculated for $C_{46}H_{68}O_{10}NaSi_2$ $(M+Na)^+$: 859.42432, Found: 859.42415. $[\alpha]_D^{20}$ = -7.3 (c = 0.29, CH_2Cl_2).

(5a*S*,6*S*,8*R*,9a*S*)-2,2,4,4-Tetraisopropyl-6-methyl-8-(((3a*R*,5*R*,5a*R*,8a*S*,8b*R*)-2,2,7,7-tetramethylhexahydro-5*H*-[1,3]dioxolo[4,5-*b*]furo[3,2-*d*]pyran-5-yl)methoxy)tetrahydro-6*H*-pyrano[3,4-*f*][1,3,5,2,4]trioxadisilepine

The title product compound is prepared according to the general procedure with 5 mol% catalyst **A** and 0.4 mmol glycal at 40 ˚C for 48 h and isolated by flash column chromatography (20:1 Pentane: Ethyl Acetate) giving a pale yellow syrup (111 mg, 0.18 mmol, 88% yield, α/β ratio 88:12).

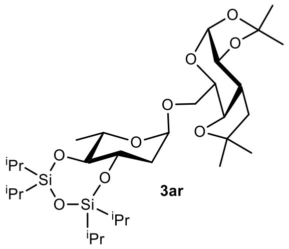

3ar

^{1}H NMR (500 MHz, $CDCl_3$) δ 5.51 (d, J = 4.9 Hz, 1 H), 4.86 (d, J = 3.4 Hz, 1 H), 4.59 (dd, J = 8.0, 2.4 Hz, 1 H), 4.29 (dd, J = 5.0, 2.4 Hz, 1 H), 4.23 (dd, J = 8.0, 1.8 Hz, 1 H), 3.99-3.94 (m, 2 H), 3.78-3.74 (m, 1 H), 3.70-3.67 (m, 1 H), 3.50 (dd, J = 10.1, 6.8 Hz, 1 H), 3.23-3.19 (m, 1 H), 2.10 (dd, J = 13.2, 5.3 Hz, 1 H), 1.70-1.64 (m, 1 H), 1.51 (s, 3 H), 1.42 (s, 3 H), 1.32 (s, 3 H), 1.30 (s, 3 H), 1.24 (d, J = 6.4 Hz, 3 H), 1.10-0.98 (m, 26 H), 0.94-0.82 (m, 2 H) ppm. **^{13}C NMR** (126 MHz, $CDCl_3$) δ 109.28, 108.68, 97.22, 96.31, 80.06, 71.61, 71.18, 70.80, 70.68, 68.10, 67.12, 65.23, 38.38, 26.16, 26.02, 25.11, 24.29, 23.98, 20.93, 18.01, 17.77, 17.51, 17.47, 17.46, 17.36, 13.07, 12.98, 12.41, 12.36, 12.32 ppm. **ESI-HRMS**: Calculated for $C_{30}H_{56}O_{10}NaSi_2$ $(M+Na)^+$: 655.33042, Found: 655.33077. $[\alpha]_D^{20}$ = -68.1 (c = 0.32, CH_2Cl_2).

(8*R*,9*S*,10*R*,13*S*,14*S*,17*S*)-10,13-Dimethyl-17-(((5a*S*,6*S*,8*R*,9a*S*)-2,2,4,4-tetraisopropyl-6-methyltetrahydro-6*H*-pyrano[3,4-*f*][1,3,5,2,4]trioxadisilepin-8-yl)oxy)-1,2,6,7,8,9,10,11,12,13,14,15,16,17-tetradecahydro-3*H*-cyclopenta[*a*]28henanthrene-3-one

The title product compound is prepared according to the general procedure with 5 mol% catalyst **A** and 0.4 mmol glycal at 30 ˚C for 48 h and isolated by flash column chromatography (20:1 to 9:1 Pentane:

Ethyl Acetate) giving a pale yellow syrup (124 mg for two anomers, 0.19 mmol, 94% yield, α/β ratio 79:21).

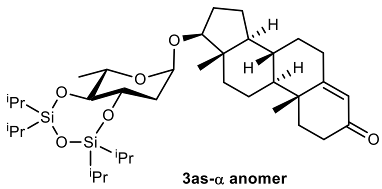

3as-α anomer

^{1}H NMR (500 MHz, $CDCl_3$) δ 5.71 (d, *J* = 4.9 Hz, 1 H), 4.83 (s, 1 H), 3.99-3.96 (m, 1 H), 3.66-3.61 (m, 1 H), 3.49-3.44 (m, 1 H), 3.22-3.17 (m, 1 H), 2.44-2.30 (m, 3 H), 2.27-2.24 (m, 1 H), 2.08-1.96 (m, 3 H), 1.84-1.79 (m, 2 H), 1.71-1.65 (m, 2 H), 1.62-1.50 (m, 4 H), 1.45-1.36 (m, 1 H), 1.29-1.21 (m, 5 H), 1.18-1.16 (m, 3 H), 1.07-0.87 (m, 31 H), 0.77-0.76 (m, 3 H) ppm. **^{13}C NMR** (126 MHz, $CDCl_3$) δ 199.72, 171.48, 123.96, 98.31, 87.46, 80.18, 71.53, 68.30, 54.00, 50.29, 42.92, 38.92, 38.76, 37.20, 35.81, 35.57, 34.05, 32.91, 31.62, 28.62, 23.48, 20.70, 18.05, 17.63, 17.54 (two carbons), 17.51 (three carbons), 17.48, 17.40 (two carbons), 13.06, 13.02, 12.50, 12.44, 11.65 ppm. **ESI-HRMS**: Calculated for $C_{37}H_{65}O_6Si_2$ $(M+H)^+$: 661.43142, Found: 661.43169. $[\alpha]_D^{20}$ = -11.3 (c = 0.50, CH_2Cl_2).

(8*R*,9*S*,10*R*,13*S*,14*S*,17*S*)-10,13-Dimethyl-17-(((5a*S*,6*S*,8*S*,9a*S*)-2,2,4,4-tetraisopropyl-6-methyltetrahydro-6*H*-pyrano[3,4-*f*][1,3,5,2,4]trioxadisilepin-8-yl)oxy)-1,2,6,7,8,9,10,11,12,13,14,15,16,17-tetradecahydro-3*H*-cyclopenta[a]29henanthrene-3-one

The title product compound is prepared according to the general procedure with 5 mol% catalyst **A** and 0.4 mmol glycal at 30 ˚C for 48 h and isolated by flash column chromatography (20:1 to 9:1 Pentane: Ethyl Acetate) giving a pale yellow syrup (124 mg for two anomers, 0.19 mmol, 94% yield, α/β ratio 79:21).

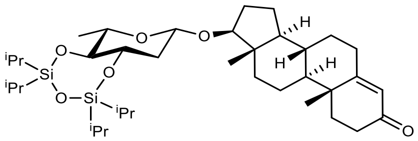

3as-β anomer

^{1}H NMR (500 MHz, $CDCl_3$) δ 5.72 (s, 1 H), 4.43 (d, *J* = 9.8 Hz, 1 H), 3.70-3.65 (m, 2 H), 3.27-3.24 (m, 1 H), 3.22-3.18 (m, 1 H), 2.41-2.34 (m, 3 H), 2.28-2.27 (m, 1 H), 2.11-2.08 (m, 1 H), 2.04-1.97 (m, 3 H), 1.84-1.81 (m, 1 H), 1.72-1.62 (m, 2 H), 1.61-1.51 (m, 4 H), 1.45-1.39 (m, 1 H), 1.32-1.25 (m, 5 H), 1.18 (s, 3 H), 1.06-0.87 (m, 31 H), 0.79 (s, 3 H) ppm. **^{13}C NMR** (126 MHz, $CDCl_3$) δ 199.91, 171.72, 123.93, 98.74, 87.08, 79.52, 74.26, 72.19, 54.11, 50.76, 42.52, 40.40, 38.80, 36.66, 35.84, 35.61, 34.09, 32.97, 31.71, 27.71, 23.34, 20.71, 18.17, 17.78, 17.54, 17.53, 17.50, 17.46 (two carbons), 17.45, 17.44, 17.35, 13.00 (two carbons), 12.36, 12.35, 11.76 ppm. **ESI-HRMS**: Calculated for $C_{37}H_{65}O_6Si_2$ $(M+H)^+$: 661.43142, Found: 661.43167. $[\alpha]_D^{20}$ = +34.2 (c = 0.15, CH_2Cl_2).

Methyl *N*-(*tert*-butoxycarbonyl)-*O*-((5a*S*,6*S*,8*R*,9a*S*)-2,2,4,4-tetraisopropyl-6-methyltetrahydro-6*H*-pyrano[3,4-*f*][1,3,5,2,4]trioxadisilepin-8-yl)-L-serinate

The title product compound is prepared according to the general procedure with 5 mol% catalyst **A** at 50 ˚C for 72 h and isolated by flash column chromatography (20:1 to 9:1 Pentane: Ethyl Acetate) giving a pale yellow syrup (70 mg, 0.12 mmol, 58% yield, α/β ratio 88:12).

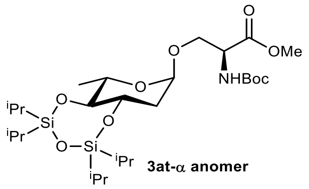

3at-α anomer

^{1}H NMR (500 MHz, $CDCl_3$) δ 5.34 (d, *J* = 8.9 Hz, 1 H), 4.78 (s, br, 1 H), 4.52-4.92 (m, 1 H), 4.04-4.00 (m, 1 H), 3.88-3.82 (m, 1 H), 3.72 (s, 3 H), 3.55-3.51 (m, 1 H), 3.45-3.38 (m, 1 H), 3.23-3.17 (m, 1 H), 2.05 (dd, *J* = 13.5, 5.3 Hz, 1 H), 1.69-1.63 (m, 1 H), 1.45 (s, 9 H), 1.26 (d, *J* = 6.2 Hz, 3 H), 1.08-0.88 (m, 28 H) ppm. **^{13}C NMR** (126 MHz, $CDCl_3$) δ 170.86, 155.52, 97.20, 80.22, 79.77, 71.42, 68.37, 67.15, 53.77, 52.33, 38.14, 28.44, 18.04, 17.74, 17.51 (two carbons), 17.47 (three carbons), 17.44, 17.36, 13.04, 12.99, 12.41, 12.26 ppm. **ESI-pos HRMS**: Calculated for $C_{27}H_{54}O_9NSi_2$ $(M+H)^+$: 592.33316, Found: 592.33316. $[\alpha]_D^{20}$ = -39.8 (c = 0.41, CH_2Cl_2).

Methyl *N*-(*tert*-butoxycarbonyl)-*O*-((5a*S*,6*S*,8*S*,9a*S*)-2,2,4,4-tetraisopropyl-6-methyltetrahydro-6*H*-pyrano[3,4-*f*][1,3,5,2,4]trioxadisilepin-8-yl)-L-serinate

The title product compound is prepared according to the general procedure with 5 mol% catalyst **A** at 50 ˚C for 72 h and isolated by flash column chromatography (20:1 to 9:1 Pentane: Ethyl Acetate) giving a pale yellow syrup (70 mg, 0.12 mmol, 58% yield, α/β ratio 88:12).

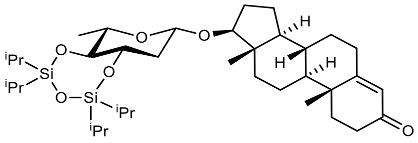

3at-β anomer

^{1}H NMR (500 MHz, $CDCl_3$) δ 5.90 (d, *J* = 8.9 Hz, 1 H), 4.42 (dd, *J* = 9.9, 2.0 Hz, 1 H), 4.37 (dt, *J* = 9.0, 2.9 Hz, 1 H), 4.14 (dd, *J* = 10.7, 3.0 Hz, 1 H), 3.92 (dd, *J* = 10.7, 2.9 Hz, 1 H), 3.75 (s, 3 H), 3.69-3.64 (m, 1 H), 3.29-3.21 (m, 2 H), 2.10 (ddd, *J* = 13.0, 5.3, 2.0 Hz, 1 H), 1.67-1.60 (m, 1 H), 1.45 (s, 9 H), 1.35 (d, *J* = 5.7 Hz, 3 H), 1.09-0.92 (m, 28 H) ppm. **^{13}C NMR** (126 MHz, $CDCl_3$) δ 171.15, 155.79, 100.33, 79.84, 79.29, 73.88, 72.48, 70.61, 54.12, 52.58, 39.64, 28.48, 18.00, 17.75, 17.54, 17.51, 17.45 (two carbons), 17.44, 17.42, 17.34, 13.00, 12.99, 12.36, 12.35 ppm. **ESI-pos HRMS**: Calculated for $C_{27}H_{54}O_9NSi_2$ $(M+H)^+$: 592.33316, Found: 592.33405. $[\alpha]_D^{20}$ = -13.9 (c = 0.06, CH_2Cl_2).

(5a*R*,8*S*,9a*R*)-2,2,4,4-Tetraisopropyl-8-(((3a*R*,5*R*,5a*S*,8a*S*,8b*R*)-2,2,7,7-tetramethyltetrahydro-5*H*-bis([1,3]dioxolo)[4,5-*b*:4',5'-*d*]pyran-5-yl)methoxy)tetrahydro-6*H*-pyrano[3,4-*f*][1,3,5,2,4]trioxadisilepine

The title product compound is prepared according to the general procedure with 5 mol% catalyst **A** and 0.3 mmol glycal at 40 ˚C for 24 h and isolated by flash column chromatography (20:1 Pentane: Ethyl Acetate) giving a pale yellow syrup (107 mg, 0.17 mmol, 86% yield, α/β ratio 83:17).

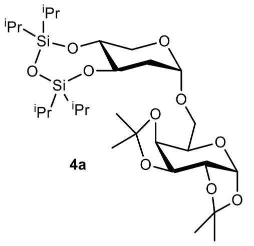

4a

^{1}H NMR (500 MHz, $CDCl_3$) δ 5.52 (d, *J* = 5.0 Hz, 1 H), 4.89 (d, *J* = 3.5 Hz, 1 H), 4.62 (dd, *J* = 8.0, 2.4 Hz, 1 H), 4.31 (dd, *J* = 5.2, 2.4 Hz, 1 H), 4.25 (dd, *J* = 7.9, 1.8 Hz, 1 H), 3.99-3.94 (m, 2 H), 3.73 (dd, *J* = 10.5, 6.4 Hz, 1 H), 3.66-3.59 (m, 3 H), 3.50-3.45 (m, 1 H), 2.11 (dd, *J* = 13.4, 5.4 Hz, 1 H), 1.61 (ddd, *J* = 14.2, 11.1, 3.7 Hz, 1 H), 1.53 (s, 3 H), 1.44 (s, 3 H), 1.34 (s, 3 H), 1.33 (s, 3 H), 1.07-0.90 (m, 28 H) ppm. **^{13}C NMR** (126 MHz, $CDCl_3$) δ 109.42, 108.69, 97.44, 96.51, 74.35, 71.81, 71.17, 70.81, 70.79, 66.06, 65.55, 62.52, 38.00, 26.15, 26.10, 25.11, 24.60, 17.74, 17.70, 17.46 (three carbons), 17.42, 17.41, 17.39, 13.14, 13.09, 12.49, 12.43 ppm. **ESI-HRMS**: Calculated for $C_{29}H_{54}O_{10}NaSi_2$ $(M+Na)^+$: 641.31477, Found: 641.31530. $[\alpha]_D^{20}$ = +17.0 (c = 0.28, CH_2Cl_2).

Methyl *N*-(*tert*-butoxycarbonyl)-*O*-((5a*R*,8*S*,9a*R*)-2,2,4,4-tetraisopropyltetrahydro-6*H*-pyrano[3,4-*f*][1,3,5,2,4]trioxadisilepin-8-yl)-L-serinate

The title product compound is prepared according to the general procedure with 4 mol% catalyst **A** and 0.4 mmol glycal at 40 ˚C for 96 h and isolated by flash column chromatography (20:1 to 9:1 Pentane: Ethyl Acetate) giving a pale yellow syrup (91 mg, 0.16 mmol, 79% yield, α/β ratio 84:16).

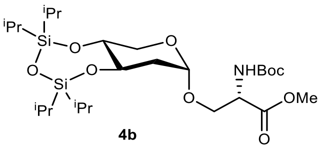

4b

^{1}H NMR (500 MHz, $CDCl_3$) δ 5.33 (d, *J* = 8.8 Hz, 1 H), 4.78 (d, *J* = 3.0 Hz, 1 H), 4.47-4.45 (m, 1 H), 3.92-3.87 (m, 1 H), 3.85 (dd, *J* = 10.2, 3.7 Hz, 1 H), 3.78-3.75 (m, 1 H), 3.75 (s, 3 H), 3.64-3.59 (m, 2 H), 3.39-3.34 (m, 1 H), 2.02 (dd, *J* = 13.4, 5.3 Hz, 1 H), 1.59 (ddd, *J* = 13.5, 11.3, 3.6 Hz, 1 H), 1.45 (s, 9 H), 1.07-0.91 (m, 28 H) ppm. **^{13}C NMR** (126 MHz, $CDCl_3$) δ 171.24, 155.42, 98.08, 80.19, 74.04, 71.68, 67.60, 62.63, 53.92, 52.59, 37.80, 28.41, 17.71, 17.67, 17.44, 17.42 (two carbons), 17.40, 17.38 (two carbons), 13.12, 13.09, 12.45, 12.44 ppm. **ESI-HRMS**: Calculated for $C_{26}H_{51}O_9NnaSi_2$ $(M+Na)^+$: 600.29946, Found: 600.29988. $[\alpha]_D^{20}$ = +53.4 (c = 0.27, CH_2Cl_2).

(5a*R*,8*R*,9a*R*)-8-(((2*R*,4a*R*,6*S*,7*R*,8*S*,8a*R*)-7-(Benzyloxy)-6-methoxy-2-phenylhexahydropyrano[3,2-*d*][1,3]dioxin-8-yl)oxy)-2,2,4,4-tetraisopropyltetrahydro-6*H*-pyrano[3,4-*f*][1,3,5,2,4]trioxadisilepine

The title product compound is prepared according to the general procedure with 5 mol% catalyst **A** and 0.4 mmol glycal at 30 ˚C for 48 h and isolated by flash column chromatography (20:1 to 9:1 Pentane: Ethyl Acetate) giving a pale yellow syrup (100 mg, 0.14 mmol, 68% yield, α/β ratio 85:15).

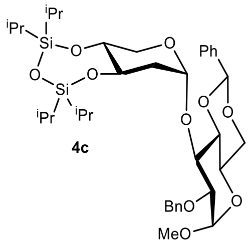

^{1}H NMR (500 MHz, $CDCl_3$) δ 7.46-7.32 (m, 10 H), 5.51 (s, 1 H), 5.43 (s, 1 H), 4.91 (d, *J* = 12.4 Hz, 1 H), 4.62 (d, *J* = 12.4 Hz, 1 H), 4.51-4.49 (m, 1 H), 4.32 (t, *J* = 9.4 Hz, 1 H), 4.24 (dd, *J* = 10.1, 4.8 Hz, 1 H), 4.12-4.07 (m, 1 H), 3.92-3.87 (m, 1 H), 3.84-3.79 (m, 1 H), 3.70-3.64 (m, 3 H), 3.59 (t, *J* = 9.4 Hz, 1 H), 3.46-3.43 (m, 1 H), 3.40 (s, 3 H), 2.18-2.14 (m, 1 H), 1.67-1.61 (m, 1 H), 1.10-0.90 (m, 28 H) ppm. **^{13}C NMR** (126 MHz, $CDCl_3$) δ 138.04, 137.34, 129.09, 128.67, 128.48, 128.38, 128.23, 126.07, 101.38, 99.37, 97.33, 83.26, 77.50, 74.30, 74.15, 72.29, 72.02, 69.16, 62.71, 62.04, 55.44, 37.95, 17.90, 17.66, 17.56, 17.44 (two carbons), 17.39, 17.37, 17.36, 13.24, 13.10, 12.41, 12.28 ppm. **ESI-pos HRMS**: Calculated for $C_{38}H_{59}O_{10}Si_2$ $(M+H)^+$: 731.36413, Found: 731.36542. $[\alpha]_D^{20}$ = +39.3 (c = 0.49, CH_2Cl_2).

(5a*R*,8*R*,9a*R*)-2,2,4,4-Tetraisopropyl-8-phenoxytetrahydro-6*H*-pyrano[3,4-*f*][1,3,5,2,4]trioxadisilepine

The title product compound is prepared according to the general procedure with 5 mol% catalyst **A** and 0.3 mmol glycal at 40 ˚C for 24 h and isolated by flash column chromatography (3:1 Pentane: CH_2Cl_2) giving a pale yellow syrup (53 mg for two anomers, 0.12 mmol, 59% yield, α/β ratio 91:9).

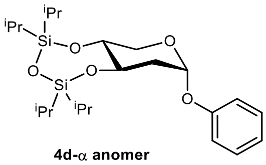

4d-α anomer

^{1}H NMR (500 MHz, $CDCl_3$) δ 7.30-7.26 (m, 2 H), 7.09-7.07 (m, 2 H), 7.03-7.02 (m, 1 H), 5.60 (dd, *J* = 3.7, 1.3 Hz, 1 H), 4.19 (ddd, *J* = 11.2, 8.2, 5.3 Hz, 1 H), 3.77-3.67 (m, 2 H), 3.60-3.55 (m, 1 H), 2.32 (dd, *J* = 13.5, 5.3 Hz, 1 H), 1.81 (ddd, *J* = 13.5, 11.2, 3.6 Hz, 1 H), 1.16-0.93 (m, 28 H) ppm. **^{13}C NMR** (126 MHz, $CDCl_3$) δ 156.79, 129.55, 122.10, 116.72, 96.20, 74.15, 71.75, 63.04, 38.07, 17.77, 17.71, 17.49, 17.47, 17.46, 17.44, 17.42, 17.39, 13.16 (two carbons), 12.43 (two carbons) ppm. **ESI-pos HRMS**: Calculated for $C_{23}H_{41}O_5Si_2$ $(M+H)^+$: 453.24870, Found: 453.24855. $[\alpha]_D^{20}$ = +101.4 (c = 0.22, CH_2Cl_2).

(5a*R*,8*S*,9a*R*)-2,2,4,4-Tetraisopropyl-8-phenoxytetrahydro-6*H*-pyrano[3,4-*f*][1,3,5,2,4]trioxadisilepine

The title product compound is prepared according to the general procedure with 5 mol% catalyst **A** and 0.3 mmol glycal at 40 ˚C for 24 h and isolated by flash column chromatography (3:1 Pentane: CH_2Cl_2) giving a pale yellow syrup (53 mg for two anomers, 0.12 mmol, 59% yield, α/β ratio 91:9).

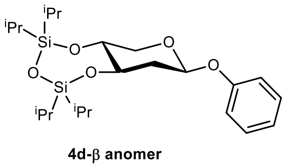

4d-β anomer

^{1}H NMR (500 MHz, $CDCl_3$) δ 7.30-7.28 (m, 3 H), 7.02-7.00 (m, 2 H), 5.12 (dd, *J* = 9.9, 2.2 Hz, 1 H), 4.01 (dd, *J* = 11.7, 5.3 Hz, 1 H), 3.82-3.72 (m, 2 H), 3.29 (dd, *J* = 11.8, 9.7 Hz, 1 H), 2.35 (ddd, *J* = 13.1, 5.0, 2.2 Hz, 1 H), 1.95-1.88 (m, 1 H), 1.09-0.96 (m, 28 H) ppm. **^{13}C NMR** (126 MHz, $CDCl_3$) δ 157.10, 129.60, 122.52, 116.64, 98.32, 74.20, 73.70, 66.45, 39.04, 17.74, 17.64, 17.42 (three carbons), 17.38, 17.36 (two carbons), 13.13, 13.10, 12.43, 12.37 ppm. **ESI-pos HRMS**: Calculated for $C_{23}H_{40}O_5NaSi_2$ $(M+Na)^+$: 475.23065, Found: 475.23109. $[\alpha]_D^{20}$ = -4.3 (c = 0.11, CH_2Cl_2).

(5a*R*,8*R*,9a*R*)-8-(((2*R*,3*S*,4*S*,5*R*,6*R*)-2-Allyl-4,5-bis(benzyloxy)-6-((benzyloxy)methyl)tetrahydro-2*H*-pyran-3-yl)oxy)-2,2,4,4-tetraisopropyltetrahydro-6*H*-pyrano[3,4-*f*][1,3,5,2,4]trioxadisilepine
The title product compound is prepared according to the general procedure with 5 mol% catalyst **A** and 0.3 mmol at 40 ˚C for 24 h and isolated by flash column chromatography (20:1 Pentane: Ethyl Acetate) giving a pale yellow syrup (112 mg for two anomers, 0.13 mmol, 67% yield, α/β ratio 84:16).

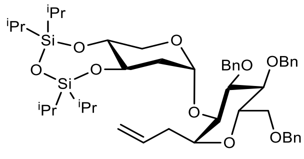

4e-α anomer

^{1}H NMR (500 MHz, $CDCl_3$) δ 7.41-7.26 (m, 13 H), 7.12-7.10 (m, 2 H), 5.87-5.84 (m, 1 H), 5.18-5.11 (m, 2 H), 5.03-4.98 (m, 2 H), 4.81 (d, *J* = 10.7 Hz, 1 H), 4.75 (d, *J* = 10.9 Hz, 1 H), 4.64 (d, *J* = 12.2 Hz, 1 H), 4.51-4.45 (m, 2 H), 4.32-4.27 (m, 1 H), 4.10-4.04 (m, 2 H), 3.82-3.62 (m, 8 H), 2.61-2.53 (m, 1 H), 2.28-2.33 (m, 1 H), 2.11 (dd, *J* = 13.4, 5.3 Hz, 1 H), 1.73-1.67 (m, 1 H), 1.11-0.91 (m, 28 H) ppm. **^{13}C NMR** (126 MHz, $CDCl_3$) δ 138.43, 138.27, 138.17, 134.53, 128.48, 128.46, 128.43, 128.06, 127.99 (two carbons), 127.77, 127.71 (two carbons), 117.25, 94.15, 81.63, 78.13, 76.02, 75.22, 74.12, 74.06, 73.53, 72.30, 72.00, 71.49, 68.97, 62.78, 37.98, 30.07, 17.70, 17.67, 17.49, 17.41, 17.39, 17.38 (three carbons), 13.11, 13.01, 12.45, 12.37 ppm. **ESI-pos HRMS**: Calculated for $C_{47}H_{68}O_9NaSi_2$ $(M+Na)^+$: 855.42941, Found: 855.43028. $[\alpha]_D^{20}$ = +31.6 (c = 0.30, CH_2Cl_2).

(5a*R*,8*S*,9a*R*)-8-(((2*R*,3*S*,4*S*,5*R*,6*R*)-2-Allyl-4,5-bis(benzyloxy)-6-((benzyloxy)methyl)tetrahydro-2*H*-pyran-3-yl)oxy)-2,2,4,4-tetraisopropyltetrahydro-6*H*-pyrano[3,4-*f*][1,3,5,2,4]trioxadisilepine
The title product compound is prepared according to the general procedure with 5 mol% catalyst **A** and 0.3 mmol at 40 ˚C for 24 h and isolated by flash column chromatography (20:1 Pentane: Ethyl Acetate) giving a pale yellow syrup (112 mg for two anomers, 0.13 mmol, 67% yield, α/β ratio 84:16).

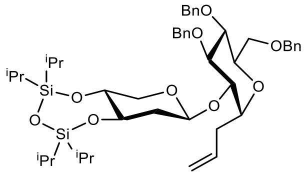

4e-β anomer

^{1}H NMR (500 MHz, $CDCl_3$) δ 7.34-7.26 (m, 13 H), 7.15-7.12 (m, 2 H), 5.87-5.79 (m, 1 H), 5.12-5.04 (m, 2 H), 4.85-4.79 (m, 3 H), 4.66-4.60 (m, 2 H), 4.50-4.46 (m, 2 H), 4.26-4.22 (m, 1 H), 3.89-3.84 (m, 2 H), 3.77-3.70 (m, 2 H), 3.63-3.57 (m, 5 H), 3.09-3.05 (m, 1 H), 2.53-2.40 (m, 2 H), 2.11-2.06 (m, 1 H), 1.62-1.55 (m, 1 H), 1.14-0.81 (m, 28 H) ppm. **^{13}C NMR** (126 MHz, $CDCl_3$) δ 138.77, 138.24, 138.22, 135.00, 128.61, 128.57, 128.47, 128.13 (two carbons), 127.92, 127.89, 127.84, 127.77, 116.84, 102.54, 82.50, 78.48, 75.70, 75.30, 75.26, 74.31, 73.81, 73.62, 71.03, 68.90, 66.23, 39.43, 29.88, 17.78, 17.67, 17.43 (four carbons), 17.37, 17.36 (two carbons), 13.10, 13.06, 12.36, 12.35 ppm. **ESI-pos HRMS**: Calculated for $C_{47}H_{68}O_9NaSi_2$ $(M+Na)^+$: 855.42941, Found: 855.43108. $[\alpha]_D^{20}$ = +79.7 (c = 0.21, CH_2Cl_2).

(5a*R*,8*R*,9a*R*)-8-(((3*S*,8*S*,9*S*,10*R*,13*R*,14*S*,17*R*)-10,13-Dimethyl-17-(l-6-methylheptan-2-yl)-2,3,4,7,8,9,10,11,12,13,14,15,16,17-tetradecahydro-1*H*-cyclopenta[*a*] 32 henanthrene-3-yl)oxy)-2,2,4,4-tetraisopropyltetrahydro-6*H*-pyrano[3,4-*f*][1,3,5,2,4]trioxadisilepine
The title product compound is prepared according to the general procedure with 5 mol% catalyst **A** and 0.3 mmol glycal at 40 ˚C for 24 h and isolated by flash column chromatography (3:1 Pentane: CH_2Cl_2) giving a white foam (135 mg, 0.18 mmol, 91% yield, α/β ratio 72:28).

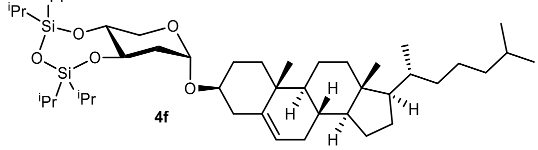

4f

^{1}H NMR (500 MHz, $CDCl_3$) δ 5.35 (d, *J* = 4.7 Hz, 1 H), 4.99 (d, *J* = 3.6 Hz, 1 H), 4.01-3.96 (m, 1 H), 3.65-3.57 (m, 2 H), 3.54-3.50 (m, 1 H), 3.45-3.39 (m, 1 H), 2.34-2.31 (m, 2 H), 2.05-1.94 (m, 3 H), 1.88-1.79 (m, 3 H), 1.65-1.62 (m, 1 H), 1.60-1.31 (m, 10 H), 1.29-1.22 (m, 1 H), 1.19-1.12 (m, 4 H), 1.11-0.93 (m, 37 H), 0.91 (d, *J* = 6.5 Hz, 3 H), 0.87 (d, *J* = 2.4 Hz, 3 H), 0.86 (d, *J* = 2.3 Hz, 3 H), 0.68 (s, 3 H) ppm. **^{13}C NMR** (126 MHz, $CDCl_3$) δ 141.12, 121.69, 95.36, 76.11, 74.56, 72.01, 62.47, 56.90, 56.28, 50.27, 42.46, 40.19, 39.92, 39.67, 38.60, 37.28, 36.89, 36.34, 35.95, 32.09, 32.02, 28.40, 28.17, 27.82,

24.44, 23.98, 22.99, 22.72, 21.21, 19.57, 18.87, 17.77, 17.74, 17.51 (two carbons), 17.49, 17.45 (three carbons), 13.15, 13.13, 12.54, 12.45, 12.00 ppm. **MS (MALDI-TOF)**: Calculated for $C_{44}H_{80}O_5NaSi_2$ $(M+Na)^+$: 767.544, Found: 767.523. $[\alpha]_D^{20}$ = +55.2 (c = 0.15, CH_2Cl_2).

(8*R*,9*S*,10*R*,13*S*,14*S*,17*S*)-10,13-Dimethyl-17-(((5a*R*,8*R*,9a*R*)-2,2,4,4-tetraisopropyltetrahydro-6*H*-pyrano[3,4-*f*][1,3,5,2,4]trioxadisilepin-8-yl)oxy)-1,2,6,7,8,9,10,11,12,13,14,15,16,17-tetradecahydro-3*H*-cyclopenta[*a*]33henanthrene-3-one

The title product compound is prepared according to the general procedure with 5 mol% catalyst **A** and 0.3 mmol glycal at 40 ˚C for 48 h and isolated by flash column chromatography (10:1 Pentane: Ethyl Acetate) giving a pale yellow syrup (97 mg for two anomers, 0.15 mmol, 75% yield, α/β ratio 79:21).

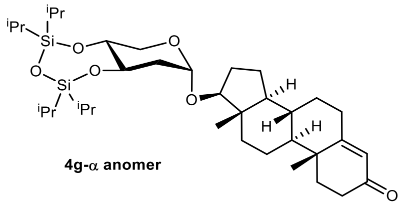

4g-α anomer

^{1}H NMR (500 MHz, $CDCl_3$) δ 5.71 (s, 1 H), 4.82 (s, 1 H), 3.97-3.95 (m, 1 H), 3.63-3.54 (m, 3 H), 3.44 (t, *J* = 9.8 Hz, 1 H), 2.44-2.24 (m, 3 H), 2.03-2.00 (m, 3 H), 1.89-1.81 (m, 2 H), 1.71-1.56 (m, 5 H), 1.44-1.40 (m, 2 H), 1.33-1.30 (m, 1 H), 1.24 (s, 1 H), 1.19-1.18 (m, 3 H), 1.06-0.92 (m, 32 H), 0.82-0.79 (m, 3 H). **^{13}C NMR** (126 MHz, $CDCl_3$) δ 199.77, 171.52, 123.96, 95.43, 83.35, 74.45, 71.83, 62.47, 54.08, 50.56, 42.46, 38.77, 38.44, 37.00, 35.81, 35.49, 34.06, 32.93, 31.65, 26.89, 23.56, 20.75, 17.67, 17.65, 17.51, 17.45, 17.43, 17.41 (two carbons), 17.39, 17.37, 13.14, 13.09, 12.54, 12.50, 11.73 ppm. **ESI-HRMS**: Calculated for $C_{36}H_{63}O_6NaSi_2$ $(M+H)^+$: 647.41577, Found: 647.41602. $[\alpha]_D^{20}$ = +92.9 (c = 0.40, CH_2Cl_2).

(8*R*,9*S*,10*R*,13*S*,14*S*,17*S*)-10,13-Dimethyl-17-(((5a*R*,8*S*,9a*R*)-2,2,4,4-tetraisopropyltetrahydro-6*H*-pyrano[3,4-*f*][1,3,5,2,4]trioxadisilepin-8-yl)oxy)-1,2,6,7,8,9,10,11,12,13,14,15,16,17-tetradecahydro-3*H*-cyclopenta[*a*]33henanthrene-3-one

The title product compound is prepared according to the general procedure with 5 mol% catalyst **A** and 0.3 mmol glycal at 40 ˚C for 48 h and isolated by flash column chromatography (10:1 Pentane: Ethyl Acetate) giving a pale yellow syrup (97 mg for two anomers, 0.15 mmol, 75% yield, α/β ratio 79:21).

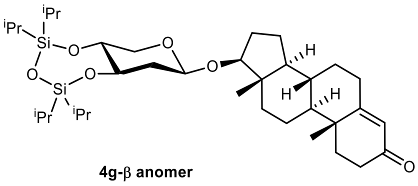

4g-β anomer

^{1}H NMR (500 MHz, $CDCl_3$) δ 5.72 (s, 1 H), 4.47 (d, *J* = 8.8 Hz, 1 H), 3.89 (dd, *J* = 11.7, 4.7 Hz, 1 H), 3.70-3.60 (m, 3 H), 3.12-3.08 (m, 1 H), 2.46-2.31 (m, 3 H), 2.29-2.25 (m, 1 H), 2.17-2.13 (m, 1 H), 2.04-2.00 (m, 2 H), 1.87-1.81 (m, 2 H), 1.70 (dd, *J* = 14.0, 4.9 Hz, 1 H), 1.67-1.54 (m, 5 H), 1.48-1.42 (m, 1 H), 1.30-1.25 (m, 2 H), 1.18 (s, 3 H), 1.17-1.14 (m, 1 H), 1.08-0.91 (m, 30 H), 0.79 (s, 3 H) ppm. **^{13}C NMR** (126 MHz, $CDCl_3$) δ 199.80, 171.52, 123.99, 101.12, 88.42, 74.44, 73.85, 66.34, 54.00, 50.44, 43.00, 39.62, 38.77, 37.48, 35.84, 35.57, 34.08, 32.92, 31.63, 29.04, 23.44, 20.73, 17.78, 17.61, 17.53, 17.44, 17.42, 17.40, 17.36, 17.34 (two carbons), 13.15, 13.03, 12.41, 12.35, 11.83 ppm. **ESI-HRMS**: Calculated for $C_{36}H_{63}O_6NaSi_2$ $(M+H)^+$: 647.41577, Found: 647.41602. $[\alpha]_D^{20}$ = +33.2 (c = 0.13, CH_2Cl_2).

(8*R*,9*S*,10*R*,13*S*,14*S*,17*S*)-10,13,17-Trimethyl-17-(((5a*R*,8*R*,9a*R*)-2,2,4,4-tetraisopropyltetrahydro-6*H*-pyrano[3,4-*f*][1,3,5,2,4]trioxadisilepin-8-yl)oxy)-1,2,6,7,8,9,10,11,12,13,14,15,16,17-tetradecahydro-3*H*-cyclopenta[*a*]33henanthrene-3-one

The title product compound is prepared according to the general procedure with 5 mol% catalyst **A** and 0.3 mmol at 40 ˚C for 24 h and isolated by flash column chromatography (9:1 to 3:1 Pentane: Ethyl Acetate) giving a pale yellow syrup (107 mg, 0.16 mmol, 81% yield, α/β ratio 80:20).

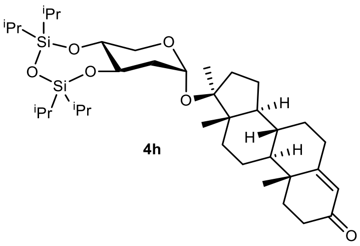

4h

^{1}H NMR (500 MHz, $CDCl_3$) δ 5.72 (s, 1 H), 5.05-5.04 (m, 1 H), 4.02-3.97 (m, 1 H), 3.63-3.55 (m, 3 H), 2.45-2.31 (m, 3 H), 2.28-2.24 (m, 1 H), 2.04-2.00 (m, 1 H), 1.95-1.91 (m, 1 H), 1.87-1.81 (m, 2 H), 1.70 (dd, *J* = 13.9, 4.9 Hz, 1 H), 1.66-1.47 (m, 7 H), 1.46-1.41 (m, 1 H), 1.35-1.29 (m, 1 H), 1.22 (s, 3 H), 1.20 (s, 3 H), 1.19 (s, 3 H), 1.19-1.17 (m, 1 H), 1.07-0.97 (m, 26 H), 0.85 (m, 4 H) ppm. **^{13}C NMR** (126 MHz, $CDCl_3$) δ 199.89, 171.66, 123.94, 93.84, 86.83, 74.65, 71.86, 62.53, 53.98, 49.26, 46.38, 39.76, 38.81, 36.26, 35.84, 35.09, 34.06, 32.99, 31.90, 23.98, 23.56, 23.23, 20.79, 17.64, 17.62, 17.51, 17.48, 17.44 (two carbons), 17.40, 17.38 (two carbons), 14.24, 13.15, 13.09, 12.55, 12.49 ppm. **ESI-HRMS**: Calculated for $C_{37}H_{65}O_6Si_2$ $(M+H)^+$: 661.43142, Found: 661.43171. $[\alpha]_D^{20}$ = +72.6 (c = 0.40, CH_2Cl_2).

(5a*S*,8*R*,9a*S*)-2,2,4,4-Tetraisopropyl-8-(((3a*R*,5*R*,5a*S*,8a*S*,8b*R*)-2,2,7,7-tetramethyltetrahydro-5*H*-bis([1,3]dioxolo)[4,5-*b*:4',5'-*d*]pyran-5-yl)methoxy)tetrahydro-6*H*-pyrano[3,4-*f*][1,3,5,2,4]trioxadisilepine

The title product compound is prepared according to the general procedure with 5 mol% catalyst **A** and 0.3 mmol glycal at 40 ˚C for 24 h and isolated by flash column chromatography (9:1 Pentane: Ethyl Acetate) giving a pale yellow syrup (115 mg, 0.19 mmol, 92% yield, α/β ratio 85:15).

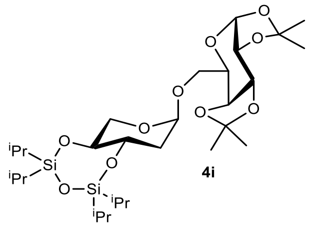

4i

^{1}H NMR (500 MHz, $CDCl_3$) δ 5.52 (d, *J* = 4.9 Hz, 1 H), 4.88 (d, *J* = 3.4 Hz, 1 H), 4.59 (dd, *J* = 7.9, 2.5 Hz, 1 H), 4.29 (dd, *J* = 5.1, 2.4 Hz, 1 H), 4.22 (dd, *J* = 8.0, 1.8 Hz, 1 H), 3.99-3.94 (m, 2 H), 3.75 (dd, *J* = 10.5, 5.0 Hz, 1 H), 3.64-3.47 (m, 4 H), 2.11 (dd, *J* = 13.2, 5.3 Hz, 1 H), 1.63-1.52 (m, 1 H), 1.52 (s, 3 H), 1.43 (s, 3 H), 1.32 (s, 3 H), 1.31 (s, 3 H), 1.06-0.87 (m, 28 H) ppm. **^{13}C NMR** (126 MHz, $CDCl_3$) δ 109.35, 108.66, 97.75, 96.35, 74.28, 71.87, 71.33, 70.75, 70.71, 67.38, 65.88, 62.49, 37.93, 26.12, 26.05, 25.12, 24.35, 17.74, 17.65, 17.46, 17.43 (two carbons), 17.39, 17.37 (two carbons), 13.13, 13.07, 12.41, 12.32 ppm. **ESI-HRMS**: Calculated for $C_{29}H_{54}O_{10}NaSi_2$ $(M+Na)^+$: 641.31477, Found: 661.31517. $[\alpha]_D^{20}$ = -62.8 (c = 0.32, CH_2Cl_2).

(5a*S*,8*S*,9a*S*)-8-(((2*R*,4a*R*,6*S*,7*R*,8*S*,8a*R*)-7-(Benzyloxy)-6-methoxy-2-phenylhexahydropyrano[3,2-*d*][1,3]dioxin-8-yl)oxy)-2,2,4,4-tetraisopropyltetrahydro-6*H*-pyrano[3,4-*f*][1,3,5,2,4]trioxadisilepine

The title product compound is prepared according to the general procedure with 5 mol% catalyst **A** and 0.3 mmol glycal at 40 ˚C for 24 h and isolated by flash column chromatography (9:1 Pentane: Ethyl Acetate) giving a pale yellow syrup (100 mg, 0.14 mmol, 68% yield, α/β ratio 91:9).

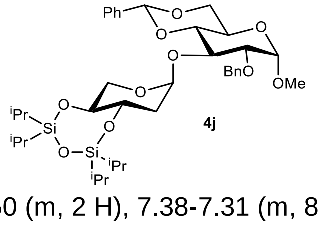

4j

^{1}H NMR (500 MHz, $CDCl_3$) δ 7.52-7.50 (m, 2 H), 7.38-7.31 (m, 8 H), 5.56 (s, 1 H), 5.27 (d, *J* = 3.6 Hz, 1 H), 4.73 (d, *J* = 12.1 Hz, 1 H), 4.63 (d, *J* = 12.1 Hz, 1 H), 4.59 (d, *J* = 3.6 Hz, 1 H), 4.27 (dd, *J* = 10.2, 4.7 Hz, 1 H), 4.14 (t, *J* = 9.4 Hz, 1 H), 3.98-3.93 (m, 1 H), 3.84-3.79 (m, 2 H), 3.70 (t, *J* = 10.3 Hz, 1 H), 3.62-3.57 (m, 1H), 3.55-3.50 (m, 2 H), 3.39 (s, 3 H), 3.37-3.34 (m, 1 H), 2.11-2.07 (m, 1 H), 1.67-1.61 (m, 1 H), 1.11-0.85 (m, 28 H) ppm. **^{13}C NMR** (126 MHz, $CDCl_3$) δ 137.85, 137.28, 128.91, 128.69, 128.28, 128.27, 128.15, 126.25, 101.57, 98.77, 98.46, 80.46, 80.24, 74.35, 74.33, 73.42, 72.02, 69.16, 62.76, 62.51, 55.42, 38.18, 17.83, 17.64, 17.49, 17.48, 17.44, 17.41, 17.38, 17.37, 13.15, 13.08, 12.44,

12.41 ppm. **ESI-HRMS**: Calculated for $C_{38}H_{58}O_{10}NaSi_2$ $(M+Na)^+$: 753.34607, Found: 753.34624. $[\alpha]_D^{20}$ = -33.1 (c = 0.23, CH_2Cl_2).

(8*R*,9*S*,10*R*,13*S*,14*S*,17*S*)-10,13,17-Trimethyl-17-(((5a*S*,8*S*,9a*S*)-2,2,4,4-tetraisopropyltetrahydro-6*H*-pyrano[3,4-*f*][1,3,5,2,4]trioxadisilepin-8-yl)oxy)-1,2,6,7,8,9,10,11,12,13,14,15,16,17-tetradecahydro-3*H*-cyclopenta[*a*]35henanthrene-3-one

The title product compound is prepared according to the general procedure with 5 mol% catalyst **A** at 40 ˚C for 24 h and isolated by flash column chromatography (9:1 Pentane: Ethyl Acetate) giving a pale yellow syrup (99 mg for two anomers, 0.15 mmol, 74% yield, α/β ratio 80:20).

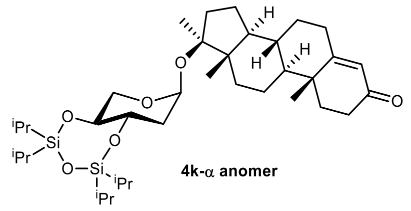

4k-α anomer

^{1}H NMR (500 MHz, $CDCl_3$) δ 5.72 (s, 1 H), 5.12 (d, *J* = 3.2 Hz, 1 H), 4.02-3.97 (m, 1 H), 3.65-3.58 (m, 3 H), 2.45-2.31 (m, 3 H), 2.28-2.25 (m, 1 H), 2.19-2.14 (m, 1 H), 2.04-2.01 (m, 1 H), 1.93 (dd, *J* = 12.8, 4.9 Hz, 1 H), 1.84 (d, *J* = 12.6 Hz, 1 H), 1.69 (td, *J* = 13.9, 4.4 Hz, 1 H), 1.64-1.51 (m, 6 H), 1.49-1.41 (m, 1 H), 1.37-1.21 (m, 2 H), 1.19 (s, 3 H), 1.18 (s, 3 H), 1.16-1.13 (m, 1 H), 1.07-0.87 (m, 33 H) ppm. **^{13}C NMR** (126 MHz, $CDCl_3$) δ 199.70, 171.45, 123.97, 93.05, 86.54, 74.65, 71.90, 62.86, 53.97, 49.54, 46.86, 39.60, 38.78, 36.30, 35.87, 35.57, 34.11, 32.98, 32.34, 31.91, 23.64, 22.19, 20.83, 17.67, 17.65, 17.52, 17.46, 17.45, 17.43 (two carbons), 17.40 (two carbons), 14.74, 13.17, 13.11, 12.55, 12.53 ppm. **ESI-HRMS**: Calculated for $C_{37}H_{65}O_6Si_2$ $(M+H)^+$: 661.43142, Found: 661.43116. $[\alpha]_D^{20}$ = +1.9 (c = 0.13, CH_2Cl_2).

(8*R*,9*S*,10*R*,13*S*,14*S*,17*S*)-10,13,17-Trimethyl-17-(((5a*S*,8*R*,9a*S*)-2,2,4,4-tetraisopropyltetrahydro-6*H*-pyrano[3,4-*f*][1,3,5,2,4]trioxadisilepin-8-yl)oxy)-1,2,6,7,8,9,10,11,12,13,14,15,16,17-tetradecahydro-3*H*-cyclopenta[*a*]35henanthrene-3-one

The title product compound is prepared according to the general procedure with 5 mol% catalyst **A** at 40 ˚C for 24 h and isolated by flash column chromatography (9:1 Pentane: Ethyl Acetate) giving a pale yellow syrup (99 mg for two anomers, 0.15 mmol, 74% yield, α/β ratio 80:20).

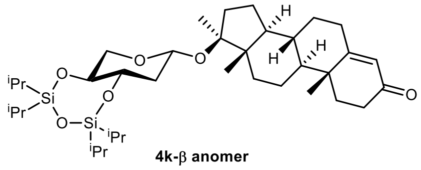

4k-β anomer

^{1}H NMR (500 MHz, $CDCl_3$) δ 5.72 (s, 1 H), 4.57 (dd, *J* = 9.9, 2.0 Hz, 1 H), 3.87 (dd, *J* = 11.7, 5.3 Hz, 1 H), 3.72-3.61 (m, 2 H), 3.12 (dd, *J* = 11.7, 9.9 Hz, 1 H), 2.45-2.30 (m, 3 H), 2.30-2.25 (m, 1 H), 2.04-2.01 (m, 2 H), 1.91-1.82 (m, 2 H), 1.70-1.55 (m, 7 H), 1.47-1.38 (m, 1 H), 1.35-1.26 (m, 1 H), 1.23 (s, 3 H), 1.21-1.17 (m, 1 H), 1.19 (s, 3 H), 1.08-0.89 (m, 31 H), 0.87 (s, 3 H) ppm. **^{13}C NMR** (126 MHz, $CDCl_3$) δ 199.80, 171.53, 123.97, 96.72, 87.60, 74.64, 73.75, 66.19, 53.98, 49.77, 46.17, 40.65, 38.82, 36.30, 35.87, 35.81, 34.13, 33.00, 31.92, 31.59, 23.44, 23.39, 20.81, 17.80, 17.60, 17.56, 17.46, 17.43, 17.42, 17.38, 17.36, 17.34, 14.11, 13.16, 13.06, 12.40, 12.35 ppm. **ESI-HRMS**: Calculated for $C_{37}H_{65}O_6Si_2$ $(M+H)^+$: 661.43142, Found: 661.43103. $[\alpha]_D^{20}$ = +24.7 (c = 0.09, CH_2Cl_2).

(6a*R*,8*R*,9a*R*)-8-(((2*S*,3*S*,4*S*,5*R*,6*R*)-2-Allyl-4,5-bis(benzyloxy)-6-((benzyloxy)methyl)tetrahydro-2*H*-pyran-3-yl)oxy)-2,2,4,4-tetraisopropyltetrahydro-6*H*-furo[3,2-*f*][1,3,5,2,4]trioxadisilocine

The title product compound is prepared according to the general procedure with 3 mol% catalyst **A** and 0.4 mmol glycal at 30 ˚C for 48 h and isolated by flash column chromatography (20:1 Pentane: Ethyl Acetate) giving a pale yellow syrup (92 mg, 0.11 mmol, 55% yield, α/β ratio >20:1).

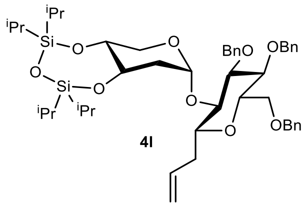

4l

^{1}H NMR (500 MHz, $CDCl_3$) δ 7.35-7.14 (m, 15 H), 6.00-5.94 (m, 1 H), 5.30-5.26 (m, 1 H), 5.18-5.07 (m, 2 H), 4.90-4.82 (m, 2 H), 4.78-4.73 (m, 1 H), 4.66-4.63 (m, 1 H), 4.60-4.54 (m, 2 H), 4.40-4.36 (m, 1 H),

4.08-4.05 (m, 1 H), 3.82-3.78 (m, 1 H), 3.75-3.65 (m, 3 H), 3.62-3.55 (m, 3 H), 3.44-3.40 (m, 1 H), 3.29-3.24 (m, 1 H), 2.71-2.67 (m, 1 H), 2.35-2.31 (m, 1 H), 2.07-2.01 (m, 1 H), 1.76-1.67 (m, 1 H), 1.09-0.88 (m, 28 H) ppm. **^{13}C NMR** (126 MHz, $CDCl_3$) δ 138.55, 138.51, 138.24, 135.45, 128.54, 128.52, 128.45, 128.05, 127.85 (two carbons), 127.69, 127.62 (two carbons), 116.83, 99.36, 87.30, 79.22, 78.80, 78.71, 75.62, 75.07, 73.53, 71.12, 69.29, 69.04, 64.38, 37.11, 35.94, 17.78, 17.71, 17.65, 17.63, 17.42 (two carbons), 17.37, 17.32, 13.91, 13.60, 13.29, 13.17 ppm. **ESI-HRMS**: Calculated for $C_{47}H_{68}O_9NaSi_2$ $(M+Na)^+$: 855.42941, Found: 855.42935. $[\alpha]_D^{20}$ = -11.9 (c = 0.23, CH_2Cl_2).

(6a*R*,8*S*,9a*R*)-2,2,4,4-Tetraisopropyl-8-(((3a*R*,5*R*,5a*S*,8a*S*,8b*R*)-2,2,7,7-tetramethyltetrahydro-5*H*-bis([1,3]dioxolo)[4,5-*b*:4',5'-*d*]pyran-5-yl)methoxy)tetrahydro-6*H*-furo[3,2-*f*][1,3,5,2,4]trioxadisilocine

The title product compound is prepared according to the general procedure with 3 mol% catalyst **A** and 0.3 mmol at 30 ˚C for 24 h and isolated by flash column chromatography (20:1 to 9:1 Pentane: Ethyl Acetate) giving a pale yellow syrup (80 mg, 0.13 mmol, 64% yield, α/β ratio >20:1).

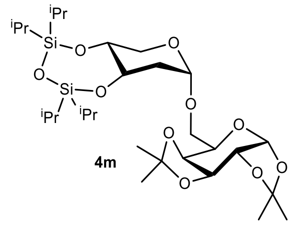

4m

^{1}H NMR (500 MHz, $CDCl_3$) δ 5.53 (d, *J* = 3.7 Hz, 1 H), 4.91 (s, 1 H), 4.58 (d, *J* = 7.8 Hz, 1 H), 4.34 (d, *J* = 8.5 Hz, 1 H), 4.30-4.29 (m, 1 H), 4.22 (d, *J* = 6.9 Hz, 1 H), 4.06-4.04 (m, 1 H), 3.99-3.96 (m, 1 H), 3.85-3.82 (m, 2 H), 3.65-3.61 (m, 2 H), 2.08-2.04 (m, 1 H), 1.83-1.78 (m, 1 H), 1.52 (s, 3 H), 1.43 (s, 3 H), 1.31 (s, 6 H), 1.05-0.84 (m, 28 H) ppm. **^{13}C NMR** (126 MHz, $CDCl_3$) δ 109.40, 108.67, 98.84, 96.46, 71.42, 70.93, 70.77, 70.58, 69.10, 67.23, 67.12, 64.24, 35.31, 26.13, 26.09, 25.06, 24.57, 17.73, 17.71, 17.66, 17.60, 17.47, 17.39, 17.34, 17.29, 13.87, 13.66, 13.56, 12.99 ppm. **ESI-HRMS**: Calculated for $C_{29}H_{54}O_{10}NaSi_2$ $(M+Na)^+$: 641.31477, Found: 641.31489. $[\alpha]_D^{20}$ = -61.8 (c = 0.40, CH_2Cl_2).

Methyl *N*-(*tert*-butoxycarbonyl)-*O*-((6aR,8S,9aR)-2,2,4,4-tetraisopropyltetrahydro-6*H*-furo[3,2-*f*][1,3,5,2,4]trioxadisilocin-8-yl)-*L*-serinate

The title product compound is prepared according to the general procedure with 5 mol% catalyst **Br-A** and 0.4 mmol at 40 ˚C for 24 h and isolated by flash column chromatography (20:1 to 9:1 Pentane: Ethyl Acetate) giving a pale yellow syrup (82 mg, 0.14 mmol, 71% yield, α/β ratio >20:1).

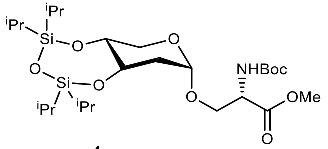

4n

^{1}H NMR (700 MHz, $CDCl_3$) δ 5.35 (d, *J* = 8.9 Hz, 1 H), 4.84-4.83 (m, 1 H), 4.48-4.45 (m, 1 H), 4.27-4.24 (m, 1 H), 4.12-4.09 (m, 1 H), 4.06-4.03 (m, 1 H), 3.75 (s, 3 H), 3.68-3.65 (m, 1 H), 3.64-3.60 (m, 2 H), 2.07-2.02 (m, 1 H), 1.80-1.76 (dt, *J* = 13.0, 3.9 Hz, 1 H), 1.46 (s, 9 H), 1.09-0.86 (m, 28 H) ppm; **^{13}C NMR** (176 MHz, $CDCl_3$) δ 171.20, 155.63, 98.64, 80.22, 70.66, 68.98, 68.02, 64.22, 53.88, 52.53, 34.72, 28.47, 17.75, 17.73, 17.71, 17.59, 17.49, 17.42, 17.35, 17.30, 14.00, 13.79, 13.49, 12.96 ppm. **ESI-HRMS**: Calculated for $C_{26}H_{51}NO_9NaSi_2$ $(M+Na)^+$: 600.3000, Found: 600.2981. $[\alpha]_D^{20}$ = -39.1 (c = 0.43, CH_2Cl_2).

(8R,9S,10R,13S,14S,17S)-10,13-Dimethyl-17-(((6aR,8S,9aR)-2,2,4,4-tetraisopropyltetrahydro-6*H*-furo[3,2-*f*][1,3,5,2,4]trioxadisilocin-8-yl)oxy)-1,2,6,7,8,9,10,11,12,13,14,15,16,17-tetradecahydro-3*H*-cyclopenta[a]36henanthrene-3-one

The title product compound is prepared according to the general procedure with 10 mol% catalyst **Br-A** and 0.4 mmol at 40 ˚C for 24 h and isolated by flash column chromatography (20:1 to 9:1 Pentane: Ethyl Acetate) giving a pale yellow syrup (70.6 mg, 0.11 mmol, 54% yield, α/β ratio 90:10).

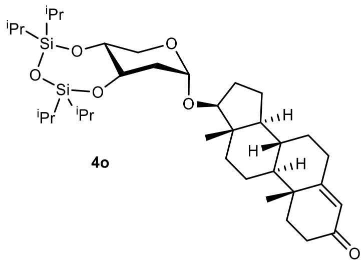

4o

¹H NMR (500 MHz, $CDCl_3$) δ 5.71 (s, 1 H), 4.92 (t, *J* = 3.6 Hz, 1 H), 4.35-4.31 (m, 1 H), 4.07-4.04 (m, 1 H), 3.79 (dd, *J* = 11.6, 2.6 Hz, 1 H), 3.63-3.55 (m, 2 H), 2.41-2.32 (m, 3 H), 2.28-2.59 (m, 1 H), 2.04-1.95 (m, 3 H), 1.87-1.75 (m, 3 H), 1.72-1.64 (m, 1 H), 1.61-1.50 (m, 4 H), 1.47-1.37 (m, 1 H), 1.31-1.23 (m, 1 H), 1.20-1.14 (m, 1 H), 1.17 (s, 3 H), 1.06-0.87 (m, 31 H), 0.79 (s, 3 H) ppm; **¹³C NMR** (126 MHz, $CDCl_3$) δ 199.65, 171.36, 123.97, 98.54, 86.95, 70.93, 69.26, 64.19, 53.94, 50.45, 42.97, 38.73, 37.47, 35.81, 35.67, 35.58, 34.06, 32.90, 31.60, 28.66, 23.38, 20.74, 17.77, 17.69 (two carbons), 17.58, 17.51, 17.50, 17.41, 17.34, 17.29, 13.89, 13.63, 13.51, 13.01, 11.84 ppm. **ESI-HRMS**: Calculated for $C_{36}H_{62}O_6NaSi_2$ $(M+Na)^+$: 669.3983, Found: 669.3978. $[\alpha]_D^{20}$ = +1.9 (c = 0.28, CH_2Cl_2).

(8R,9S,10R,13S,14S,17S)-10,13,17-Trimethyl-17-(((6aR,8R,9aR)-2,2,4,4-tetraisopropyltetrahydro-6*H*-furo[3,2-*f*][1,3,5,2,4]trioxadisilocin-8-yl)oxy)-1,2,6,7,8,9,10,11,12,13,14,15,16,17-tetradecahydro-3H-cyclopenta[a]37henanthrene-3-one

The title product compound is prepared according to the general procedure with 10 mol% catalyst **Br-A** and 0.4 mmol at 40 ˚C for 24 h and isolated by flash column chromatography (20:1 to 9:1 Pentane: Ethyl Acetate) giving a white foam (85.2 mg, 0.13 mmol, 64% yield, α/β ratio >20:1).

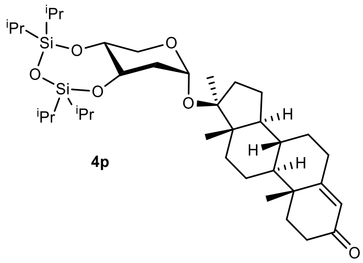

4p

¹H NMR (500 MHz, $CDCl_3$) δ 5.71 (s, 1 H), 5.12-5.10 (m, 1 H), 4.37-4.33 (m, 1 H), 4.07-4.03 (m, 1 H), 3.86 (dd, *J* = 11.5, 2.8 Hz, 1 H), 3.61 (dd, *J* = 11.5, 5.7 Hz, 1 H), 2.45-2.29 (m, 3 H), 2.28-2.23 (m, 1 H), 2.18-2.13 (m, 1 H), 2.05-1.99 (m, 2 H), 1.86-1.80 (m, 1 H), 1.73-1.64 (m, 2 H), 1.63-1.53 (m, 5 H), 1.47-1.34 (m, 2 H), 1.20 (s, 3 H), 1.18 (s, 3 H), 1.18-1.13 (m, 1 H), 1.06-0.90 (m, 30 H), 0.90-0.85 (m, 1 H), 0.88 (s, 3 H) ppm. **¹³C NMR** (126 MHz, $CDCl_3$) δ 199.70, 171.47, 123.94, 93.90, 86.98, 71.06, 69.64, 64.26, 53.89, 49.93, 46.66, 38.75, 36.29, 35.99, 35.83, 34.08, 32.96, 32.70, 31.87, 23.51, 22.50, 20.79, 17.78, 17.69, 17.68, 17.59, 17.52, 17.51, 17.42, 17.36, 17.31, 14.72, 13.79, 13.61, 13.57, 13.10 (one carbon was overlapped in the range of 17.78-17.31) ppm. **ESI-HRMS**: Calculated for $C_{37}H_{65}O_6Si_2$ $(M+H)^+$: 661.4320, Found: 661.4312. $[\alpha]_D^{20}$ = +6.3 (c = 0.63, CH_2Cl_2).

(((2*R*,3*S*,4*R*,5*R*,6*S*)-2-(((*Tert*-Butyldimethylsilyl)oxy)methyl)-6-isopropoxytetrahydro-2*H*-pyran-3,4-diyl-5-*d*)bis(oxy))bis(*tert*-butyldimethylsilane)

The title product compound is prepared according to the general procedure with 3 mol% catalyst **A** at 30 ˚C for 48 h and isolated by flash column chromatography (3:1 Pentane: CH_2Cl_2) giving a pale yellow syrup (78 mg, 0.14 mmol, 71% yield, α/β ratio >20:1).

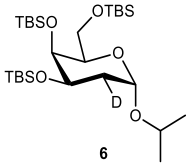

6

¹H NMR (500 MHz, $CDCl_3$) δ 4.97 (d, *J* = 3.7 Hz, 1 H), 4.03 (dd, *J* = 11.7, 2.4 Hz, 1 H), 3.87-3.81 (m, 2 H), 3.69-3.63 (m, 3 H), 2.05 (dd, *J* = 11.8, 3.7 Hz, 1 H), 1.16 (d, *J* = 6.1 Hz, 3 H), 1.09 (d, *J* = 6.1 Hz, 3 H), 0.92-0.89 (m, 27 H), 0.11-0.04 (m, 18 H) ppm. ²H NMR (107 MHz, $CDCl_3$) δ 1.55 (s, 1 H) ppm. **¹³C NMR** (126 MHz, $CDCl_3$) δ 95.89, 72.70, 70.49, 68.51, 68.38, 62.89, 33.94 (t, *J* = 20.2 Hz), 26.37, 26.29, 25.98, 23.52, 21.66, 18.73, 18.65, 18.34, -3.74, -4.20, -4.54, -4.77, -5.17, -5.25 ppm. **ESI-HRMS**:

Calculated for $C_{27}H_{59}DO_5NaSi_3$ $(M+Na)^+$: 572.37035, Found: 572.37014. $[\alpha]_D^{20}$ = +56.7 (c = 0.22, CH_2Cl_2).

(((2*R*,3*S*,4*R*,5*S*,6*S*)-2-(((*Tert*-Butyldimethylsilyl)oxy)methyl)-6-isopropoxytetrahydro-2*H*-pyran-3,4-diyl-5-*d*)bis(oxy))bis(*tert*-butyldimethylsilane)

The title product compound is prepared according to the general procedure with 3 mol% catalyst **A** at 30 ˚C for 48 h and isolated by flash column chromatography (3:1 Pentane: CH_2Cl_2) giving a pale yellow syrup (65 mg, 0.12 mmol, 59% yield, α/β ratio >20:1).

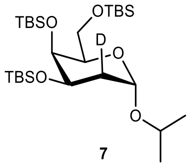

7

^{1}H NMR (500 MHz, $CDCl_3$) δ 4.97 (s, 1 H), 4.03 (dd, *J* = 4.0, 2.4 Hz, 1 H), 3.86-3.81 (m, 2 H), 3.68-3.63 (m, 3 H), 1.52 (d, *J* = 4.0 Hz, 1 H), 1.16 (d, *J* = 6.3 Hz, 3 H), 1.09 (d, *J* = 6.1 Hz, 3 H), 0.91-0.89 (m, 27 H), 0.10-0.04 (m, 18 H) ppm. **^{13}C NMR** (126 MHz, $CDCl_3$) δ 95.90, 72.70, 70.53, 68.52, 68.37, 62.89, 33.99 (t, *J* = 17.6 Hz), 26.37, 26.30, 25.99, 23.52, 21.65, 18.73, 18.65, 18.34, -3.73, -4.20, -4.54, -4.77, -5.16, -5.25 ppm. **ESI-HRMS**: Calculated for $C_{27}H_{59}DO_5NaSi_3$ $(M+Na)^+$: 572.37035, Found: 572.37016. $[\alpha]_D^{20}$ = +60.6 (c = 0.19, CH_2Cl_2).

Supplementary Notes

Supplementary Note 1: The 3,4-di-acetyl-D-ribal was synthesized according to the literature[6]. The proposed 5-membered furanosyl ring in the original paper was incorrect and then the authors corrected to be the structure of 6-membered ring, please see the correction[7]. And the starting material D-ribal was derived from the reaction of deprotection of 3,4-di-acetyl-D-ribal. Therefore, D-ribal **1j** should also have the 6-membered pyranosyl ring structure.

Supplementary Note 2: 3m, **3ad**, **4f** can only be detected by MALDI-TOF, and detection by other HRMS technique (ESI+) was unsuccessful.

Supplementary Note 3: In table S1 (page S47), we attempted to synthesize the bromide/iodide salt of the catalyst **A** instead of a triflate salt by two methods including the anion exchange and the iodination of imidazolium according to the recently reported procedure,[10] however, both methods did not work. It is pertinent to note that the exact iodide/bromide derivative of **A** are not known compounds in the literature.

Supplementary Note 4: In figure S1 (page S48), when the control experiments with 1 mol% and 5 mol% HOTf were performed, low NMR yields were observed in both cases. The low yields might have resulted from the decomposition of glycosyl donor **1b** by HOTf. The key difference between HOTf catalysis and XB catalysis was that TBAI competition poisoned the reaction catalyzed by XB (active catalyst is oxyanion-catalyst complex), while TBAI did not poison any of the reactions catalyzed by HOTf, strongly suggesting against conversion of adduct **11** into HOTf resulting in trace acid catalysis. Interestingly when 1 mol% TBAI as additive was used together with 1 mol% HOTf, the reaction (Supplementary table S1, entry 24) gave quantitative NMR yield. We speculate that anionic exchange might be operative in this case, and the HI formed *in-situ* might be the actual catalyst for the reaction (Supplementary Table S1, entry 24). However, even in this case, we did not observe poisoning of reaction as a result of TBAI competition, further supporting crucial XB influences in **11** and **12**.

Supplementary Note 5: In figure S2 (page S49), fitting of the titration curve using the expression $y = y_0 / 0.02 \times (0.5 \times [i\text{PrOH}] + 0.5 \times (0.02 + K_d) - 0.5 \times ([i\text{PrOH}]^2 + 2 \times [i\text{PrOH}] \times (K_d\text{-}0.02) + (K_d + 0.02)^2)^{0.5}))$[11,12] yielded a dissociation constant (K_d) of 2.22 M, so binding constant (K_a) equals to 0.45 M^{-1}, calculated from the equation $K_a \times K_d = 1$. y_0 means the maximum chemical shift change.

Supplementary Note 6: In figure S15 (page S64), in the sequential instance, an accelerated product formation is observed which ended at 400 min. In contrast, the standard conditions involving concomitant *in-situ* catalyst generation amplification and product retardation is more gradual (Figure 6a in the manuscript, Supplementary figure S11) and needed a significantly longer reaction time of 1100 minutes.

Supplementary Note 7: In figure S17 (page S67), the retardation of catalysis observed in Supplementary figure S17 when 20 mol% of **3al** was initially added to the standard glycosylation conditions indicate that product **3al** can possibly interact with catalyst precursor **A** *via* XB interactions, preventing amplification of the active catalyst **12** by siphoning off the “off-cycle” reservoir of **A**. This experiment provides further evidence to exclude autocatalytic effects.

Supplementary Note 8: In table S8 (page S69), due to the overlap of other peaks, the accurate integration of **2al** was not possible, so here only **1c** and **3al** were listed.

Supplementary Note 9: In figure S20 (page S70), to evaluate if the sequestration of glycosidic product to precursor **A** has an analogous effect on the *in-situ* catalyst, we performed a sequential control experiment (Supplementary figure S20, gray points) reacting **2al** with **A** for 6 h to pre-generate the *in-situ* catalyst, followed by **1c** and 20 mol% **3al**, and commencing the NMR monitoring. By overlaying the temporal formation kinetic of **3al** from this sequential condition and that from the conditions of **Figure 6a**, a substantial increment of NMR yield of **3al** of 23.5% (sequential) over 6.5% (**3al** added at onset) is observed at 1200 min, supporting the higher propensity of **3al** in establishing deactivating XB interactions with **A** but not with the *in-situ* catalyst. However, the yields were still much lower in this sequential experiment as compared to standard conditions at 1200 min, supporting that any unconverted **A** in the reaction mixture after 6h will be sequestered by **3al** upon sequential addition, halting any further amplification of the *in-situ* catalyst.

The preferential product **3al** to **A** rather than **3al** to *in-situ* catalyst deactivation could be accounted for by a possible stronger XB interaction between **A** and the acceptor derived oxyanion in the *in-situ* catalyst. This also explains why our protocol at standard conditions did not terminate upon formation of 20 mol% **3al**, due to the formation and presence of substantial *in-situ* catalyst in the reaction flask for reaction to proceed, unlike in **Figure 6a**, where initial sequestration of **3al** to **A** precluded progression of the reaction to generate the crucial *in-situ* catalyst required for catalysis

Supplementary Note 10: In figure S46 (page S87), Calculation the secondary KIE is described as follows:
The amount (moles) of **3al** is denoted as x, and the amount (moles) of **7** is denoted as y.
According to the previous KIE experiments (Supplementary figure S40), the ratio of **7** to **6** is 13.3;
So the amount (moles) of **6** is y /13.3.
From the integration shown in the ^{1}H NMR spectra, and by solving the following two simultaneous equations:
$x + y/13.3 = 1$ and $x + y = 1.81$
so $x = 0.935$ and $y = 0.875$
The secondary KIE is the ratio of **3al** to **7** + **6**
So, KIE value is equal to 0.935 / (0.875+0.875/13.3) = 0.994

Supplementary Note 11: In figure S49 (page S89), during the monitoring for the control reaction involving dynamic acceptor exchange, no observable anomerization of **3al** was detected, and the formation of **3ak** was α-selective at all time points. Therefore, we propose the first step of the dynamic acceptor exchange cycle involves cleavage of C-O bond of **3al** forming the oxocarbenium ion, followed by the S_N1 attack by acceptor **2r** at the anomeric carbon forming **3ak**. Analogously, we propose that in a reaction mixture with only 1 acceptor, the dynamic acceptor exchange cycle involves glycosidic bond cleavage and reforming of the same bond catalyzed by the XB catalyst.

Supplementary Note 12: In figure S53 (page S92), we further conducted a confirmatory experiment by first performing the reaction under standard conditions. Upon reaching the maximal plateau of the sigmoidal profile at 21h, we added 0.2 mmol of propargyl alcohol **2r** to investigate the perturbation on the mechanistic network. The main difference distinguishing this experiment from that in **Figure 6c** lies in understanding catalyst activity of **A** in the dynamic acceptor exchange under actual glycosylation conditions after completion of productive turnover cycles. In contrast, **Figure 6c** evidences the dynamic exchange using fresh catalyst **A** in the absence of the original glycal donor and isopropanol acceptor. The reduction of **3al** and the concomitant increment of **3ak** further confirmed that the XB catalyst activity for dynamic acceptor exchange is operative throughout the timeframe studied under standard conditions, including the post-period after observed completion of reaction.

Supplementary Note 13: In table S15 (page S97), comparing with the results obtained from XB catalysis for analogues **3af** and **4h**, the yields obtained by *p*TsOH catalysis were substantially lower. In line with decomposition kinetics observed in Supplementary figures S59-60, we postulate that this observation is due to the enhanced Brønsted acid catalyzed hydrolytic cleavage of labile glycosidic bonds, when tertiary alcohol aglycones are employed.

Supplementary Note 14: In line with Ref 65 of the manuscript, we have conducted benchmarking 2-deoxyglycoside decomposition experiments comparing XB catalysis and strong Brønsted acid catalysis. These experiments revealed substantially enhanced decomposition kinetics using HCl und *p*TsOH, and almost negligible decomposition using XB catalyst **A** under identical conditions (See Supplementary figures 59-60). Moreover, in control 2-deoxyglycosylation experiments involving *p*TsOH catalysis in the presence of tertiary glycosyl acceptors, substantially reduced yields due to decomposition was observed (See Supplementary Table S15), ascertaining 2-deoxyglycoside instability in the presence of strong Brønsted acids.

Supplementary Note 15: While such non-iodinated catalyst **E** worked in the facile model glycosylation through non-classical HB with fairly good yields of **3a**, we noticed in control experiments that more challenging acceptor substrates like phenolic functionalities in tyrosine resulted in significantly more inferior (12% yield of **3aj**) than catalyst **A** (75% yield).

Supplementary Note 16: When thiols are used as *S*-acceptors in control experiments, benzimidazolium adducts due to S_NAr reactions on catalyst **A** were detected by LC-MS (ESI+ mode)

and also in ^{1}H NMR, proving that positively charged benzimidazolium adducts are suitable for LC-MS detection (See Supplementary Figure S5-S9 for detailed analysis). However, no such adducts are detected for *O*-acceptors. Hence, fortuitous acid catalysis could be operative when sulfur based nucleophiles are used, but not with *O*-acceptors.

Supplementary Note 17: The possibility of ion pair **12** converting into trace amounts of HOTf, which then catalyzes the reaction is excluded through further competition control experiments (See Supplementary Figure S1 for more details). While the use of HOTf as a catalyst resulted in reaction, however, the diagnostic TBAB/TBAI competition experiments did not poison HOTf catalysis. This is unlike our protocol, which is terminated by TBAB/TBAI addition in multiple control experiments, confirming the cruciality of XB participation in **12**, as well as in our entire catalytic mechanism.

Supplementary Note 18: There exists the plausibility of NMR signal averaging in the NMR timescale in a weak acid dissociation reaction, making both the undissociated acid adduct **11** and the solvent separated ion pair **12** indistinguishable on the NMR timescale.

Supplementary Discussions

Supplementary Discussion 1

Current literature reports on 2-deoxygalactosylation generally lack information on cross methodological comparison and feature mainly hexoses donors such as galactal and glucal donors. It is hence unclear from literature whether important glycosyl donor classes (e.g. pentoses) can also be incorporated into literature known methodologies, which constitutes a clear scientific gap our manuscript addresses. Further benchmarking experiments were conducted to better understand the advantages of XB catalysis over key methods (Supplementary Table S16, SI Page S98-S99). The original protocols with exact reported substrates employed in these methods were also reproduced in our hands.

For comparing against purely thiourea catalysis, our studies indicated that XB catalysis have general clear advantages in terms of broadness of donor and acceptor substrate tolerances (e.g. in particular glucals, rhamnals and a wide range of Pentoses, silylated galactals). Notably, while ether protecting group schemes on galactals work generally well in thiourea catalysis, other glycosyl donors and silylated galactals were inefficient using thiourea catalysis (See Figure 10, also Table S16-2 in page S98 of SI).

Regarding *p*TsOH,[4] we have done comparative decomposition experiments (see SI Page S97, Supplementary Table S14-S15, Supplementary Figure 60), which revealed that strong acids resulted in a higher propensity of product decomposition, despite them being more tolerant towards glucals and rhamnals than thiourea. Significantly, decomposition was not observed using XB catalysis, and this observation is particularly pronounced using *p*TsOH catalysis with sterically hindered tertiary acceptors giving much reduced yields of product (see SI page S97). This constitutes the advantage of using the milder XB catalyzed conditions without concerns of decomposition issues brought about by strong Brønsted acids.

The pyridinium salts initially reported by Berkessel[3] operates through a covalent mechanism via reaction between glycosyl acceptor and pyridinium salts, which generates a Brønsted acid, rather than *via* noncovalent mechanisms. While pentoses tolerance were not described nor known in the initial publication from Berkessel, our control further experiments (SI Page S99, Table S16-4) revealed that XB catalysis (especially using the **Br-A** variant) provided overall robustness, giving consistently very high yields (>80%) over Berkessel's method (**Method E**). Moreover, the authors noted in the manuscript[3] that "*In the more challenging glucal series, the benzyl-protected donor **11a** reacted smoothly with the primary acceptor galactose acetonide **6c** with very high anomeric selectivity (entry 10). The same holds for the silyl-protected glucal **11b** (entry 11), although the yield of isolated glycoside was somewhat lower (63% vs. quantitative).*" This also suggest that pyridinium salts tolerate etheric protecting groups better than silyl-protecting groups which gave lower yield, whereas our XB method favour silyl-protecting groups much better.

For both Pd and Au catalysis,[1-2] our control experiments indicated that they performed often poorer than XB in pentoses donors, generating glycosides with lower anomeric selectivity and yields (SI Page S99, Table S16-4). Furthermore, Pd and Au catalysis have practical drawbacks. The Pd catalysis report required high catalyst loadings (25% catalyst and 30% ligand),[1] and both Pd and Au catalysis reports required strict moisture exclusion by rigorous vacuum suctioning prior to the start of the reaction. In our XB catalytic case, it is milder, less sensitive to air and moisture and do not require such pre-suctioning protocols. Furthermore, galactal substrates bearing two protecting groups TIPS and Bn (**3am** in Figure 3 of manuscript), an important substrate for orthogonal protection/deprotection purposes, appeared to give much lower anomeric selectivity (6:1) in Au catalysis.[2]

In all, we noted that various factors including carbohydrate substrate specificity and acid decomposition play different roles in all reported catalytic systems. However, in comparative experiments, we show that the mildness and tunability of XB catalysis have the overall advantage of accessing a very broad scope on both the donor and the acceptor portion with very high anomeric selectivity without substantial decomposition, particularly in pentoses, which places it as a top forerunner amongst these leading methods.

Supplementary Discussion 2

We postulate that the formation of solvated adduct **12** from A and ROH is thermodynamically described by a reversible weak acid dissociation constant (K_a), which approaches dynamic equilibrium slowly. Pretreatment of **A** and ROH allows time for this dynamic equilibrium (equal forward and backward rates) to be established, ensuring the presence of maximal "*in-situ*" catalyst for reaction progression.

Without this pretreatment, the reaction does not attain dynamic equilibrium in the rate determining step, and encounters the complication of product formation before attaining dynamic equilibrium. The product can establish XB interactions with **A**, which then siphons **A** from the left hand side of the acid dissociation equation, inhibiting formation of maximal "*in-situ*" catalyst described by the acid dissociation constant.

Supplementary Discussion 3

In response to the concern by a reviewer, we generated **3t** in the pure alpha form and mixed with catalyst **A** and diacetone-galactose acceptor (See SI Supplementary Discussion 3), however, NMR analysis after 24 h revealed no change in anomeric selectivities (Supplementary Figure S54, Page S93).

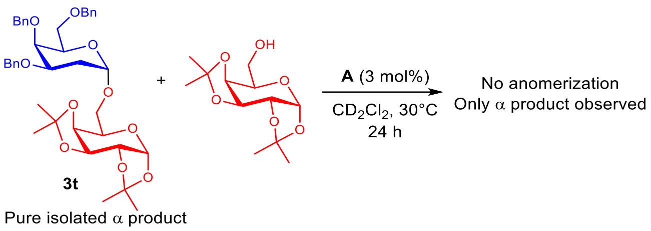

Further dynamic acceptor exchange experiments were not successful with benzylated galactal (Supplementary Figure S55, Page S94 of SI, i.e. no acceptor exchange product detected), supporting the hypothesis that benzylated substrates do not undergo transacetalization under XB catalysis, while silylated substrates do.

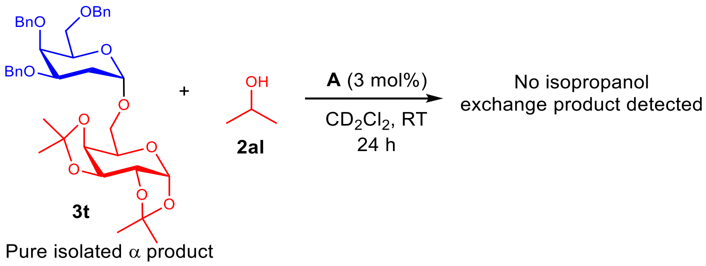

A last confirmatory anomerization control experiment was also conducted, where **3t** is mixed with catalyst **A** directly to ascertain possibility of catalyst controlled anomerization (Supplementary Figure 56, SI Page S94). In this control experiment, no anomerization was detected by ^{1}H NMR after 4 h.

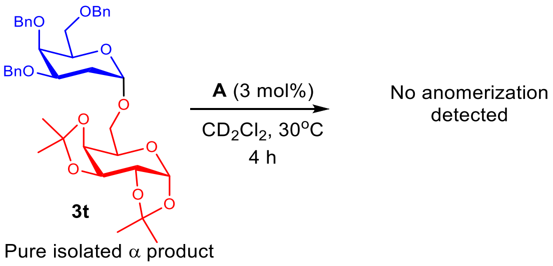

Hence from these control experiments, the lower anomeric selectivity in **3t** generated from XB catalysis is less likely to be indicative of anomerization due to transacetalization. Hence suggesting that the issue of lower stereoselectivities in benzylated substrates is not related to thermodynamic equilibria of transacetalization. For silylated substrates, our acceptor exchange experiments revealed that no beta product was observed throughout the entire timeframe of study, also indicating that the

stereoselectivities is not related to thermodynamic equilibria, since bond cleavage and acceptor reattack occur α-selectively before and after attaining steady state.

Supplementary References

[1]. Sau, A.; Williams, R.; Palo-Nieto, C.; Franconetti, A.; Medina, S.; Galan, M. Palladium-catalyzed direct stereoselective synthesis of deoxyglycosides from glycals. *Angew. Chem. Int. Ed*. **2017**, *56*, 3640-3644.

[2]. Palo-Nieto, C.; Sau, A.; Galan, M. Gold(I)-catalyzed direct stereoselective synthesis of deoxyglycosides from glycals. *J. Am. Chem. Soc*. **2017**, *139*, 14041-14044.

[3]. Das, S.; Pekel, D.; Neudörfl, J.; Berkessel, A. Organocatalytic glycosylation by electron deficient pyridinium salts. *Angew. Chem. Int. Ed*. **2015**, *54*, 12479-12483.

[4]. Balmond, E, I.; Benito-Alifonso, D.; Coe, D. M.; Alder, R. W.; McGarrigle, E. M.; Galan, M. C. A 3,4-trans-fused cyclic protecting group facilitates α-selective catalytic synthesis of 2-deoxyglycosides. *Angew. Chem. Int. Ed*. **2014**, *53*, 8190-8194.

[5]. Balmond, E. I.; Coe, D. M.; Galan, M. C.; McGarrigle, E. M. α-Selective organocatalytic synthesis of 2-deoxygalactosides. *Angew. Chem. Int. Ed*. **2012**, *51*, 9152-9155.

[6]. Brawn, R.; Panek, J. Stereoselective *C*-glycosidations with achiral and enatioenriched allenylsilanes. *Org. Lett*. **2010**, *12*, 4624-4627.

[7]. Brawn, R.; Panek, J. Stereoselective *C*-glycosidations with achiral and enatioenriched allenylsilanes. *Org. Lett*. **2010**, *12*, 5600-5600.

[8]. Thompson, M. S.; Meyer, T. J. Mechanisms of oxidation of 2-propanol by polypyridyl complexes of ruthenium(III) and ruthenium(IV). *J. Am. Chem. Soc*. **1982**, *104*, 4106-4115.

[9]. Brammer, L.; Bruton, E. A.; Sherwood, P. Understanding the behavior of halogens as hydrogen bond acceptors. *Crystal Growth&Design* **2001**, *1*, 277-290.

[10]. Squitieri, R. A.; Fitzpatrick, K. P.; Jaworski, A. A.; Scheidt, K. A. Synthesis and evaluation of azolium-based halogen-bond donors. *Chem. Eur. J*. **2019**, *25*, 10069-10073.

[11]. Li, W.; Jiang, D.; Suna, Y.; Aida, T. Cooperativity in chiroptical sensing with dendritic zinc porphyrins. *J. Am. Chem. Soc*. **2005**, *127*, 7700-7702.

[12]. Benz, S.; Poblador-Bahamonde, A. I.; Low-Ders, N.; Matile, S. Catalysis with Pnictogen, Chalcogen and Halogen Bonds. *Angew. Chem. Int. Ed.* **2018**, *57*, 5408-5412.

[13]. Bradschaw, G. A.; Colgan, A. C.; Allen, N. P.; Pongener, I.; Boland, M. B.; Ortin, Y.; Mcgarrigle, E. M. *Chem. Sci*. **2019**, *10*, 508-514.

Supplementary Tables and Figures

Table number	Title	Page
S1	Optimization for XB catalyzed glycosylation of D-glucal	S47
S2	NMR Titration data	S49
S3	Experimental setup for isothermal titration calorimetry	S49
S4	Concentration table for **1c**, **2al**, **3al** calculated by ^{1}H NMR analysis for the monitoring under standard conditions	S57
S5	Concentration table for **1c**, **2al**, **3al** calculated by ^{1}H NMR analysis for the reaction catalyzed by HCl	S60
S6	Concentration table for **1c**, **2al**, **3al** calculated by ^{1}H NMR analysis for the sequential reaction monitoring	S63
S7	Concentration table for **1c**, **2al**, **3al** calculated by ^{1}H NMR analysis for the reaction with initial addition of 20 mol% **3al**	S66
S8	Concentration table for **1c**, **3al** calculated by ^{1}H NMR analysis for the sequential reaction with following addition of 20 mol% **3al**	S69
S9	Concentration for the product calculated by ^{1}H NMR analysis for the reaction of benzylated galactal **1d** and isopropanol **2al**	S72
S10	Concentration table of **3al** and **3ak** for the acceptor exchange experiments	S89
S11	Concentration table of **3al** and **3ak** for the *in situ* acceptor exchange experiments	S91
S12	Benchmarking experiments comparing XB catalysis with established thiourea and thiouracil catalyzed protocols	S96
S13	Temporal concentrations of product in the presence of catalyst **A** and HCl in CD_2Cl_2	S96
S14	Temporal concentrations of product in the presence of catalyst **A** and TsOH in CD_3CN	S97
S15	Decomposition cases due to *p*TsOH catalysis when challenging tertiary acceptors are employed	S97
S16	Comparison between halogen bond catalysis and other reported leading systems.	S98
S17	Overview for the concentration dependence experiments	S99
S18	Concentration for **3al** calculated by ^{1}H NMR analysis for the experiments of entry 1-3 in supplementary table S17	S103
S19	Concentration for **3al** calculated by ^{1}H NMR analysis for the experiments of entry 1 and entries 4-5 in supplementary table S17	S106
S20	Concentration for **3al** calculated by ^{1}H NMR analysis for the experiments of entry 1 and entries 6-8 in supplementary table S17	S110

Supplementary table S1: Optimization for XB catalyzed glycosylation of D-glucal

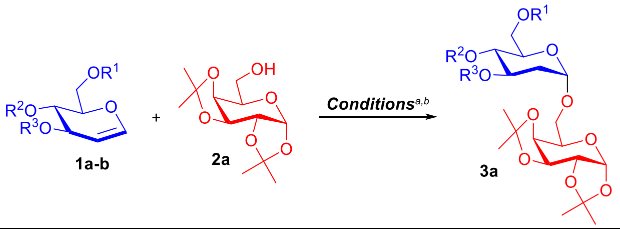

Entry	Cat. (mol%)	Solvent	Temp. /°C	Additive (mol%)	Time /h	R^1	R^2	R^3	**3a** Yield% (α:β)
1	**A** (5 %)	CH_2Cl_2	50	--	10	Bn	Bn	Bn	49% (n.d.)[c]
2	**A** (5 %)	PhF	50	--	10	Bn	Bn	Bn	27% (n.d.)[c]
3	**A** (5 %)	THF	50	--	10	Bn	Bn	Bn	35% (n.d.)[c]
4	**A** (5 %)	CH_2Cl_2	50	--	10	TIPS	$^{i}Pr_2SiOSi^{i}Pr_2$		91% (>20:1)
5	**B** (5 %)	CH_2Cl_2	50	--	10	TIPS	$^{i}Pr_2SiOSi^{i}Pr_2$		86% (>20:1)
6	**C** (5 %)	CH_2Cl_2	50	--	10	TIPS	$^{i}Pr_2SiOSi^{i}Pr_2$		79% (>20:1)
7	**D** (5 %)	CH_2Cl_2	50	--	10	TIPS	$^{i}Pr_2SiOSi^{i}Pr_2$		7% (>20:1)[d]
8	**E** (5 %)	CH_2Cl_2	50	--	10	TIPS	$^{i}Pr_2SiOSi^{i}Pr_2$		83% (>20:1)
9	**A** (5%)	CH_2Cl_2	50	TBAB (10%)	10	TIPS	$^{i}Pr_2SiOSi^{i}Pr_2$		<5% (n.d.)
10	**A** (5%)	CH_2Cl_2	50	TBAI (10%)	10	TIPS	$^{i}Pr_2SiOSi^{i}Pr_2$		<5% (n.d.)
11	**HCl** (5%)	CH_2Cl_2	50	--	10	TIPS	$^{i}Pr_2SiOSi^{i}Pr_2$		86% (>20:1)
12	**HCl** (5%)	CH_2Cl_2	50	TBAB (10%)	10	TIPS	$^{i}Pr_2SiOSi^{i}Pr_2$		80% (>20:1)
13	**A** (5%)	CH_2Cl_2	50	**F** (10%)	10	TIPS	$^{i}Pr_2SiOSi^{i}Pr_2$		<5% (n.d.)
14	**A** (5%)	CH_2Cl_2	50	**F** (5%)	10	TIPS	$^{i}Pr_2SiOSi^{i}Pr_2$		<5% (n.d.)
15	**A** (5%)	CH_2Cl_2	50	K_2CO_3 (10%)	10	TIPS	$^{i}Pr_2SiOSi^{i}Pr_2$		<5% (n.d.)
16	**A** (5%)	CH_2Cl_2	50	--	24	TIPS	$^{i}Pr_2SiOSi^{i}Pr_2$		92% (93:7)[e]
17	--	CH_2Cl_2	50	--	24	TIPS	$^{i}Pr_2SiOSi^{i}Pr_2$		<5% (n.d.)
18	**A** (3%)	CH_2Cl_2	40	--	48	TIPS	$^{i}Pr_2SiOSi^{i}Pr_2$		89% (>20:1)[f]
19	**E** (5 %)	CH_2Cl_2	50	TBAI (10%)	10	TIPS	$^{i}Pr_2SiOSi^{i}Pr_2$		<5% (n.d.)[g]
20	**TfOH** (5 %)	CH_2Cl_2	50	--	10	TIPS	$^{i}Pr_2SiOSi^{i}Pr_2$		32% (n.d.)[h]
21	**TfOH** (5 %)	CH_2Cl_2	50	TBAB (10%)	10	TIPS	$^{i}Pr_2SiOSi^{i}Pr_2$		24% (n.d.)[h]
22	**TfOH** (5 %)	CH_2Cl_2	50	TBAI (10%)	10	TIPS	$^{i}Pr_2SiOSi^{i}Pr_2$		28% (n.d.)[h]
23	**TfOH** (1 %)	CH_2Cl_2	50	--	10	TIPS	$^{i}Pr_2SiOSi^{i}Pr_2$		19% (n.d.)[h]
24	**TfOH** (1 %)	CH_2Cl_2	50	TBAI (1%)	10	TIPS	$^{i}Pr_2SiOSi^{i}Pr_2$		*quantitative* (>20:1)[f]

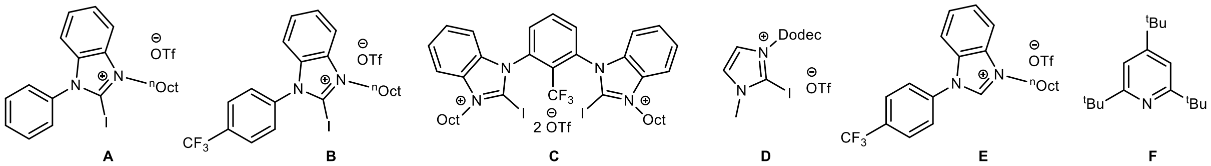

Conditions:[a]**2a** (0.1 mmol), **1a-b** (0.15 mmol), catalyst, temp.; solvent (0.2 M), time, argon; [b]yield and α/β ratio were determined by crude ^{1}H NMR spectra analysis using 1,3,5-trimethoxybenzene as an internal standard; [c]40-50% NMR yields of Ferrier rearrangement side-products detected; [d]92% **1b** remaining; [e]**2a** (0.2 mmol), **1b** (0.3 mmol), CH_2Cl_2 (0.2 M); [f]isolated yield; [g]*Halides are known to be good hydrogen bond acceptors in the literature*[9] *and lead to inhibition of hydrogen bond catalyst.* The spectra in Supplementary figure S34-35 show the interaction between compound **E** and TBAI; [h]see Supplementary figure S1 and Supplementary Note 17. n.d. = not determined. ^{i}Pr: isopropyl. TBAB: tetrabutylammonium bromide. TBAI: tetrabutylammonium iodide.

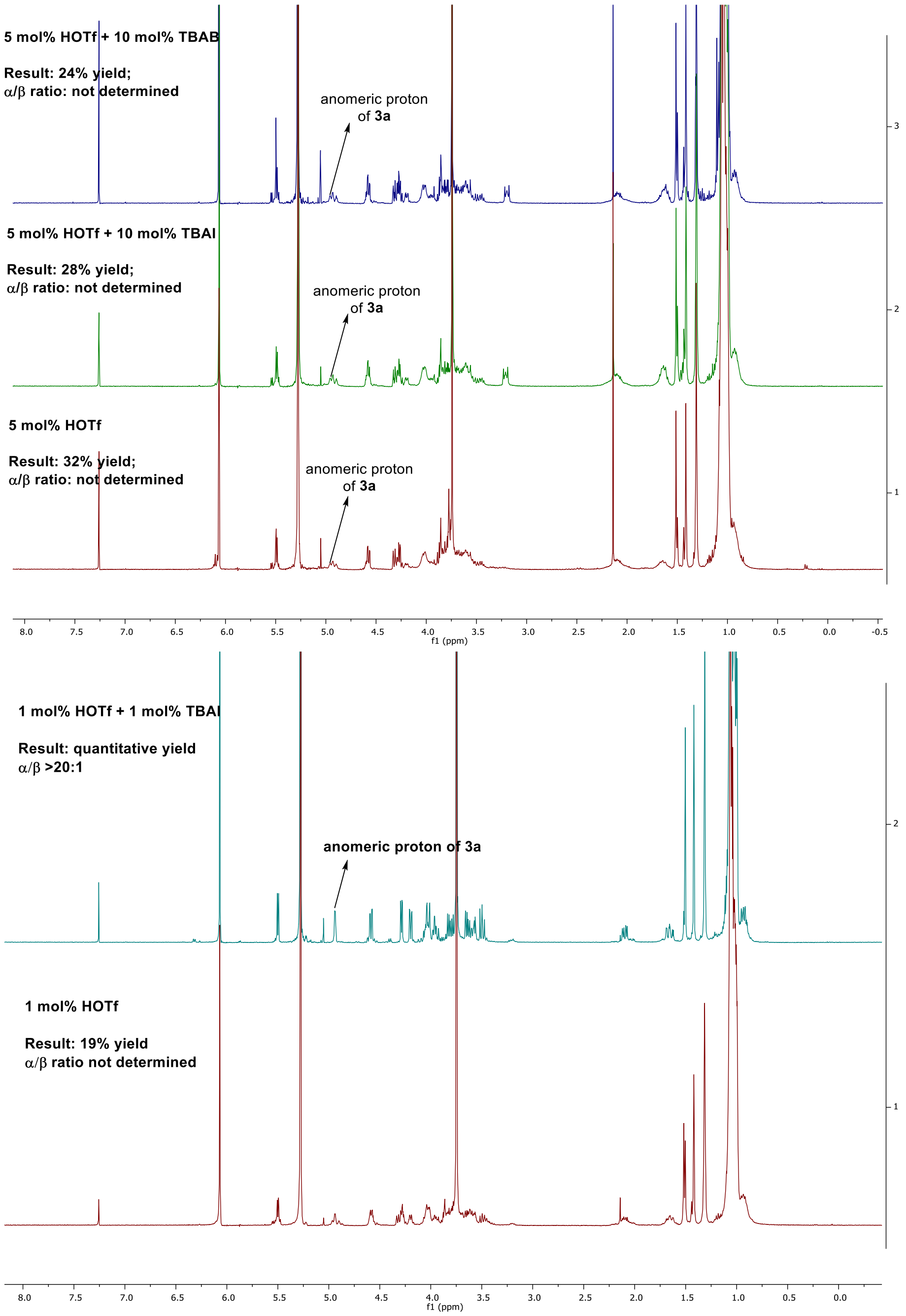

Supplementary figure S1: Crude ^{1}H NMR spectra for the reactions with HOTf (5 mol% or 1 mol%) in the absence of additive or in the presence of TBAB or TBAI

Supplementary table S2. NMR Titration data (Related to Figure 4a-b in the manuscript)

The results of the titration of halogen bond catalyst **A** with isopropanol **2al** are depicted below.

Entry	Concentration of **A** (M)	Equivalents of isopropanol **2al**	Concentration of isopropanol **2al**	δ (*C*-I) /ppm	Change of δ (*C*-I)/ppm
1	0.02	0	0	112.91	0
2	0.02	1	0.02	112.97	0.053
3	0.02	4	0.08	112.95	0.034
4	0.02	7	0.14	113.04	0.130
5	0.02	10	0.20	113.47	0.560
6	0.02	15	0.30	113.34	0.431
7	0.02	20	0.40	113.51	0.598
8	0.02	25	0.50	113.63	0.716
9	0.02	35	0.70	113.95	1.035
10	0.02	100	2.00	114.95	2.037
11	0.02	150	3.00	115.34	2.427
12	0.02	200	4.00	115.58	2.663

Graphical depiction:

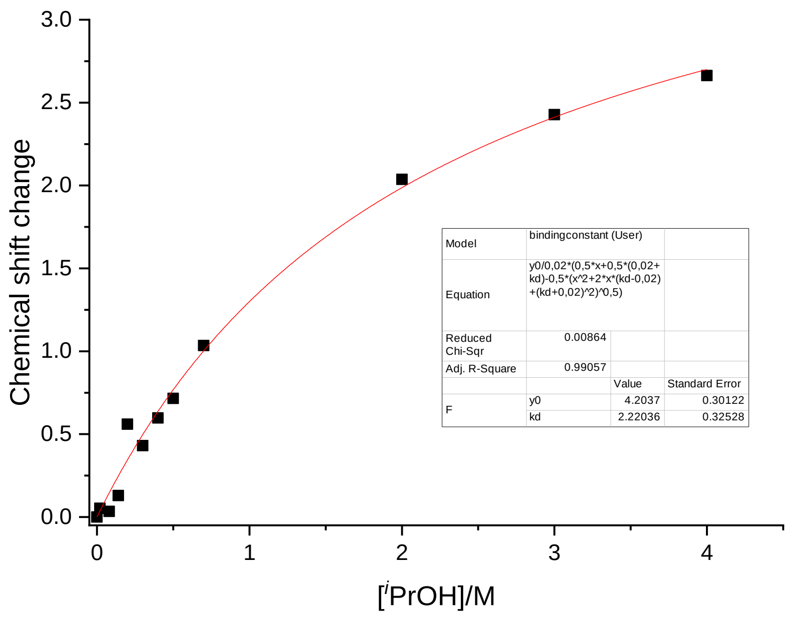

Supplementary figure S2: Experimental (black points) and fitted (red line) titration curve; *y*-axis: change of *C*-I NMR shift in ppm; *x*-axis: concentration of isopropanol **2al.**

Supplementary table S3: Experimental setup for isothermal titration calorimetry

**Host**	**Guest**	***Setup***	***c(syringe)* [mM]**	***c(cell)* [mM]**
Catalyst **A**	TBAB	Guest to Host	10	1
Catalyst **A**	TBAI	Guest to Host	10	1

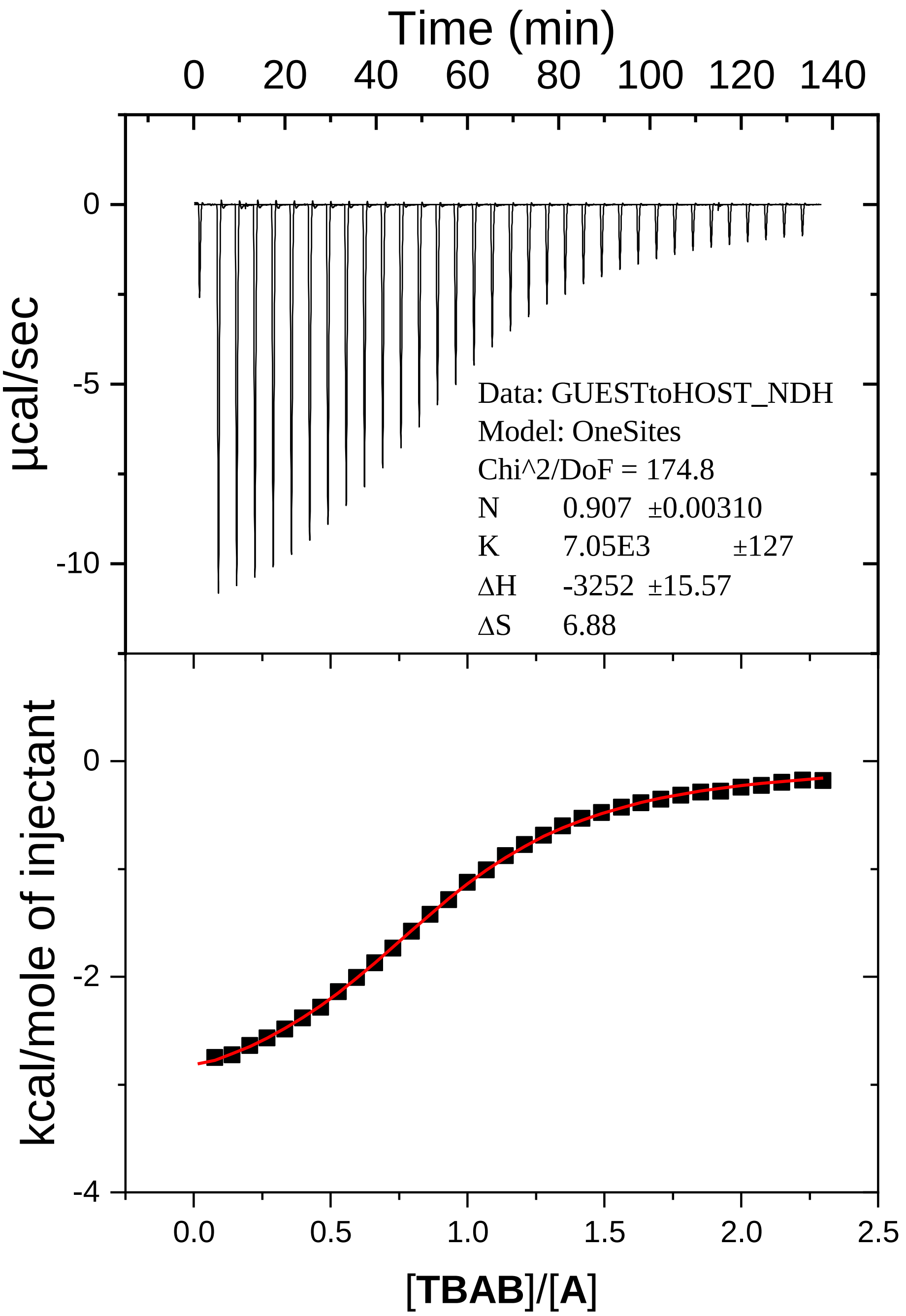

Supplementary figure S3: Titration of $^{n}Bu_4NBr$ to catalyst **A** in MeCN at 30 ˚C

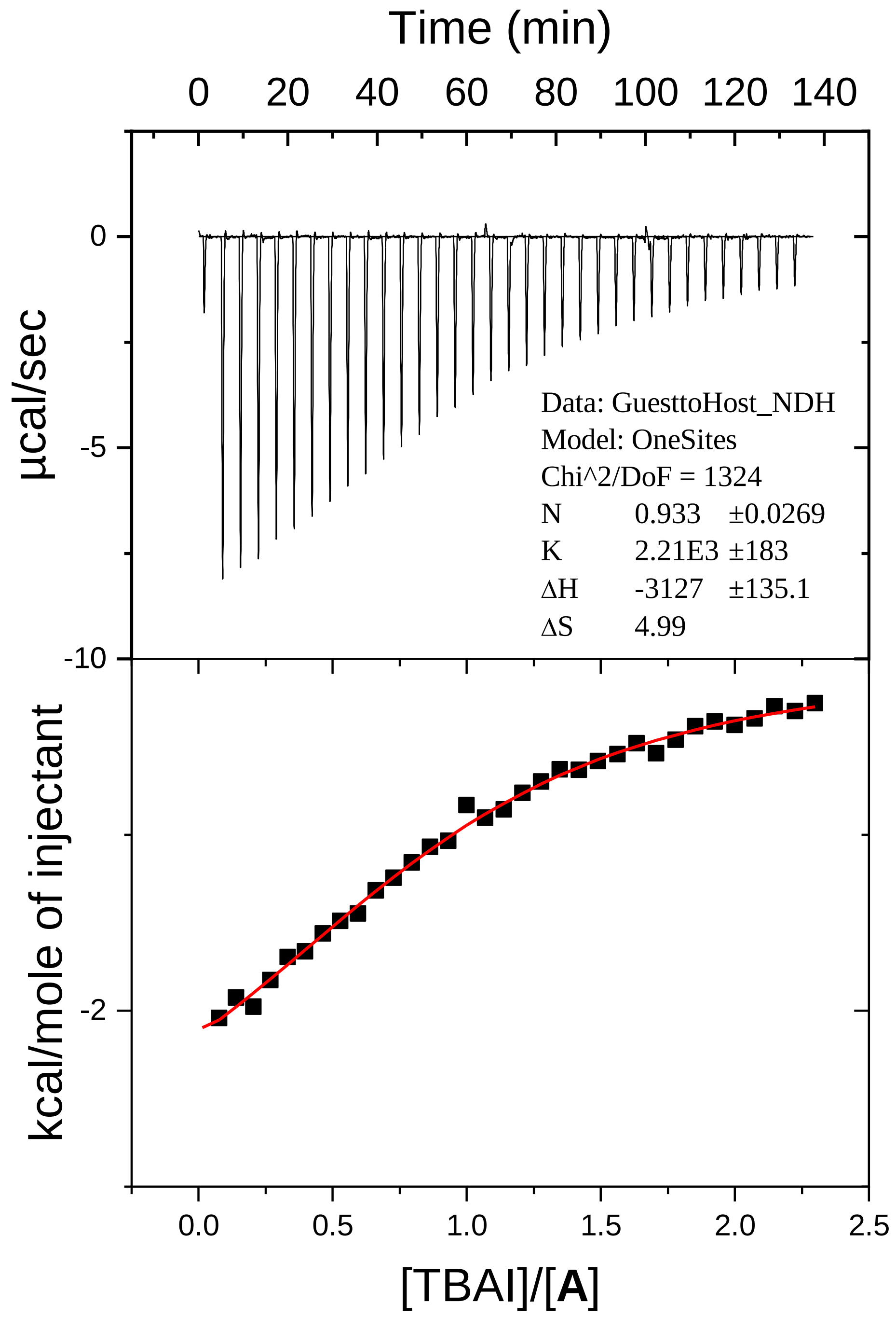

Supplementary figure S4: Titration of ${}^{n}Bu_4NI$ to catalyst **A** in MeCN at 30 ˚C

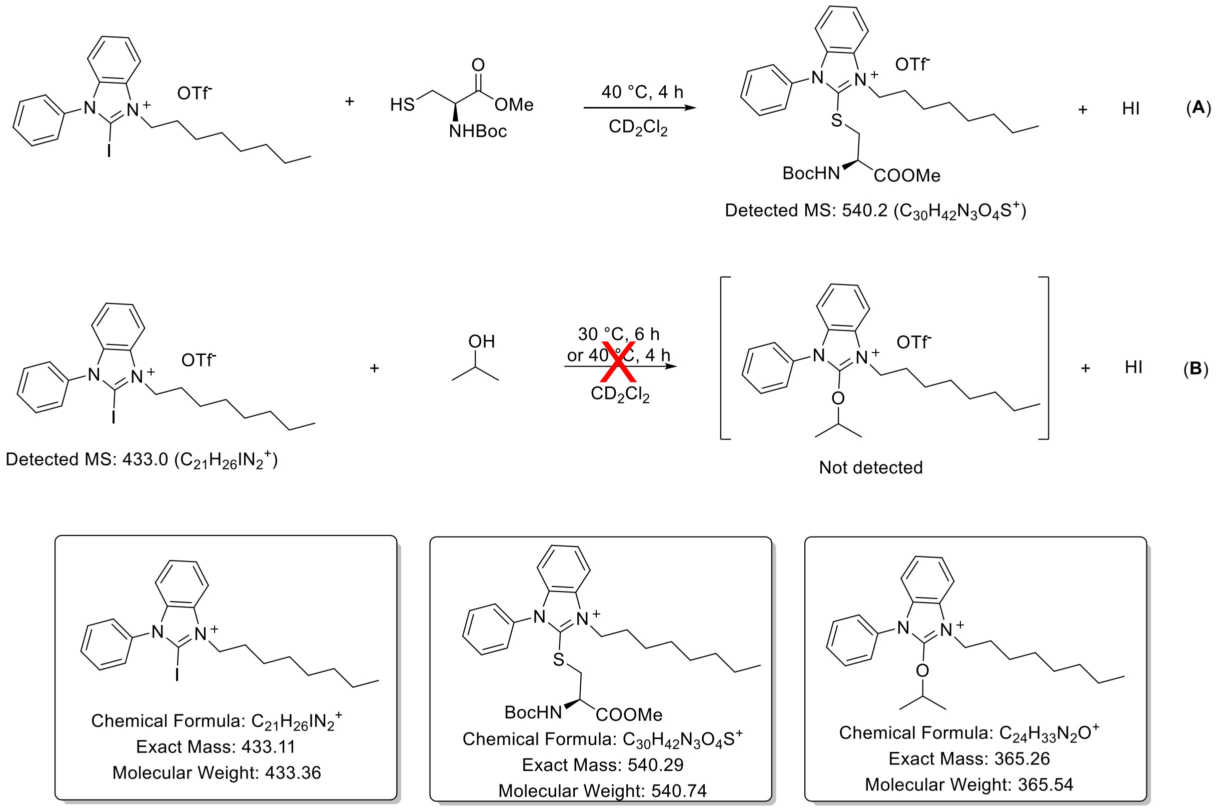

Supplementary figure S5. Reactions of catalyst **A** with *N*-(*tert*-Butoxycarbonyl)-L-cysteine methyl ester or ^{i}PrOH **2al**

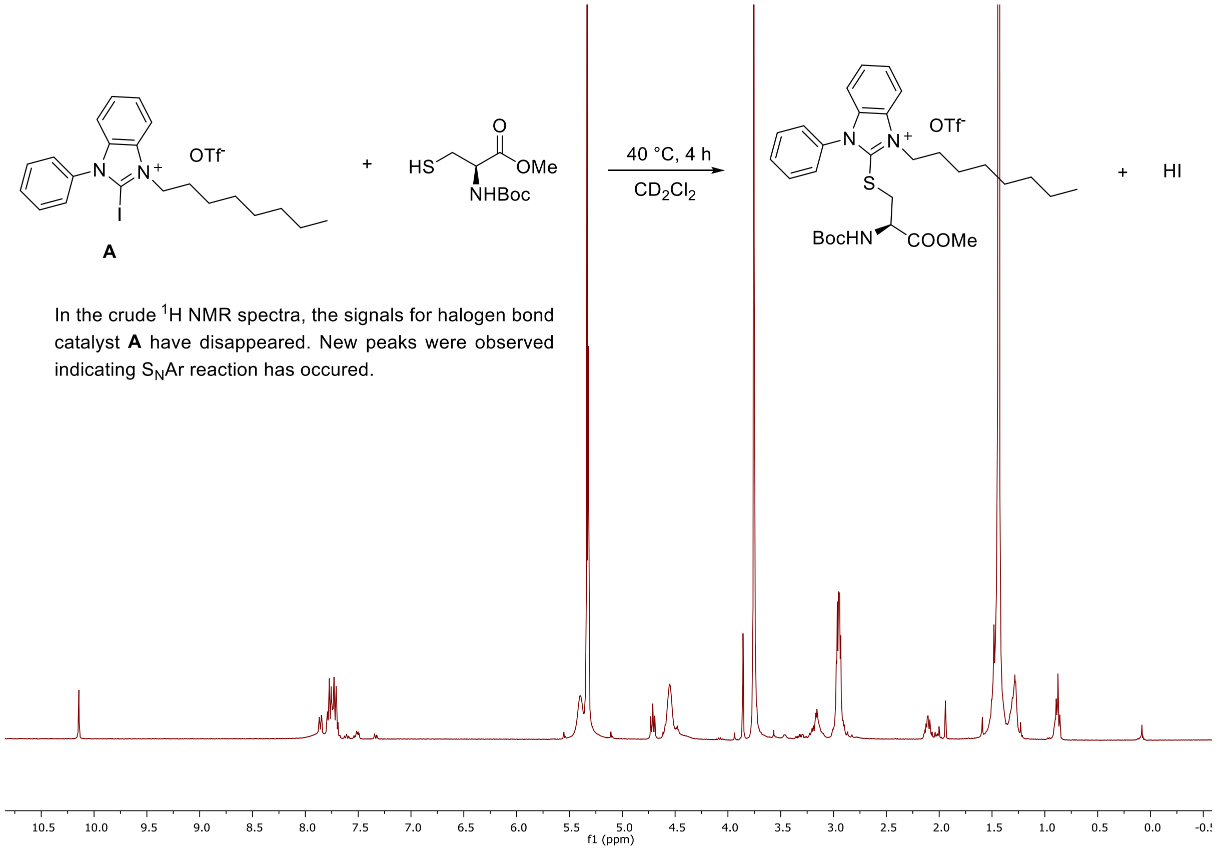

Supplementary figure S6. Crude ^{1}H NMR spectrum for reaction (**A**) in the supplementary figure S5

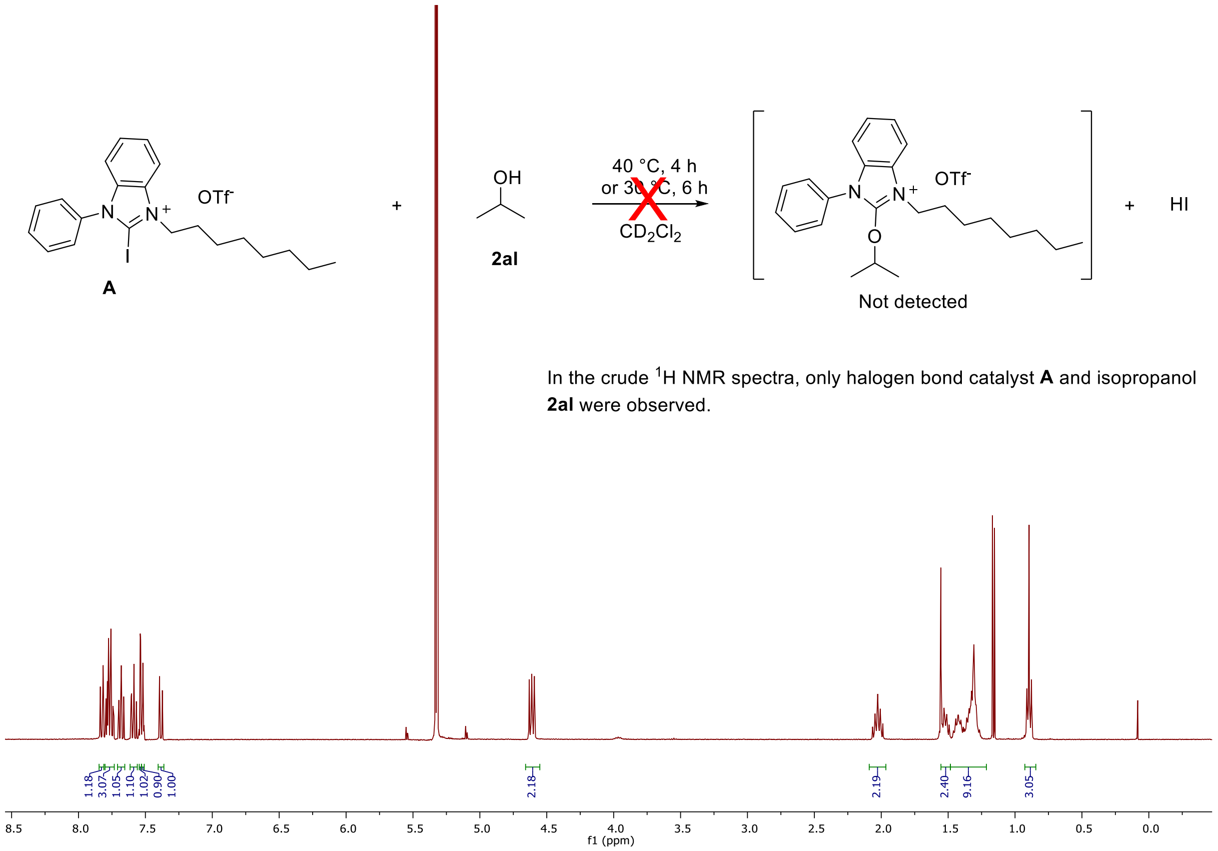

Supplementary figure S7. Crude ^{1}H NMR spectrum for reaction (**B**) in the supplementary figure S5

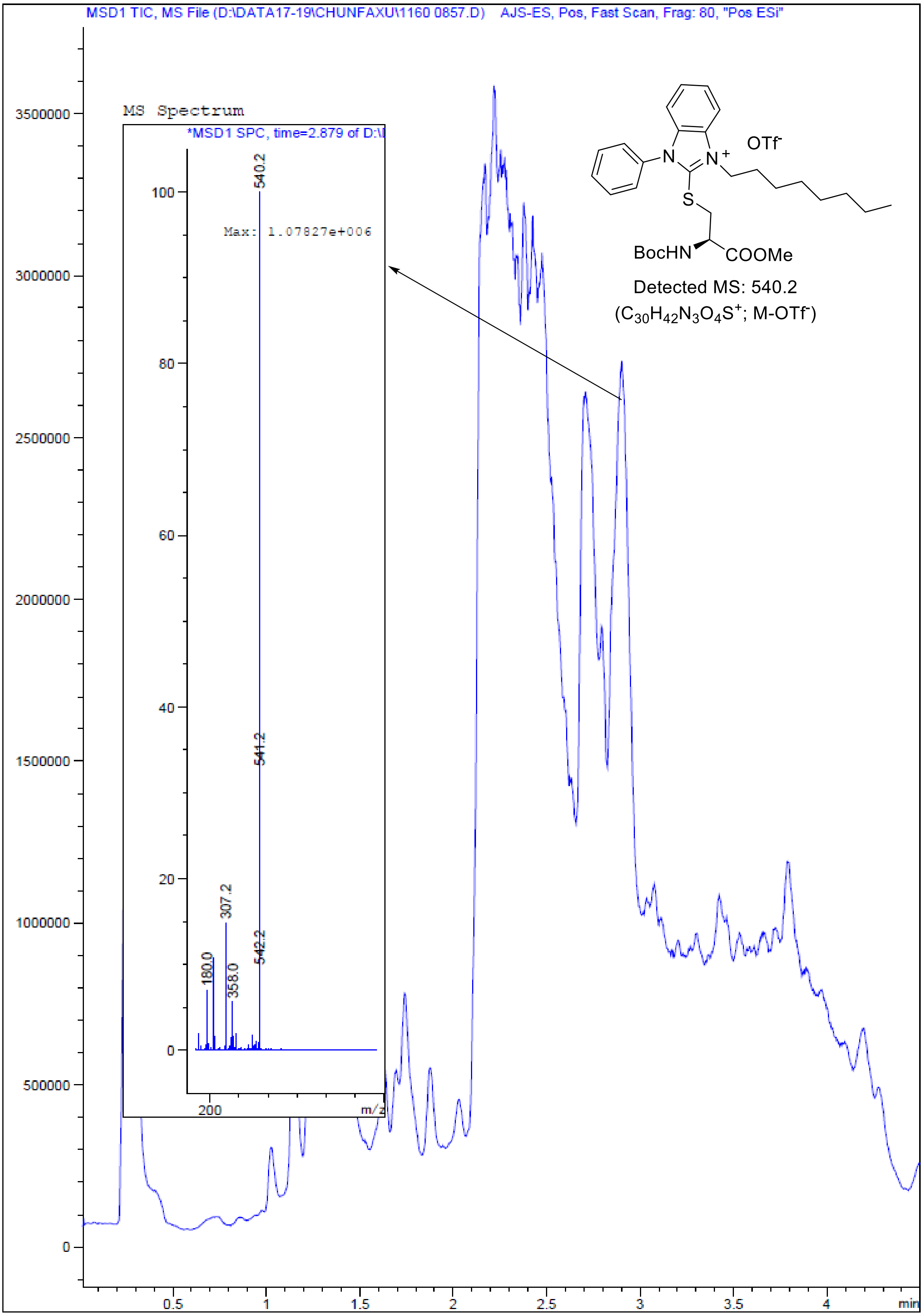

Supplementary figure S8: Crude LC-MS spectrum for reaction (**A**) in the supplementary figure S5 confirming the S_NAr product

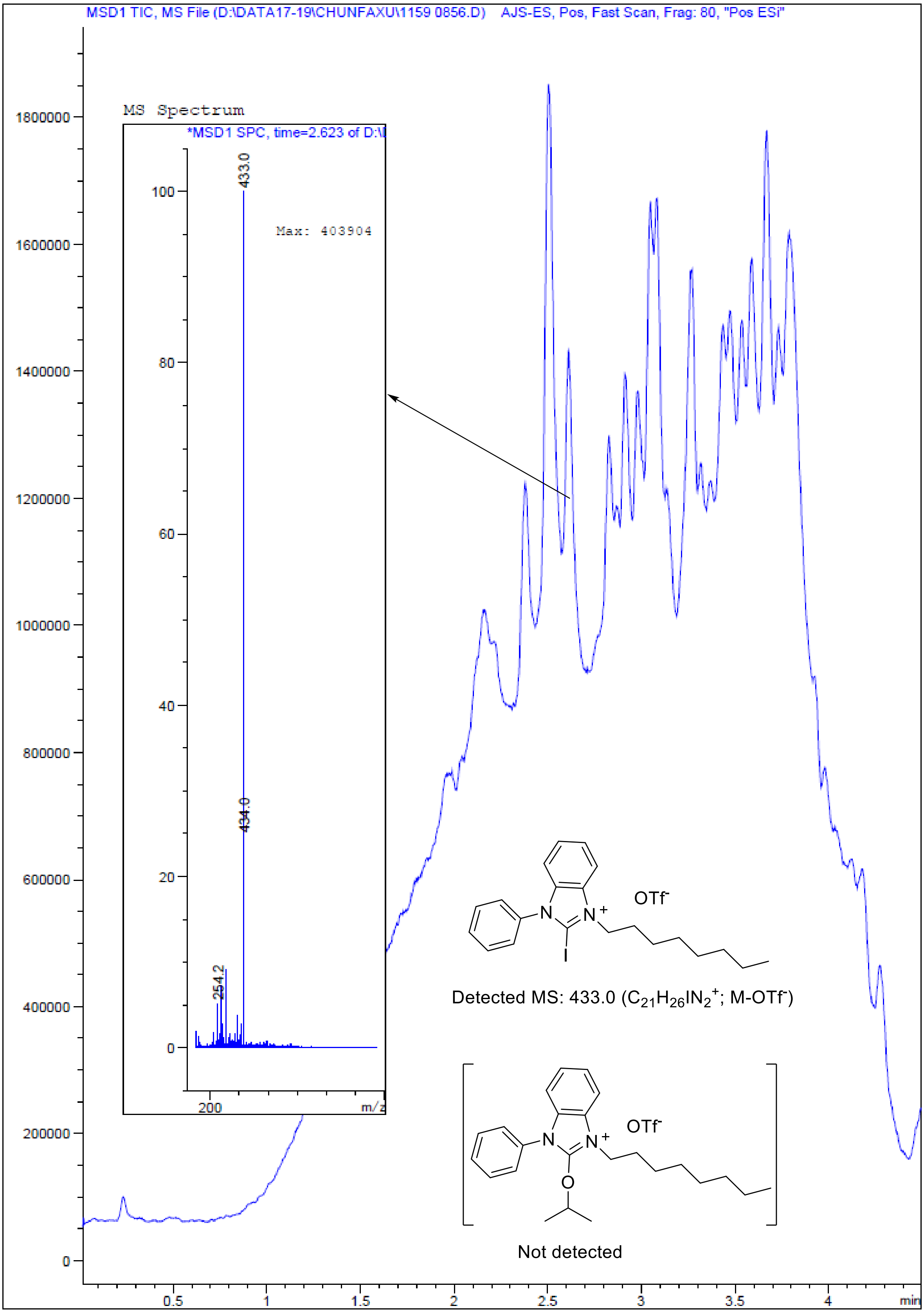

Supplementary figure S9: Crude LC-MS spectrum for reaction (**B**) in the supplementary figure S5 confirming presence of catalyst **A** and excluding the S_NAr reaction

***In situ* NMR monitoring data for kinetics studies:**

1. *In situ* NMR monitoring under standard conditions (Related to Figure 6a in the manuscript)

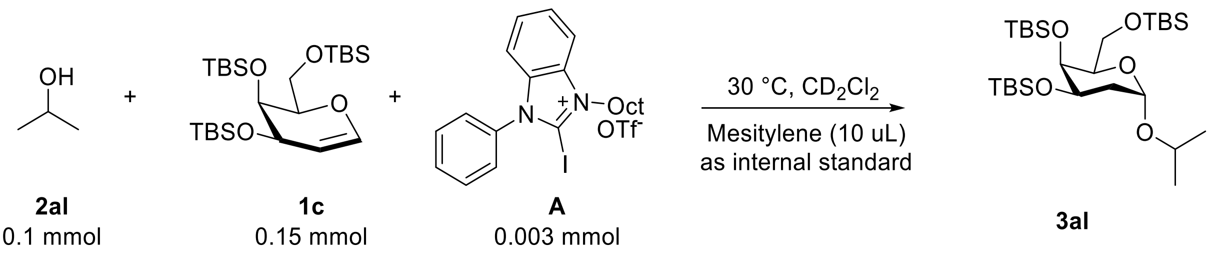

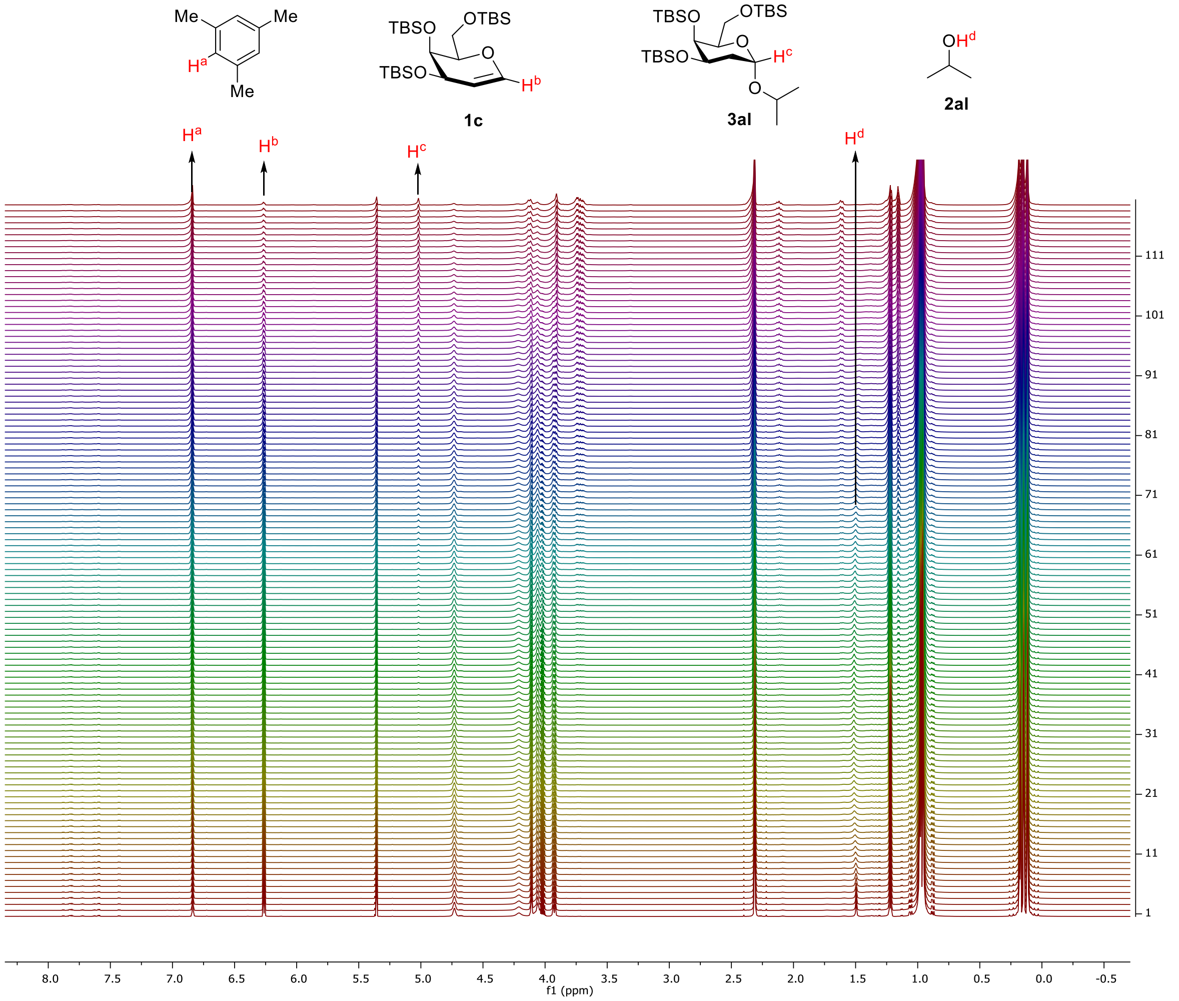

Supplementary figure S10: Stacked ^1H NMR spectra for the monitoring under standard conditions

Supplementary table S4: Concentration for **1c**, **2al**, **3al** calculated by ^{1}H NMR analysis for the monitoring under standard conditions

Time /min	[1c] /M	[2al] /M	[3al] /M	Time /min	[1c] /M	[2al] /M	[3al] /M	Time /min	[1c] /M	[2al] /M	[3al] /M
**7**	0.286	0.182	0.000	**397**	0.275	0.172	0.005	**787**	0.221	0.124	0.051
**17**	0.287	0.181	0.000	**407**	0.273	0.171	0.005	**797**	0.218	0.121	0.054
**27**	0.287	0.181	0.000	**417**	0.273	0.170	0.006	**807**	0.216	0.119	0.056
**37**	0.287	0.180	0.000	**427**	0.273	0.169	0.006	**817**	0.213	0.118	0.059
**47**	0.285	0.180	0.000	**437**	0.272	0.166	0.008	**827**	0.211	0.117	0.059
**57**	0.287	0.180	0.000	**447**	0.272	0.167	0.009	**837**	0.206	0.114	0.061
**67**	0.287	0.179	0.000	**457**	0.271	0.164	0.009	**847**	0.203	0.111	0.065
**77**	0.287	0.172	0.000	**467**	0.270	0.162	0.009	**857**	0.201	0.109	0.069
**87**	0.286	0.177	0.000	**477**	0.269	0.162	0.010	**867**	0.198	0.104	0.070
**97**	0.286	0.177	0.000	**487**	0.267	0.161	0.010	**877**	0.195	0.102	0.074
**107**	0.287	0.176	0.000	**497**	0.267	0.159	0.013	**887**	0.191	0.098	0.075
**117**	0.286	0.177	0.000	**507**	0.265	0.158	0.013	**897**	0.187	0.095	0.079
**127**	0.286	0.179	0.000	**517**	0.265	0.156	0.015	**907**	0.183	0.094	0.080
**137**	0.285	0.176	0.000	**527**	0.264	0.160	0.015	**917**	0.177	0.088	0.086
**147**	0.286	0.176	0.000	**537**	0.262	0.157	0.017	**927**	0.173	0.084	0.092
**157**	0.286	0.177	0.000	**547**	0.260	0.156	0.017	**937**	0.170	0.080	0.095
**167**	0.285	0.177	0.000	**557**	0.260	0.155	0.018	**947**	0.164	0.079	0.097
**177**	0.286	0.177	0.000	**567**	0.257	0.154	0.018	**957**	0.158	0.077	0.101
**187**	0.285	0.176	0.000	**577**	0.258	0.154	0.019	**967**	0.152	0.076	0.103
**197**	0.283	0.179	0.000	**587**	0.256	0.153	0.020	**977**	0.146	0.065	0.110
**207**	0.283	0.177	0.001	**597**	0.255	0.152	0.023	**987**	0.141	0.059	0.115
**217**	0.283	0.179	0.000	**607**	0.254	0.152	0.024	**997**	0.135	0.057	0.119
**227**	0.282	0.179	0.000	**617**	0.252	0.151	0.024	**1007**	0.128	0.054	0.123
**237**	0.282	0.181	0.000	**627**	0.250	0.151	0.025	**1017**	0.123	0.046	0.125
**247**	0.282	0.176	0.000	**637**	0.250	0.147	0.026	**1027**	0.116	0.043	0.129
**257**	0.282	0.176	0.001	**647**	0.249	0.146	0.027	**1037**	0.109	0.039	0.134
**267**	0.282	0.180	0.000	**657**	0.246	0.145	0.027	**1047**	0.103	0.031	0.139
**277**	0.281	0.177	0.001	**667**	0.244	0.142	0.031	**1057**	0.098	0.028	0.141
**287**	0.281	0.177	0.001	**677**	0.244	0.142	0.032	**1067**	0.093	0.026	0.144
**297**	0.281	0.177	0.001	**687**	0.241	0.142	0.032	**1077**	0.088	0.026	0.147
**307**	0.281	0.177	0.000	**697**	0.238	0.140	0.035	**1087**	0.084	0.025	0.149
**317**	0.280	0.176	0.002	**707**	0.238	0.139	0.036	**1097**	0.081	0.022	0.150
**327**	0.279	0.176	0.003	**717**	0.236	0.137	0.037	**1107**	0.079	0.025	0.151
**337**	0.279	0.177	0.003	**727**	0.232	0.133	0.040	**1117**	0.076	0.025	0.151
**347**	0.279	0.173	0.003	**737**	0.233	0.131	0.041	**1127**	0.075	0.025	0.151
**357**	0.277	0.175	0.003	**747**	0.230	0.134	0.042	**1137**	0.072	0.025	0.151
**367**	0.277	0.173	0.003	**757**	0.227	0.129	0.045	**1147**	0.071	0.025	0.150
**377**	0.275	0.171	0.004	**767**	0.226	0.130	0.046	**1157**	0.071	0.024	0.149
**387**	0.275	0.172	0.005	**777**	0.224	0.125	0.050				

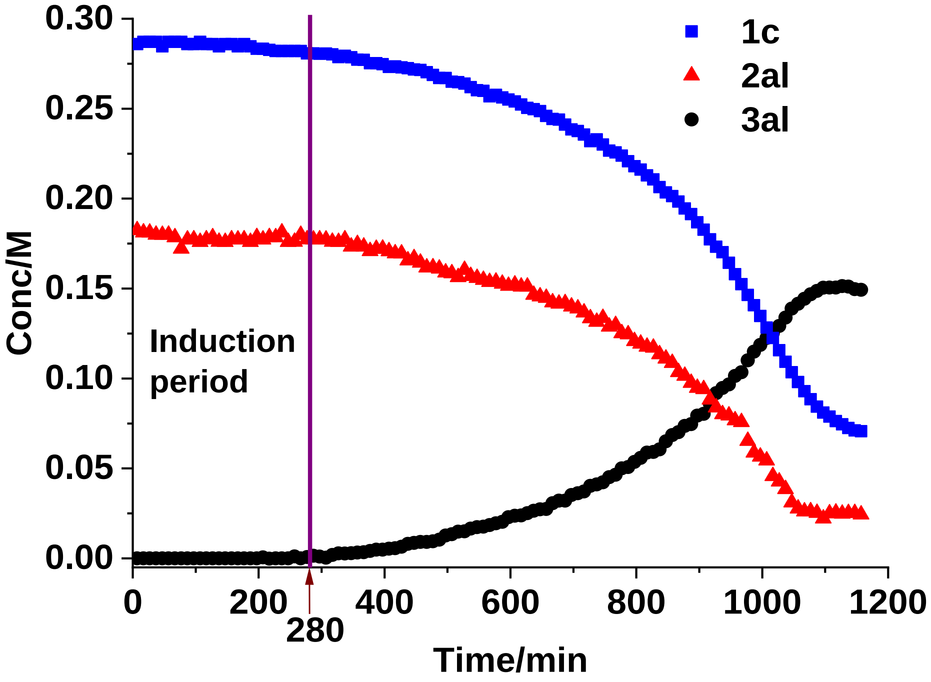

Supplementary figure S11: Temporal kinetics profile for the monitoring under standard conditions

2. *In situ* NMR monitoring for the reaction utilizing HCl as catalyst (Related to Figure 6b in the manuscript)

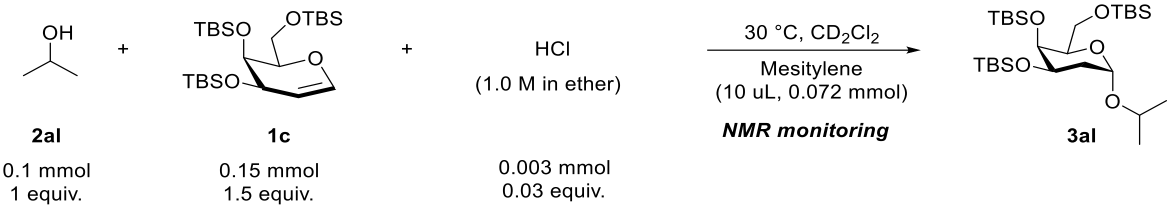

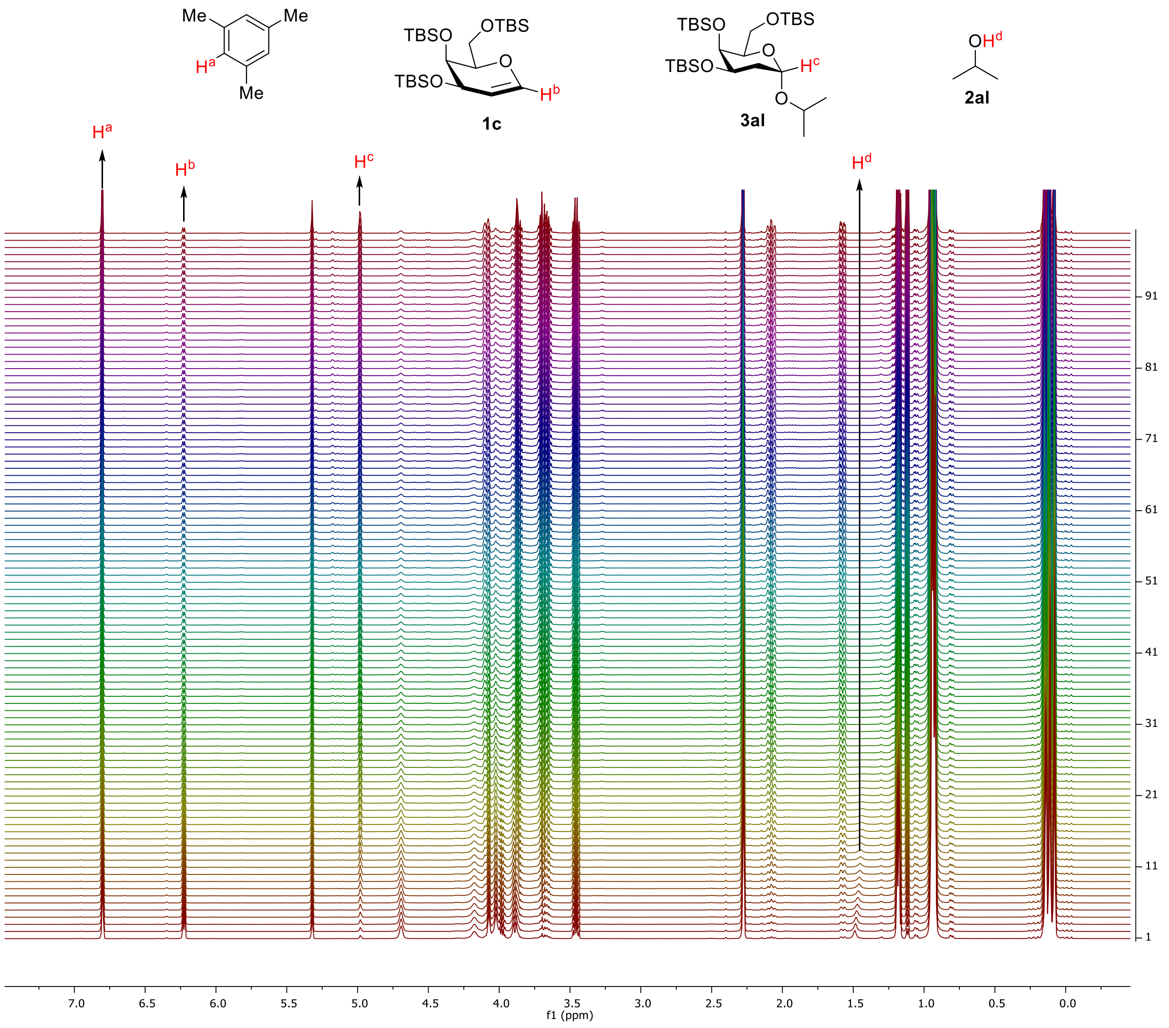

Supplementary figure S12: Stacked ^{1}H NMR spectra for the reaction utilizing HCl as catalyst

Supplementary table S5: Concentration for **1c**, **2al**, **3al** calculated by ^{1}H NMR analysis for the reaction catalyzed by HCl

Time /min	[1c] /M	[2al] /M	[3al] /M	Time /min	[1c] /M	[2al] /M	[3al] /M	Time /min	[1c] /M	[2al] /M	[3al] /M
**14.5**	0.272	0.170	0.036	**179.0**	0.0788	0.001	0.189	**343.3**	0.0489	0.002	0.199
**19.5**	0.252	0.159	0.041	**183.8**	0.0767	0.003	0.191	**348.1**	0.0493	0.003	0.200
**24.3**	0.244	0.152	0.048	**188.6**	0.0745	0.004	0.192	**353.0**	0.0484	0.002	0.199
**29.1**	0.237	0.143	0.054	**193.5**	0.0726	0.004	0.193	**357.8**	0.0483	0.001	0.200
**34.0**	0.230	0.134	0.061	**198.3**	0.0706	0.002	0.194	**362.6**	0.0481	0.001	0.200
**38.8**	0.222	0.127	0.067	**203.1**	0.0687	0.001	0.194	**367.5**	0.0481	0.001	0.200
**43.6**	0.215	0.120	0.074	**208.0**	0.0674	0.004	0.196	**372.3**	0.0476	0.001	0.199
**48.5**	0.208	0.112	0.080	**212.8**	0.0657	0.002	0.196	**377.1**	0.0479	0.002	0.200
**53.3**	0.202	0.105	0.086	**217.6**	0.064	0.001	0.196	**382.0**	0.0478	0.002	0.200
**58.1**	0.195	0.096	0.092	**222.5**	0.0628	0.001	0.197	**386.8**	0.0476	0.001	0.200
**63.0**	0.188	0.089	0.098	**227.3**	0.0613	0.001	0.197	**391.6**	0.0472	0.001	0.199
**67.8**	0.182	0.079	0.104	**232.1**	0.0607	0.003	0.198	**396.5**	0.0473	0.001	0.199
**72.6**	0.176	0.071	0.109	**237.0**	0.0593	0.001	0.197	**401.3**	0.0475	0.003	0.200
**77.5**	0.169	0.064	0.115	**241.8**	0.0581	0.002	0.198	**406.1**	0.0470	0.002	0.200
**82.3**	0.163	0.058	0.120	**246.6**	0.0575	0.002	0.198	**411.0**	0.0468	0.000	0.199
**87.1**	0.157	0.051	0.125	**251.5**	0.0565	0.001	0.198	**415.8**	0.0468	0.001	0.200
**92.0**	0.152	0.047	0.131	**256.3**	0.0559	0.002	0.199	**420.6**	0.0469	0.002	0.200
**96.8**	0.146	0.042	0.136	**261.1**	0.0549	0.001	0.198	**425.5**	0.0465	0.002	0.200
**101.6**	0.140	0.038	0.141	**266.0**	0.0545	0.003	0.199	**430.3**	0.0468	0.003	0.200
**106.5**	0.135	0.035	0.146	**270.8**	0.0539	0.003	0.199	**435.1**	0.0465	0.001	0.200
**111.3**	0.130	0.031	0.150	**275.6**	0.0531	0.000	0.199	**440.0**	0.0464	0.001	0.200
**116.1**	0.125	0.028	0.154	**280.5**	0.0527	0.001	0.199	**444.8**	0.0465	0.002	0.200
**121.0**	0.120	0.025	0.159	**285.3**	0.0526	0.003	0.200	**449.6**	0.0466	0.003	0.200
**125.8**	0.115	0.022	0.163	**290.1**	0.0522	0.003	0.200	**454.5**	0.0461	0.001	0.200
**130.6**	0.111	0.019	0.166	**295.0**	0.0517	0.003	0.200	**459.3**	0.0461	0.002	0.200
**135.5**	0.107	0.013	0.170	**299.8**	0.0514	0.003	0.200	**464.1**	0.0461	0.001	0.200
**140.3**	0.103	0.014	0.173	**304.6**	0.0512	0.003	0.200	**469.0**	0.0460	0.001	0.200
**145.1**	0.0993	0.010	0.175	**309.5**	0.0505	0.002	0.199	**473.8**	0.0461	0.005	0.200
**150.0**	0.0959	0.009	0.178	**314.3**	0.0504	0.003	0.200	**478.6**	0.0461	0.003	0.200
**154.8**	0.0927	0.007	0.181	**319.1**	0.0499	0.001	0.199	**483.5**	0.0458	0.003	0.200
**159.6**	0.0895	0.006	0.183	**324.0**	0.0499	0.002	0.200	**488.3**	0.0458	0.002	0.200
**164.5**	0.0868	0.003	0.185	**328.8**	0.0496	0.003	0.200	**493.1**	0.0454	0.001	0.199
**169.3**	0.0840	0.005	0.187	**333.6**	0.0495	0.001	0.200				
**174.1**	0.0812	0.002	0.188	**338.5**	0.0490	0.001	0.199				

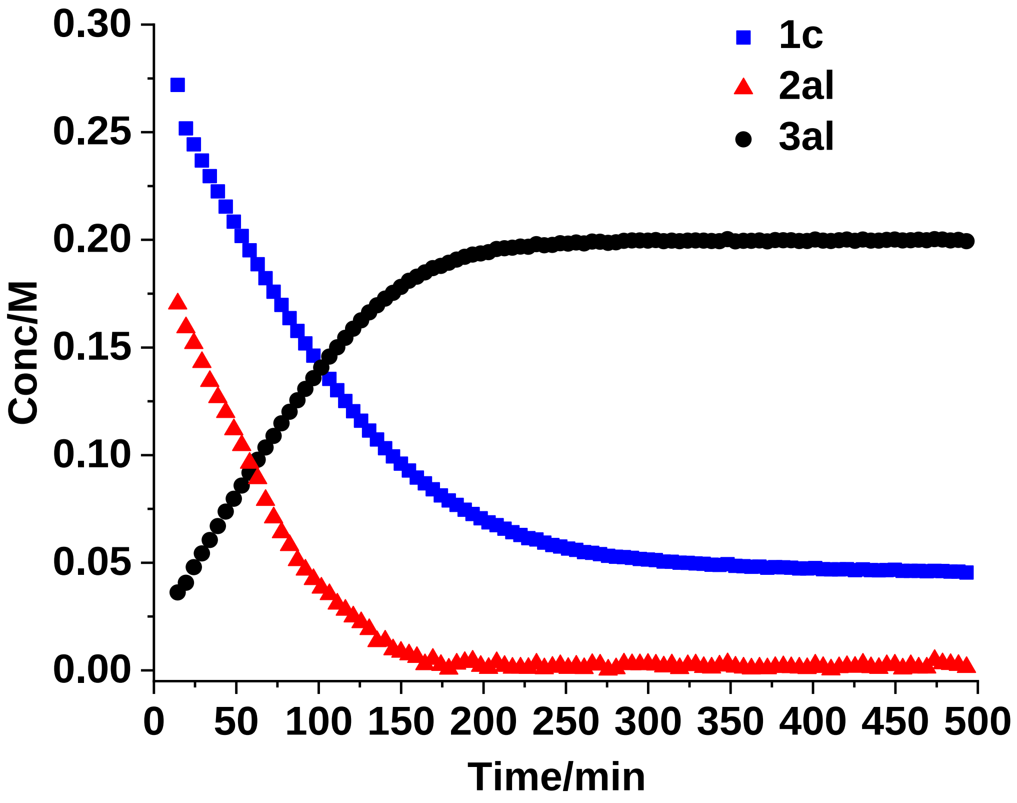

Supplementary figure S13: Temporal kinetics profile for the reaction utilizing HCl as catalyst

3. *In situ* NMR monitoring for the sequential reaction (Related to Figure 7a in the manuscript)

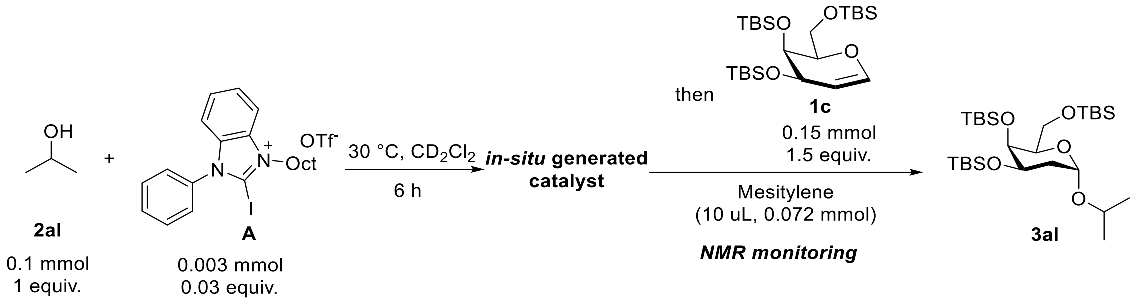

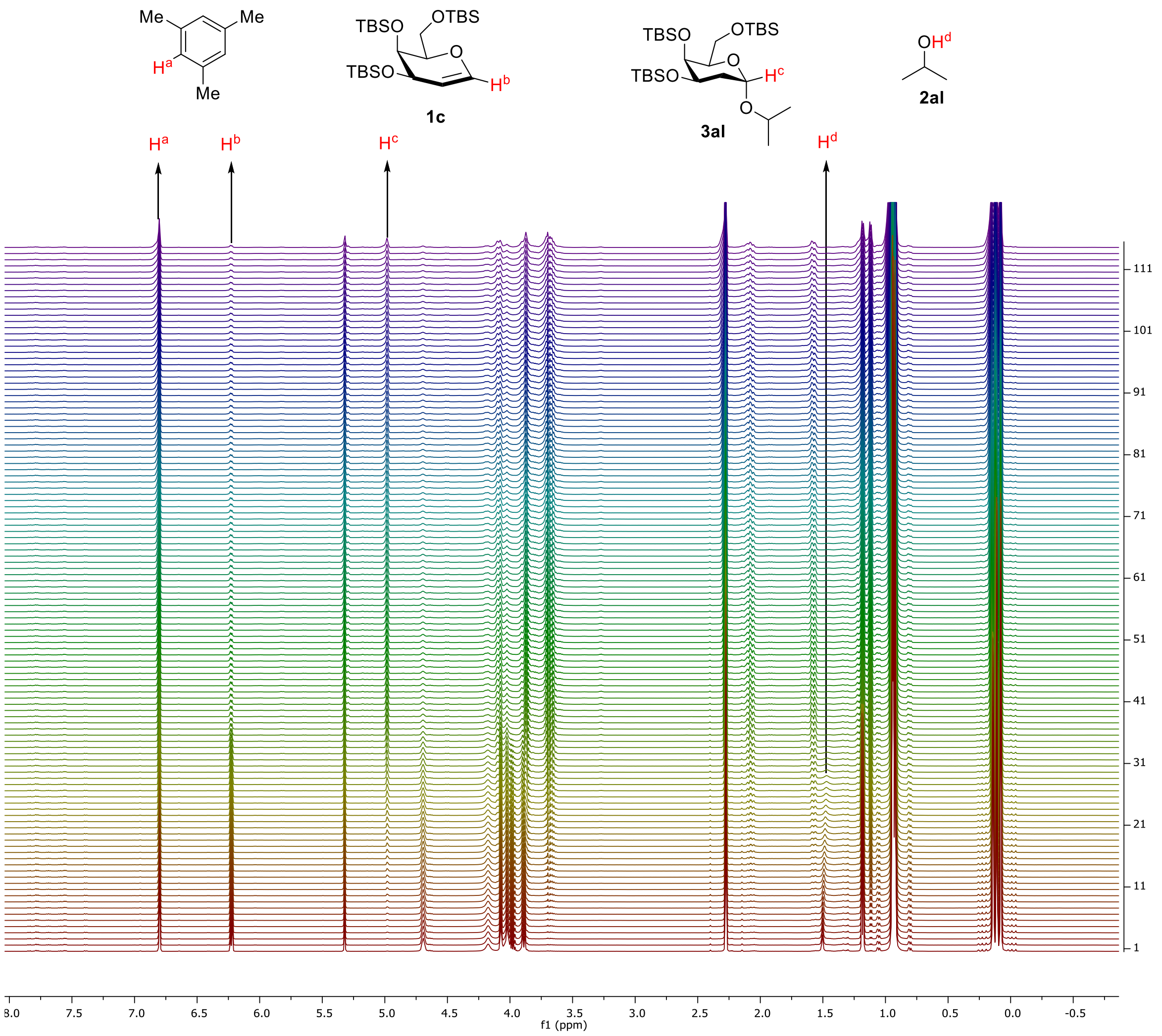

Supplementary figure S14: Stacked ^{1}H NMR spectra for the sequential reaction monitoring

Supplementary table S6: Concentration for **1c**, **2al**, **3al** calculated by ^{1}H NMR analysis for the sequential reaction monitoring

Time /min	[1c] /M	[2al] /M	[3al] /M	Time /min	[1c] /M	[2al] /M	[3al] /M	Time /min	[1c] /M	[2al] /M	[3al] /M
**9.5**	0.313	0.216	0.003	**285.0**	0.194	0.105	0.096	**560.3**	0.049	0.014	0.189
**19.5**	0.298	0.215	0.004	**294.8**	0.185	0.099	0.103	**570.1**	0.049	0.016	0.189
**29.3**	0.296	0.211	0.006	**304.6**	0.175	0.089	0.112	**580.0**	0.049	0.018	0.190
**39.1**	0.295	0.209	0.007	**314.5**	0.163	0.078	0.122	**589.8**	0.048	0.017	0.188
**49.0**	0.293	0.207	0.009	**324.3**	0.150	0.067	0.134	**599.6**	0.048	0.016	0.188
**58.8**	0.291	0.205	0.011	**334.1**	0.132	0.055	0.147	**609.5**	0.049	0.018	0.189
**68.6**	0.289	0.202	0.013	**344.0**	0.111	0.038	0.164	**619.3**	0.048	0.019	0.189
**78.5**	0.286	0.199	0.015	**353.8**	0.089	0.022	0.179	**629.1**	0.048	0.017	0.190
**88.3**	0.284	0.195	0.017	**363.6**	0.074	0.017	0.185	**639.0**	0.048	0.018	0.189
**98.1**	0.281	0.192	0.019	**373.5**	0.067	0.015	0.188	**648.8**	0.047	0.016	0.188
**108.0**	0.277	0.187	0.022	**383.3**	0.063	0.015	0.188	**658.6**	0.046	0.020	0.187
**117.8**	0.276	0.186	0.024	**393.1**	0.060	0.017	0.189	**668.5**	0.046	0.018	0.187
**127.6**	0.272	0.182	0.027	**403.0**	0.059	0.016	0.189	**678.3**	0.046	0.018	0.188
**137.5**	0.269	0.178	0.029	**412.8**	0.057	0.017	0.189	**688.1**	0.046	0.017	0.187
**147.3**	0.266	0.174	0.032	**422.6**	0.056	0.017	0.189	**698.0**	0.047	0.016	0.188
**157.1**	0.263	0.171	0.035	**432.5**	0.055	0.016	0.189	**707.8**	0.045	0.018	0.187
**167.0**	0.259	0.168	0.039	**442.3**	0.054	0.014	0.189	**717.6**	0.045	0.017	0.186
**176.8**	0.255	0.163	0.042	**452.1**	0.054	0.016	0.189	**727.5**	0.045	0.021	0.186
**186.6**	0.251	0.160	0.045	**462.0**	0.053	0.017	0.189	**737.3**	0.045	0.016	0.185
**196.5**	0.247	0.155	0.049	**471.8**	0.052	0.017	0.189	**747.1**	0.045	0.018	0.187
**206.3**	0.242	0.150	0.053	**481.6**	0.052	0.016	0.189	**757.0**	0.044	0.019	0.185
**216.1**	0.238	0.145	0.057	**491.5**	0.052	0.016	0.189	**766.8**	0.044	0.020	0.184
**226.0**	0.233	0.140	0.062	**501.3**	0.051	0.019	0.189	**776.6**	0.043	0.020	0.184
**235.8**	0.228	0.135	0.066	**511.1**	0.051	0.016	0.189	**786.5**	0.043	0.018	0.184
**245.6**	0.222	0.129	0.071	**521.0**	0.050	0.016	0.189	**796.3**	0.043	0.021	0.183
**255.5**	0.216	0.126	0.077	**530.8**	0.050	0.016	0.189	**806.1**	0.043	0.018	0.183
**265.3**	0.209	0.120	0.083	**540.6**	0.050	0.016	0.189				
**275.1**	0.202	0.113	0.089	**550.5**	0.049	0.013	0.188				

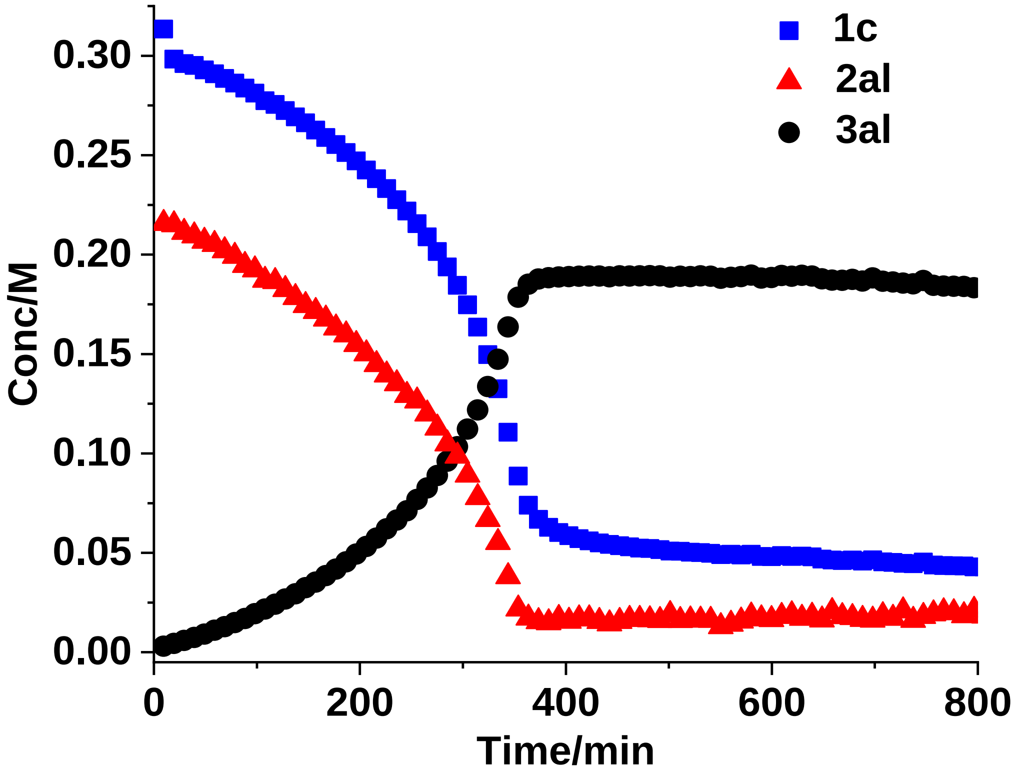

Supplementary figure S15: Temporal kinetics profile for the sequential reaction

4. *In situ* NMR monitoring for the reaction initial addition of 20 mol% of product 3al to the standard XB catalytic conditions (Related to the Figure 8a in the manuscript)

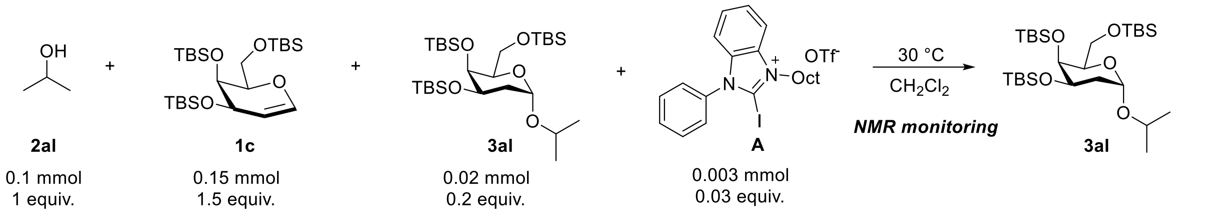

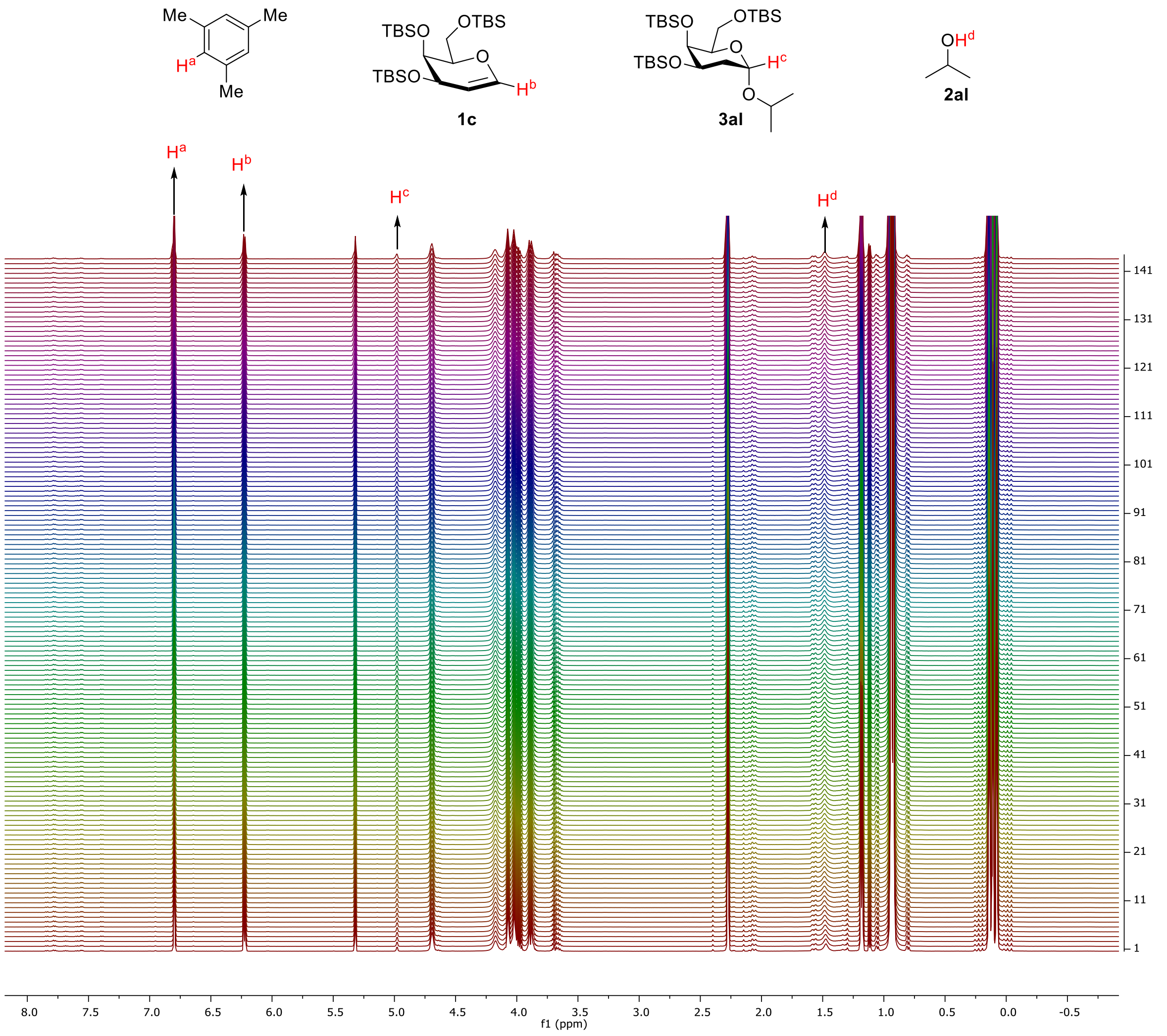

Supplementary figure S16: Stacked ^{1}H NMR spectra for the reaction with initial addition of 20 mol% **3al** to the standard XB catalytic conditions

Supplementary table S7: Concentration for **1c**, **2al**, **3al** calculated by NMR analysis for the reaction with initial addition of 20 mol% **3al**

Time /min	[1c] /M	[2al] /M	[3al] /M	Time /min	[1c] /M	[2al] /M	[3al] /M	Time /min	[1c] /M	[2al] /M	[3al] /M
**9.5**	0.309	0.207	0.035	**481.6**	0.294	0.211	0.038	**953.6**	0.287	0.204	0.045
**19.5**	0.299	0.204	0.034	**491.5**	0.294	0.208	0.038	**963.5**	0.287	0.202	0.045
**29.3**	0.299	0.203	0.034	**501.3**	0.294	0.204	0.038	**973.3**	0.287	0.201	0.045
**39.1**	0.299	0.206	0.034	**511.1**	0.294	0.206	0.039	**983.1**	0.287	0.203	0.045
**49.0**	0.299	0.206	0.033	**521.0**	0.294	0.207	0.038	**993.0**	0.286	0.202	0.045
**58.8**	0.299	0.208	0.034	**530.8**	0.293	0.207	0.039	**1002.8**	0.286	0.202	0.045
**68.6**	0.299	0.209	0.034	**540.6**	0.293	0.205	0.039	**1012.6**	0.286	0.202	0.046
**78.5**	0.299	0.211	0.034	**550.5**	0.293	0.213	0.038	**1022.5**	0.286	0.198	0.044
**88.3**	0.298	0.211	0.033	**560.3**	0.293	0.207	0.039	**1032.3**	0.286	0.201	0.046
**98.1**	0.298	0.212	0.034	**570.1**	0.293	0.206	0.039	**1042.1**	0.286	0.205	0.046
**108.0**	0.298	0.211	0.034	**580.0**	0.293	0.207	0.039	**1052.0**	0.285	0.202	0.046
**117.8**	0.298	0.211	0.035	**589.8**	0.292	0.205	0.040	**1061.8**	0.285	0.202	0.046
**127.6**	0.298	0.212	0.035	**599.6**	0.292	0.205	0.040	**1071.6**	0.286	0.200	0.046
**137.5**	0.298	0.211	0.035	**609.5**	0.292	0.204	0.040	**1081.5**	0.285	0.201	0.046
**147.3**	0.298	0.211	0.035	**619.3**	0.292	0.207	0.040	**1091.3**	0.285	0.205	0.047
**157.1**	0.298	0.212	0.035	**629.1**	0.292	0.203	0.040	**1101.1**	0.284	0.201	0.047
**167.0**	0.298	0.213	0.035	**639.0**	0.292	0.205	0.040	**1111.0**	0.284	0.200	0.047
**176.8**	0.298	0.207	0.034	**648.8**	0.292	0.205	0.040	**1120.8**	0.284	0.200	0.048
**186.6**	0.298	0.209	0.034	**658.6**	0.291	0.206	0.040	**1130.6**	0.284	0.201	0.048
**196.5**	0.298	0.212	0.035	**668.5**	0.291	0.205	0.040	**1140.5**	0.284	0.199	0.048
**206.3**	0.297	0.210	0.036	**678.3**	0.291	0.205	0.041	**1150.3**	0.284	0.196	0.048
**216.1**	0.297	0.211	0.036	**688.1**	0.291	0.205	0.041	**1160.1**	0.283	0.200	0.048
**226.0**	0.298	0.209	0.036	**698.0**	0.291	0.206	0.041	**1170.0**	0.283	0.198	0.048
**235.8**	0.297	0.210	0.036	**707.8**	0.290	0.206	0.041	**1179.8**	0.283	0.201	0.048
**245.6**	0.297	0.211	0.036	**717.6**	0.290	0.206	0.041	**1189.6**	0.283	0.198	0.049
**255.5**	0.297	0.209	0.036	**727.5**	0.291	0.206	0.041	**1199.5**	0.282	0.201	0.049
**265.3**	0.296	0.210	0.036	**737.3**	0.290	0.205	0.041	**1209.3**	0.282	0.198	0.049
**275.1**	0.297	0.210	0.036	**747.1**	0.290	0.206	0.042	**1219.1**	0.282	0.199	0.049
**285.0**	0.296	0.207	0.036	**757.0**	0.290	0.205	0.042	**1229.0**	0.282	0.199	0.050
**294.8**	0.296	0.209	0.036	**766.8**	0.290	0.205	0.042	**1238.8**	0.282	0.201	0.050
**304.6**	0.296	0.208	0.036	**776.6**	0.290	0.206	0.042	**1248.6**	0.282	0.198	0.050
**314.5**	0.296	0.209	0.037	**786.5**	0.290	0.205	0.042	**1258.5**	0.281	0.196	0.050
**324.3**	0.296	0.211	0.037	**796.3**	0.290	0.204	0.042	**1268.3**	0.281	0.198	0.051
**334.1**	0.296	0.209	0.037	**806.1**	0.289	0.206	0.042	**1278.1**	0.281	0.198	0.051
**344.0**	0.296	0.210	0.037	**816.0**	0.289	0.208	0.042	**1287.9**	0.280	0.198	0.051
**353.8**	0.295	0.207	0.037	**825.8**	0.289	0.208	0.042	**1297.8**	0.280	0.199	0.051
**363.6**	0.295	0.208	0.037	**835.6**	0.289	0.204	0.042	**1307.6**	0.280	0.200	0.051
**373.5**	0.295	0.209	0.037	**845.5**	0.289	0.200	0.042	**1317.4**	0.280	0.196	0.051
**383.3**	0.295	0.209	0.037	**855.3**	0.288	0.203	0.043	**1327.3**	0.280	0.193	0.052
**393.1**	0.295	0.208	0.038	**865.1**	0.288	0.204	0.043	**1337.1**	0.279	0.196	0.052
**403.0**	0.295	0.208	0.037	**875.0**	0.288	0.205	0.043	**1346.9**	0.279	0.191	0.051
**412.8**	0.294	0.208	0.037	**884.8**	0.288	0.208	0.043	**1356.8**	0.279	0.198	0.052
**422.6**	0.294	0.209	0.038	**894.6**	0.288	0.204	0.044	**1366.6**	0.279	0.195	0.052
**432.5**	0.294	0.209	0.037	**904.5**	0.288	0.204	0.044	**1376.4**	0.278	0.195	0.053
**442.3**	0.294	0.208	0.038	**914.3**	0.288	0.205	0.044	**1386.3**	0.278	0.191	0.052
**452.1**	0.295	0.209	0.038	**924.1**	0.288	0.204	0.044	**1396.1**	0.277	0.195	0.053
**462.0**	0.294	0.207	0.039	**934.0**	0.288	0.202	0.044	**1405.9**	0.277	0.193	0.053
**471.8**	0.294	0.206	0.038	**943.8**	0.287	0.202	0.044	**1415.8**	0.277	0.193	0.054

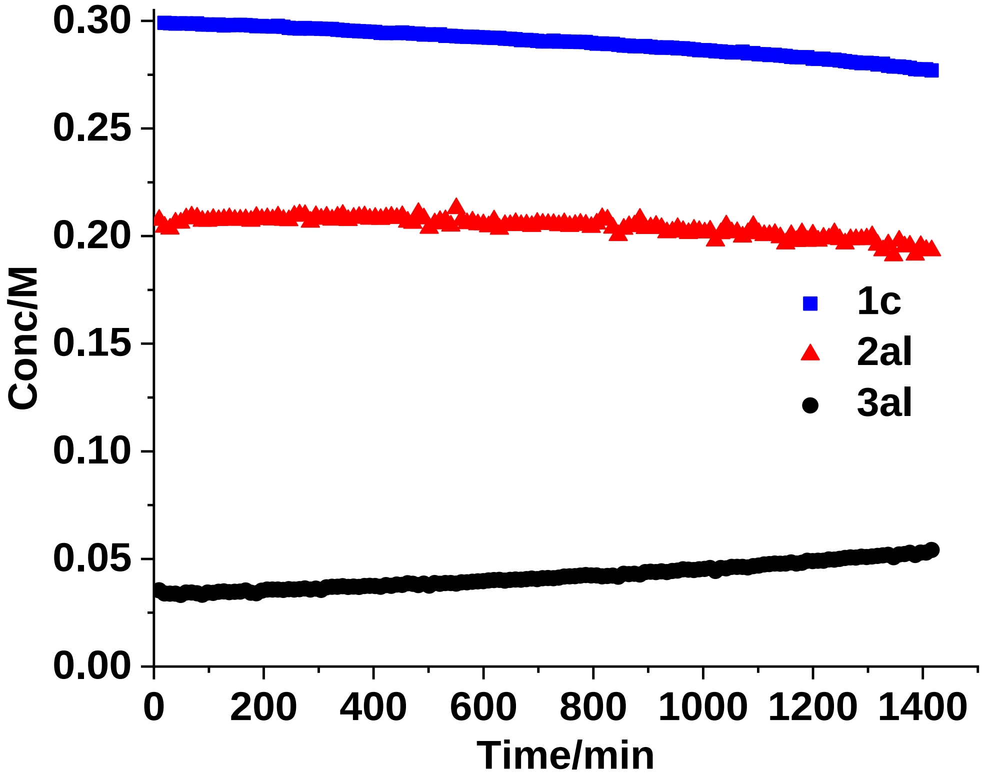

Supplementary figure S17: Temporal kinetics profile for the reaction with initial addition of 20 mol% **3al** to the standard XB catalytic conditions

5. *In situ* NMR monitoring for the temporal 3al formation for sequential reaction with following addition of 20 mol% 3al after *in situ* generated catalyst 12

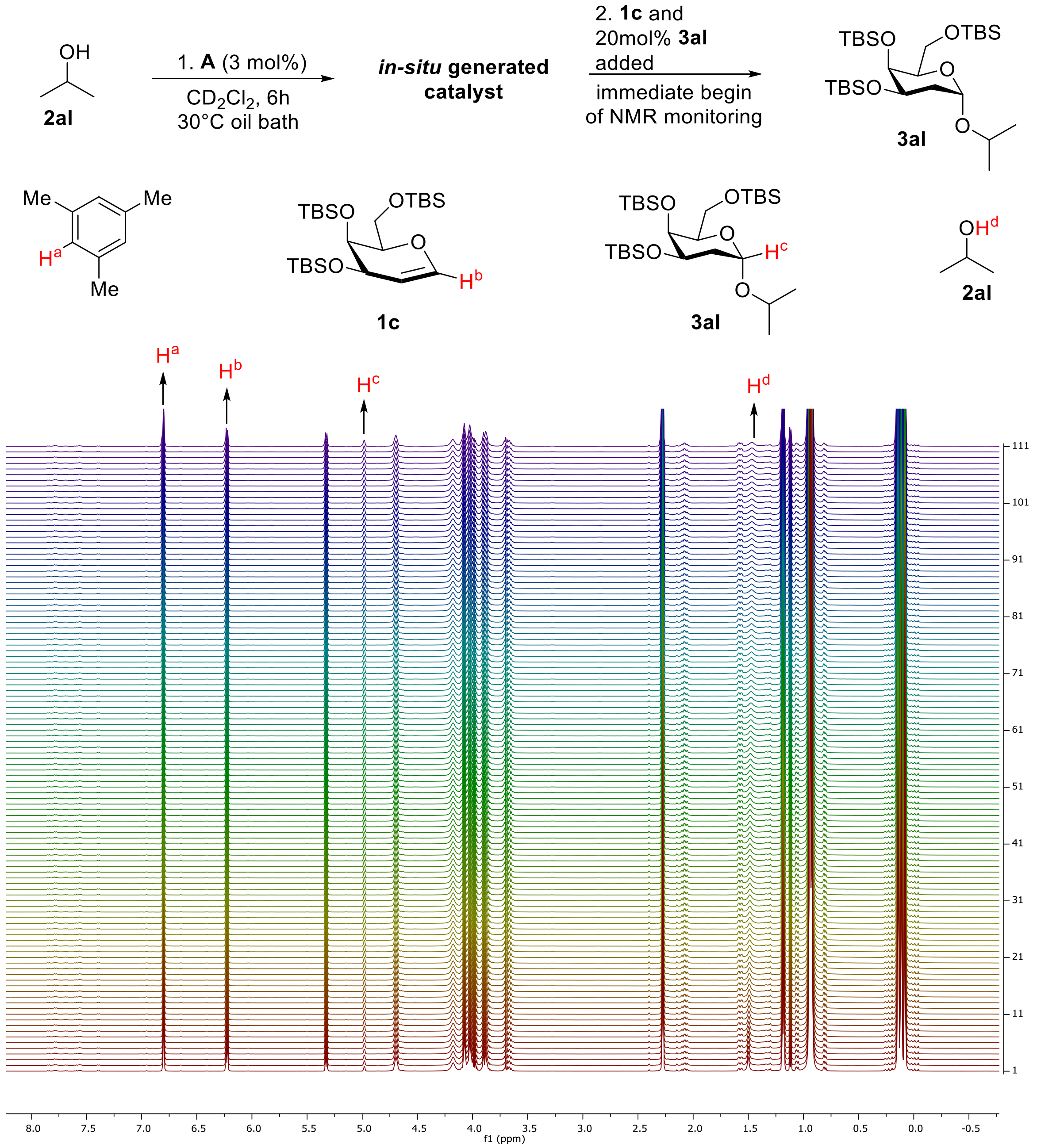

Supplementary figure S18: Stacked ^{1}H NMR spectra for the reaction sequential addition of 20 mol% **3al** after *in situ* generated catalyst **12**

Supplementary table S8: Concentration for **1c**, **3al** calculated by ^{1}H NMR analysis for the sequential reaction with following addition of 20 mol% **3al**

Time /min	[1c] /M	[3al] /M	Time /min	[1c] /M	[3al] /M	Time /min	[1c] /M	[3al] /M
**0**	0.300	0.040	**400**	0.287	0.06897	**780**	0.272	0.08017
**30**	0.299	0.05433	**410**	0.287	0.06857	**790**	0.271	0.07945
**40**	0.335	0.05709	**420**	0.287	0.06937	**800**	0.271	0.081
**50**	0.306	0.05054	**430**	0.286	0.06978	**810**	0.271	0.08082
**60**	0.304	0.05266	**440**	0.285	0.07044	**820**	0.270	0.08106
**70**	0.303	0.05381	**450**	0.285	0.07048	**830**	0.271	0.08097
**80**	0.302	0.05526	**460**	0.284	0.07068	**840**	0.270	0.08108
**90**	0.301	0.05587	**470**	0.284	0.07097	**850**	0.270	0.08146
**100**	0.300	0.05582	**480**	0.284	0.07126	**860**	0.270	0.08146
**110**	0.299	0.05788	**490**	0.283	0.07163	**870**	0.269	0.08192
**120**	0.298	0.05745	**500**	0.282	0.07229	**880**	0.269	0.08184
**130**	0.298	0.05907	**510**	0.283	0.07236	**890**	0.269	0.08202
**140**	0.297	0.05954	**520**	0.282	0.07243	**900**	0.268	0.0826
**150**	0.297	0.05994	**530**	0.281	0.07357	**910**	0.268	0.08267
**160**	0.296	0.06084	**540**	0.281	0.07346	**920**	0.268	0.08306
**170**	0.295	0.06114	**550**	0.280	0.07339	**930**	0.268	0.08276
**180**	0.295	0.06135	**560**	0.280	0.07405	**940**	0.268	0.08316
**190**	0.294	0.06194	**570**	0.280	0.07437	**950**	0.267	0.08307
**200**	0.294	0.06247	**580**	0.279	0.07532	**960**	0.267	0.08342
**210**	0.294	0.06276	**590**	0.279	0.07495	**970**	0.266	0.08398
**220**	0.293	0.06322	**600**	0.279	0.0758	**980**	0.266	0.08399
**230**	0.293	0.06334	**610**	0.278	0.07599	**990**	0.266	0.08466
**240**	0.293	0.06401	**620**	0.277	0.07602	**1000**	0.266	0.08458
**250**	0.292	0.06363	**630**	0.277	0.07682	**1010**	0.266	0.08459
**260**	0.292	0.06338	**640**	0.277	0.07715	**1020**	0.265	0.08458
**270**	0.292	0.06465	**650**	0.276	0.07731	**1030**	0.265	0.08483
**280**	0.292	0.06454	**660**	0.276	0.0775	**1040**	0.265	0.08507
**290**	0.291	0.06535	**670**	0.276	0.0773	**1050**	0.261	0.08512
**300**	0.290	0.06543	**680**	0.275	0.07764	**1060**	0.264	0.08535
**310**	0.291	0.06565	**690**	0.275	0.07845	**1070**	0.264	0.08548
**320**	0.290	0.06625	**700**	0.274	0.07858	**1080**	0.263	0.08583
**330**	0.289	0.06664	**710**	0.274	0.07859	**1090**	0.263	0.08582
**340**	0.289	0.06683	**720**	0.274	0.07858	**1100**	0.263	0.08635
**350**	0.289	0.06685	**730**	0.274	0.07822	**1110**	0.263	0.08643
**360**	0.289	0.06716	**740**	0.273	0.07939	**1120**	0.262	0.0868
**370**	0.288	0.06814	**750**	0.273	0.07928	**1130**	0.262	0.08677
**380**	0.288	0.06791	**760**	0.273	0.07965	**1140**	0.262	0.08683
**390**	0.287	0.06853	**770**	0.272	0.08021			

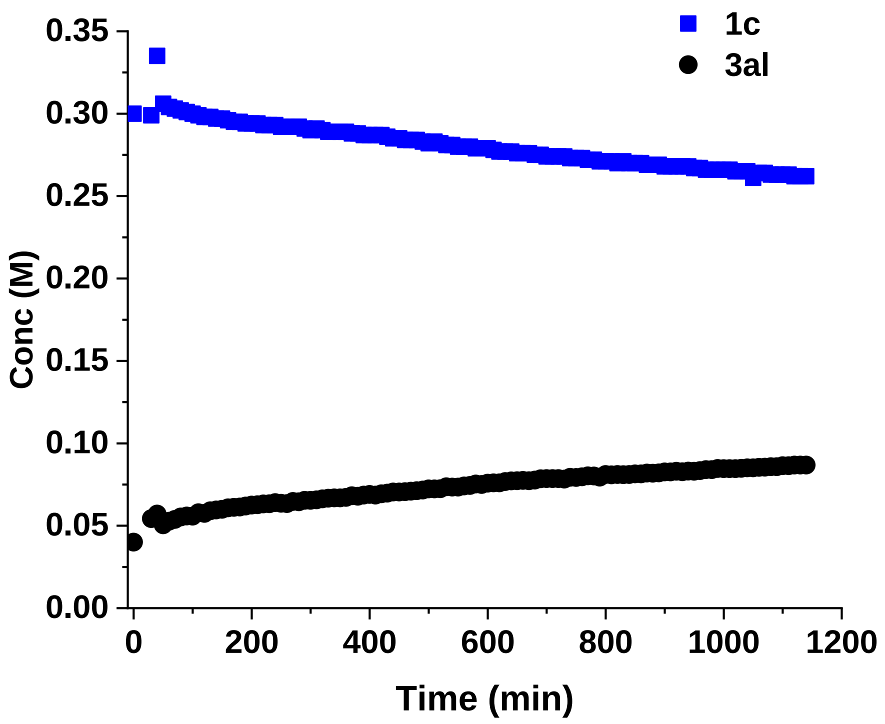

Supplementary figure S19: Temporal kinetics profile for the temporal **3al** formation for sequential reaction with following addition of 20 mol% **3al** after *in situ* generated catalyst **12**

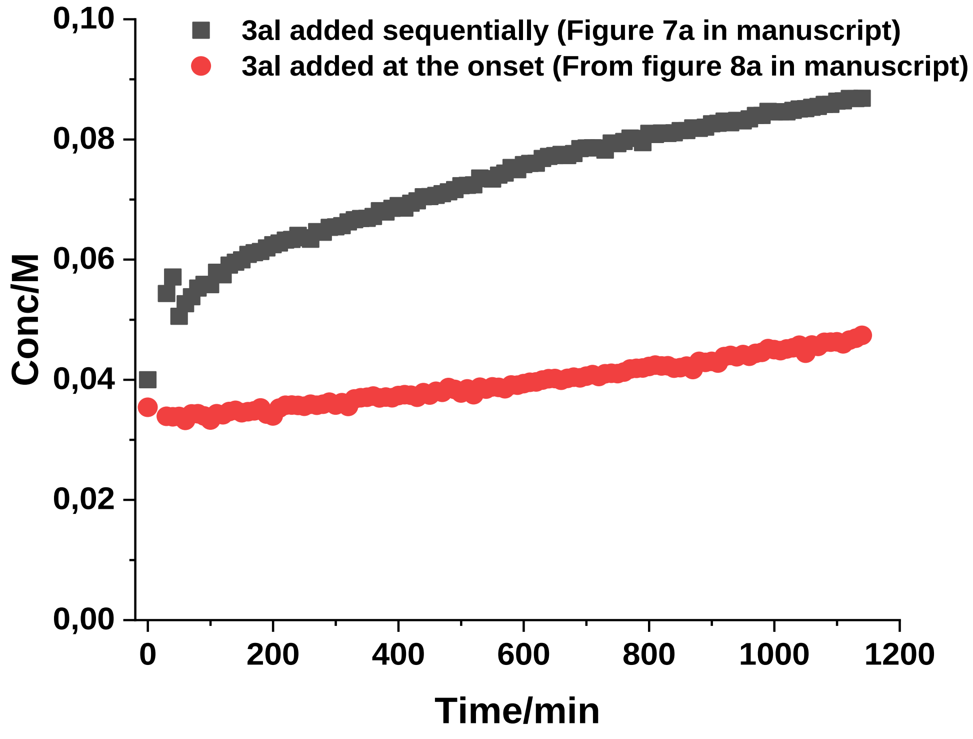

Supplementary figure S20: Overlap of temporal **3al** formation for sequential addition of 20 mol% **3al** and conditions in Supplementary figure S16.

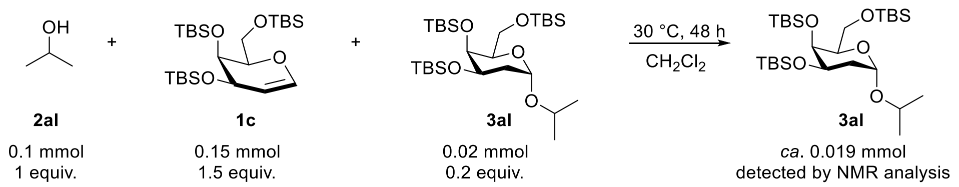

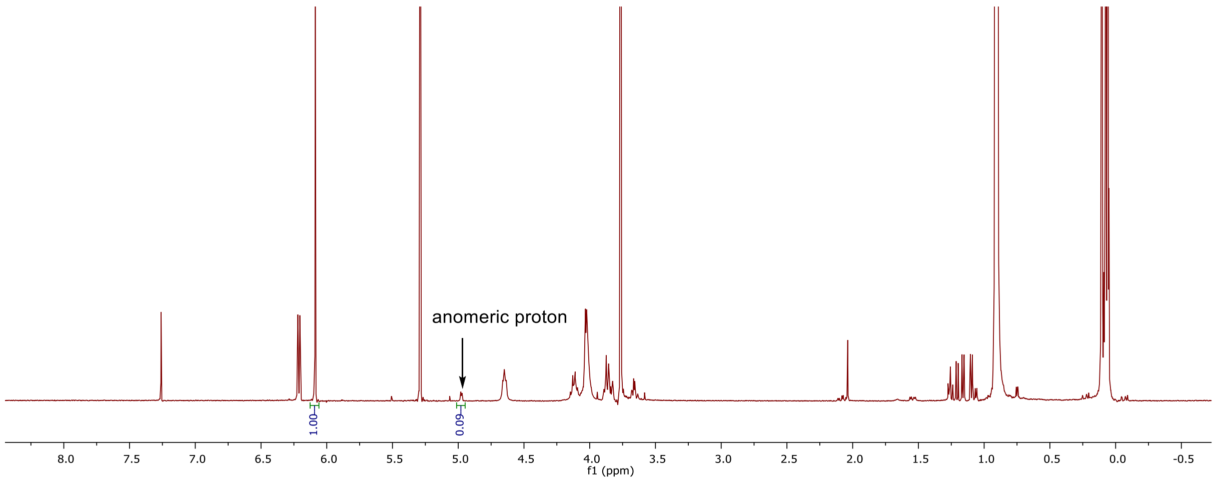

Supplementary figure S21: Crude ^{1}H NMR spectrum for the control reaction with initial addition of 20 mol% of **3al** to exclude pure autocatalysis (Related to Figure 7d in the manuscript)

***In situ* NMR monitoring for the reaction of benzylated galactal 1d and isopropanol 2al using halogen bond A as catalyst**

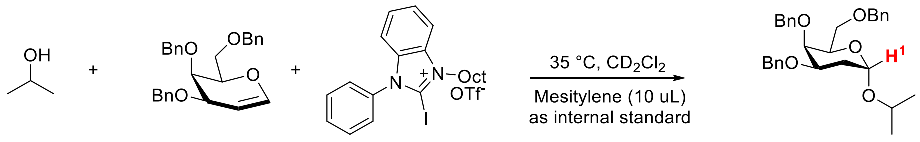

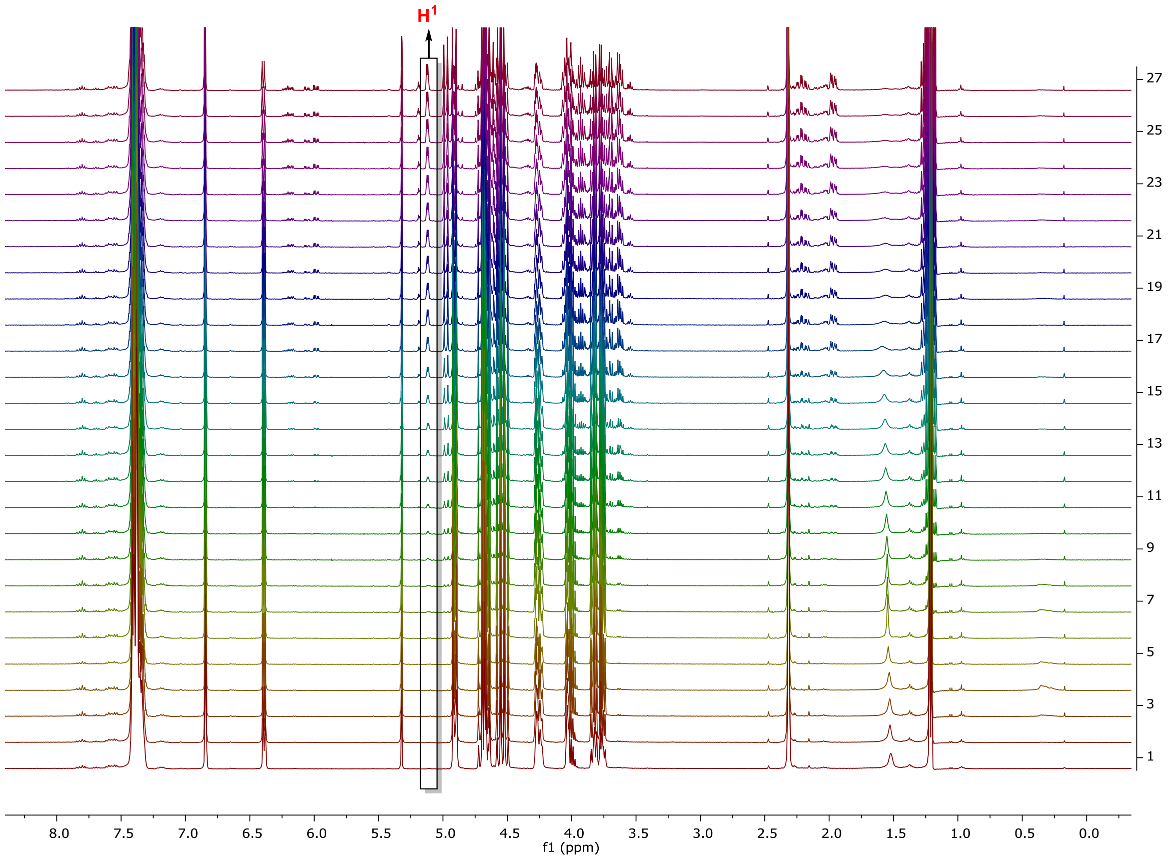

Supplementary figure S22: Stacked ^1H NMR spectra for the reaction of benzylated galactal and isopropanol

Supplementary table S9: Concentration for the product calculated by ^1H NMR analysis for the reaction of benzylated galactal **1d** and isopropanol **2al**.

Entry	Time/min	[3-Bn-IPA] /M	Entry	Time/min	[3-Bn-IPA] /M	Entry	Time/min	[Product] /M
1	10	0	10	120	0.01699	19	280	0.0937
2	20	0	11	135	0.02294	20	300	0.09616
3	30	0	12	150	0.02893	21	330	0.10435
4	40	0	13	165	0.0354	22	360	0.1166
5	50	0	14	180	0.04299	23	390	0.12656
6	60	0	15	200	0.05653	24	420	0.13497
7	75	0.00233	16	220	0.0752	25	450	0.14462
8	90	0.00772	17	240	0.08275	26	480	0.14902
9	105	0.01194	18	260	0.08844	27	510	0.1495

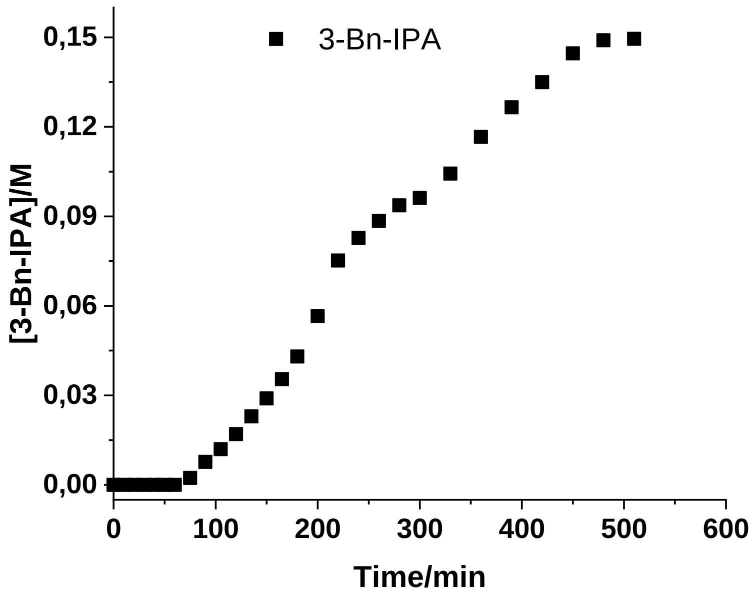

Supplementary figure S23: Temporal kinetic of 3-Bn-IPA for 2-deoxygalactosylation of benzylated galactal

Interaction between halogen bond catalyst A and TBAB

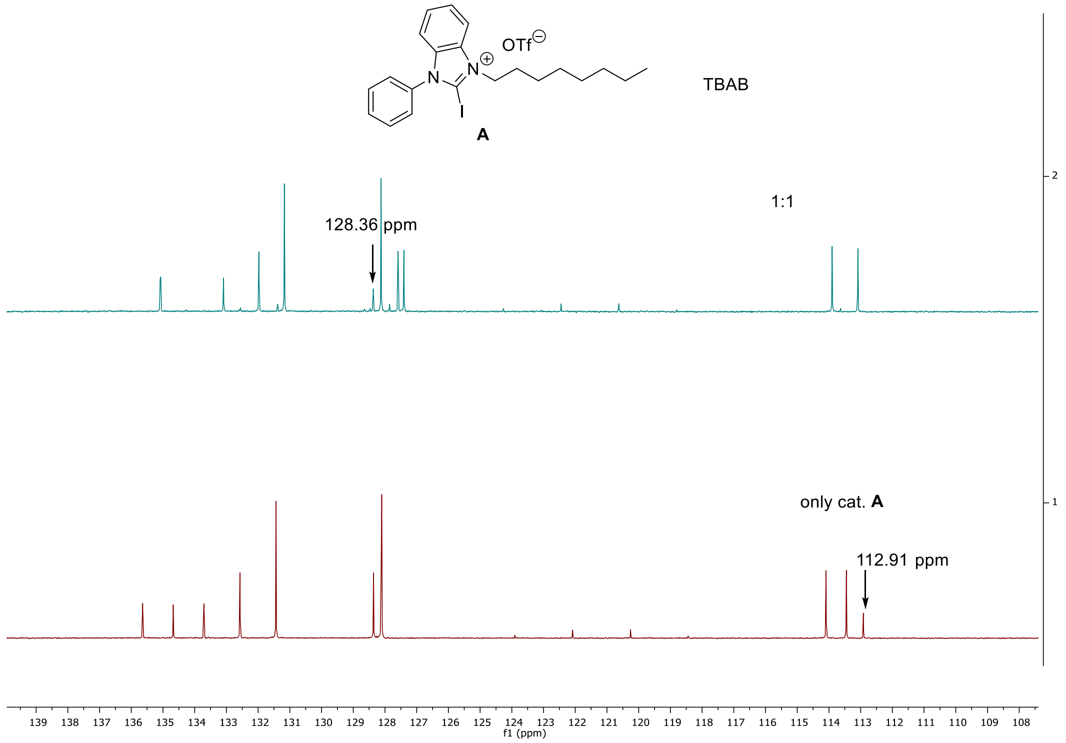

Supplementary figure S24: The chemical shift of ^{13}C of *C*-I shifts downfield from 112.91 ppm (solely halogen bond **A**) to 128.36 ppm (molar ratio 1:1).

NMR spectra for the solution of halogen bond catalyst A and TBAB (molar ratio 1:1)

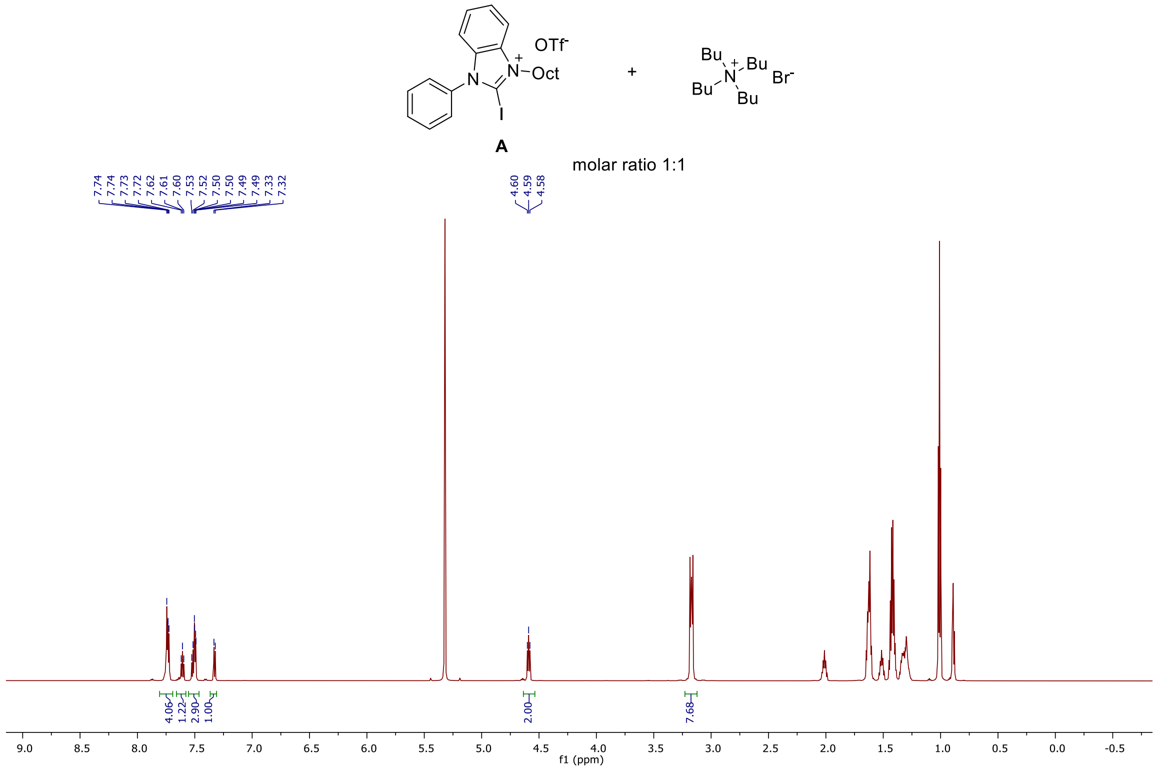

Supplementary figure S25: ^{1}H NMR spectrum for the mixture of halogen bond catalyst **A** and TBAB (molar ratio 1:1)

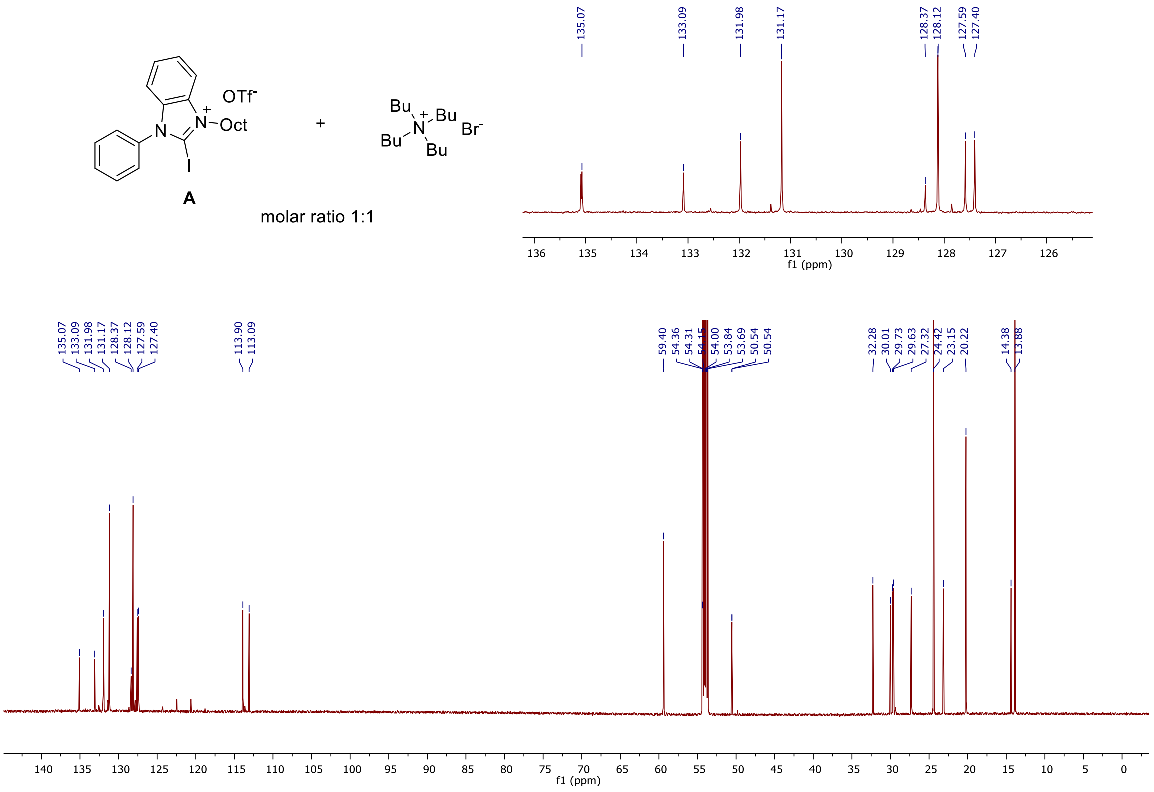

Supplementary figure S26: ^{13}C NMR spectrum for the mixture of halogen bond catalyst **A** and TBAB (molar ratio 1:1)

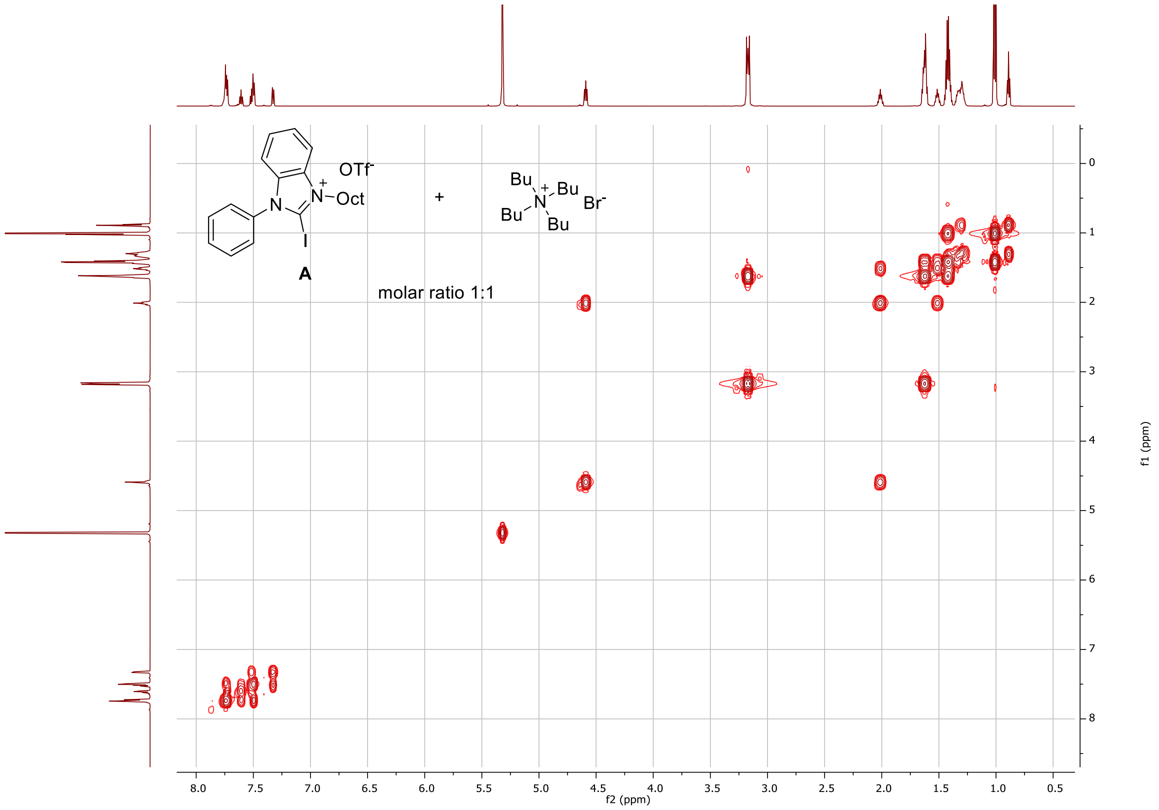

Supplementary figure S27: COSY spectrum for the mixture of halogen bond catalyst **A** and TBAB (molar ratio 1:1)

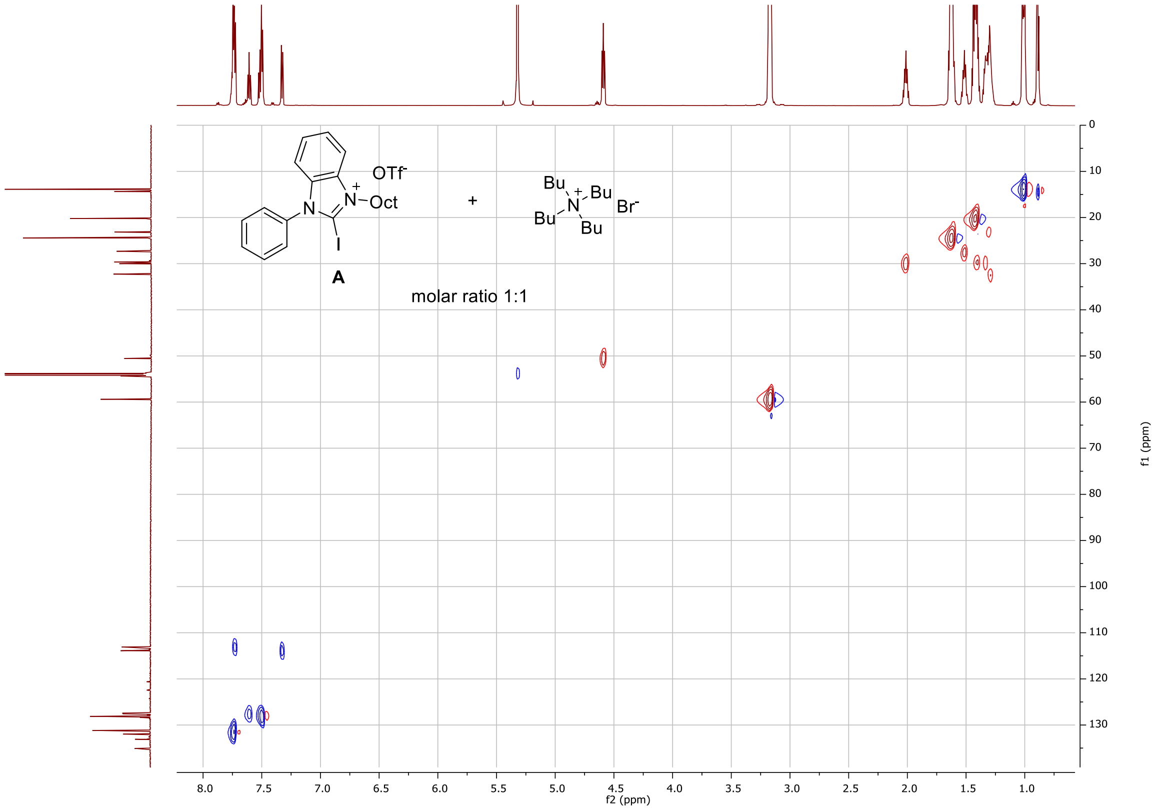

Supplementary figure S28: HSQC spectrum for the mixture of halogen bond catalyst **A** and TBAB (molar ratio 1:1)

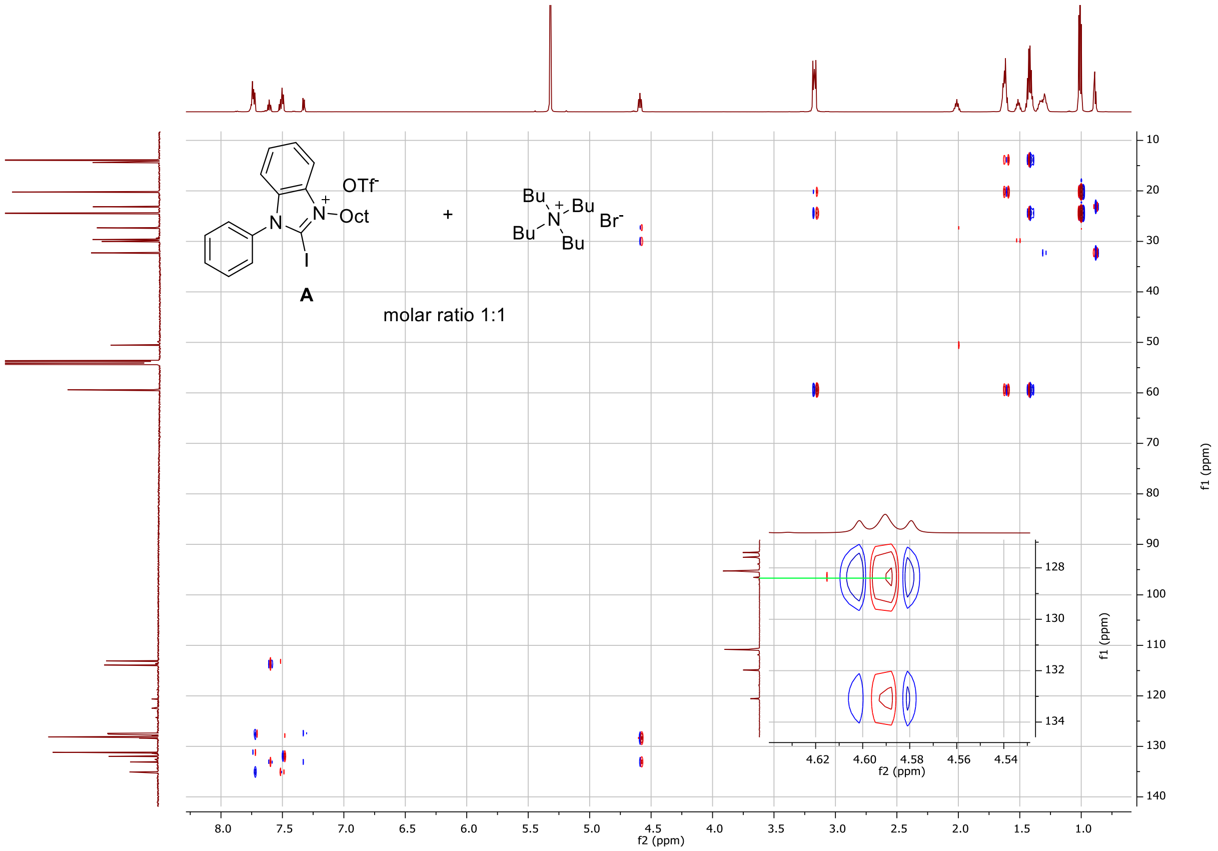

Supplementary figure S29: HMBC spectrum for the mixture of halogen bond catalyst **A** and TBAB (molar ratio 1:1)

Interaction between halogen bond catalyst A and TBAI

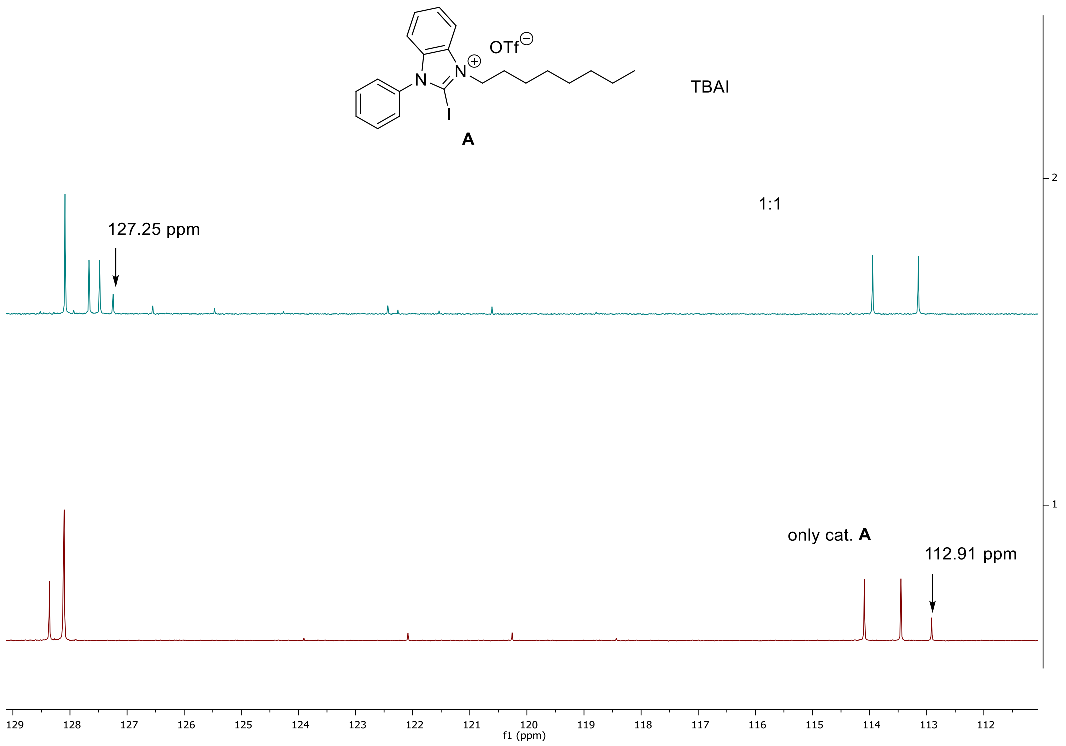

Supplementary figure S30: The chemical shift of ^{13}C of *C*-I shifts downfield from 112.91 ppm (solely halogen bond **A**) to 127.25 ppm (molar ratio 1:1).

NMR spectra for the solution of halogen bond catalyst A and TBAI (molar ratio 1:1)

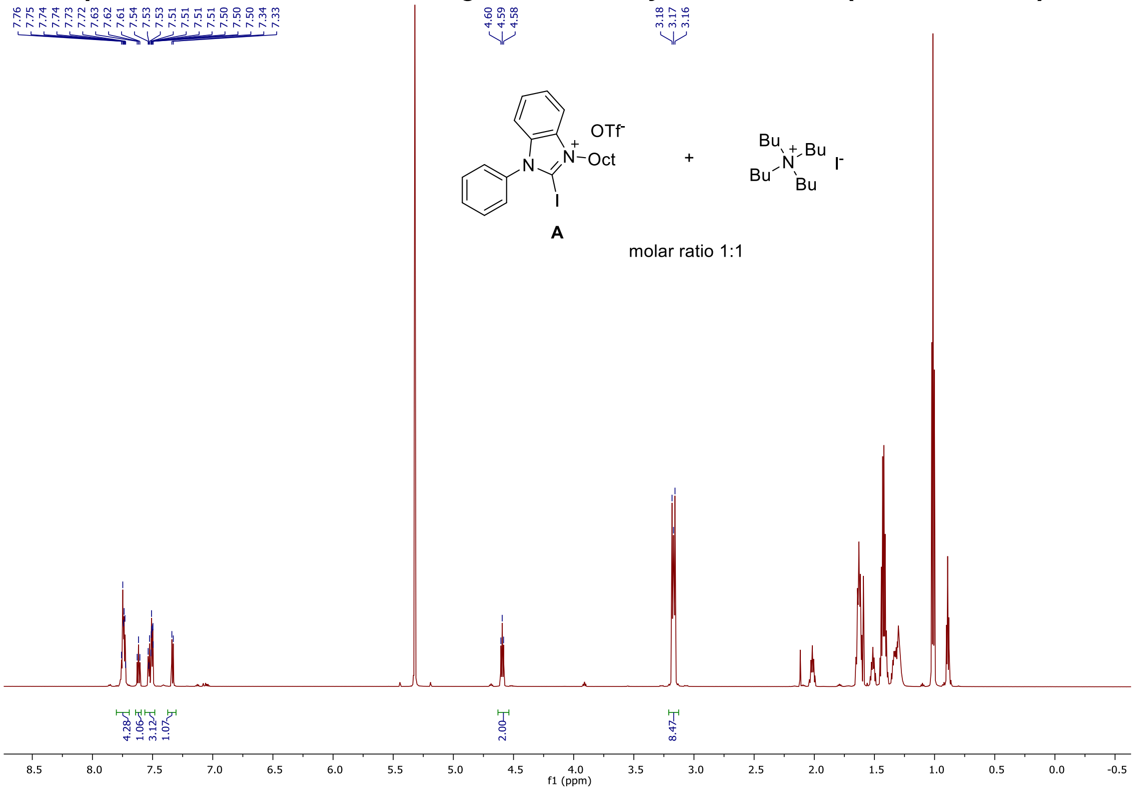

Supplementary figure S31: ^{1}H spectrum for the mixture of halogen bond catalyst **A** and TBAI (molar ratio 1:1)

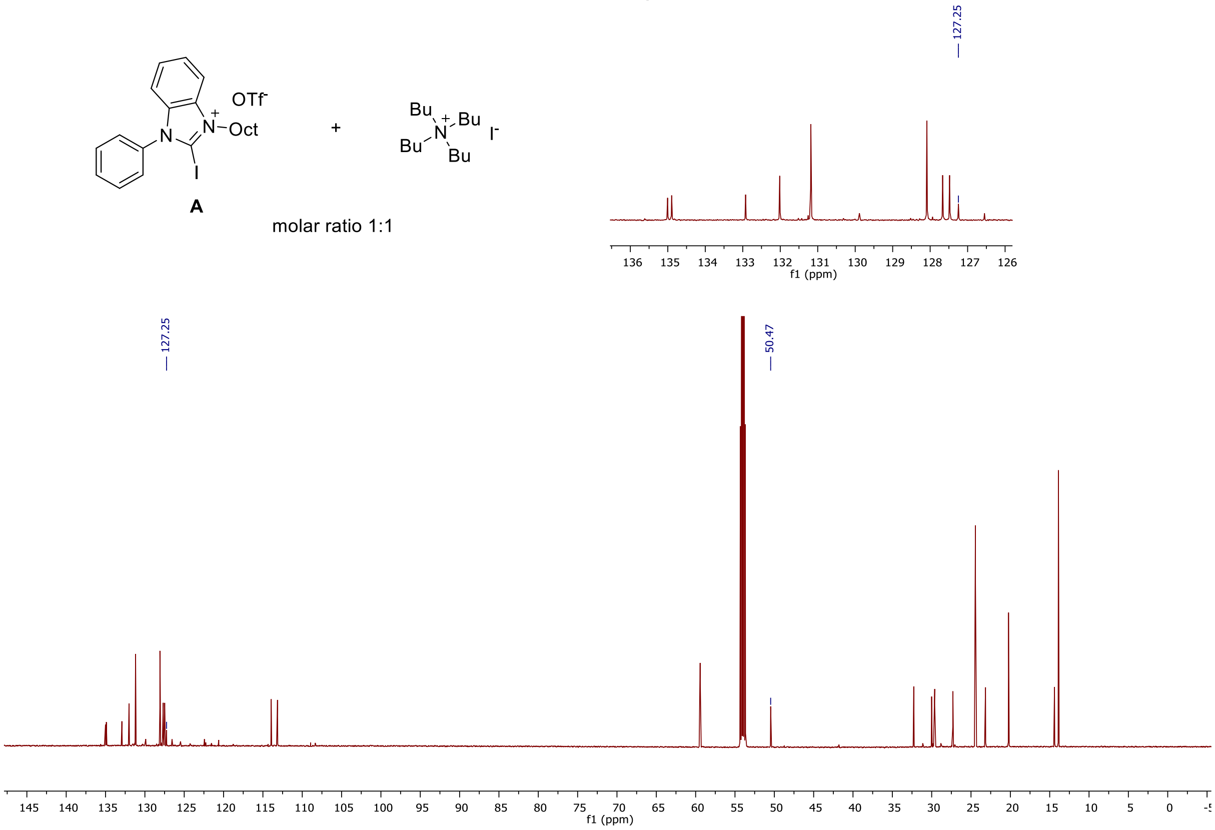

Supplementary figure S32: ^{13}C spectrum for the mixture of halogen bond catalyst **A** and TBAI (molar ratio 1:1)

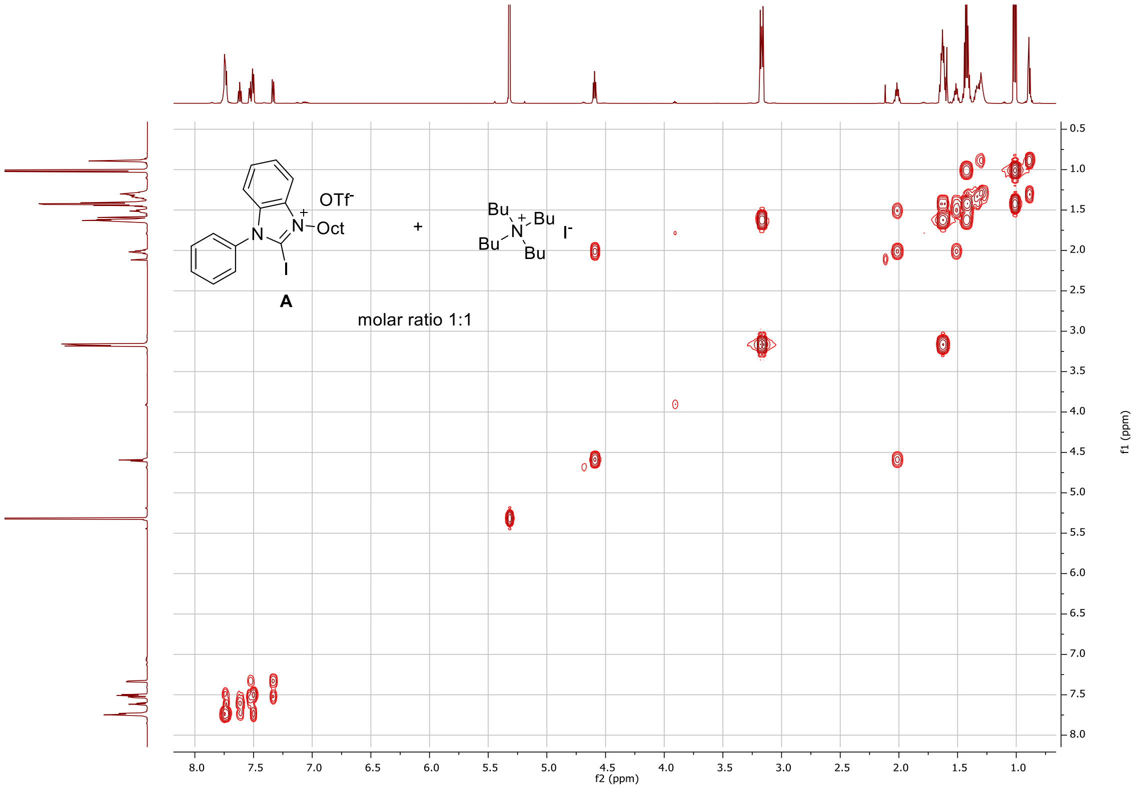

Supplementary figure S33: COSY spectrum for the mixture of halogen bond catalyst **A** and TBAI (molar ratio 1:1)

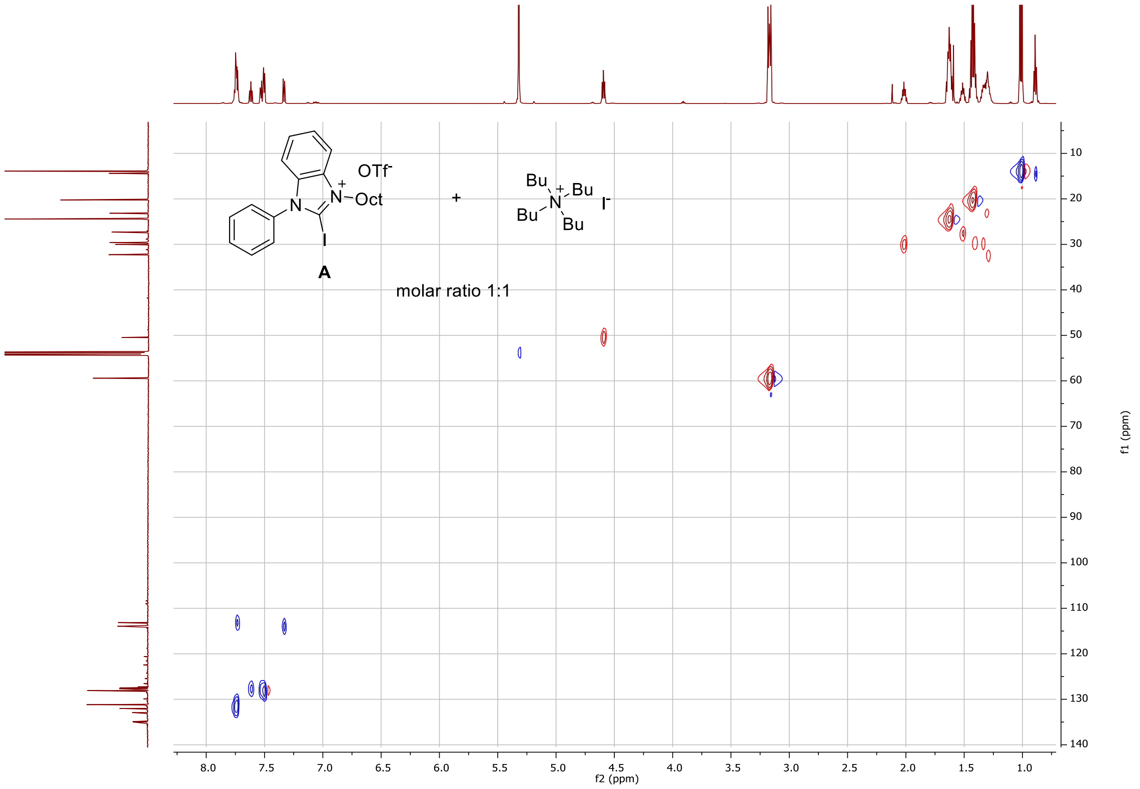

Supplementary figure S34: HSQC spectrum for the mixture of halogen bond catalyst **A** and TBAI (molar ratio 1:1)

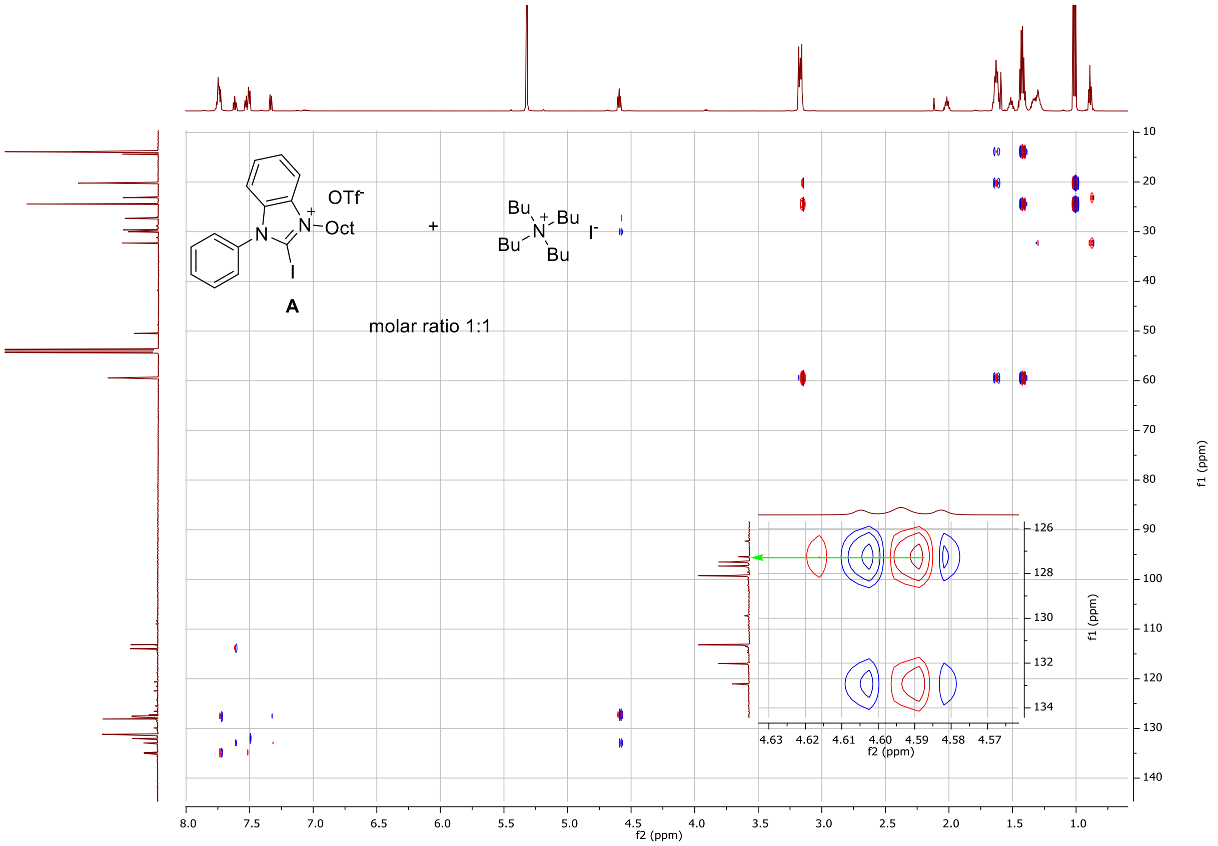

Supplementary figure S35: HMBC spectrum for the mixture of halogen bond catalyst **A** and TBAI (molar ratio 1:1)

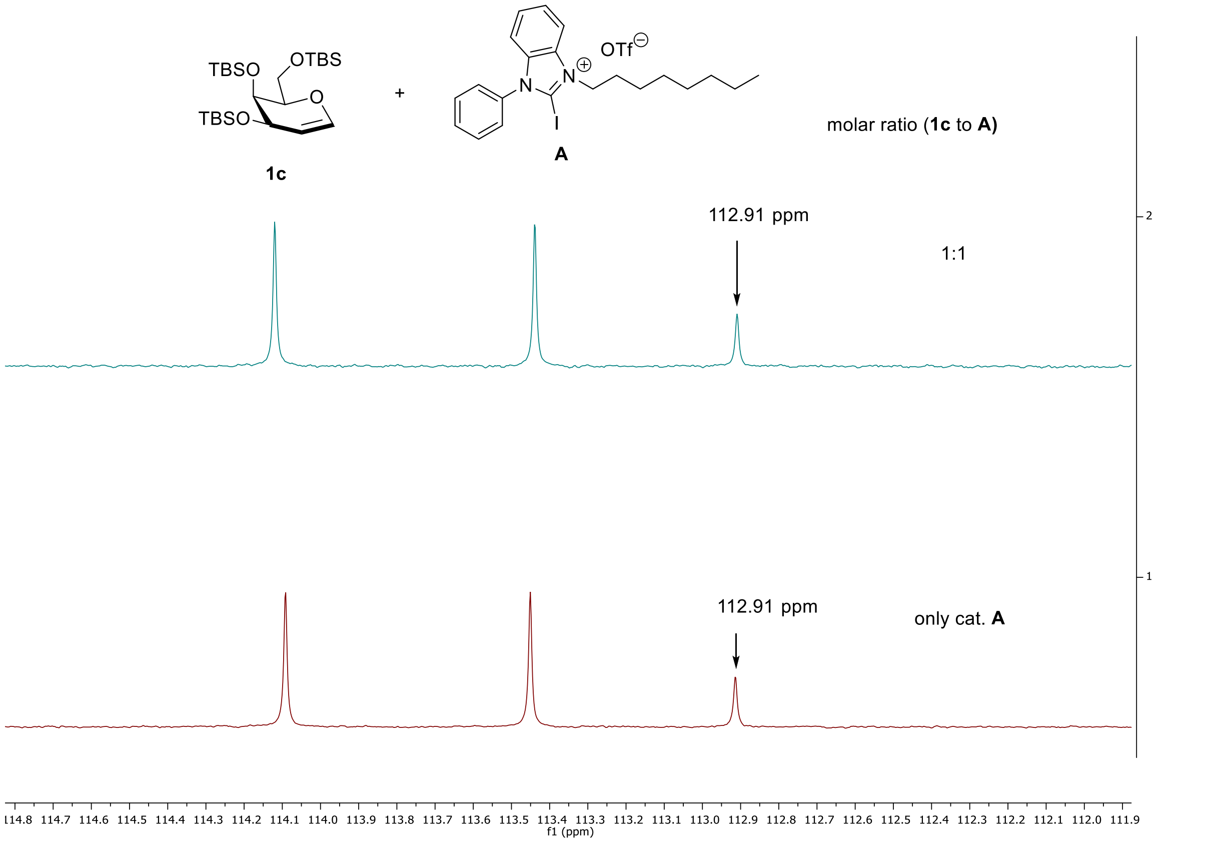

Supplementary figure S36: Stacked ^{13}C spectra for the ^{13}C NMR shift experiment of halogen bond catalyst **A** and **1c**

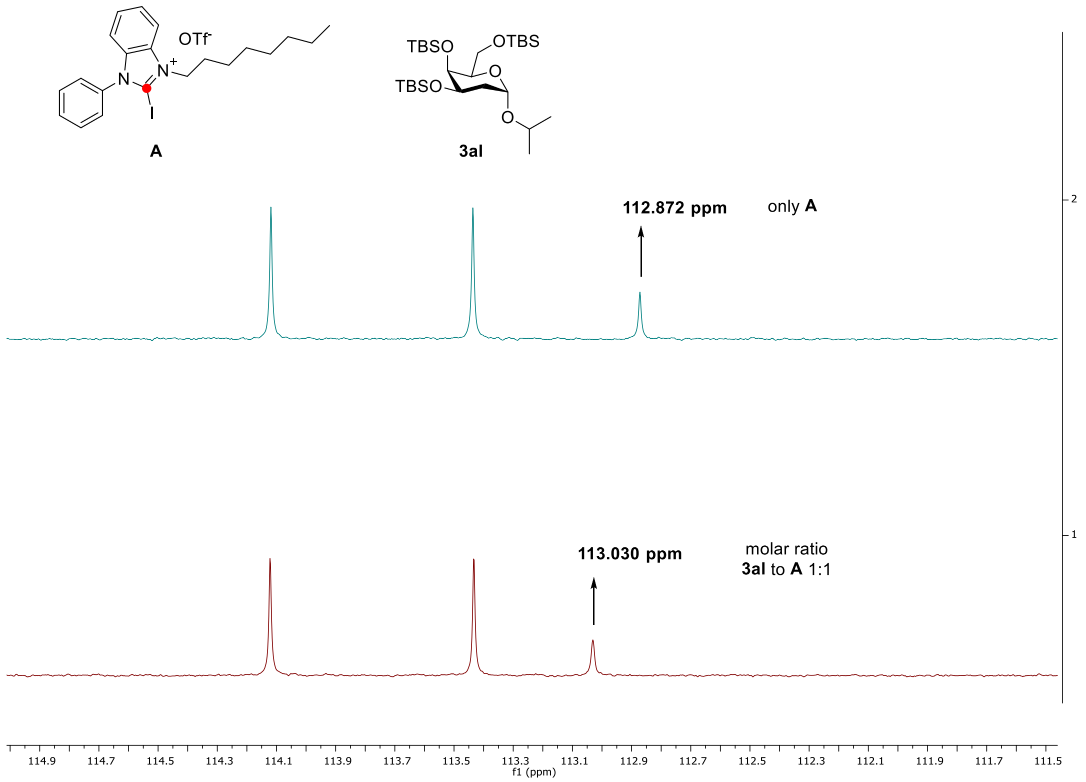

Supplementary figure S37: Stacked ^{13}C spectra for the ^{13}C NMR shift experiment of halogen bond catalyst **A** and **3al**

Sequential reaction terminated by TBAI

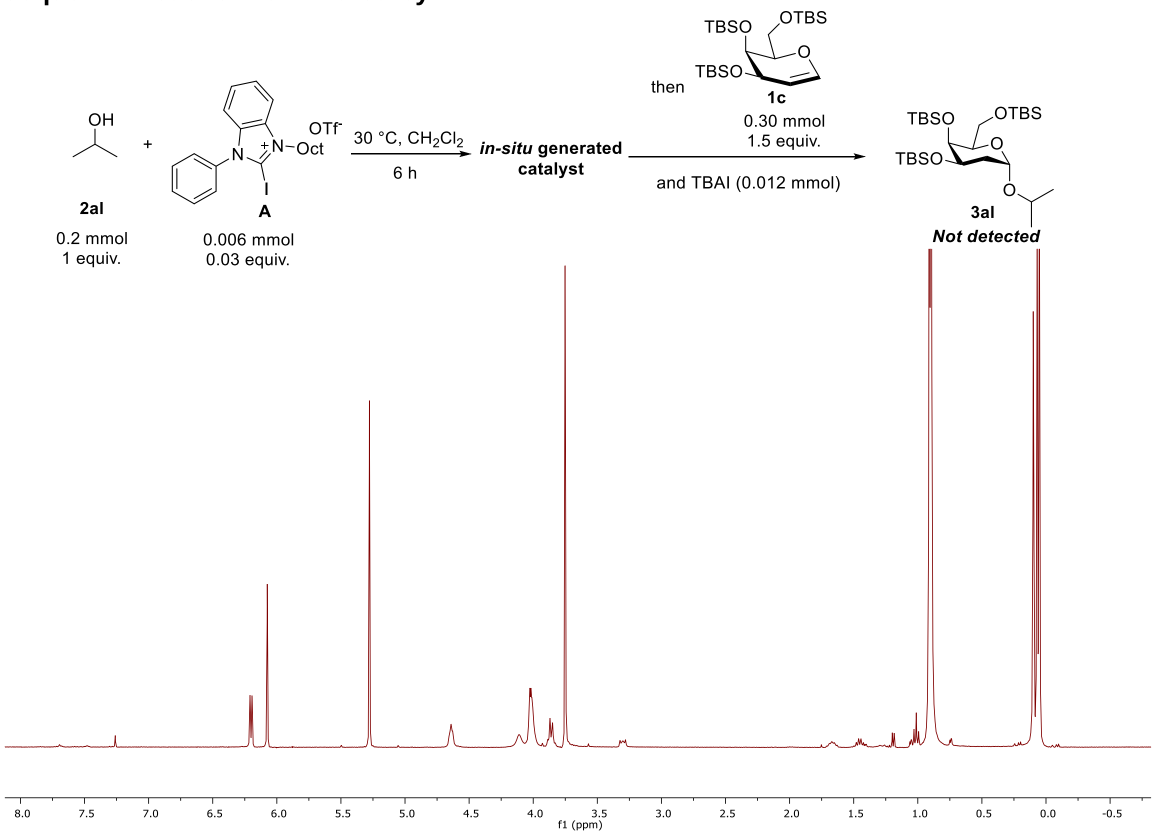

Supplementary figure S38: Crude ^{1}H NMR spectra for the sequential reaction terminated by TBAI (Related to Figure 7c in the manuscript)

Primary kinetic isotope effect studies using deuterated substrate (Related to Figure 5, equation (3) (4) and (5) in the manuscript)

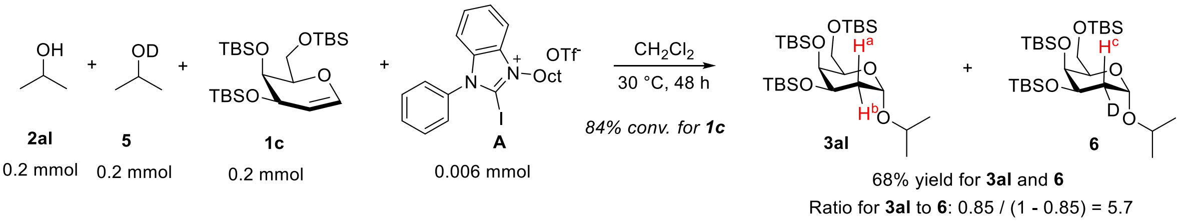

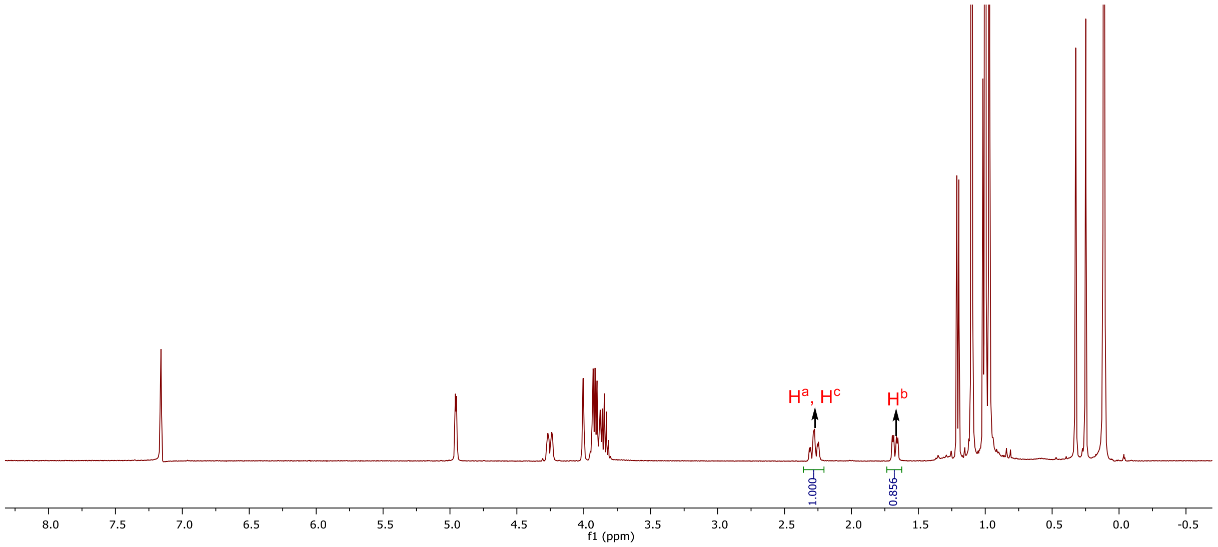

Supplementary figure S39: ^{1}H spectra for the isolated product and calculation for the ratio of **3al** to **6** (Related to Figure 5, equation (3) at high conversion in the manuscript)

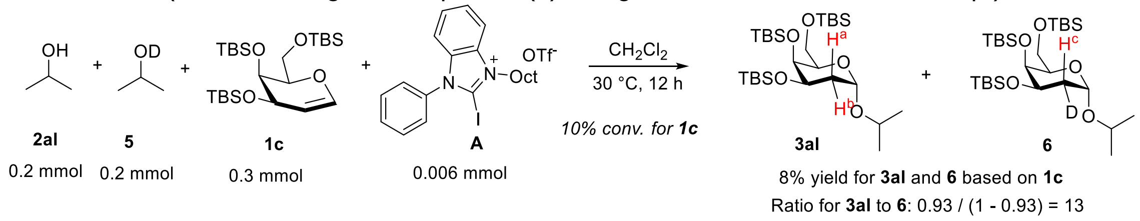

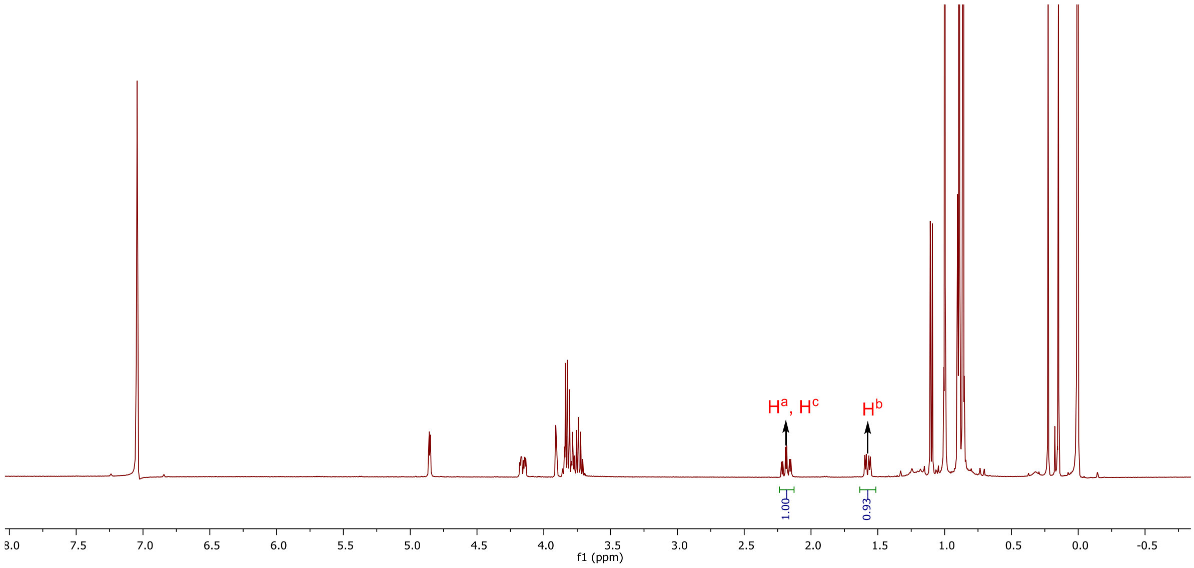

Supplementary figure S40: ^{1}H spectra for the isolated product and calculation for the ratio of **3al** to **6** (Related to Figure 5, equation (4) at low conversion in the manuscript)

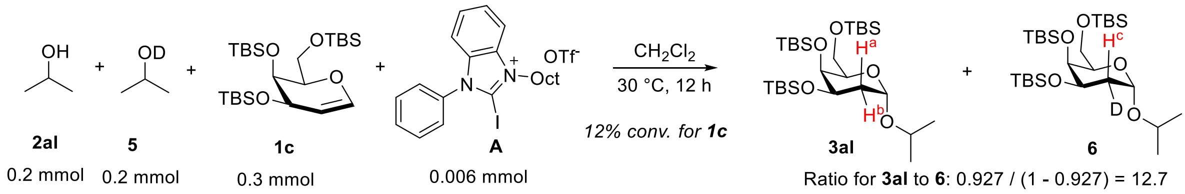

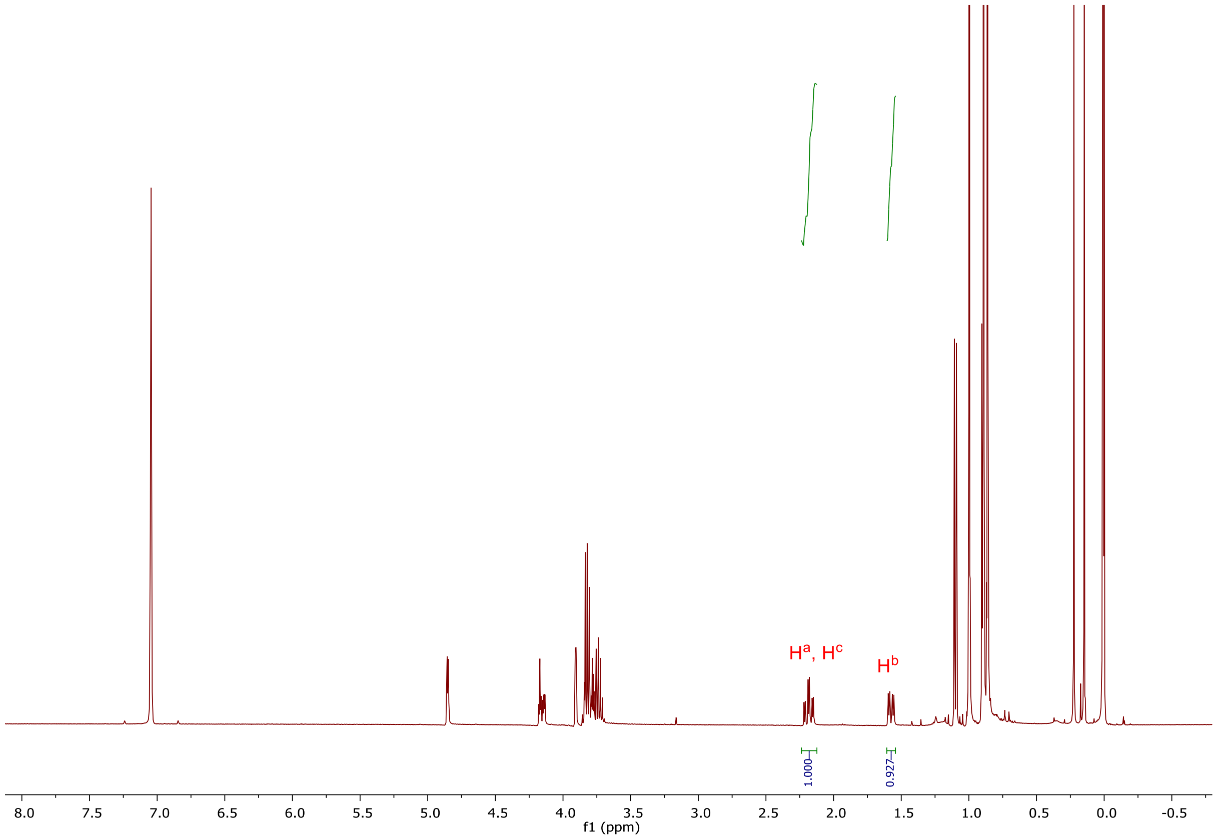

Supplementary figure S41: Repeated Primary KIE experiment purified on normal silica gel

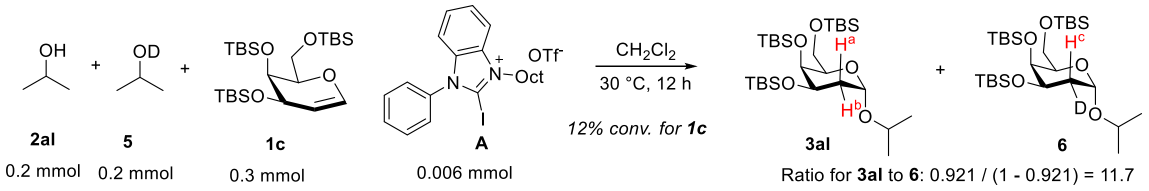

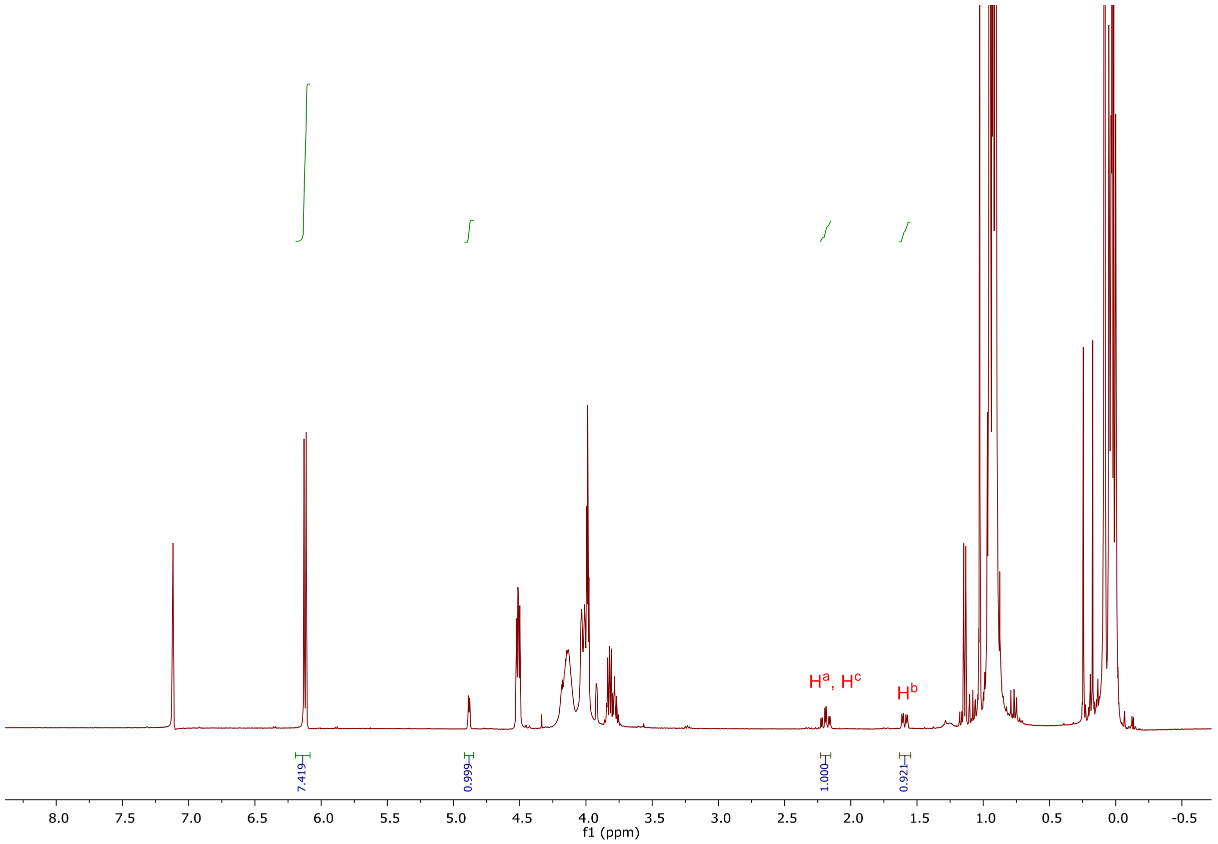

Supplementary figure S42: Repeat experiment purified on basified silica gel

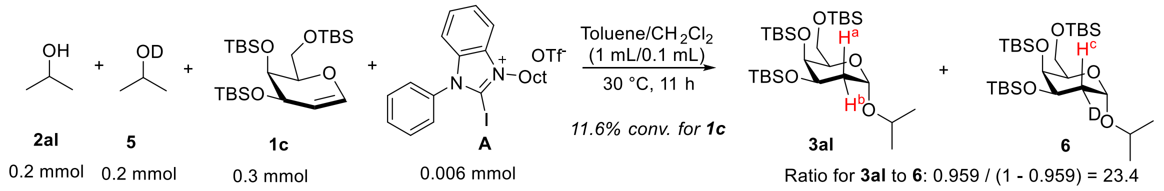

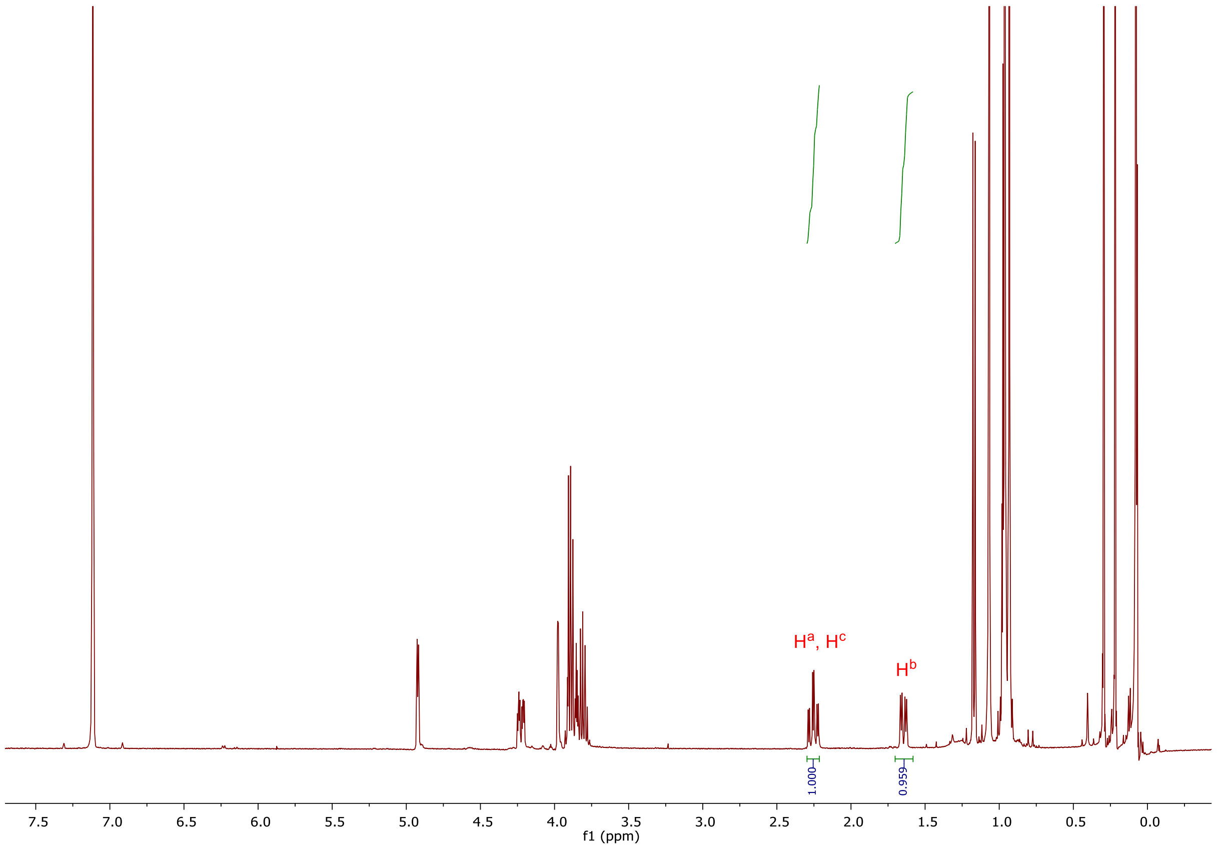

Supplementary figure S43: ^{1}H spectra for the isolated product and calculation for the ratio of **3al** to **6** in 10:1 $PhCH_3$:CH_2Cl_2 (Related to Figure 5, equation (5) in the manuscript)

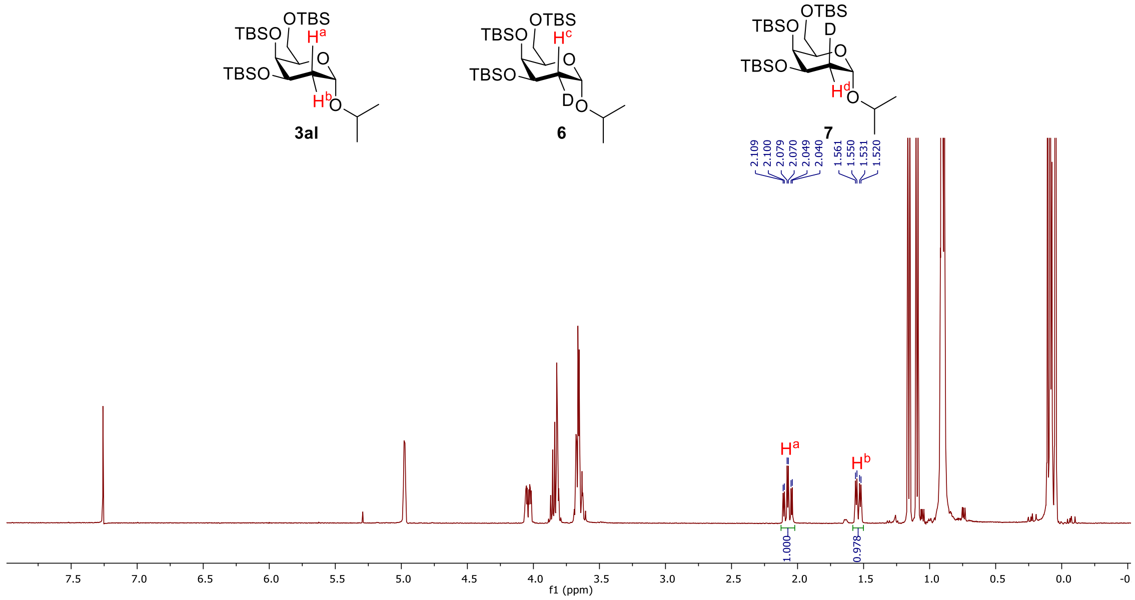

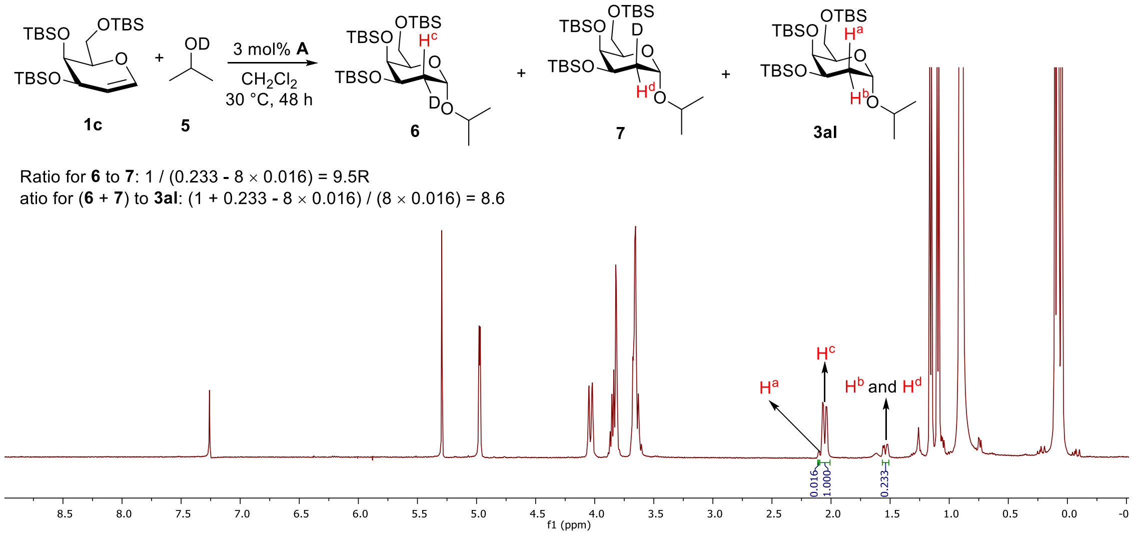

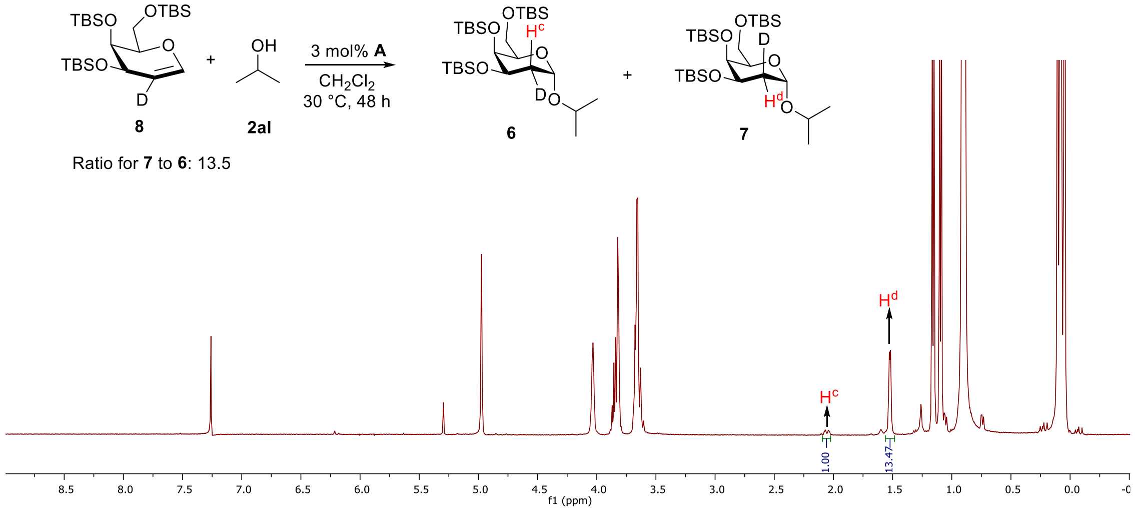

Supplementary figure S44: Deuterated experiments show deuterium label scrambling at C2 (Related to Figure 5, equation (1) and (2) in the manuscript)

Secondary kinetic isotope effect studies using deuterated substrate (Related to Figure 5, equation (6) and (7) in the manuscript)

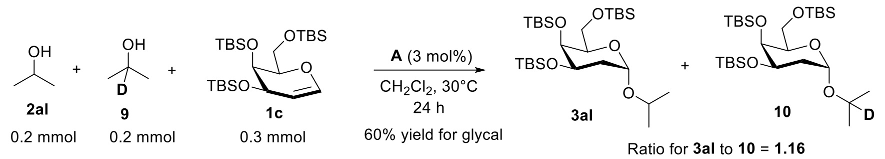

Ratio for **3al** to **10** = **1.16**

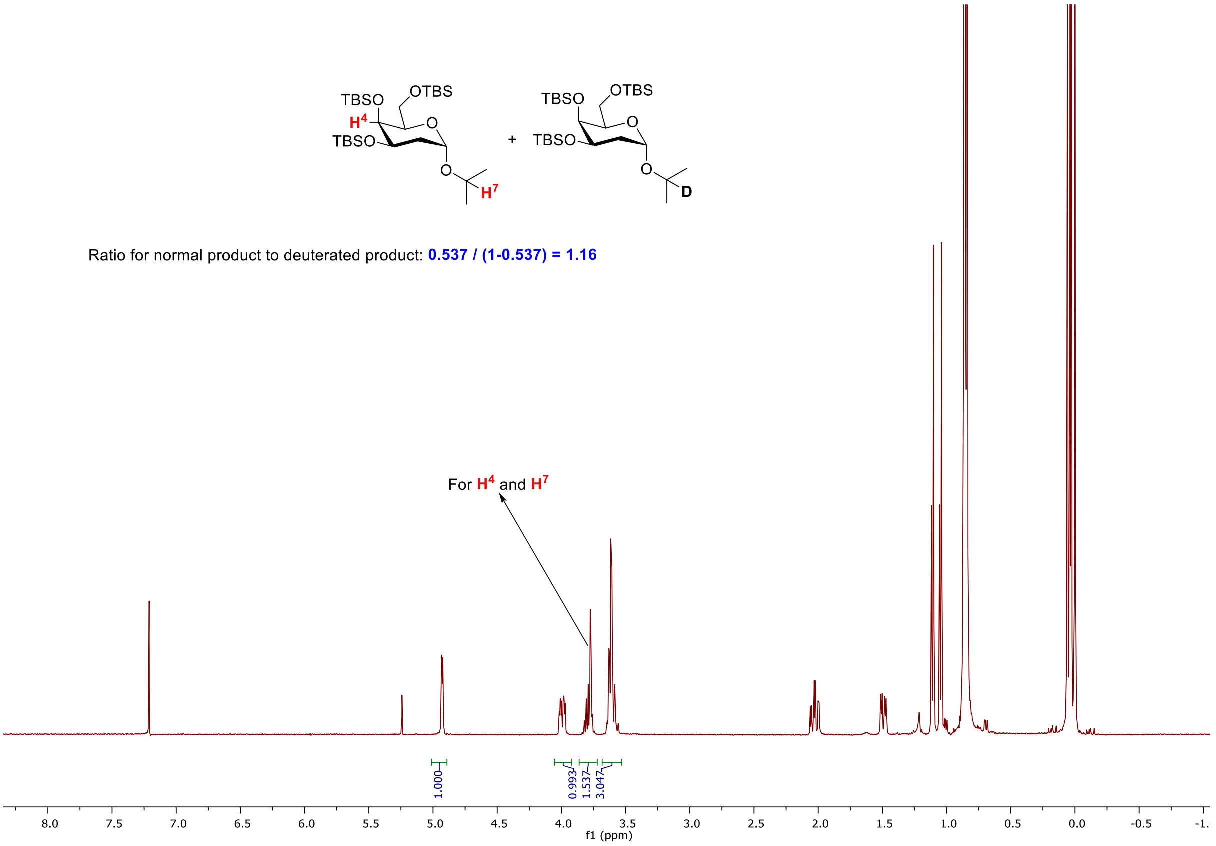

Supplementary figure S45: Intermolecular competition experiments to understand secondary kinetic isotopic effects for isopropanol (Related to Figure 5, equation (6) in the manuscript)

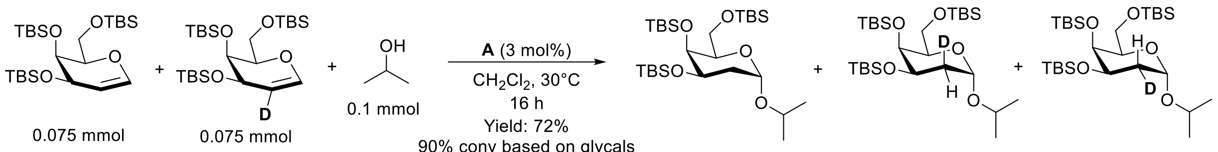

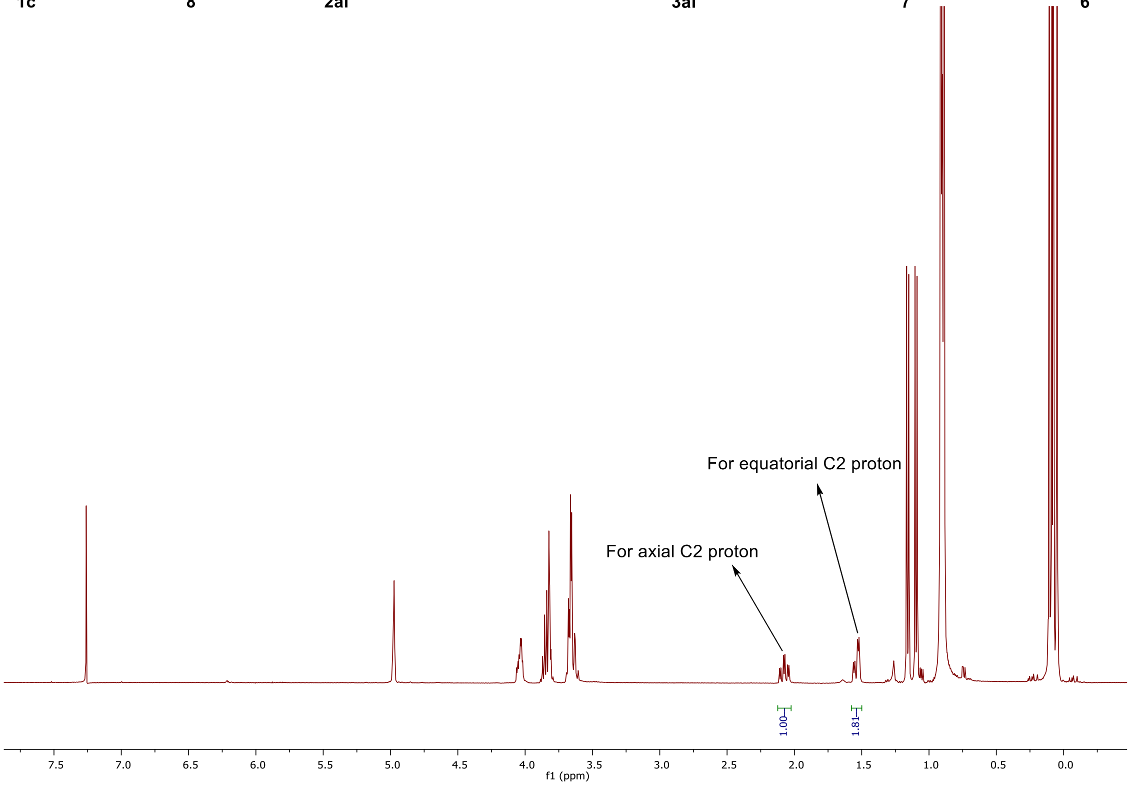

Supplementary figure S46: Intermolecular competition experiments to understand secondary kinetic isotopic effects for **1c** and **8** (Related to Figure 5, equation (7) in the manuscript)

NMR experiments to elucidate the interaction between compound E and TBAI

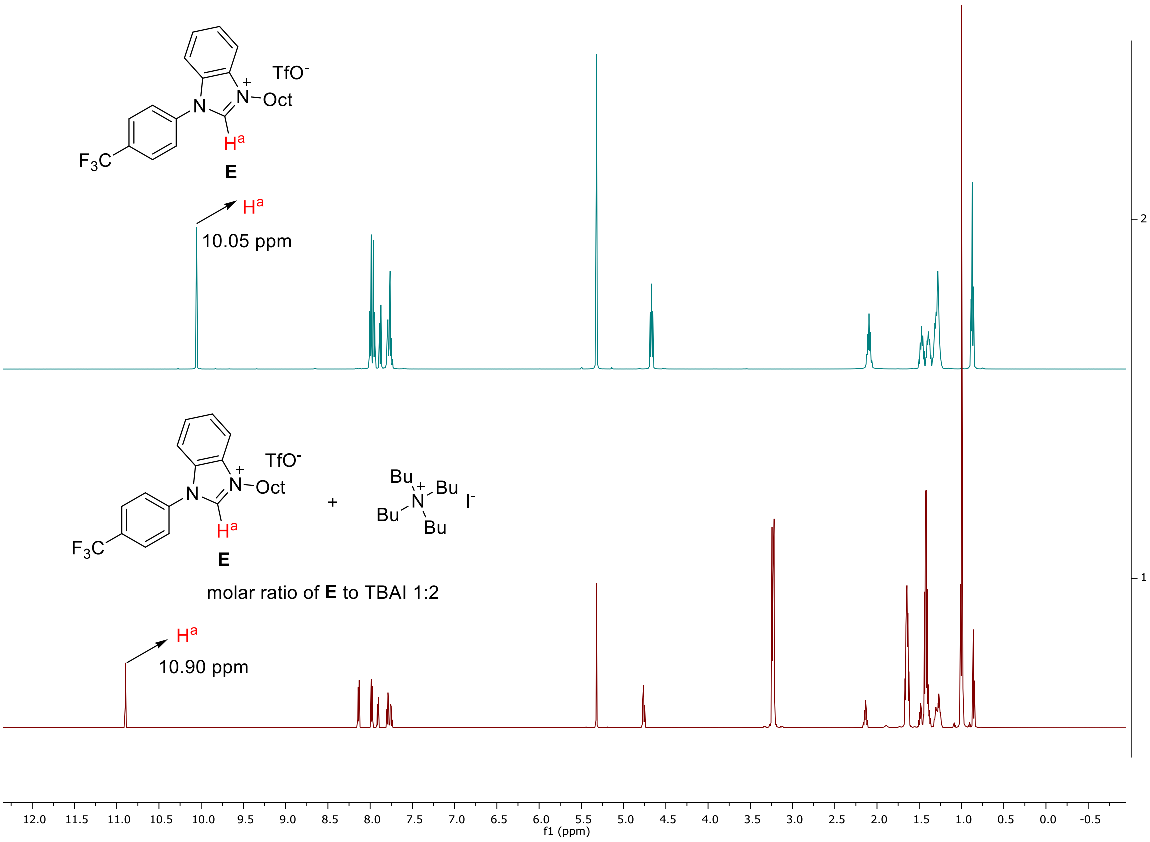

Supplementary figure S47: Stacked ^{1}H NMR spectra analysis for the interaction of compound **E** and TBAI

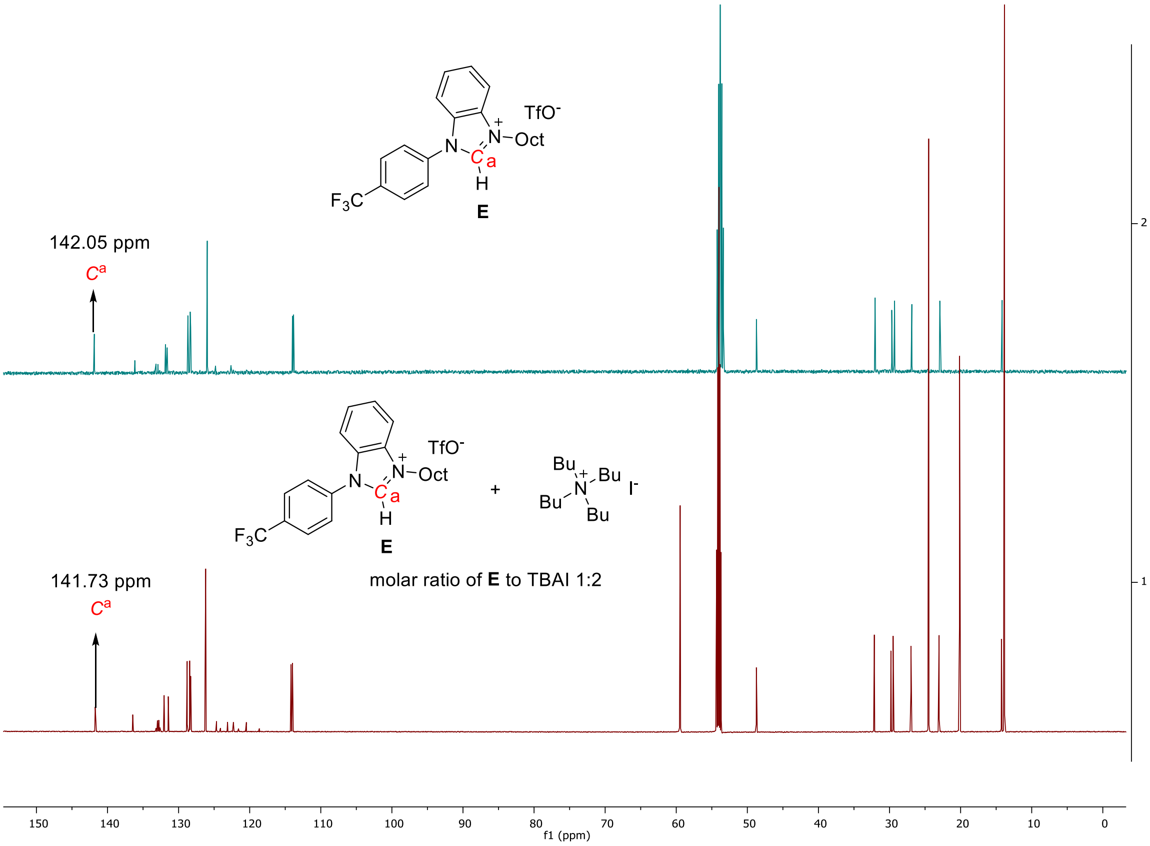

Supplementary figure S48: Stacked ^{13}C NMR spectra analysis for the interaction of compound **E** and TBAI

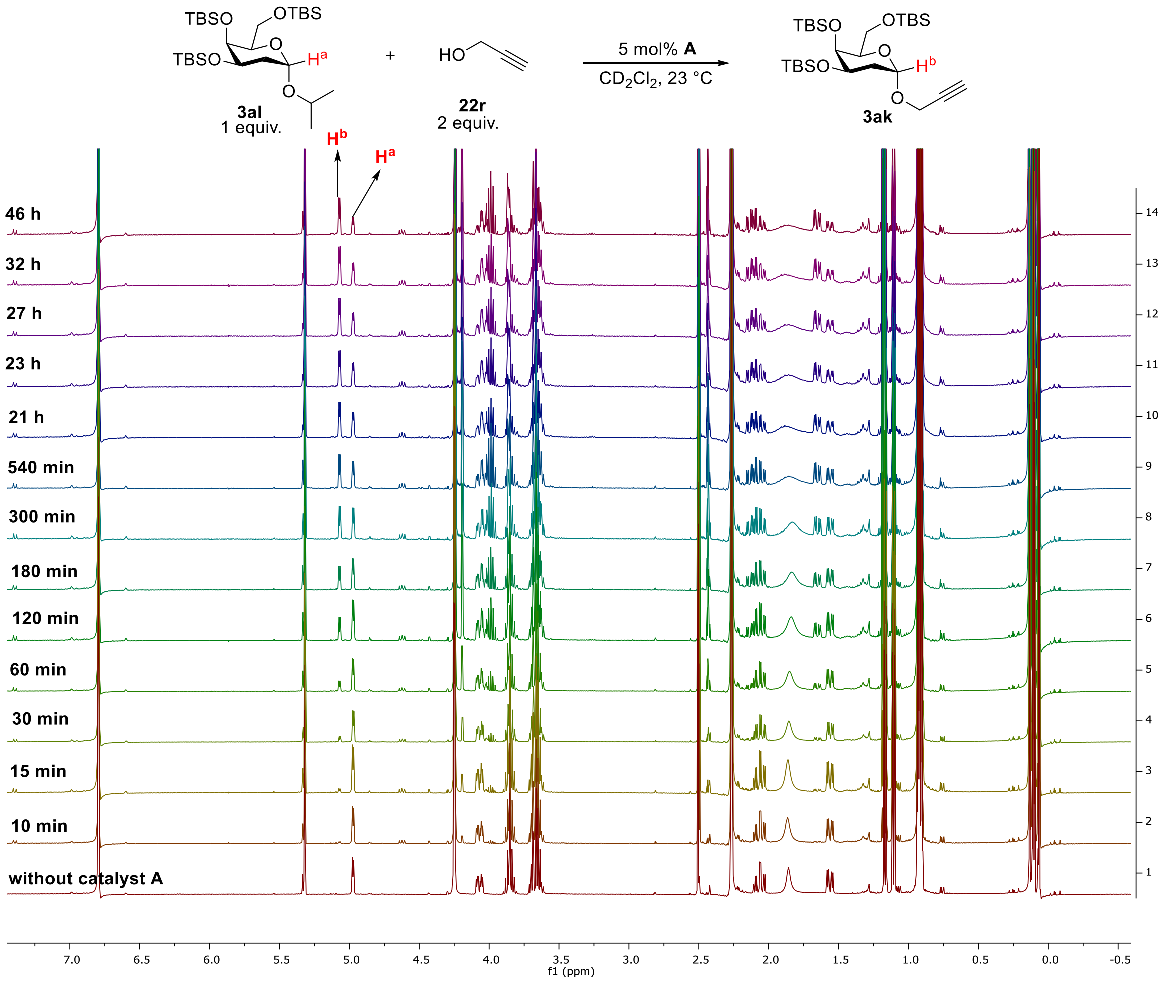

Supplementary figure S49: Stacked ^{1}H NMR spectra for the dynamic acceptor exchange experiment

Supplementary Table S10: Concentration of **3al** and **3ak** for the acceptor exchange experiments

Time/min	[3al]/M	[3ak]/M
10	0.00302	0.0815
15	0.00604	0.07977
30	0.01164	0.07287
60	0.01897	0.06511
120	0.02889	0.05433
180	0.03493	0.04916
300	0.0414	0.04183
540	0.04441	0.03751
1260	0.047	0.03493
1380	0.04873	0.03406
1620	0.05045	0.03234
1920	0.05304	0.02717
2760	0.05433	0.02717

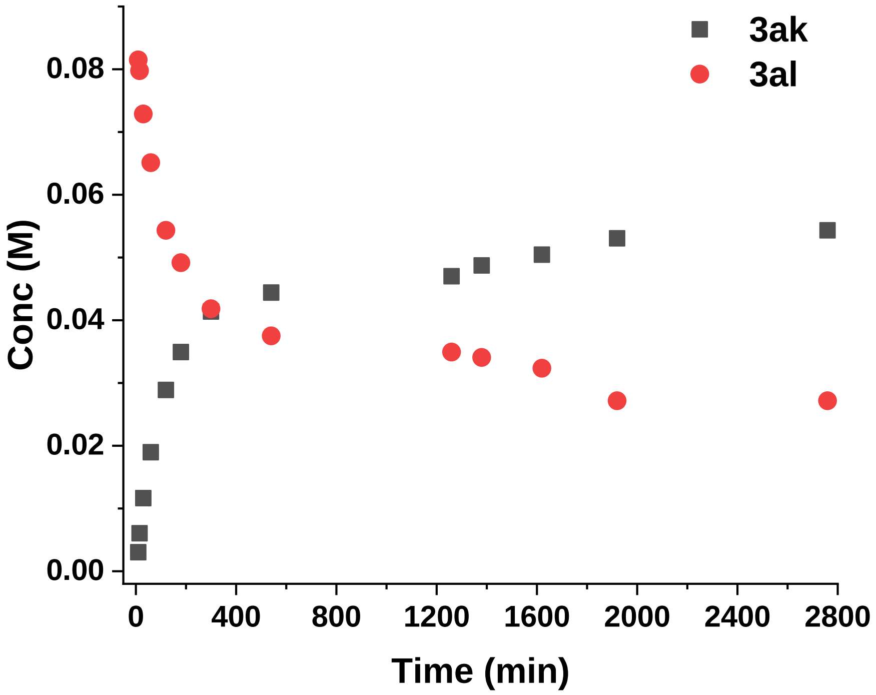

Supplementary figure S50: Temporal kinetic for the dynamic acceptor exchange experiment (Related to Figure 8c in the manuscript)

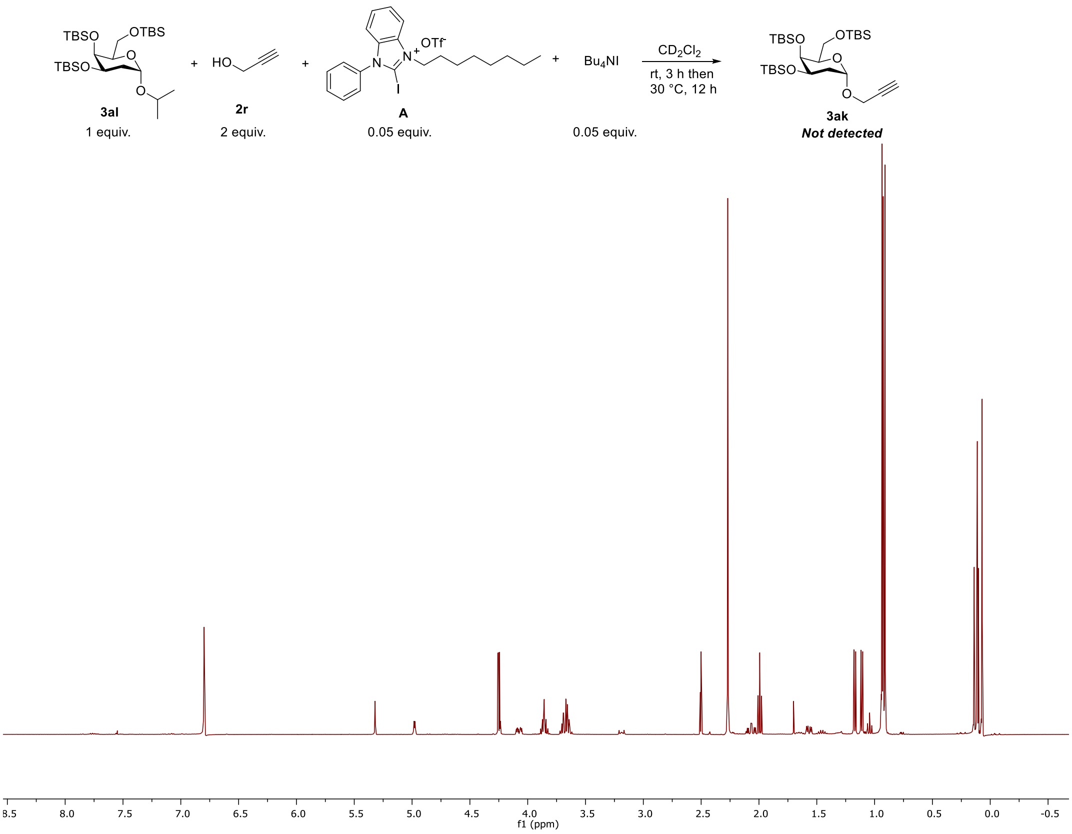

Supplementary figure S51: Competition experiment with TBAI showed XB catalyst poisoning halted the dynamic acceptor exchange

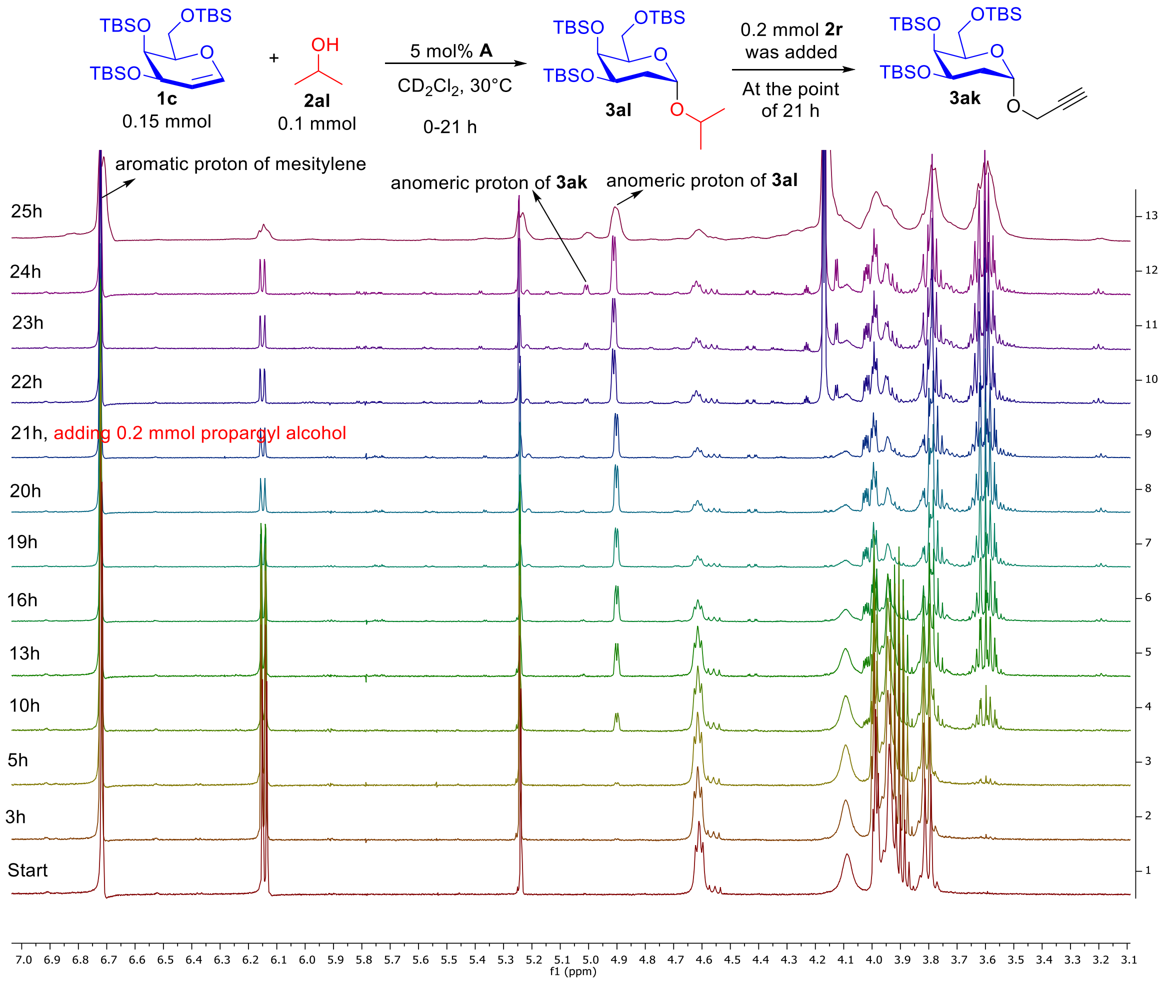

Supplementary figure S52: Stacked ^{1}H NMR spectra for the *in-situ* acceptor exchange experiment

Supplementary Table S11: Concentration of **3al** and **3ak** for the in situ acceptor exchange experiments

Time/h	[3al]/M	[3ak]/M	Comments
0	0		
3	0		
5	0.0021		
10	0.0360		
13	0.0770		
16	0.1304		
19	0.1844		
20	0.1881		
21	0.1882	0	0.2 mmol acceptor **2r** was added
22	0.1717	0.0118	
23	0.1663	0.0151	
24	0.1633	0.0227	
25	0.1597	0.0320	

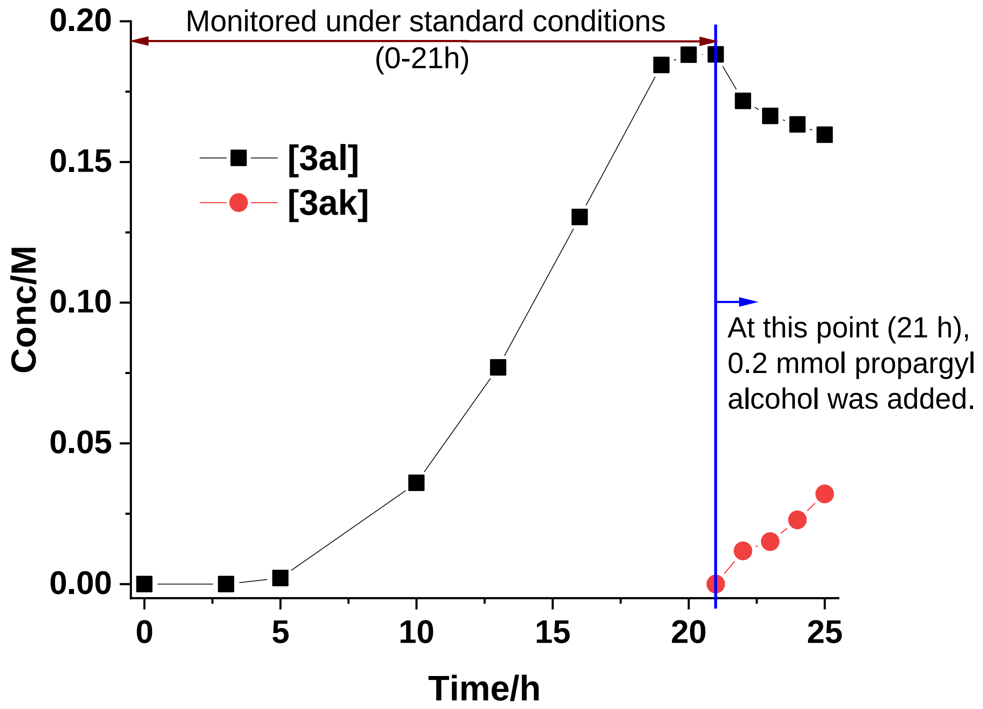

Supplementary figure S53: Temporal kinetic for the dynamic *in-situ* acceptor exchange experiment

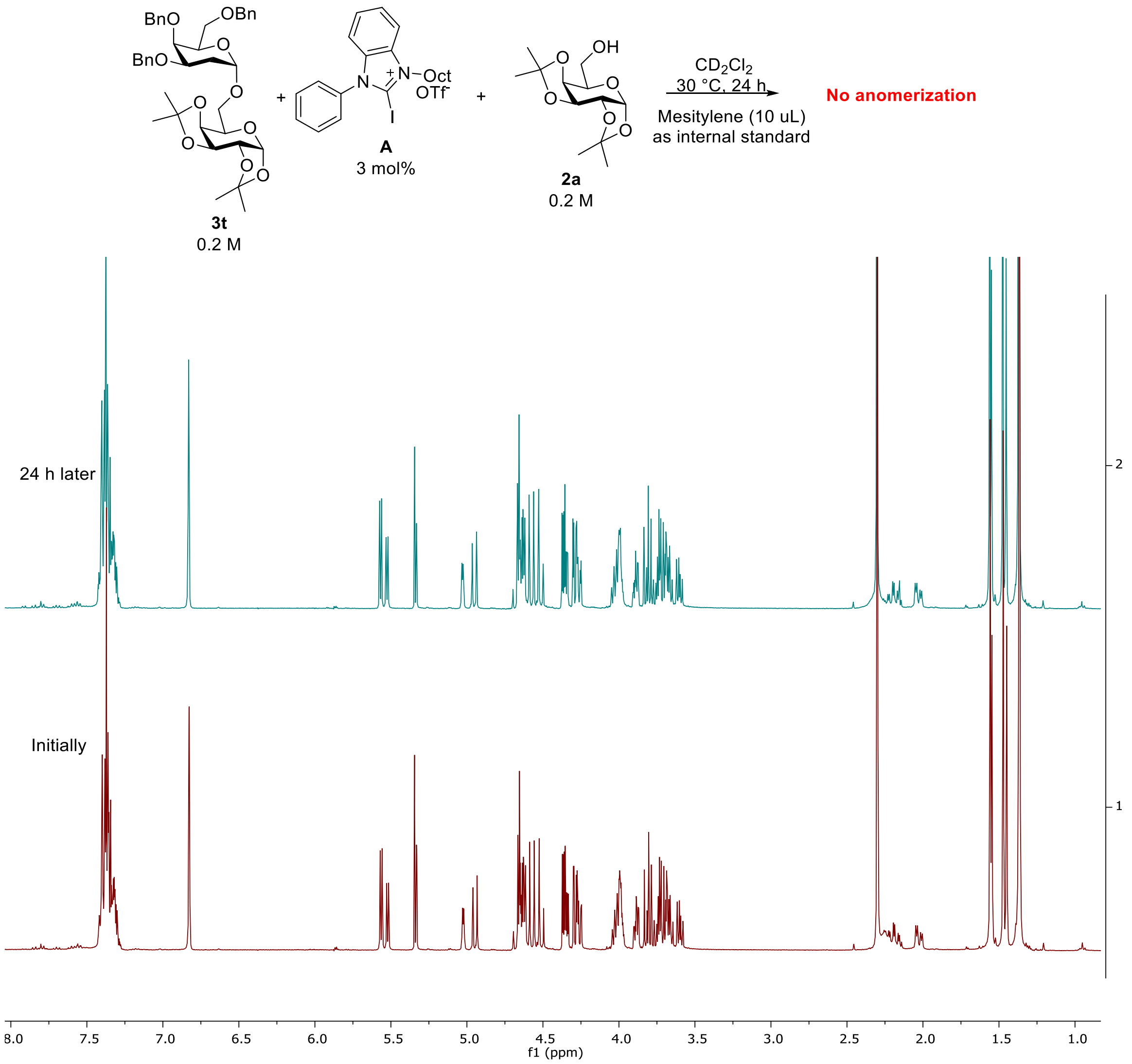

Supplementary figure S54: Determination of anomerization for the compound **3t**

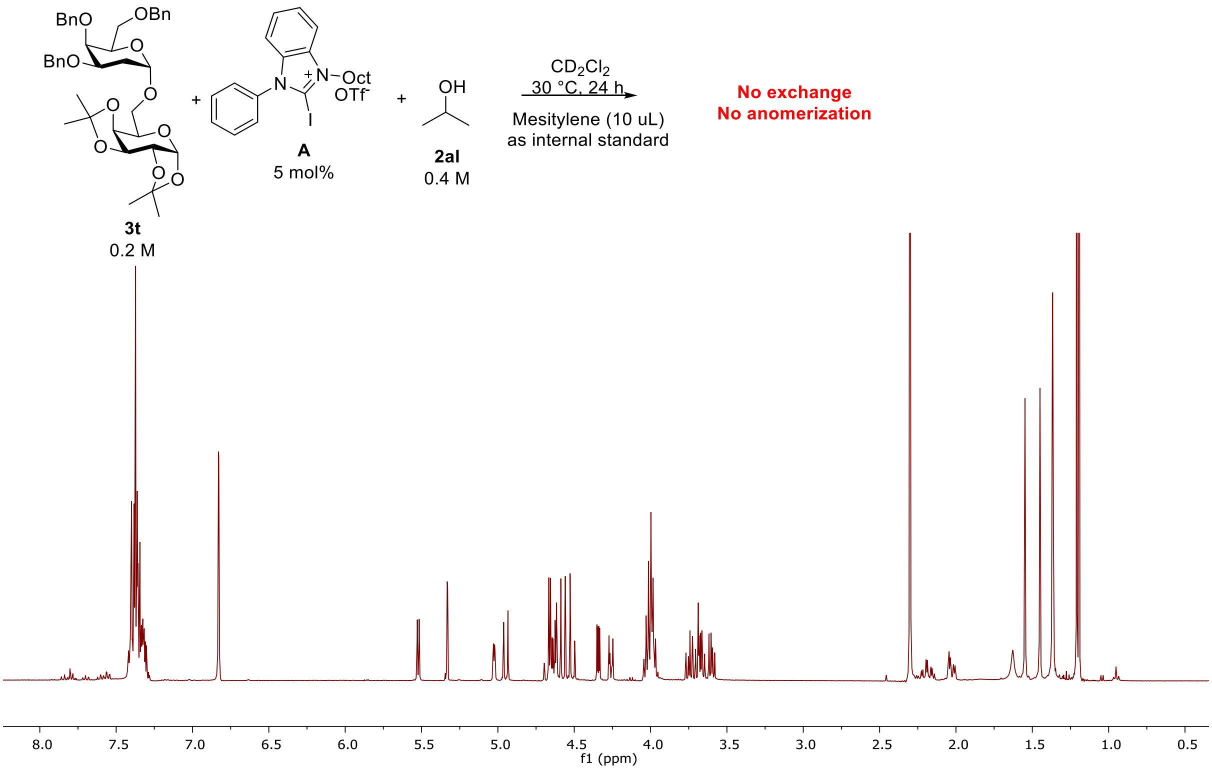

Supplementary figure S55: Determination of anomerization and acceptor exchange for the compound **3t**

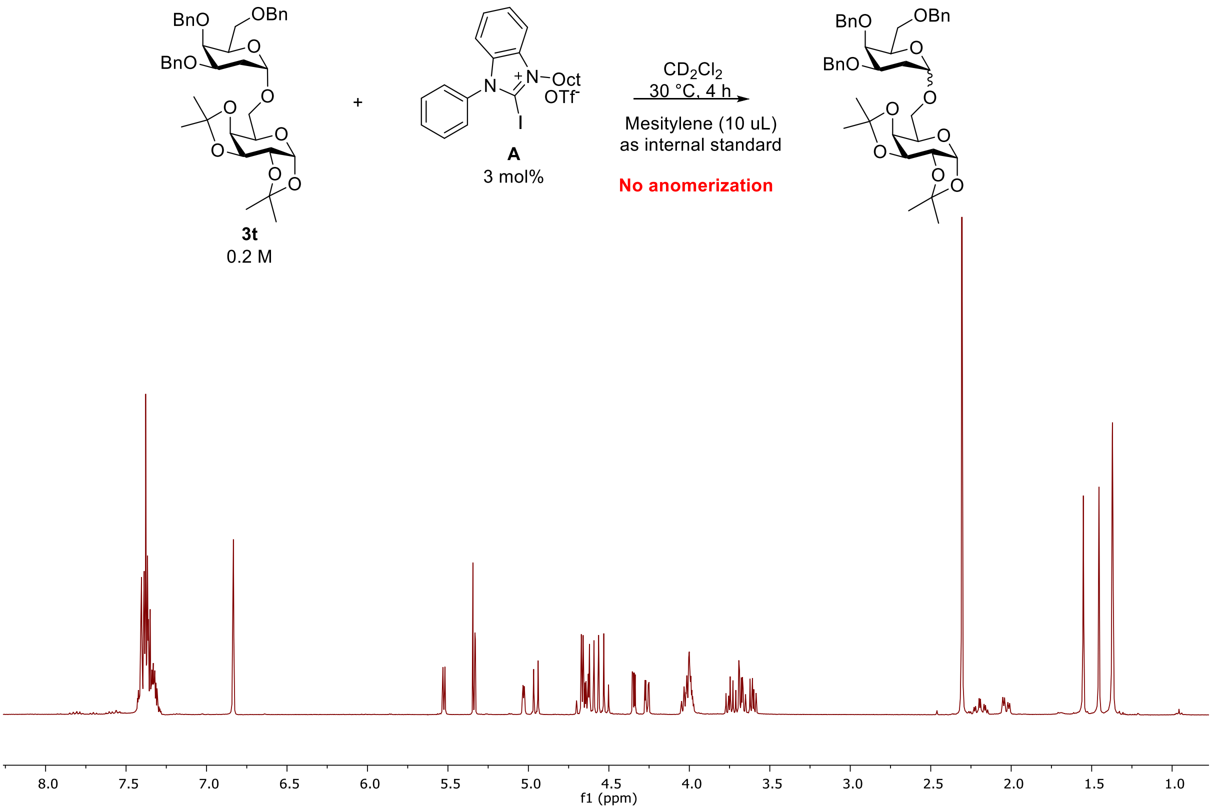

Supplementary figure S56: Determination of anomerization for the compound **3t** in the absence of acceptor

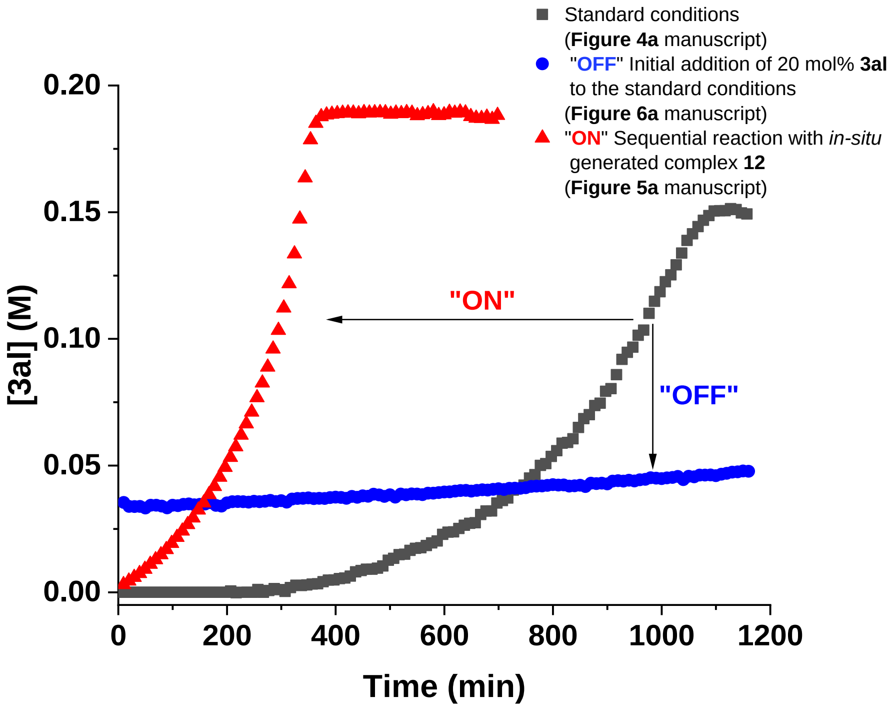

Supplementary figure S57: Overlap of temporal formation of **3al** under three different switchable conditions

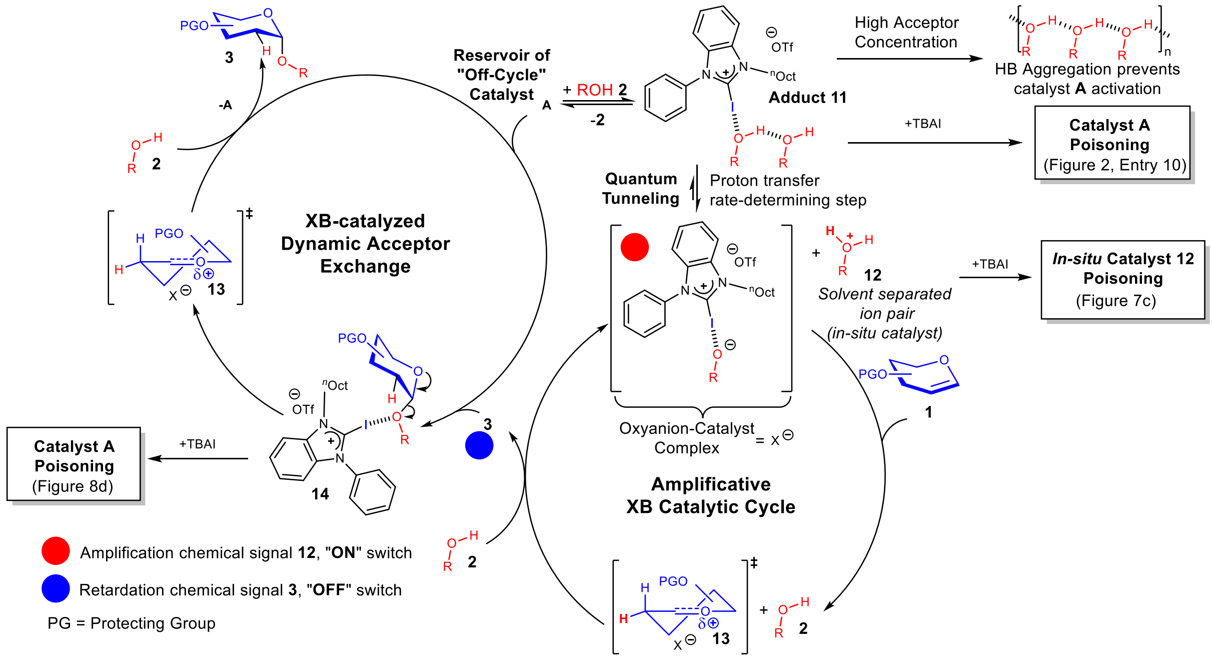

Supplementary figure S58: Proposed XB-catalyzed autoinductive glycosylation mechanism within an intrinsic biomimetic systems network

Supplementary table S12: Benchmarking experiments comparing XB catalysis with established thiourea and thiouracil catalyzed protocols.

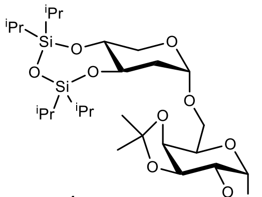

4a

Thiourea **15**: Yield <5%[a]
Thiouracil **16**: Yield <5%[b]
XB catalyst **A**: 86%, α:β 83:17

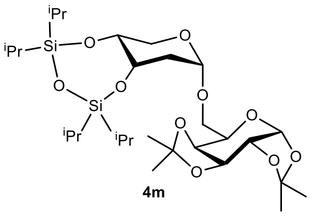

4m

Thiourea **15**: 20%, α:β >20:1[a]
Thiouracil **16**: Yield <5%[b]
XB catalyst **A**: 55%, α:β >20:1

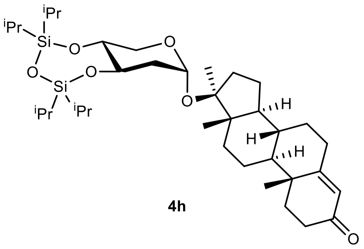

4h

Thiourea **15**: Yield <5%[a,c]
Thiouracil **16**: Yield <5%[b,c]
XB catalyst **A**: 81%, α:β 80:20

[a]Using the benchmarking experiments modified procedure method **A**, acceptor (0.2 mmol), glycosyl donor (0.24 mmol), catalyst **15** (1 mol%), CH_2Cl_2 (0.6 mL), 40˚C, 48 h; [b]Using the benchmarking experiments modified procedure method **B**, acceptor (0.2 mmol), glycosyl donor (0.24 mmol), catalyst **16** (1 mol%), CH_2Cl_2 (1 mL), 40˚C, 18 h; [c]5 mol% catalyst, 24 h.

Supplementary table S13: Temporal concentrations of product in the presence of catalyst **A** and HCl in CD_2Cl_2

Time/min	[**3al**]/M In the present of 3 mol% **A**	[**3al**]/M In the present of 3 mol% HCl
8	0.100	0.0752
26	0.0989	0.0703
40	0.0988	0.0688
50	0.101	0.0652
60	0.0992	0.0637
90	0.101	0.0591
120	0.0977	0.0524
270	0.103	0.0404
360	0.101	0.0362
540	0.0995	0.0270
1170	0.0988	0.0217

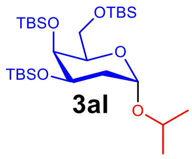

3al

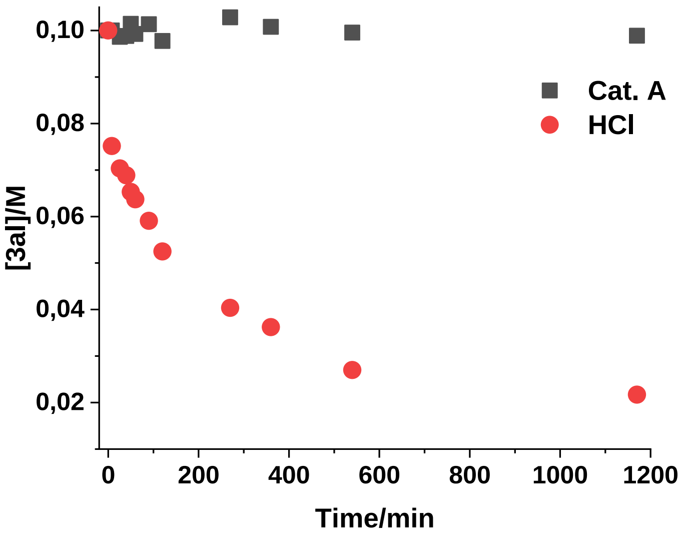

Supplementary figure 59: Decomposition kinetics in the presence of catalyst **A** and HCl performed in CD_2Cl_2

Supplementary table S14: Temporal concentrations of product in the presence of catalyst **A** and TsOH in CD_3CN

Time/min	[**3al**]/M In the present of 3 mol% **A**	[**3al**]/M In the present of 3 mol% TsOH
5	0.0774	0.0420
15	0.0781	0.0373
30	0.0773	0.0311
60	0.0779	0.0267
120	0.0774	0.0223
180	0.0779	0.0195
240	0.0787	0.0171
360	0.0782	0.0152
480	0.0779	0.0140
720	0.0782	0.0116

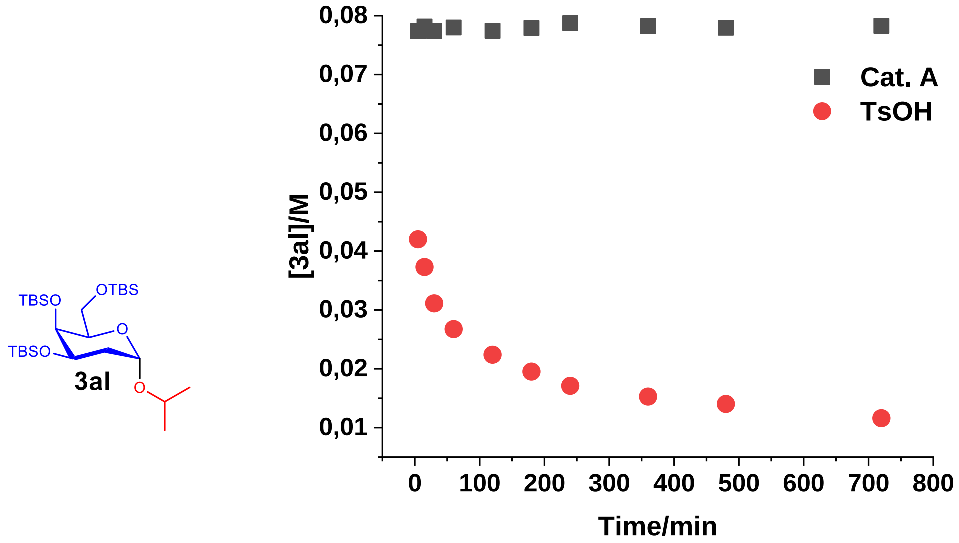

Supplementary figure 60: Decomposition kinetics in the presence of catalyst **A** and TsOH performed in CD_3CN

Supplementary table S15: Decomposition cases due to *p*TsOH catalysis when challenging tertiary acceptors are employed.

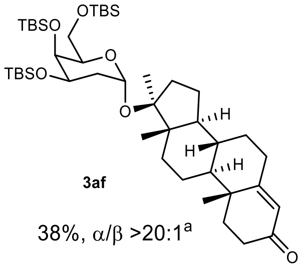

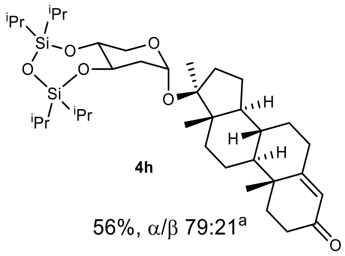

[a]Procedure: To a dry tube, acceptor (0.1 mmol) and donor (0.12 mmol) were added under argon atmosphere and put under high vacuum for 1 h, then $TsOH•H_2O$ (1 mol%) and CH_2Cl_2 (1 mL) was injected. After that, the mixture was stirred at 40˚C oil bath for 18 h. The anomeric ratio was determined by crude ^1H NMR spectra; yields were isolated yield.

Supplementary table S16: Comparison between halogen bond catalysis and other reported leading systems. (Related to Figure 10 in the manuscript)

S16-1. General reaction and overview of acceptors and donors used for the comparison

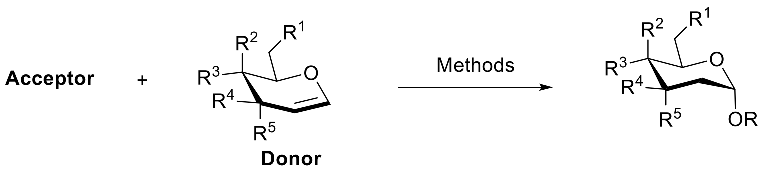

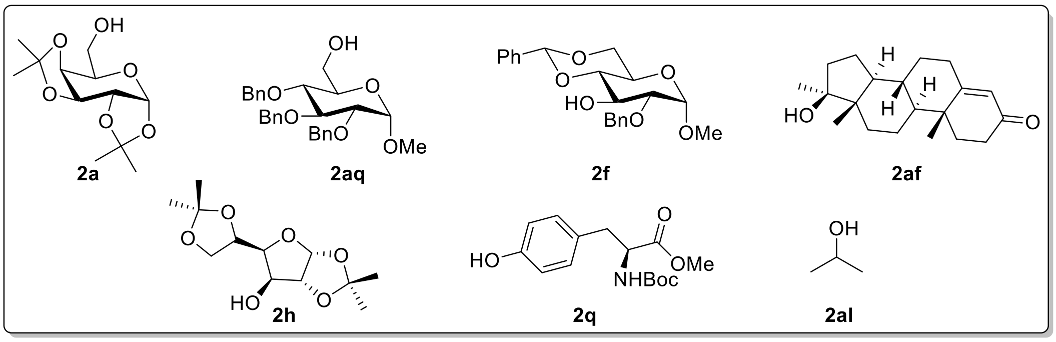

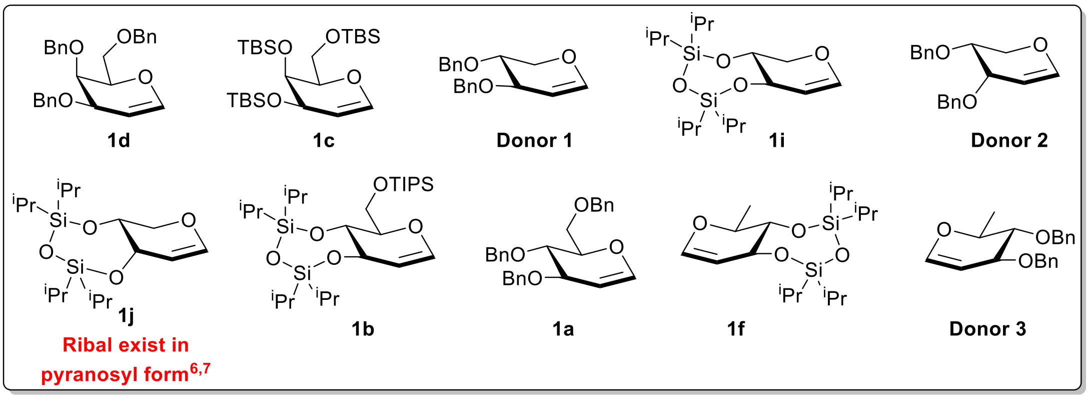

S16-2. The results for the cases with the reported leading systems and the halogen bond catalysis

Entry	Acceptor	Donor	Schreiner thiourea catalysis (**Method A**)		Thiouracil catalysis (**Method B**)		XB
			Reported	Reproduced	Reported	Reproduced	
1	**2a**	**1d**	96%, α/β >20:1	94%,[a] α/β >20:1	95%, α/β >20:1	96%,[a] α/β >20:1	55%,[a] α/β 85:15
2	**2aq**	**1d**	98%, α/β >20:1	93%,[a] α/β >20:1	Not reported	-	70%,[a] α/β >20:1
3	**2f**	**1d**	96%, α/β >20:1	72%,[a] α/β >20:1	89%, α/β >20:1	72%,[a] α/β >20:1	46%[b] α/β >20:1
4	**2a**	**1c**	72%, α/β >20:1	68%,[a] α/β >20:1	11%,[c,e] α/β >20:1	<5%[d,e]	91%,[a] α/β 91:9

[a]Isolated yield after flash column chromatography. [b]Isolated yield with ca. 5% inseparable Ferrier type product impurity. [c]68% yield was obtained when 2-methyl-THF was used as solvent at reflux temperature for 18 h. [d]The NMR yield was determined by using ^{1}H NMR with 1,3,5-trimethoxybenzene as an internal standard. [e] 48h reaction.

S16-3. Reproducing the reaction with reported transition metal catalysis and pyridinium catalysis

Entry	Acceptor	Donor	Palladium catalysis (**Method C**)		Gold catalysis (**Method D**)		Pyridinium catalysis (**Method E**)	
			Reported	Reproduced	Reported	Reproduced	Reported	Reproduced
1	**2a**	**1d**					94%, α	79%, α 10% SM recovered
2	**2aq**	**1d**			89%, α/β >20:1	76%, α/β >20:1		
3	**2h**	**1d**	74%, α/β >20:1	68%, α/β >20:1				

S16-4. Comparison for the reaction with rarely explored D-xylal donor **1i** and D-ribal donor **1j**

Entry	Acceptor	Donor	Palladium catalysis (**Method C**)	Gold catalysis (**Method D**)	Pyridinium catalysis (**Method E**)	XB catalysis
1	**2a**	**1i**	51%<br>α/β 73:27	70%<br>α/β 76:24	66%<br>α/β 84:16	86%<br>α/β 83:17
2	**2a**	**1j**	60%<br>α/β >20:1	45%<br>α/β 60:40	86%<br>α/β >20:1	64%, α/β >20:1<br>(catalyst **A**)<br>81%, α/β >20:1<br>(catalyst **Br-A**)

S16-5. Detail cases for the comparison between thiourea catalysis, thiouracil catalysis and XB catalysis

Entry	Acceptor	Donor	Schreiner thiourea Catalysis (**Method A**)	Thiouracil catalysis (**Method B**)	XB catalysis
1	**2a**	**Donor 1**	< 5%[a]	< 5%[a]	< 5%[a]
2	**2a**	**1i**	< 5%[b]	< 5%[b]	86%, α/β 83:17
3	**2a**	**Donor 2**	< 5%[a]	< 5%[a]	< 5%[a]
4	**2a**	**1j**	44%, α/β >20:1	52%, α/β >20:1	64%, α/β >20:1
5	**2af**	**Donor 1**	< 5%[a]	< 5%[a]	< 5%[a]
6	**2af**	**1i**	< 5%[b]	< 5%[b]	81%, α/β 80:20
7	**2a**	**1b**	< 5%[b]	< 5%[b]	89%, >20:1
8	**2a**	**1f**	< 5%[b]	< 5%[b]	88%, α/β 88:12
9	**2a**	**1a**	44% (NMR yield)[b]<br>α/β 76:24	64% (NMR yield)[b]<br>α/β 77:23<br>Lit[13]: 46%, α/β 5:1	64% (NMR yield)[b]<br>α/β 75:25
10	**2a**	**Donor 3**	78% (NMR yield)[b]<br>α/β 83:17	39% (NMR yield)[b]<br>α/β 87:13<br>Lit[13]: 40%, α/β 81:19	83% (NMR yield)[b]<br>α/β 86:14
11	**2q**	**1c**	< 5%[c]	< 5%[c]	75%, α/β >20:1[c]
12	**2al**	**1c**	< 5%[c]	< 5%[c]	88%, α/β >20:1[c]

[a]A mixture with inseparable Ferrier type product impurity was obtained after flash chromatography purification, the yield was calculated by ^{1}H NMR analysis. [b]The NMR yield was determined by using ^{1}H NMR spectra with 1,3,5-trimethoxybenzene as an internal standard. [c] Isolated yield after flash column chromatography.

Concentration dependence studies (Related to Figure 9a-c in the manuscript)

Supplementary table S17. Overview for the concentration dependence experiments

Entry	Concentration of catalyst **A**	Concentration of donor **1c**	Concentration of acceptor **2al**
1	3 mol% (50 uL of 0.06 M **A** CD_2Cl_2 solution)	0.3 M (73.3 mg **1c** dissolved in 450 uL CD_2Cl_2)	0.2 M (6.0 mg, 7.5 uL)
2	8 mol% (4.6 mg **A**)	0.3 M (73.3 mg **1c** dissolved in 500 uL CD_2Cl_2)	0.2 M (6.0 mg, 7.5 uL)
3	13 mol% (7.5 mg **A**)	0.3 M (73.3 mg **1c** dissolved in 500 uL CD_2Cl_2)	0.2 M (6.0 mg, 7.5 uL)
4	3 mol% (50 uL of 0.06 M **A** CD_2Cl_2 solution)	0.2 M (48.9 mg **1c** dissolved in 450 uL CD_2Cl_2)	0.2 M (6.0 mg, 7.5 uL)
5	3 mol% (50 uL of 0.06 M **A** CD_2Cl_2 solution)	0.4 M (97.7 mg **1c** dissolved in 450 uL CD_2Cl_2)	0.2 M (6.0 mg, 7.5 uL)
6	3 mol% (50 uL of 0.06 M **A** CD_2Cl_2 solution)	0.3 M (73.3 mg **1c** dissolved in 450 uL CD_2Cl_2)	0.1 M (3.0 mg, 3.8 uL)
7	3 mol% (50 uL of 0.06 M **A** CD_2Cl_2 solution)	0.3 M (73.3 mg **1c** dissolved in 450 uL CD_2Cl_2)	0.3 M (9.0 mg, 11.4 uL)
8	3 mol% (50 uL of 0.06 M **A** CD_2Cl_2 solution)	0.3 M (73.3 mg **1c** dissolved in 450 uL CD_2Cl_2)	0.4 M (12.0 mg, 15.2 uL)

1. Catalyst concentration dependence (Supplementary table S17, entries 1-3)

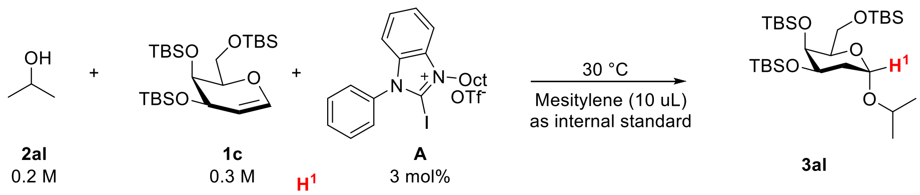

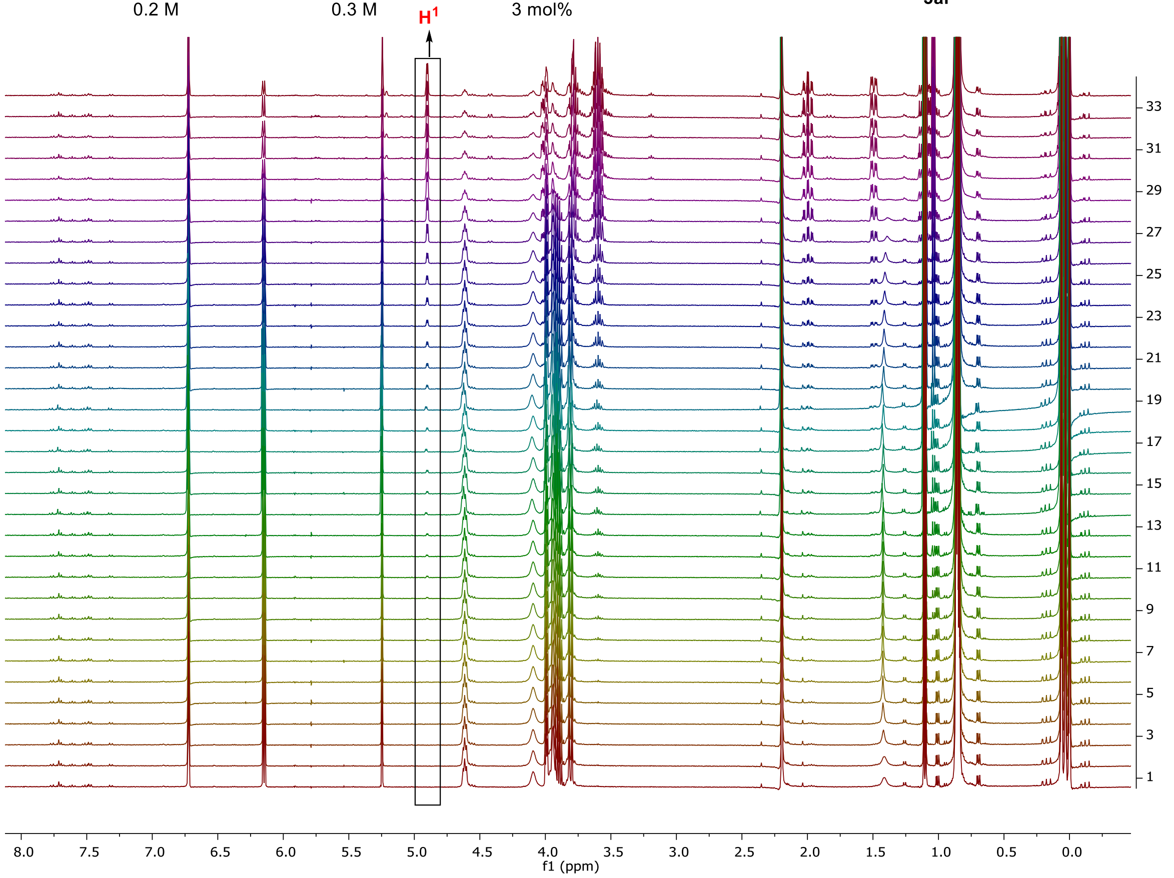

Supplementary figure S61: Stacked ^{1}H spectra for the experiment of entry 1 in supplementary table S17

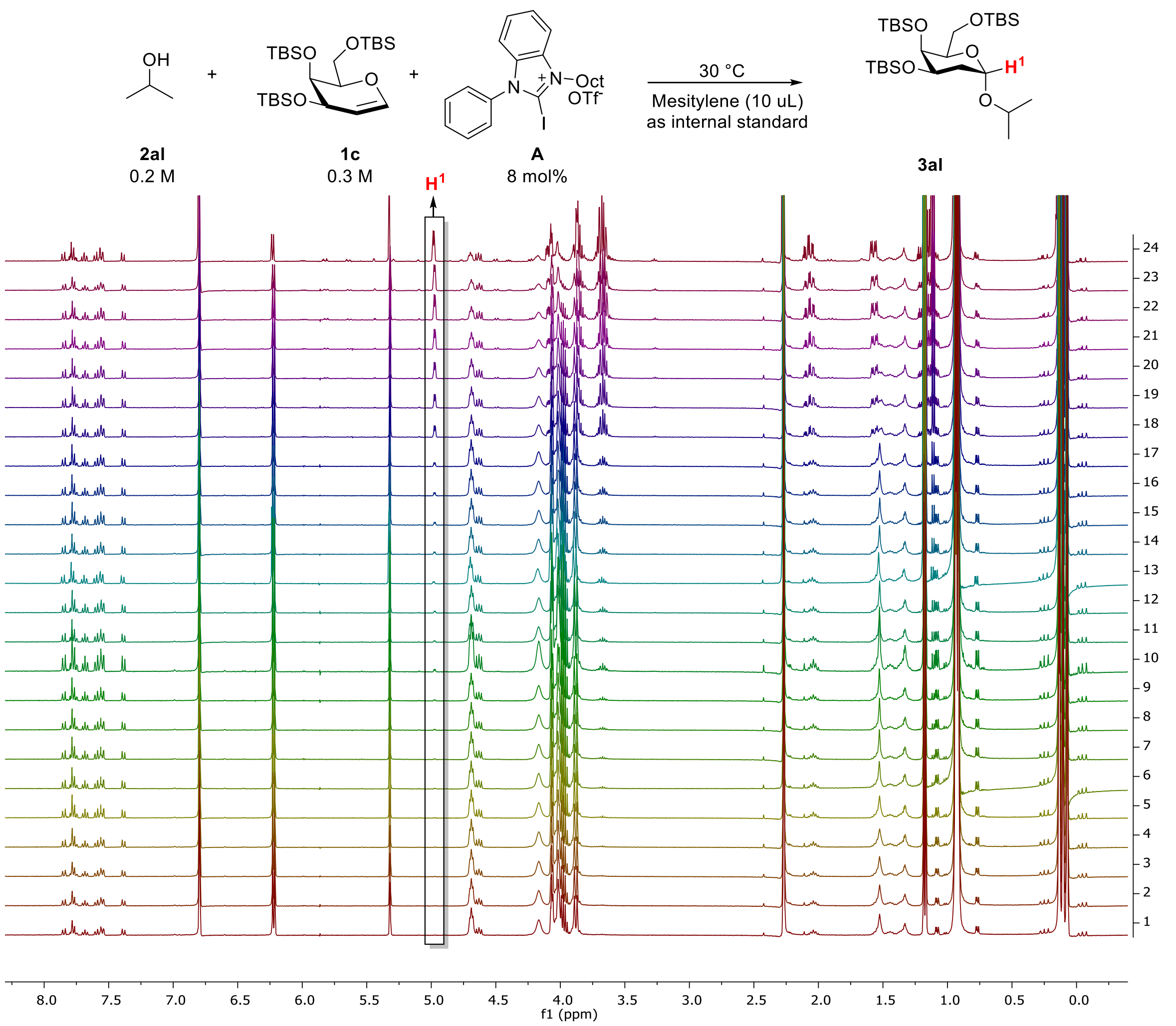

Supplementary figure S62: Stacked ^{1}H spectra for the experiment of entry 2 in supplementary table S17

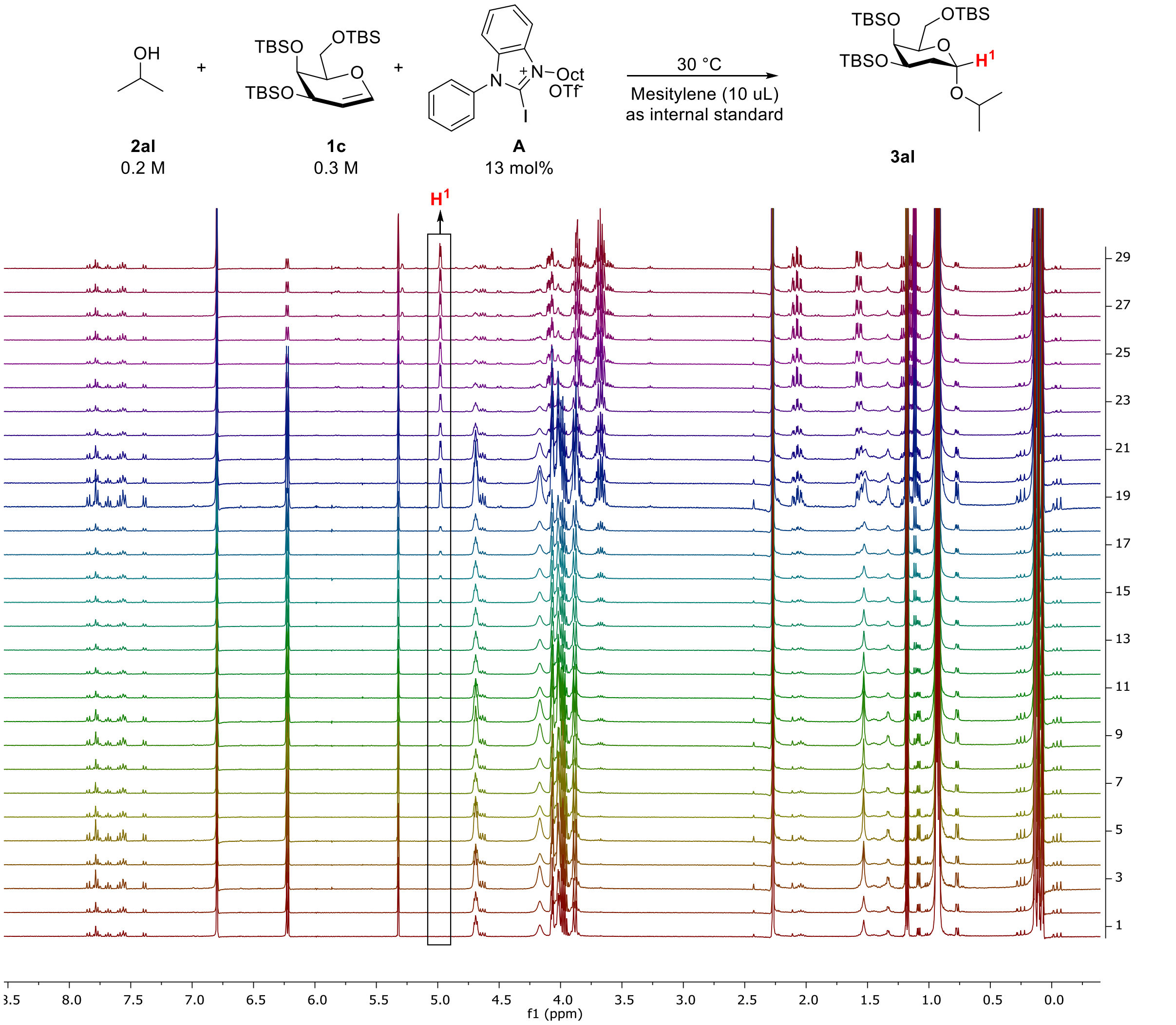

Supplementary figure S63: Stacked ^{1}H spectra for the experiment of entry 3 in supplementary table S17

Supplementary table S18. Concentration for **3al** calculated by ^{1}H NMR analysis for the experiments of entry 1-3 in supplementary table S17

Entry	3 mol% catalyst		8 mol% catalyst		13 mol% catalyst	
	Time/min	[**3al**]	Time/min	[**3al**]	Time/min	[**3al**]
1	0	0	0	0	0	0
2	25	0	33	0	30	0
3	55	0	48	0	46	0
4	85	0	63	0	62	0
5	115	0	83	0	78	0
6	145	0	103	0	93	0
7	175	0	123	0.00043	108	0
8	205	0.00043	143	0.00129	123	0.00173
9	225	0.00043	163	0.00216	143	0.00259
10	245	0.00129	183	0.00259	159	0.00431
11	265	0.00129	203	0.00561	174	0.00561
12	280	0.00388	223	0.00733	190	0.00863
13	295	0.00604	243	0.00776	207	0.01165
14	310	0.00733	263	0.01208	225	0.01466
15	325	0.00862	283	0.01294	241	0.01941
16	340	0.01164	303	0.01596	256	0.02329
17	355	0.01337	323	0.01898	276	0.0289
18	370	0.01337	443	0.06383	296	0.03752
19	385	0.0138	473	0.07548	316	0.05046
20	405	0.02027	503	0.09316	336	0.06211
21	425	0.02328	533	0.11646	356	0.07677
22	445	0.02673	563	0.14492	386	0.10697
23	465	0.03234	593	0.16218	416	0.15096
24	485	0.03924	623	0.1777	446	0.19194
25	505	0.04743			476	0.1928
26	525	0.05649			506	0.19582
27	590	0.10737			536	0.19452
28	620	0.13971			596	0.19625
29					626	0.19496

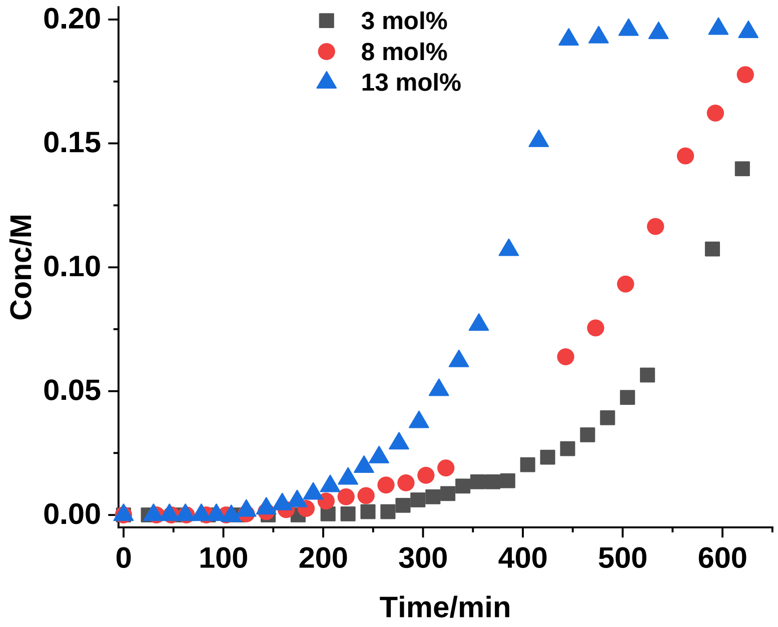

Supplementary figure S64: Overlapped profile for the catalyst concentration dependence experiments

2. Donor concentration dependence (Supplementary table S17, entries 1 and entries 4-5)

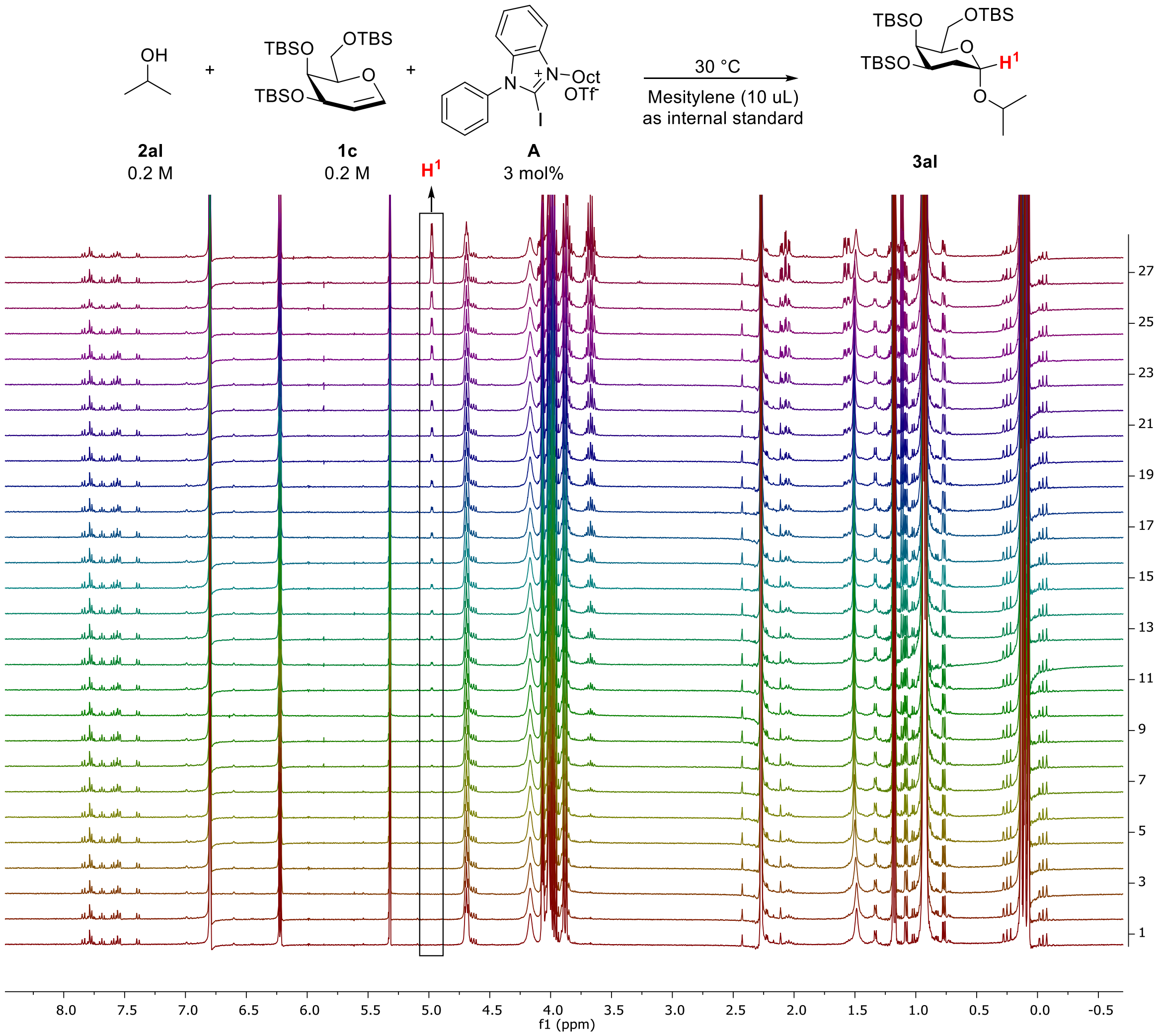

Supplementary figure S65: Stacked ^{1}H spectra for the experiment of entry 4 in supplementary table S17

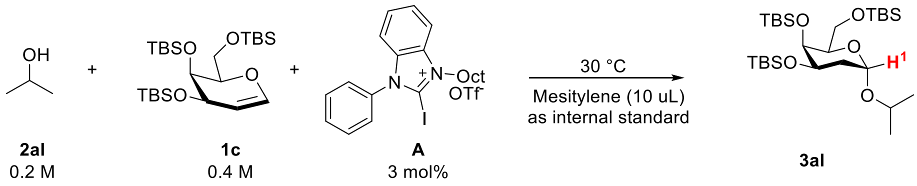

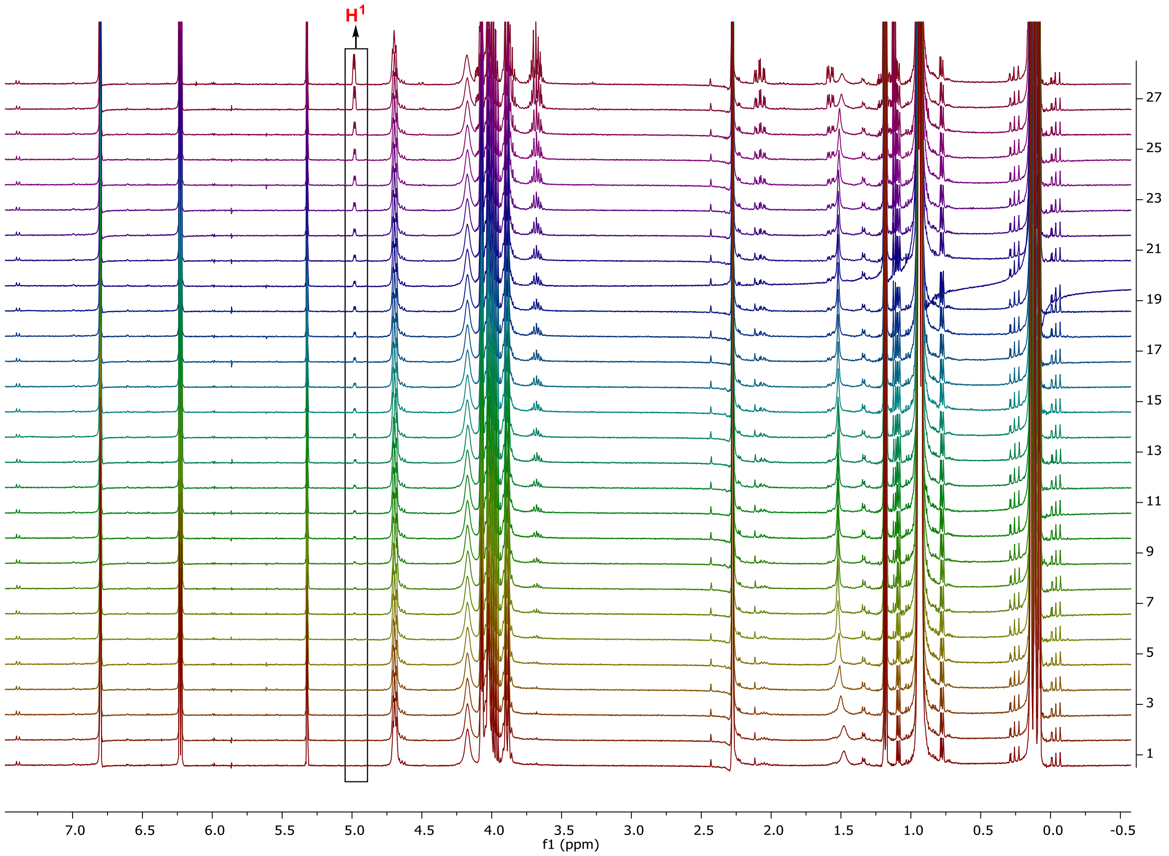

Supplementary figure S66: Stacked ^{1}H spectra for the experiment of entry 5 in supplementary table S17

Supplementary table S19. Concentration for **3al** calculated by ^{1}H NMR analysis for the experiments of entry 1 and entries 4-5 in supplementary table S17

Entry	0.2 M Donor **1c**		0.3 M Donor **1c**		0.4 M Donor **1c**	
	Time/min	[**3al**]	Time/min	[**3al**]	Time/min	[**3al**]
1	0	0	0	0	0	0
2	20	0	25	0	30	0
3	50	0	55	0	60	0
4	80	0	85	0	90	0
5	110	0	115	0	120	0
6	140	0	145	0	150	0
7	170	0	175	0	180	0.00172
8	200	0	205	0.00043	200	0.00172
9	220	0.00086	225	0.00043	215	0.00086
10	240	0.00129	245	0.00129	235	0.00086
11	260	0.00216	265	0.00129	255	0.00345
12	275	0.00172	280	0.00388	275	0.00474
13	290	0.00431	295	0.00604	290	0.00647
14	305	0.00517	310	0.00733	305	0.00733
15	320	0.00561	325	0.00862	320	0.00862
16	335	0.00733	340	0.01164	335	0.00949
17	350	0.00647	355	0.01337	350	0.01035
18	365	0.00819	370	0.01337	365	0.01121
19	380	0.01035	385	0.0138	380	0.01207
20	400	0.01121	405	0.02027	400	0.0125
21	420	0.0138	425	0.02328	420	0.01595
22	440	0.01725	445	0.02673	440	0.01897
23	460	0.02027	465	0.03234	460	0.02199
24	480	0.02458	485	0.03924	480	0.0263
25	500	0.02932	505	0.04743	500	0.03148
26	520	0.03363	525	0.05649	520	0.03665
27	585	0.05951	590	0.10737	590	0.06985
28	615	0.07072	620	0.13971	620	0.08796

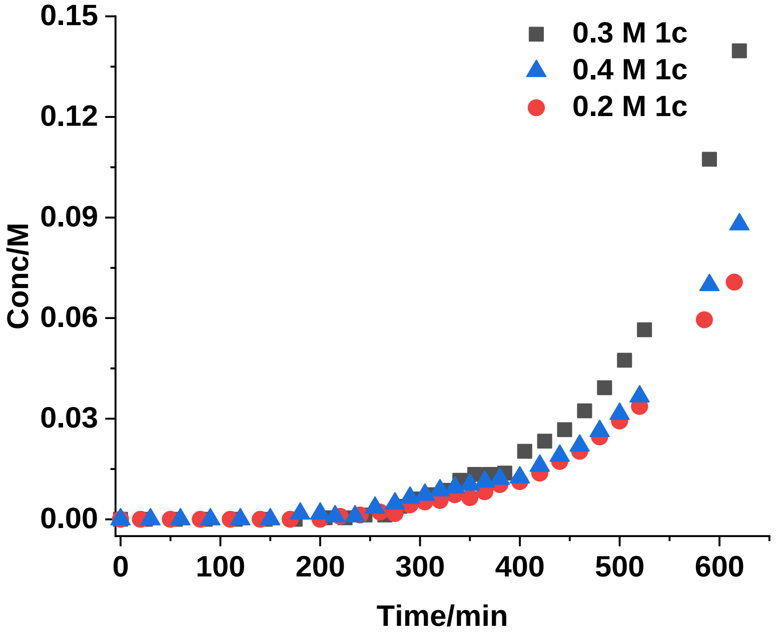

Supplementary figure S67: Overlapped profile for the donor concentration dependence experiments

3. Acceptor concentration dependence (Supplementary table S17, entries 1 and entries 6-8)

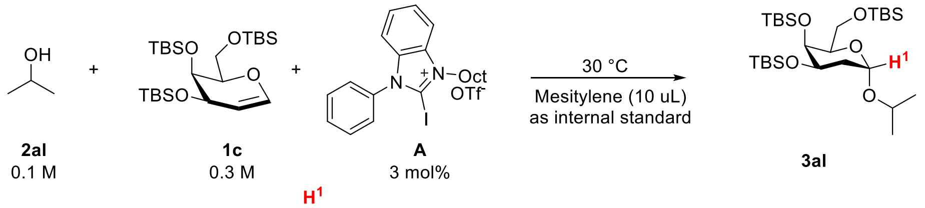

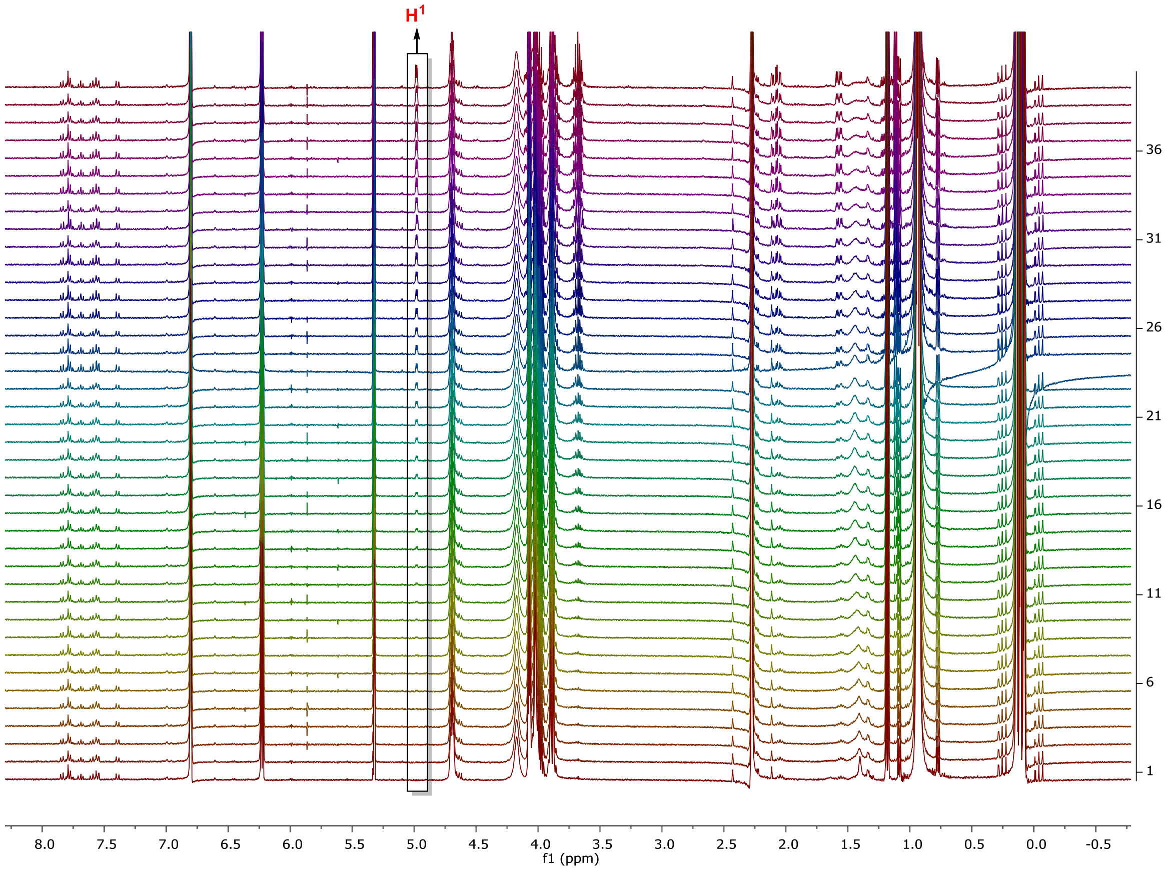

Supplementary figure S68: Stacked ^{1}H spectra for the experiment of entry 6 in supplementary table S17

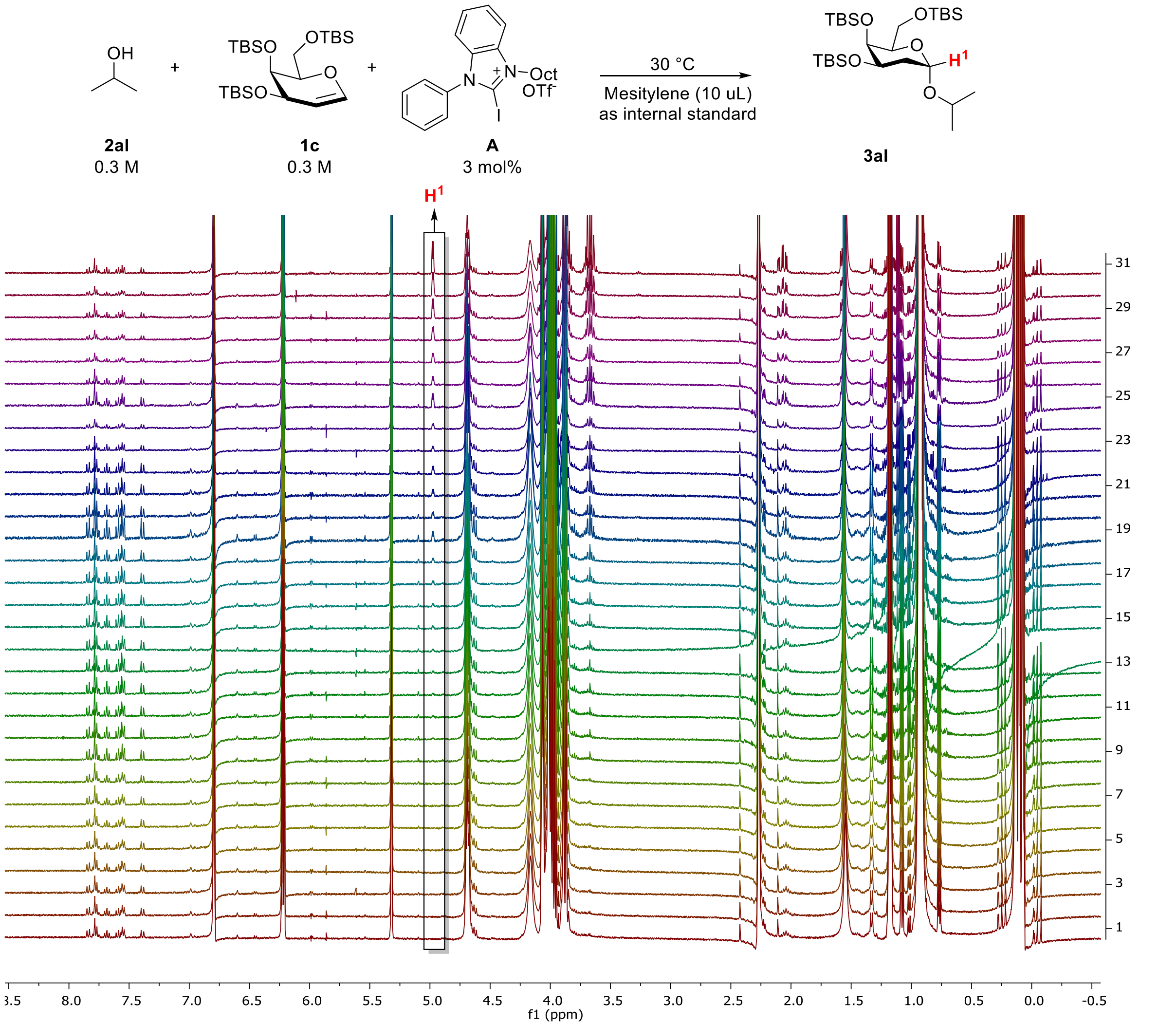

Supplementary figure S69: Stacked ^{1}H spectra for the experiment of entry 7 in supplementary table S17

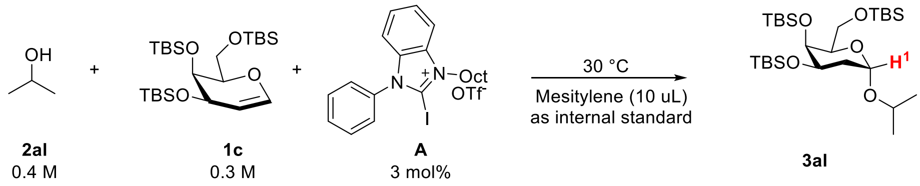

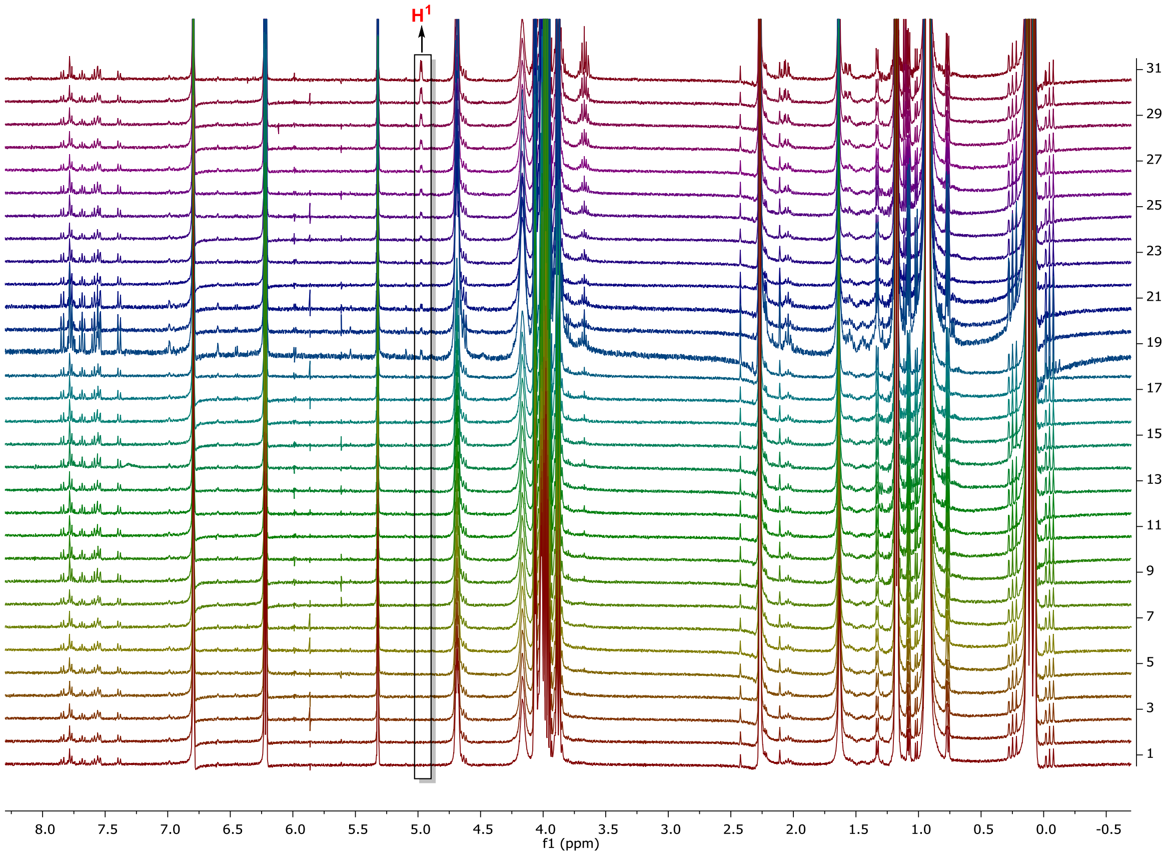

Supplementary figure S70: Stacked ^{1}H spectra for the experiment of entry 8 in supplementary table S17

Supplementary table S20. Concentration for **3al** calculated by ^{1}H NMR analysis for the experiments of entry 1 and entries 6-8 in supplementary table S17

Entry	0.1 M **2al**		0.2 M **2al**		0.3 M **2al**		0.4 M **2al**	
	Time/min	[**3al**]	Time/min	[**3al**]	Time/min	[**3al**]	Time/min	[**3al**]
1	0	0	0	0	0	0	0	0
2	60	0	25	0	42	0	34	0
3	120	0	55	0	58	0	50	0
4	135	0	85	0	74	0	66	0
5	150	0	115	0	90	0	82	0
6	165	0	145	0	105	0	97	0
7	180	0	175	0	120	0	112	0
8	210	0	205	0.00043	135	0	127	0
9	225	0	225	0.00043	155	0	147	0
10	240	0	245	0.00129	171	0	163	0
11	270	0	265	0.00129	186	0	178	0
12	292	0	280	0.00388	202	0	194	0
13	307	0.00043	295	0.00604	219	0	211	0
14	322	0.00173	310	0.00733	247	0	229	0
15	337	0.00216	325	0.00862	263	0	245	0
16	352	0.00345	340	0.01164	278	0.00043	260	0
17	367	0.00388	355	0.01337	298	0.00129	280	0
18	382	0.00388	370	0.01337	321	0.00129	301	0
19	397	0.00518	385	0.0138	341	0.00259	327	0
20	412	0.00561	405	0.02027	361	0.00345	347	0
21	427	0.0069	425	0.02328	381	0.00431	367	0
22	442	0.00863	445	0.02673	411	0.00431	397	0.00043
23	457	0.00949	465	0.03234	437	0.0069	425	0.00086
24	472	0.01121	485	0.03924	467	0.00906	455	0.00216
25	487	0.01208	505	0.04743	499	0.01165	487	0.00216
26	502	0.0138	525	0.05649	529	0.0138	517	0.00345
27	517	0.0151	590	0.10737	559	0.02027	547	0.00474
28	532	0.01639	620	0.13971	619	0.02717	607	0.00776
29	547	0.01812			679	0.03752	667	0.01337
30	562	0.01941			739	0.0509	727	0.01768
31	577	0.02113			799	0.06383	787	0.02243
32	592	0.02243						
33	607	0.02459						
34	622	0.02588						
35	637	0.02804						
36	652	0.0289						
37	667	0.03192						
38	682	0.03364						
39	697	0.03451						
40	712	0.03623						

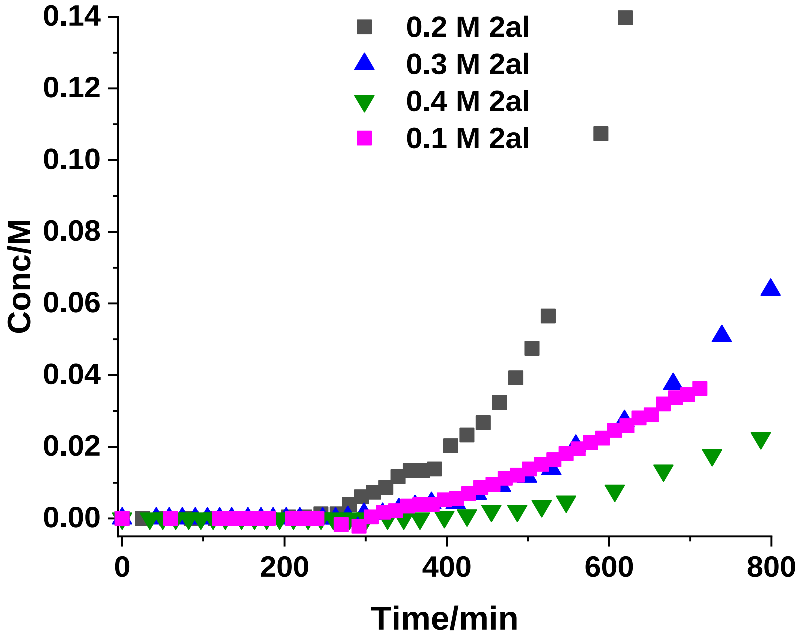

Supplementary figure S71: Overlapped profile for the acceptor concentration dependence experiments

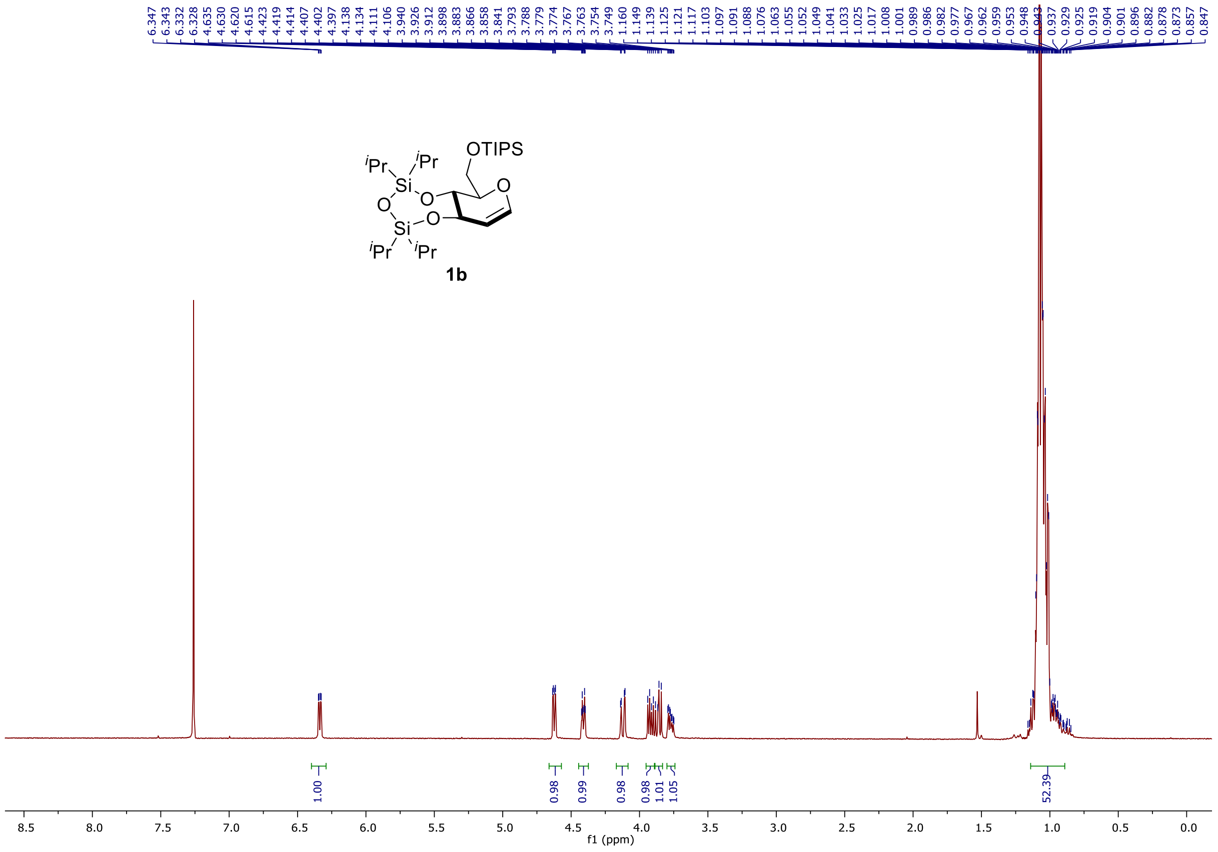

Supplementary figure S72: ^{1}H spectra for **1b**

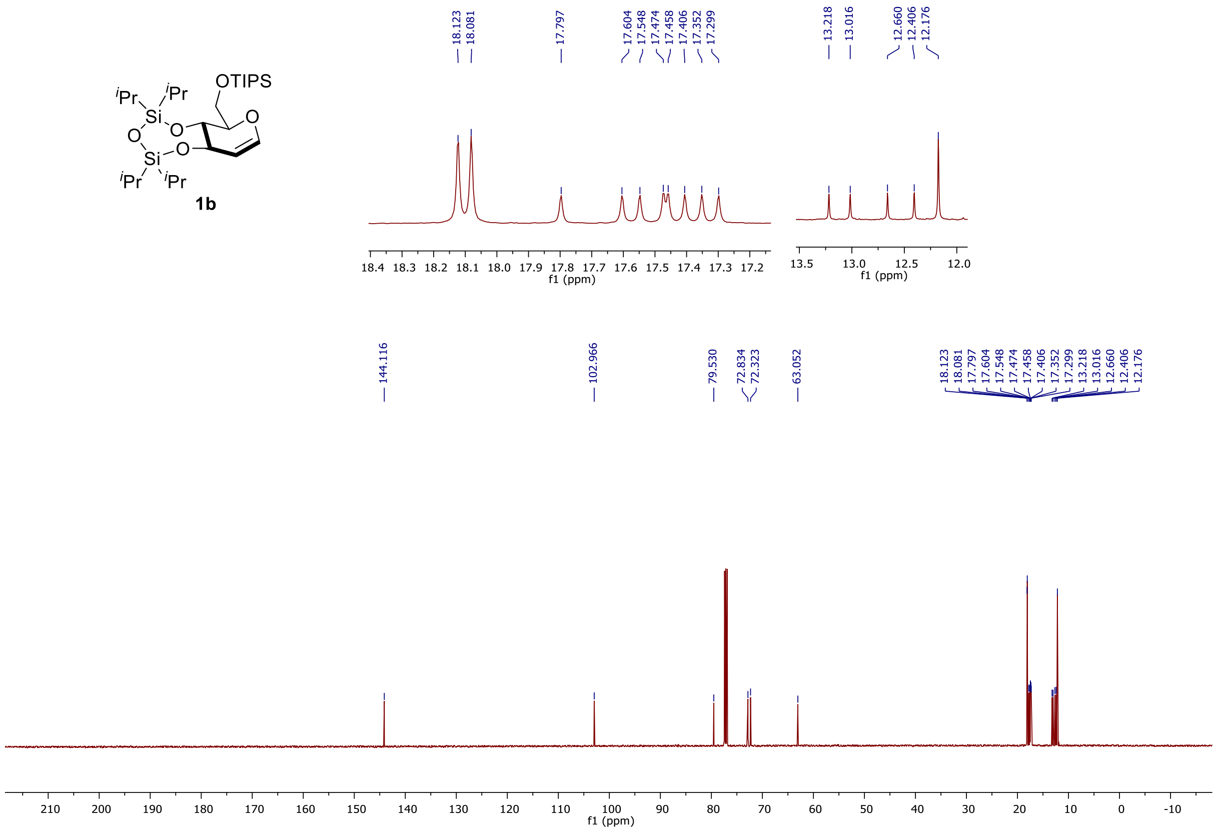

Supplementary figure S73: ^{13}C spectra for **1b**

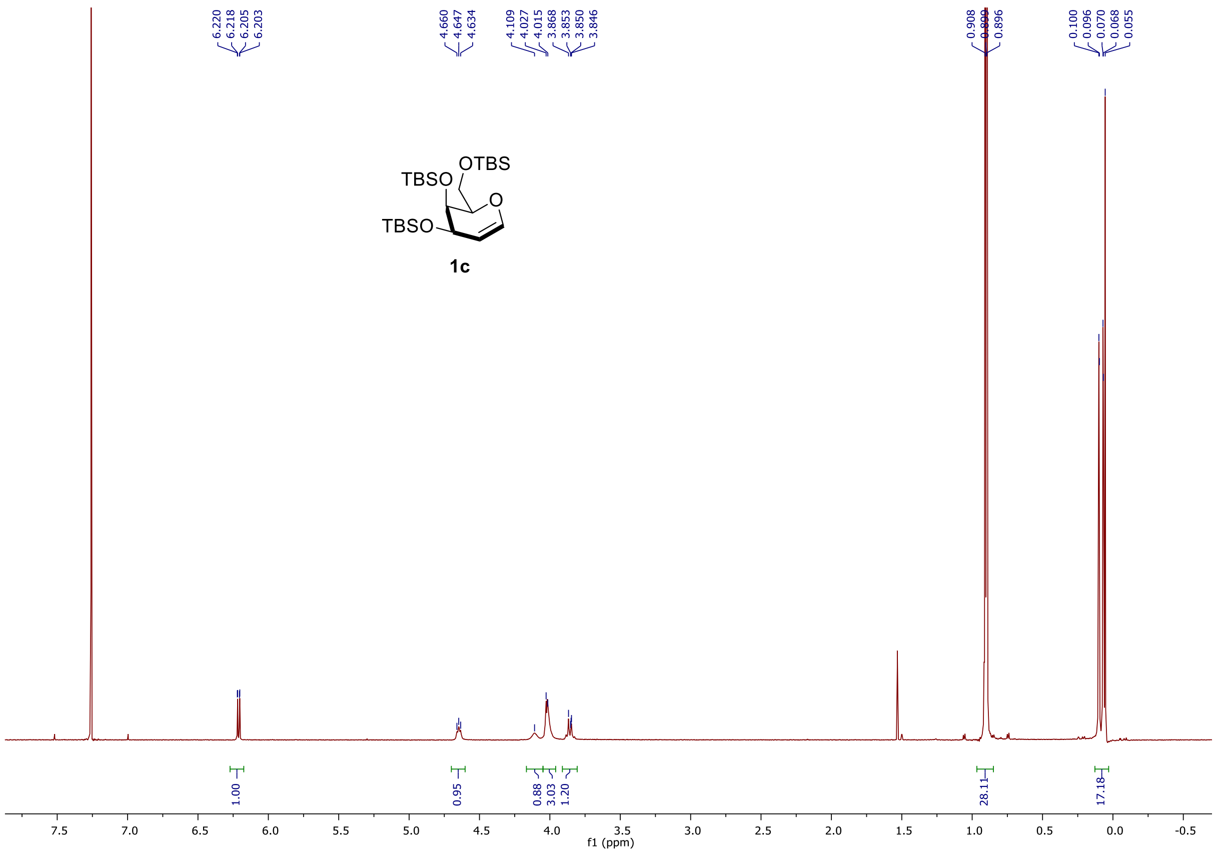

Supplementary figure S74: ^{1}H spectra for **1c**

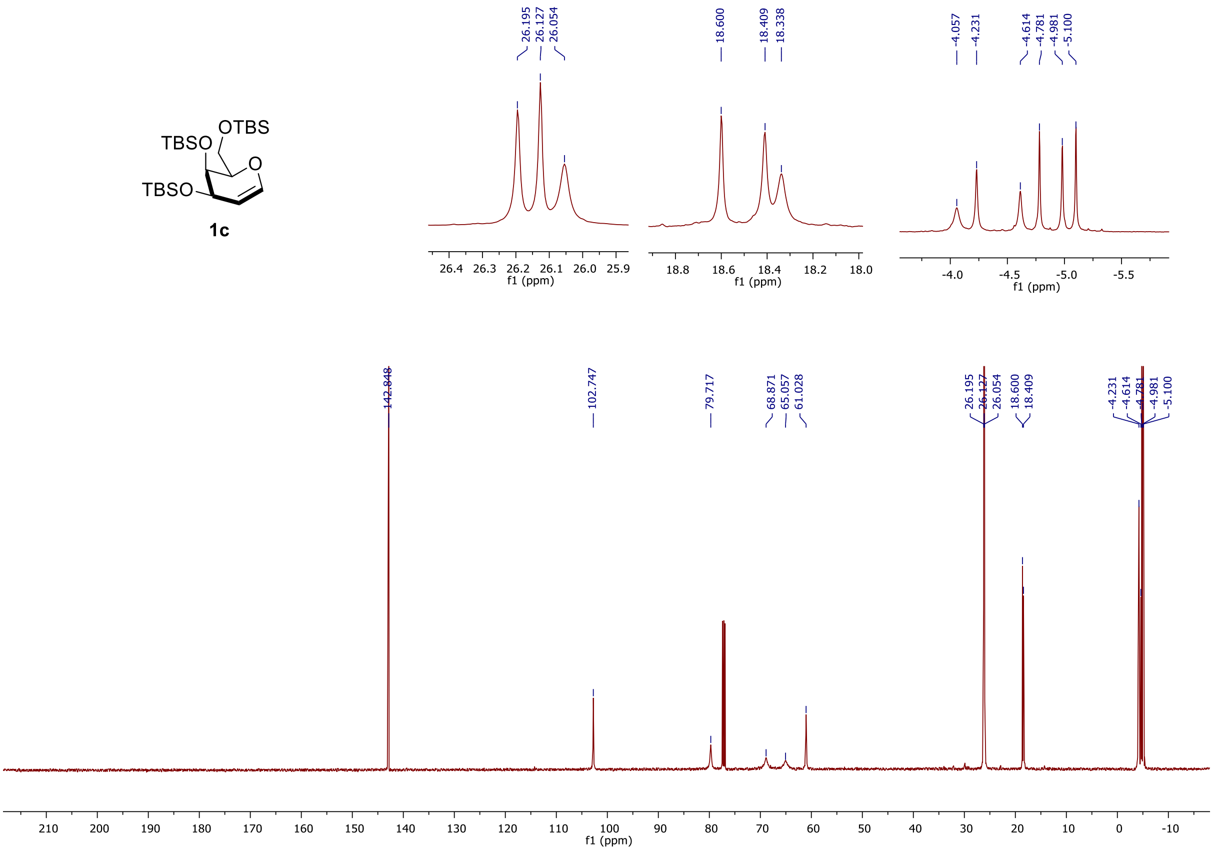

Supplementary figure S75: ^{13}C spectra for **1c**

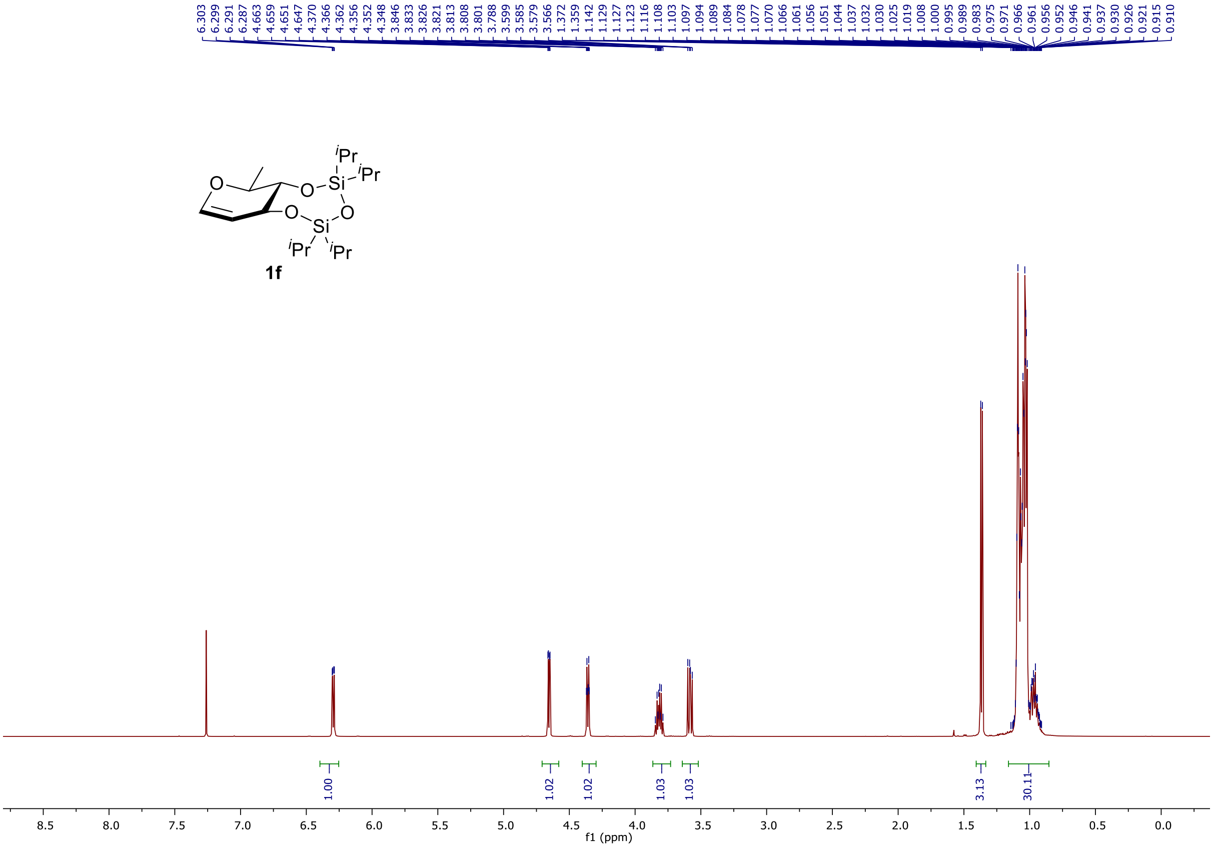

Supplementary figure S76: ^{1}H spectra for **1f**

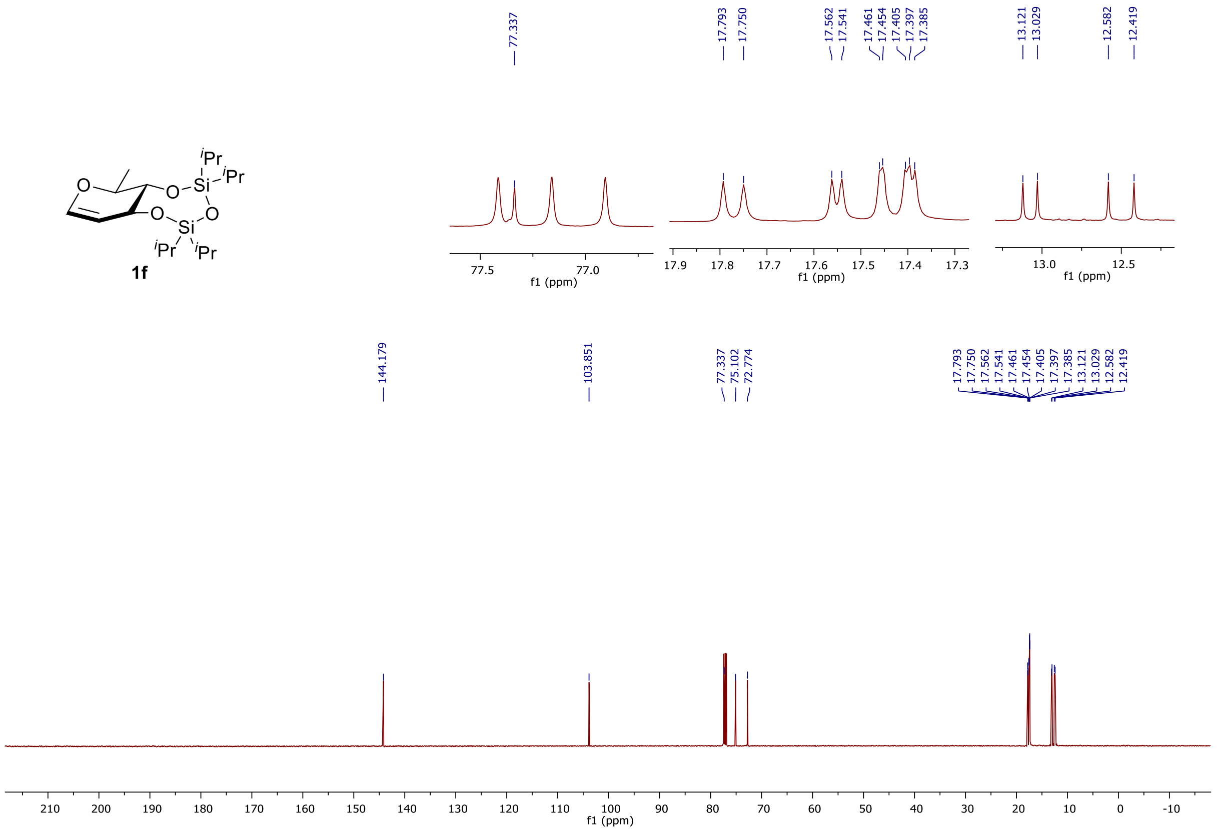

Supplementary figure S77: ^{13}C spectra for **1f**

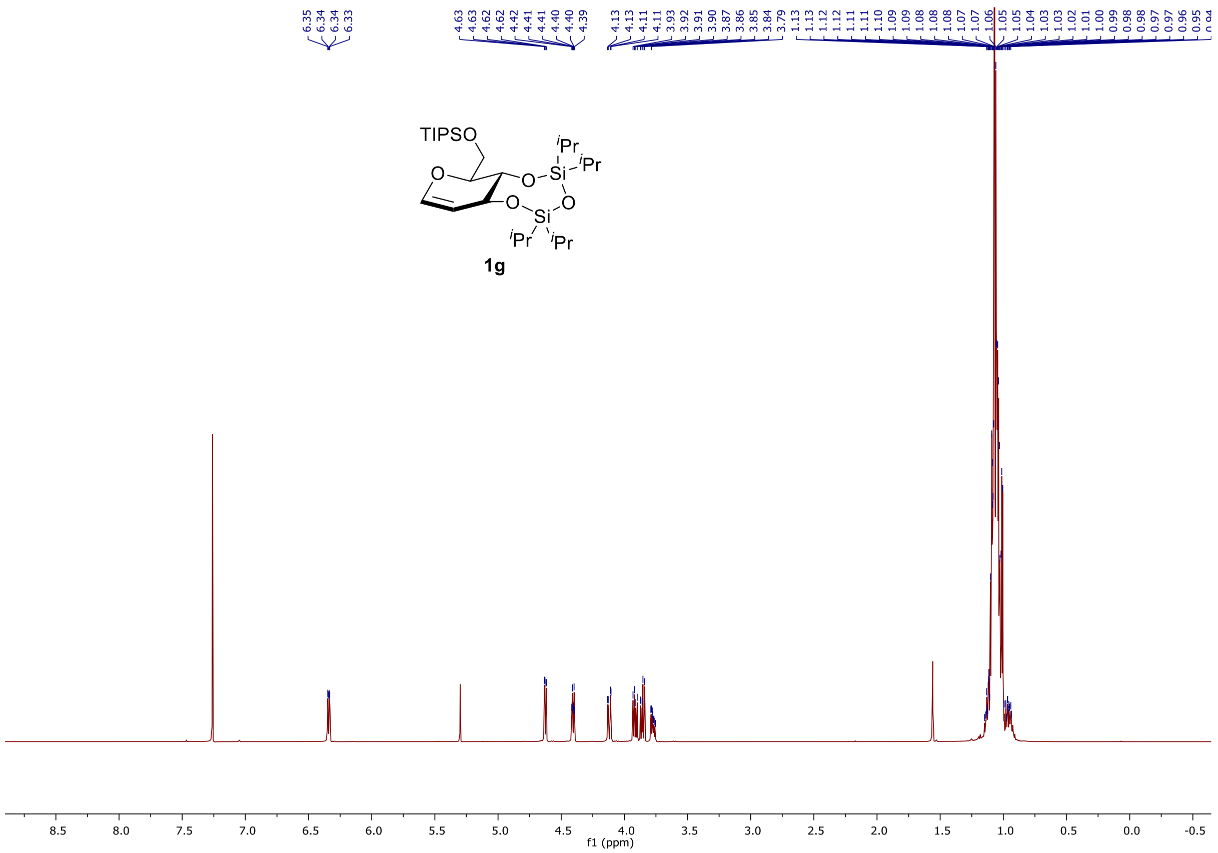

Supplementary figure S78: ^{1}H spectra for **1g**

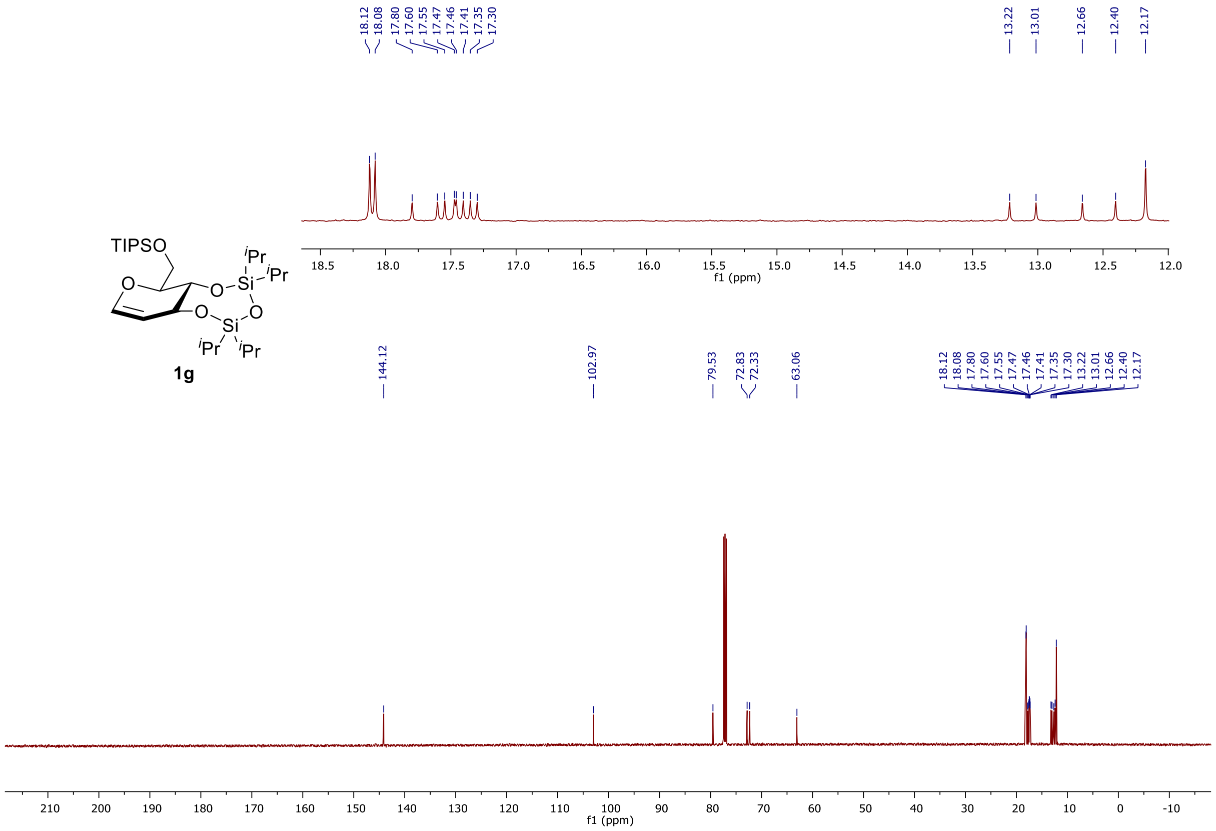

Supplementary figure S79: ^{13}C spectra for **1g**

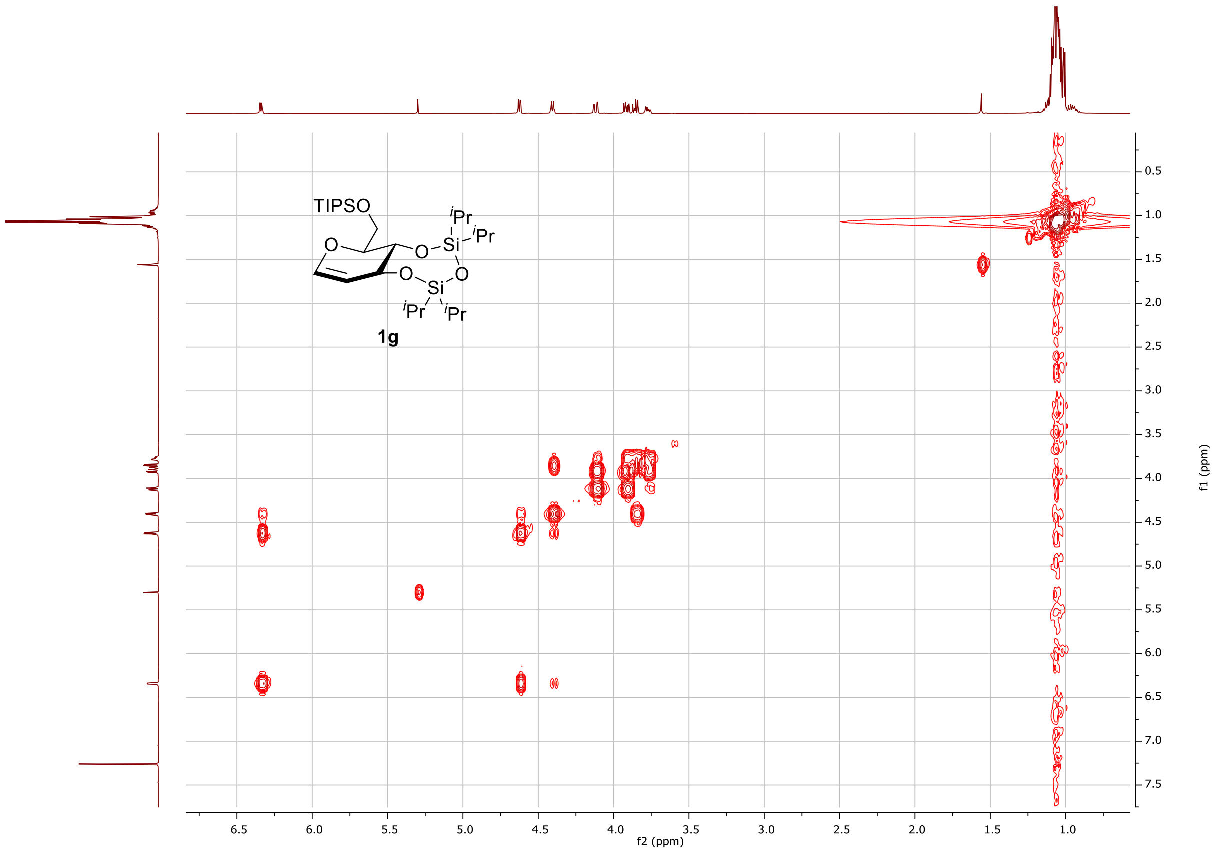

Supplementary figure S80: COSY spectra for **1g**

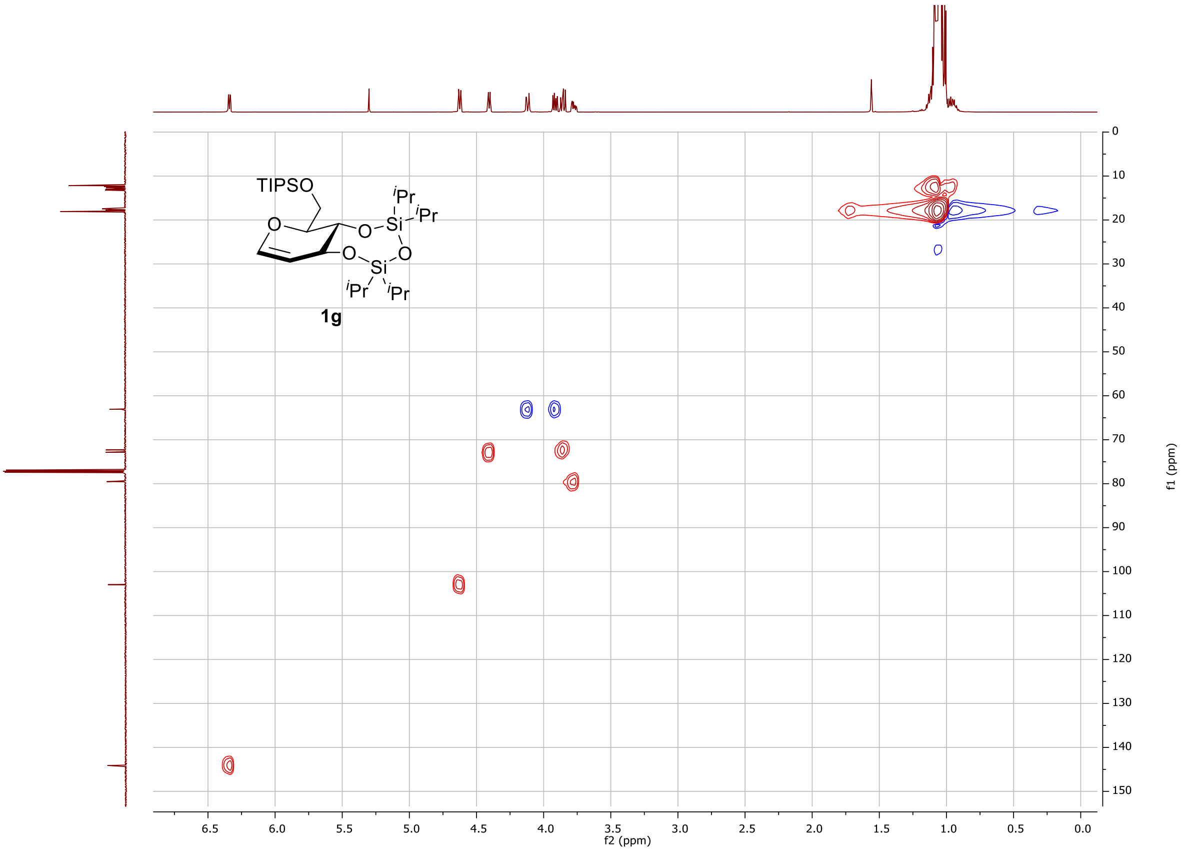

Supplementary figure S81: HSQC spectra for **1g**

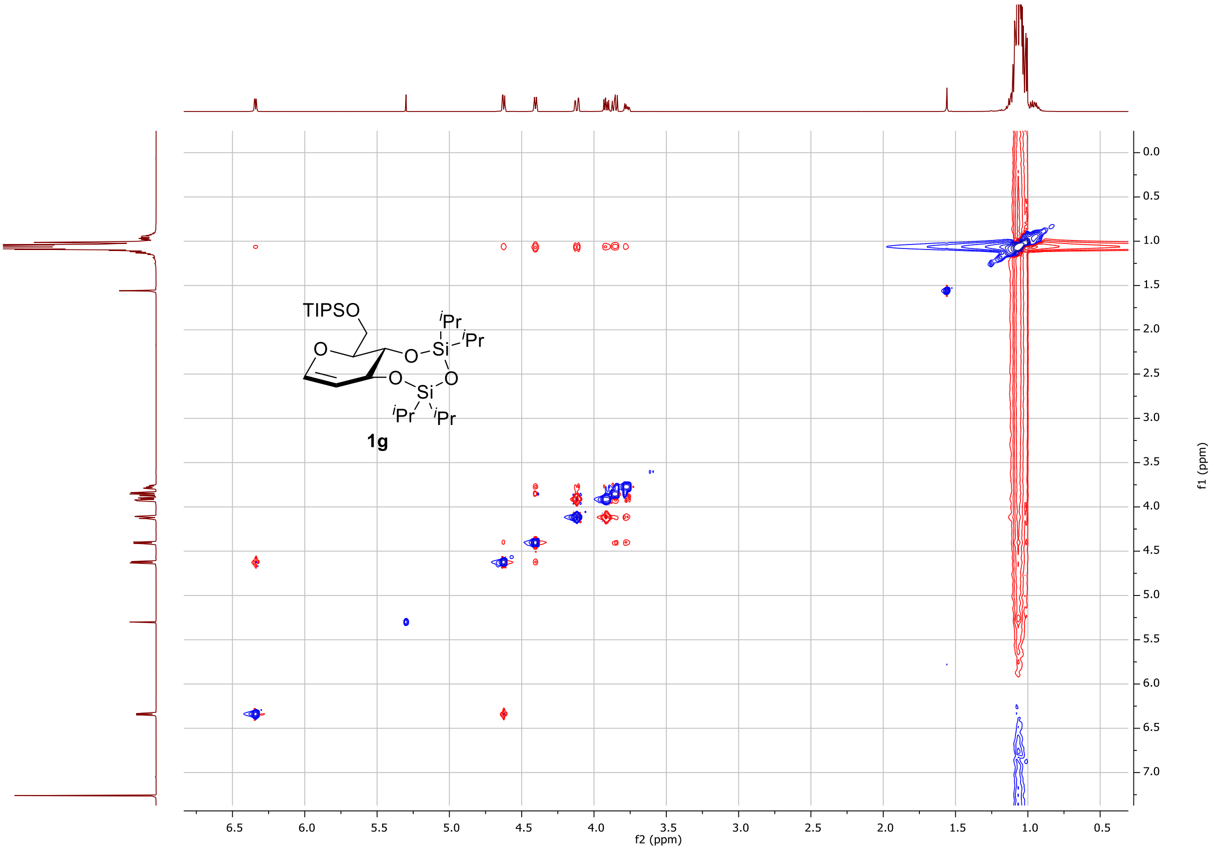

Supplementary figure S82: NOESY spectra for **1g**

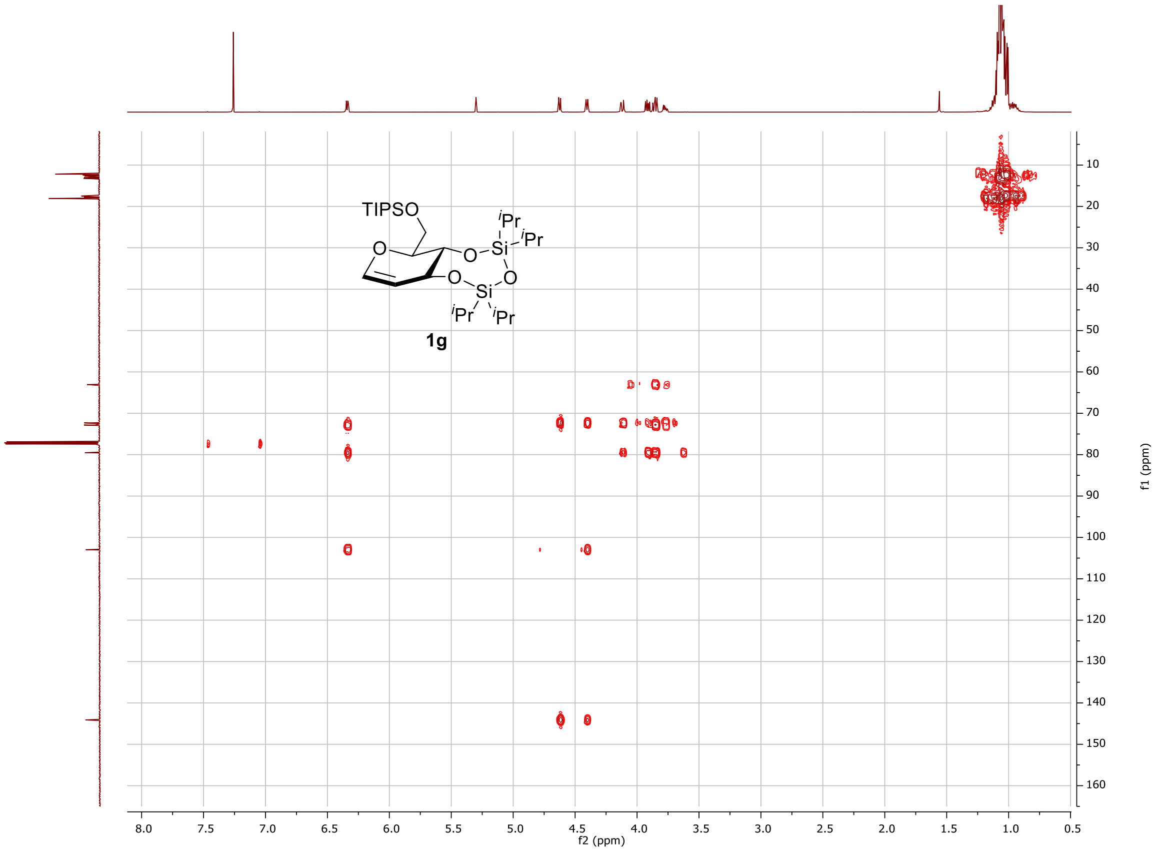

Supplementary figure S83: HMBC spectra for **1g**

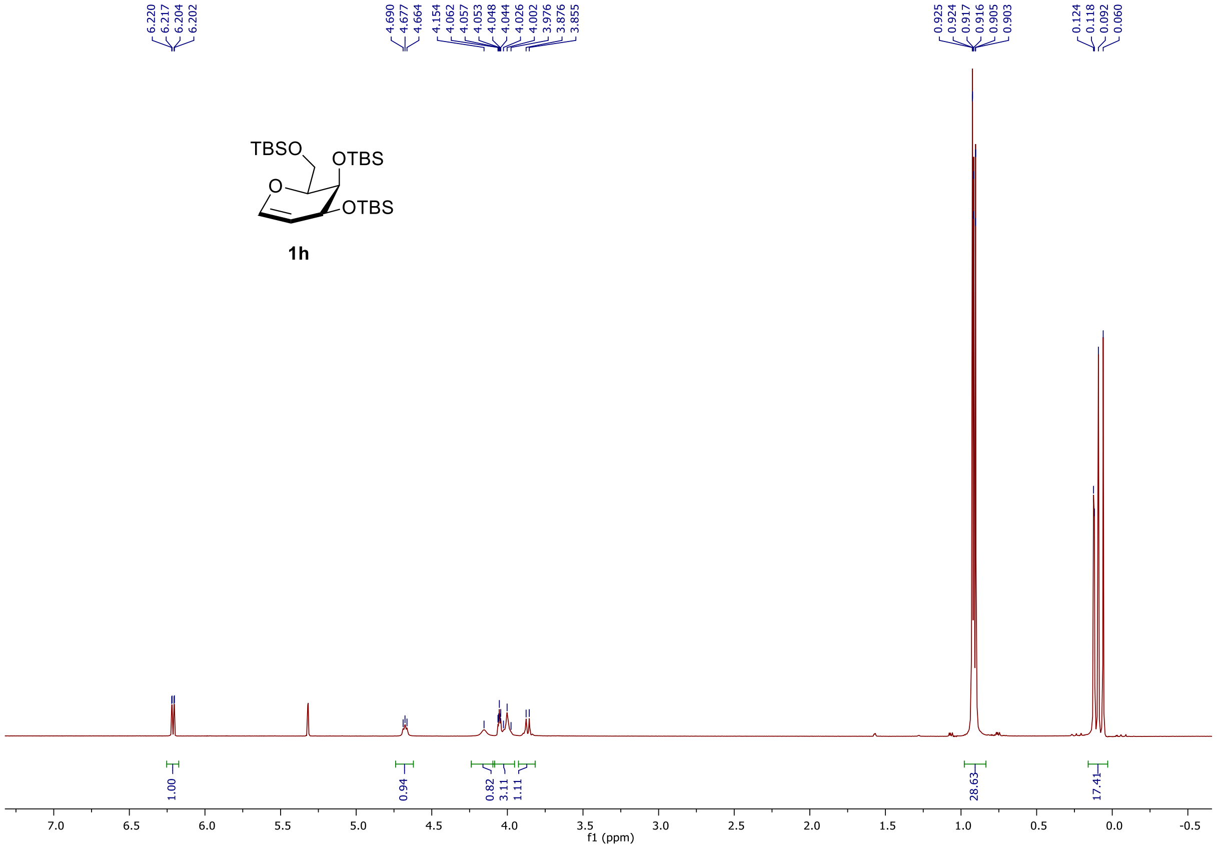

Supplementary figure S84: ^{1}H spectra for **1h**

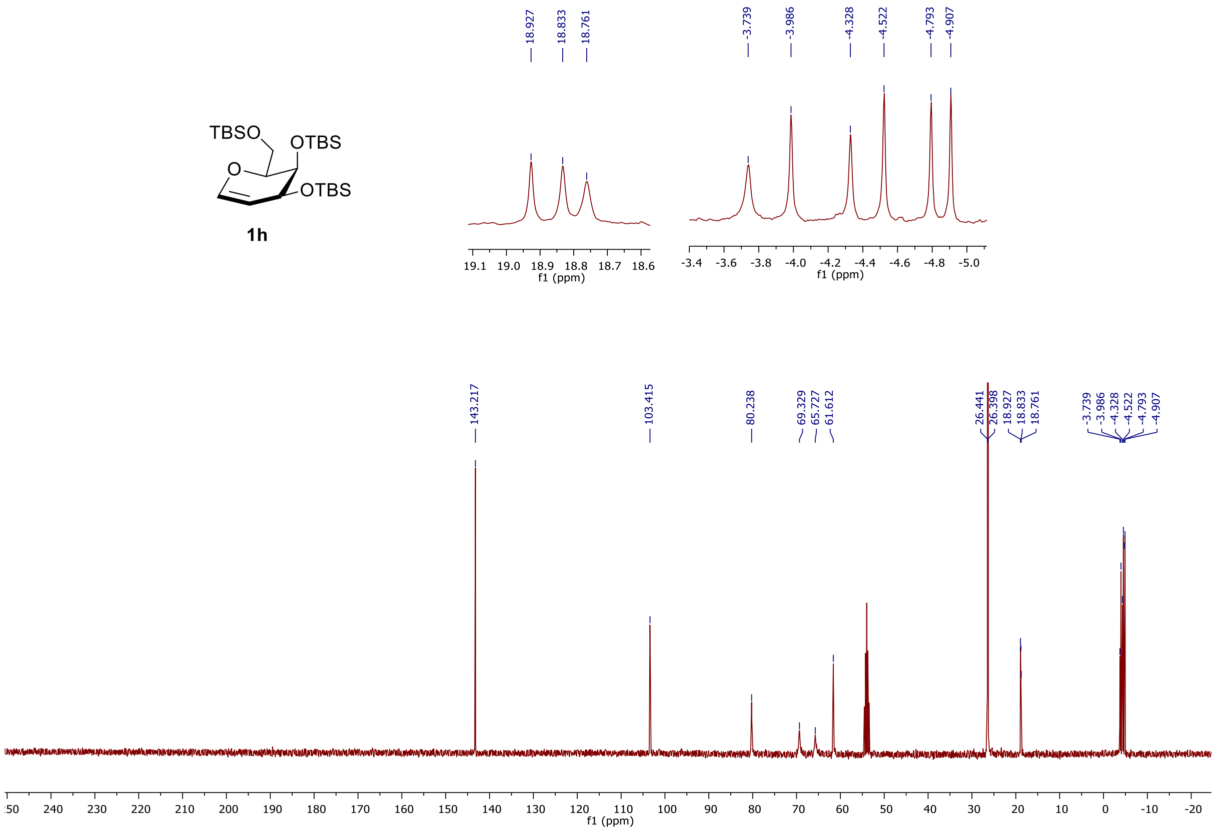

Supplementary figure S85: ^{13}C spectra for **1h**

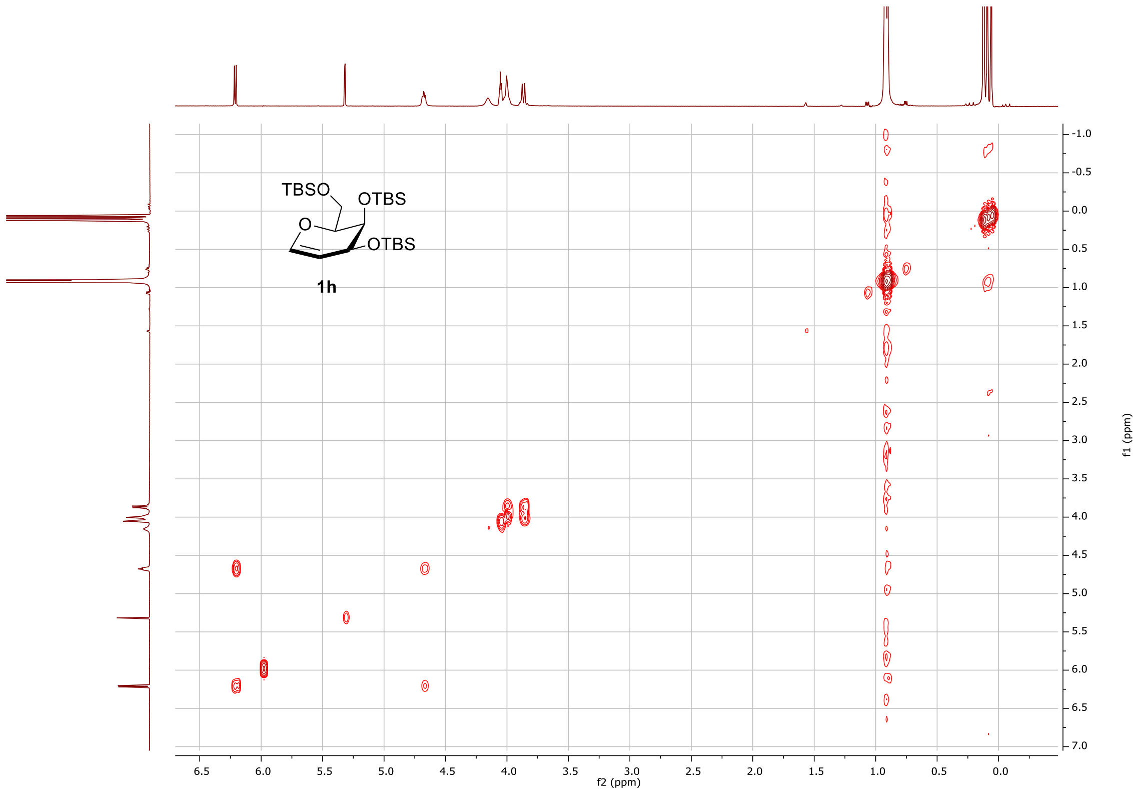

Supplementary figure S86: COSY spectra for **1h**

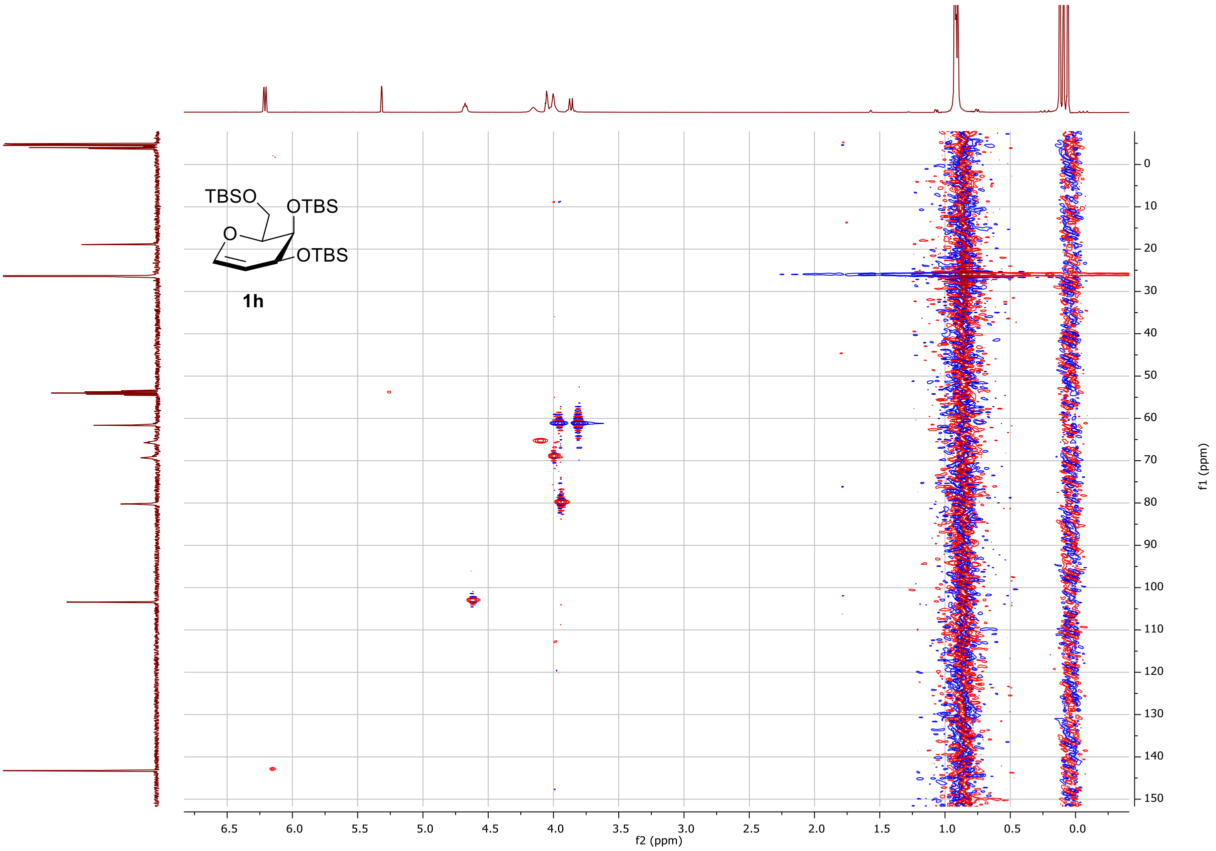

Supplementary figure S87: HSQC spectra for **1h**

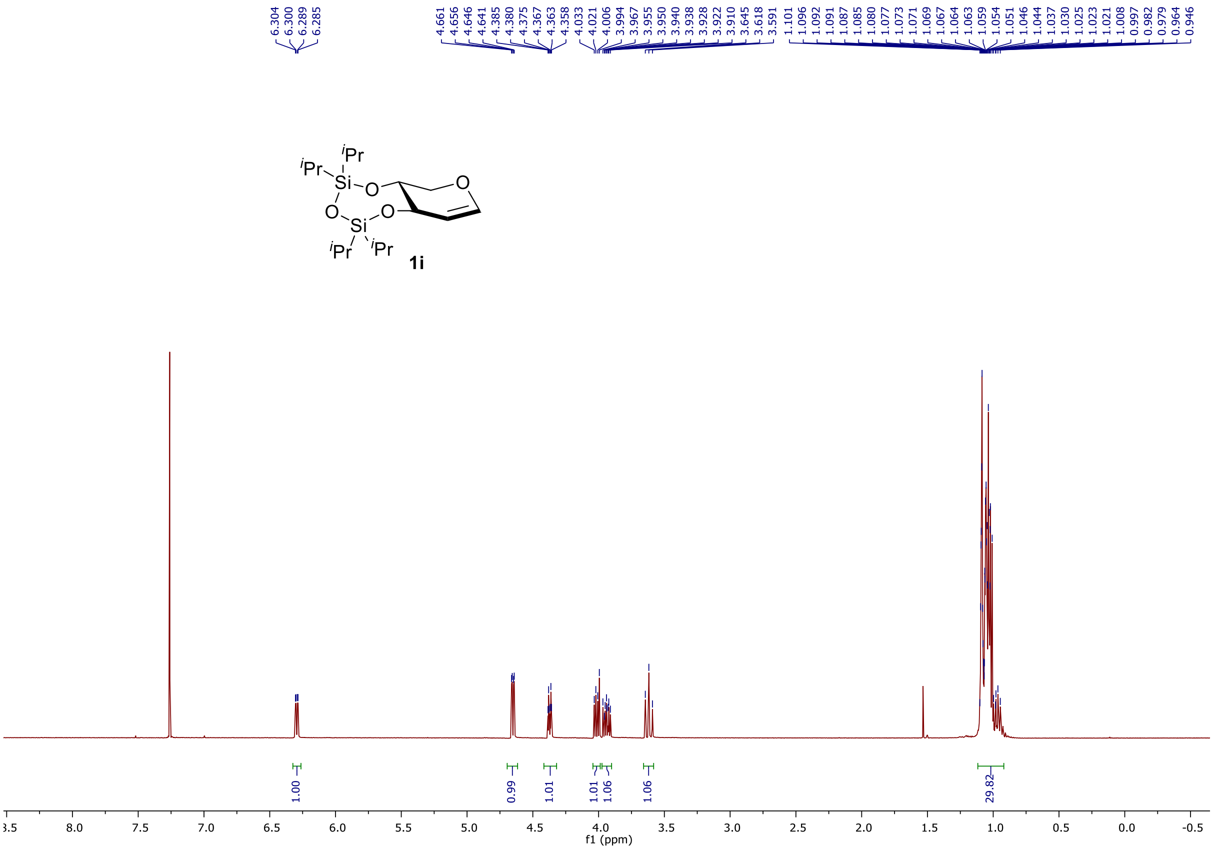

Supplementary figure S88: ^{1}H spectra for **1i**

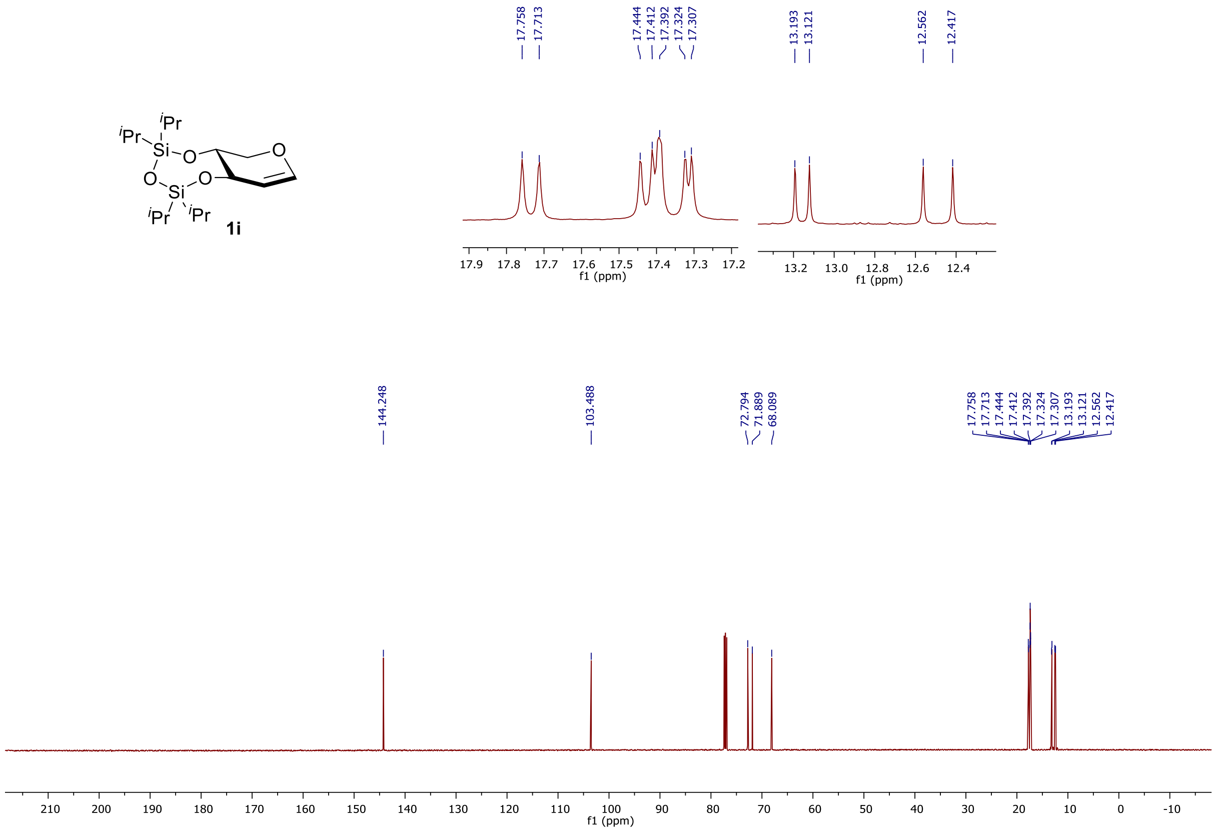

Supplementary figure S89: ^{13}C spectra for **1i**

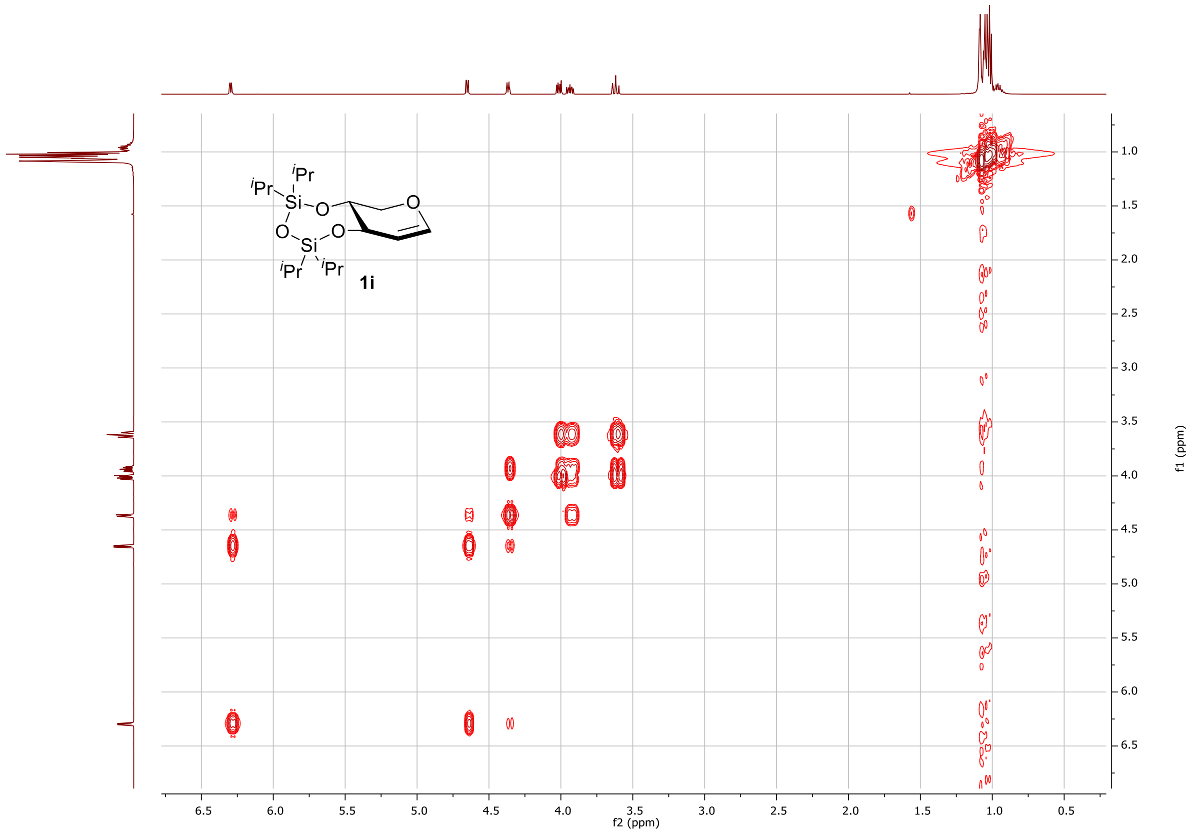

Supplementary figure S90: COSY spectra for **1i**

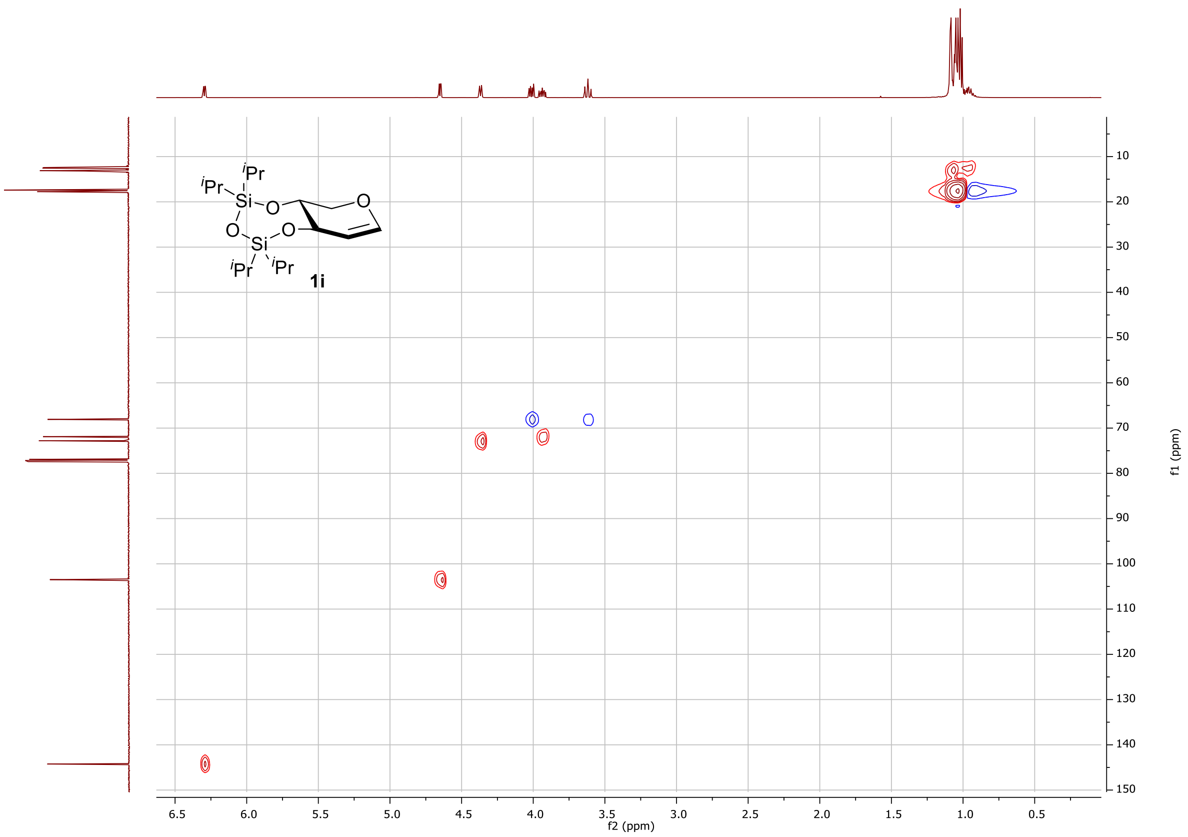

Supplementary figure S91: HSQC spectra for **1i**

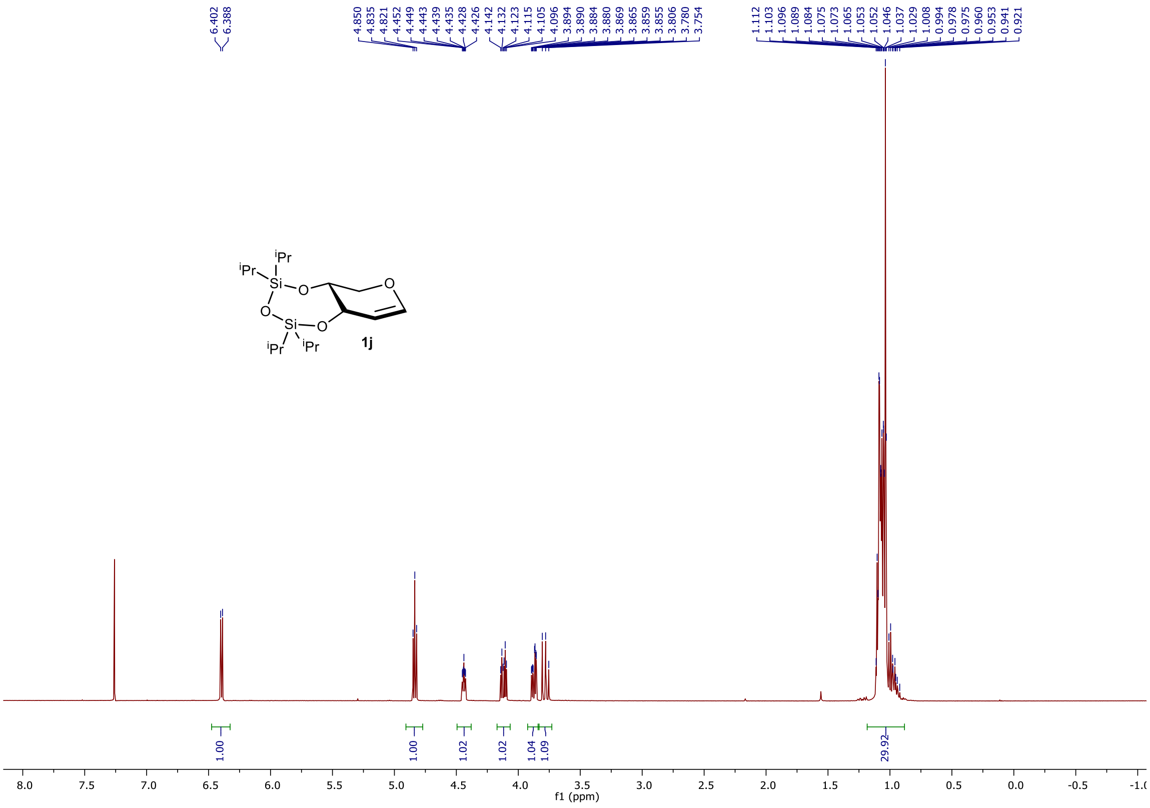

Supplementary figure S92: ^{1}H spectra for **1j**

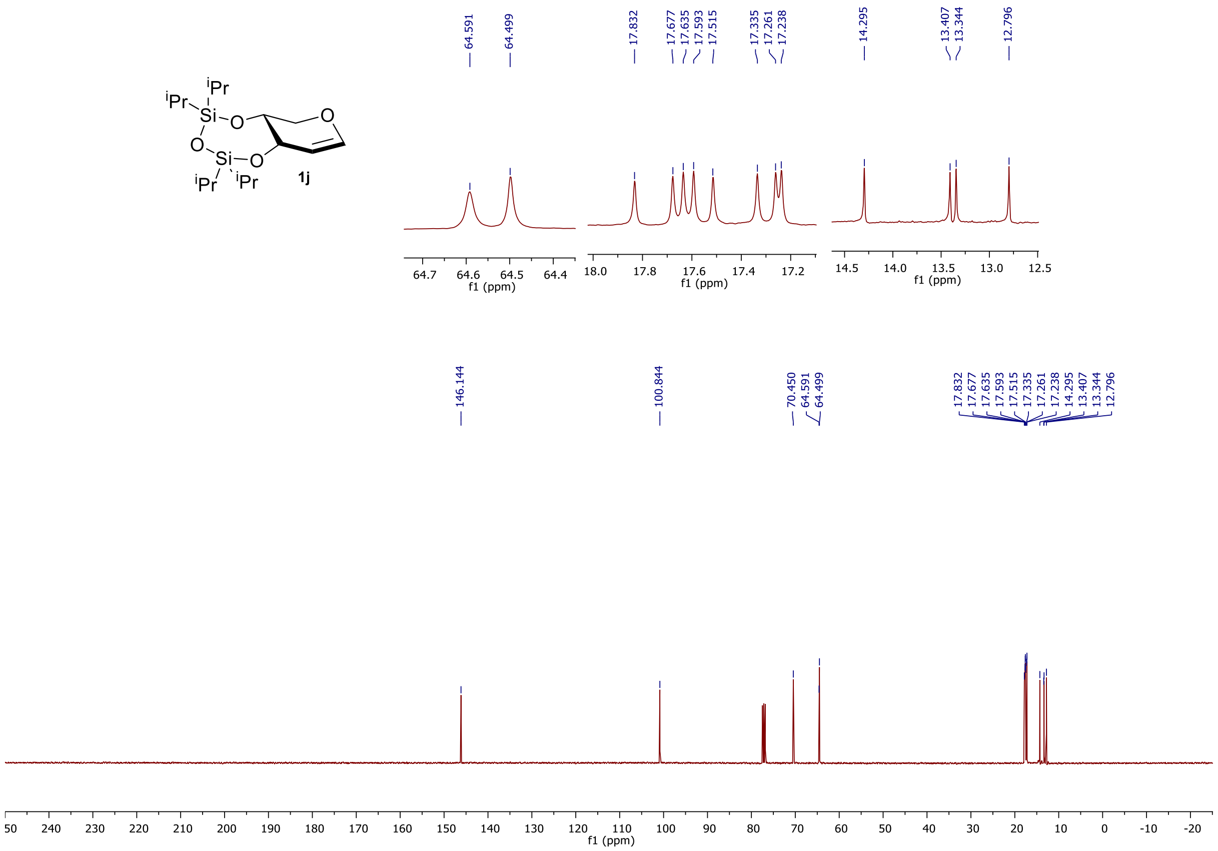

Supplementary figure S93: ^{13}C spectra for **1j**

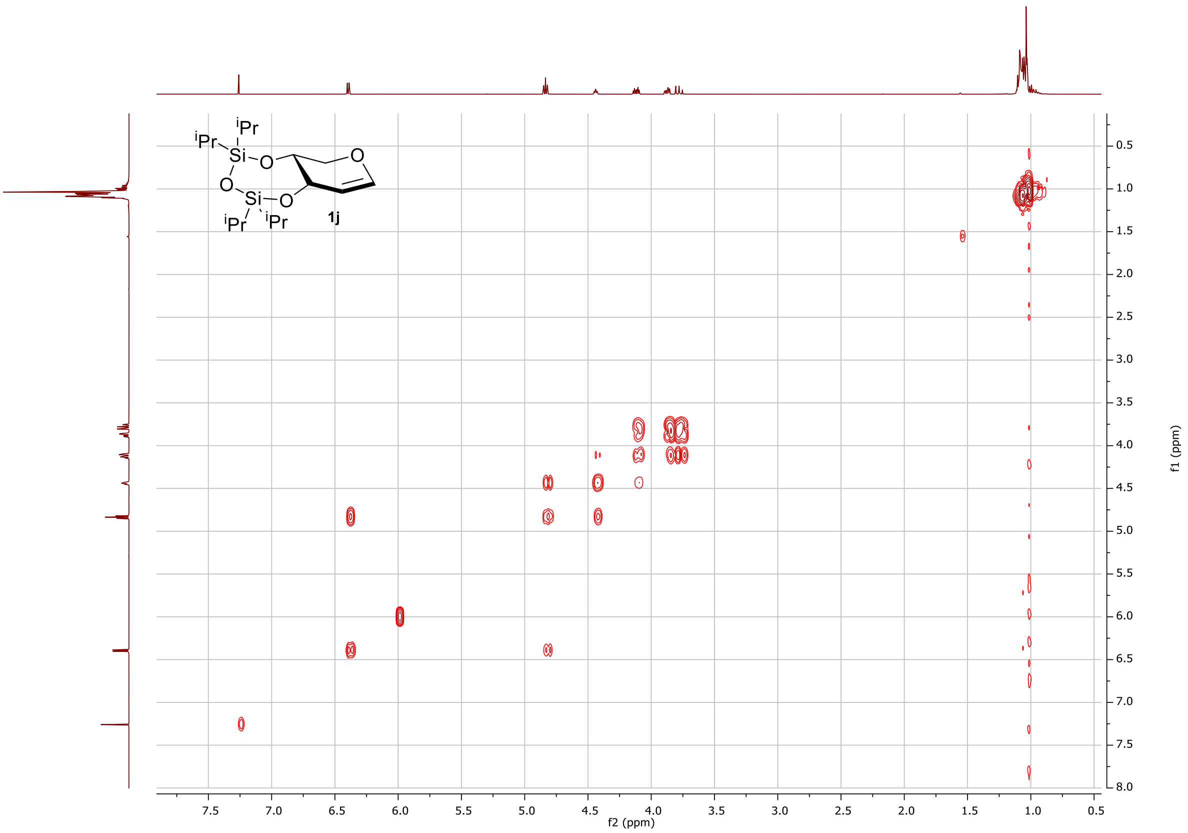

Supplementary figure S94: COSY spectra for **1j**

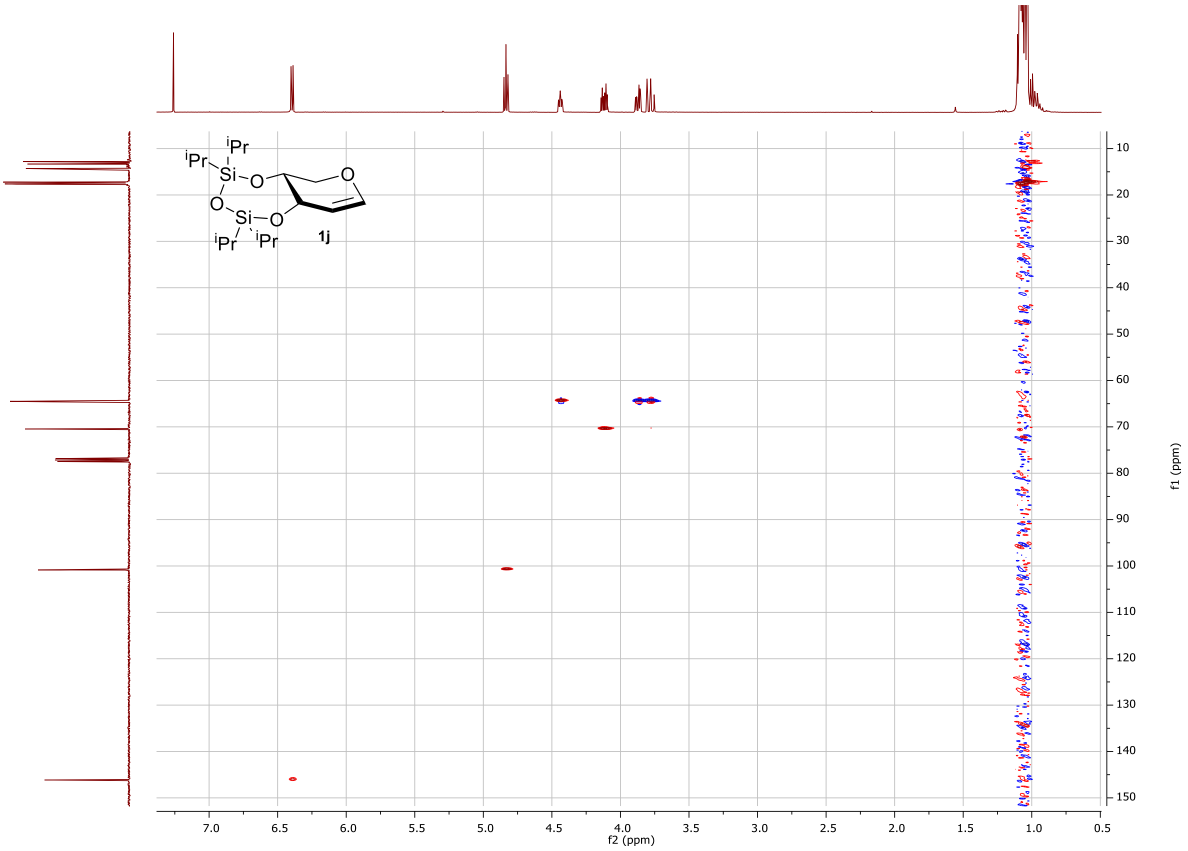

Supplementary figure S95: HSQC spectra for **1j**

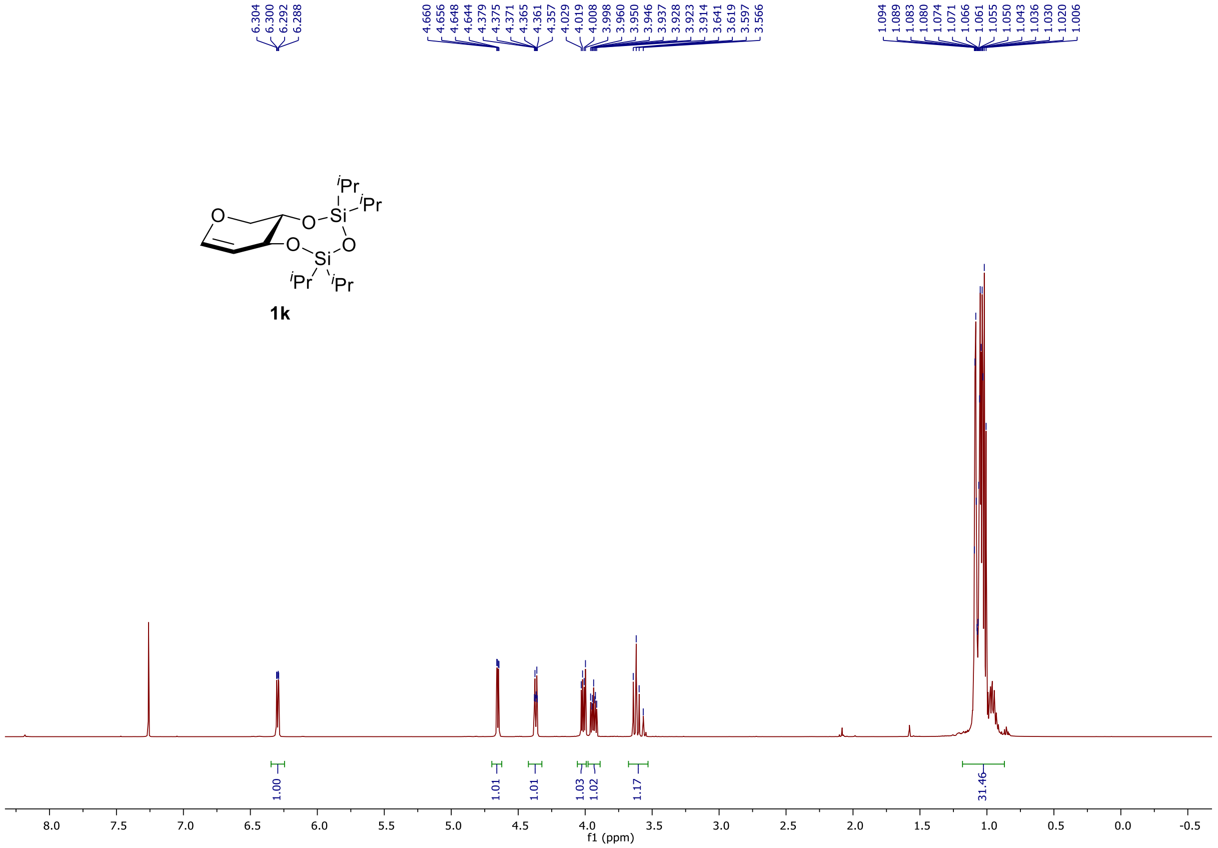

Supplementary figure S96: ^{1}H spectra for **1k**

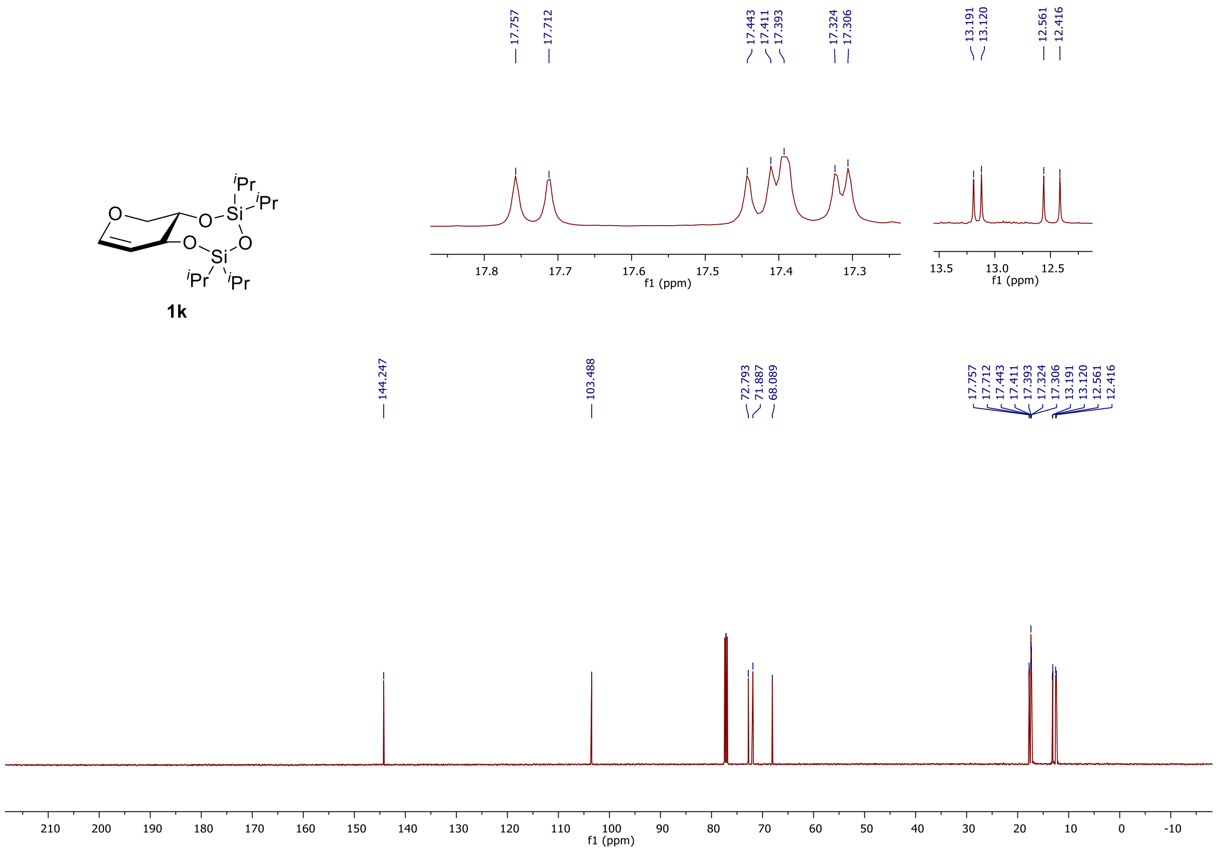

Supplementary figure S97: ^{13}C spectra for **1k**

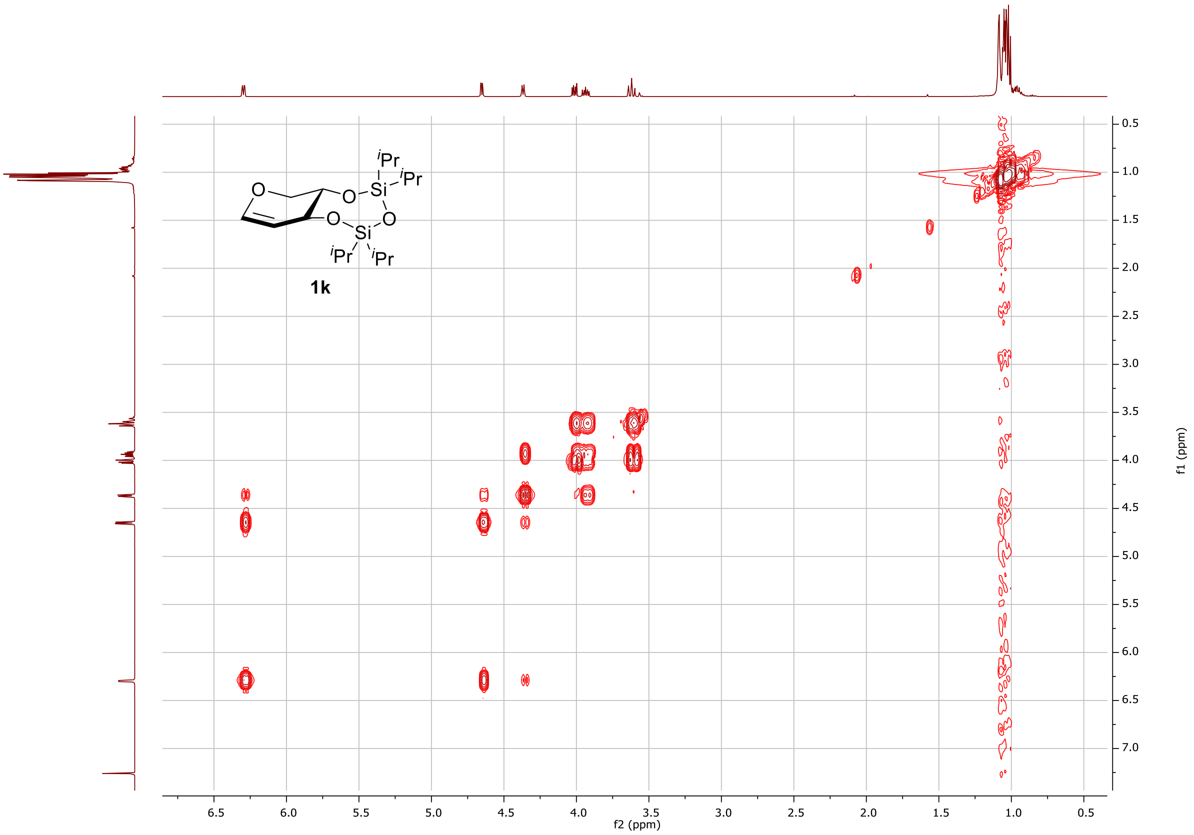

Supplementary figure S98: COSY spectra for **1k**

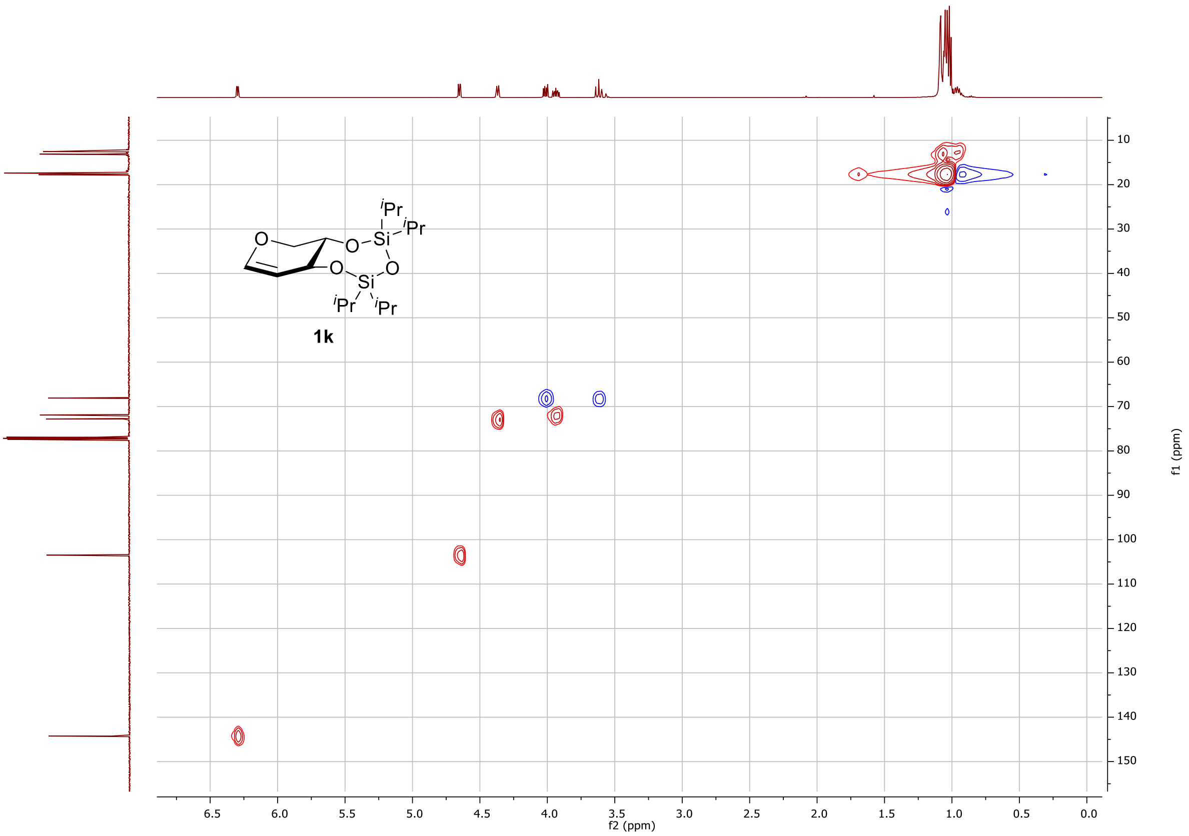

Supplementary figure S99: HSQC spectra for **1k**

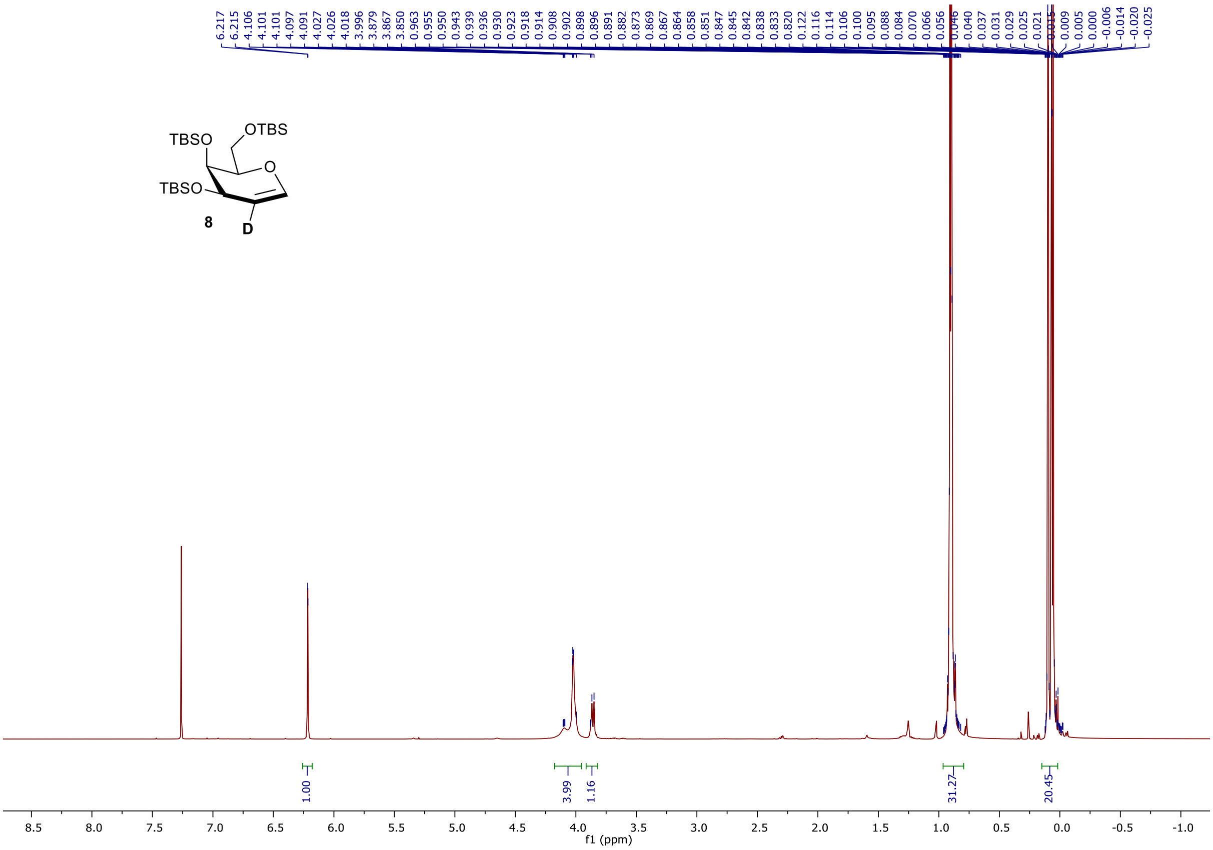

Supplementary figure S100: ^{1}H spectra for **8**

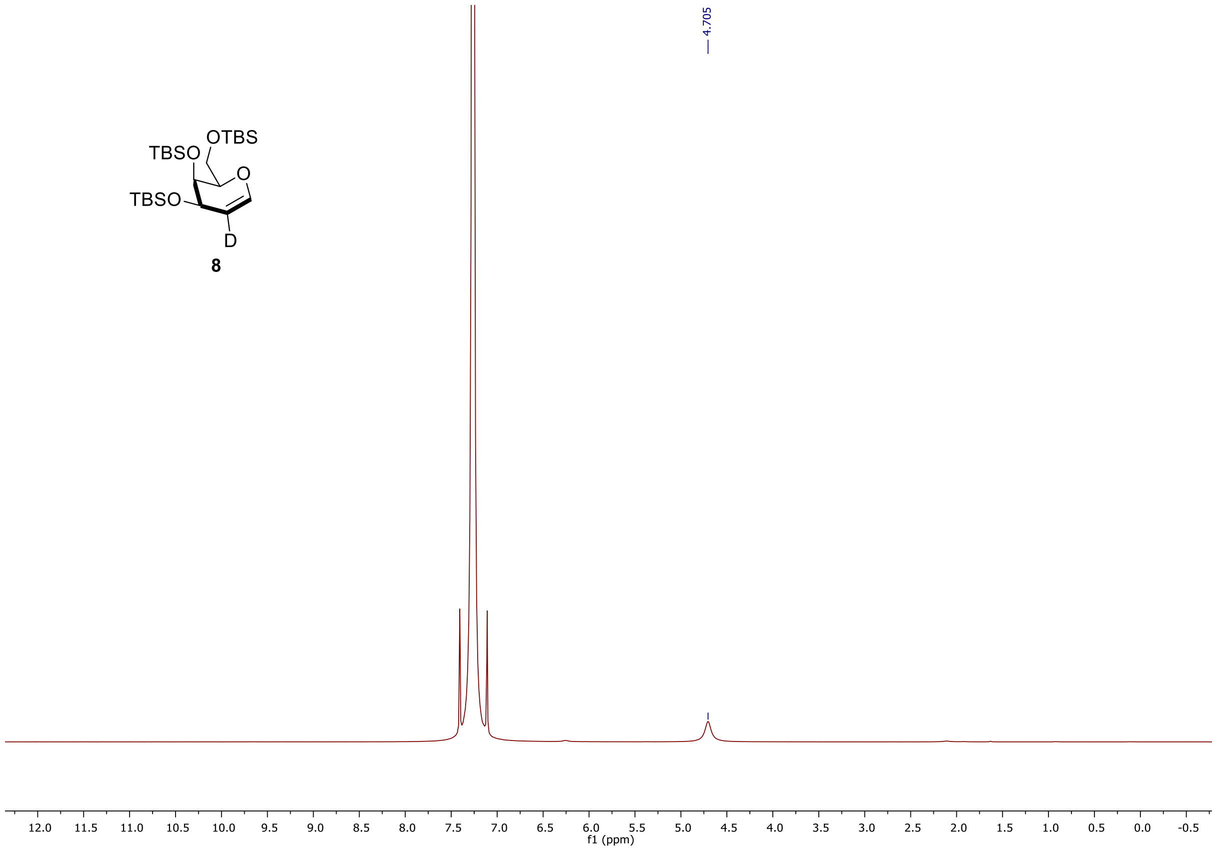

Supplementary figure S101: ^{2}H spectra for **8**

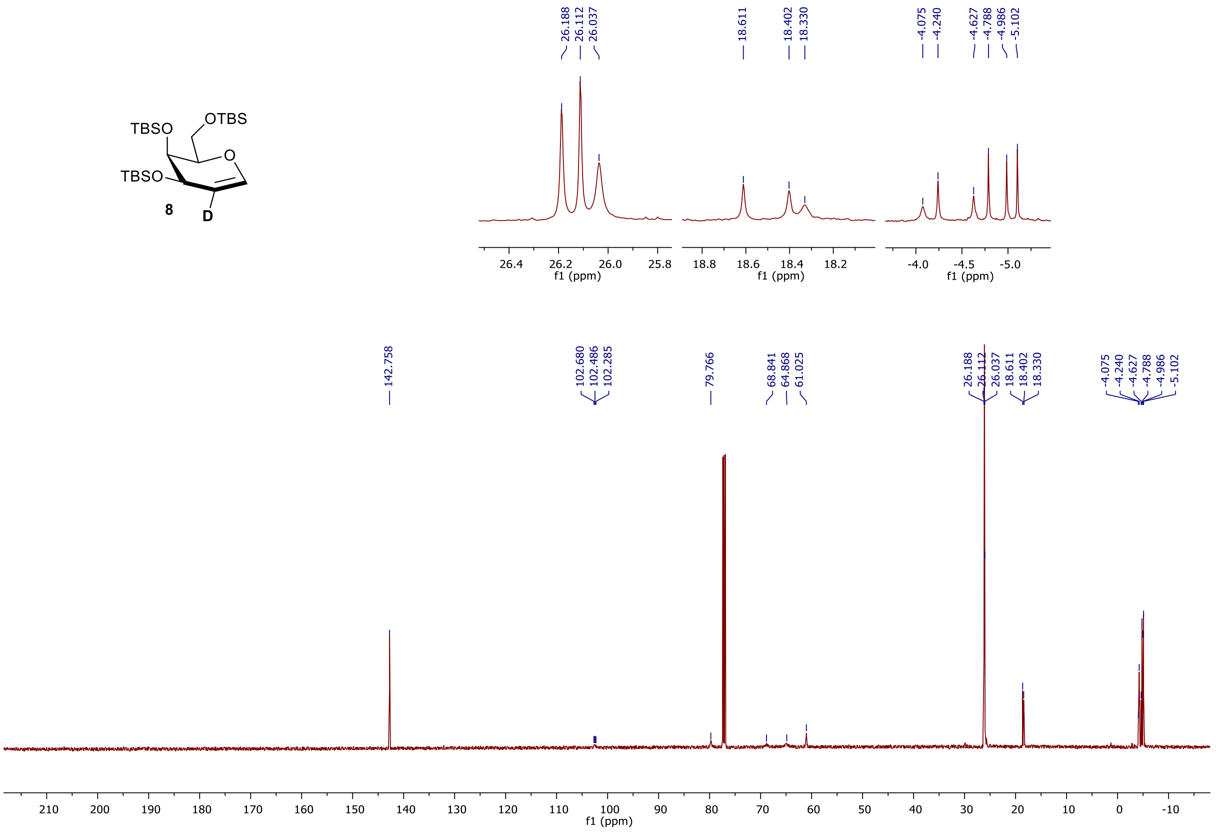

Supplementary figure S102: ^{13}C spectra for **8**

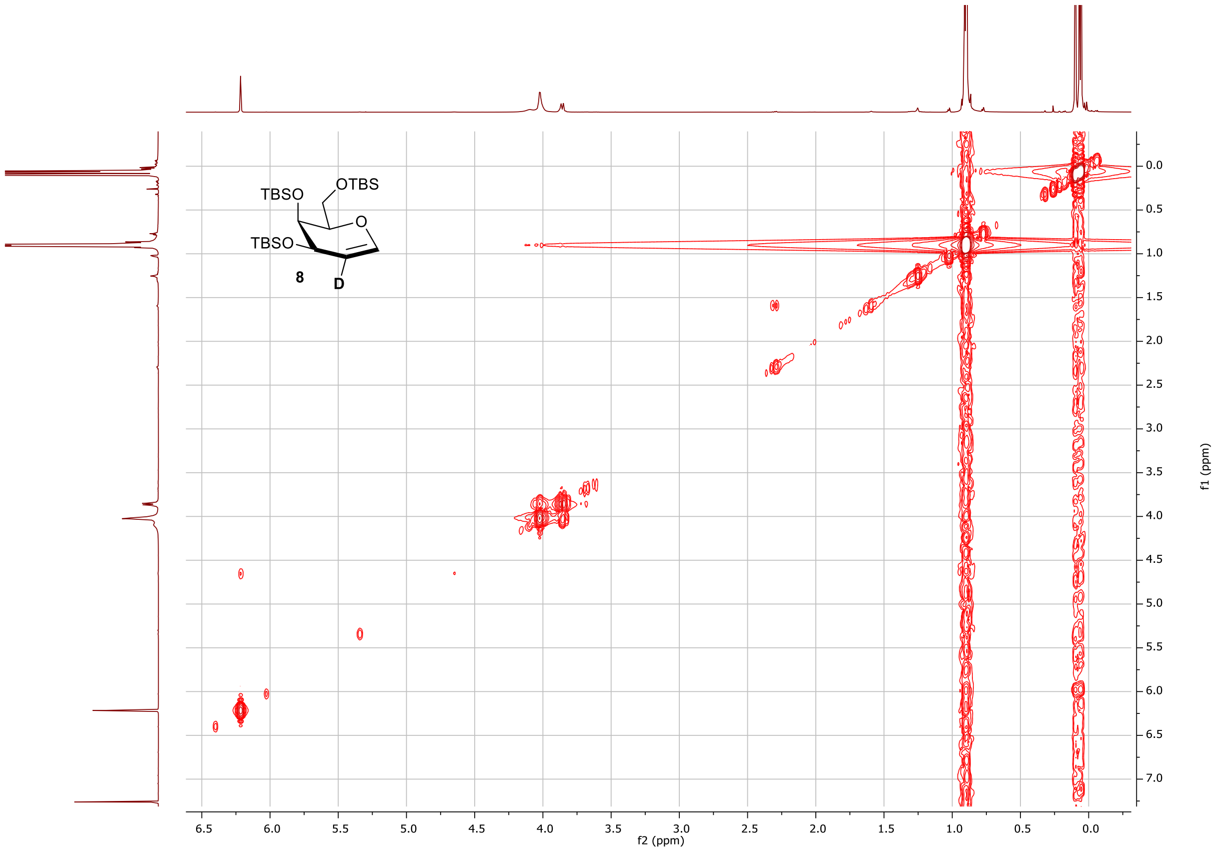

Supplementary figure S103: COSY spectra for **8**

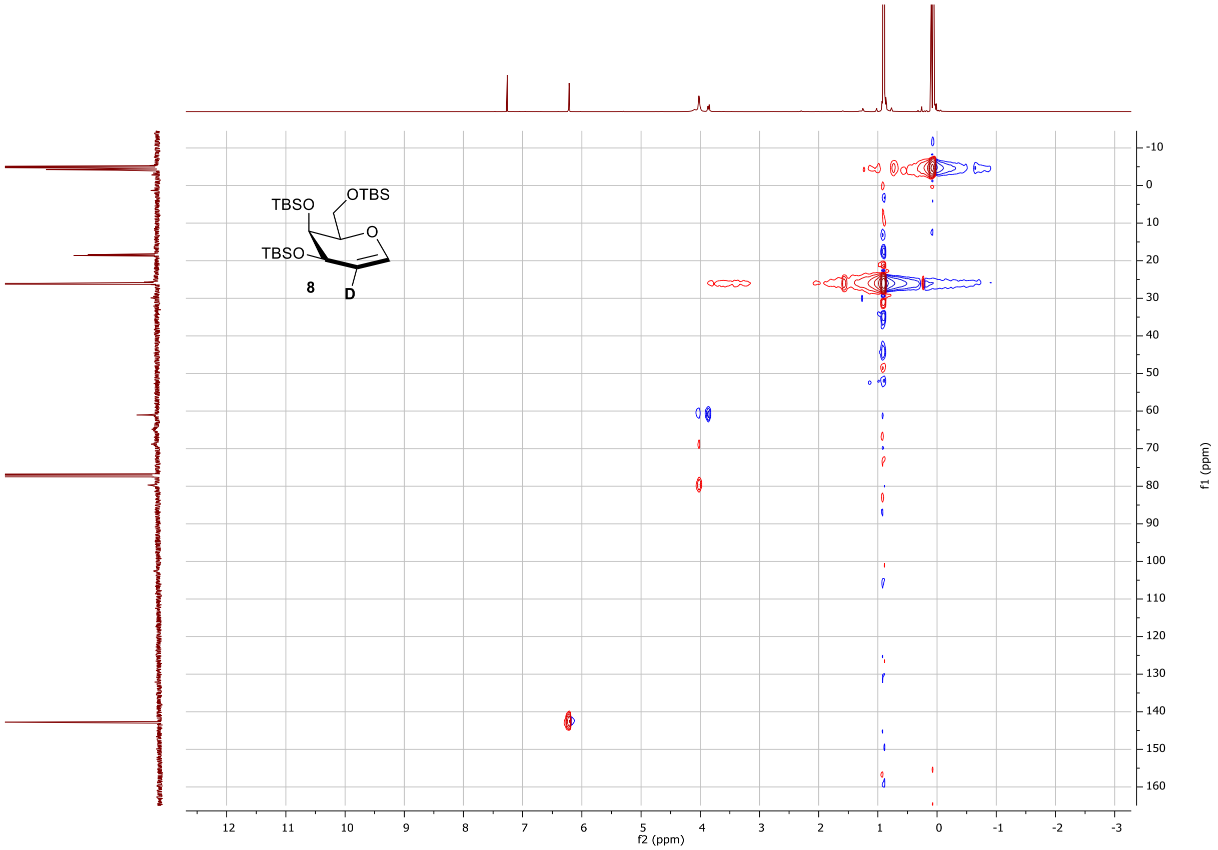

Supplementary figure S104: HSQC spectra for **8**

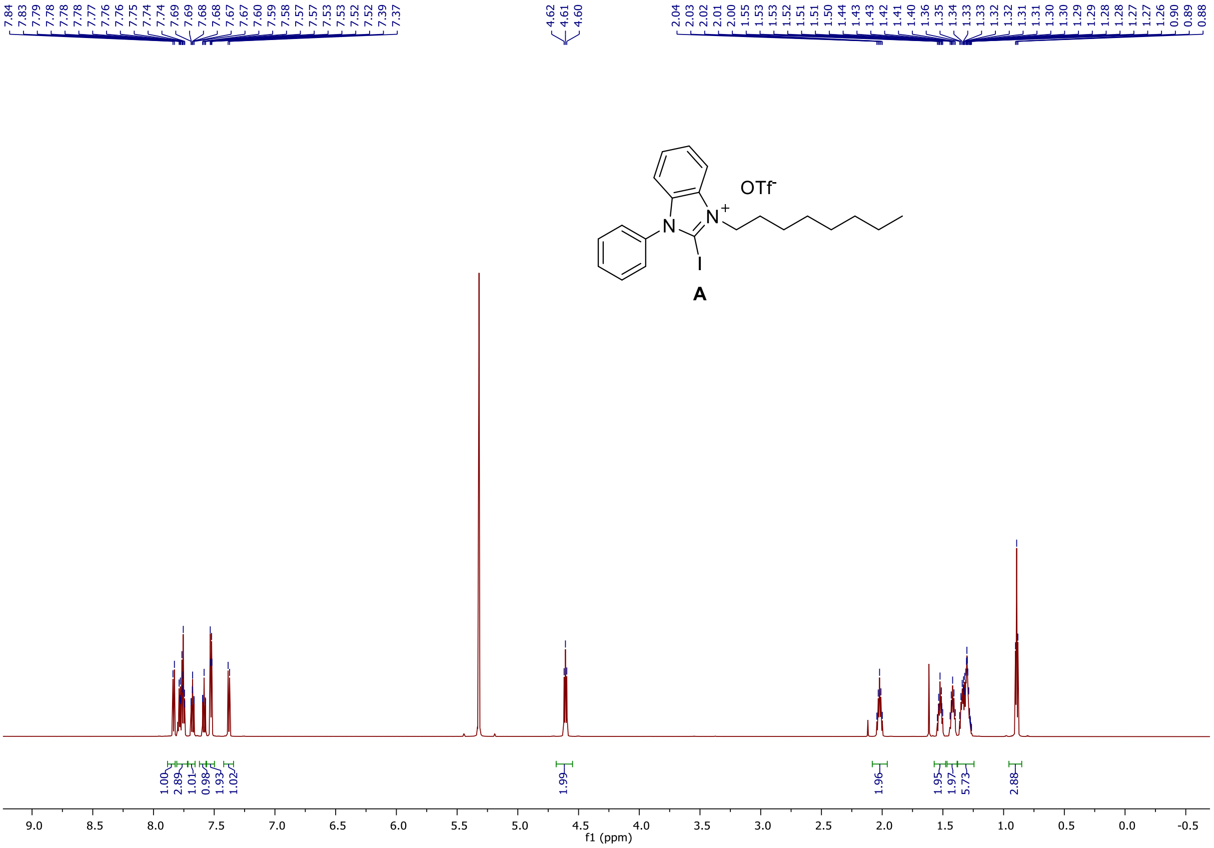

Supplementary figure S105: ^{1}H spectra for compound **A**

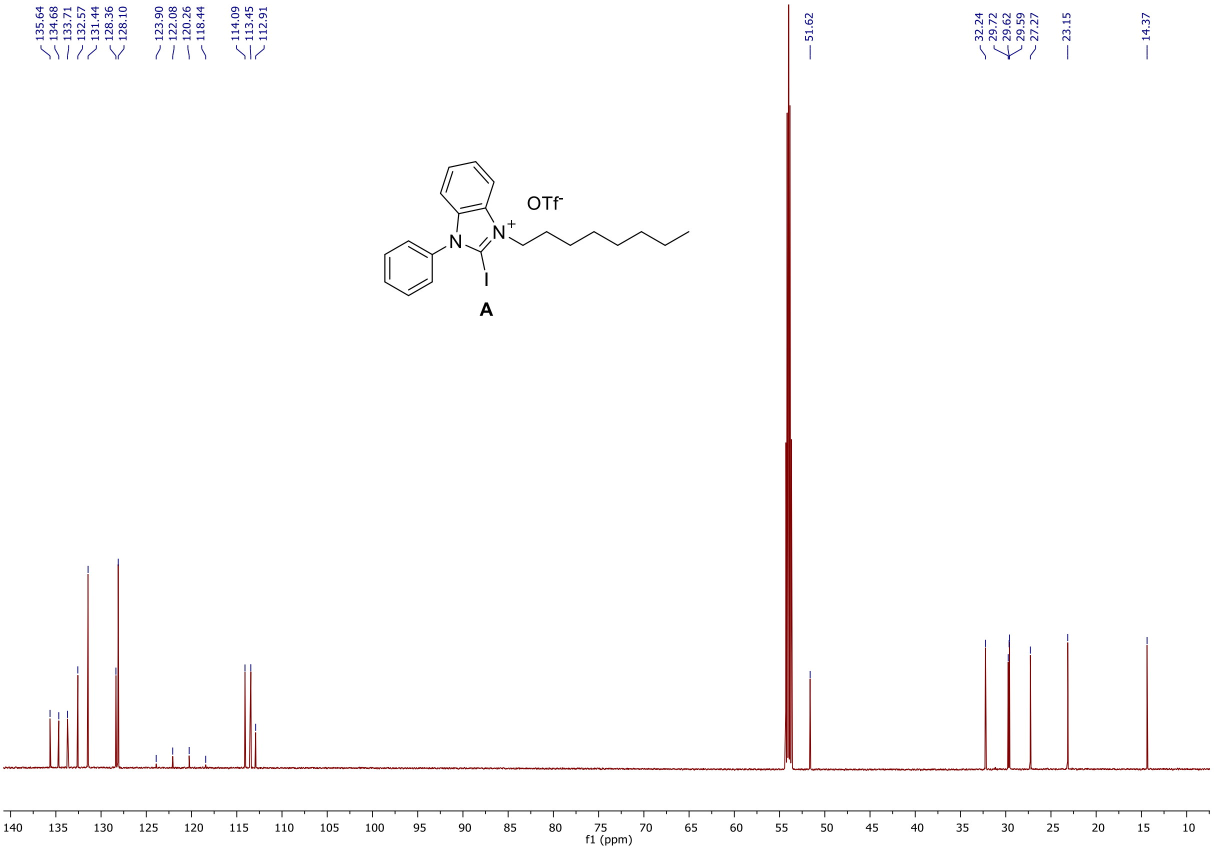

Supplementary figure S106: ^{13}C spectra for compound **A**

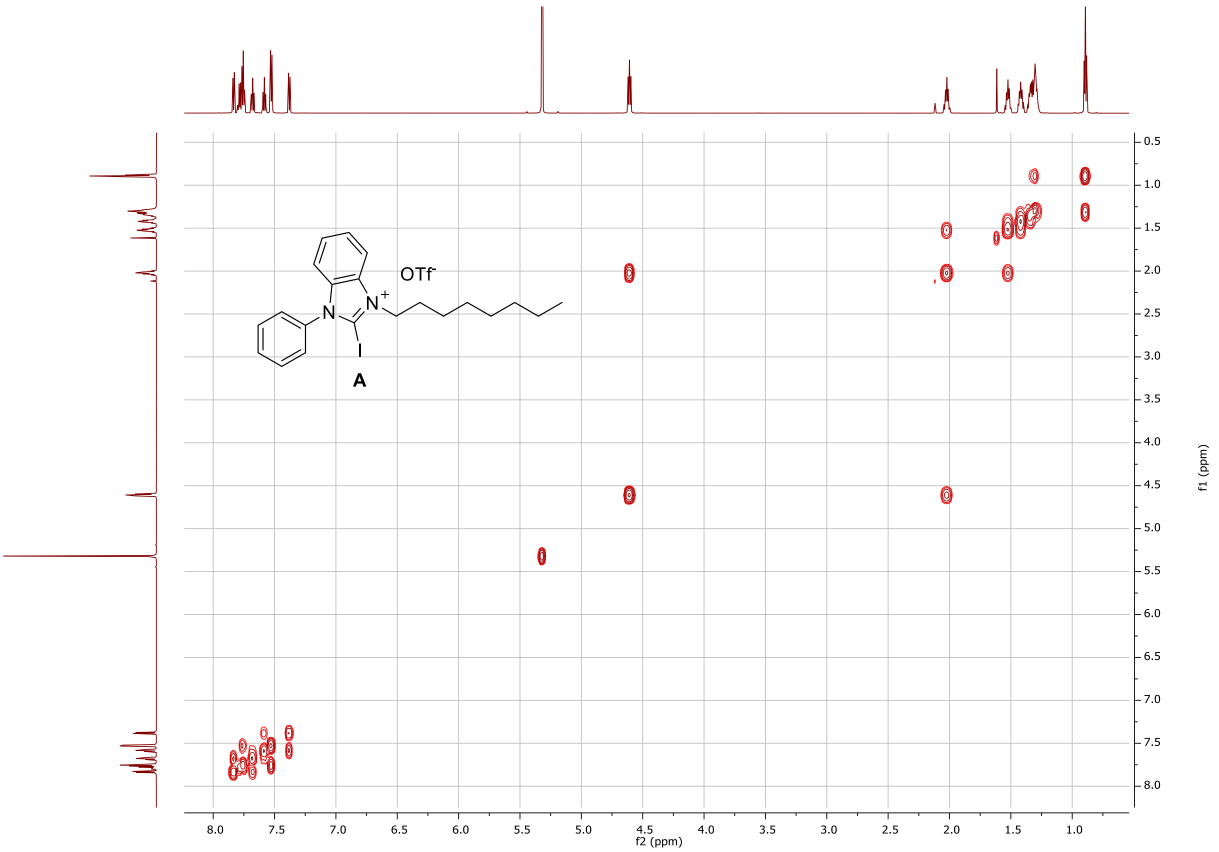

Supplementary figure S107: COSY spectra for compound **A**

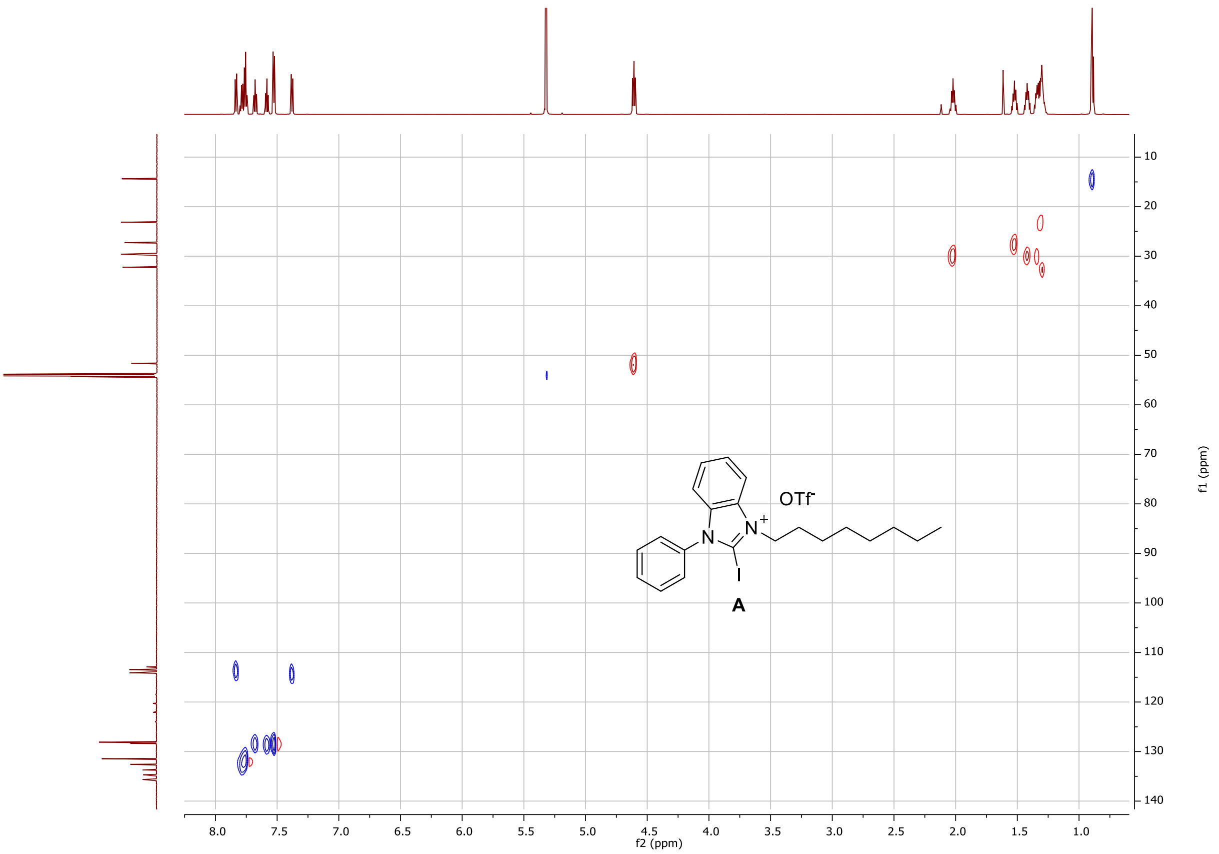

Supplementary figure S108: HSQC spectra for compound **A**

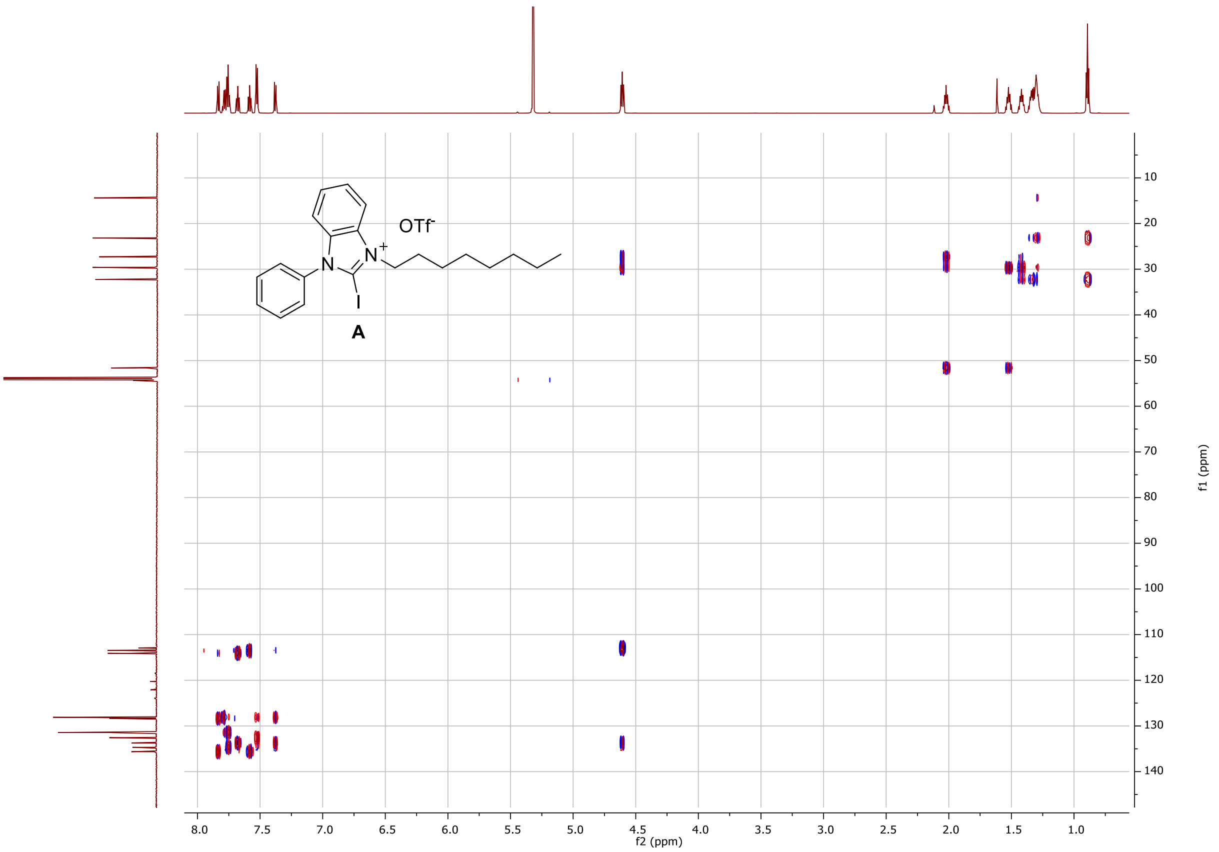

Supplementary figure S109: HMBC spectra for compound **A**

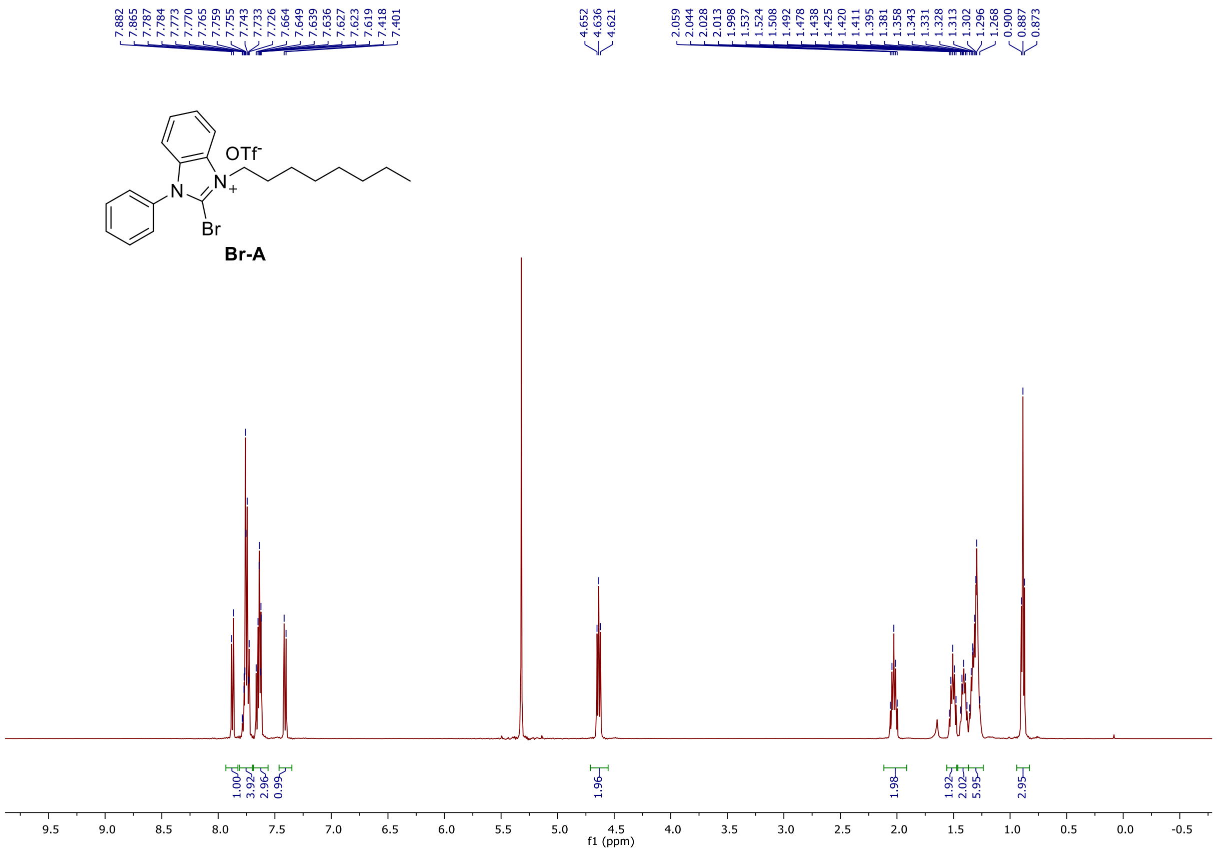

Supplementary figure S110: ^{1}H spectra for compound **Br-A**

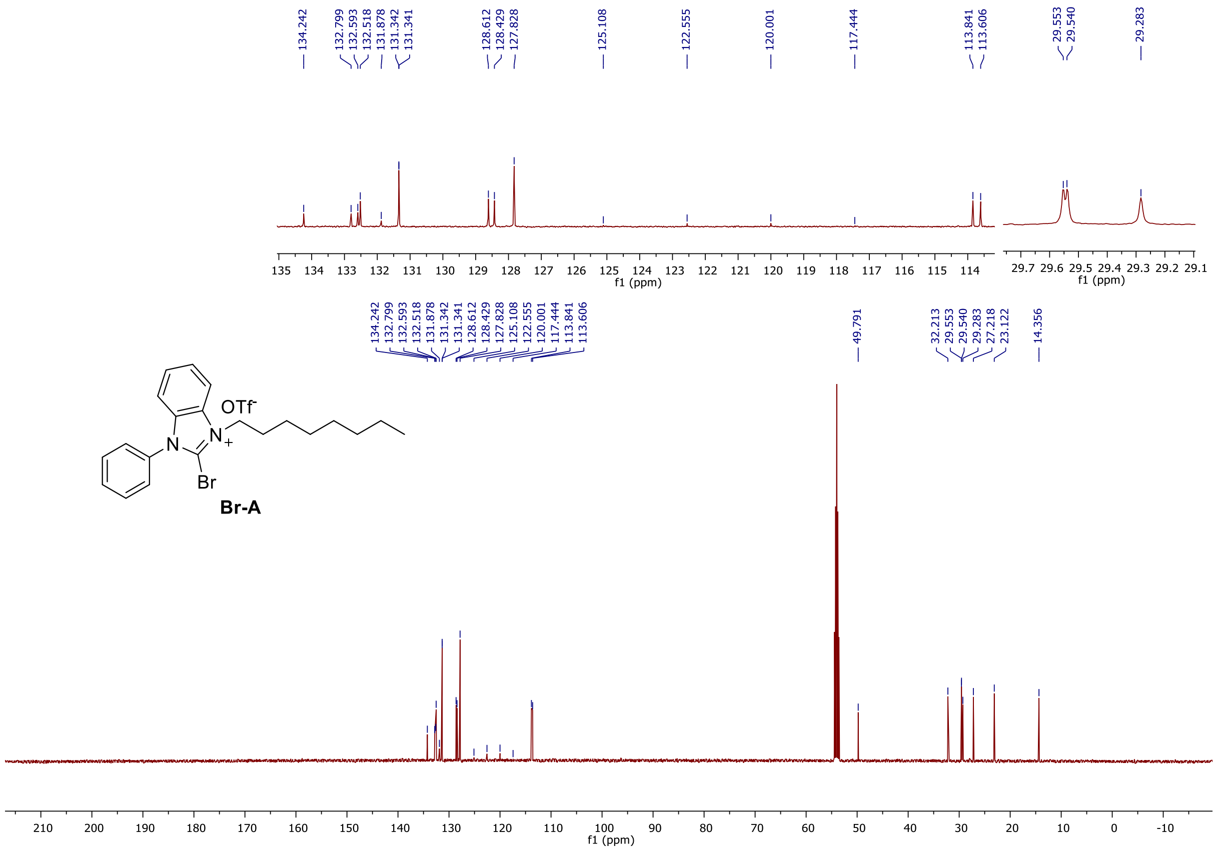

Supplementary figure S111: ^{13}C spectra for compound **Br-A**

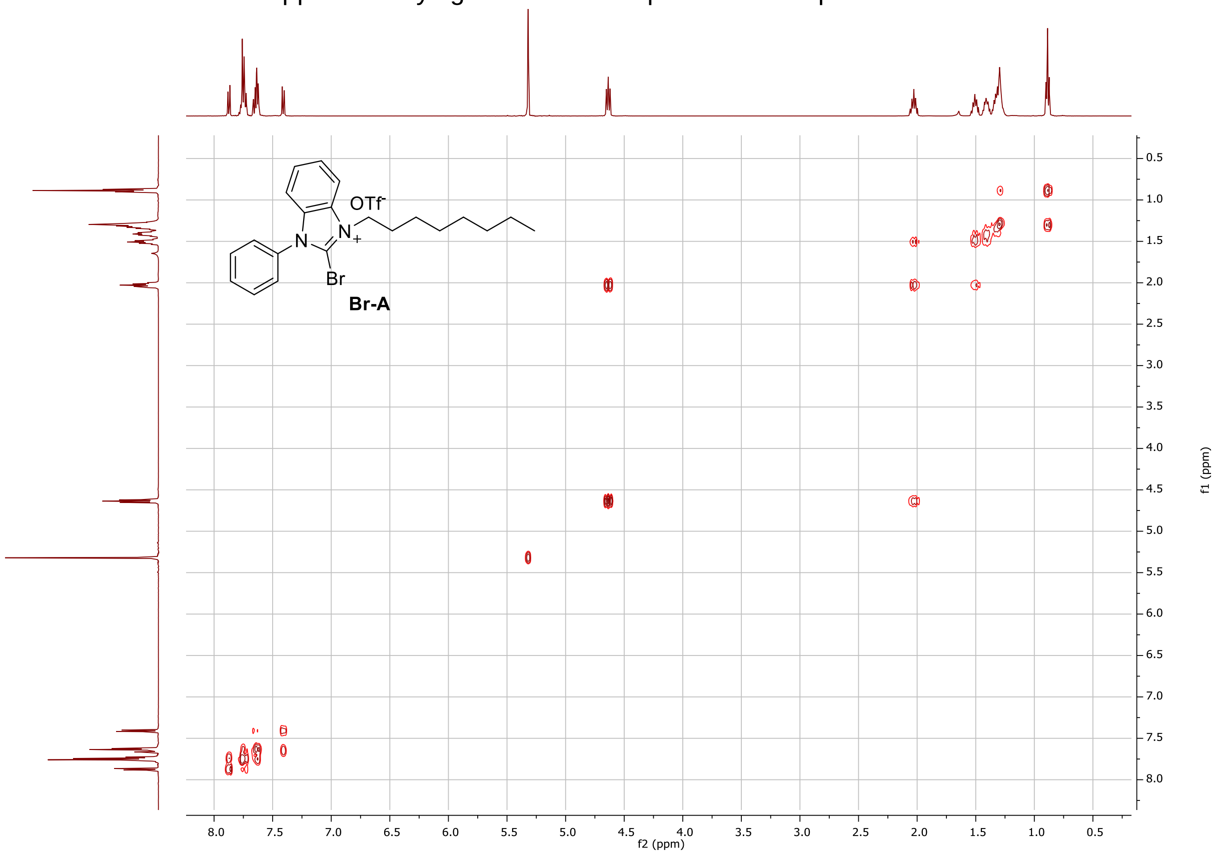

Supplementary figure S112: COSY spectra for compound **Br-A**

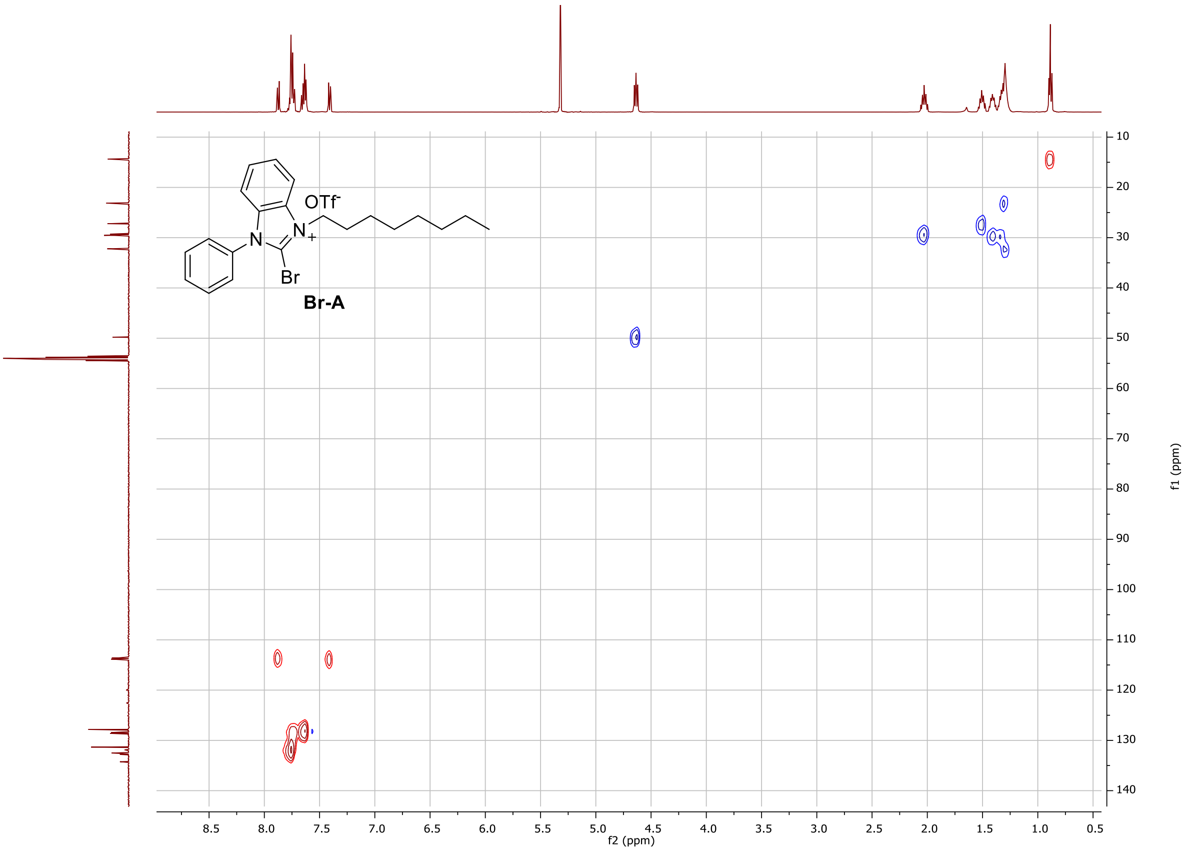

Supplementary figure S113: HSQC spectra for compound **Br-A**

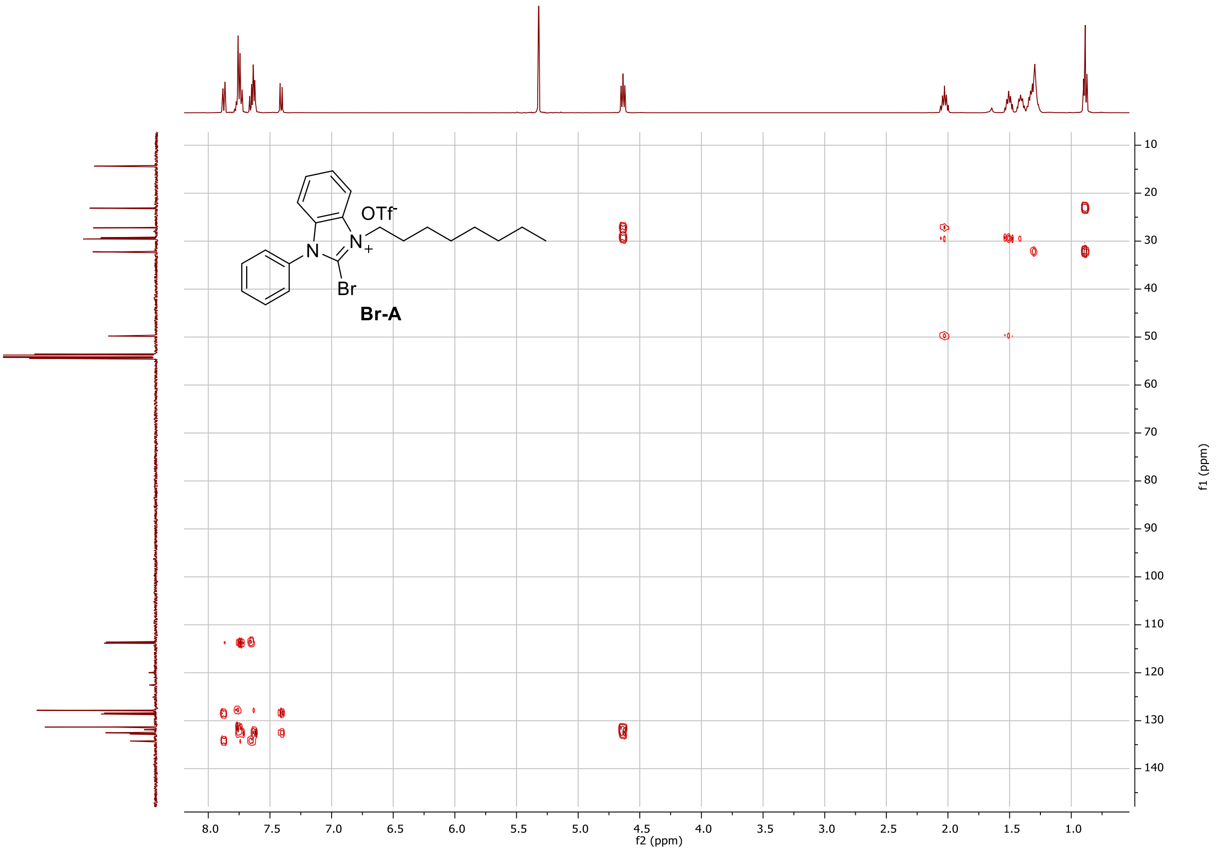

Supplementary figure S114: HMBC spectra for compound **Br-A**

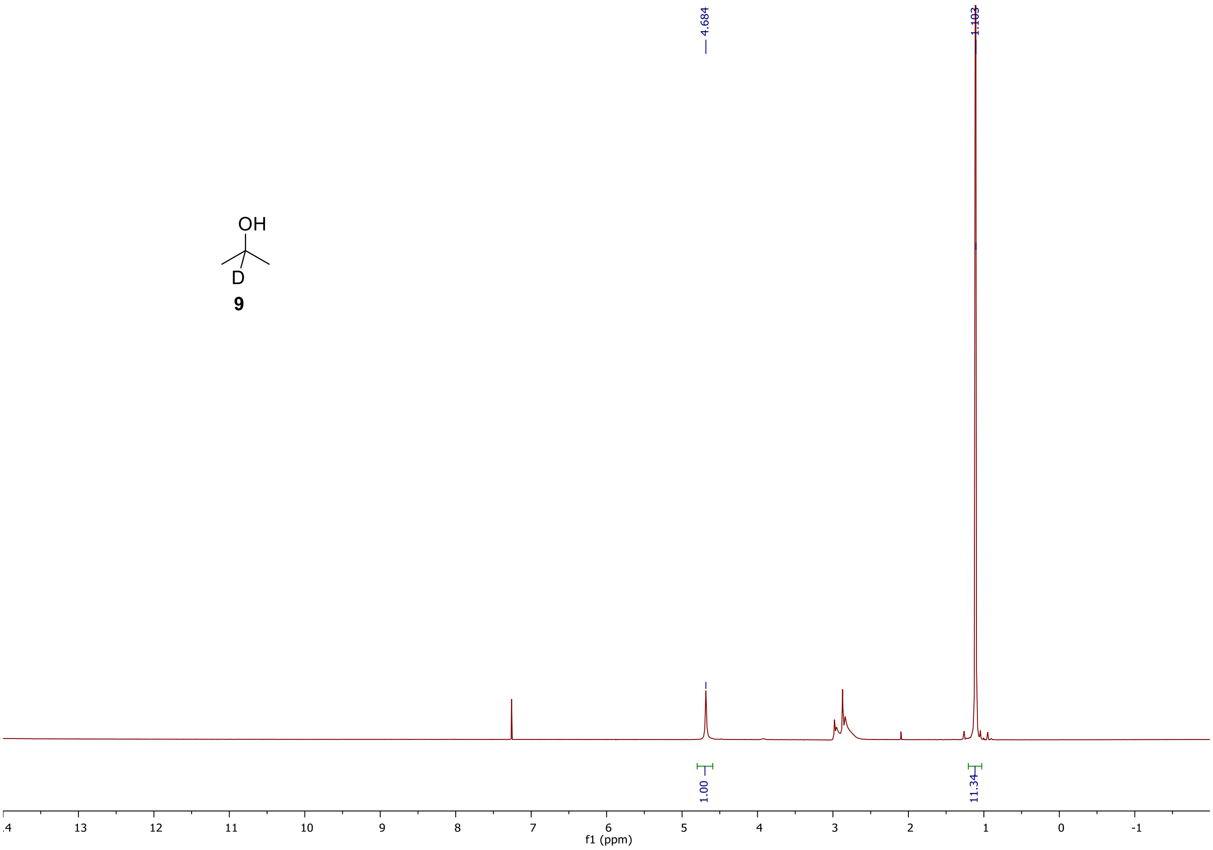

Supplementary figure S115: ^{1}H spectra for compound **9**

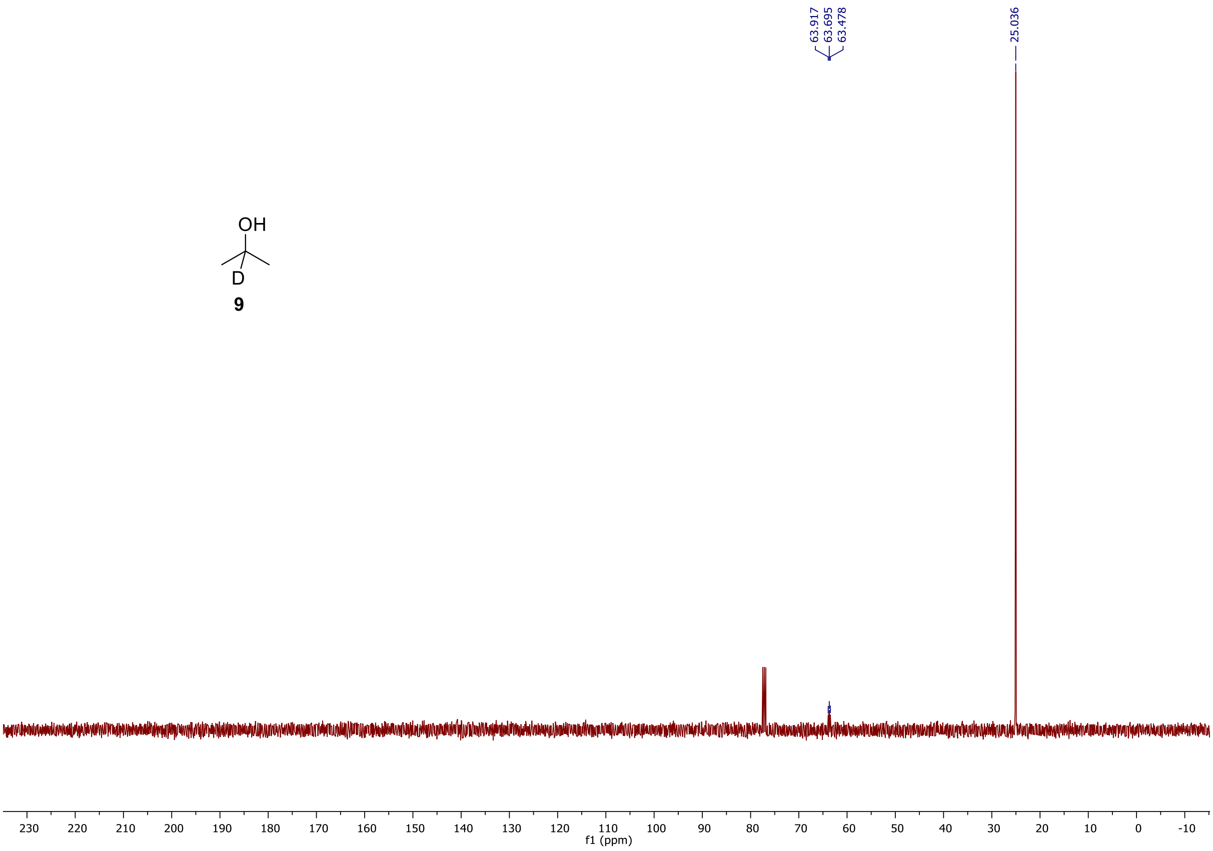

Supplementary figure S116: ^{13}C spectra for compound **9**

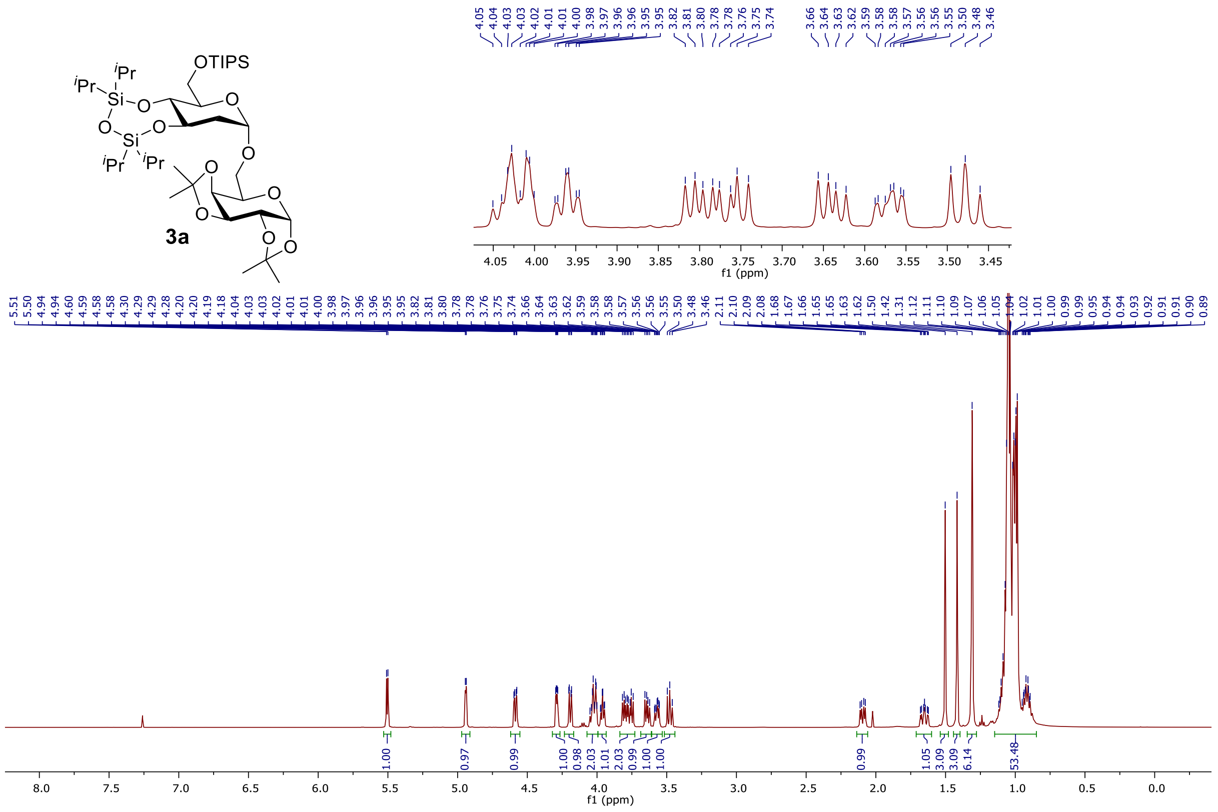

Supplementary figure S117: ^{1}H spectra for **3a**

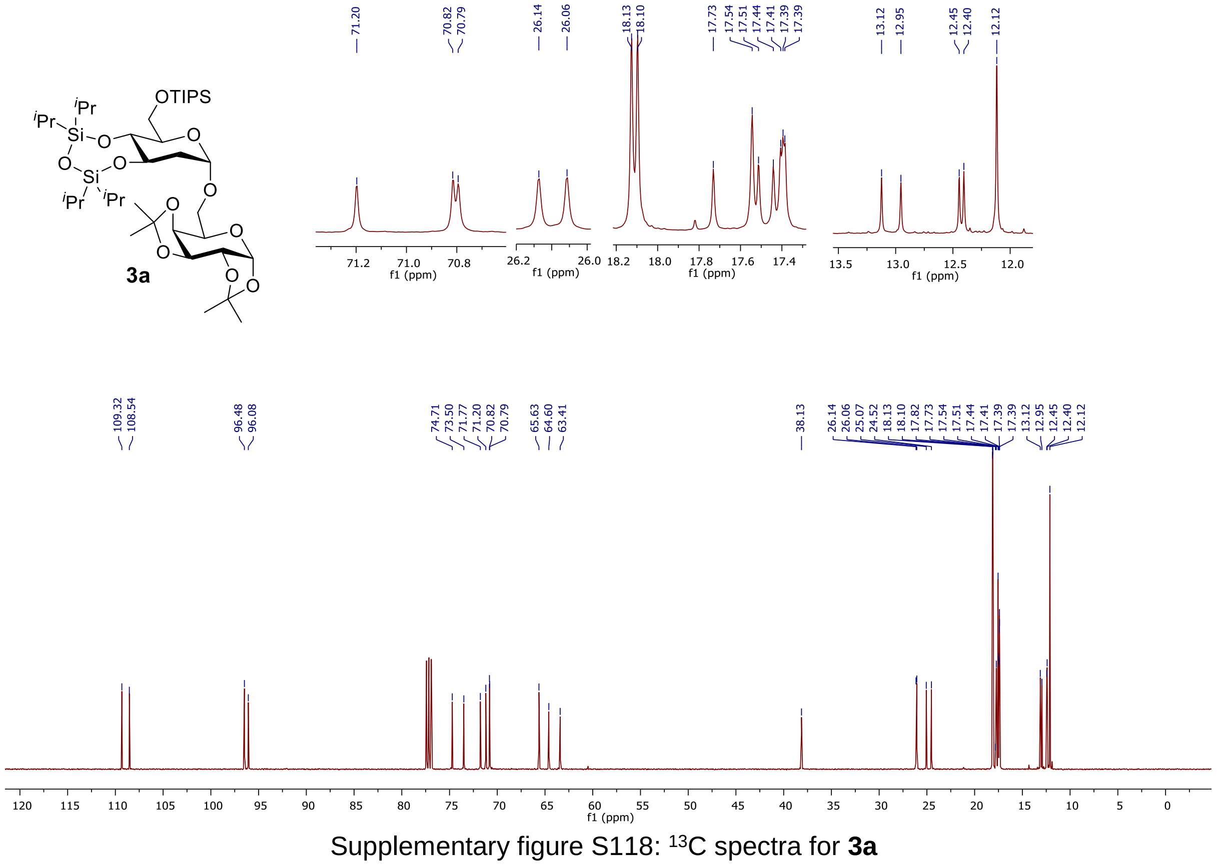

Supplementary figure S118: ^{13}C spectra for **3a**

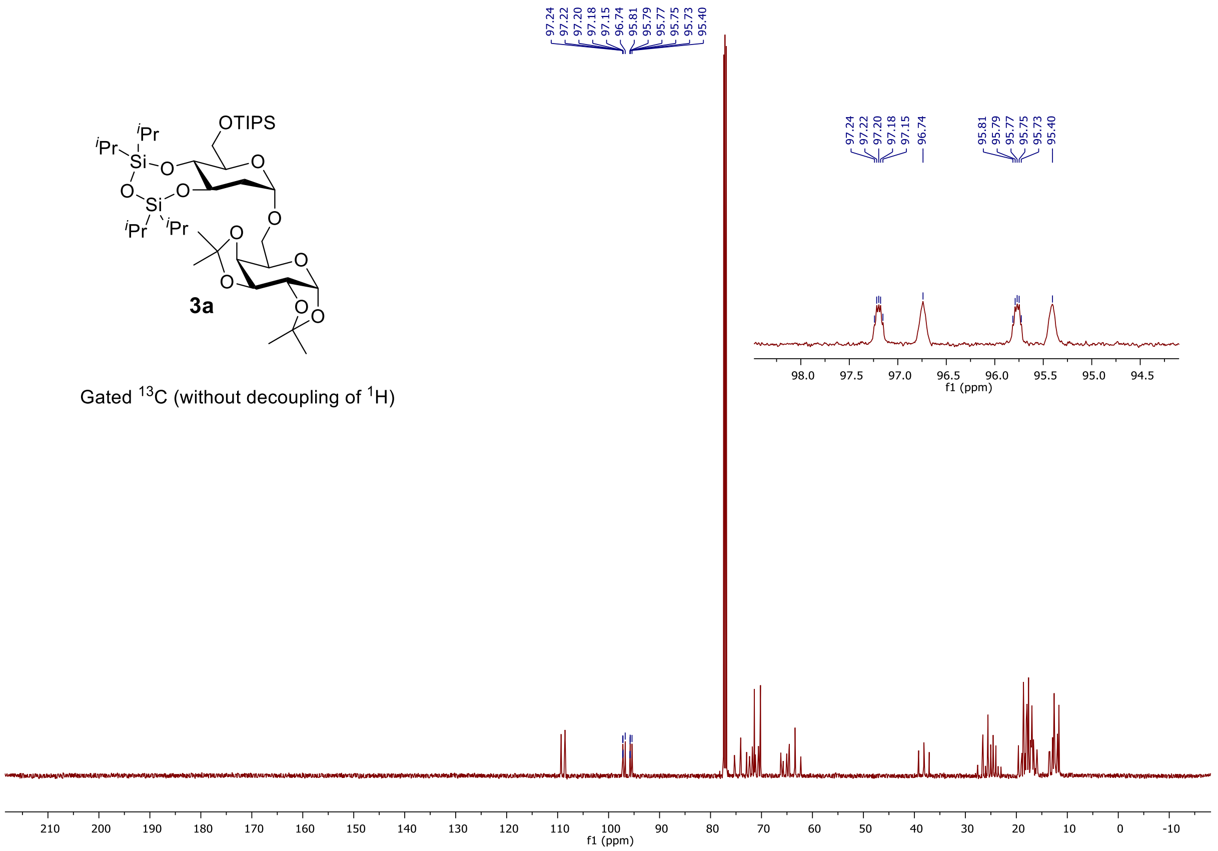

Supplementary figure S119: Gated ^{13}C spectra for **3a**

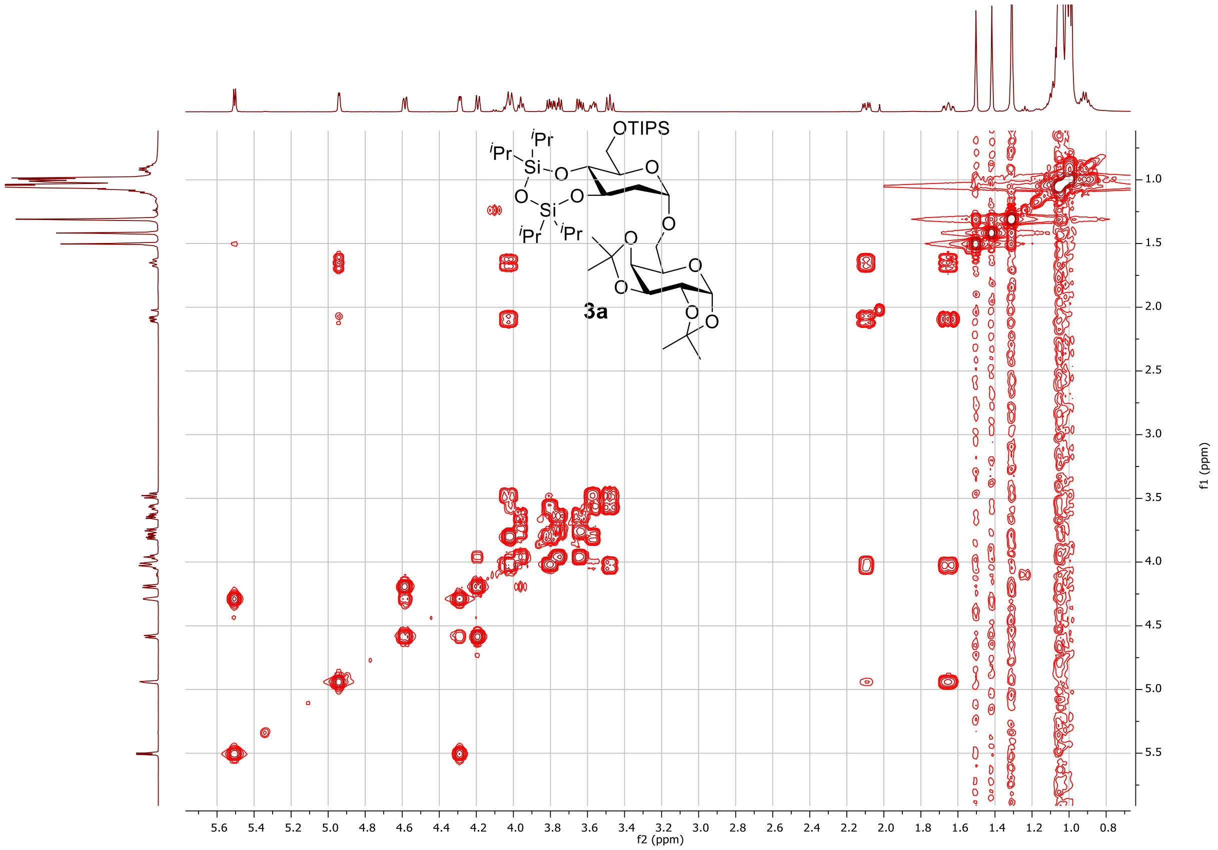

Supplementary figure S120: COSY spectra for **3a**

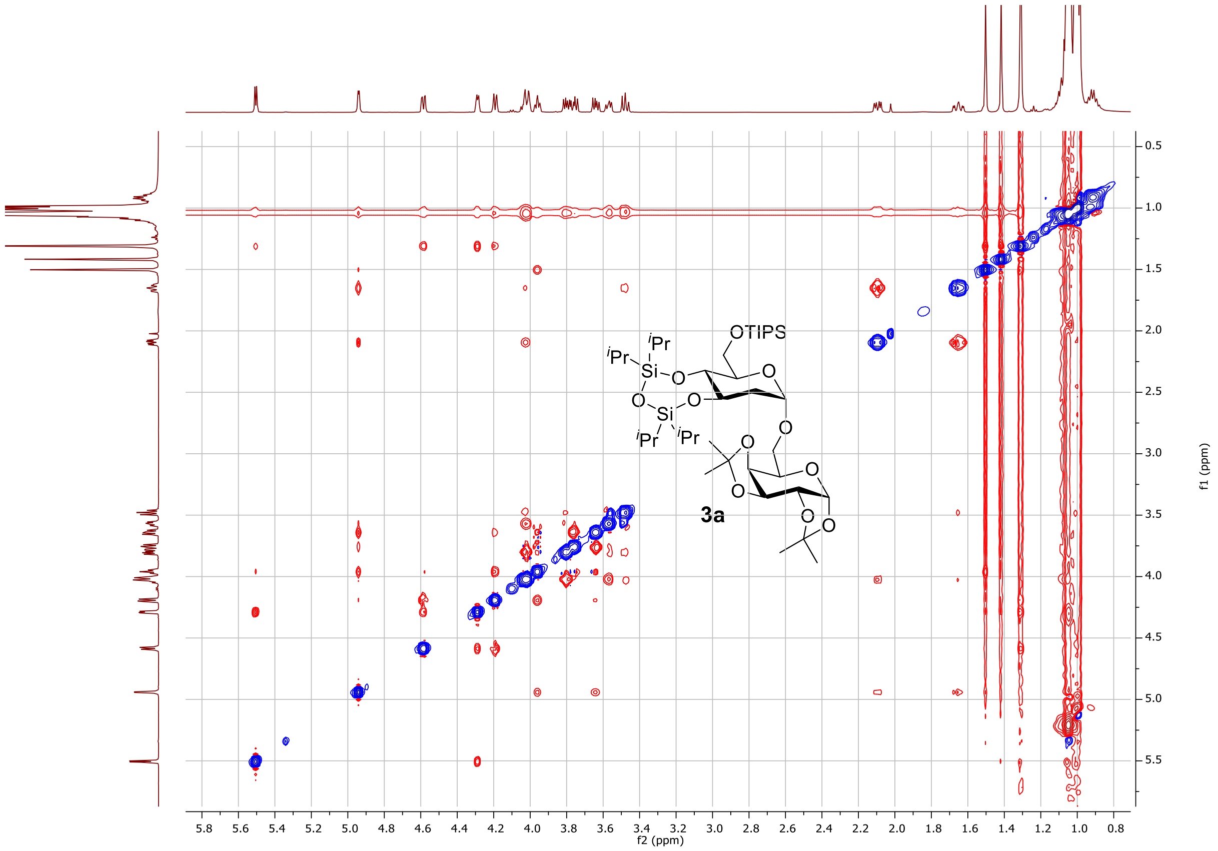

Supplementary figure S121: NOESY spectra for **3a**

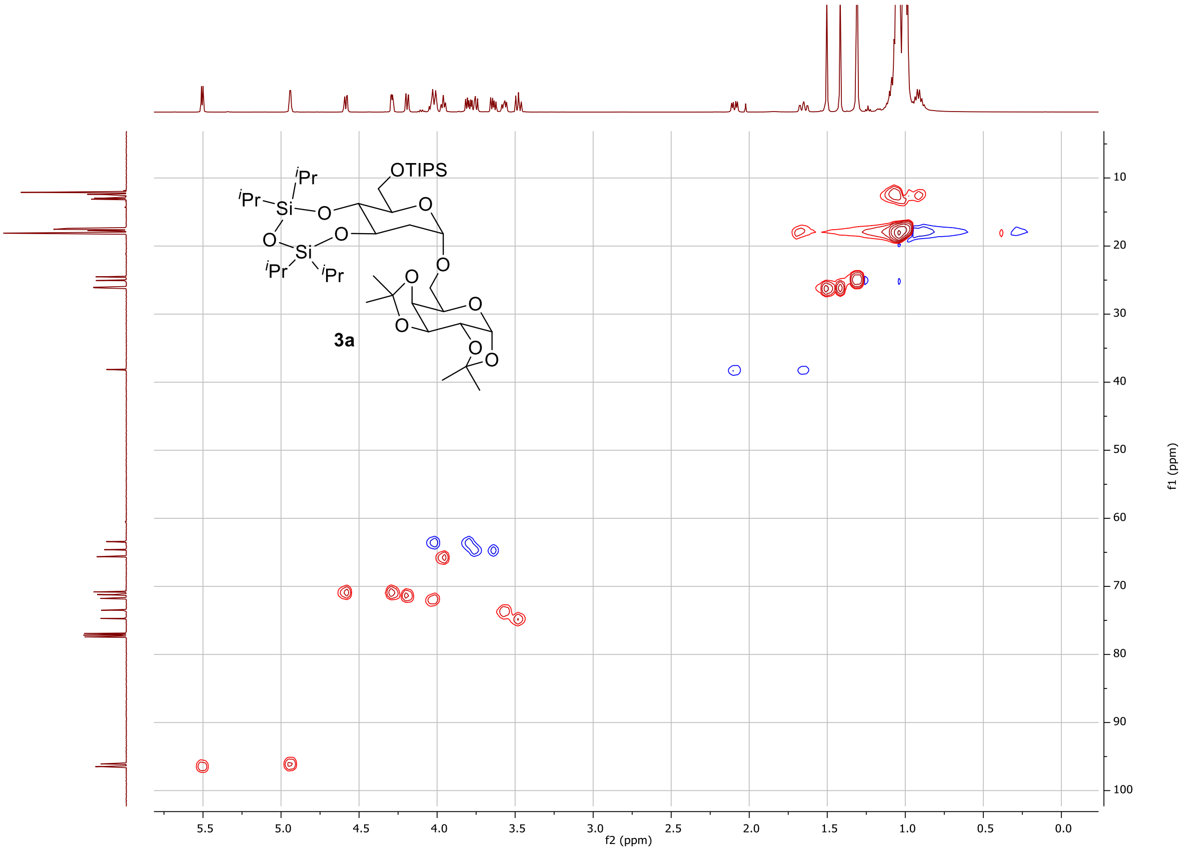

Supplementary figure S122: HSQC spectra for **3a**

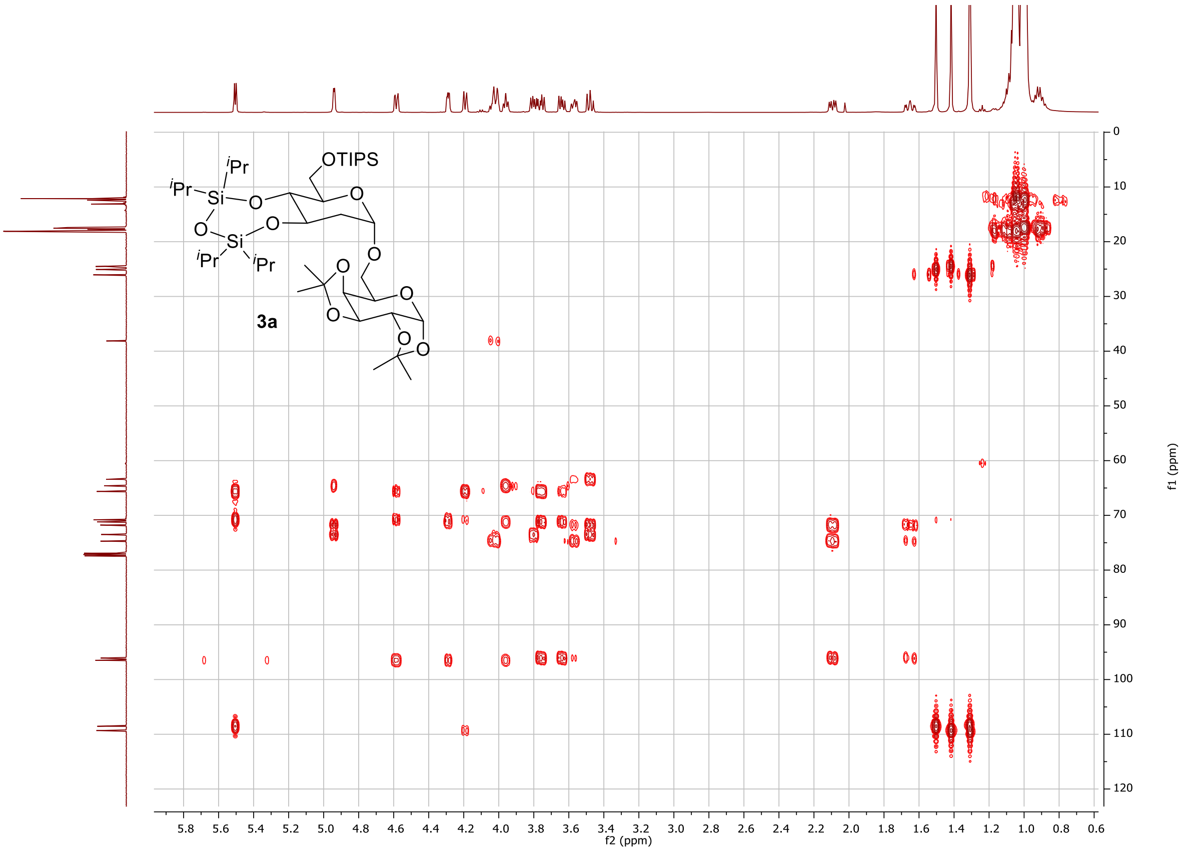

Supplementary figure S123: HMBC spectra for **3a**

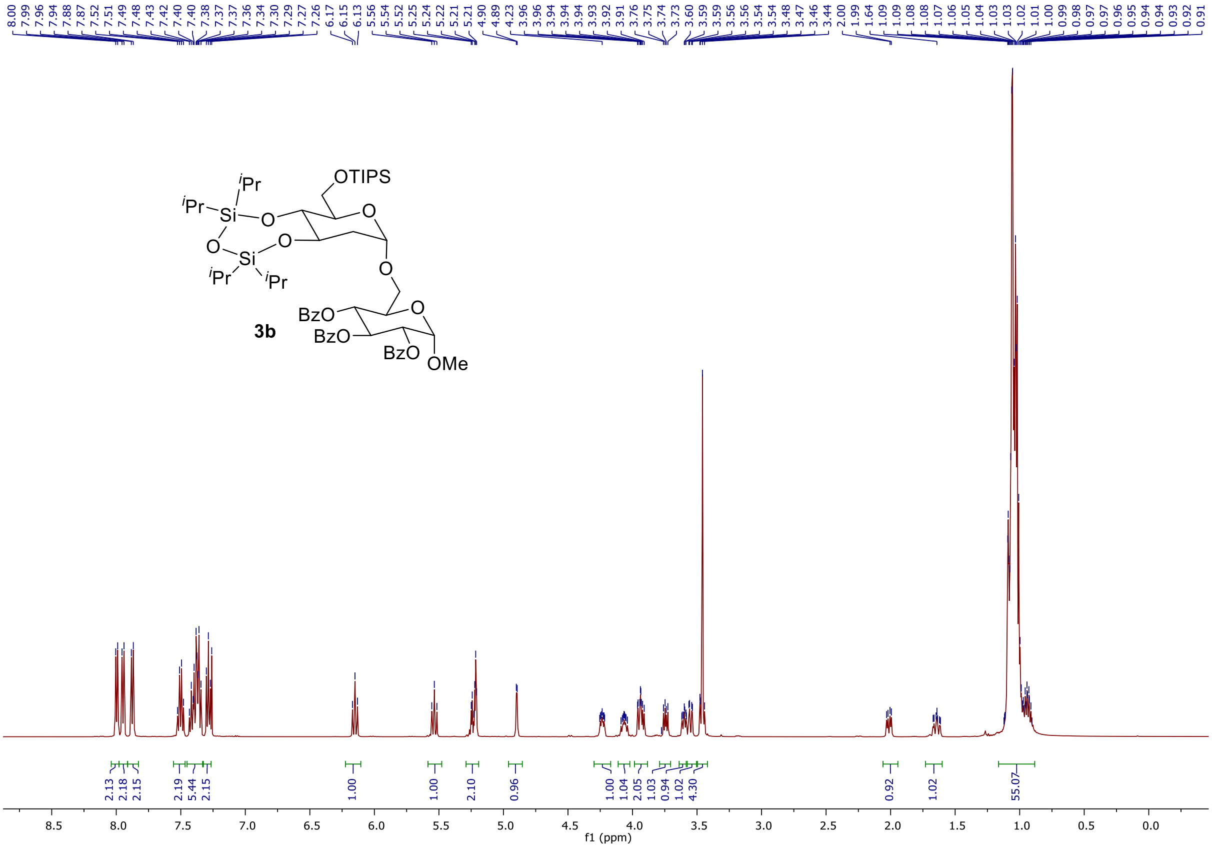

Supplementary figure S124: ^{1}H spectra for **3b**

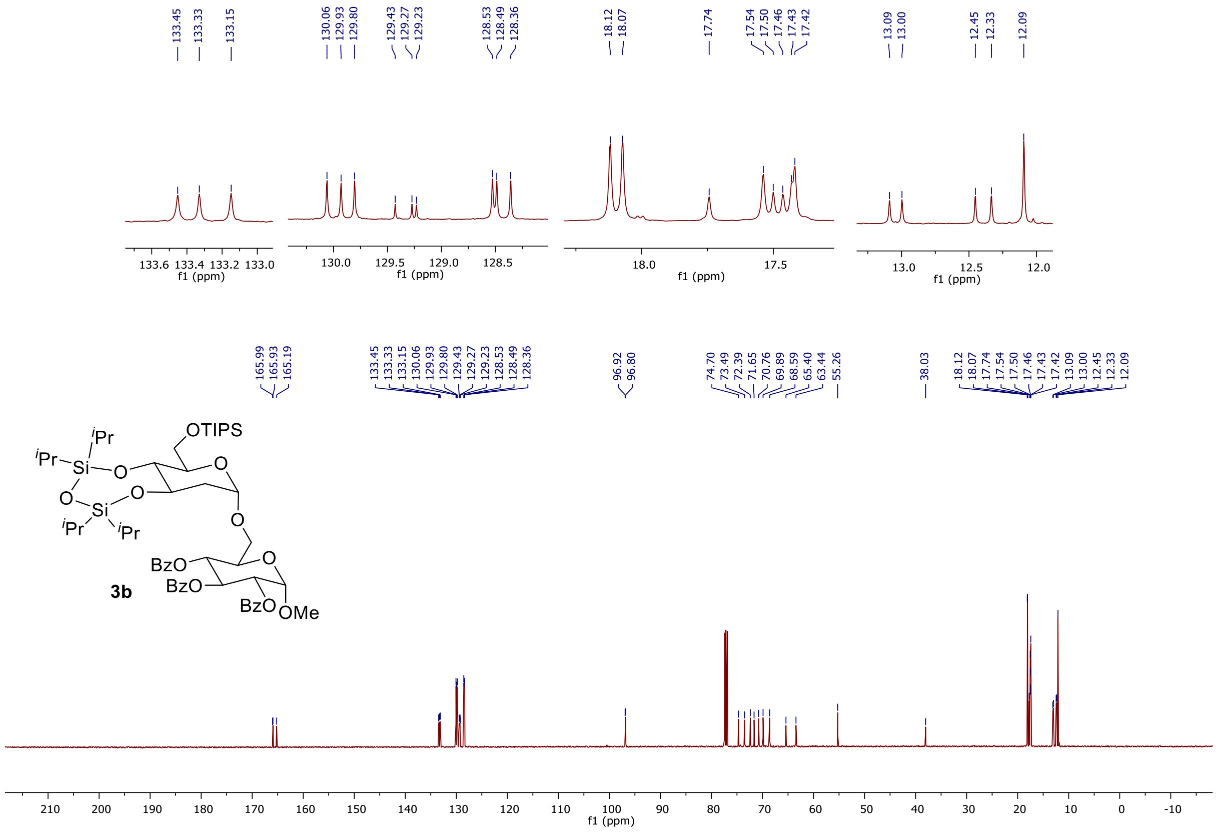

Supplementary figure S125: ^{13}C spectra for **3b**

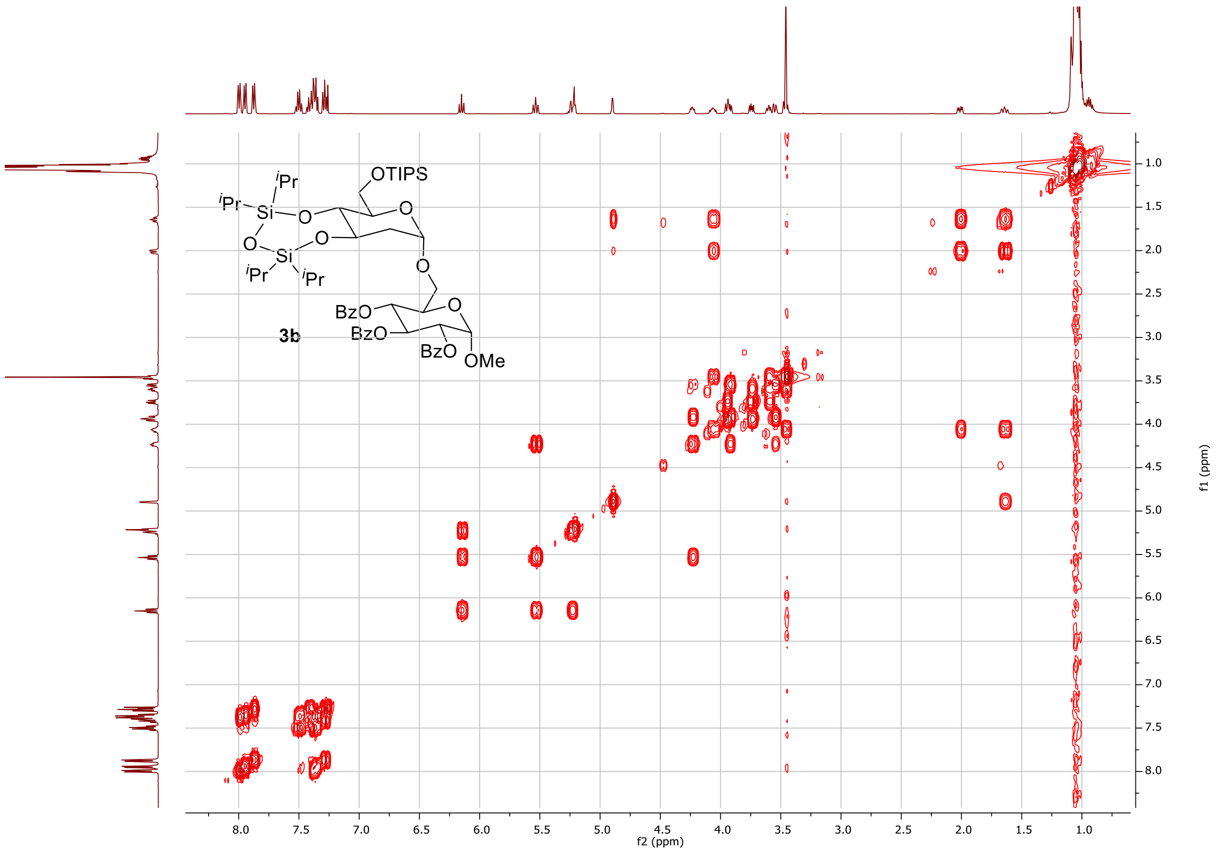

Supplementary figure S126: COSY spectra for **3b**

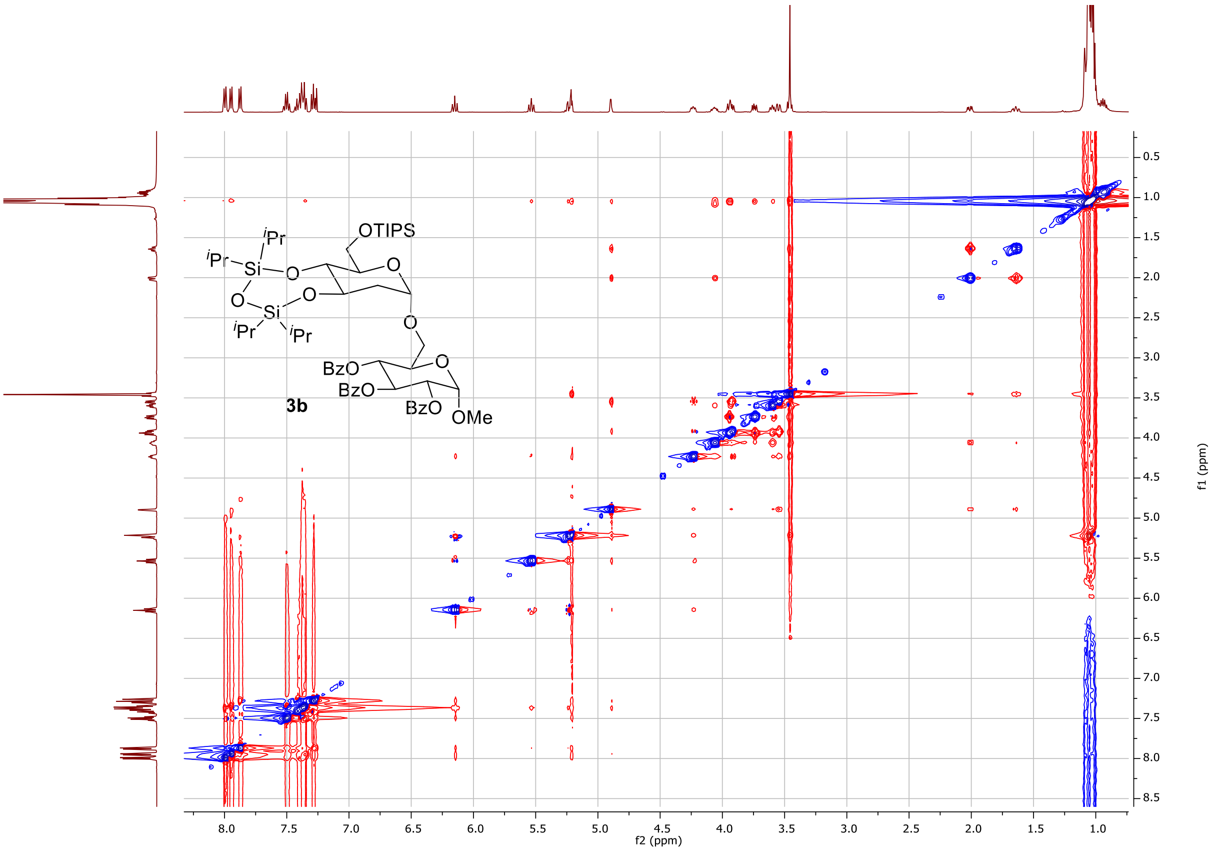

Supplementary figure S127: NOESY spectra for **3b**

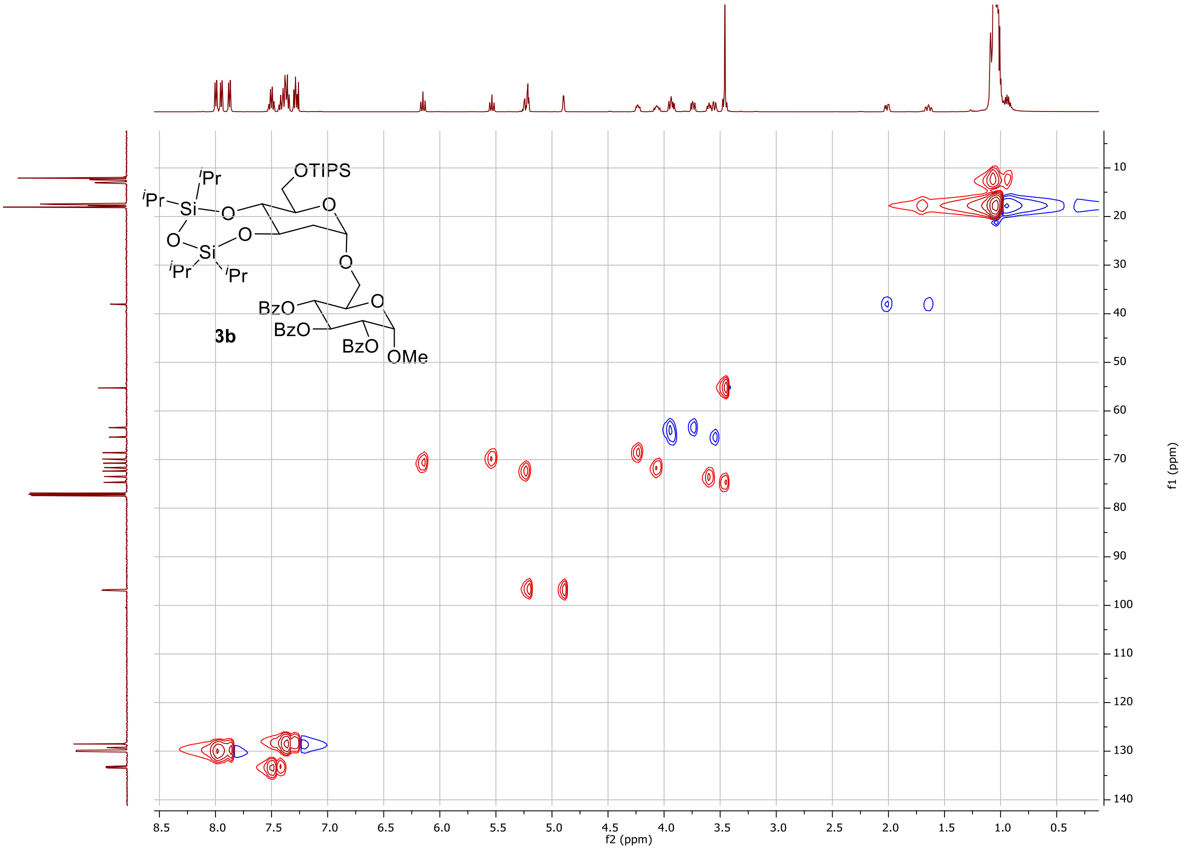

Supplementary figure S128: HSQC spectra for **3b**

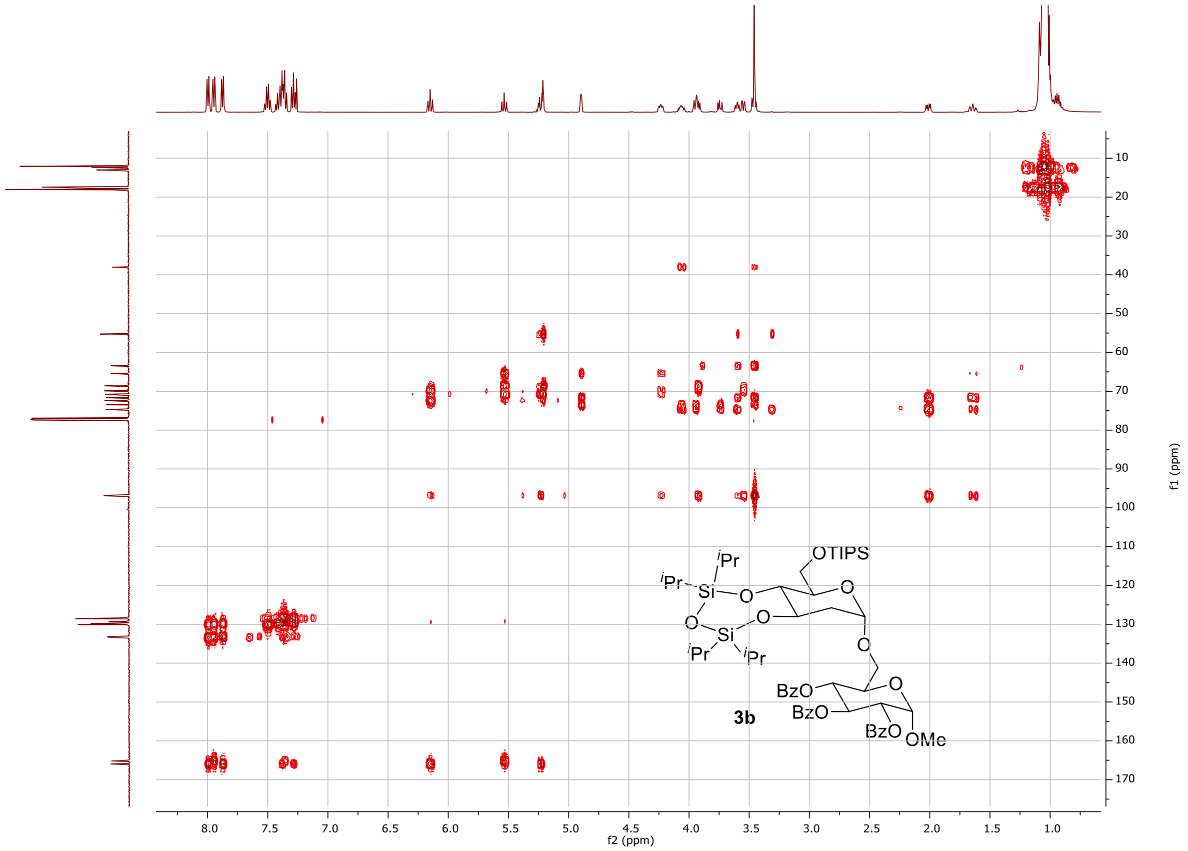

Supplementary figure S129: HMBC spectra for **3b**

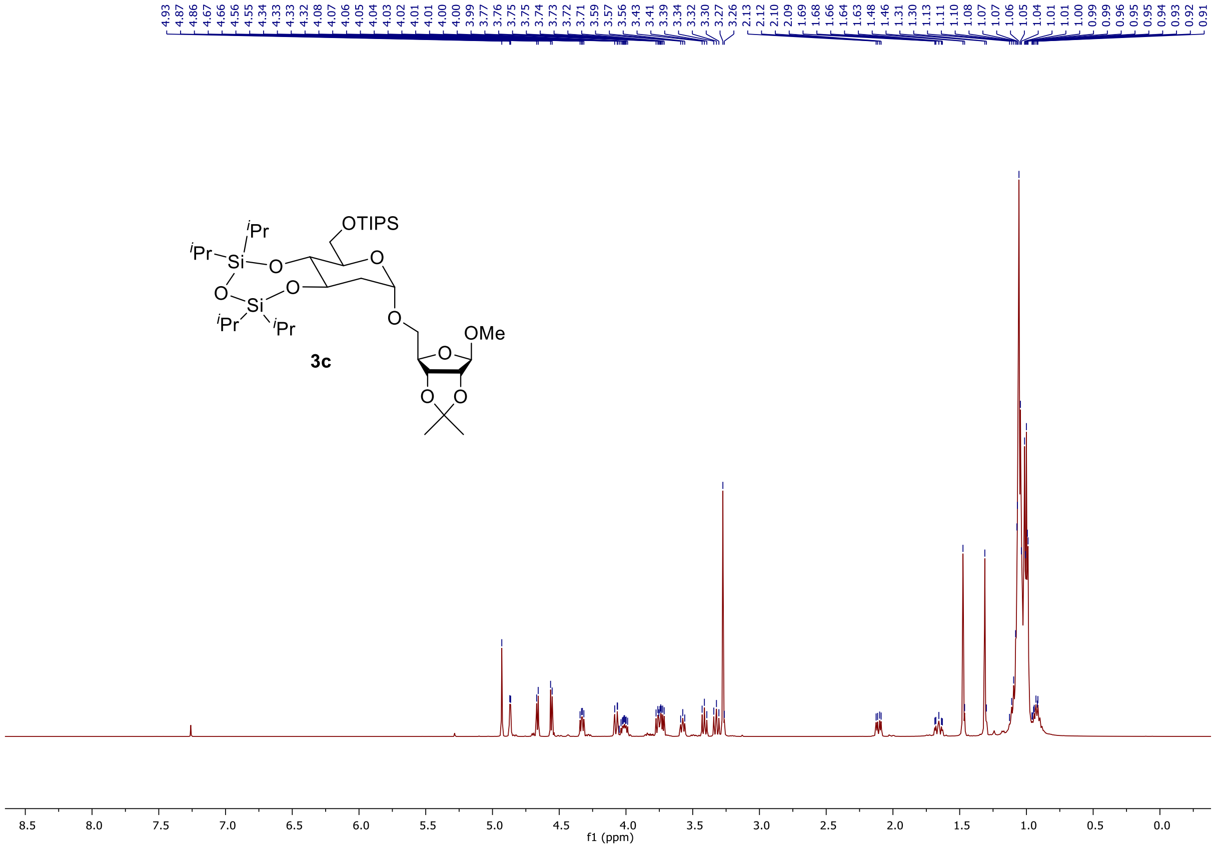

Supplementary figure S130: ^{1}H spectra for **3c**

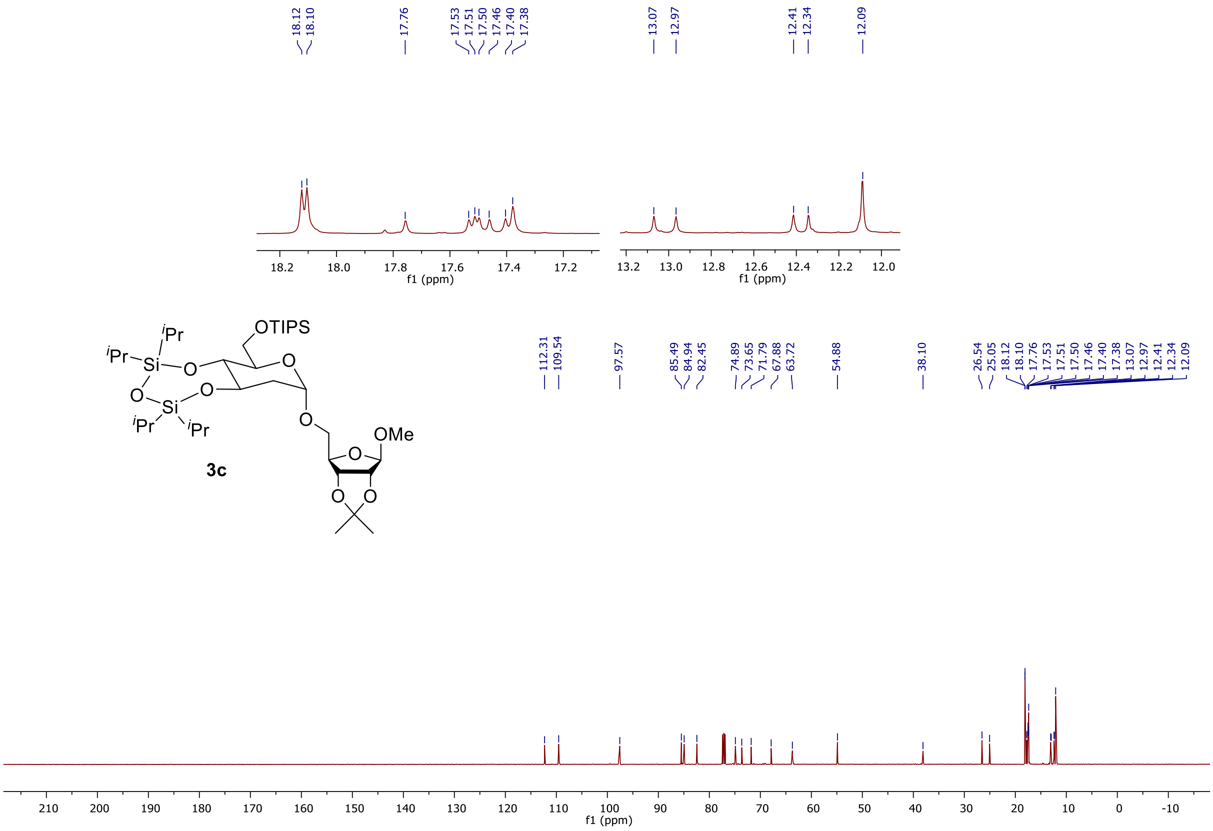

Supplementary figure S131: ^{13}C spectra for **3c**

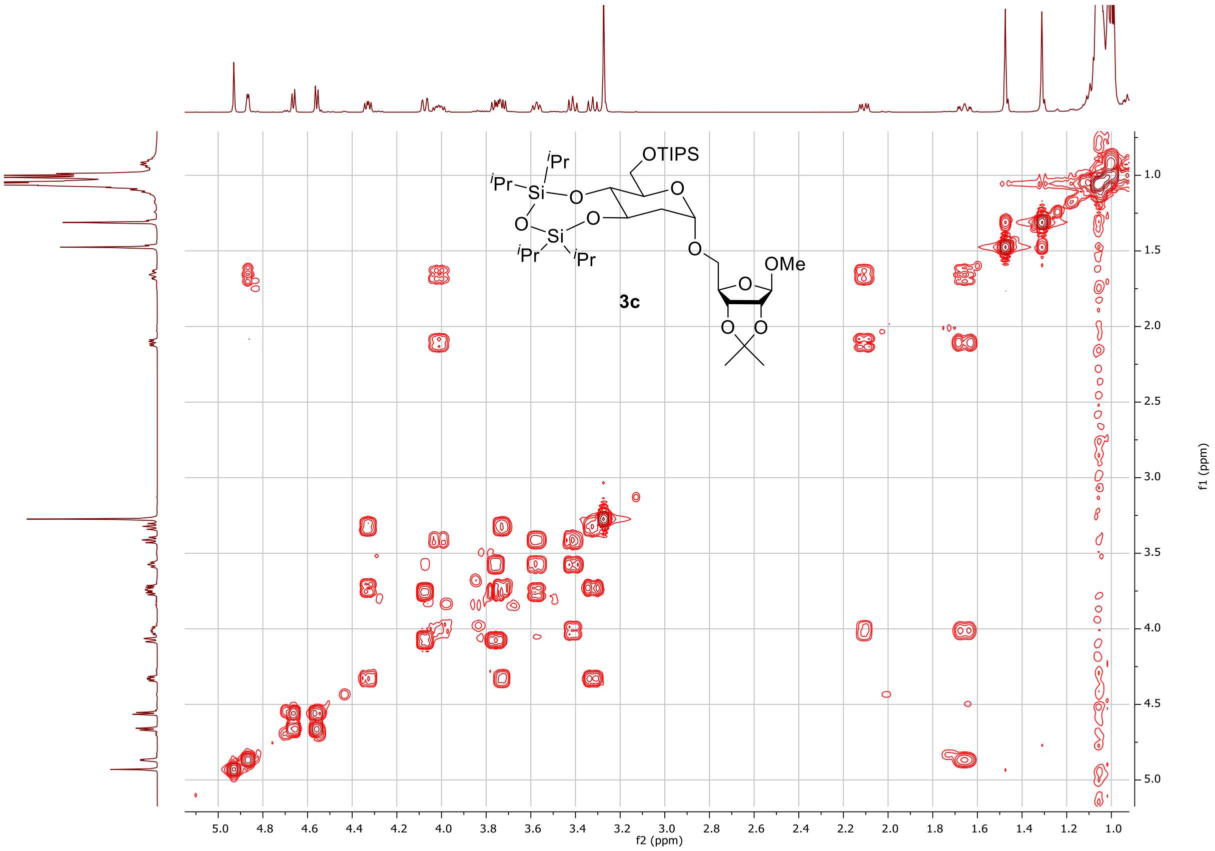

Supplementary figure S132: COSY spectra for **3c**

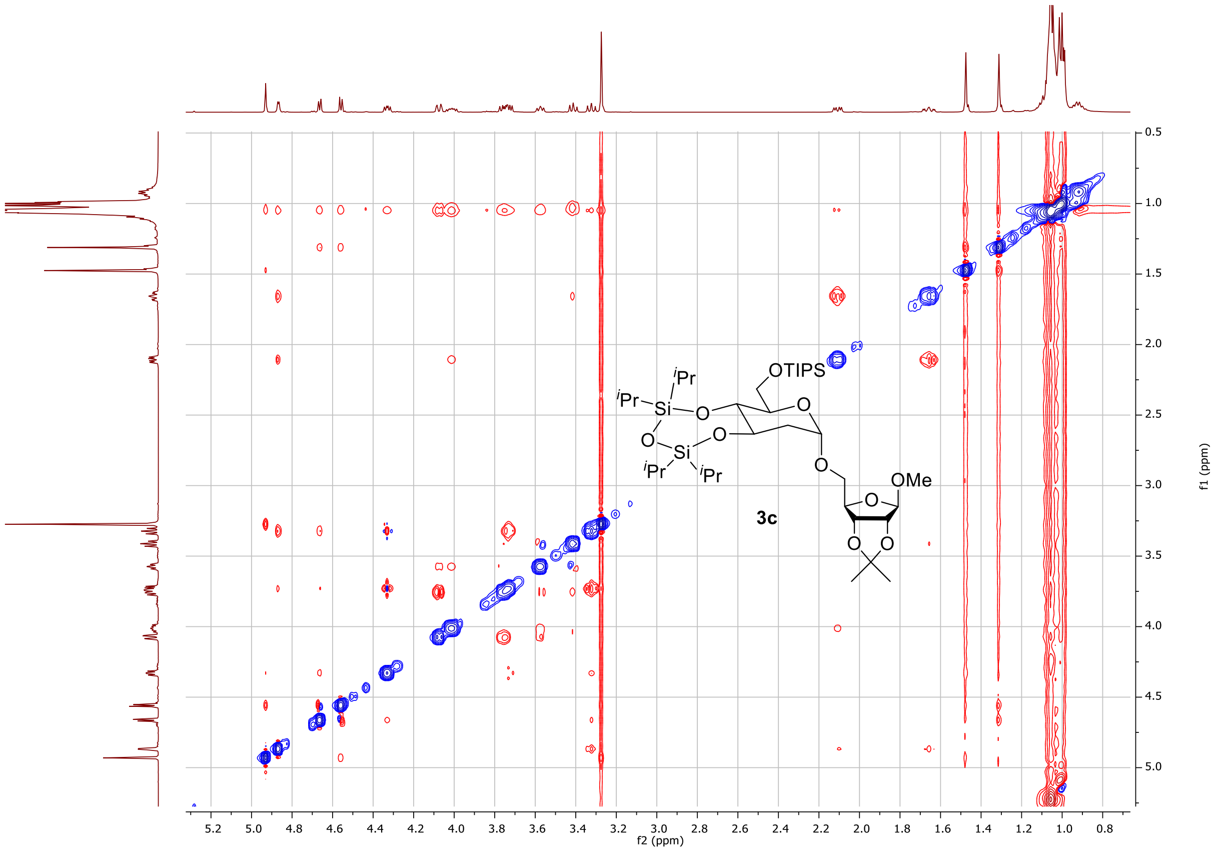

Supplementary figure S133: NOESY spectra for **3c**

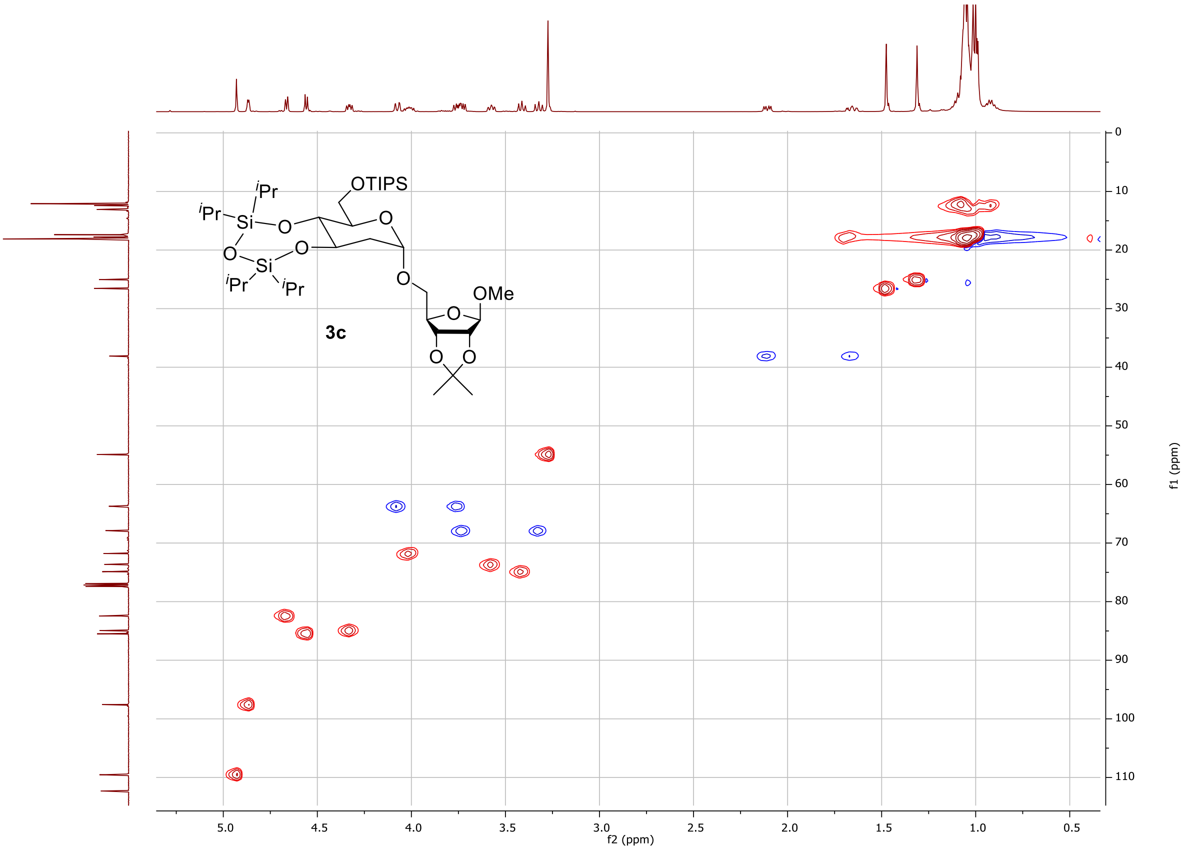

Supplementary figure S134: HSQC spectra for **3c**

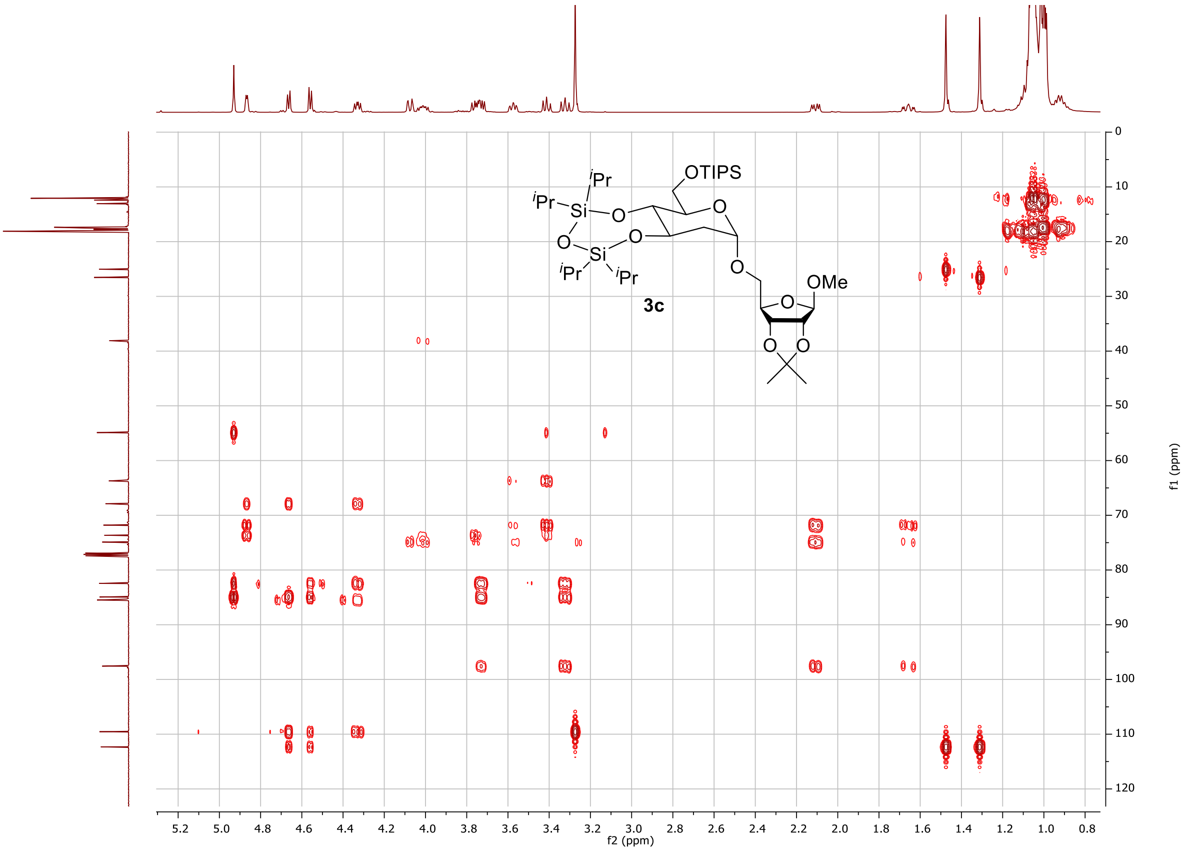

Supplementary figure S135: HMBC spectra for **3c**

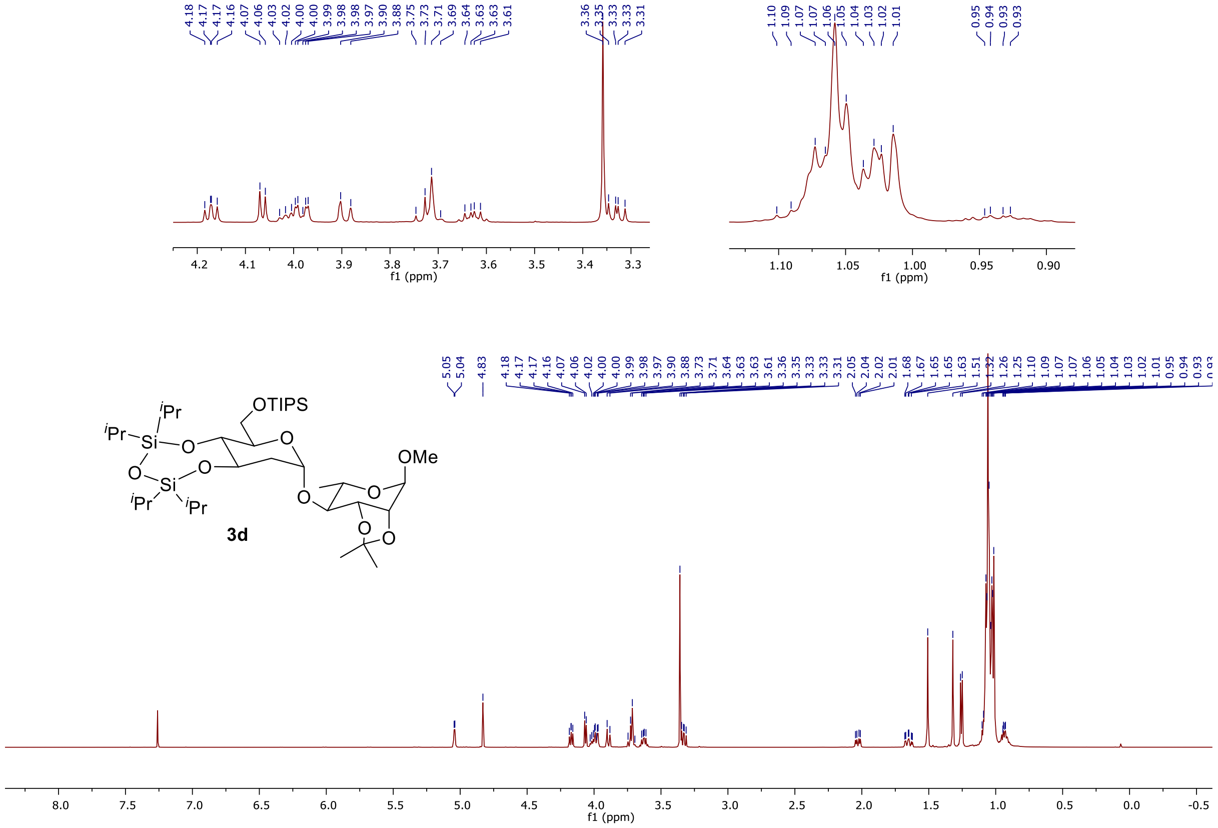

Supplementary figure S136: ^{1}H spectra for **3d**

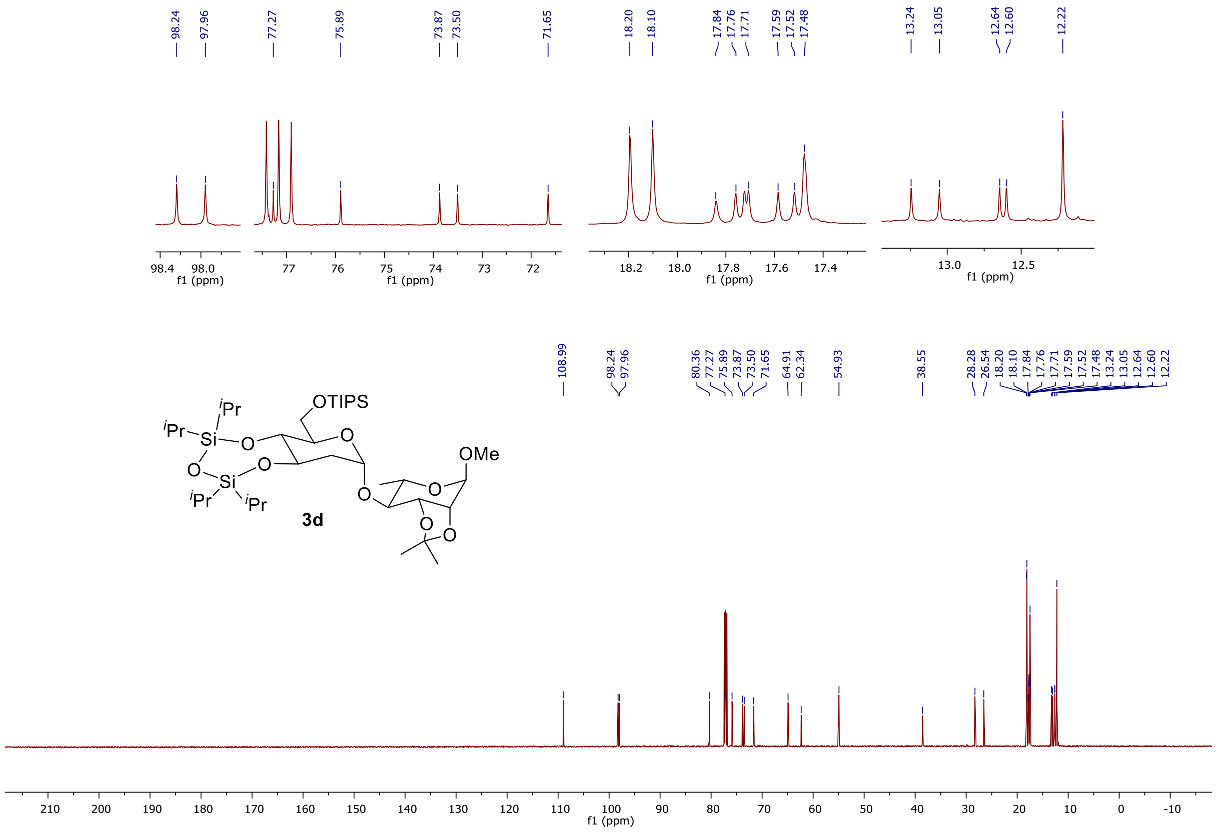

Supplementary figure S137: ^{13}C spectra for **3d**

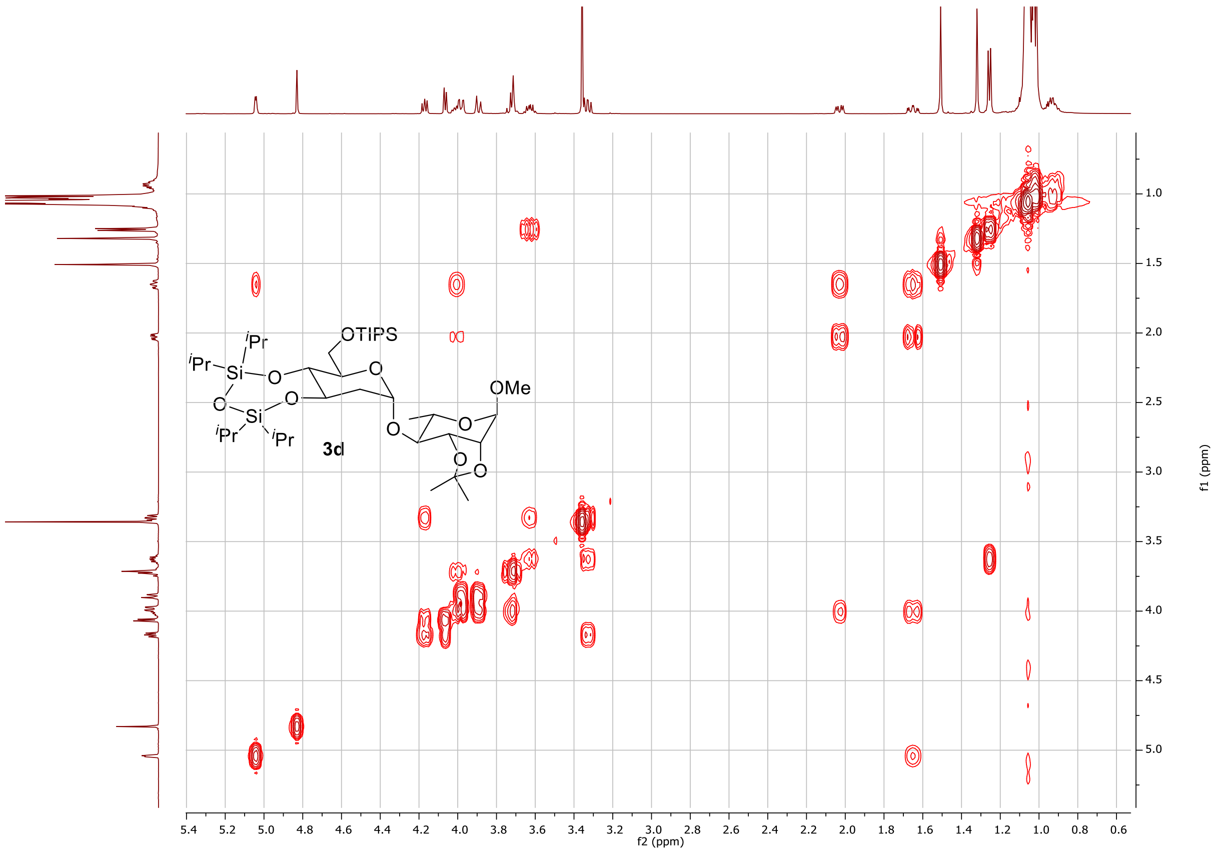

Supplementary figure S138: COSY spectra for **3d**

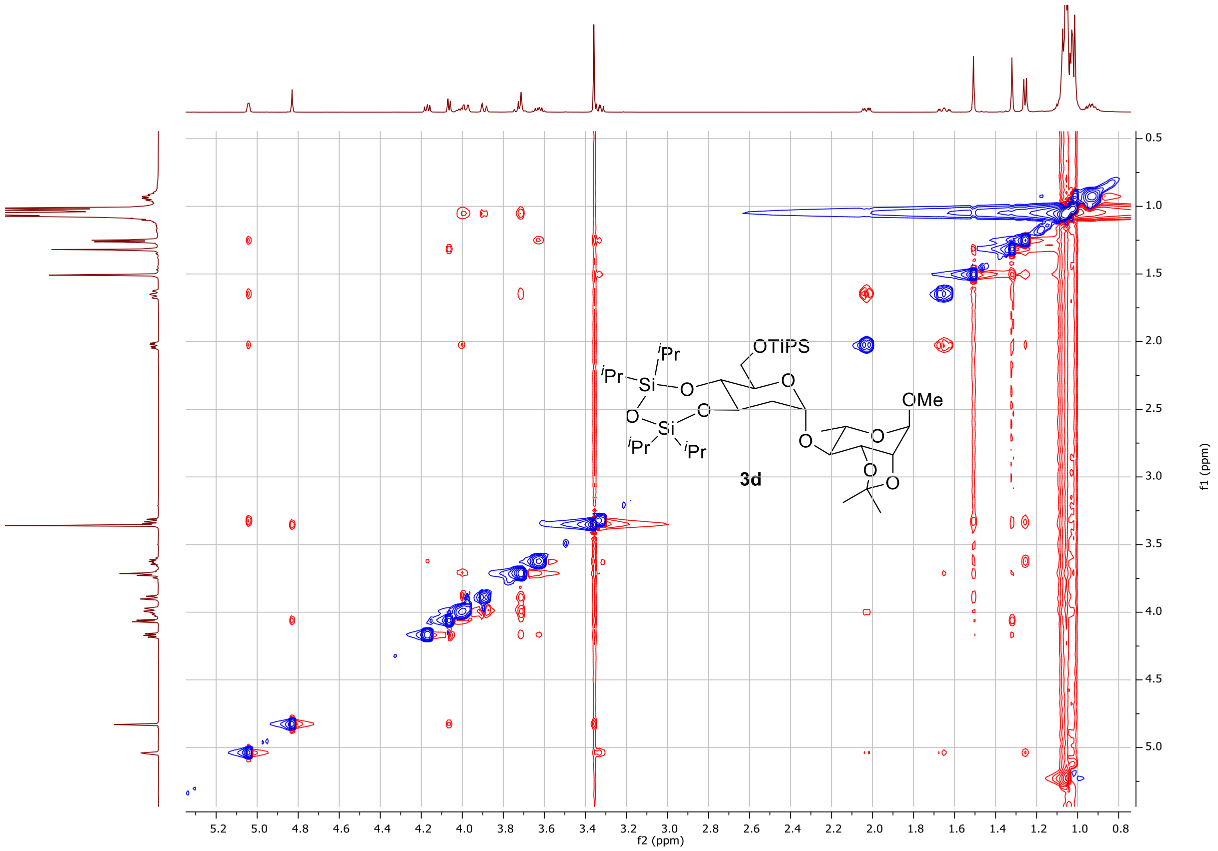

Supplementary figure S139: NOESY spectra for **3d**

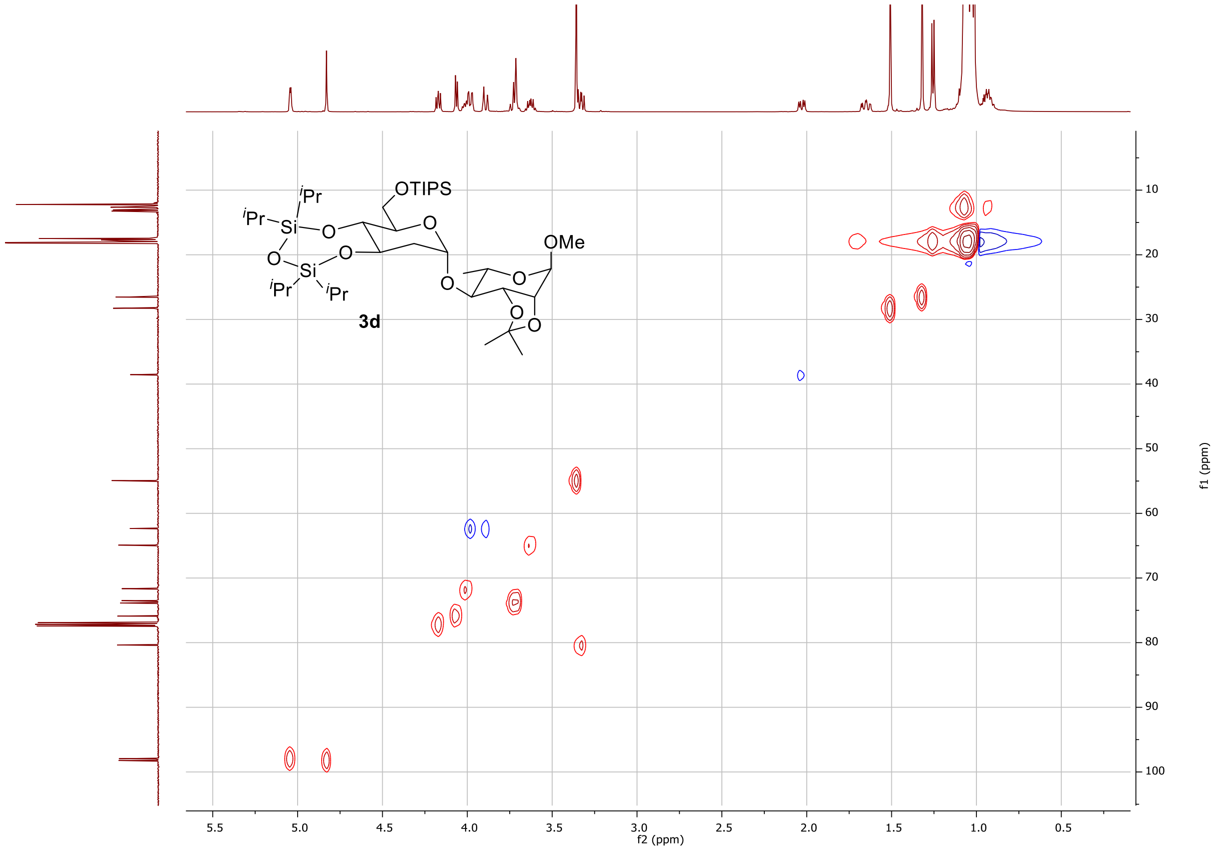

Supplementary figure S140: HSQC spectra for **3d**

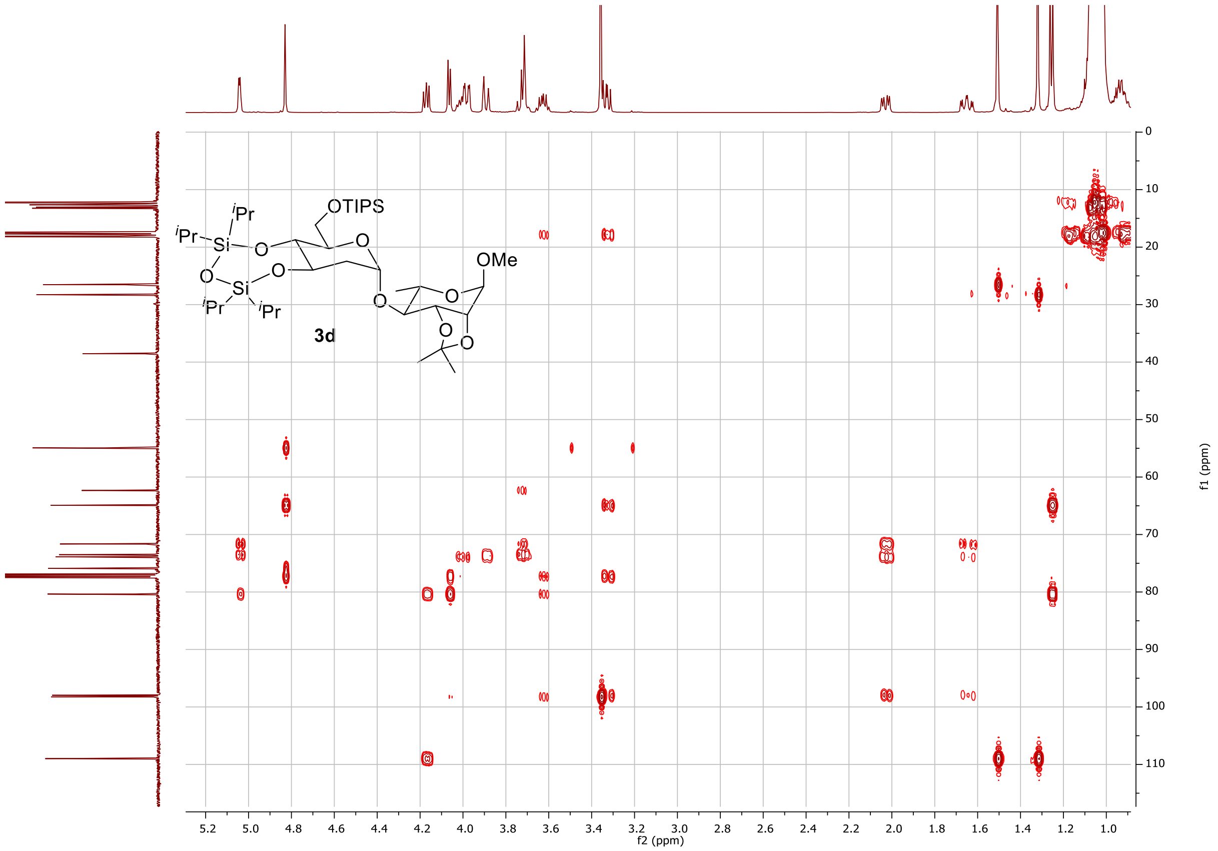

Supplementary figure S141: HMBC spectra for **3d**

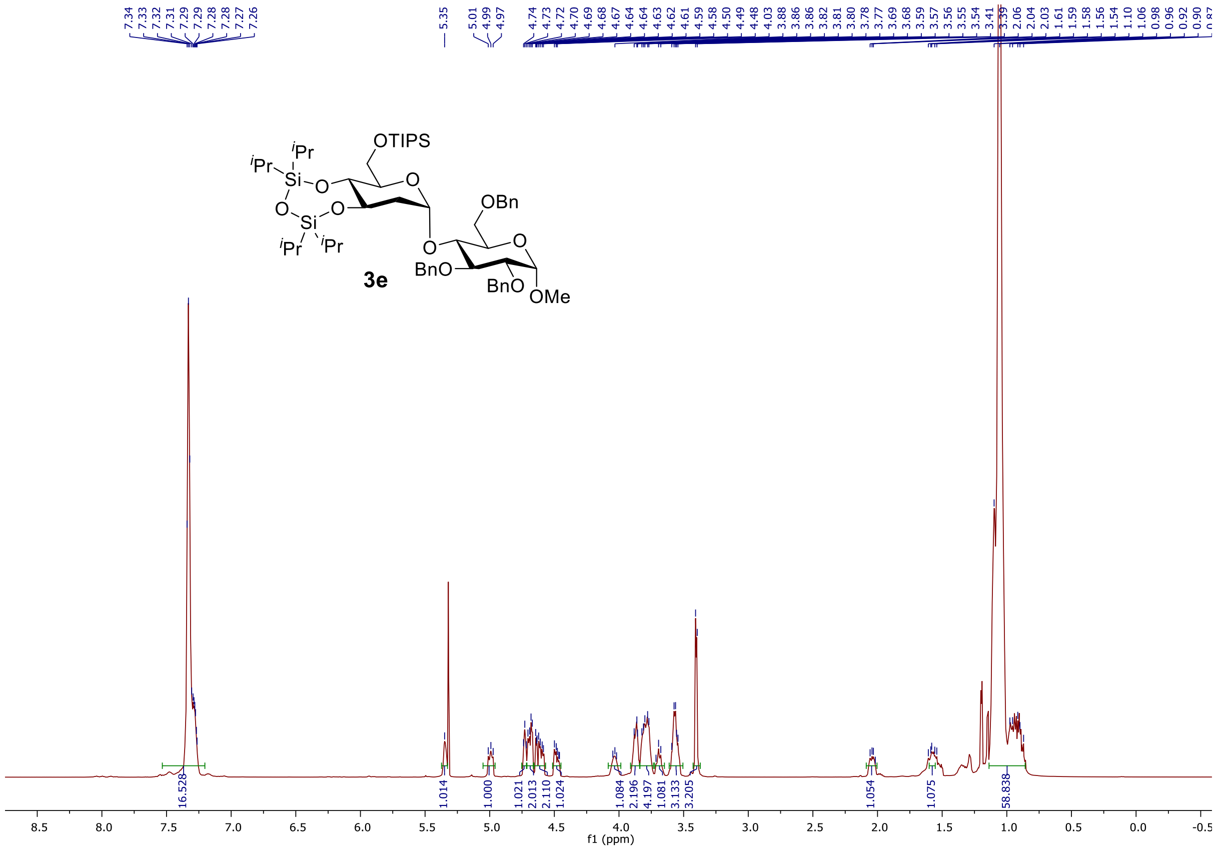

Supplementary figure S142: ^{1}H spectra for **3e**

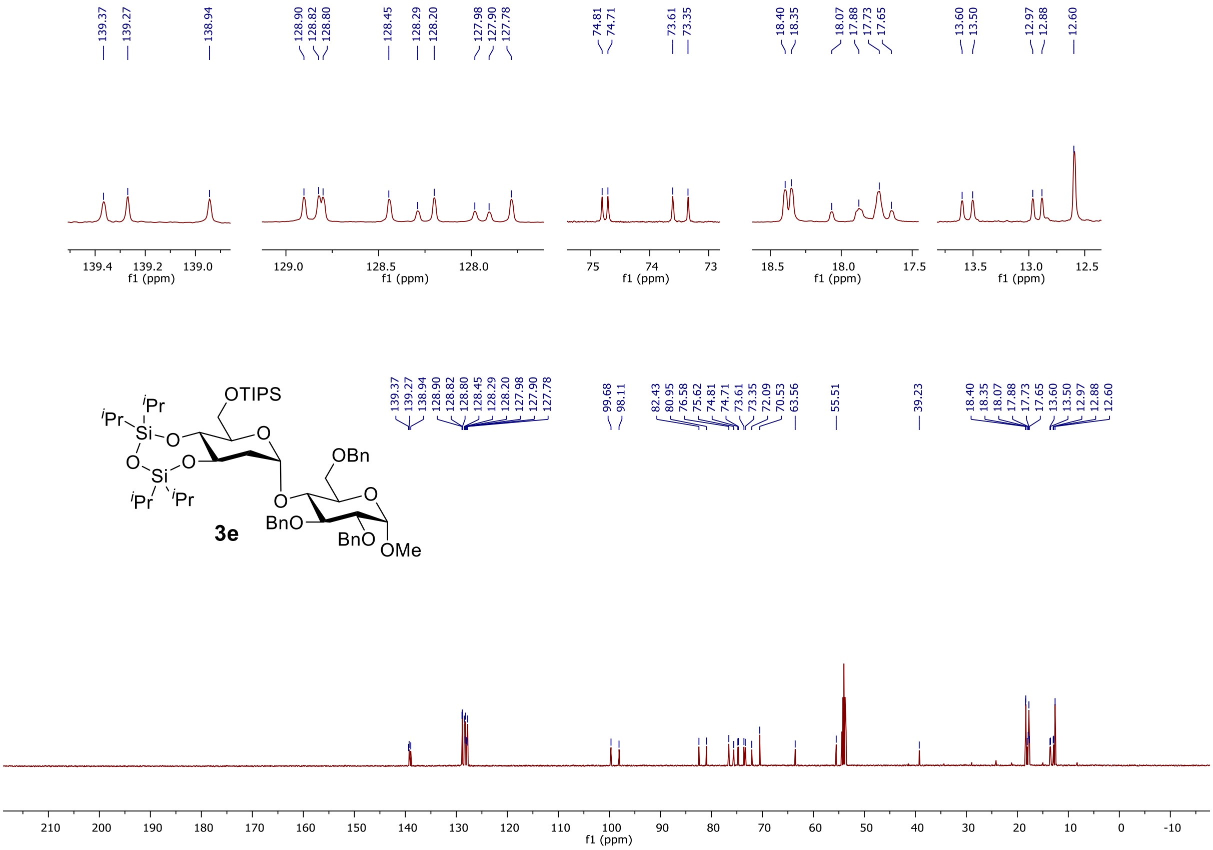

Supplementary figure S143: ^{13}C spectra for **3e**

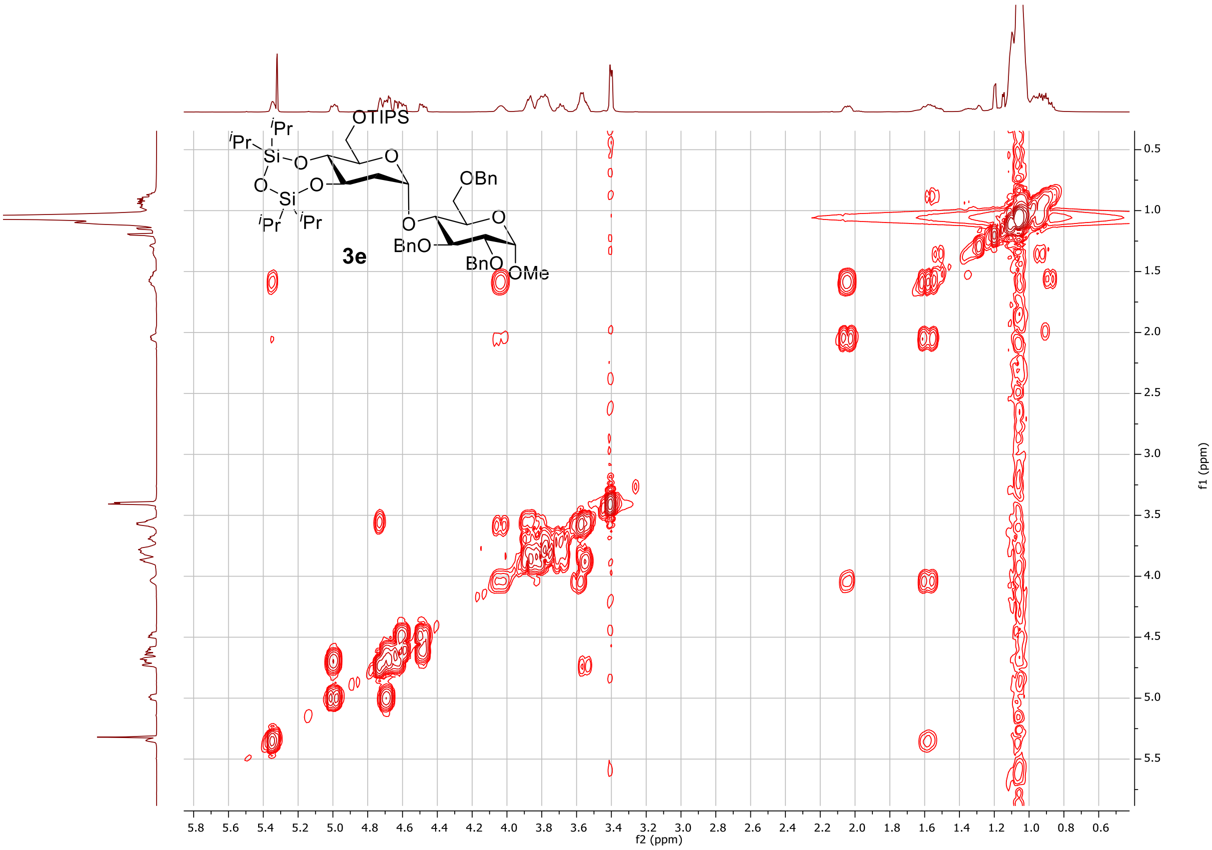

Supplementary figure S144: COSY spectra for **3e**

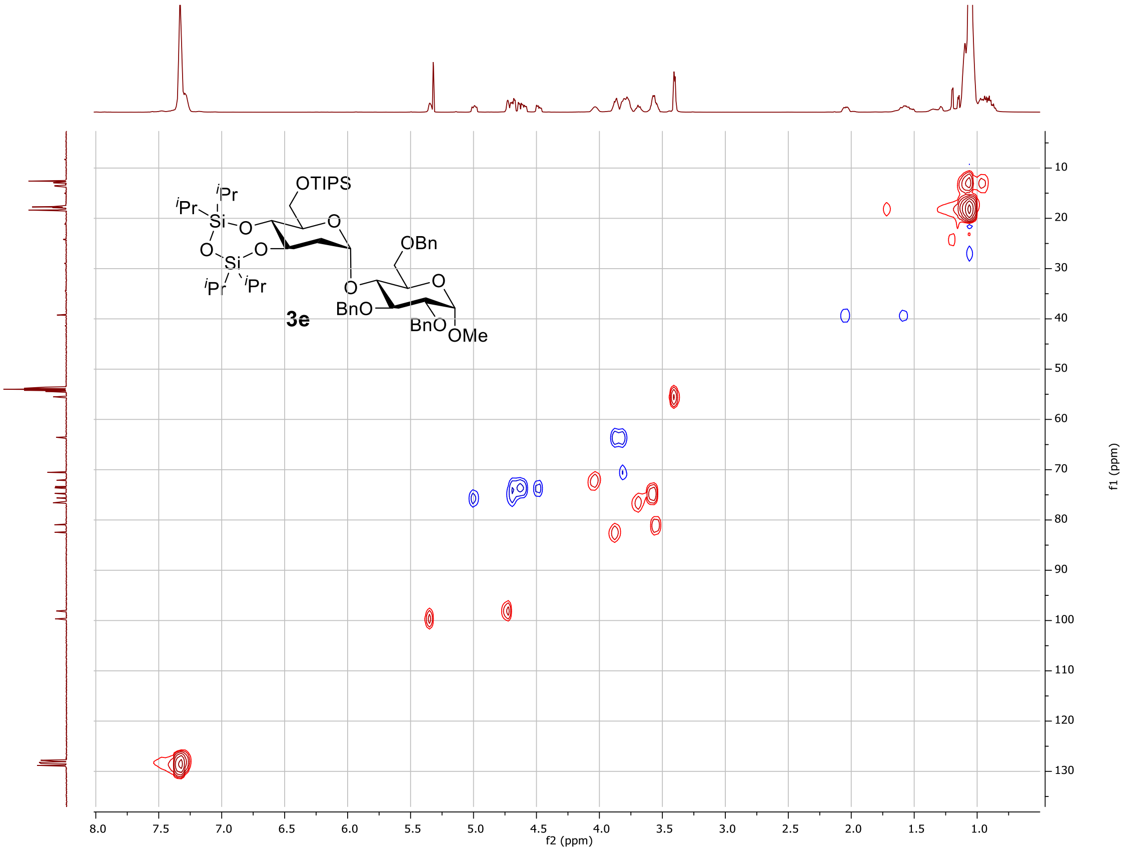

Supplementary figure S145: HSQC spectra for **3e**

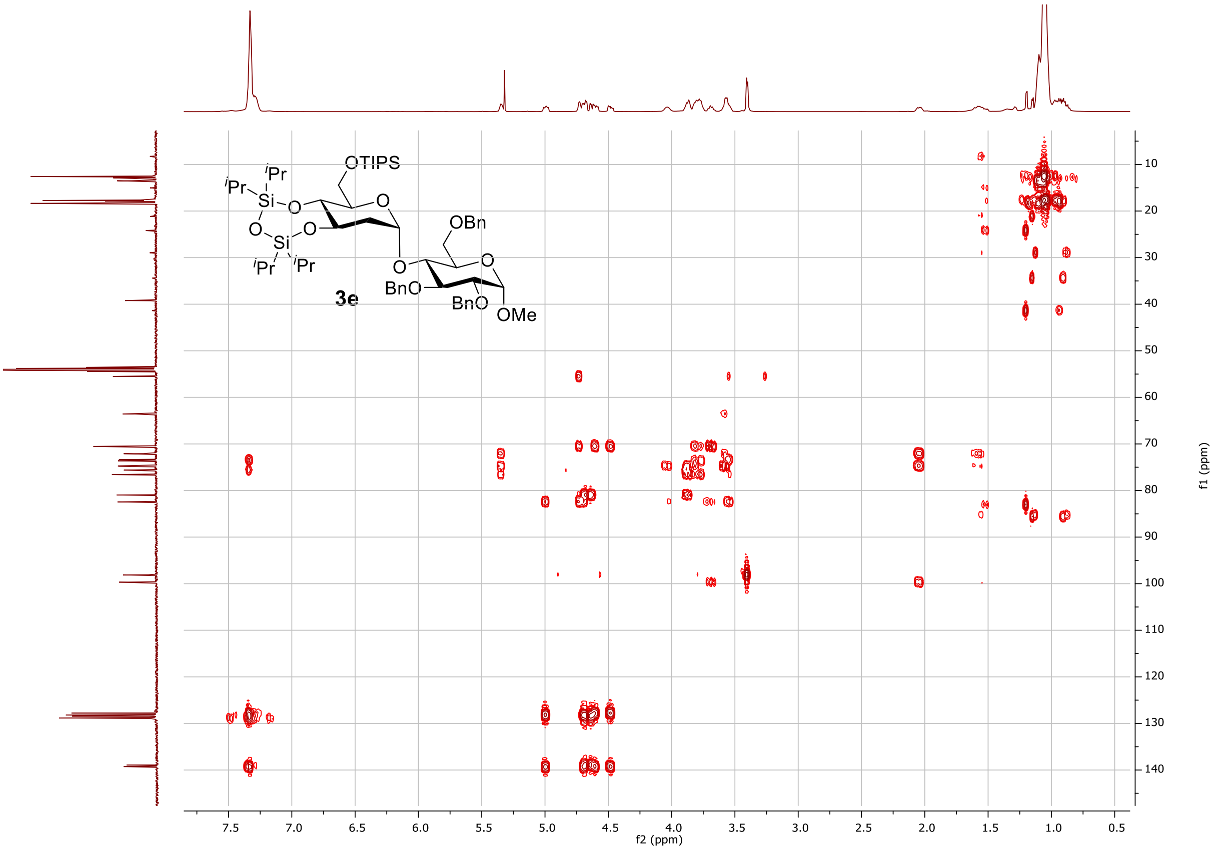

Supplementary figure S146: HMBC spectra for **3e**

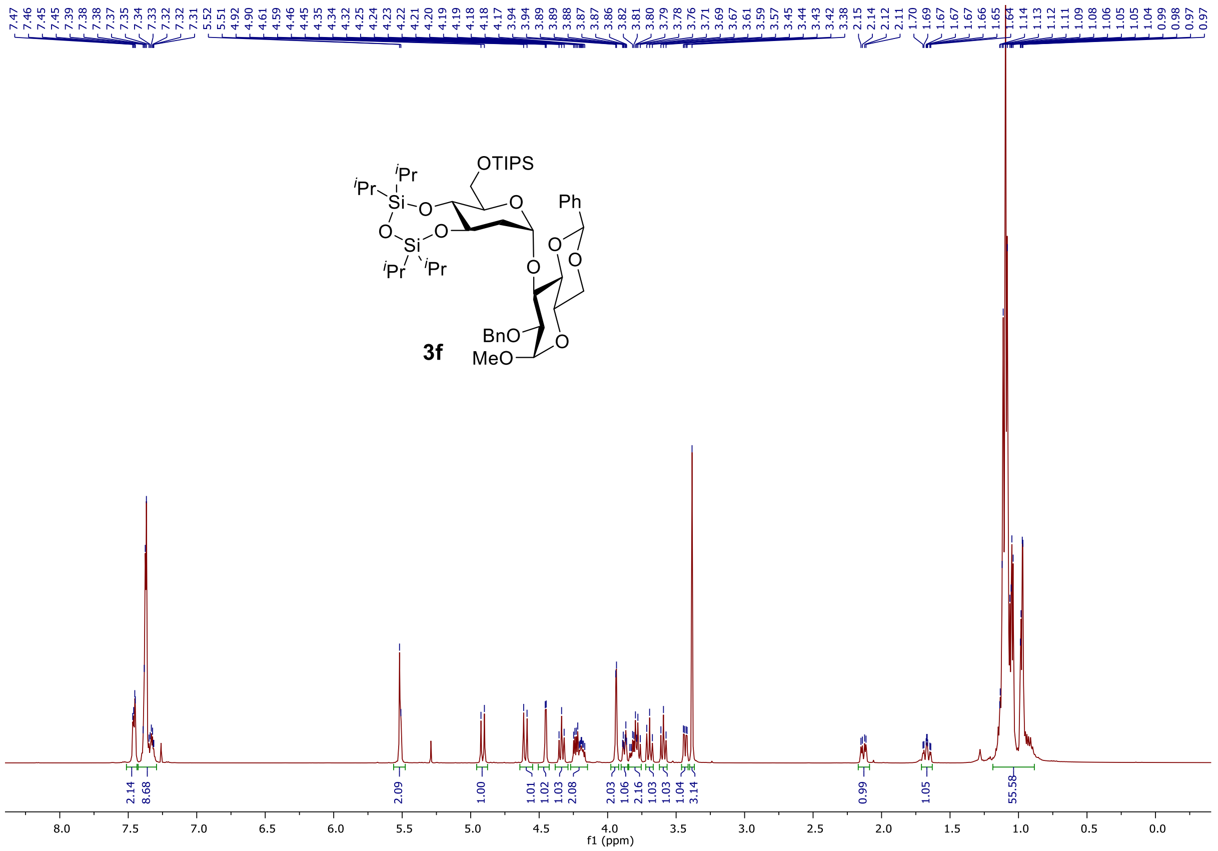

Supplementary figure S147: ^{1}H spectra for **3f**

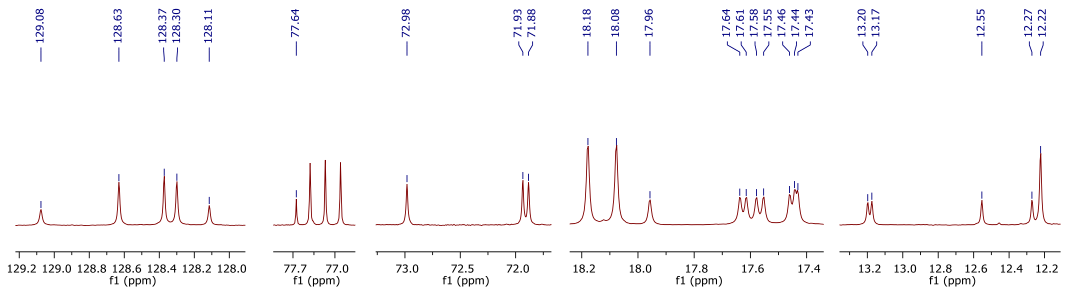

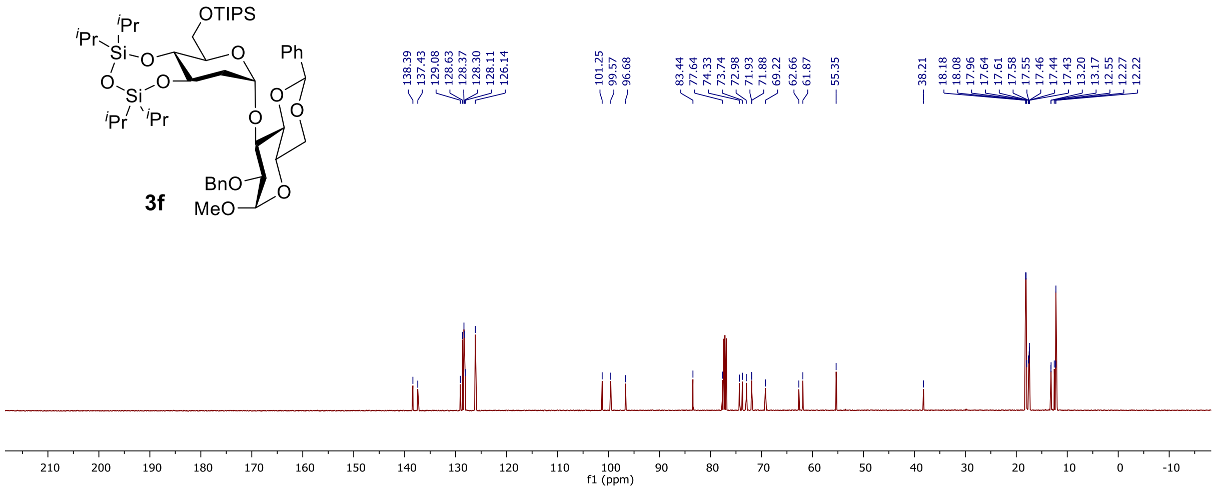

Supplementary figure S148: ^{13}C spectra for **3f**

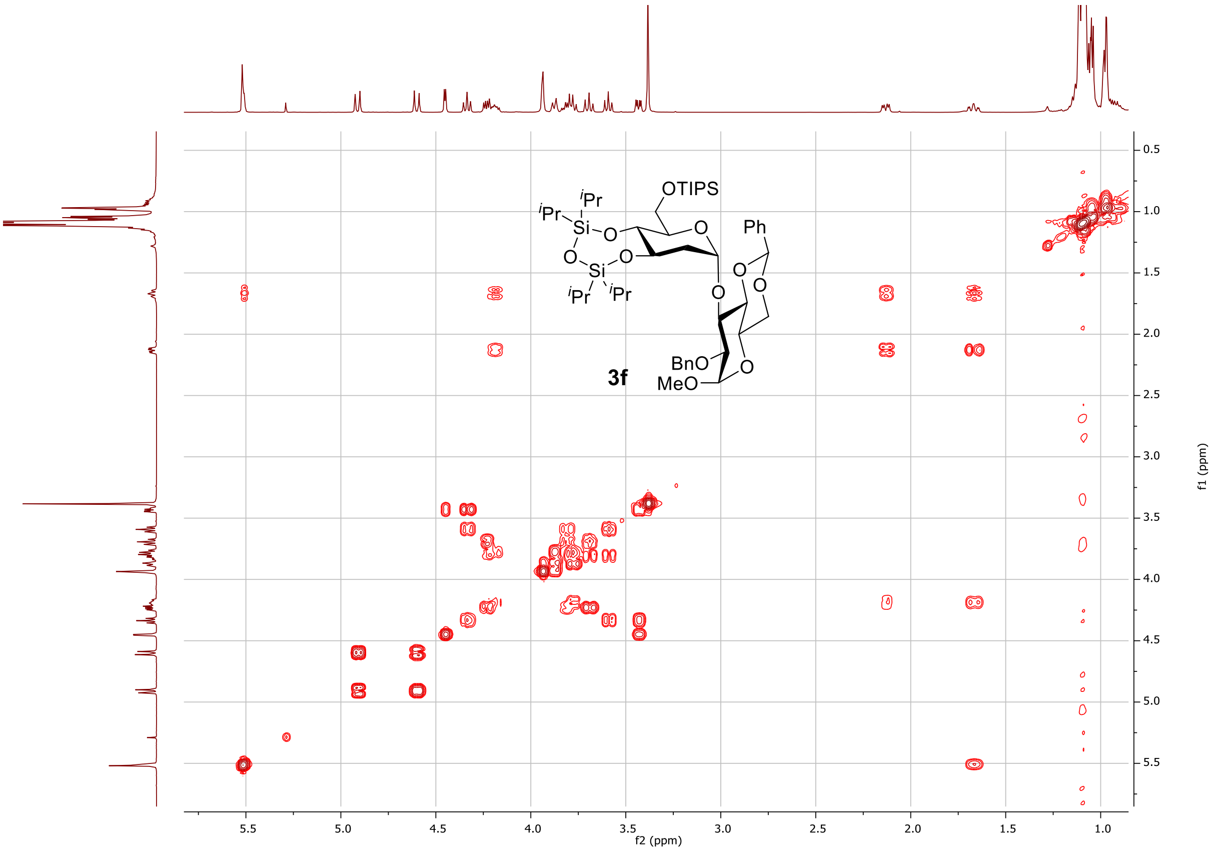

Supplementary figure S149: COSY spectra for **3f**

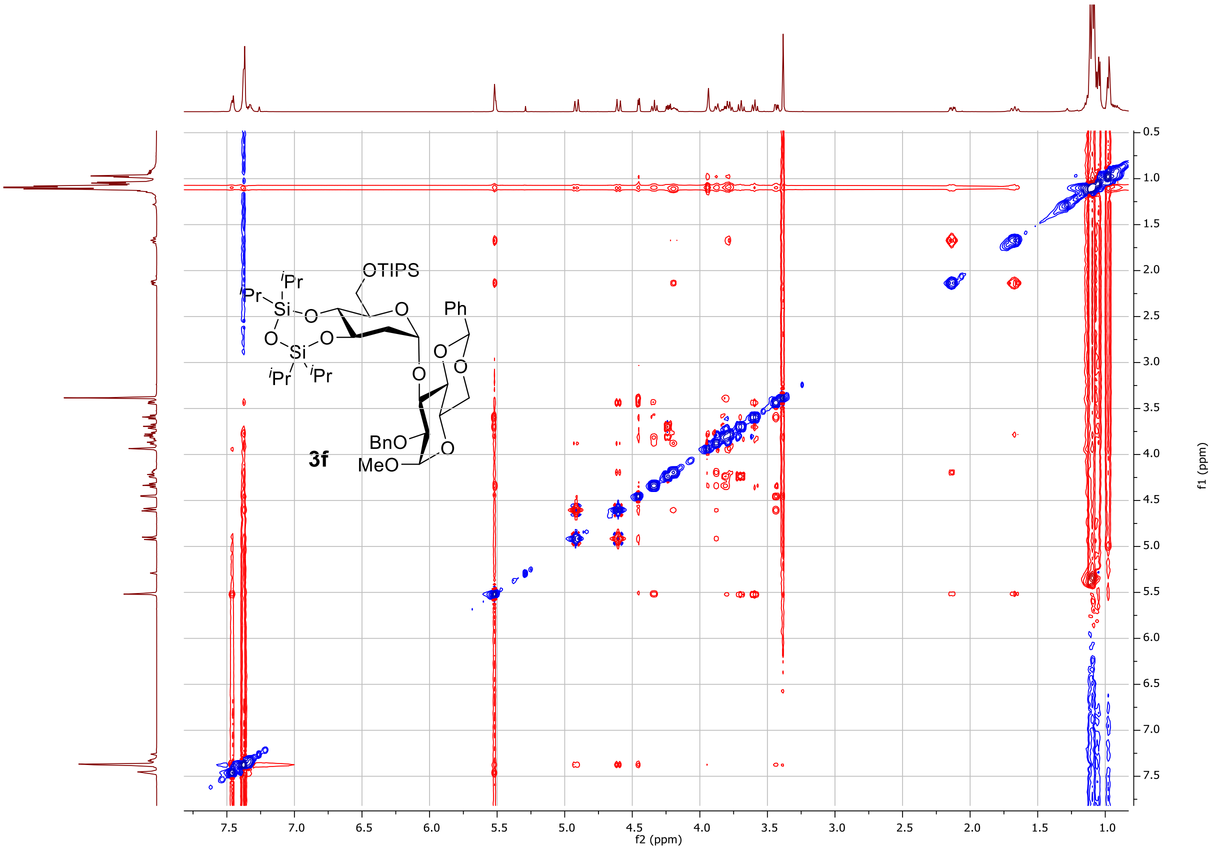

Supplementary figure S150: NOESY spectra for **3f**

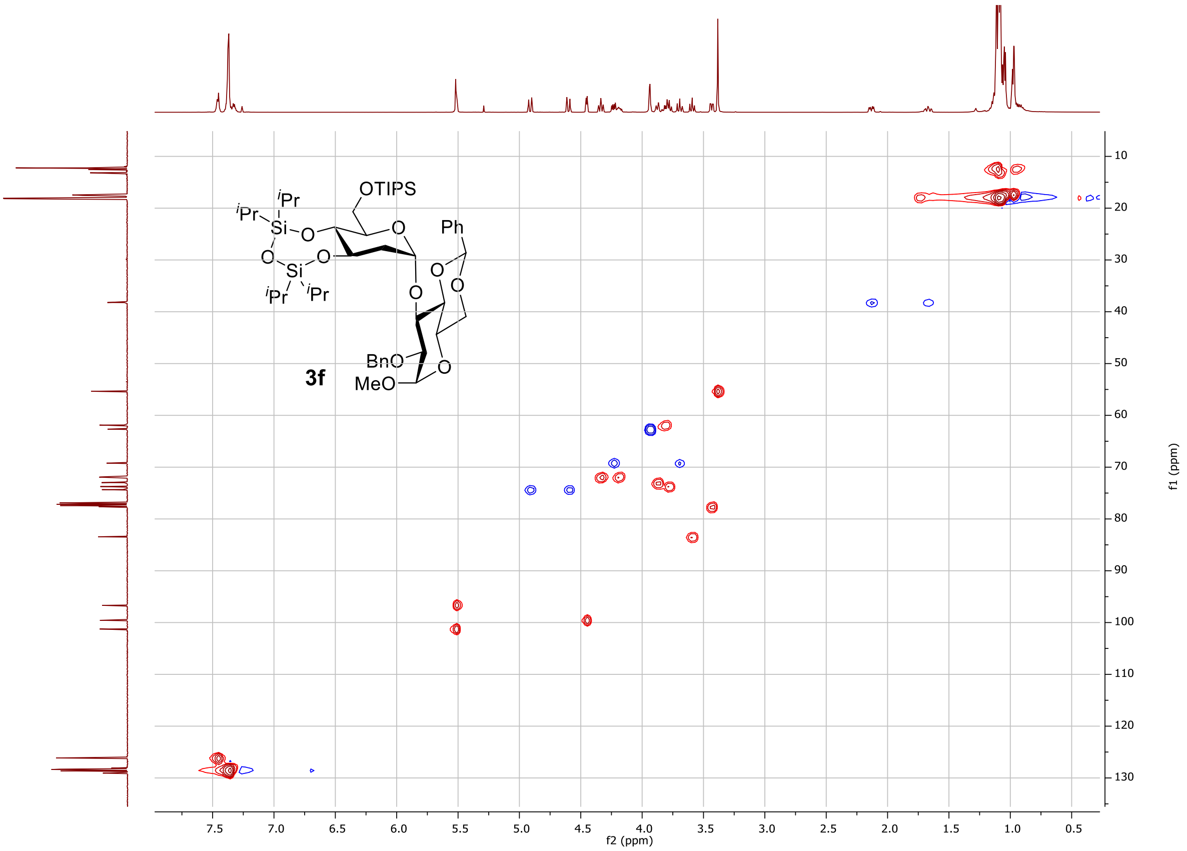

Supplementary figure S151: HSQC spectra for **3f**

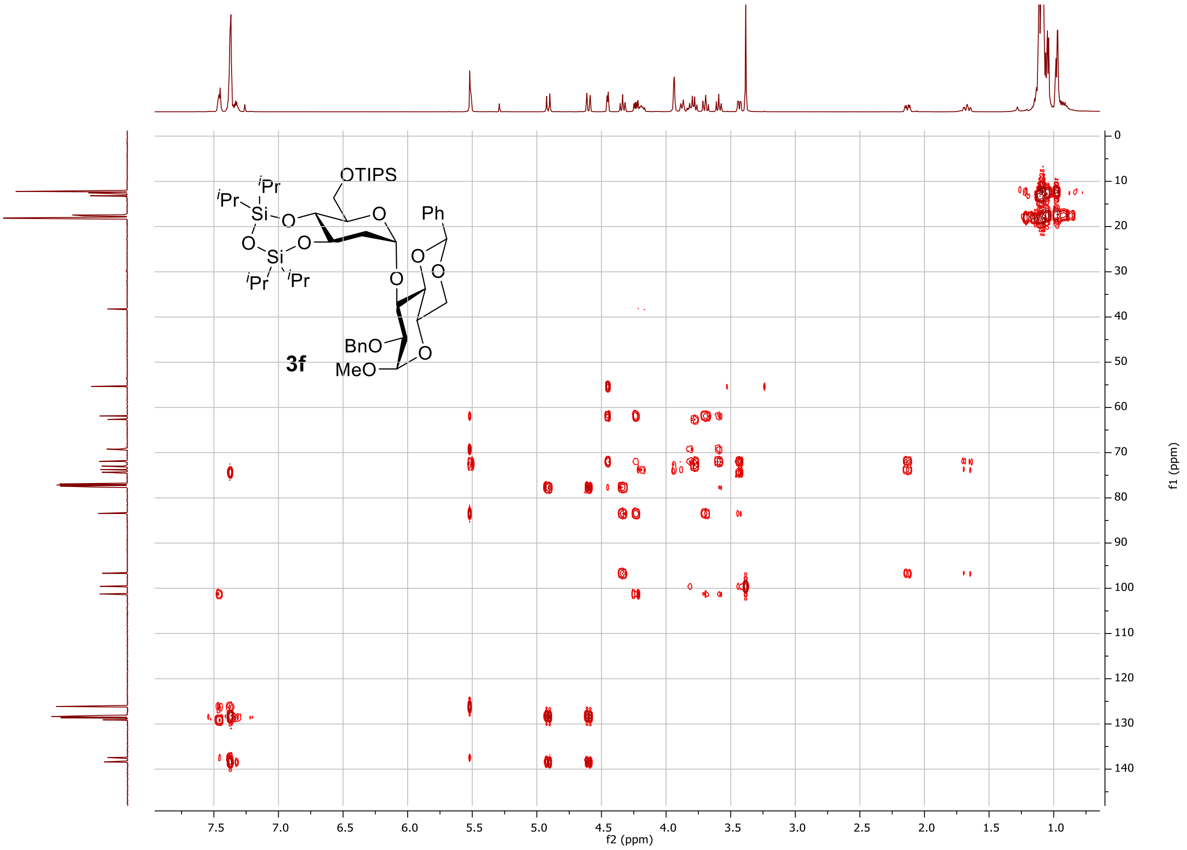

Supplementary figure S152: HMBC spectra for **3f**

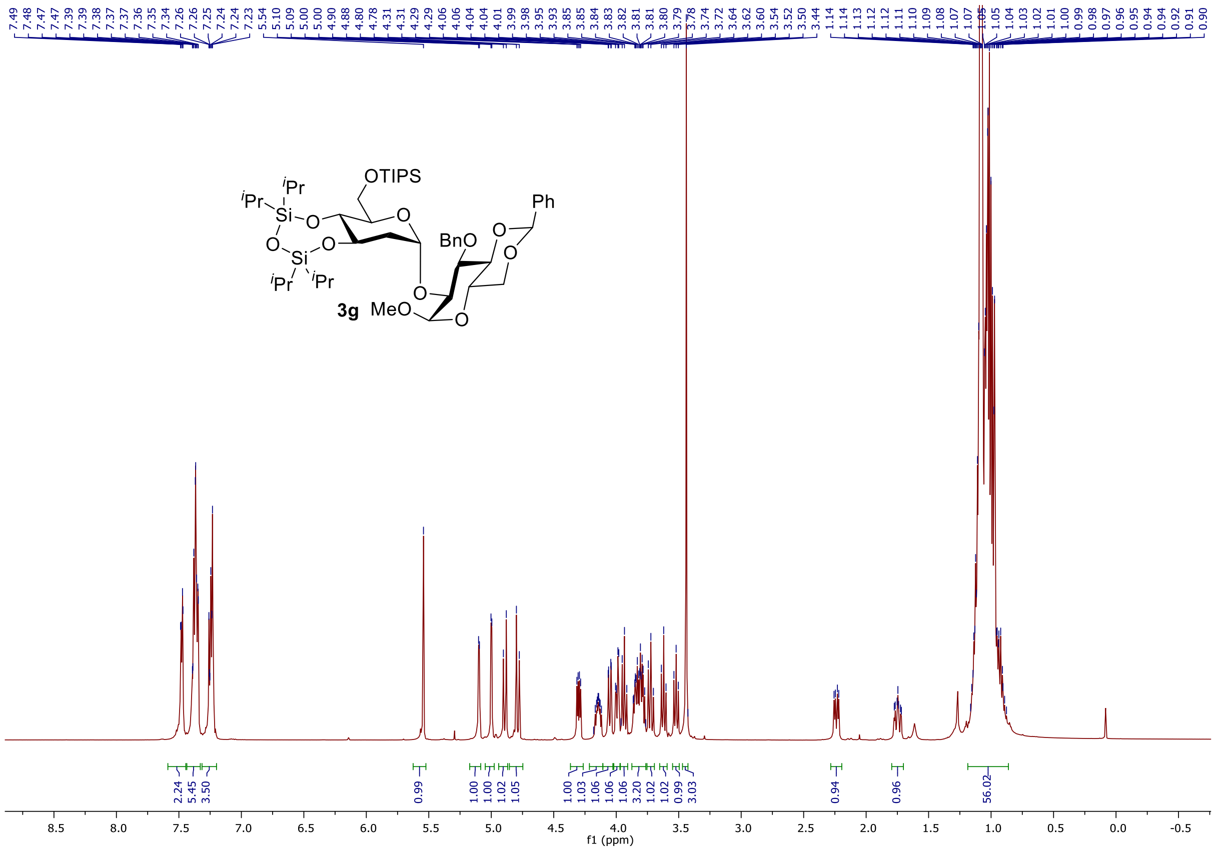

Supplementary figure S153: ^{1}H spectra for **3g**

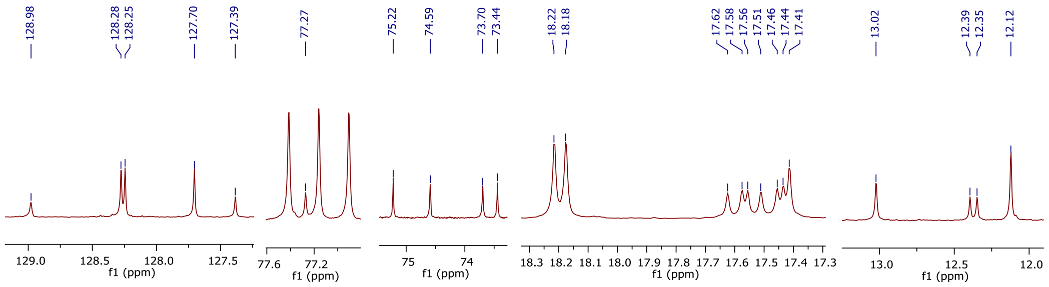

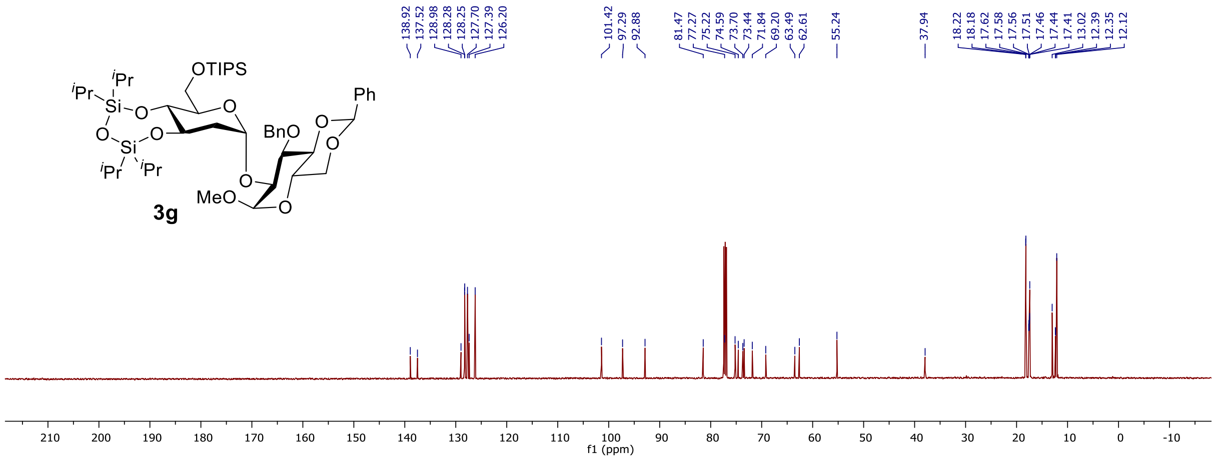

Supplementary figure S154: ^{13}C spectra for **3g**

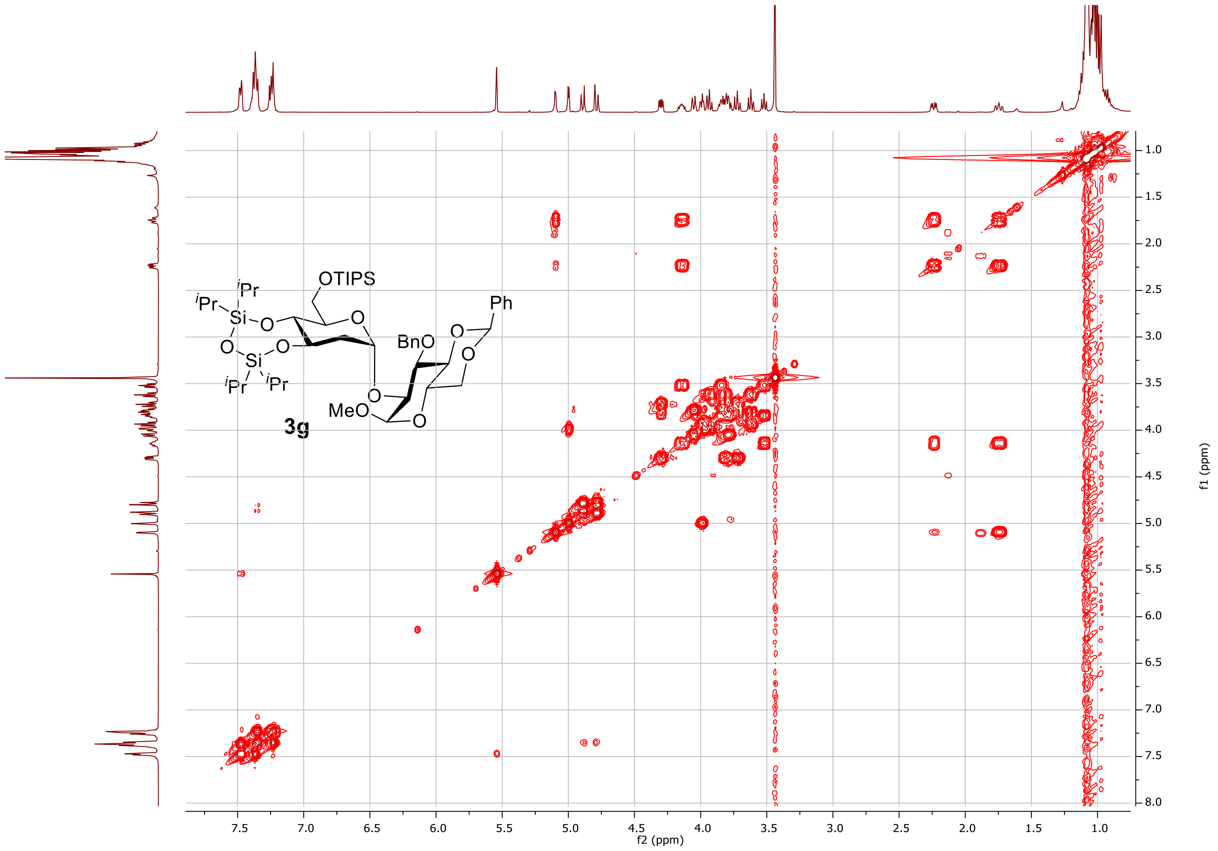

Supplementary figure S155: COSY spectra for **3g**

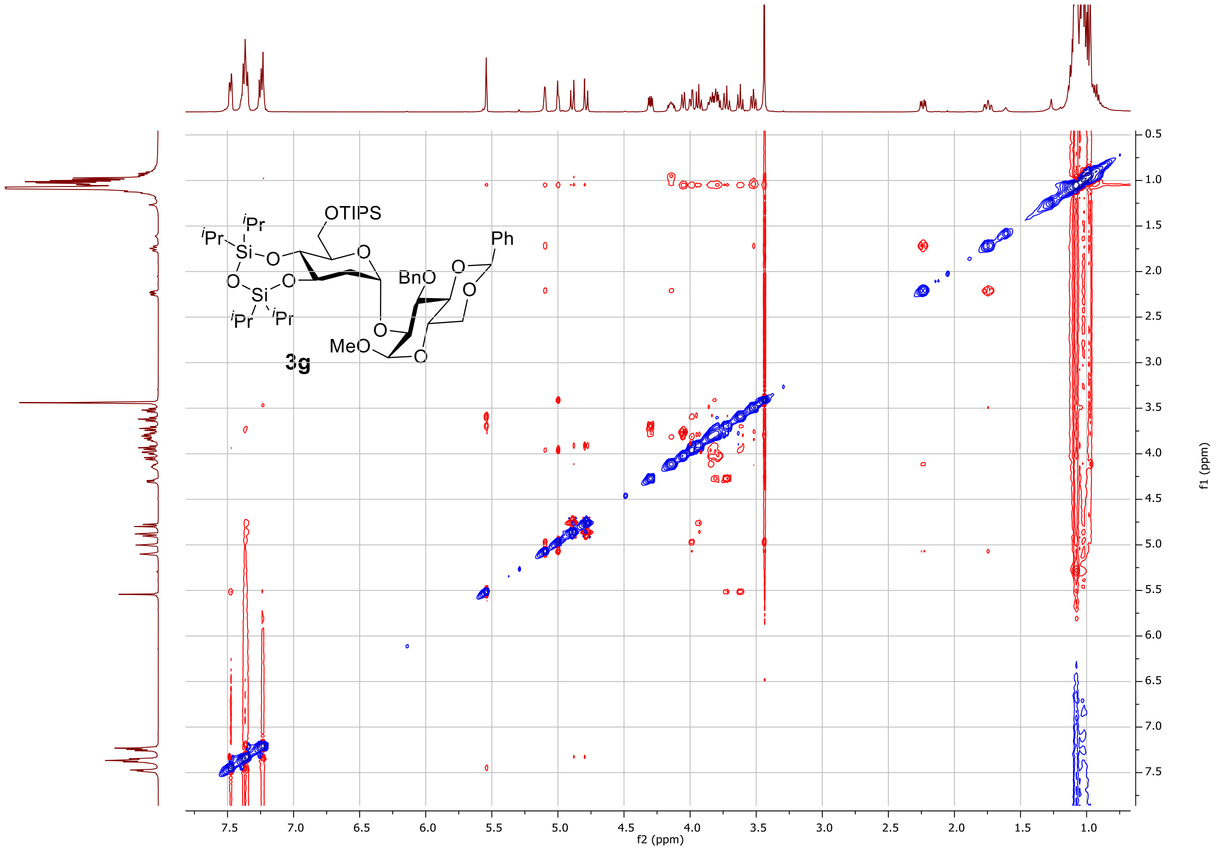

Supplementary figure S156: NOESY spectra for **3g**

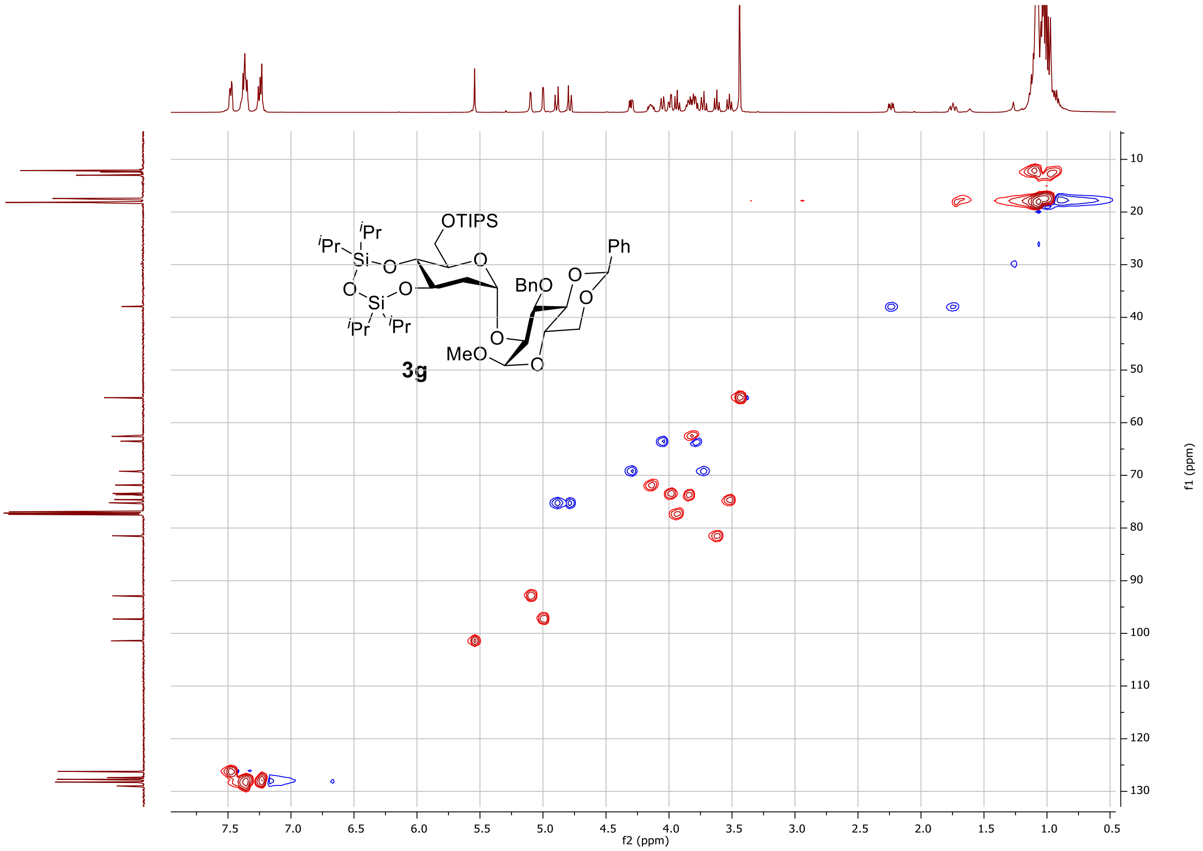

Supplementary figure S157: HSQC spectra for **3g**

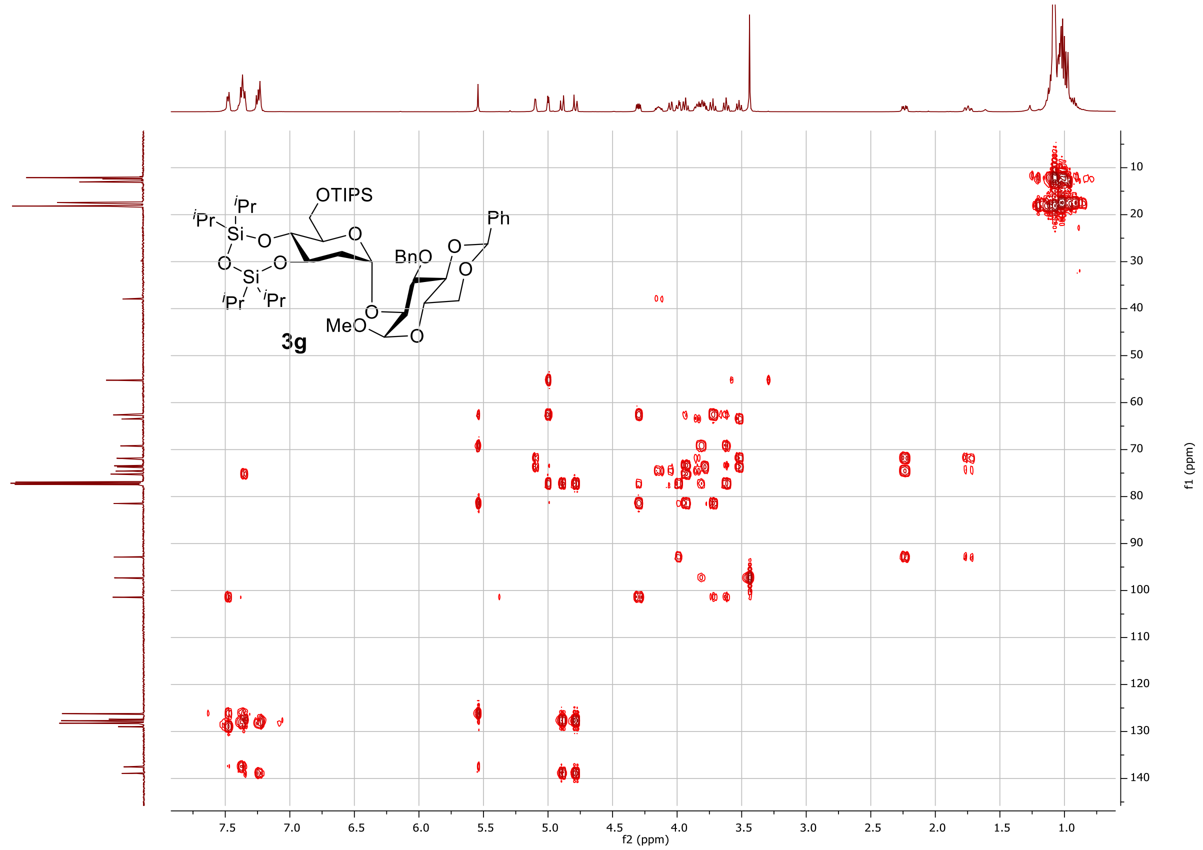

Supplementary figure S158: HMBC spectra for **3g**

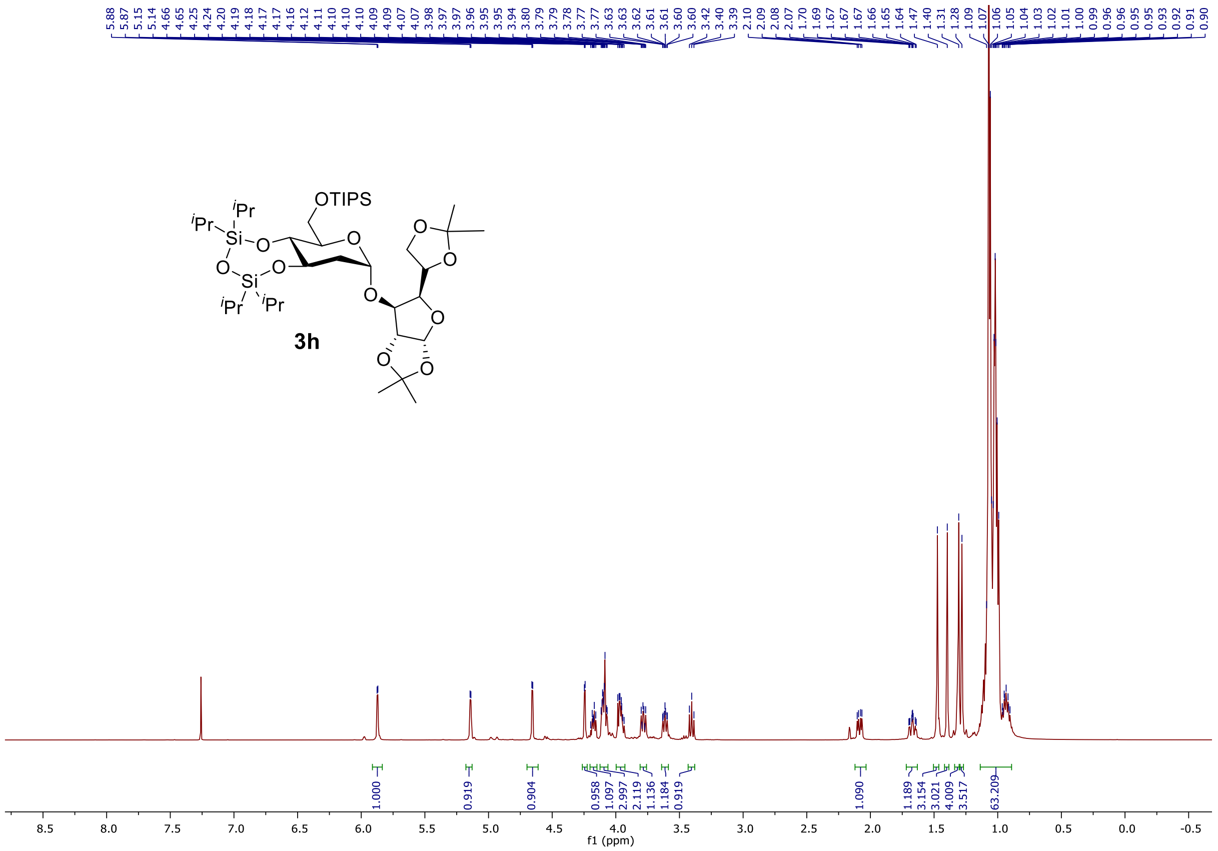

Supplementary figure S159: ^{1}H spectra for **3h**

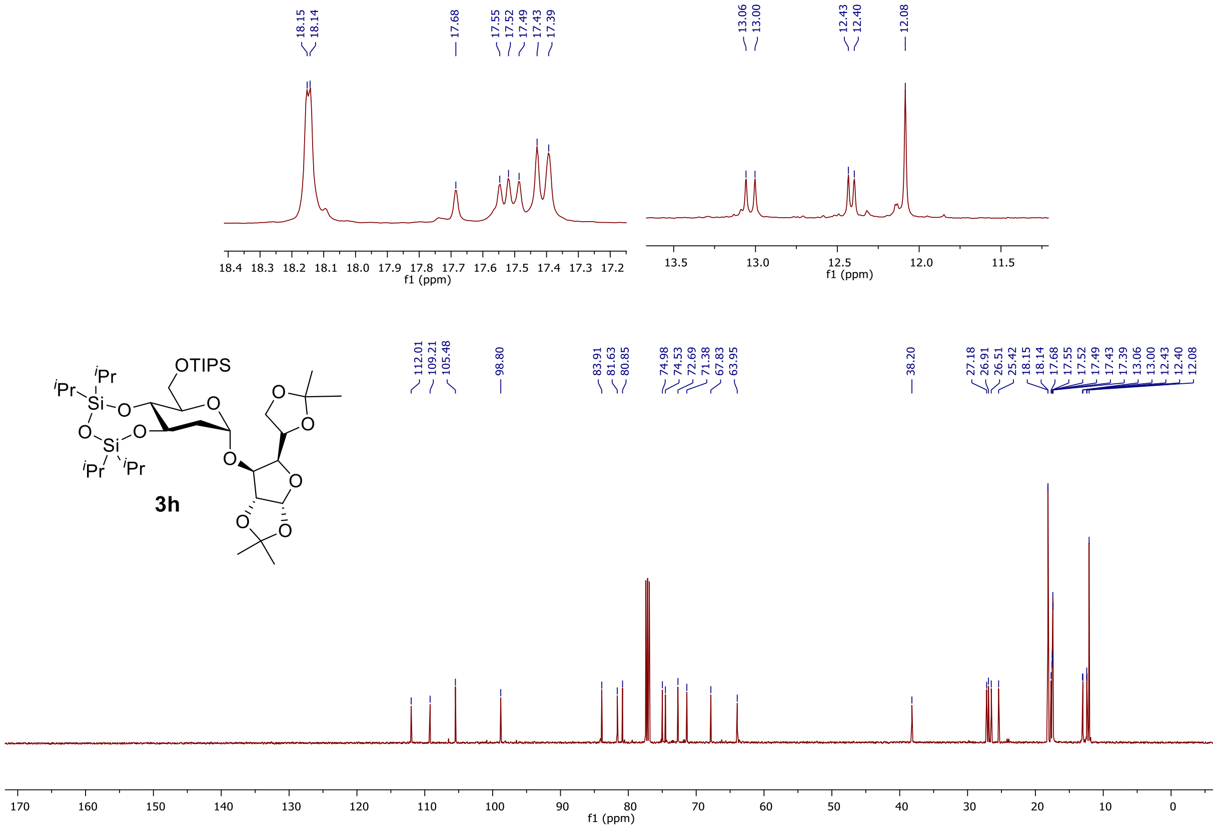

Supplementary figure S160: ^{13}C spectra for **3h**

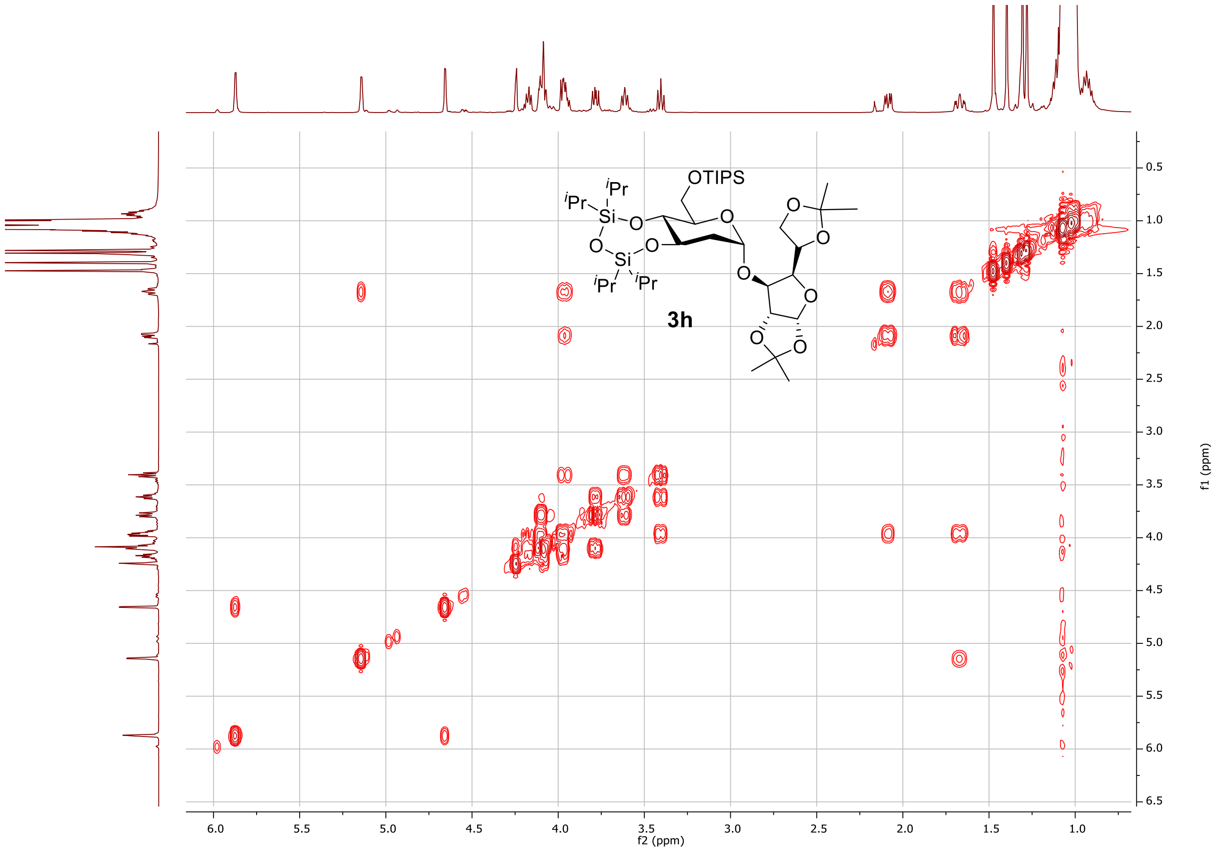

Supplementary figure S161: COSY spectra for **3h**

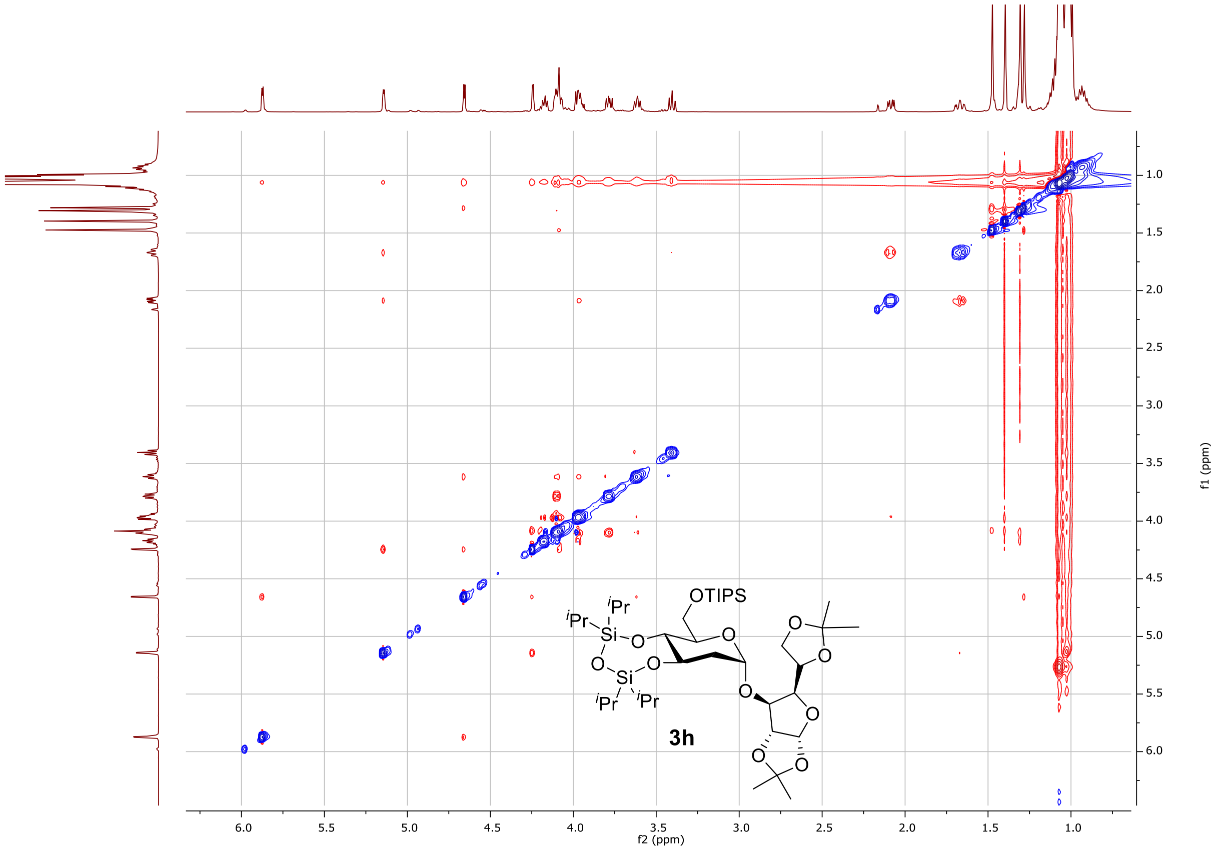

Supplementary figure S162: NOESY spectra for **3h**

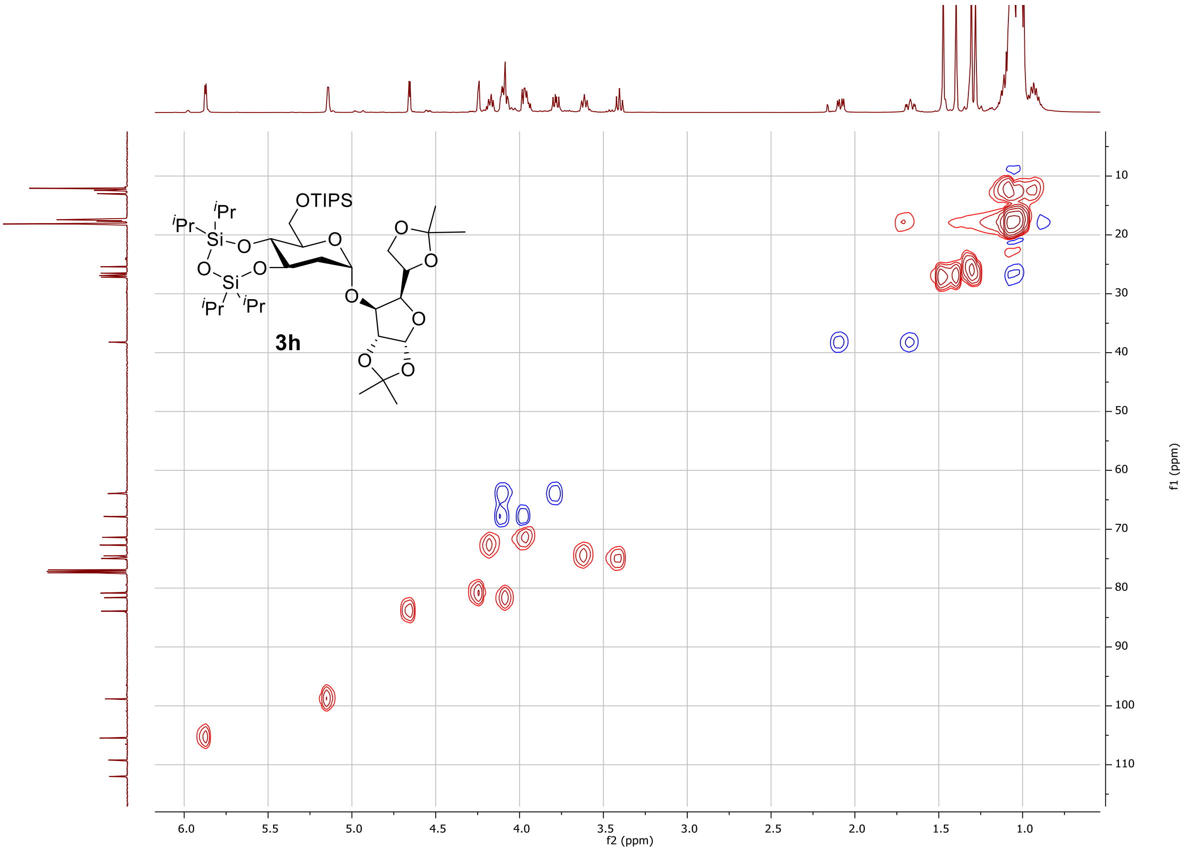

Supplementary figure S163: HSQC spectra for **3h**

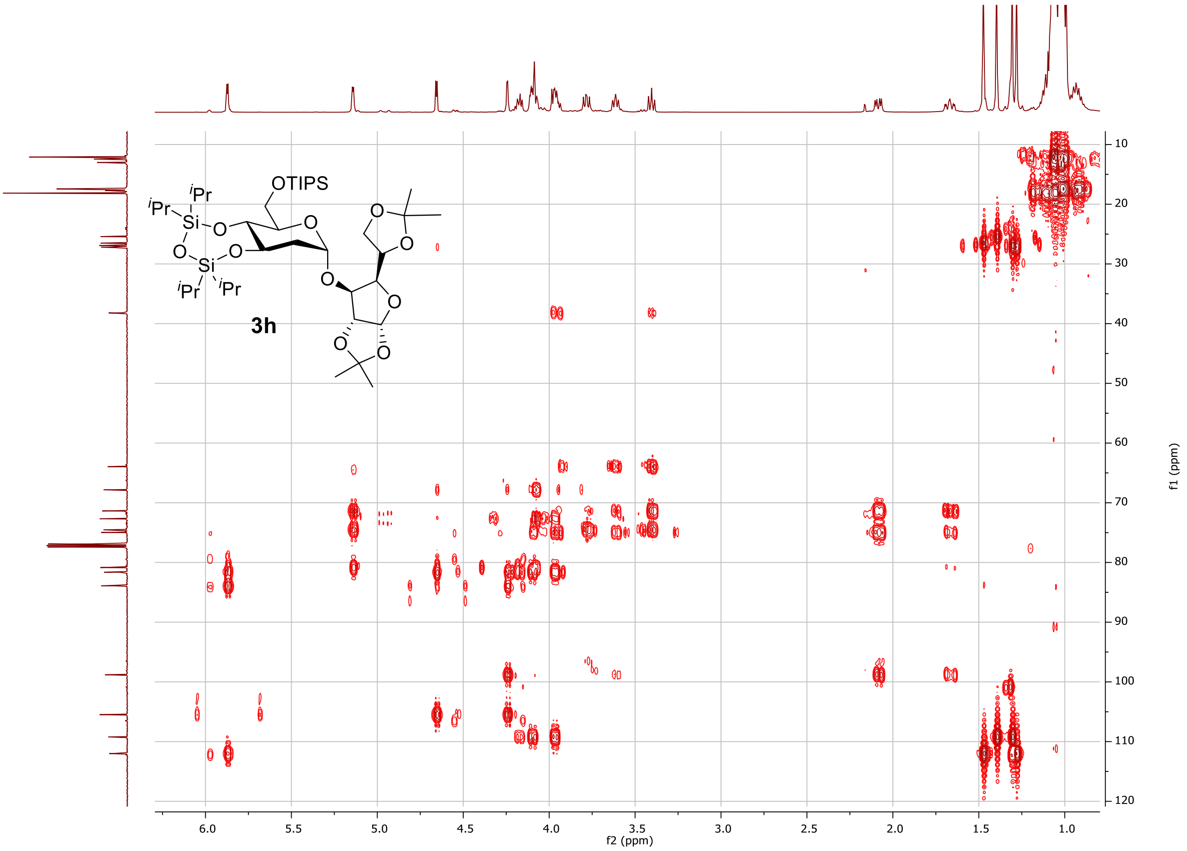

Supplementary figure S164: HMBC spectra for **3h**

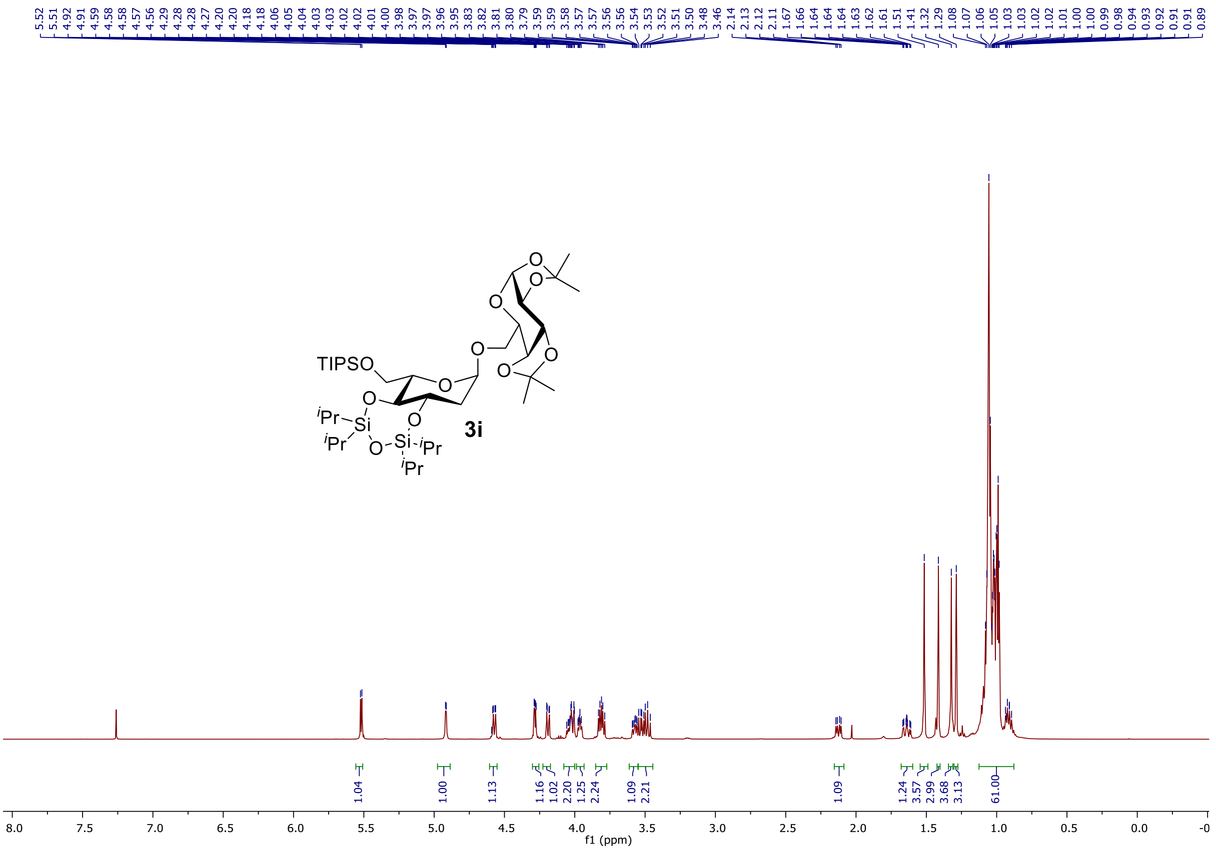

Supplementary figure S165: ^{1}H spectra for **3i**

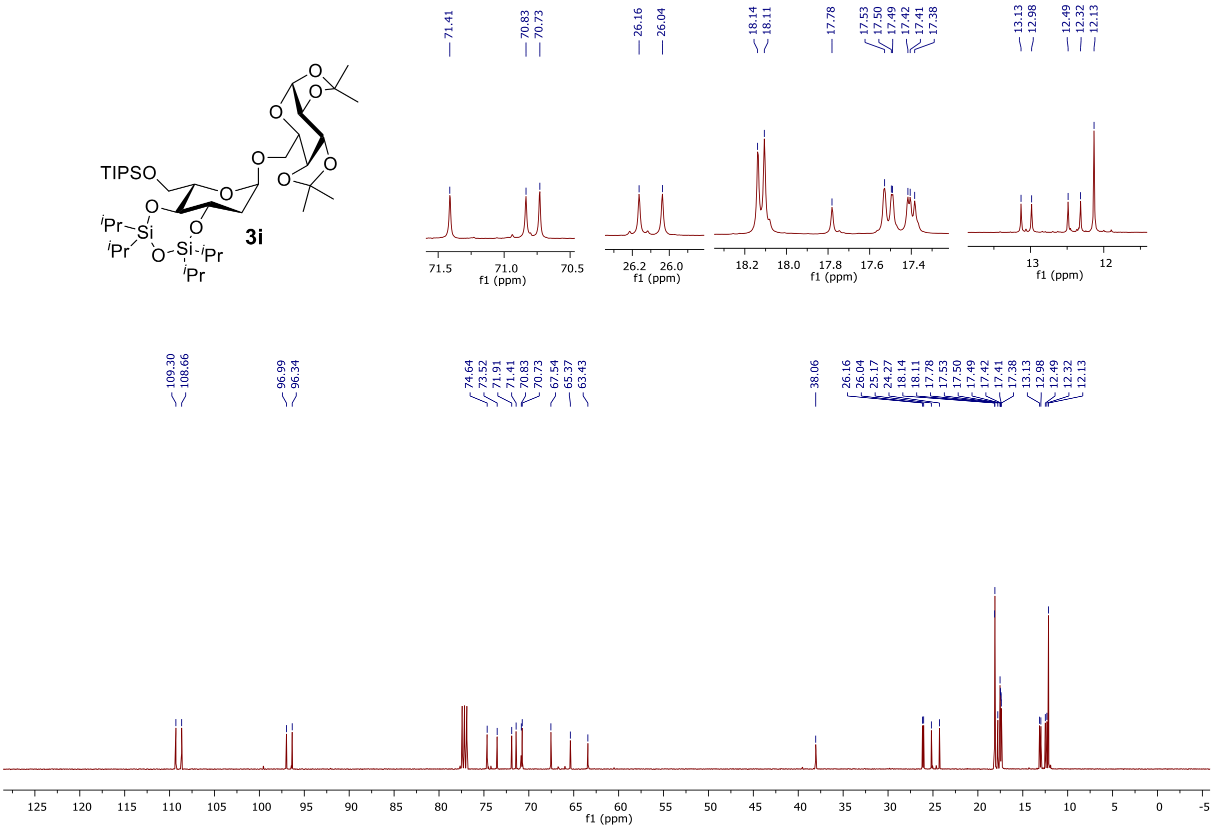

Supplementary figure S166: ^{13}C spectra for **3i**

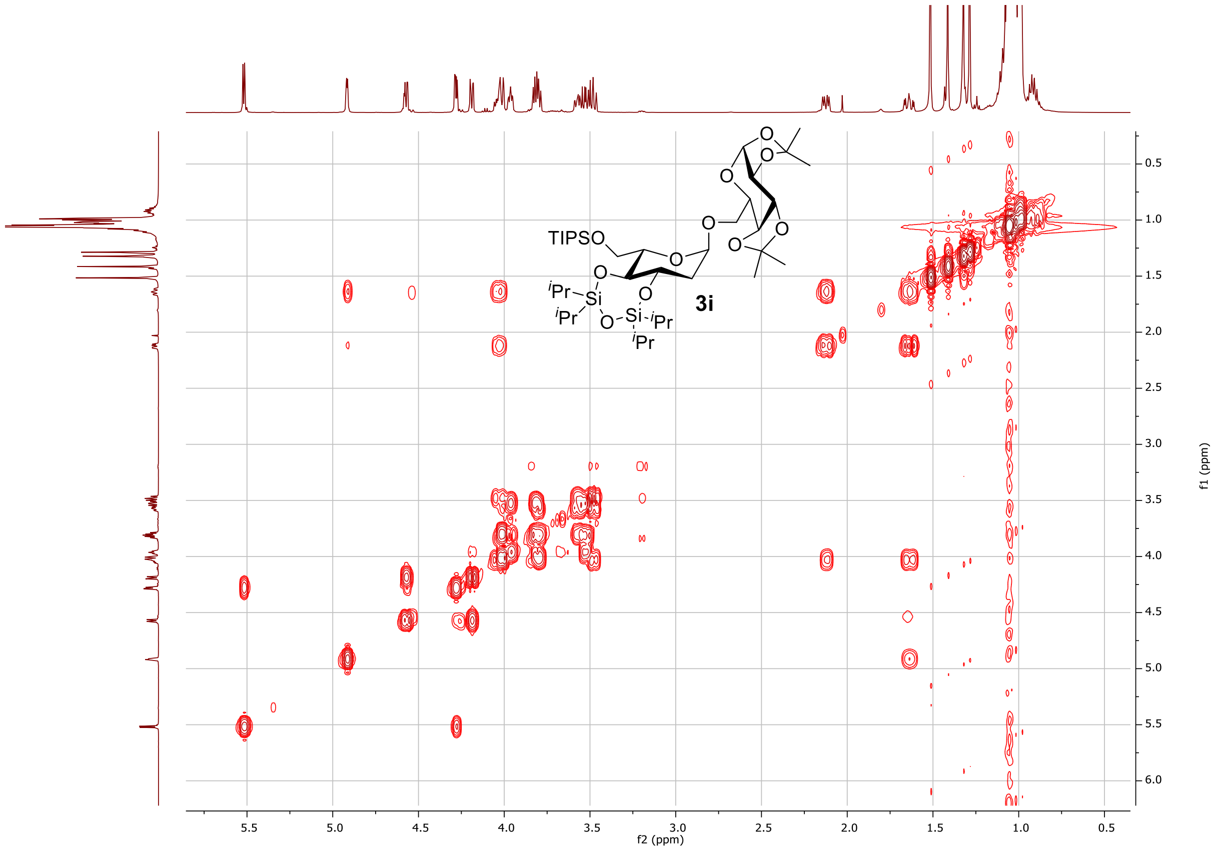

Supplementary figure S167: COSY spectra for **3i**

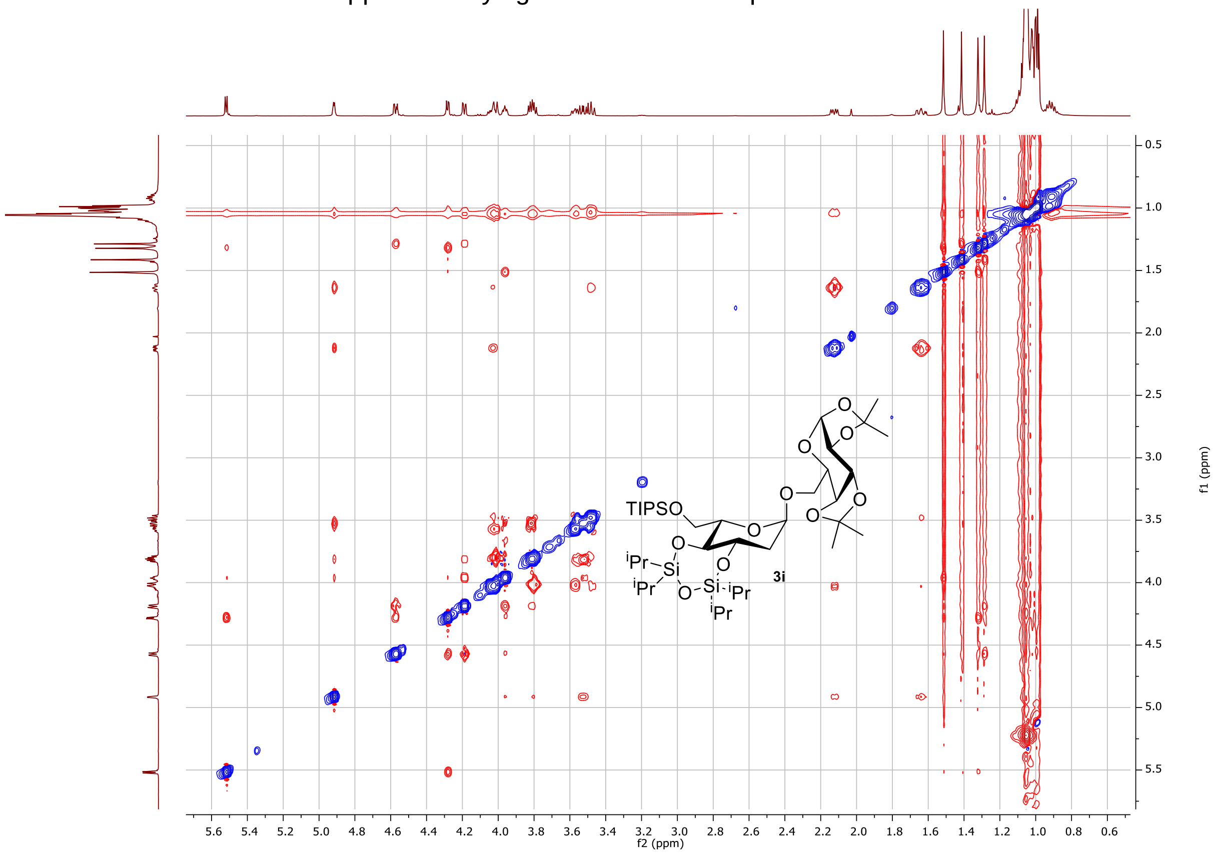

Supplementary figure S168: NOESY spectra for **3i**

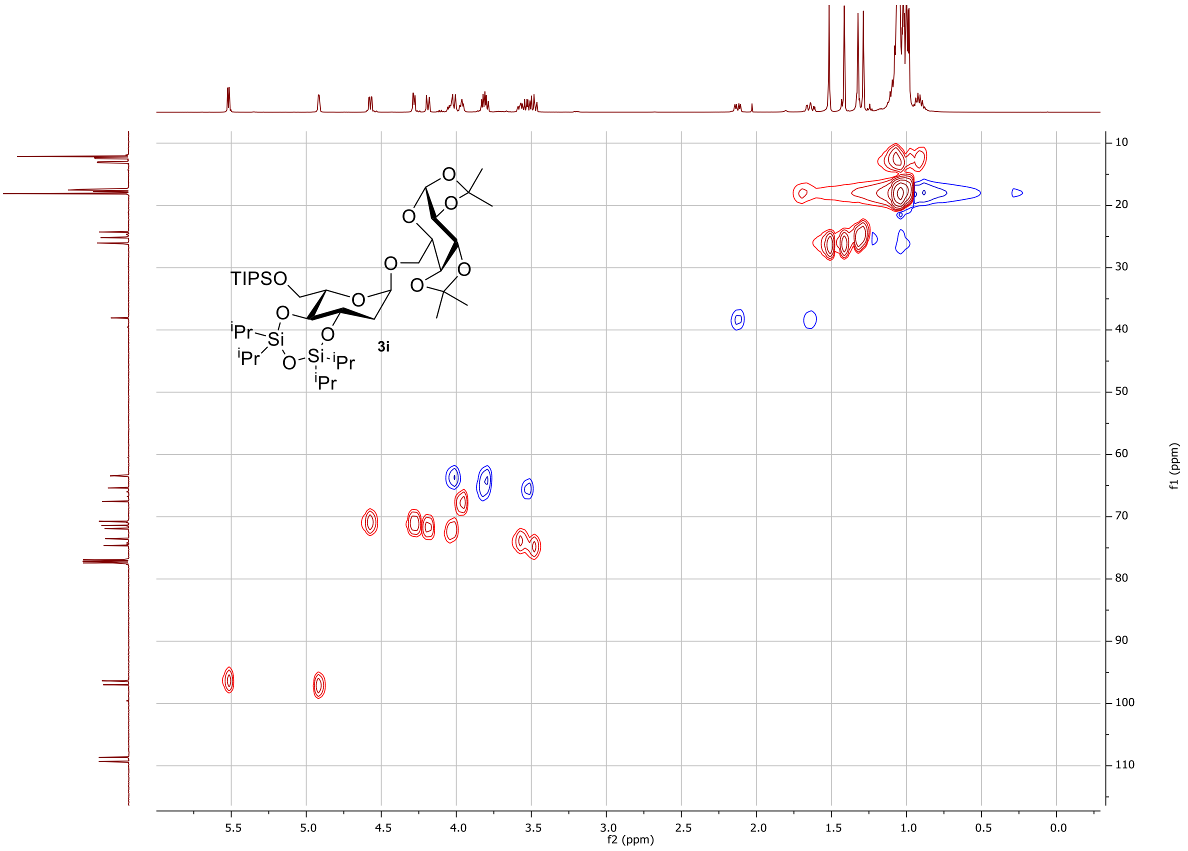

Supplementary figure S169: HSQC spectra for **3i**

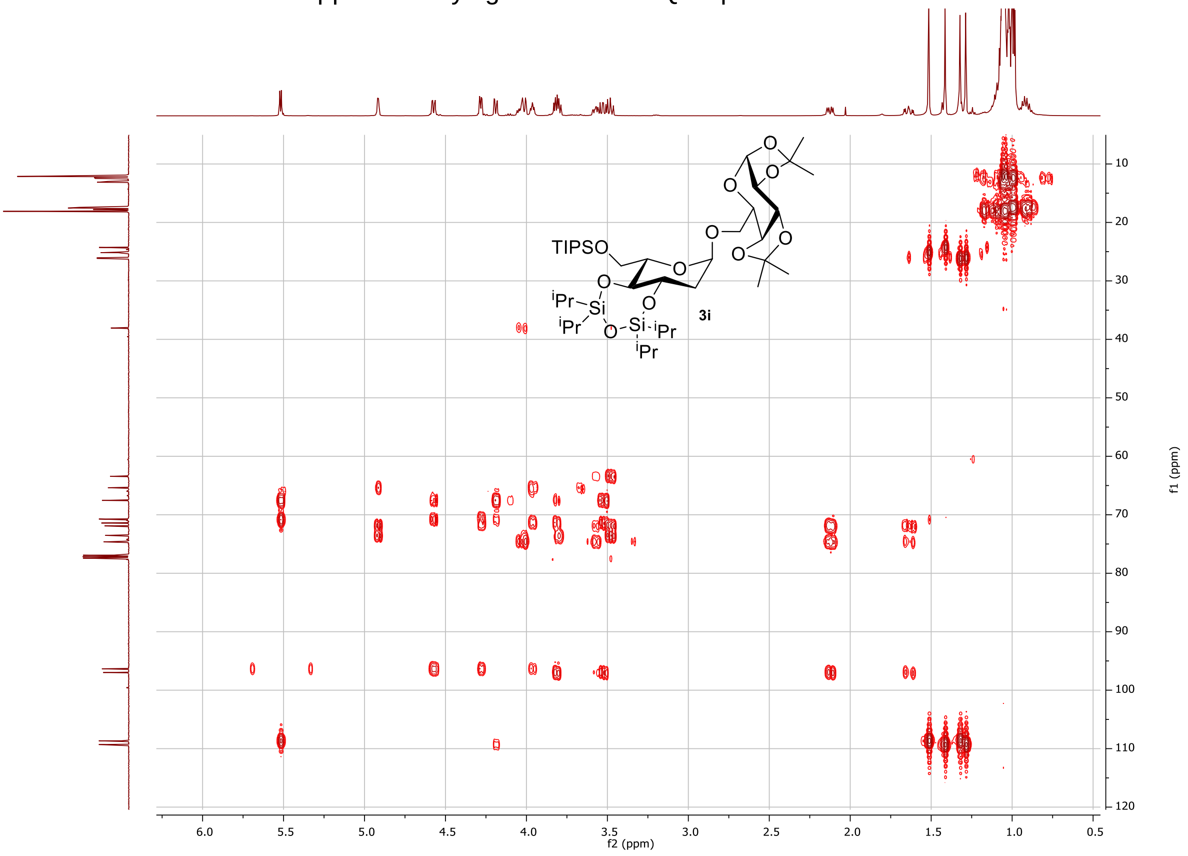

Supplementary figure S170: HMBC spectra for **3i**

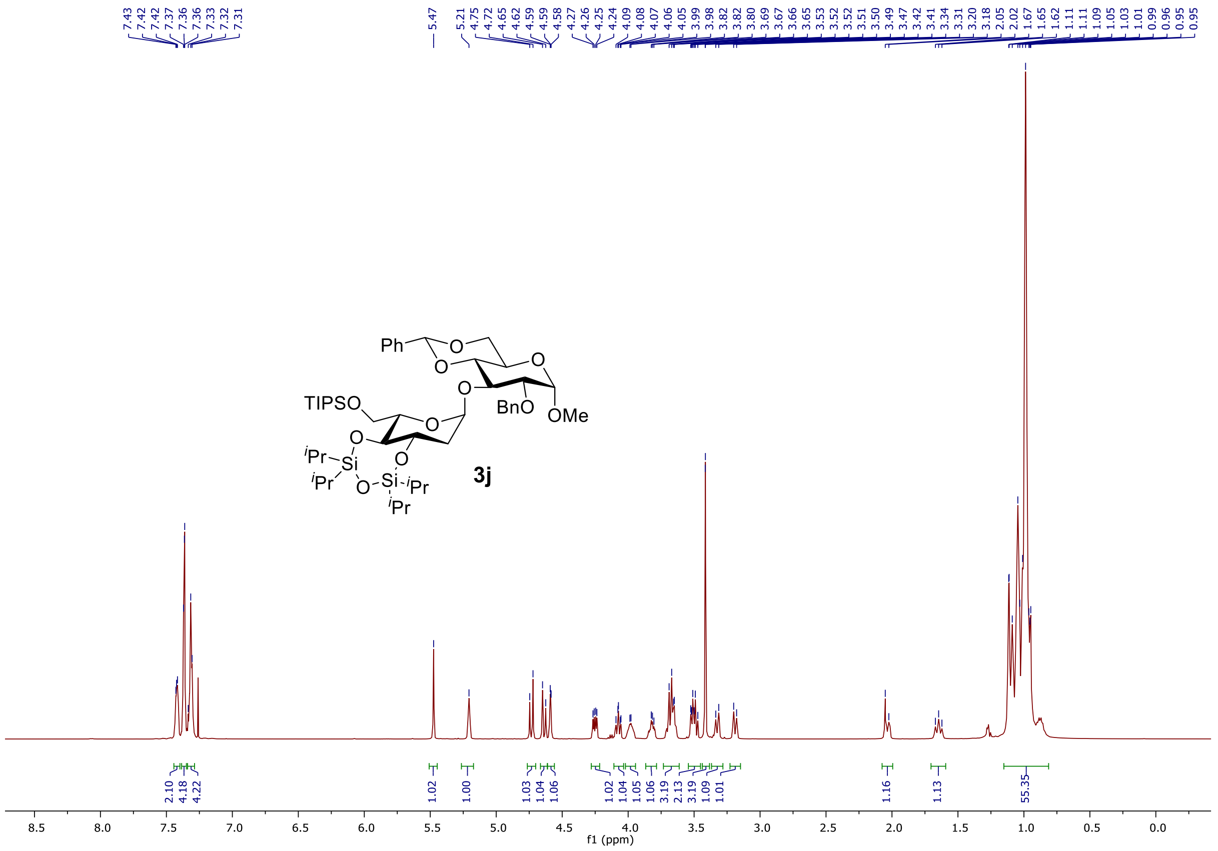

Supplementary figure S171: ^{1}H spectra for **3j**

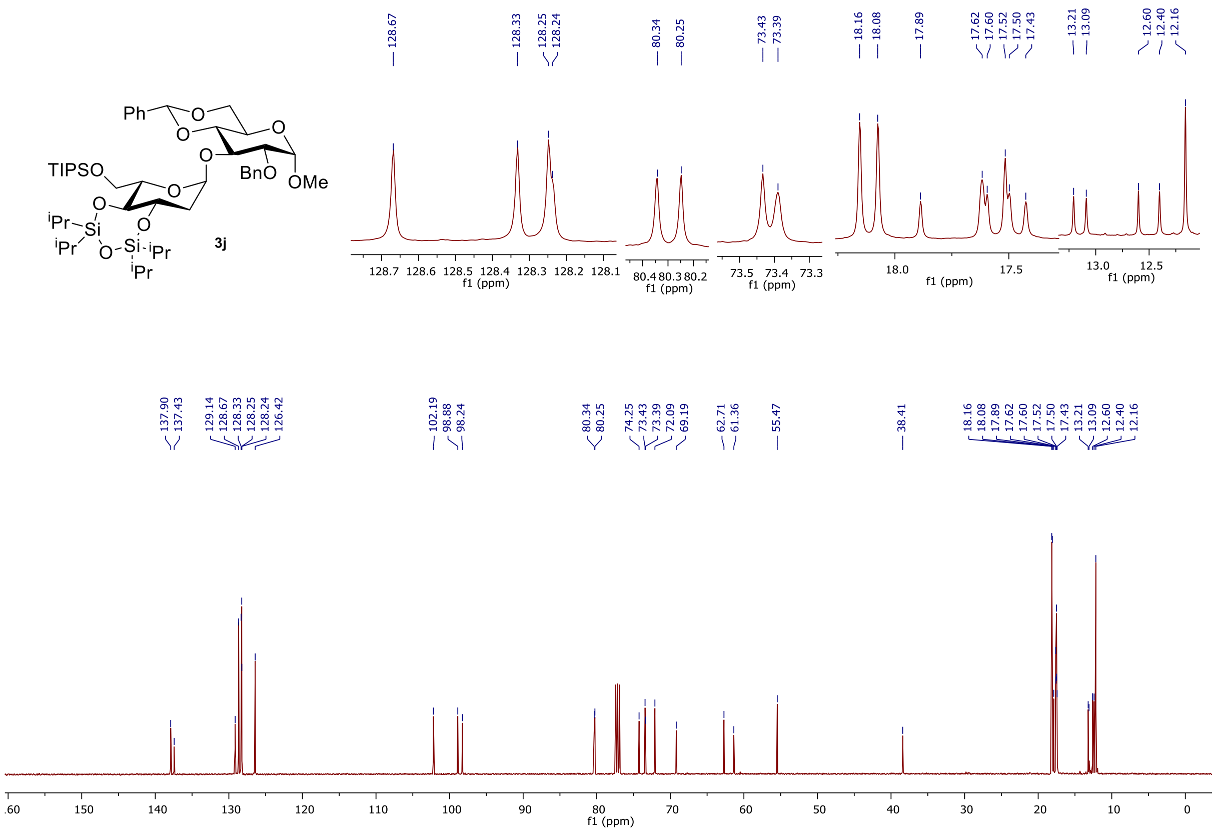

Supplementary figure S172: ^{13}C spectra for **3j**

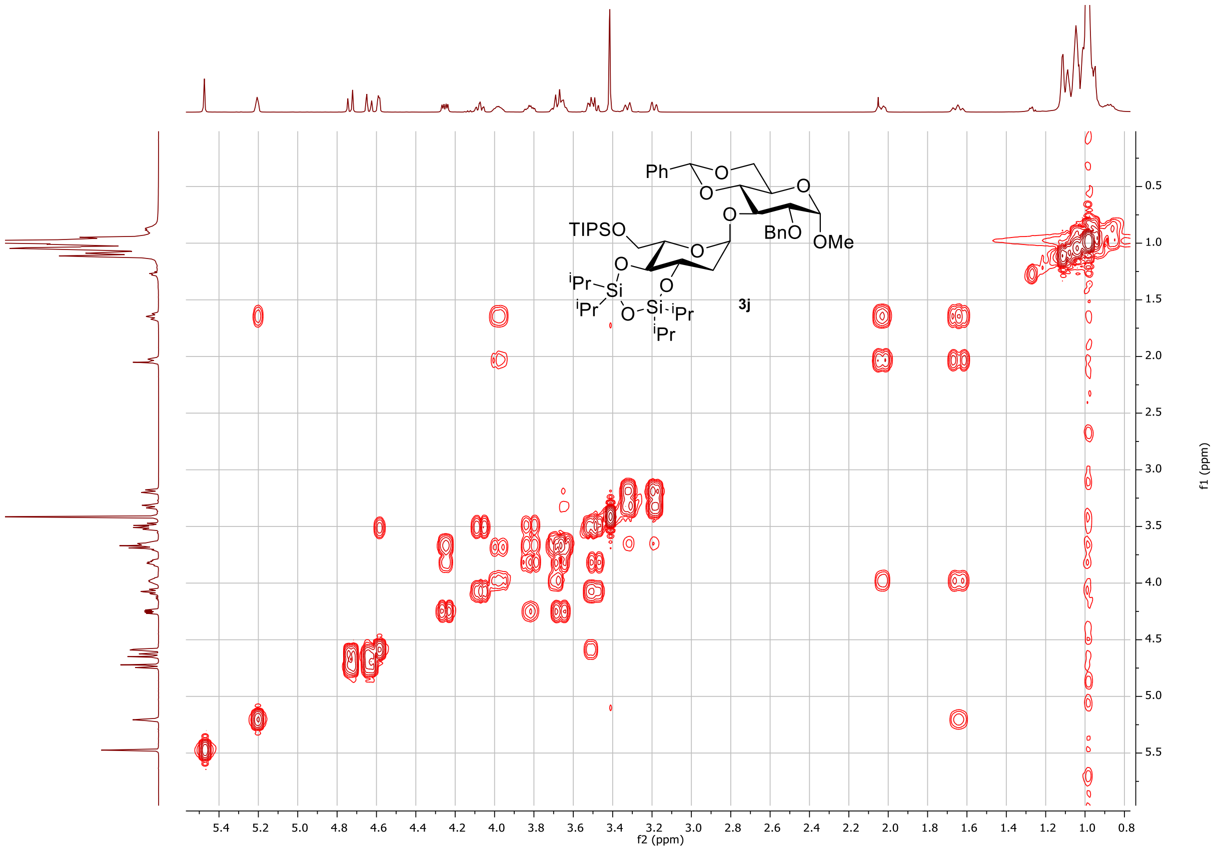

Supplementary figure S173: COSY spectra for **3j**

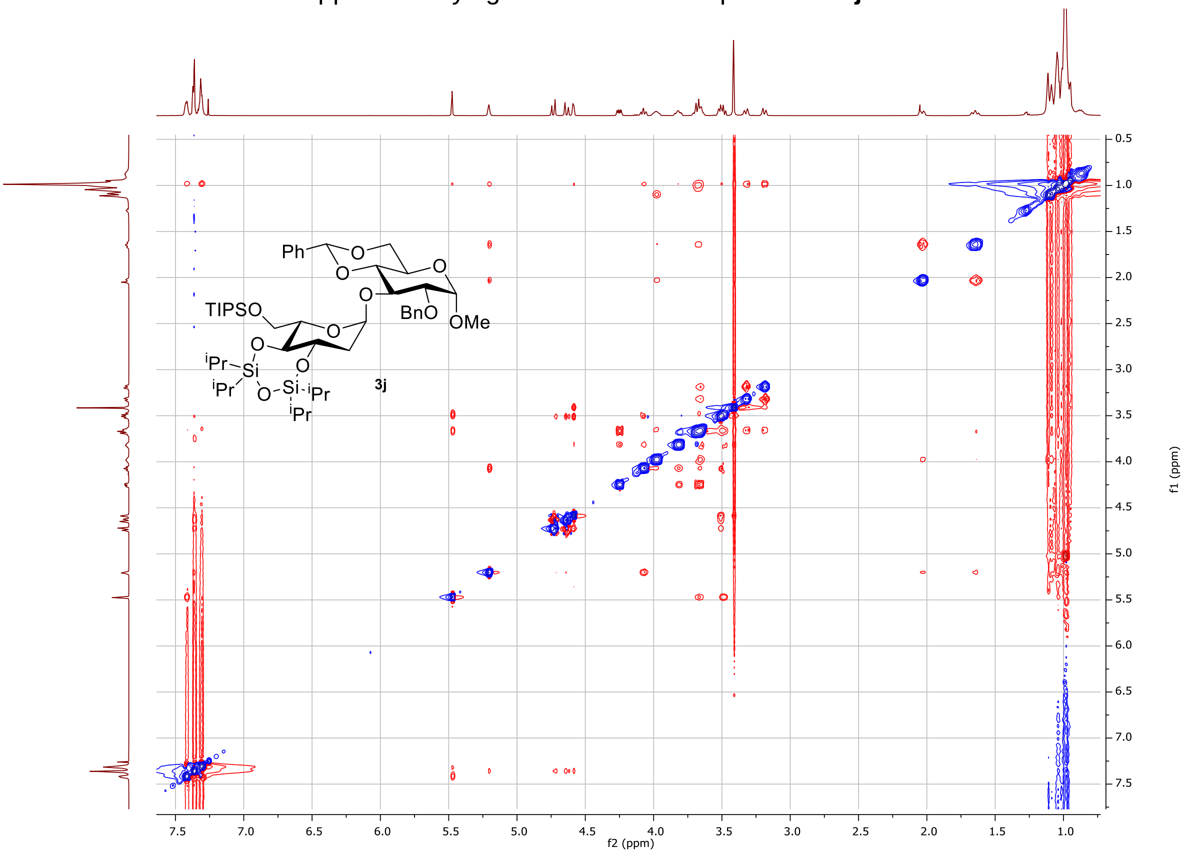

Supplementary figure S174: NOESY spectra for **3j**

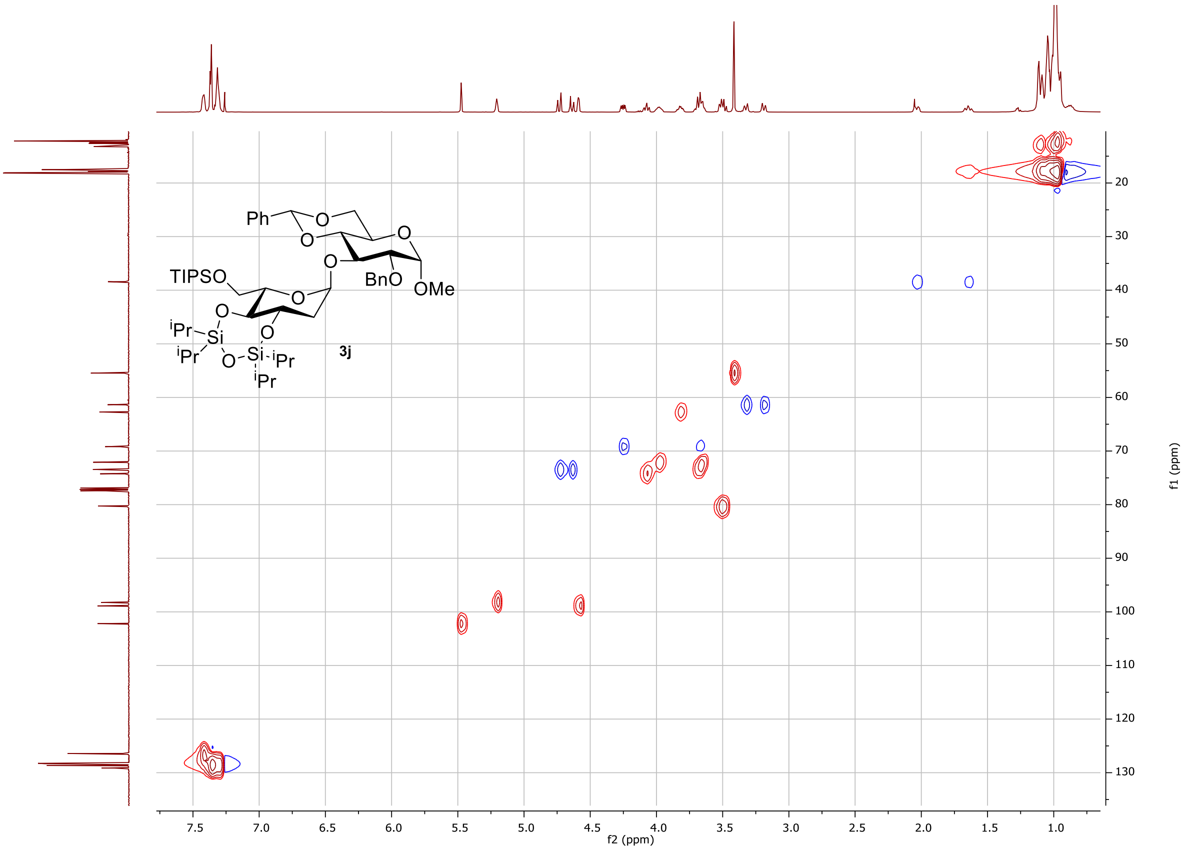

Supplementary figure S175: HSQC spectra for **3j**

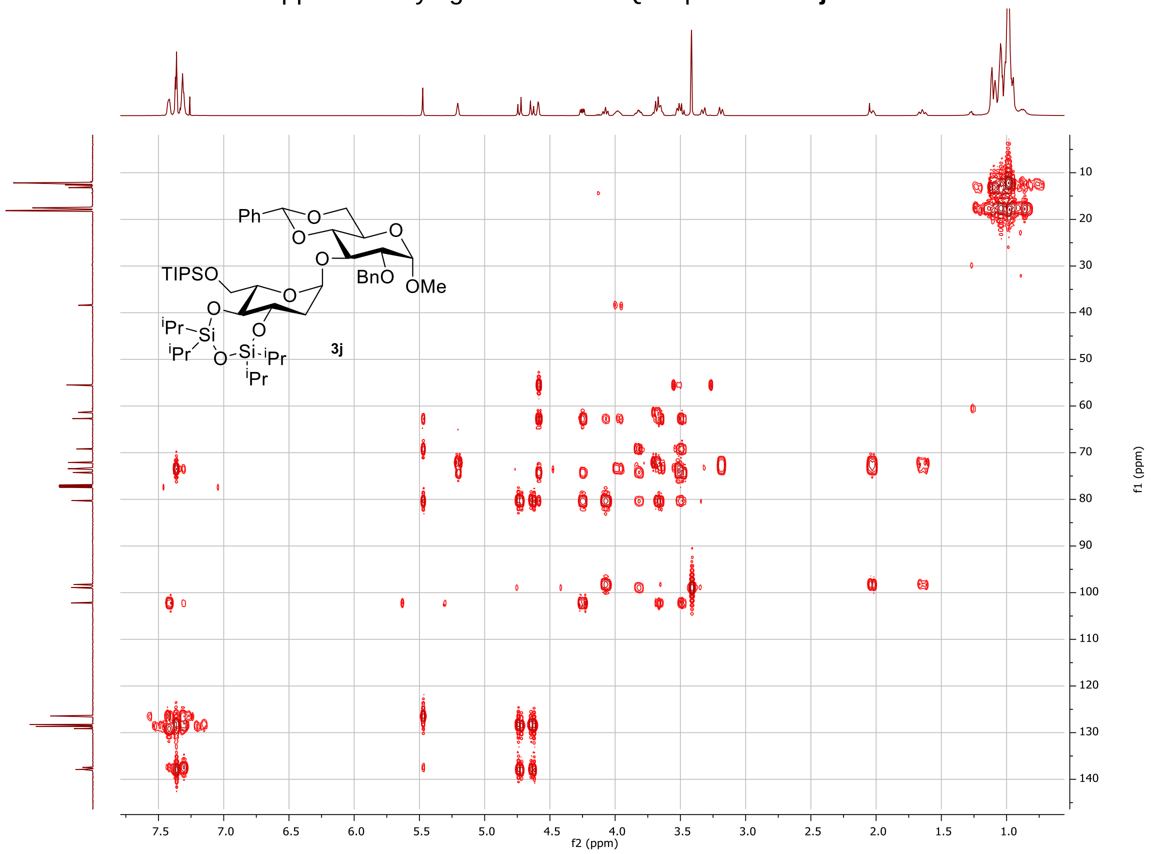

Supplementary figure S176: HMBC spectra for **3j**

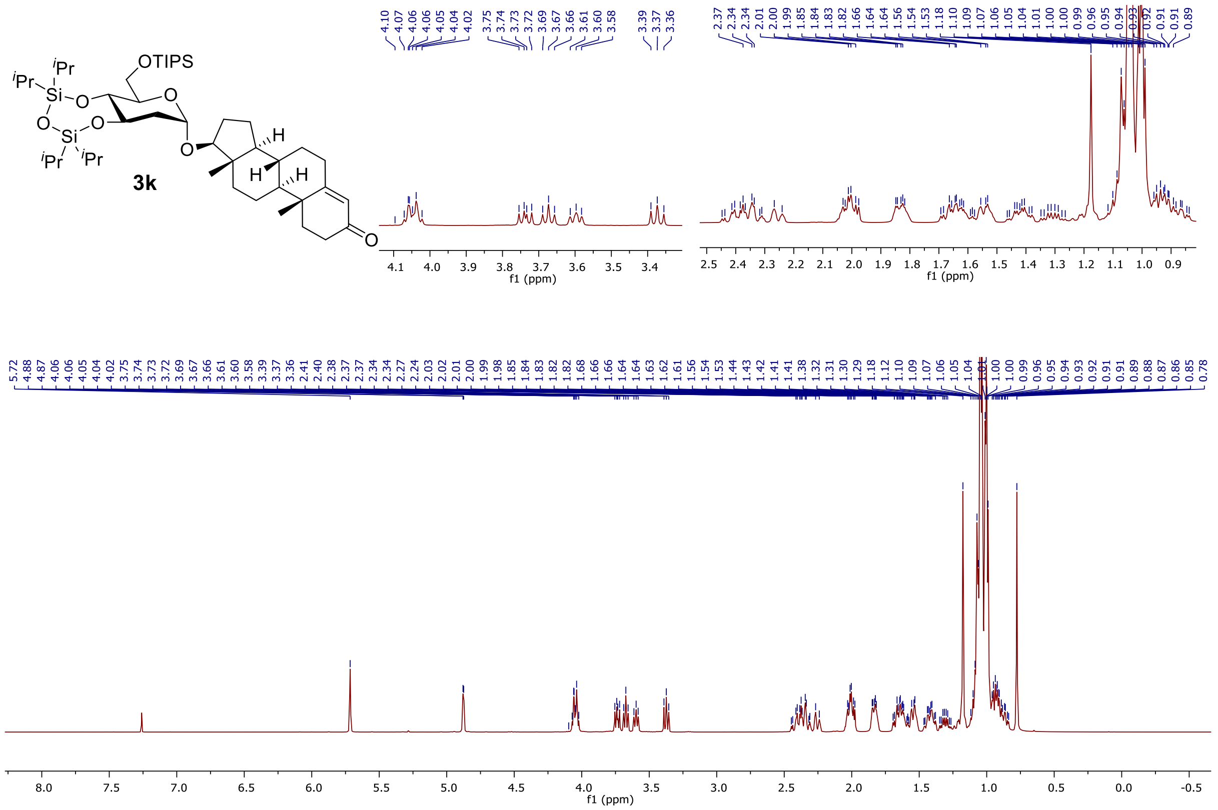

Supplementary figure S177: ^{1}H spectra for **3k**

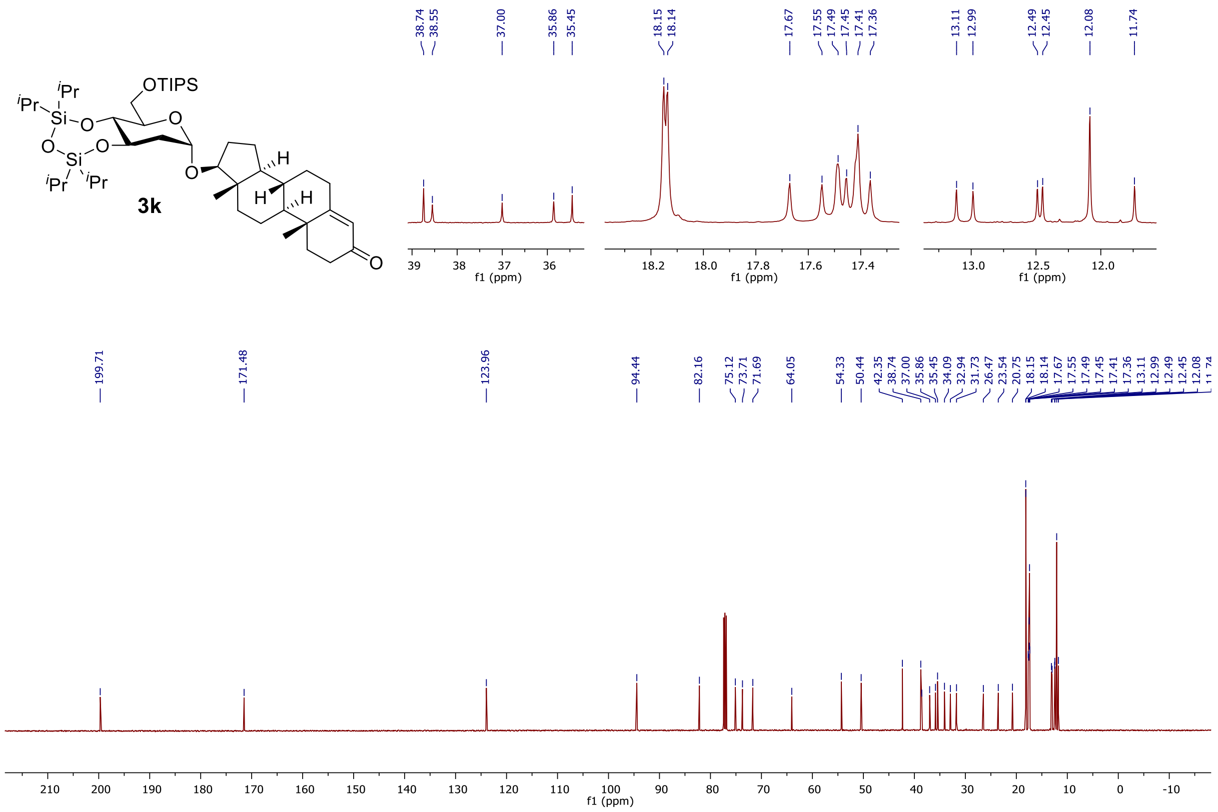

Supplementary figure S178: ^{13}C spectra for **3k**

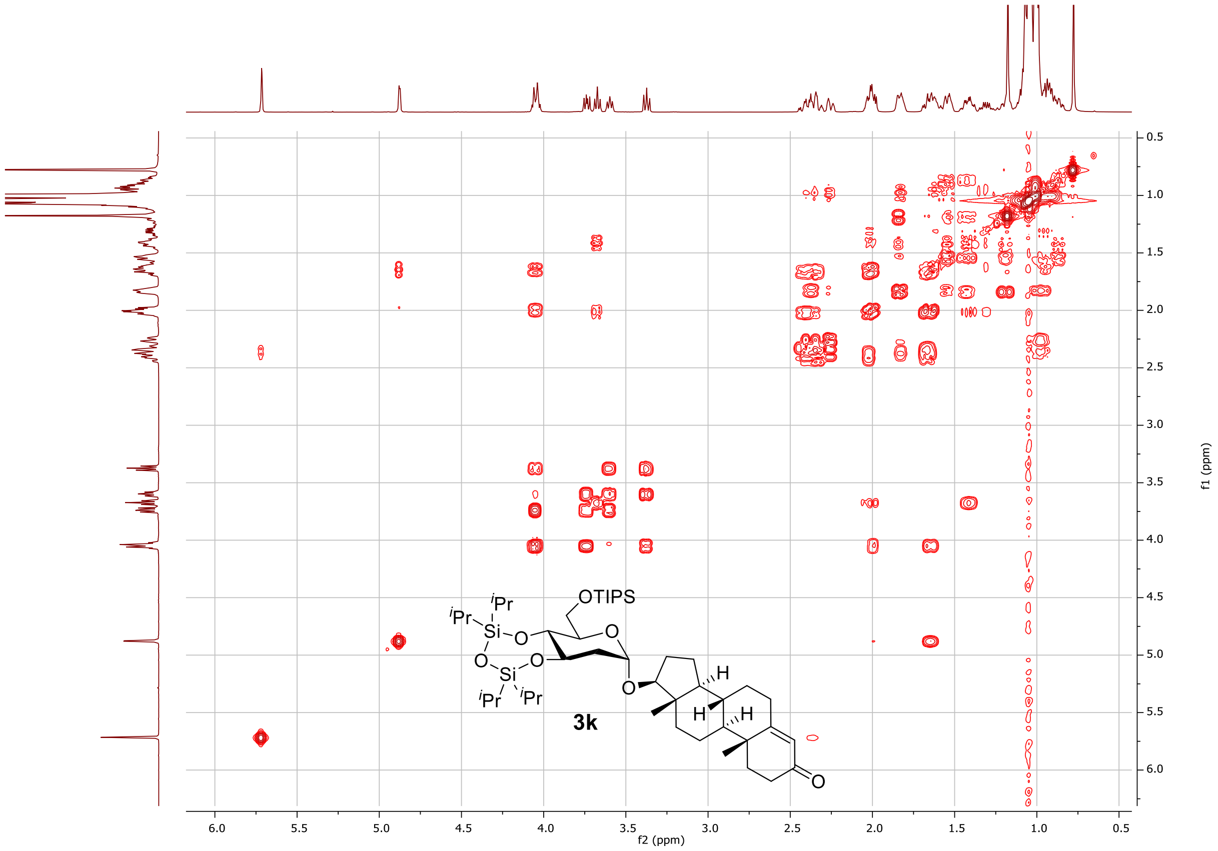

Supplementary figure S179: COSY spectra for **3k**

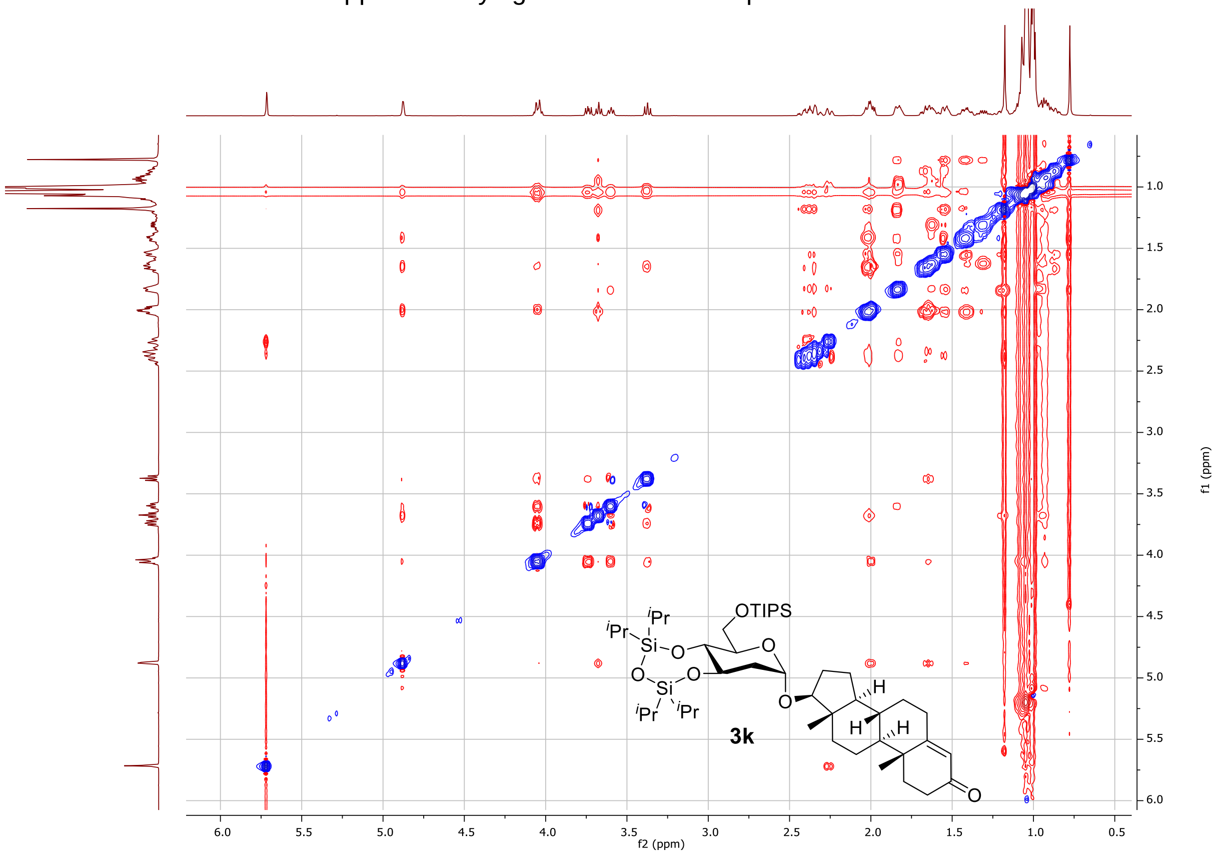

Supplementary figure S180: NOESY spectra for **3k**

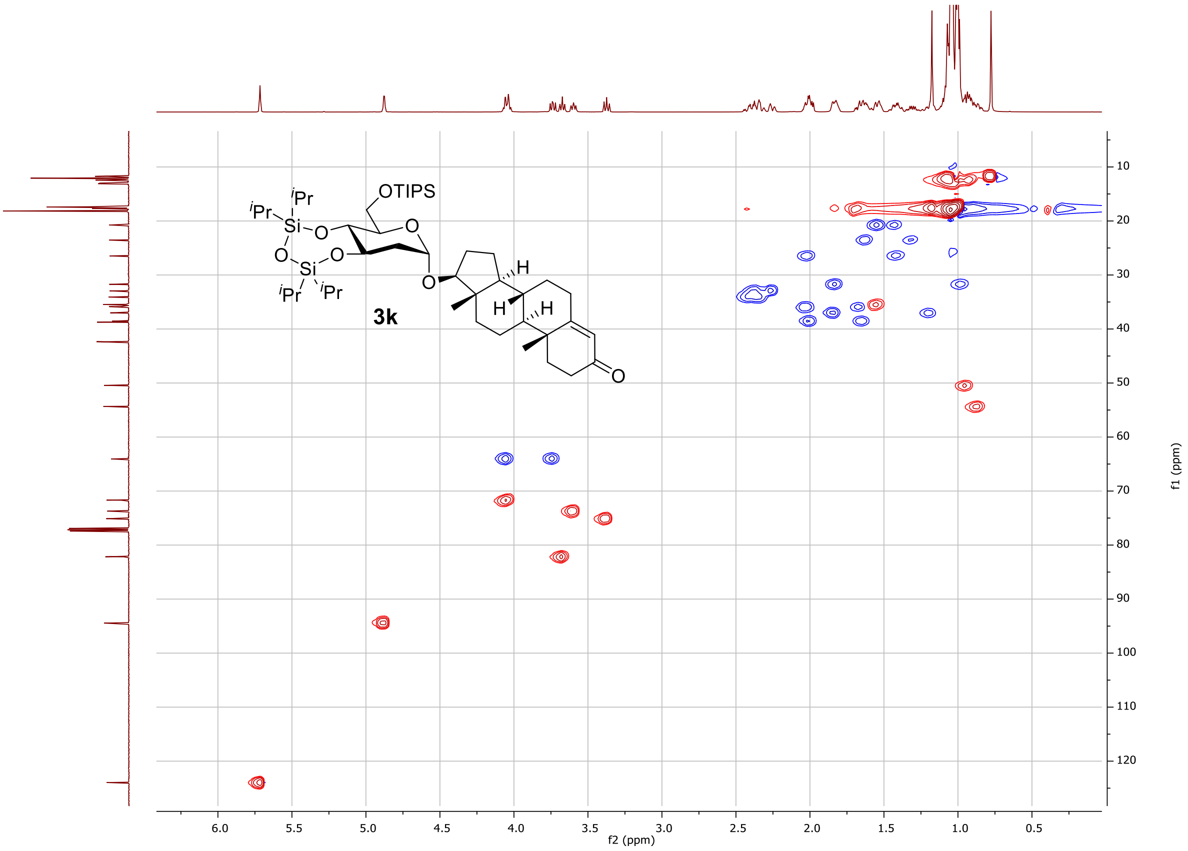

Supplementary figure S181: HSQC spectra for **3k**

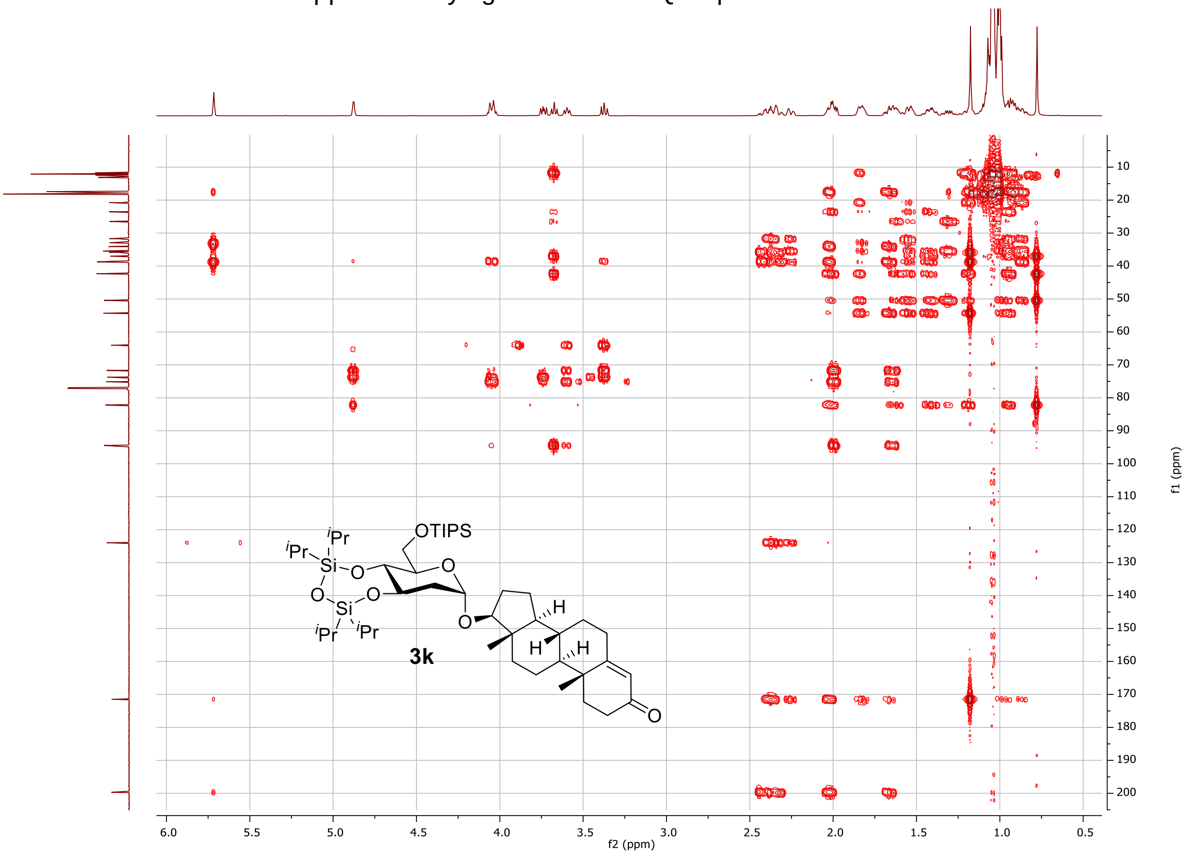

Supplementary figure S182: HMBC spectra for **3k**

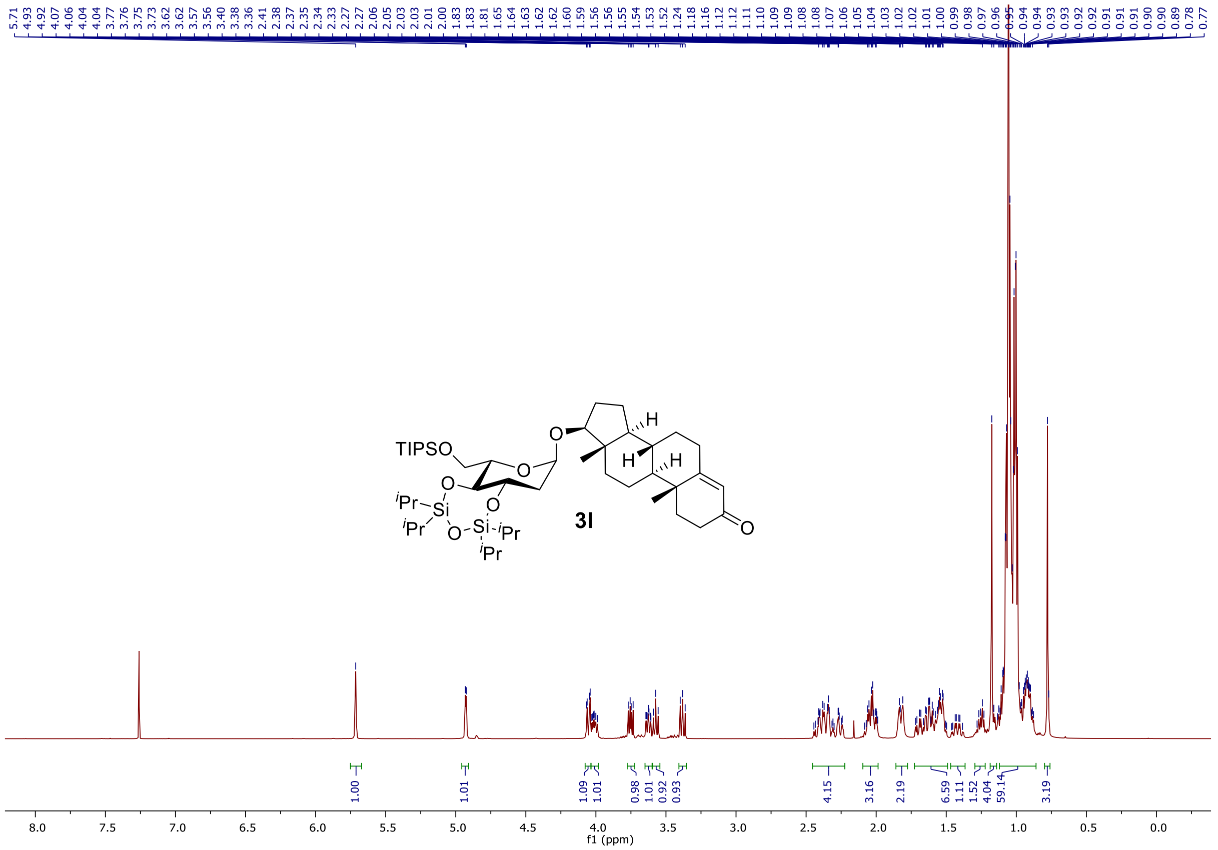

Supplementary figure S183: ^{1}H spectra for **3l**

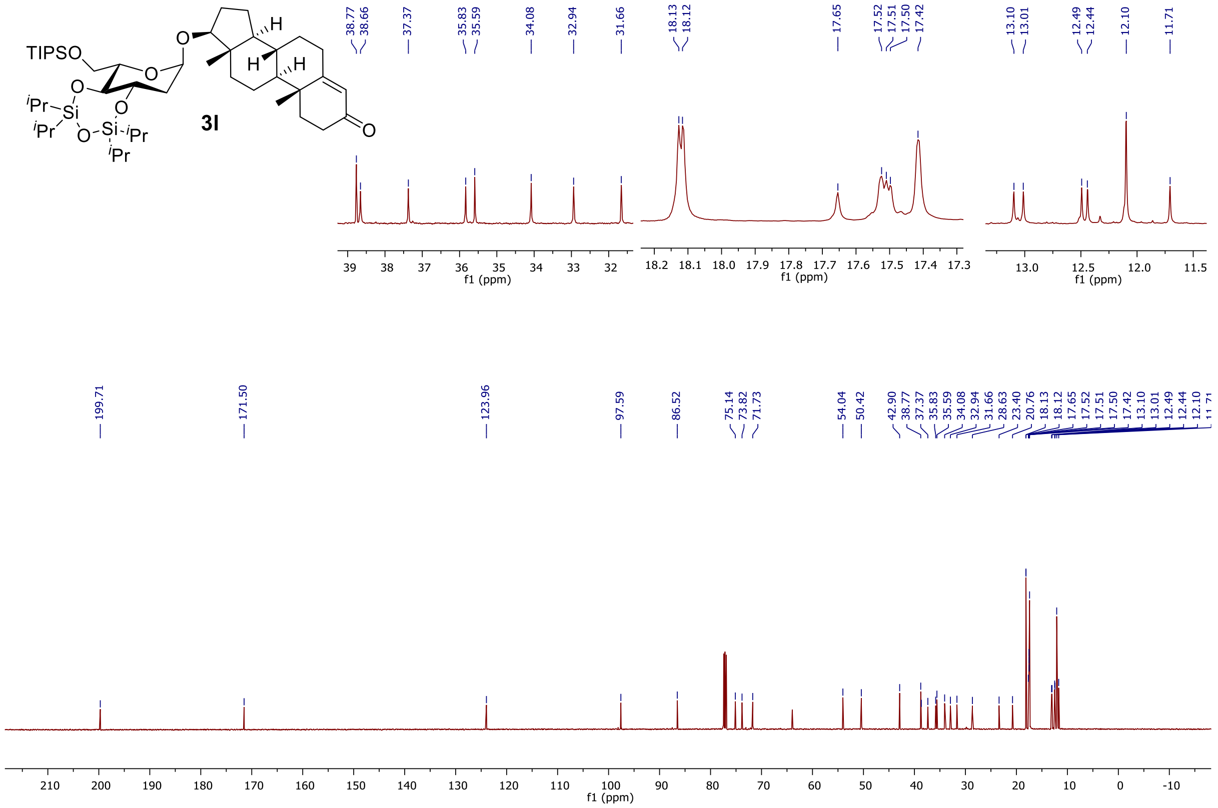

Supplementary figure S184: ^{13}C spectra for **3l**

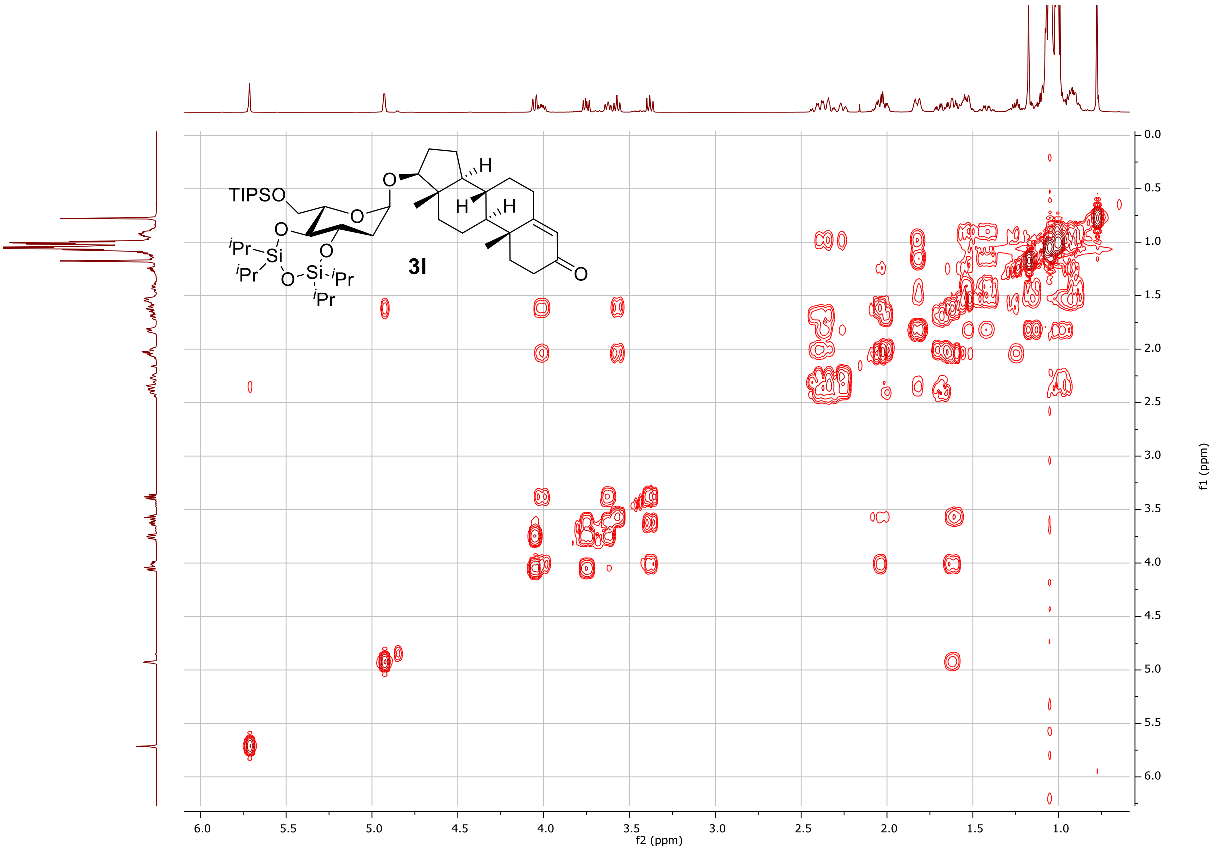

Supplementary figure S185: COSY spectra for **3l**

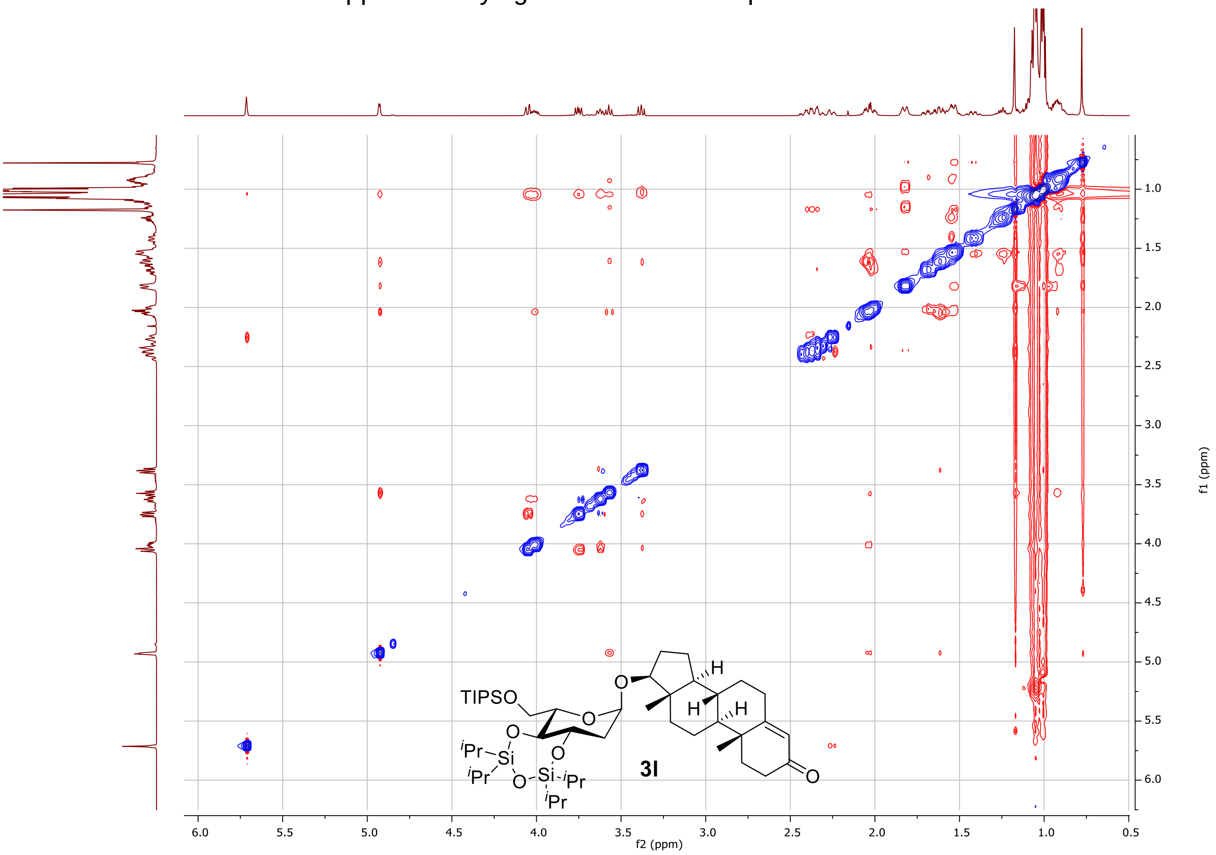

Supplementary figure S186: NOESY spectra for **3l**

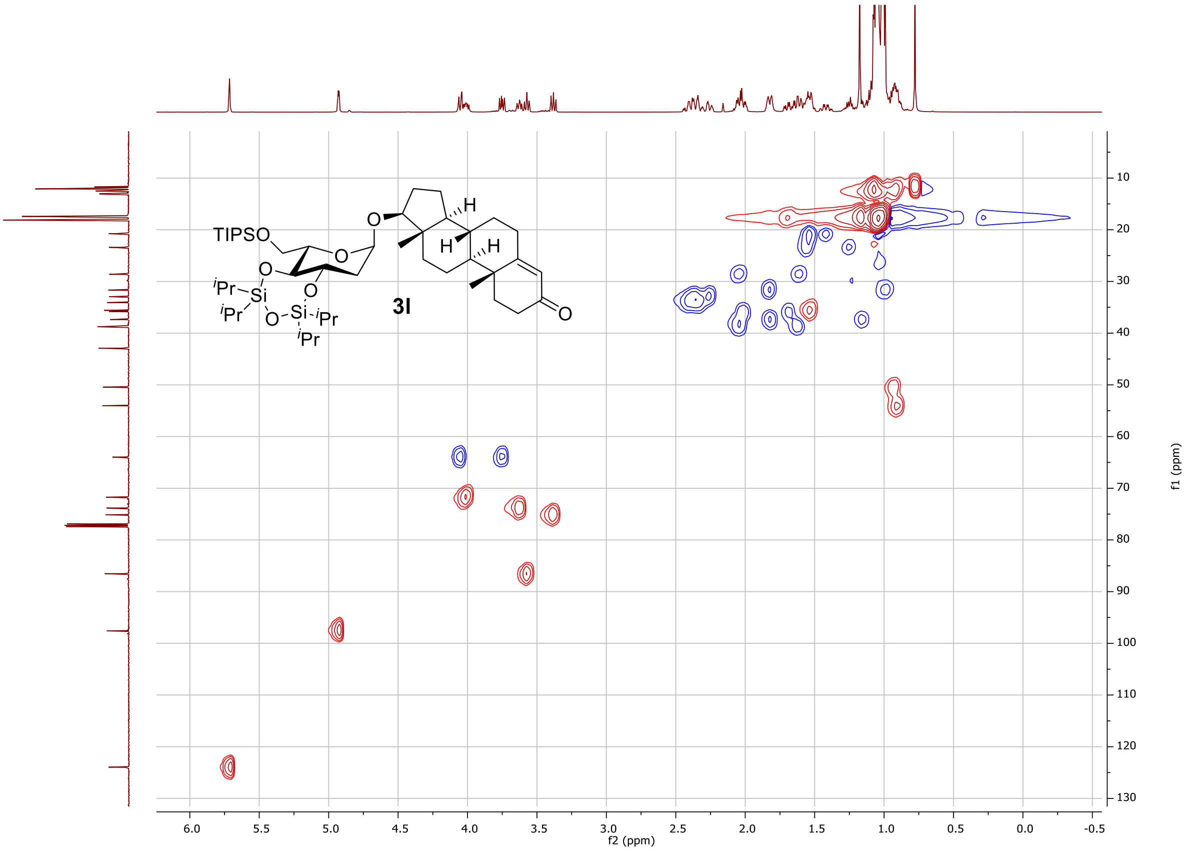

Supplementary figure S187: HSQC spectra for **3l**

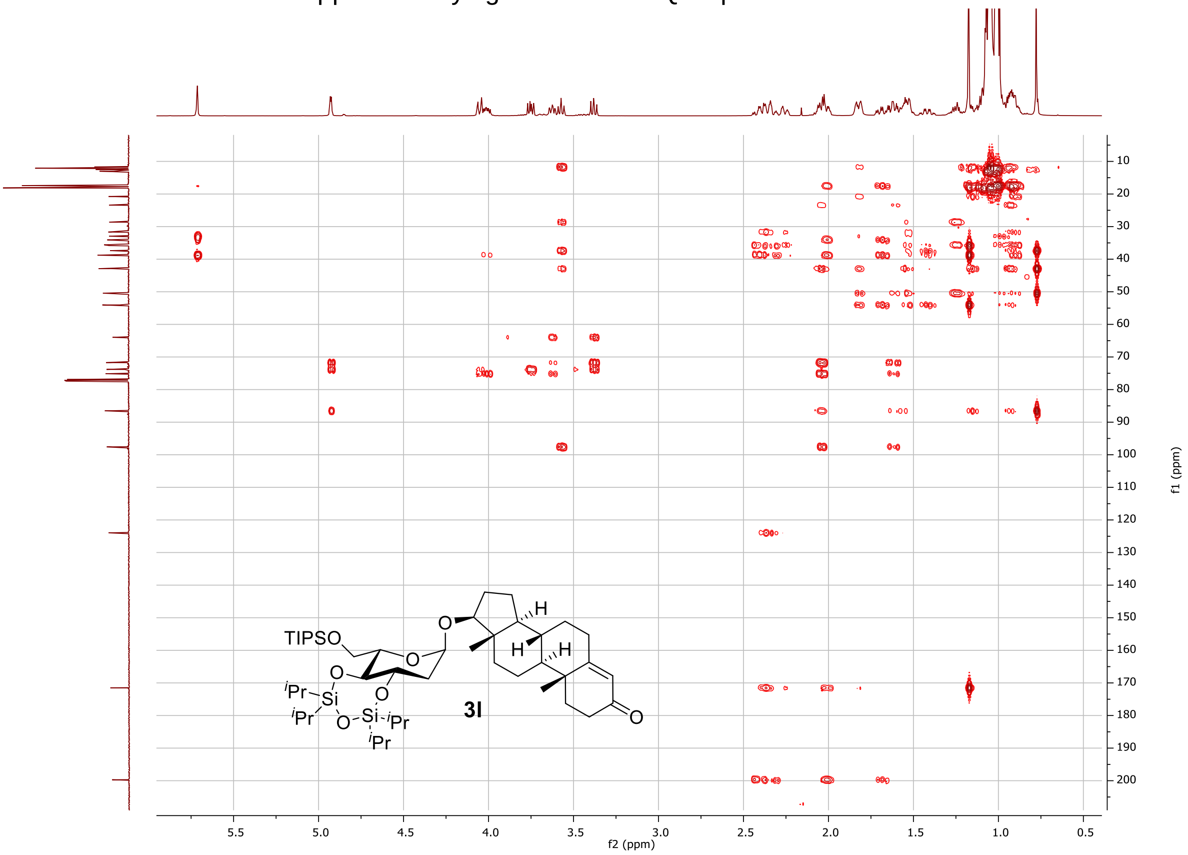

Supplementary figure S188: HMBC spectra for **3l**

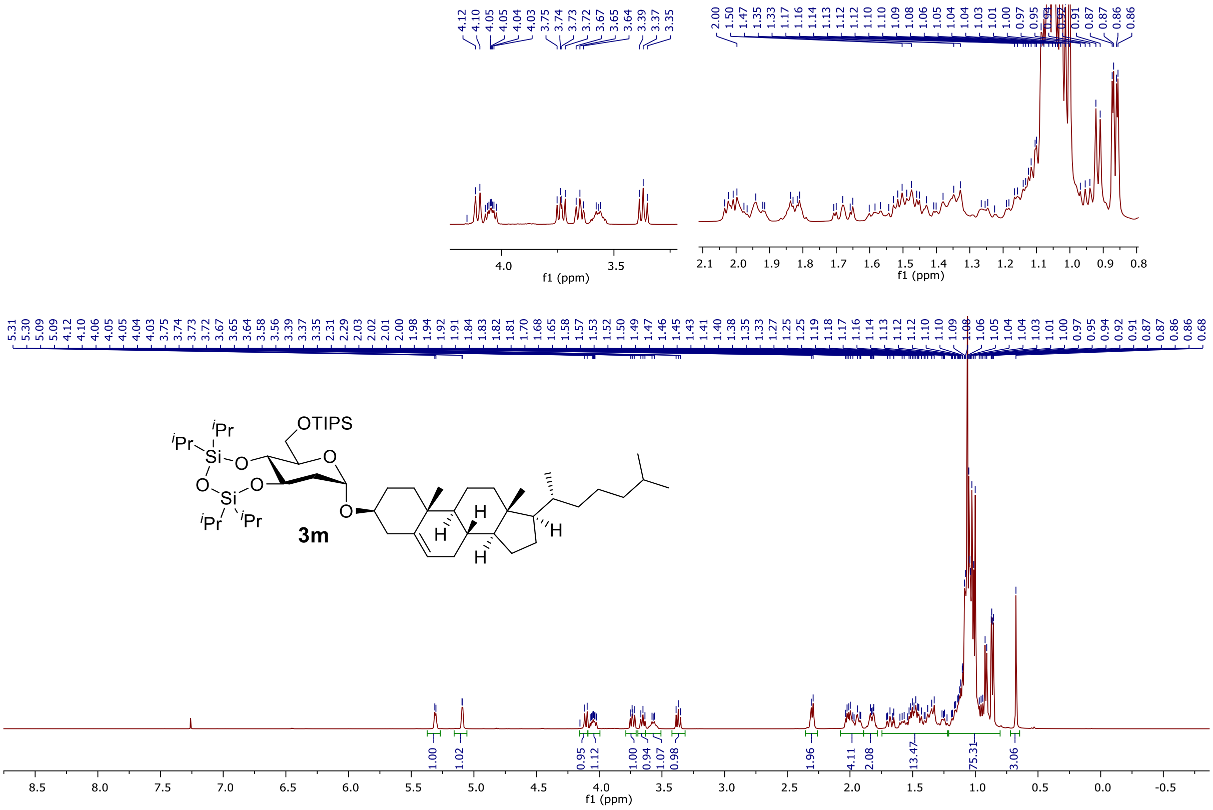

Supplementary figure S189: ^{1}H spectra for **3m**

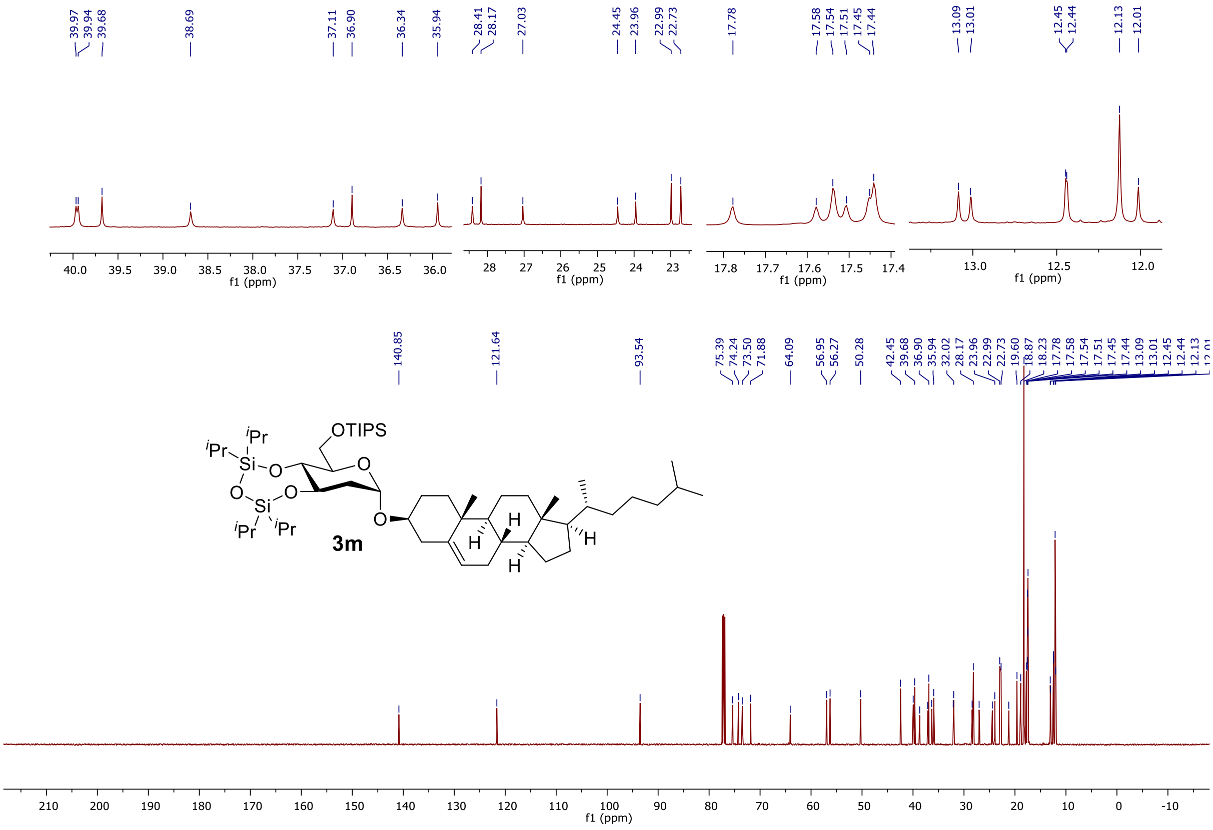

Supplementary figure S190: ^{13}C spectra for **3m**

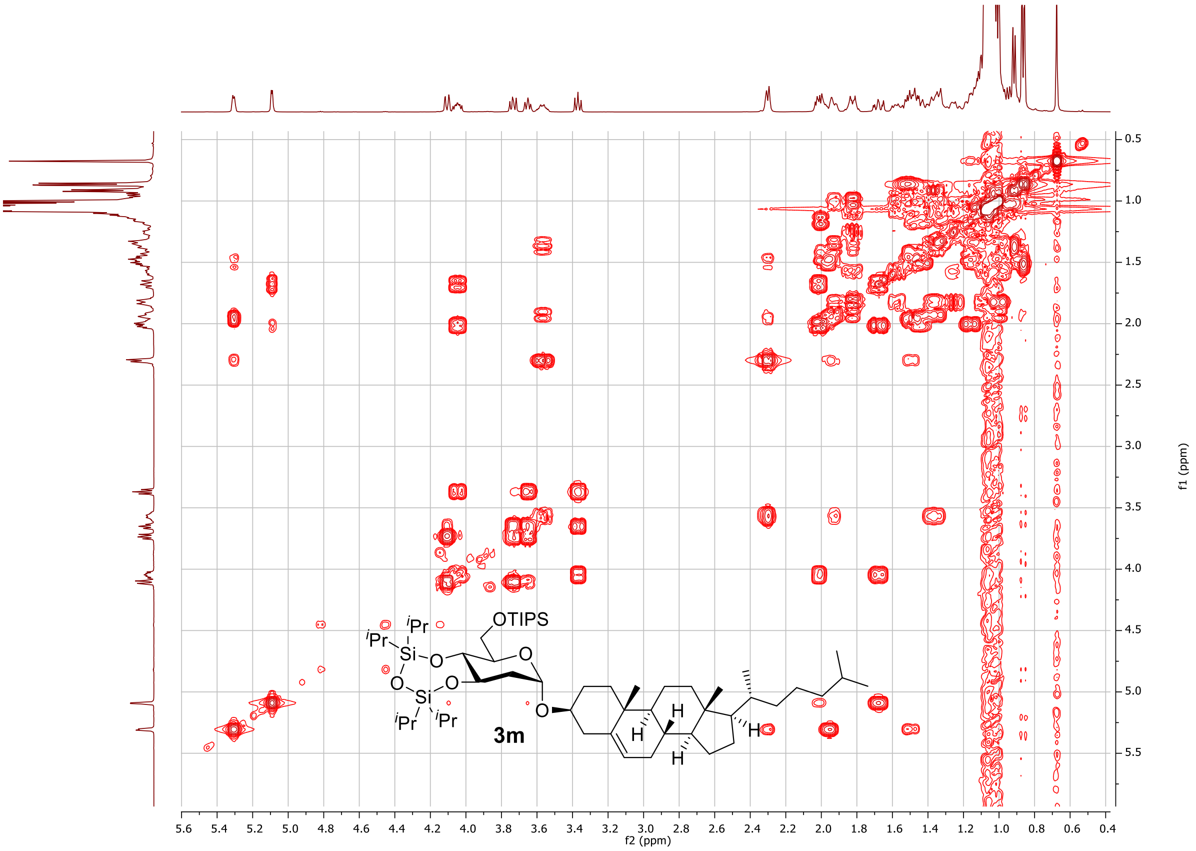

Supplementary figure S191: COSY spectra for **3m**

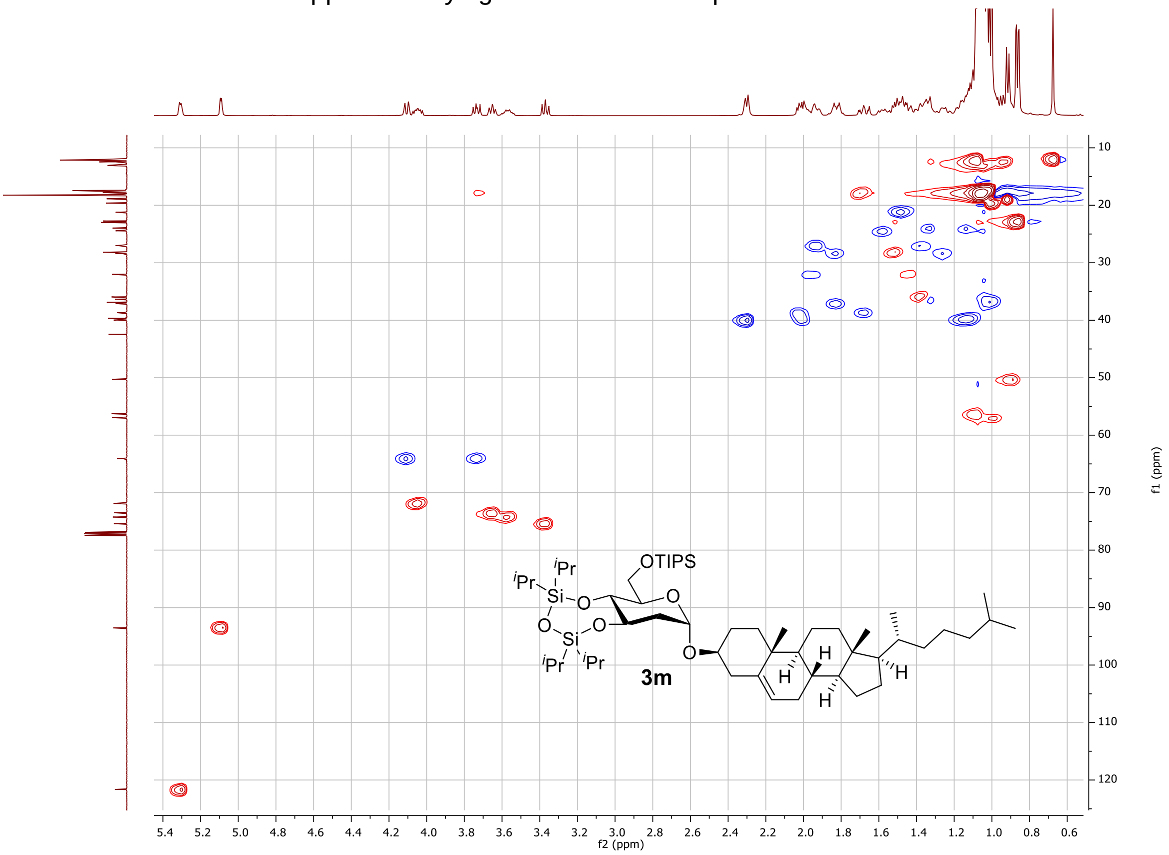

Supplementary figure S192: HSQC spectra for **3m**

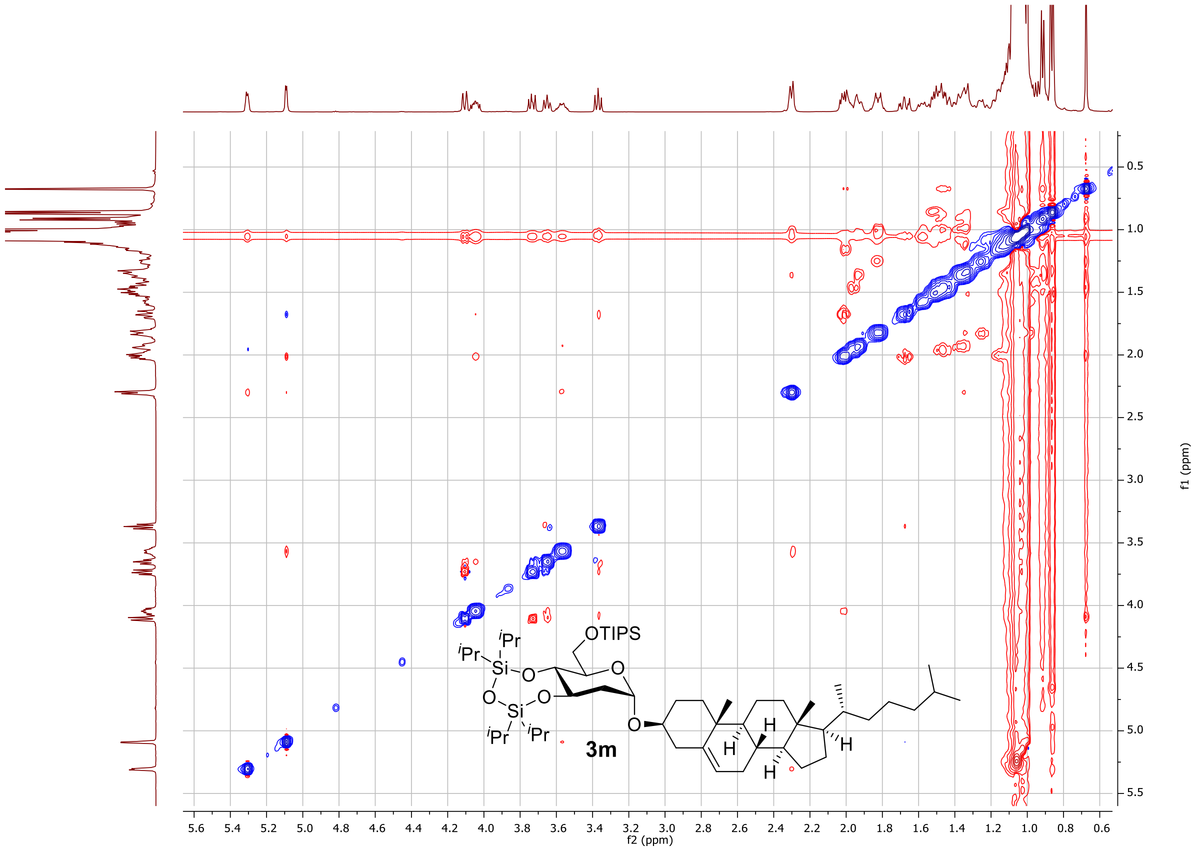

Supplementary figure S193: NOESY spectra for **3m**

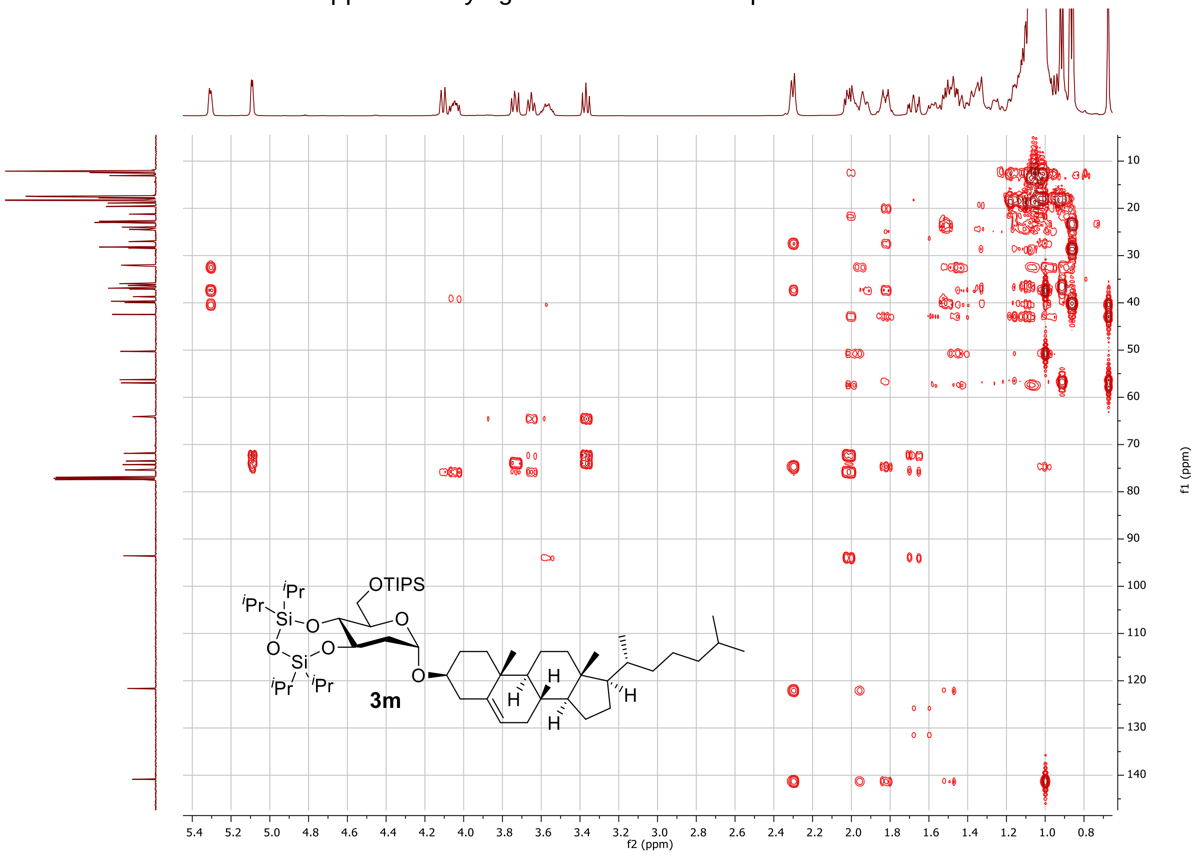

Supplementary figure S194: HMBC spectra for **3m**

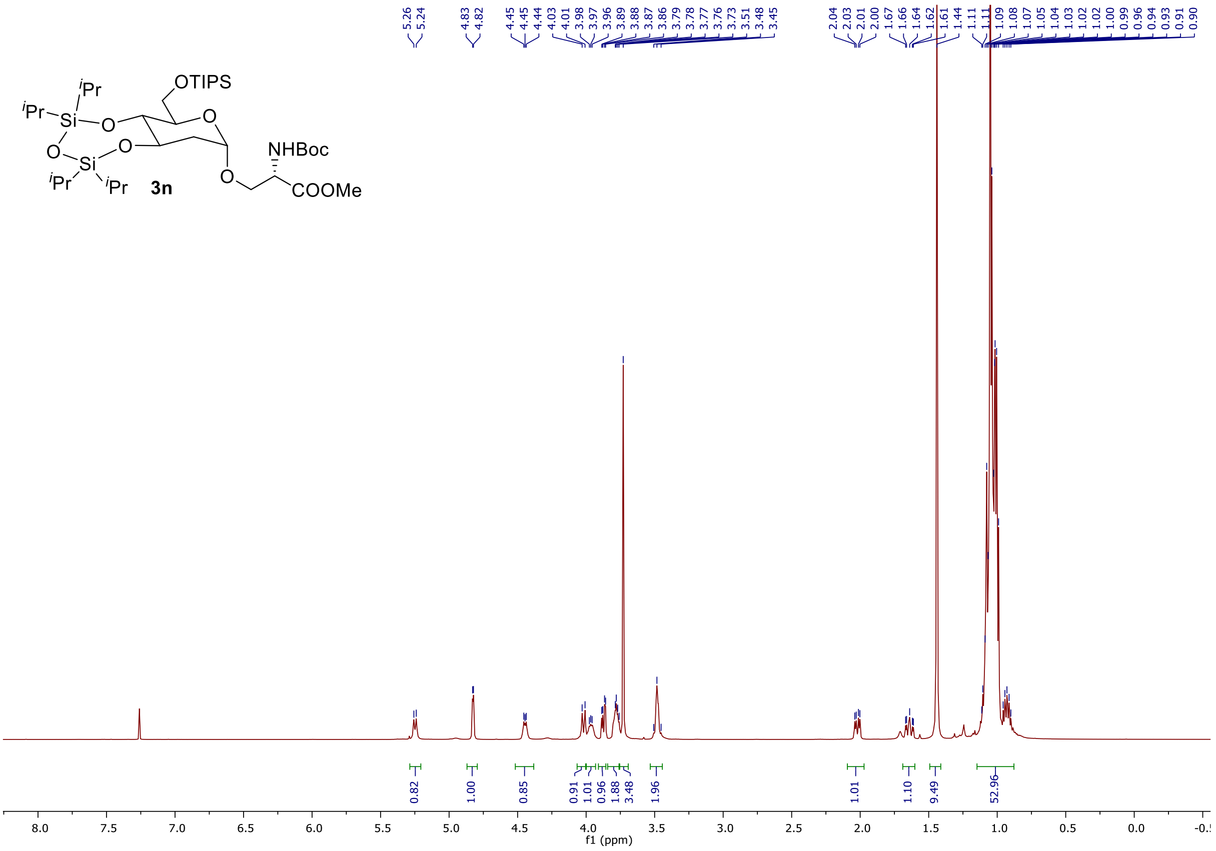

Supplementary figure S195: ^{1}H spectra for **3n**

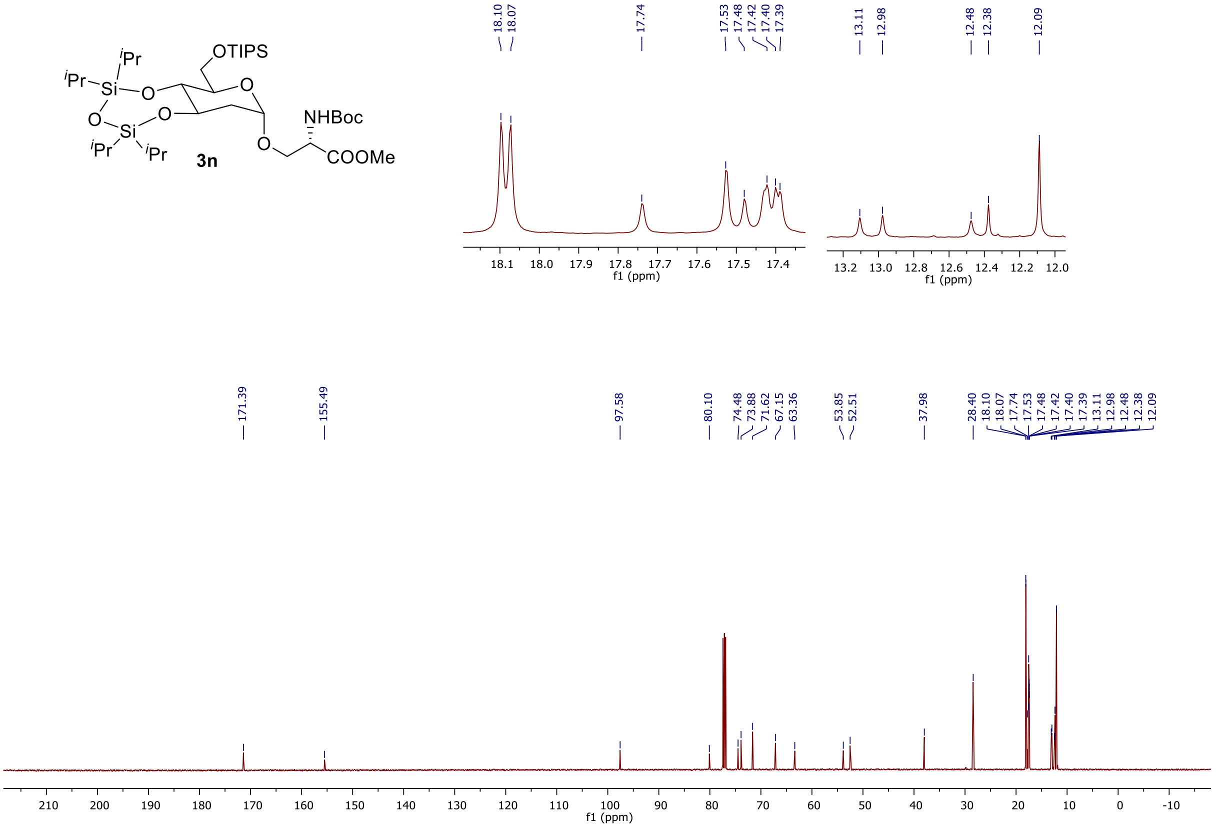

Supplementary figure S196: ^{13}C spectra for **3n**

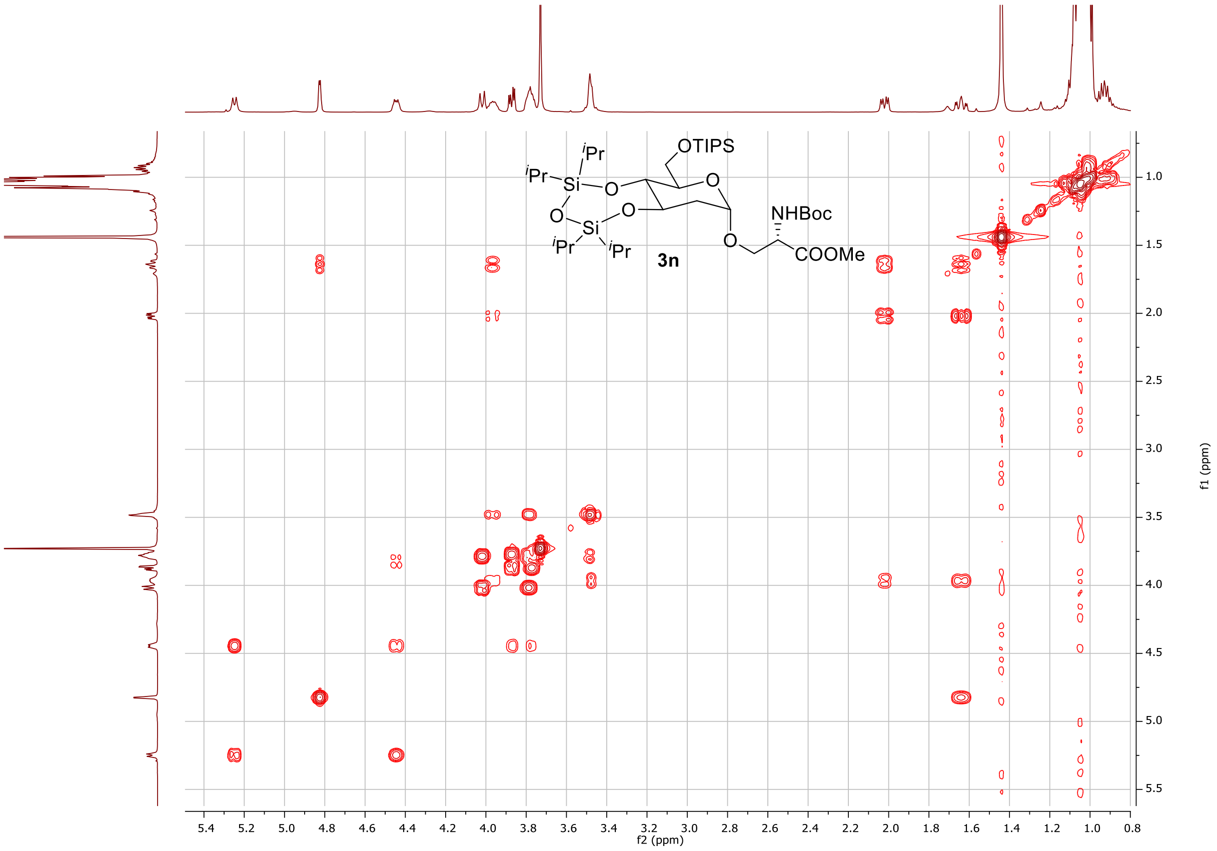

Supplementary figure S197: COSY spectra for **3n**

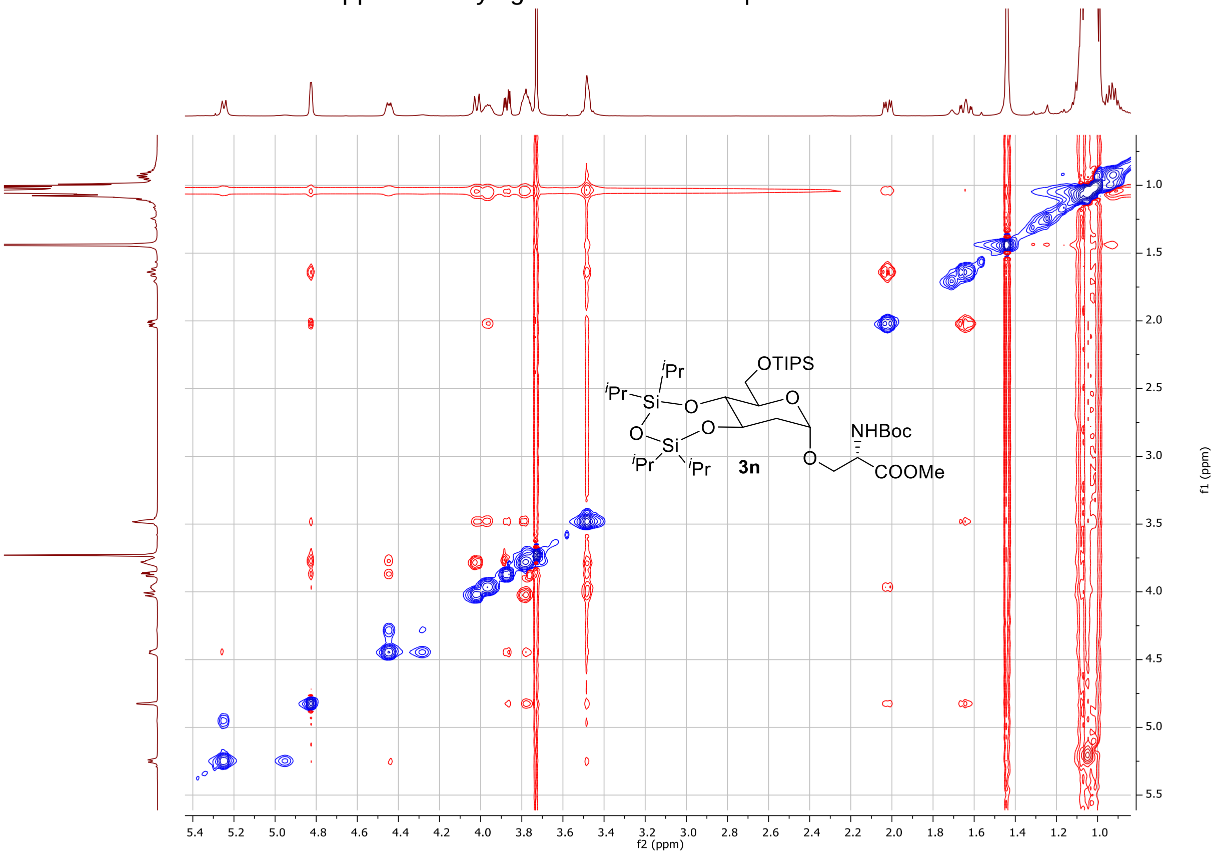

Supplementary figure S198: NOESY spectra for **3n**

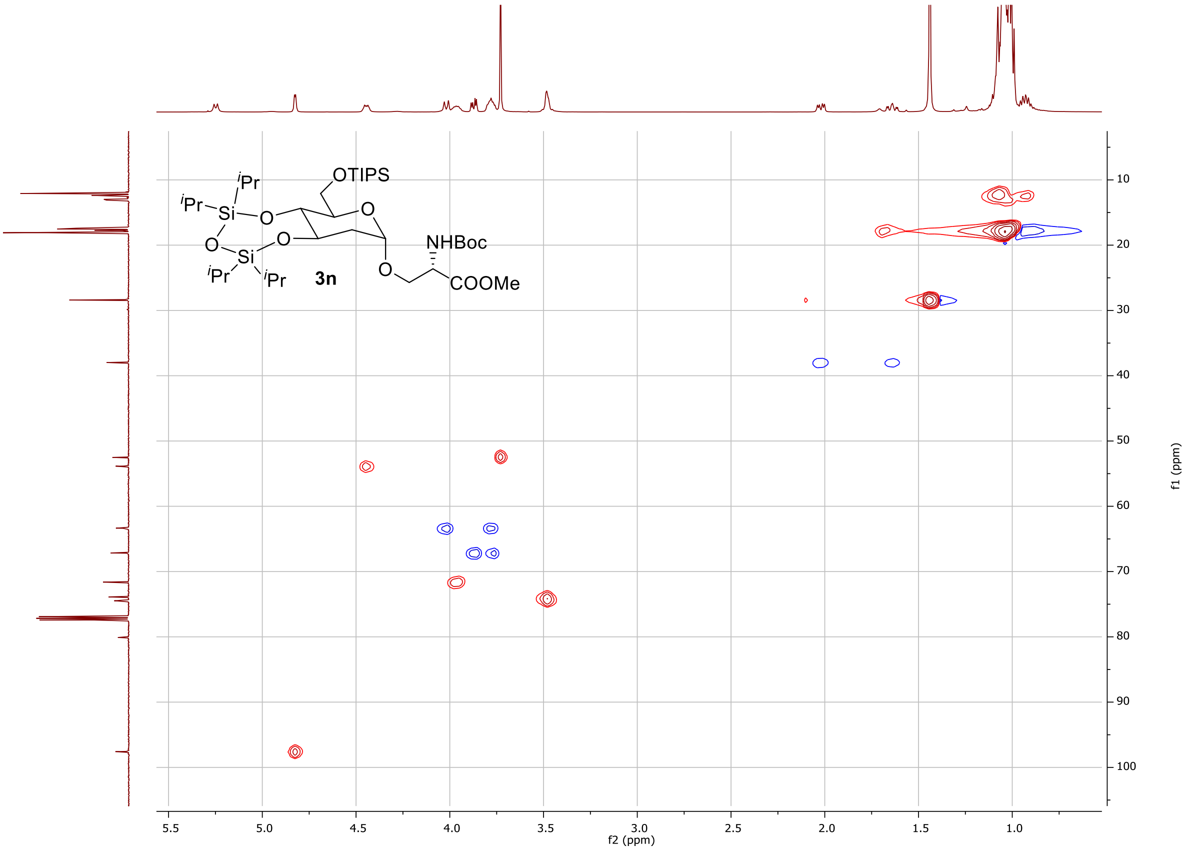

Supplementary figure S199: HSQC spectra for **3n**

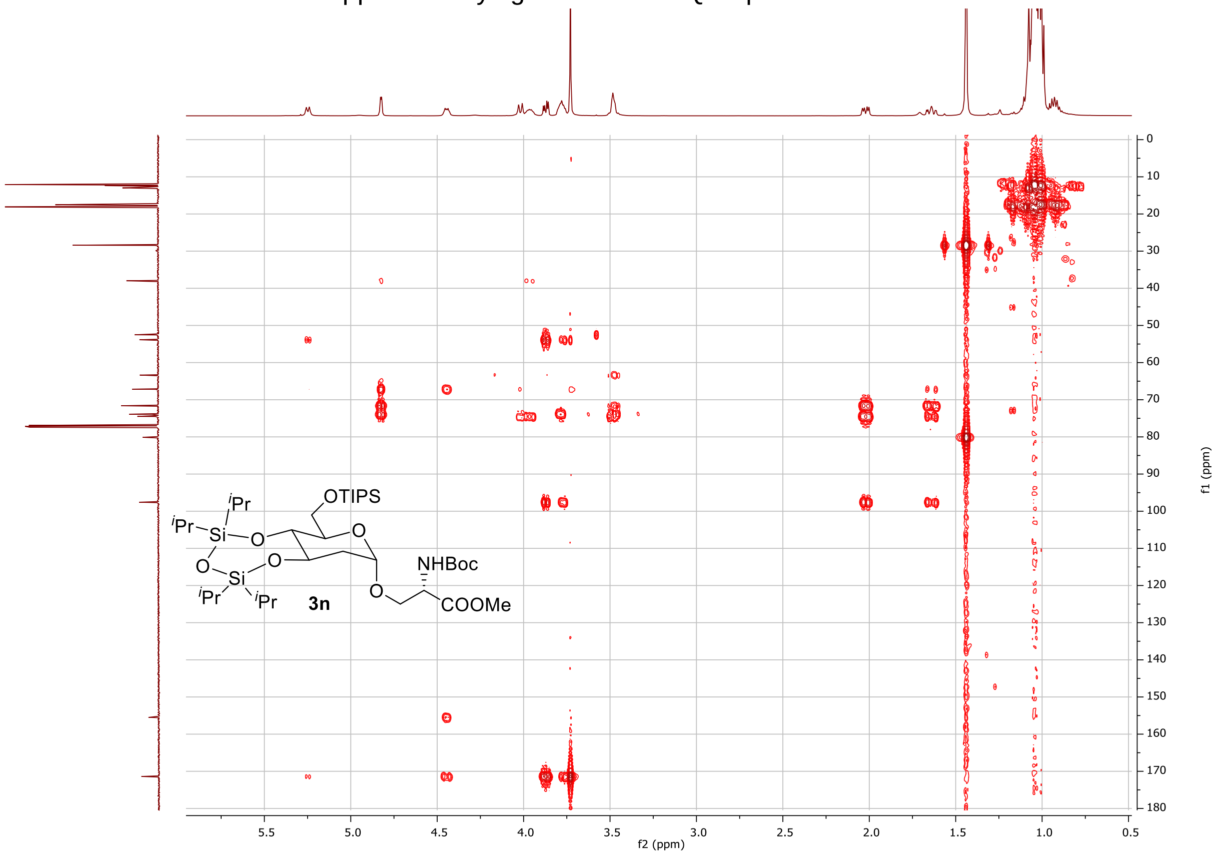

Supplementary figure S200: HMBC spectra for **3n**

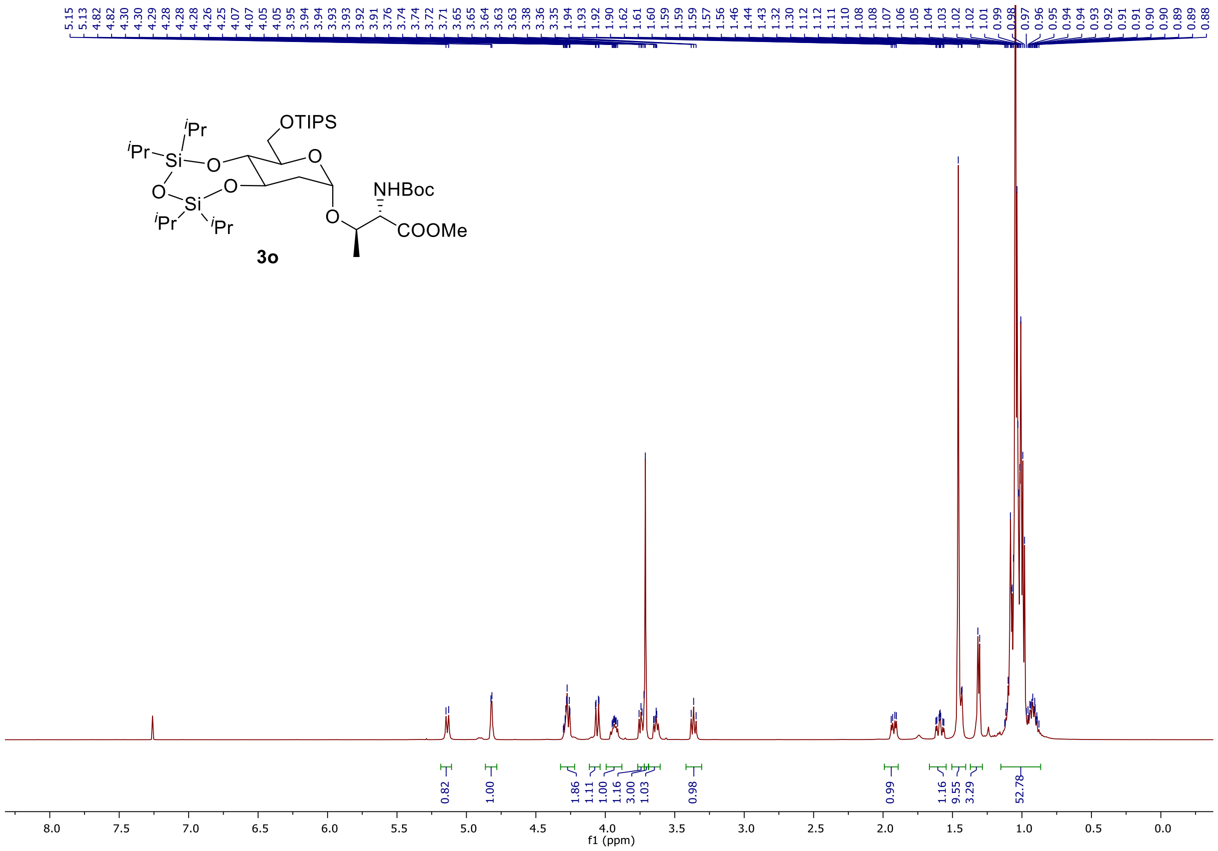

Supplementary figure S201: ^{1}H spectra for **3o**

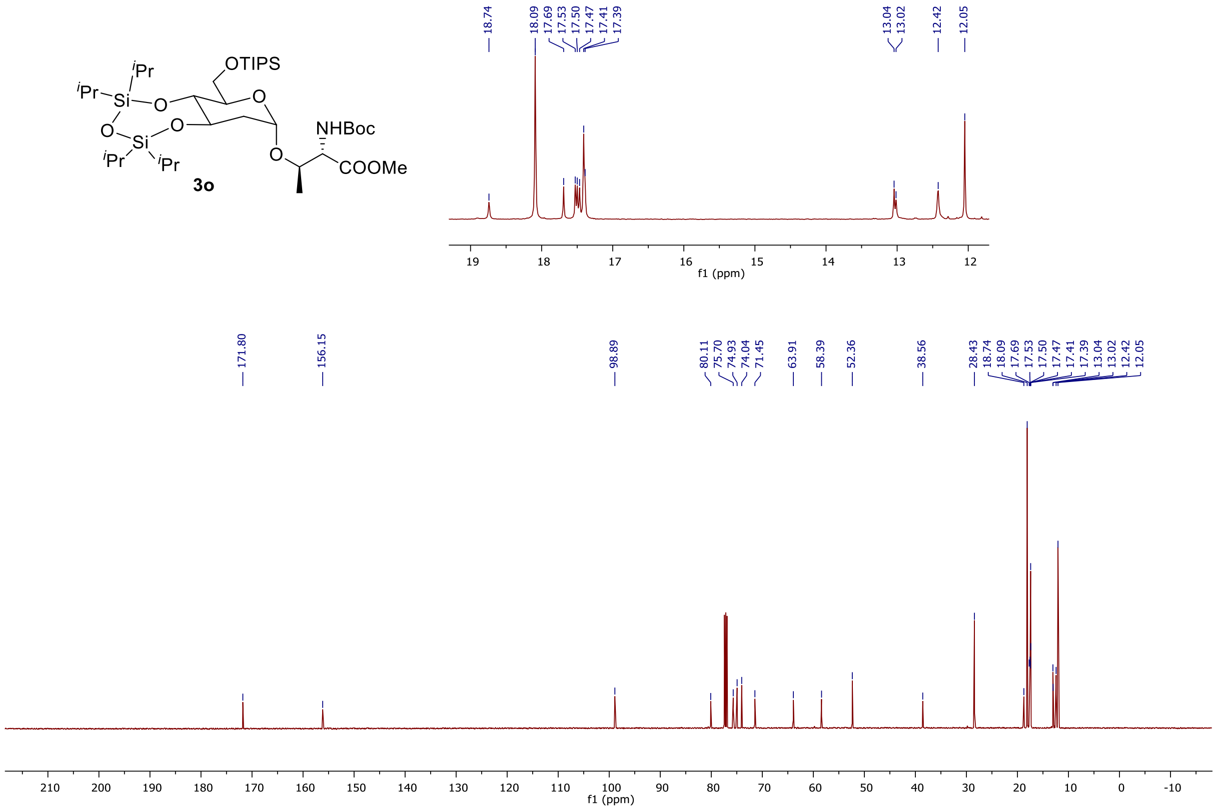

Supplementary figure 202: ^{13}C spectra for **3o**

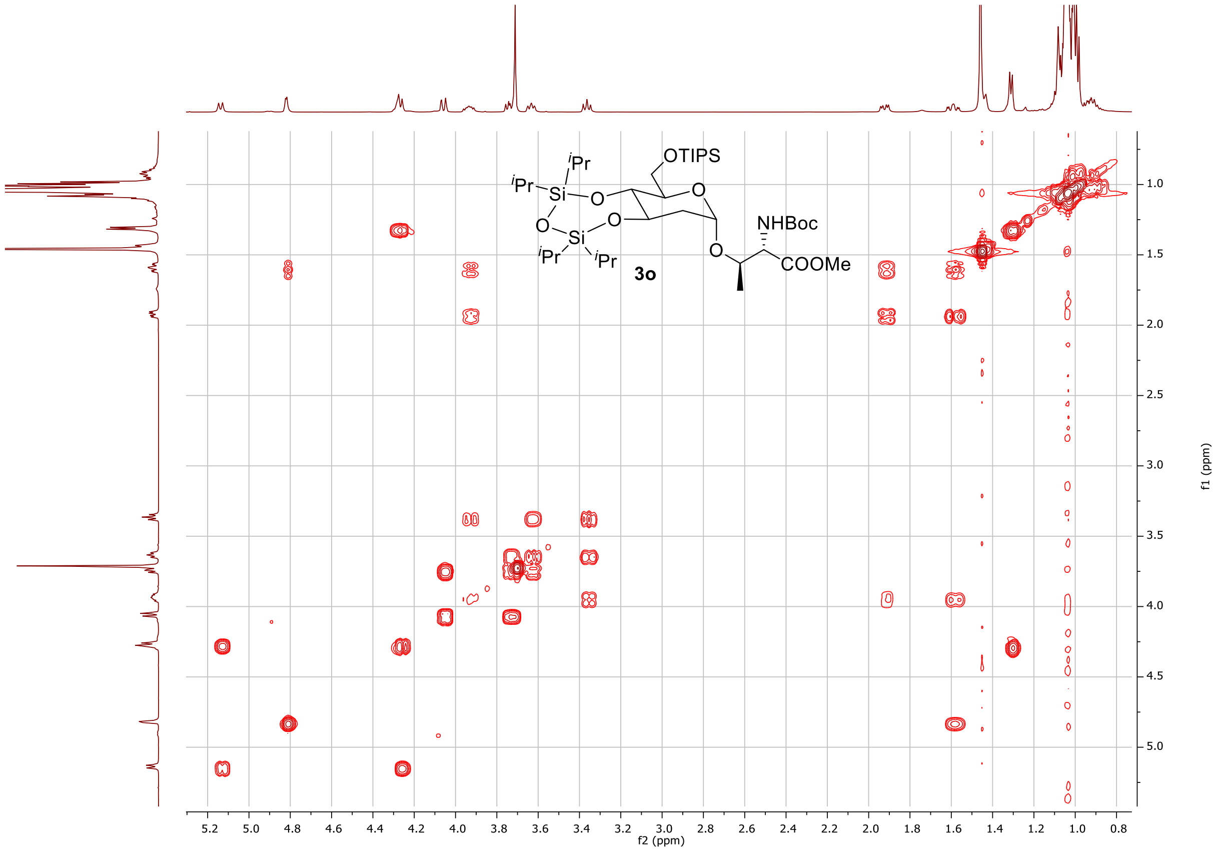

Supplementary figure S203: COSY spectra for **3o**

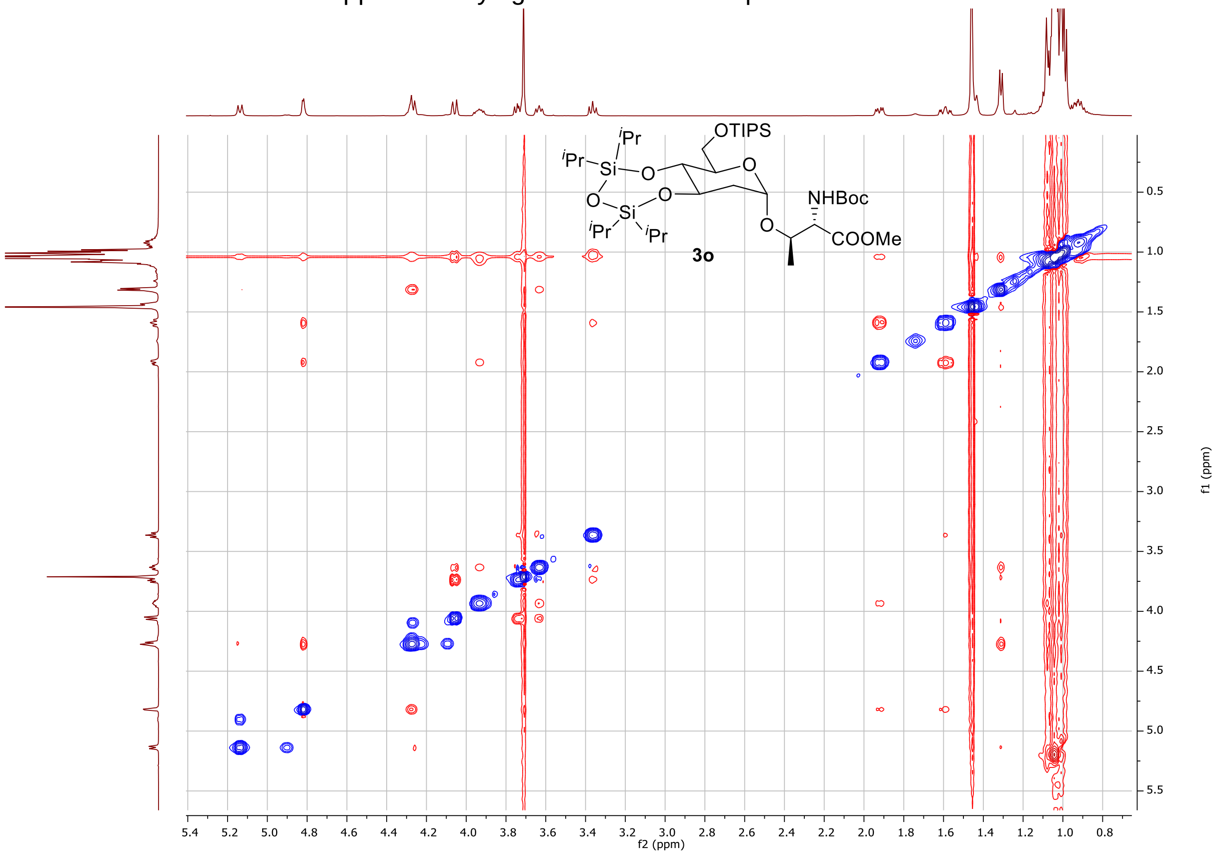

Supplementary figure S204: NOESY spectra for **3o**

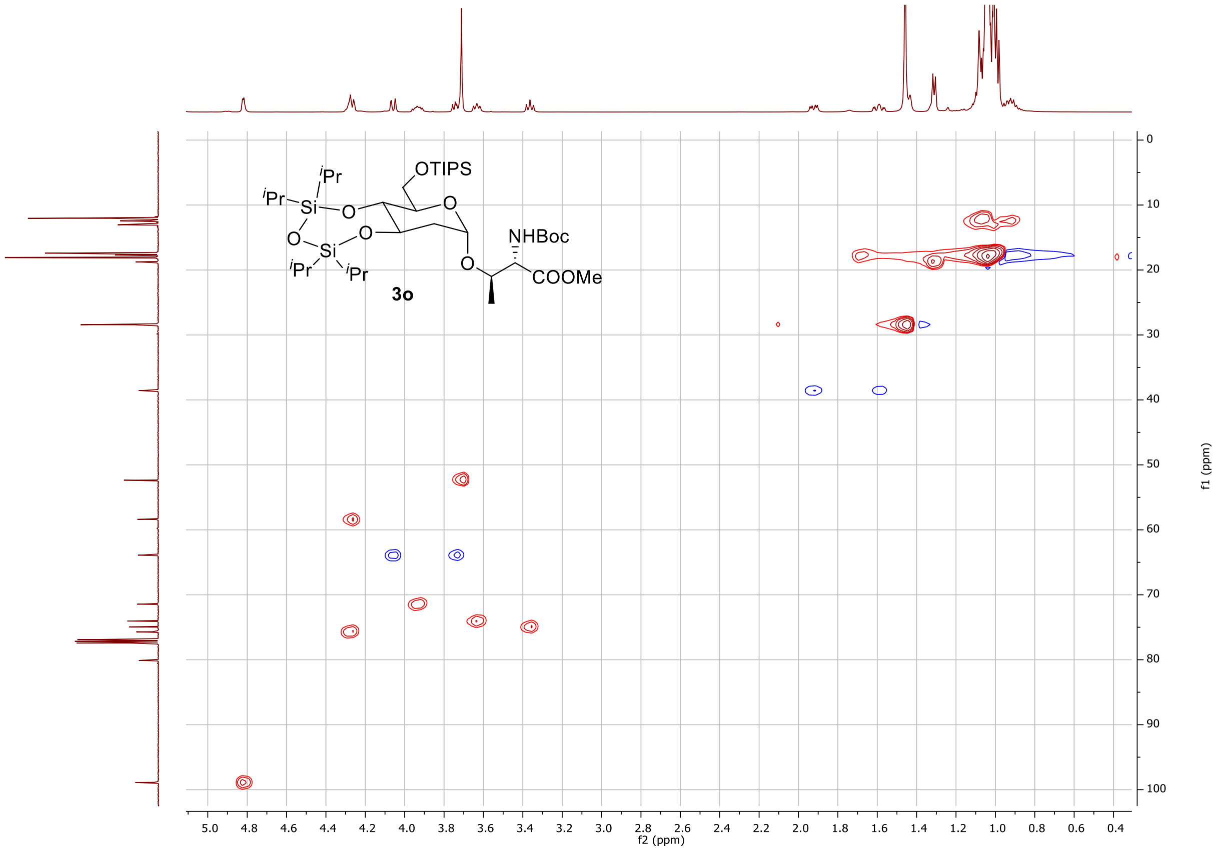

Supplementary figure S205: HSQC spectra for **3o**

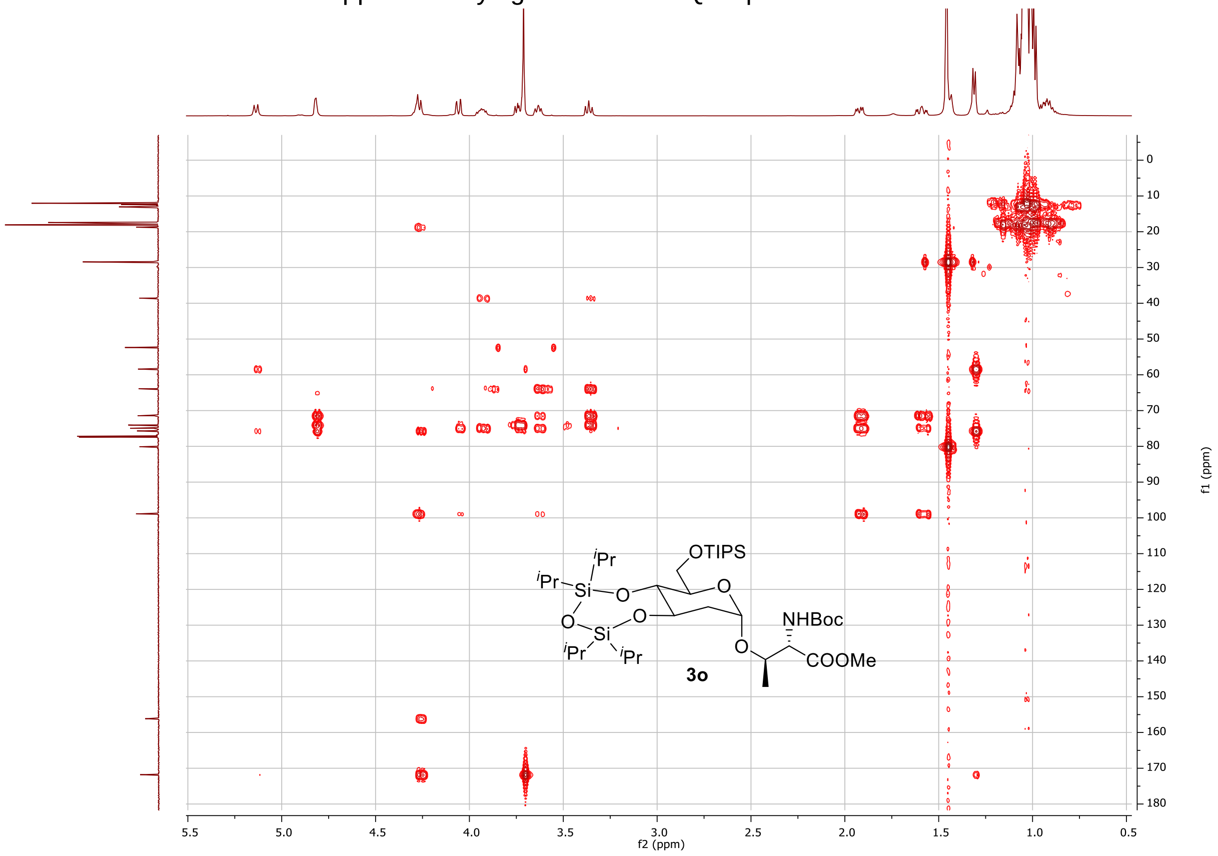

Supplementary figure S206: HMBC spectra for **3o**

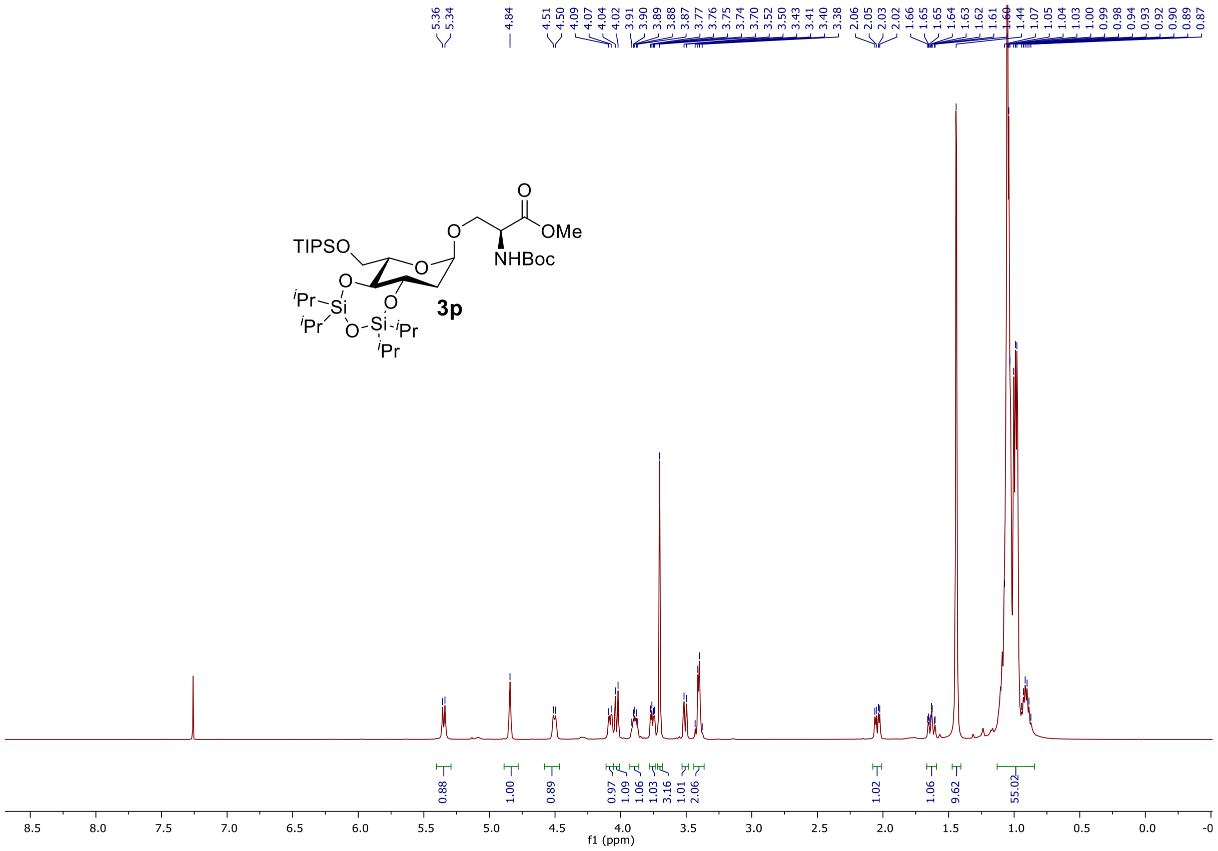

Supplementary figure S207: ^{1}H spectra for **3p**

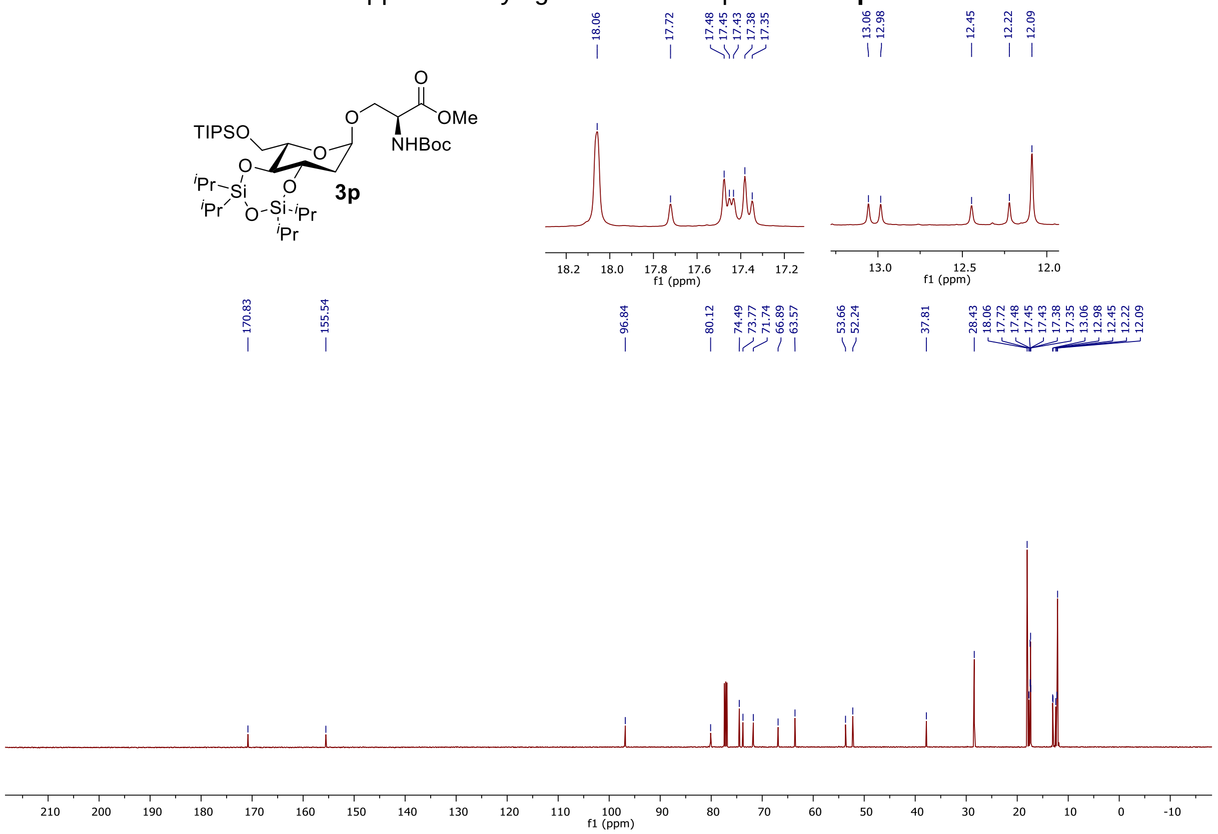

Supplementary figure S208: ^{13}C spectra for **3p**

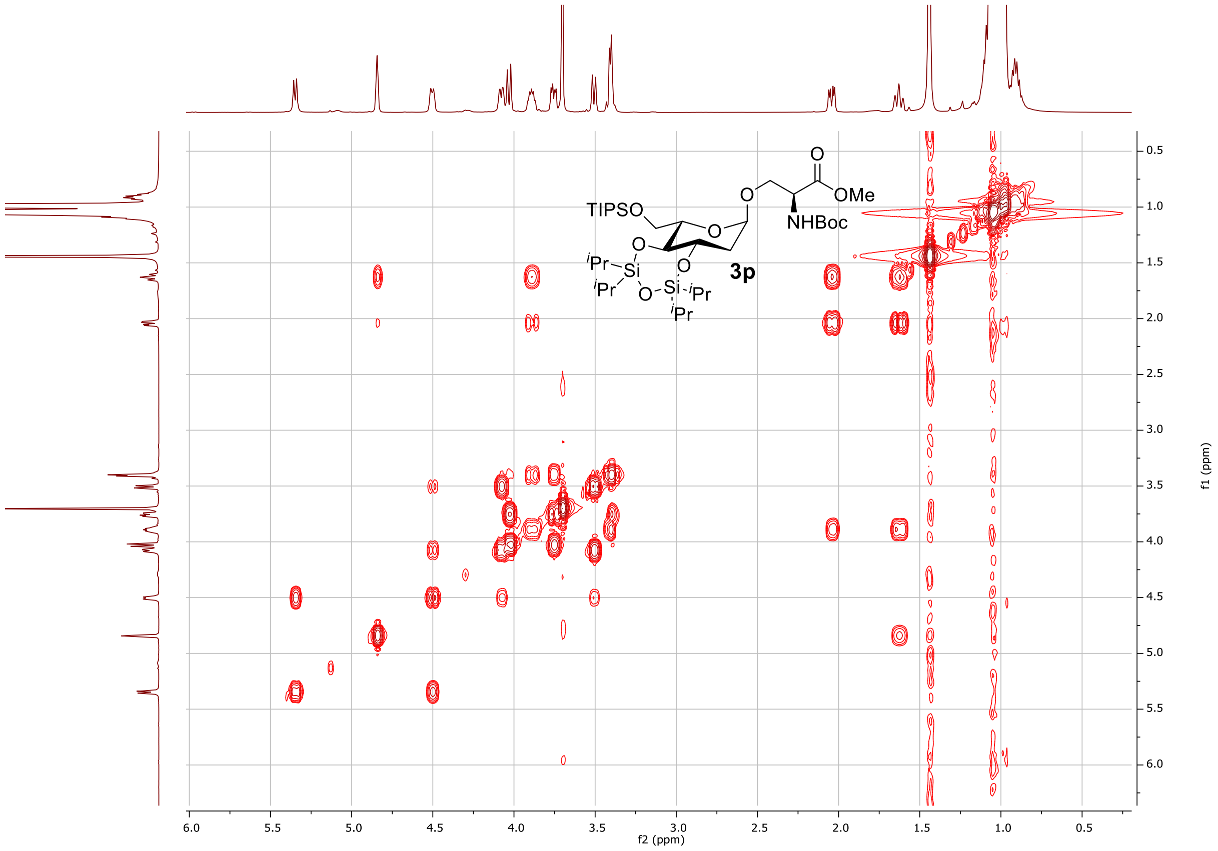

Supplementary figure S209: COSY spectra for **3p**

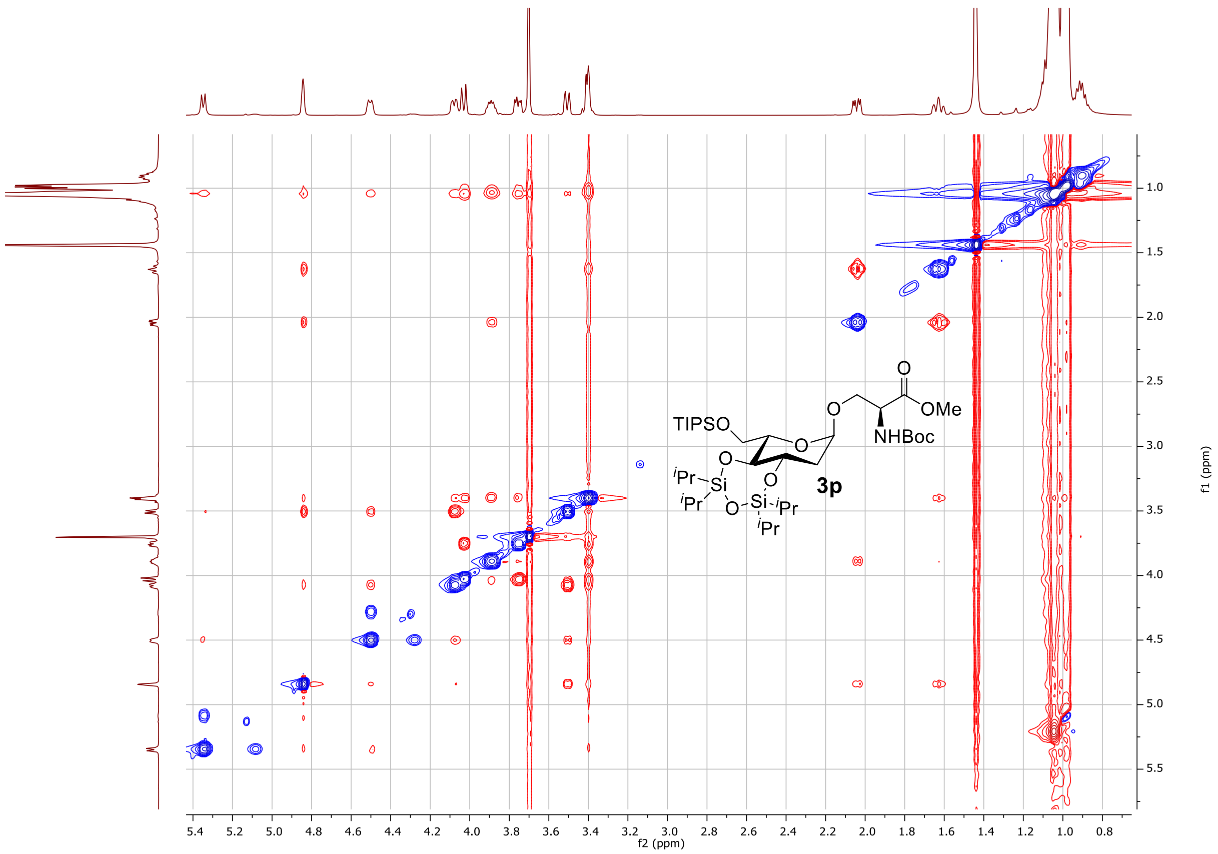

Supplementary figure S210: NOESY spectra for **3p**

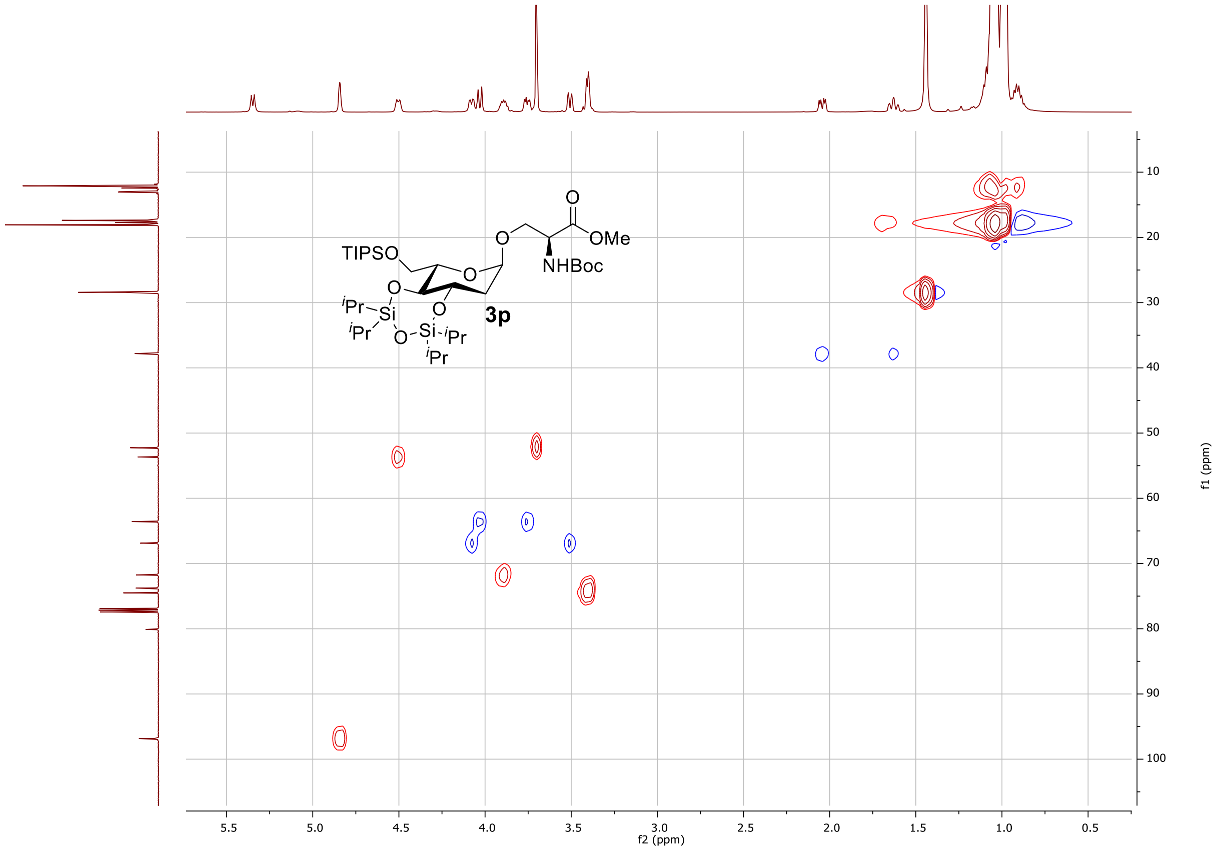

Supplementary figure S211: HSQC spectra for **3p**

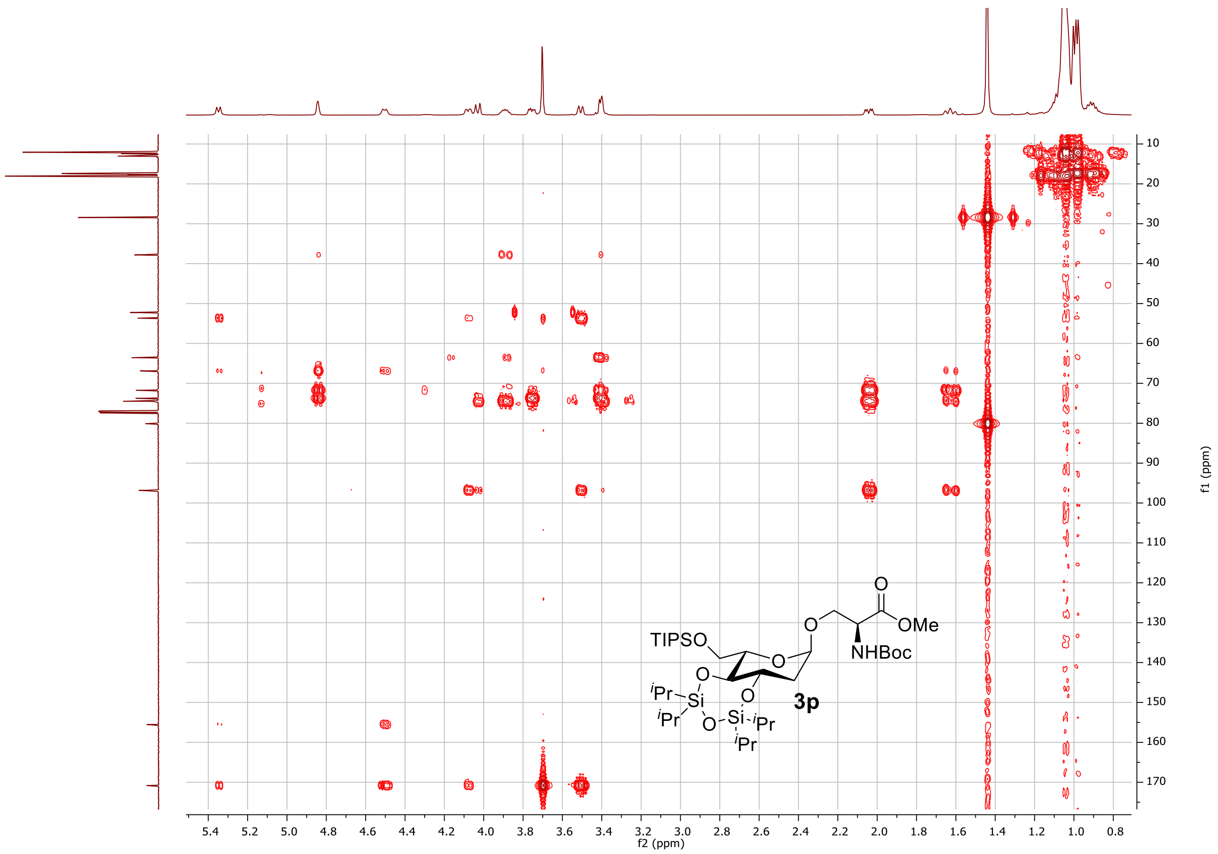

Supplementary figure S212: HMBC spectra for **3p**

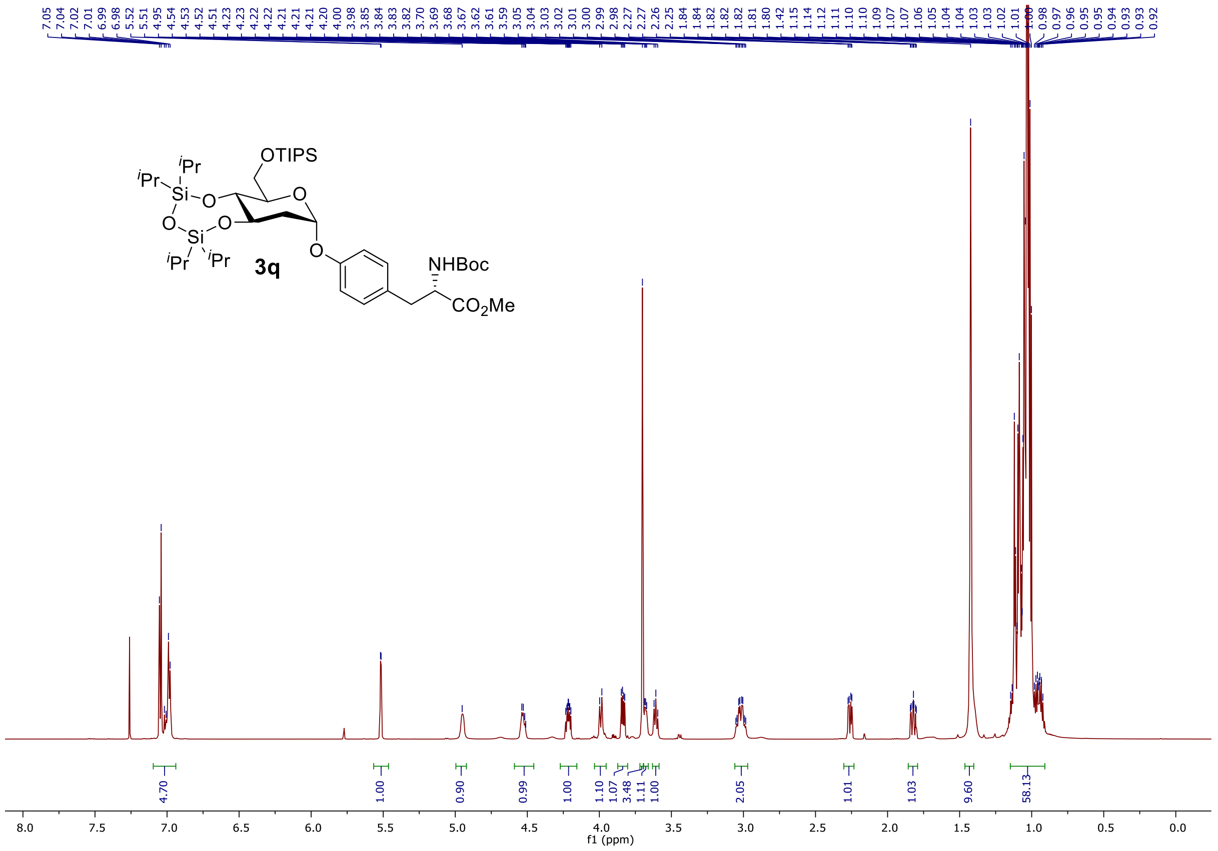

Supplementary figure S213: ^{1}H spectra for **3q**

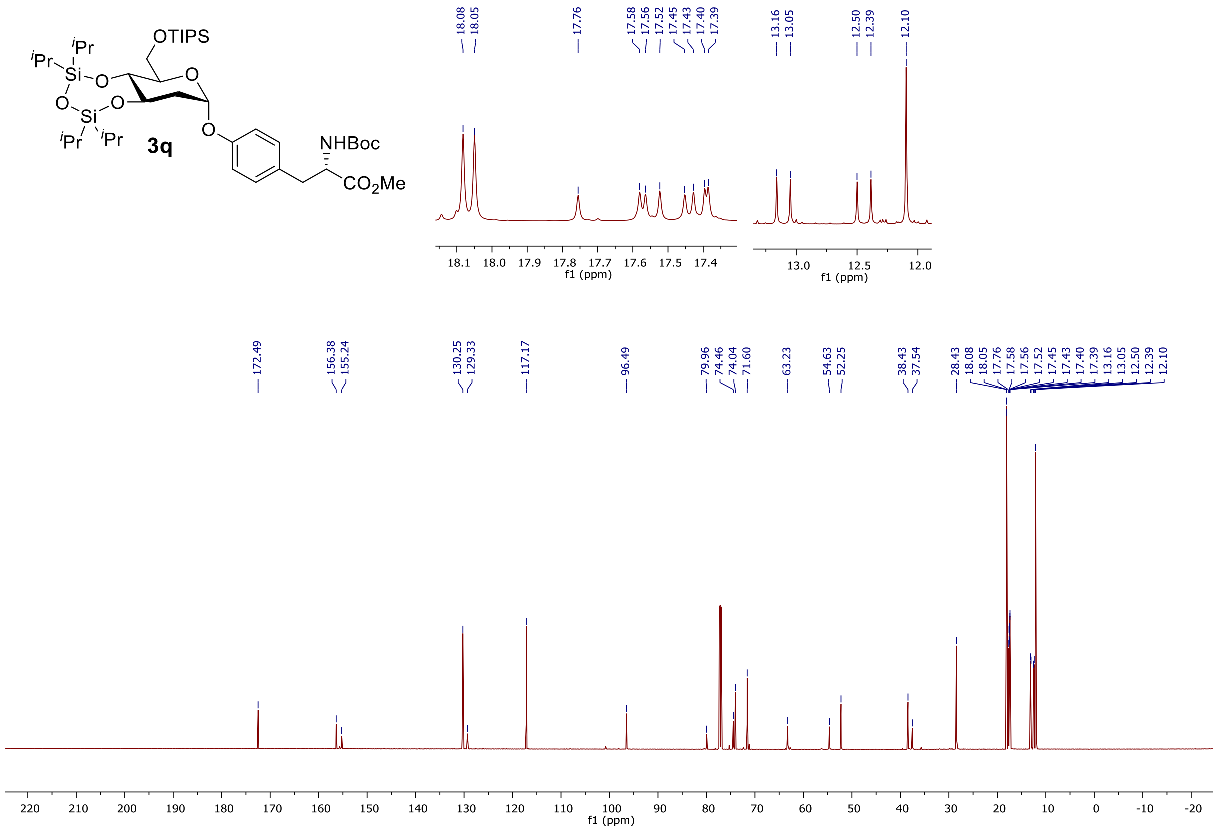

Supplementary figure S214: ^{13}C spectra for **3q**

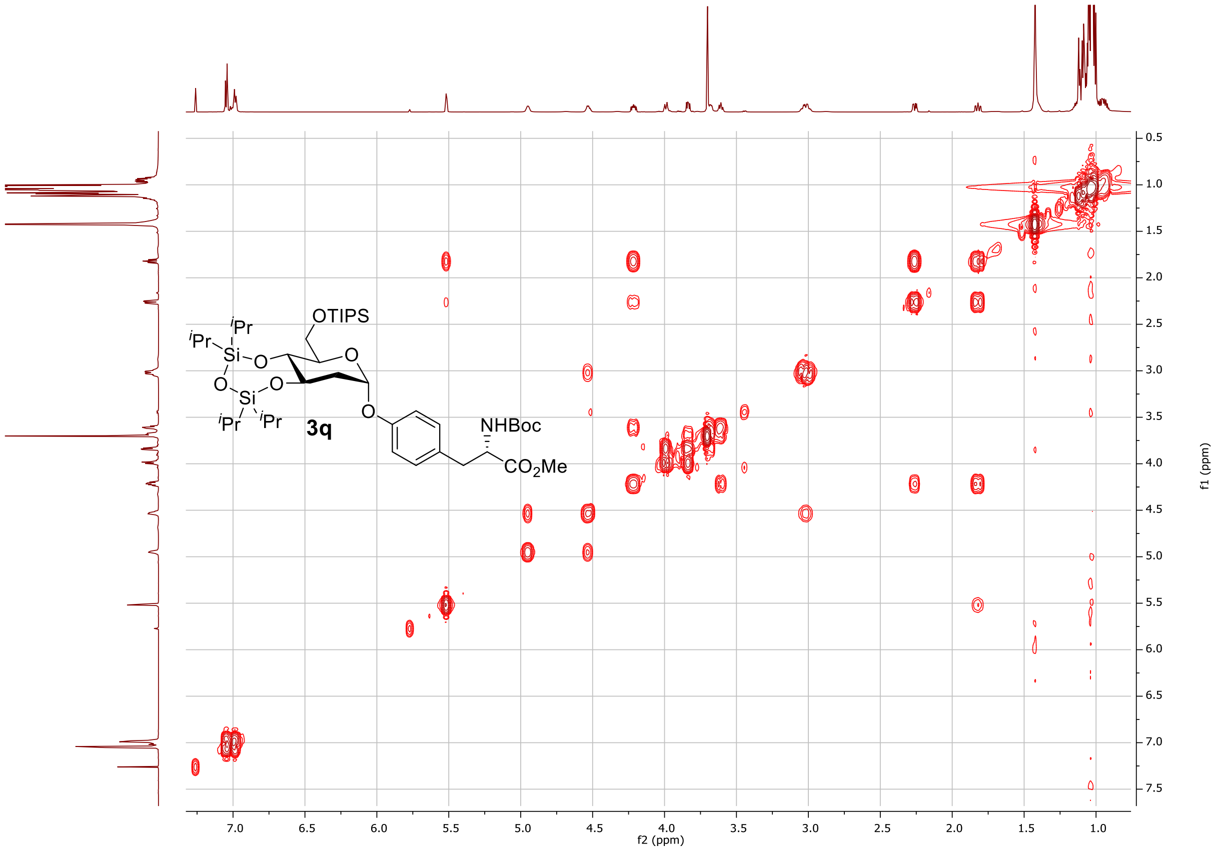

Supplementary figure S215: COSY spectra for **3q**

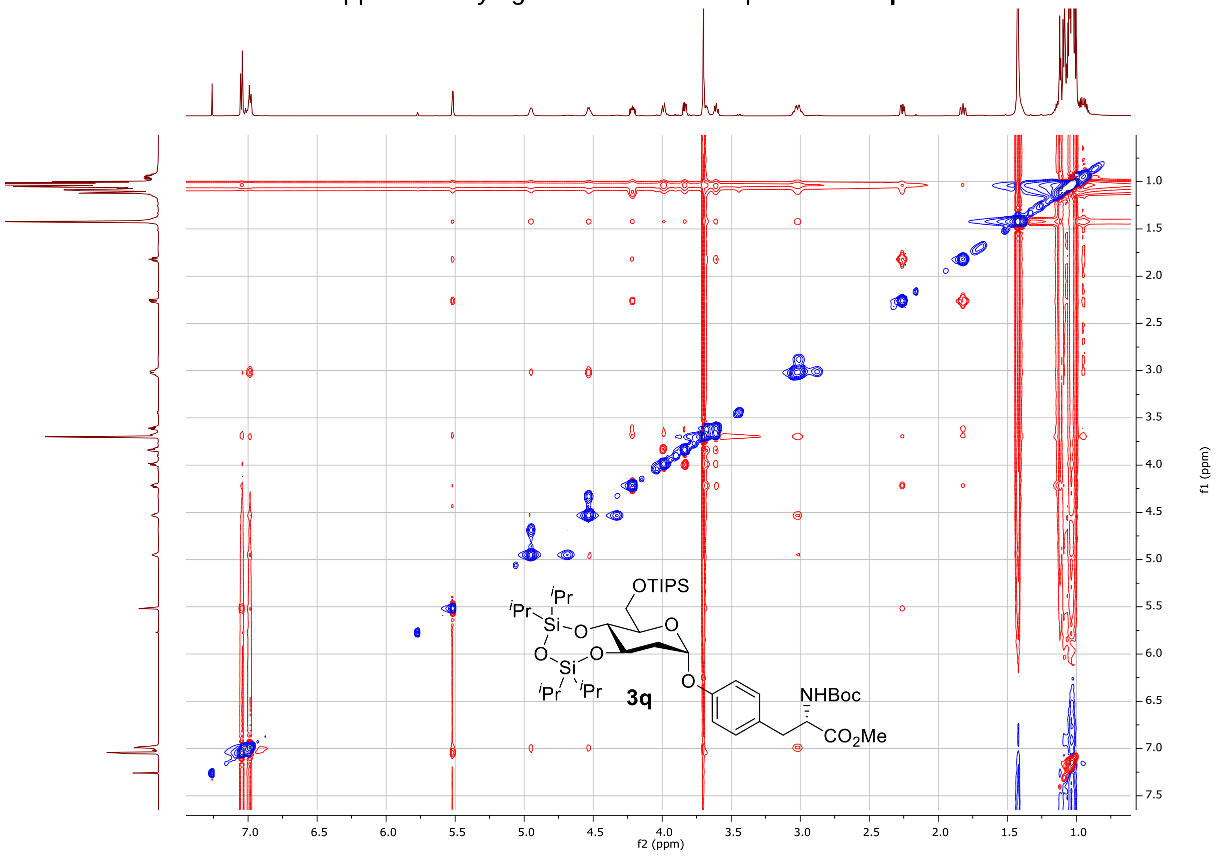

Supplementary figure S216: NOESY spectra for **3q**

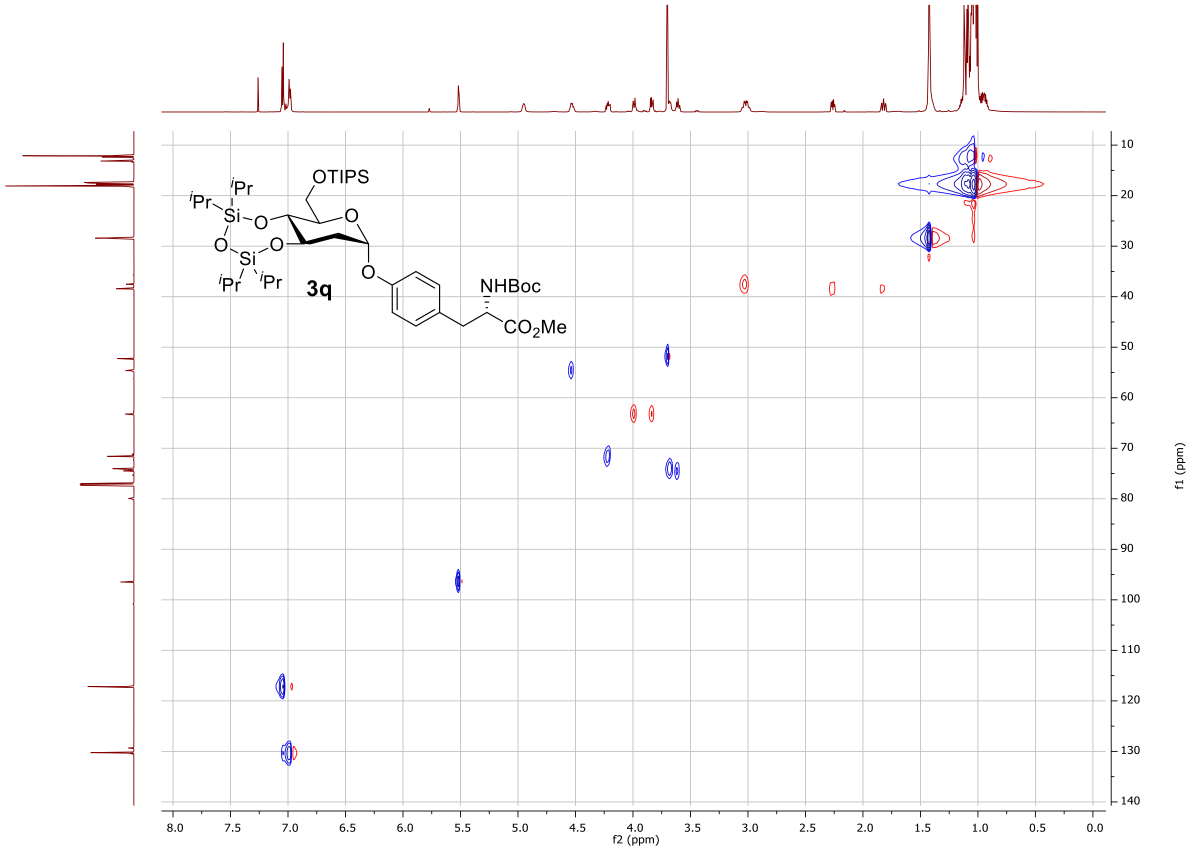

Supplementary figure S217: HSQC spectra for **3q**

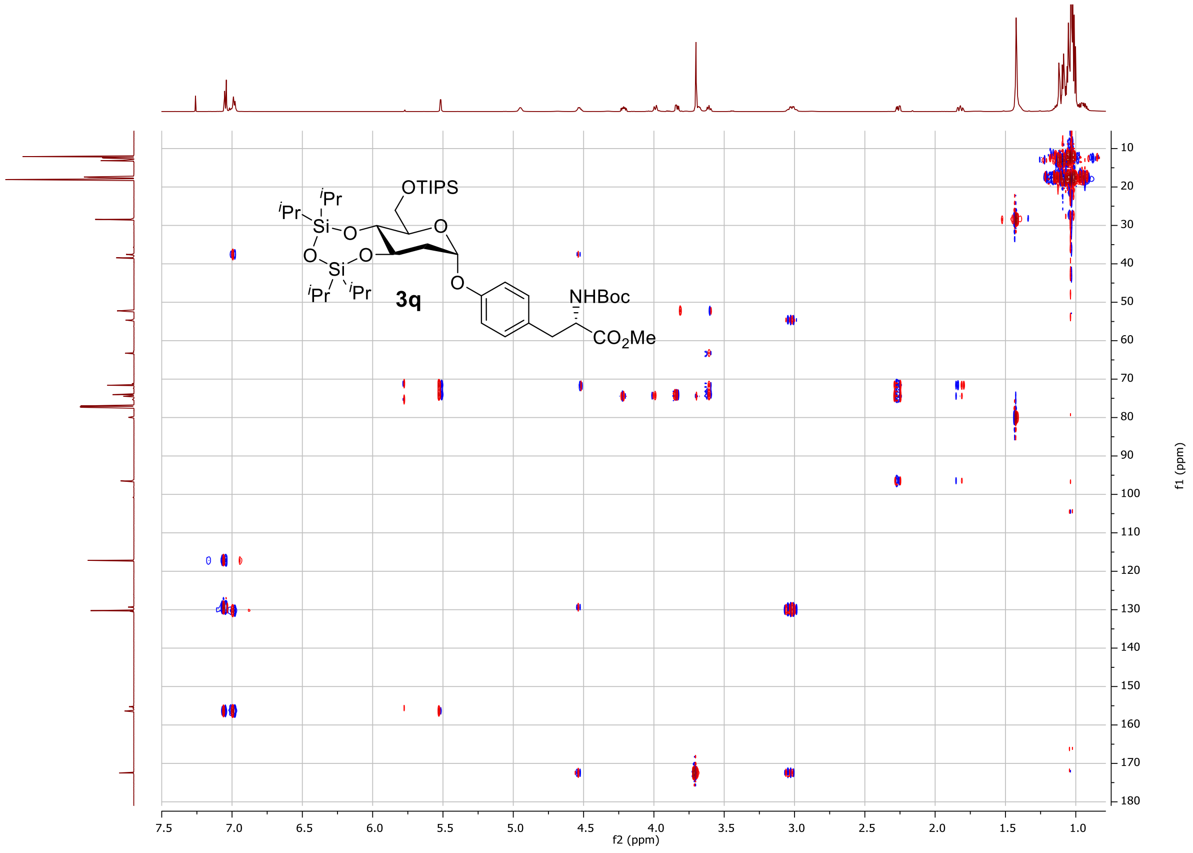

Supplementary figure S218: HMBC spectra for **3q**

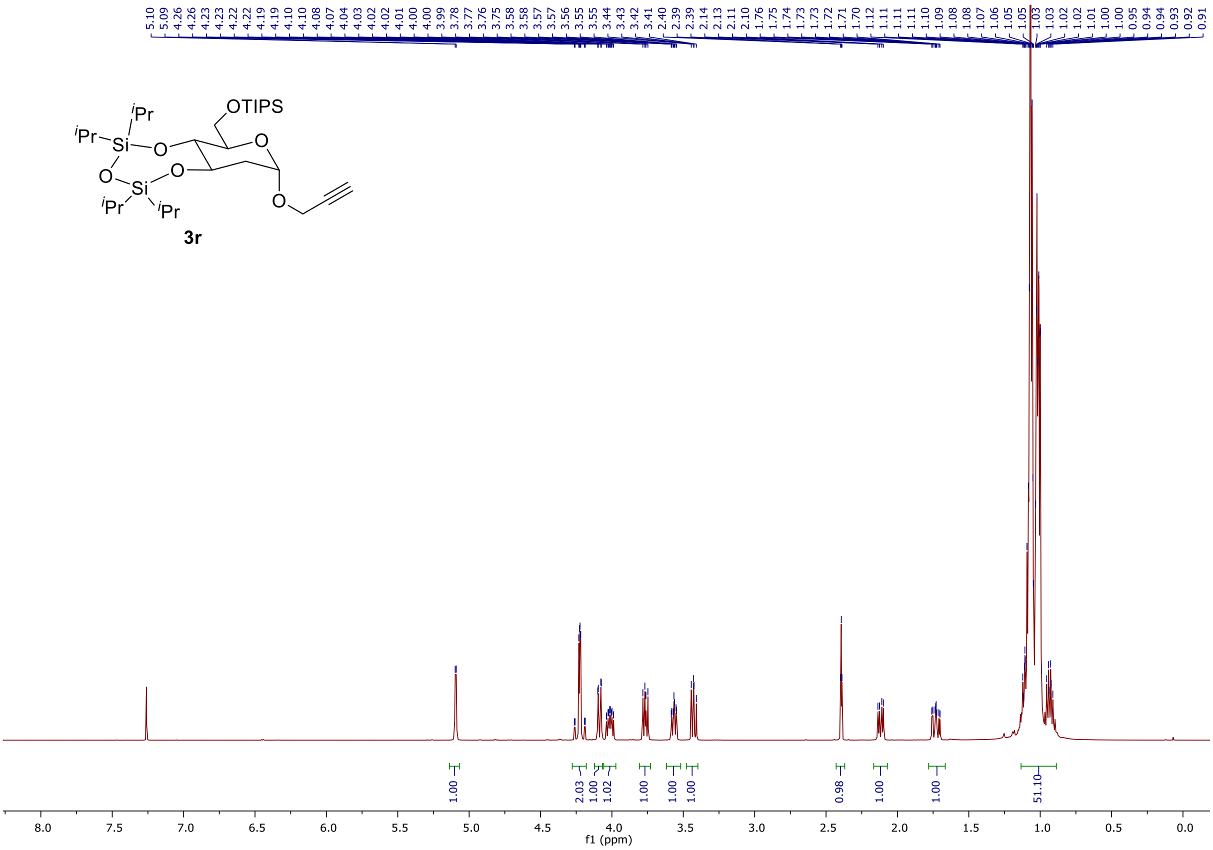

Supplementary figure S219: ^{1}H spectra for **3r**

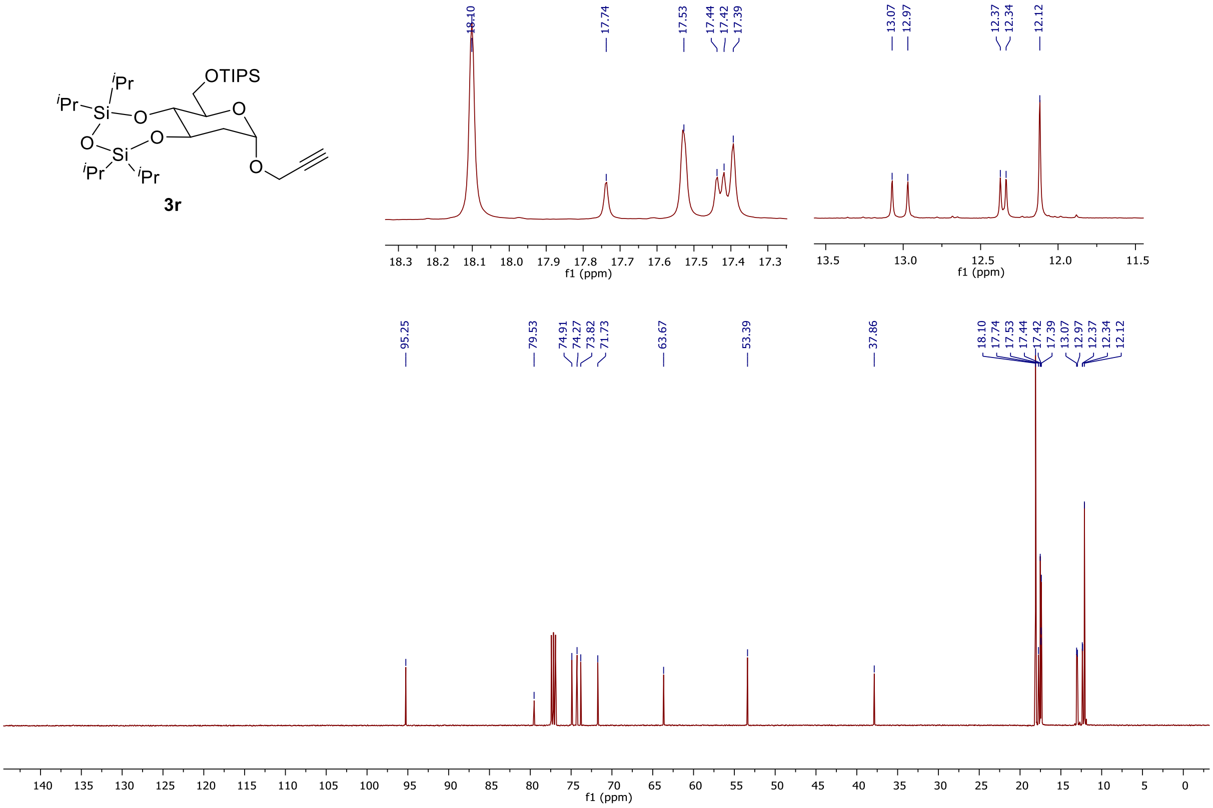

Supplementary figure S220: ^{13}C spectra for **3r**

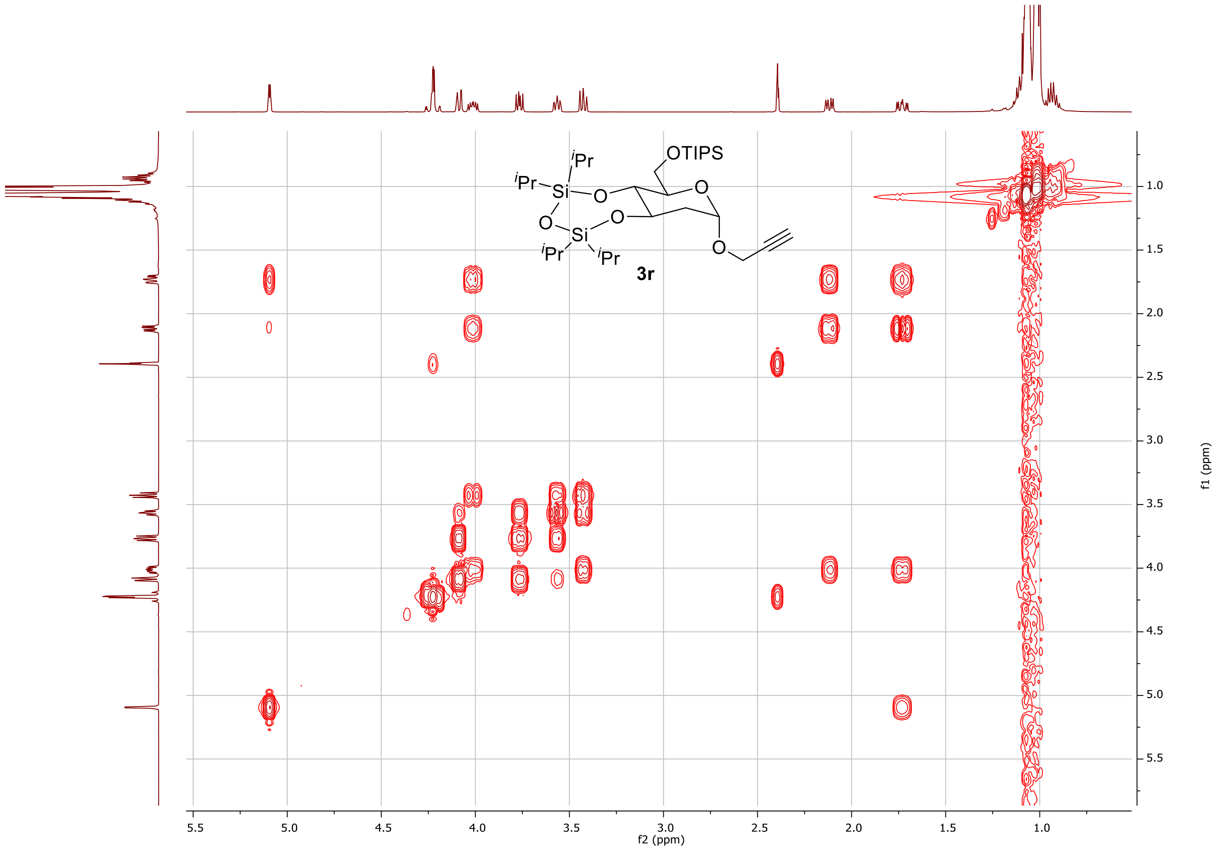

Supplementary figure S221: COSY spectra for **3r**

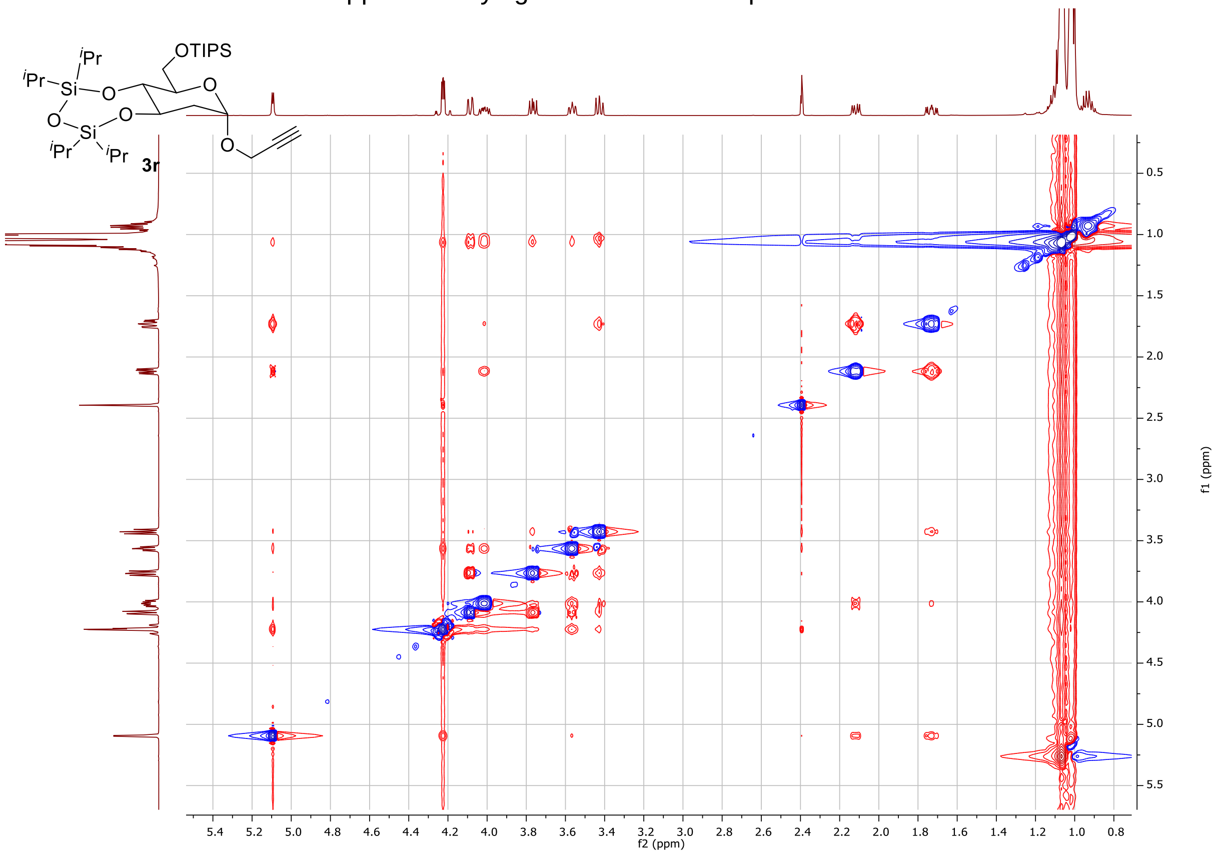

Supplementary figure S222: NOESY spectra for **3r**

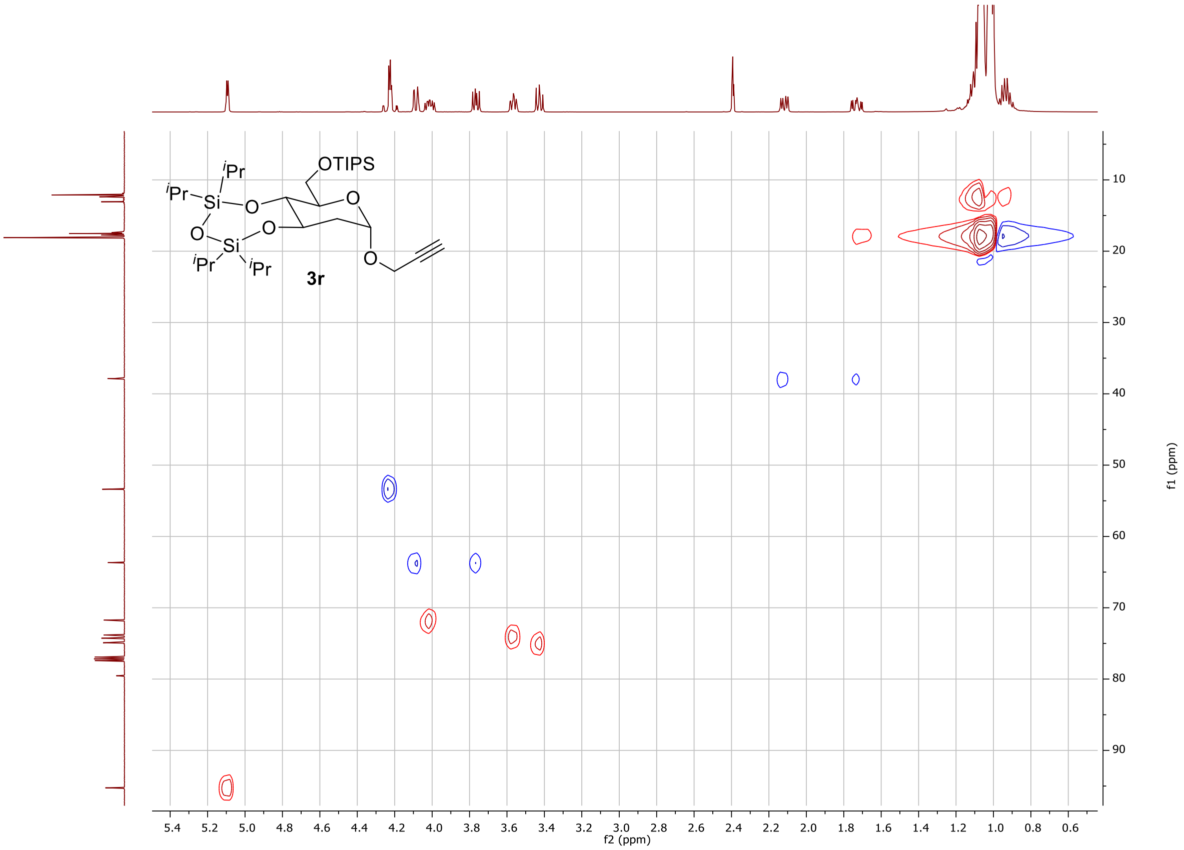

Supplementary figure S223: HSQC spectra for **3r**

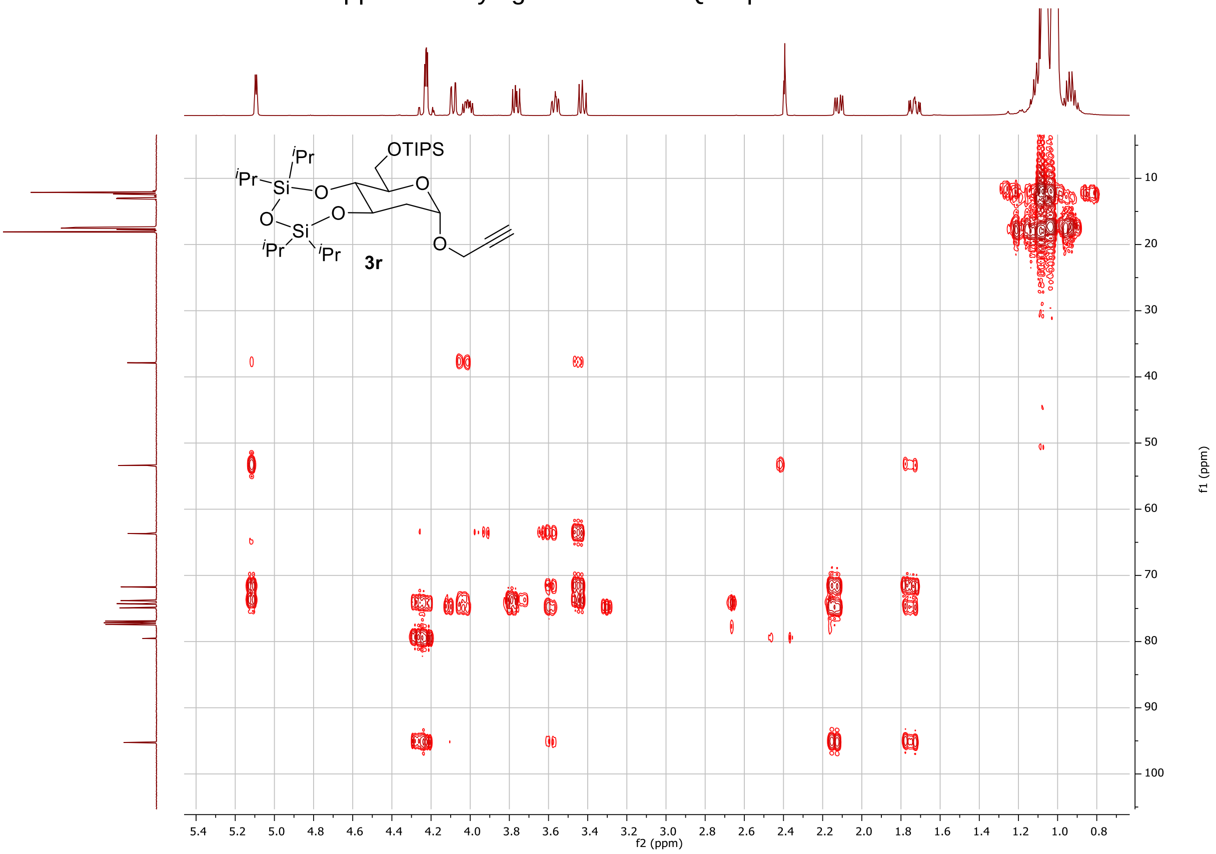

Supplementary figure S224: HMBC spectra for **3r**

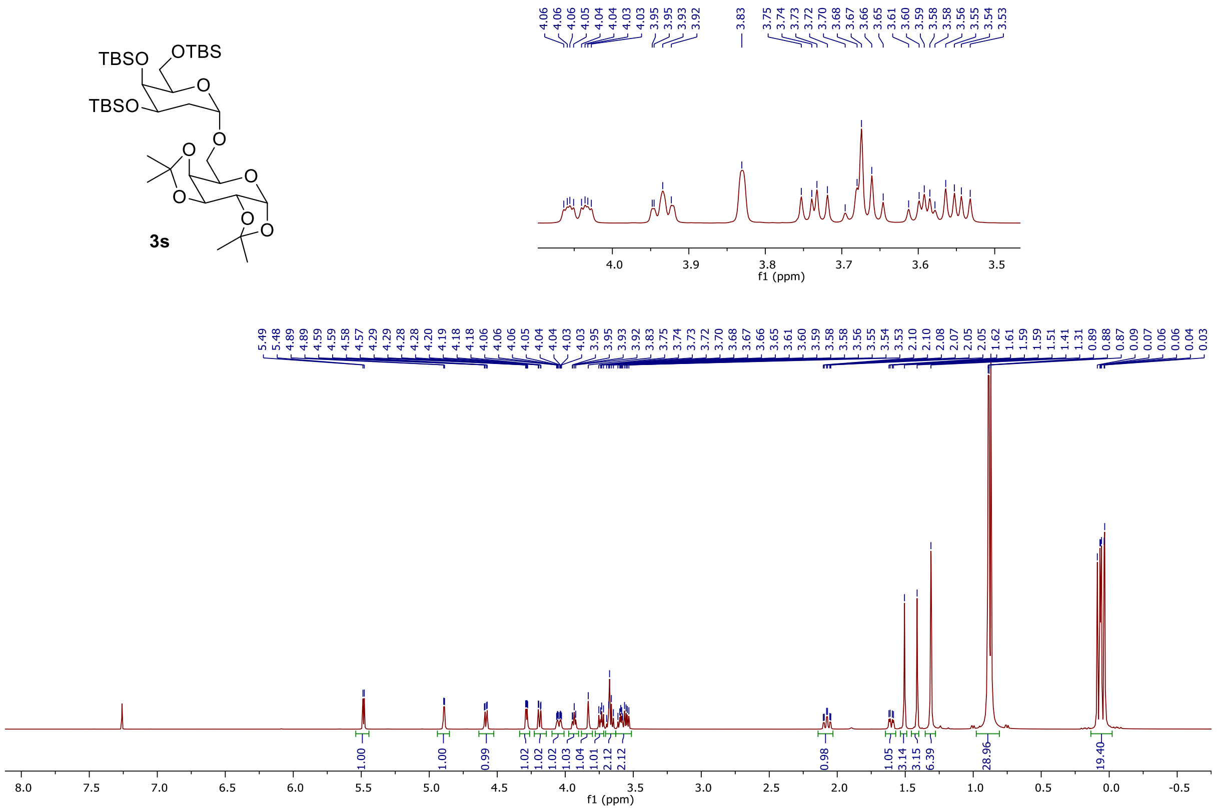

Supplementary figure S225: ^{1}H spectra for **3s**

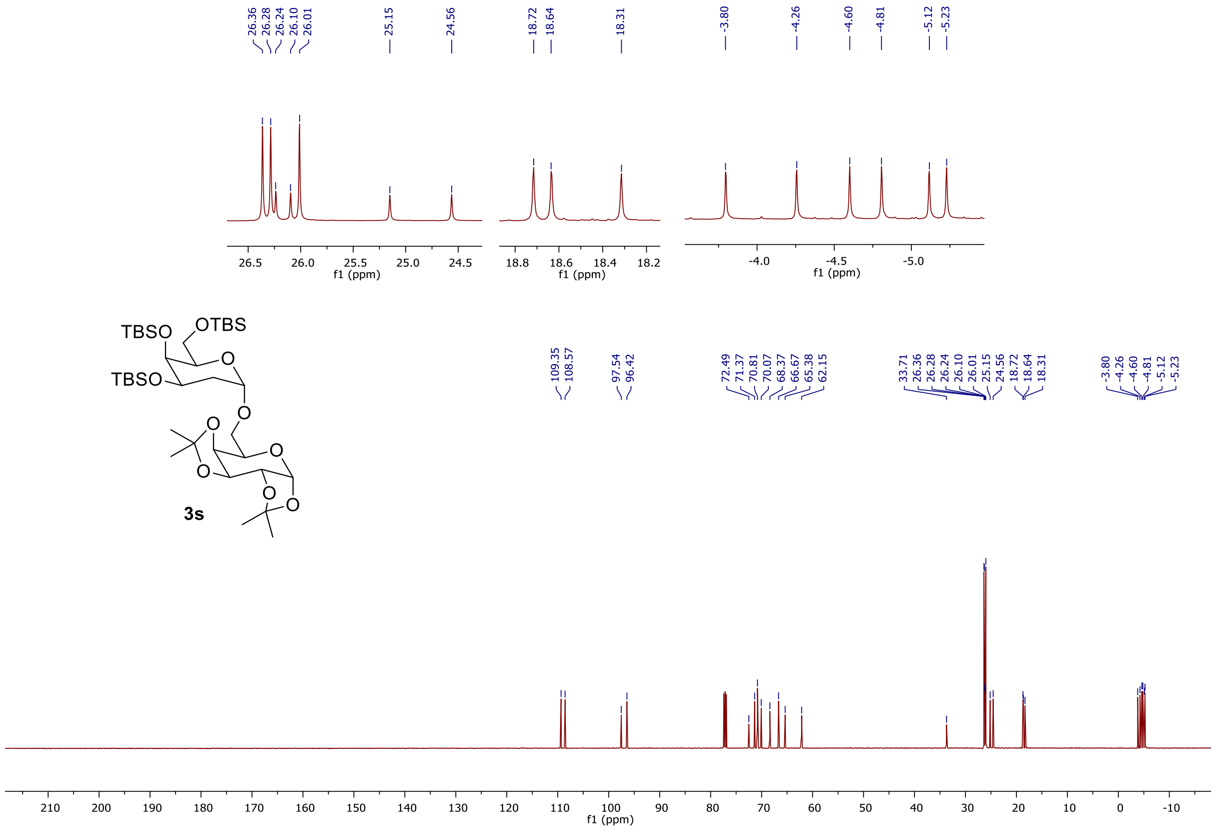

Supplementary figure S226: ^{13}C spectra for **3s**

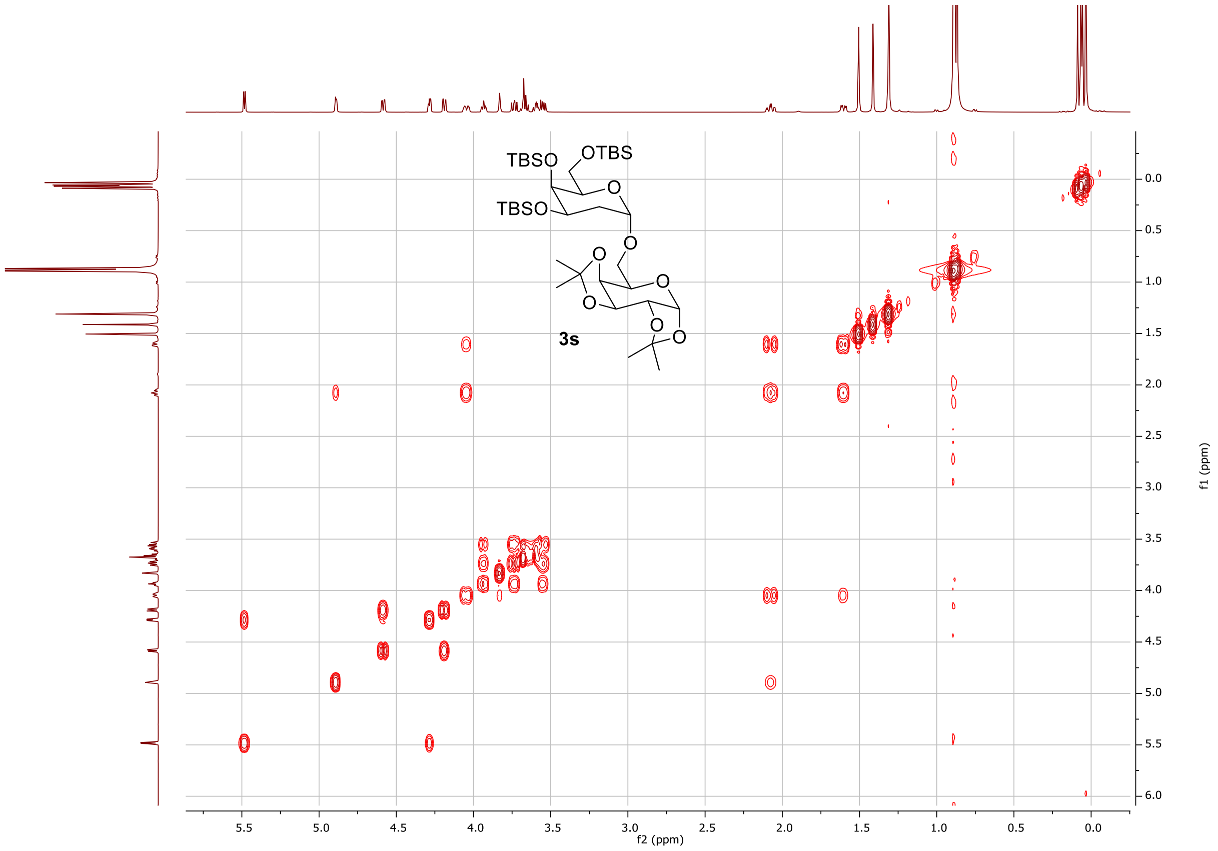

Supplementary figure S227: COSY spectra for **3s**

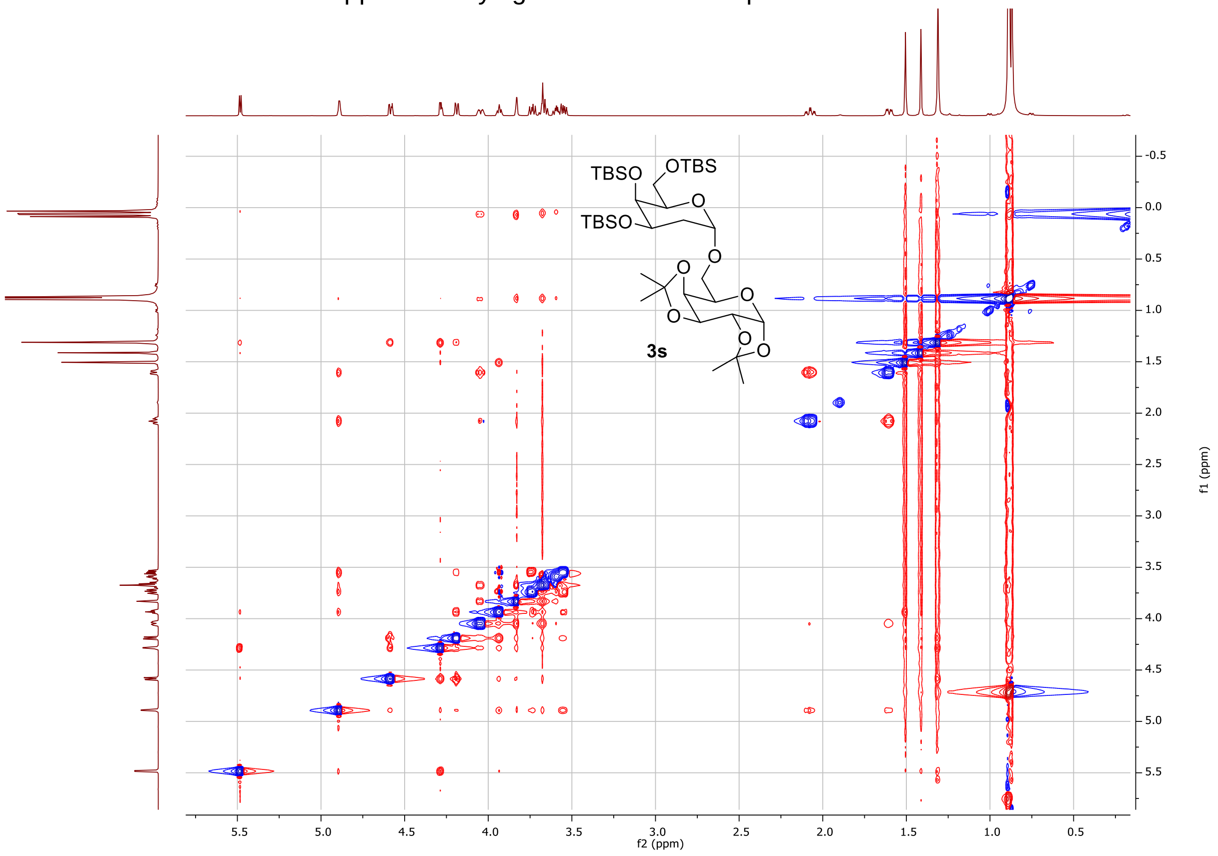

Supplementary figure S228: NOESY spectra for **3s**

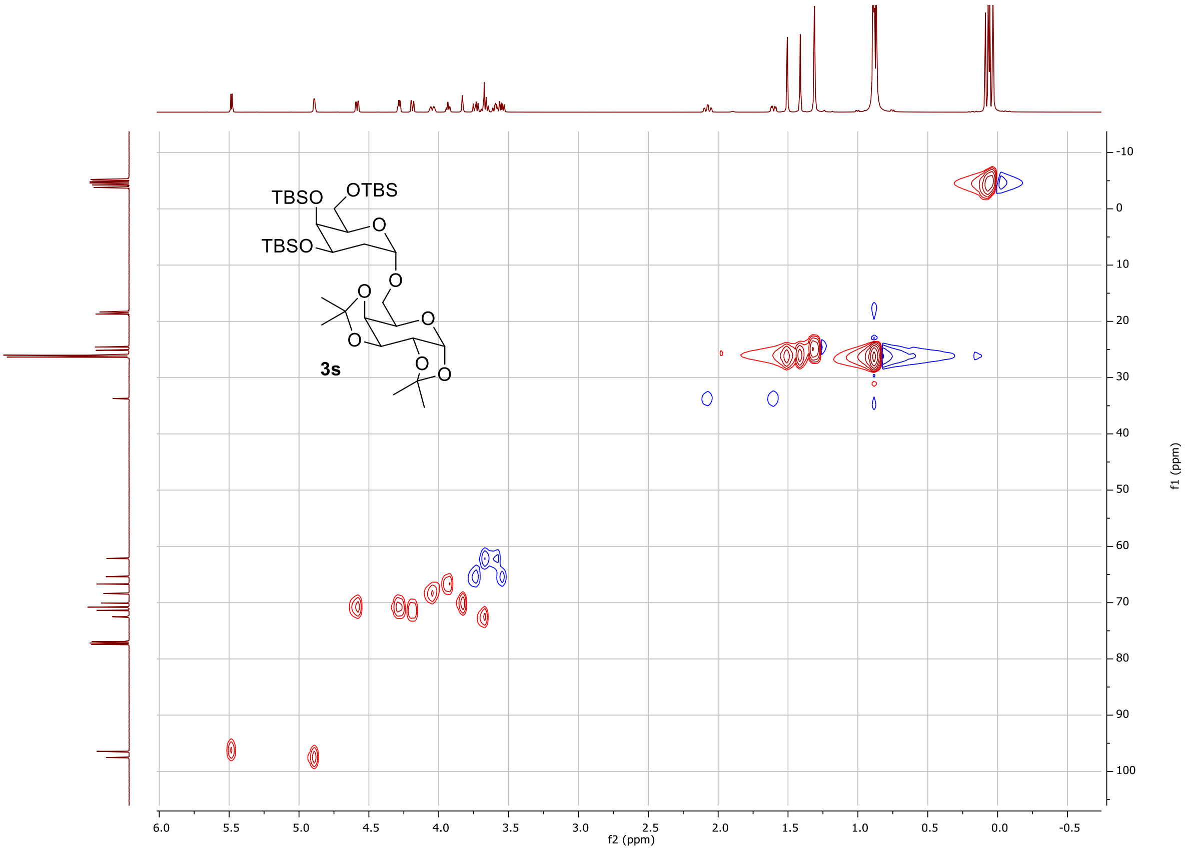

Supplementary figure S229: HSQC spectra for **3s**

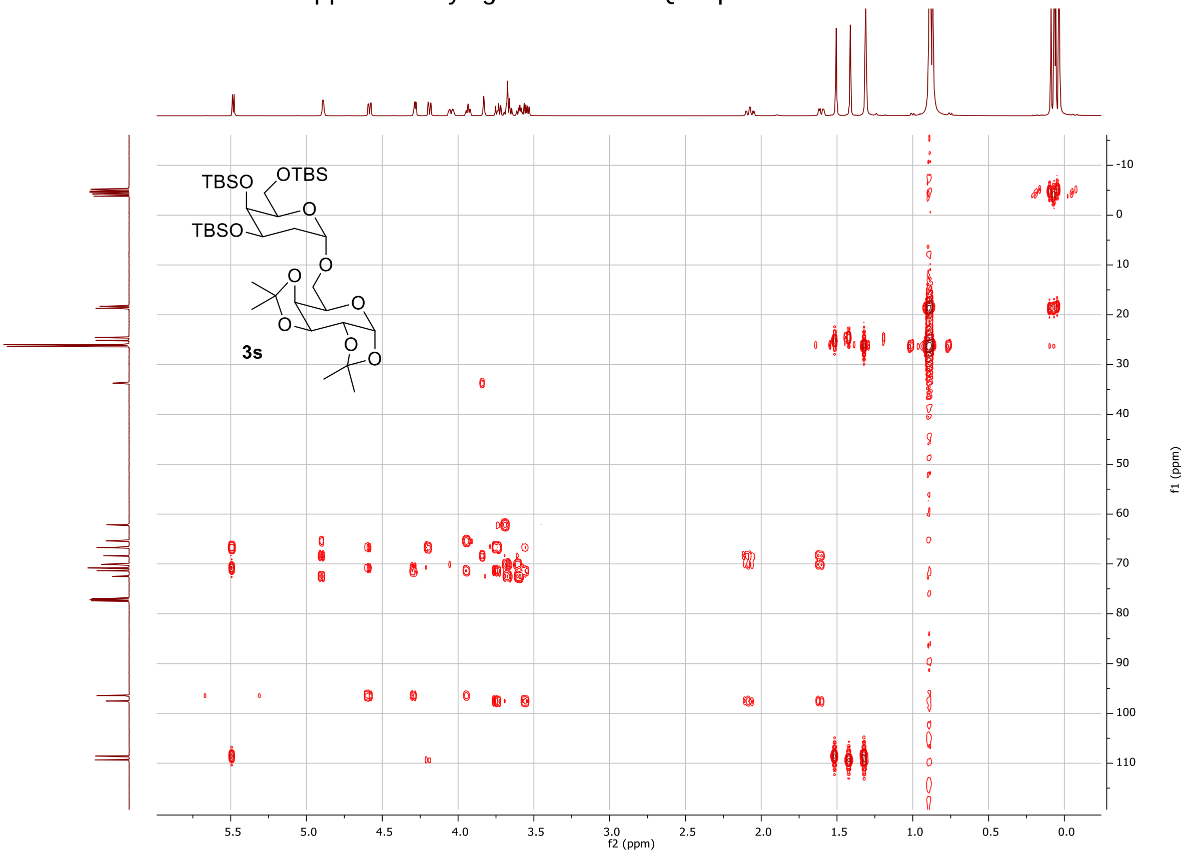

Supplementary figure S230: HMBC spectra for **3s**

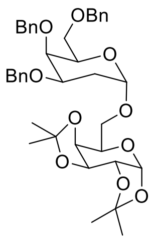

3t

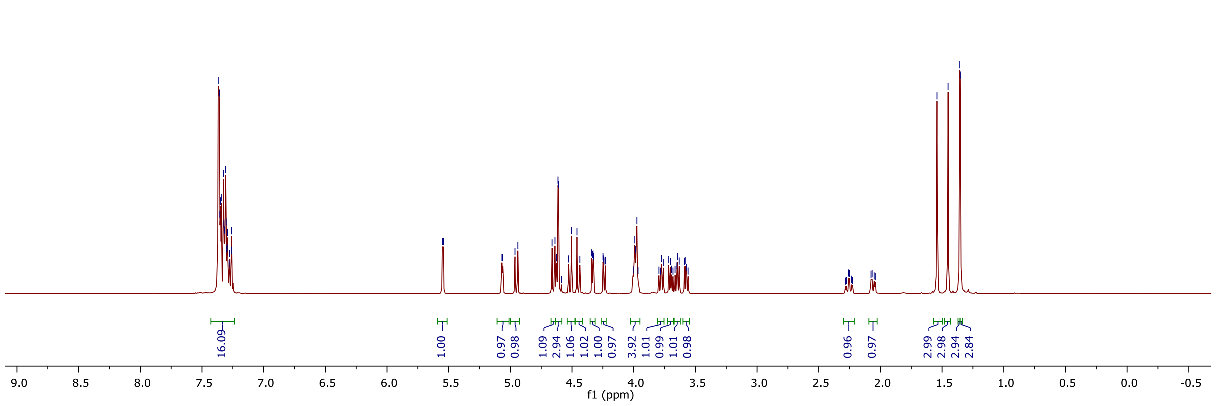

Supplementary figure S231: ^{1}H spectra for **3t**

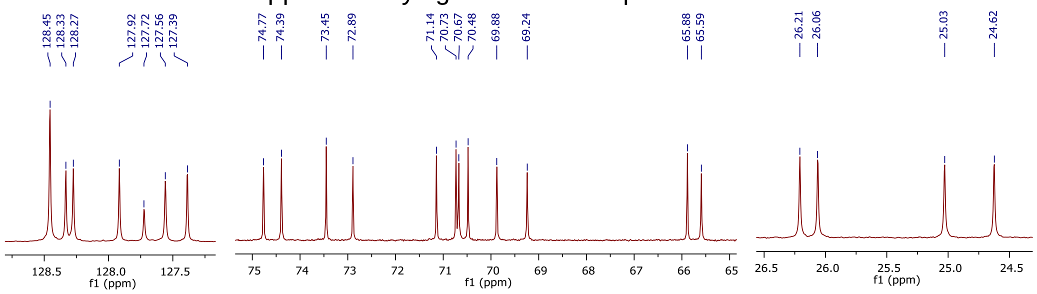

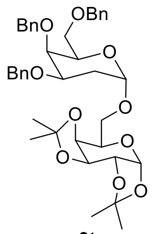

3t

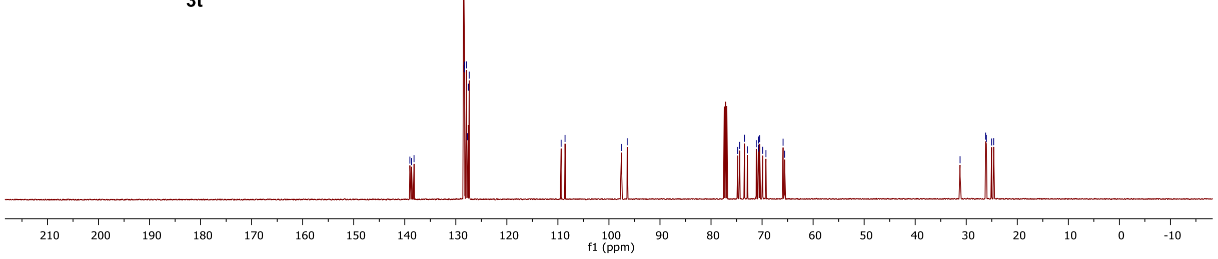

Supplementary figure S232: ^{13}C spectra for **3t**

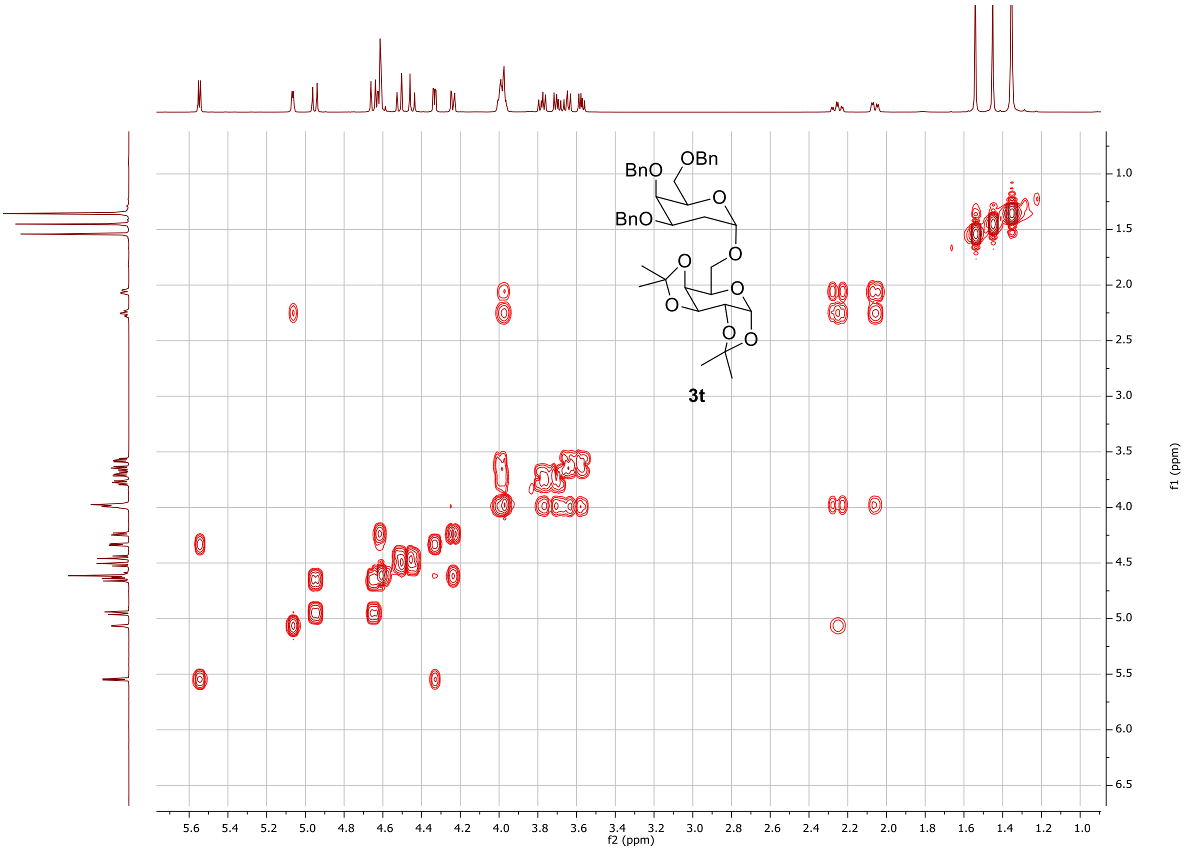

Supplementary figure S233: COSY spectra for **3t**

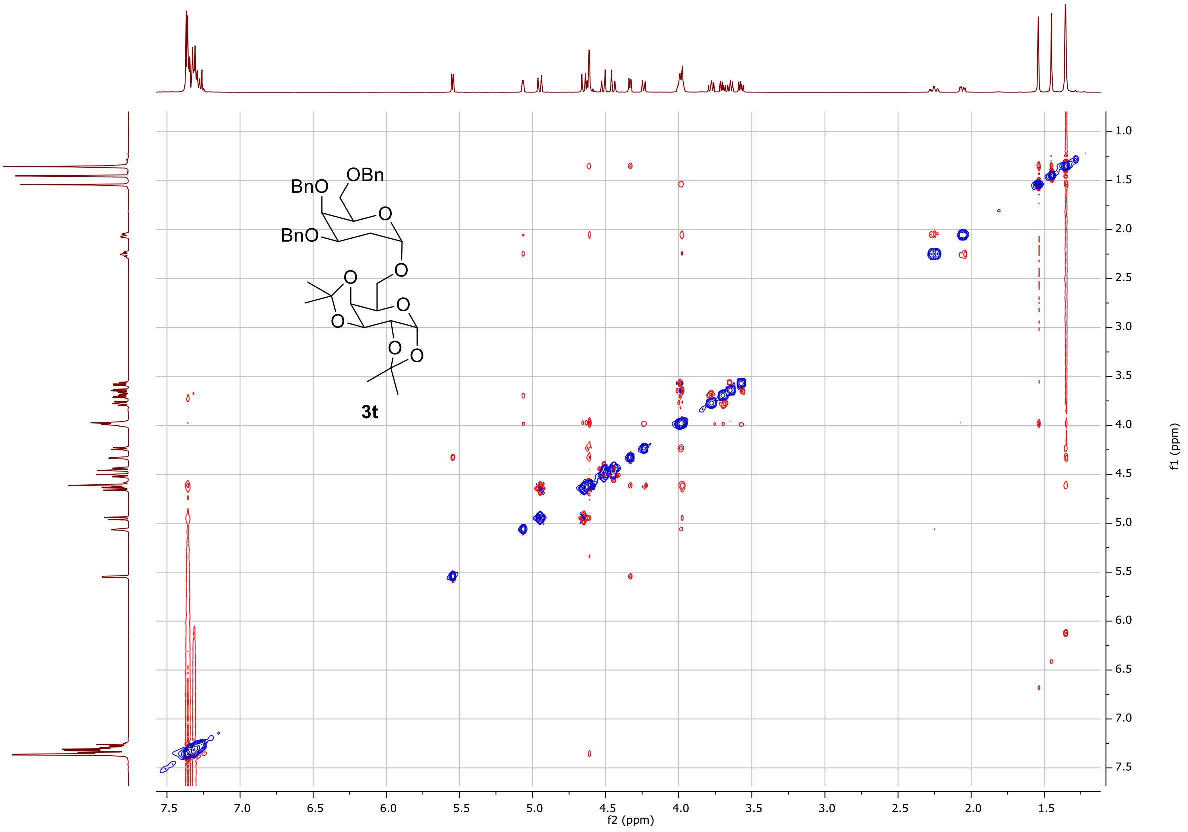

Supplementary figure S234: NOESY spectra for **3t**

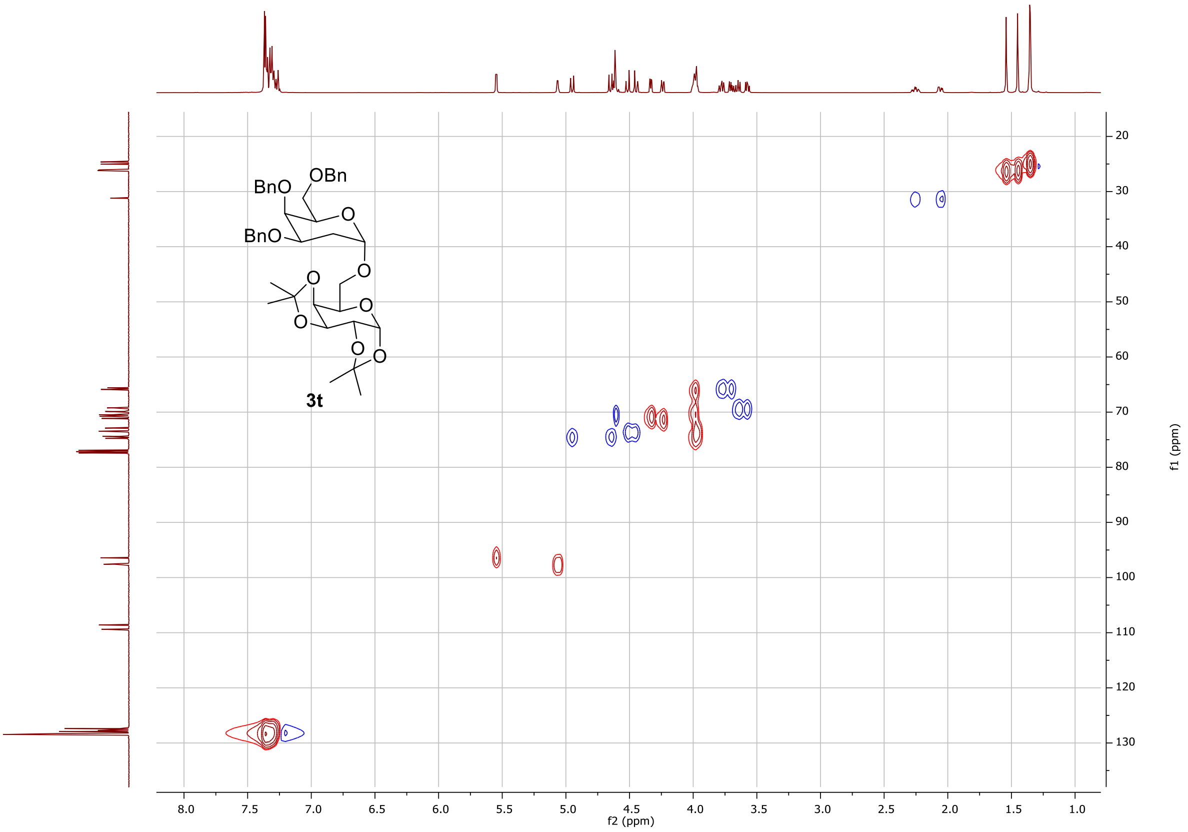

Supplementary figure S235: HSQC spectra for **3t**

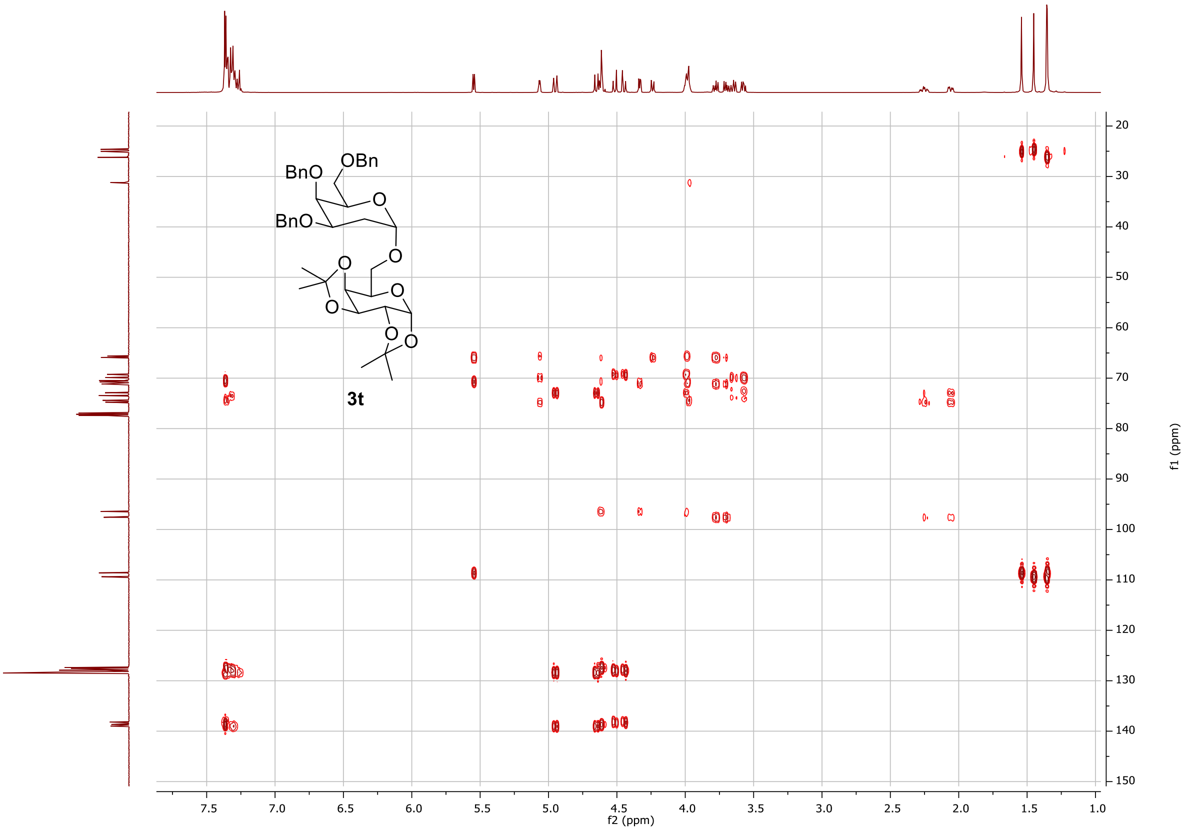

Supplementary figure S236: HMBC spectra for **3t**

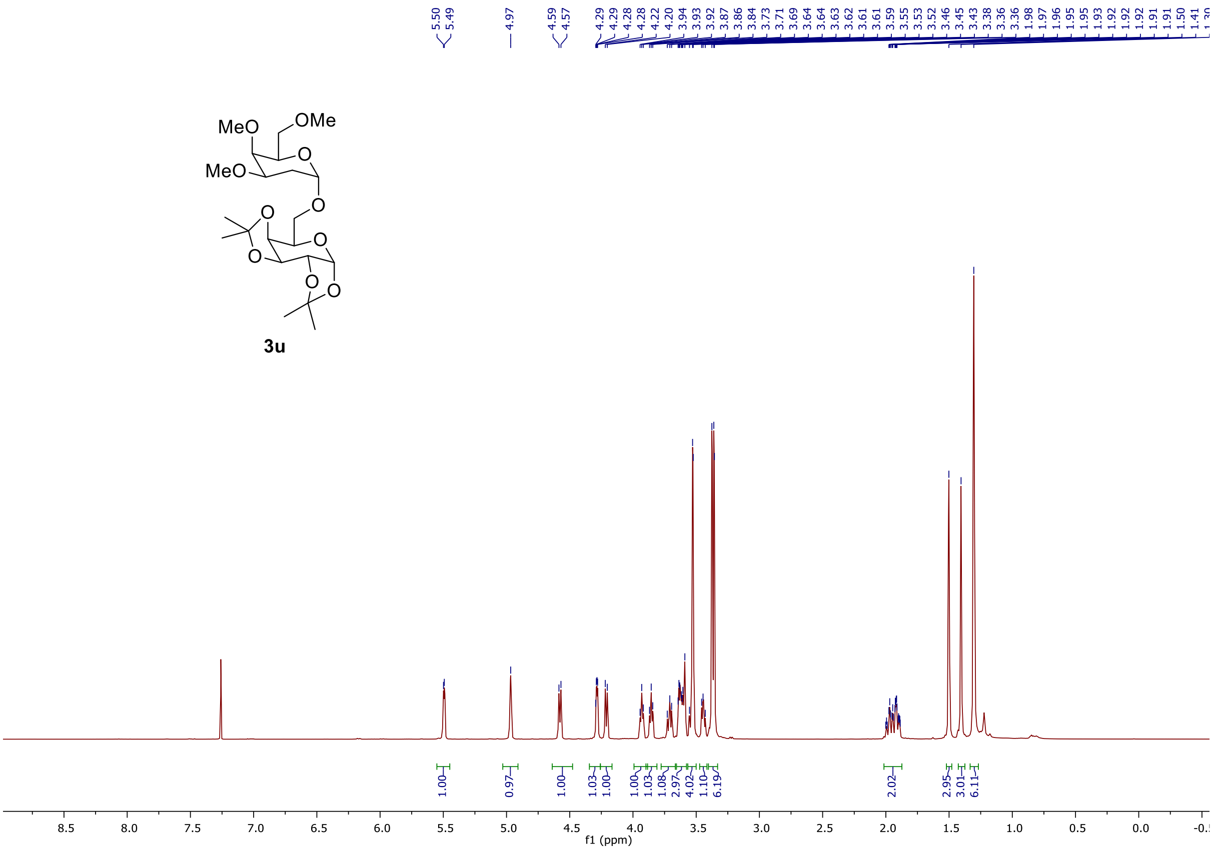

Supplementary figure S237: ^{1}H spectra for **3u**

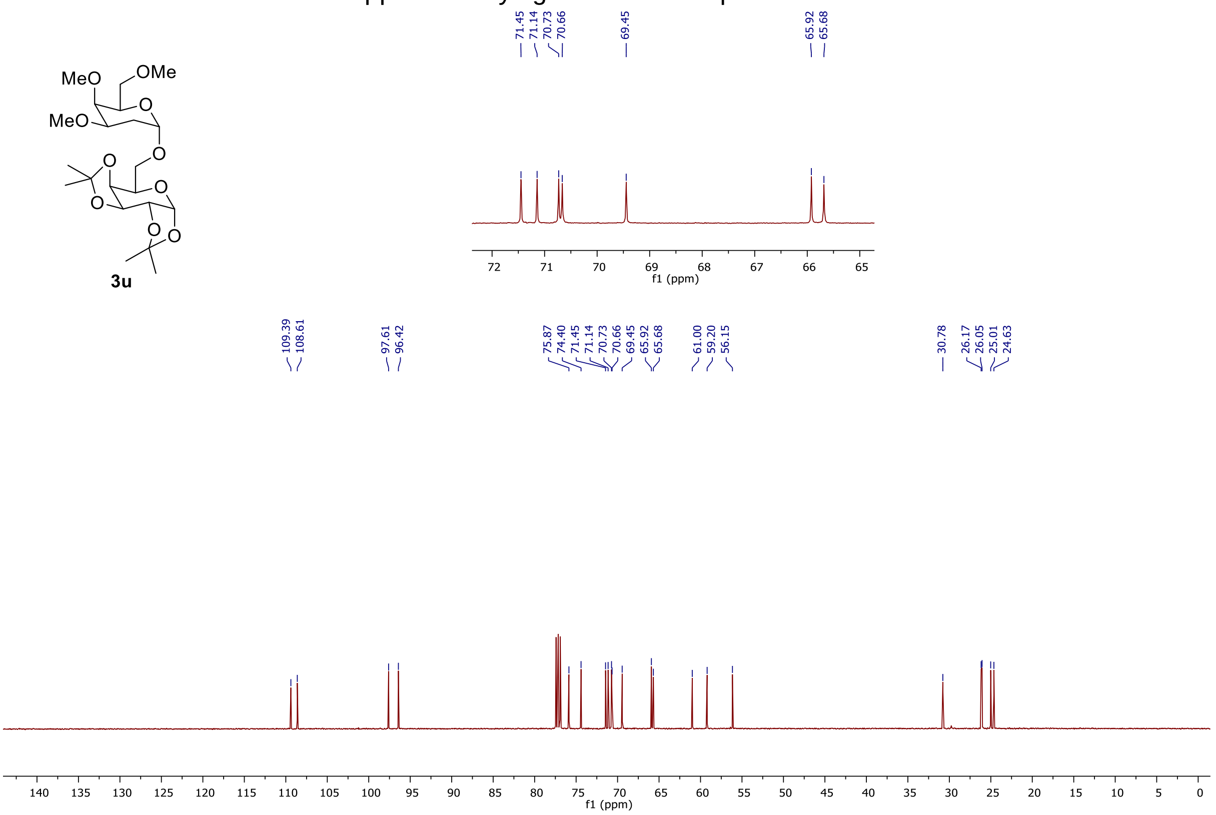

Supplementary figure S238: ^{13}C spectra for **3u**

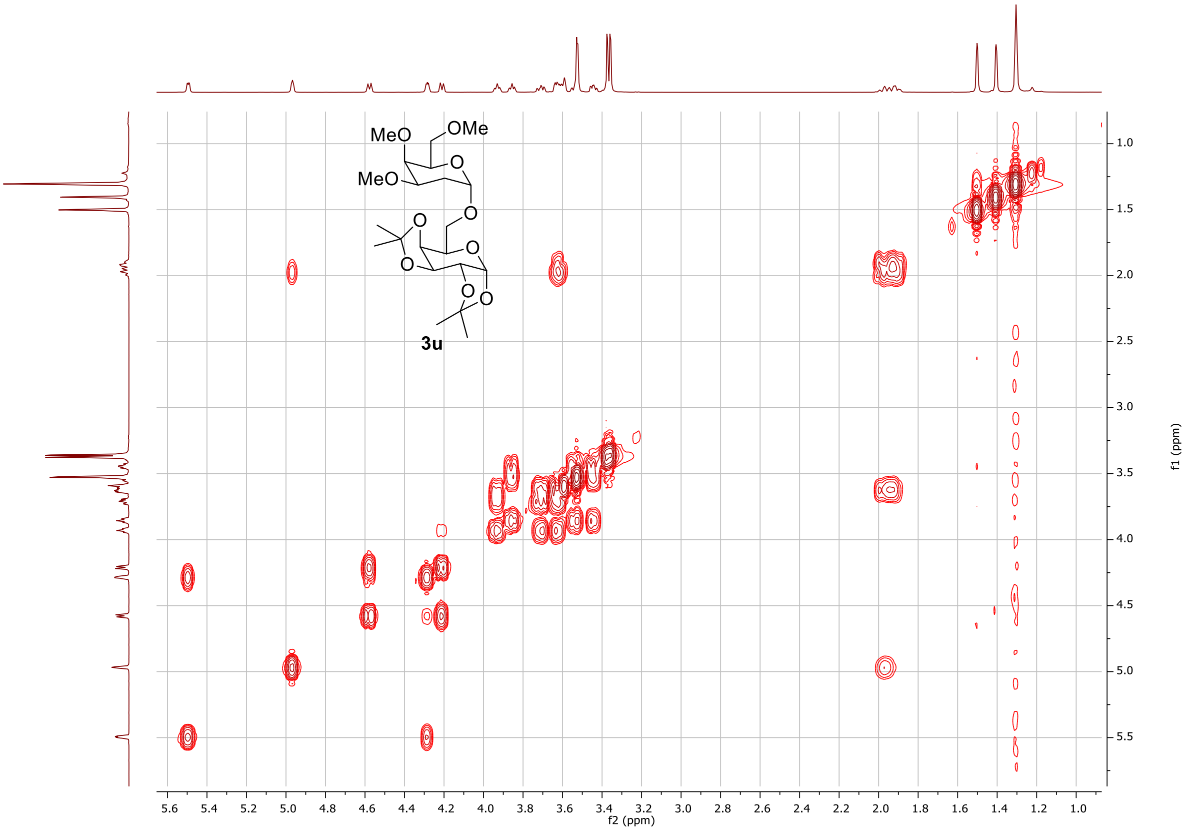

Supplementary figure S239: COSY spectra for **3u**

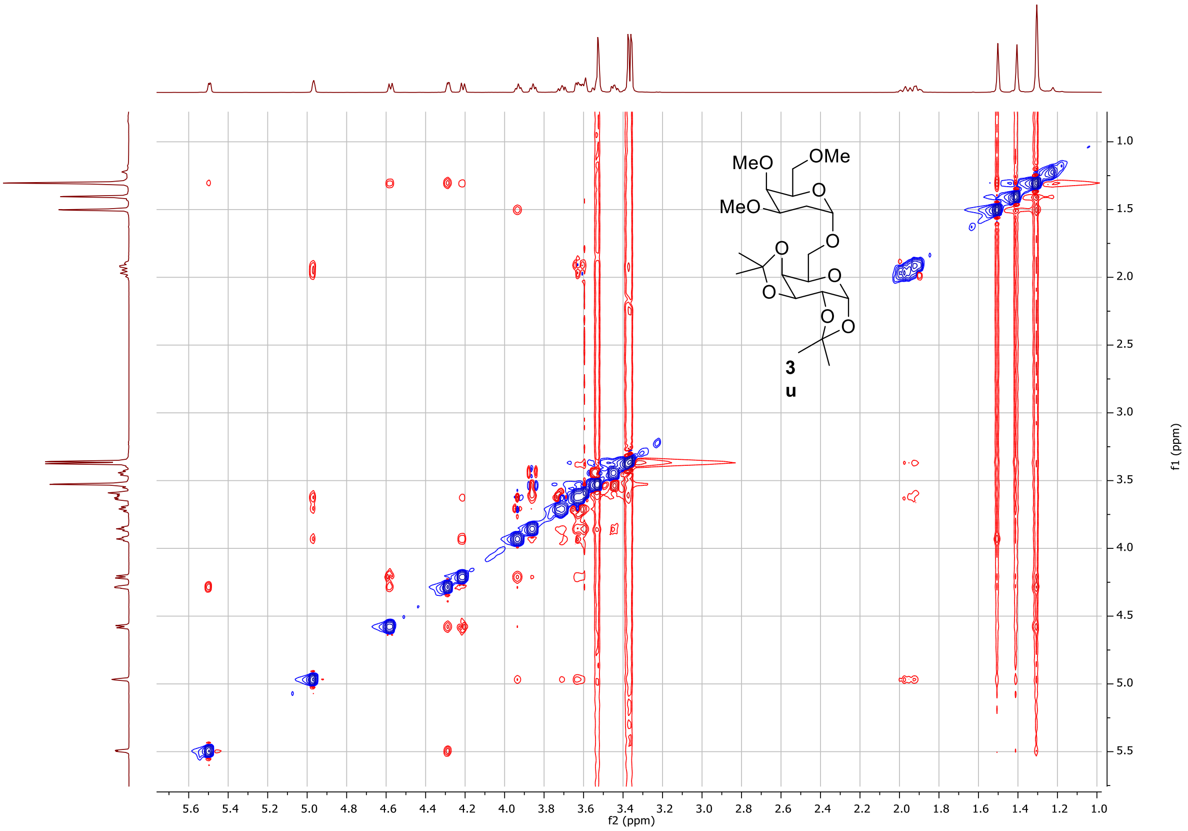

Supplementary figure S240: NOESY spectra for **3u**

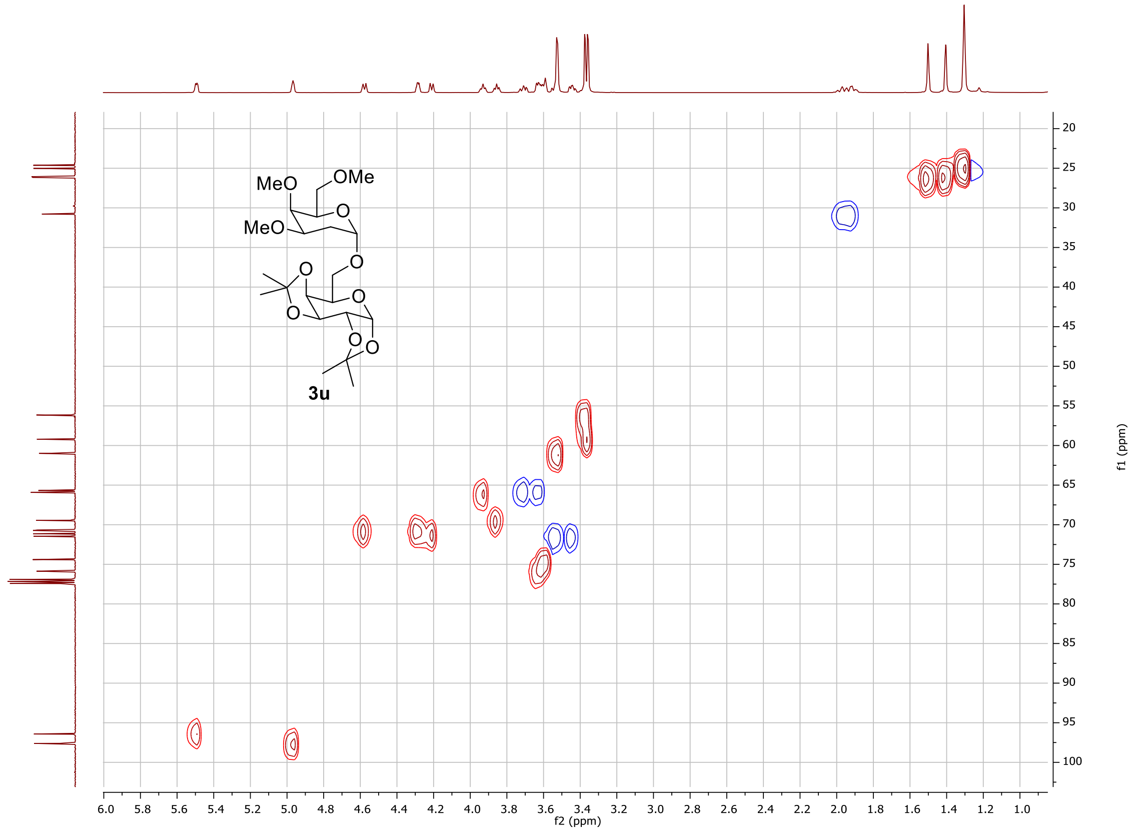

Supplementary figure S241: HSQC spectra for **3u**

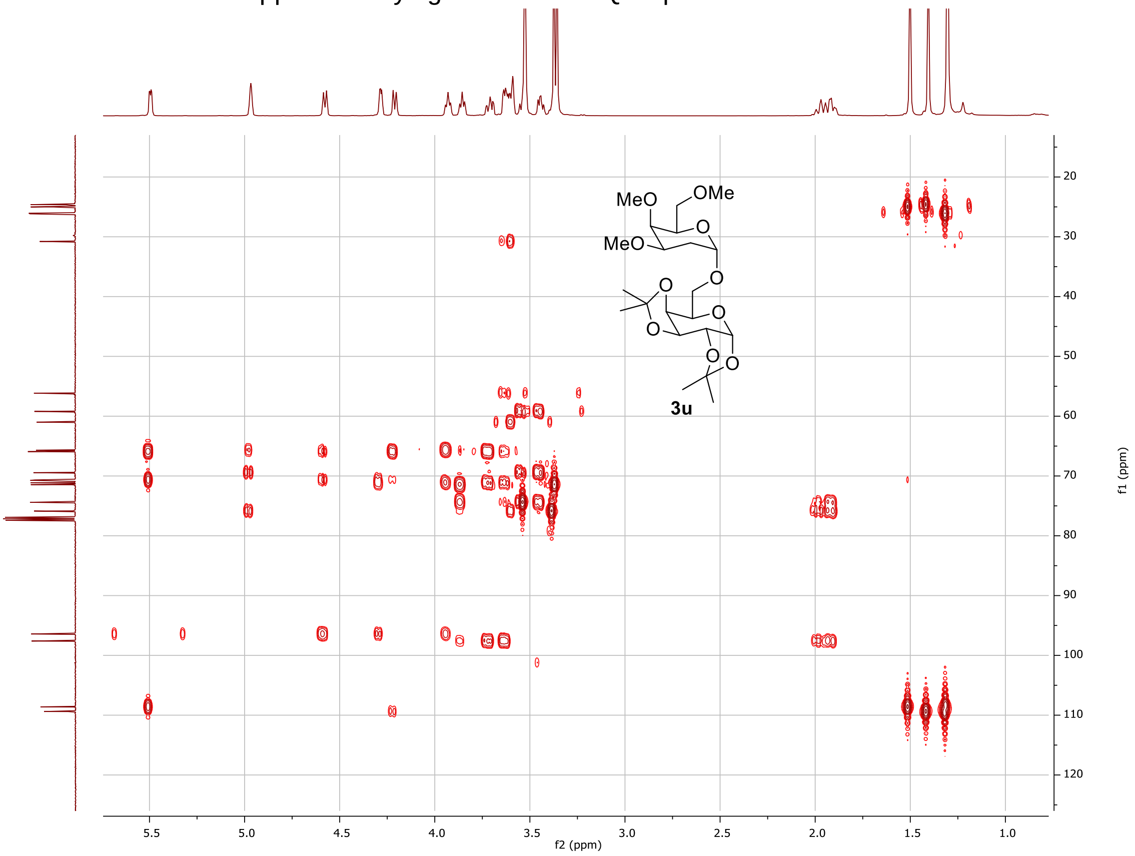

Supplementary figure S242: HMBC spectra for **3u**

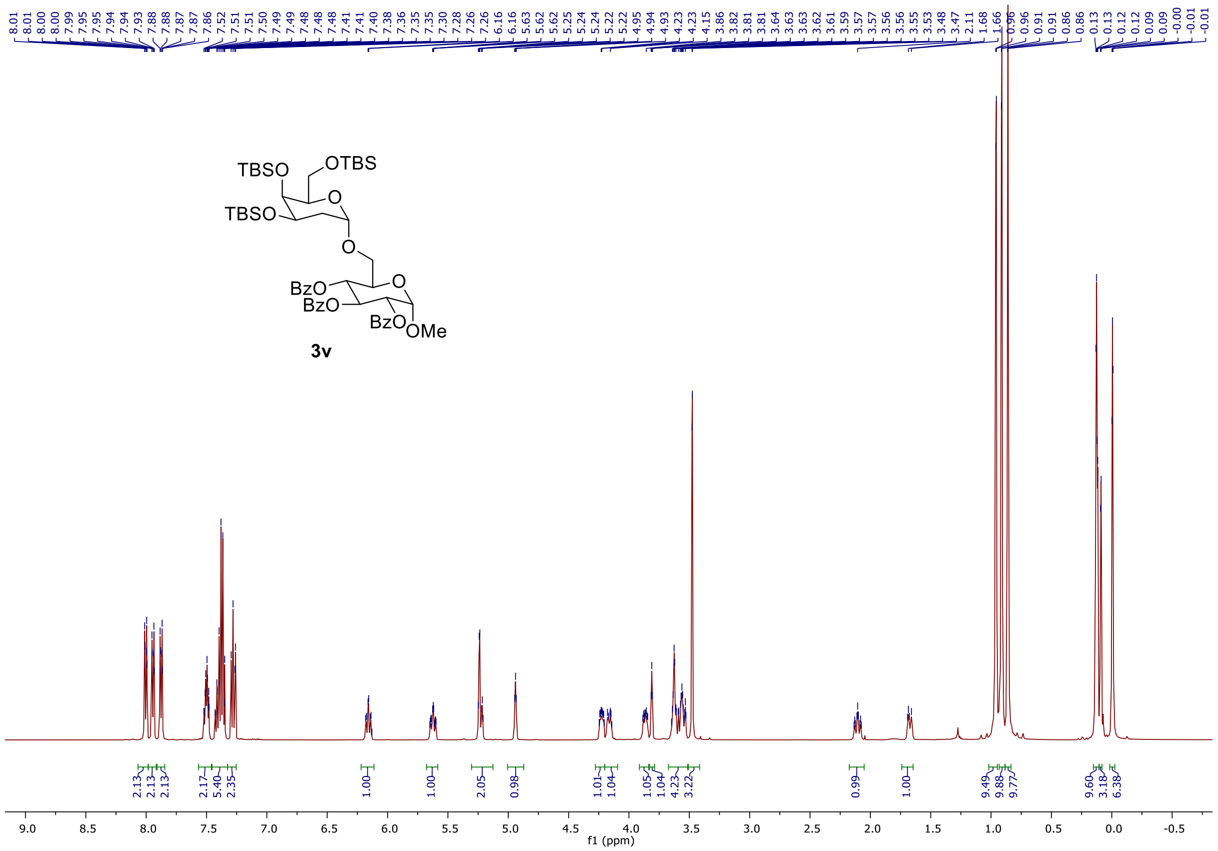

Supplementary figure S243: ^{1}H spectra for **3v**

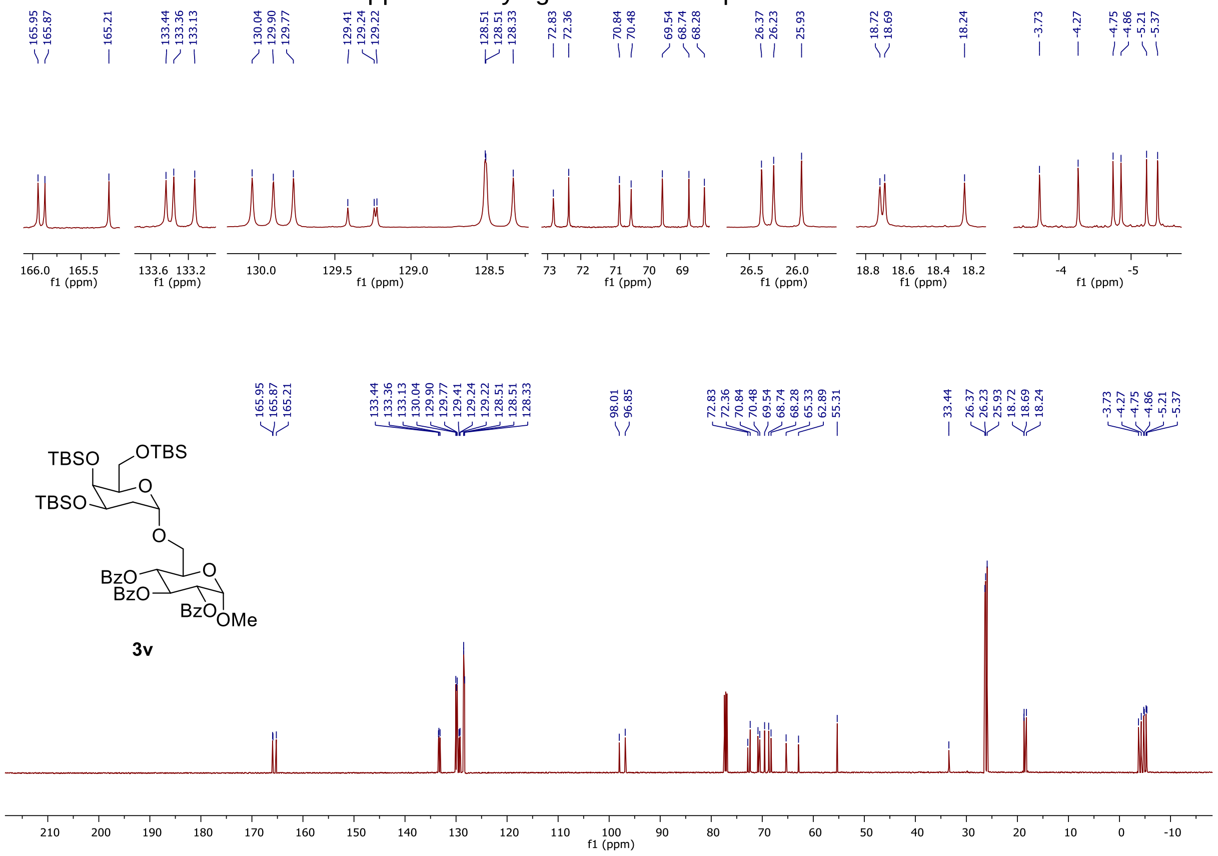

Supplementary figure S244: ^{13}C spectra for **3v**

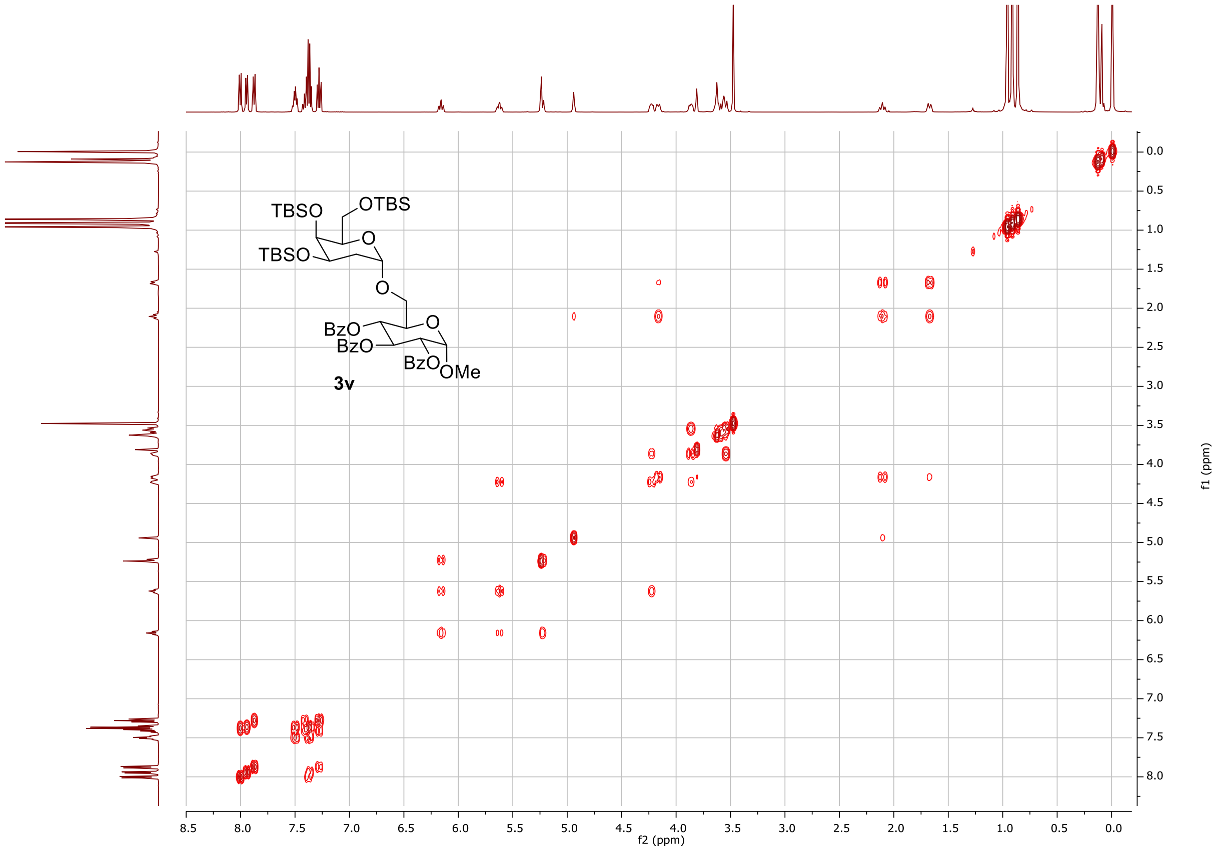

Supplementary figure S245: COSY spectra for **3v**

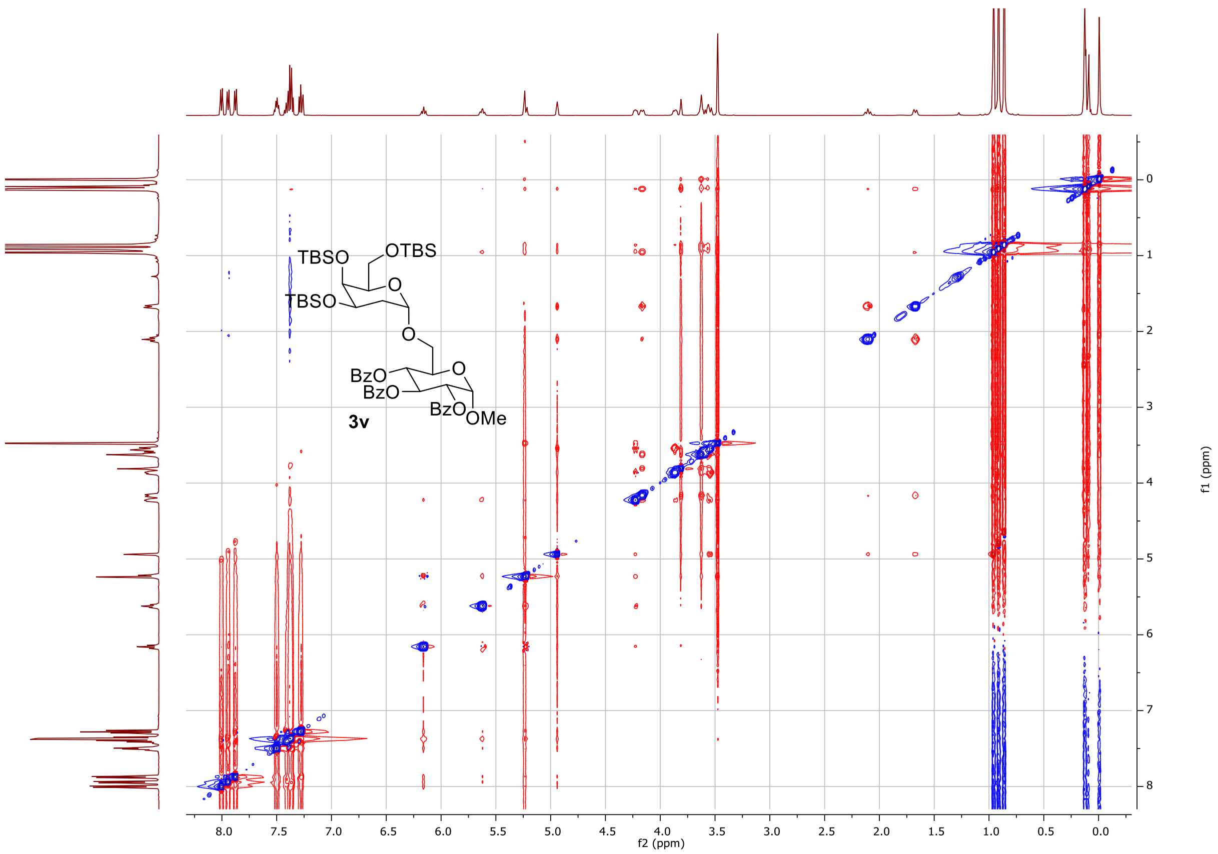

Supplementary figure S246: NOESY spectra for **3v**

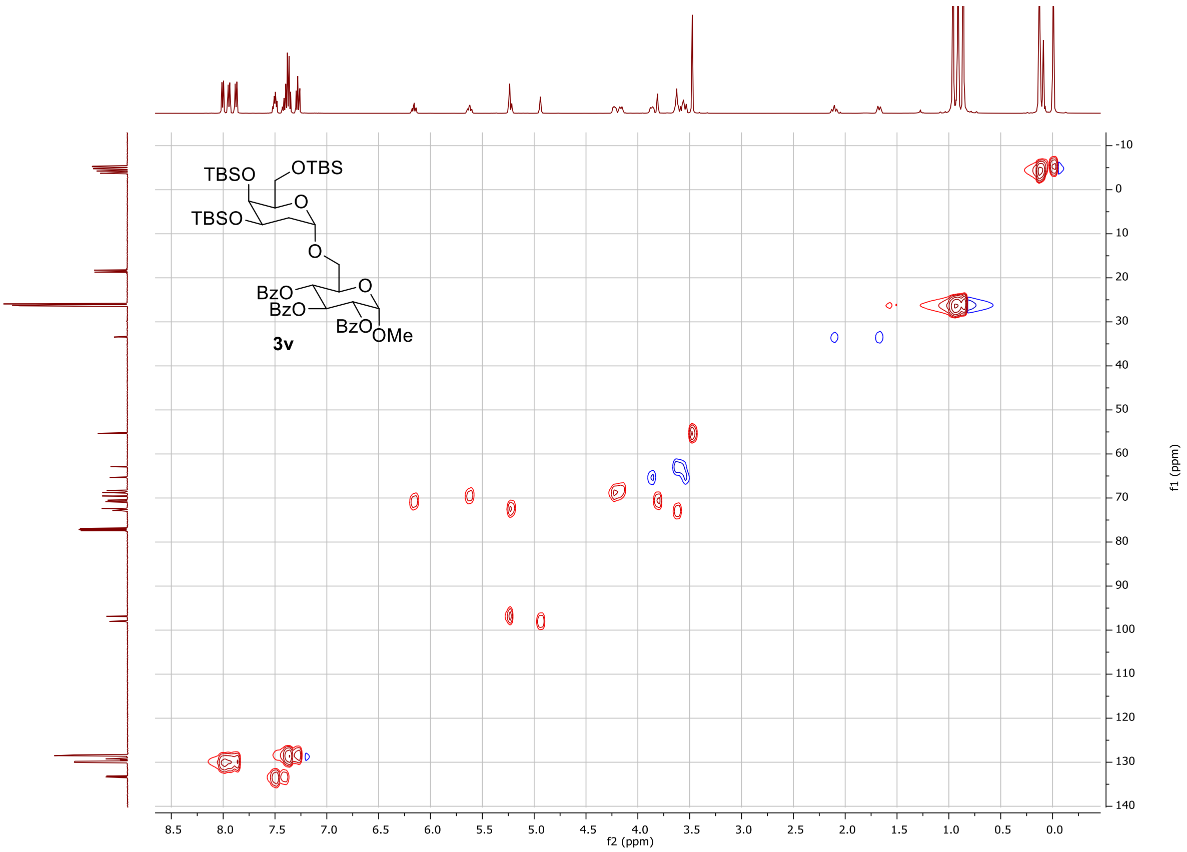

Supplementary figure S247: HSQC spectra for **3v**

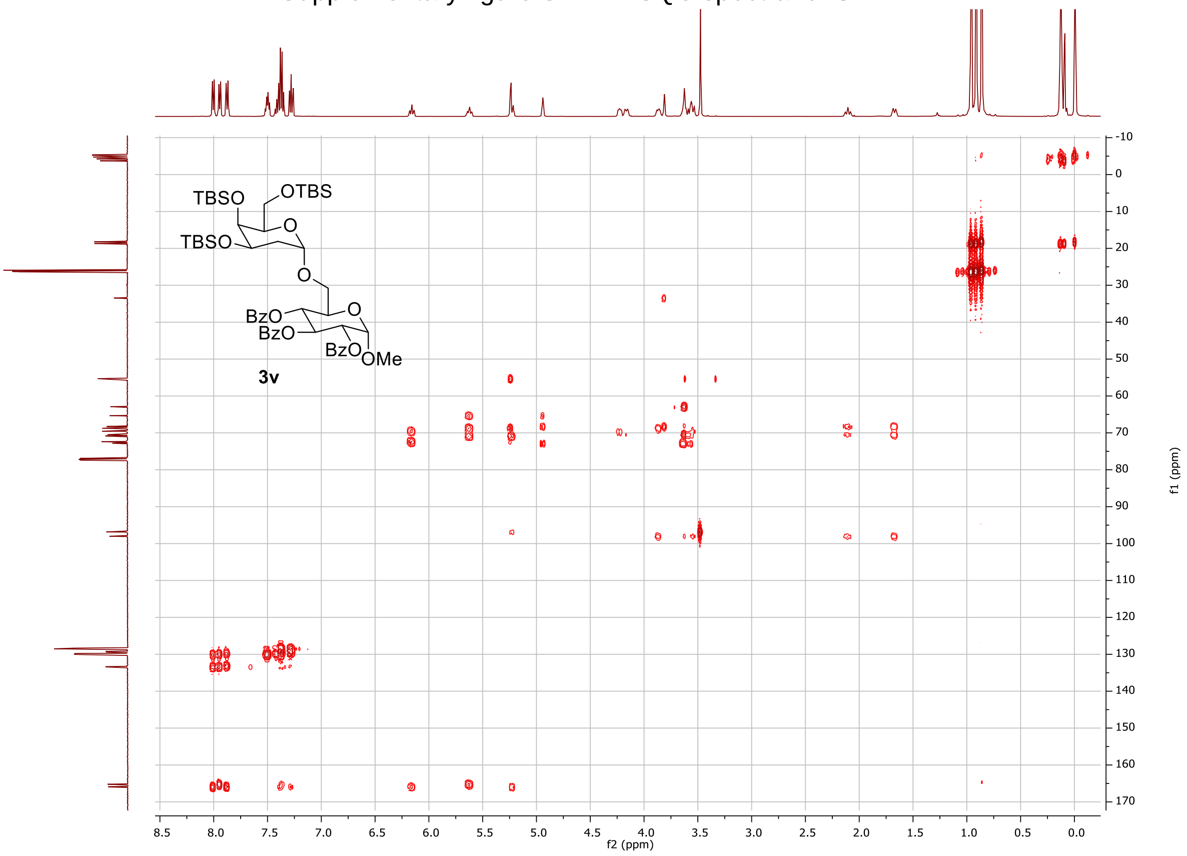

Supplementary figure S248: HMBC spectra for **3v**

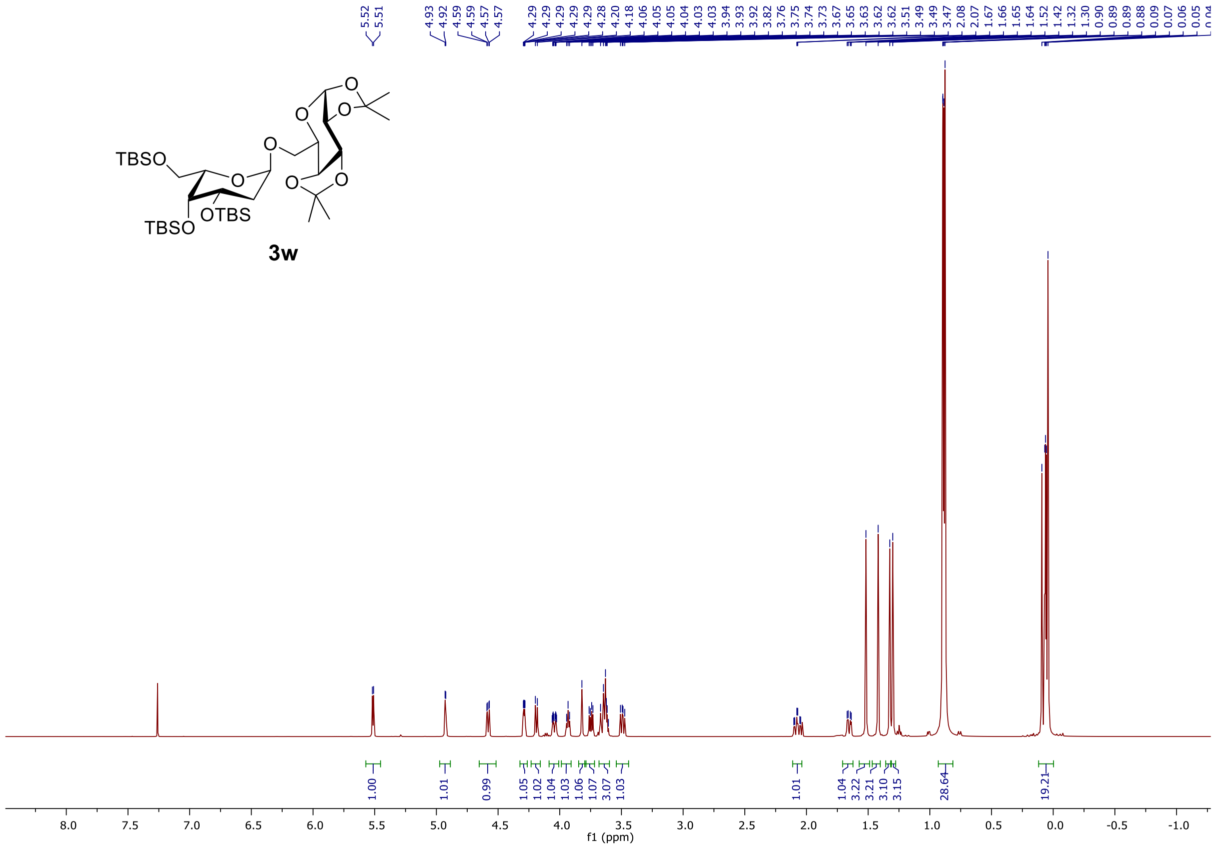

Supplementary figure S249: ^{1}H spectra for **3w**

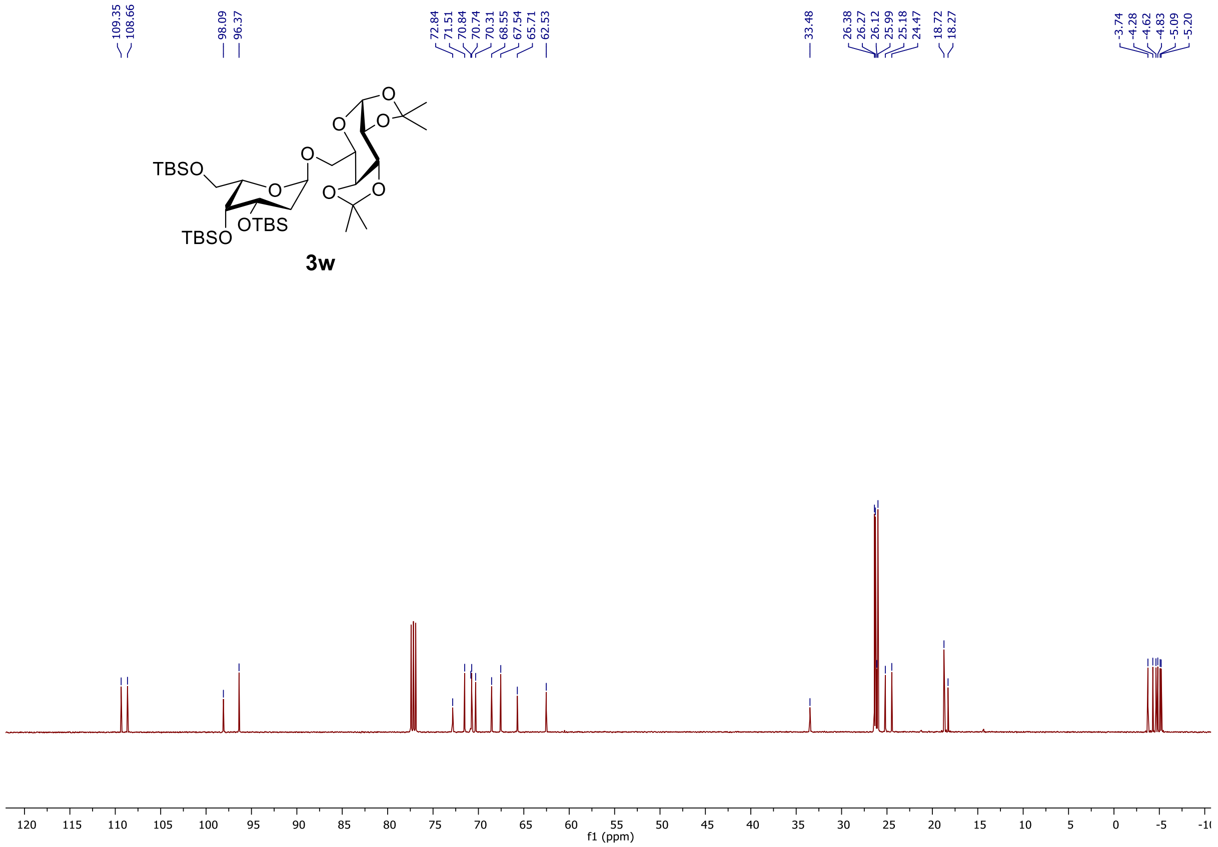

Supplementary figure S250: ^{13}C spectra for **3w**

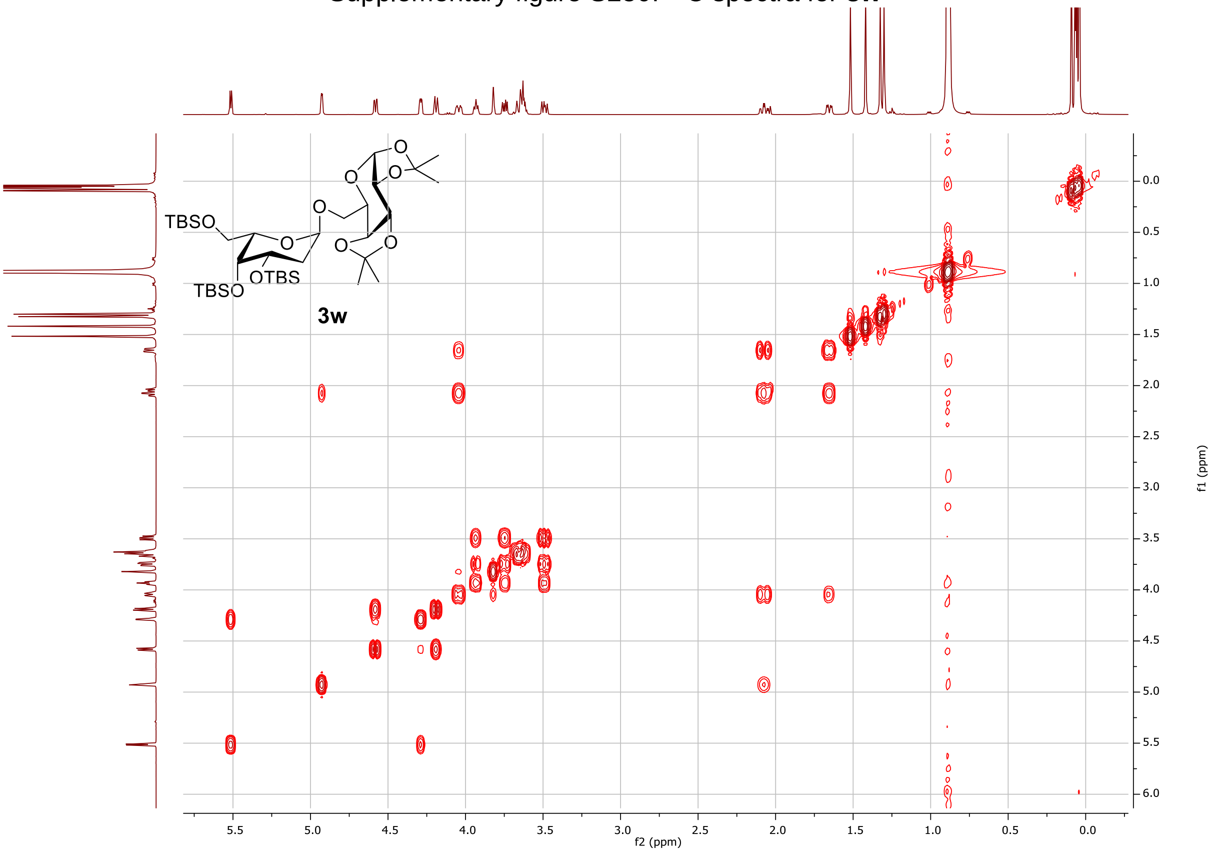

Supplementary figure S251: COSY spectra for **3w**

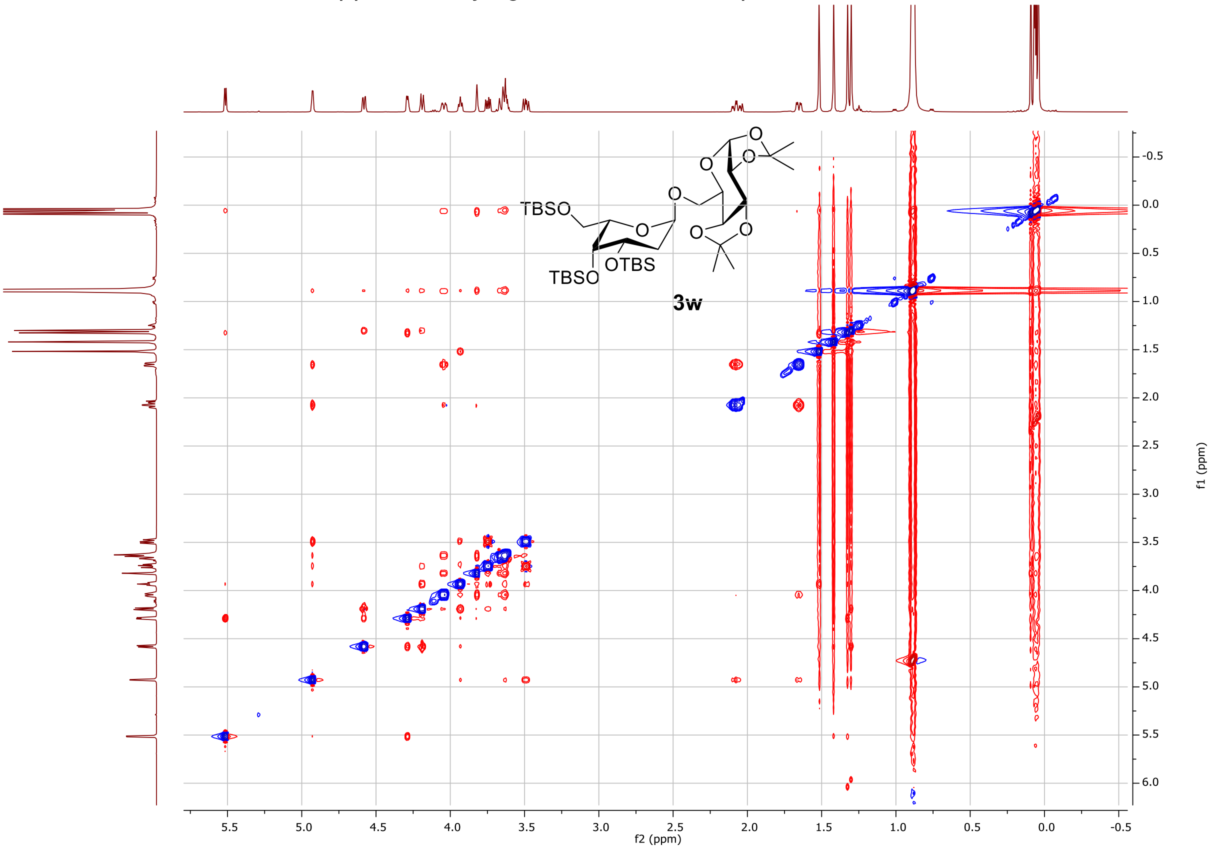

Supplementary figure S252: NOESY spectra for **3w**

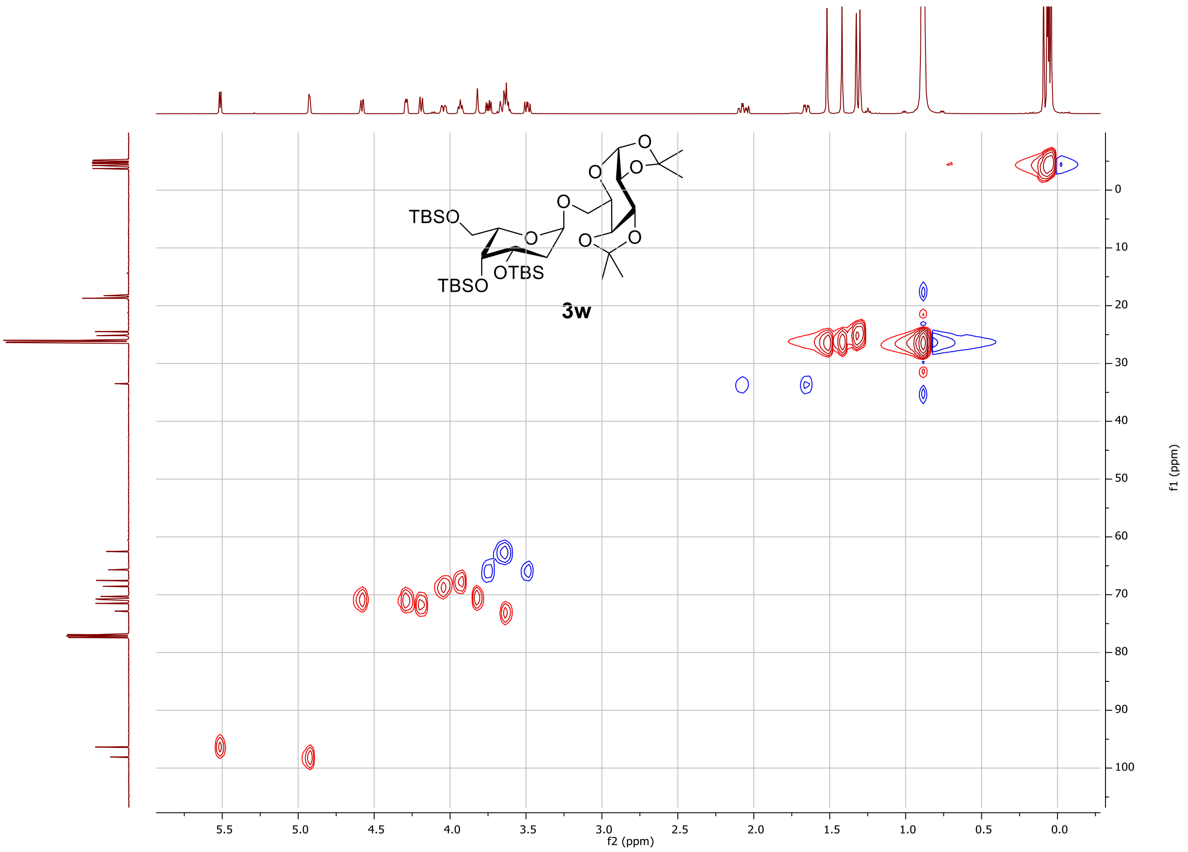

Supplementary figure S253: HSQC spectra for **3w**

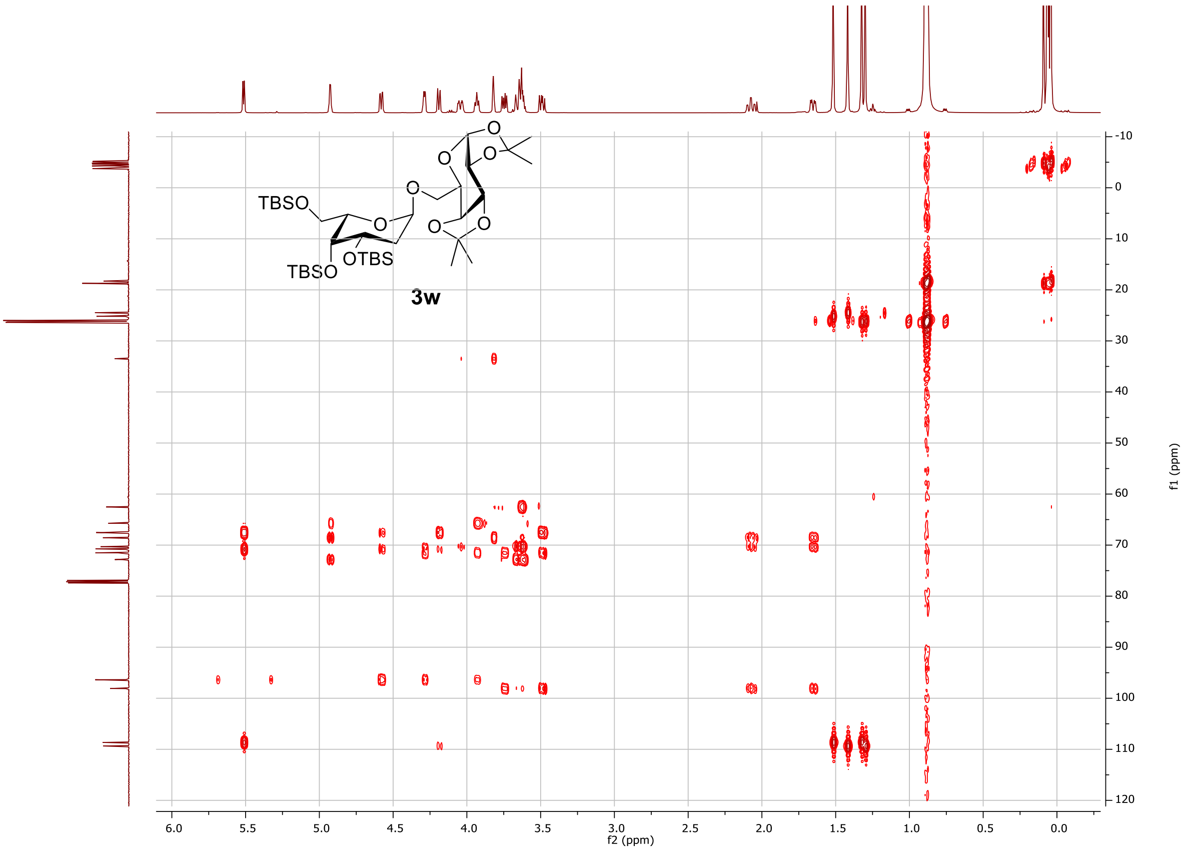

Supplementary figure S254: HMBC spectra for **3w**

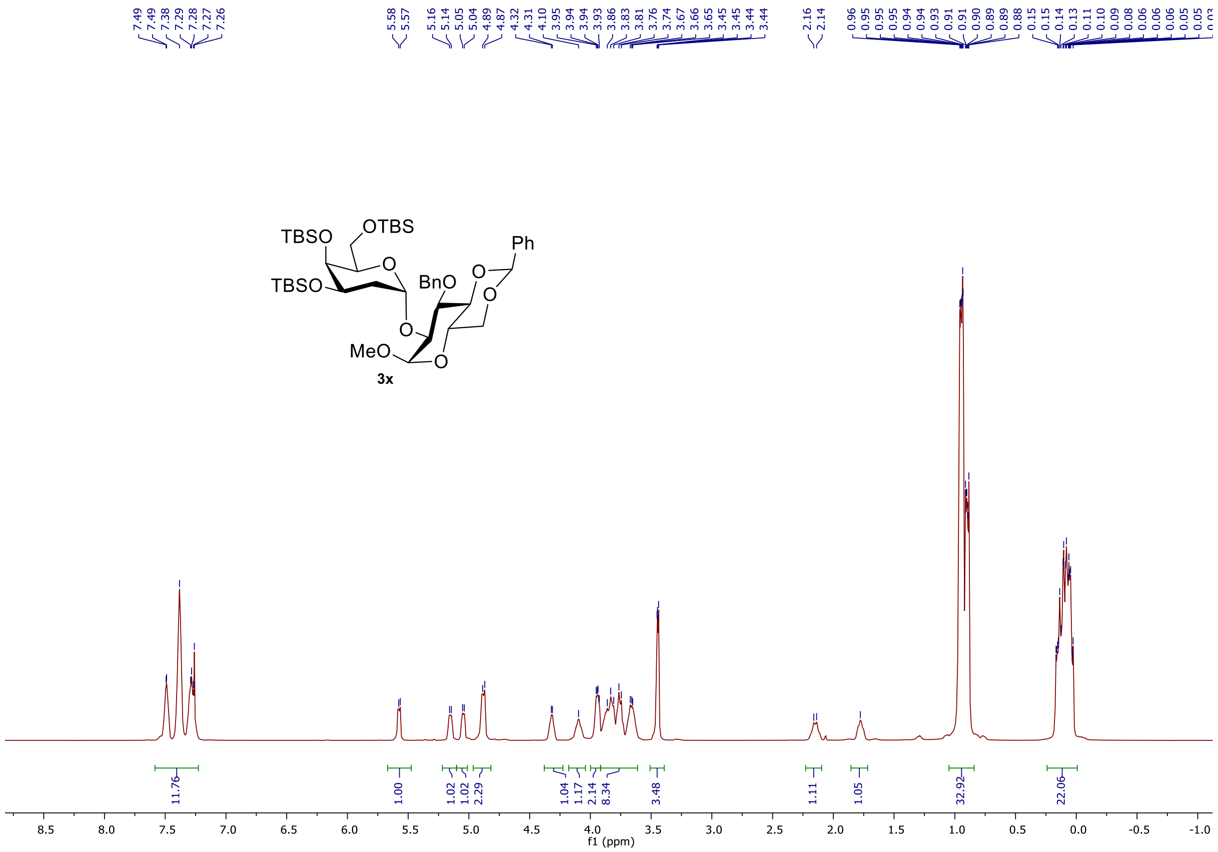

Supplementary figure S255: ^{1}H spectra for **3x**

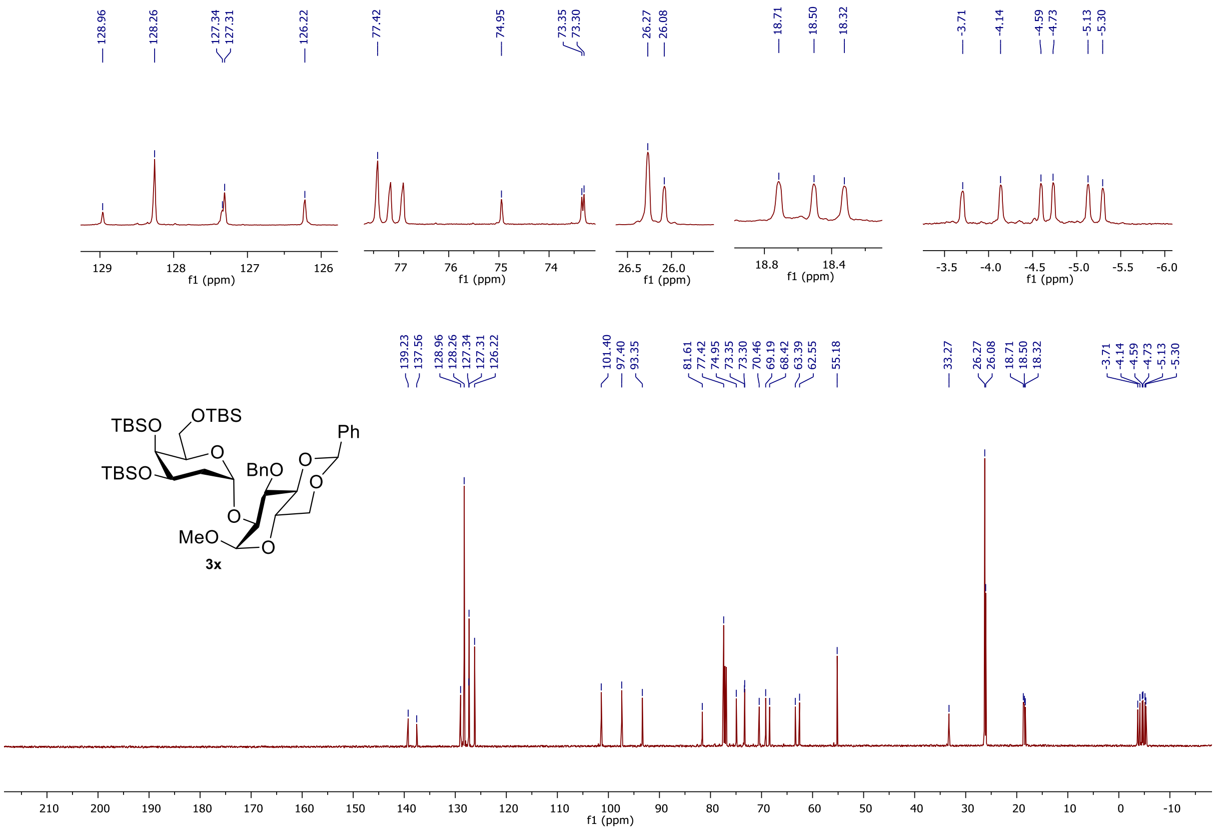

Supplementary figure S256: ^{13}C spectra for **3x**

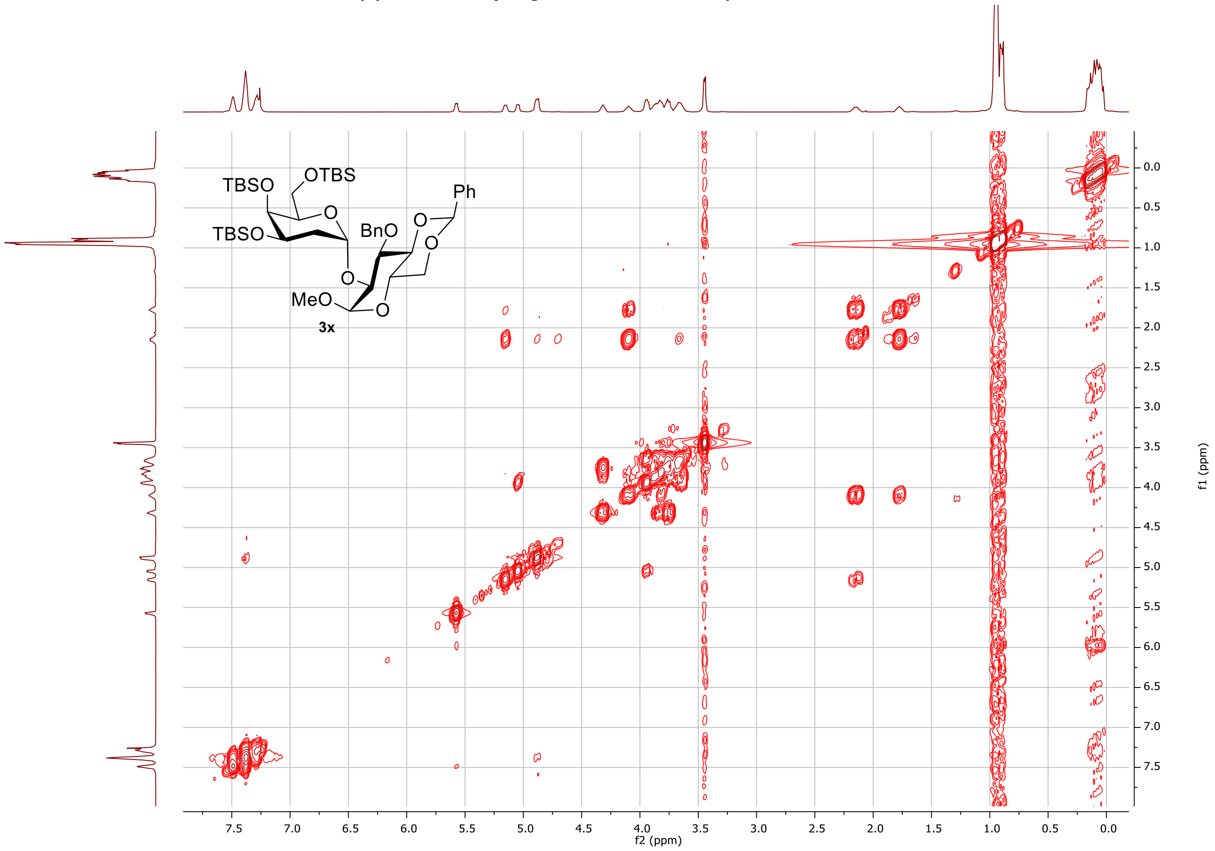

Supplementary figure S257: COSY spectra for **3x**

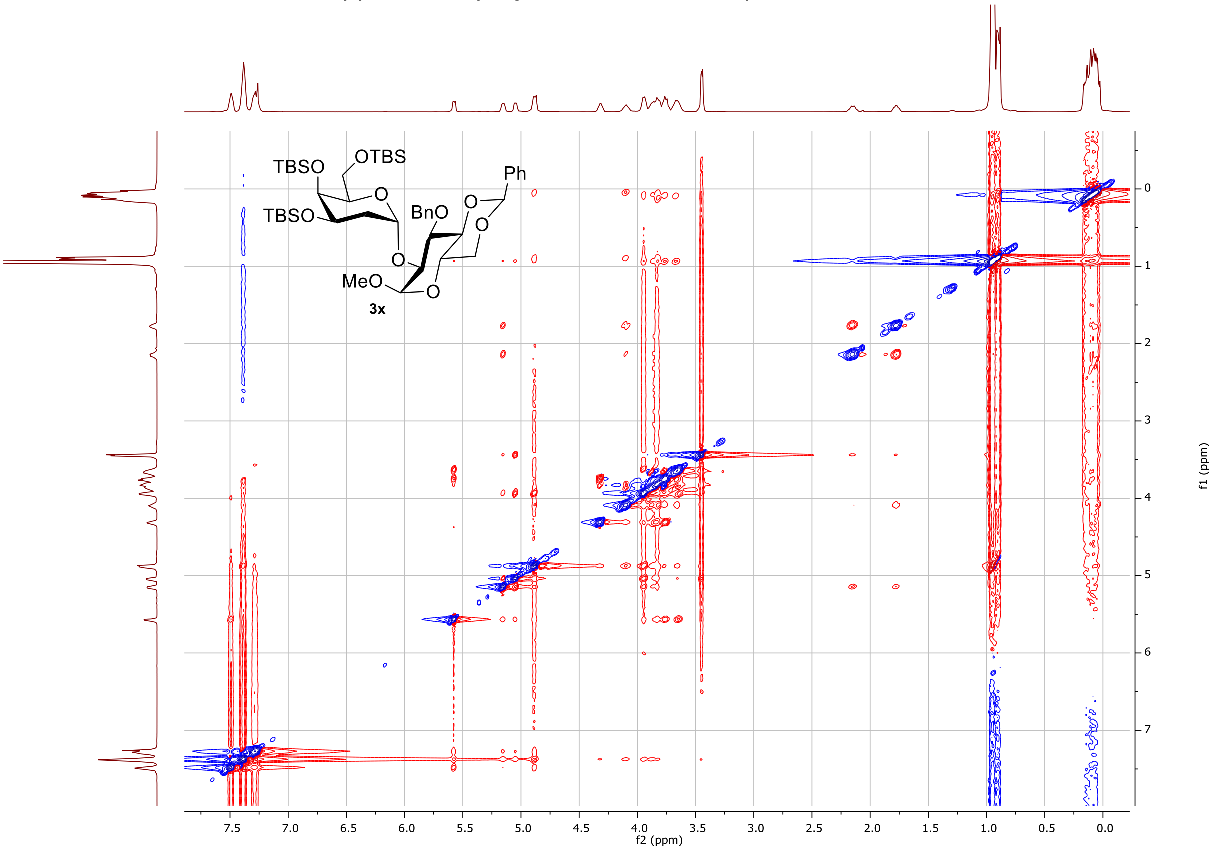

Supplementary figure S258: NOESY spectra for **3x**

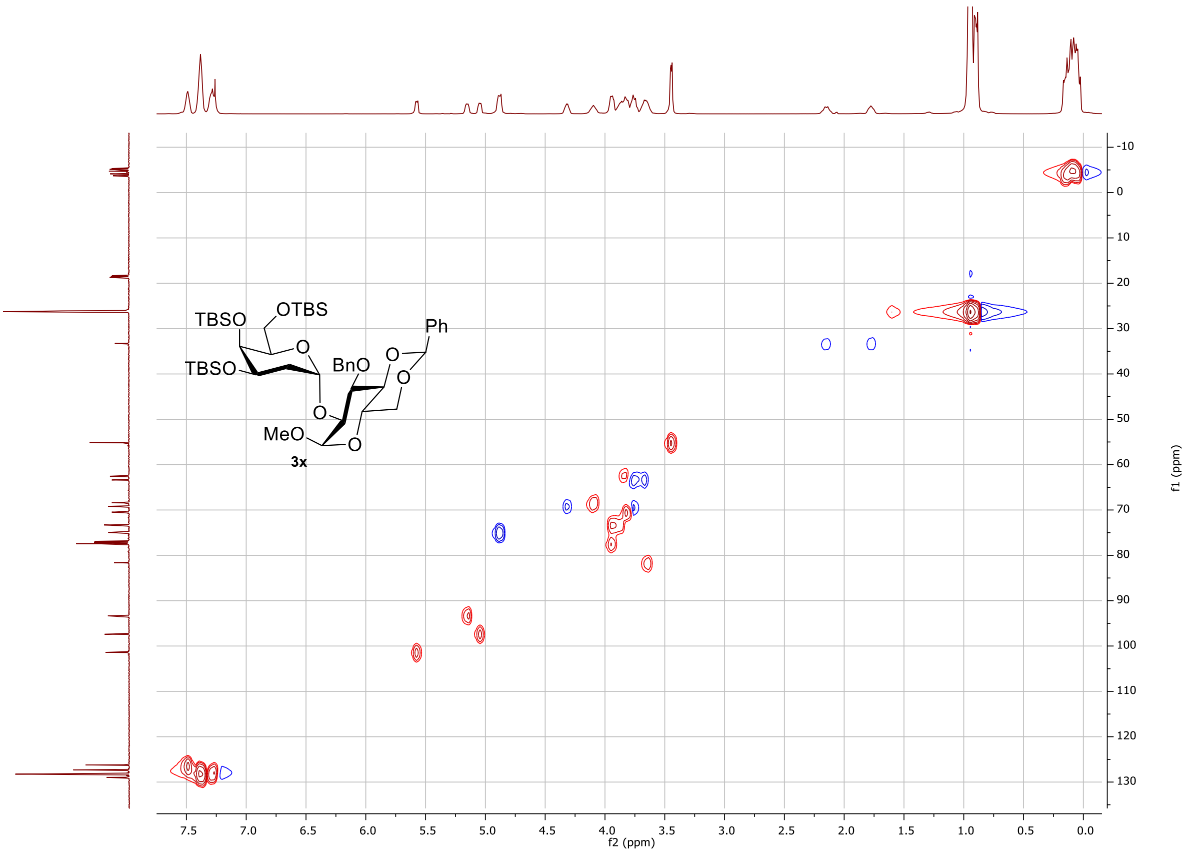

Supplementary figure S259: HSQC spectra for **3x**

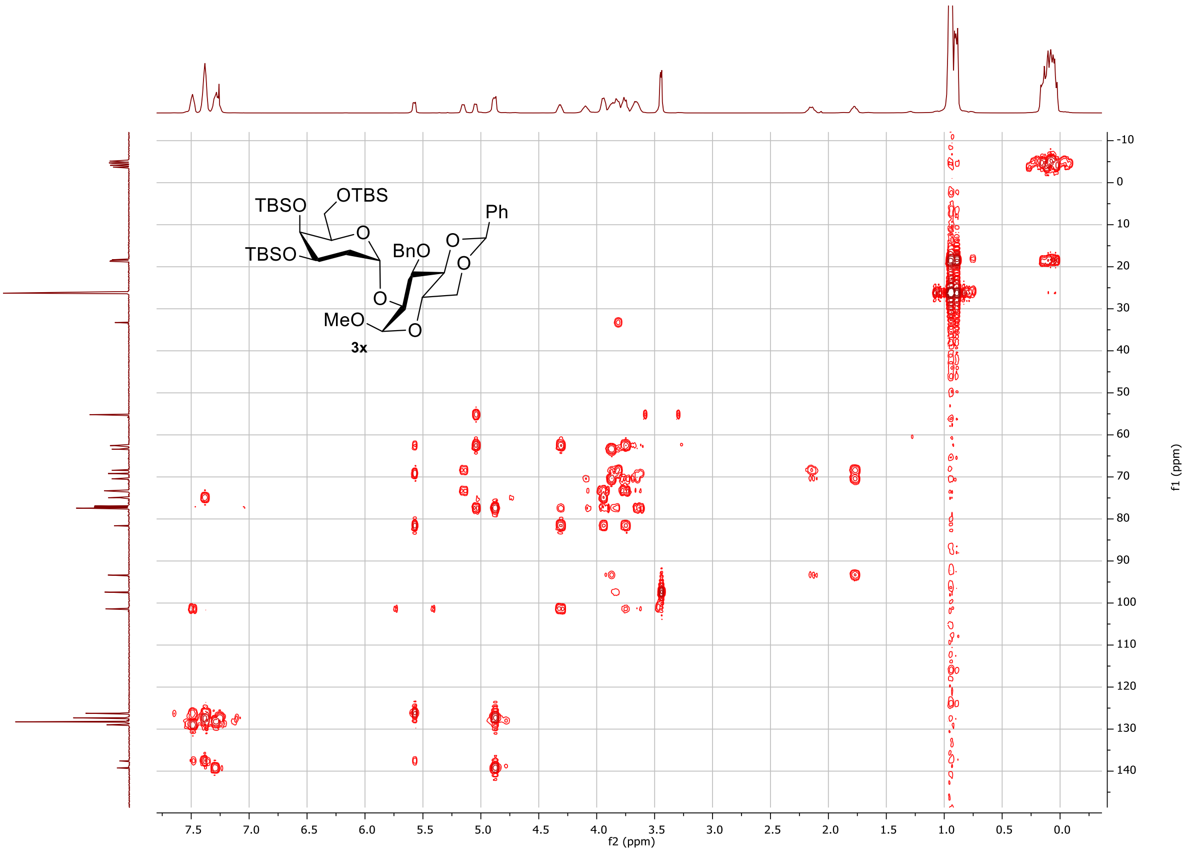

Supplementary figure S260: HMBC spectra for **3x**

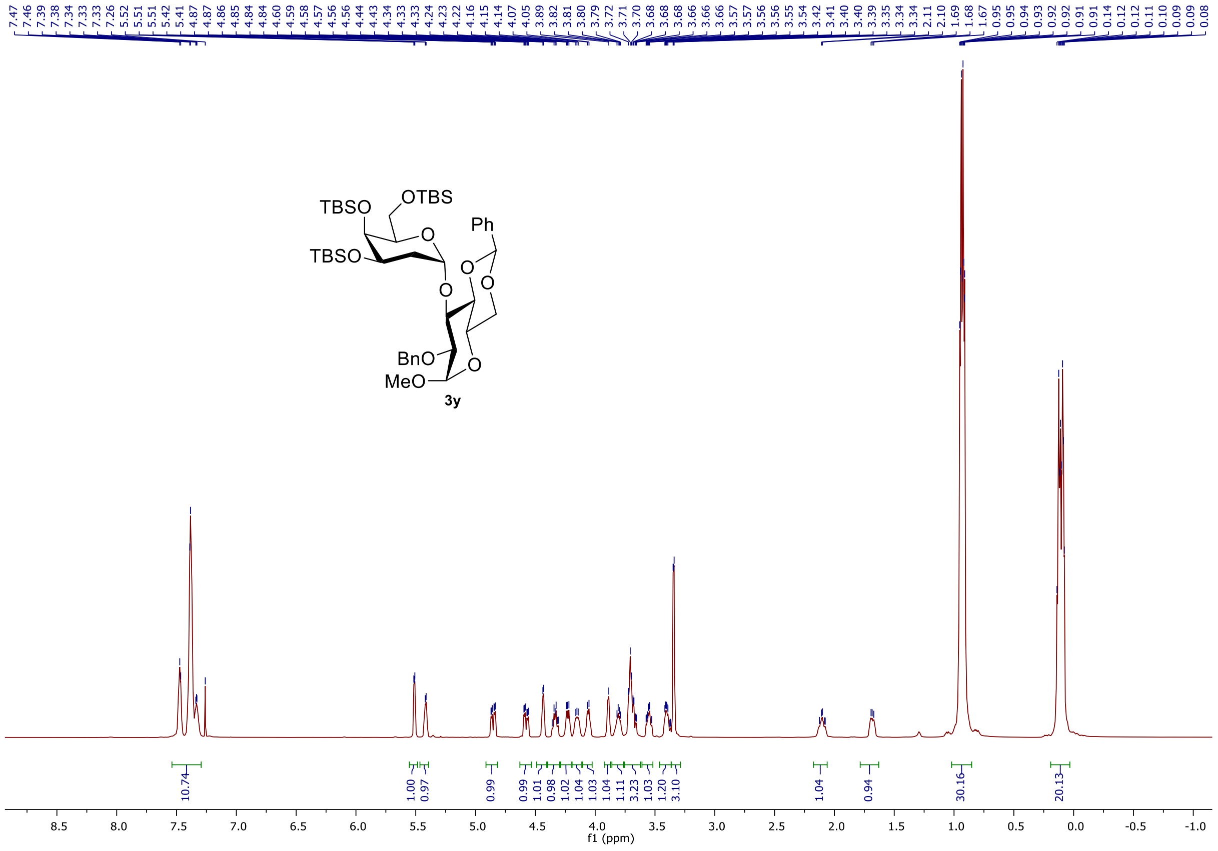

Supplementary figure S261: ^{1}H spectra for **3y**

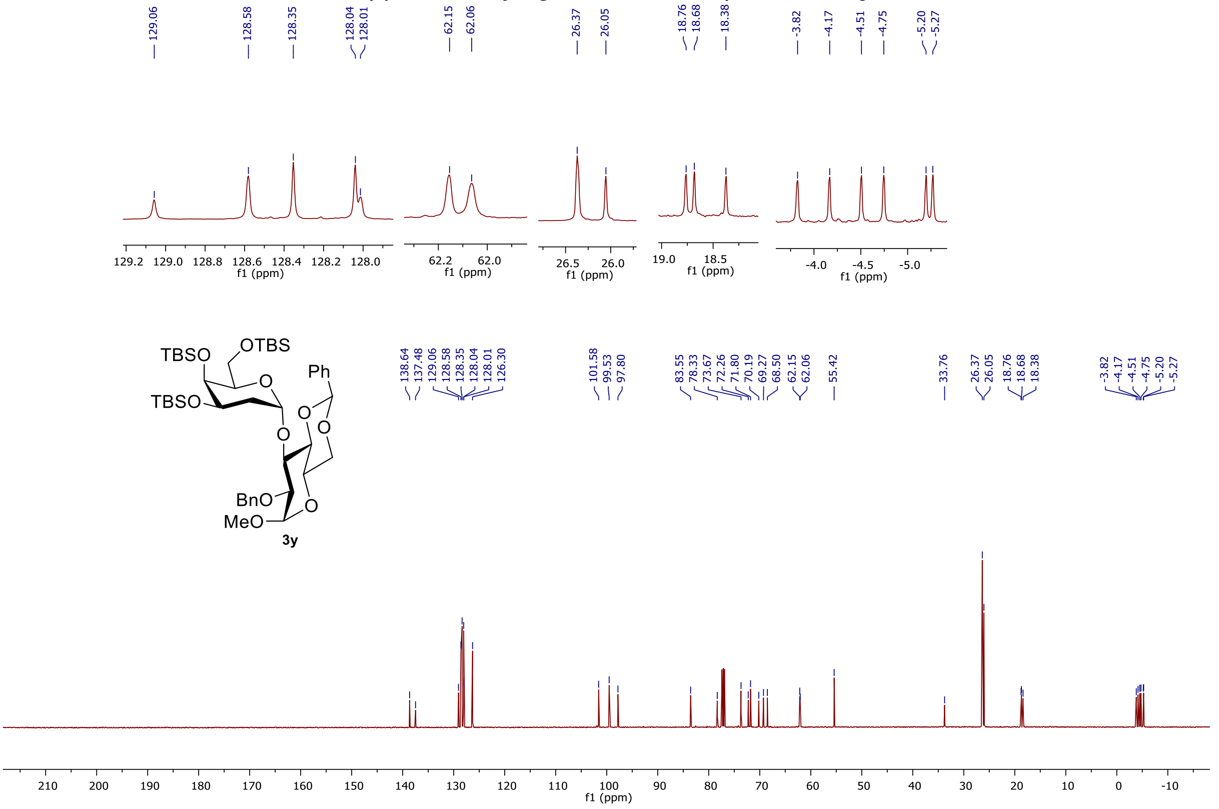

Supplementary figure S262: ^{13}C spectra for **3y**

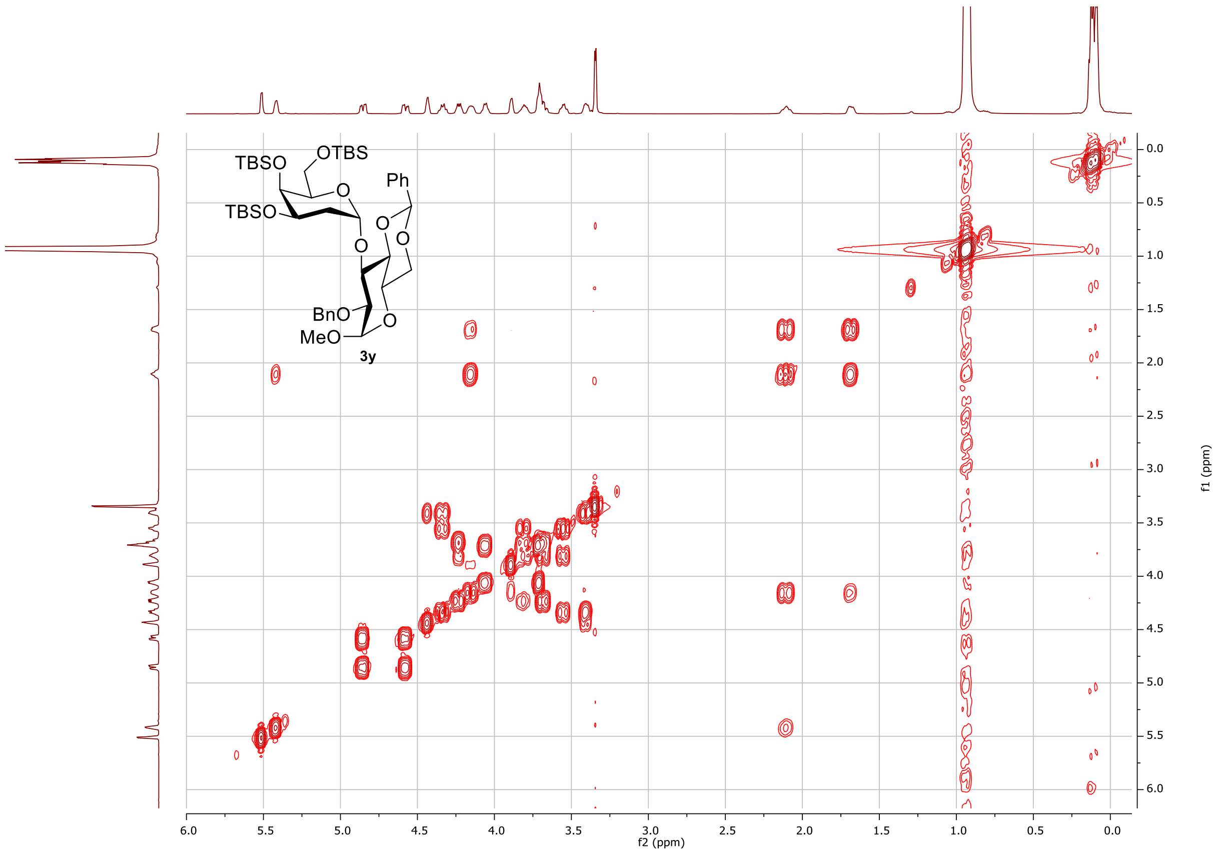

Supplementary figure S263: COSY spectra for **3y**

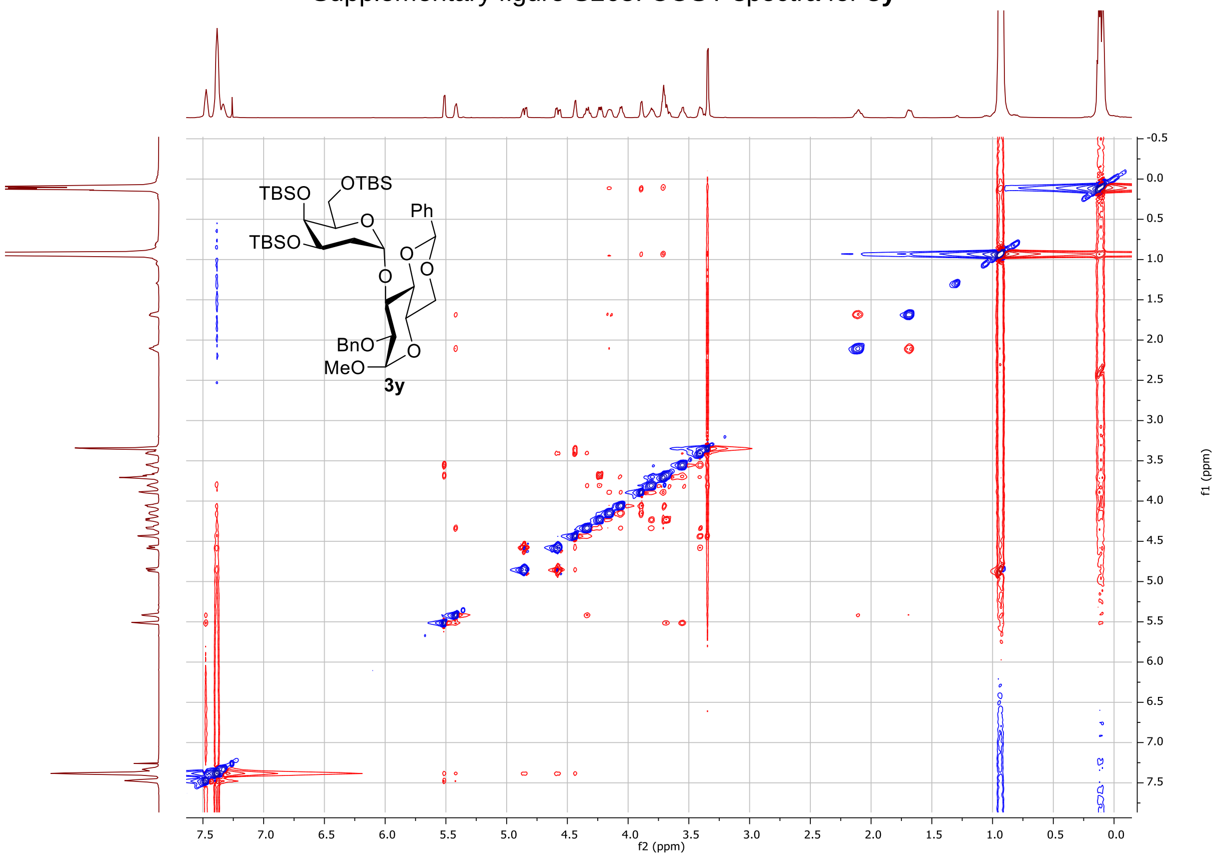

Supplementary figure S264: NOESY spectra for **3y**

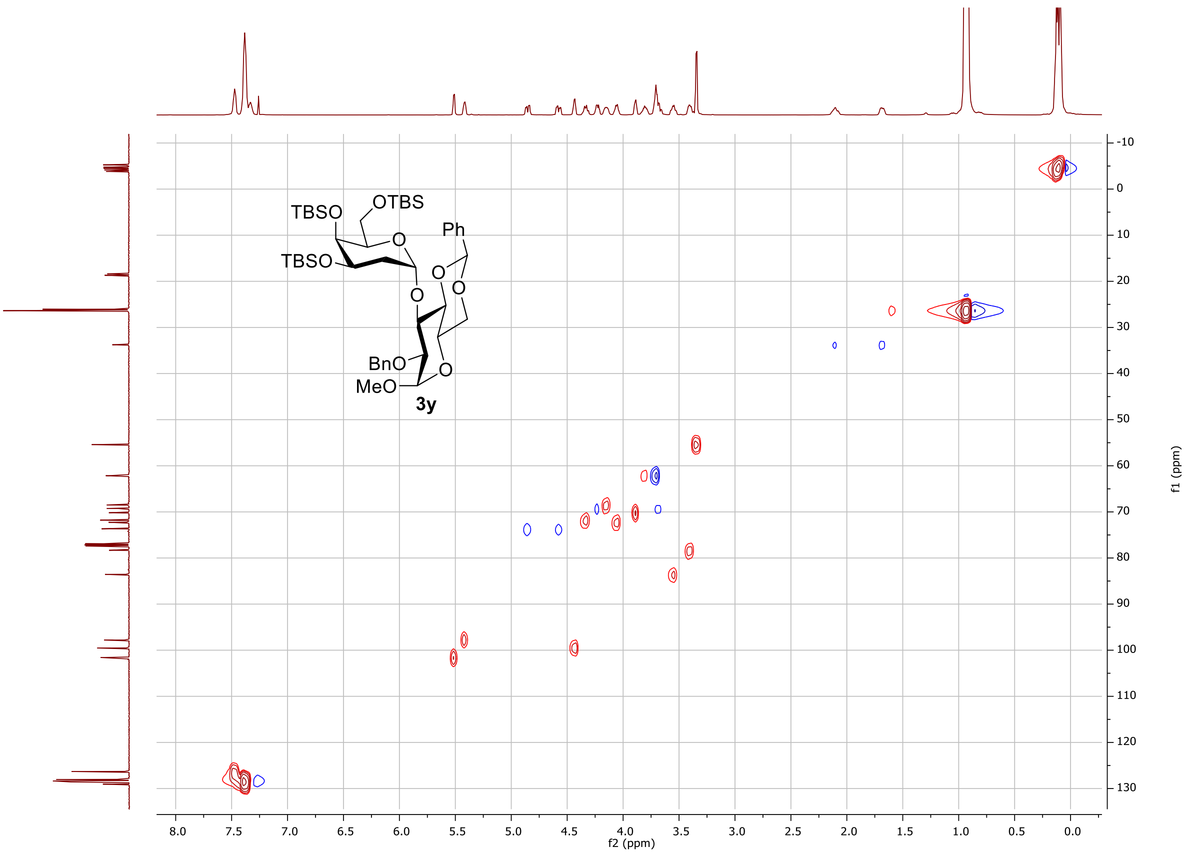

Supplementary figure S265: HSQC spectra for **3y**

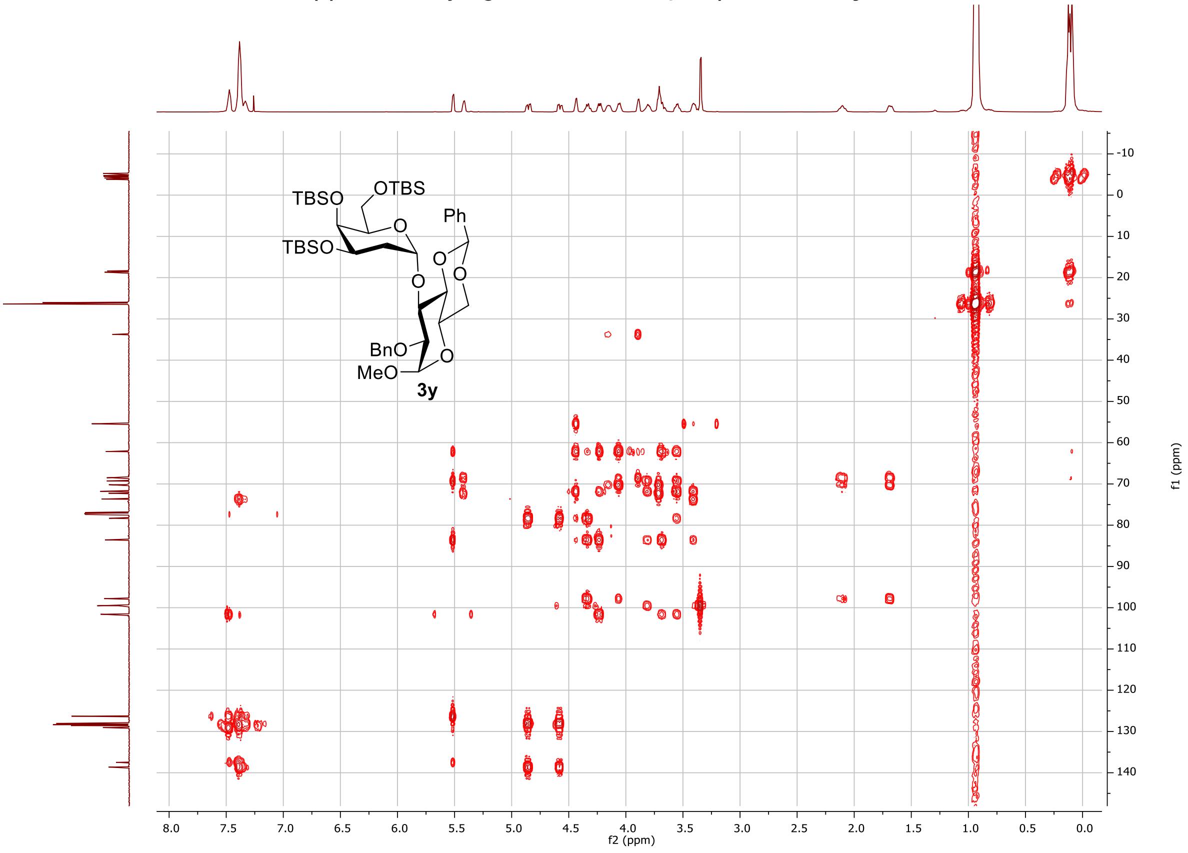

Supplementary figure S266: HMBC spectra for **3y**

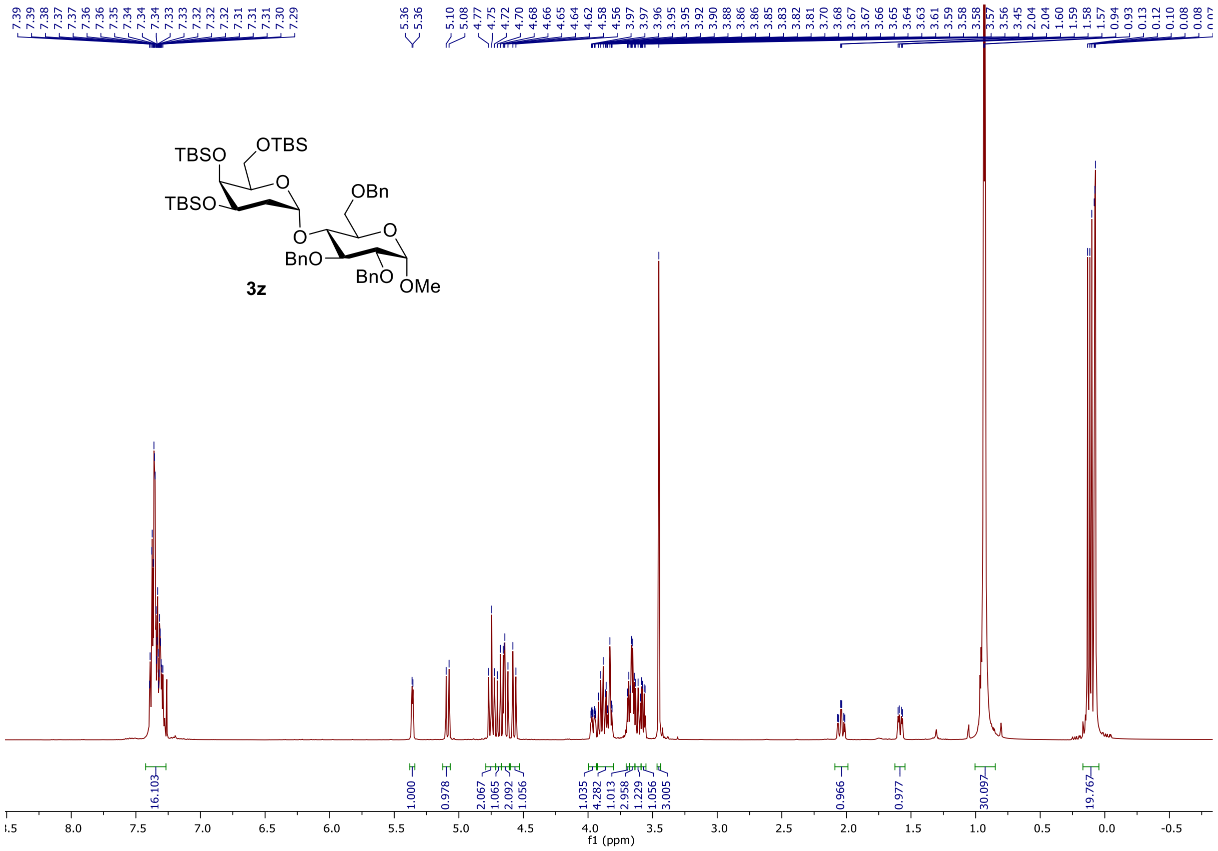

Supplementary figure S267: ^{1}H spectra for **3z**

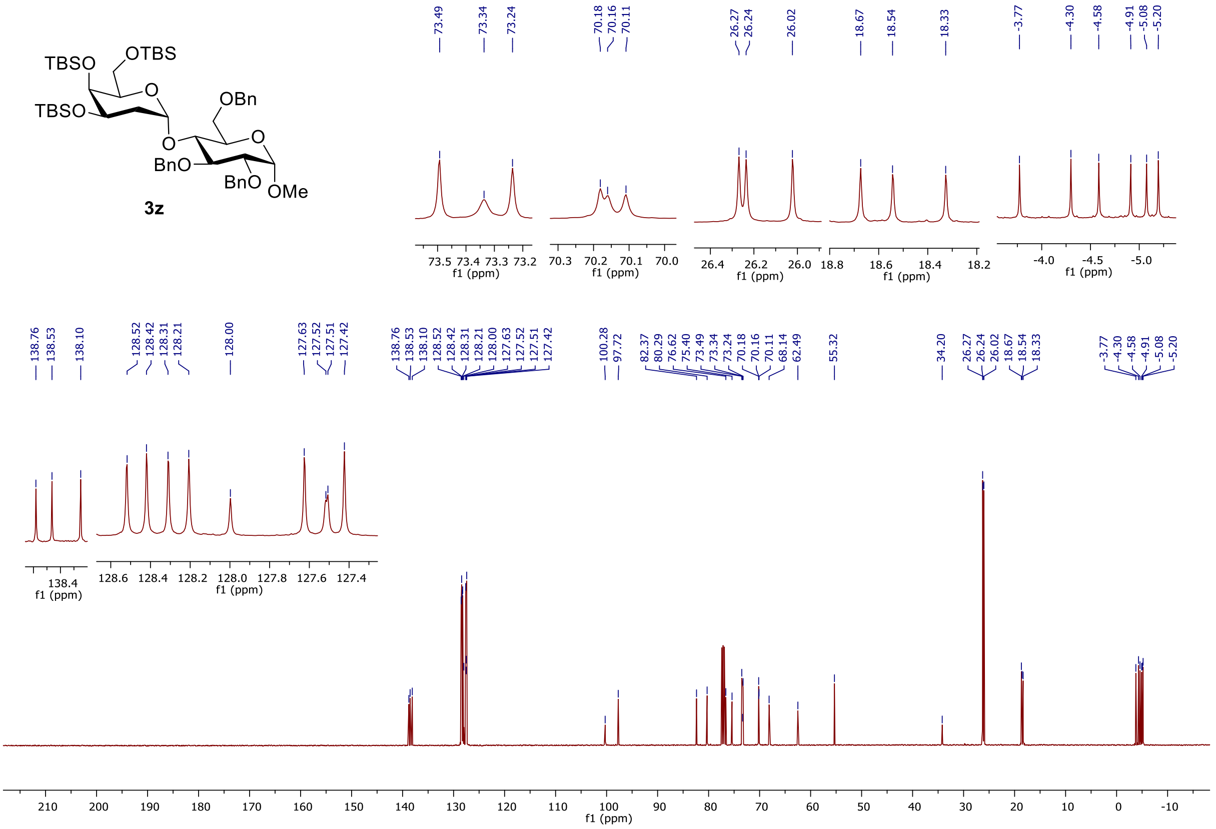

Supplementary figure S268: ^{13}C spectra for **3z**

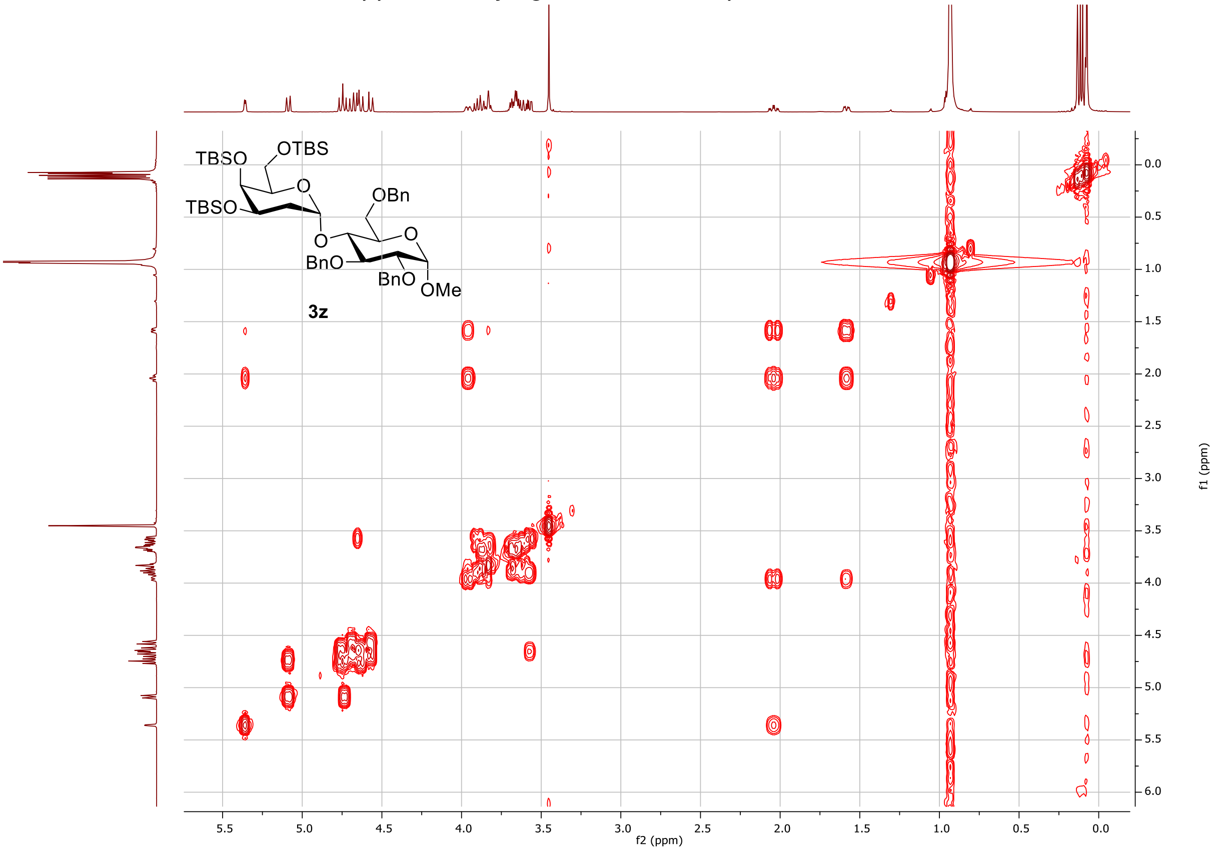

Supplementary figure S269: COSY spectra for **3z**

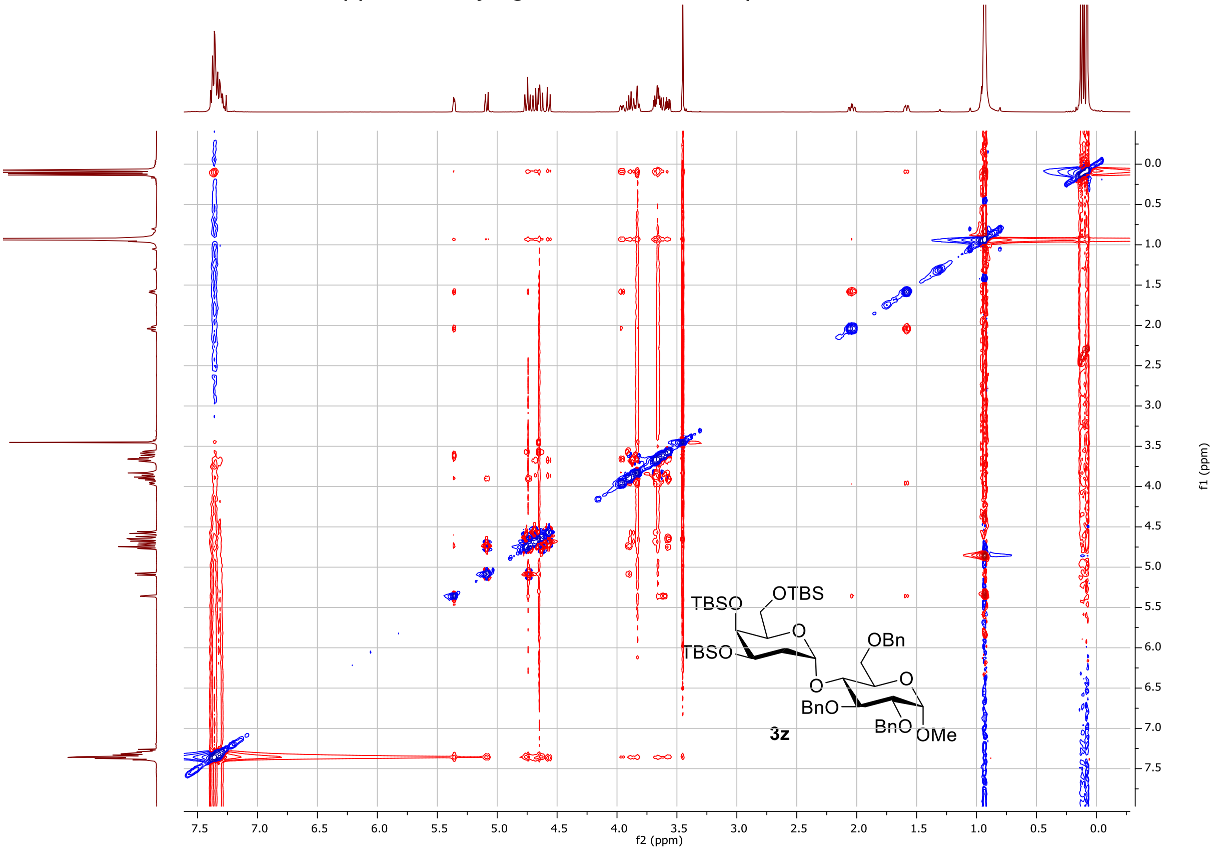

Supplementary figure S270: NOESY spectra for **3z**

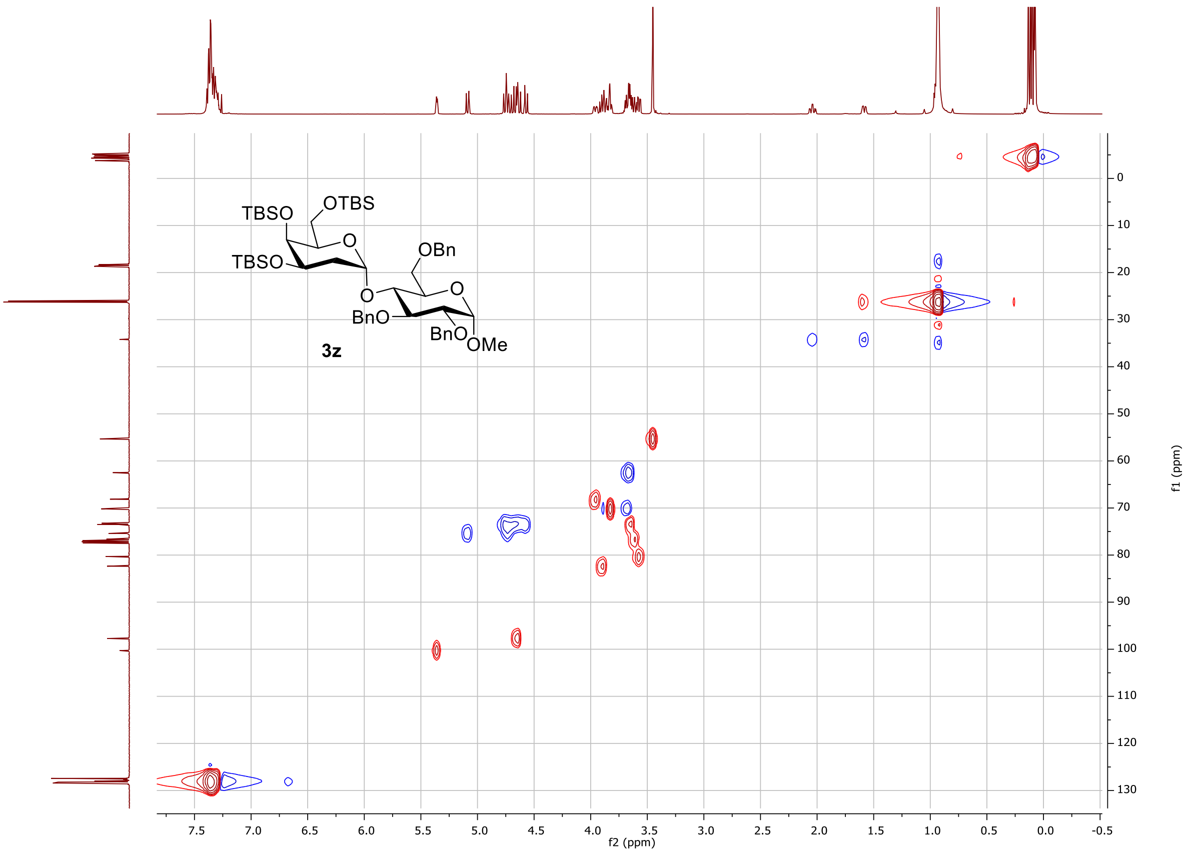

Supplementary figure S271: HSQC spectra for **3z**

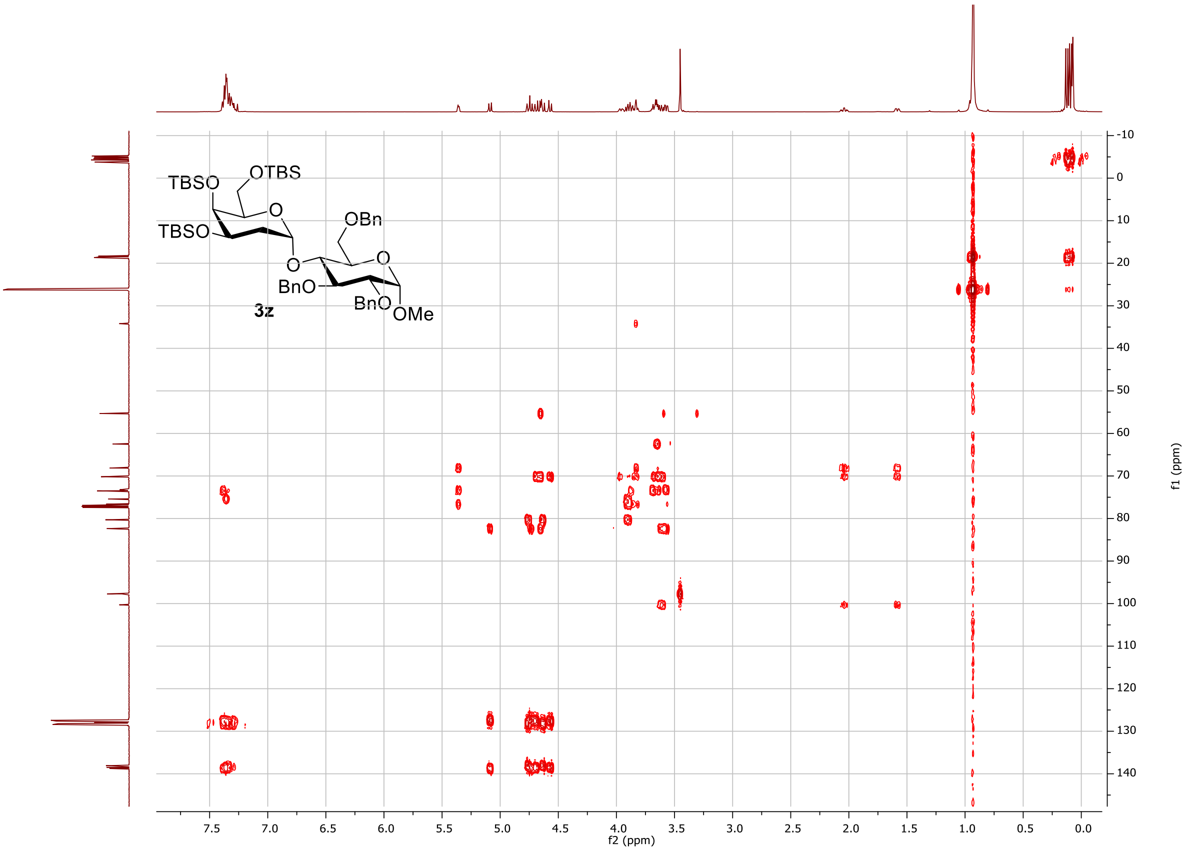

Supplementary figure S272: HMBC spectra for **3z**

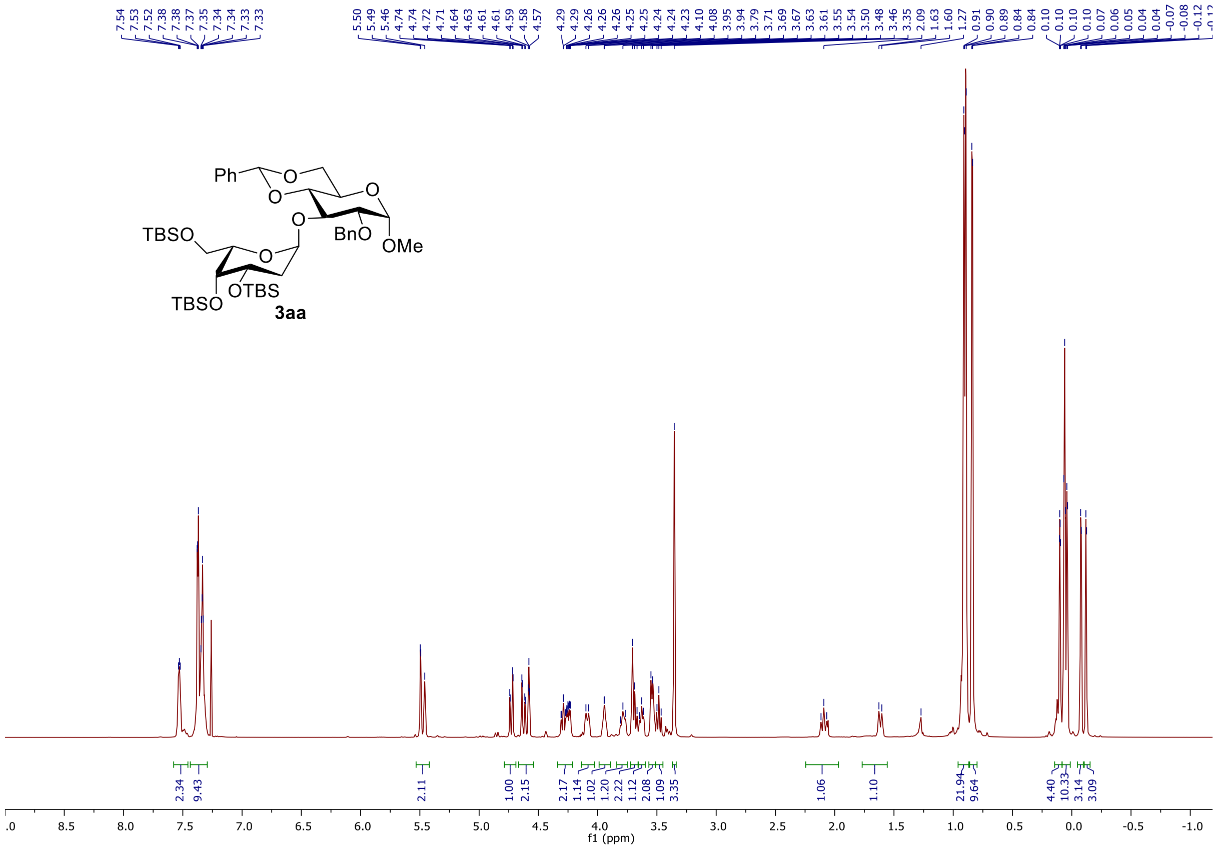

Supplementary figure S273: ^{1}H spectra for **3aa**

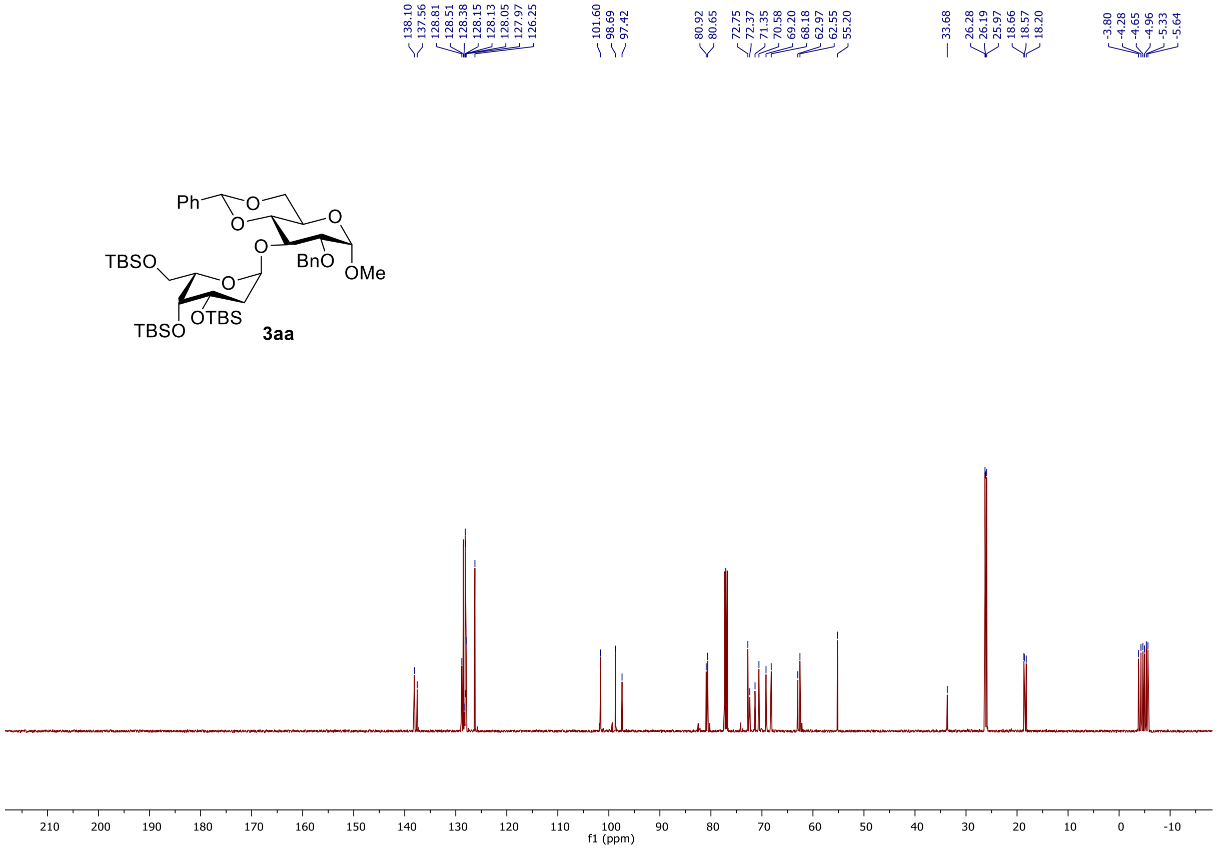

Supplementary figure S274: ^{13}C spectra for **3aa**

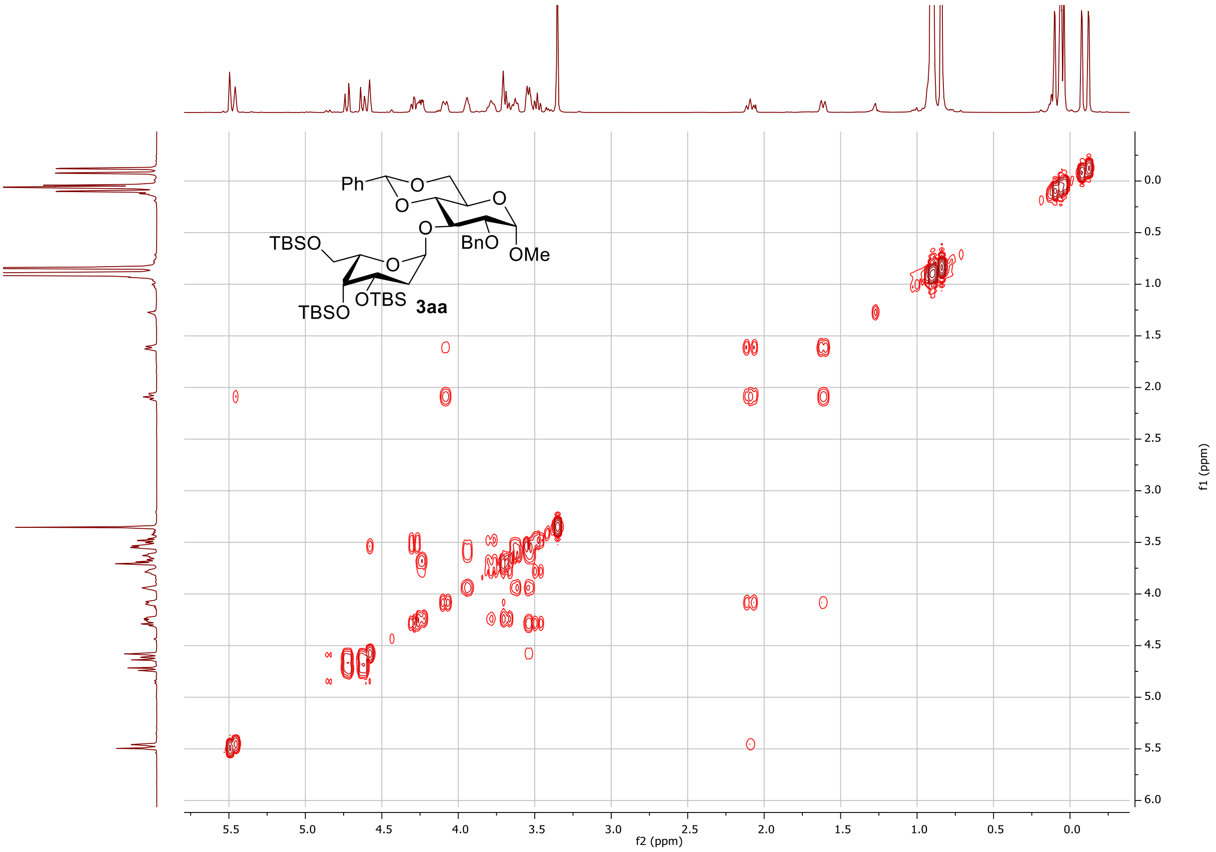

Supplementary figure S275: COSY spectra for **3aa**

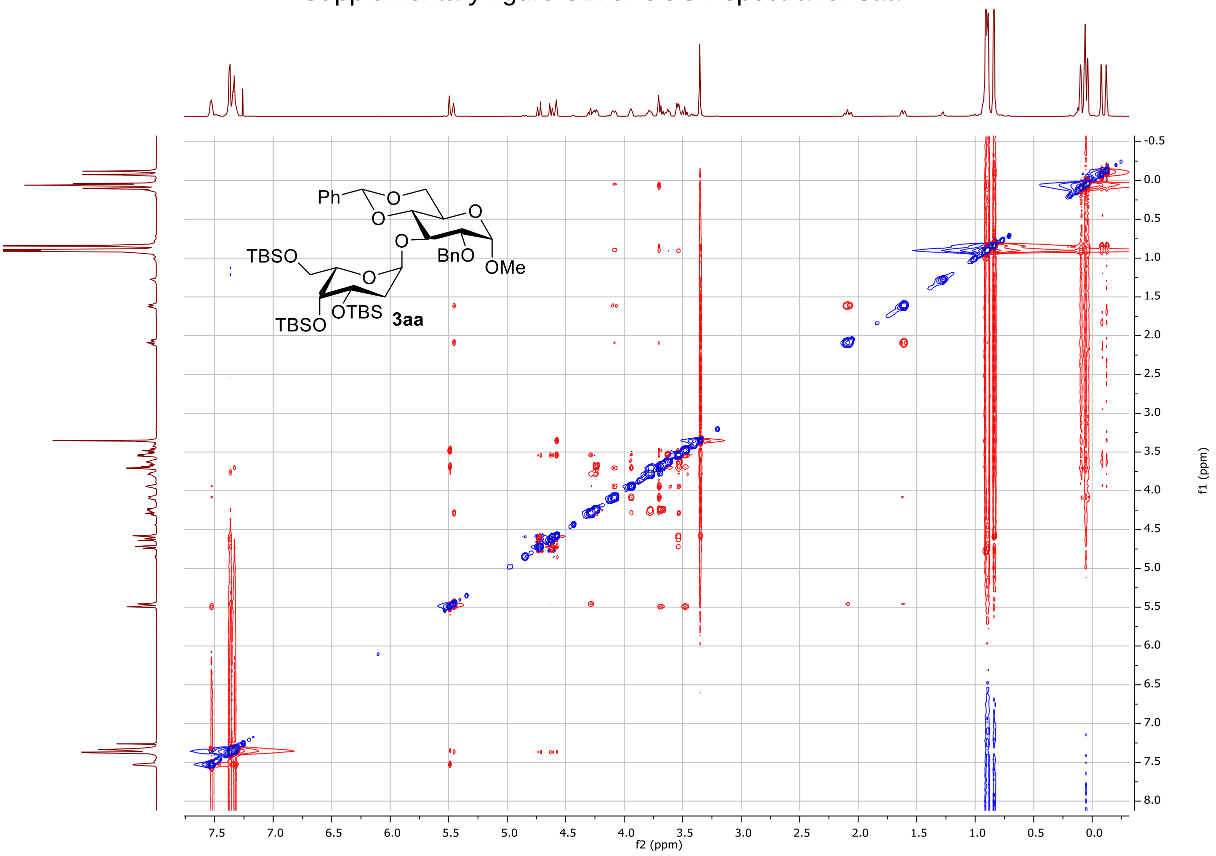

Supplementary figure S276: NOESY spectra for **3aa**

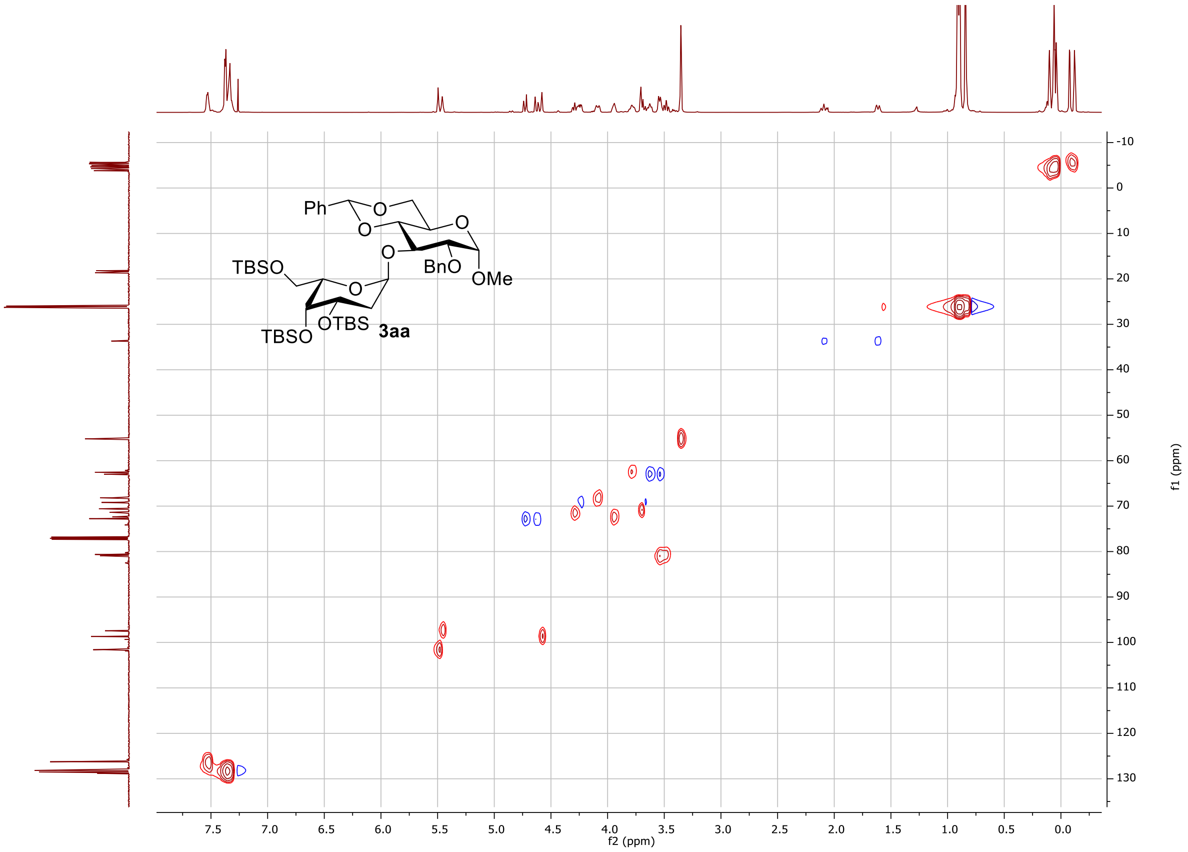

Supplementary figure S277: HSQC spectra for **3aa**

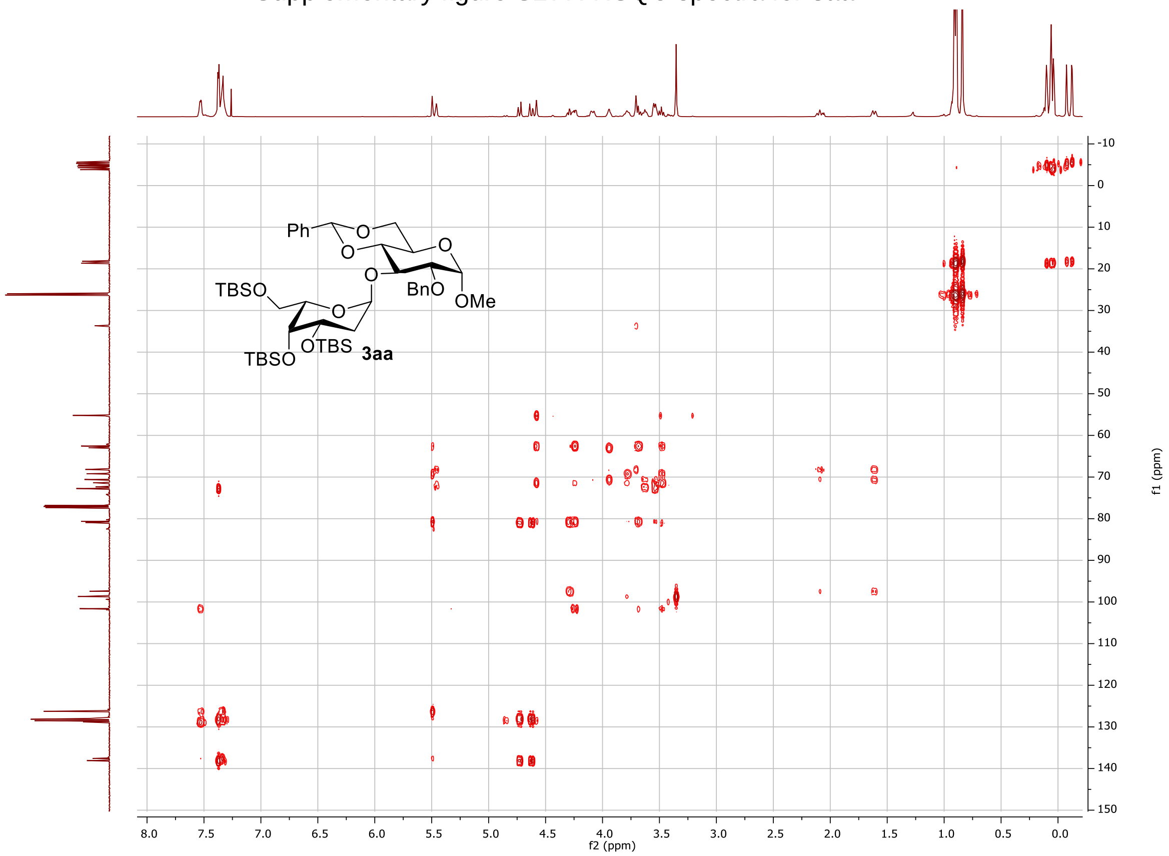

Supplementary figure S278: HMBC spectra for **3aa**

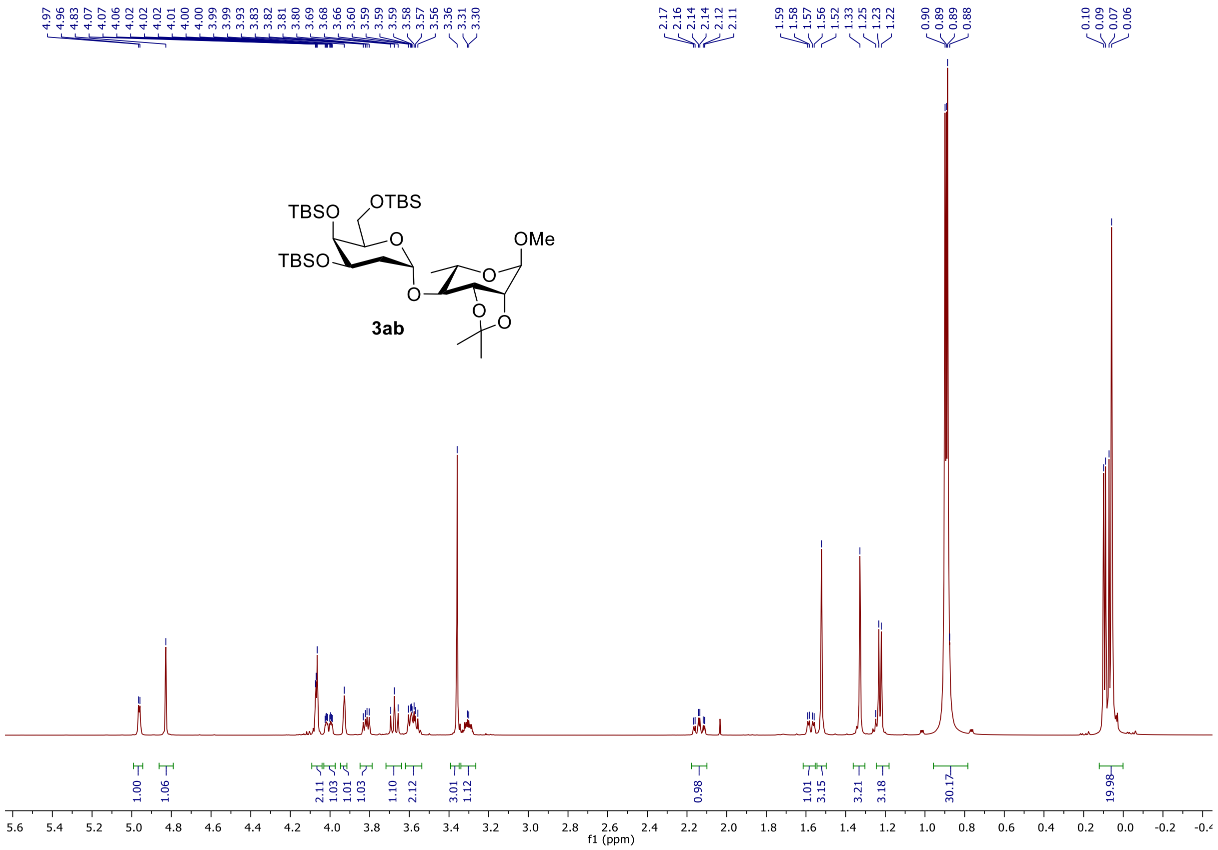

Supplementary figure S279: ^{1}H spectra for **23ab**

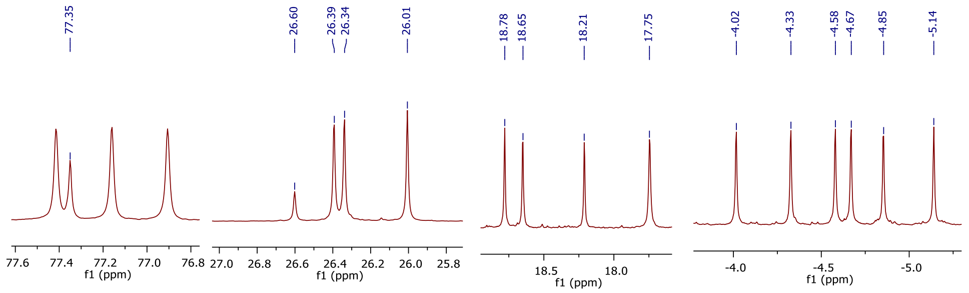

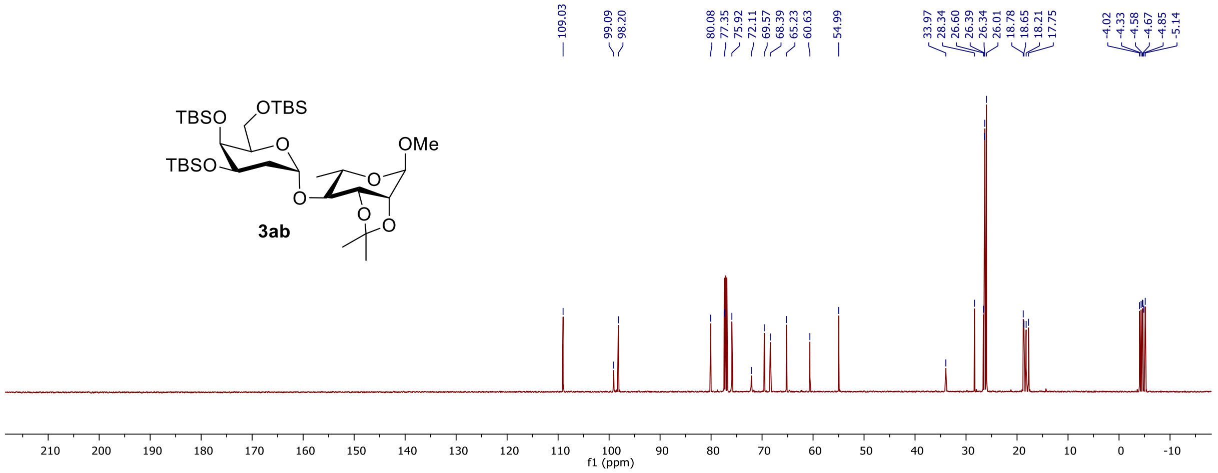

Supplementary figure S280: ^{13}C spectra for **3ab**

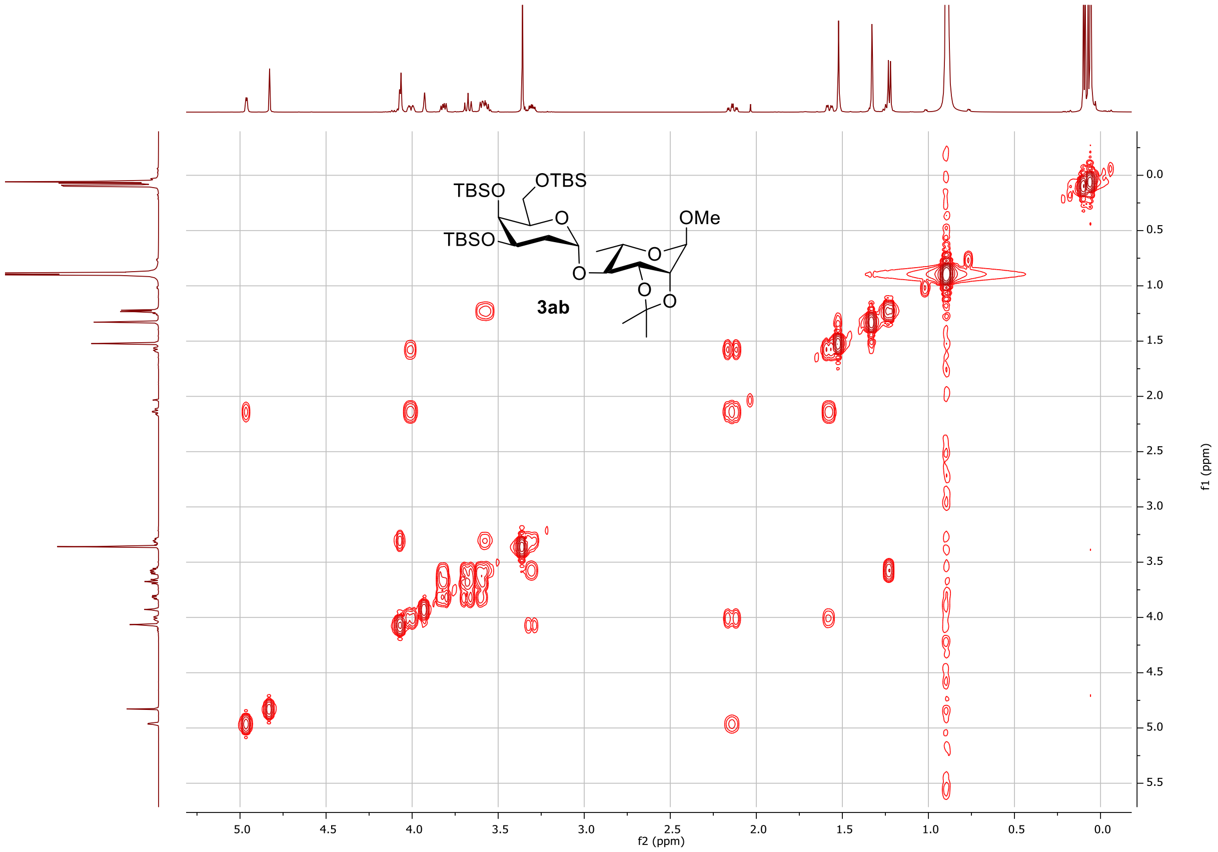

Supplementary figure S281: COSY spectra for **3ab**

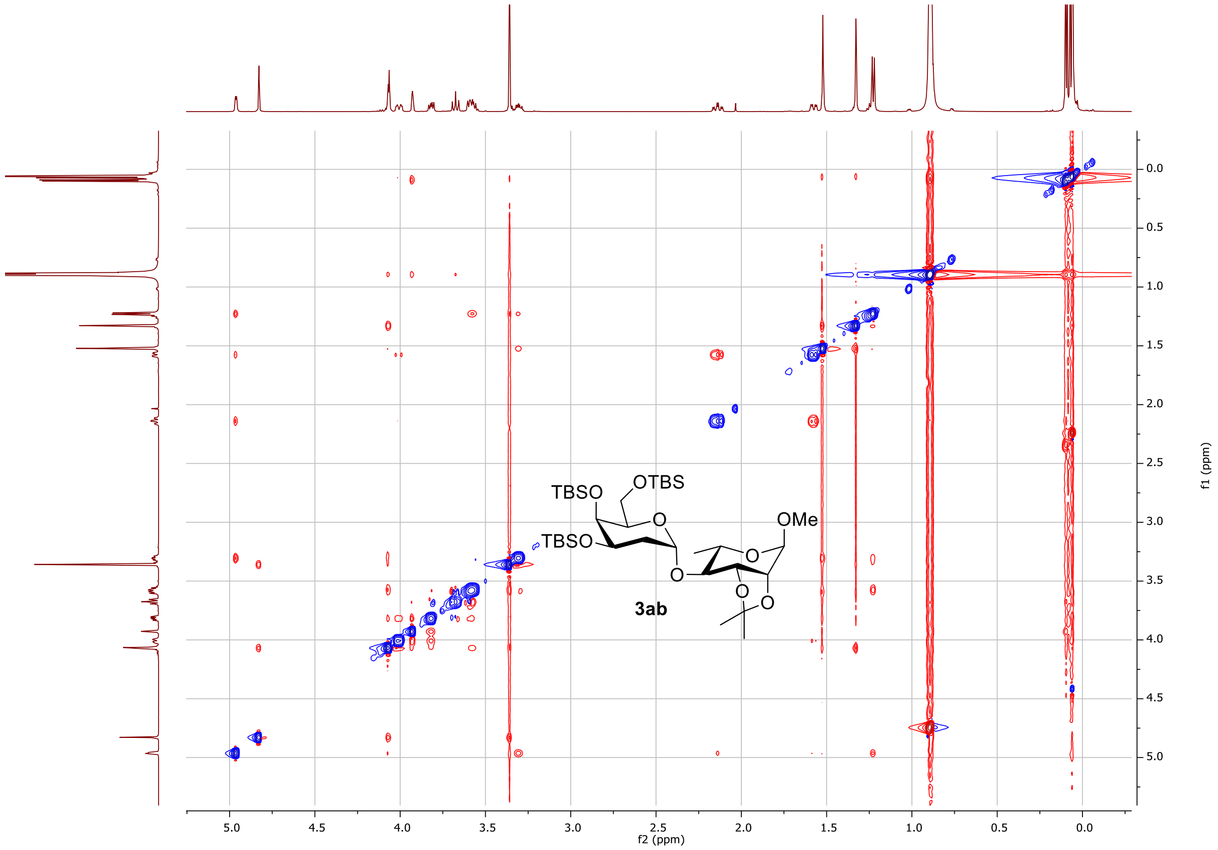

Supplementary figure S282: NOESY spectra for **3ab**

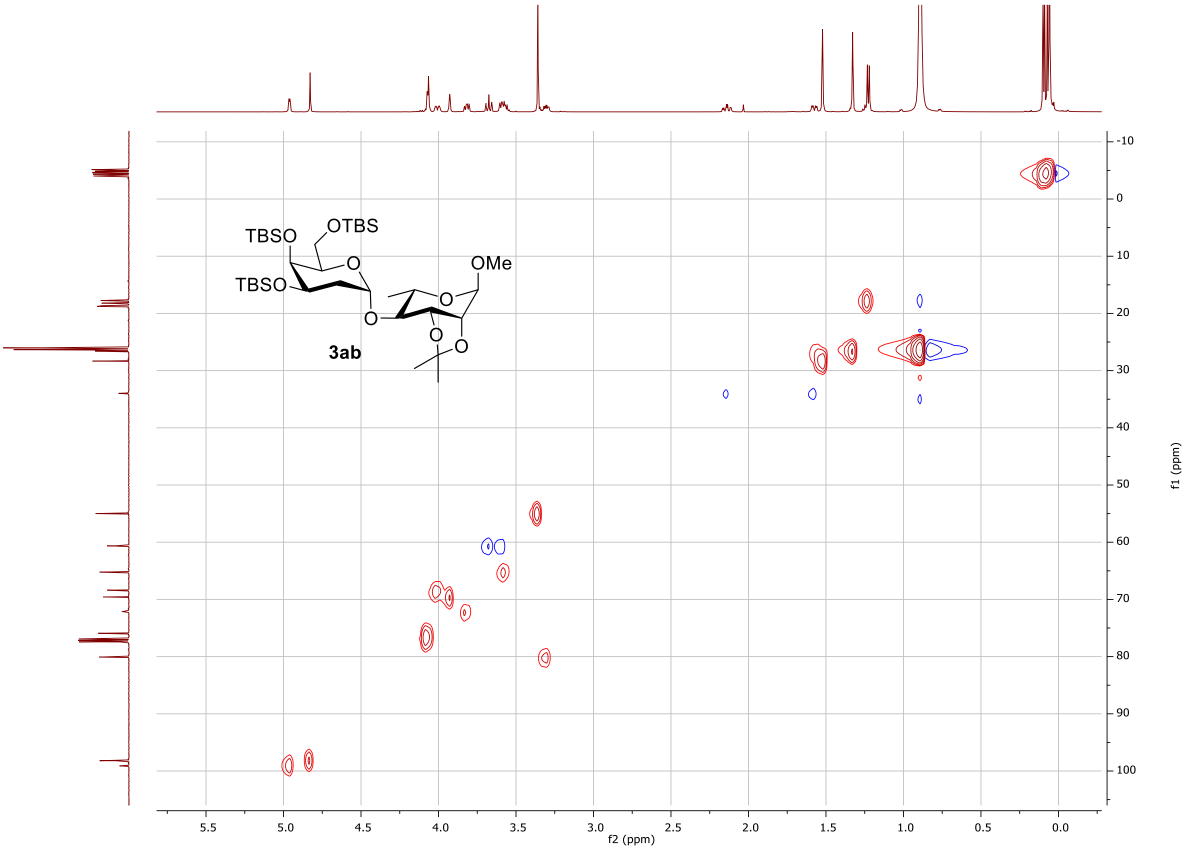

Supplementary figure S283: HSQC spectra for **3ab**

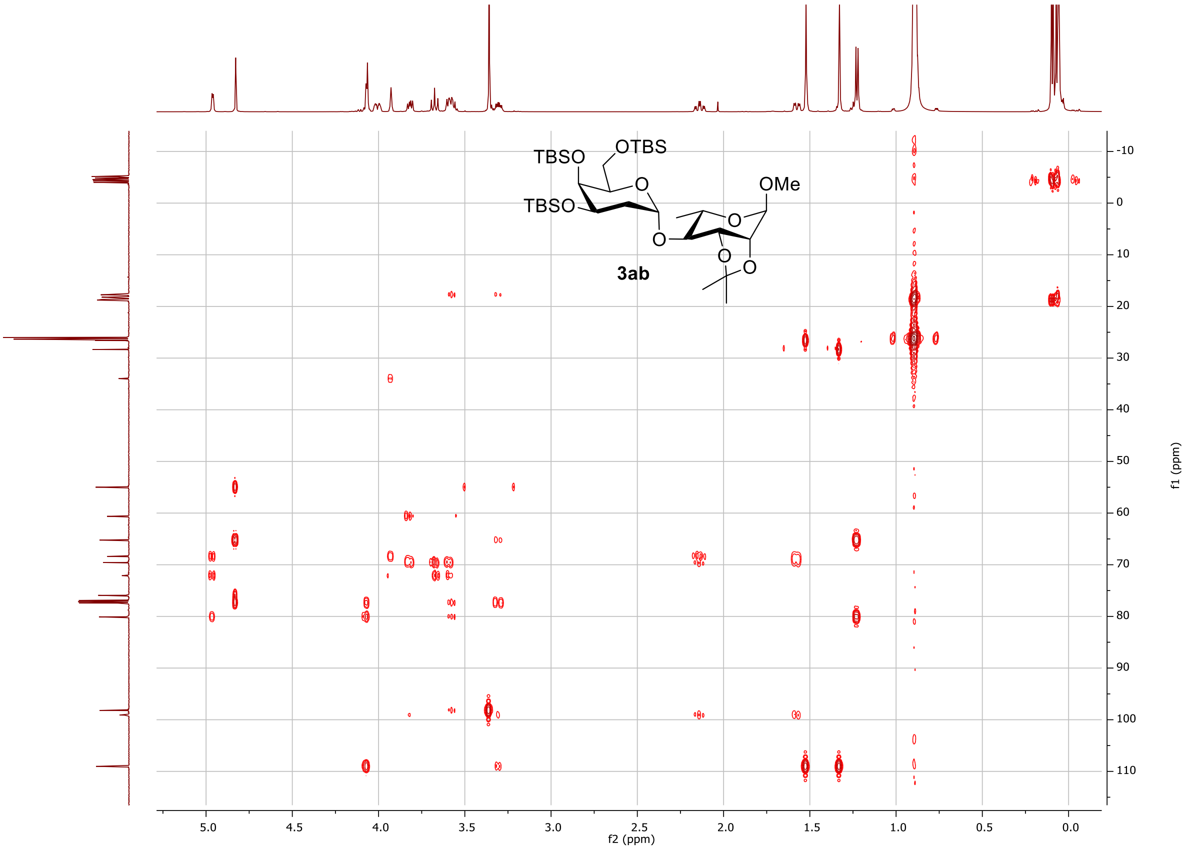

Supplementary figure S284: HMBC spectra for **3ab**

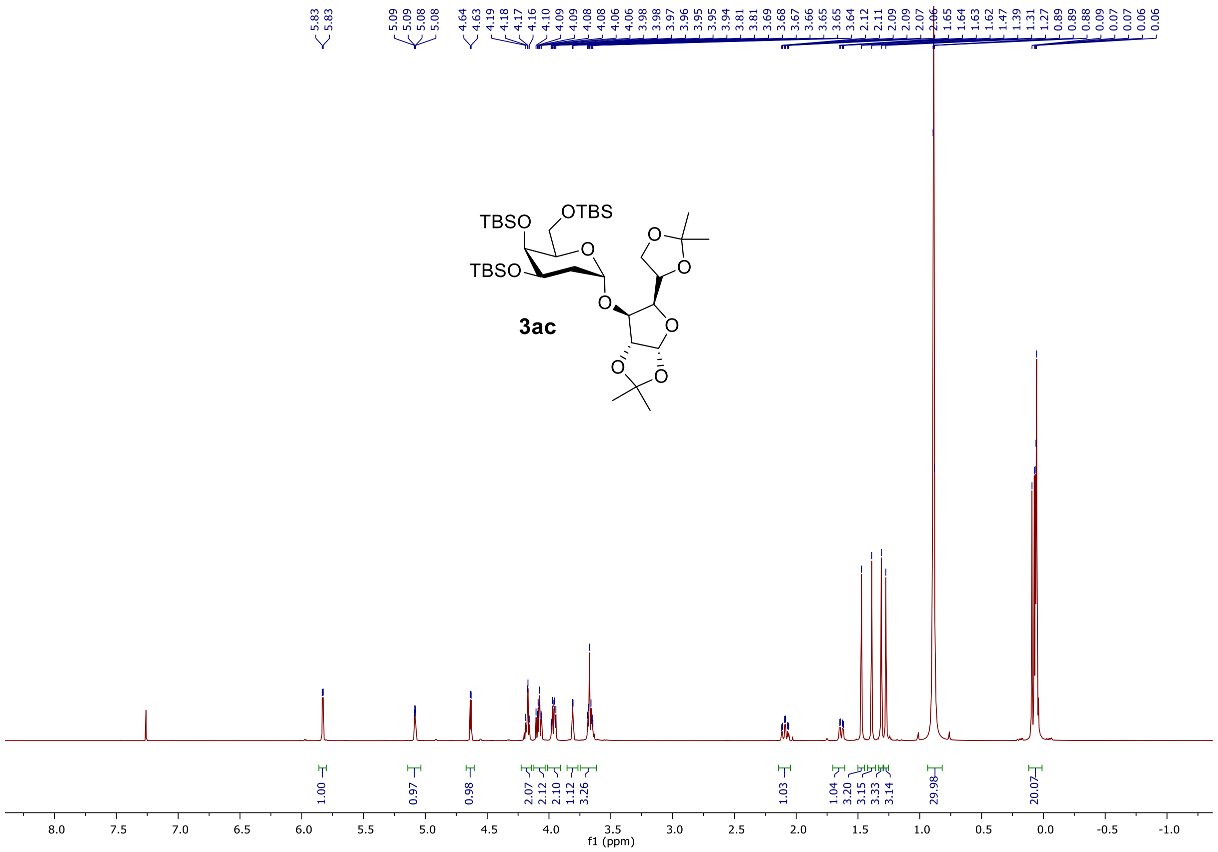

Supplementary figure S285: ^{1}H spectra for **3ac**

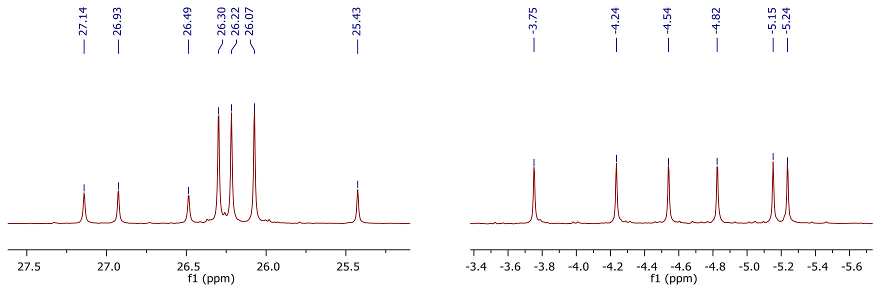

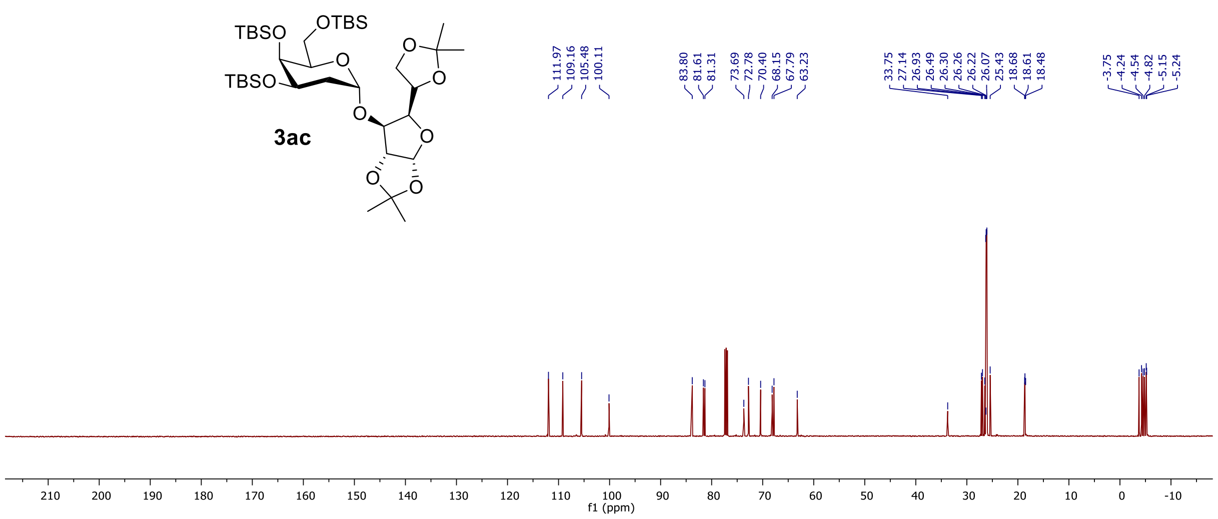

Supplementary figure S286: ^{13}C spectra for **3ac**

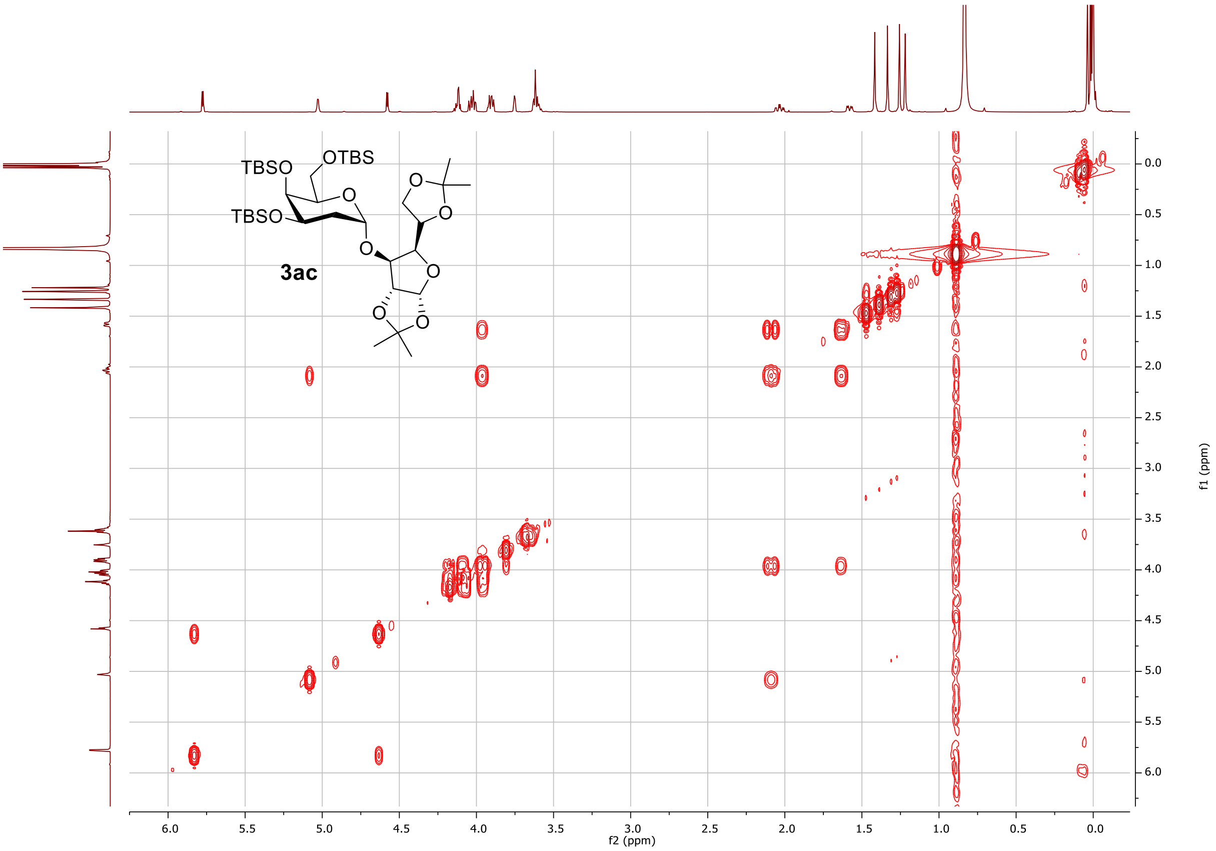

Supplementary figure S287: COSY spectra for **3ac**

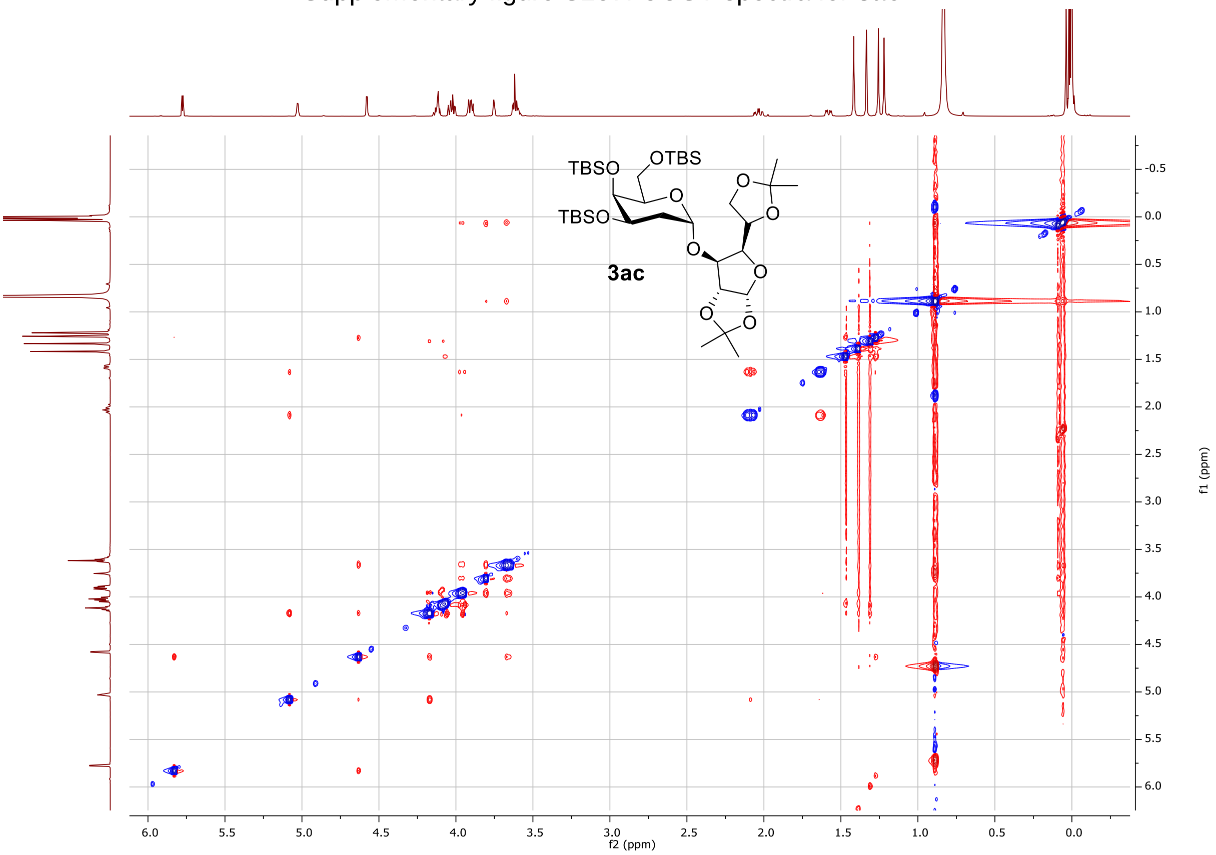

Supplementary figure S288: NOESY spectra for **3ac**

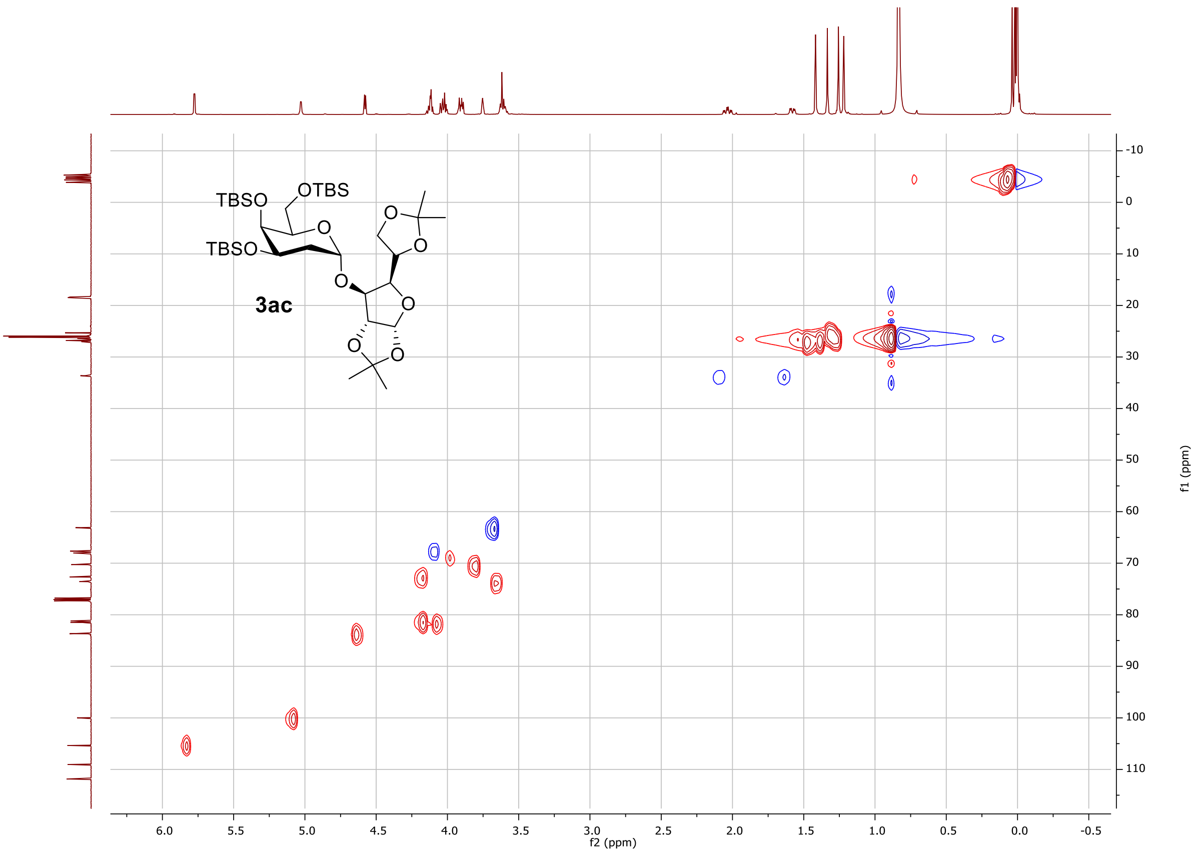

Supplementary figure S289: HSQC spectra for **3ac**

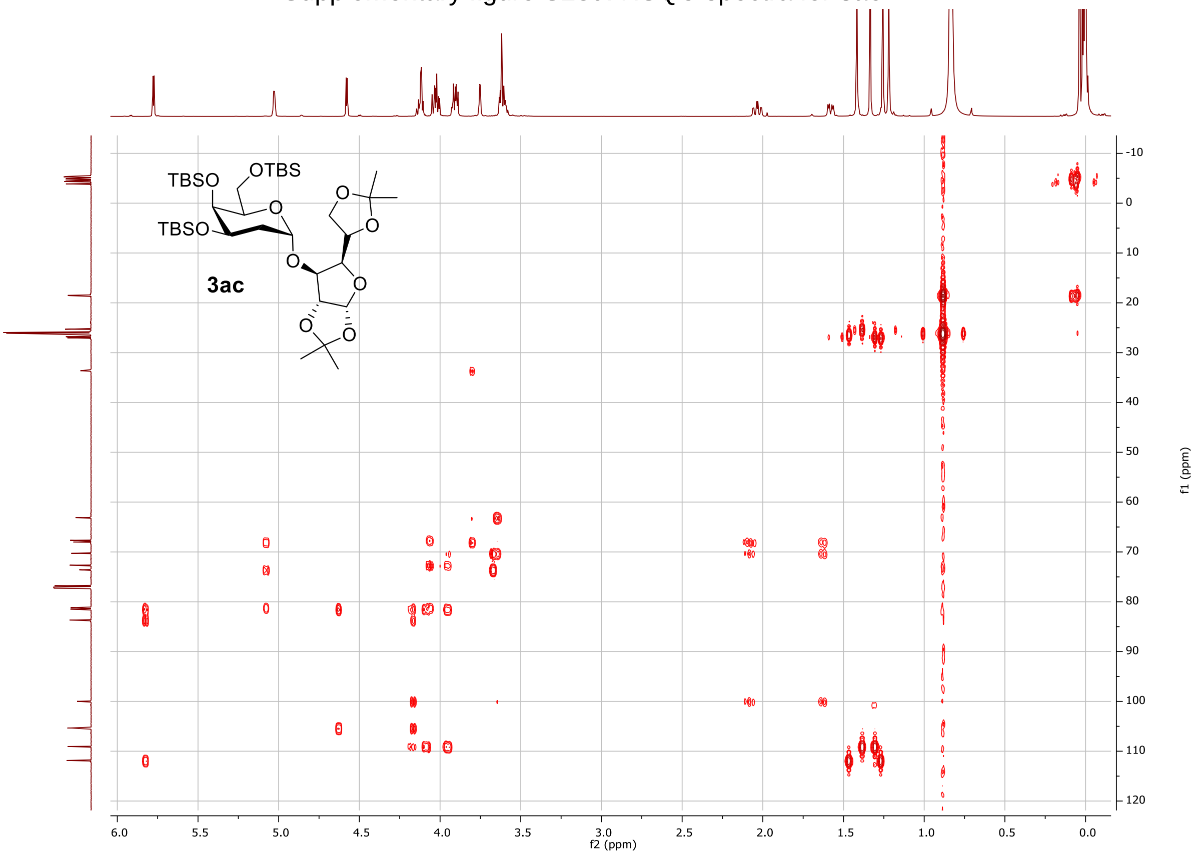

Supplementary figure S290: HMBC spectra for **3ac**

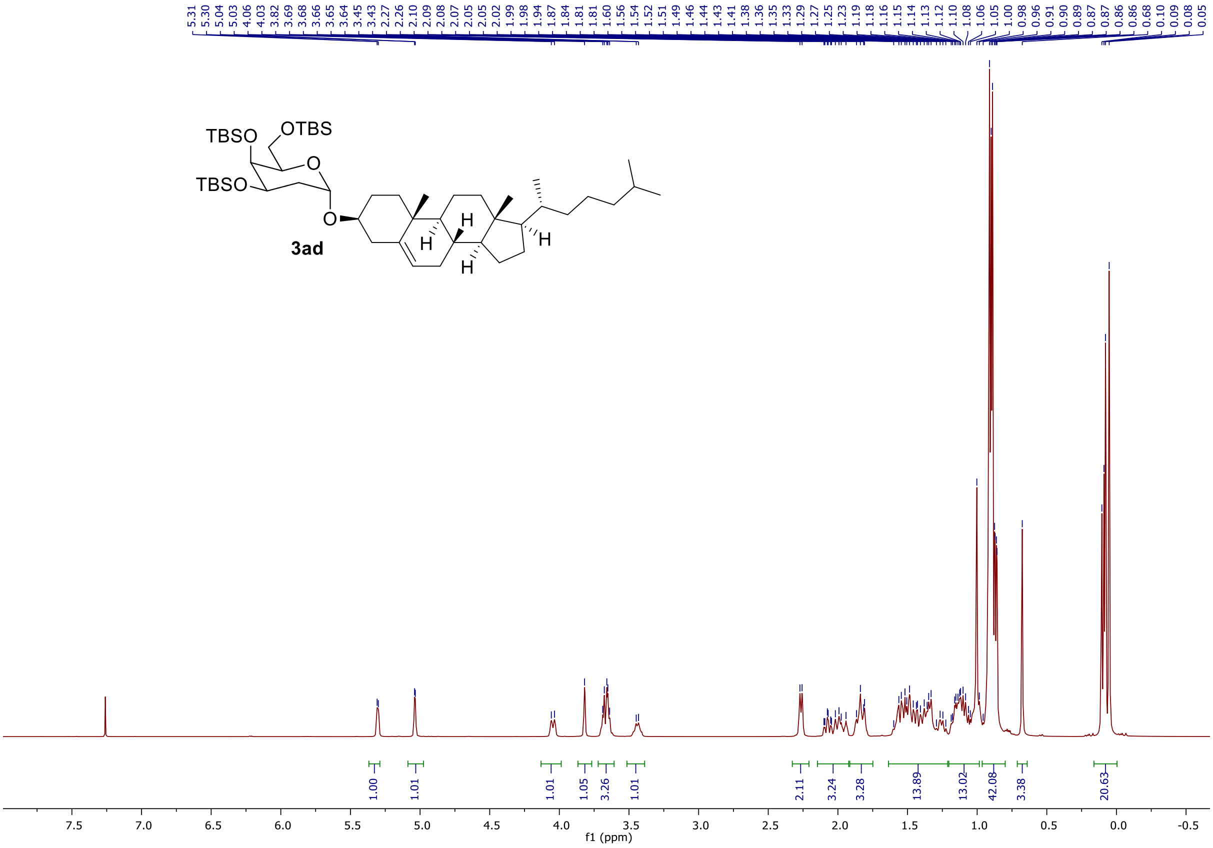

Supplementary figure S291: ^{1}H spectra for **3ad**

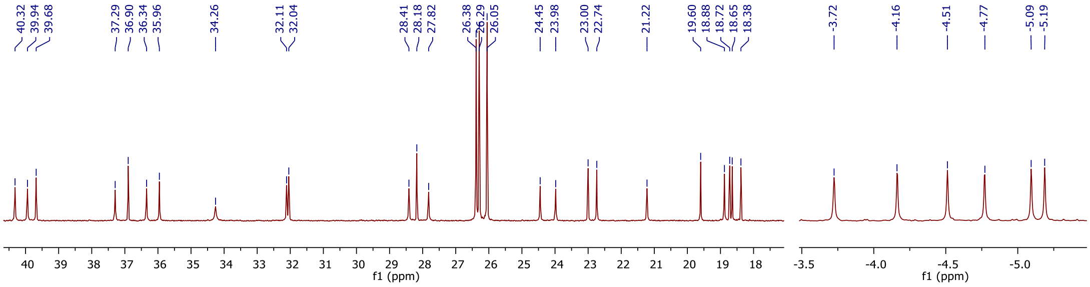

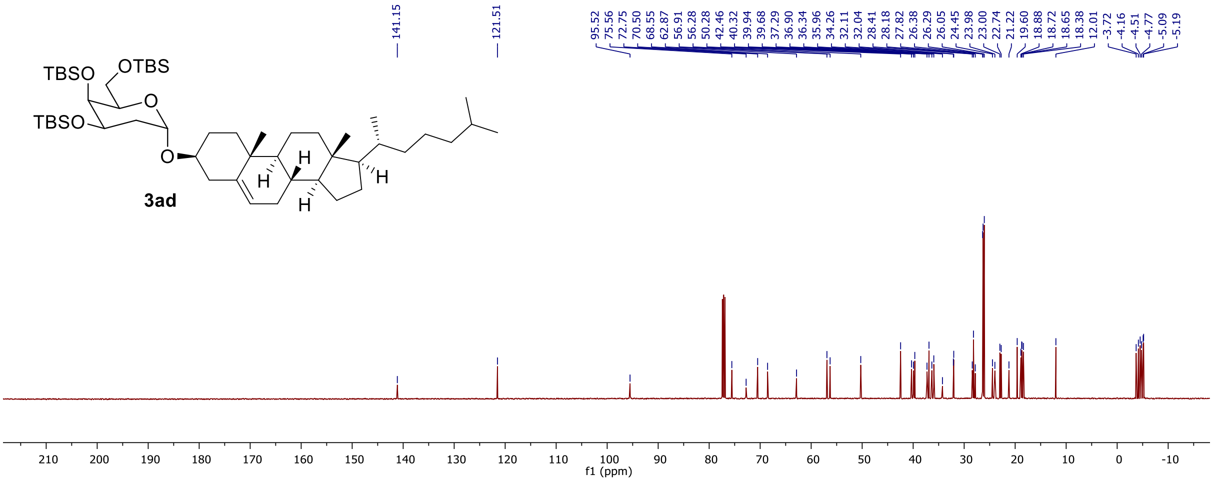

Supplementary figure S292: ^{13}C spectra for **3ad**

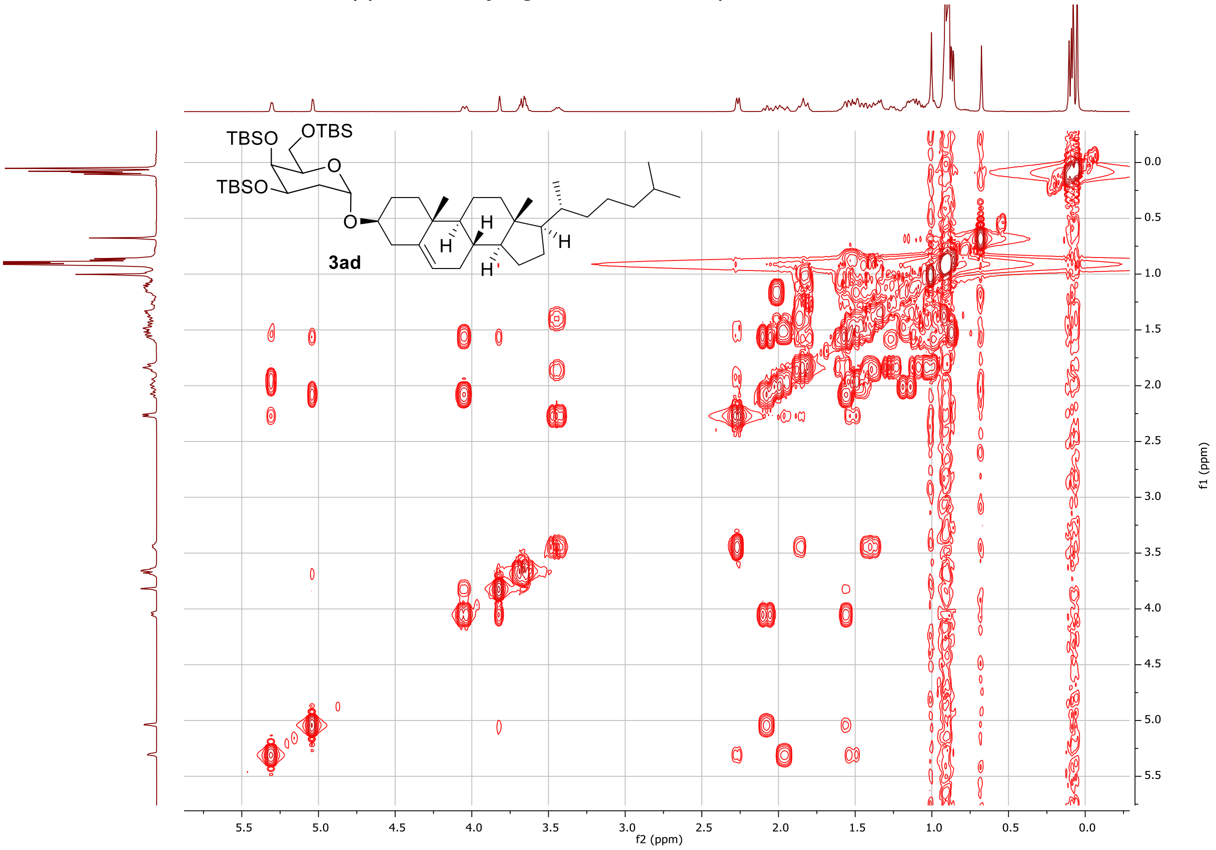

Supplementary figure S293: COSY spectra for **3ad**

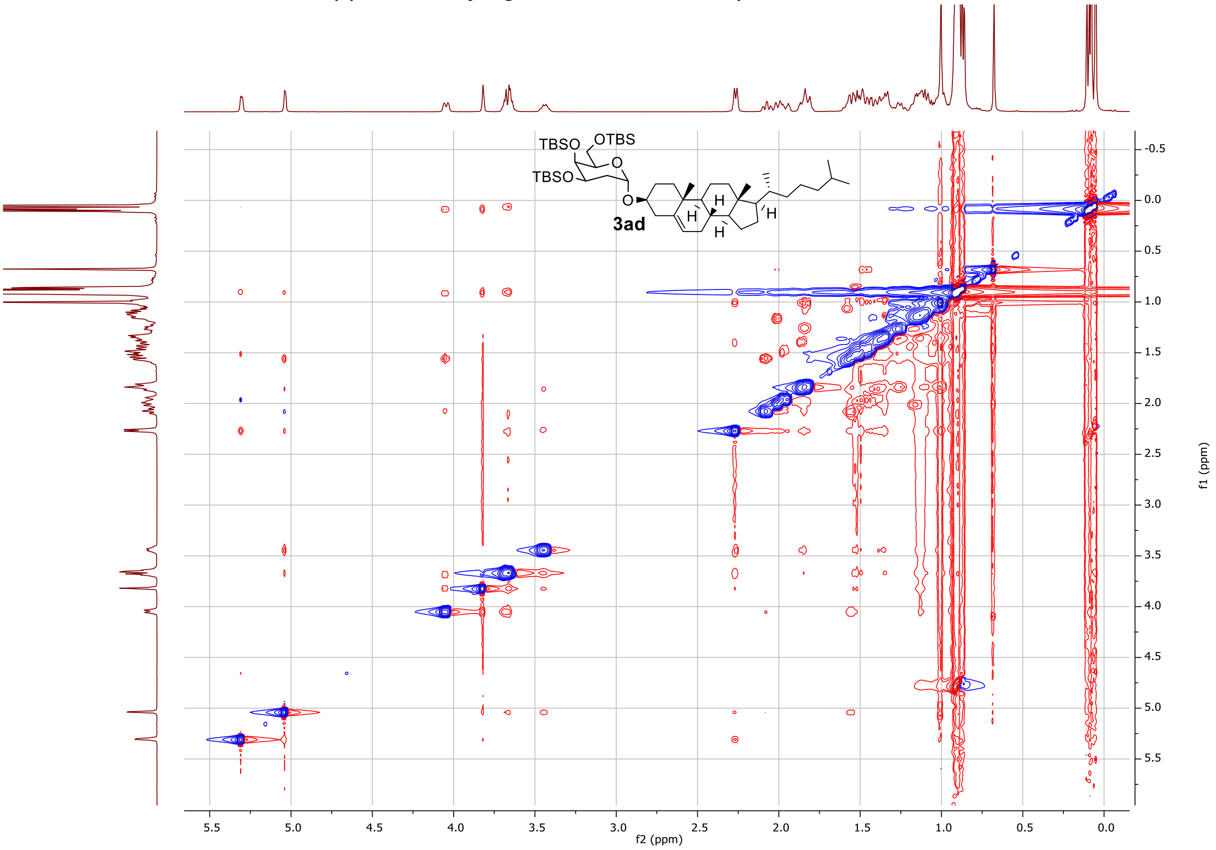

Supplementary figure S294: NOESY spectra for **3ad**

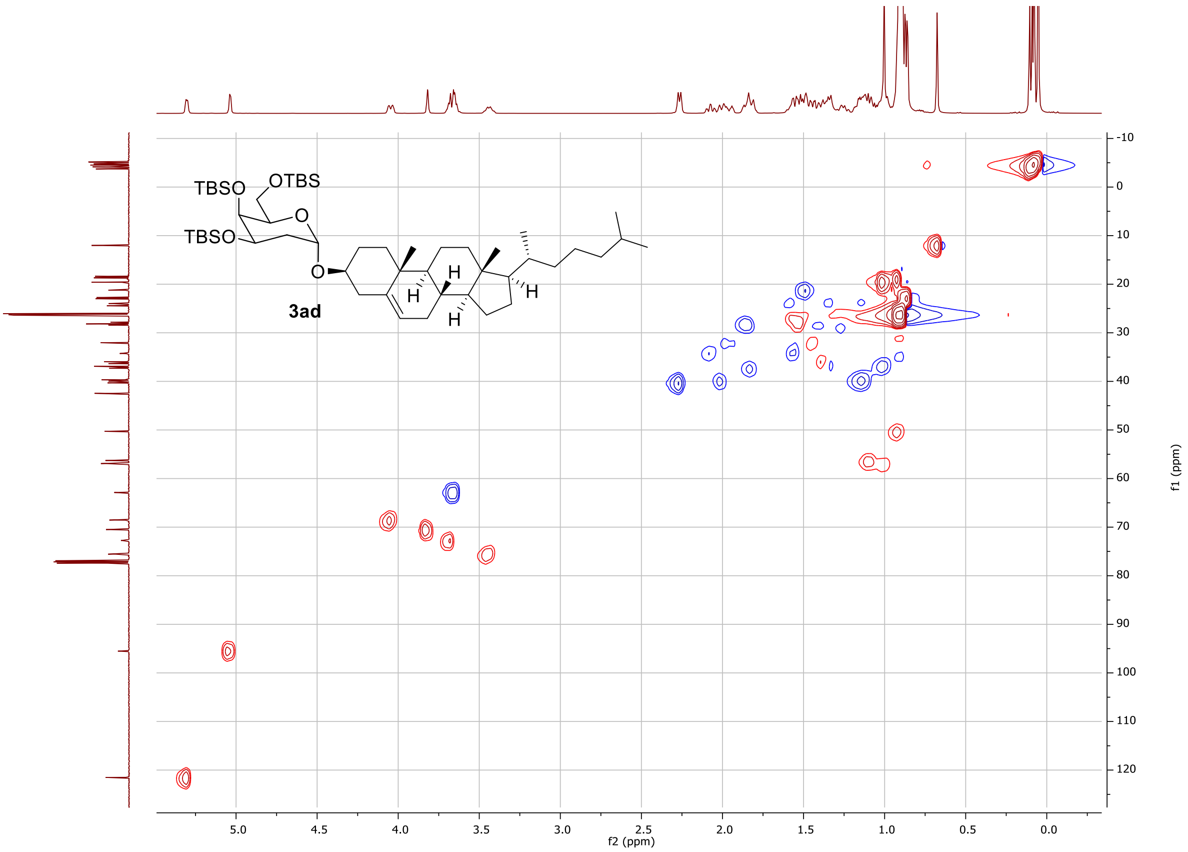

Supplementary figure S295: HSQC spectra for **3ad**

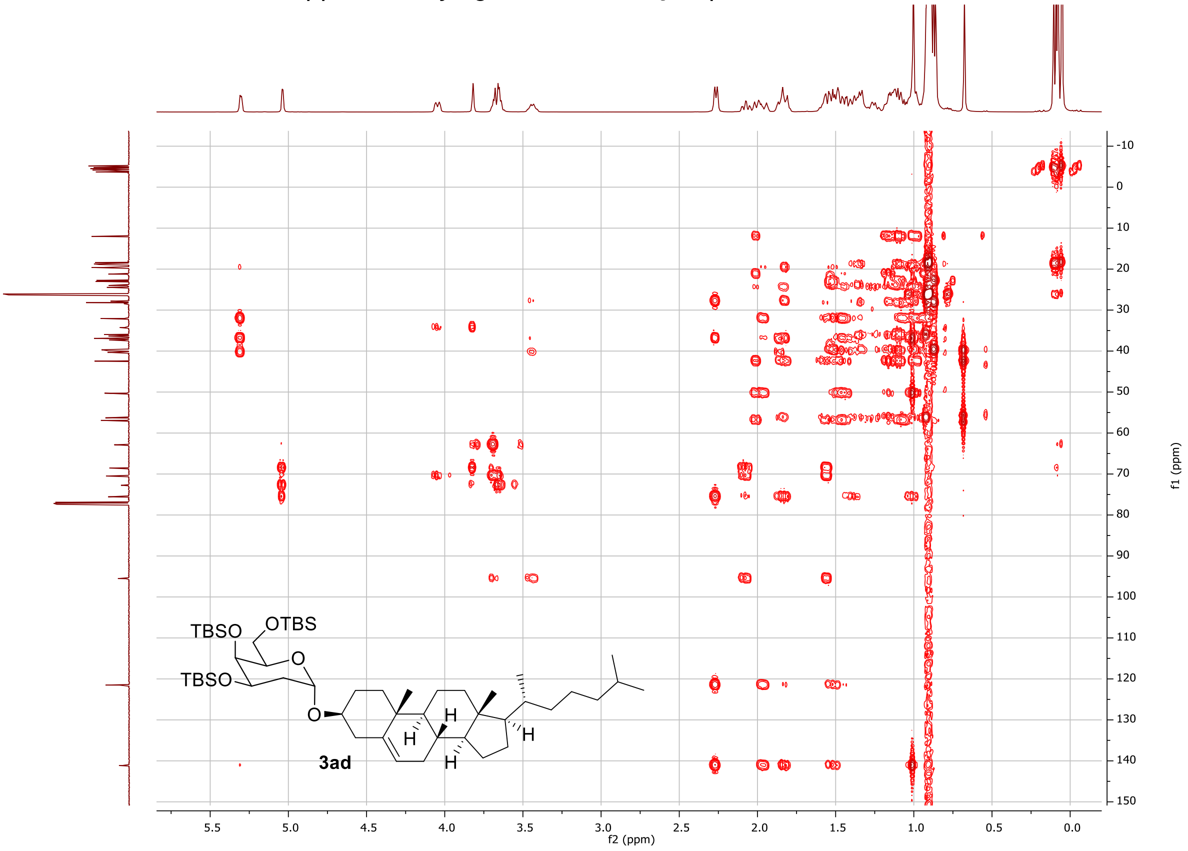

Supplementary figure S296: HMBC spectra for **3ad**

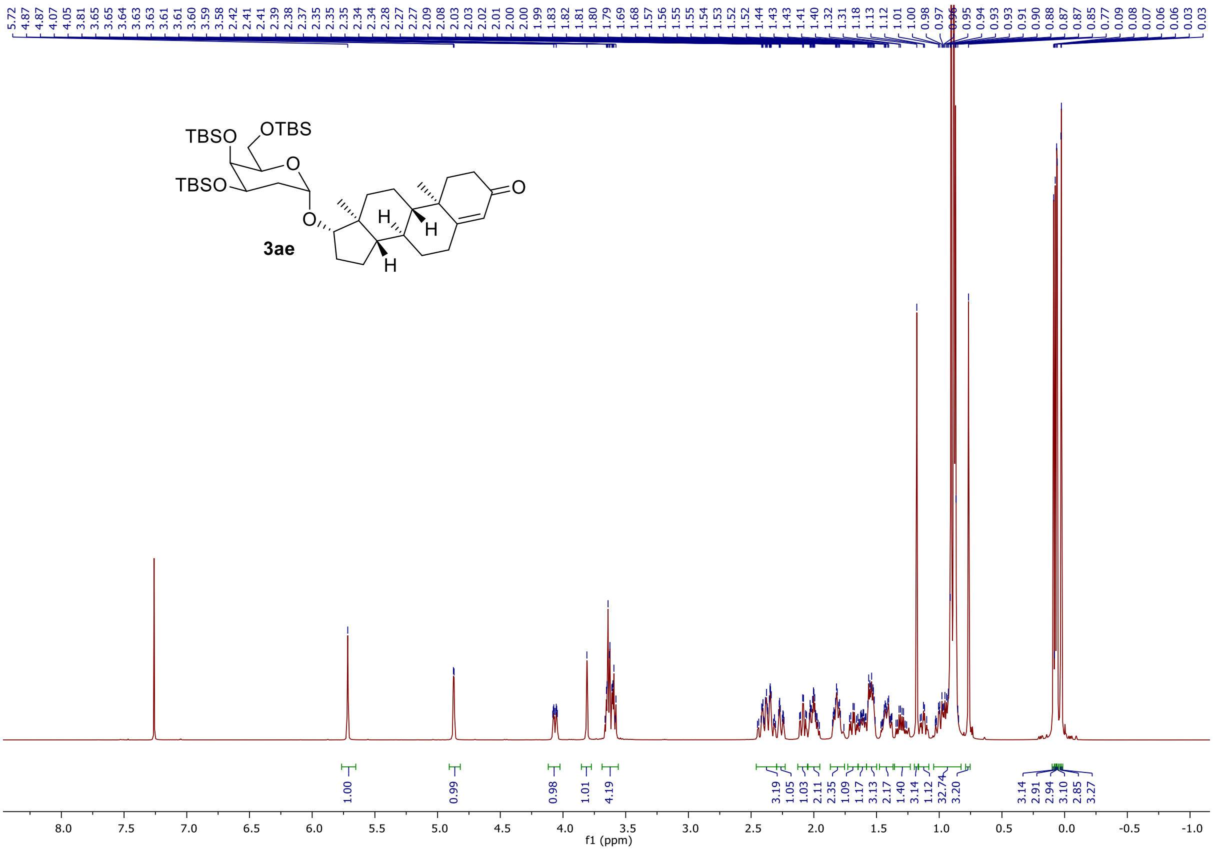

Supplementary figure S297: ^{1}H spectra for **3ae**

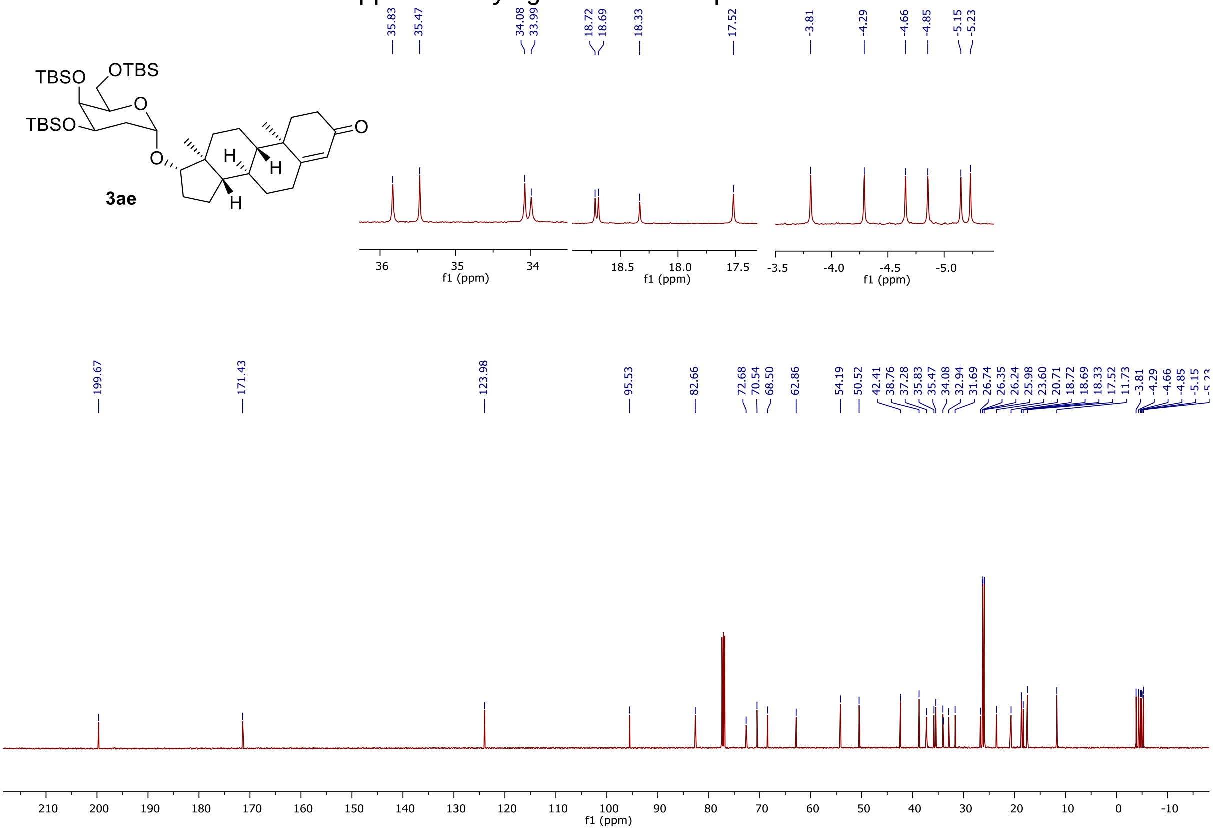

Supplementary figure S298: ^{13}C spectra for **3ae**

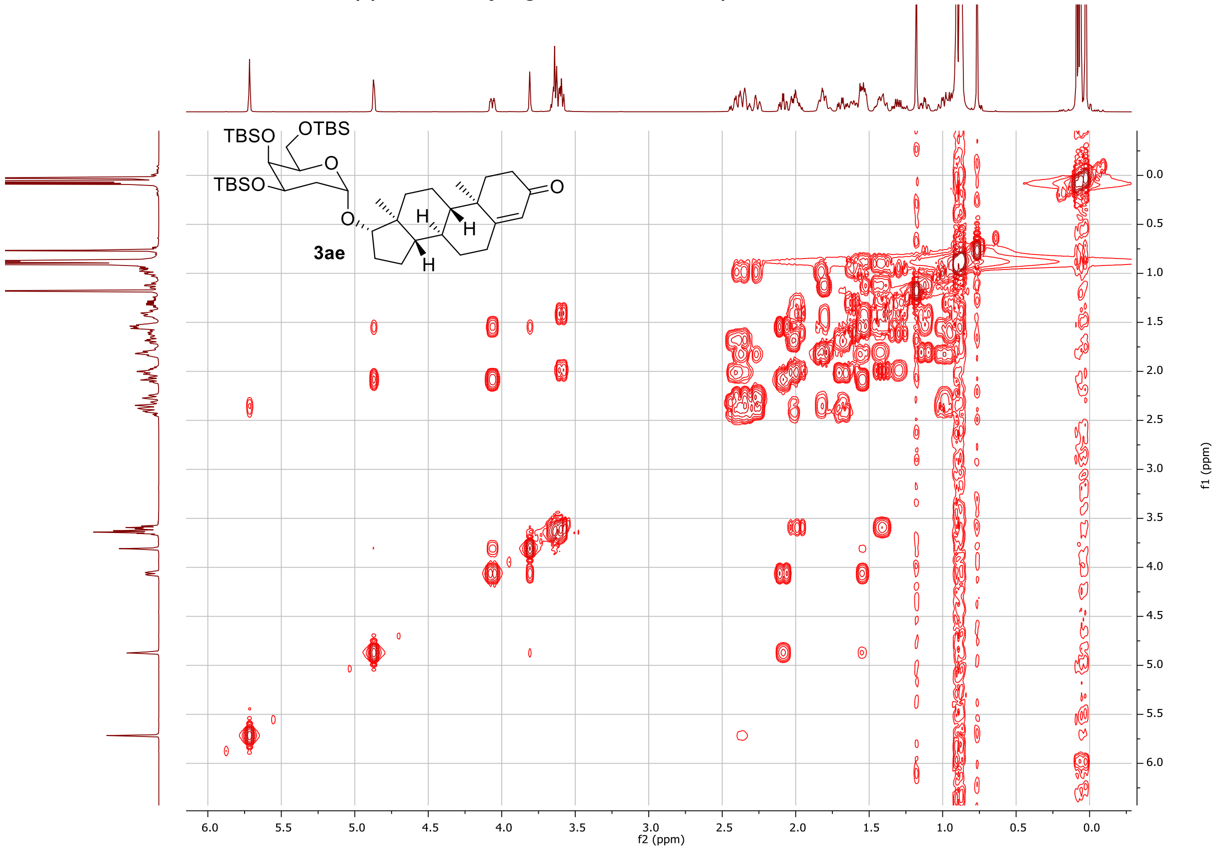

Supplementary figure S299: COSY spectra for **3ae**

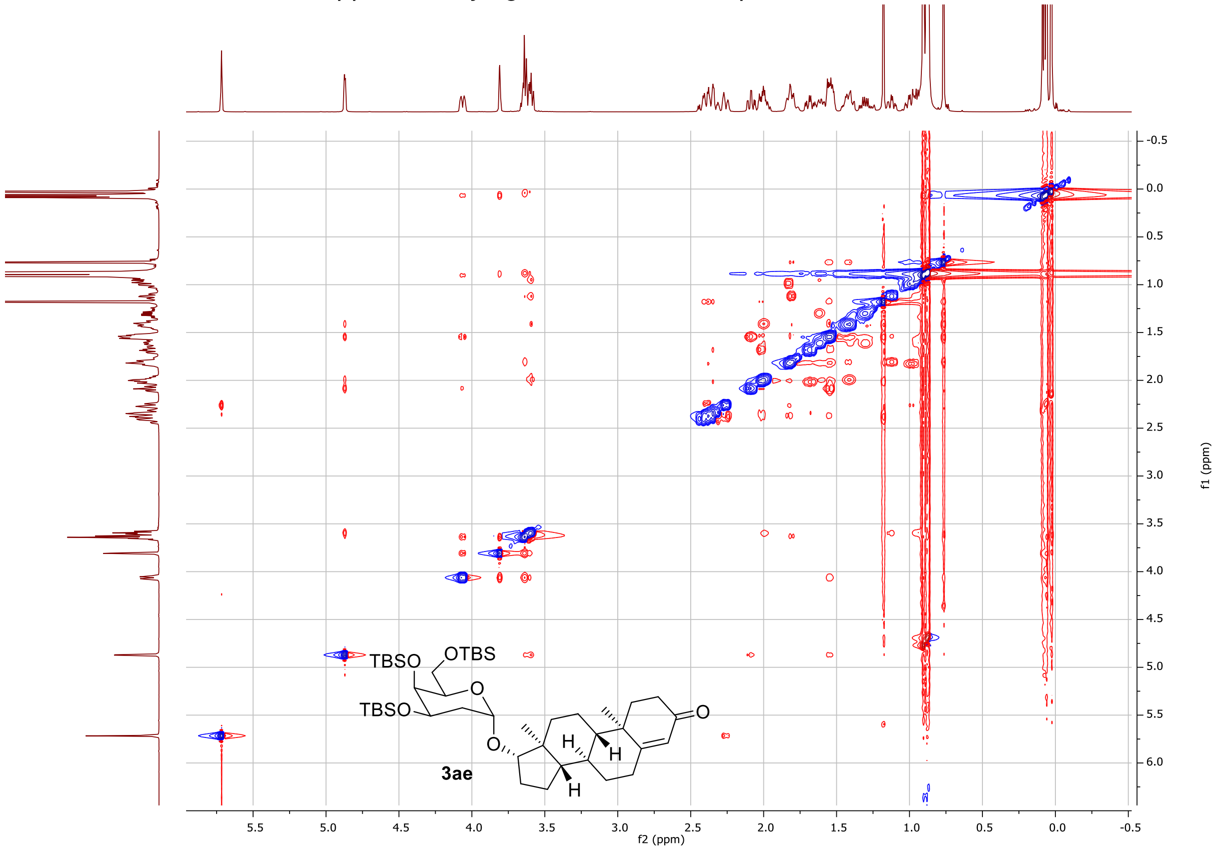

Supplementary figure S300: NOESY spectra for **3ae**

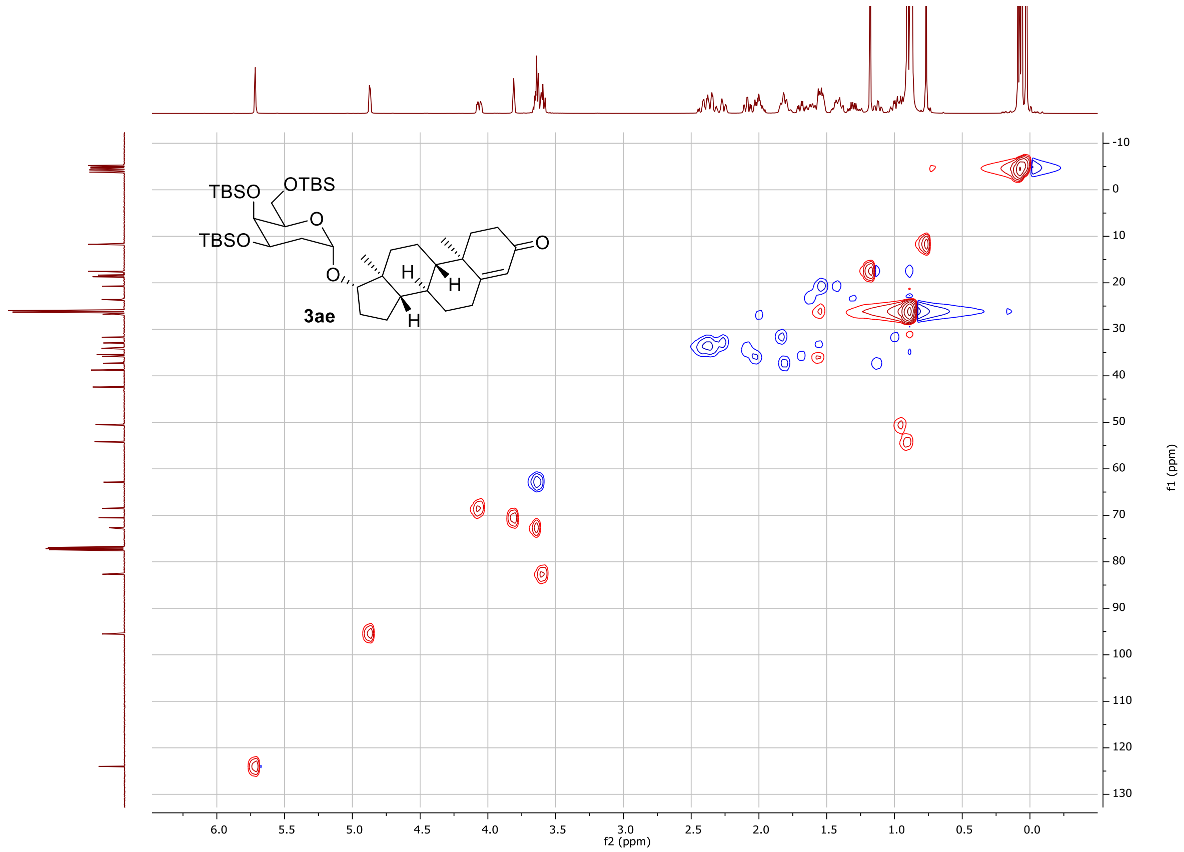

Supplementary figure S301: HSQC spectra for **3ae**

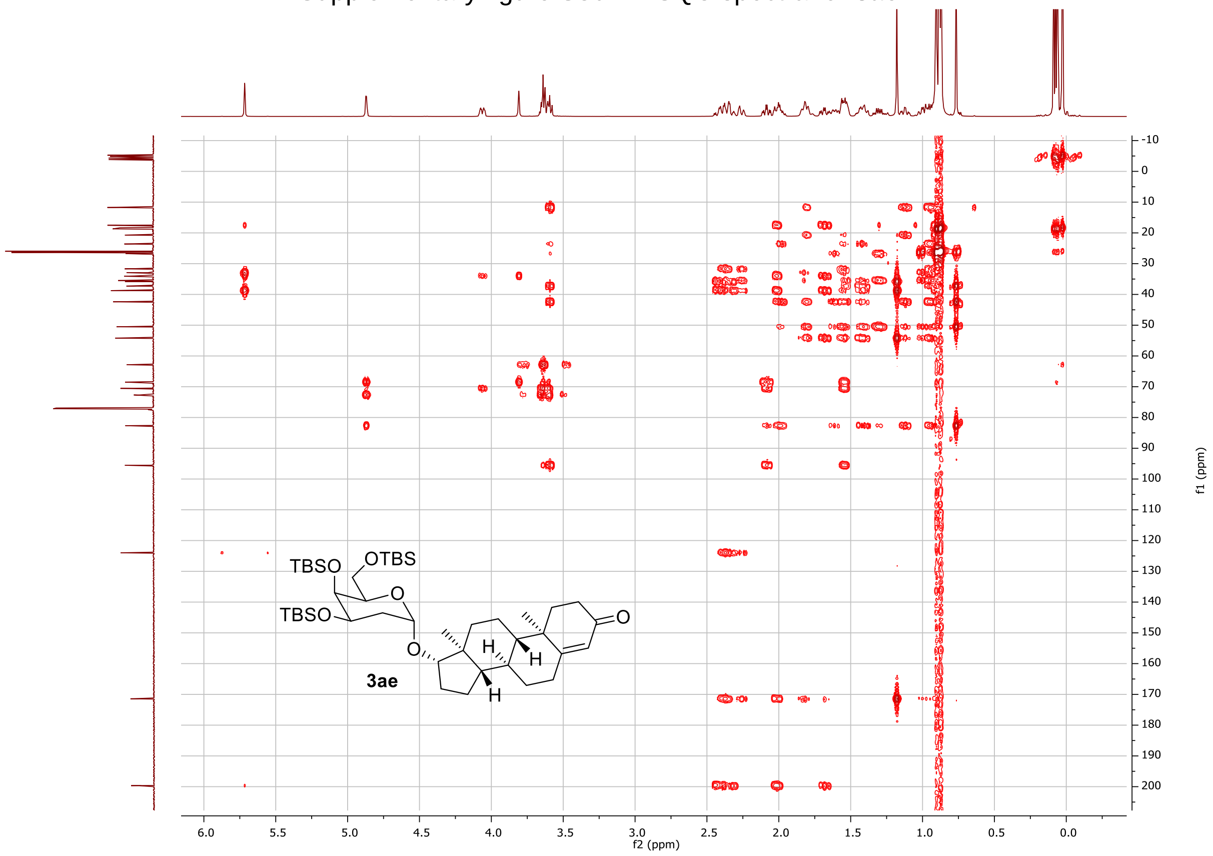

Supplementary figure S302: HMBC spectra for **3ae**

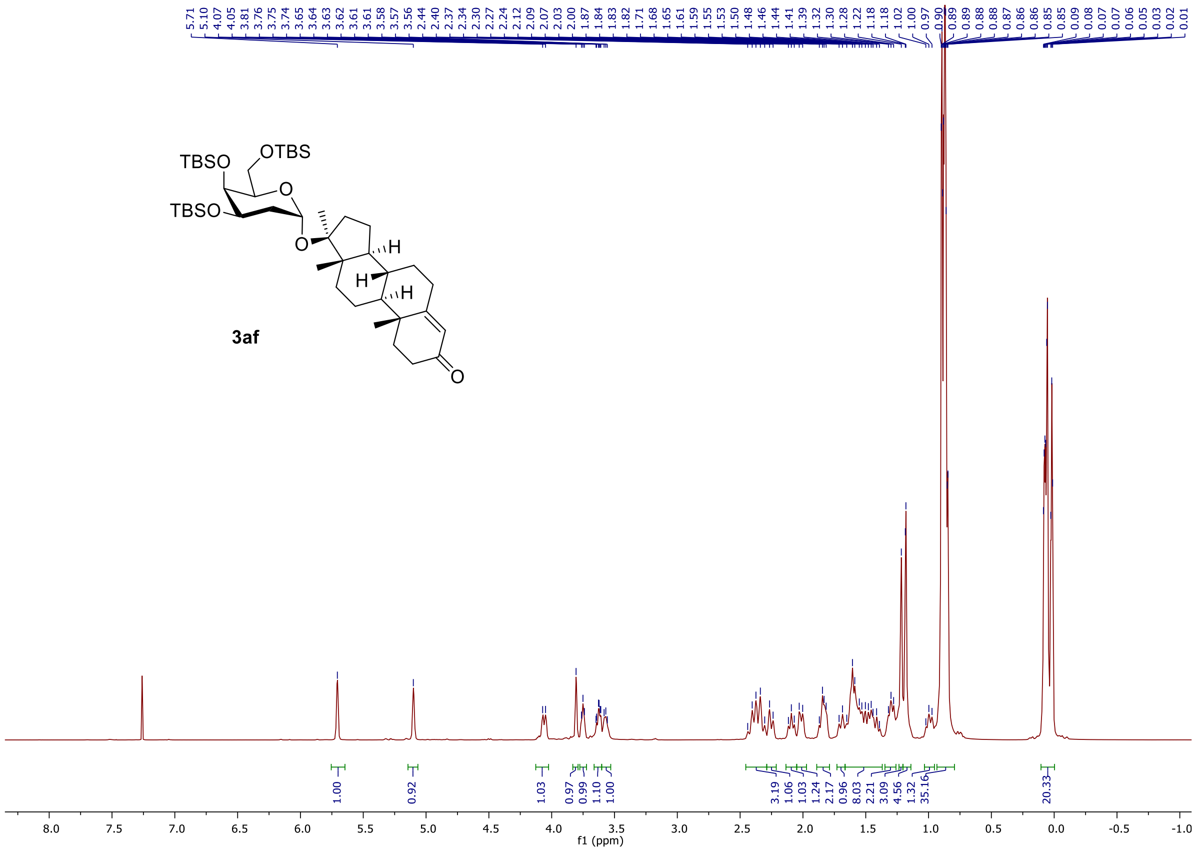

Supplementary figure S303: ^{1}H spectra for **3af**

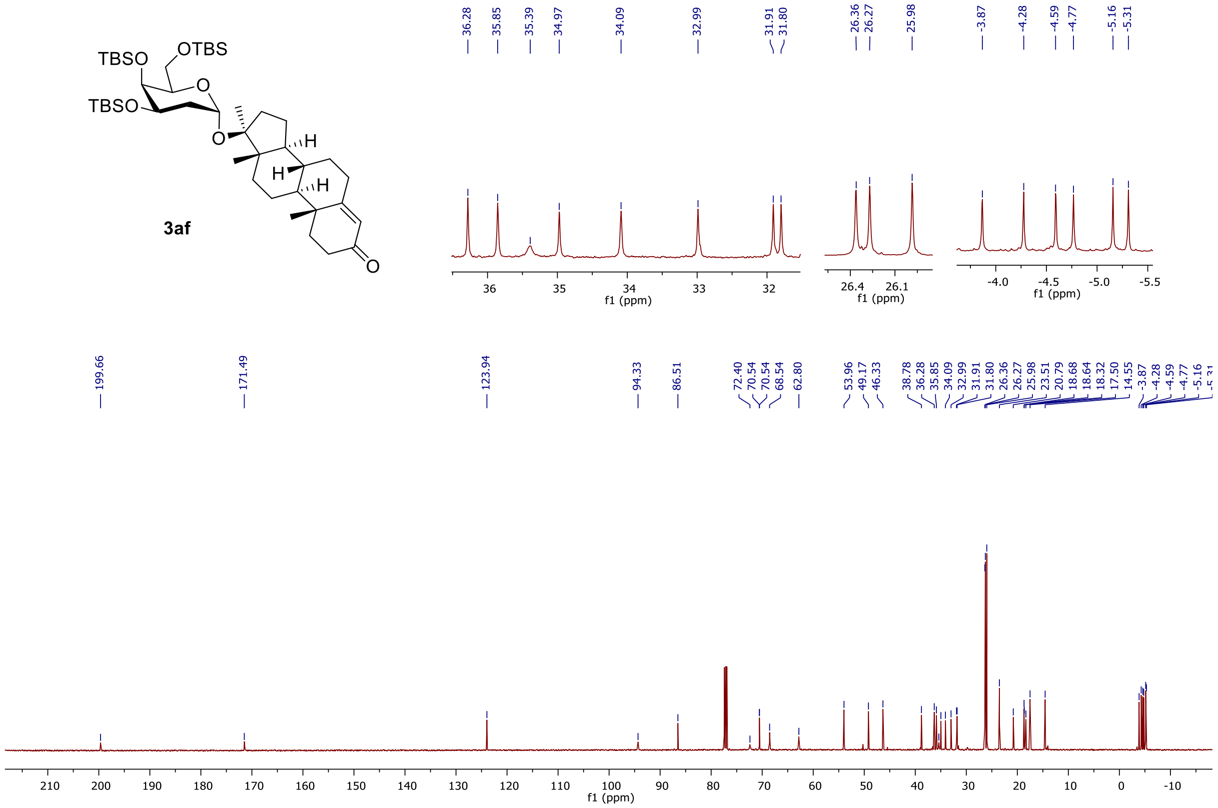

Supplementary figure S304: ^{13}C spectra for **3af**

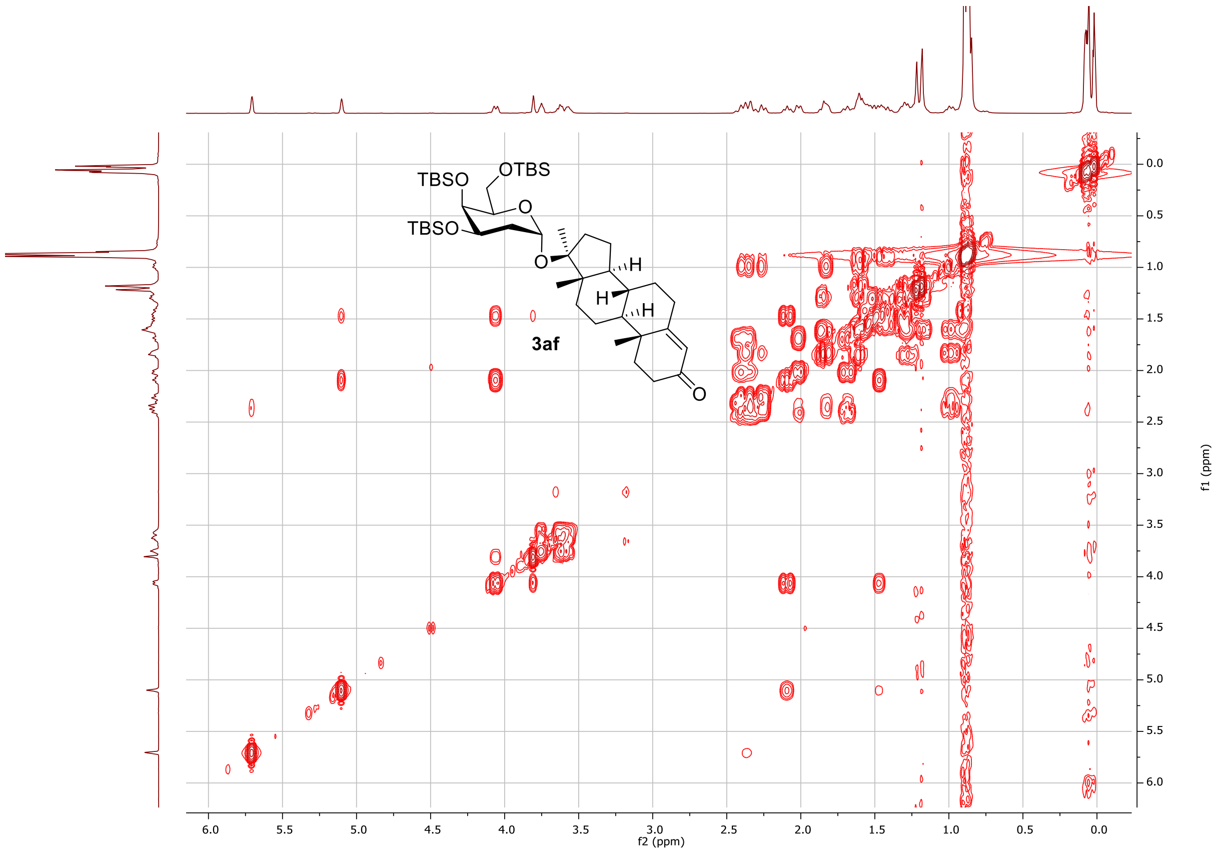

Supplementary figure S305: COSY spectra for **3af**

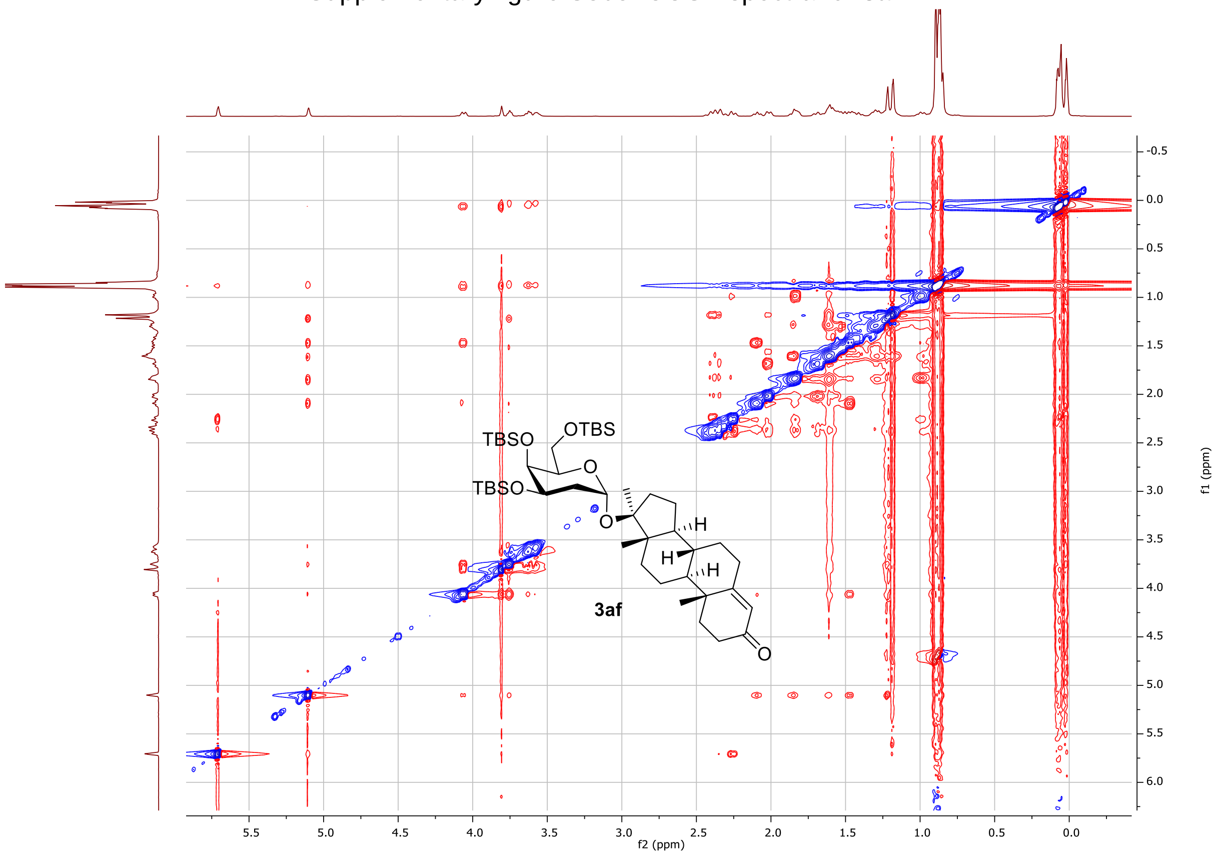

Supplementary figure S306: NOESY spectra for **3af**

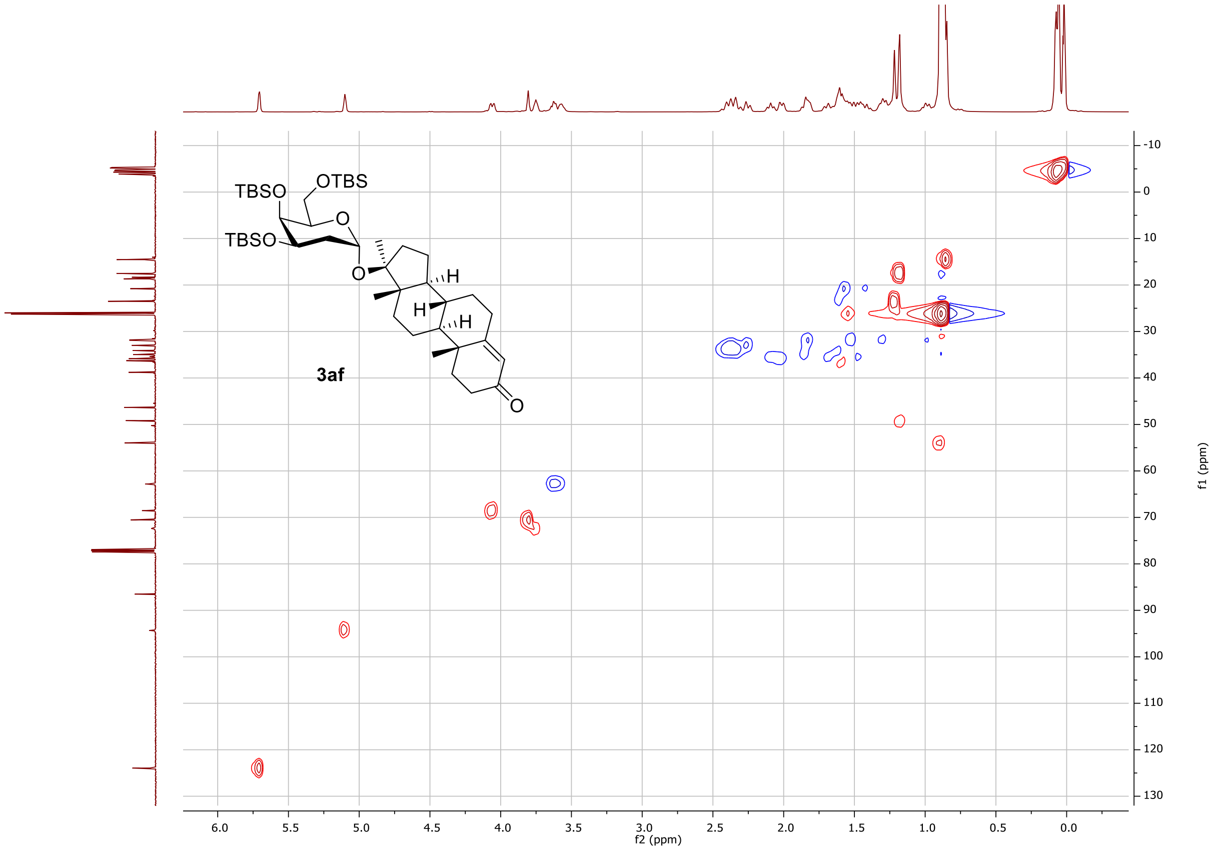

Supplementary figure S307: HSQC spectra for **3af**

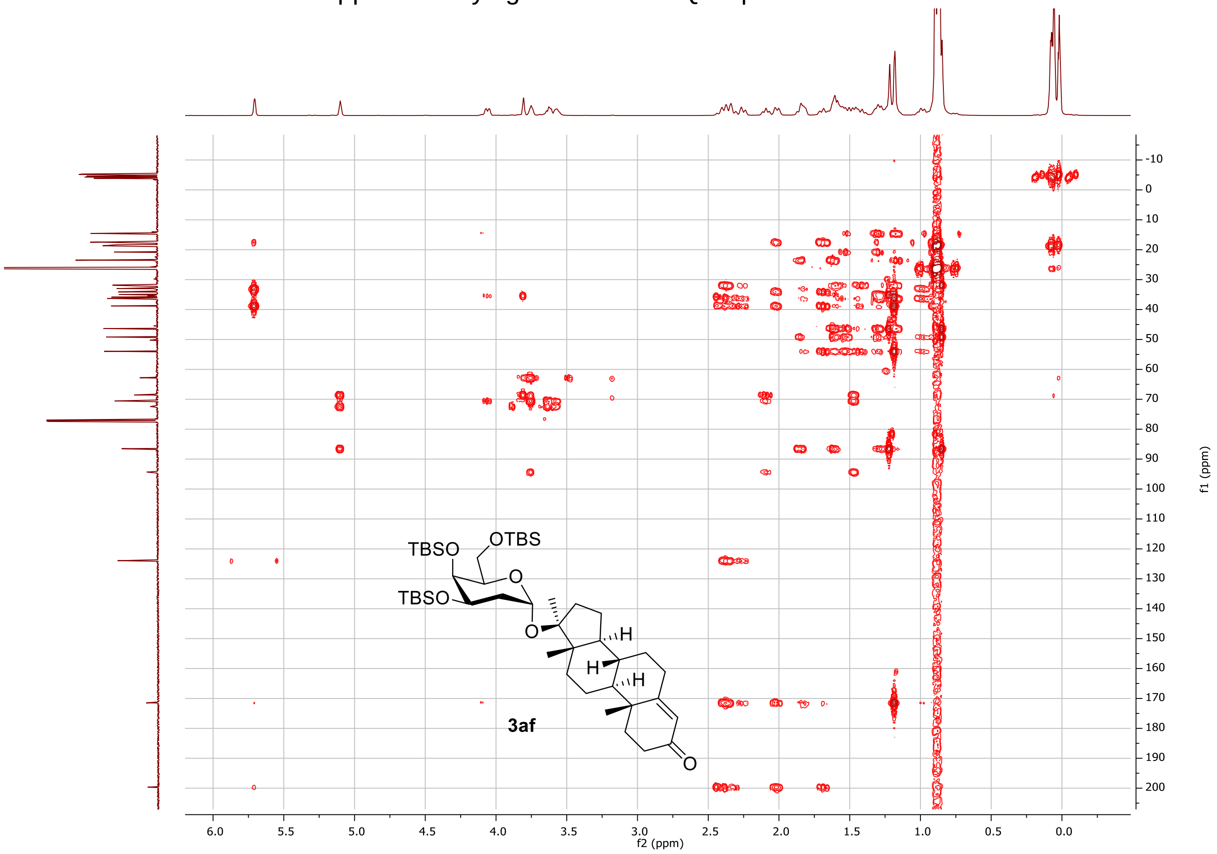

Supplementary figure S308: HMBC spectra for **3af**

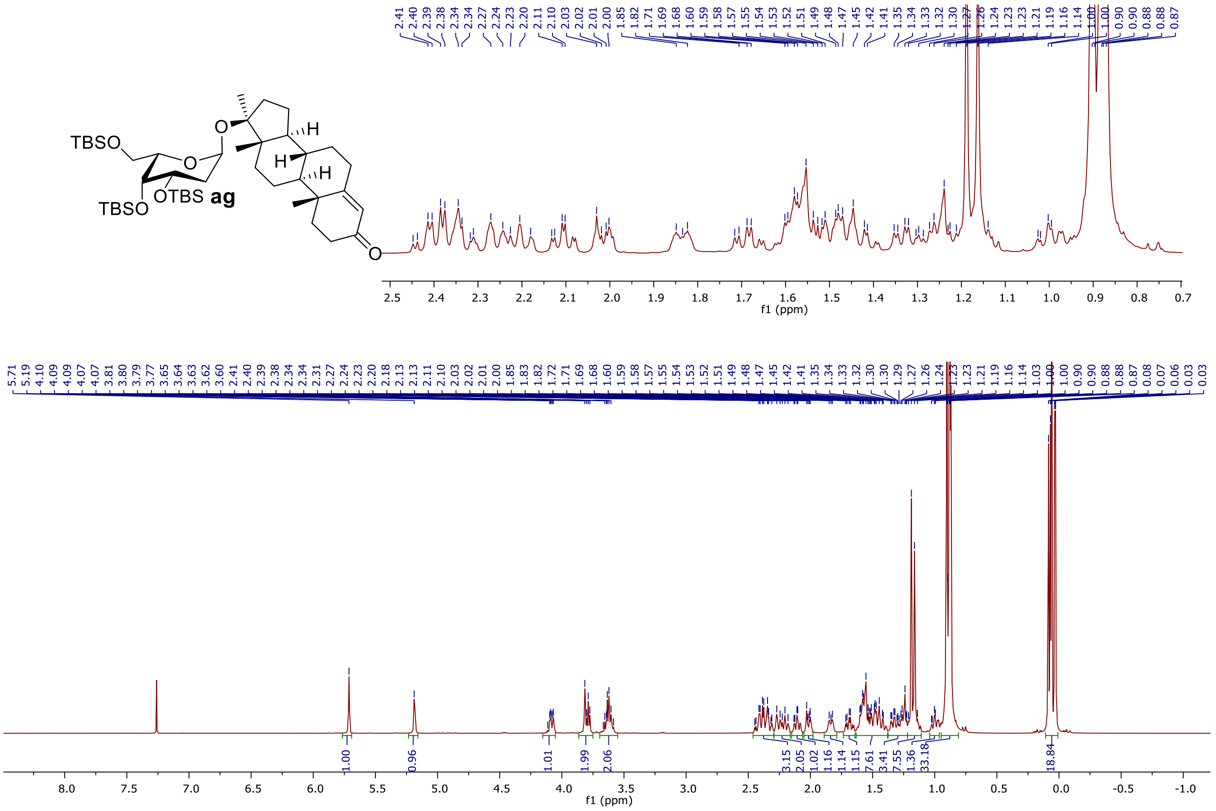

Supplementary figure S309: ^{1}H spectra for **3ag**

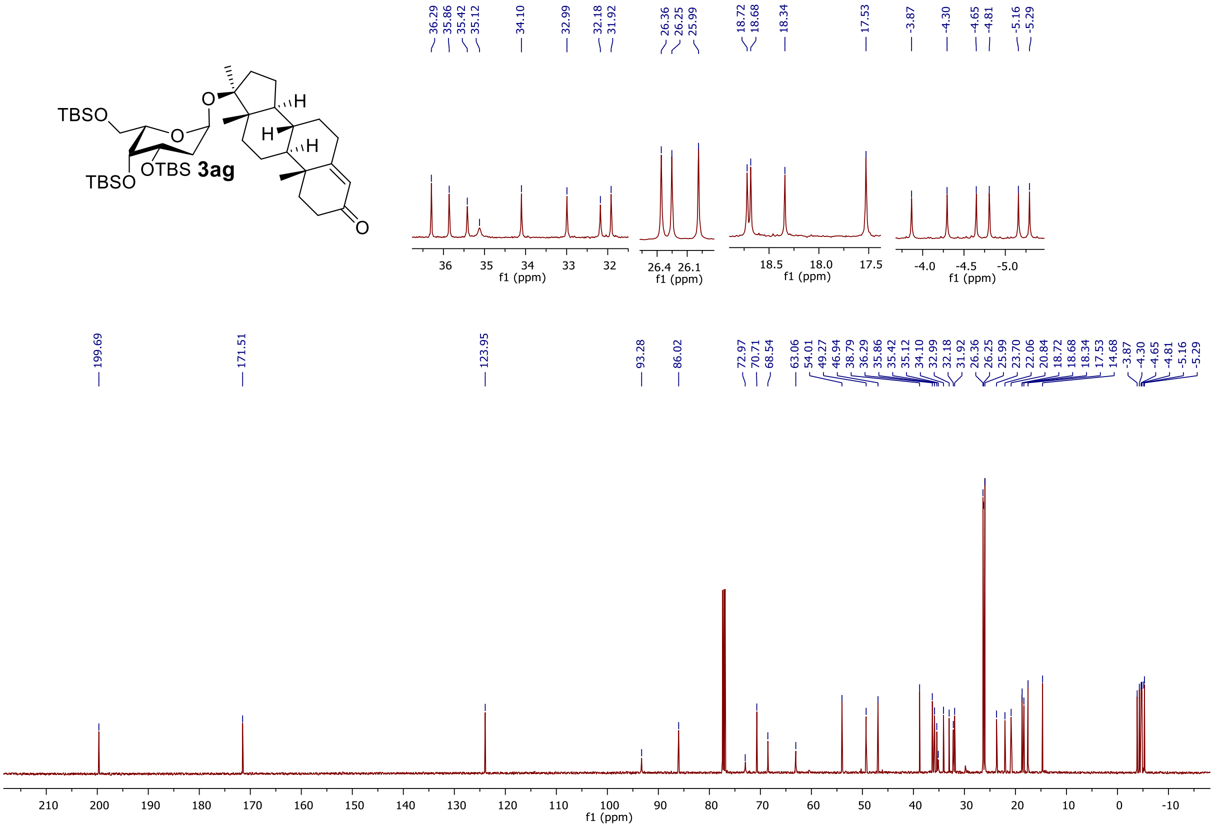

Supplementary figure S310: ^{13}C spectra for **3ag**

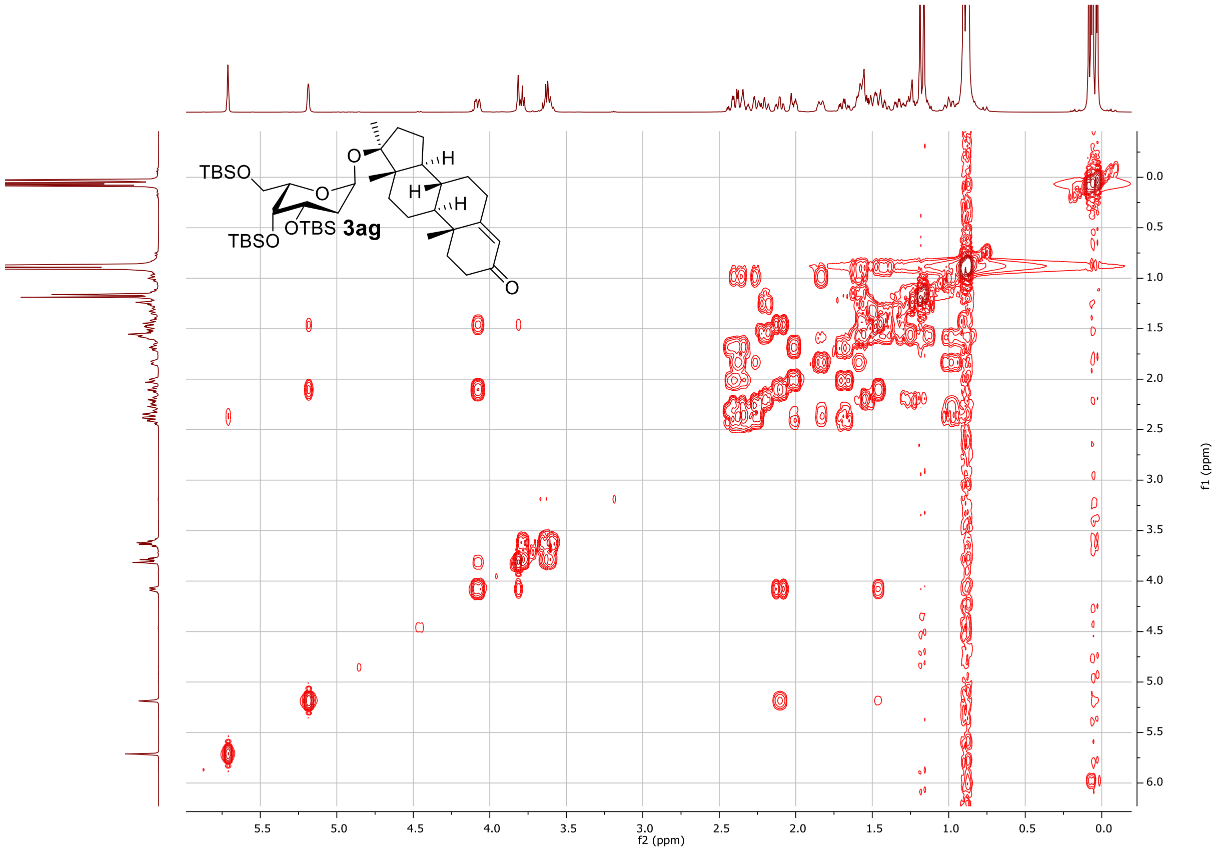

Supplementary figure S311: COSY spectra for **3ag**

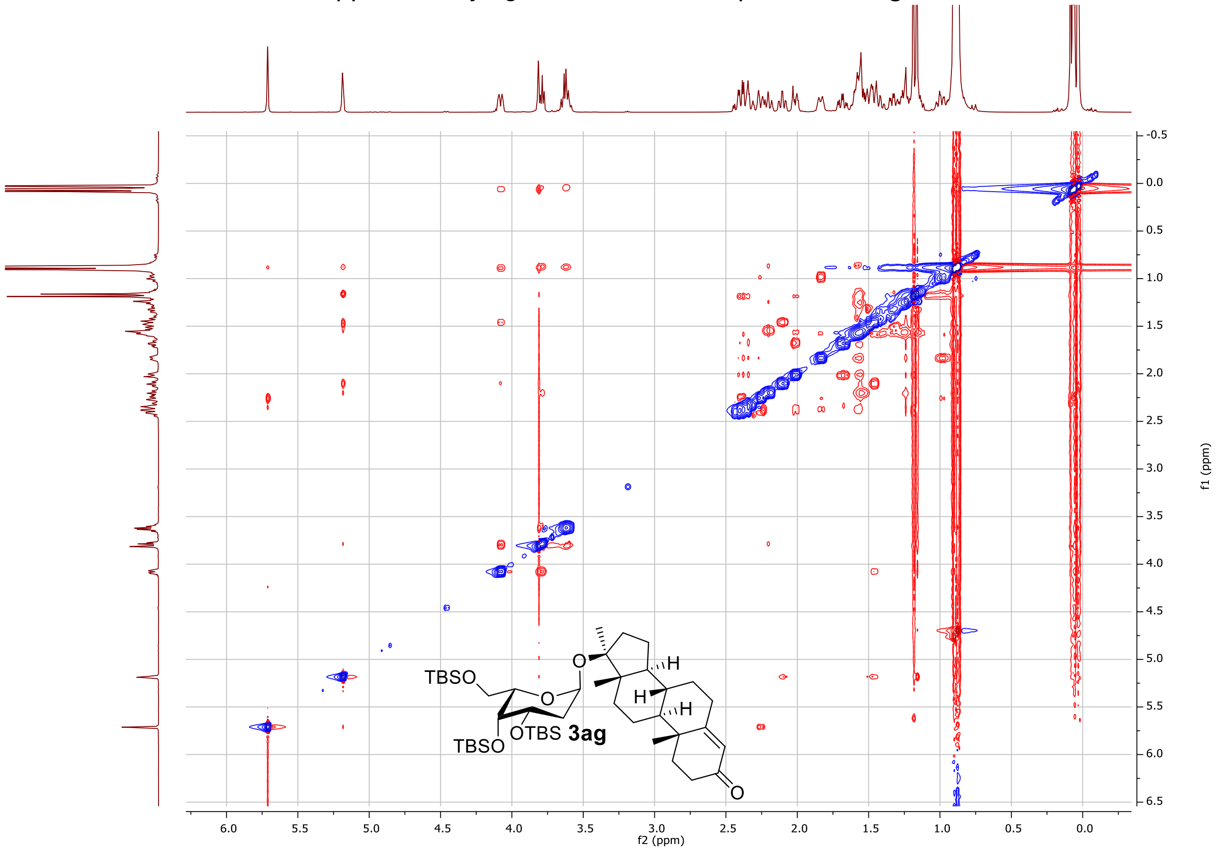

Supplementary figure S312: NOESY spectra for **3ag**

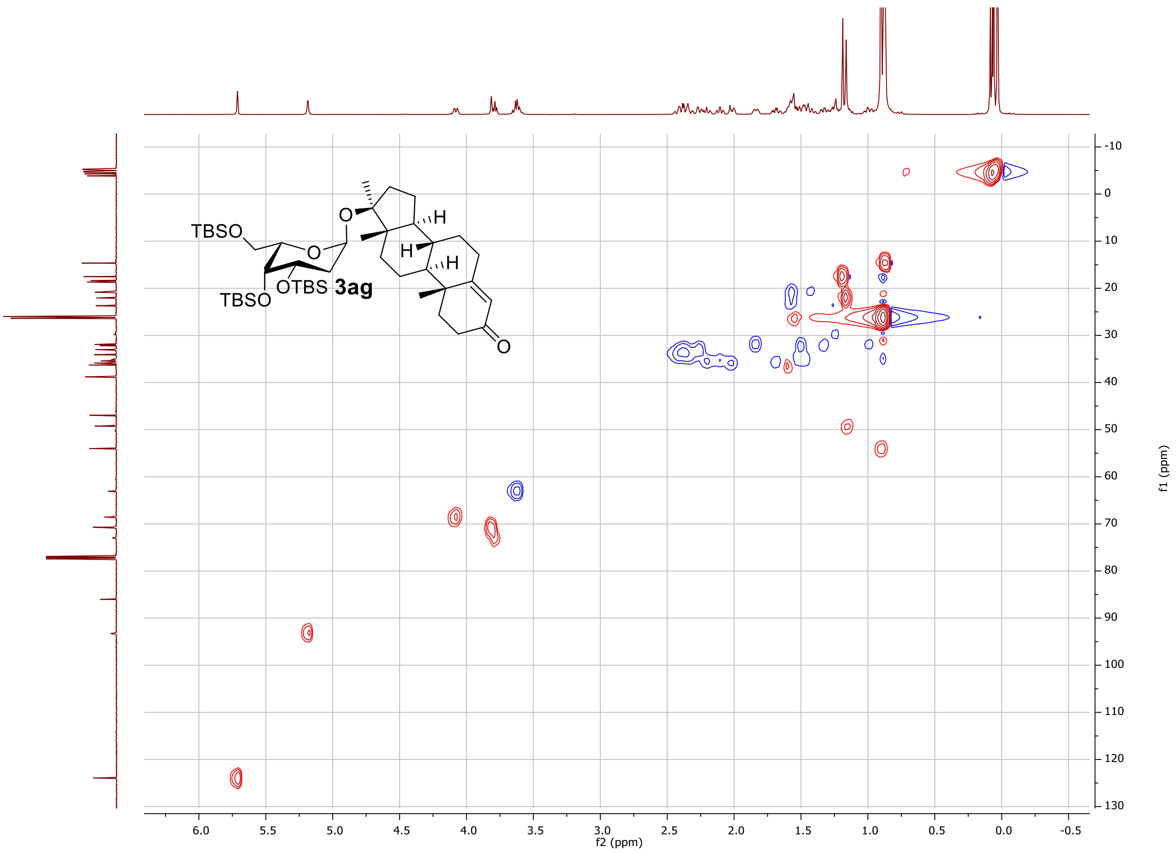

Supplementary figure S313: HSQC spectra for **3ag**

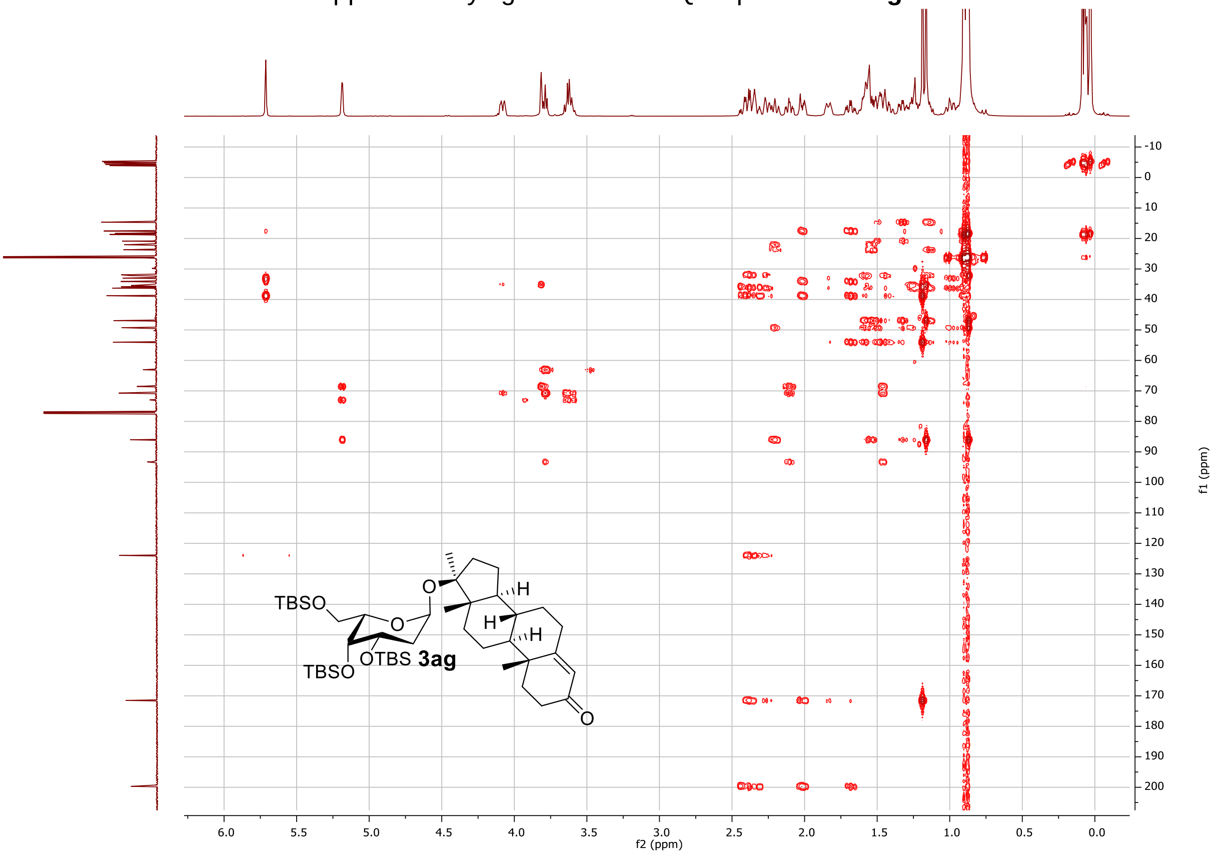

Supplementary figure S314: HMBC spectra for **3ag**

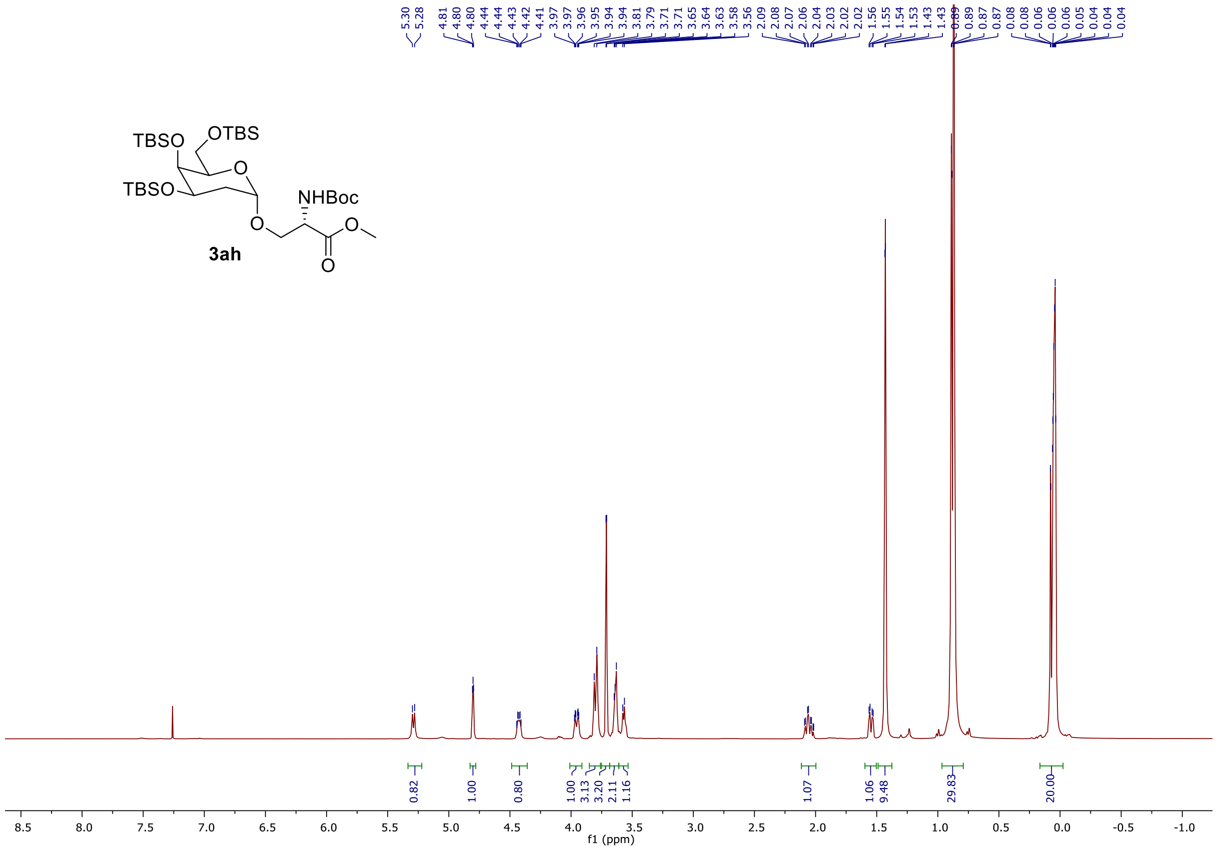

Supplementary figure S315: ^{1}H spectra for **3ah**

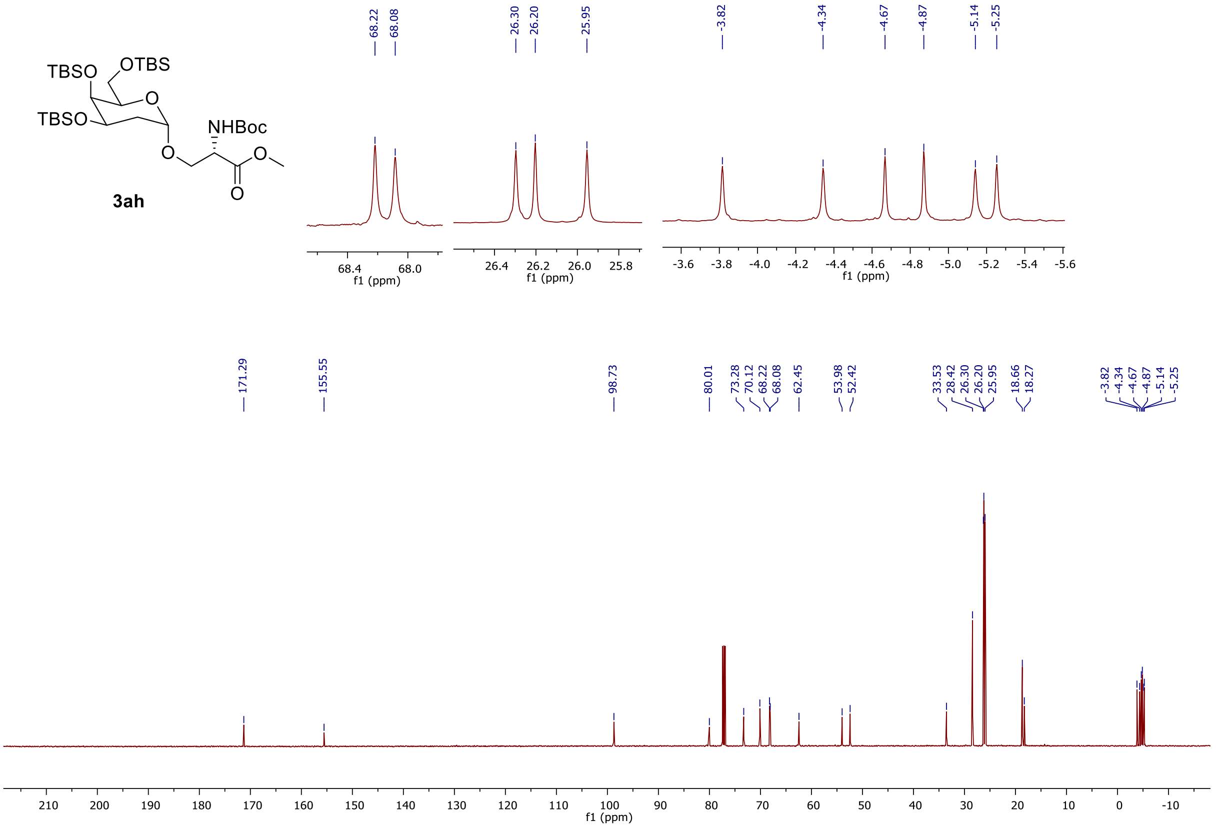

Supplementary figure S316: ^{13}C spectra for **3ah**

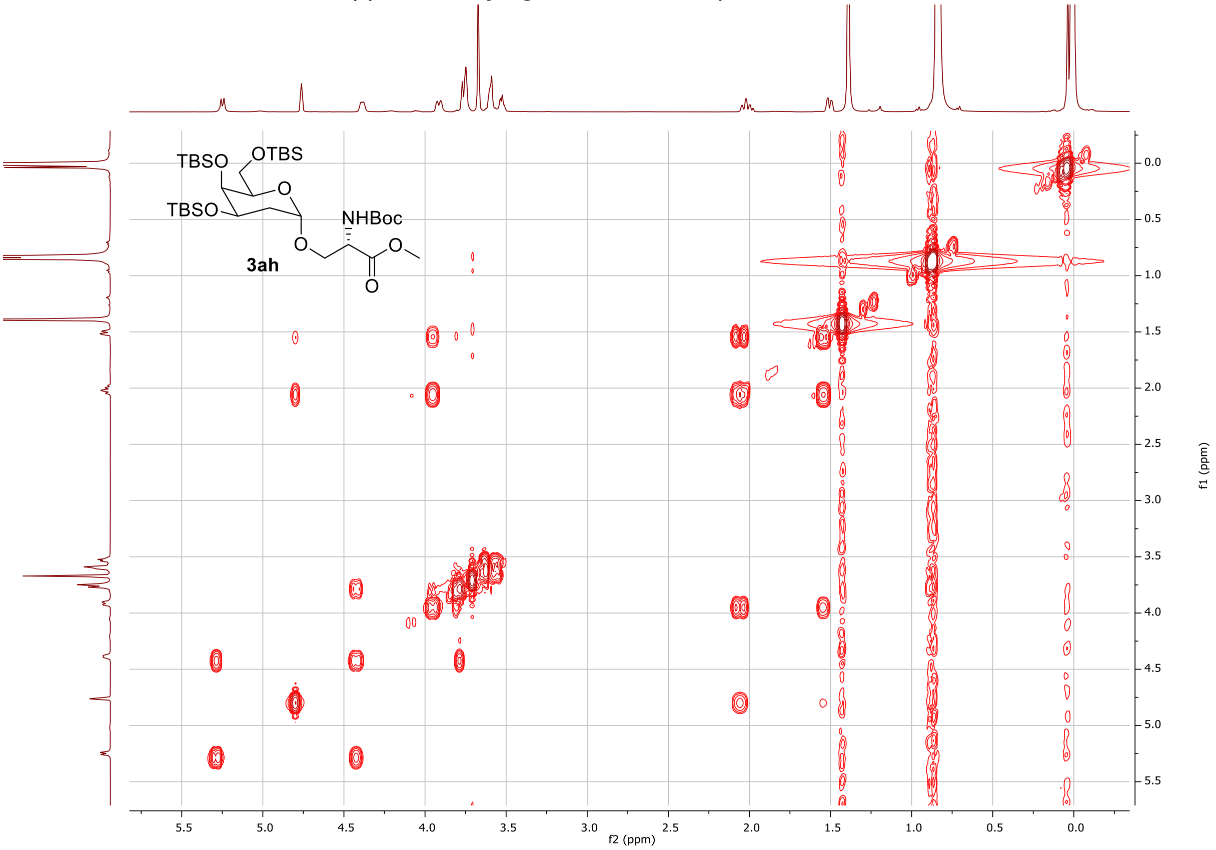

Supplementary figure S317: COSY spectra for **3ah**

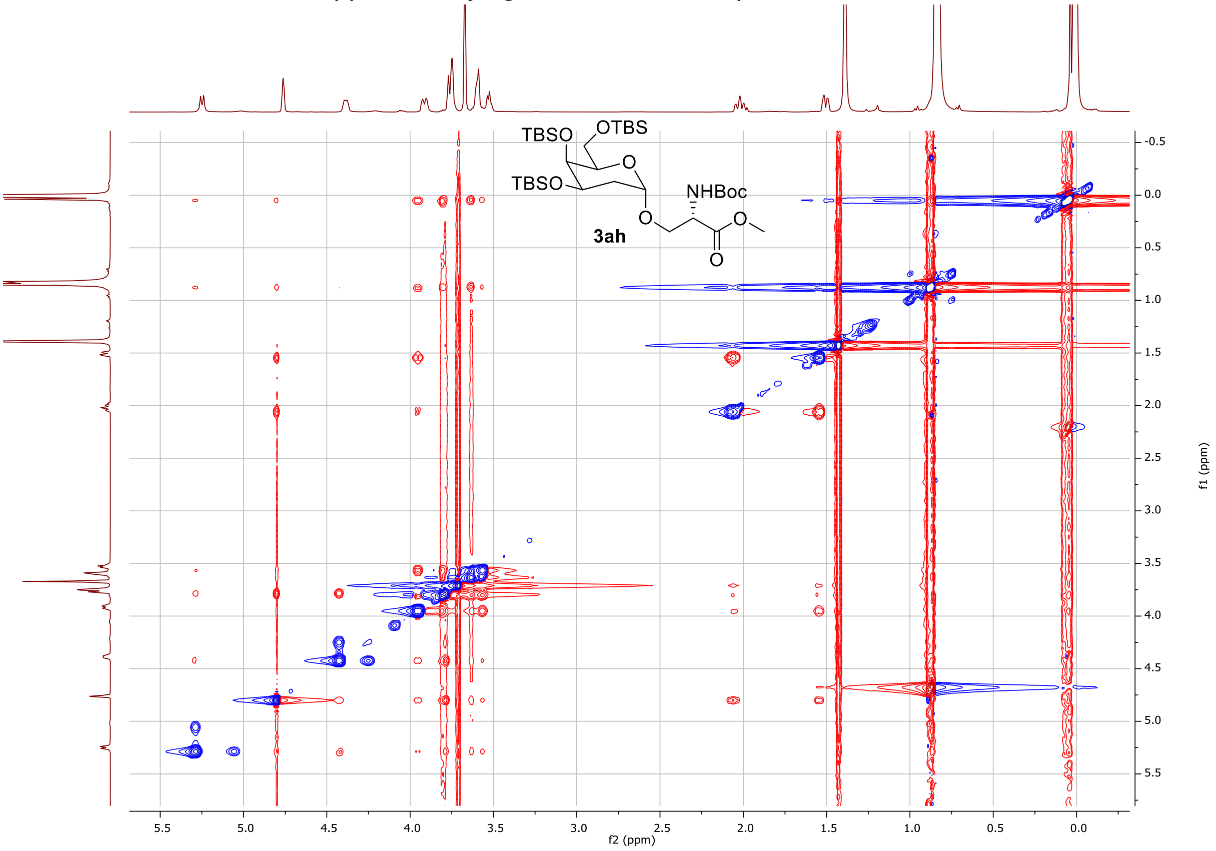

Supplementary figure S318: NOESY spectra for **3ah**

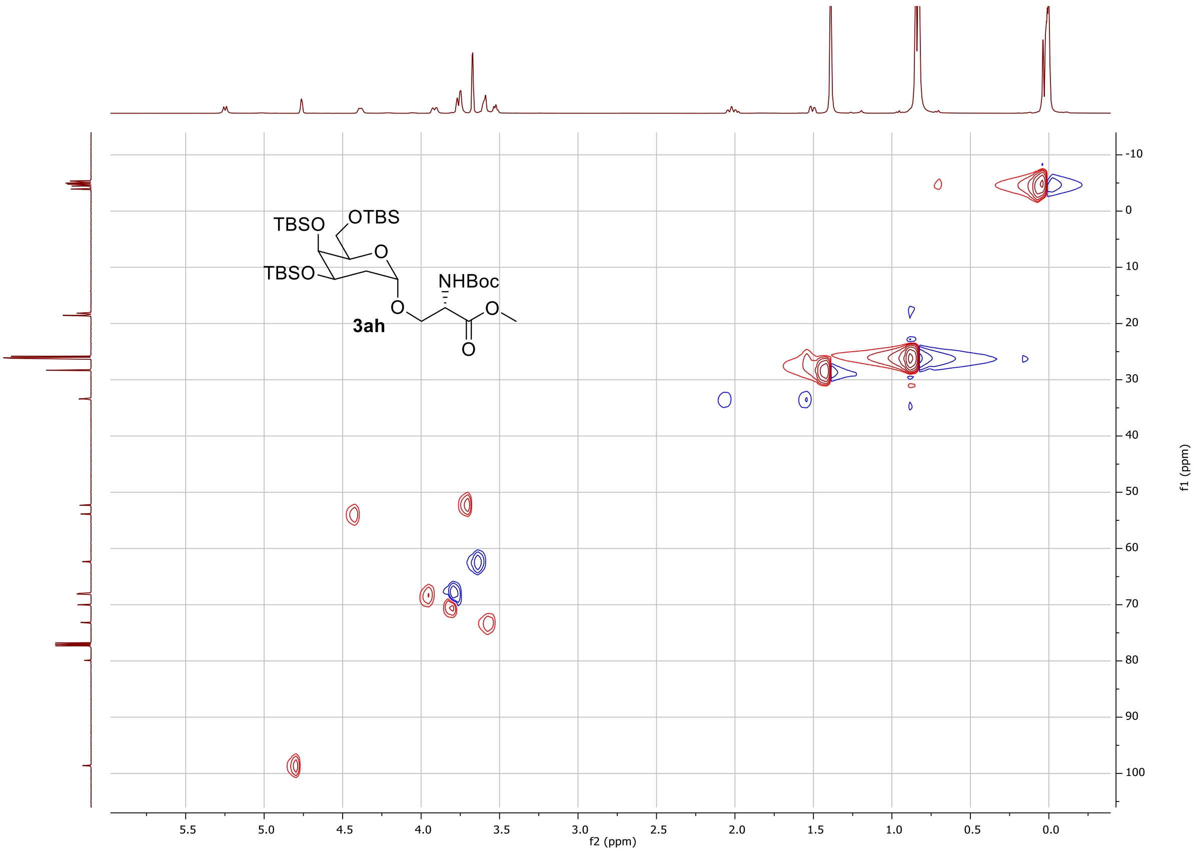

Supplementary figure S319: HSQC spectra for **3ah**

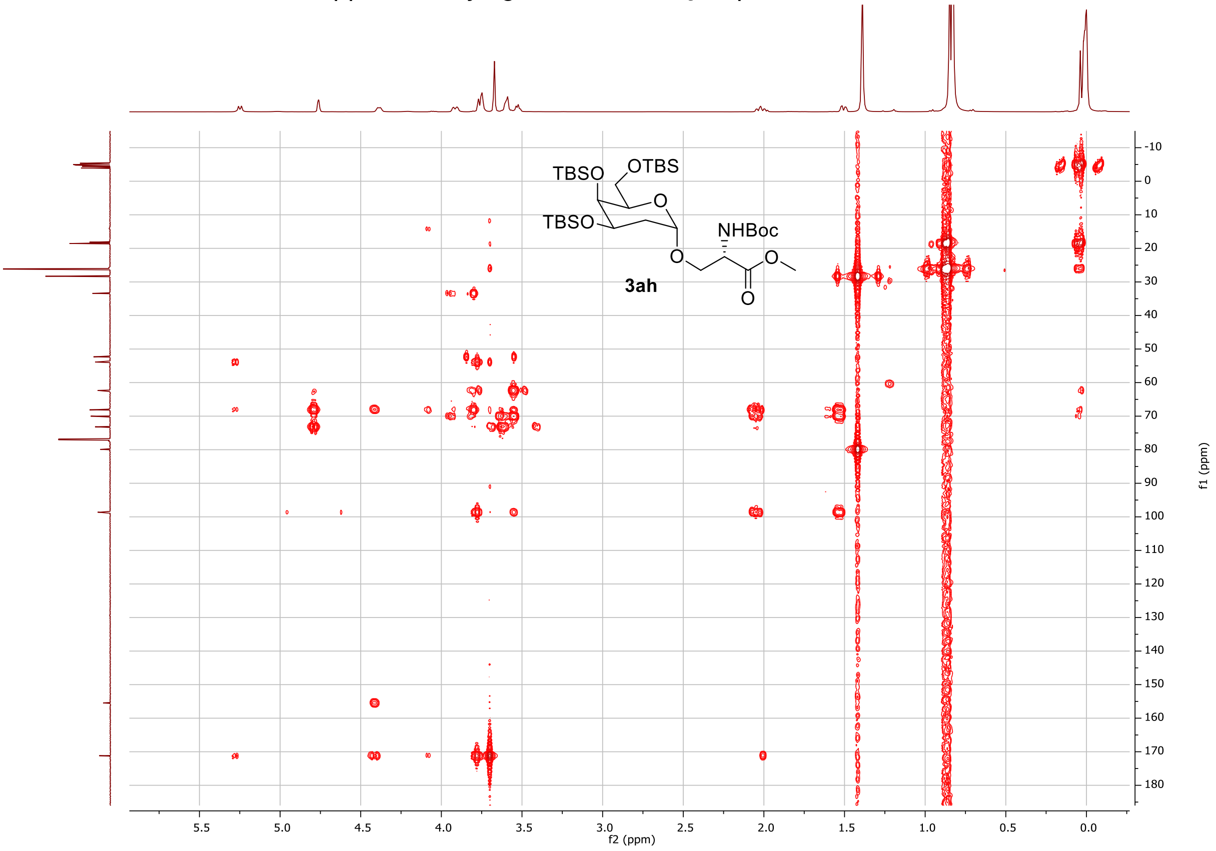

Supplementary figure S320: HMBC spectra for **3ah**

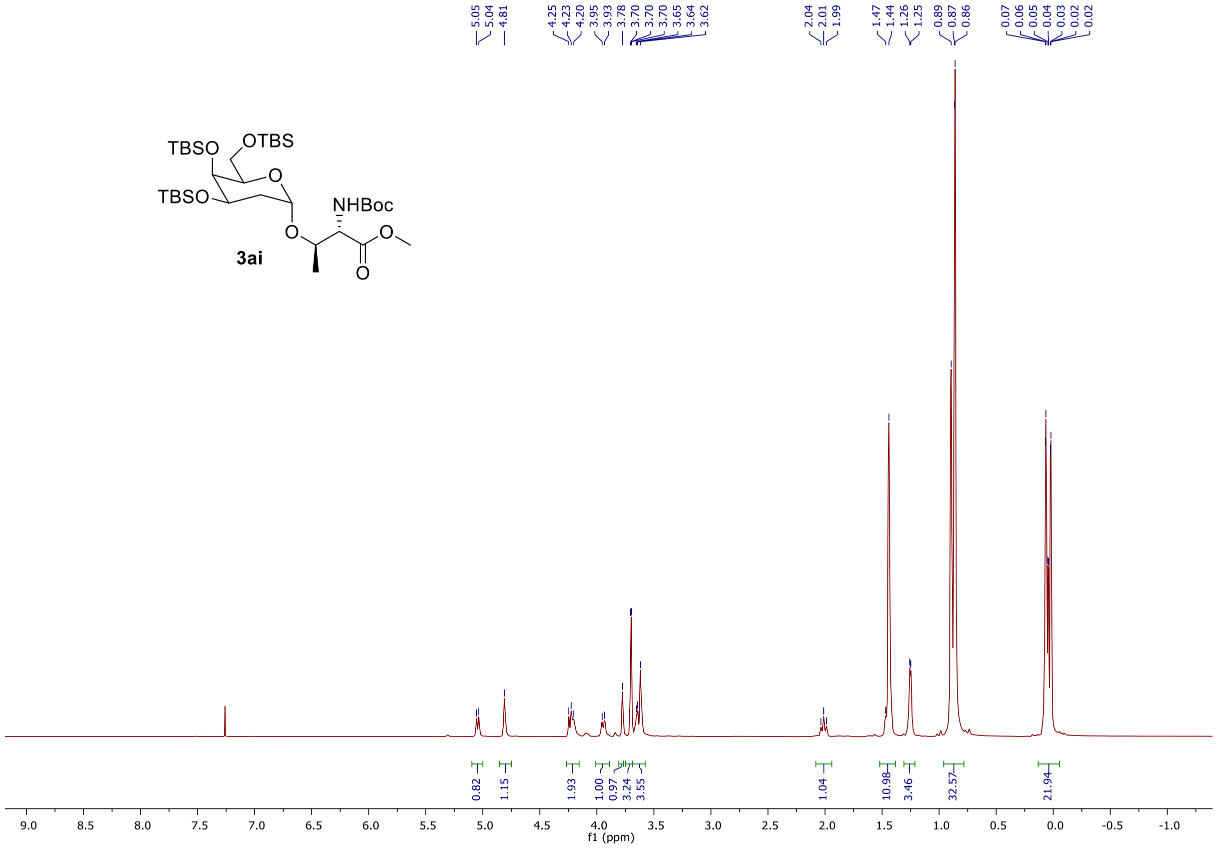

Supplementary figure S321: ^{1}H spectra for **3ai**

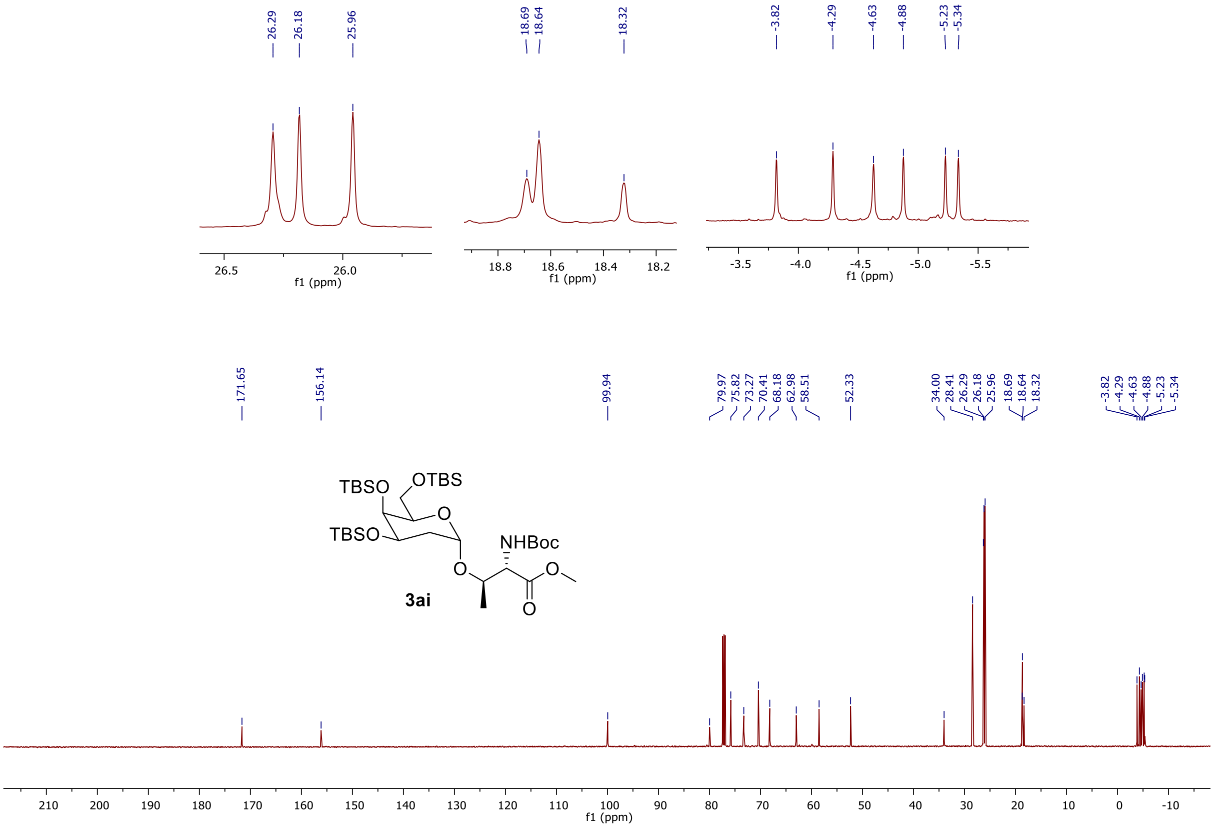

Supplementary figure S322: ^{13}C spectra for **3ai**

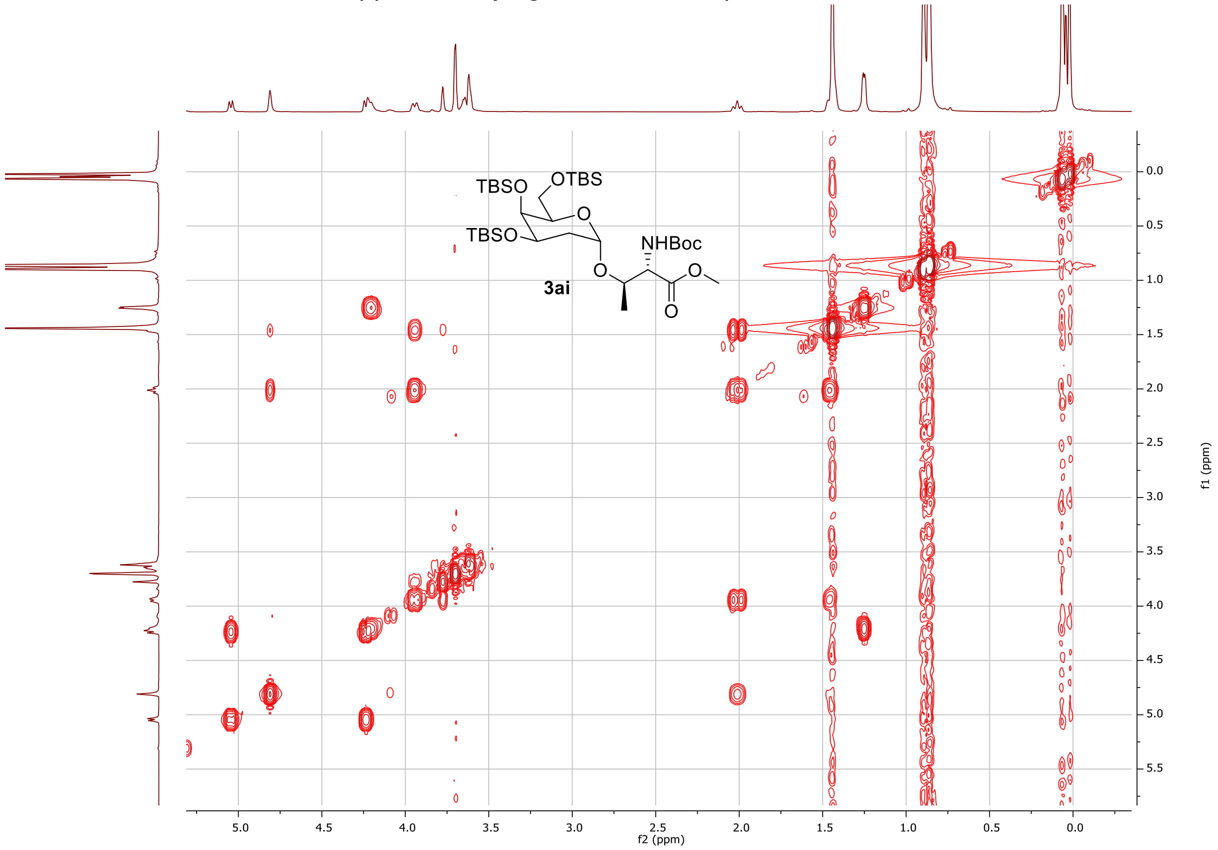

Supplementary figure S323: COSY spectra for **3ai**

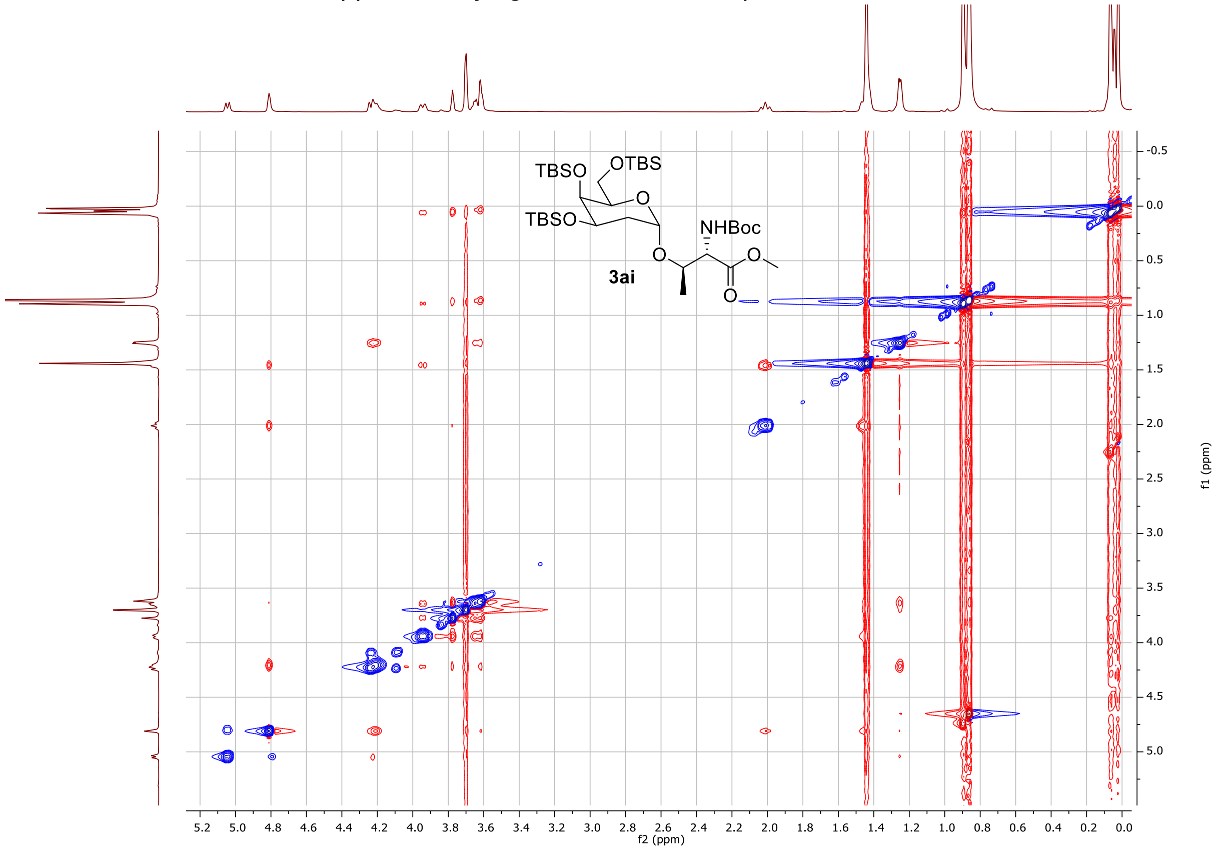

Supplementary figure S324: NOESY spectra for **3ai**

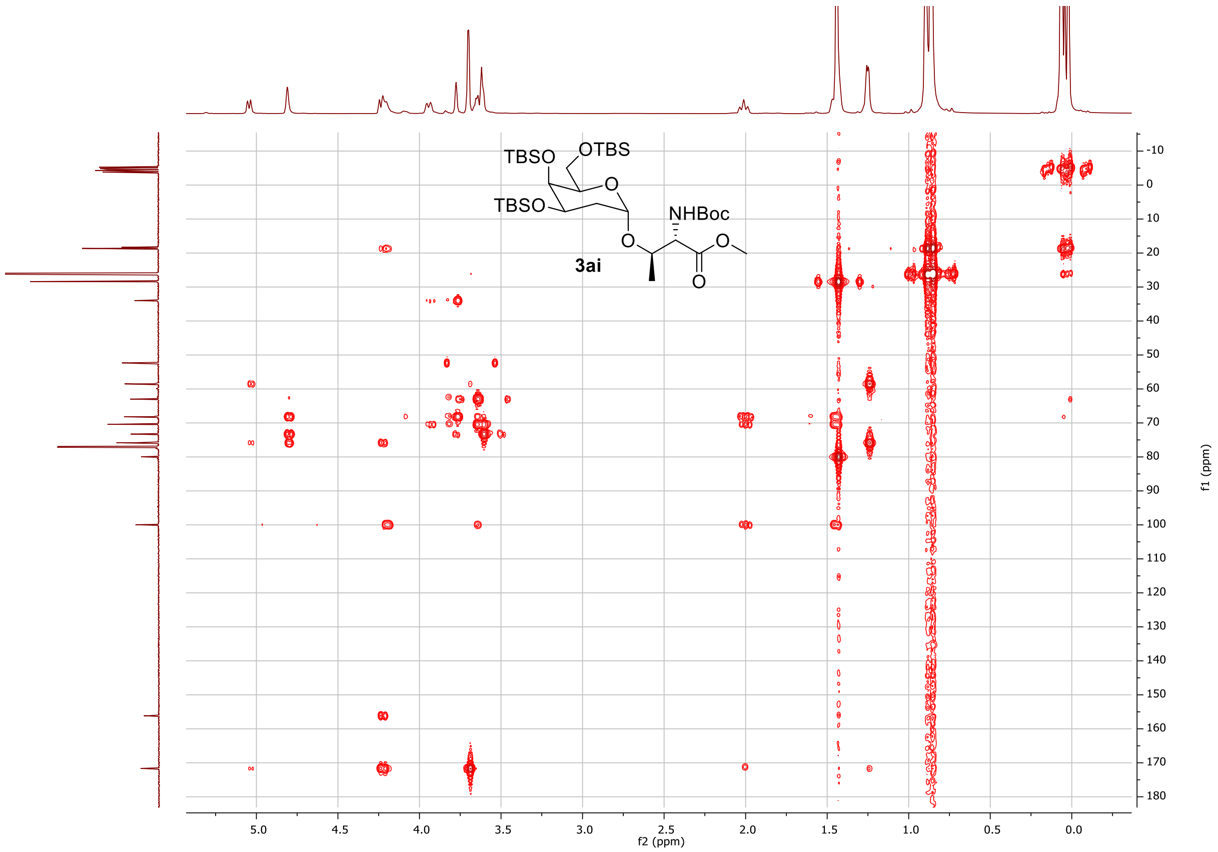

Supplementary figure S325: HMBC spectra for **3ai**

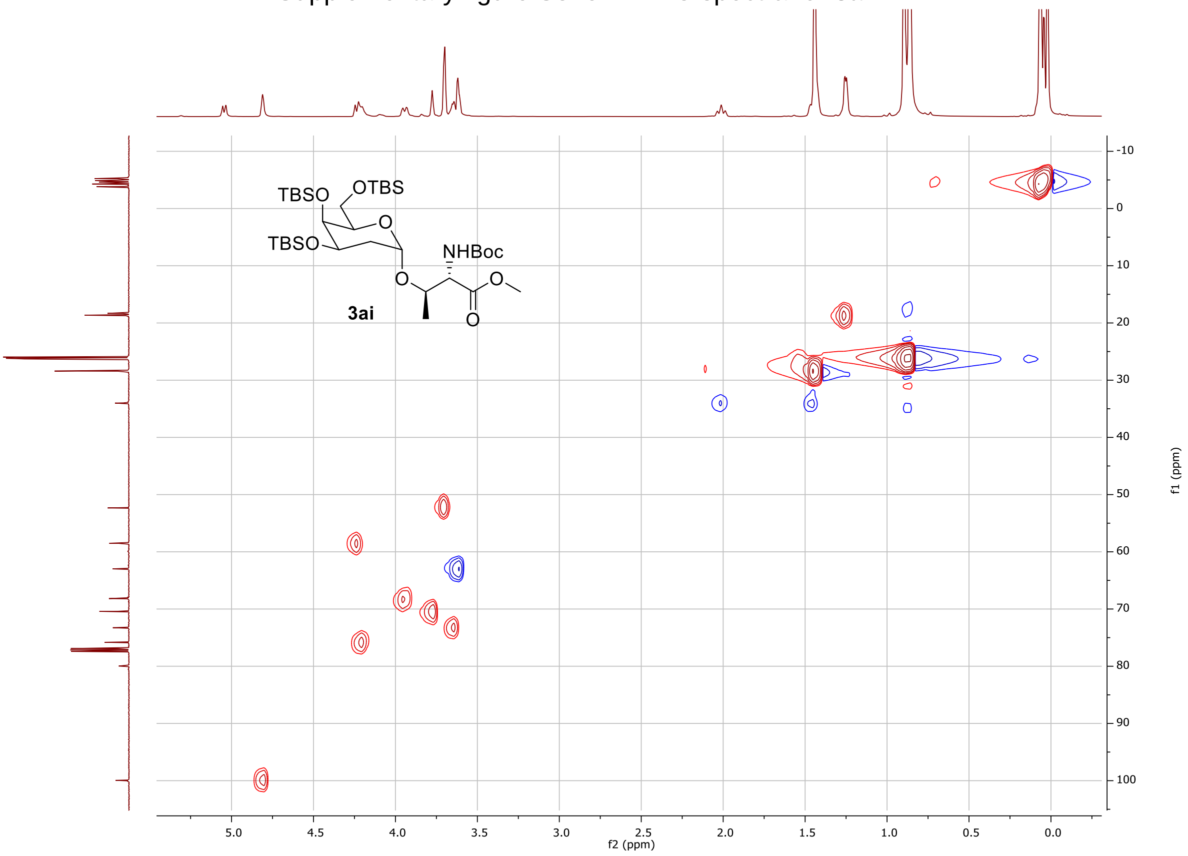

Supplementary figure S326: HSQC spectra for **3ai**

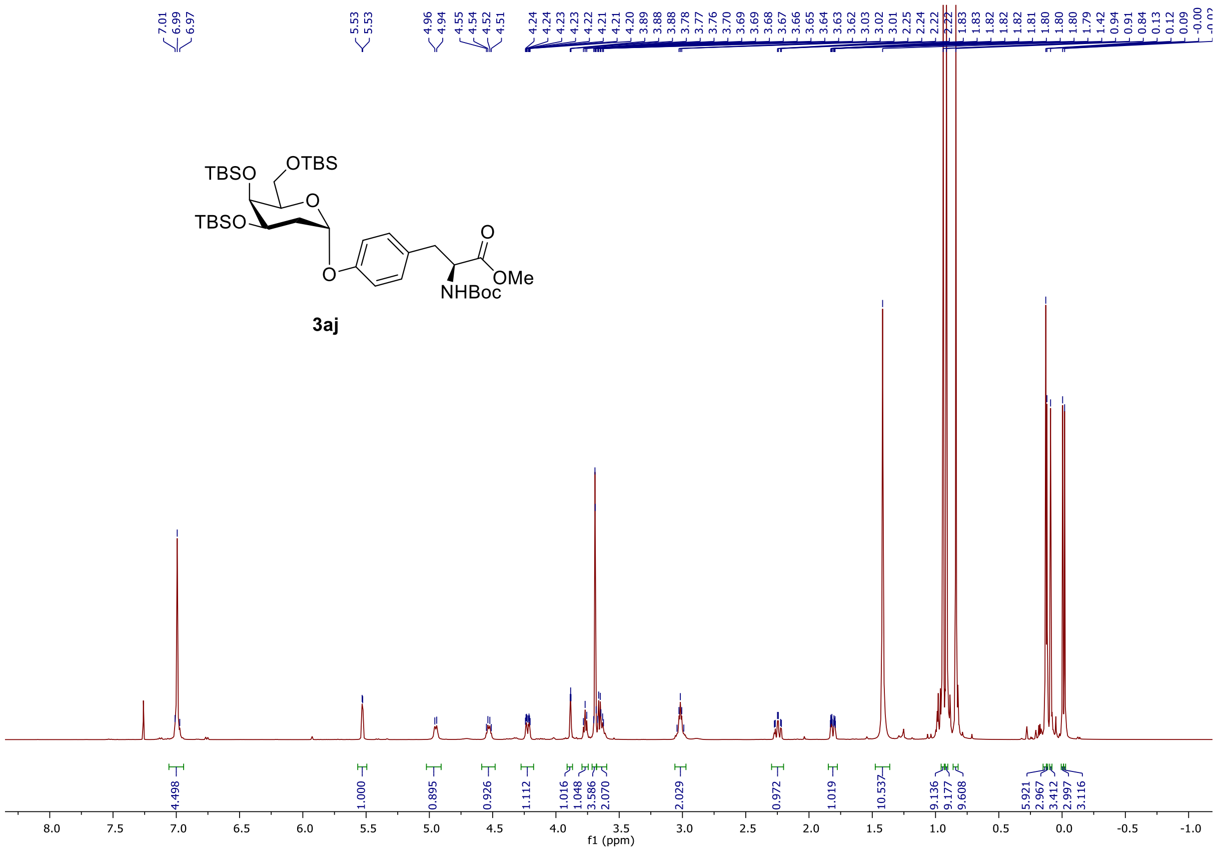

Supplementary figure S327: ^{1}H spectra for **3aj**

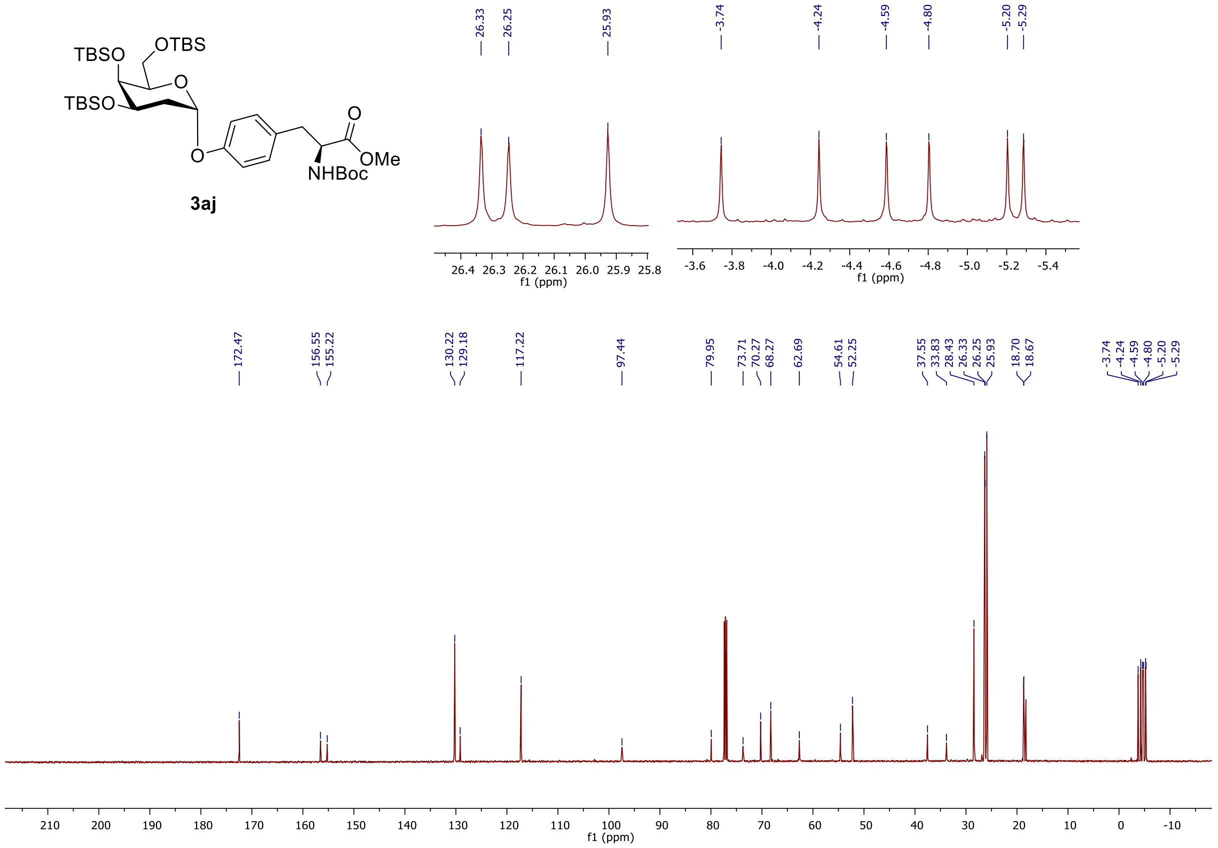

Supplementary figure S328: ^{13}C spectra for **3aj**

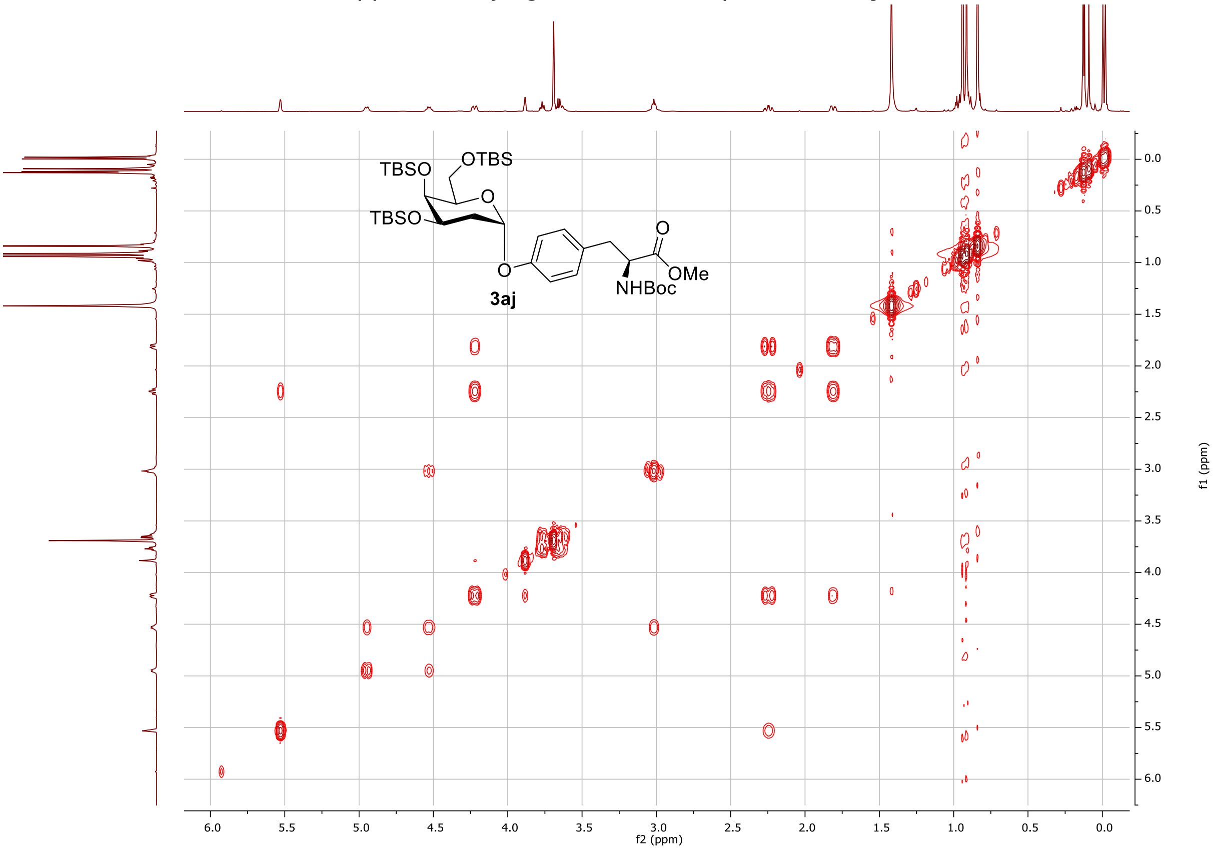

Supplementary figure S329: COSY spectra for **3aj**

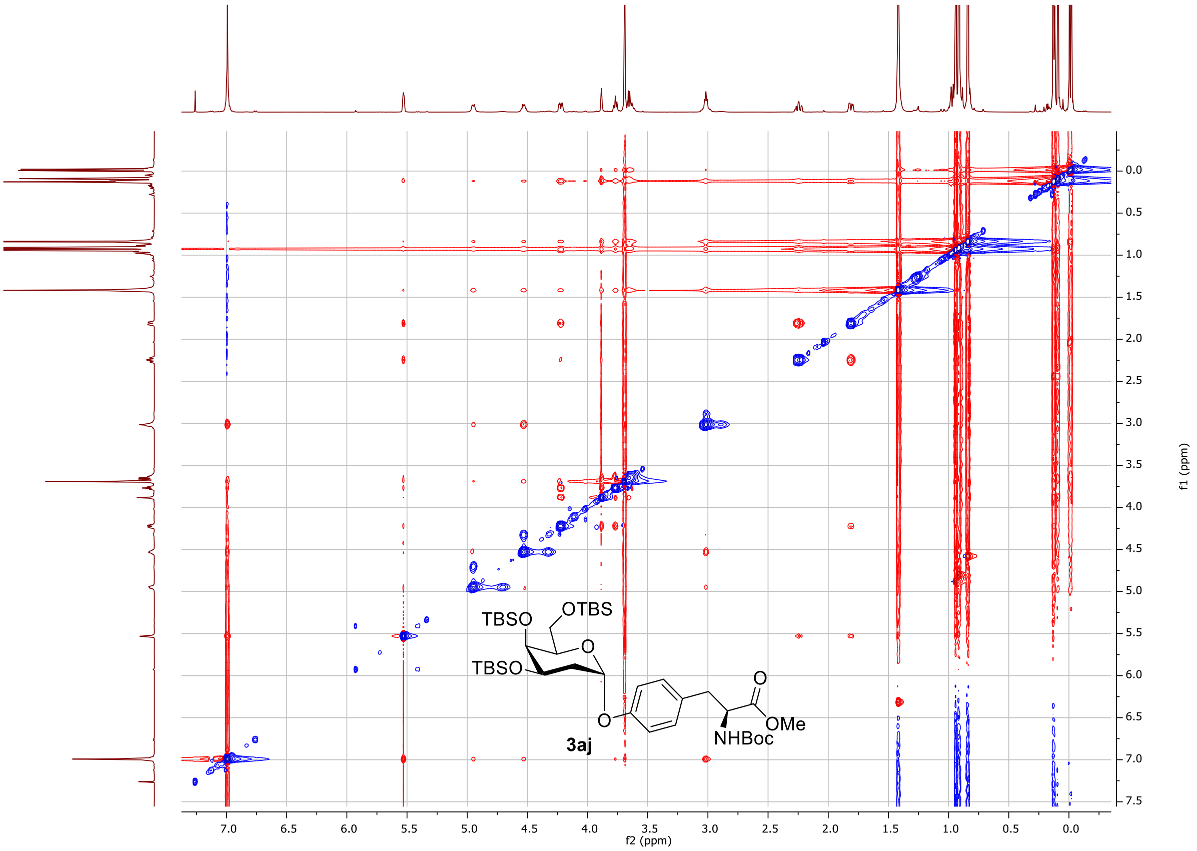

Supplementary figure S330: NOESY spectra for **3aj**

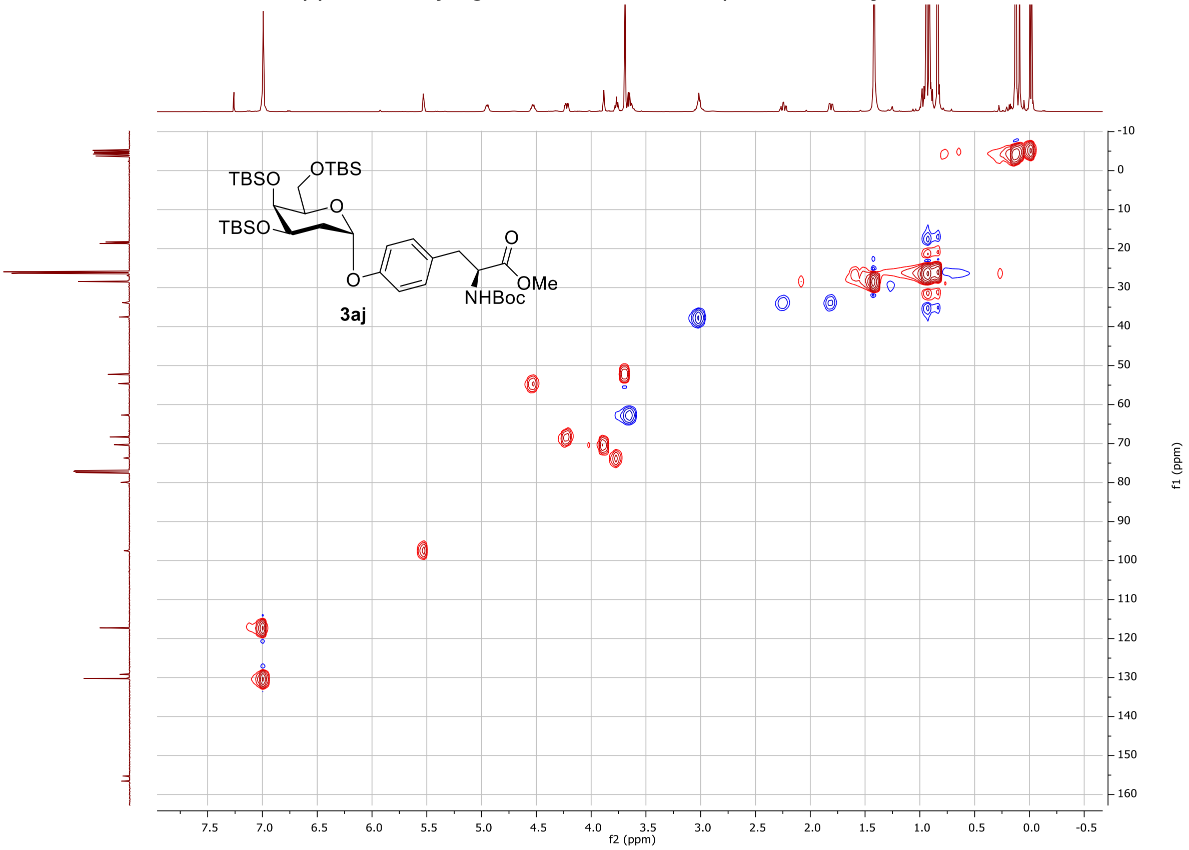

Supplementary figure S331: HSQC spectra for **3aj**

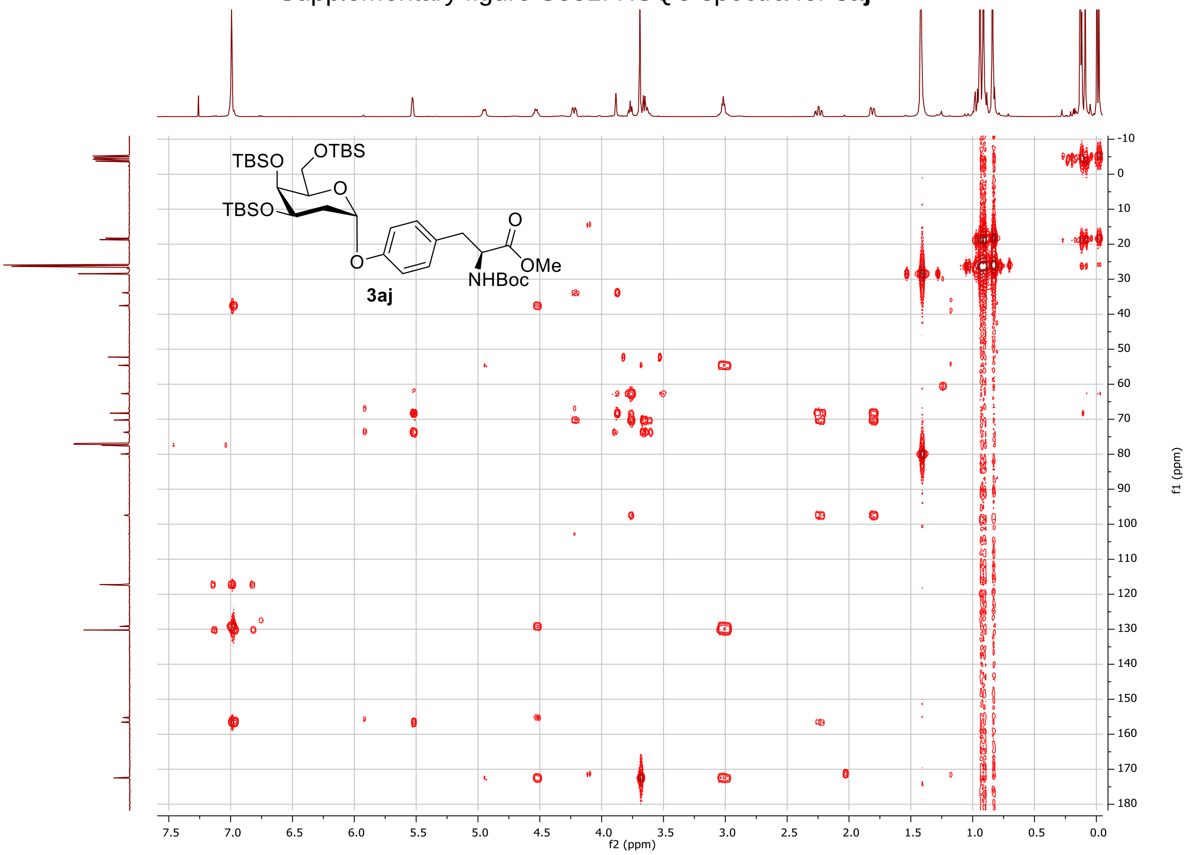

Supplementary figure S332: HMBC spectra for **23aj**

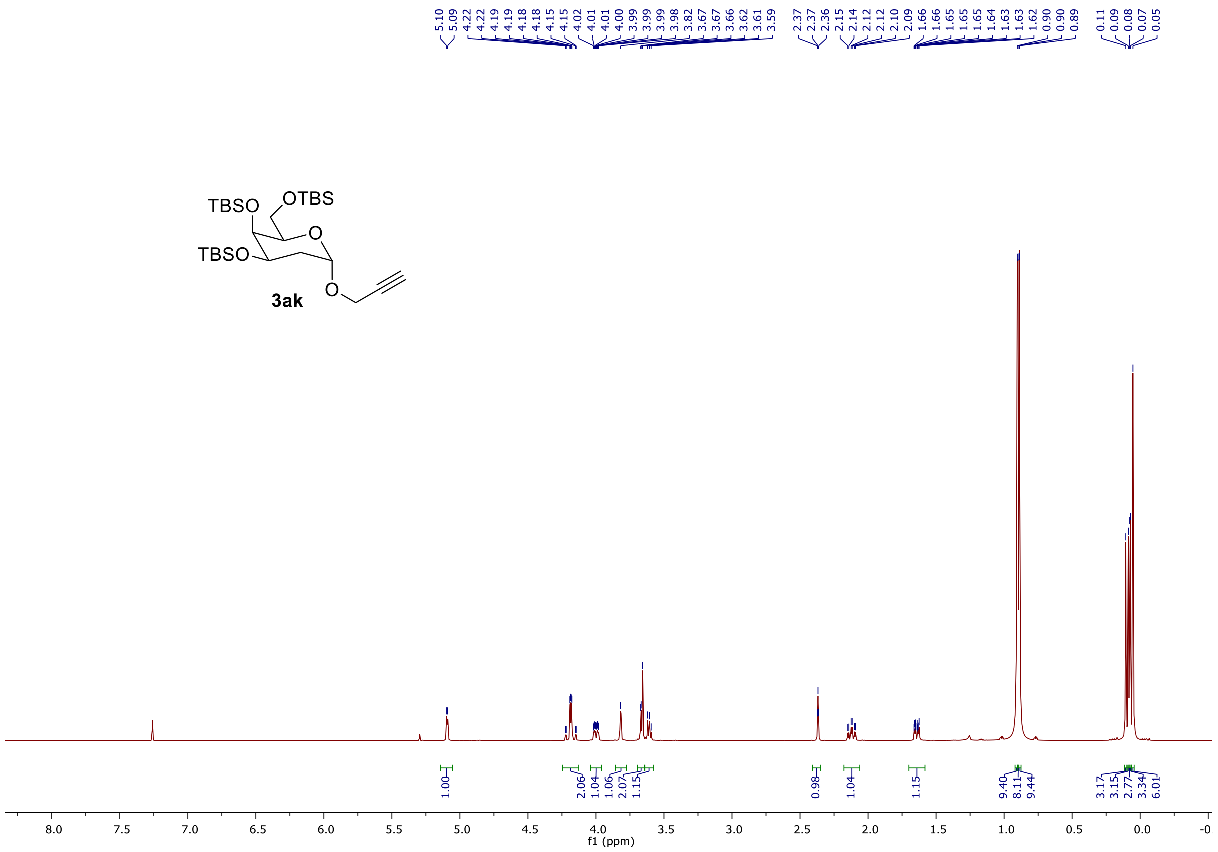

Supplementary figure S333: ^{1}H spectra for **3ak**

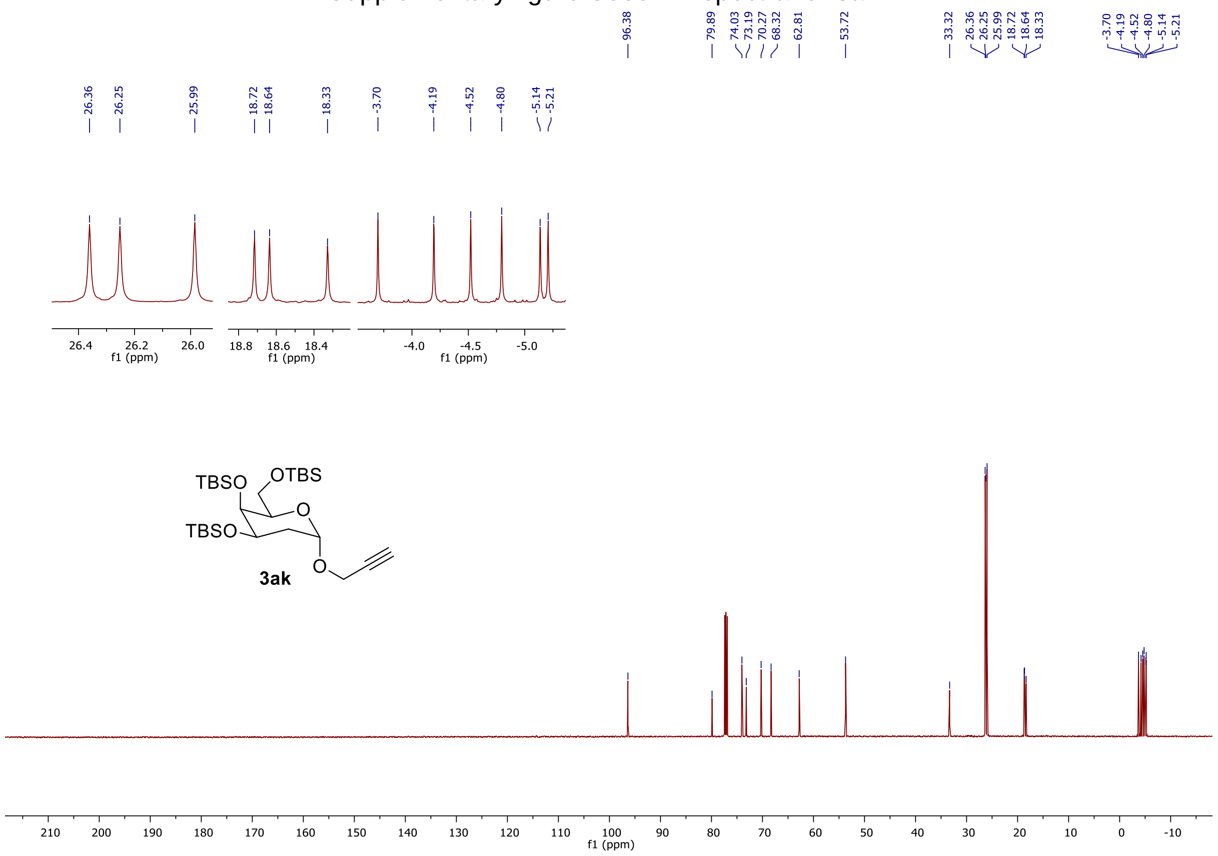

Supplementary figure S334: ^{13}C spectra for **3ak**

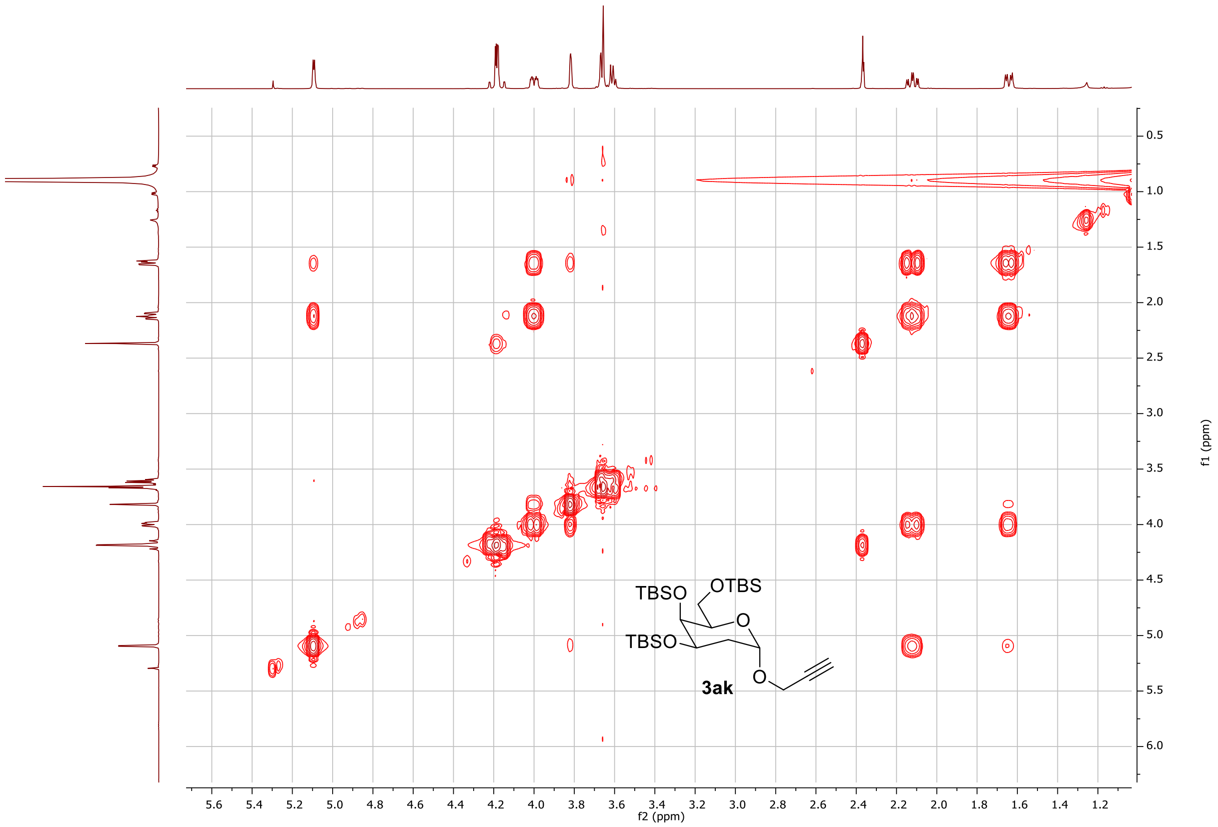

Supplementary figure S335: COSY spectra for **3ak**

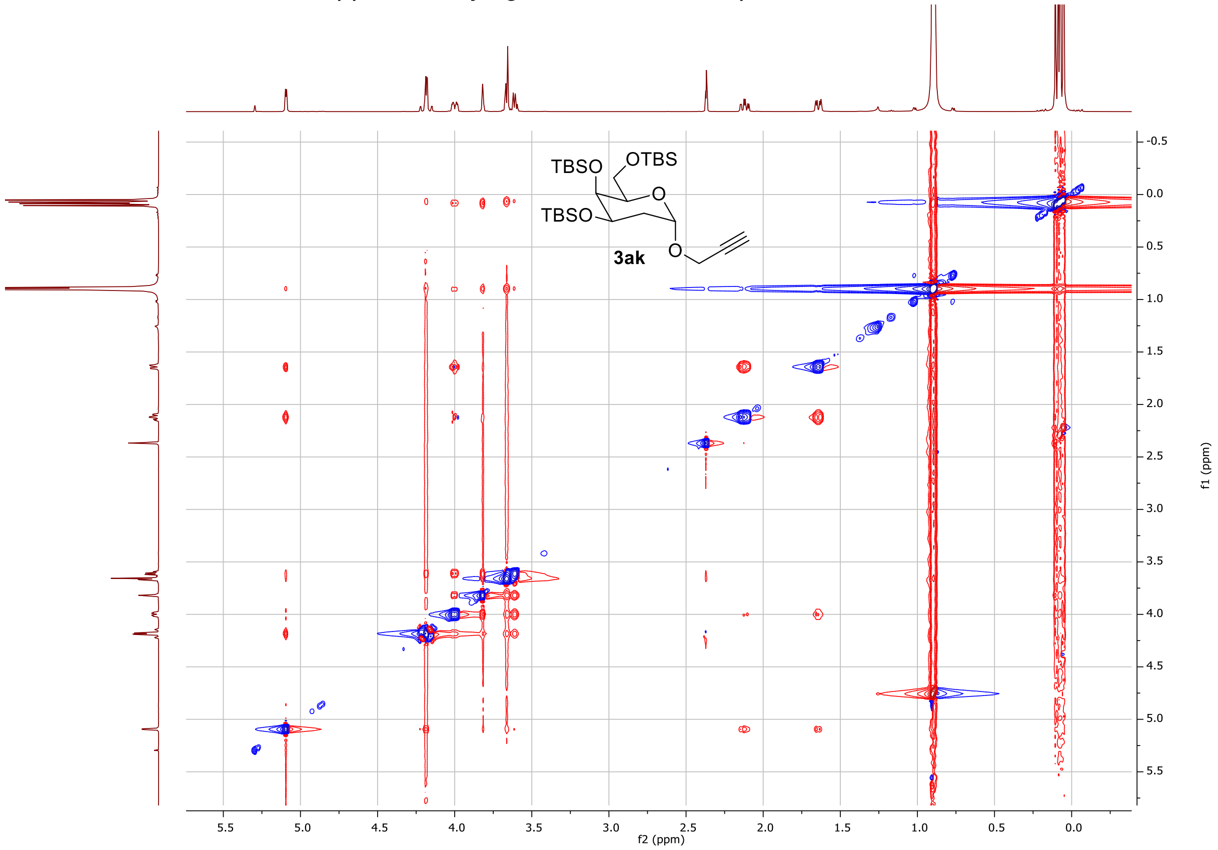

Supplementary figure S336: NOESY spectra for **3ak**

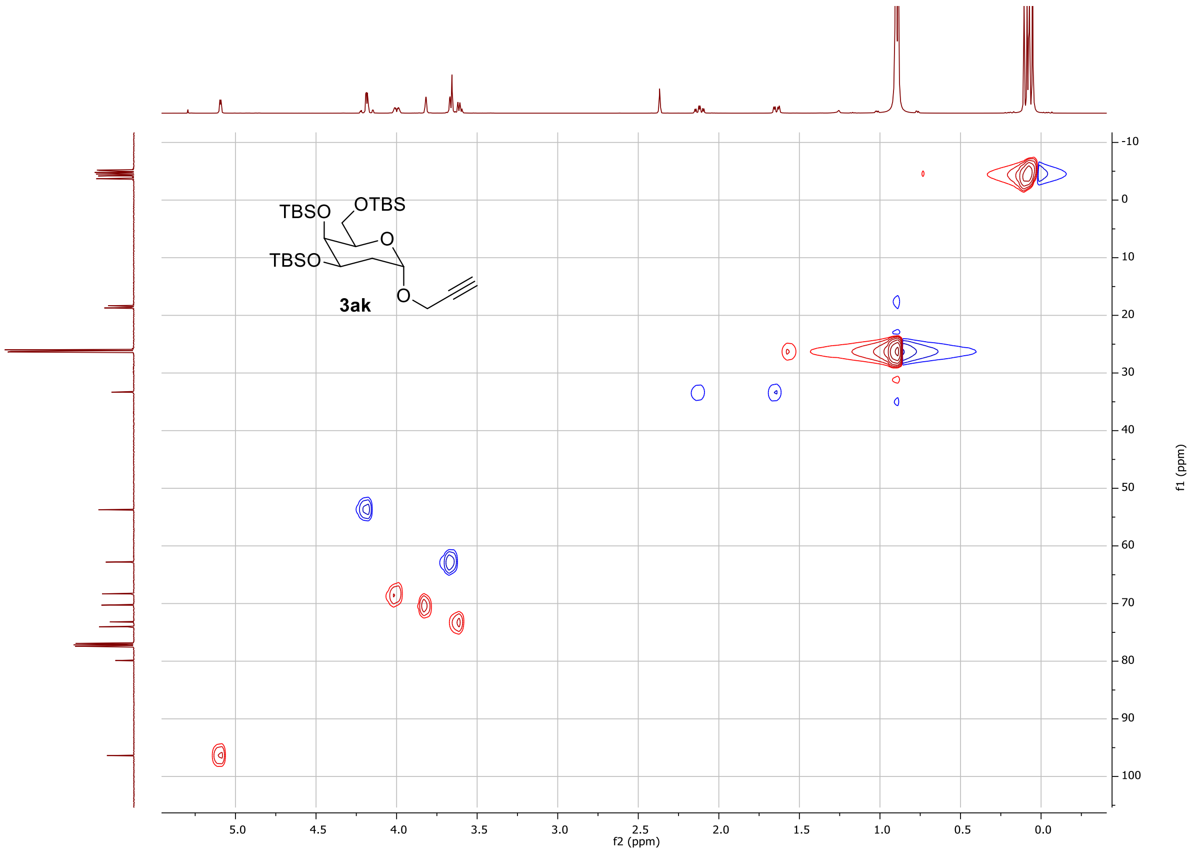

Supplementary figure S337: HSQC spectra for **3ak**

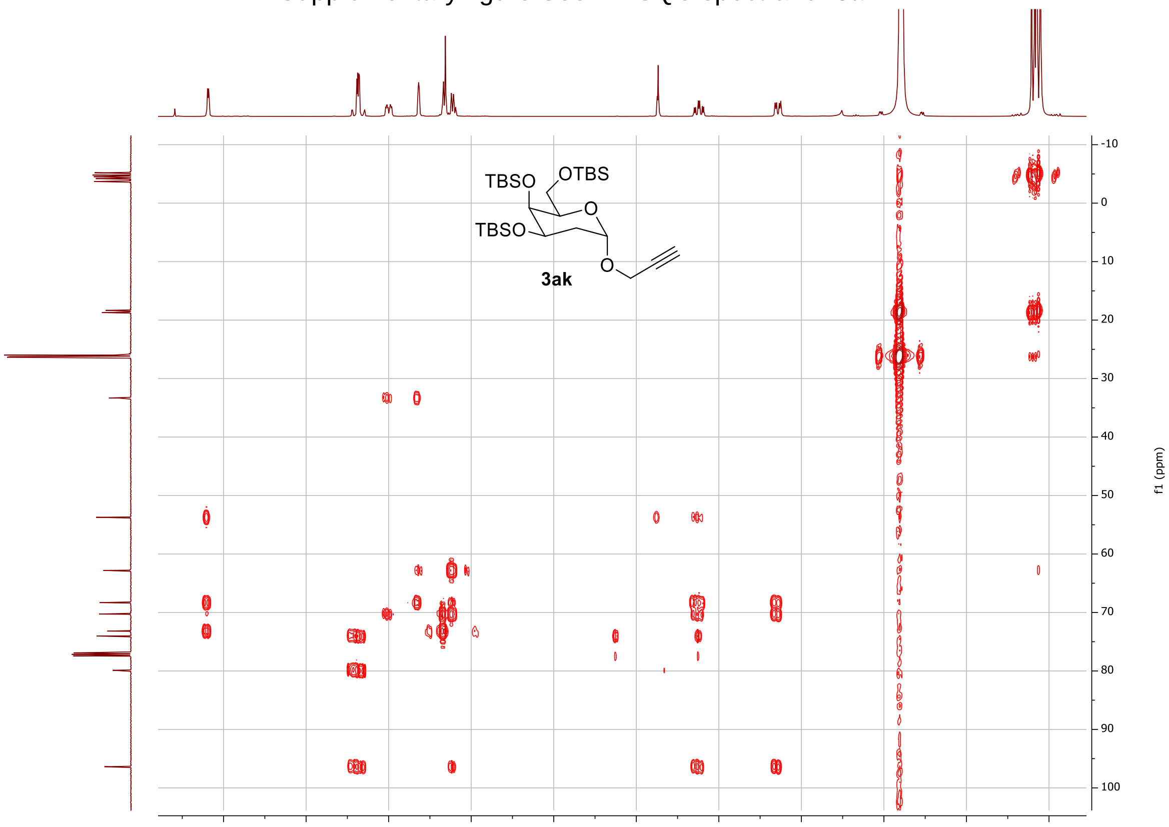

Supplementary figure S338: HMBC spectra for **3ak**

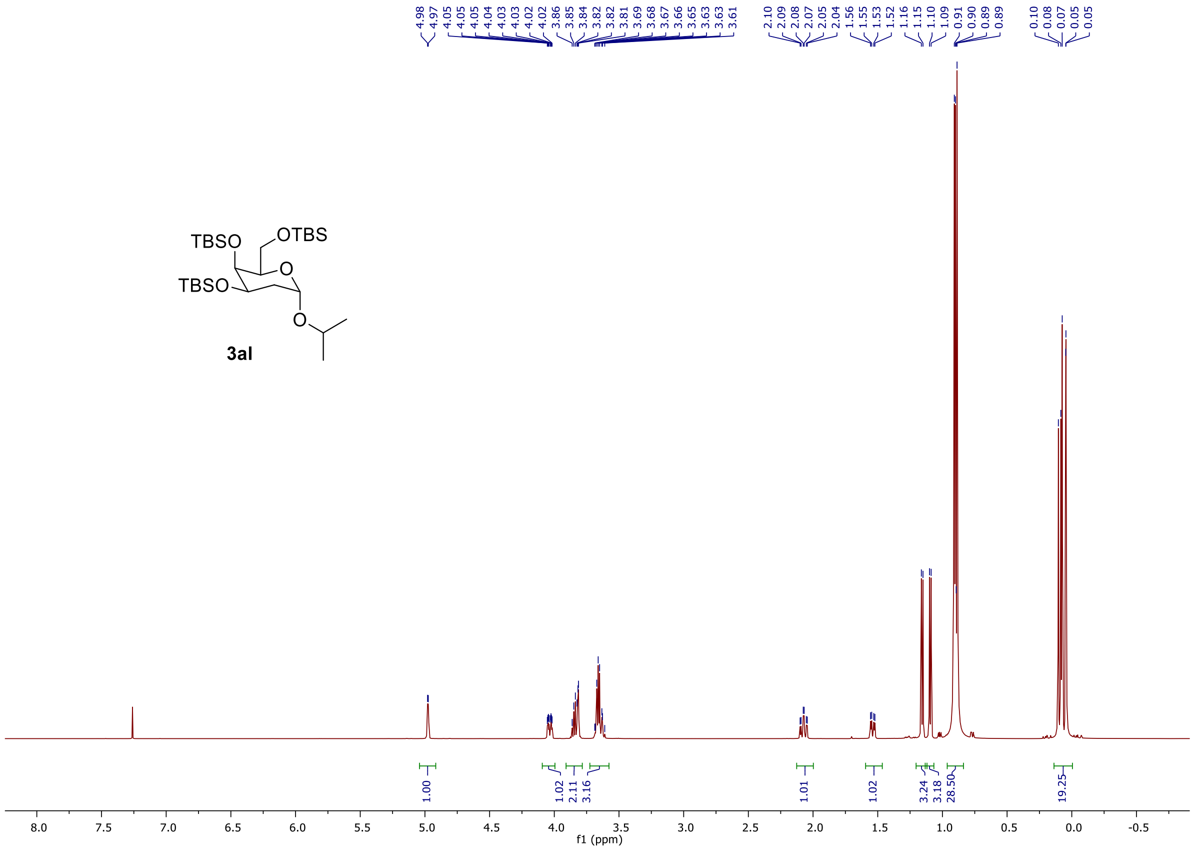

Supplementary figure S339: ^{1}H spectra for **3al**

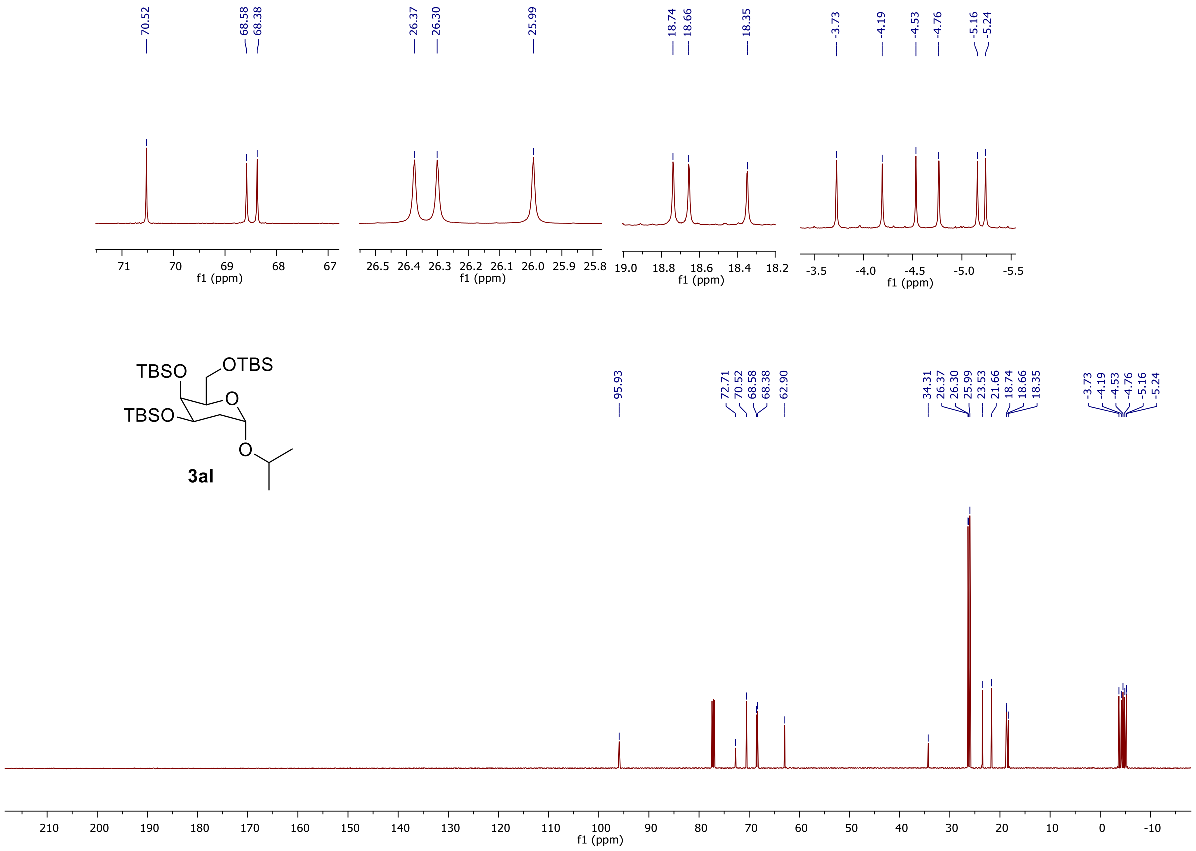

Supplementary figure S340: ^{13}C spectra for **3al**

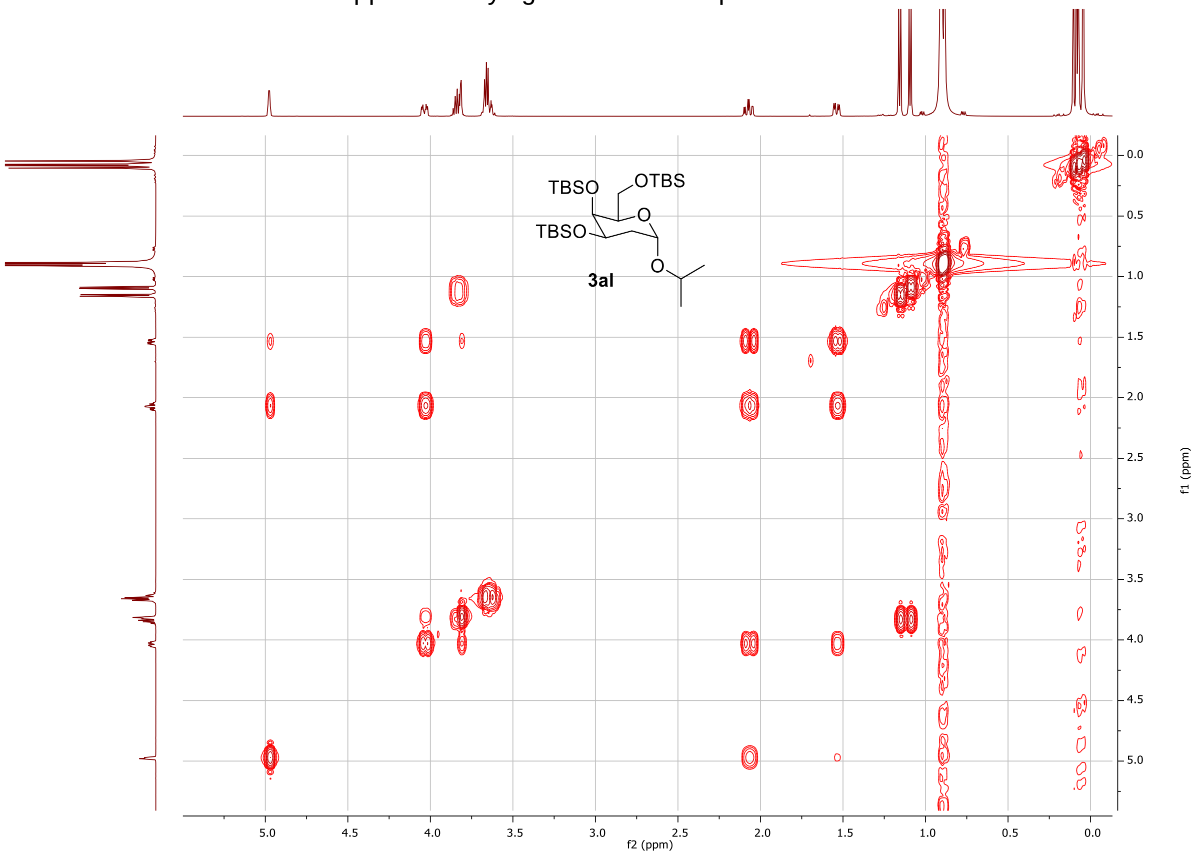

Supplementary figure S341: COSY spectra for **3al**

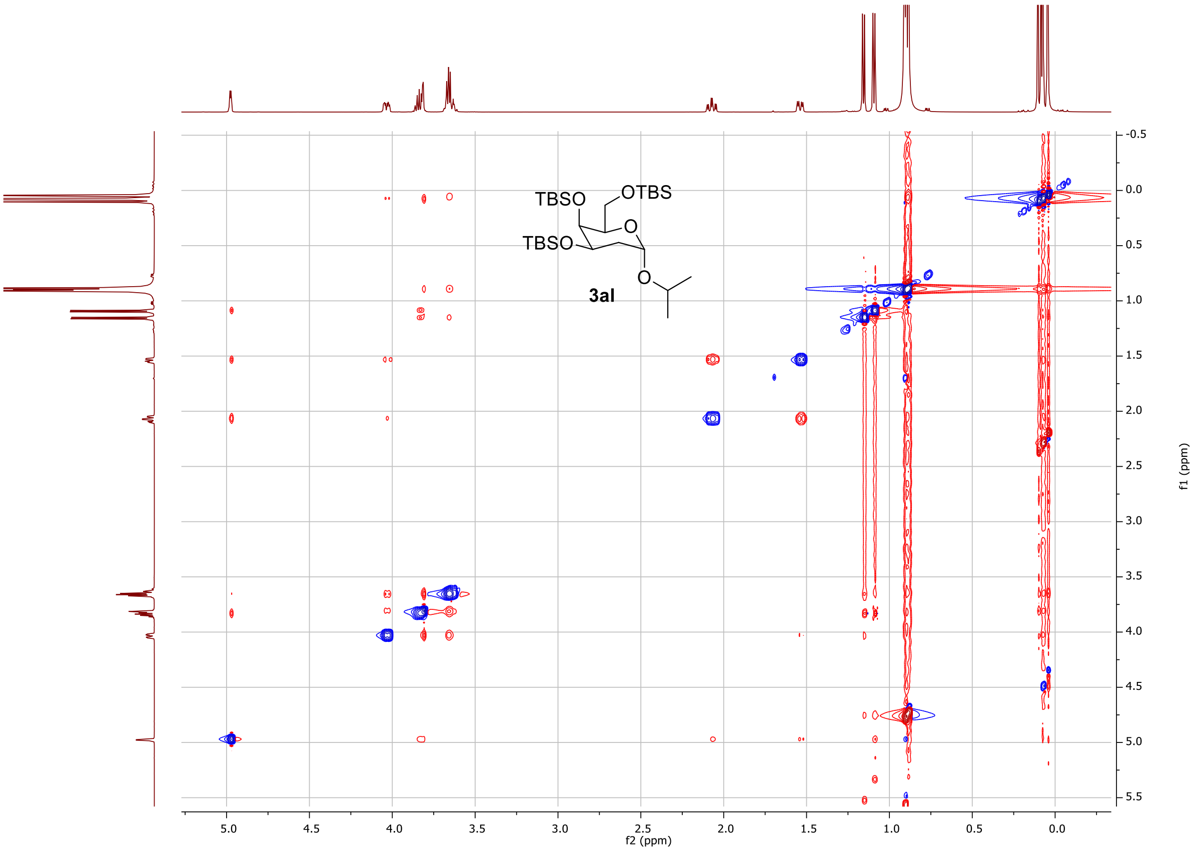

Supplementary figure S342: NOESY spectra for **3al**

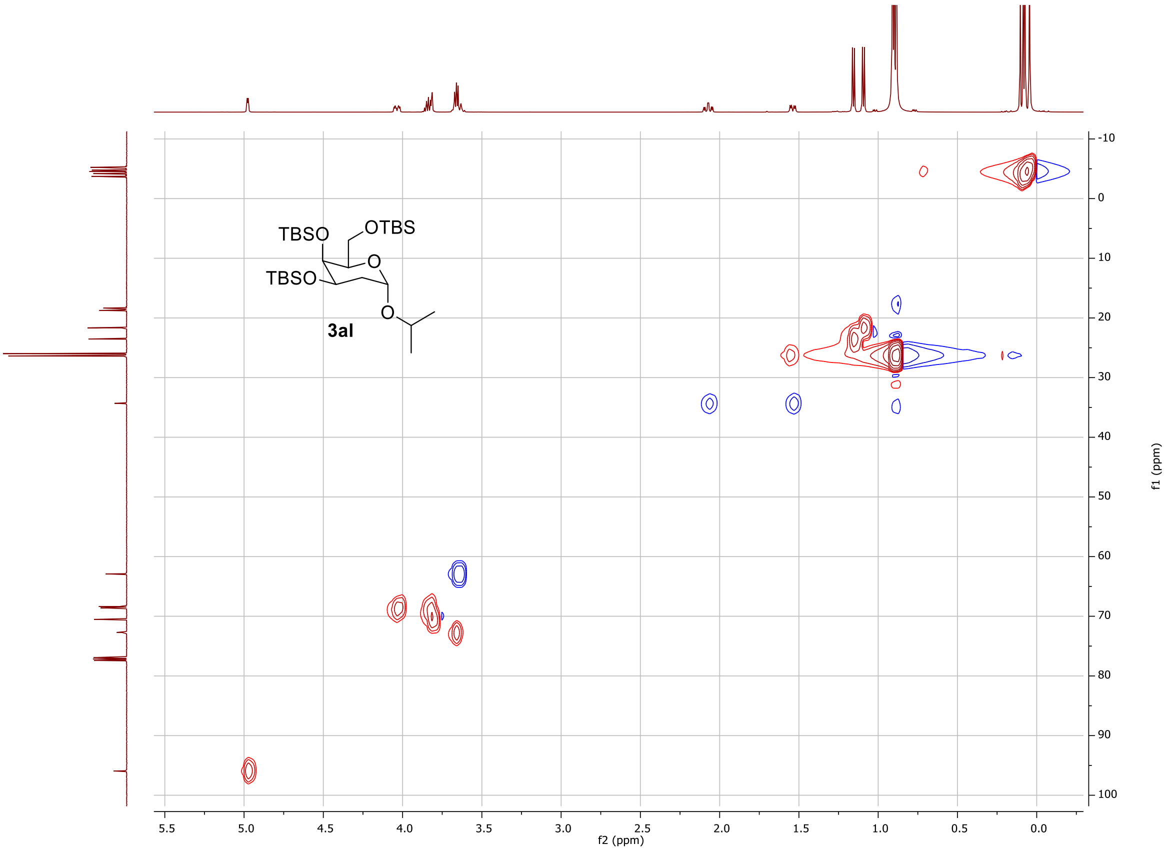

Supplementary figure S343: HSQC spectra for **3al**

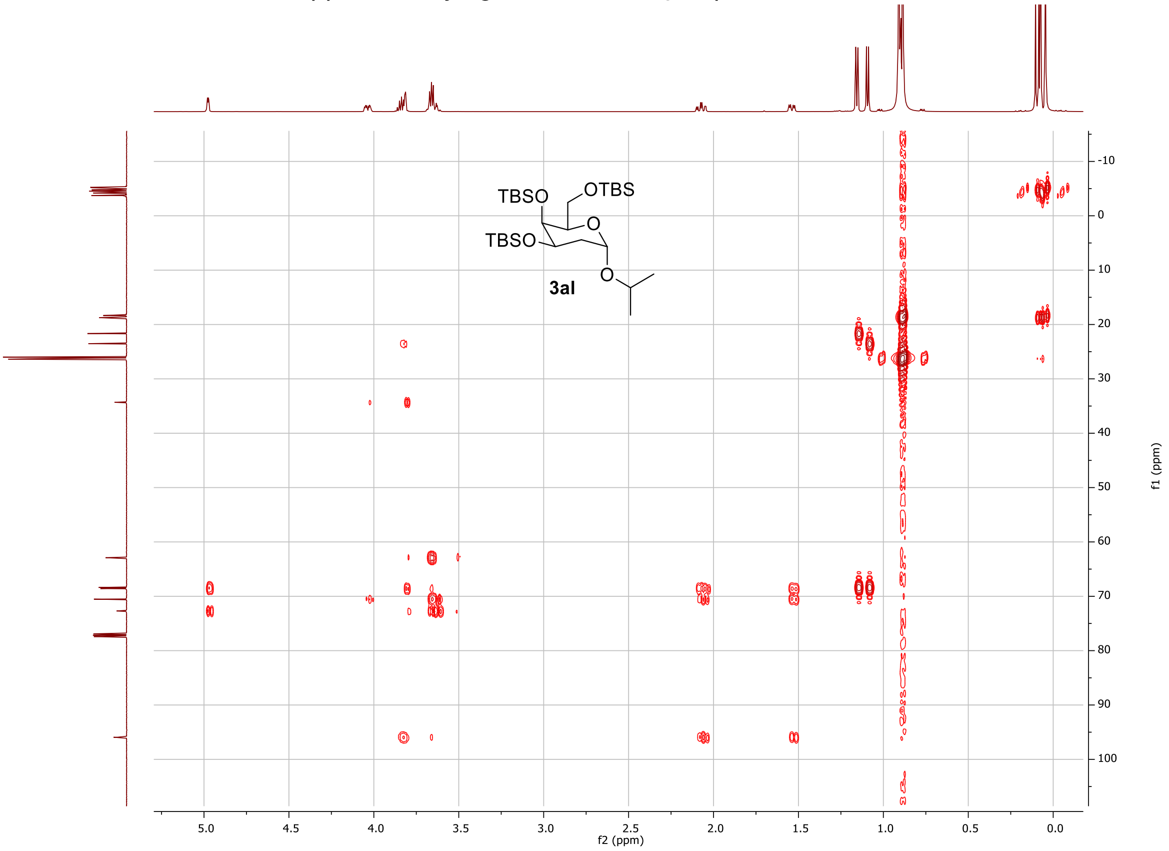

Supplementary figure S344: HMBC spectra for **3al**

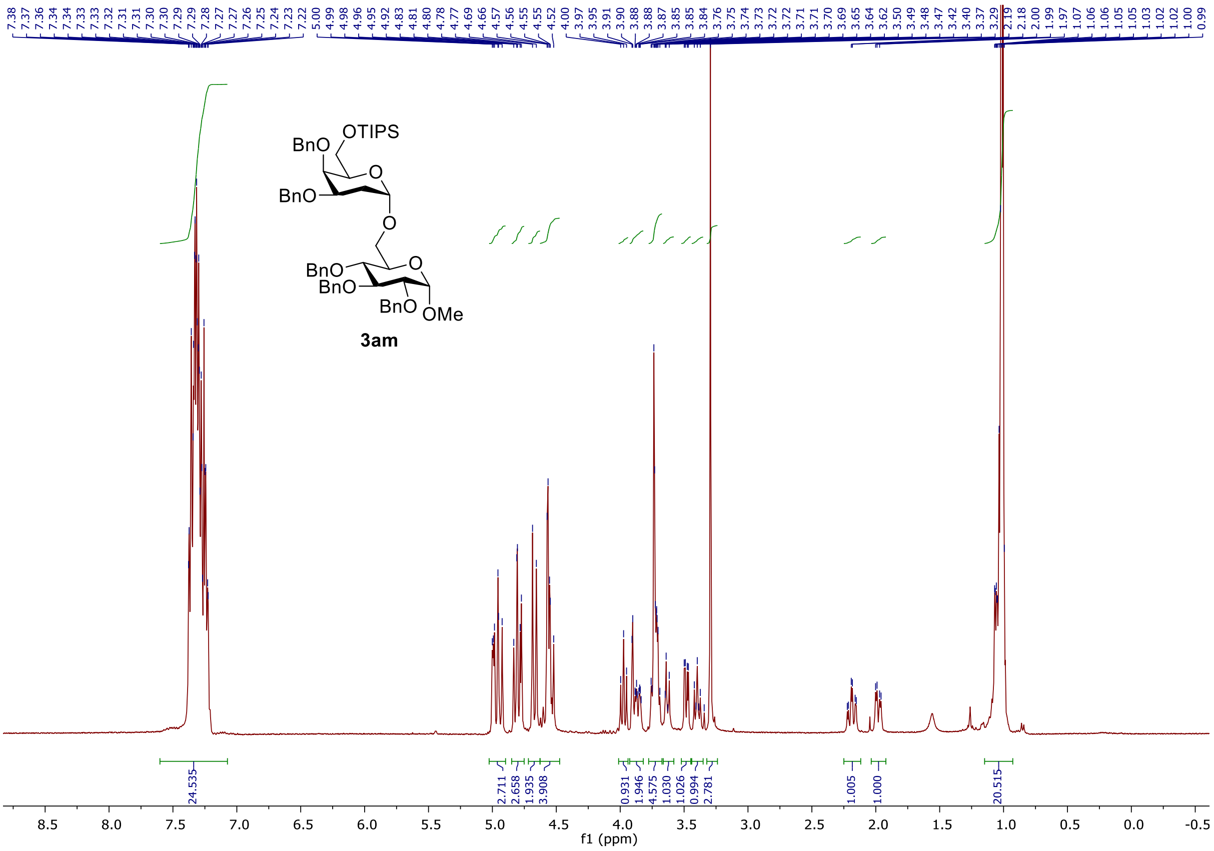

Supplementary figure S345: ¹H spectra for **3am**

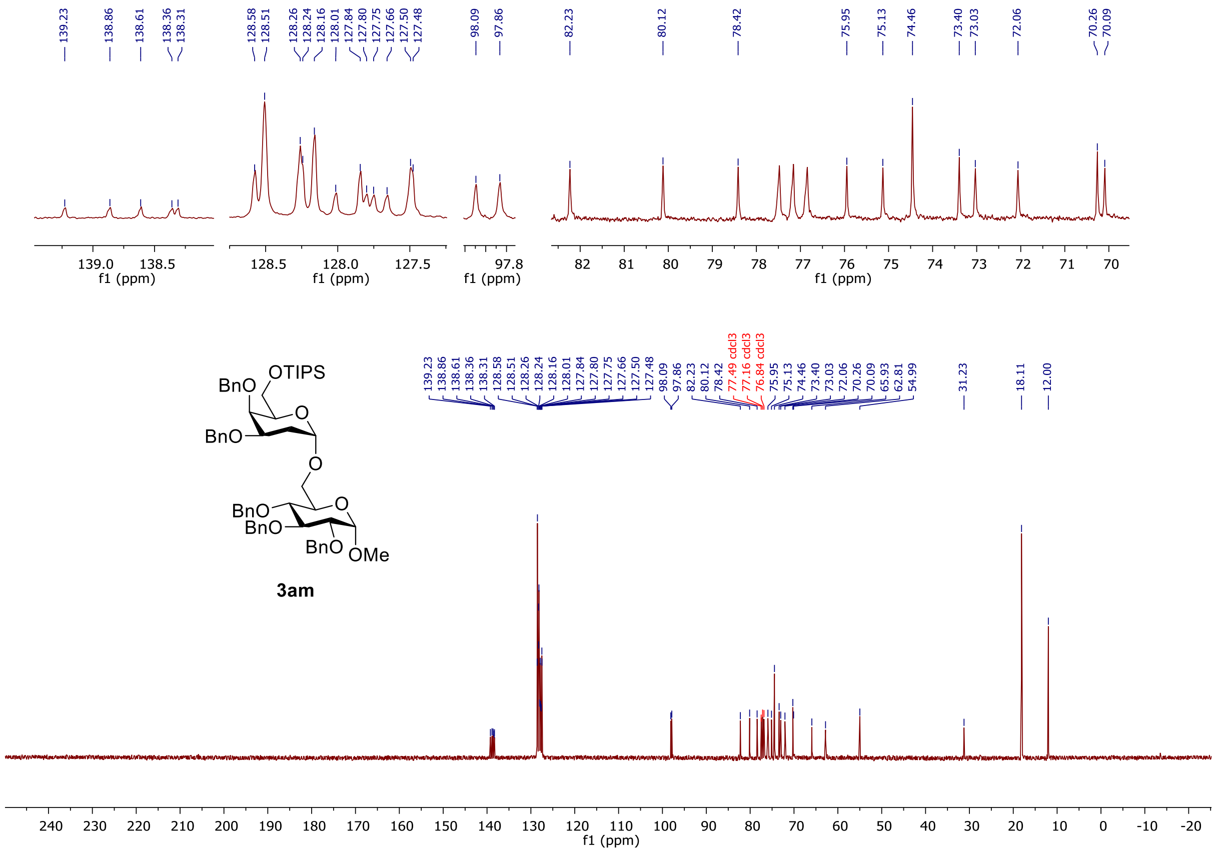

Supplementary figure S346: ^{13}C spectra for **3am**

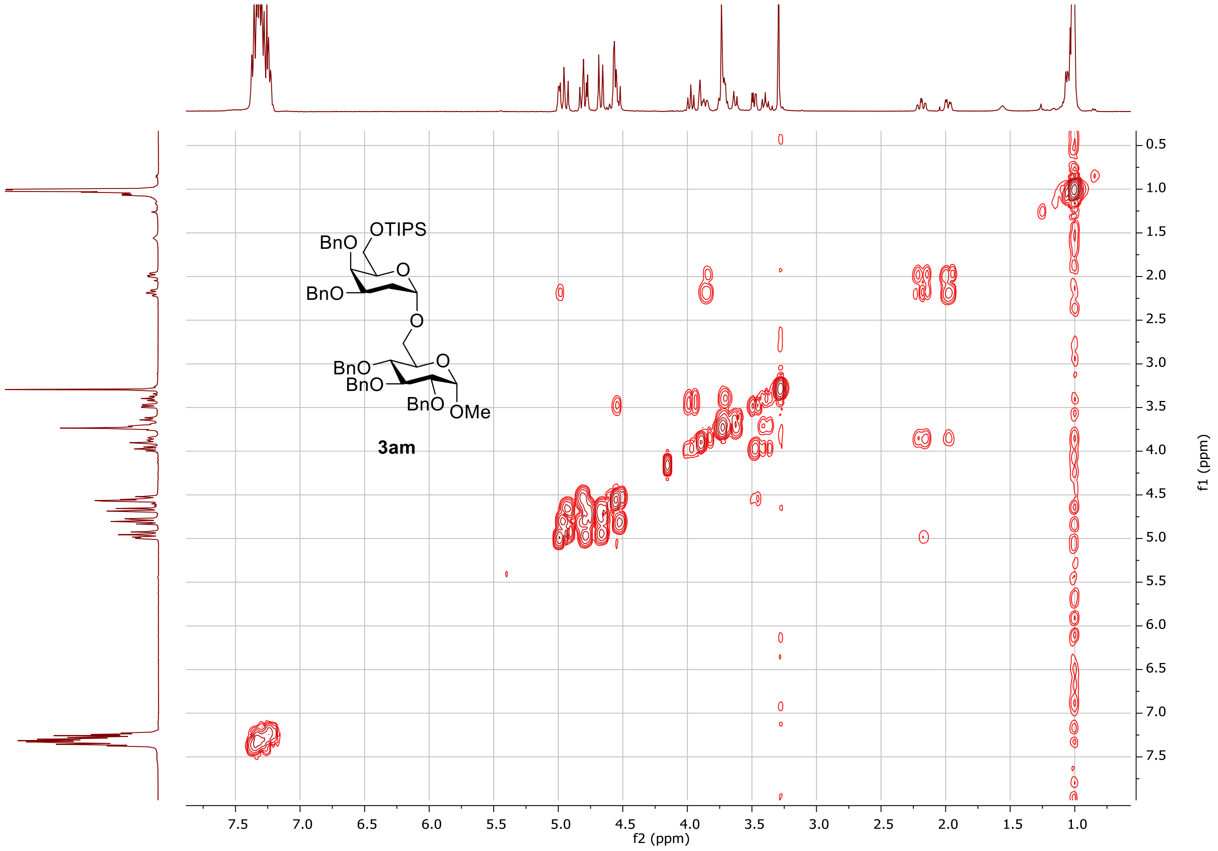

Supplementary figure S347: COSY spectra for **3am**

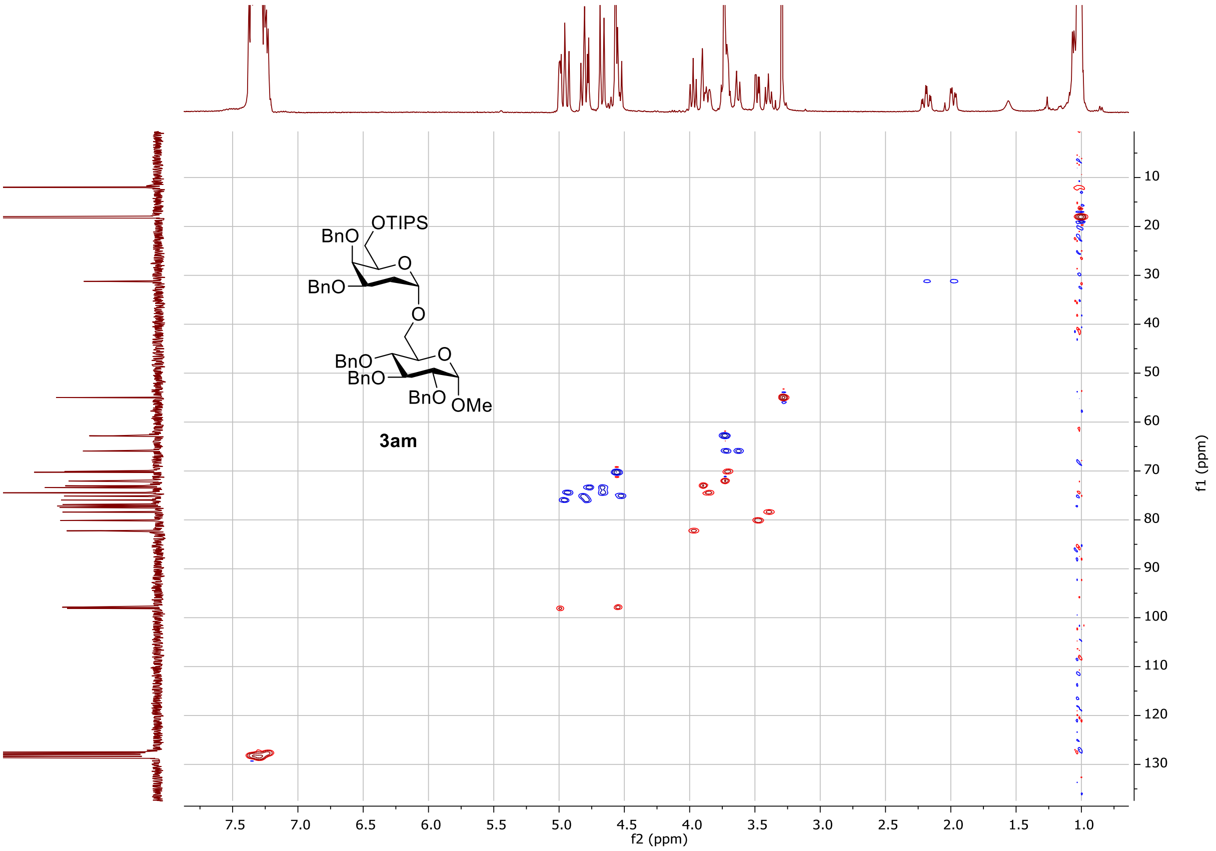

Supplementary figure S348: HSQC spectra for **3am**

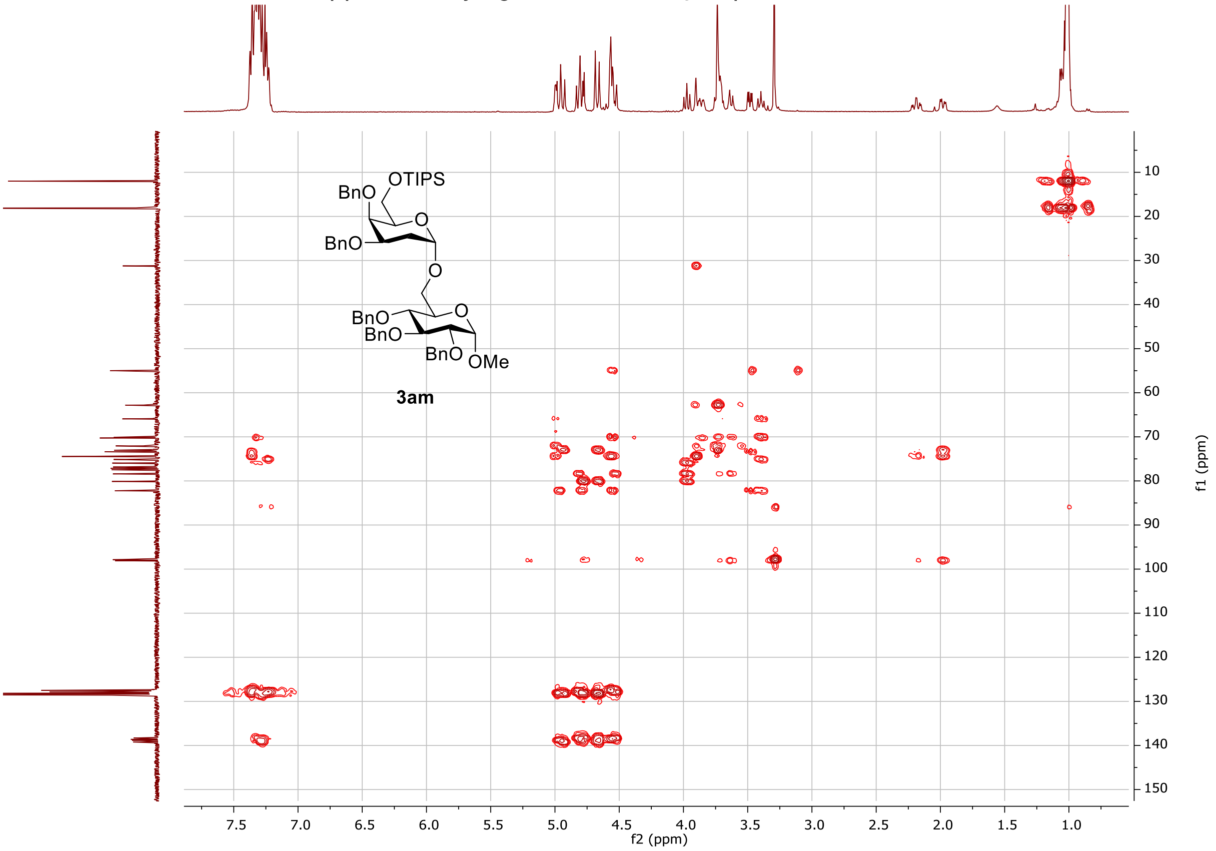

Supplementary figure S349: HMBC spectra for **3am**

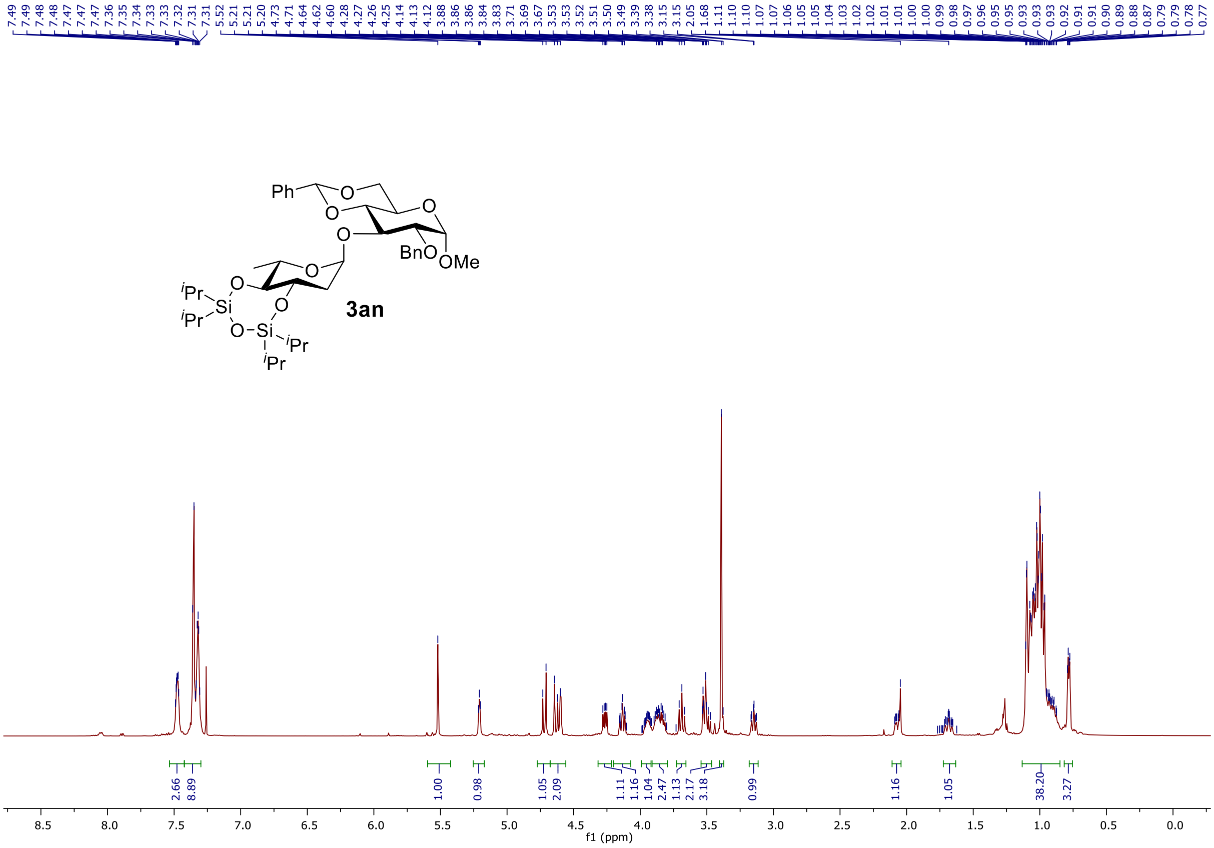

Supplementary figure S350: ^{1}H spectra for **3an**

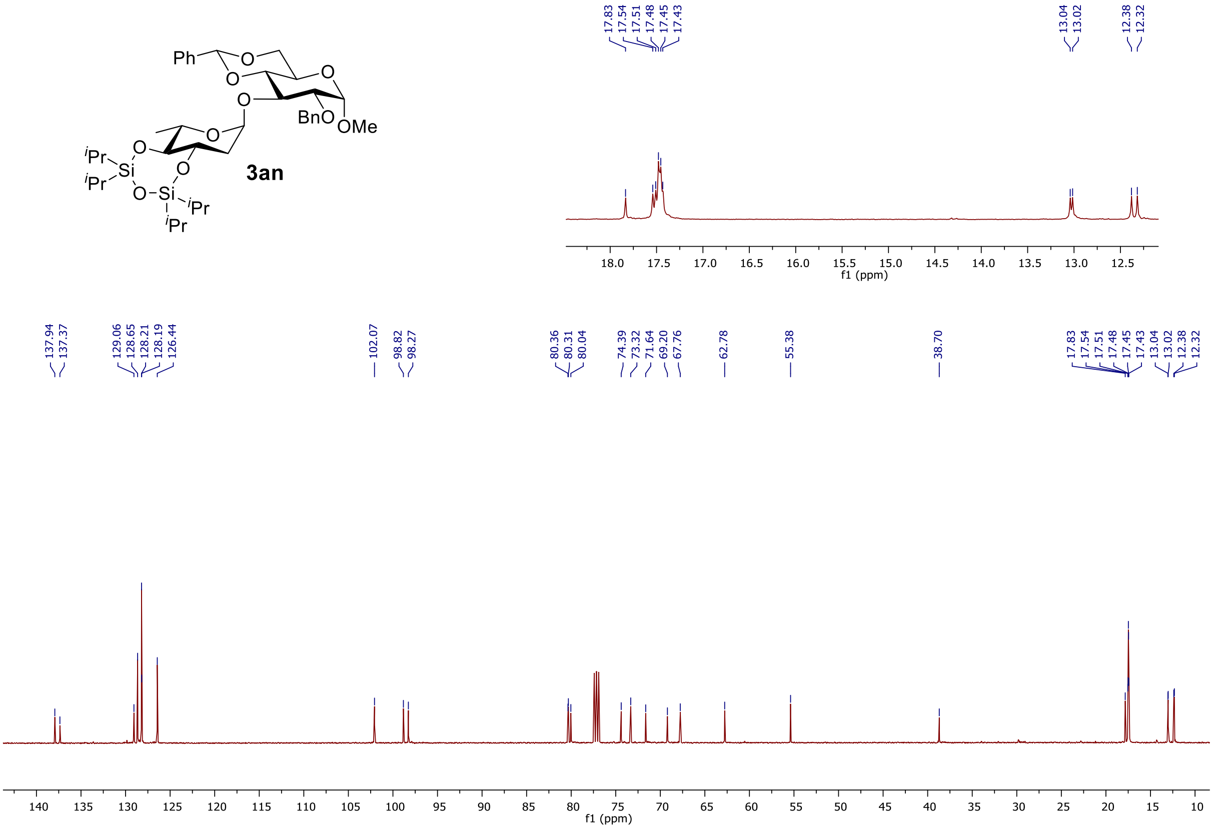

Supplementary figure S351: ^{13}C spectra for **3an**

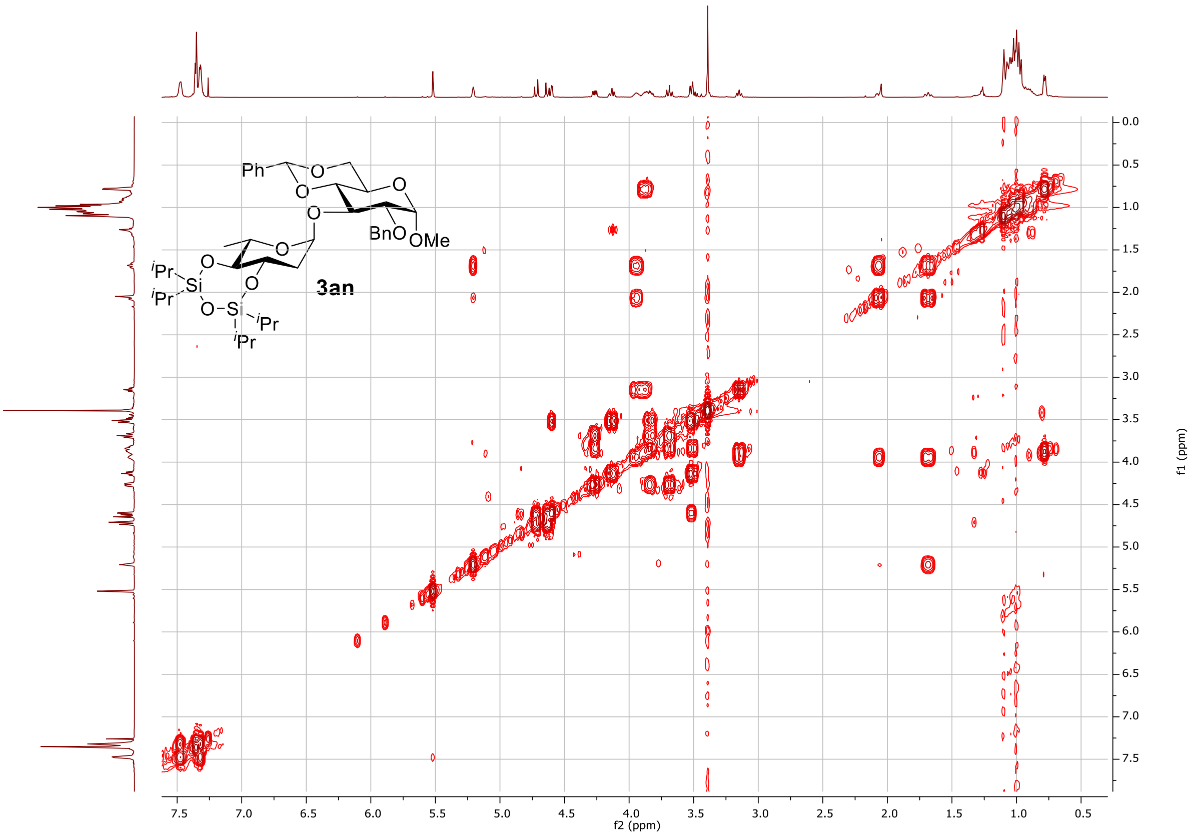

Supplementary figure S352: COSY spectra for **3an**

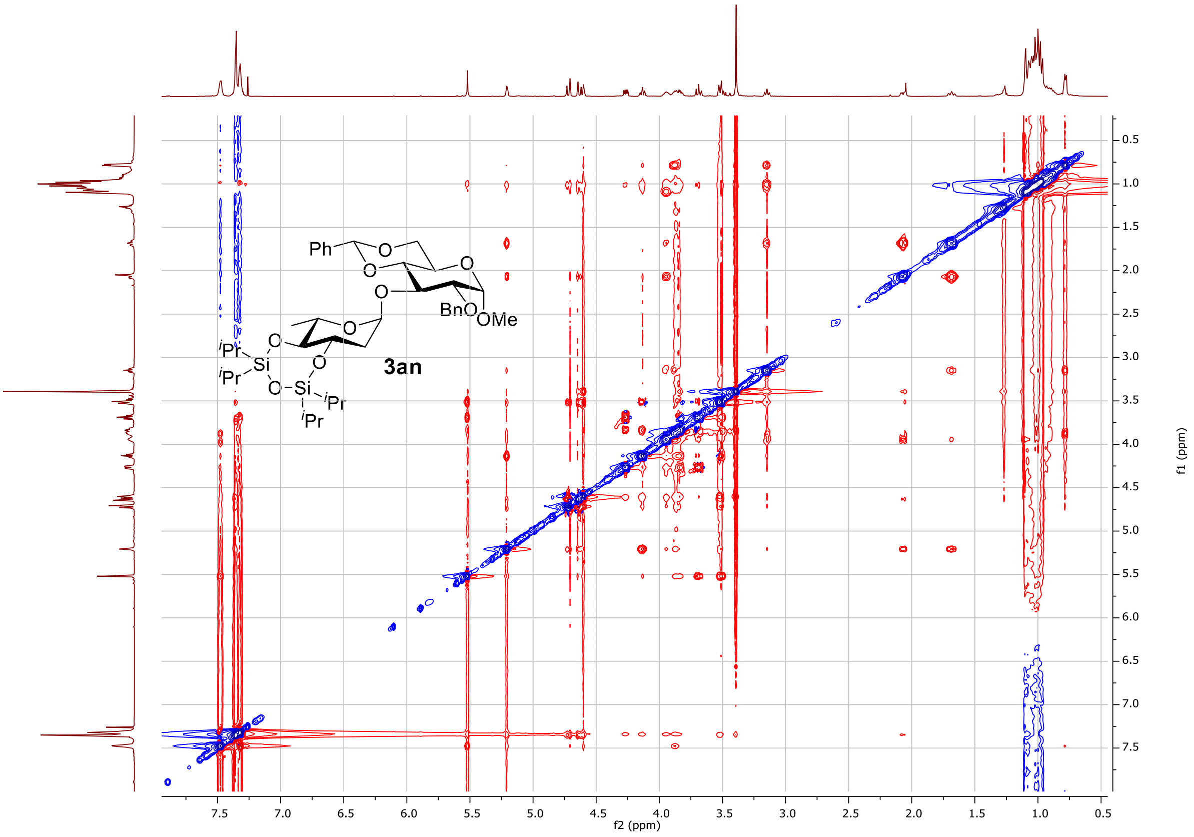

Supplementary figure S353: NOESY spectra for **3an**

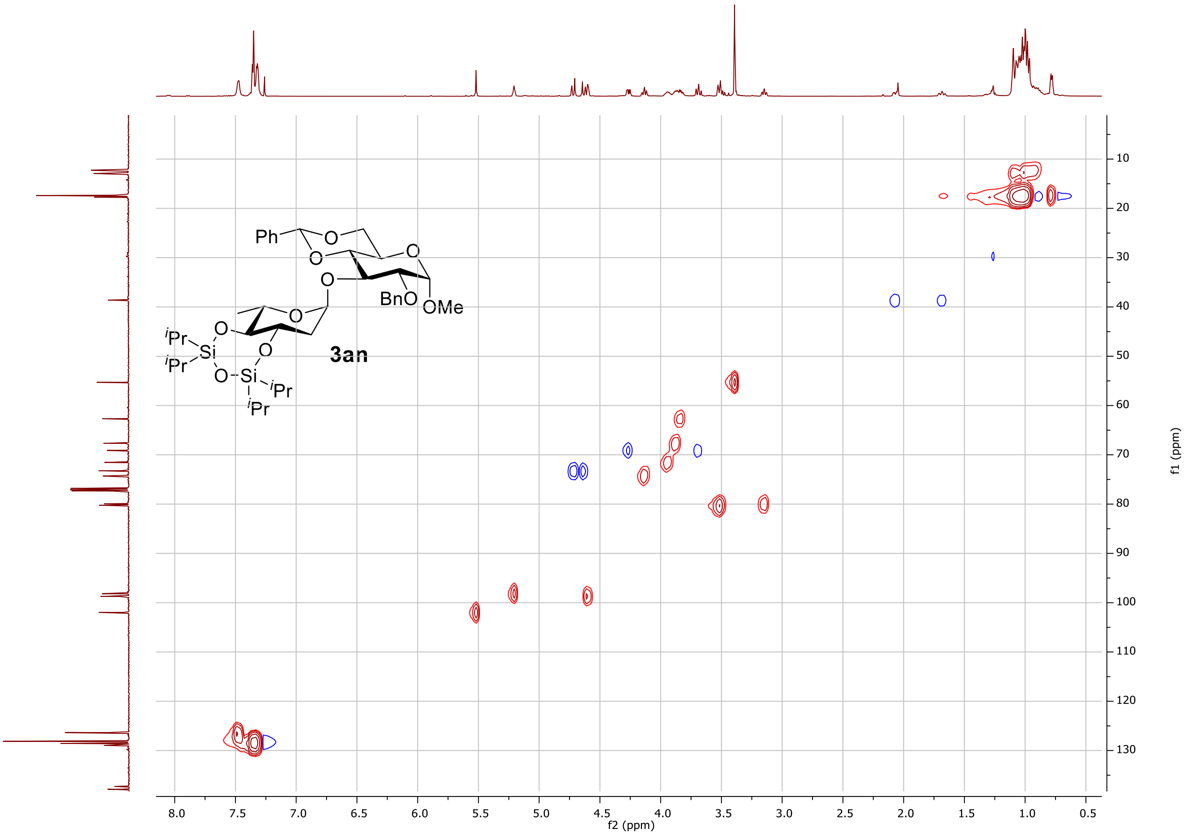

Supplementary figure S354: HSQC spectra for **3an**

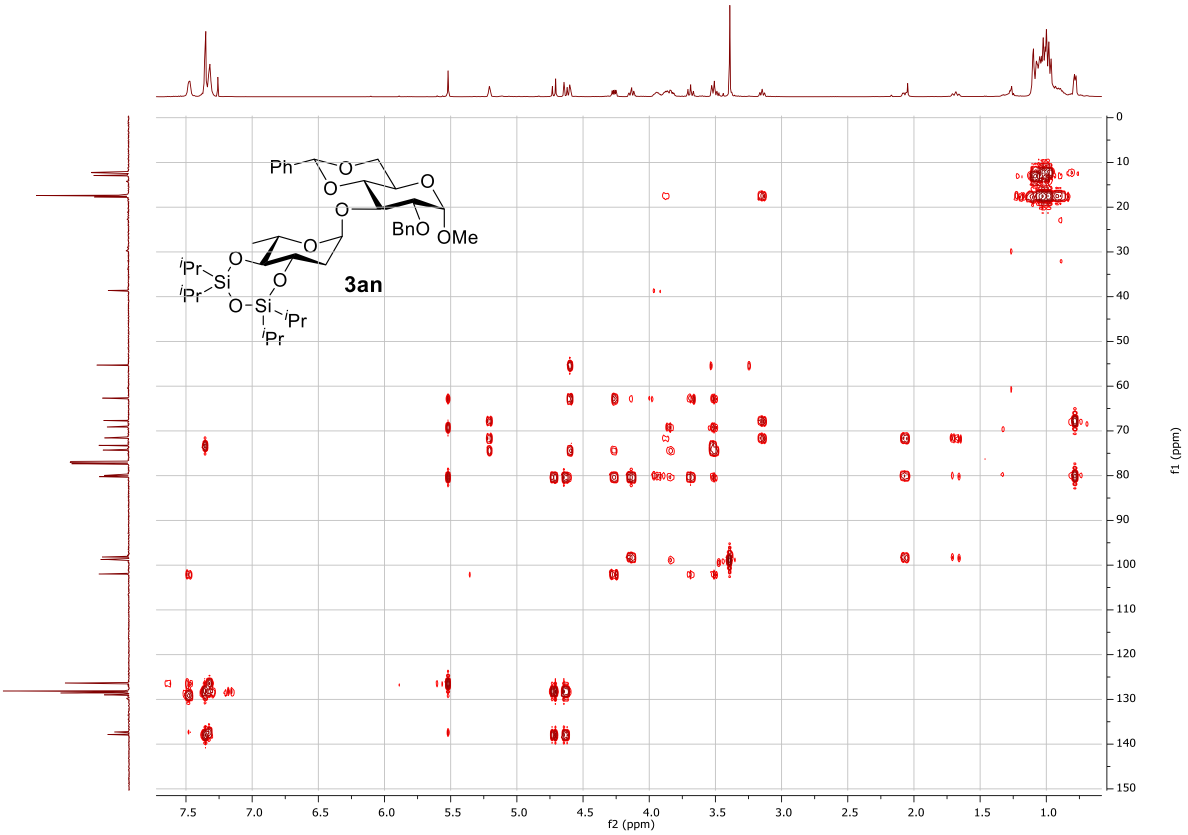

Supplementary figure S355: HMBC spectra for **3an**

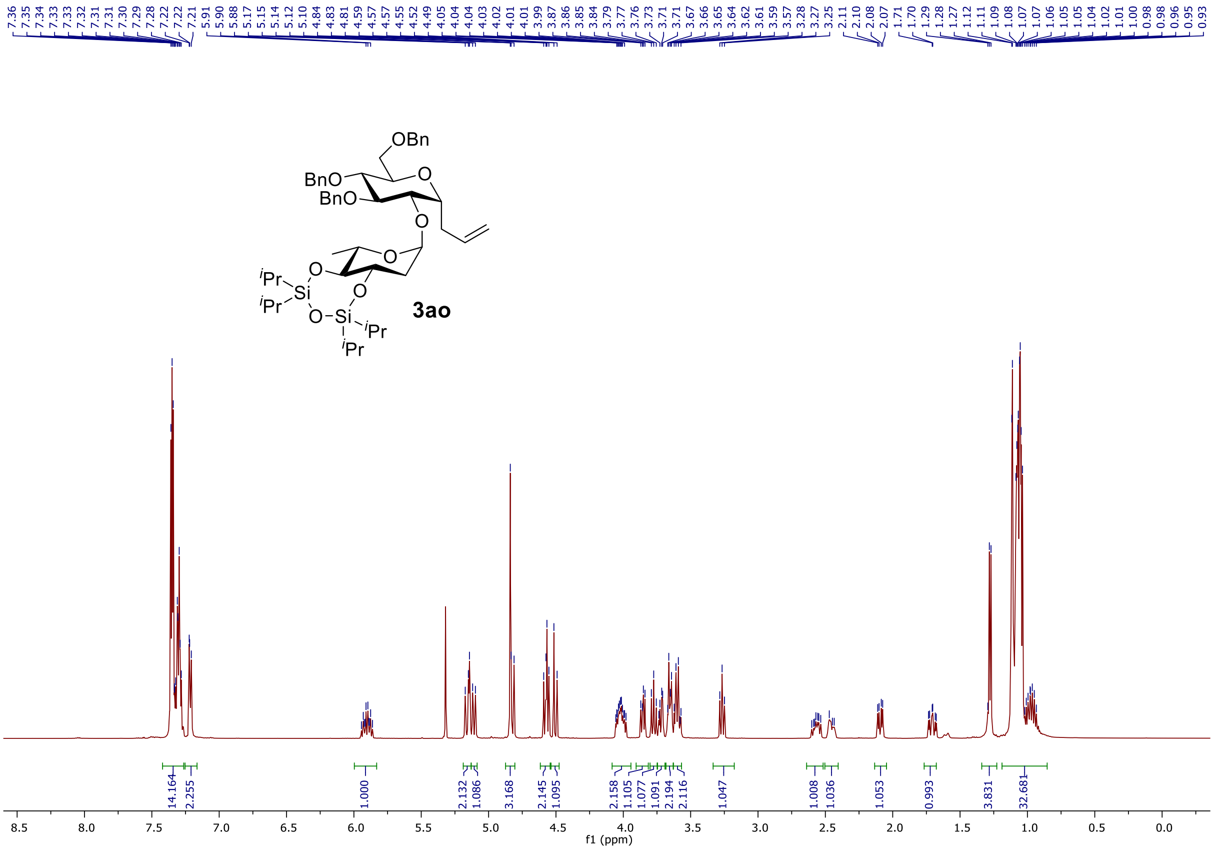

Supplementary figure S356: ^{1}H spectra for **3ao**

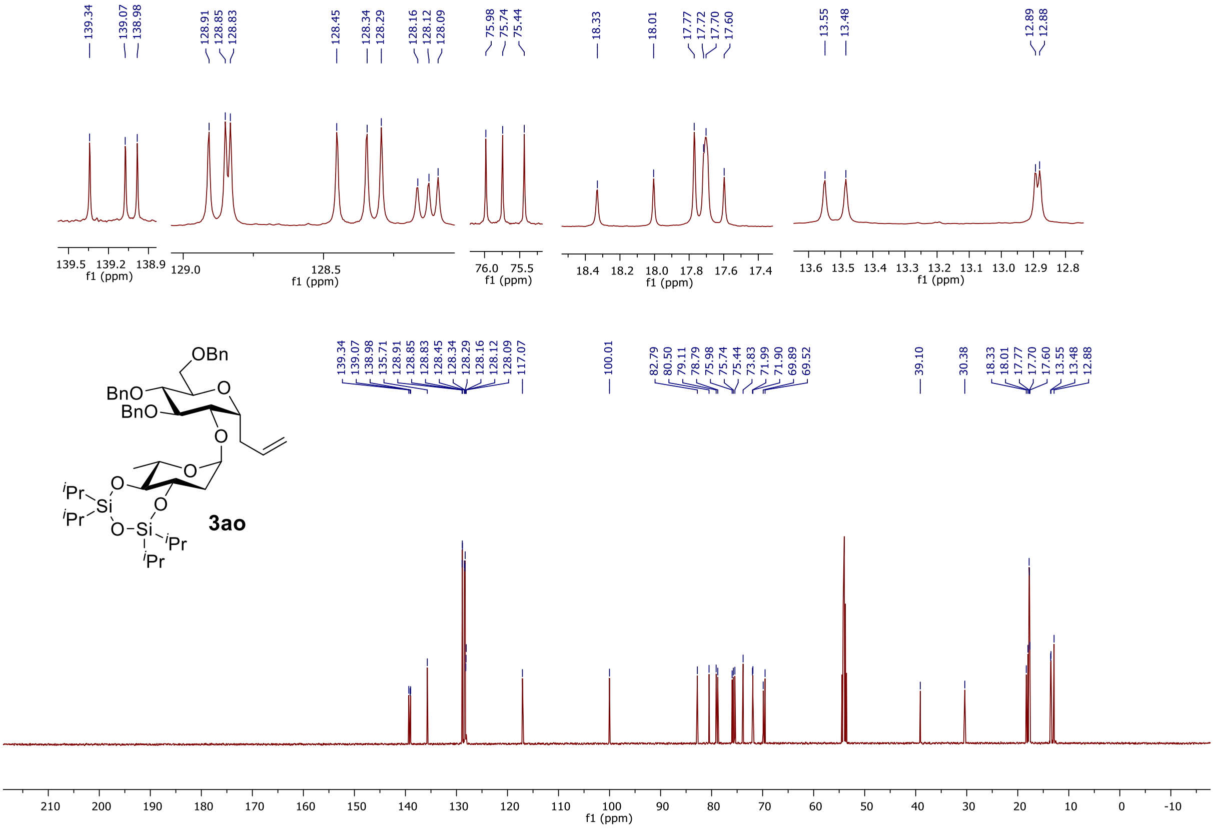

Supplementary figure S357: ^{13}C spectra for **3ao**

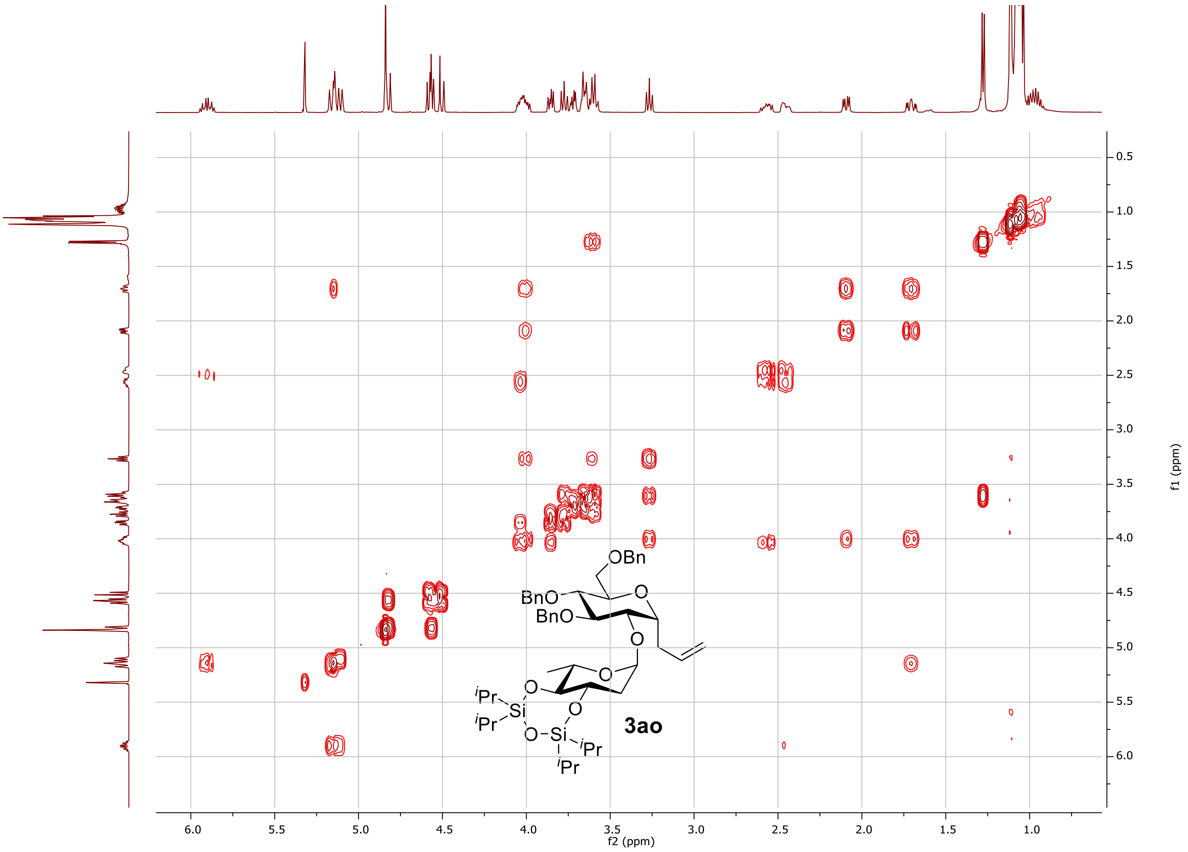

Supplementary figure S358: COSY spectra for **3ao**

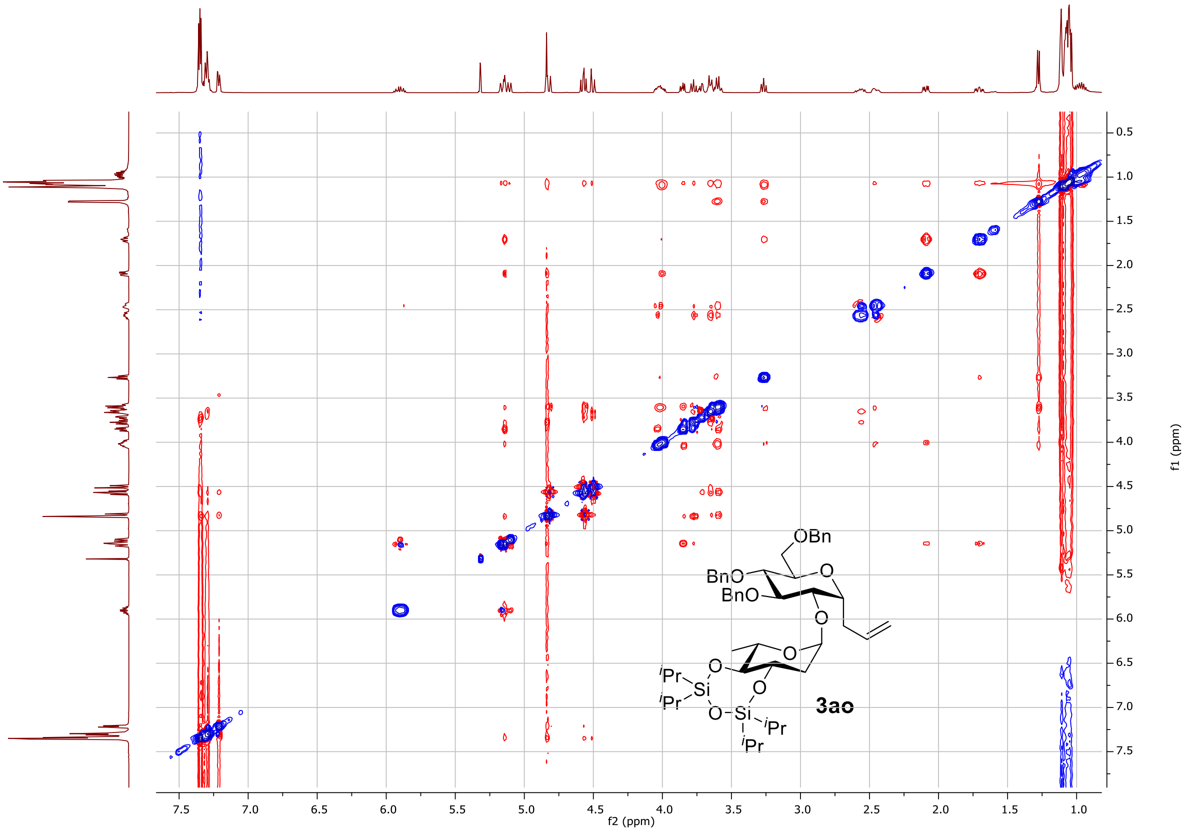

Supplementary figure S359: NOESY spectra for **3ao**

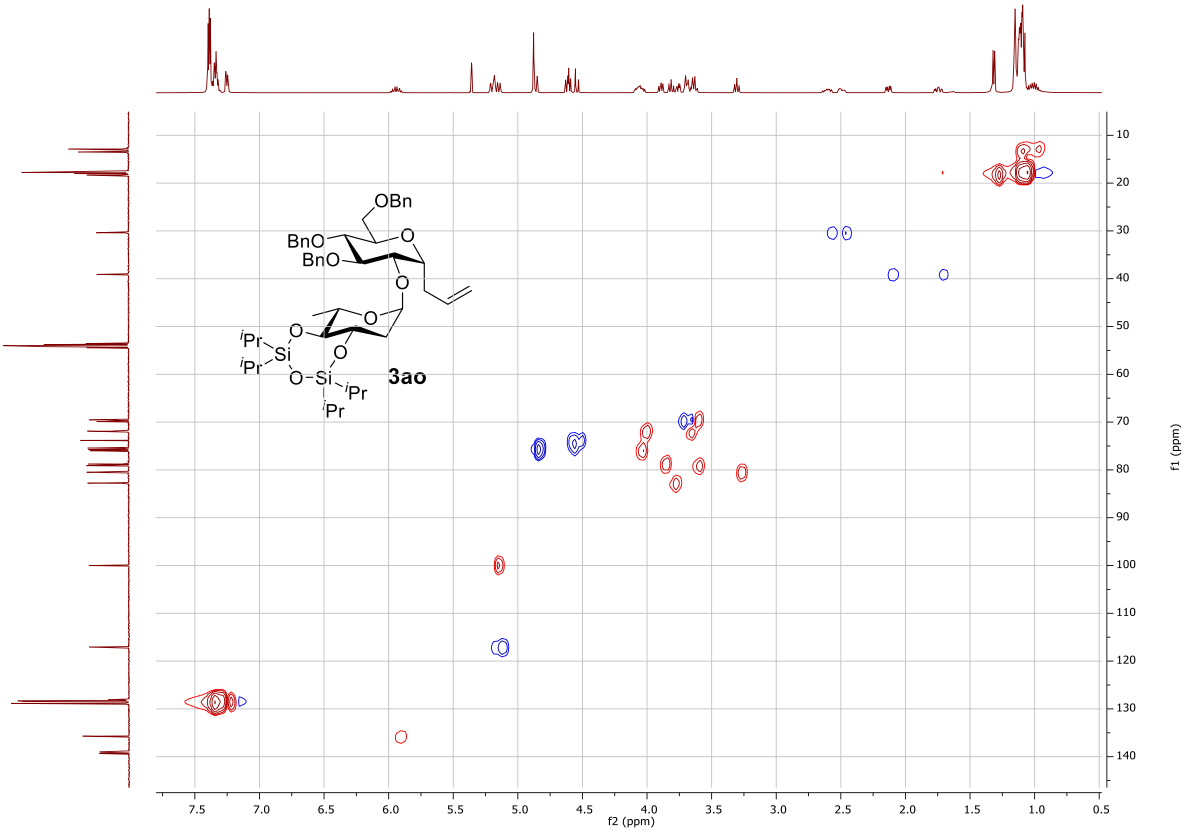

Supplementary figure S360: HSQC spectra for **3ao**

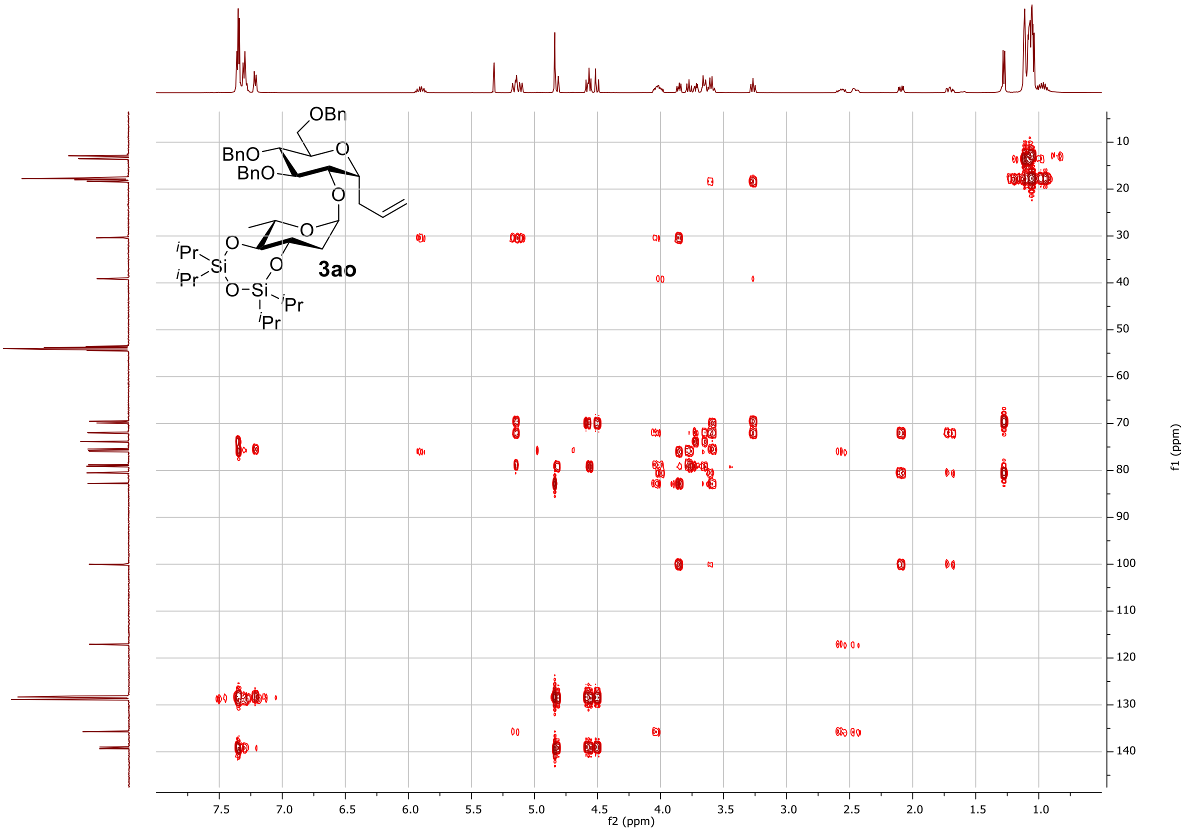

Supplementary figure S361: HMBC spectra for **3ao**

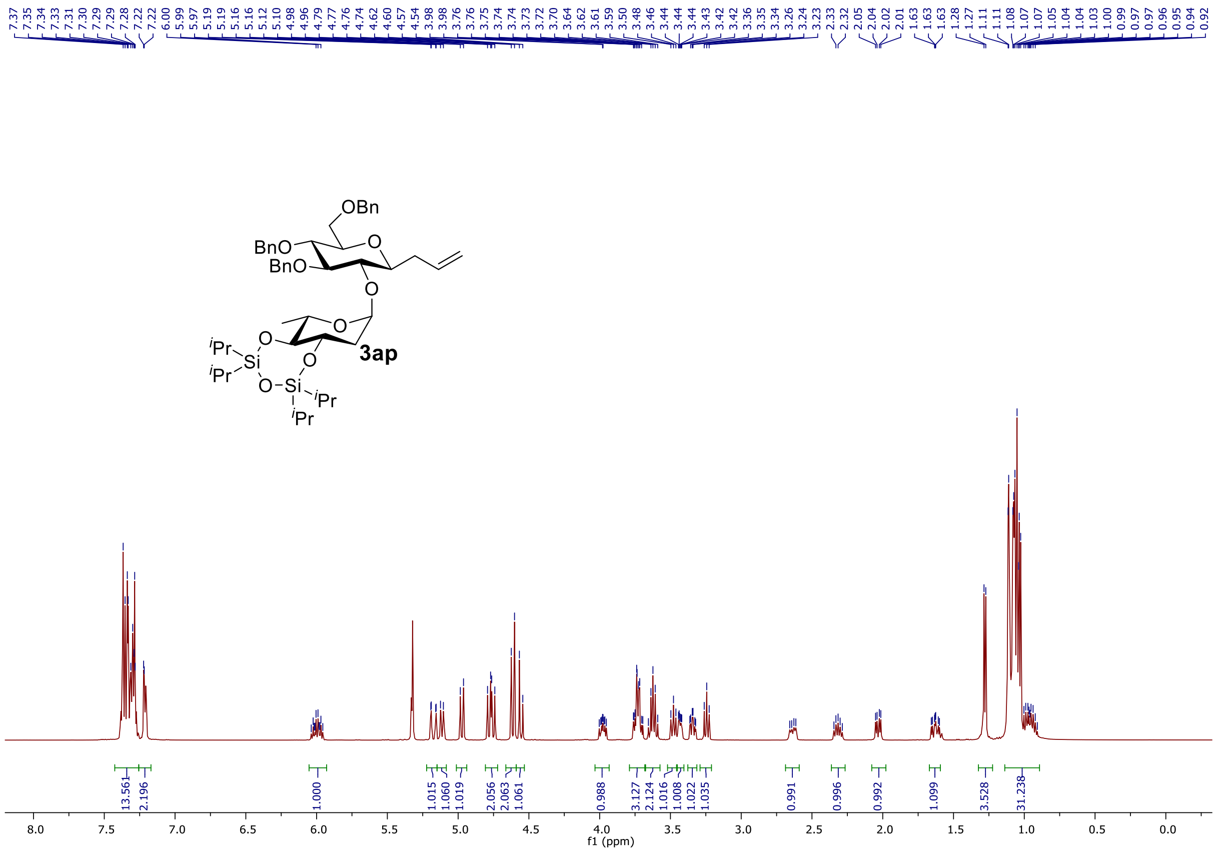

Supplementary figure S362: ^{1}H spectra for **3ap**

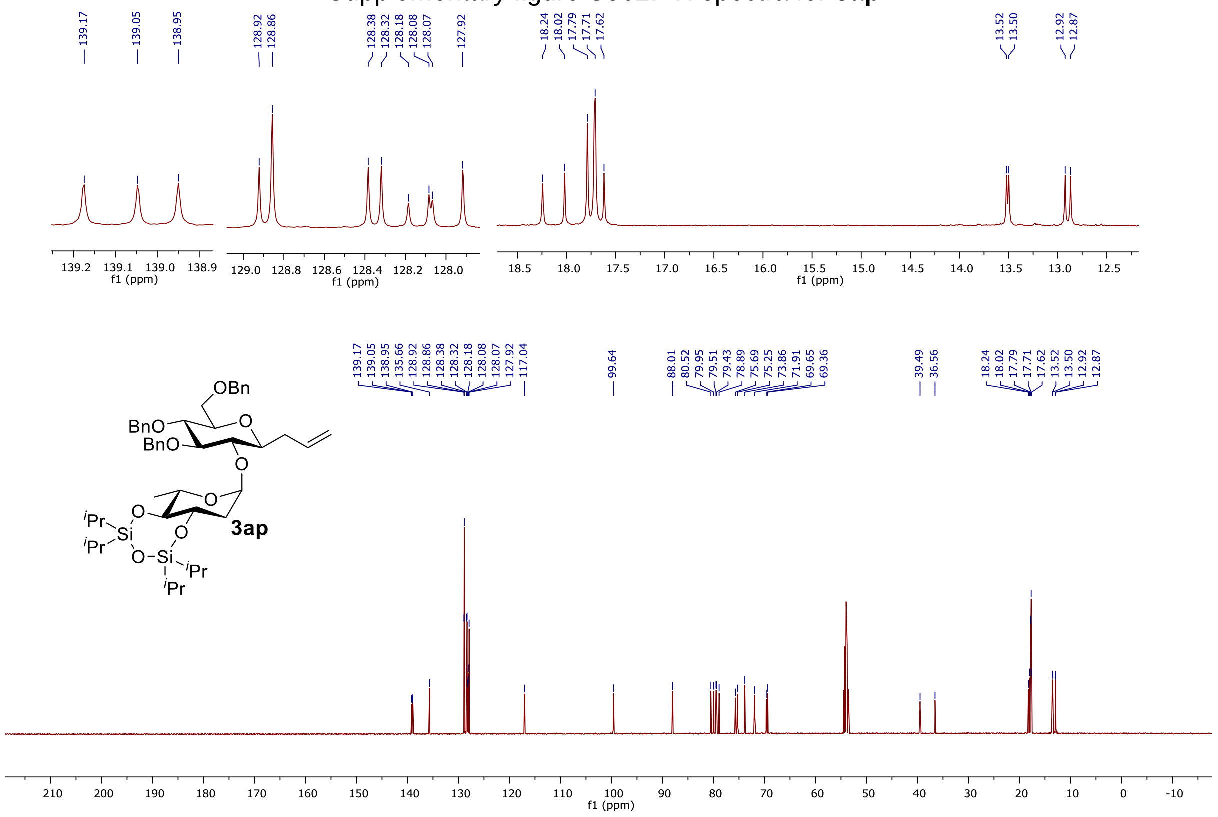

Supplementary figure S363: ^{13}C spectra for **3ap**

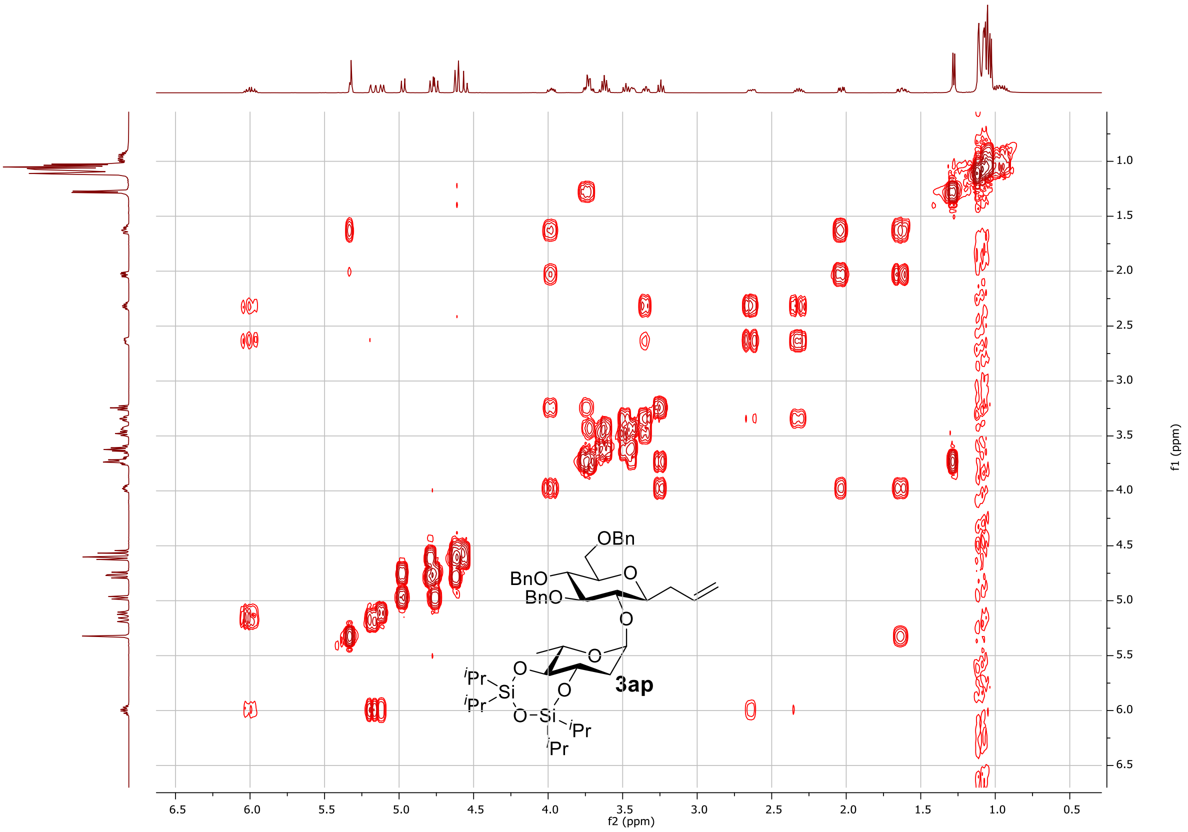

Supplementary figure S364: COSY spectra for **3ap**

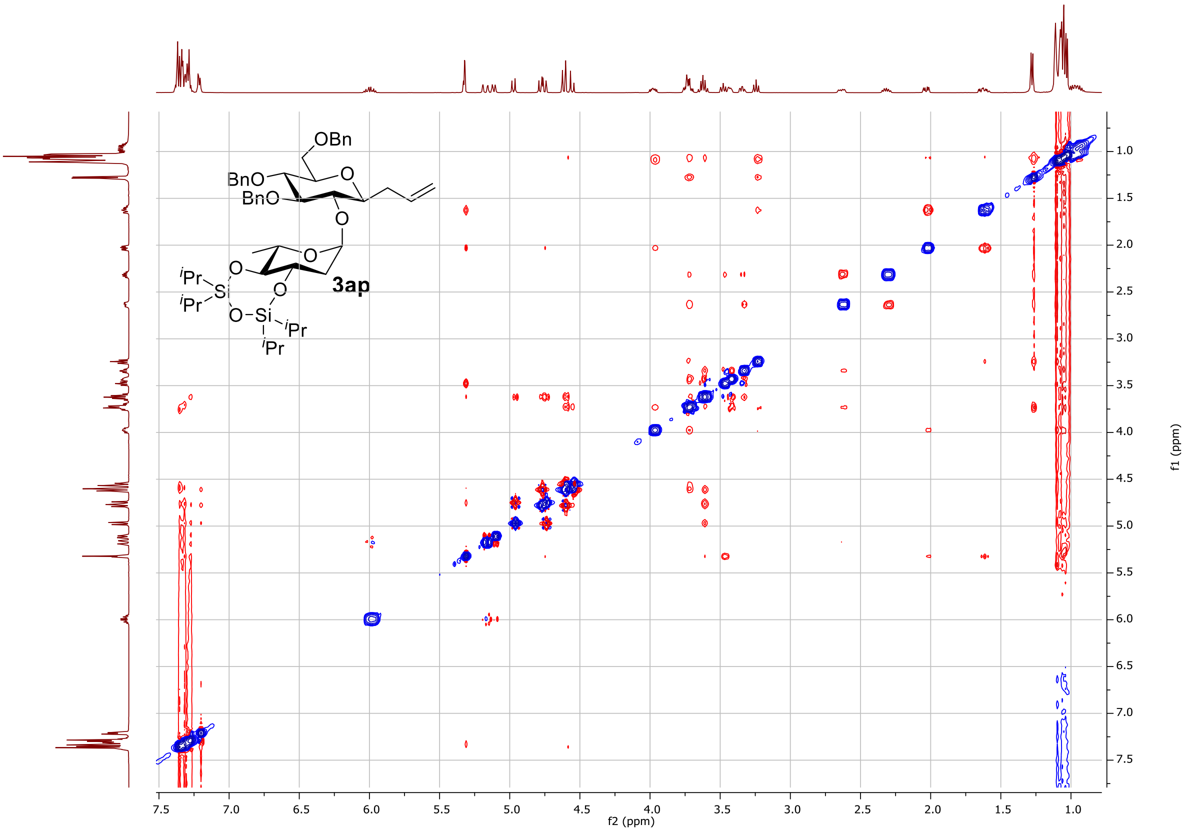

Supplementary figure S365: NOESY spectra for **3ap**

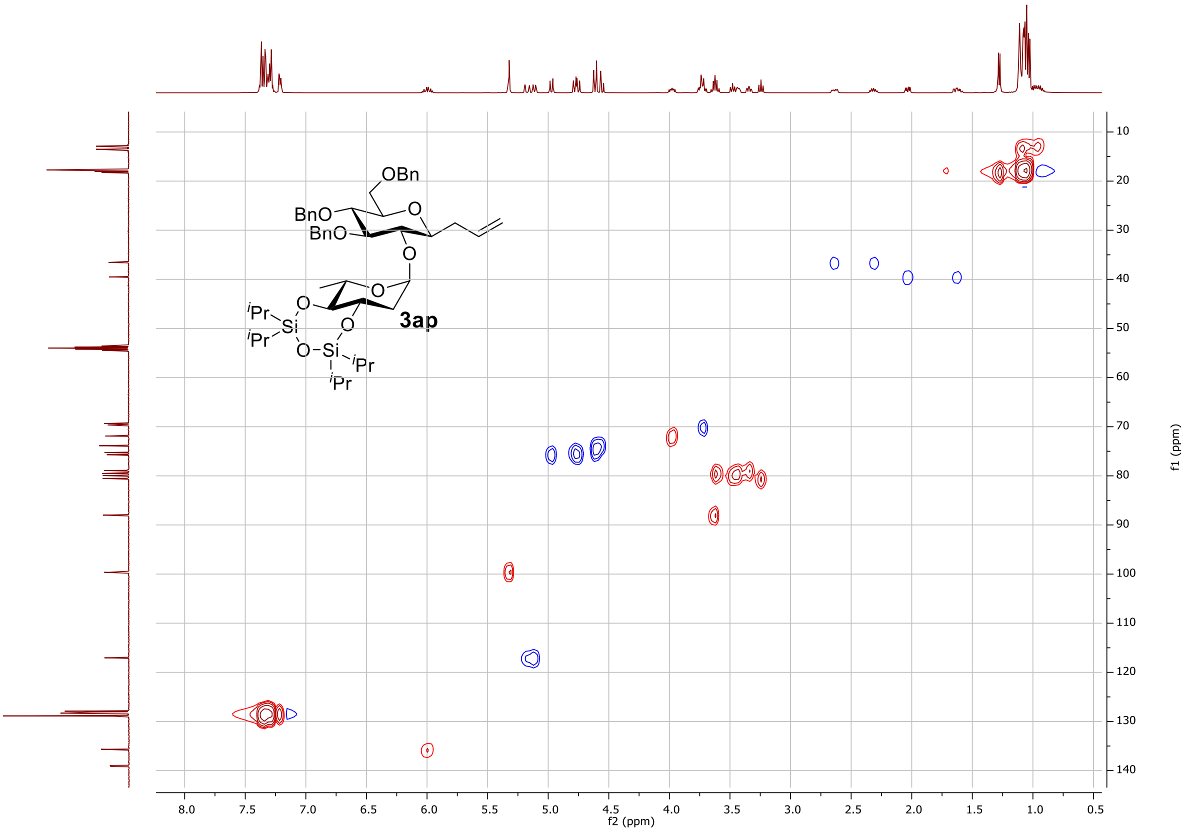

Supplementary figure S366: HSQC spectra for **3ap**

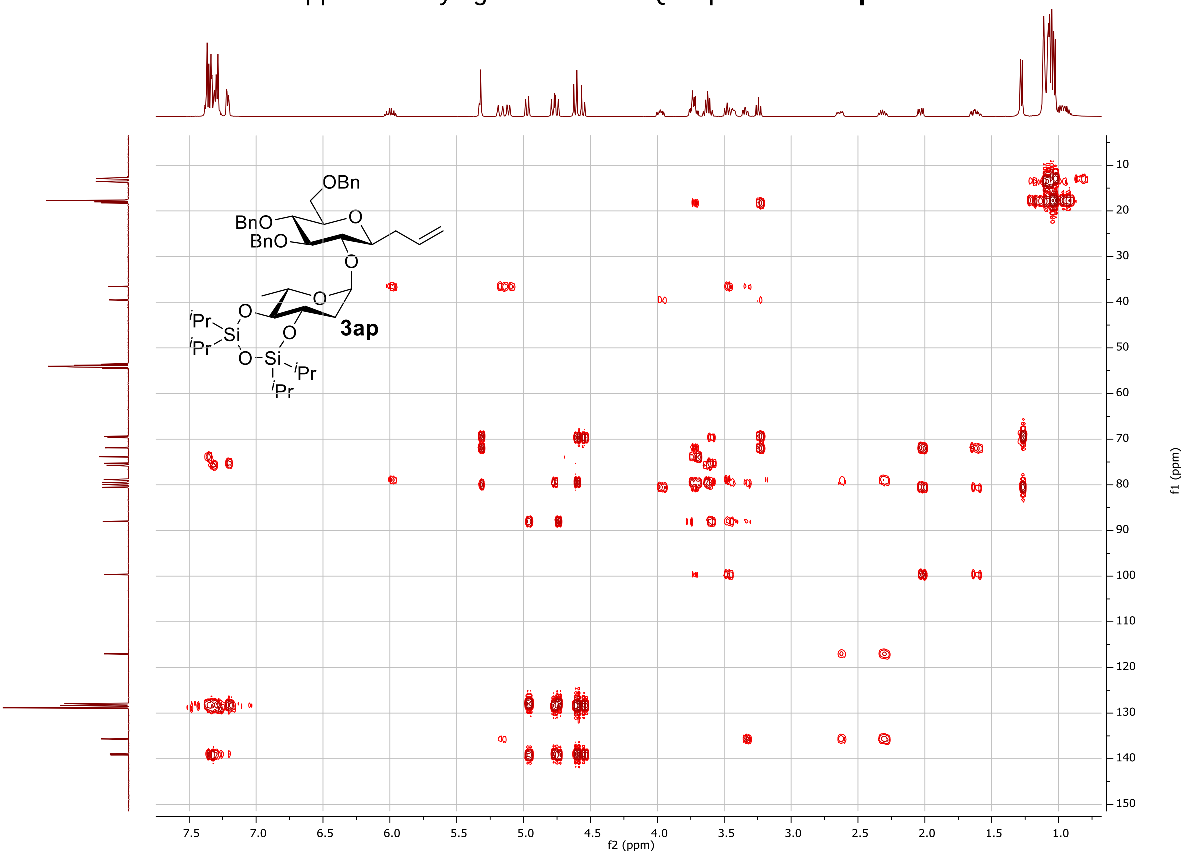

Supplementary figure S367: HMBC spectra for **3ap**

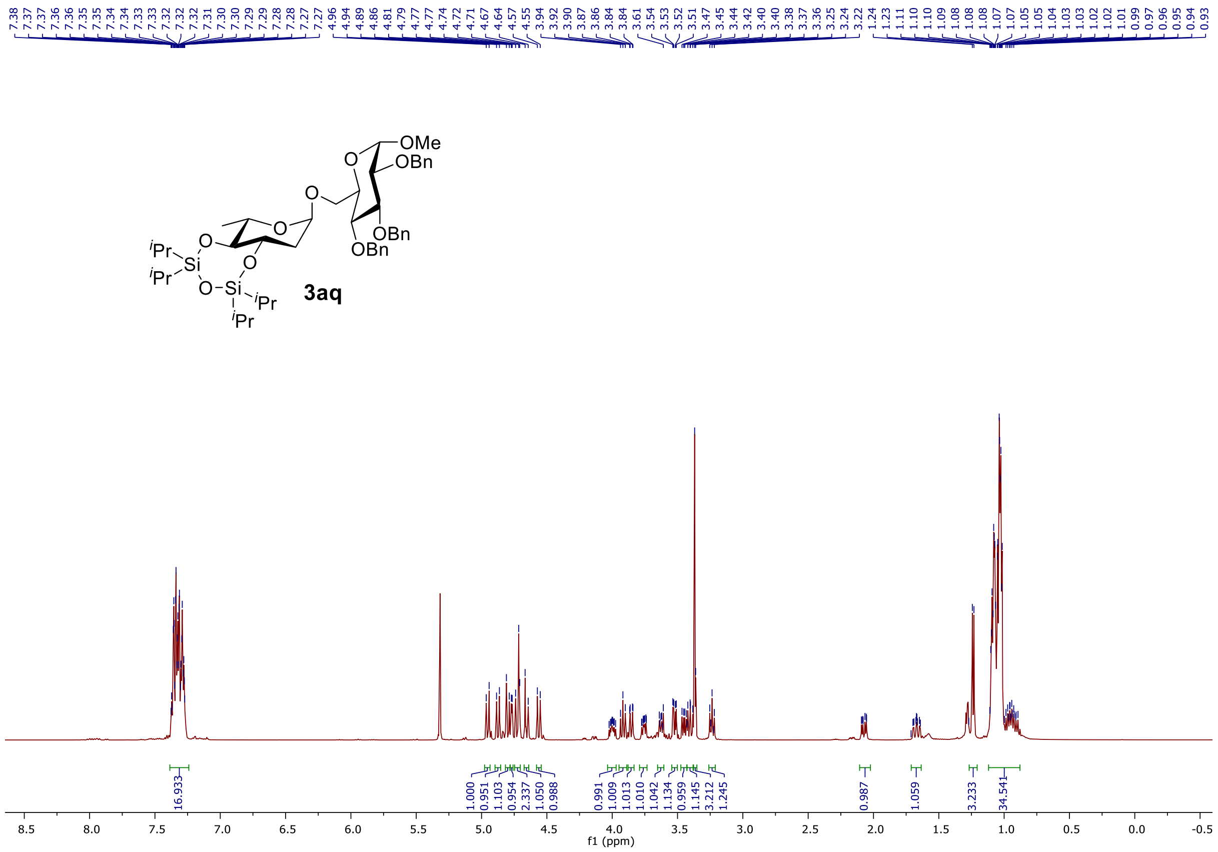

Supplementary figure S368: ^{1}H spectra for **3aq**

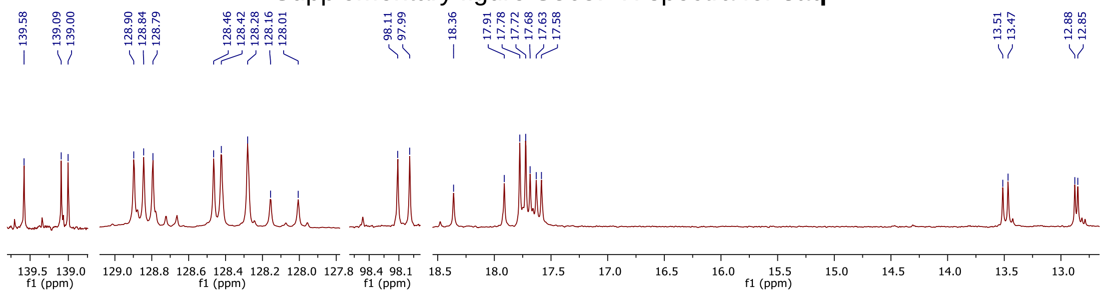

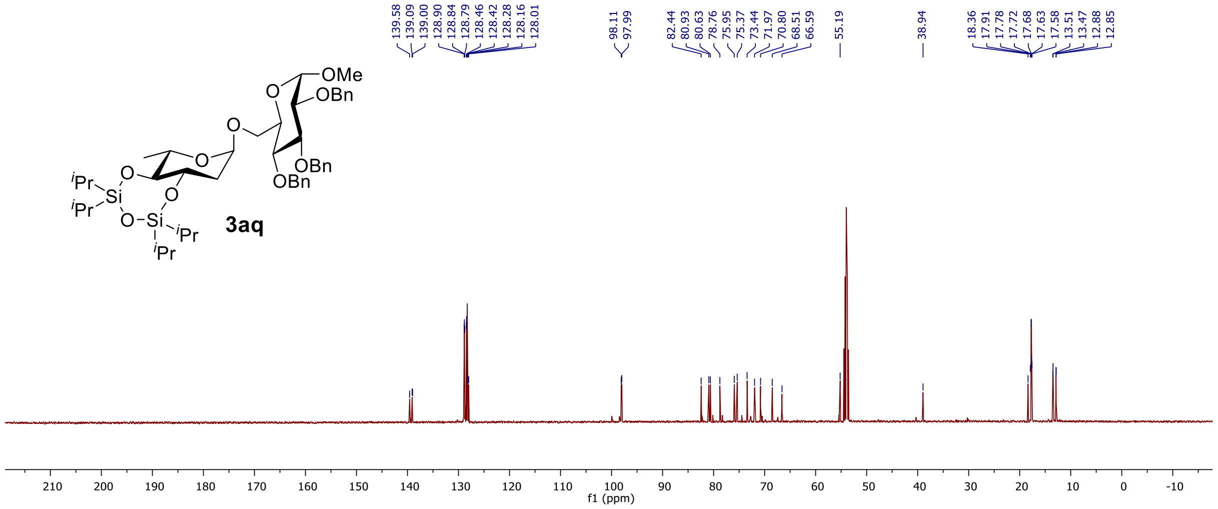

Supplementary figure S369: ^{13}C spectra for **3aq**

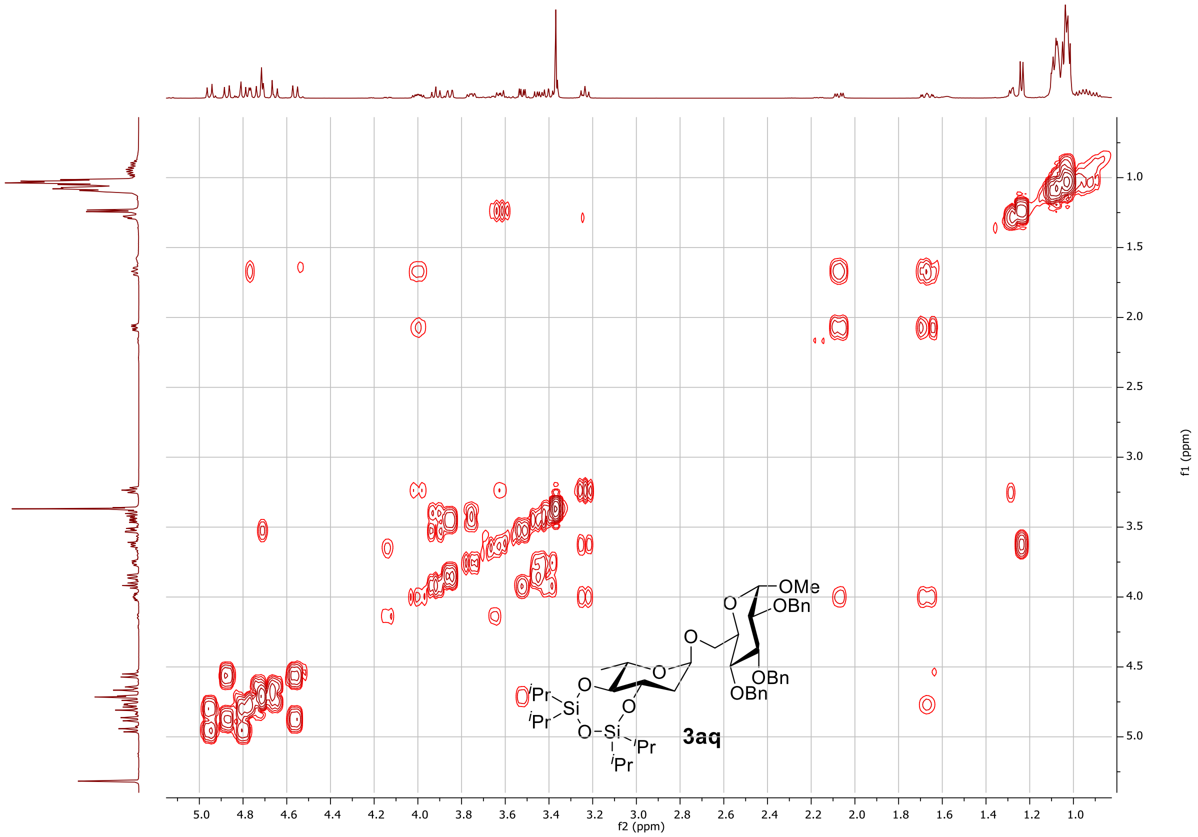

Supplementary figure S370: COSY spectra for **3aq**

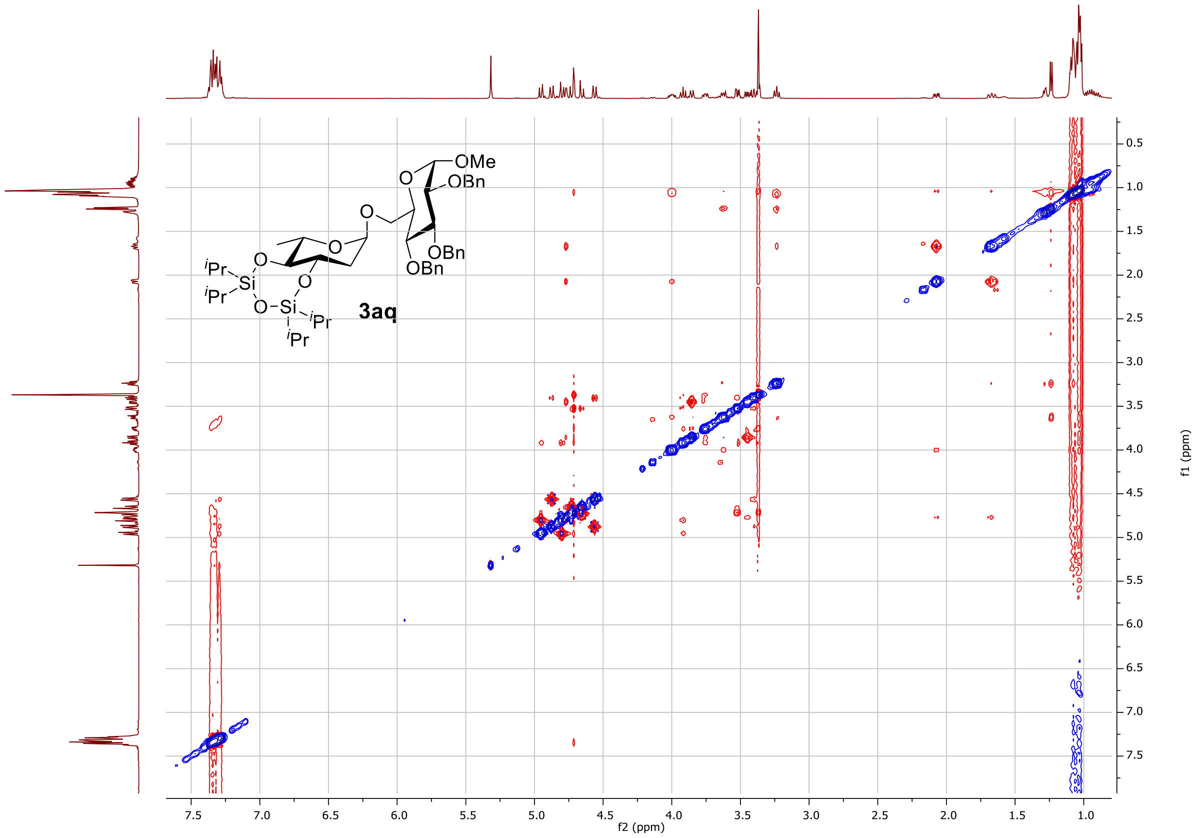

Supplementary figure S371: NOESY spectra for **3aq**

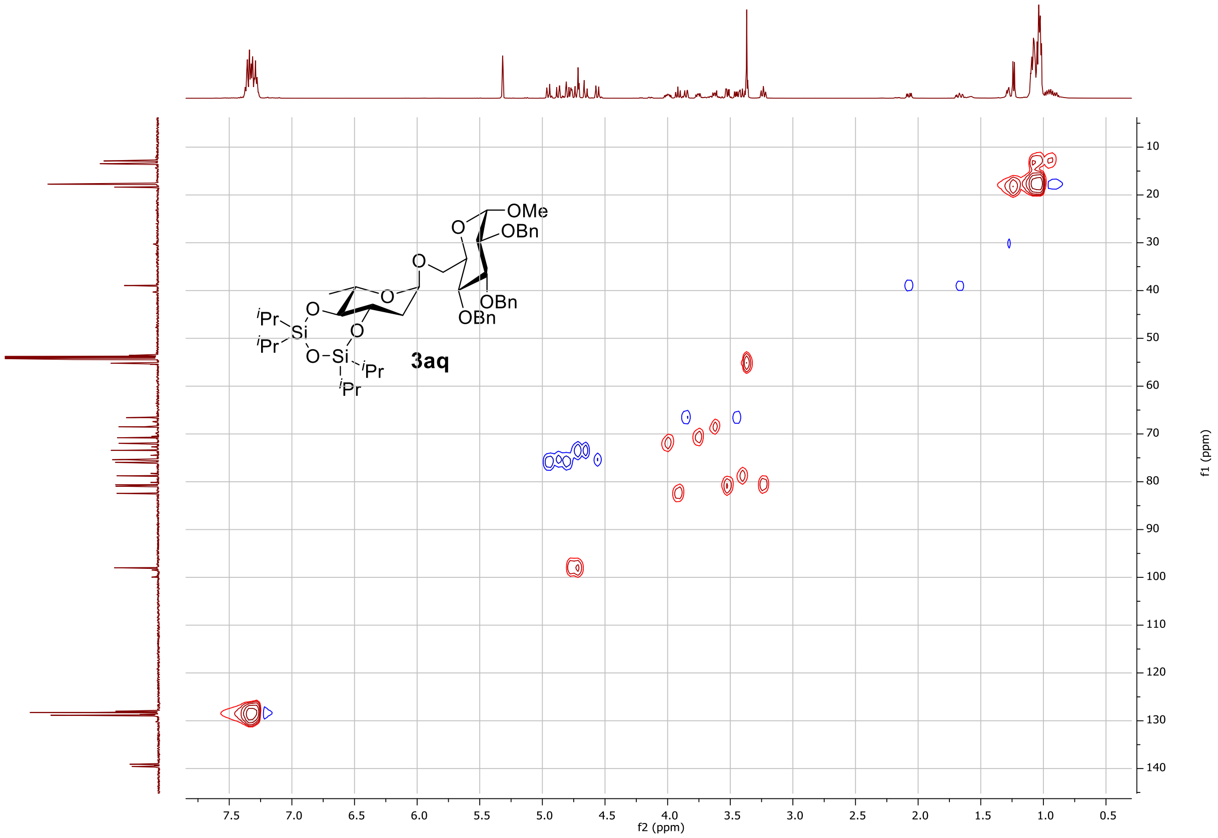

Supplementary figure S372: HSQC spectra for **3aq**

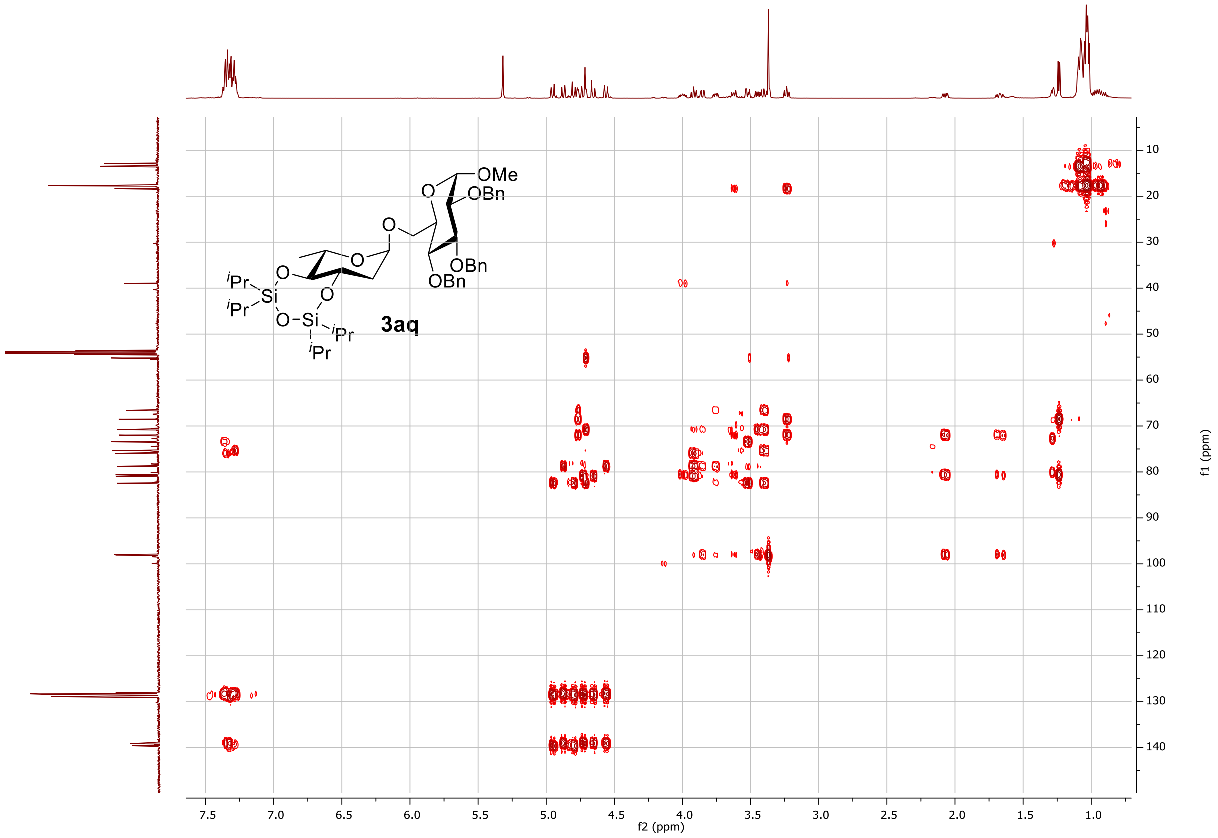

Supplementary figure S373: HMBC spectra for **3aq**

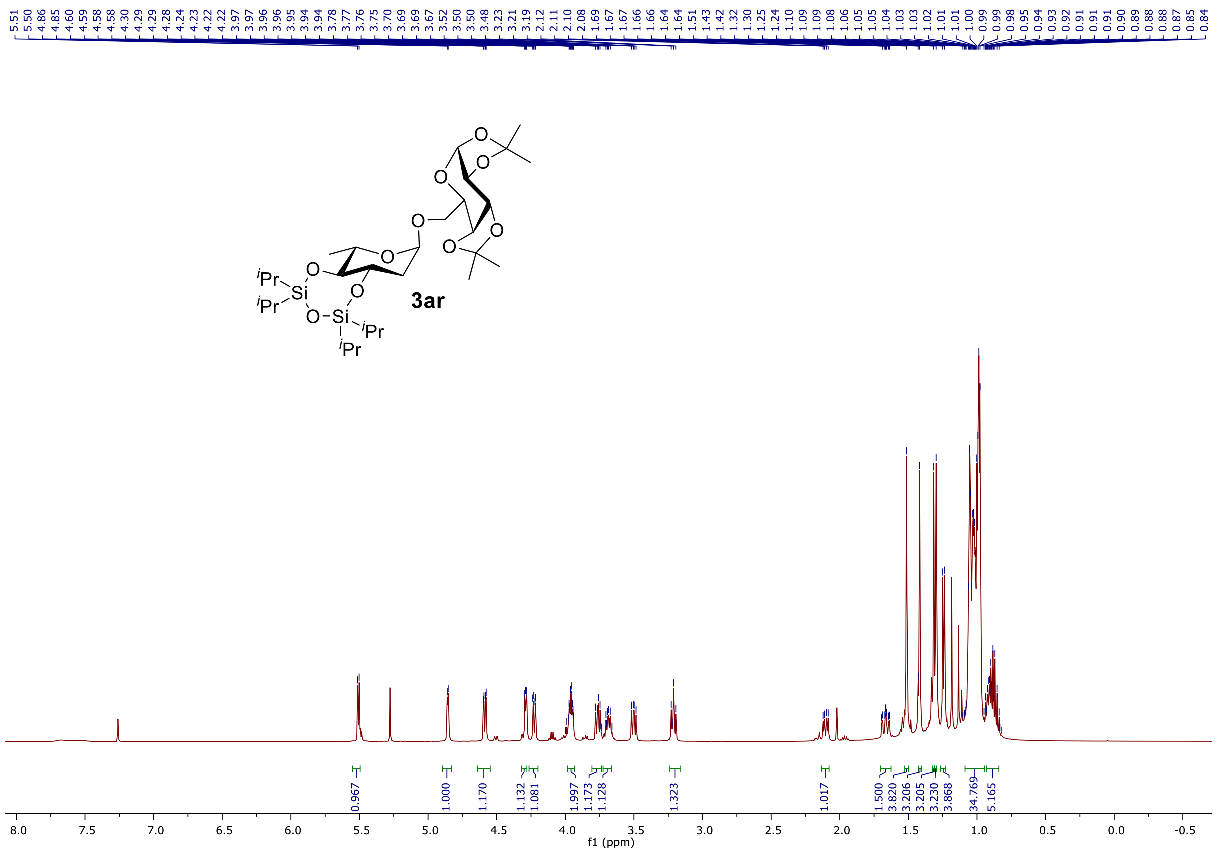

Supplementary figure S374: ^{1}H spectra for **3ar**

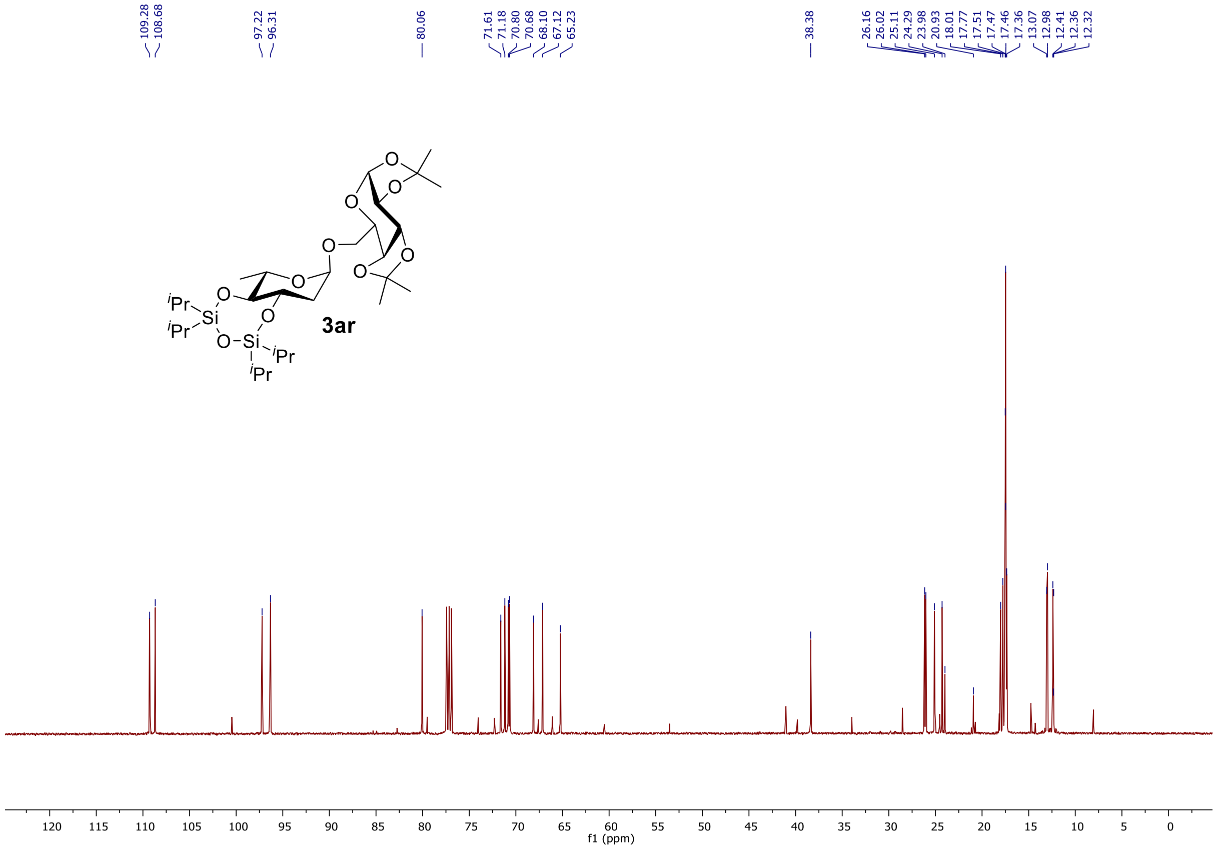

Supplementary figure S375: ^{13}C spectra for **3ar**

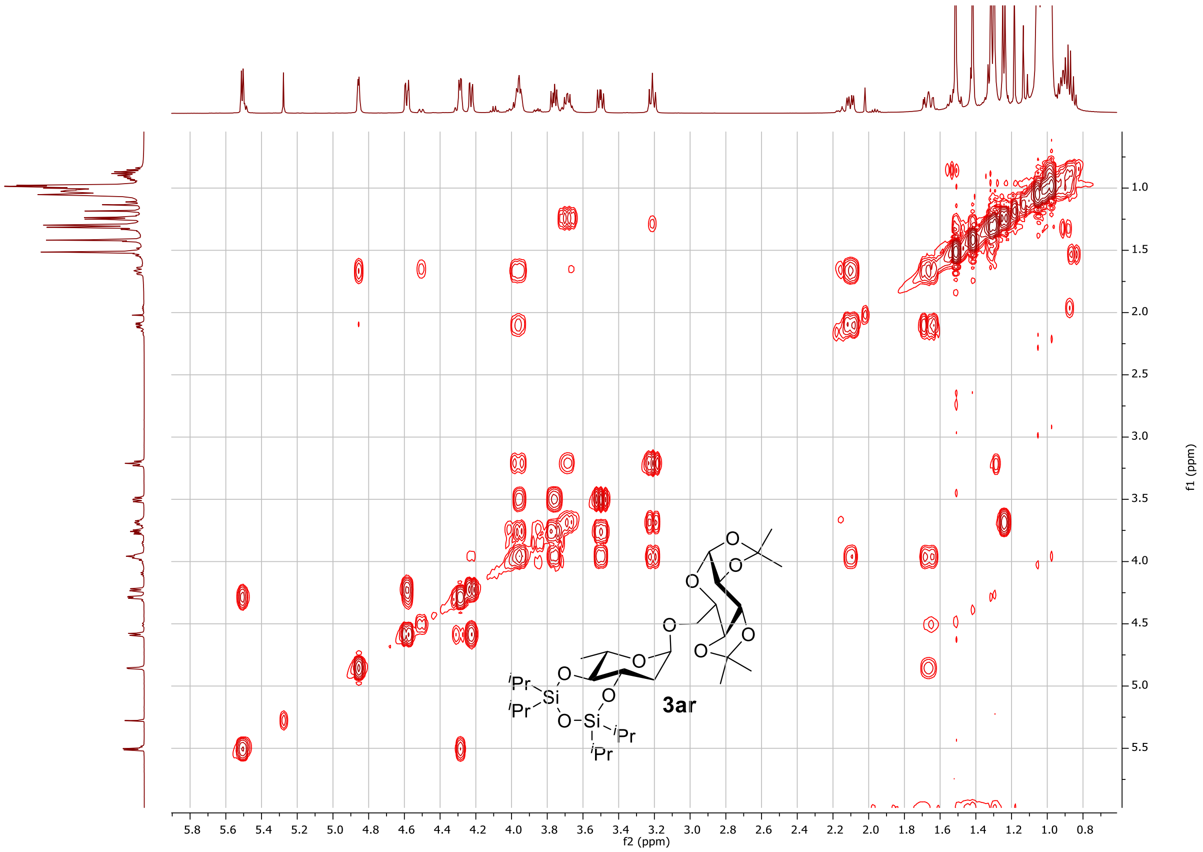

Supplementary figure S376: COSY spectra for **3ar**

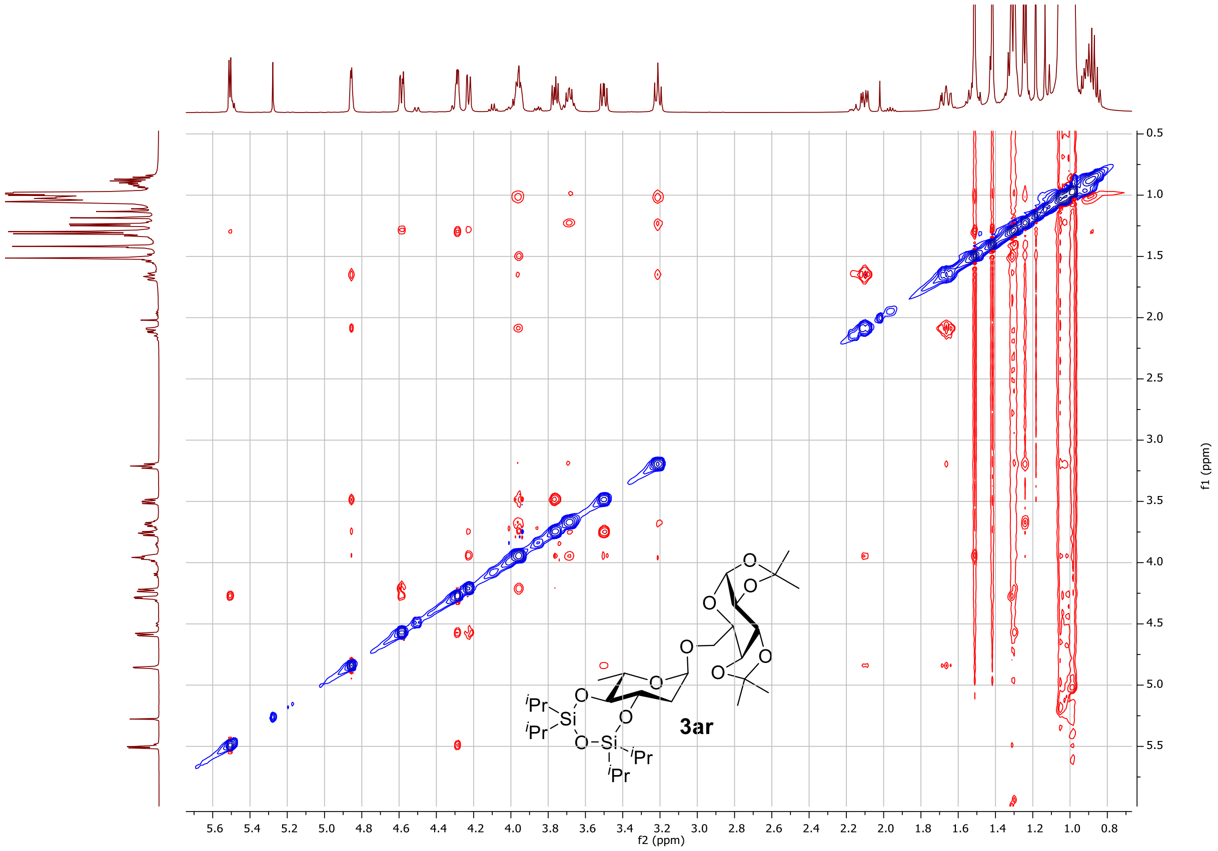

Supplementary figure S377: NOESY spectra for **3ar**

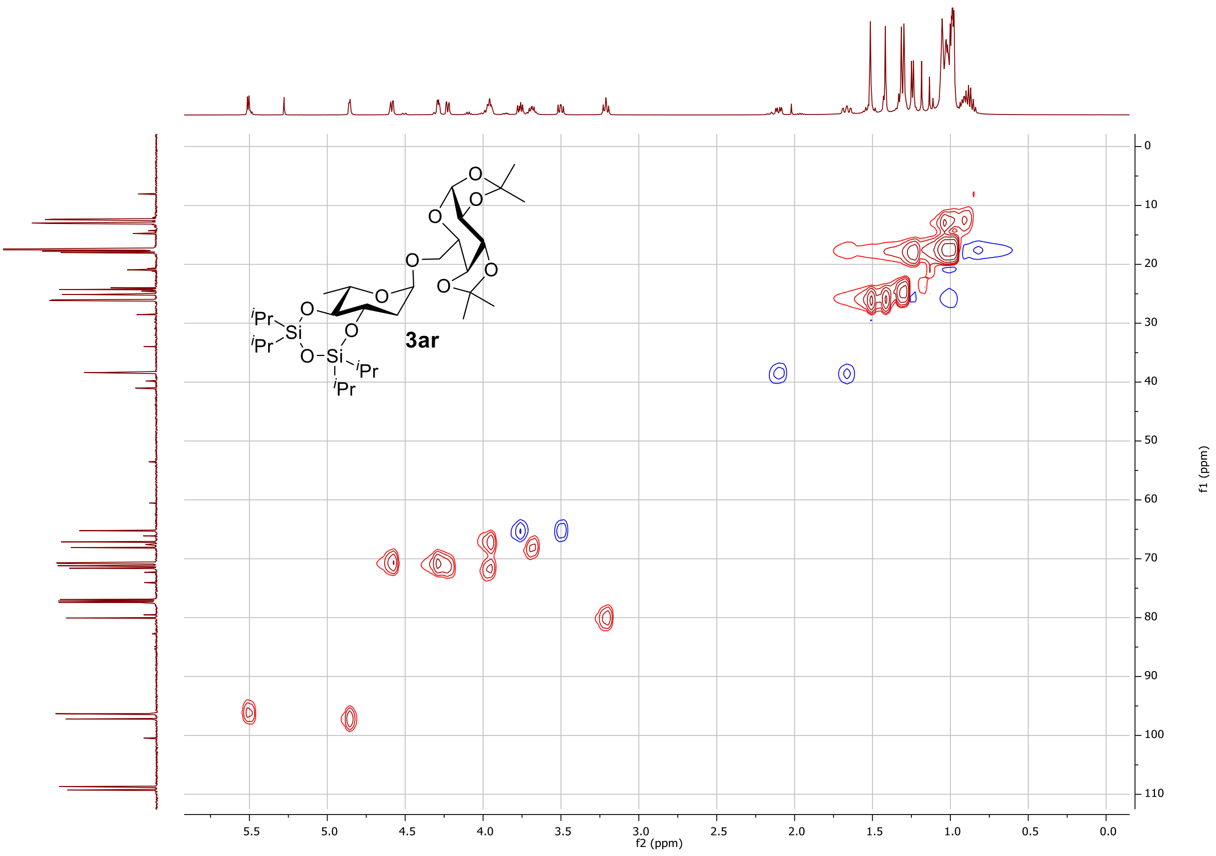

Supplementary figure S378: HSQC spectra for **3ar**

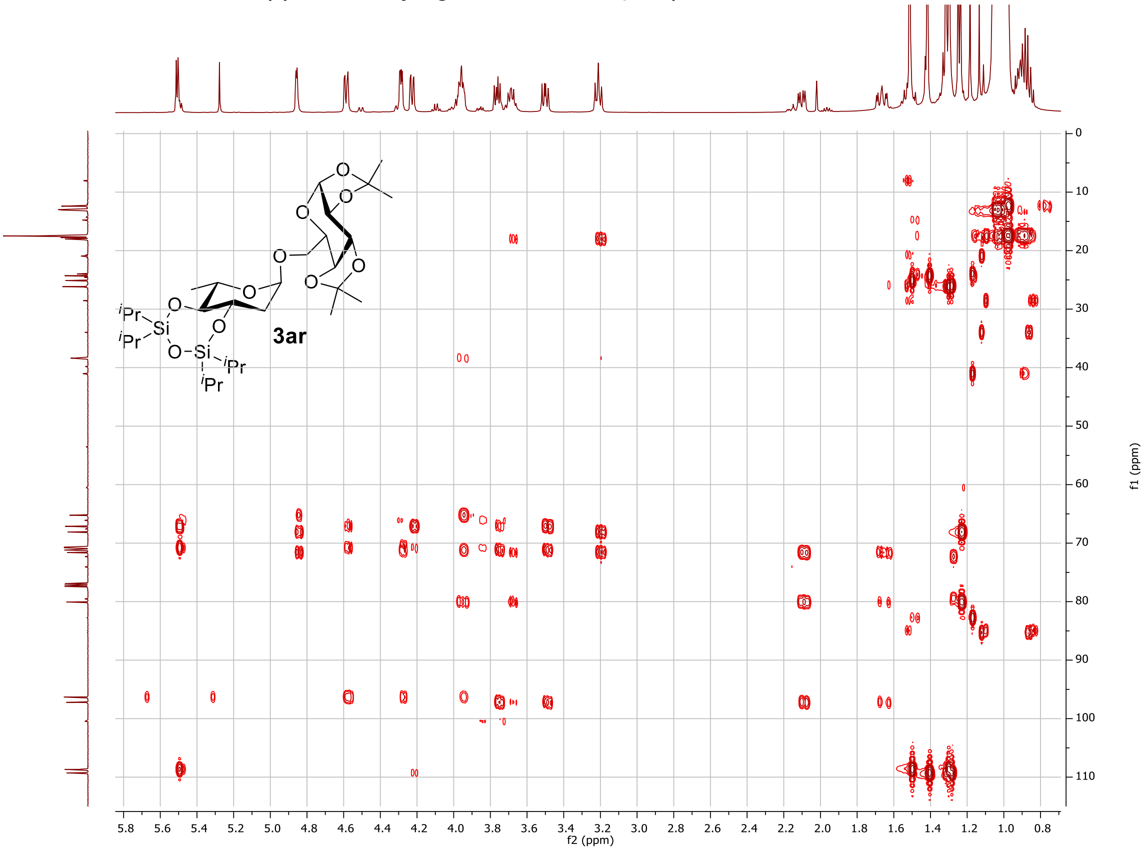

Supplementary figure S379: HMBC spectra for **3ar**

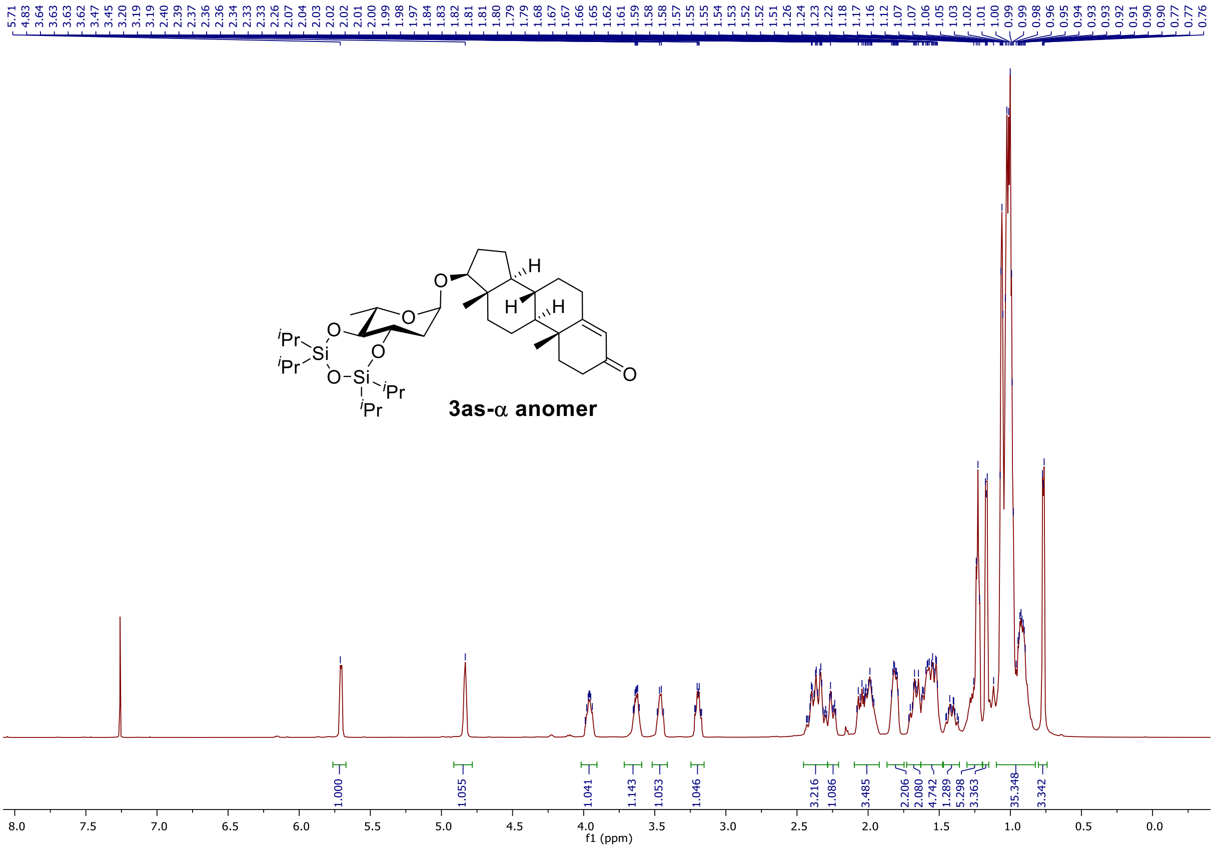

Supplementary figure S380: ^{1}H spectra for **3as-α anomer**

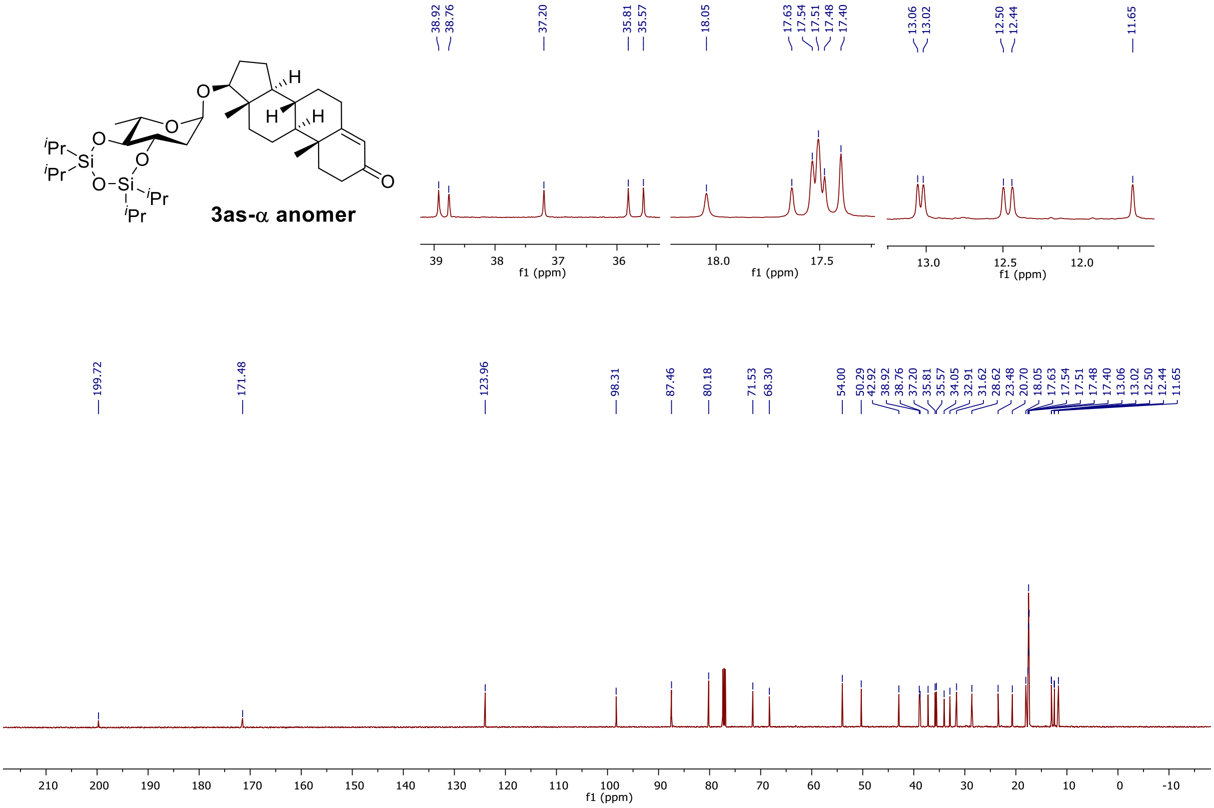

Supplementary figure S381: ^{13}C spectra for **3as-α anomer**

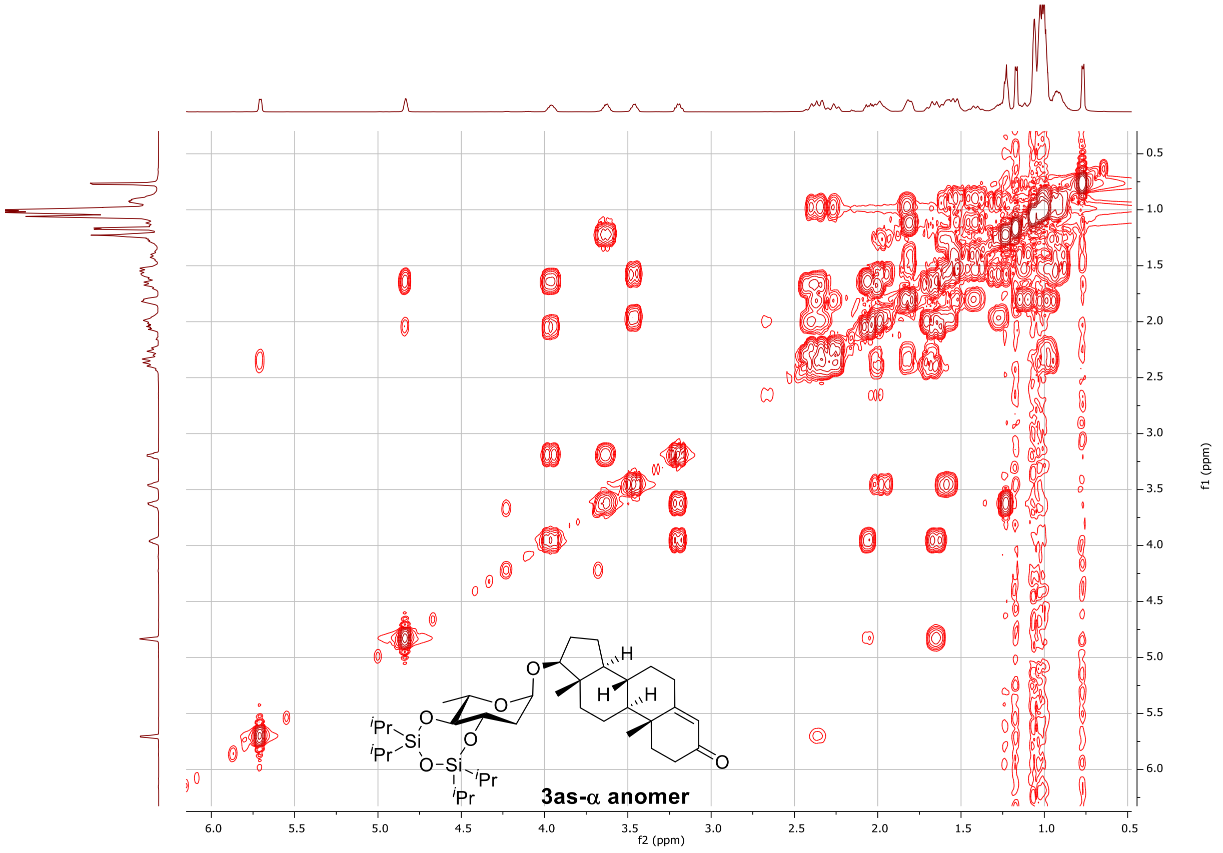

Supplementary figure S382: COSY spectra for **3as-α anomer**

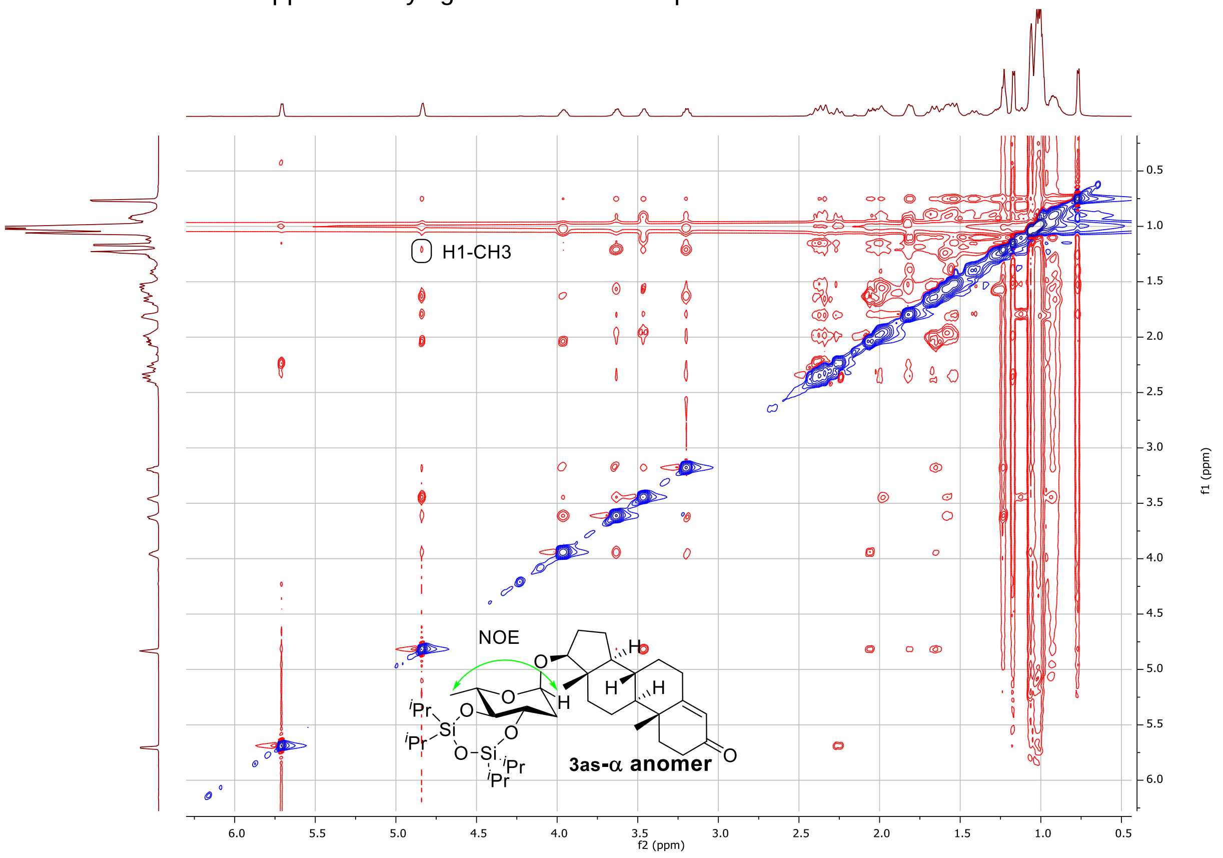

Supplementary figure S383: NOESY spectra for **3as-α anomer**

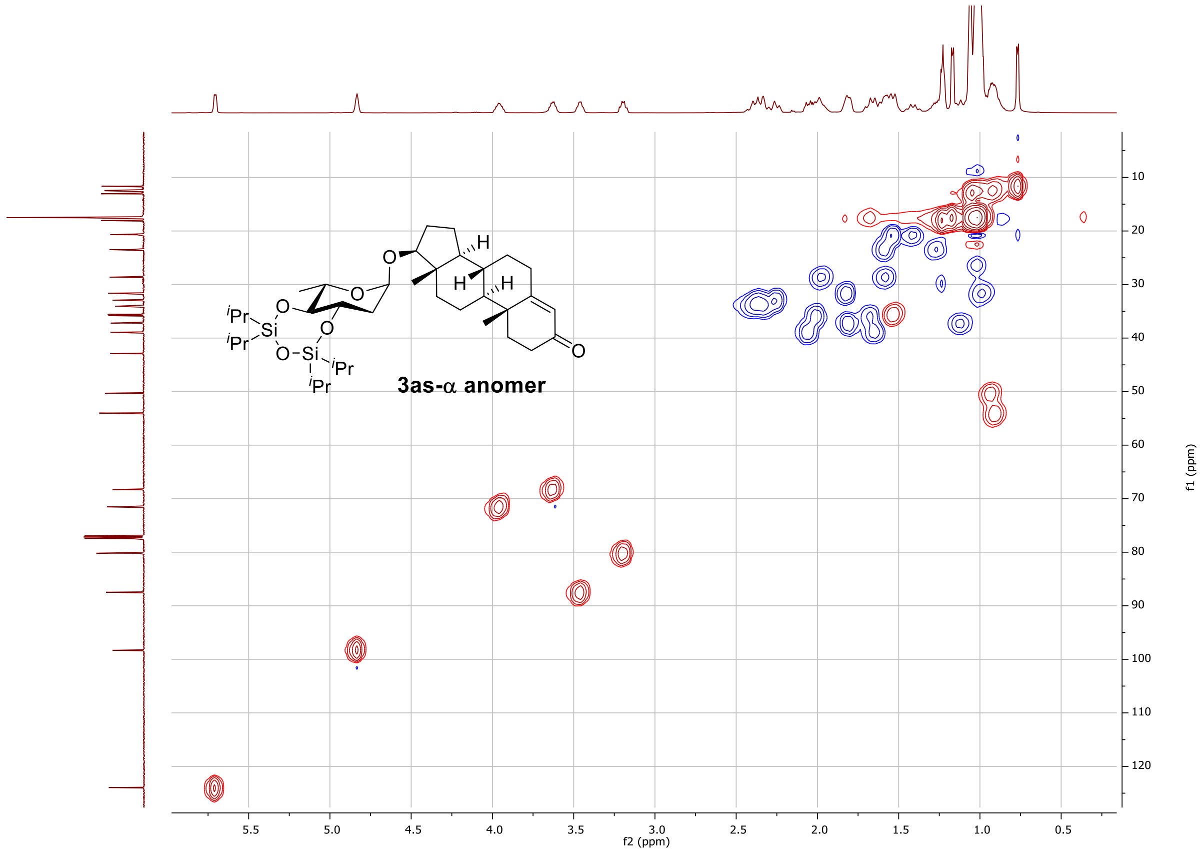

Supplementary figure S384: HSQC spectra for **3as-α anomer**

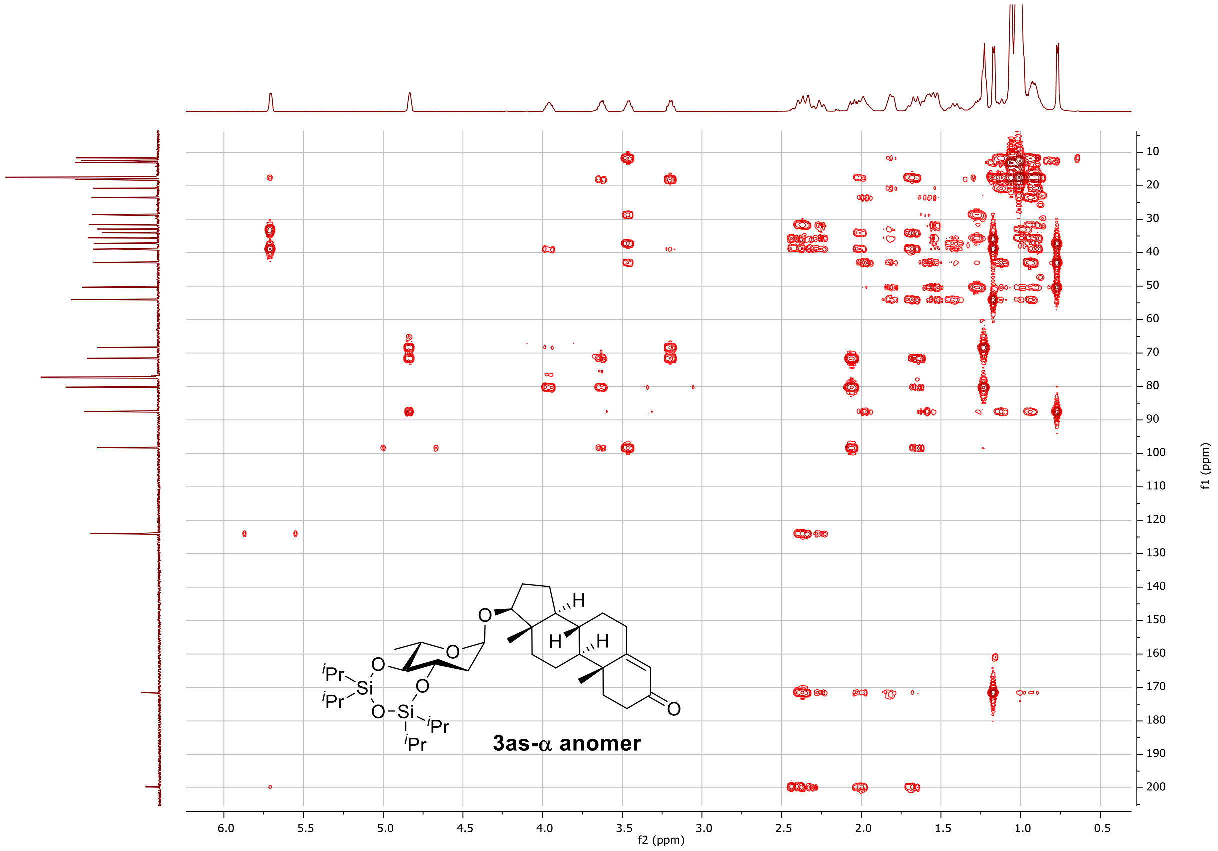

Supplementary figure S385: HMBC spectra for **3as-α anomer**

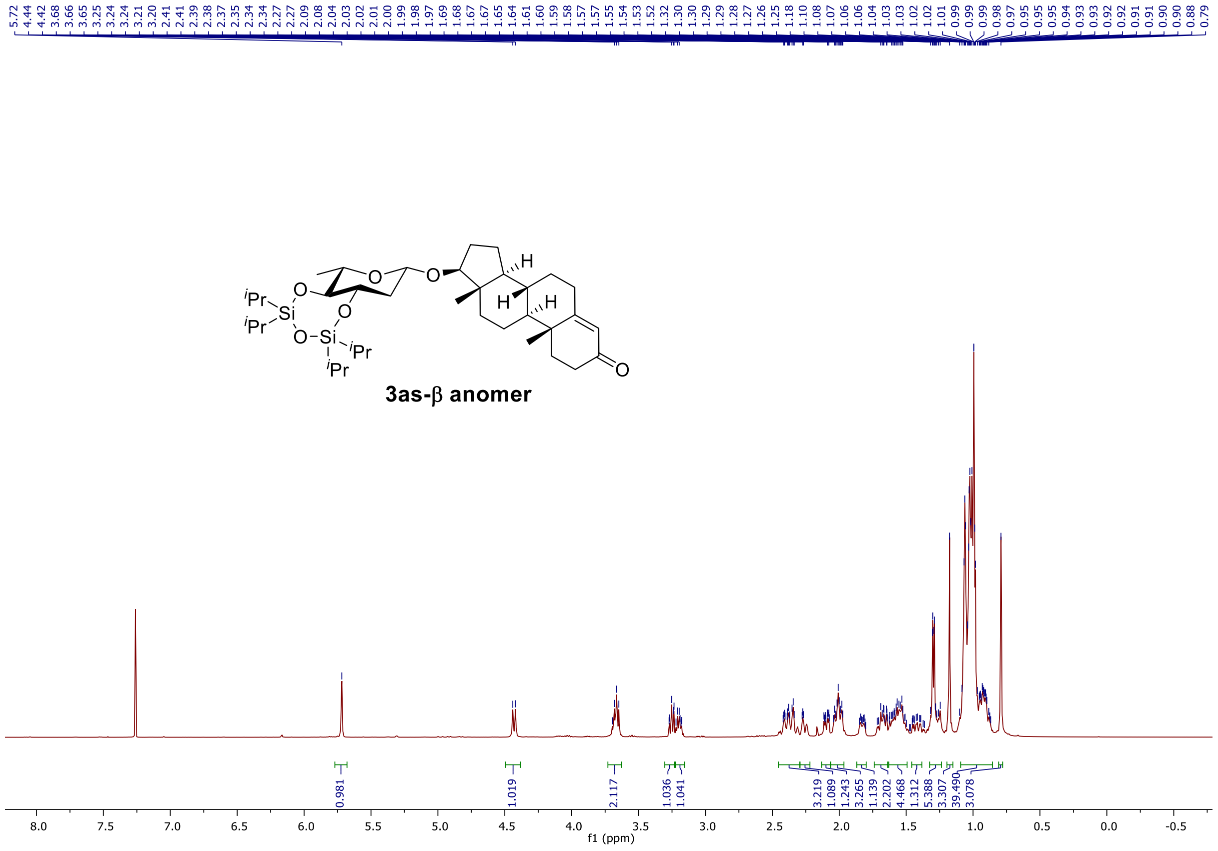

Supplementary figure S386: ^{1}H spectra for **3as-β anomer**

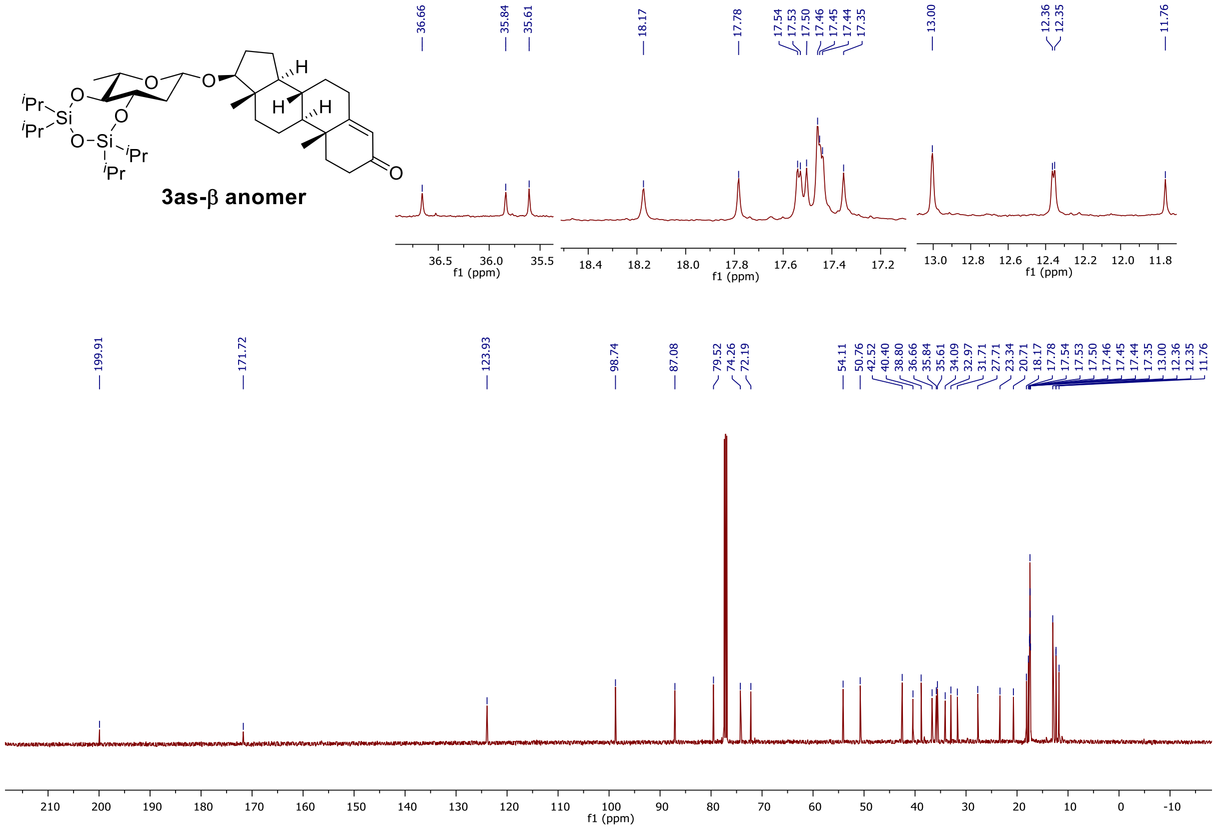

Supplementary figure S387: ^{13}C spectra for **3as-β anomer**

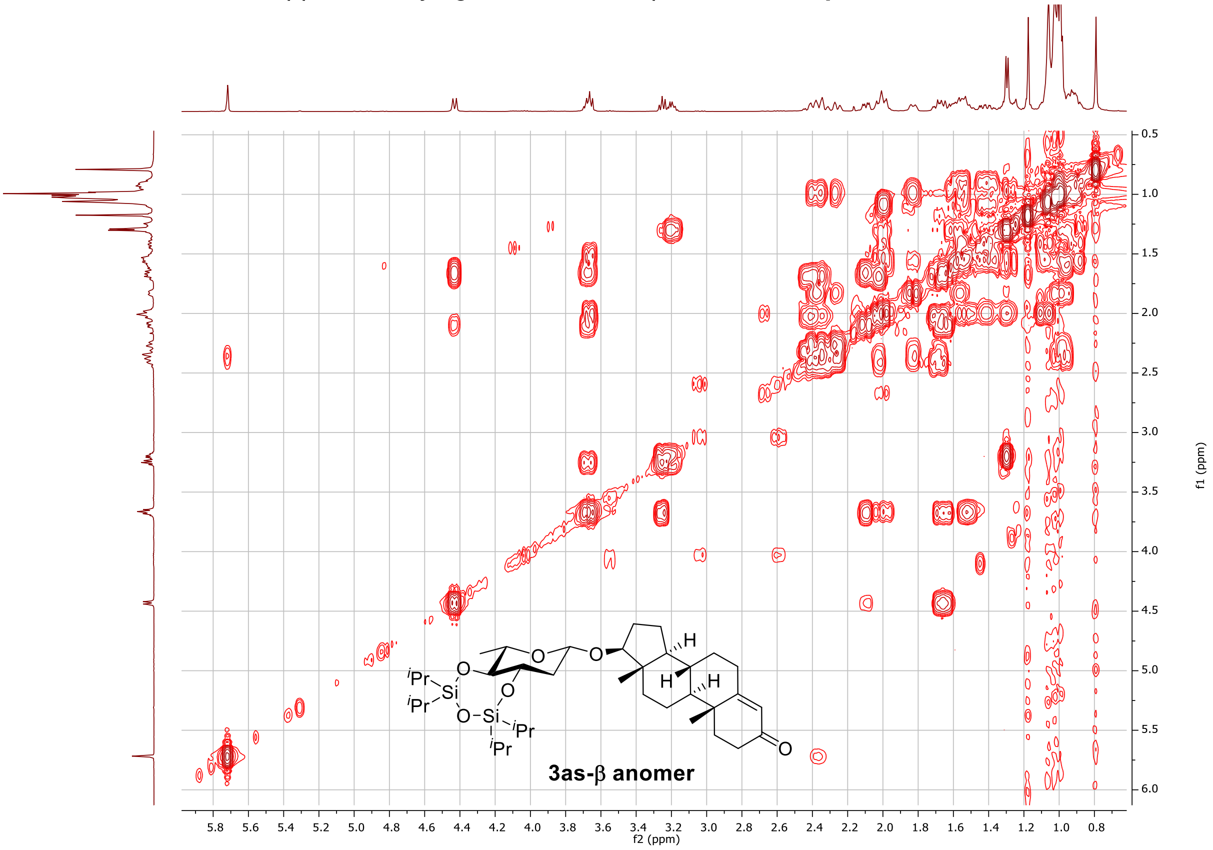

Supplementary figure S388: COSY spectra for **3as-β anomer**

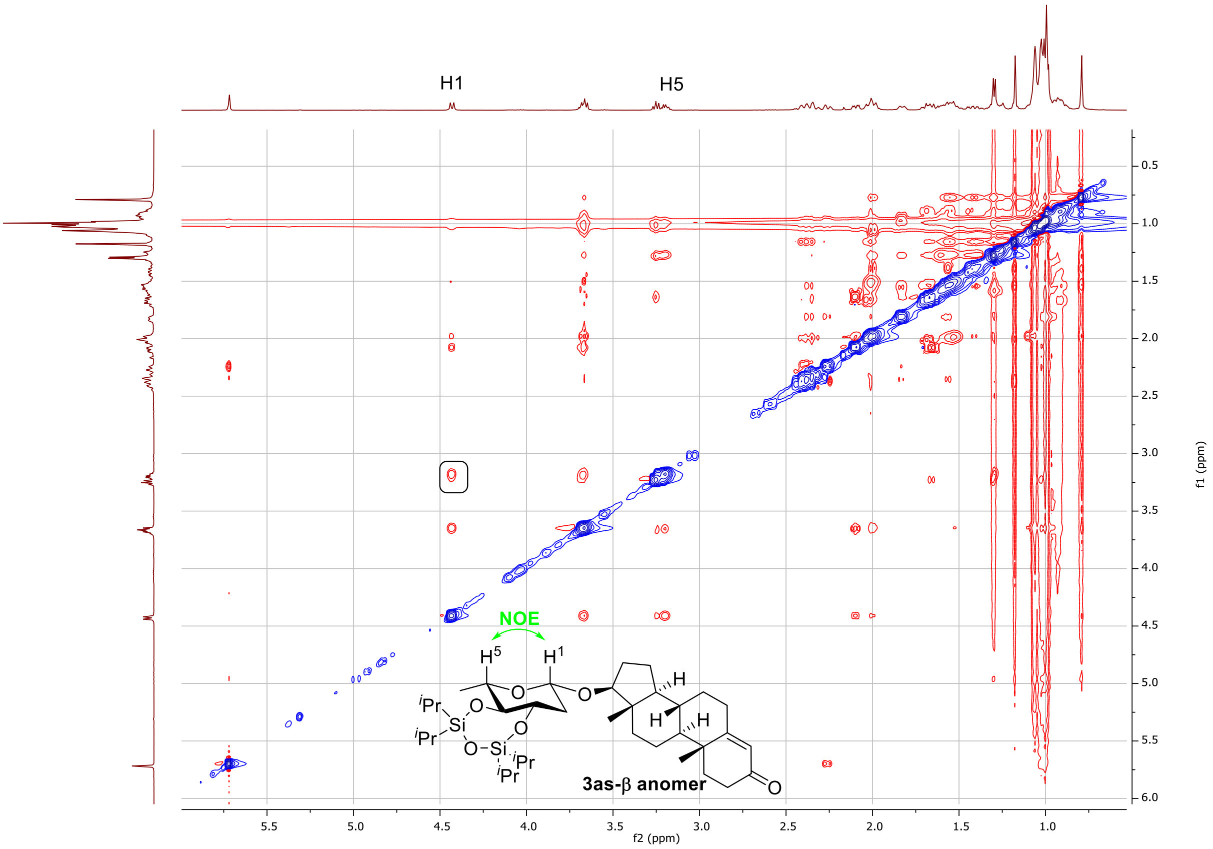

Supplementary figure S389: NOESY spectra for **3as-β anomer**

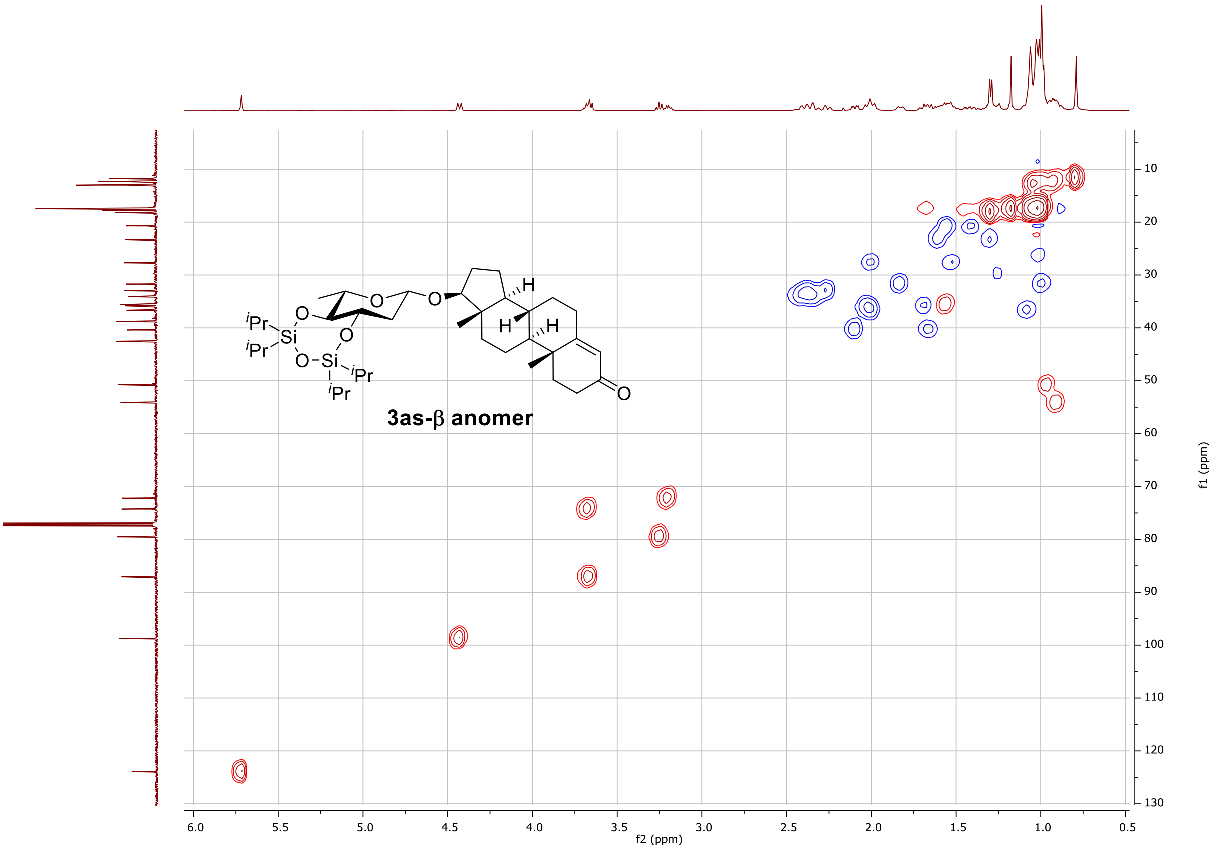

Supplementary figure S390: HSQC spectra for **3as-β anomer**

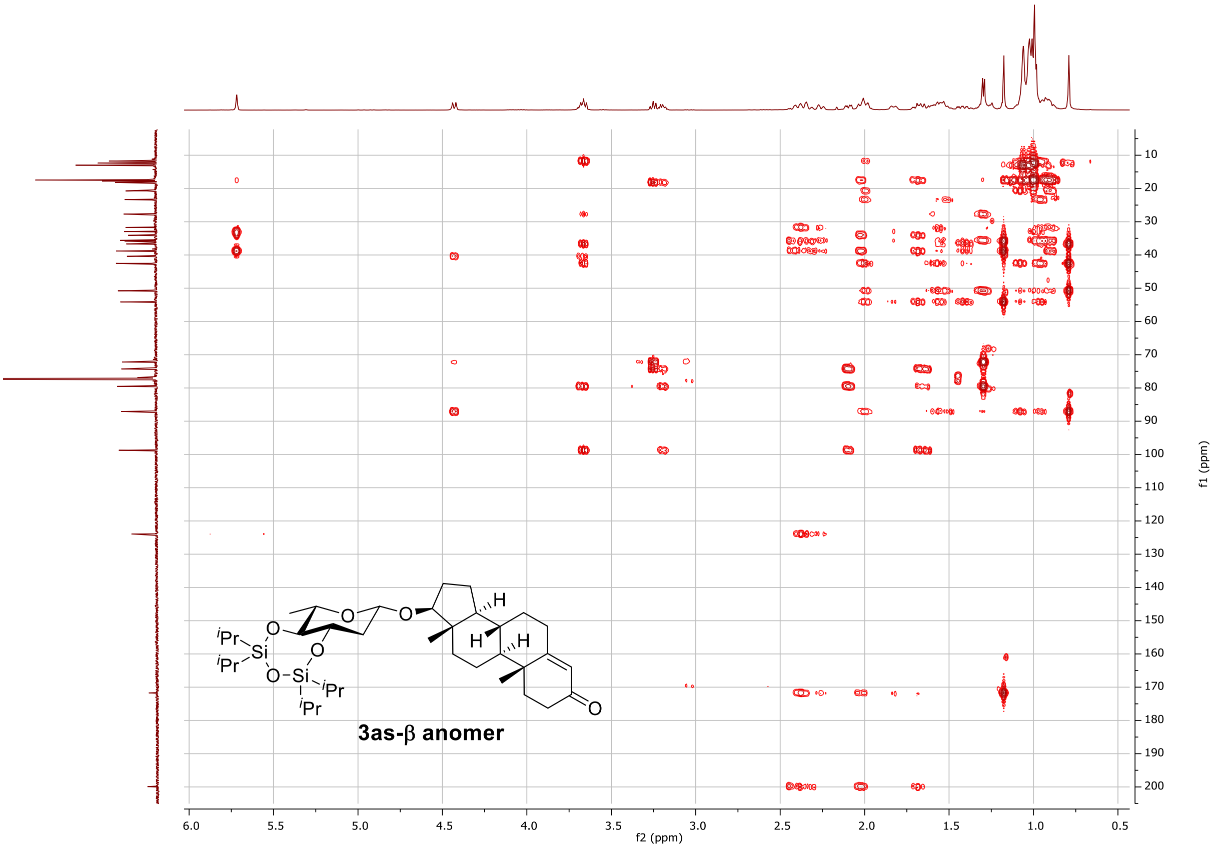

Supplementary figure S391: HMBC spectra for **3as-β anomer**

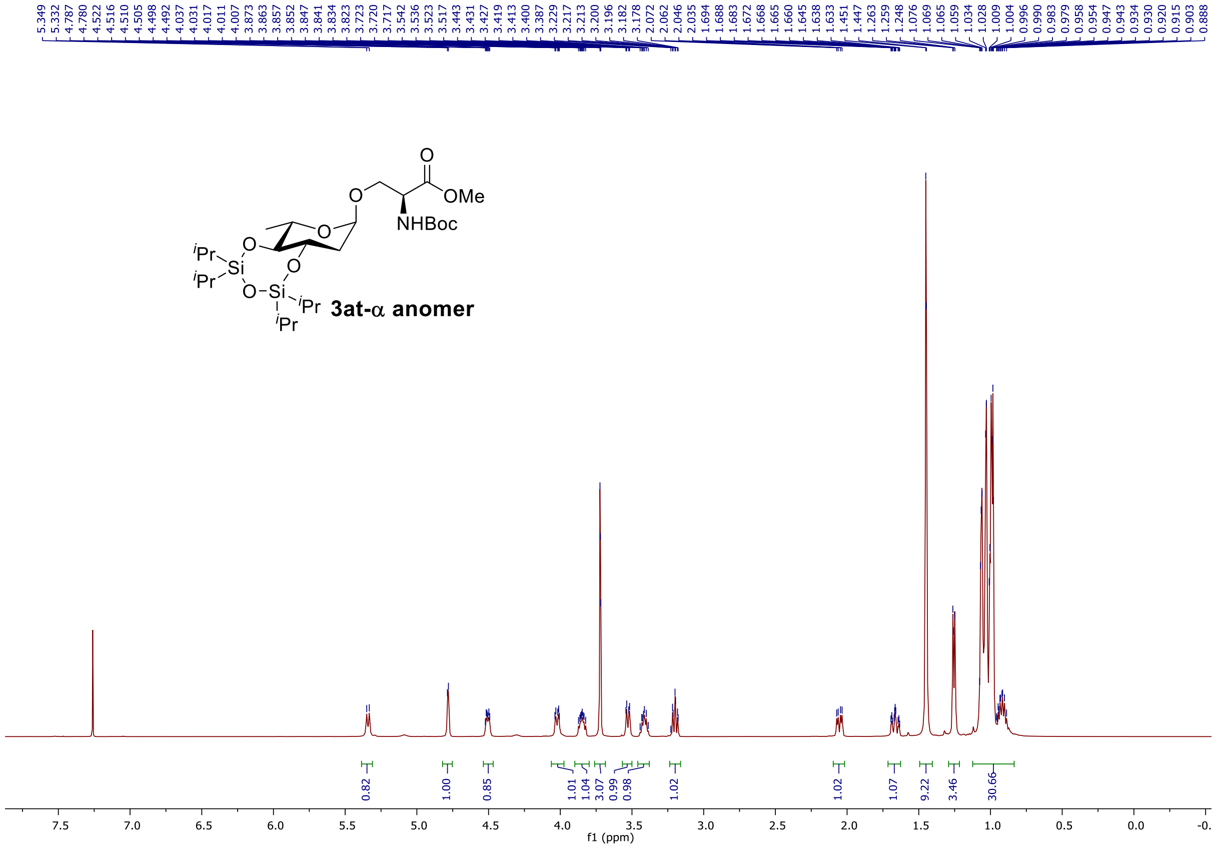

Supplementary figure S392: ^{1}H spectra for **3at-α anomer**

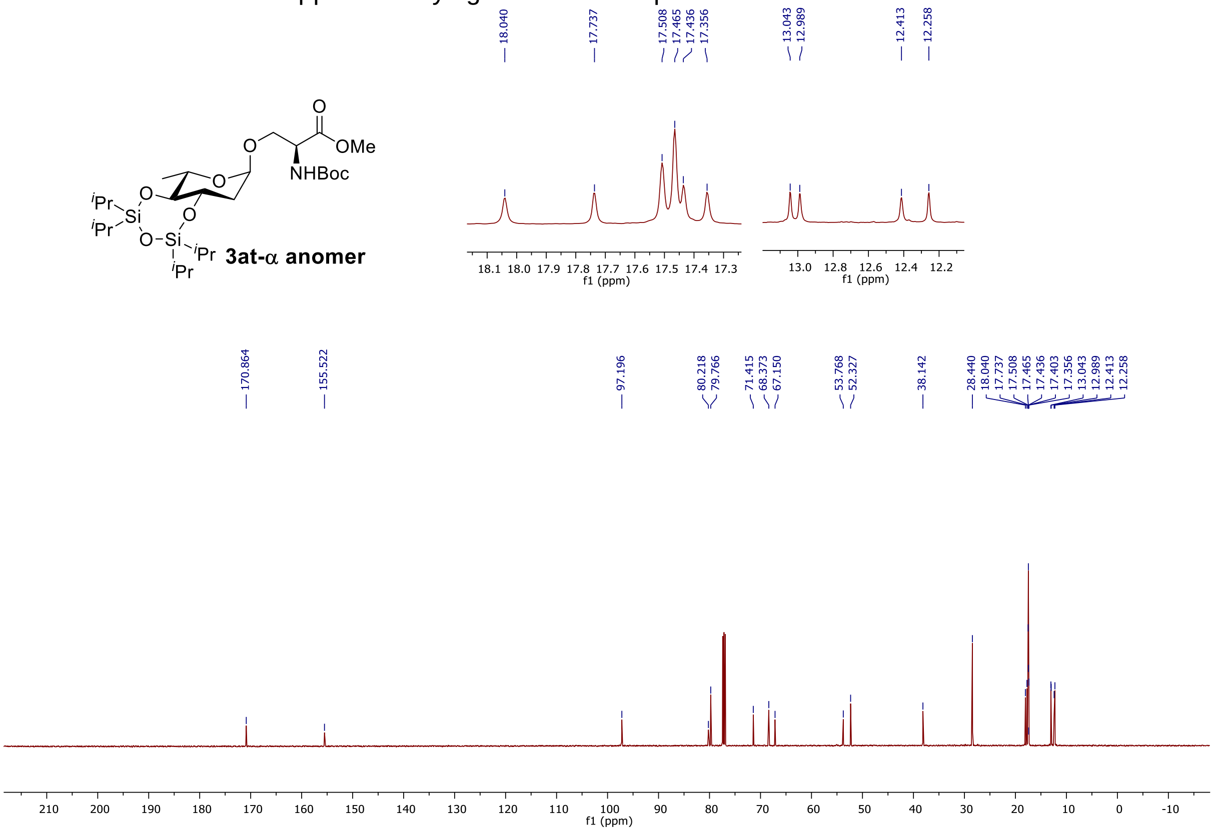

Supplementary figure S393: ^{13}C spectra for **3at-α anomer**

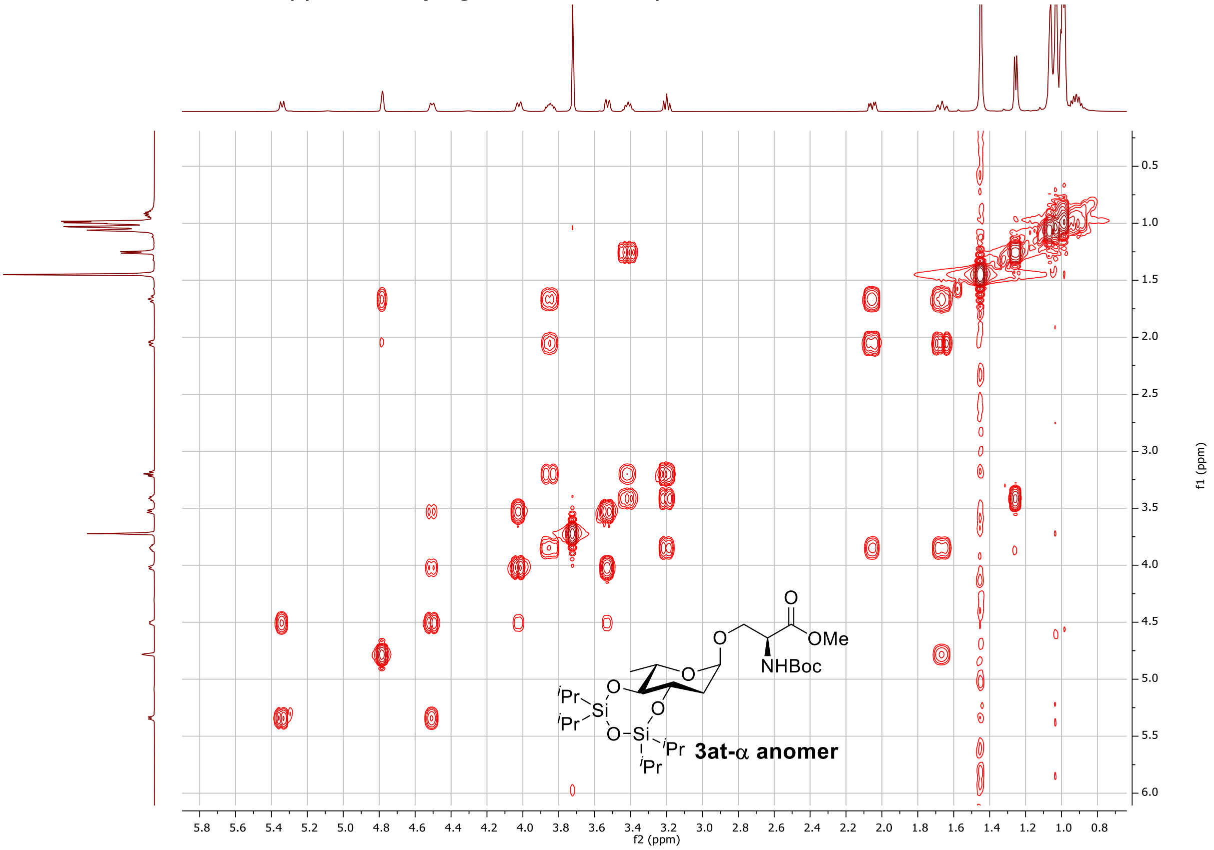

Supplementary figure S394: COSY spectra for **3at-α anomer**

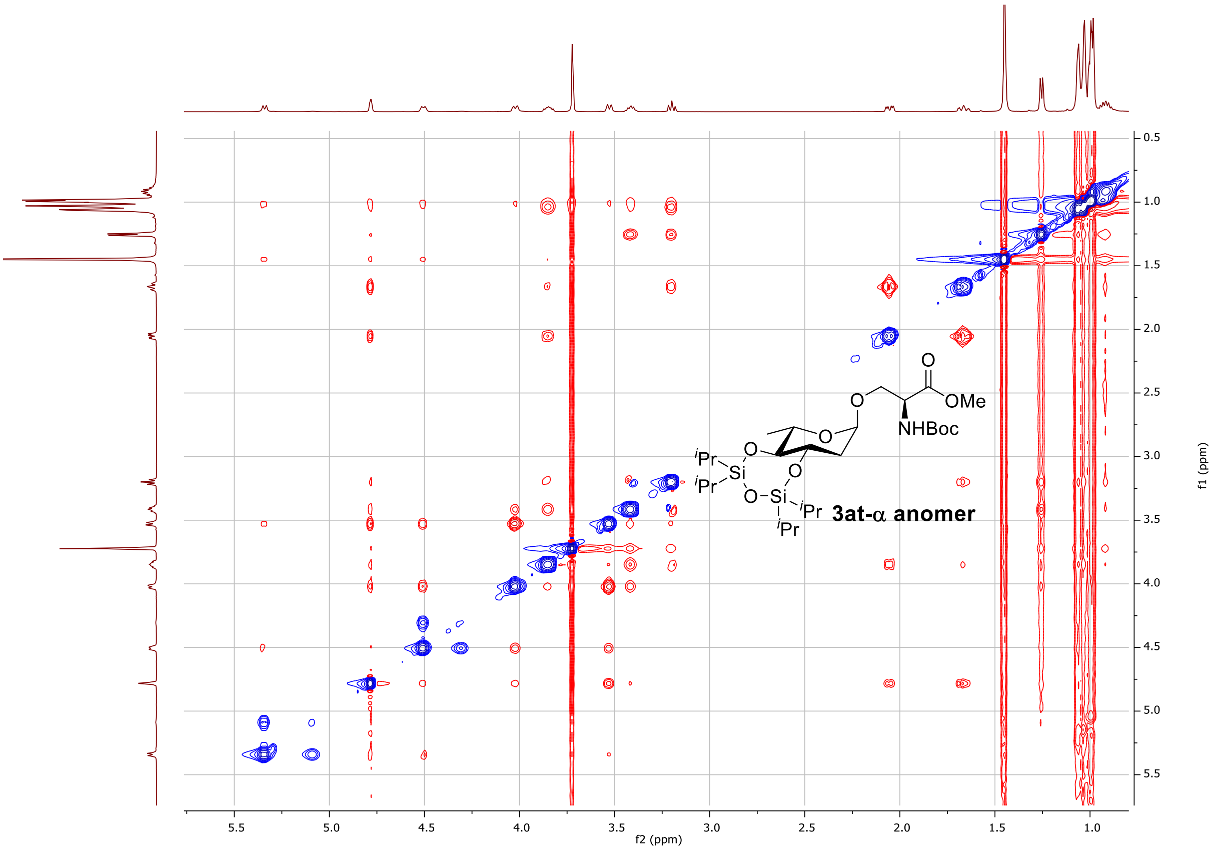

Supplementary figure S395: NOESY spectra for **3at-α anomer**

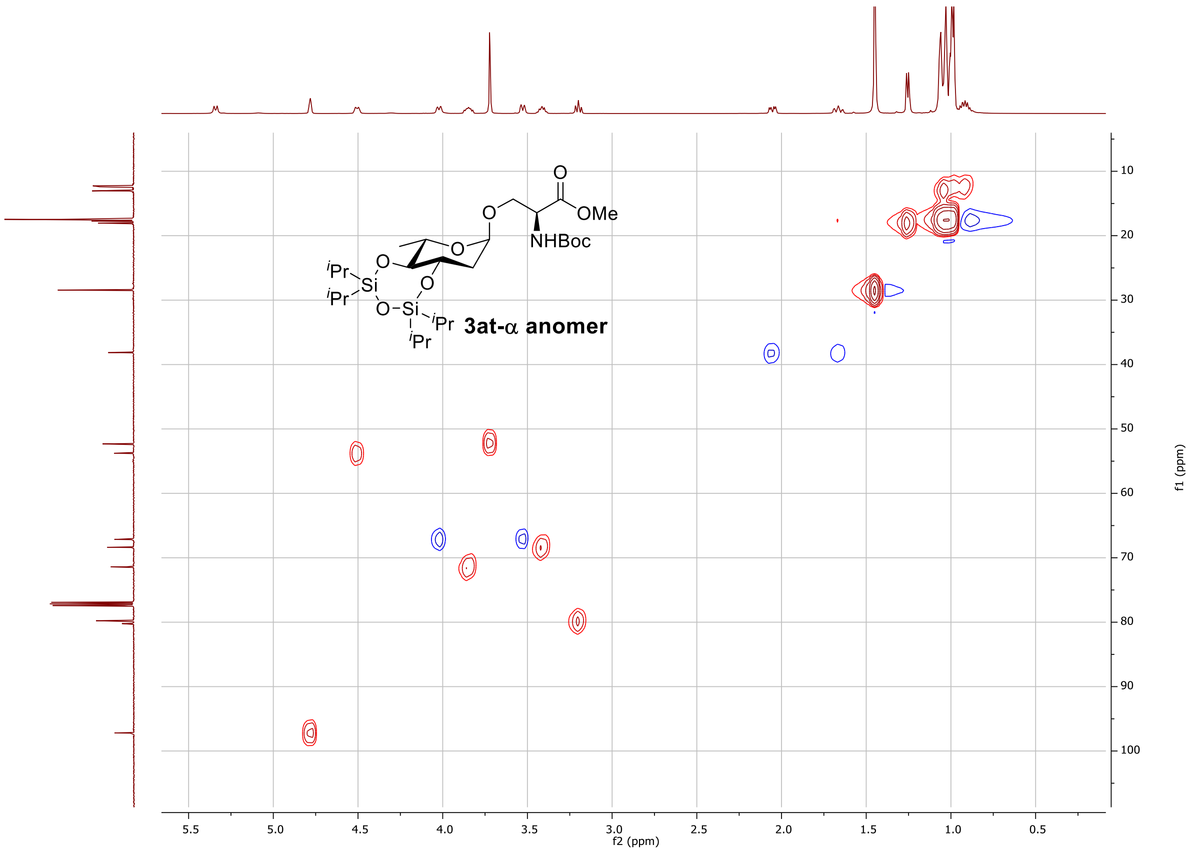

Supplementary figure S396: HSQC spectra for **3at-α anomer**

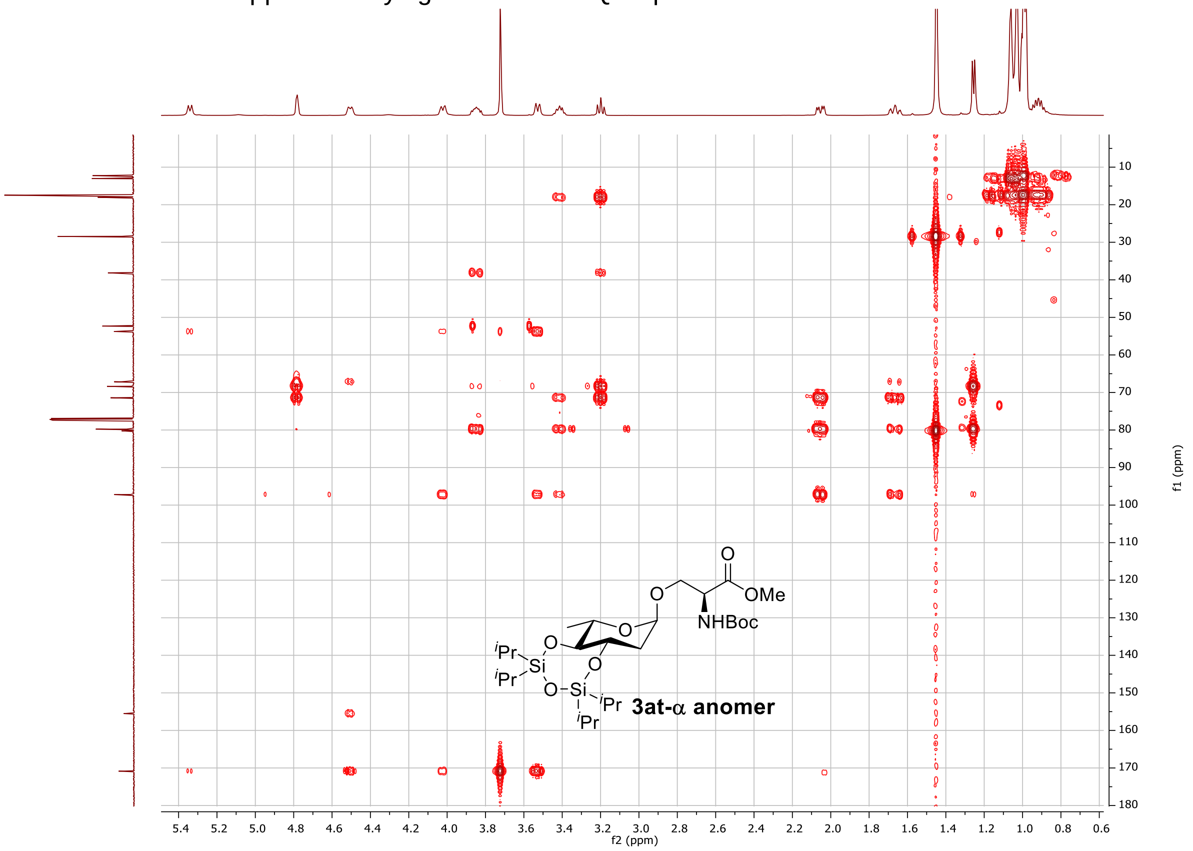

Supplementary figure S397: HMBC spectra for **3at-α anomer**

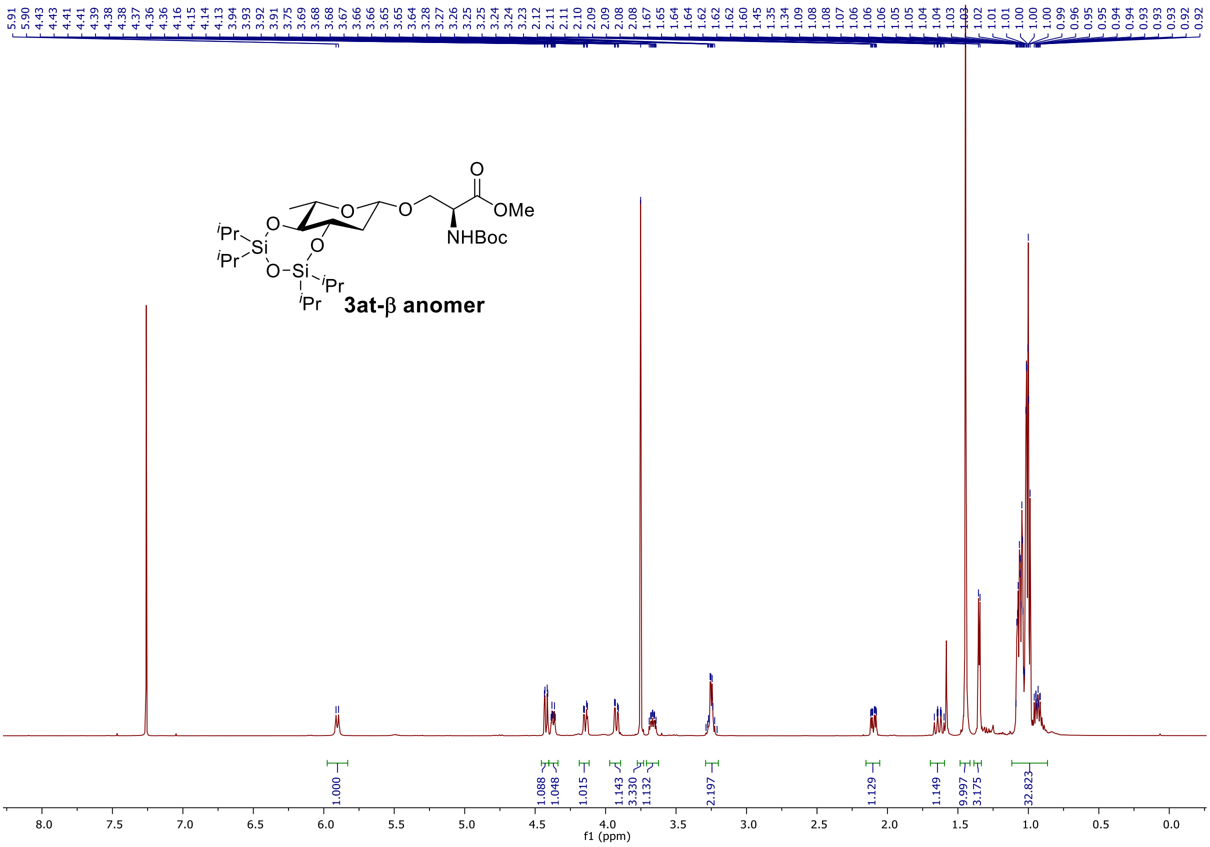

Supplementary figure S398: ^{1}H spectra for **3at-β anomer**

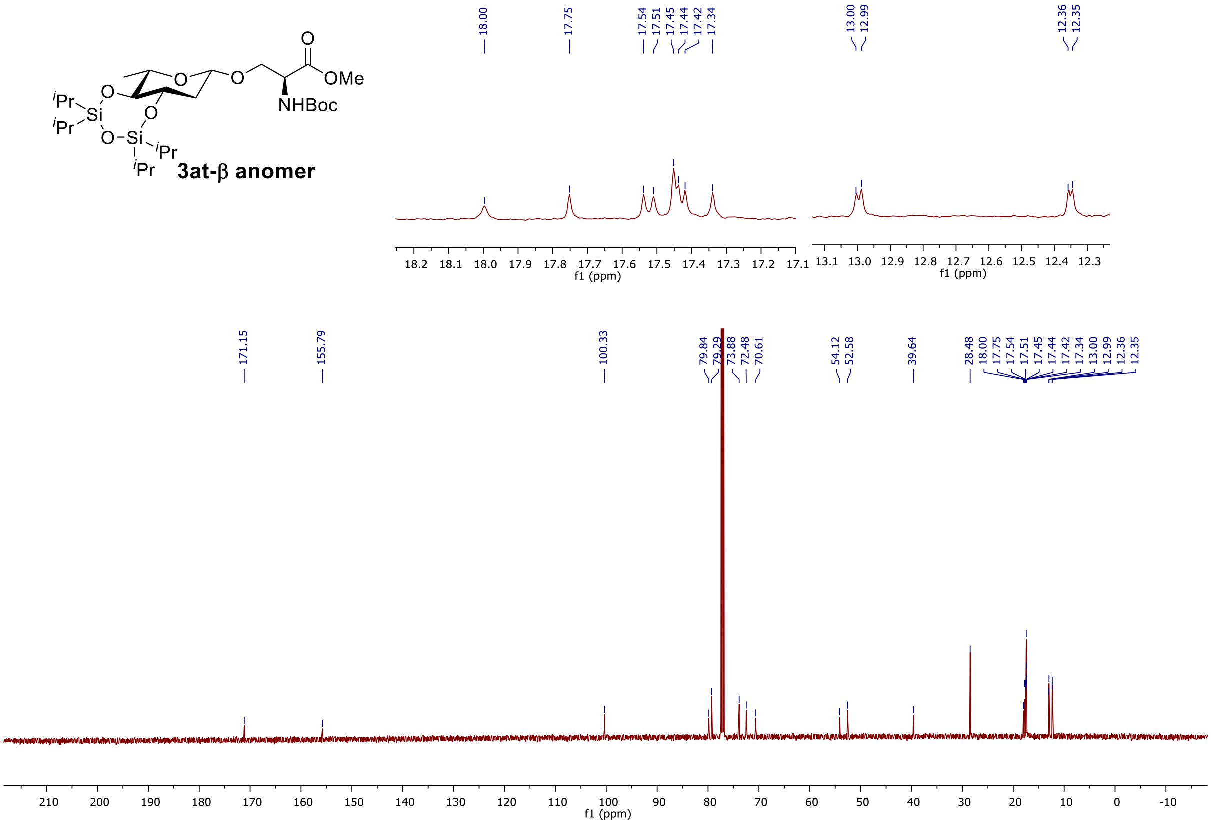

Supplementary figure S399: ^{13}C spectra for **3at-β anomer**

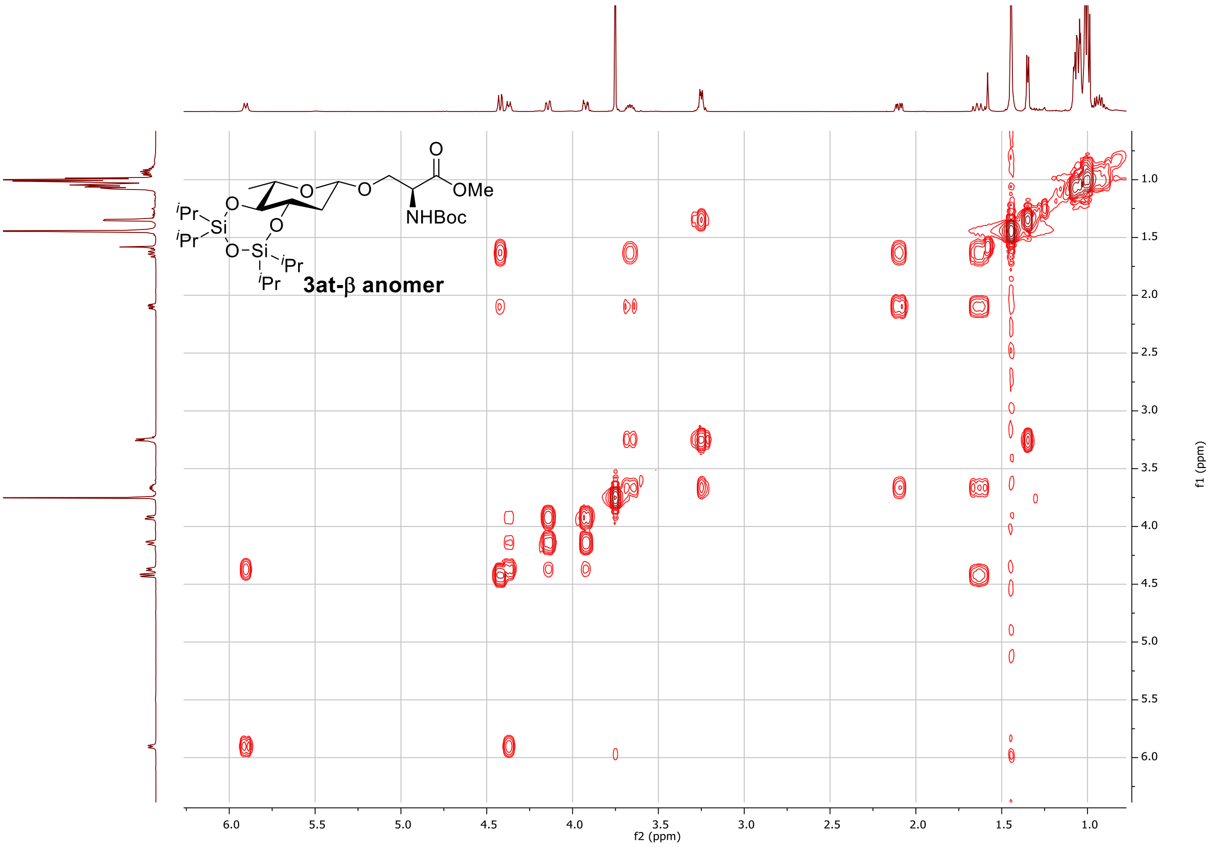

Supplementary figure S400: COSY spectra for **3at-β anomer**

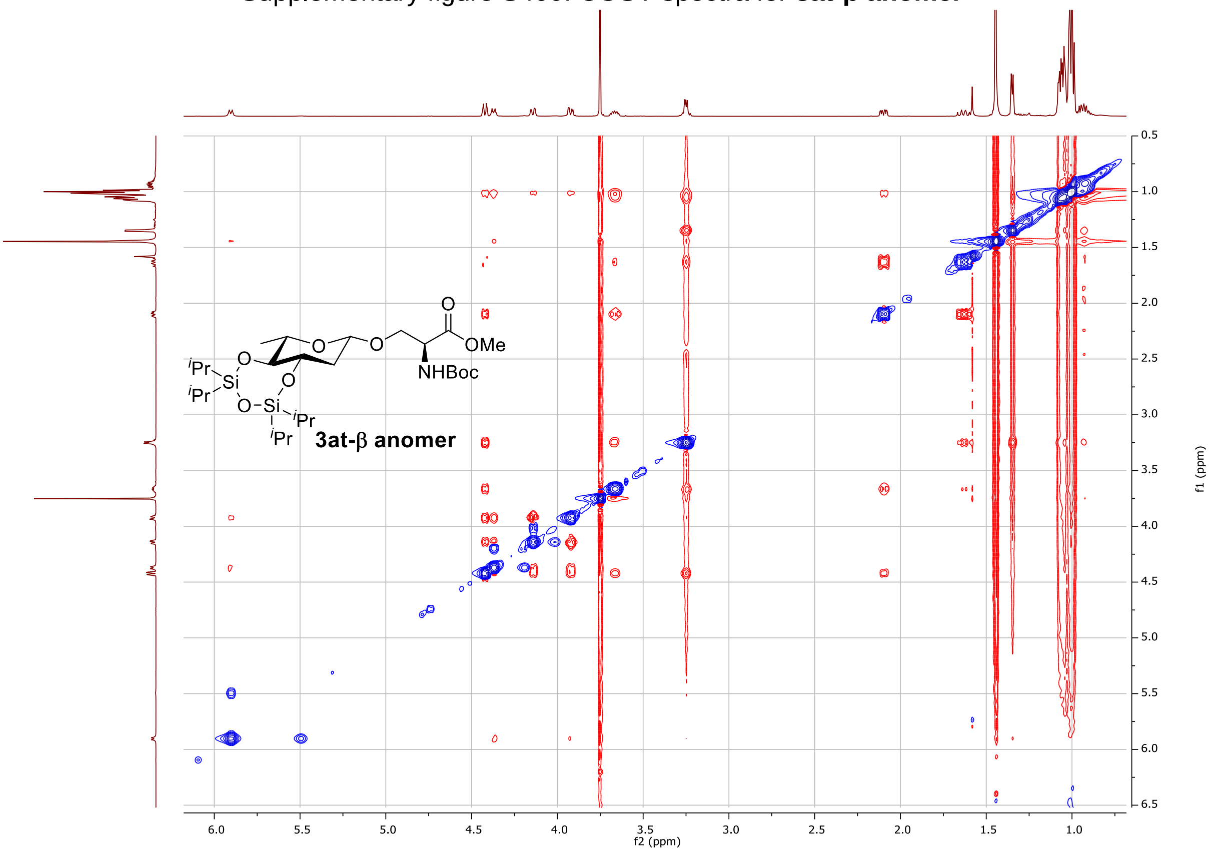

Supplementary figure S401: NOESY spectra for **3at-β anomer**

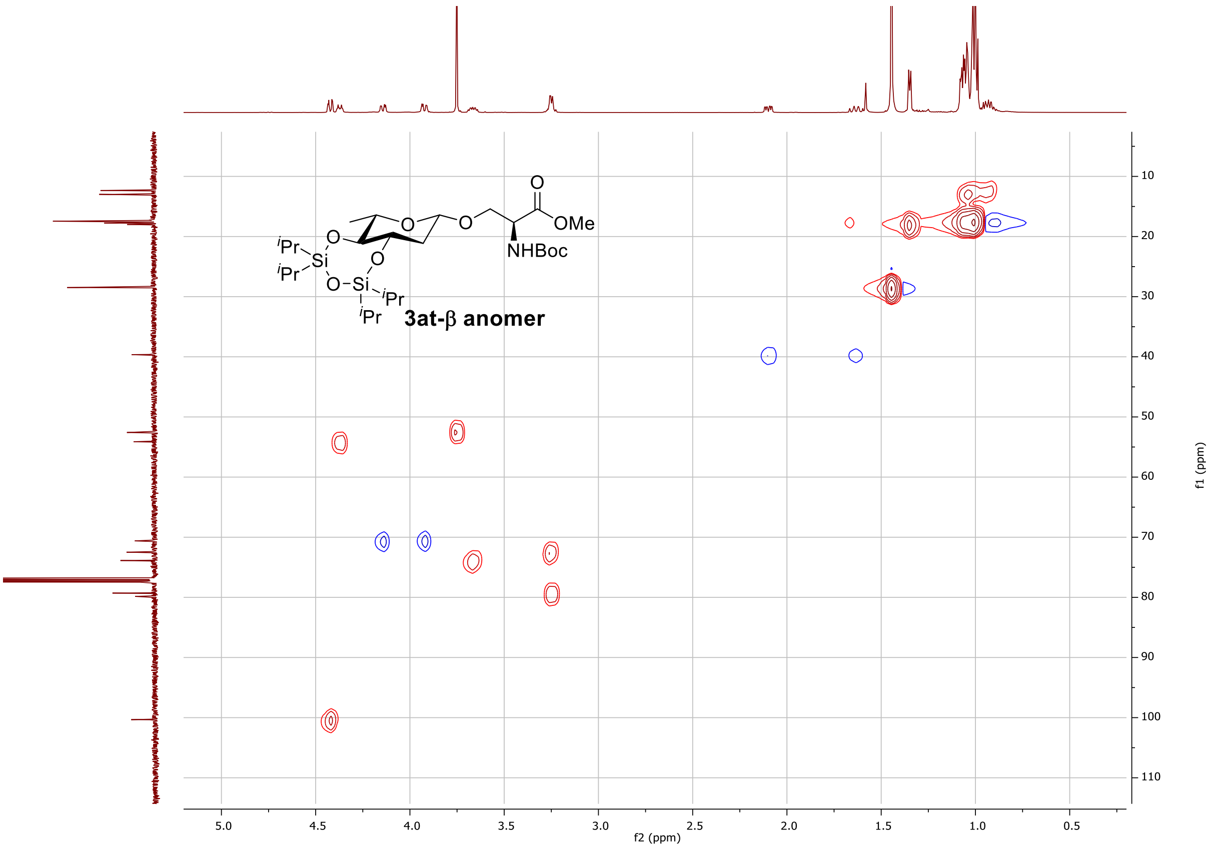

Supplementary figure S402: HSQC spectra for **3at-β anomer**

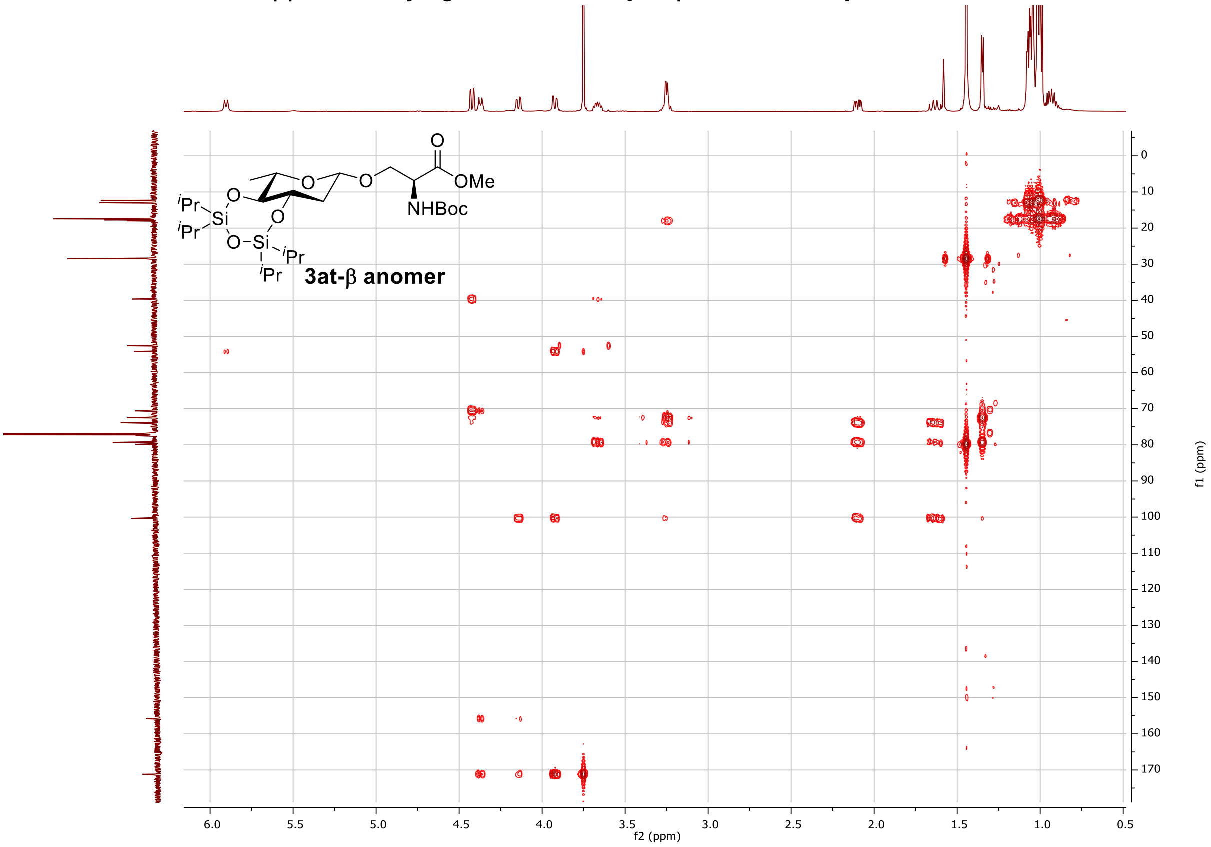

Supplementary figure S043: HMBC spectra for **3at-β anomer**

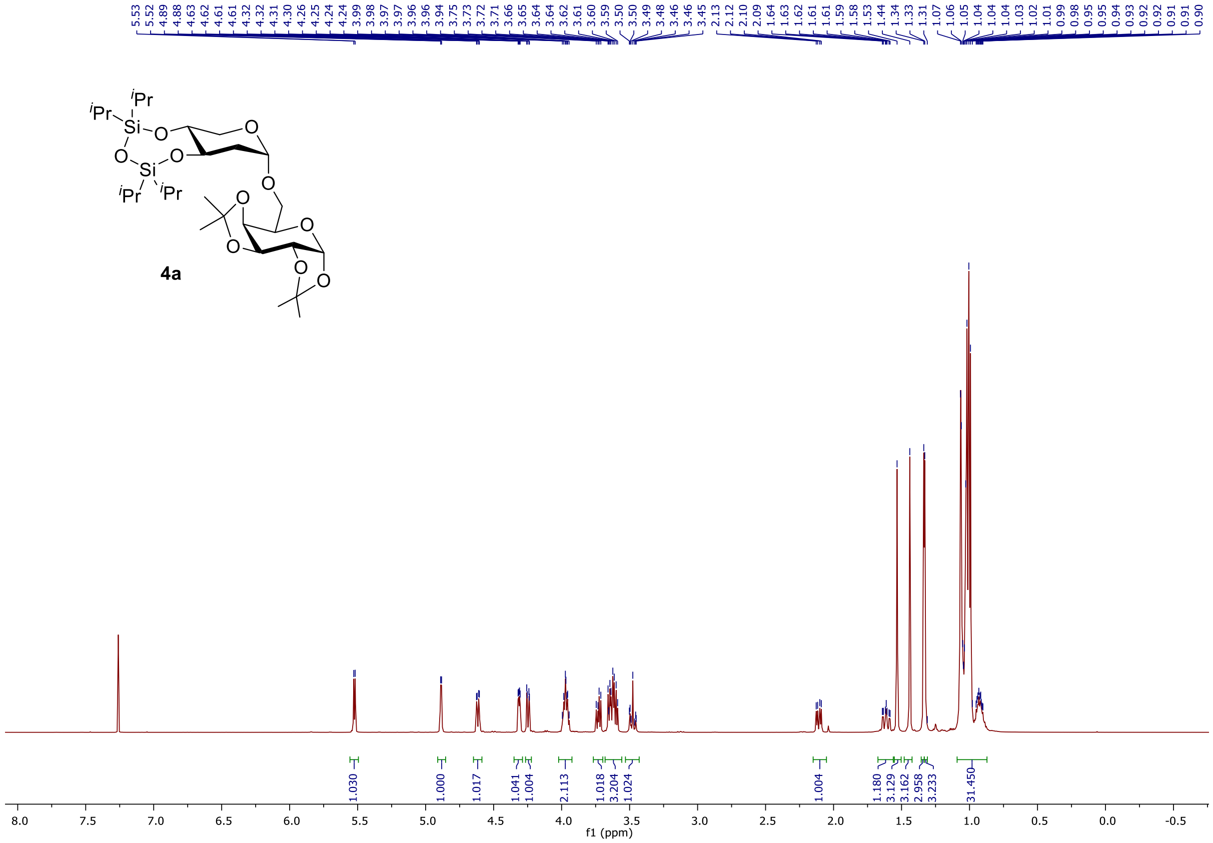

Supplementary figure S404: ^{1}H spectra for **4a**

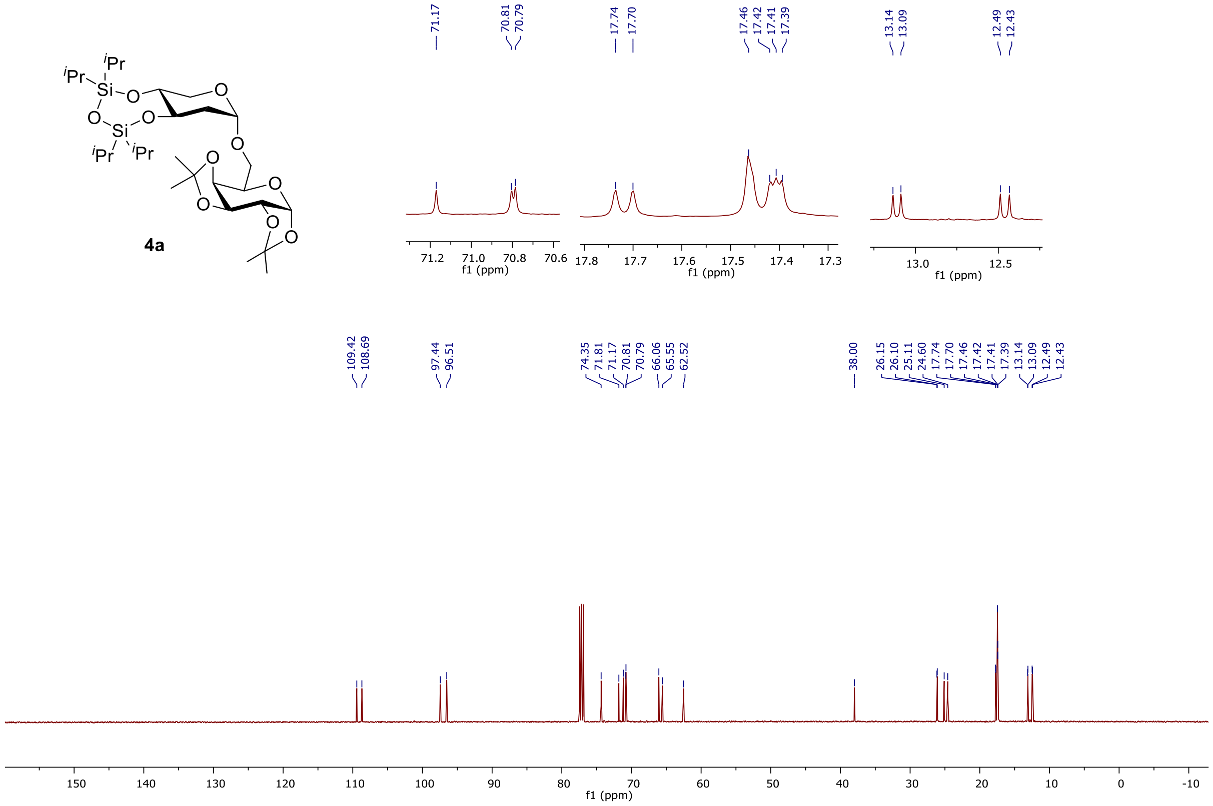

Supplementary figure S405: ^{13}C spectra for **4a**

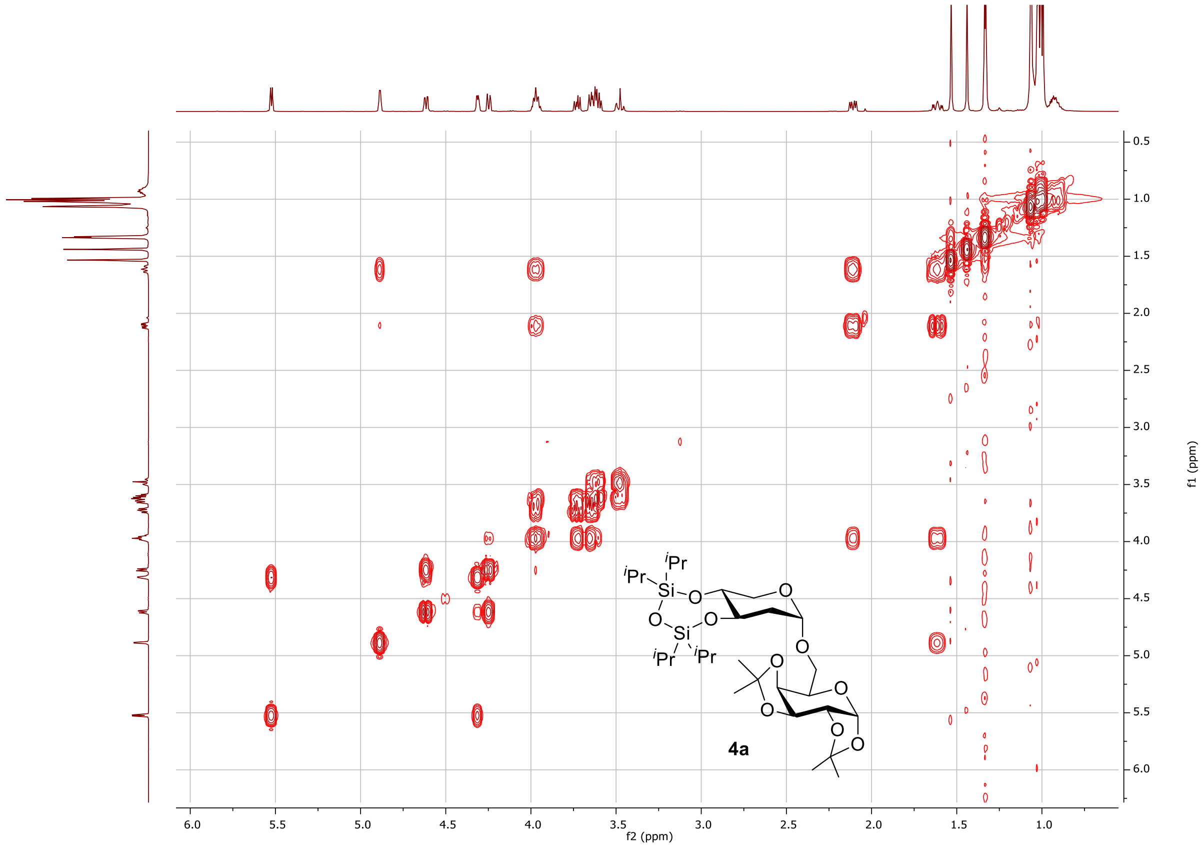

Supplementary figure S406: COSY spectra for **4a**

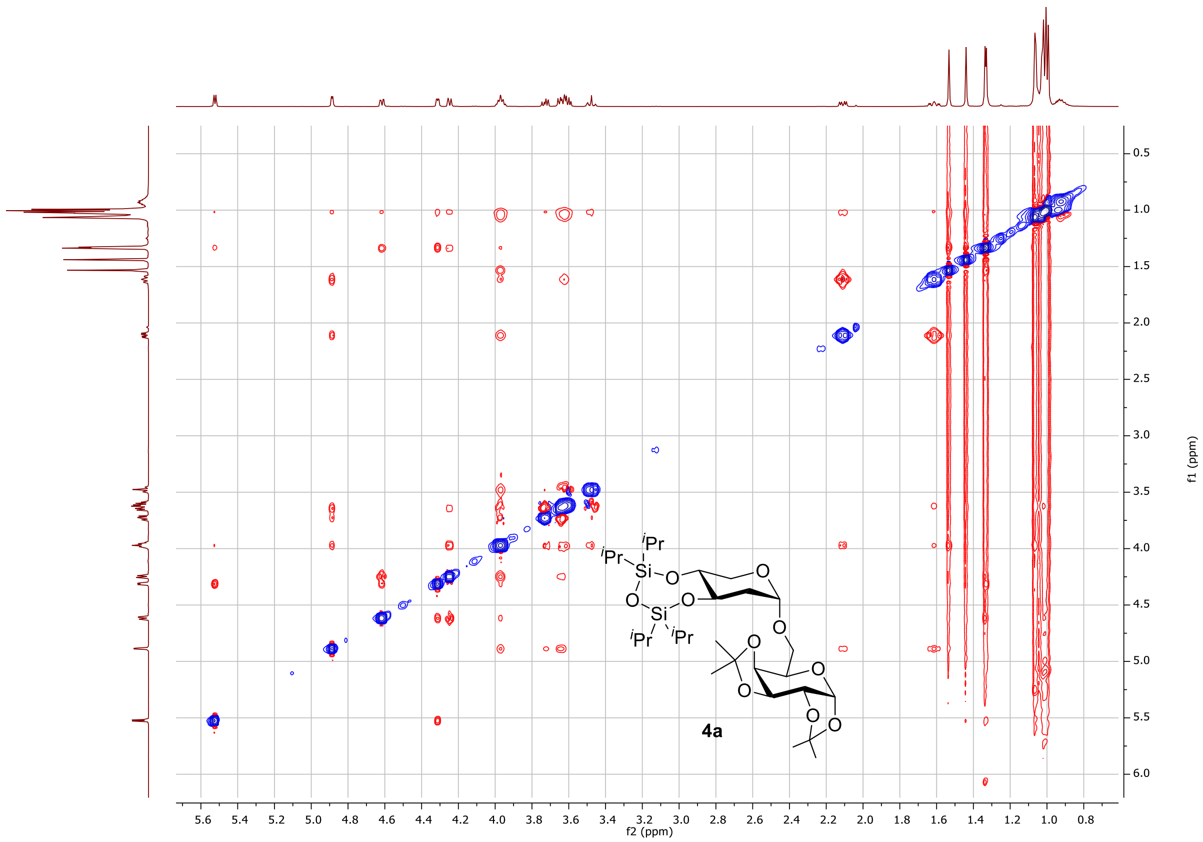

Supplementary figure S407: NOESY spectra for **4a**

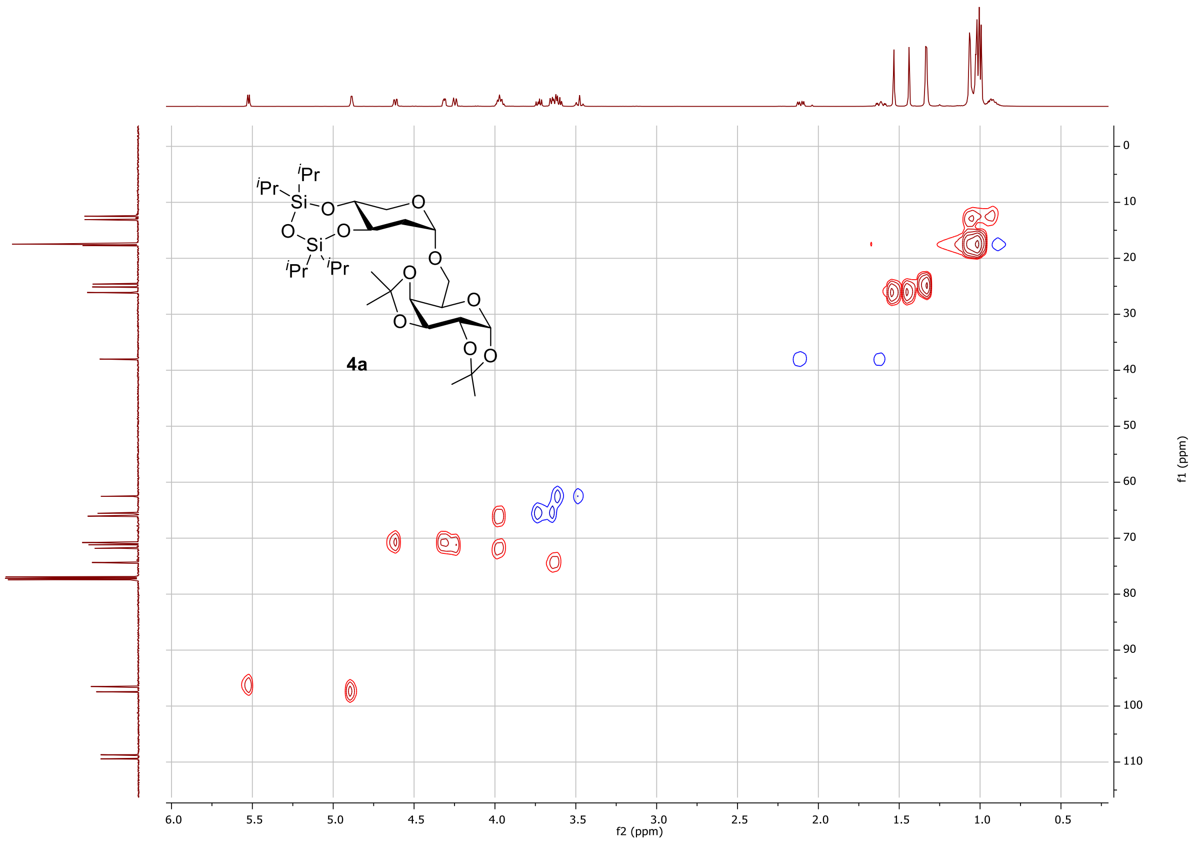

Supplementary figure S408: HSQC spectra for **4a**

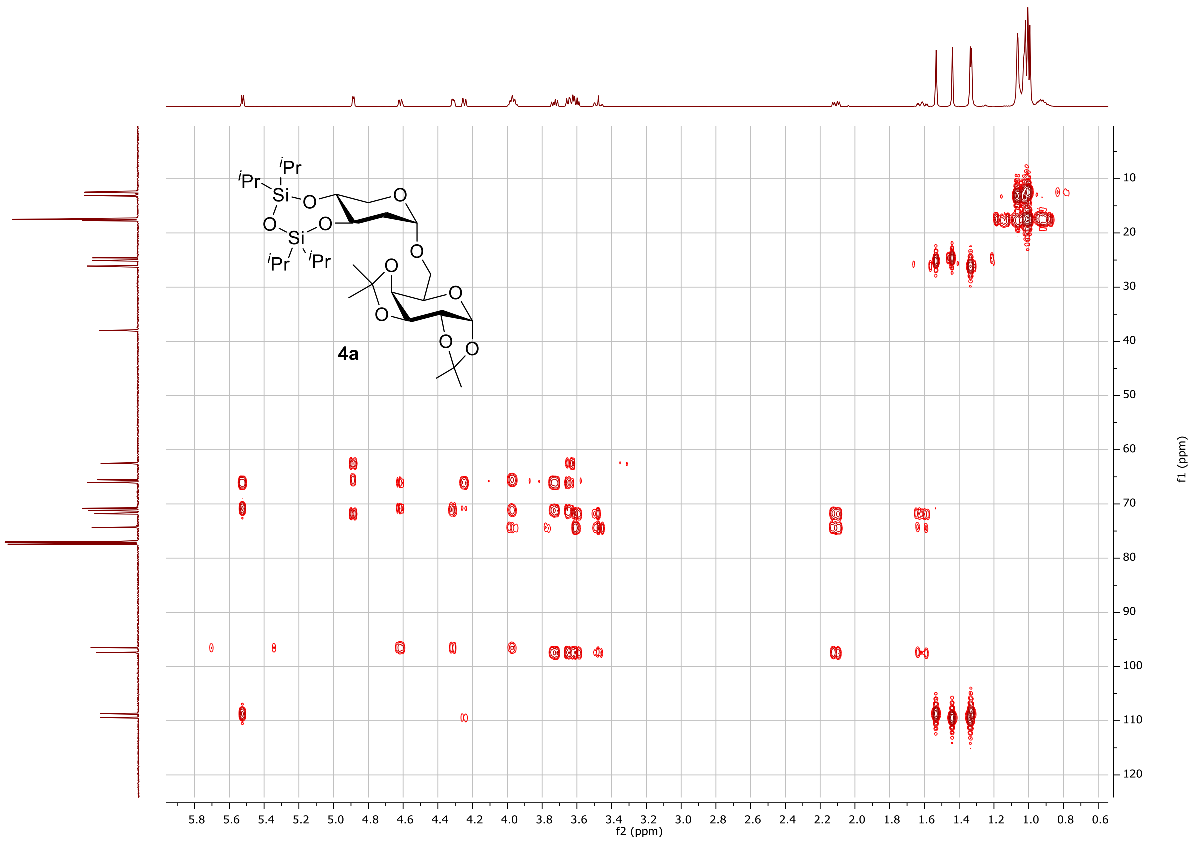

Supplementary figure S409: HMBC spectra for **4a**

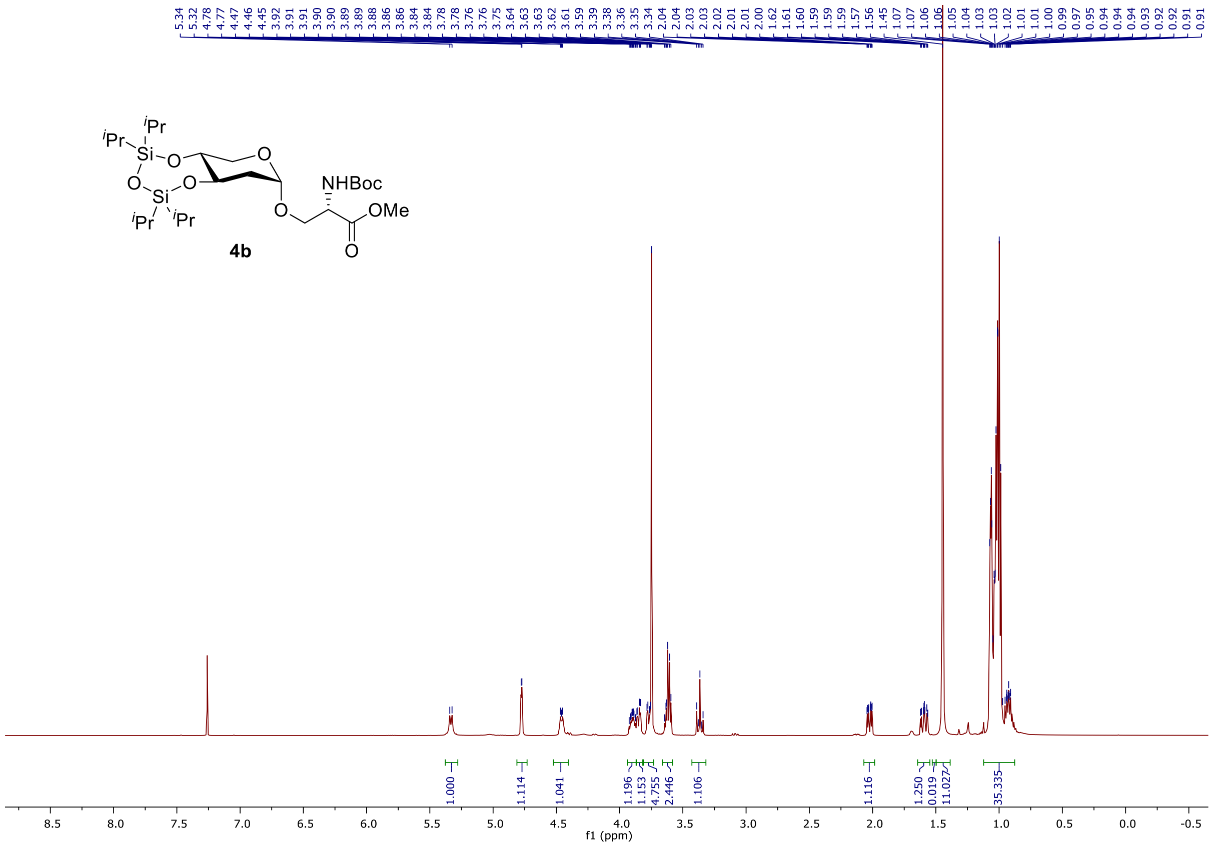

Supplementary figure S410: ^{1}H spectra for **4b**

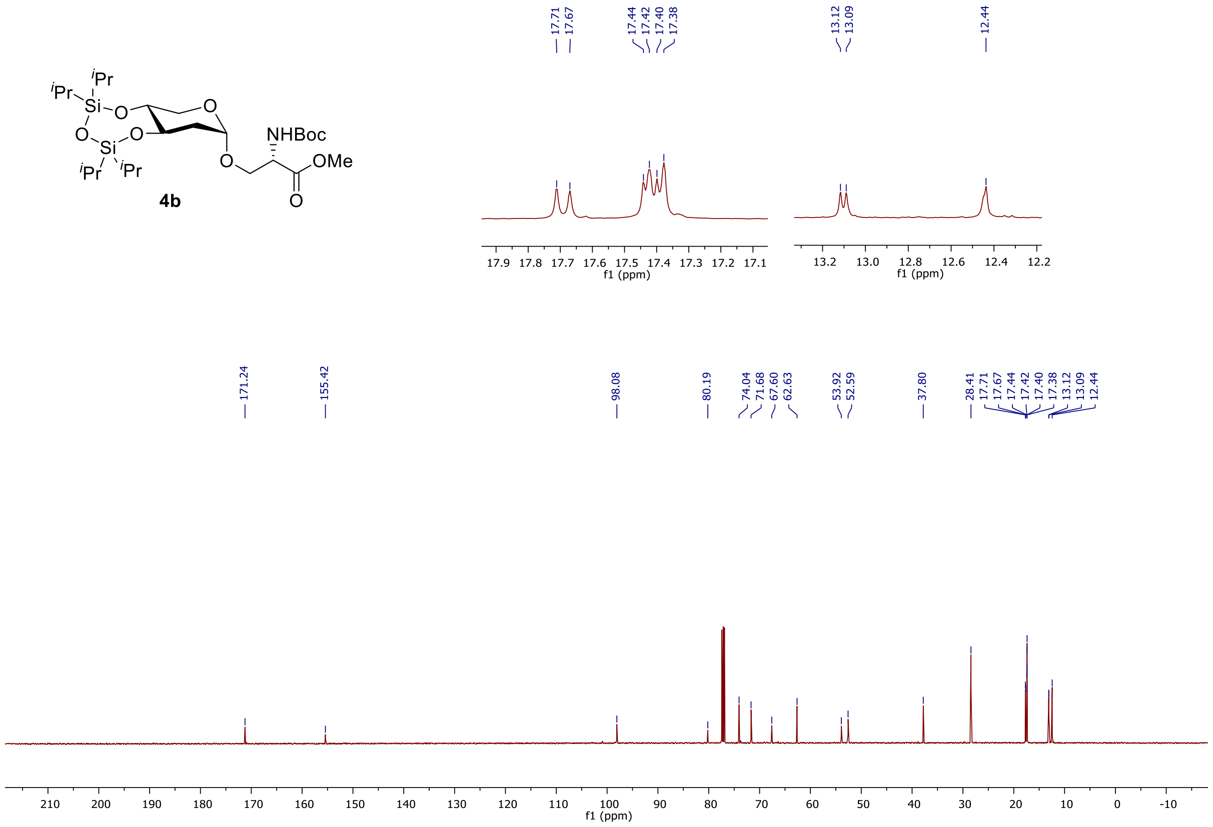

Supplementary figure S411: ^{13}C spectra for **4b**

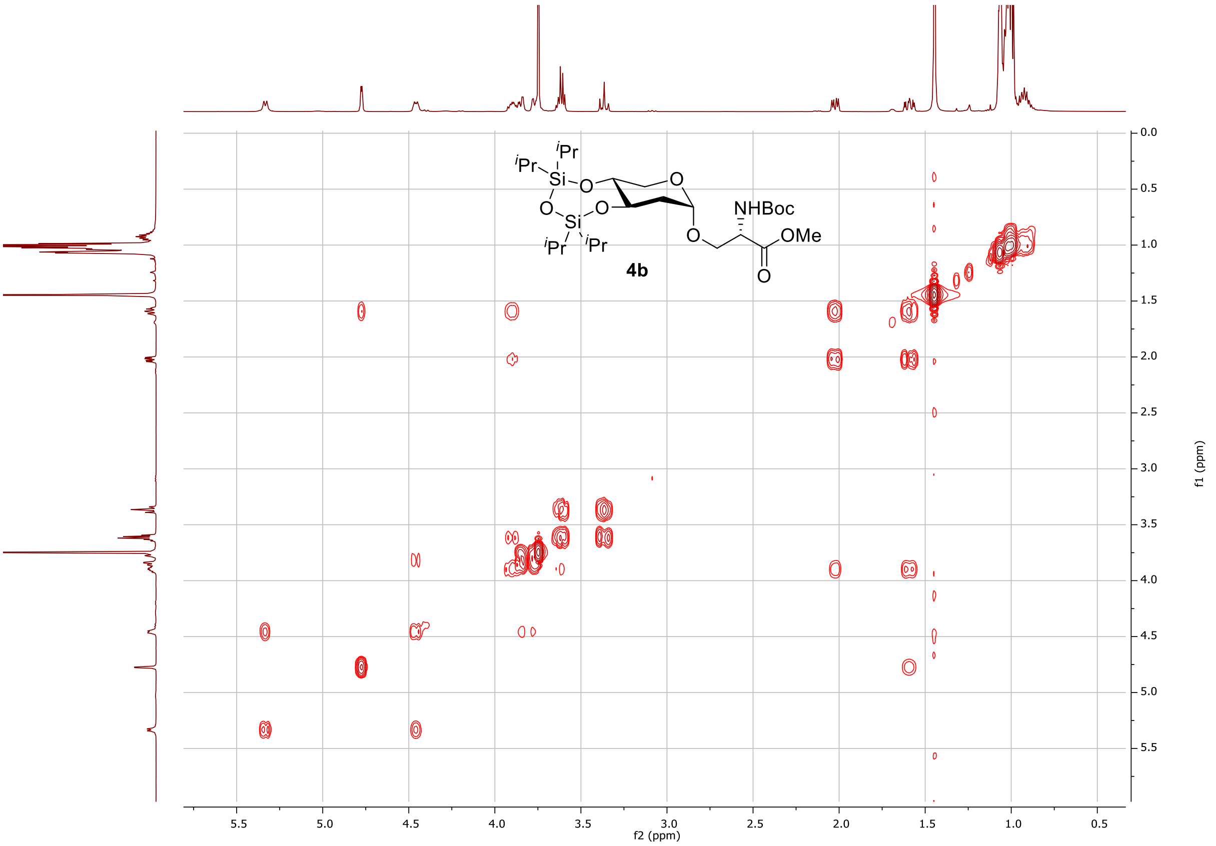

Supplementary figure S412: COSY spectra for **4b**

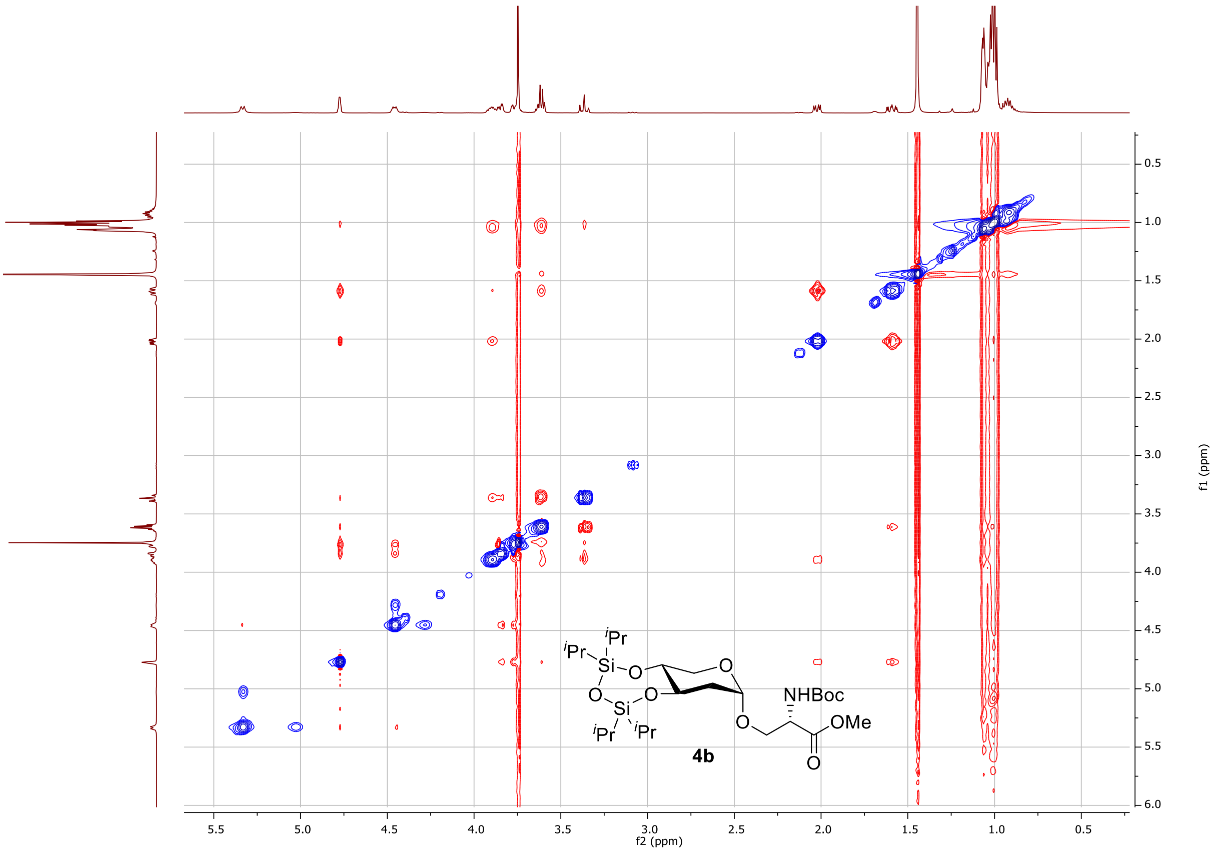

Supplementary figure S413: NOESY spectra for **4b**

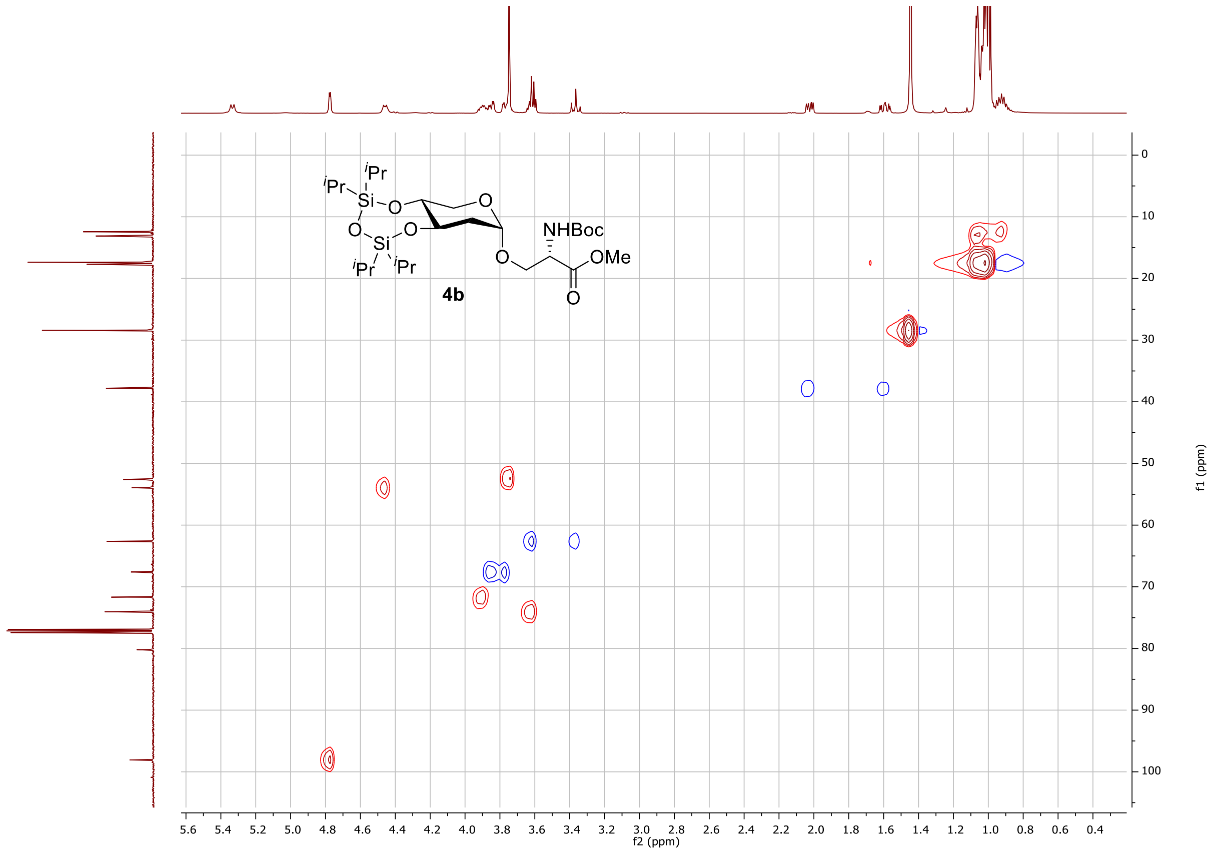

Supplementary figure S414: HSQC spectra for **4b**

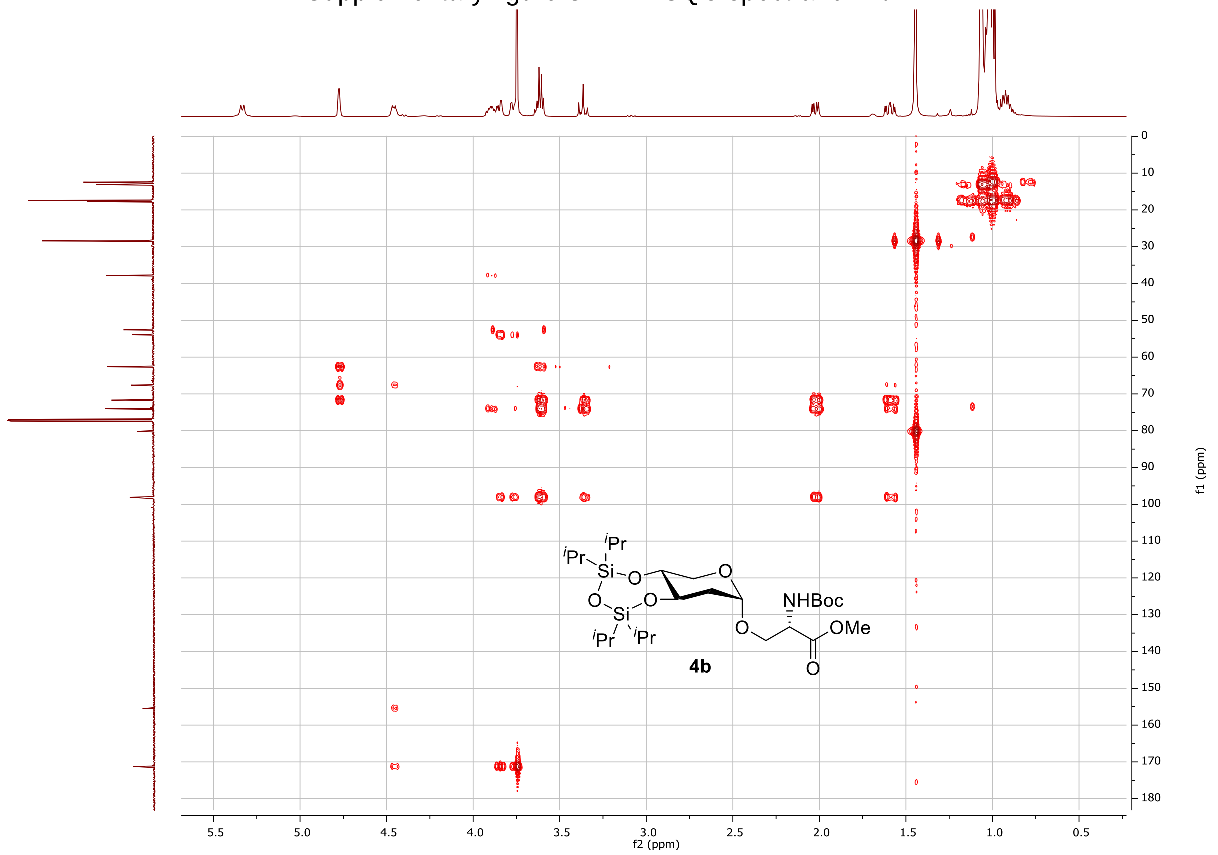

Supplementary figure S415: HMBC spectra for **4b**

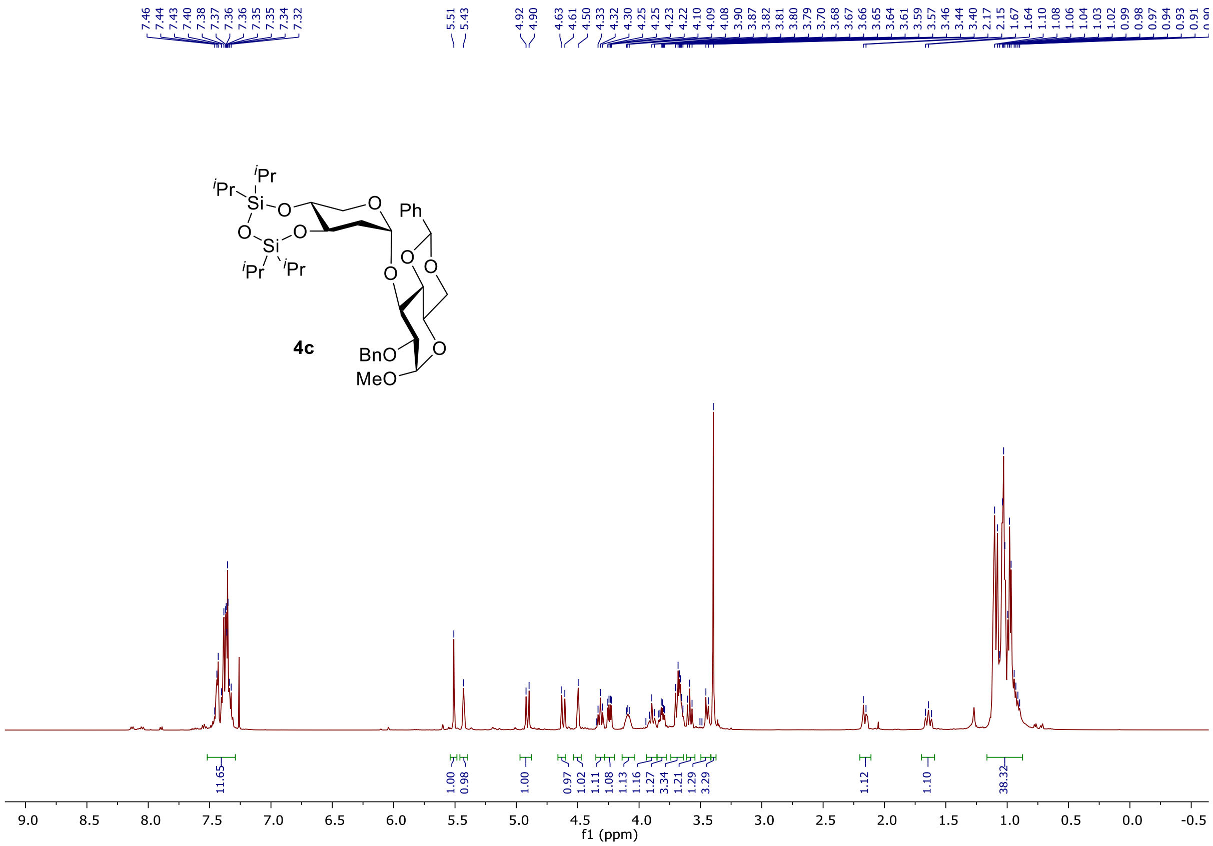

Supplementary figure S416: ^{1}H spectra for **4c**

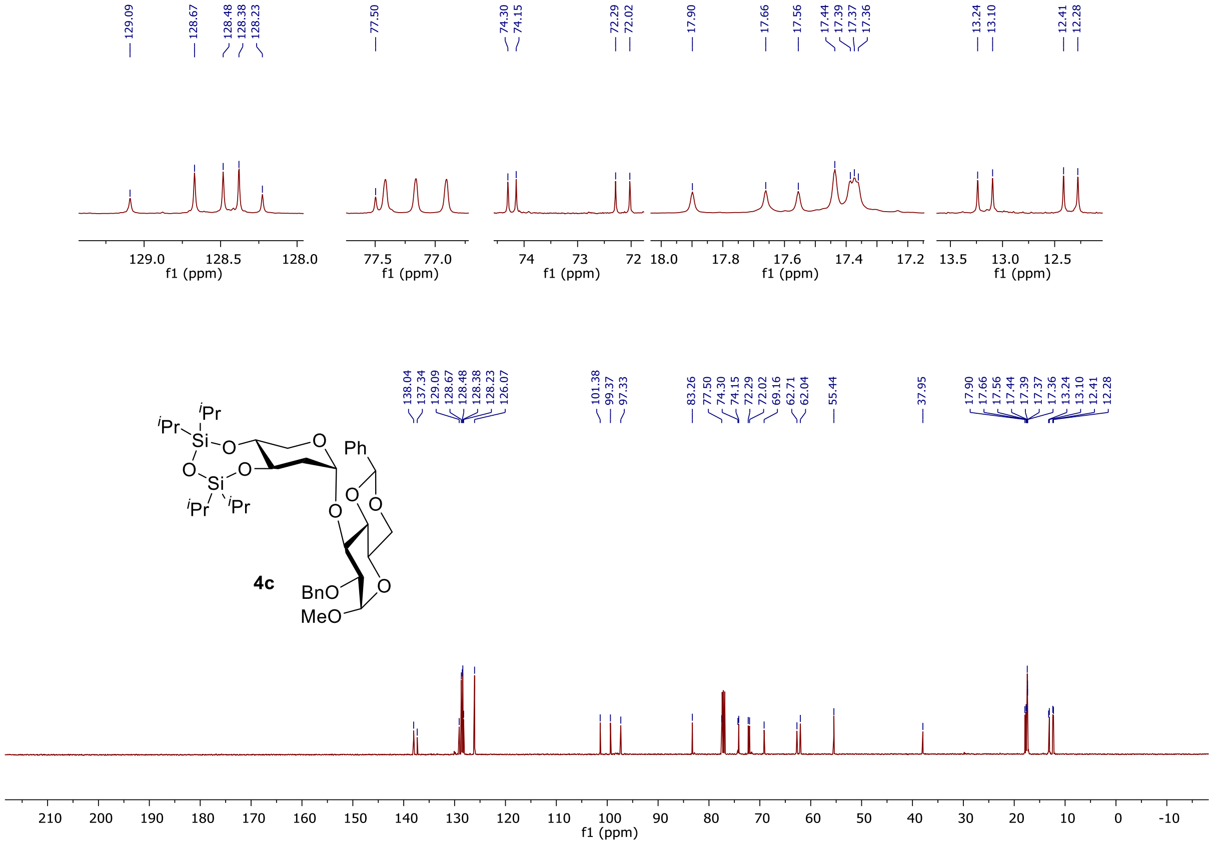

Supplementary figure S417: ^{13}C spectra for **4c**

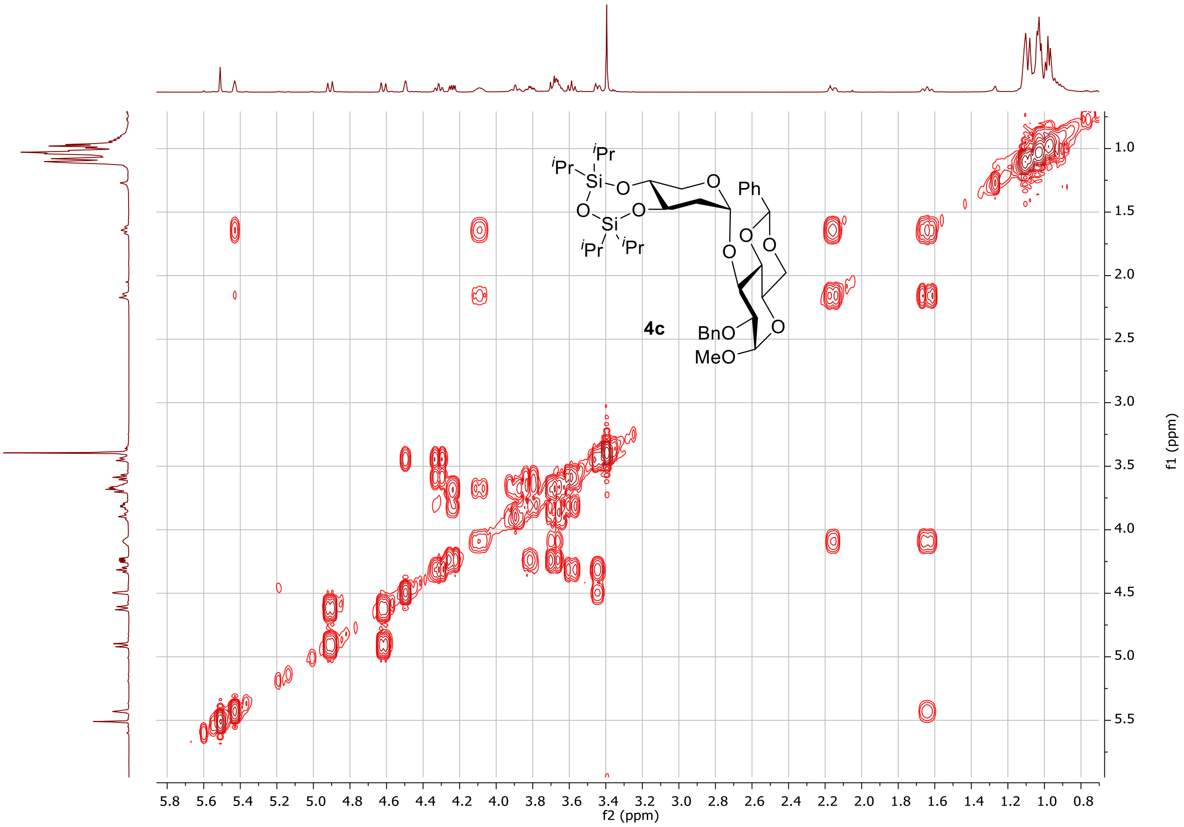

Supplementary figure S418: COSY spectra for **4c**

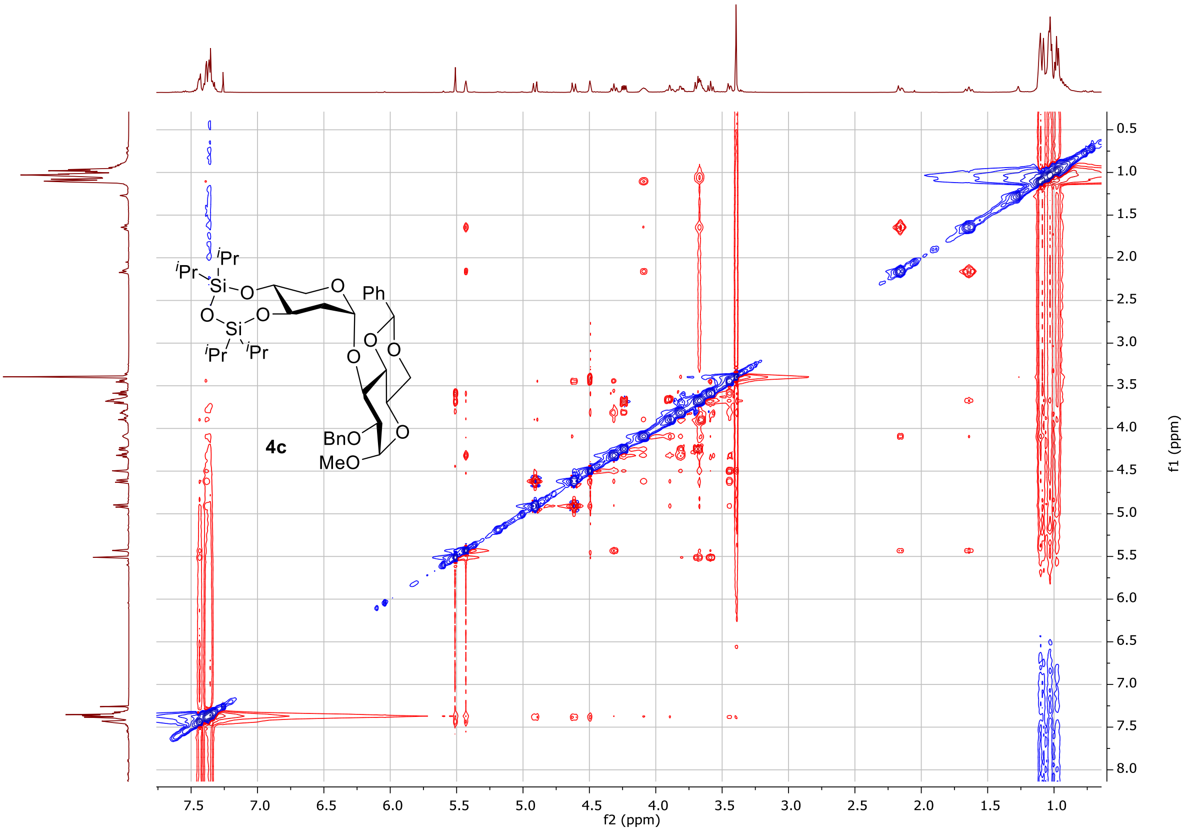

Supplementary figure S419: NOESY spectra for **4c**

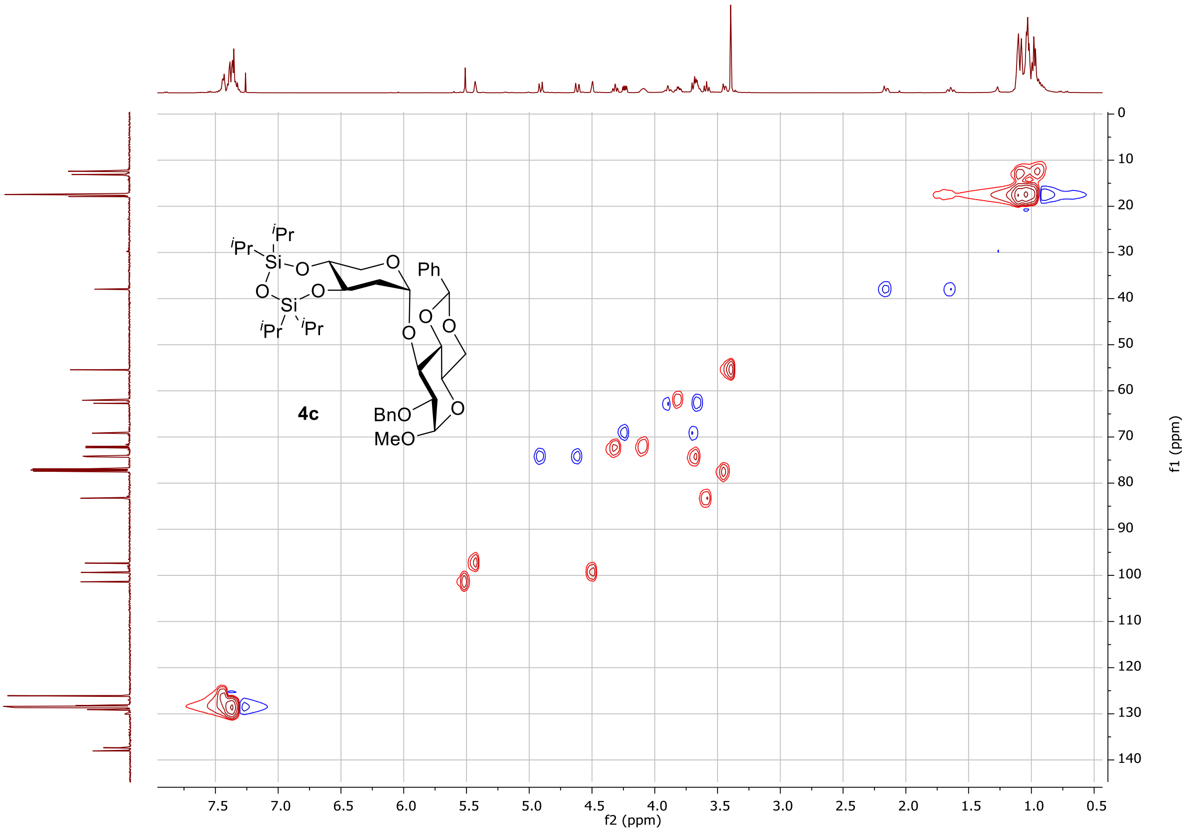

Supplementary figure S420: HSQC spectra for **4c**

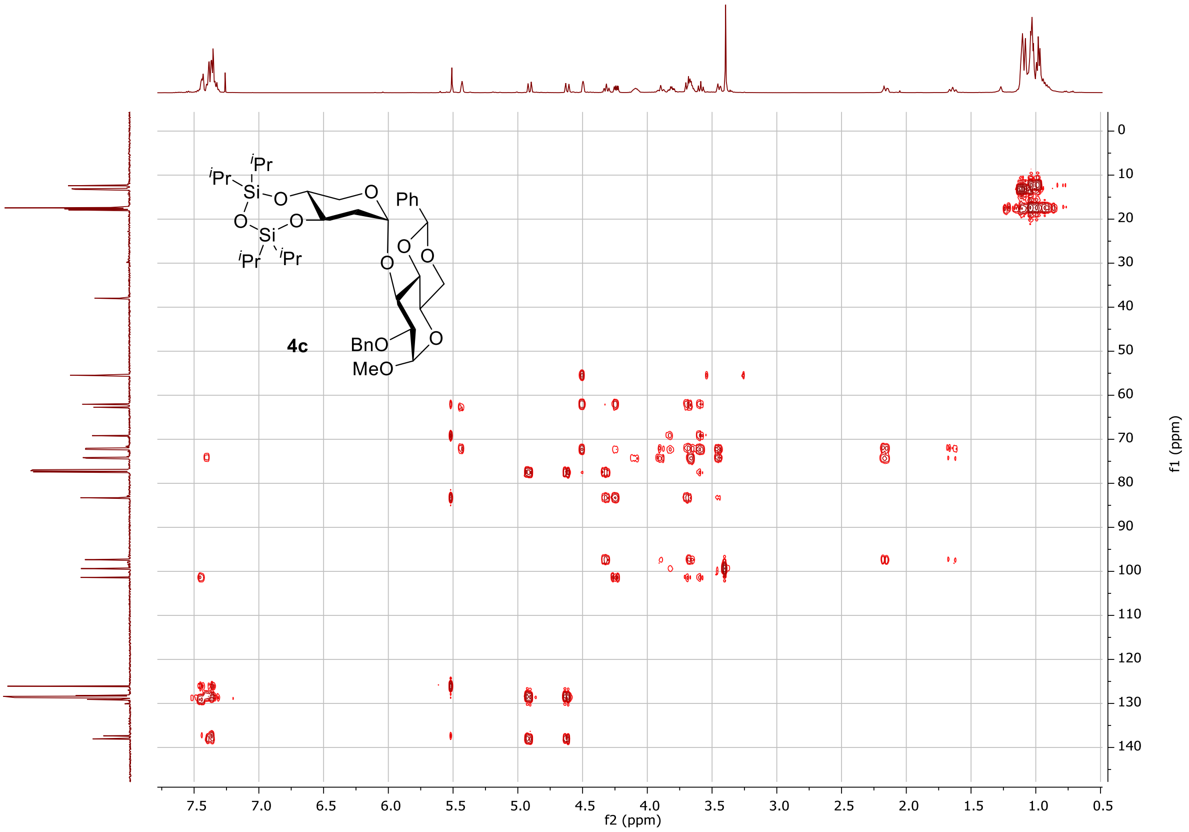

Supplementary figure S421: HMBC spectra for **4c**

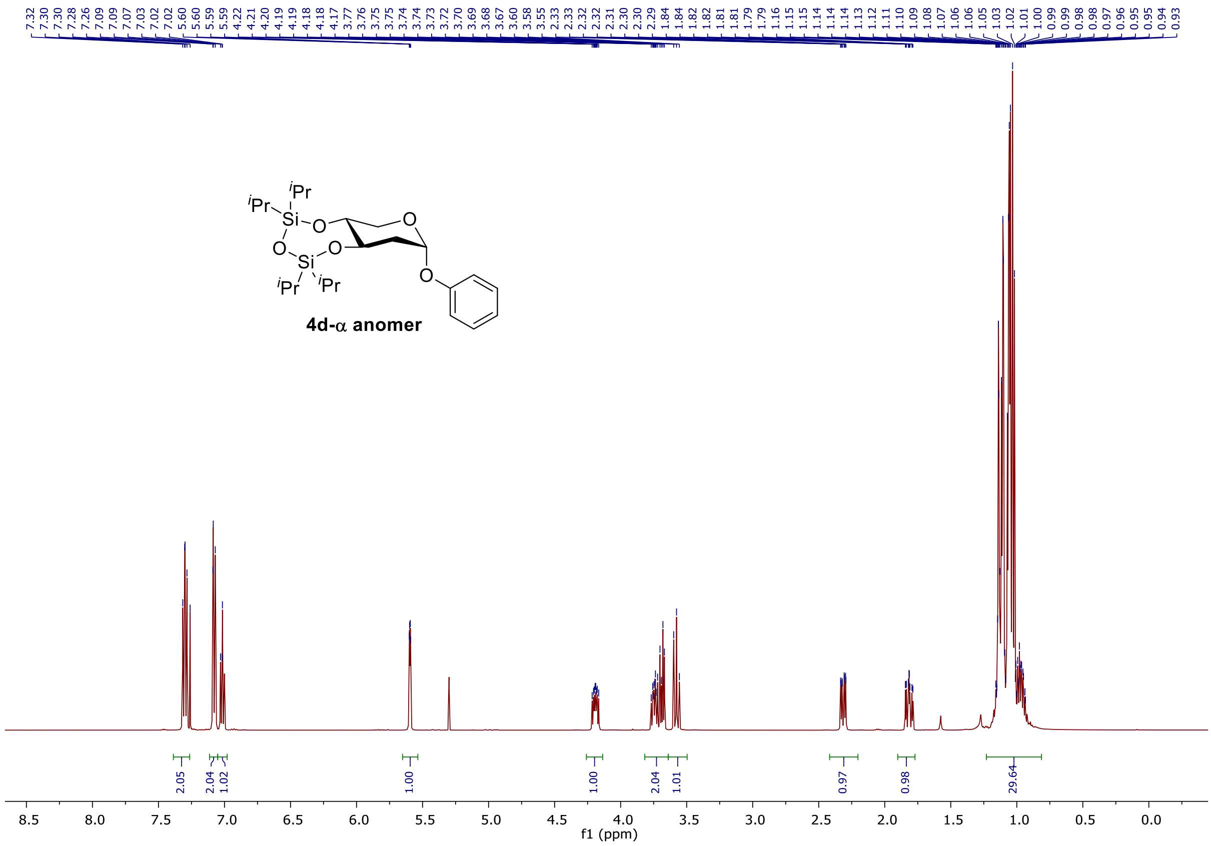

Supplementary figure S422: ^{1}H spectra for **4d-α anomer**

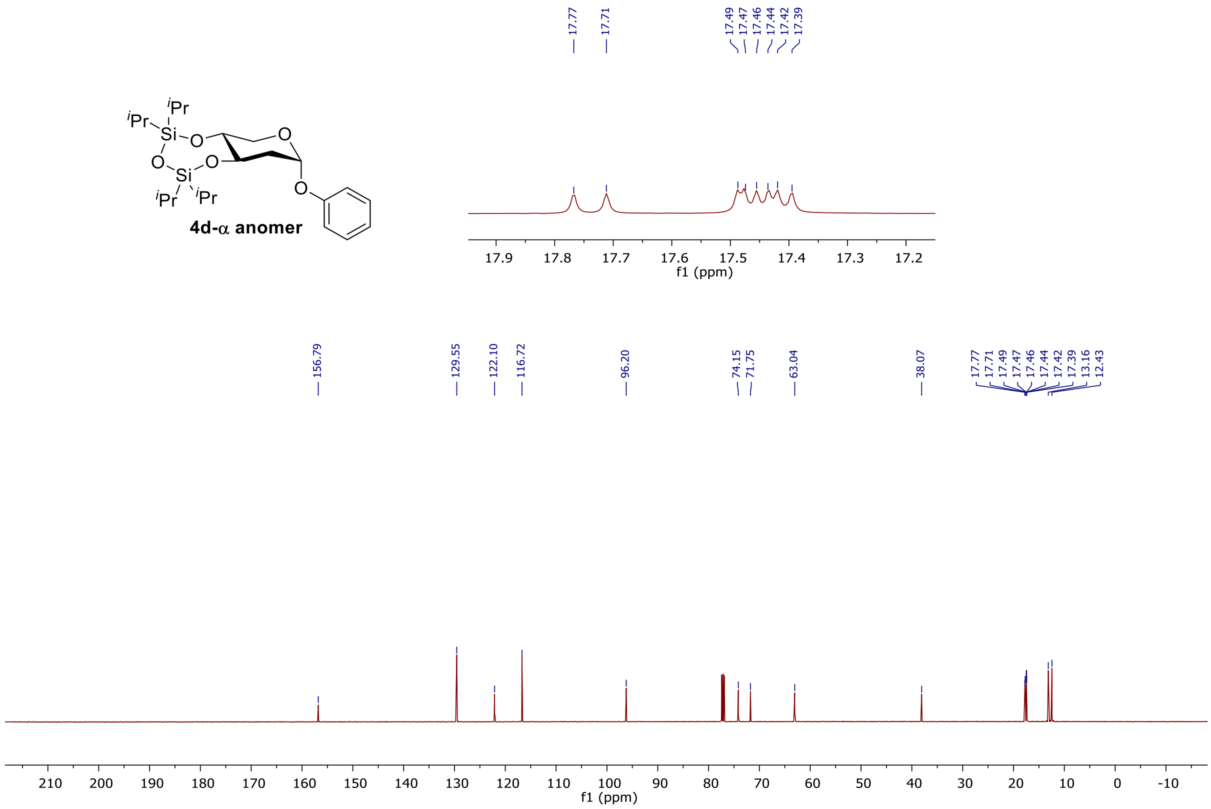

Supplementary figure S423: ^{13}C spectra for **4d-α anomer**

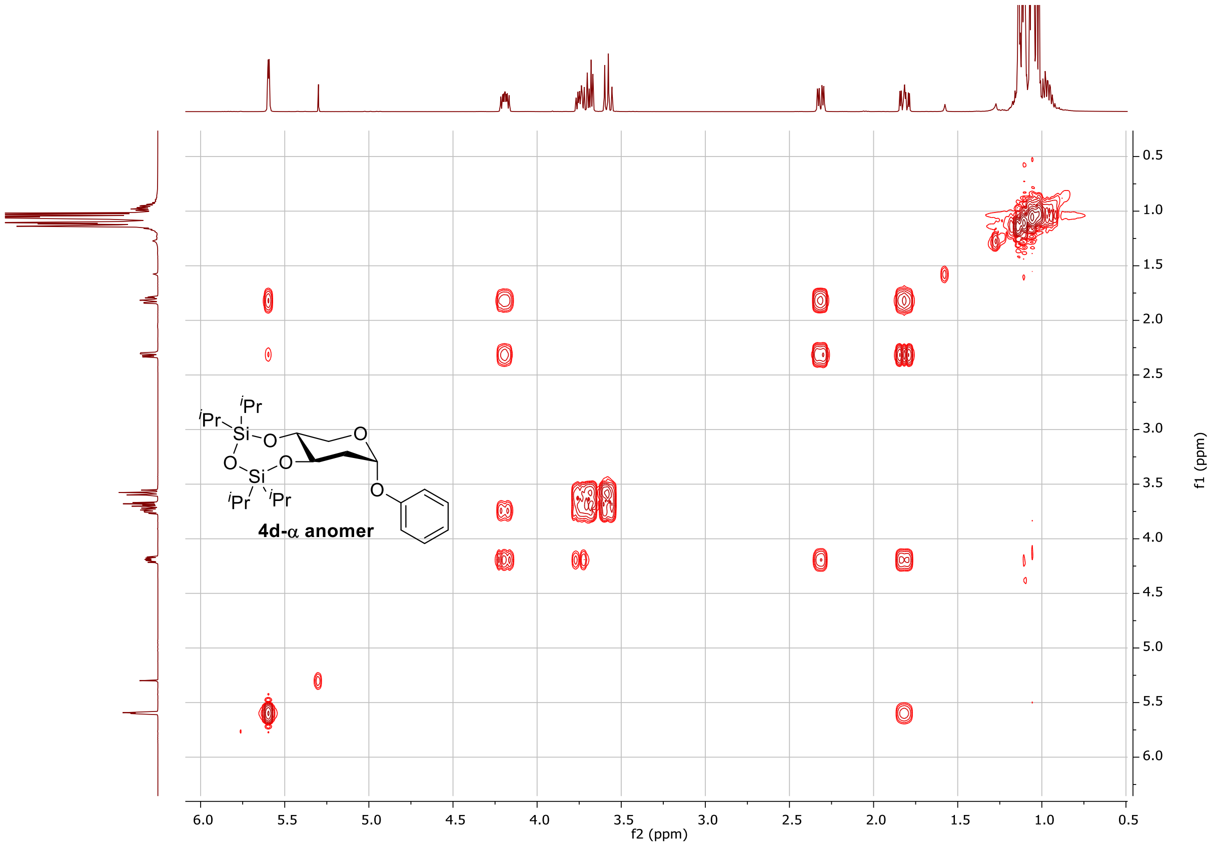

Supplementary figure S424: COSY spectra for **4d-α anomer**

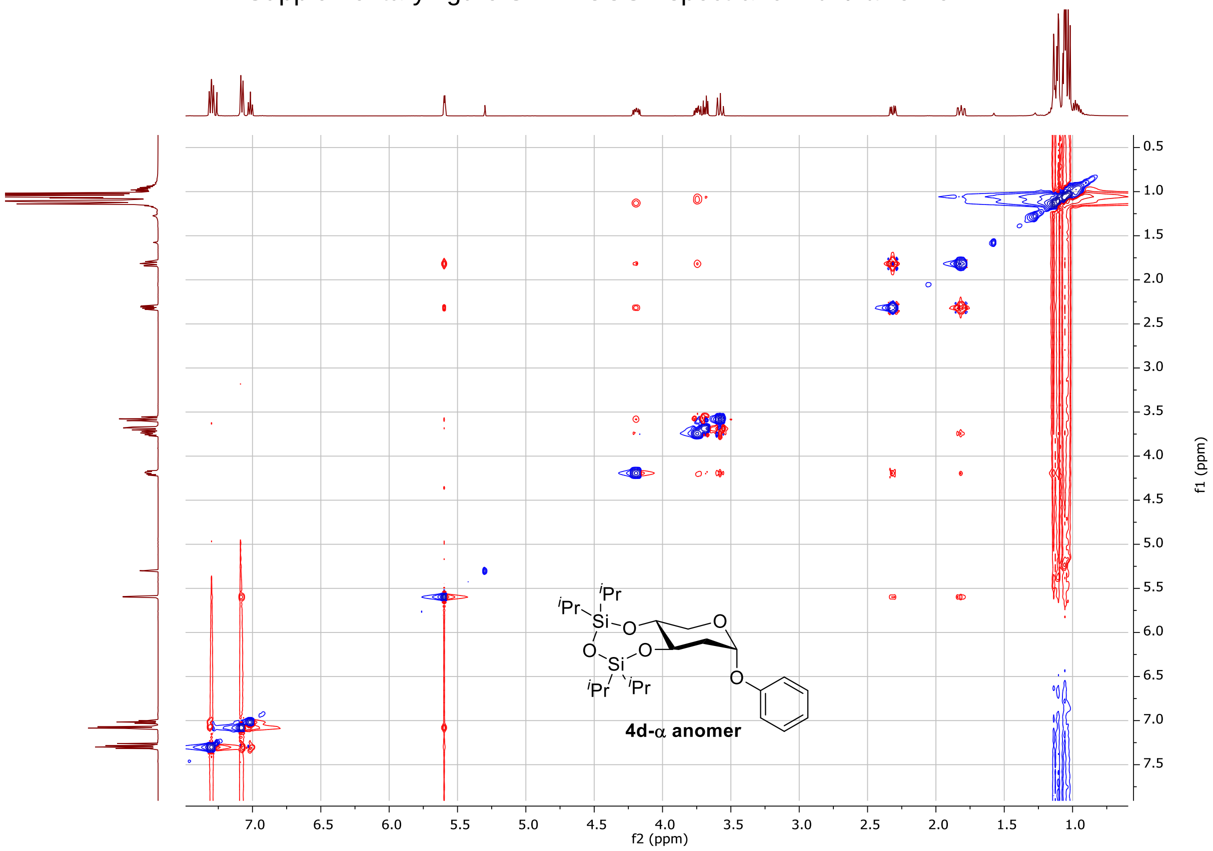

Supplementary figure S425: NOESY spectra for **4d-α anomer**

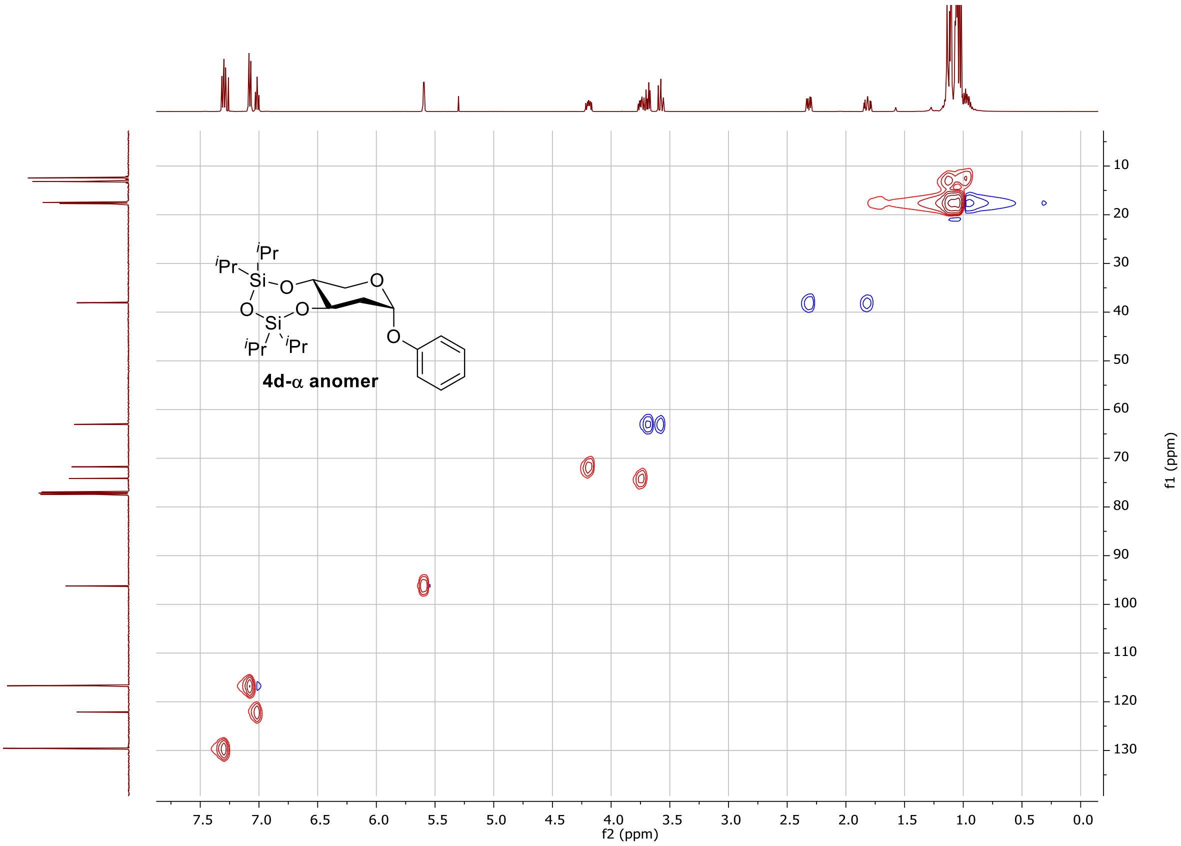

Supplementary figure S426: HSQC spectra for **4d-α anomer**

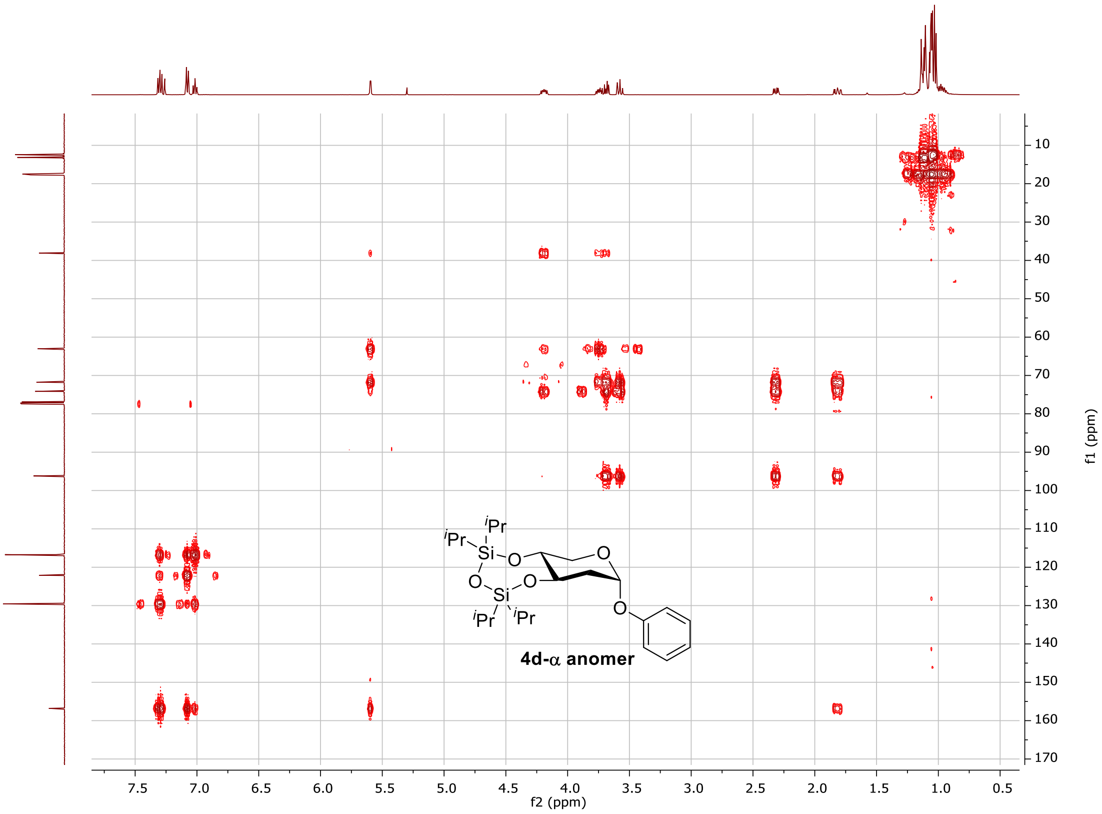

Supplementary figure S427: HMBC spectra for **4d-α anomer**

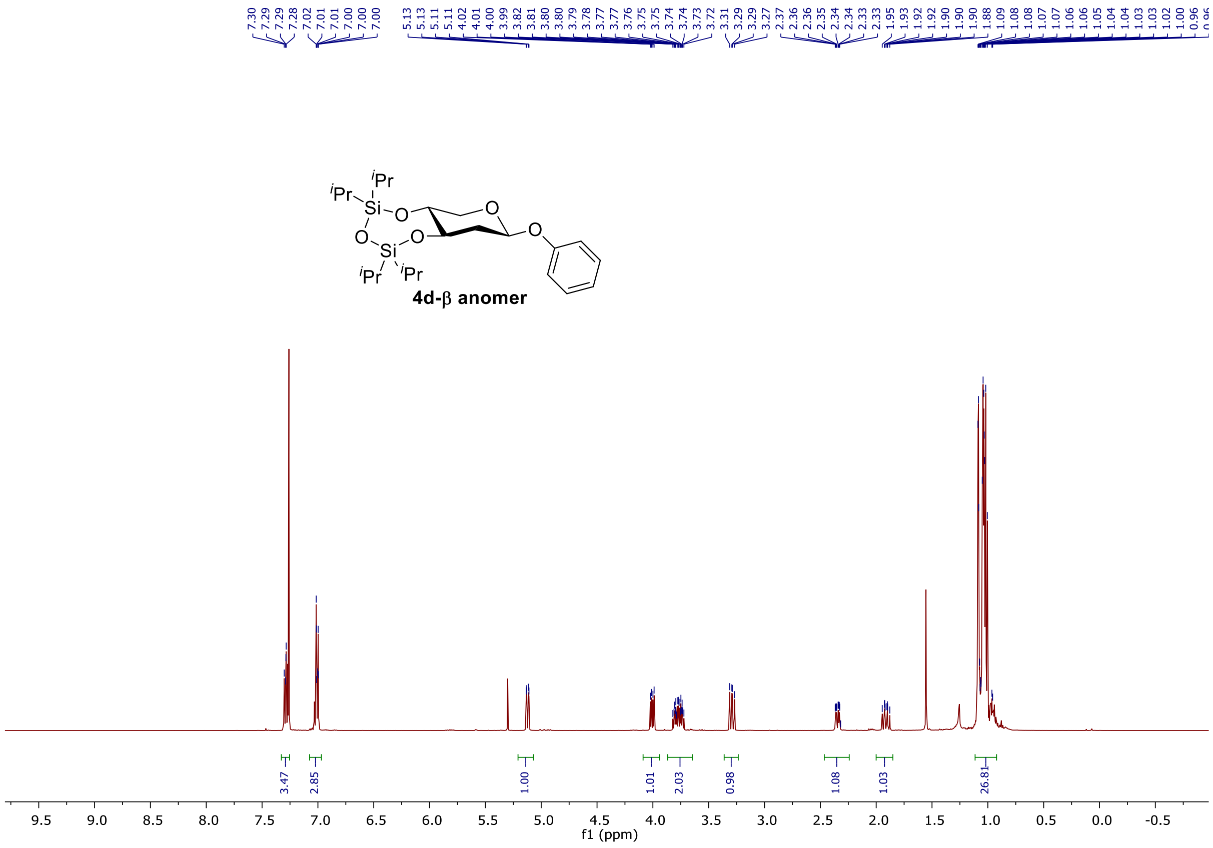

Supplementary figure S428: ^{1}H spectra for **4d-β anomer**

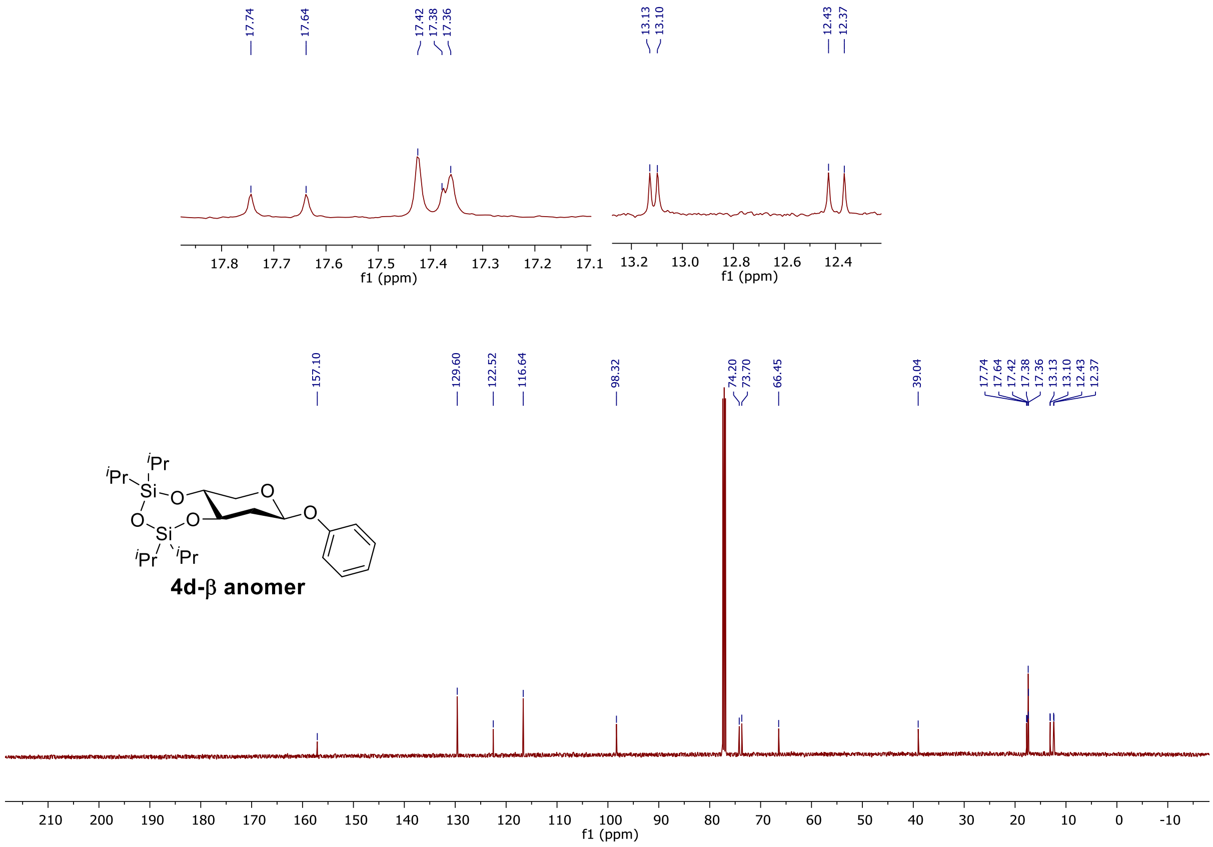

Supplementary figure S429: ^{13}C spectra for **4d-β anomer**

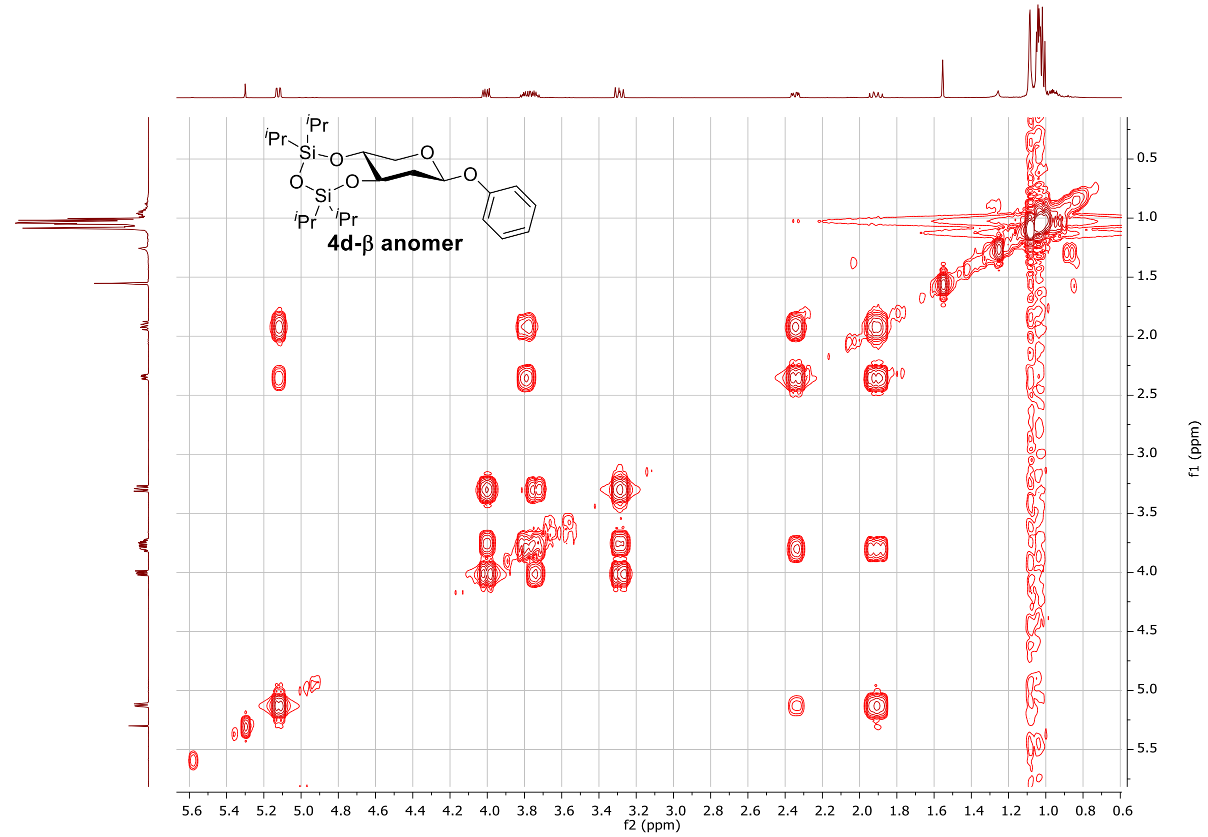

Supplementary figure S430: COSY spectra for **4d-β anomer**

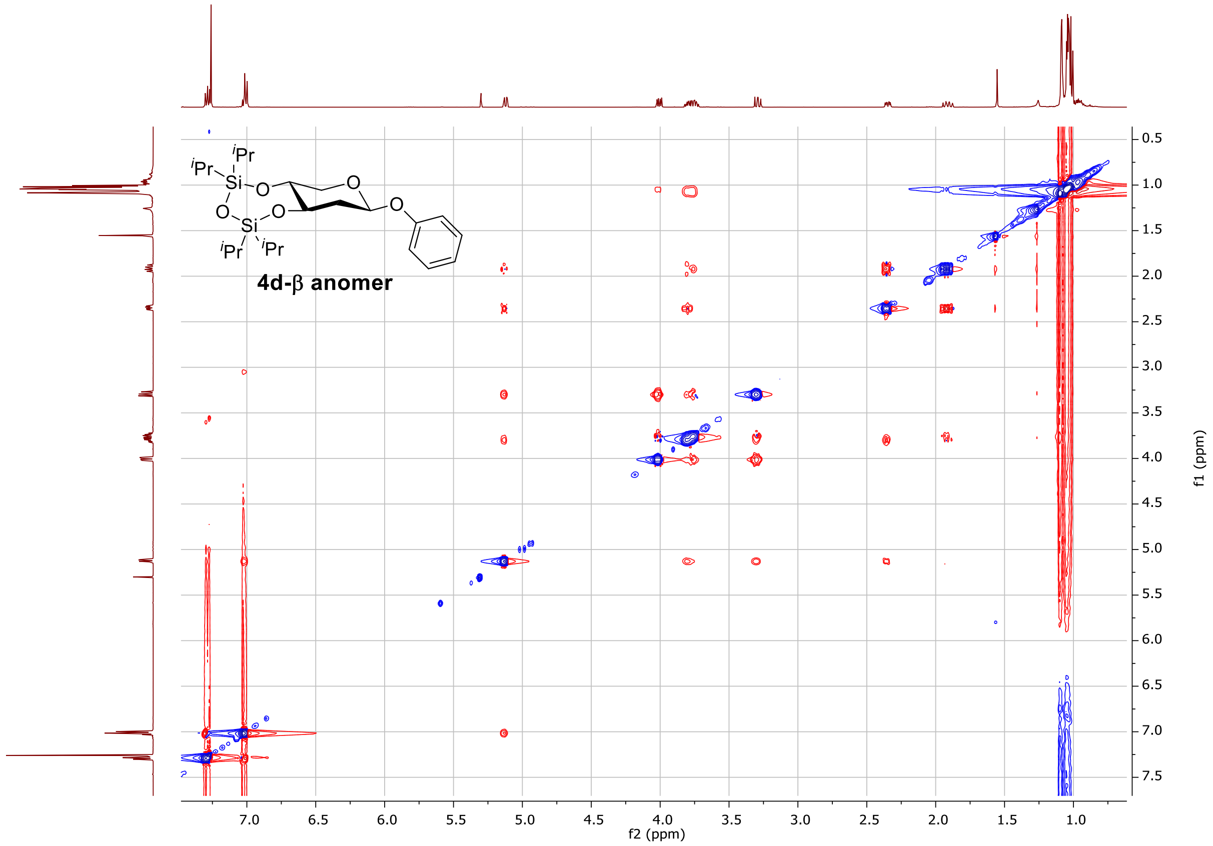

Supplementary figure S431: NOESY spectra for **4d-β anomer**

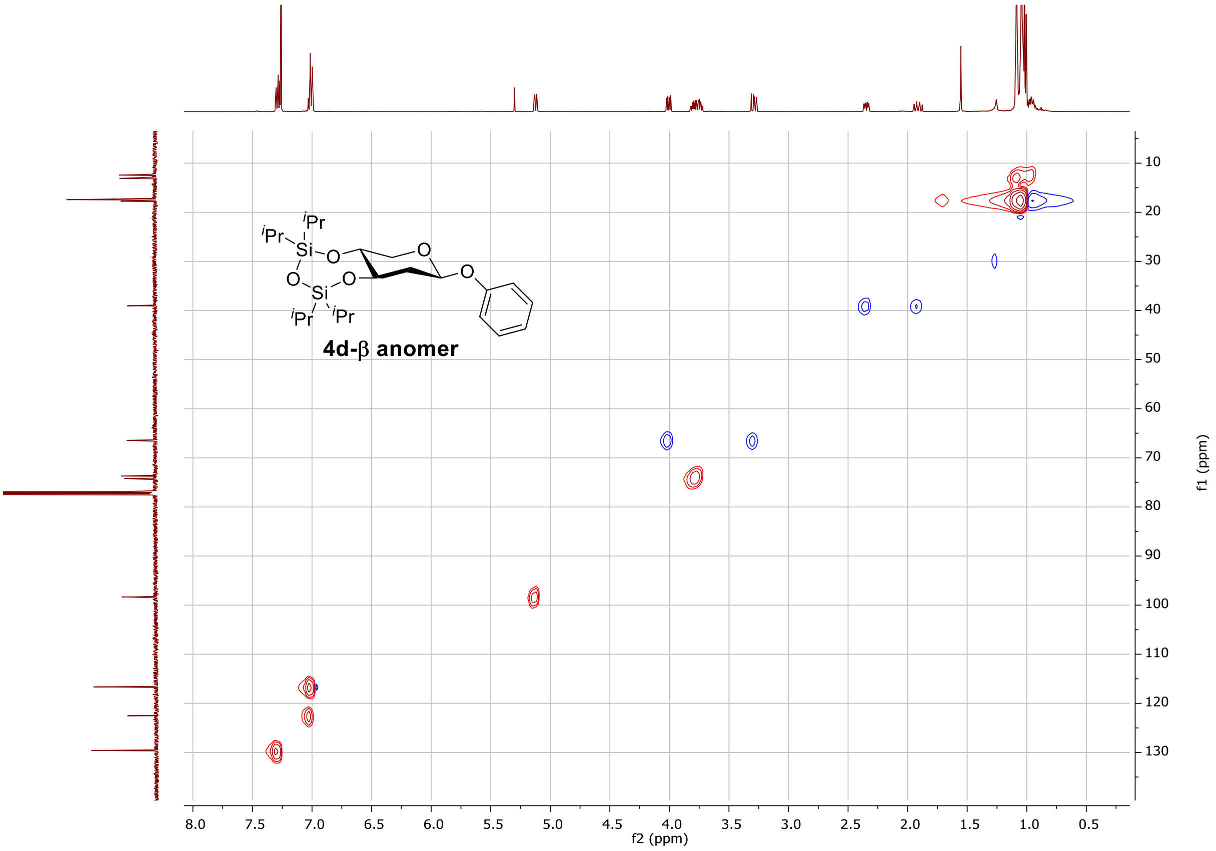

Supplementary figure S432: HSQC spectra for **4d-β anomer**

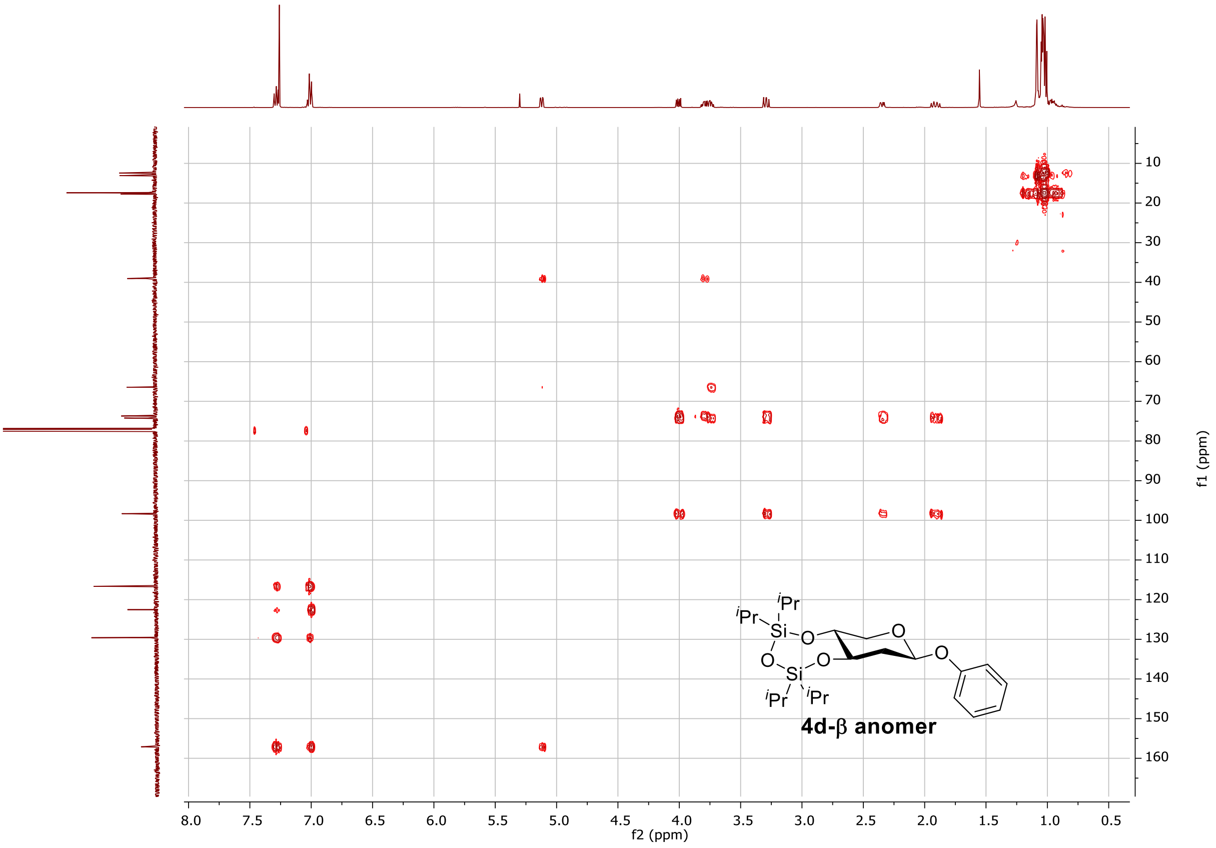

Supplementary figure S433: HMBC spectra for **4d-β anomer**

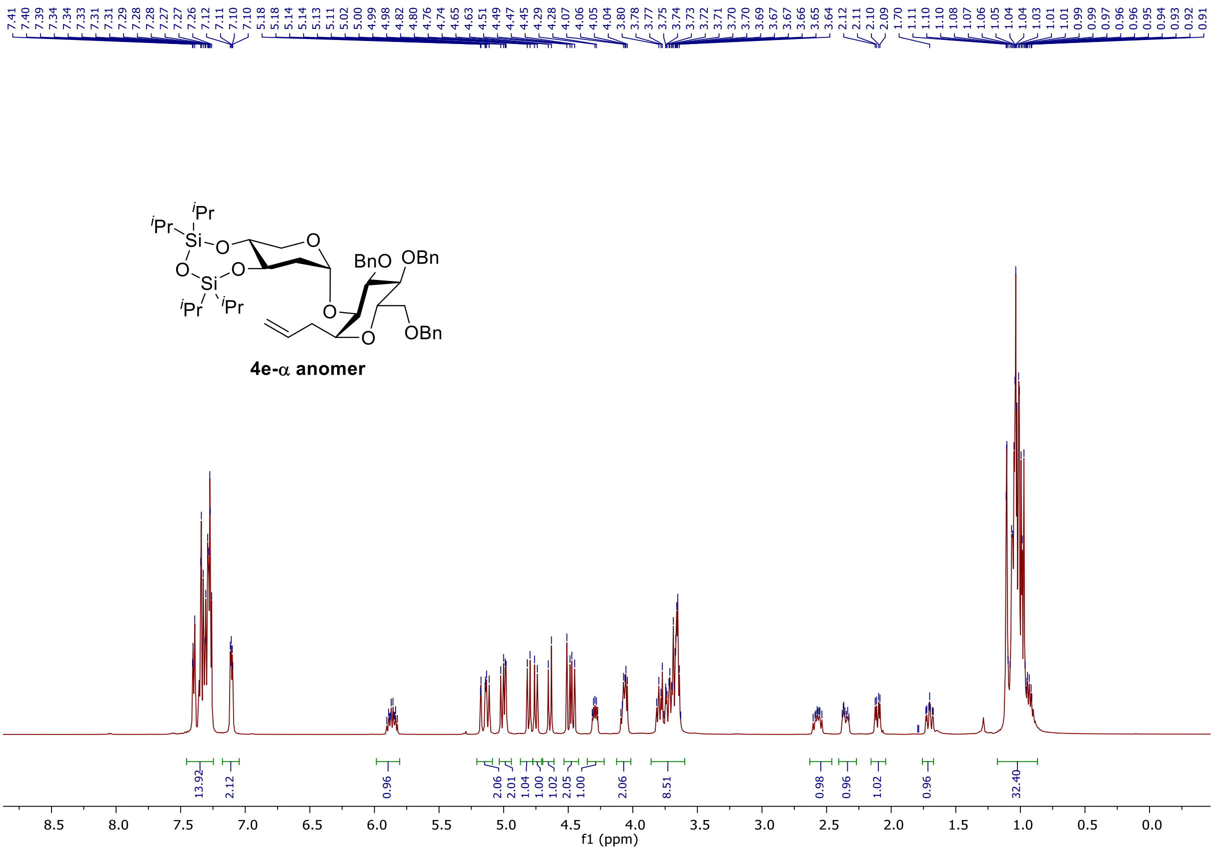

Supplementary figure S434: ^{1}H spectra for **4e-α anomer**

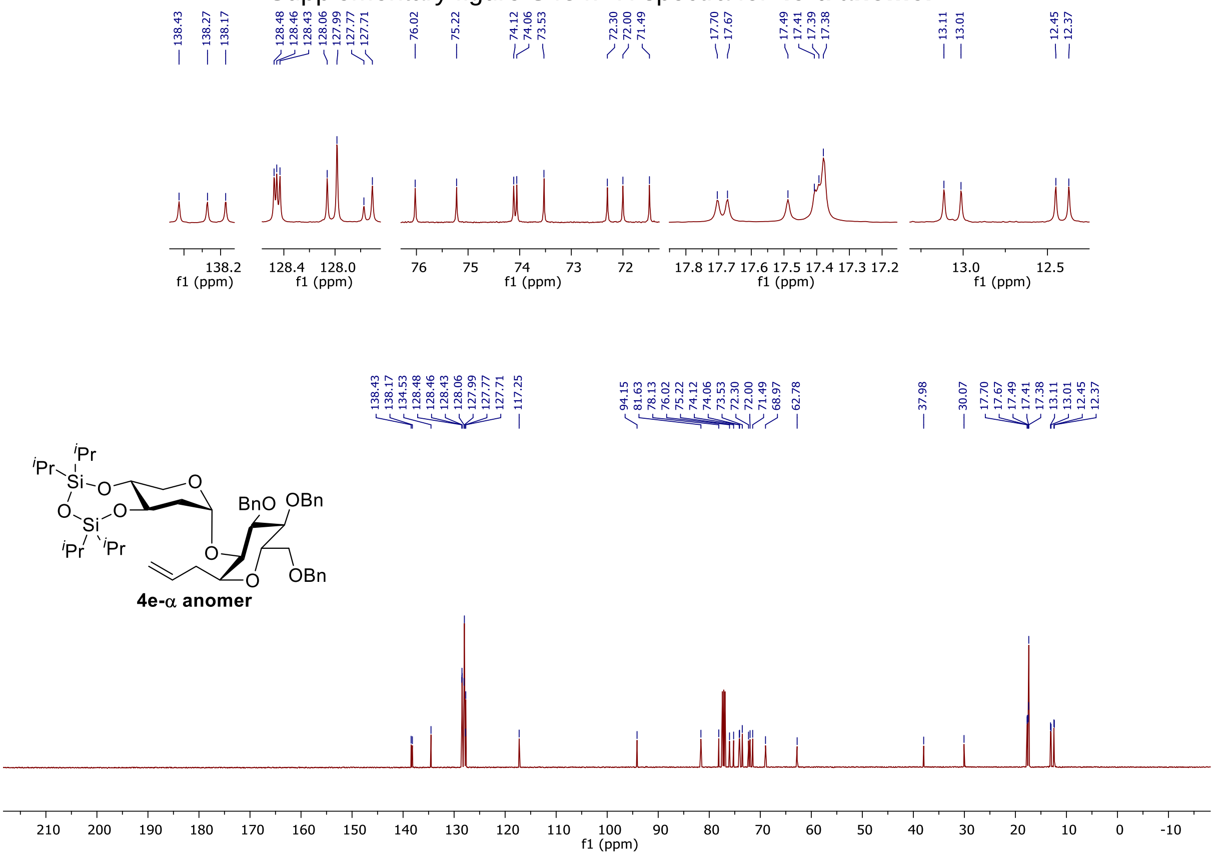

Supplementary figure S435: ^{13}C spectra for **4e-α anomer**

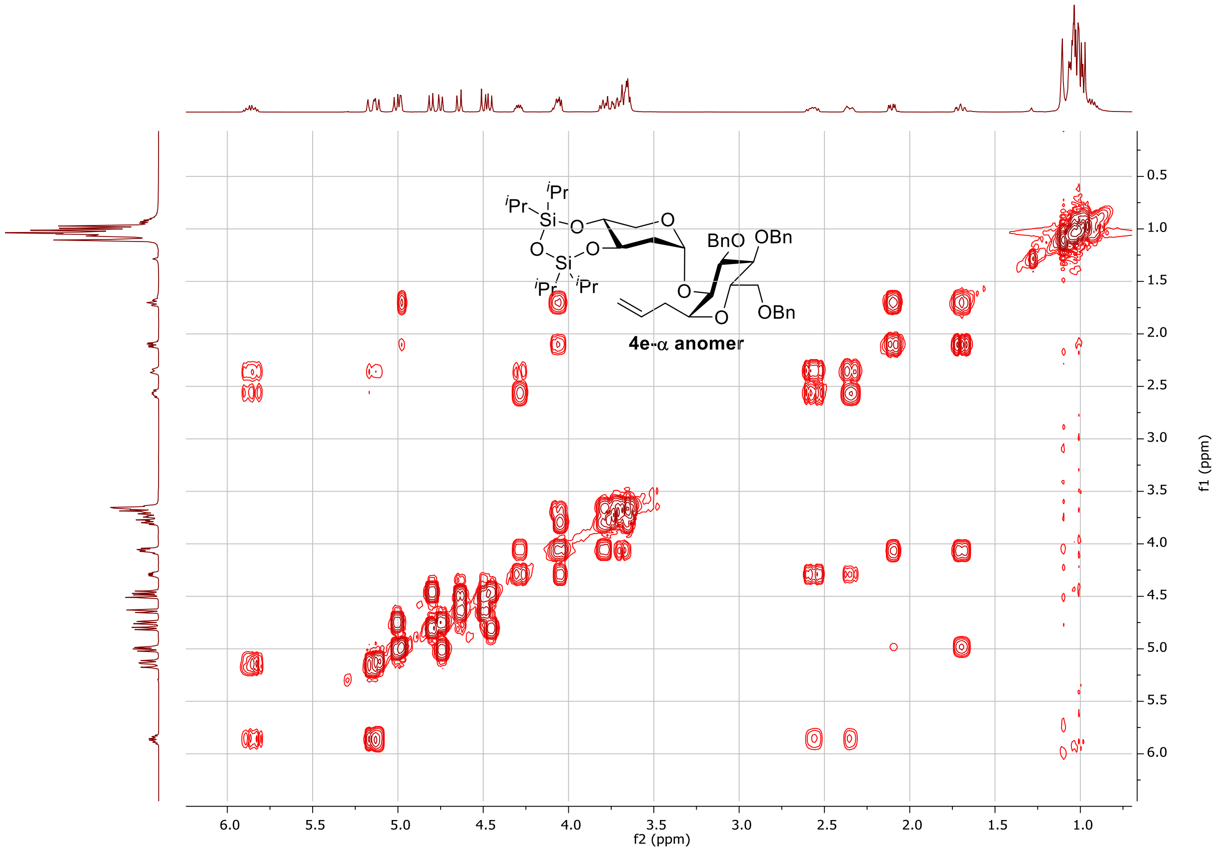

Supplementary figure S436: COSY spectra for **4e-α anomer**

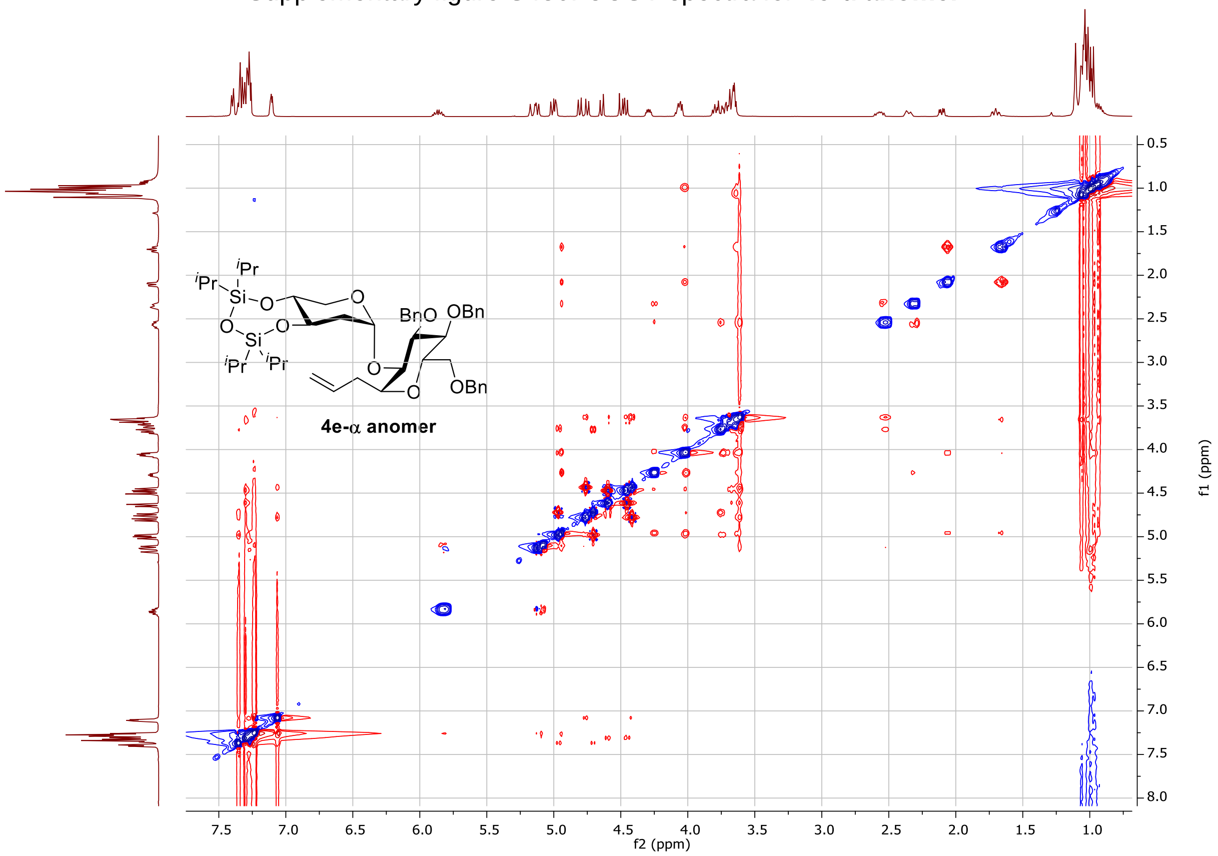

Supplementary figure S437: NOESY spectra for **4e-α anomer**

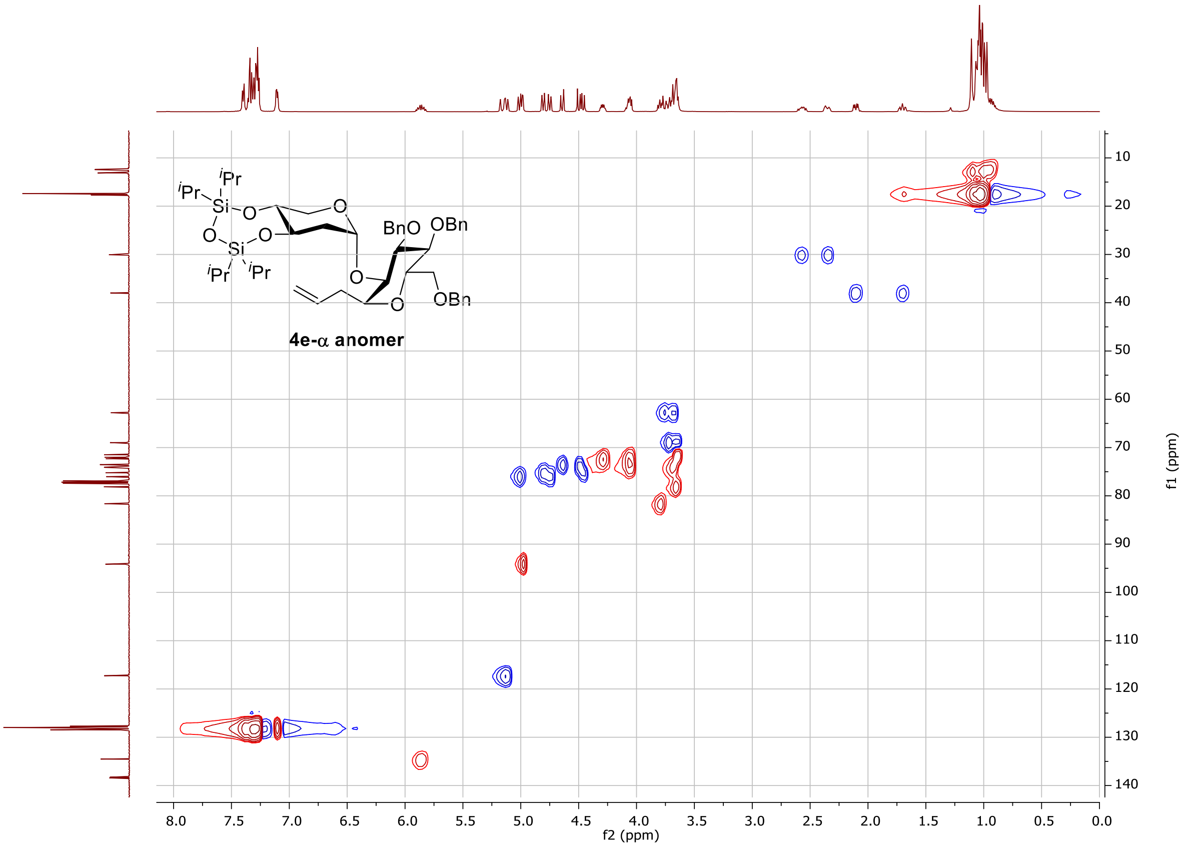

Supplementary figure S438: HSQC spectra for **4e-α anomer**

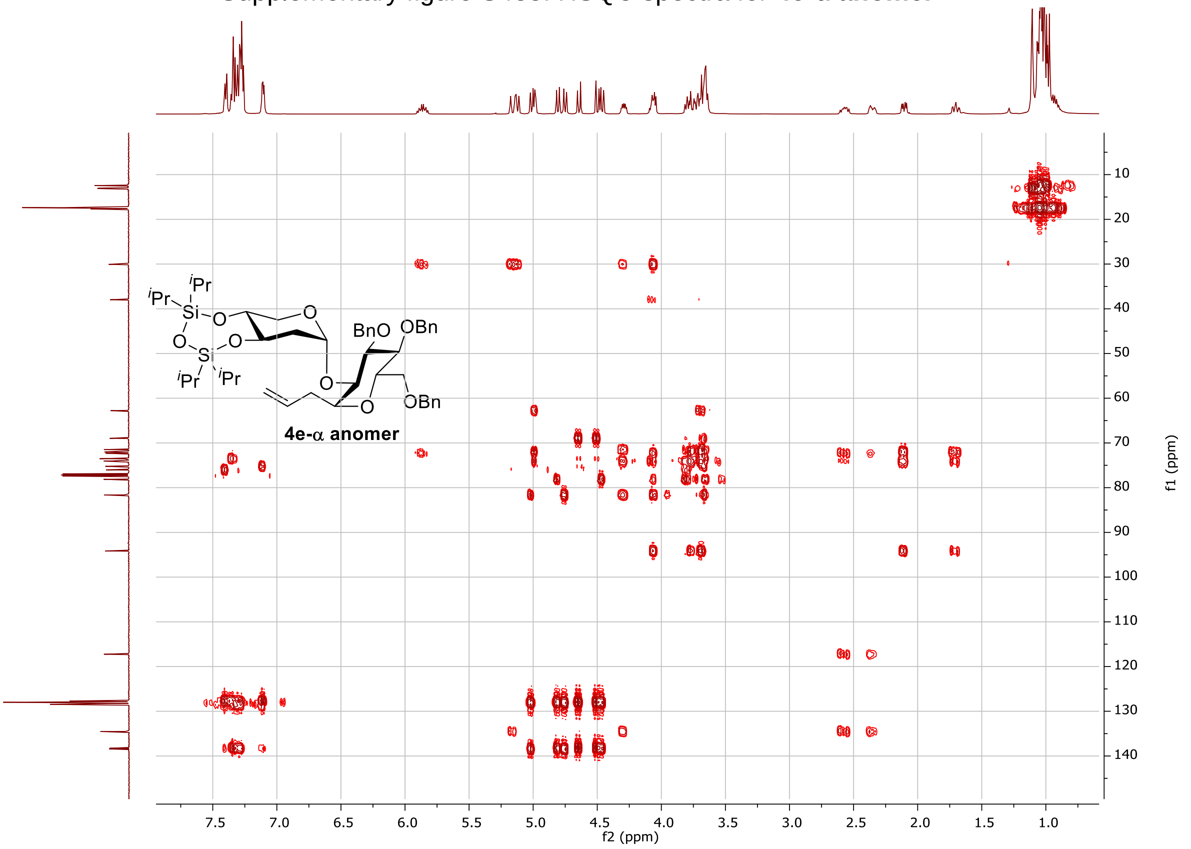

Supplementary figure S439: HMBC spectra for **4e-α anomer**

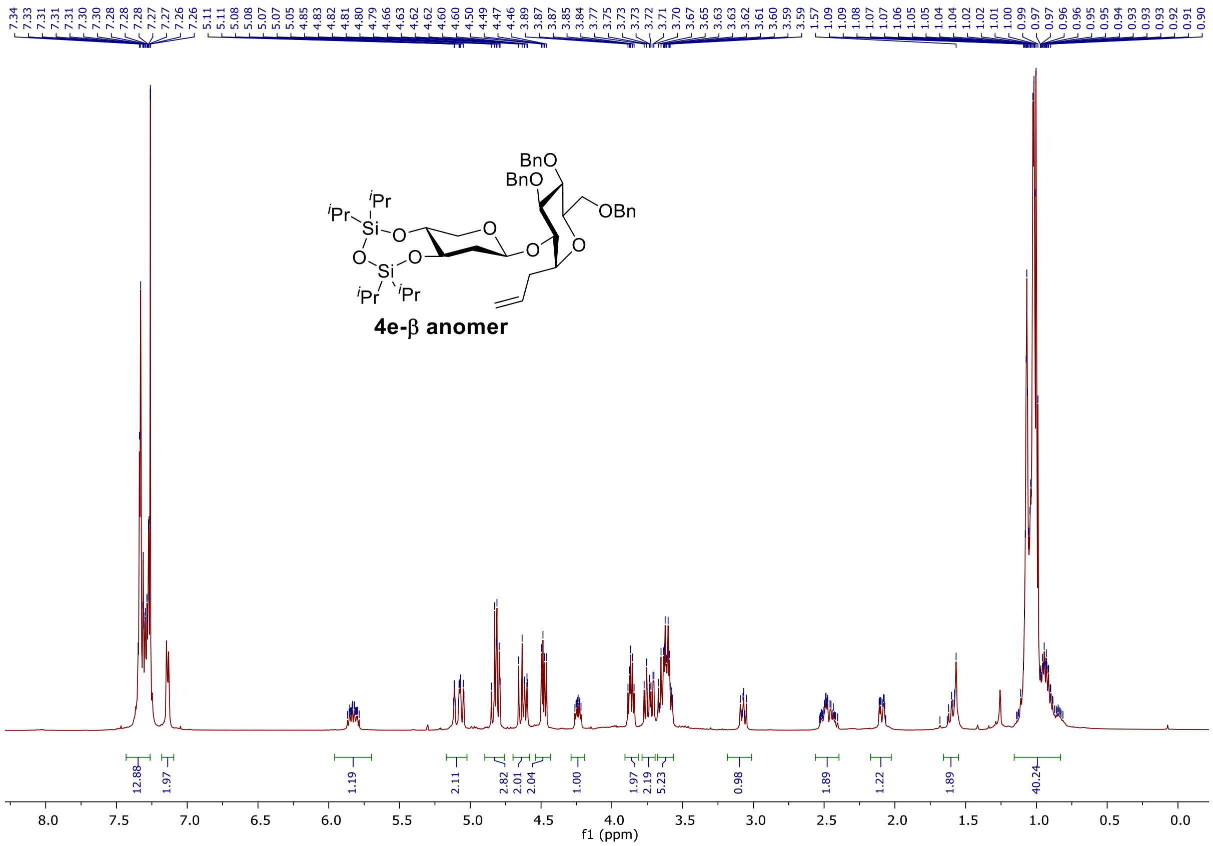

Supplementary figure S440: ^{1}H spectra for **4e-β anomer**

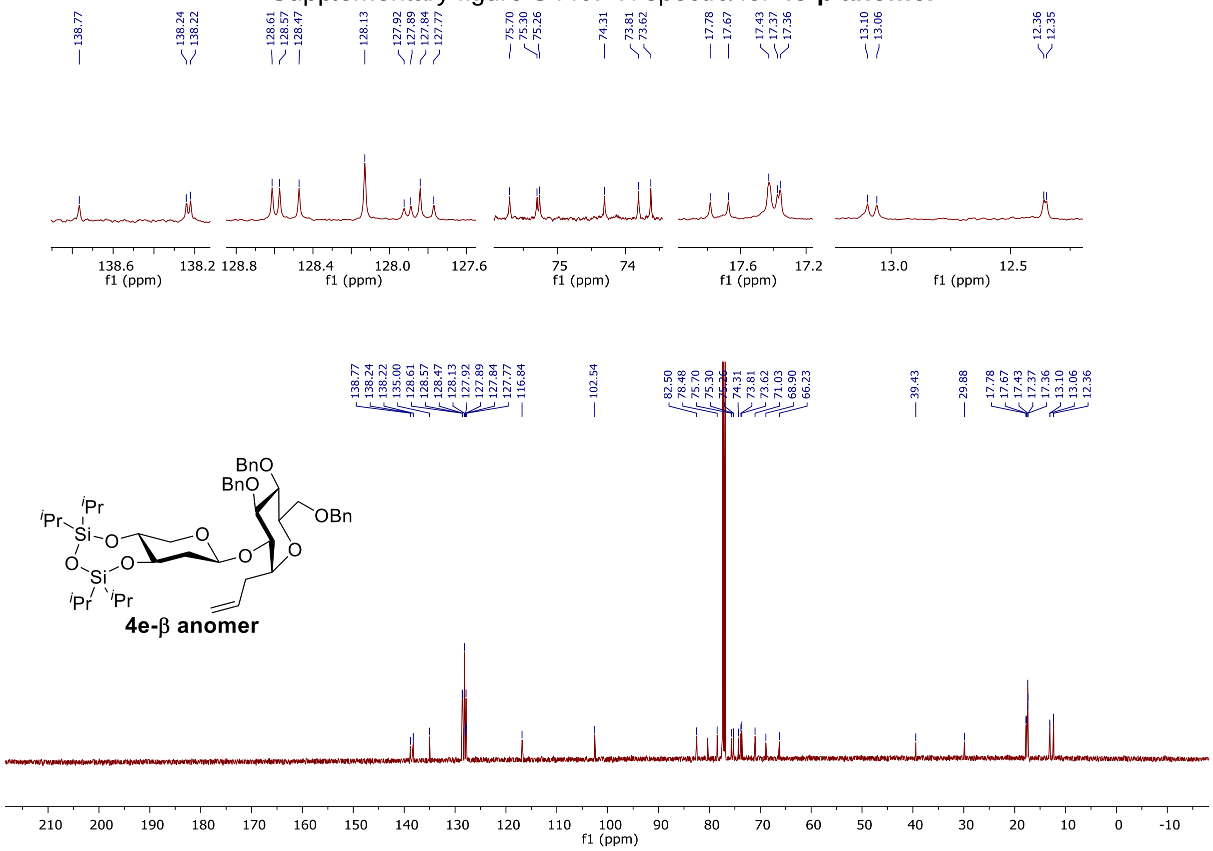

Supplementary figure S441: ^{13}C spectra for **4e-β anomer**

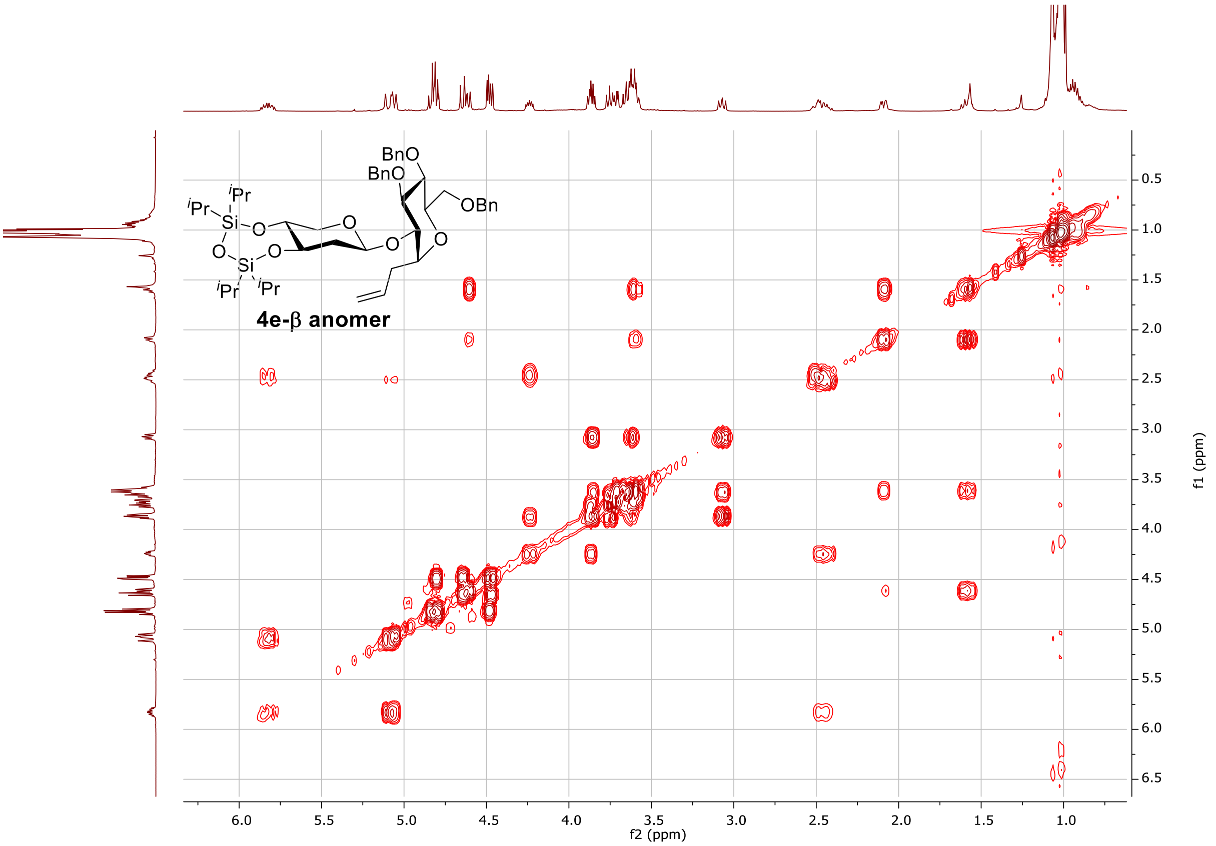

Supplementary figure S442: COSY spectra for **4e-β anomer**

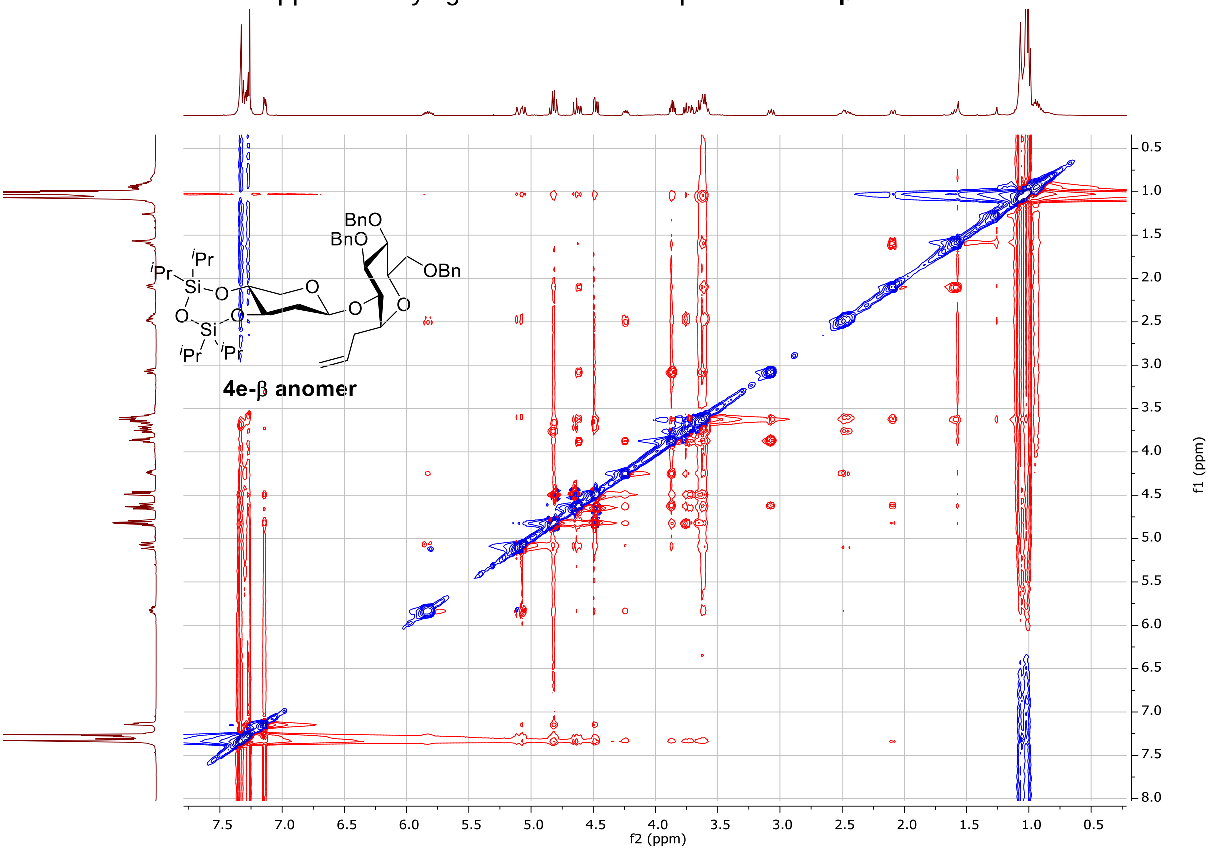

Supplementary figure S443: NOESY spectra for **4e-β anomer**

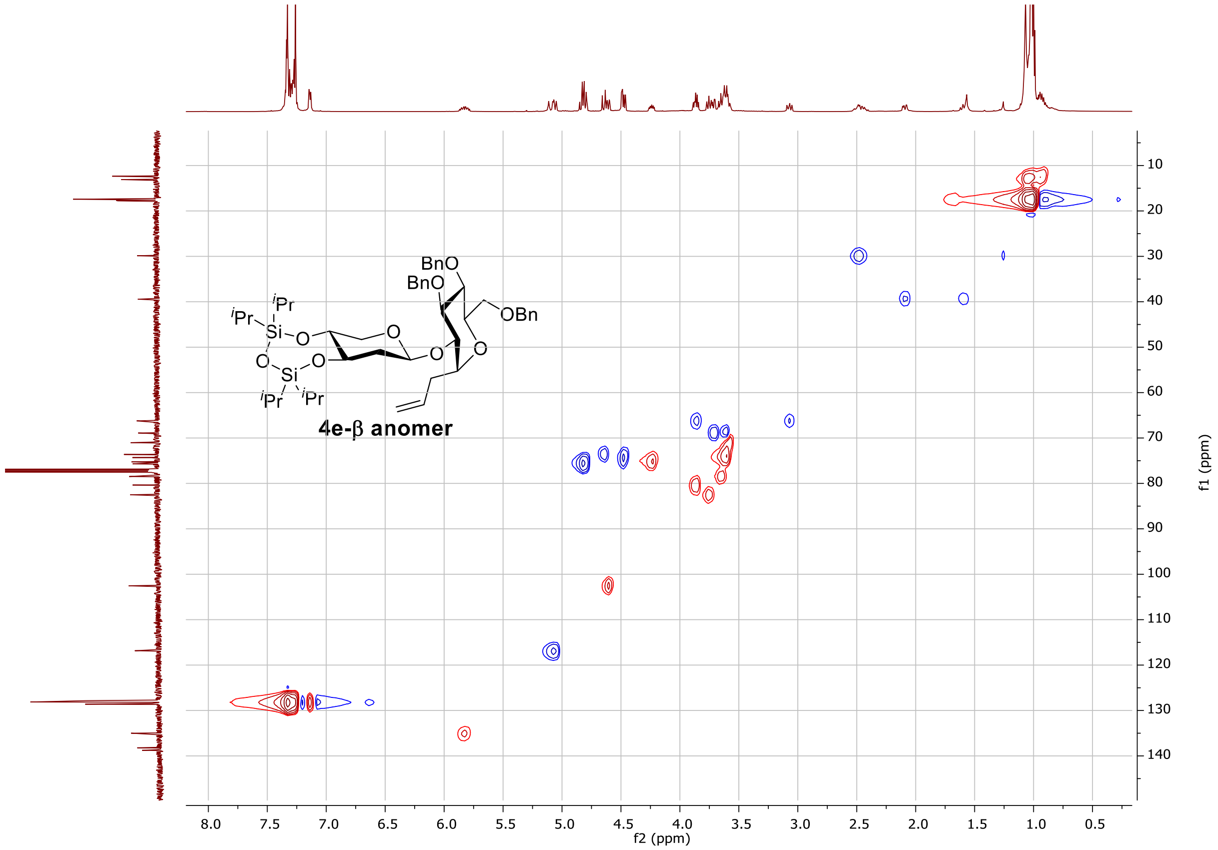

Supplementary figure S444: HSQC spectra for **4e-β anomer**

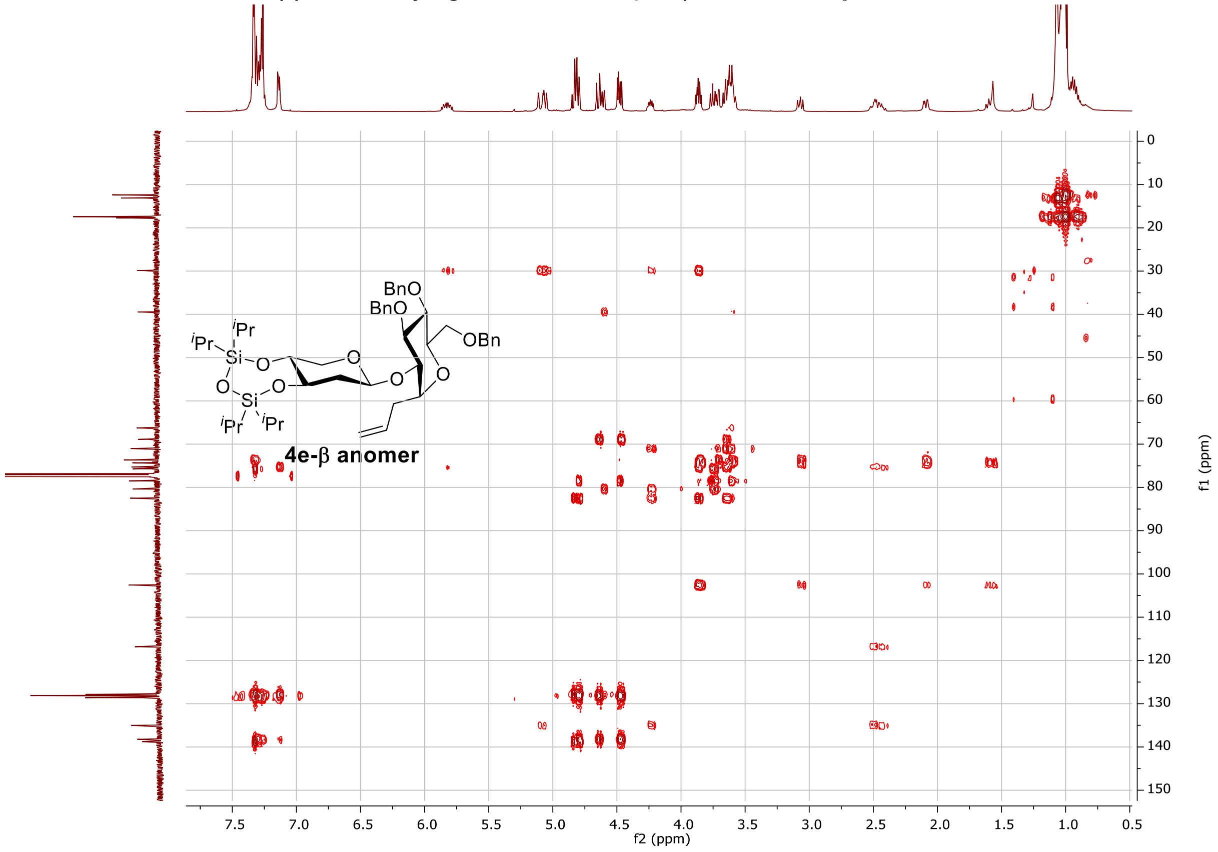

Supplementary figure S445: HMBC spectra for **4e-β anomer**

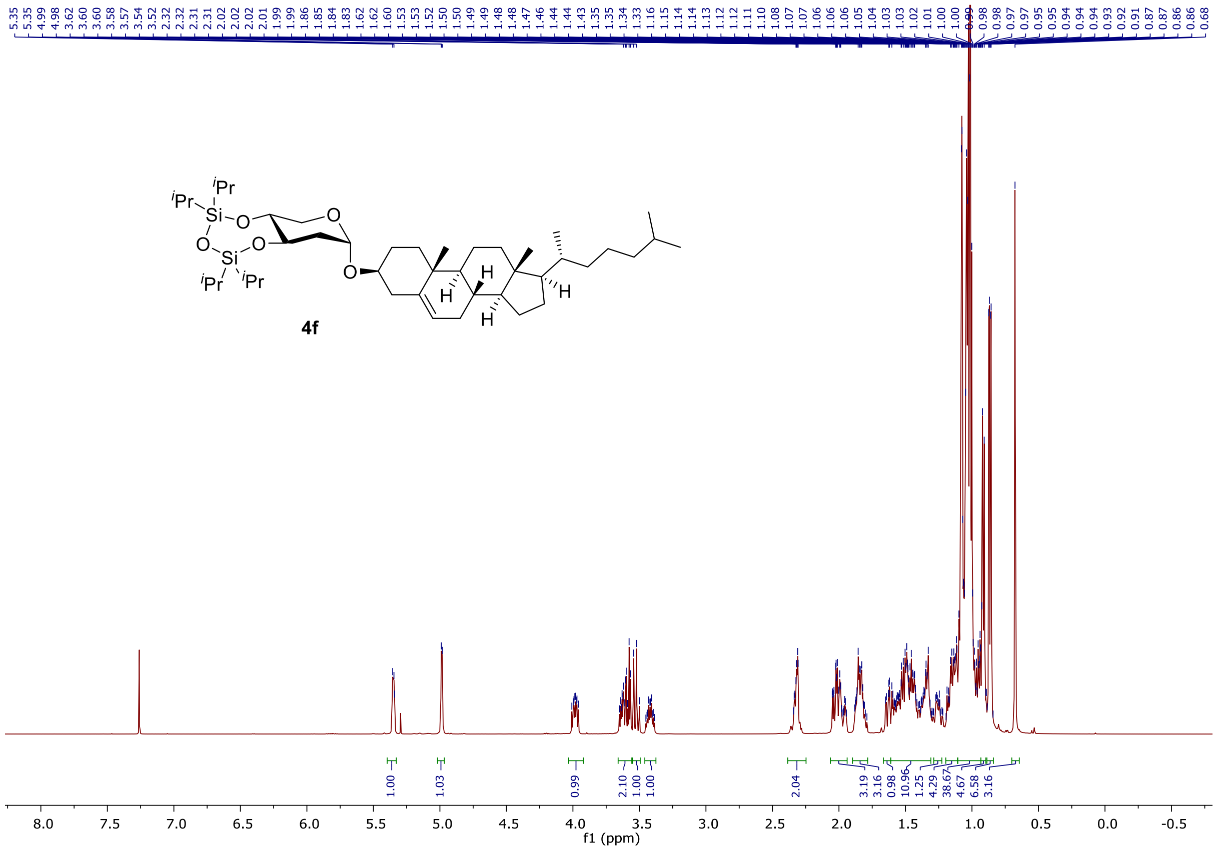

Supplementary figure S446: ^{1}H spectra for **4f**

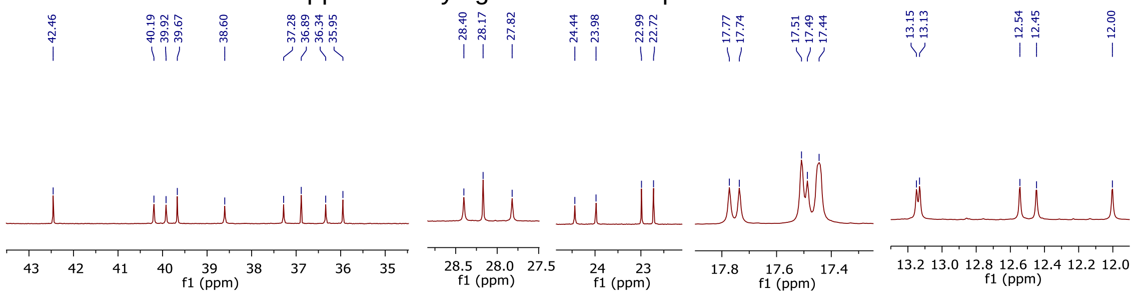

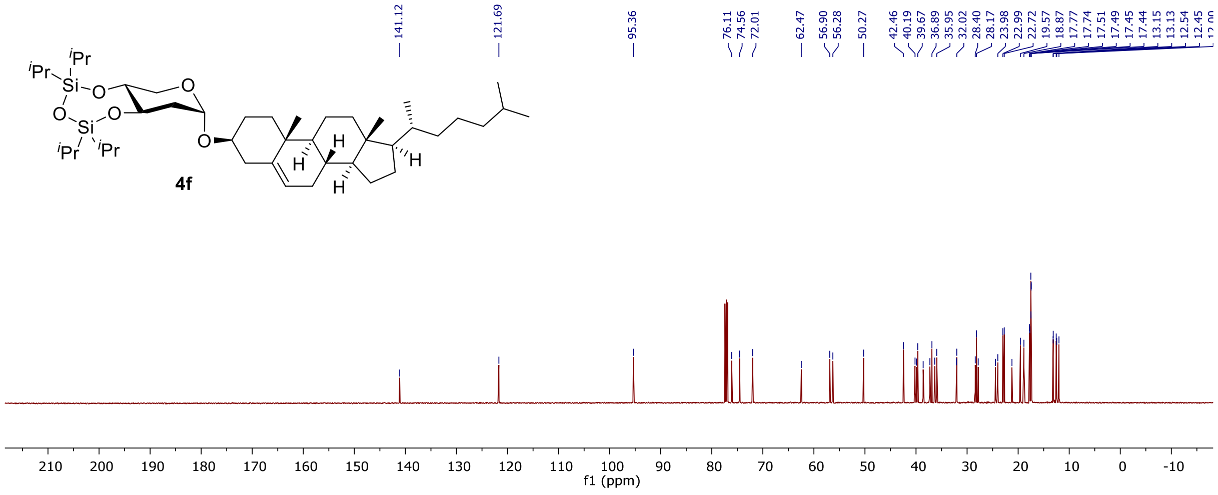

Supplementary figure S447: ^{13}C spectra for **4f**

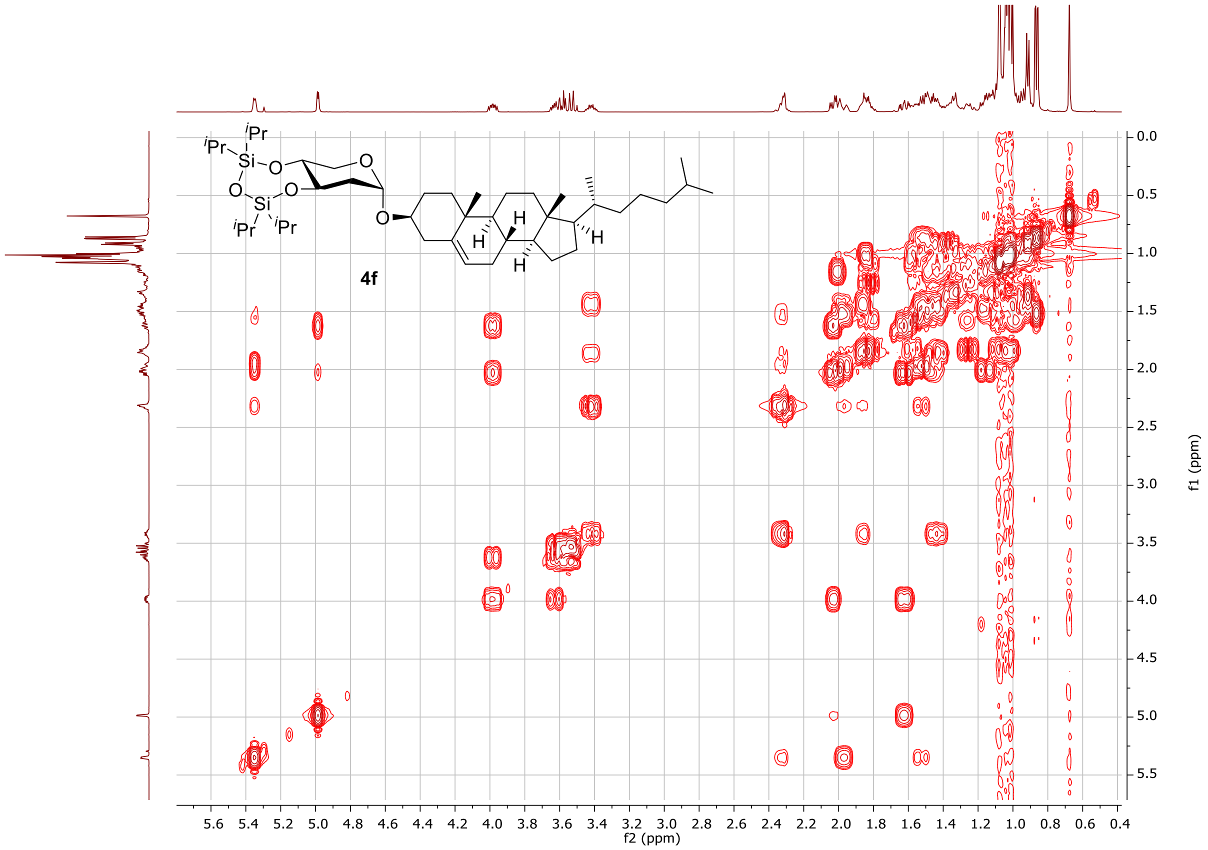

Supplementary figure S448: COSY spectra for **4f**

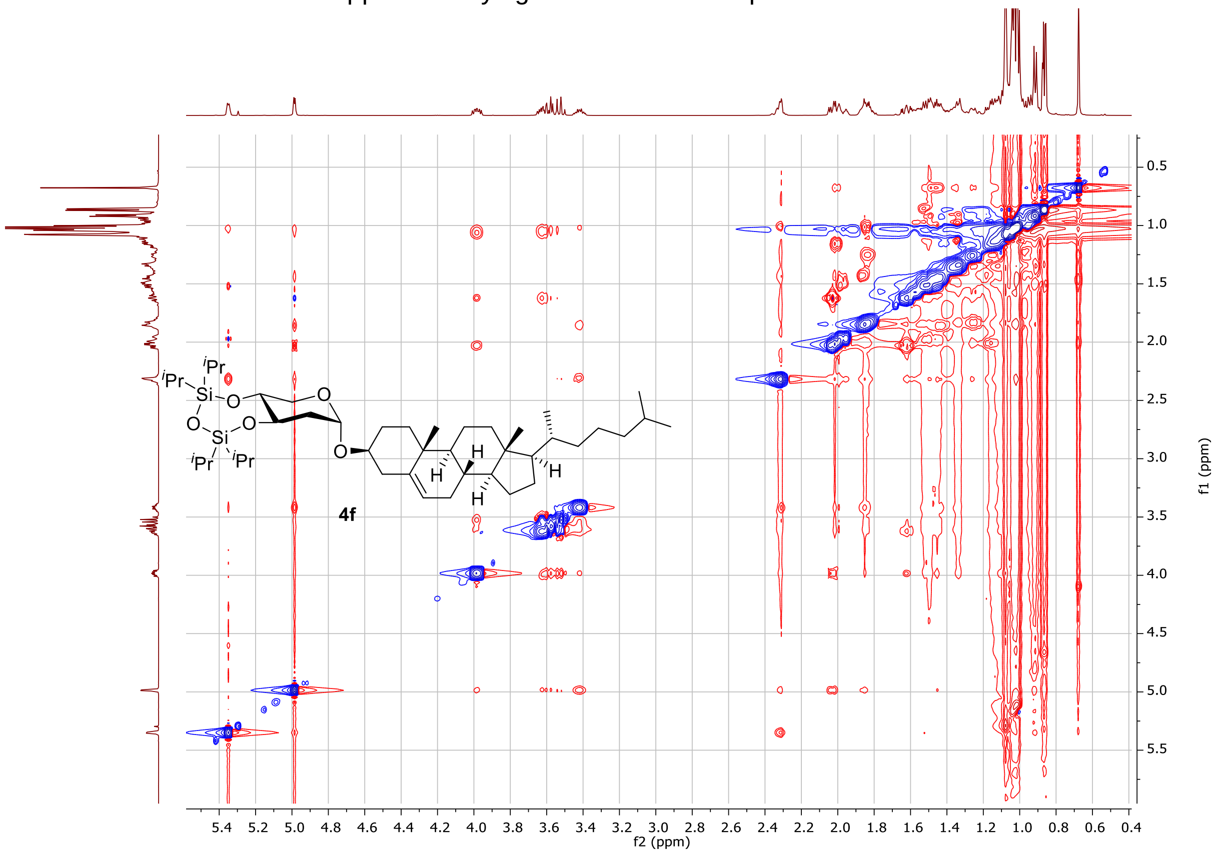

Supplementary figure S449: NOESY spectra for **4f**

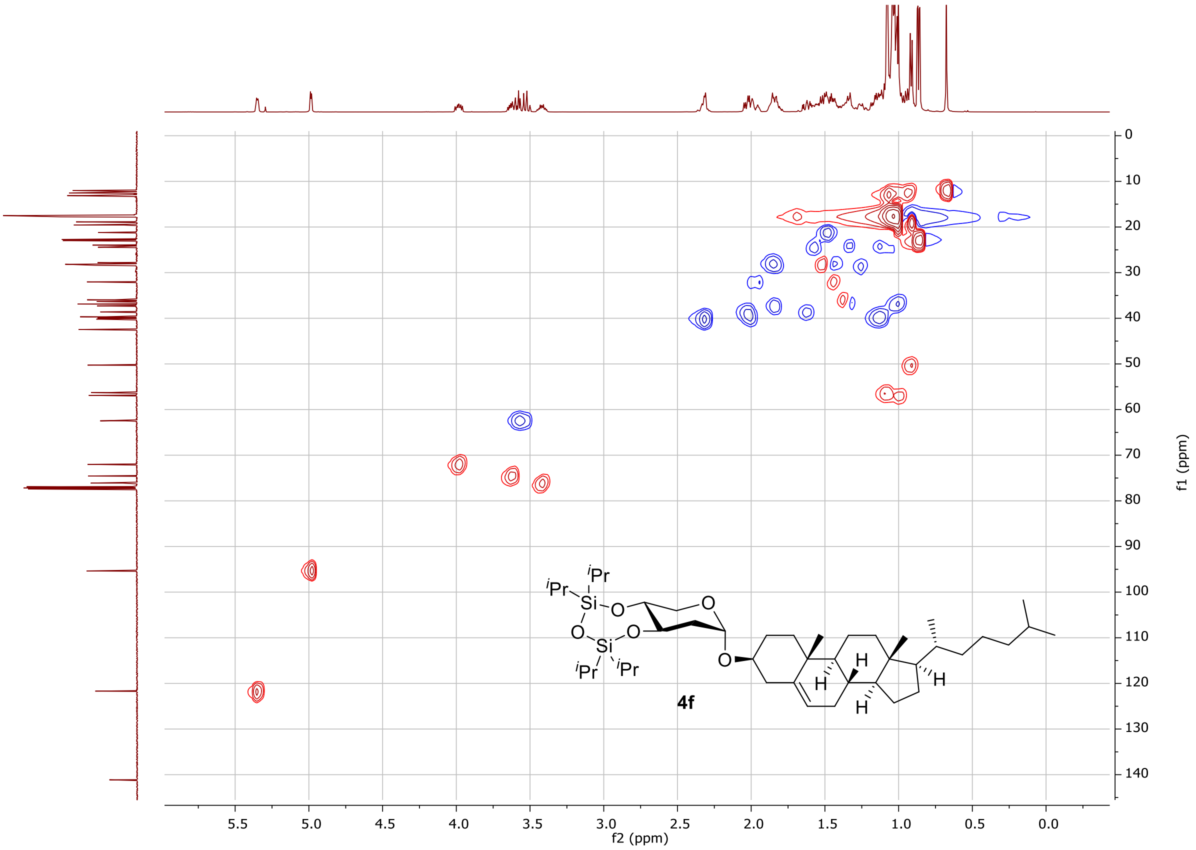

Supplementary figure S450: HSQC spectra for **4f**

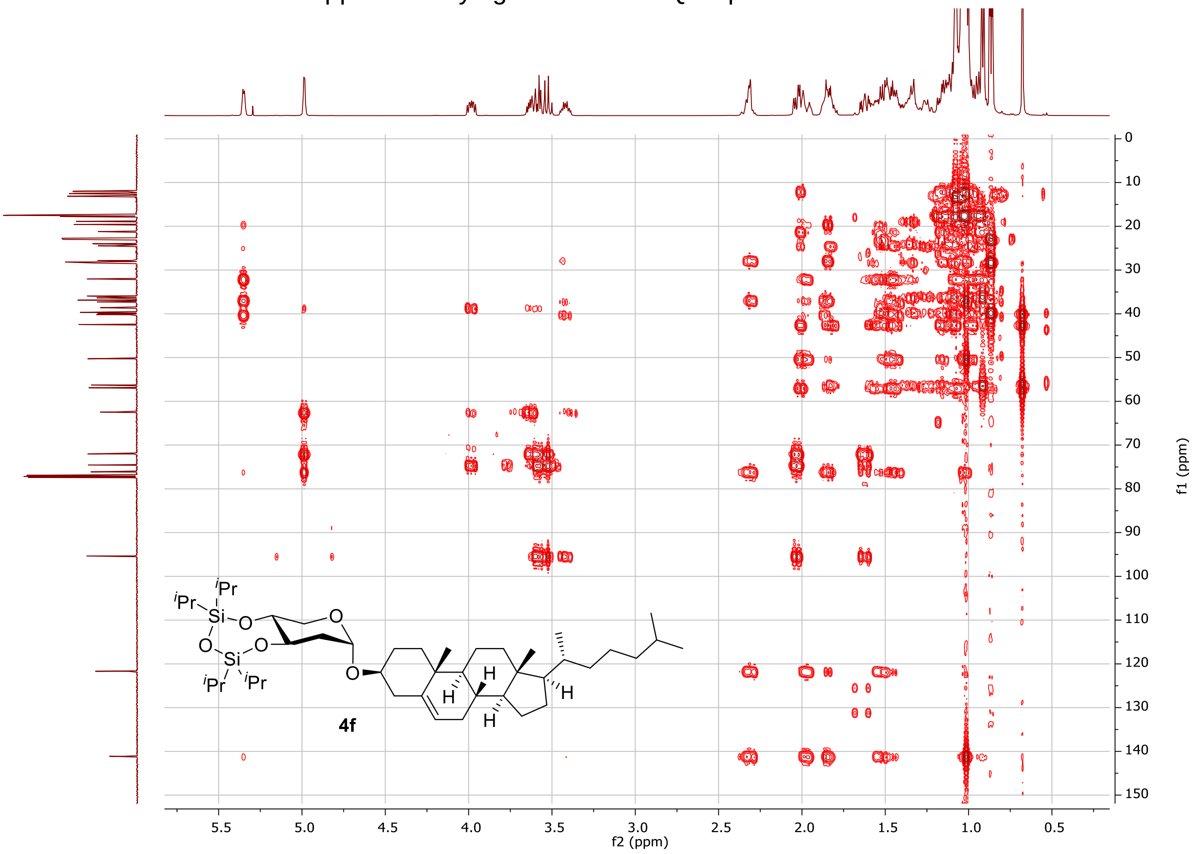

Supplementary figure S451: HMBC spectra for **4f**

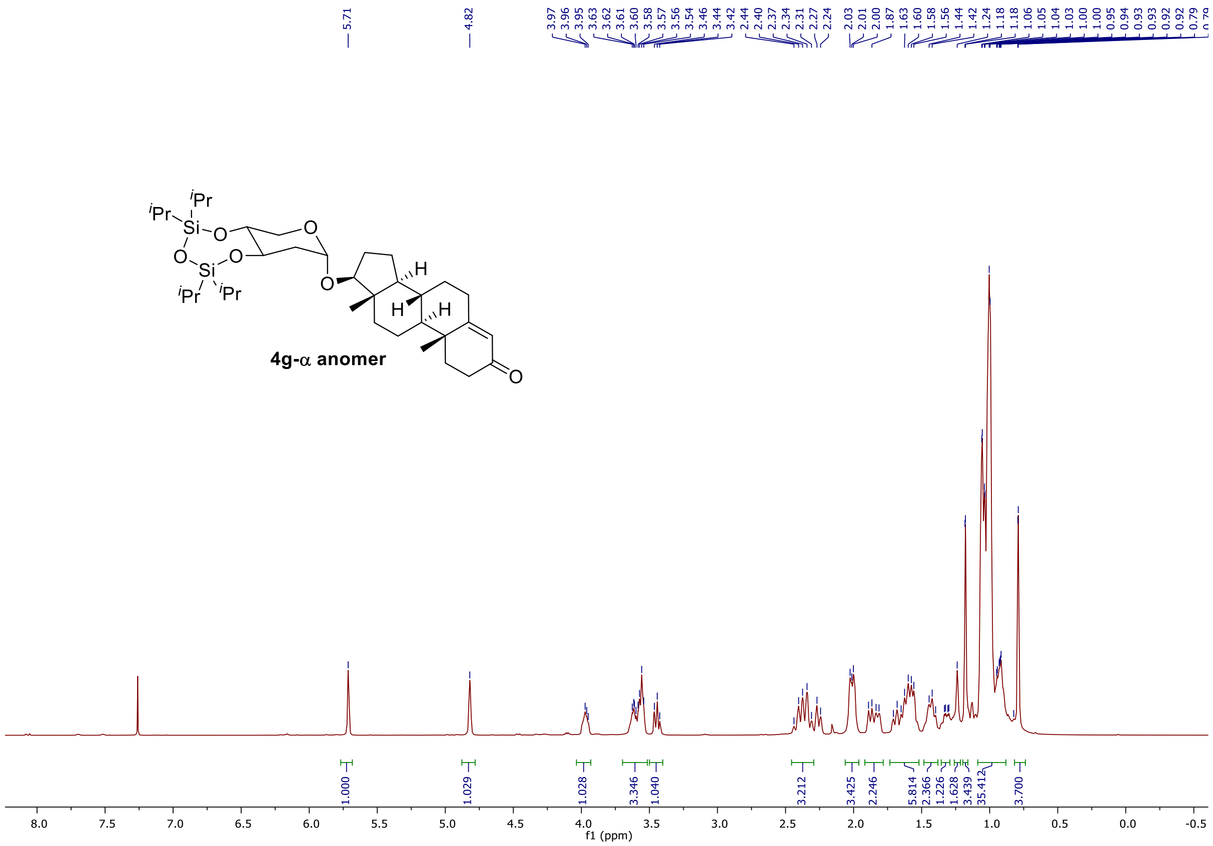

Supplementary figure S452: ^{1}H spectra for **4g-α anomer**

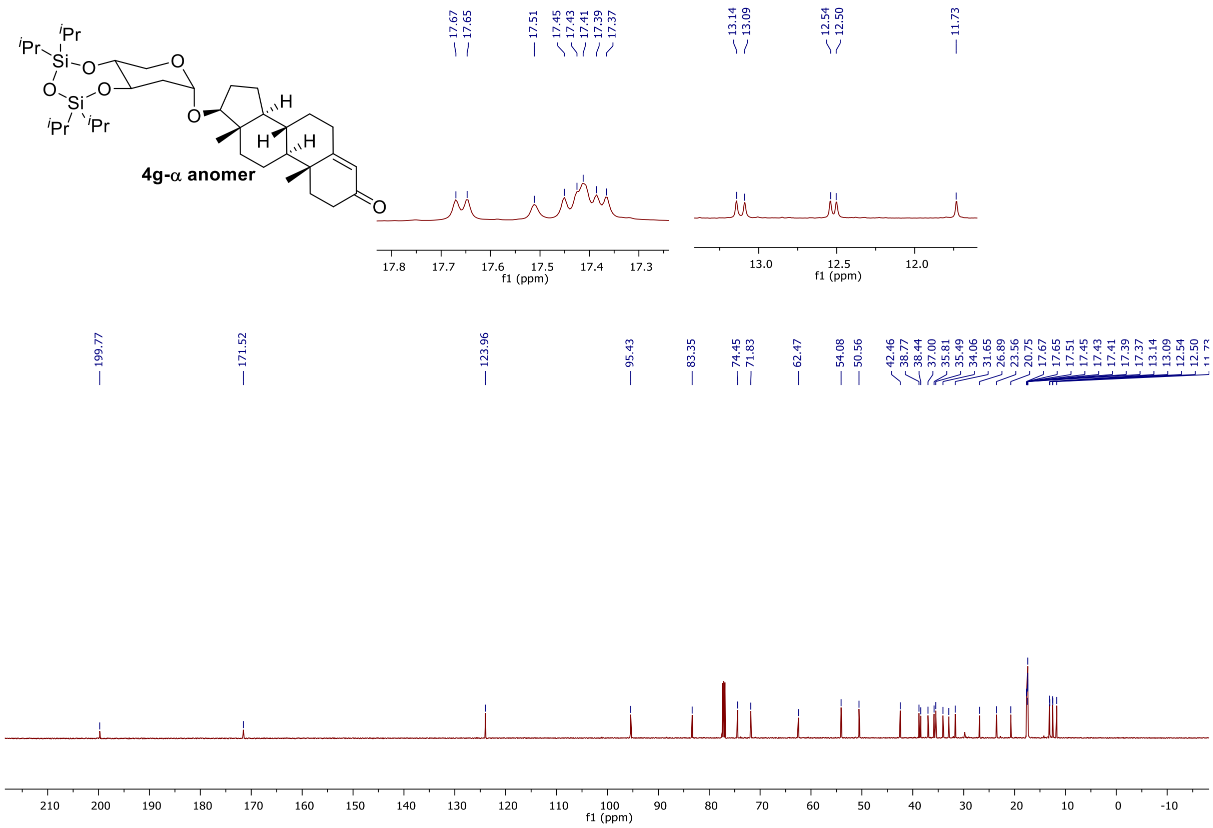

Supplementary figure S453: ^{13}C spectra for **4g-α anomer**

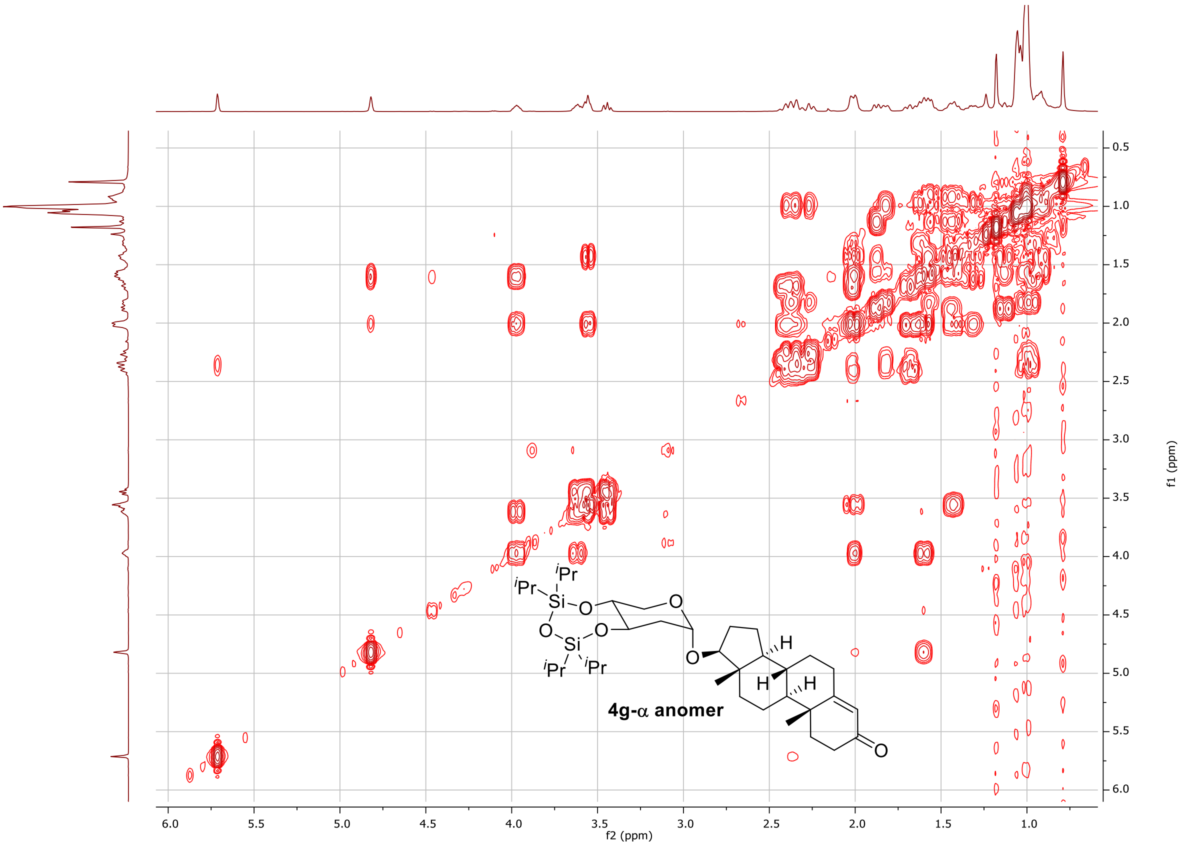

Supplementary figure S454: COSY spectra for **4g-α anomer**

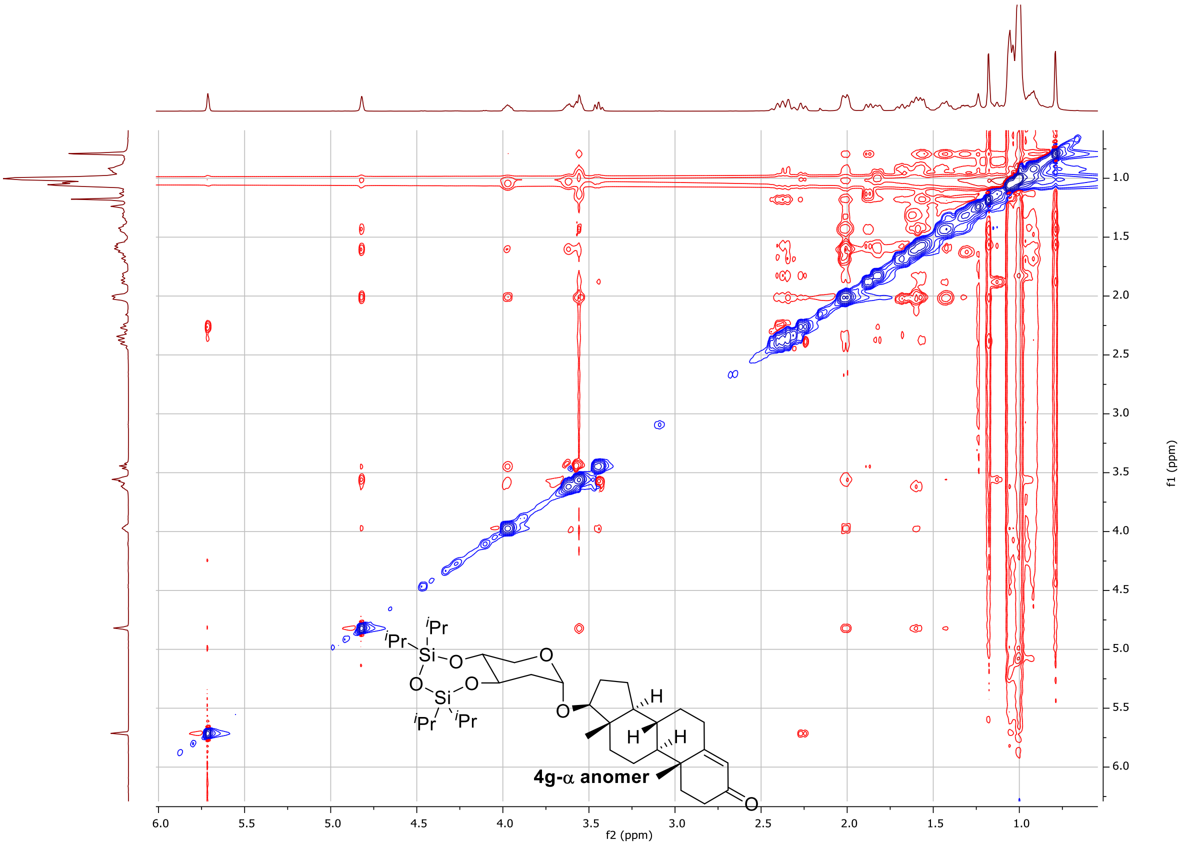

Supplementary figure S455: NOESY spectra for **4g-α anomer**

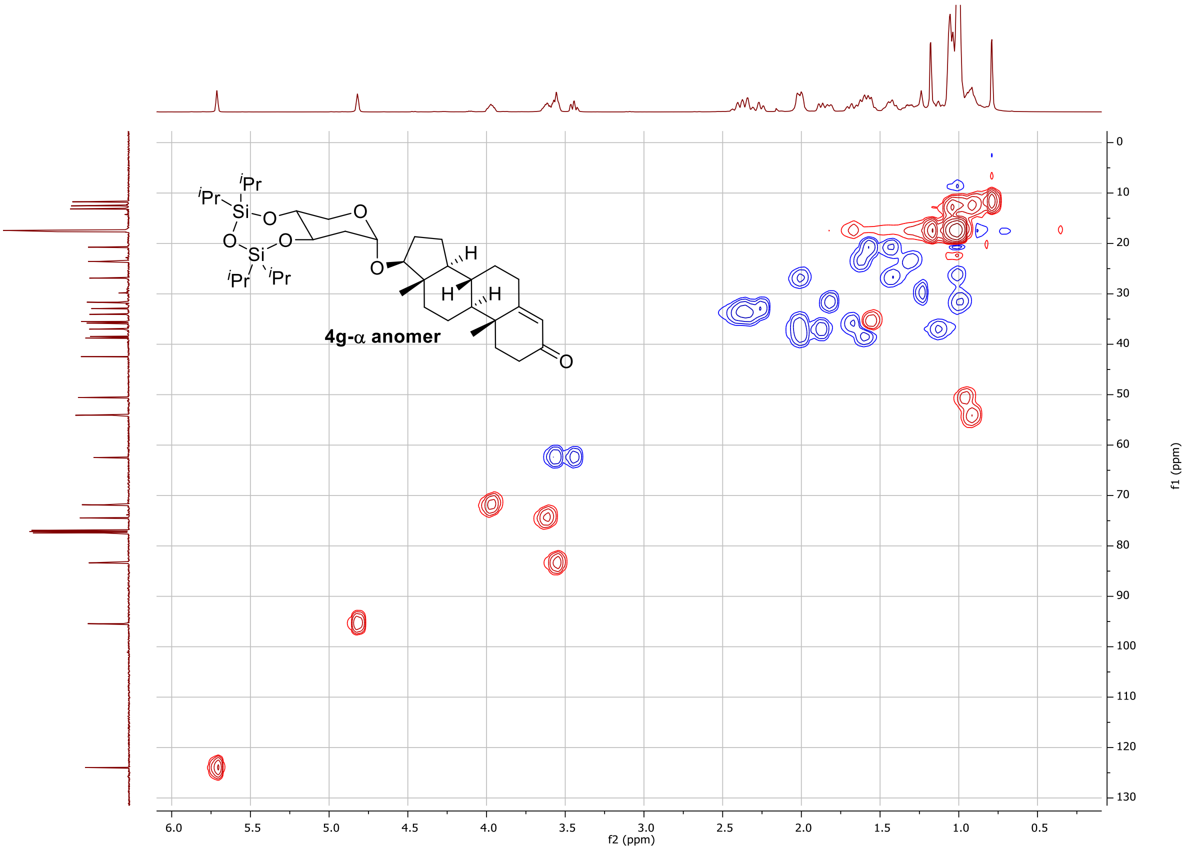

Supplementary figure S456: HSQC spectra for **4g-α anomer**

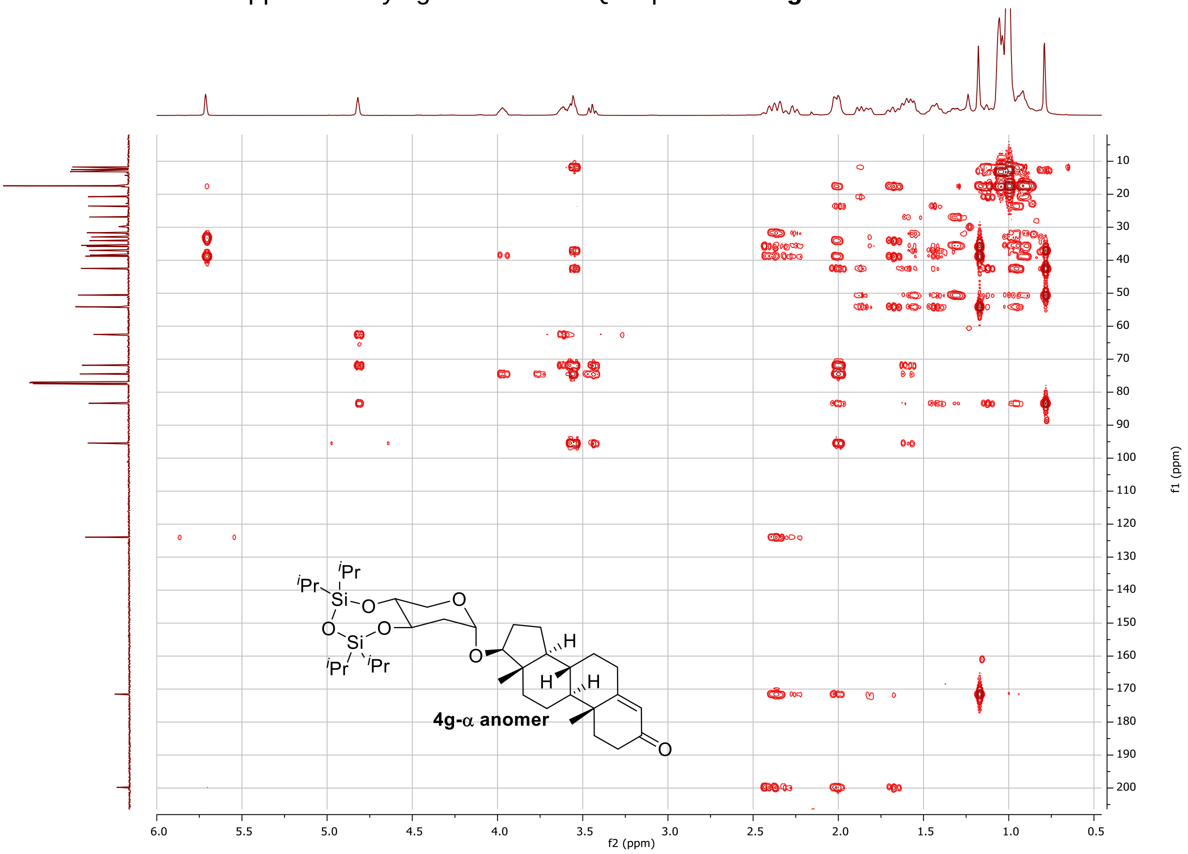

Supplementary figure S457: HMBC spectra for **4g-α anomer**

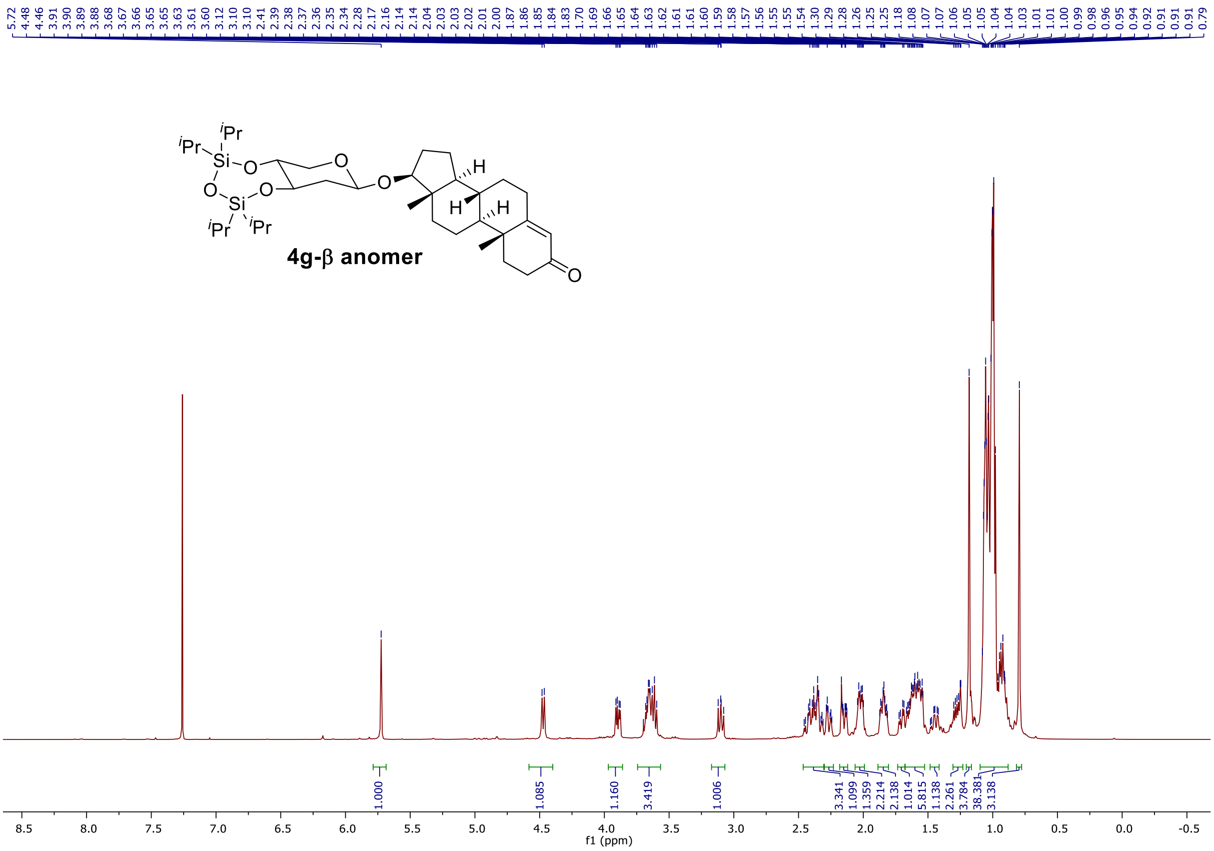

Supplementary figure S458: ^{1}H spectra for **4g-β anomer**

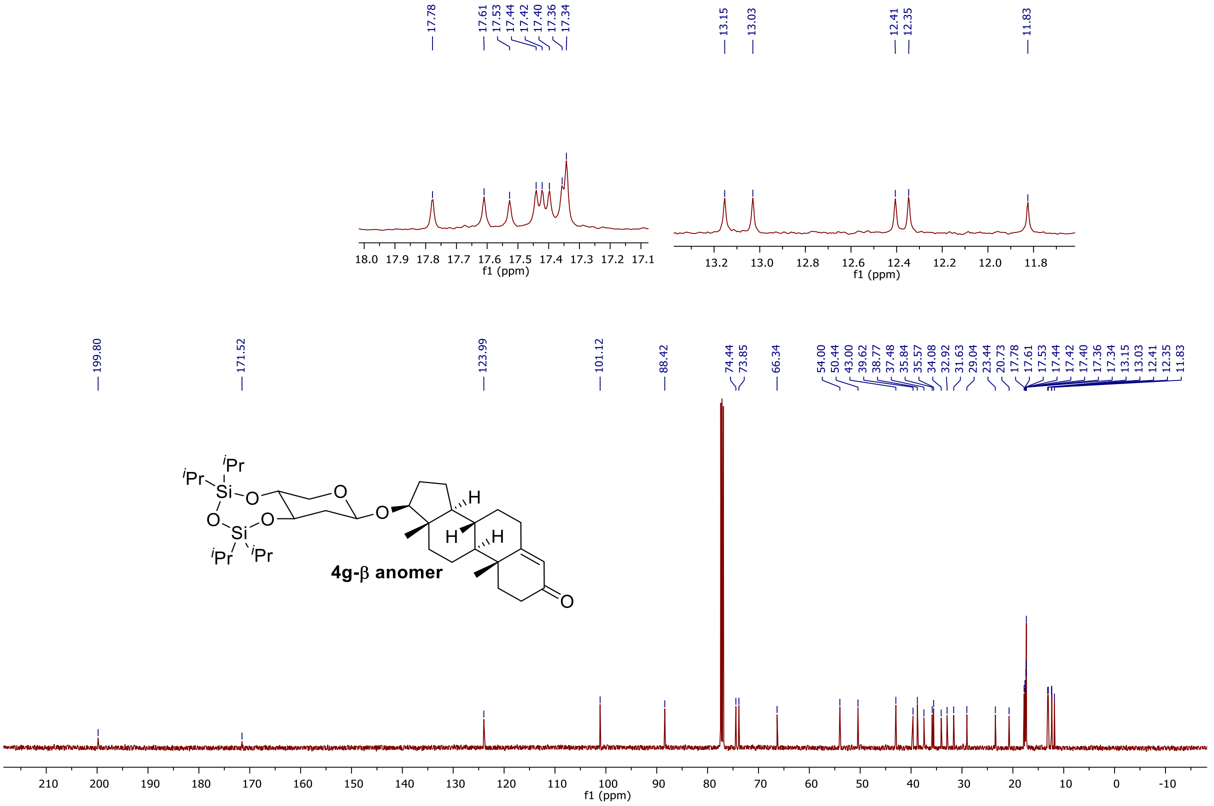

Supplementary figure S459: ^{13}C spectra for **4g-β anomer**

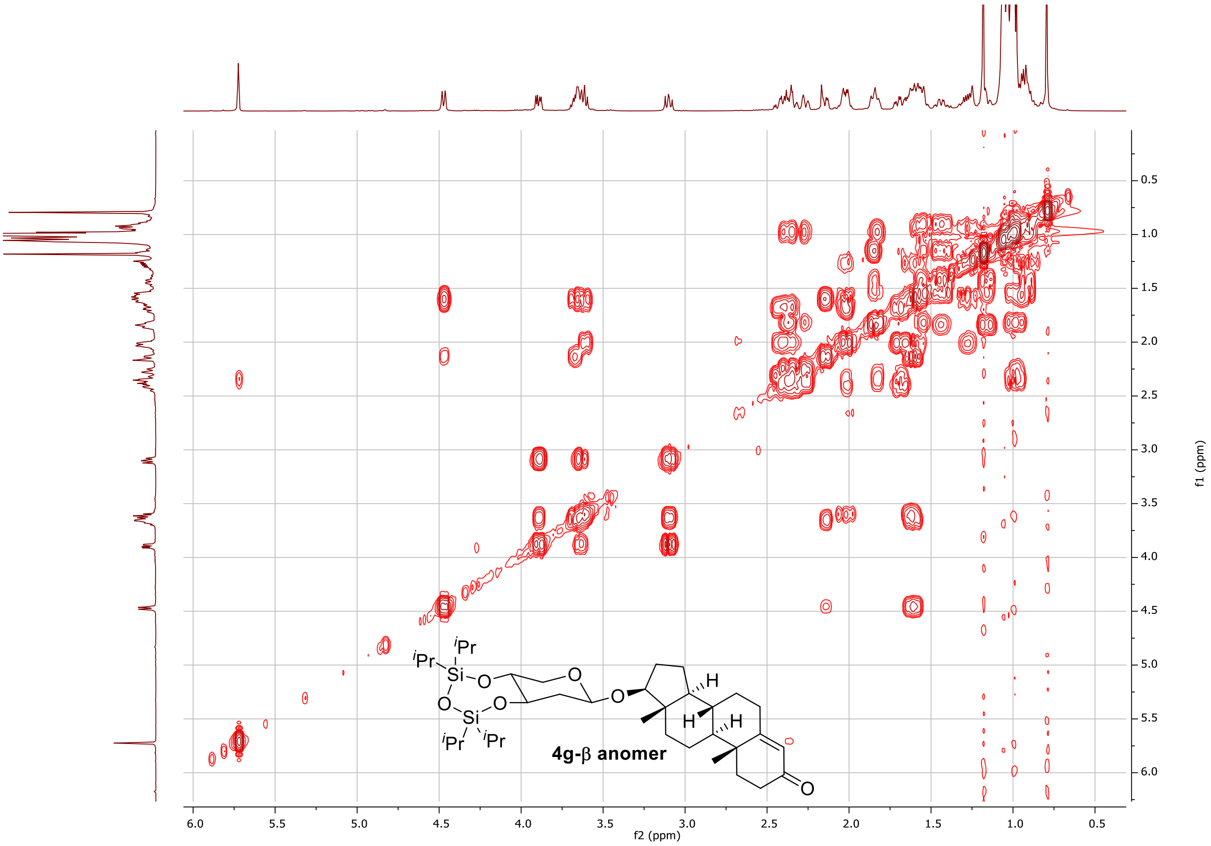

Supplementary figure S460: COSY spectra for **4g-β anomer**

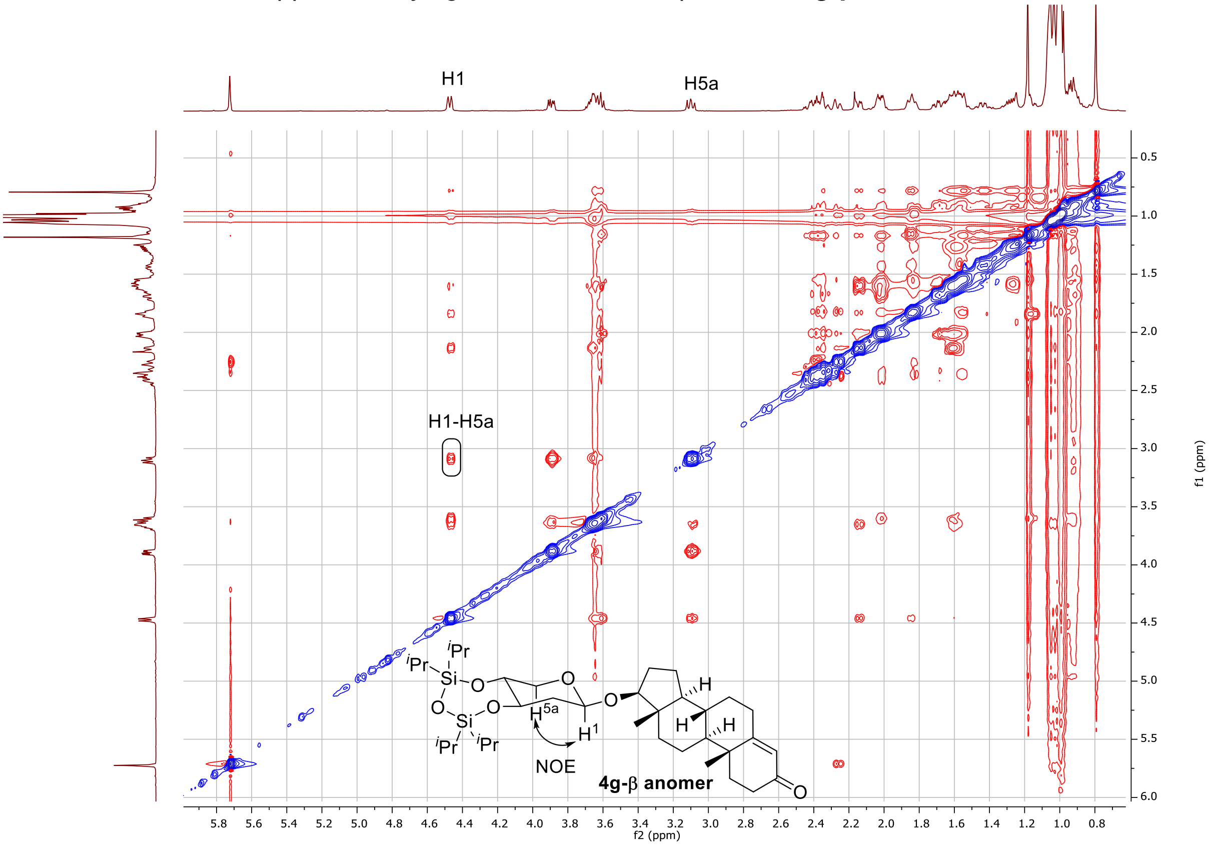

Supplementary figure S461: NOESY spectra for **4g-β anomer**

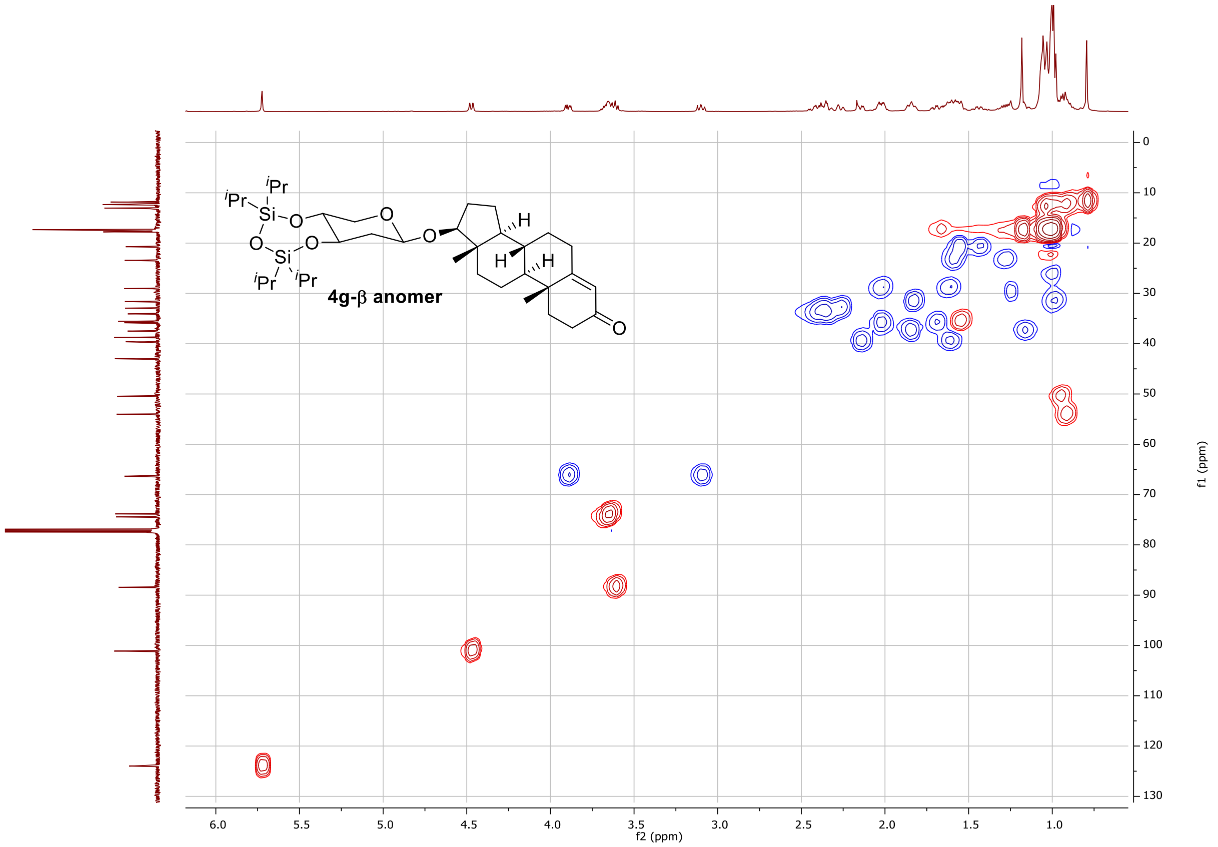

Supplementary figure S462: HSQC spectra for **4g-β anomer**

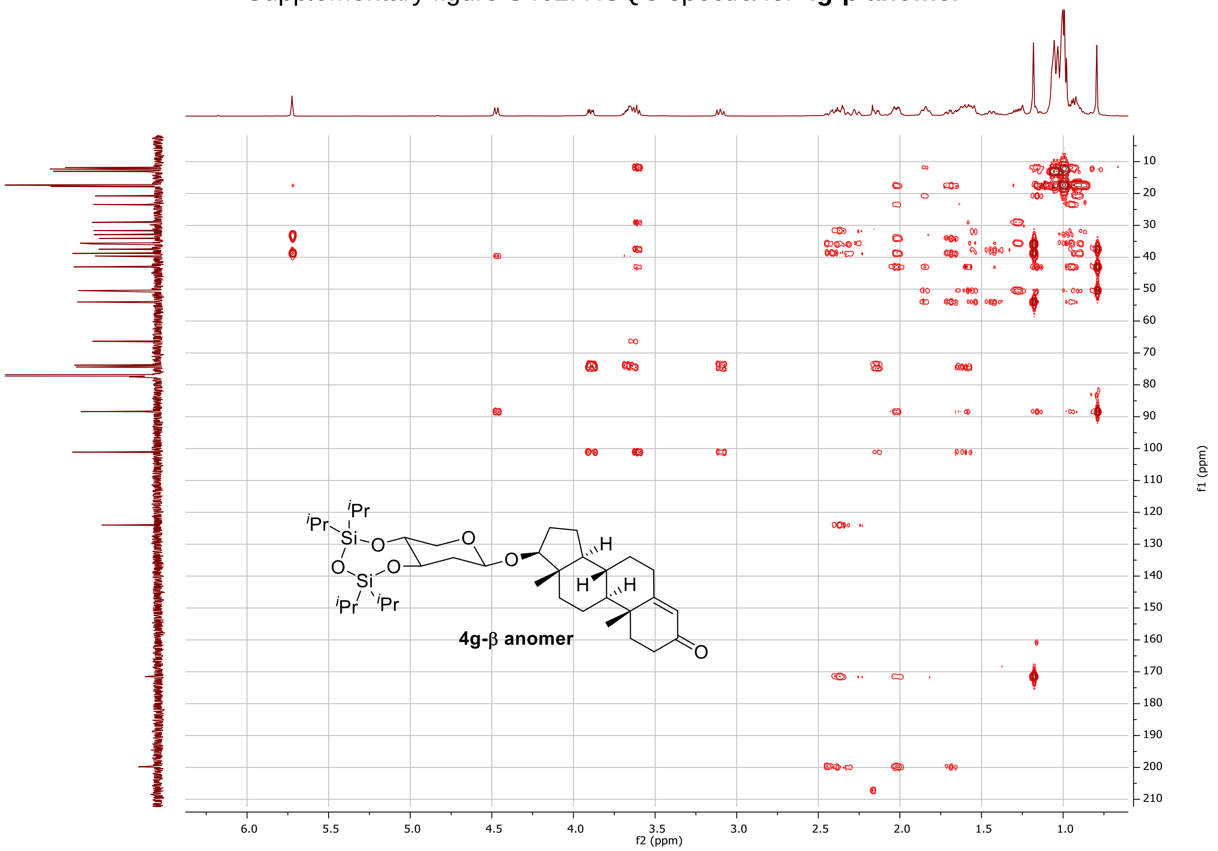

Supplementary figure S463: HMBC spectra for **4g-β anomer**

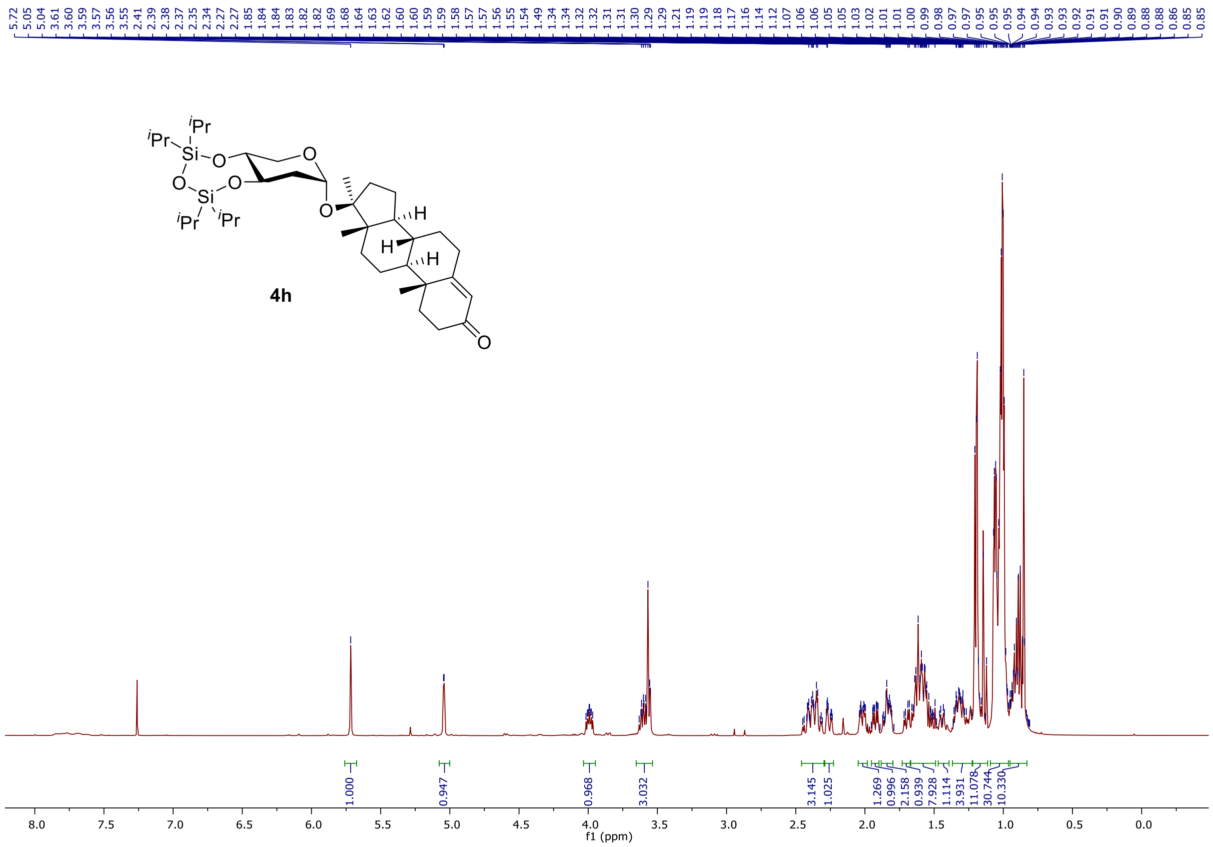

Supplementary figure S464: ^{1}H spectra for **4h**

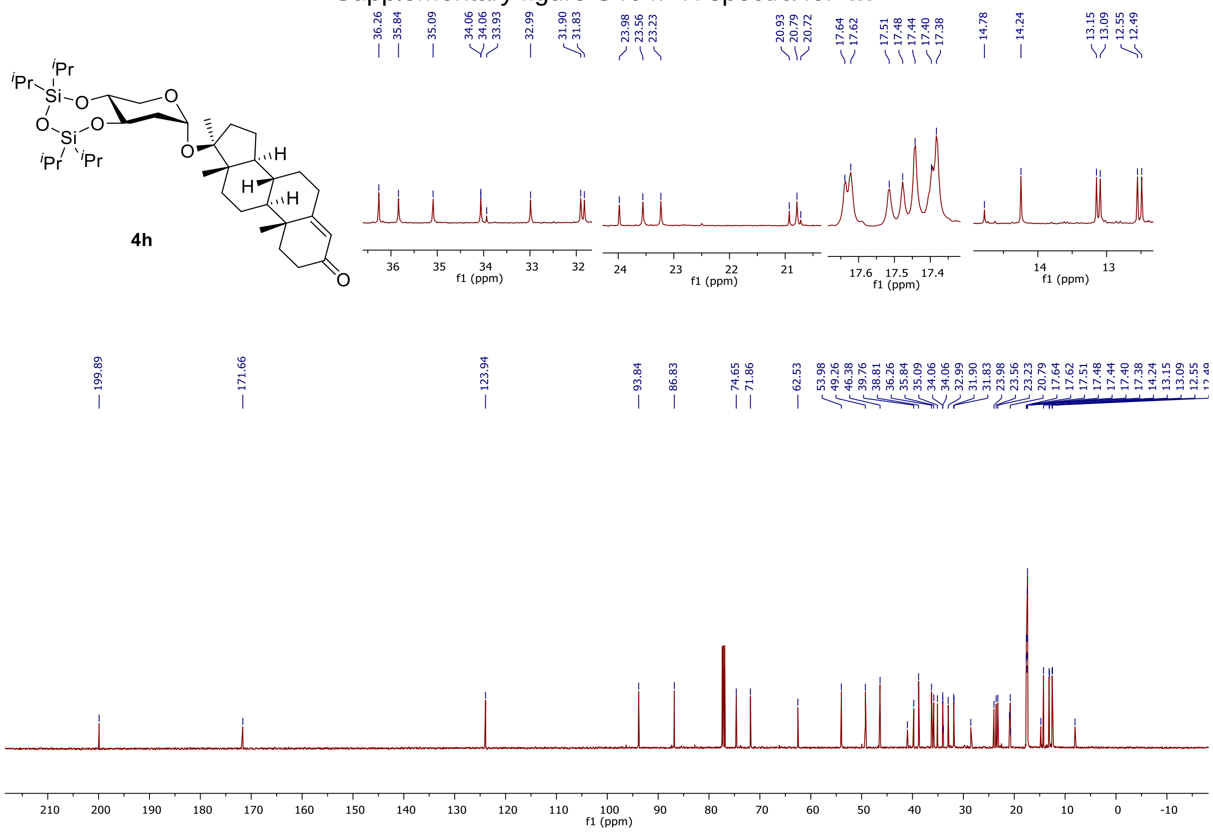

Supplementary figure S465: ^{13}C spectra for **4h**

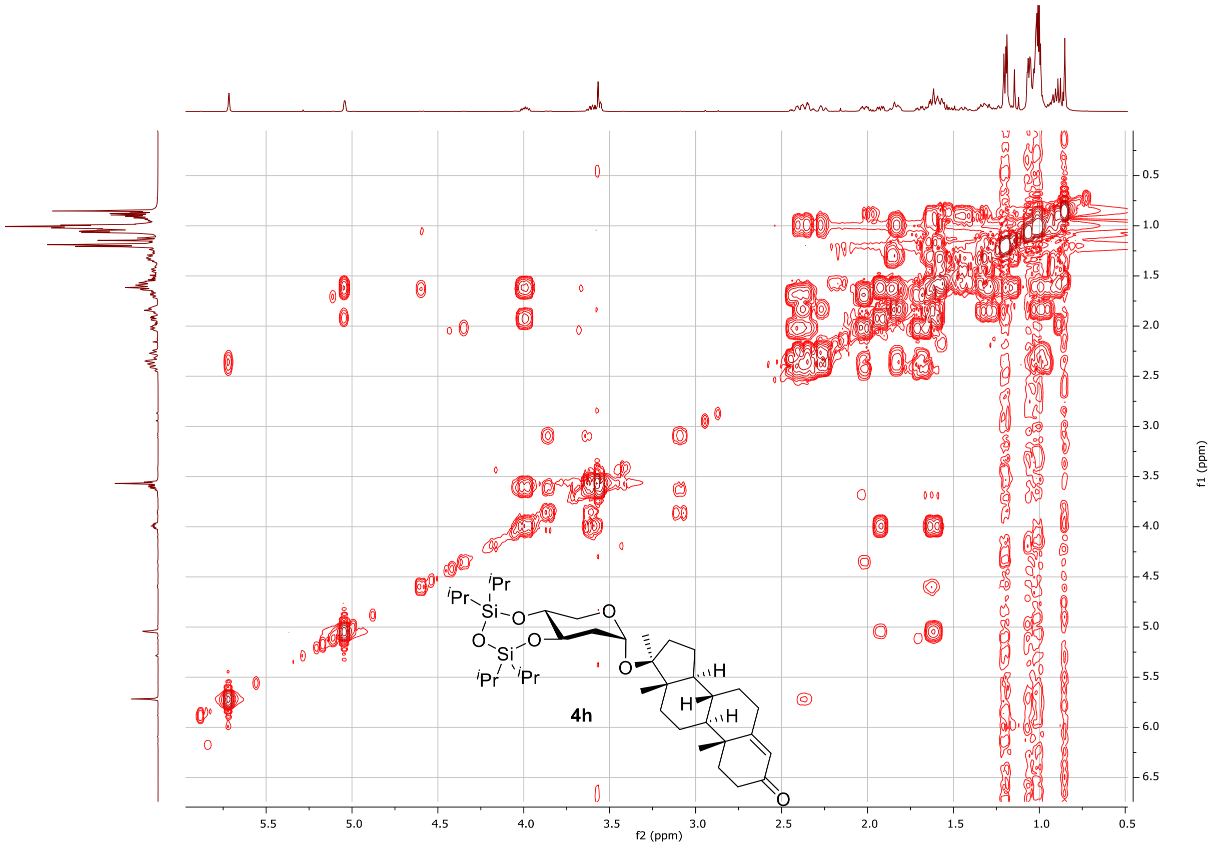

Supplementary figure S466: COSY spectra for **4h**

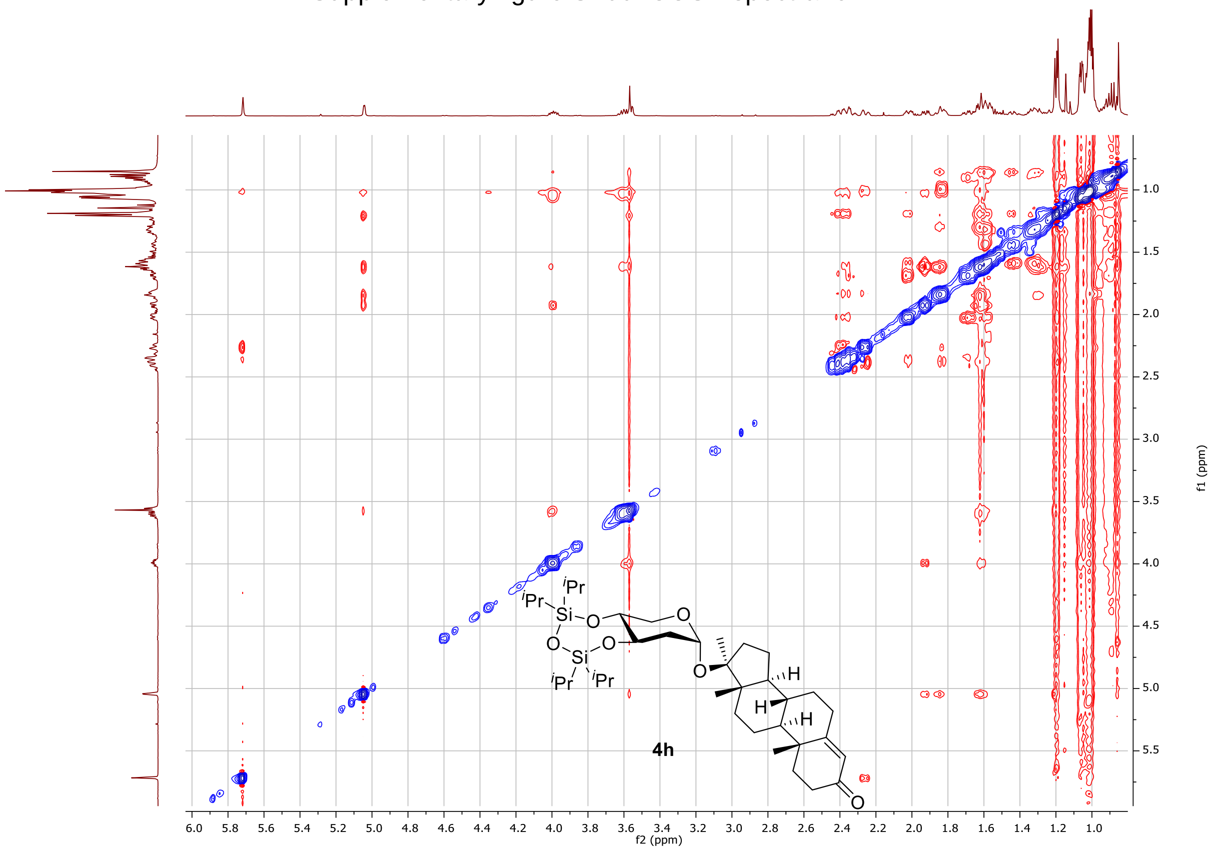

Supplementary figure S467: NOESY spectra for **4h**

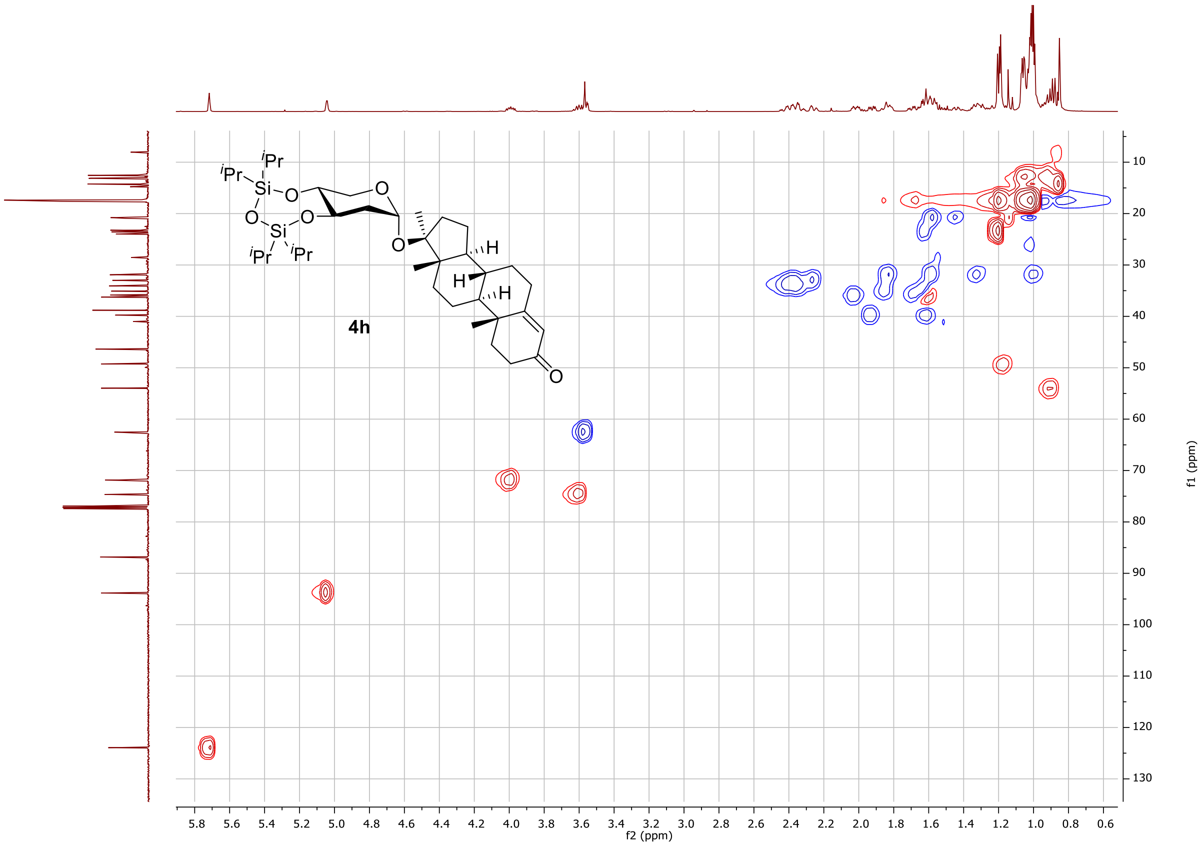

Supplementary figure S468: HSQC spectra for **4h**

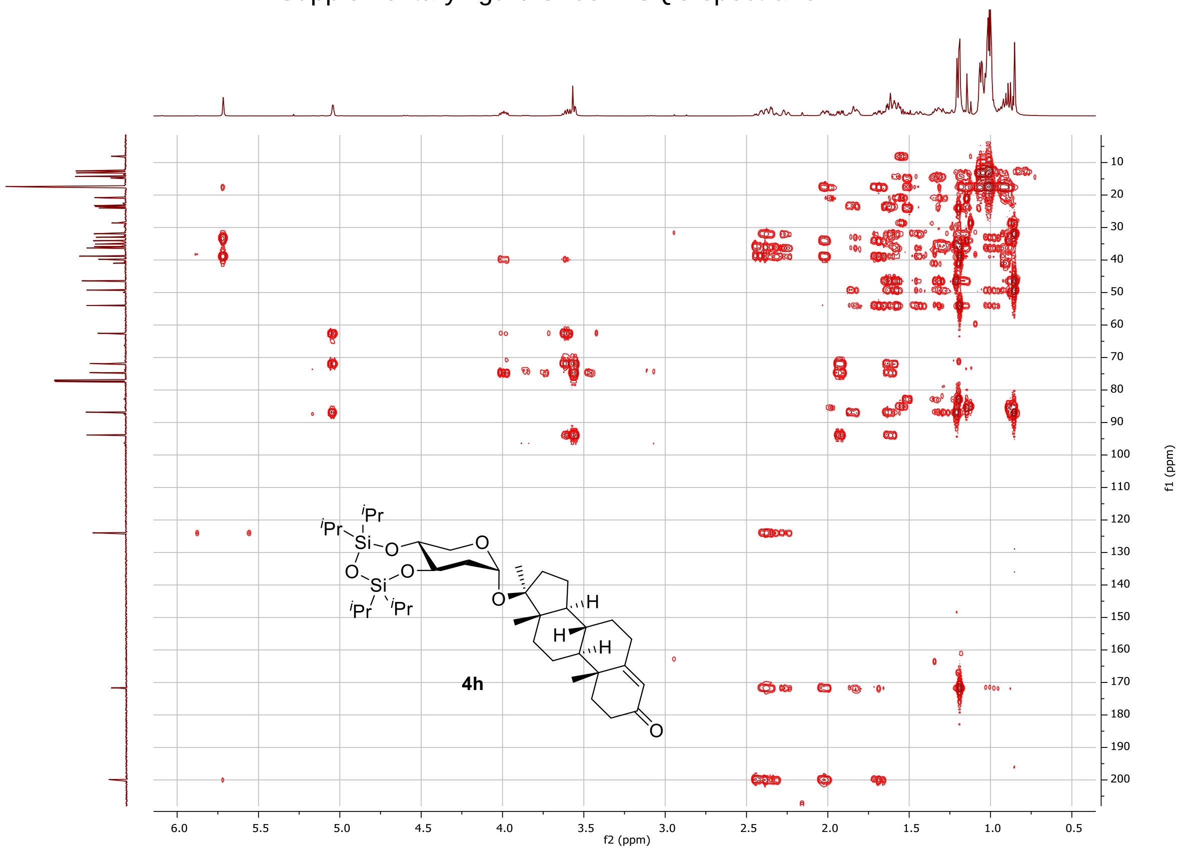

Supplementary figure S469: HMBC spectra for **4h**

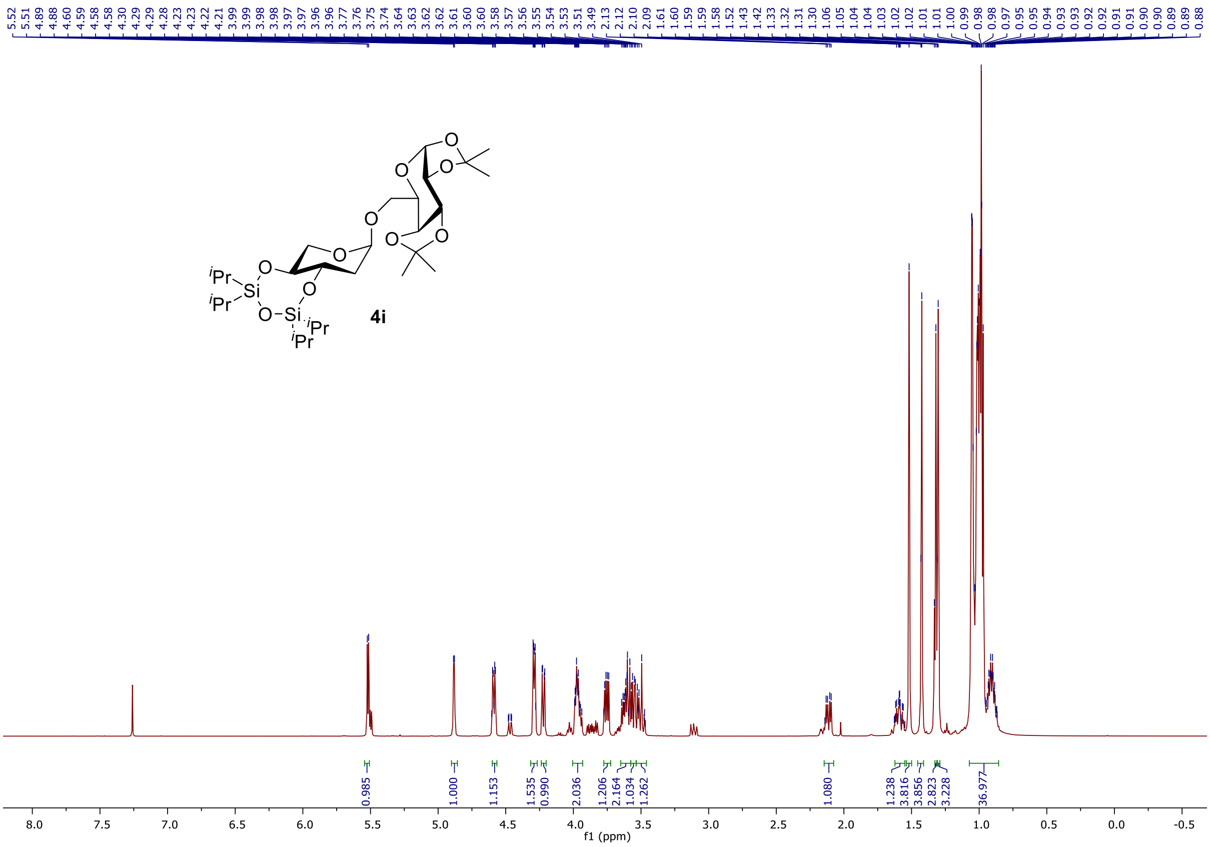

Supplementary figure S470: ^{1}H spectra for **4i**

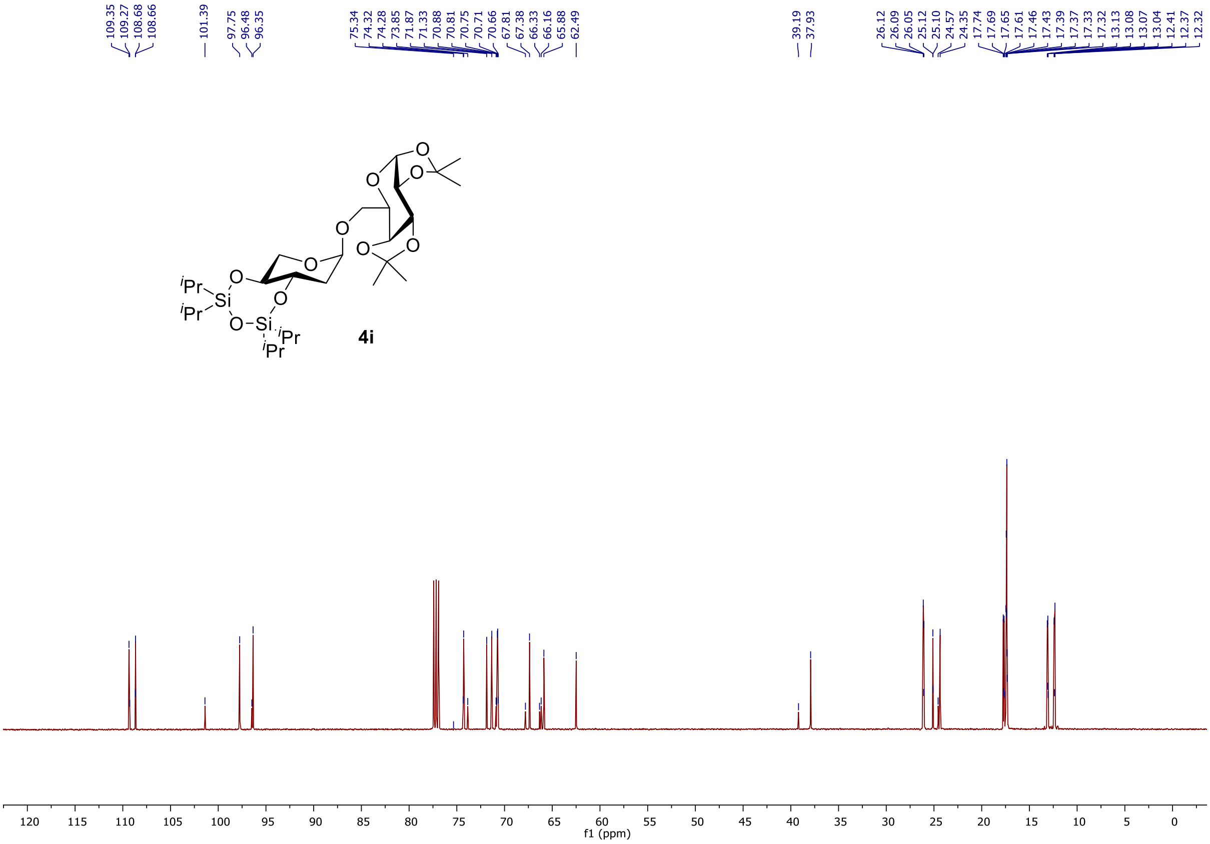

Supplementary figure S471: ^{13}C spectra for **4i**

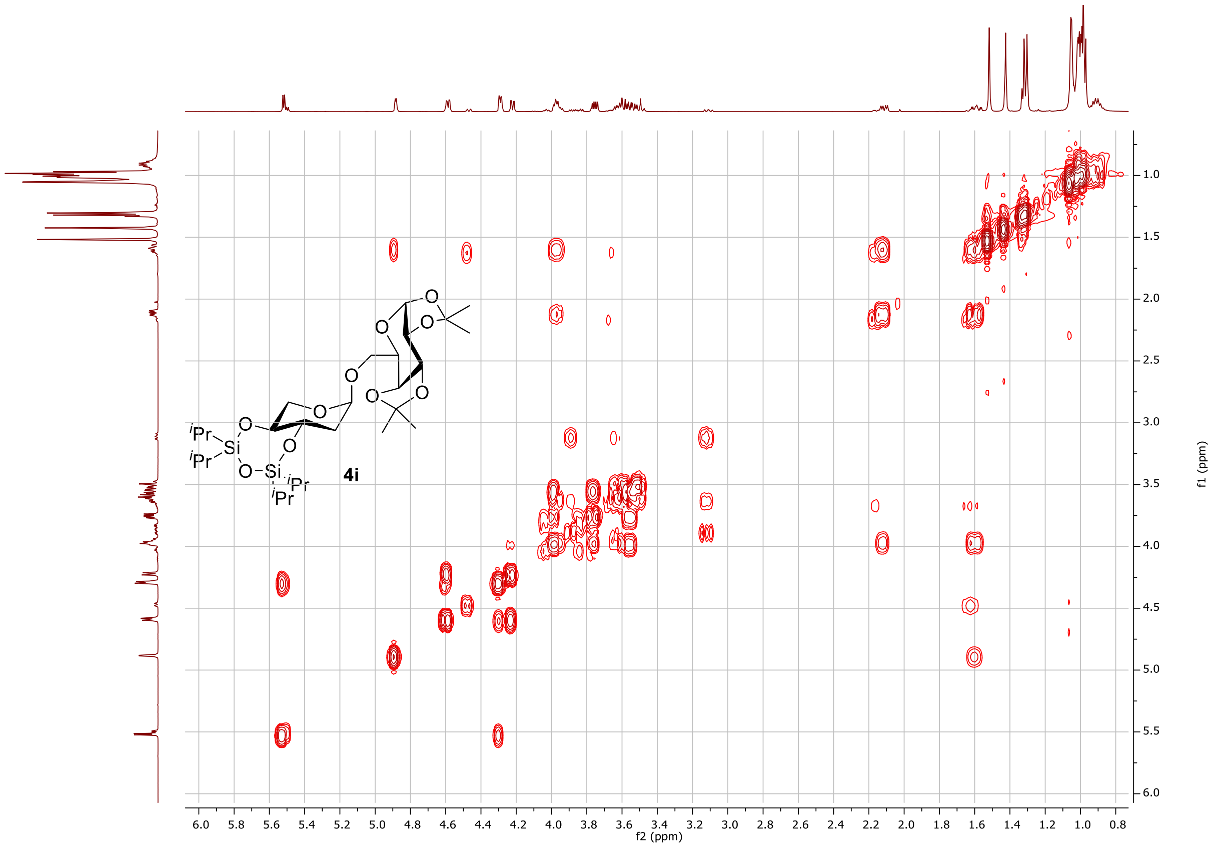

Supplementary figure S472: COSY spectra for **4i**

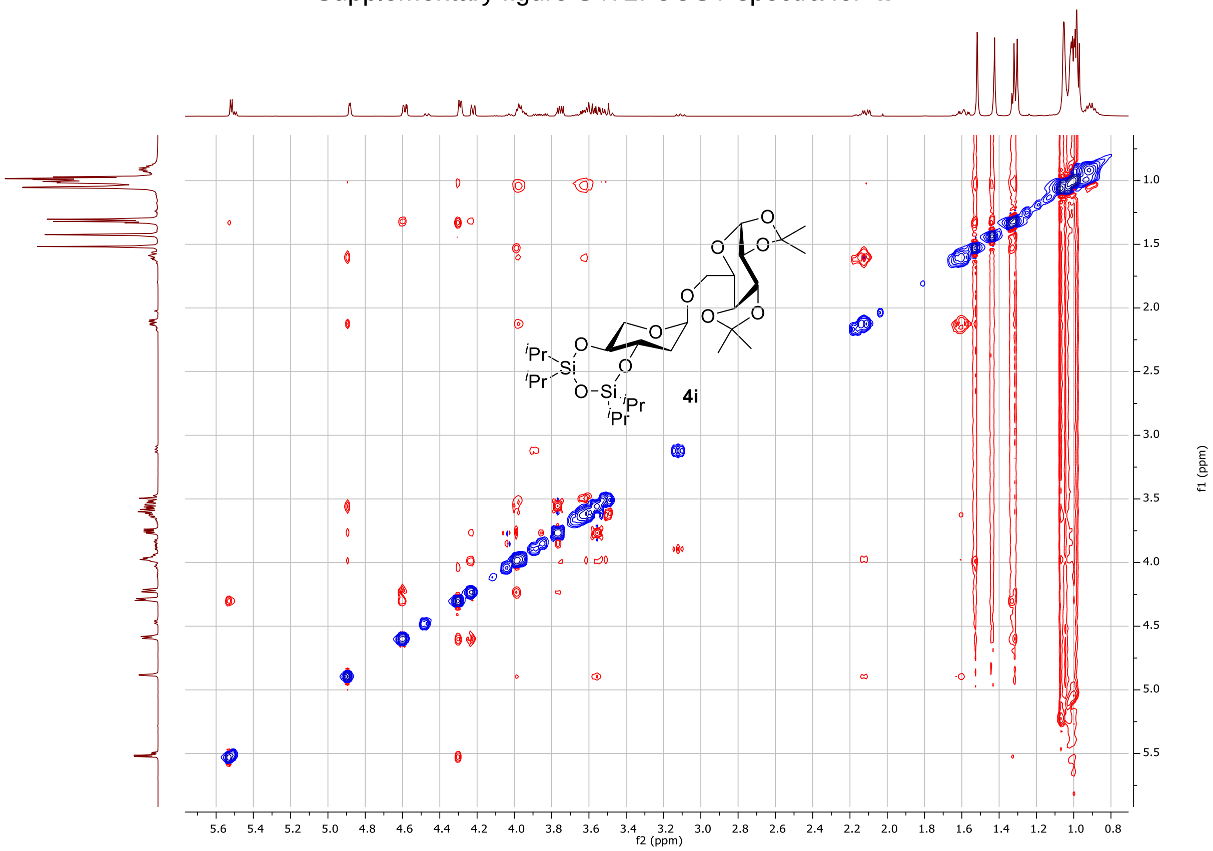

Supplementary figure S473: NOESY spectra for **4i**

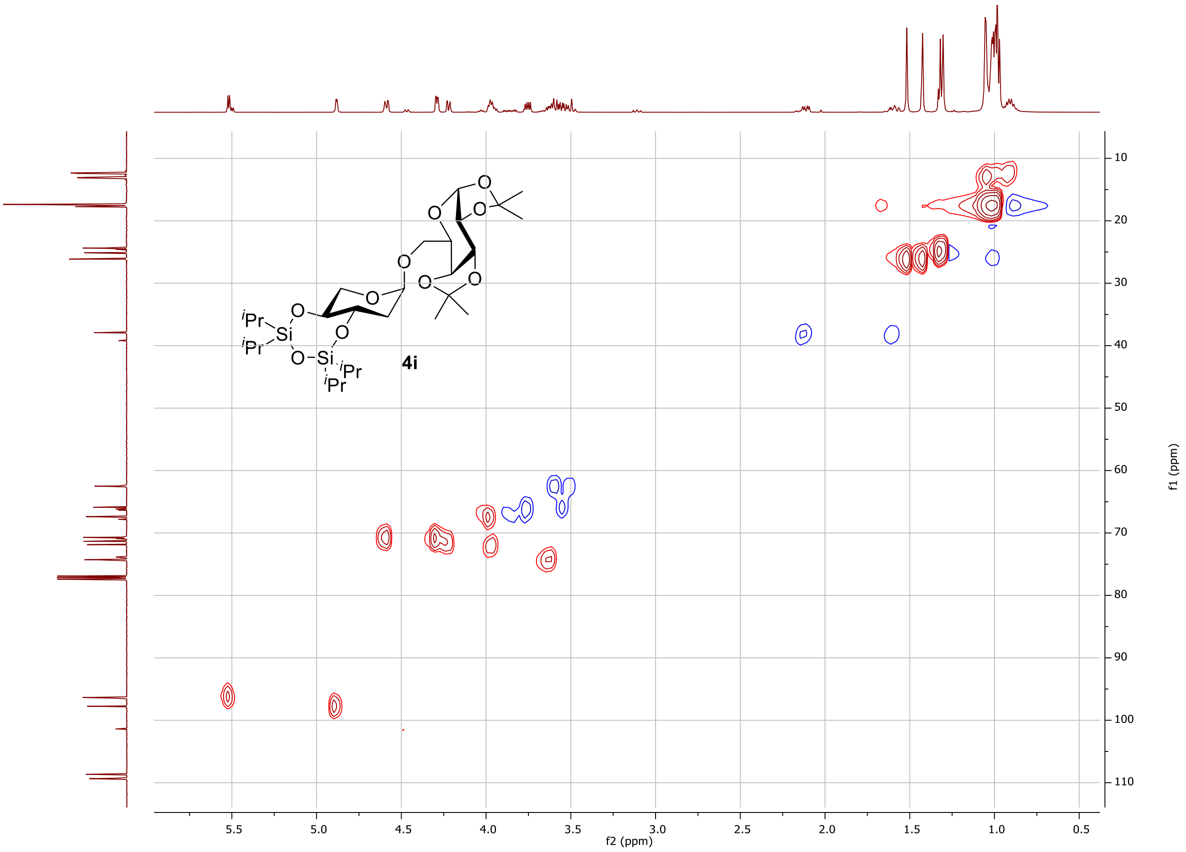

Supplementary figure S474: HSQC spectra for **4i**

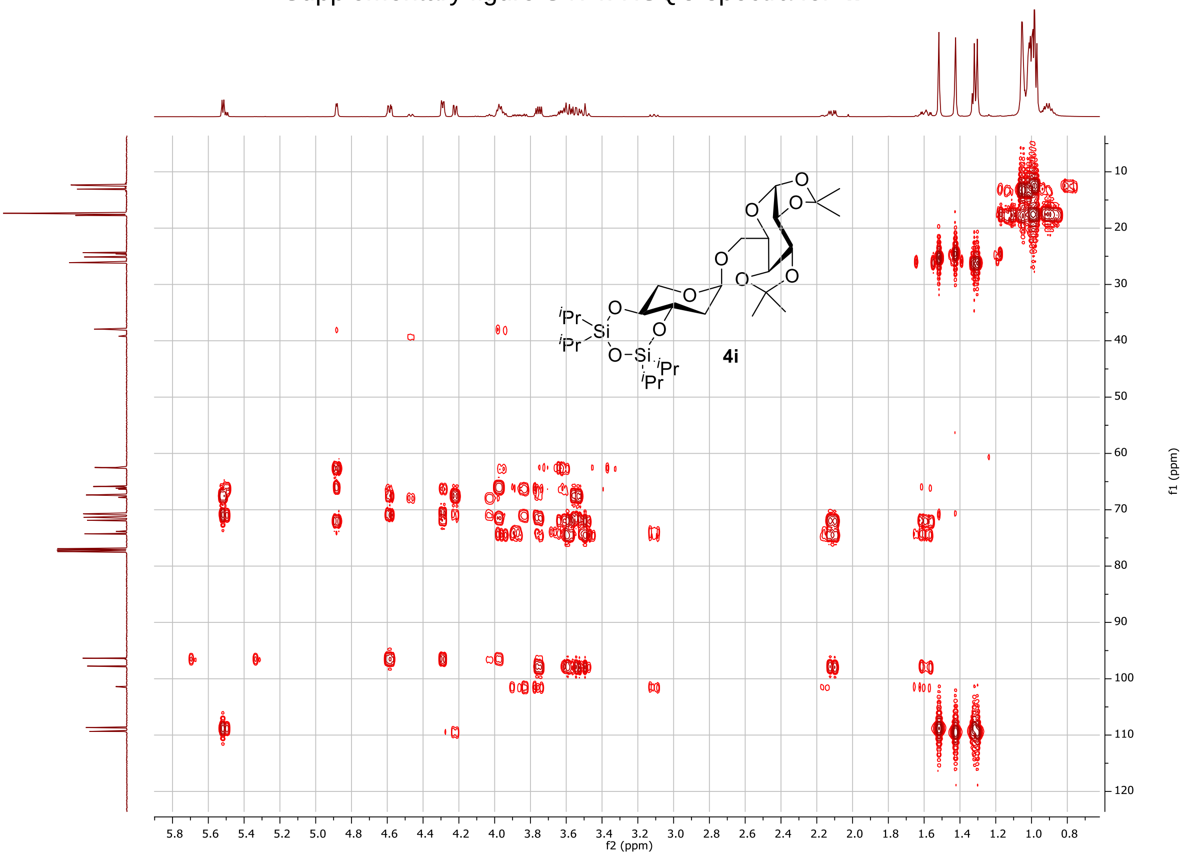

Supplementary figure S475: HMBC spectra for **4i**

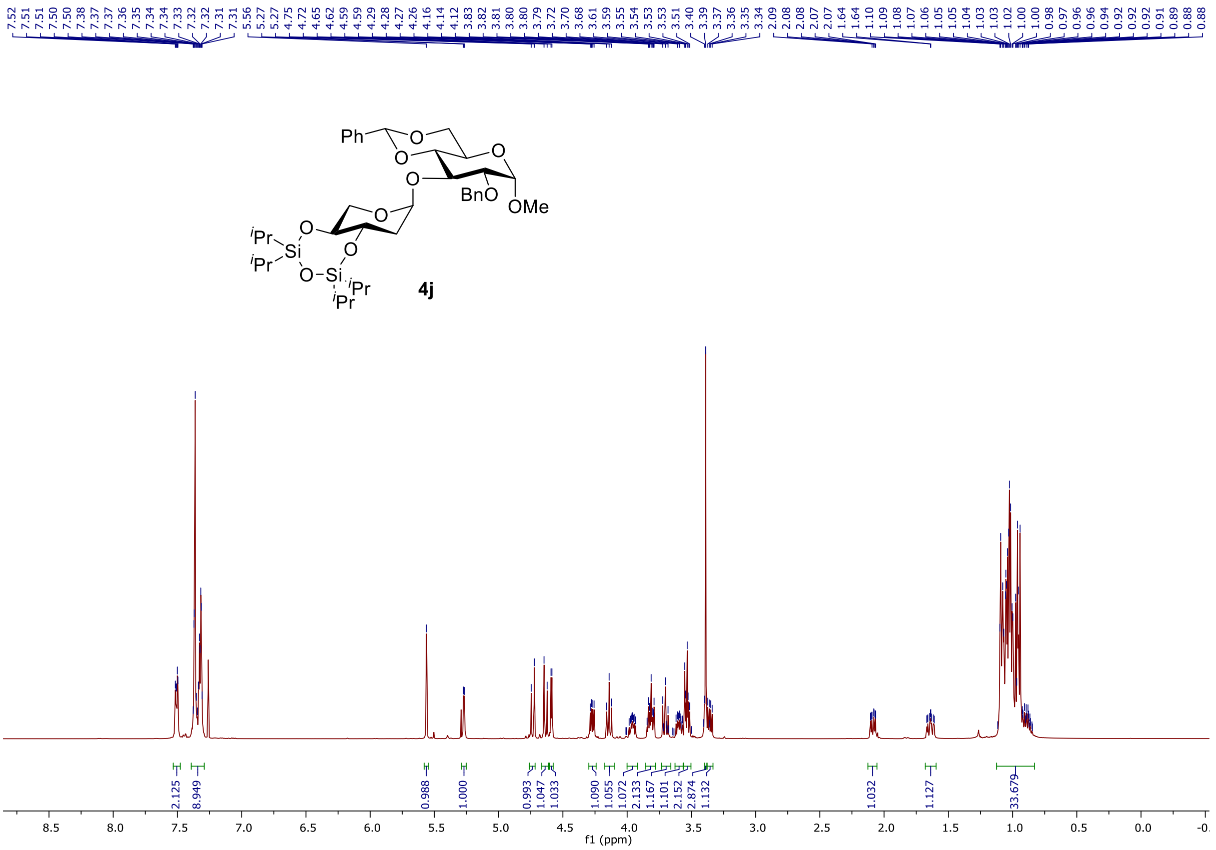

Supplementary figure S476: ^{1}H spectra for **4j**

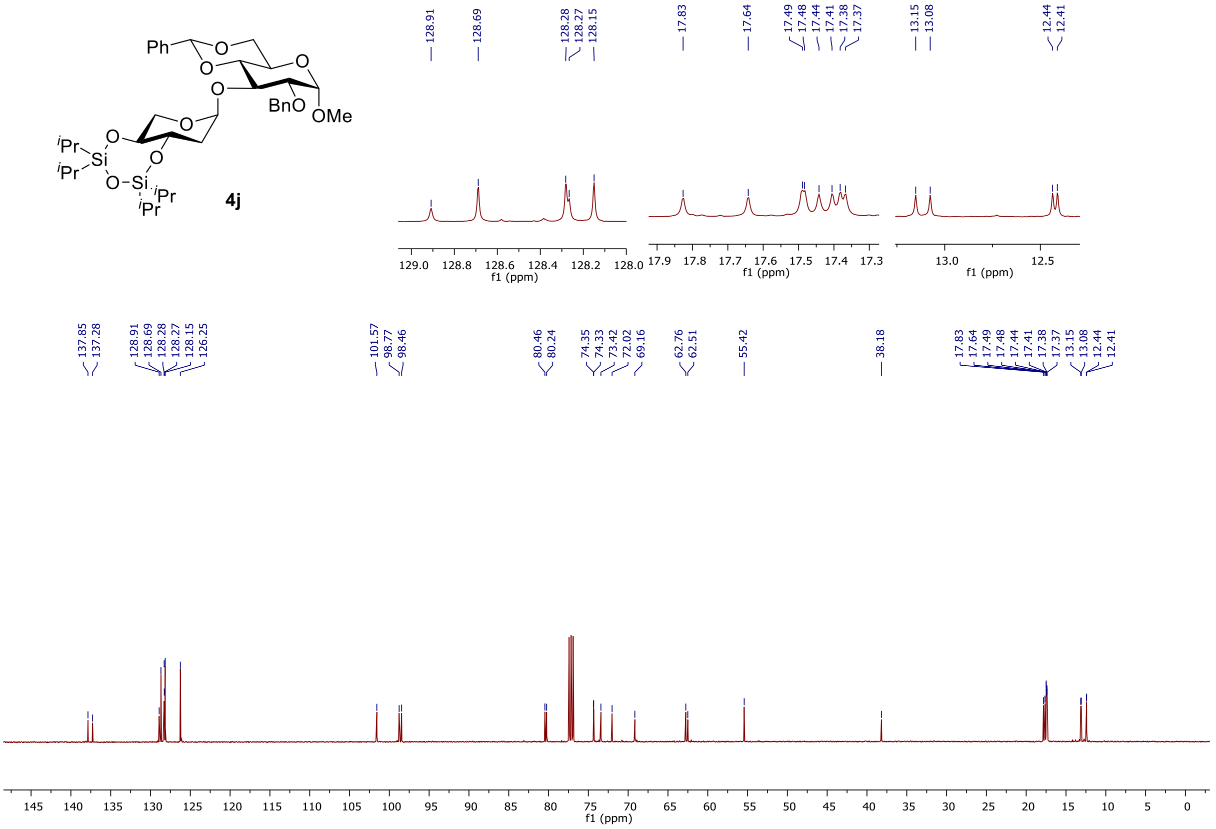

Supplementary figure S477: ^{13}C spectra for **4j**

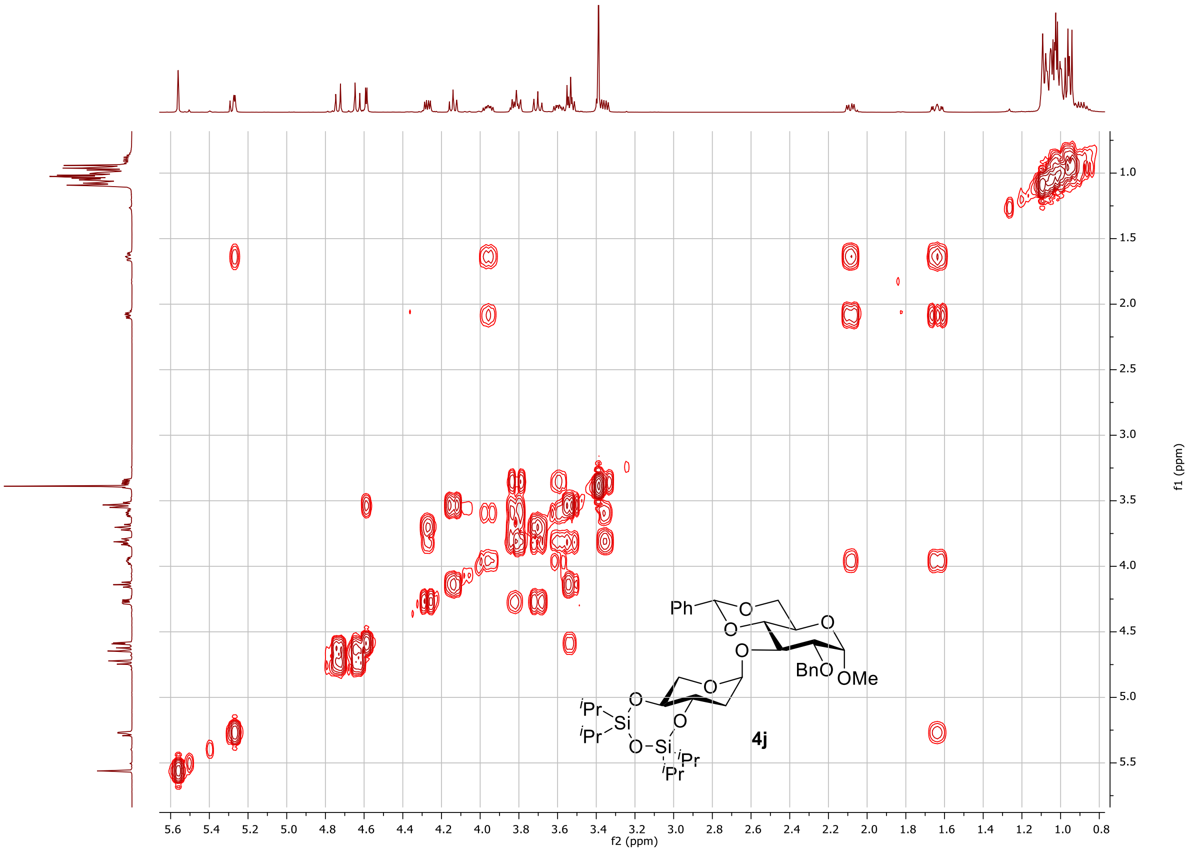

Supplementary figure S478: COSY spectra for **4j**

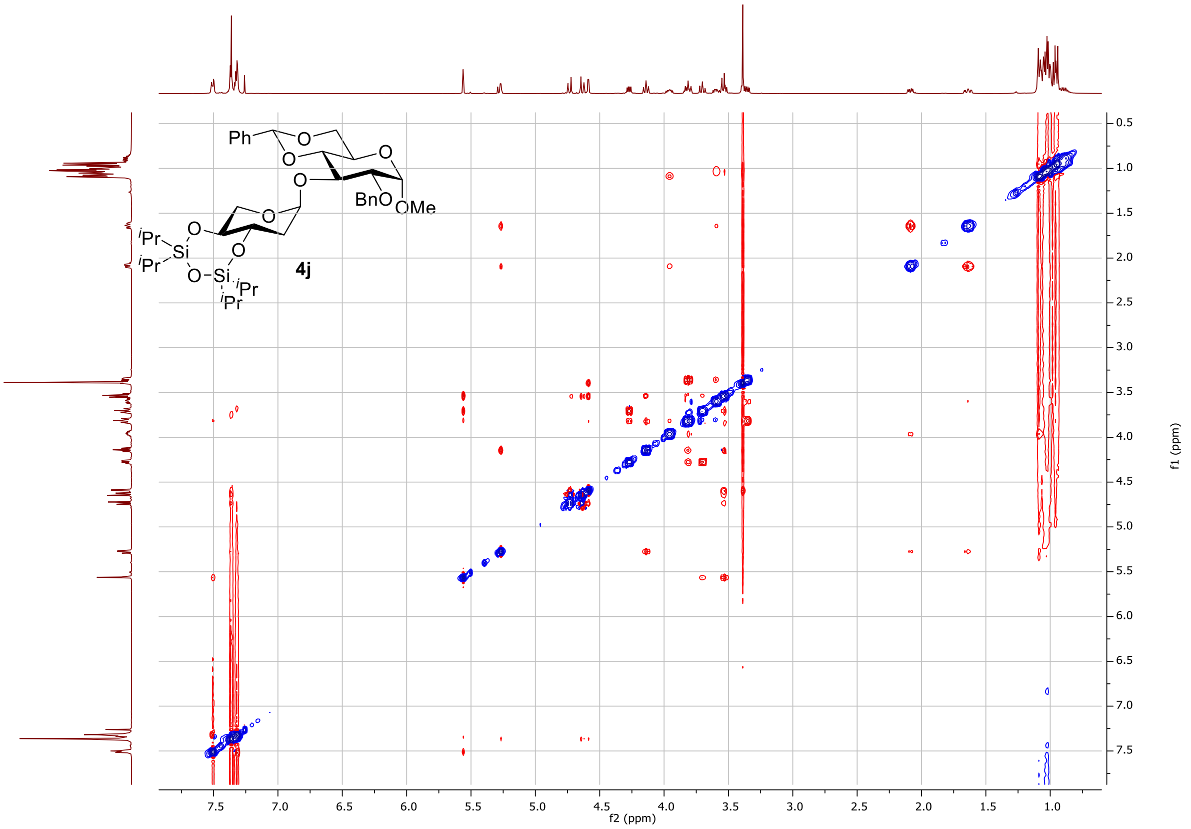

Supplementary figure S479: NOESY spectra for **4j**

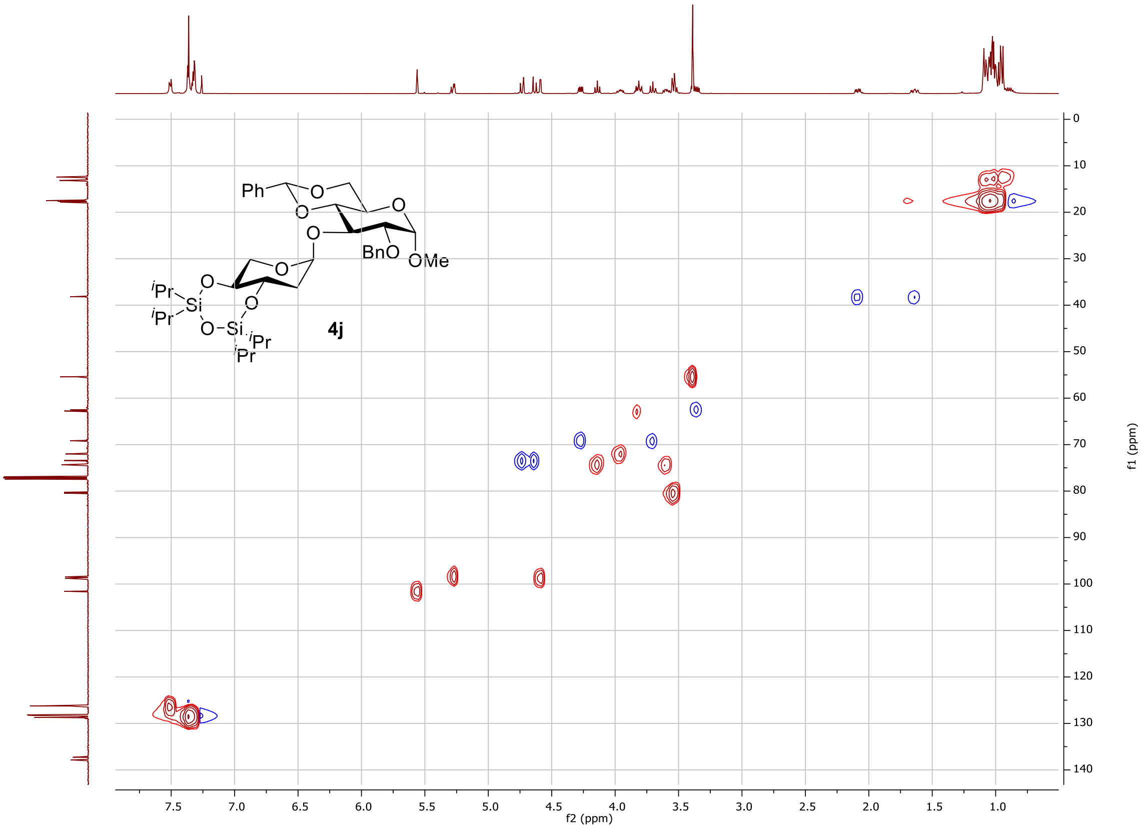

Supplementary figure S480: HSQC spectra for **4j**

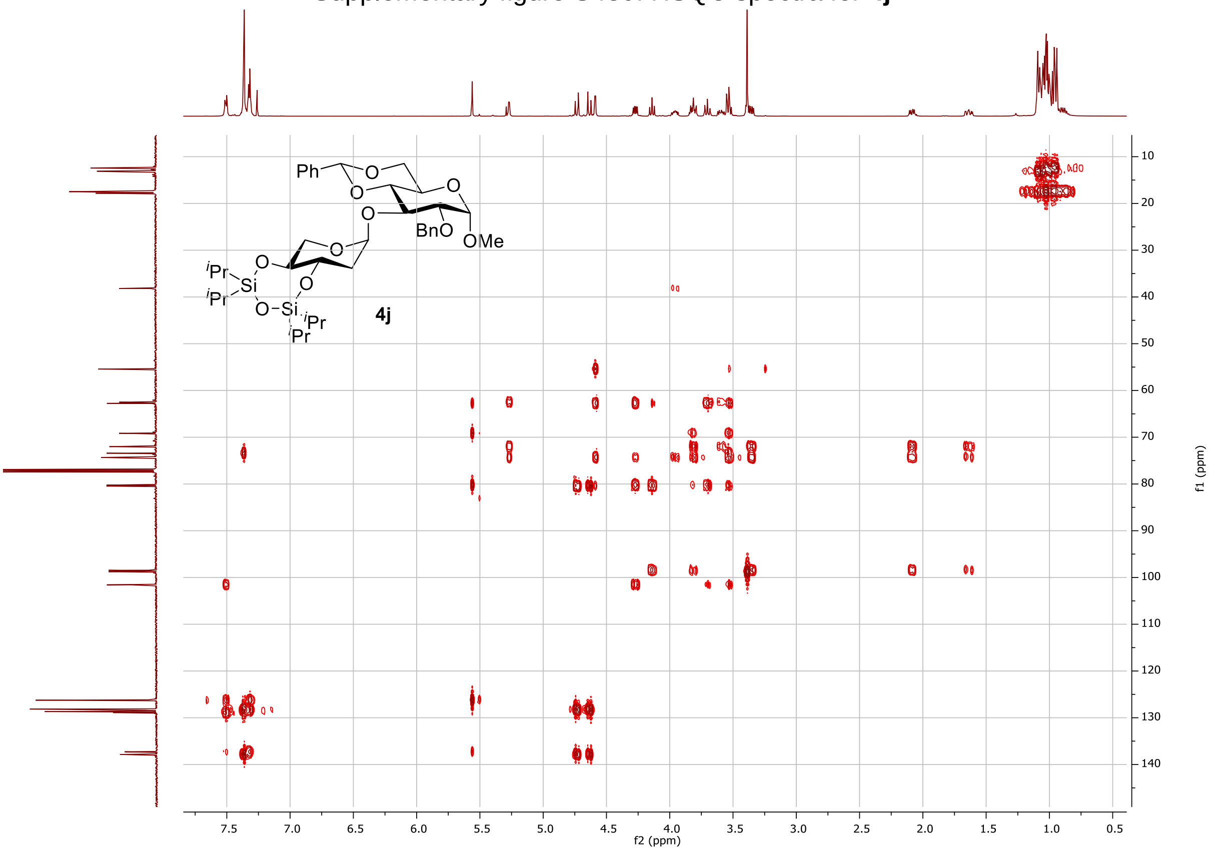

Supplementary figure S481: HMBC spectra for **4j**

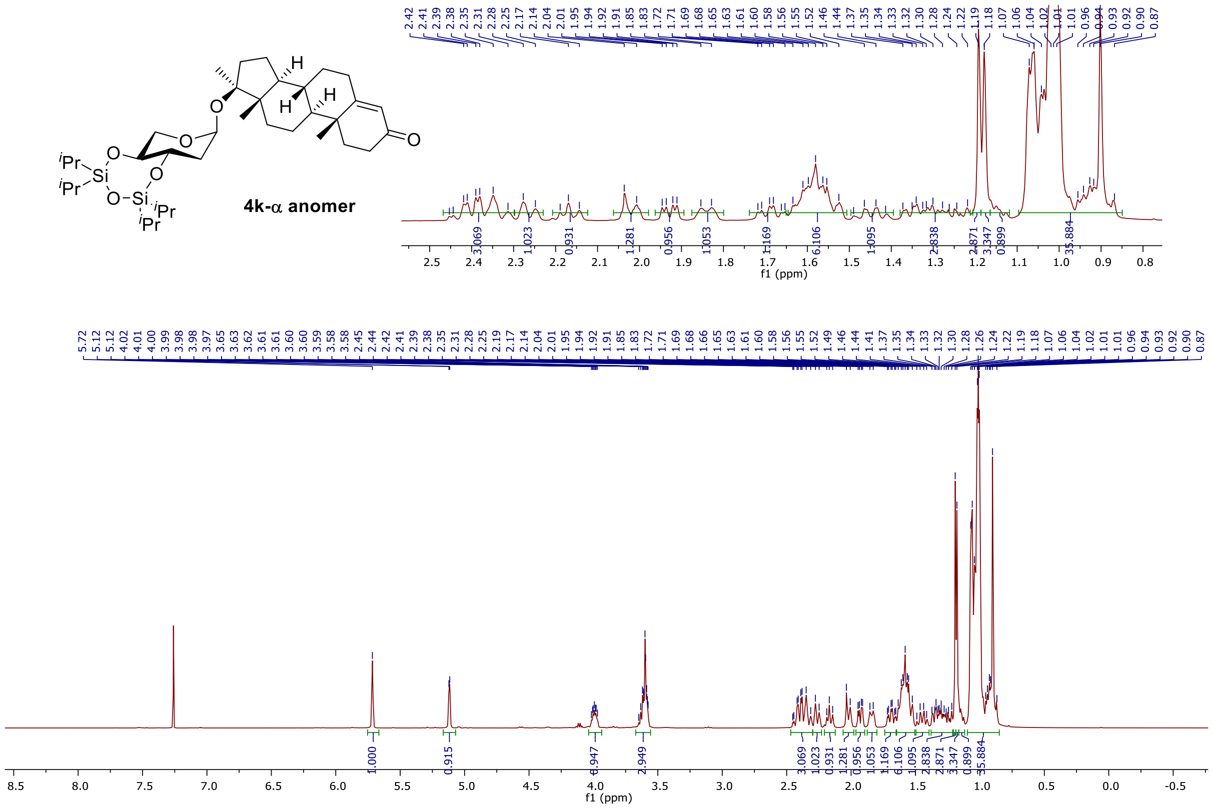

Supplementary figure S482: ^{1}H spectra for **4k-α anomer**

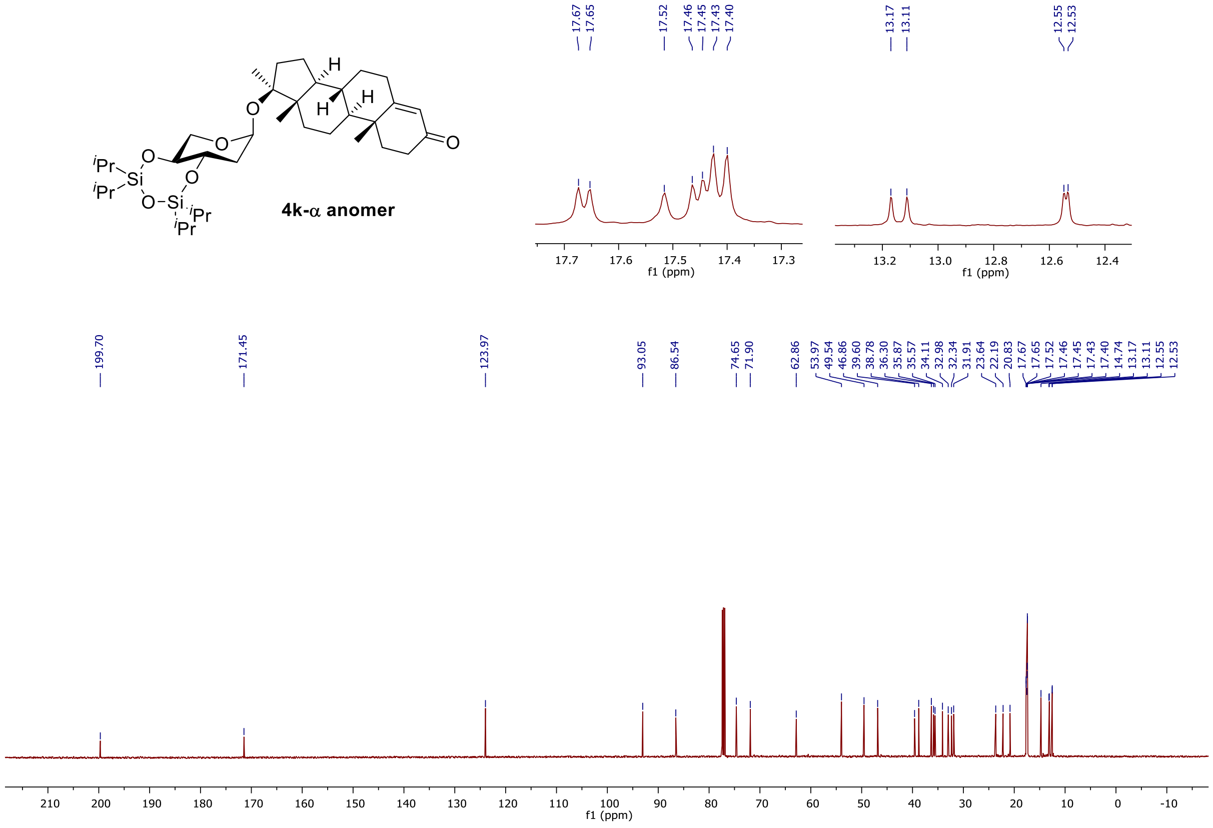

Supplementary figure S483: ^{13}C spectra for **4k-α anomer**

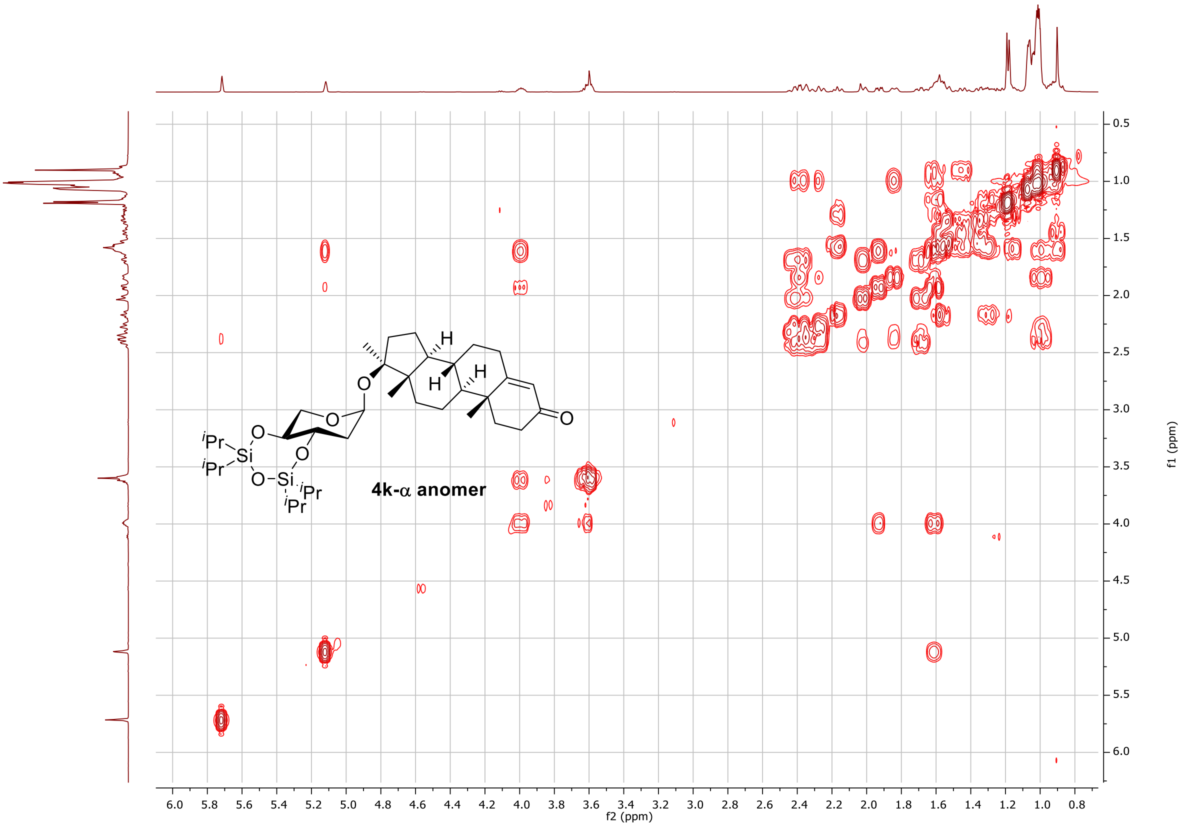

Supplementary figure S484: COSY spectra for **4k-α anomer**

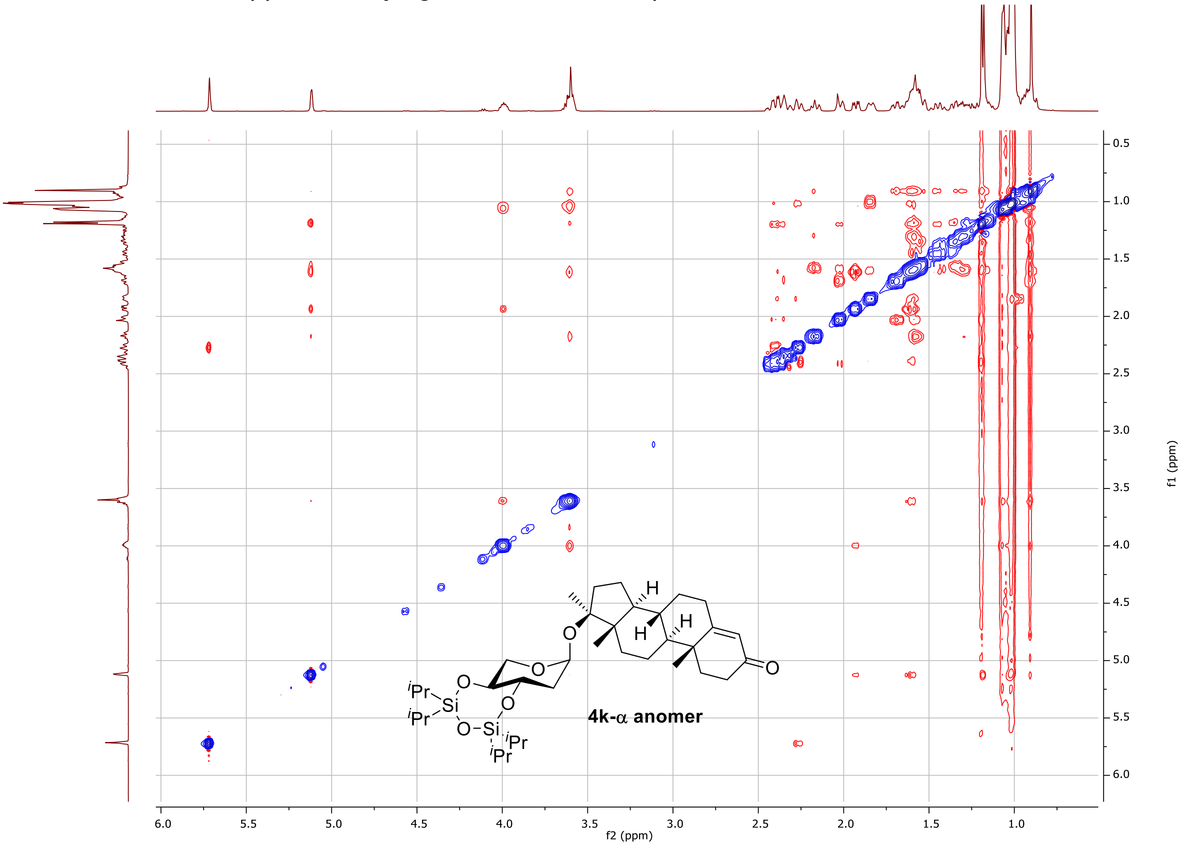

Supplementary figure S485: NOESY spectra for **4k-α anomer**

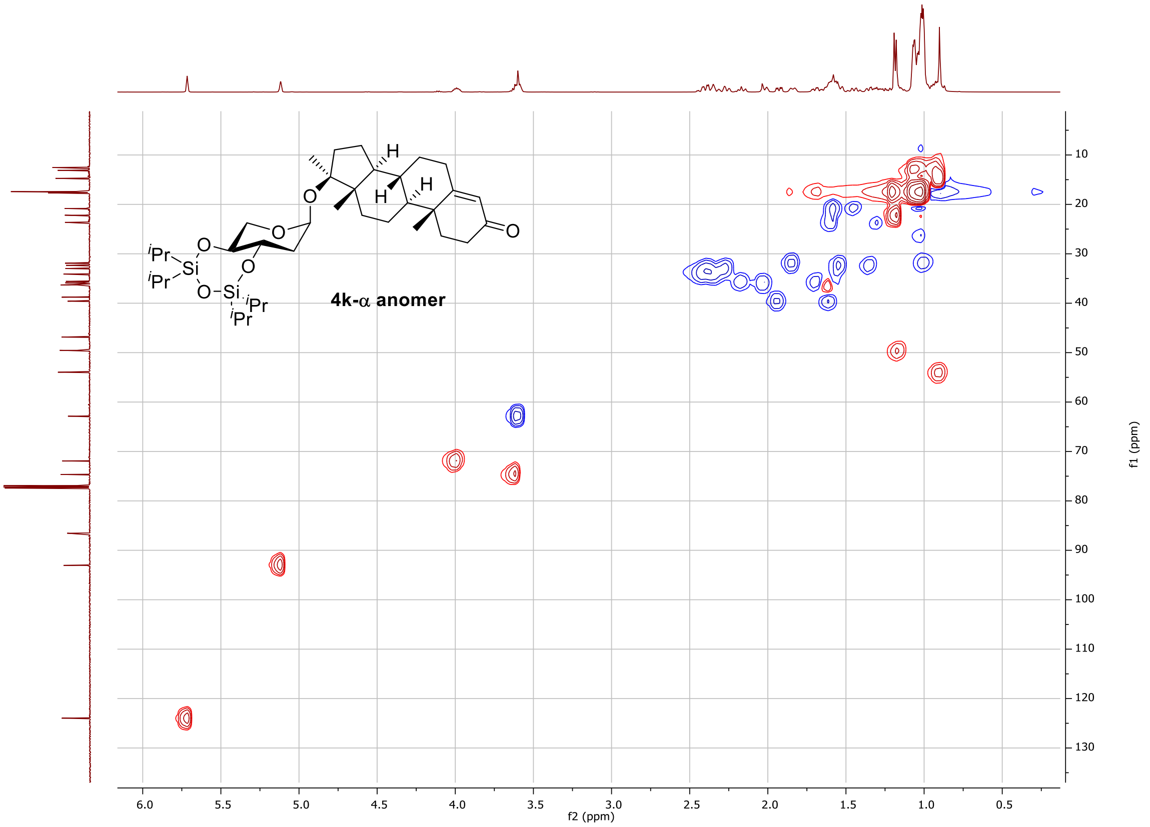

Supplementary figure S486: HSQC spectra for **4k-α anomer**

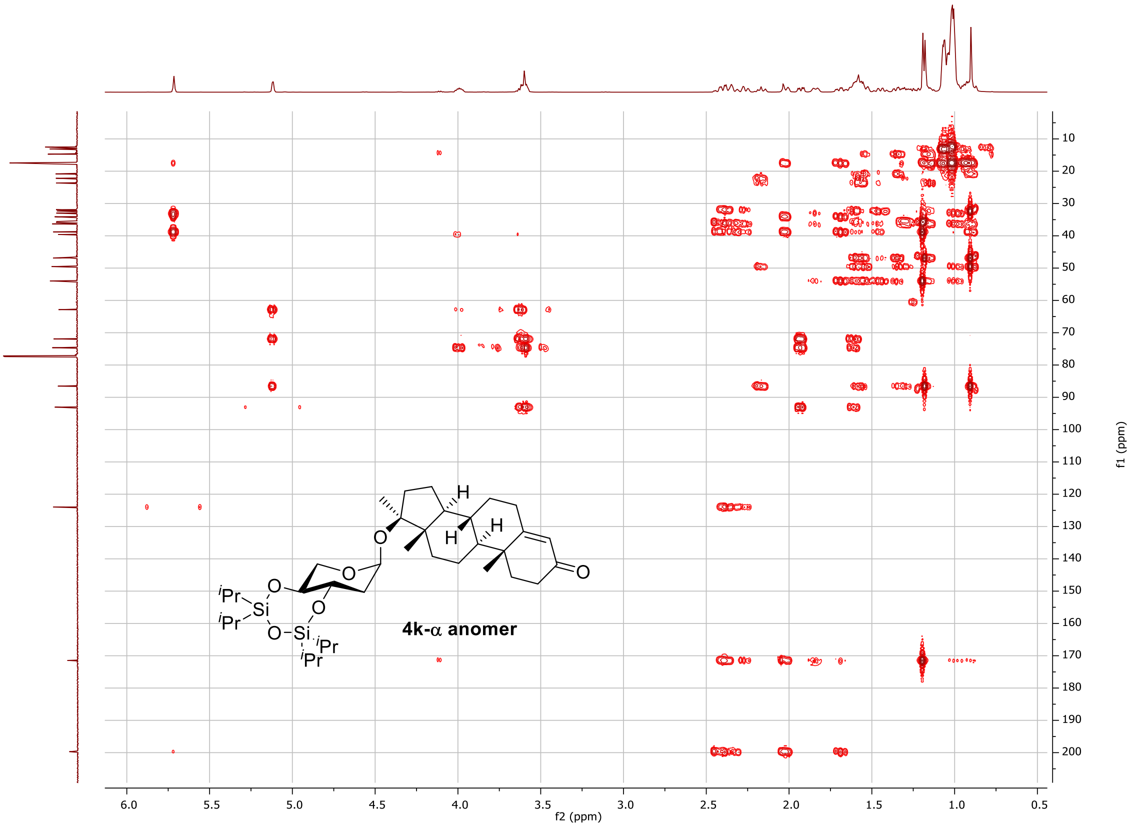

Supplementary figure S487: HMBC spectra for **4k-α anomer**

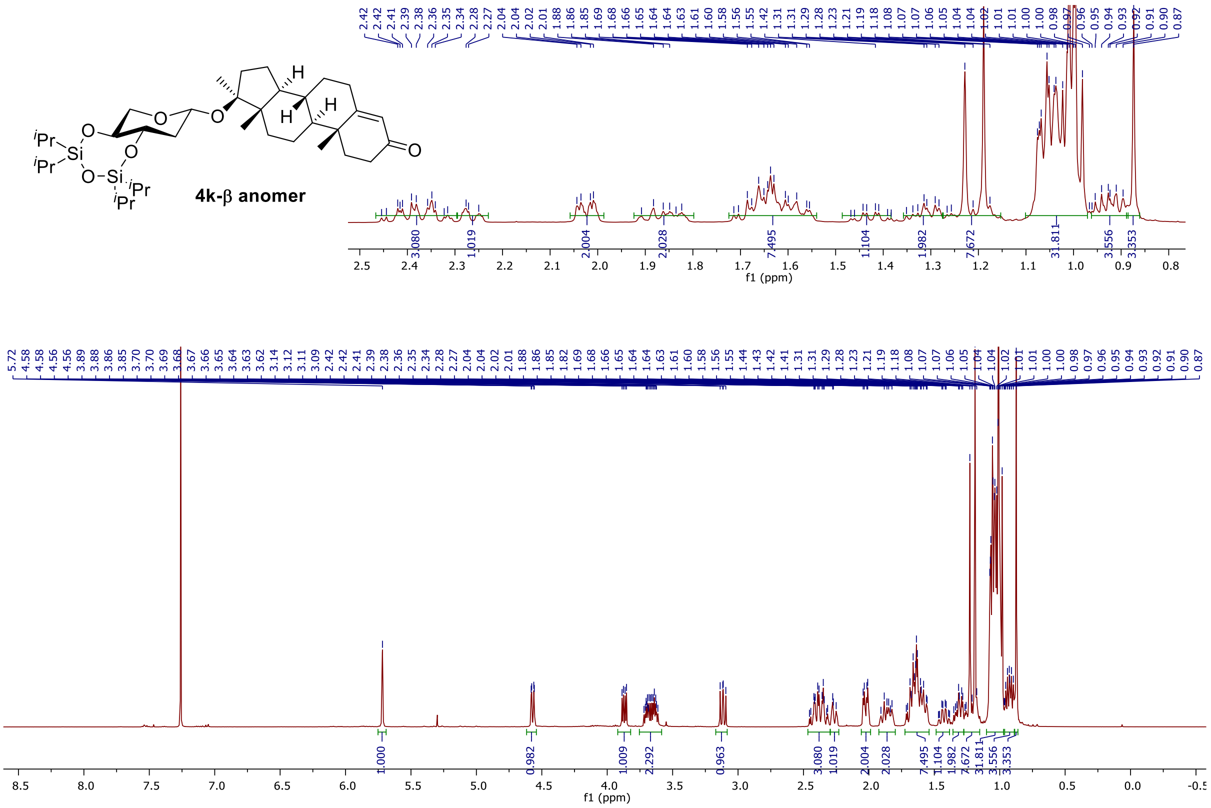

Supplementary figure S488: ^{1}H spectra for **4k-β anomer**

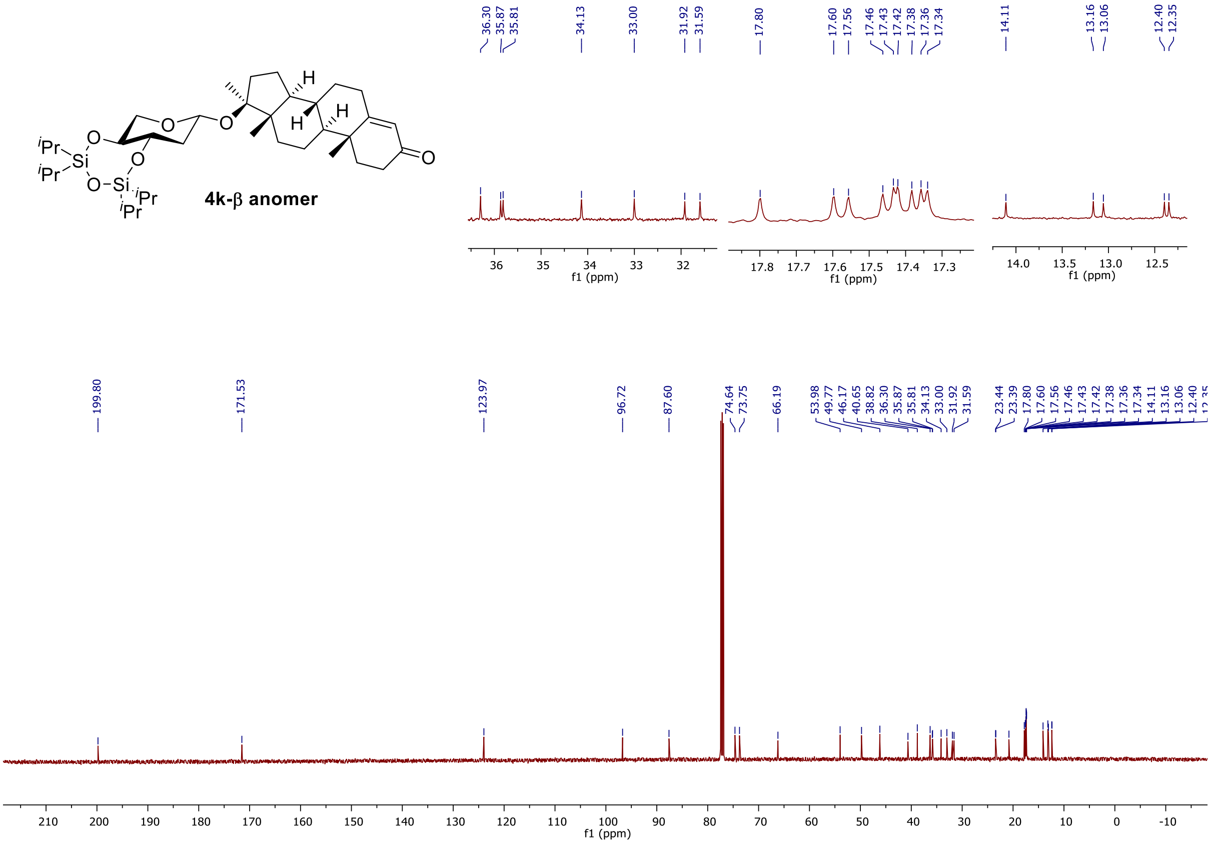

Supplementary figure S489: ^{13}C spectra for **4k-β anomer**

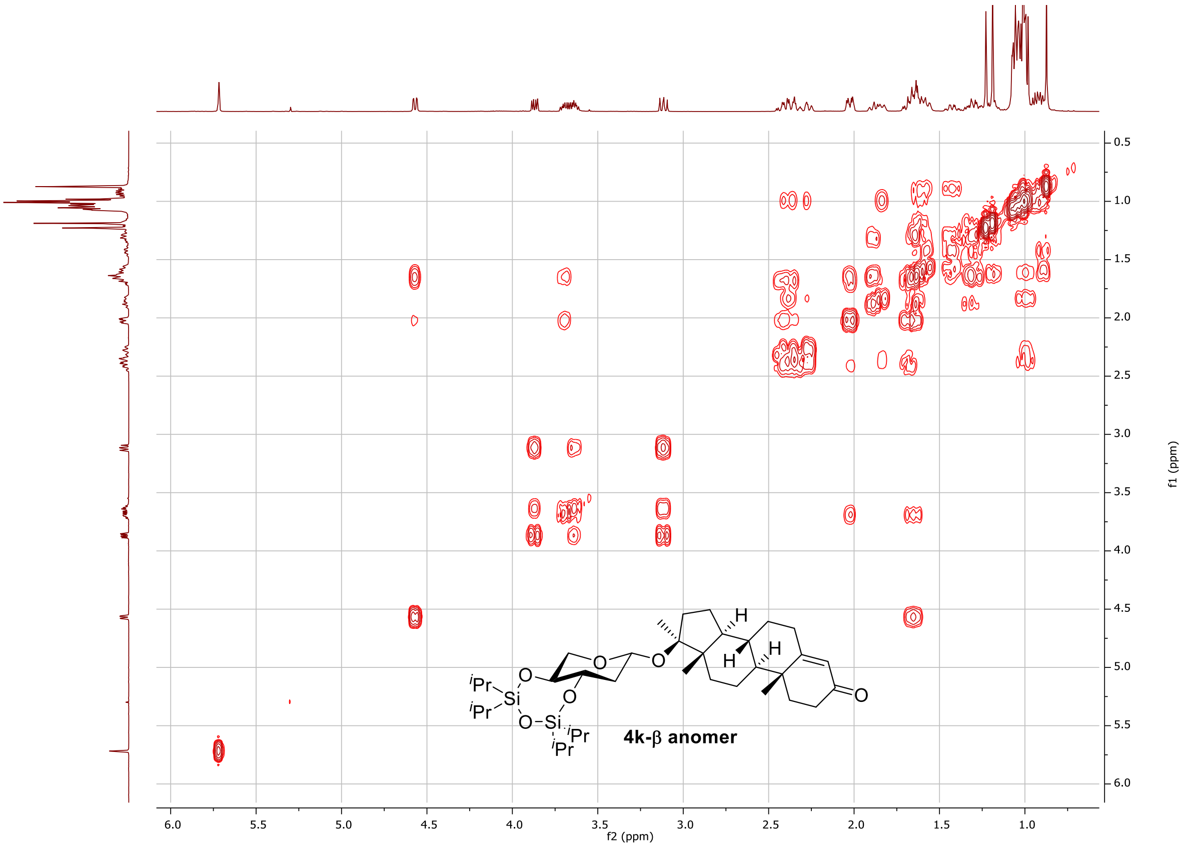

Supplementary figure S490: COSY spectra for **4k-β anomer**

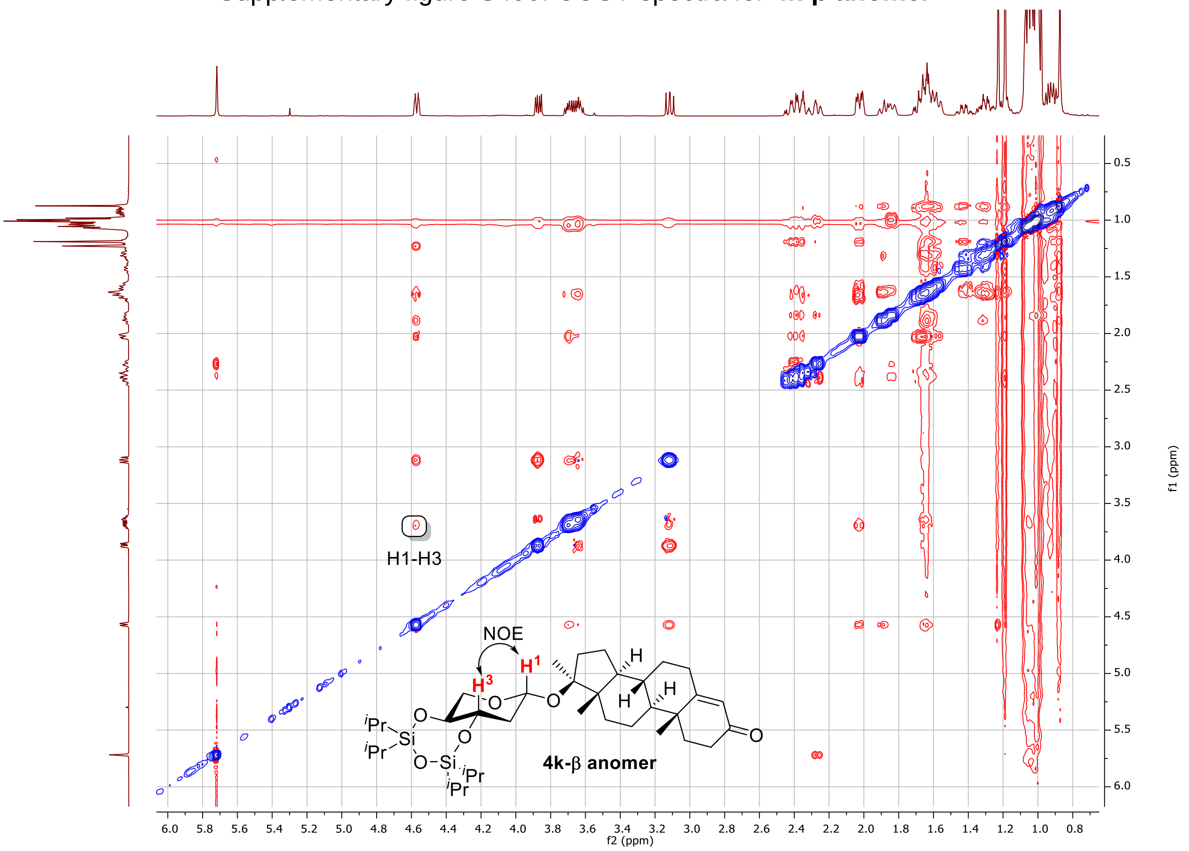

Supplementary figure S491: NOESY spectra for **4k-β anomer**

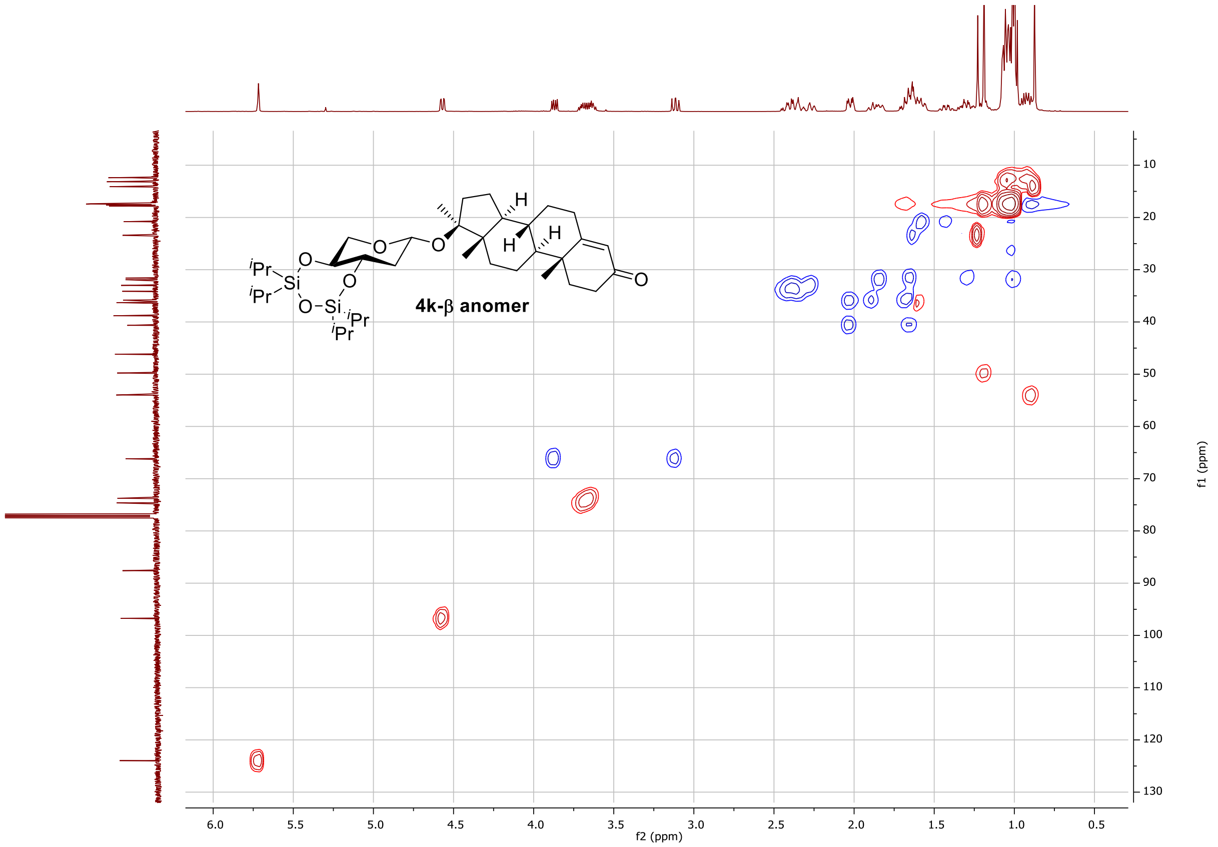

Supplementary figure S492: HSQC spectra for **4k-β anomer**

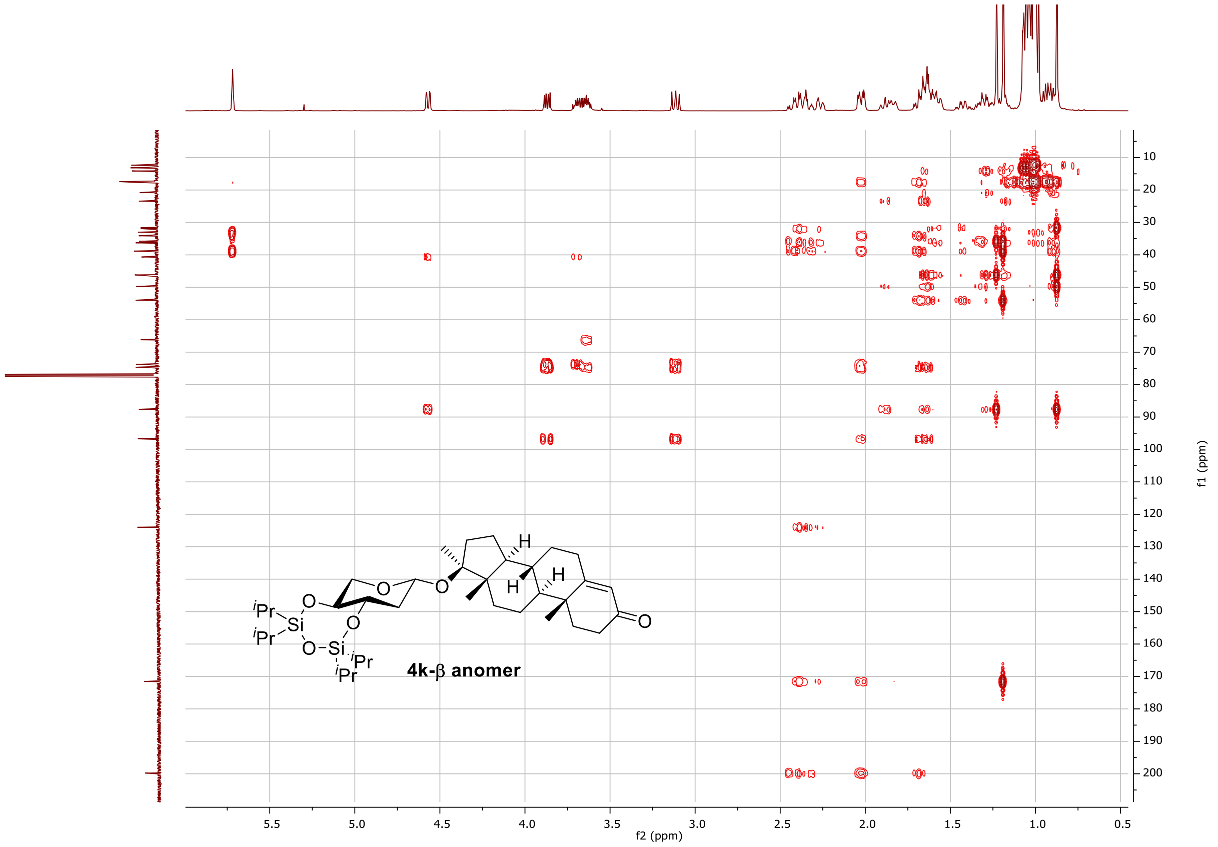

Supplementary figure S493: HMBC spectra for **4k-β anomer**

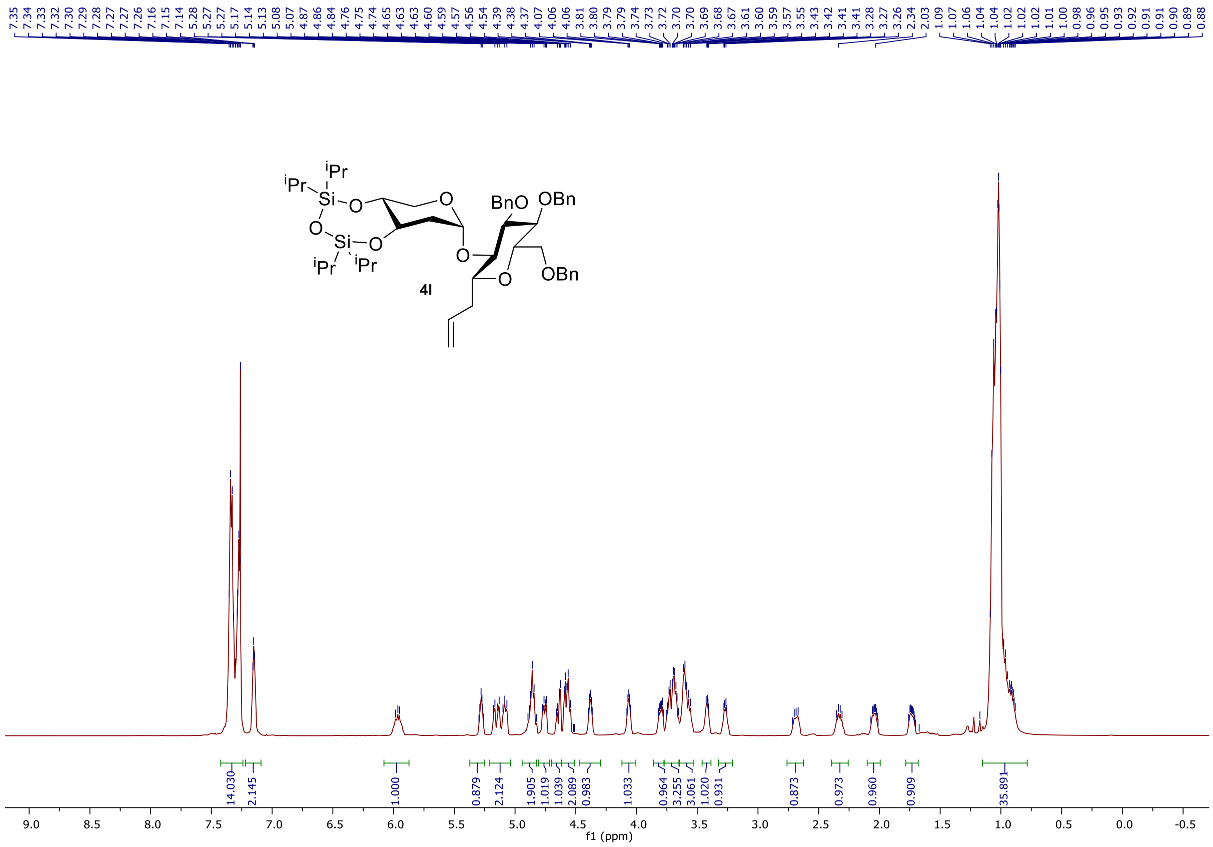

Supplementary figure S494: ^{1}H spectra for **4l**

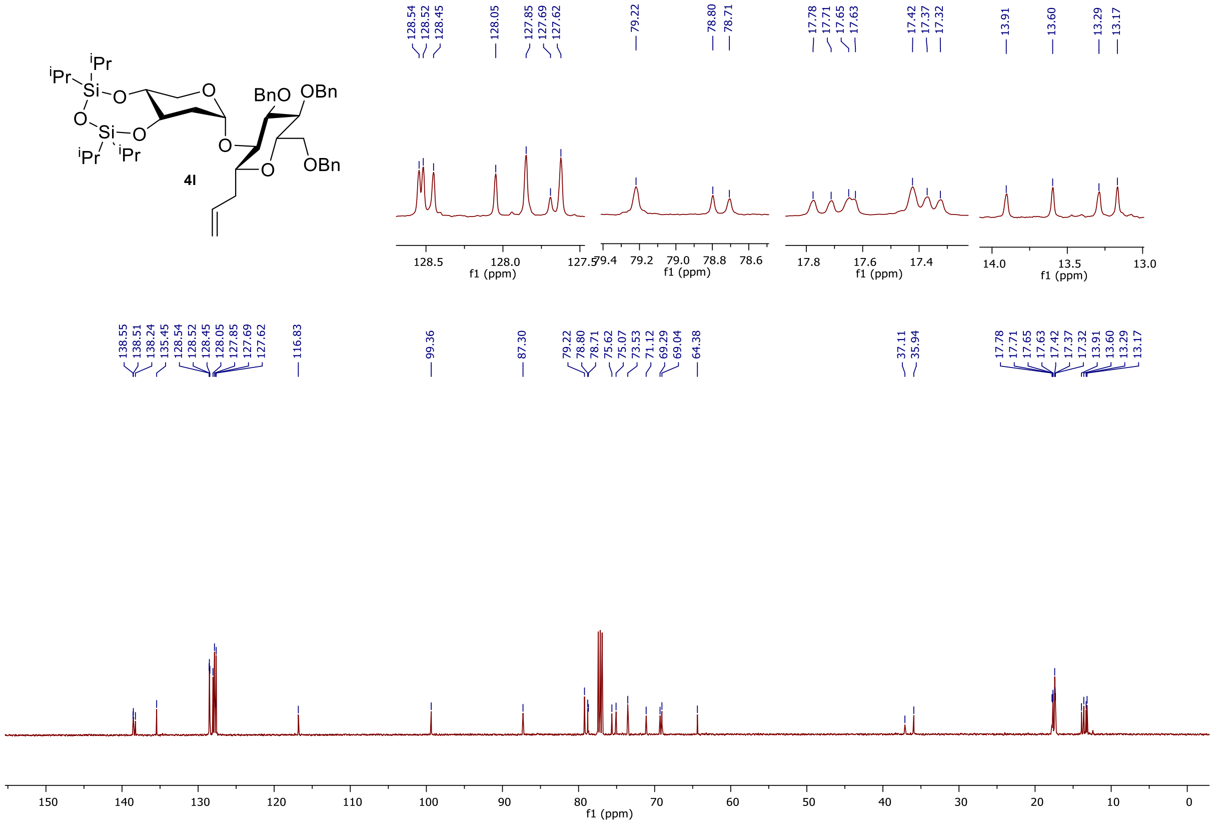

Supplementary figure S495: ^{13}C spectra for **4l**

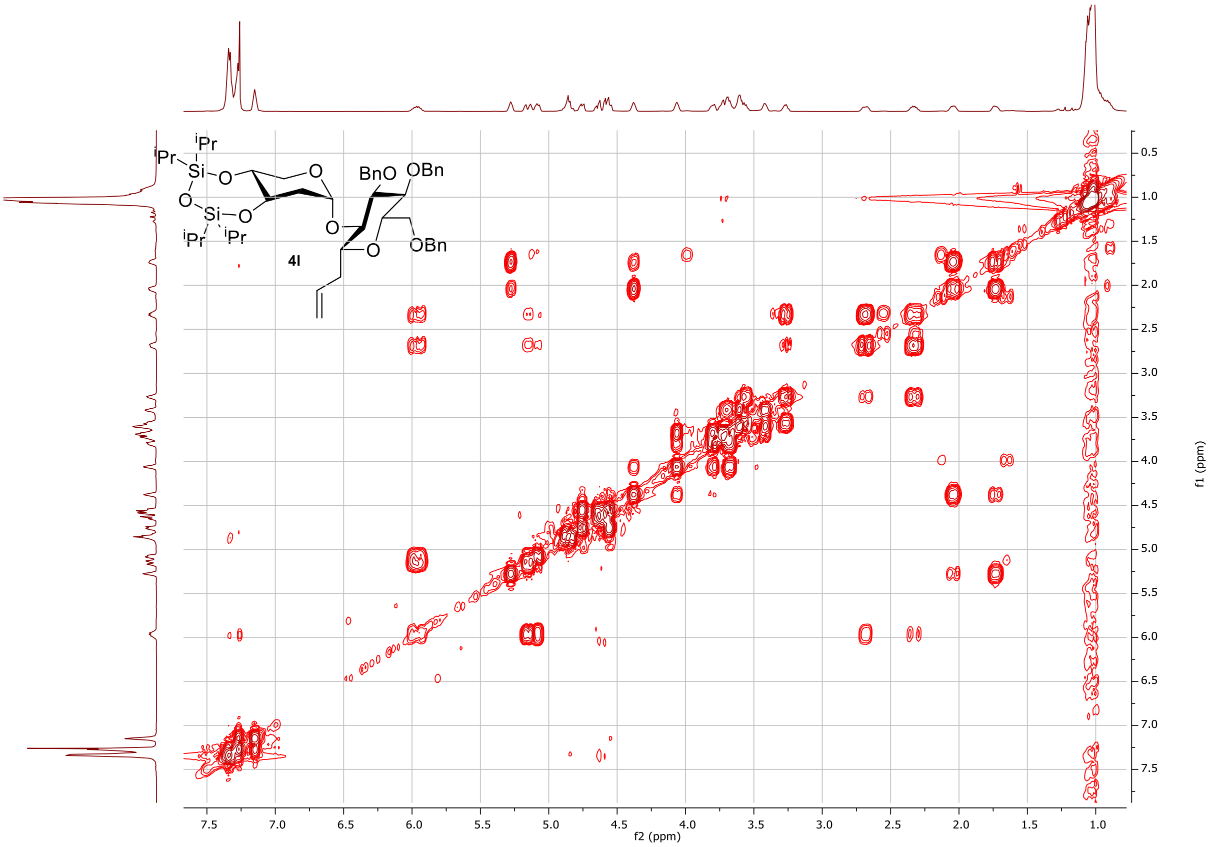

Supplementary figure S496: COSY spectra for **4l**

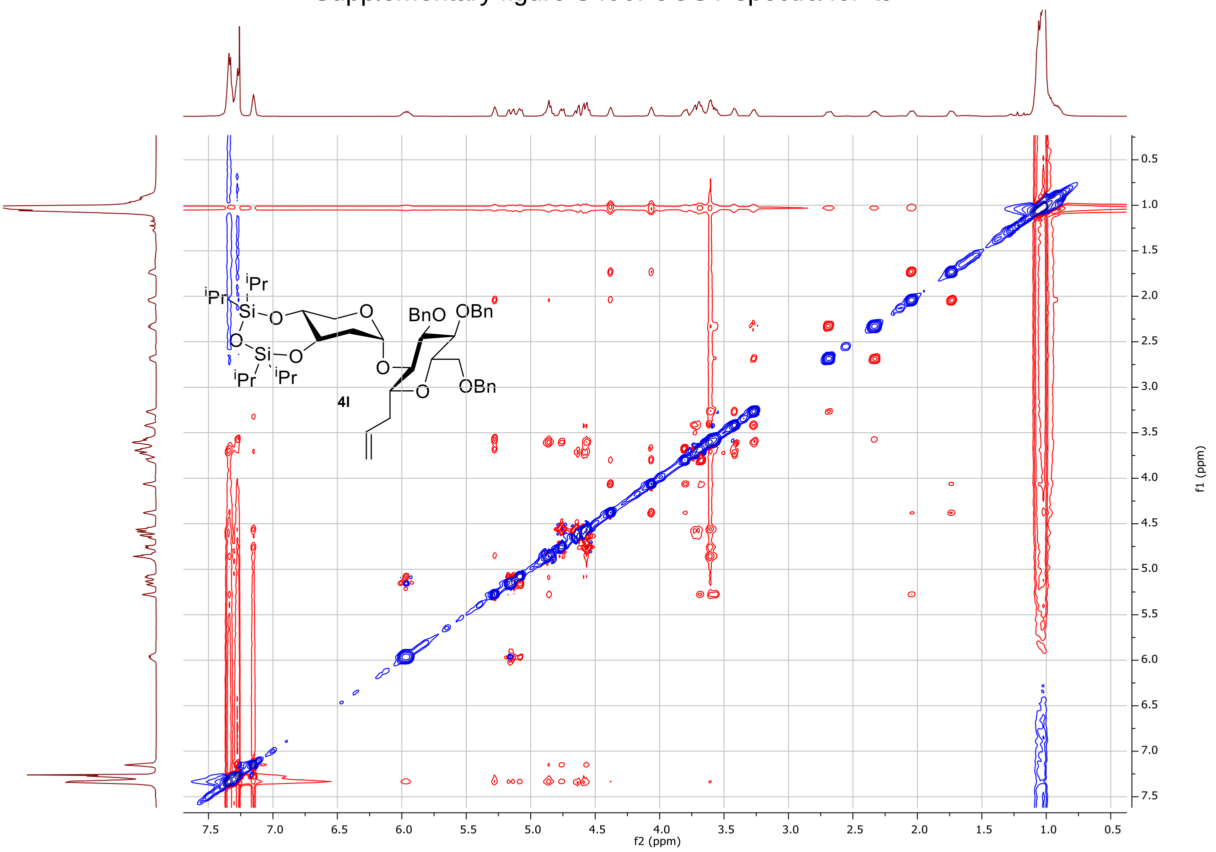

Supplementary figure S497: NOESY spectra for **4l**

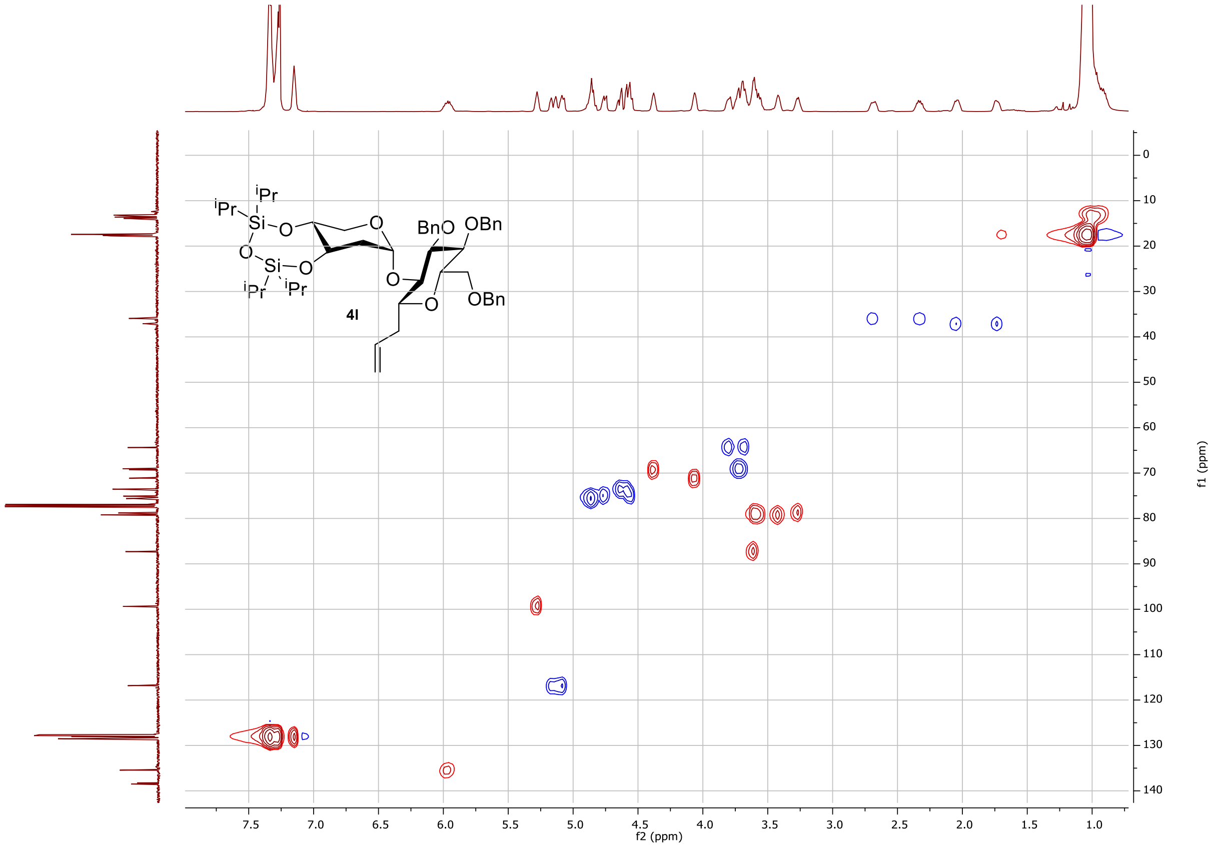

Supplementary figure S498: HSQC spectra for **4l**

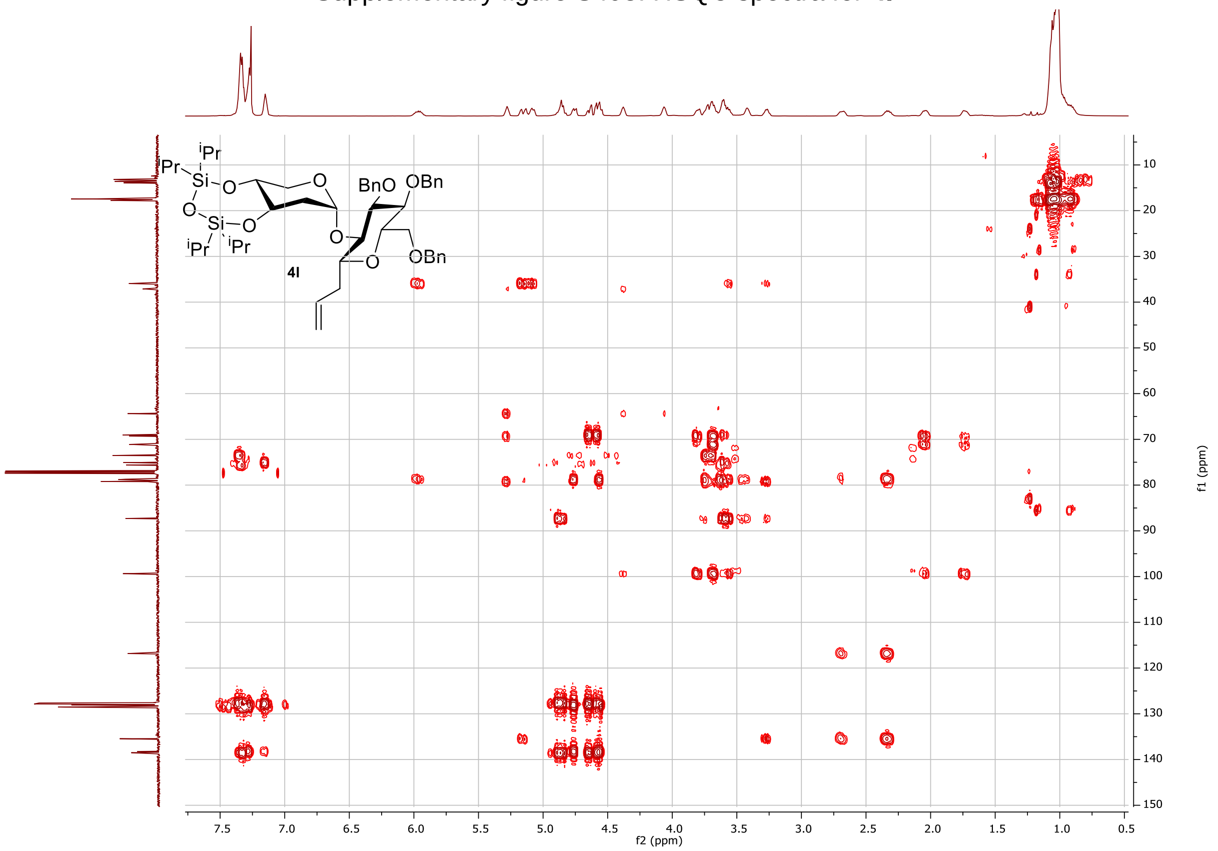

Supplementary figure S499: HMBC spectra for **4l**

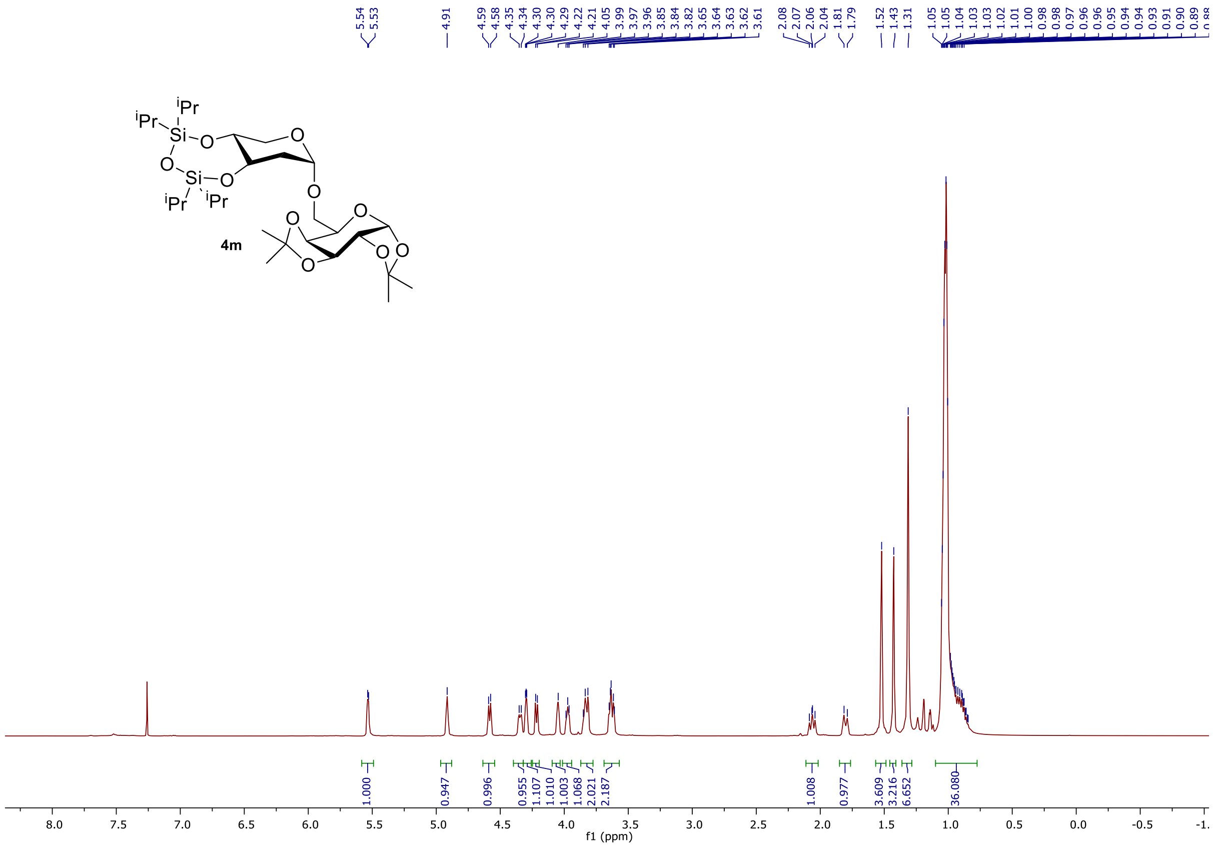

Supplementary figure S500: ^{1}H spectra for **4m**

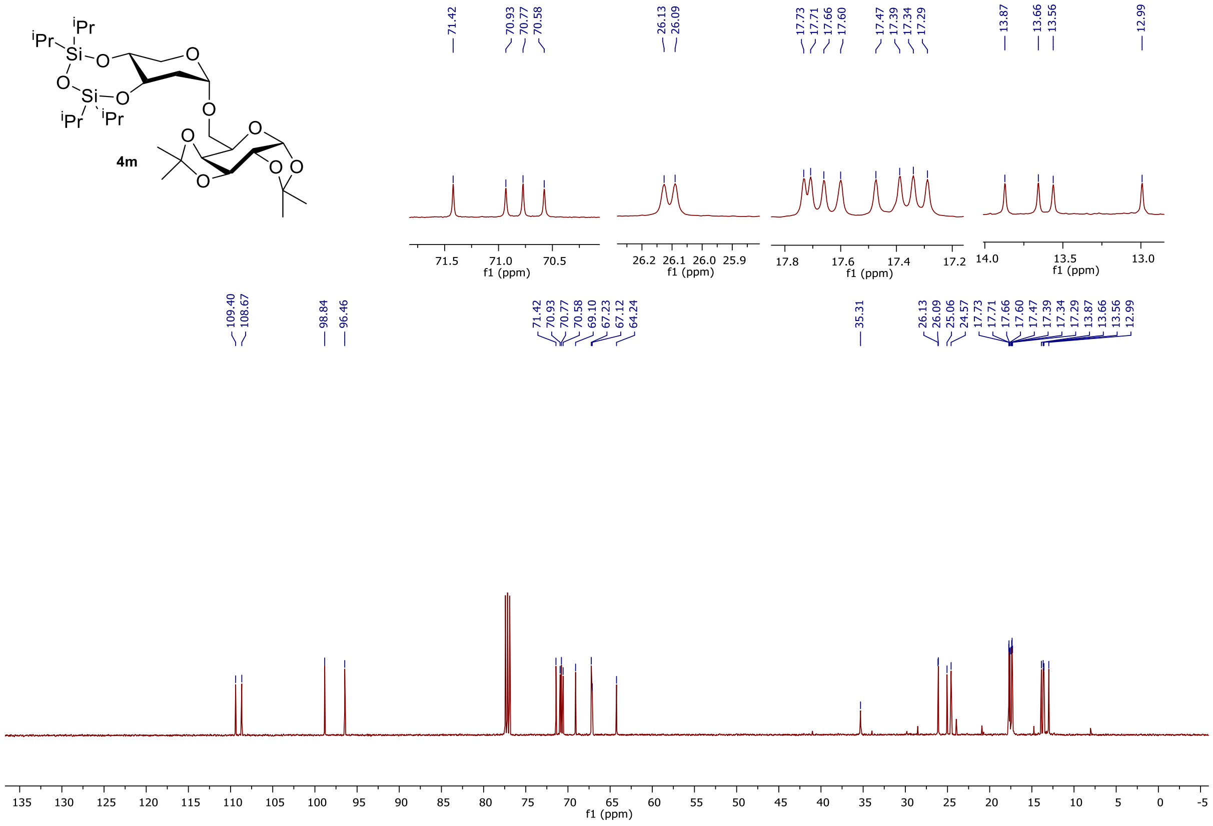

Supplementary figure S501: ^{13}C spectra for **4m**

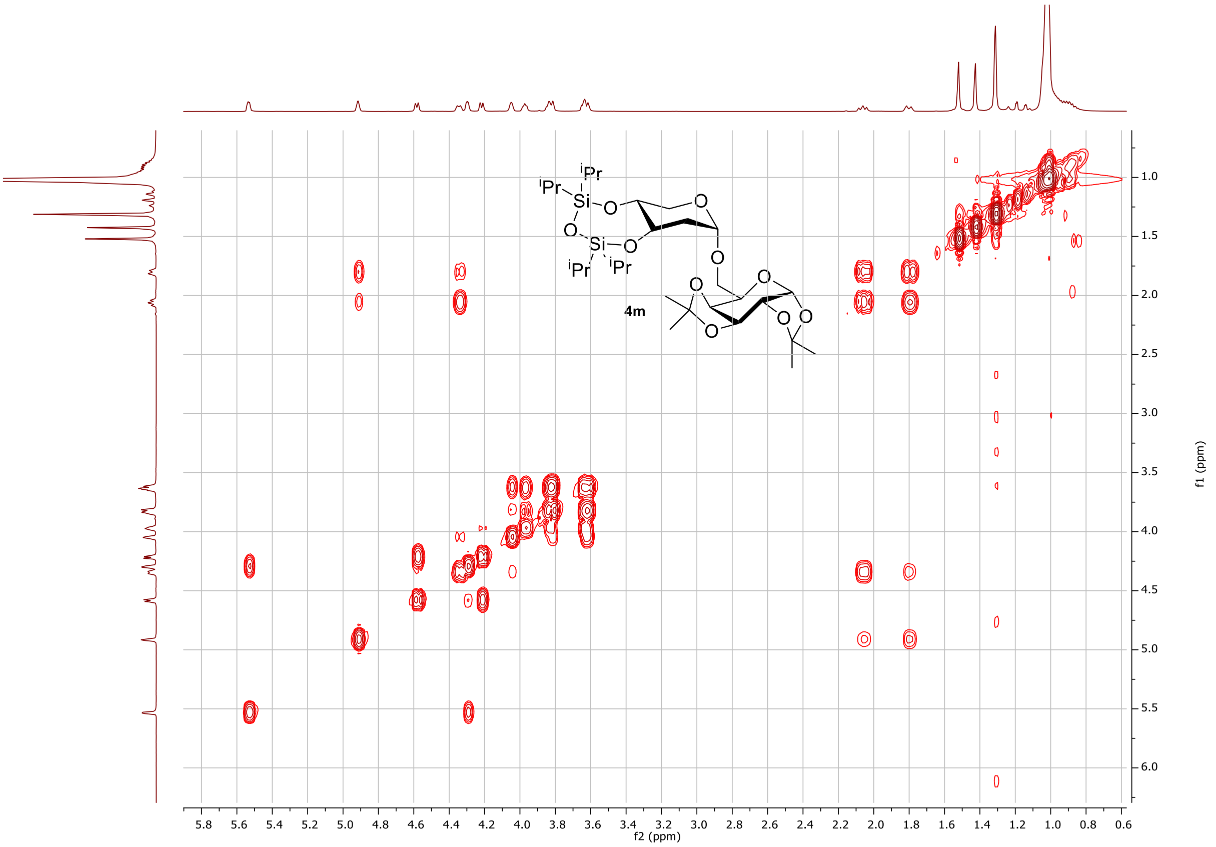

Supplementary figure S502: COSY spectra for **4m**

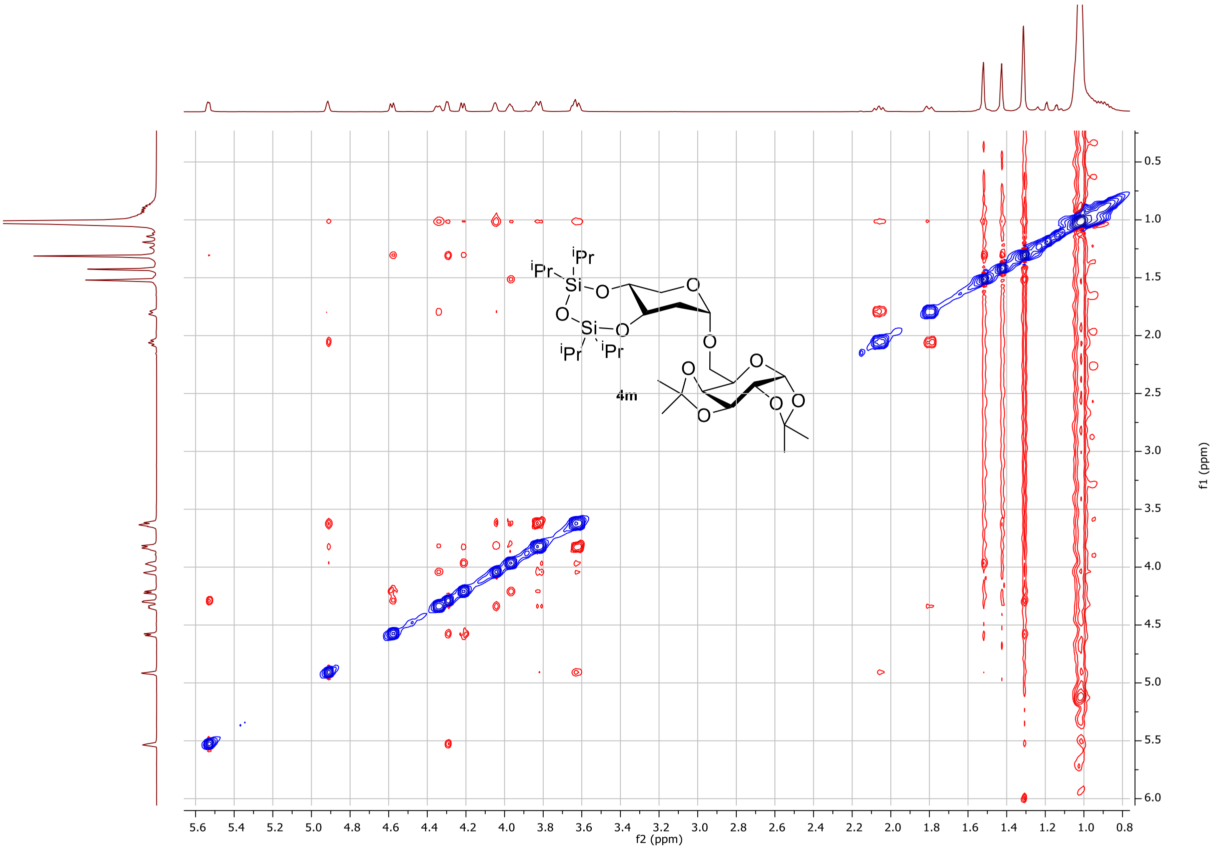

Supplementary figure S503: NOESY spectra for **4m**

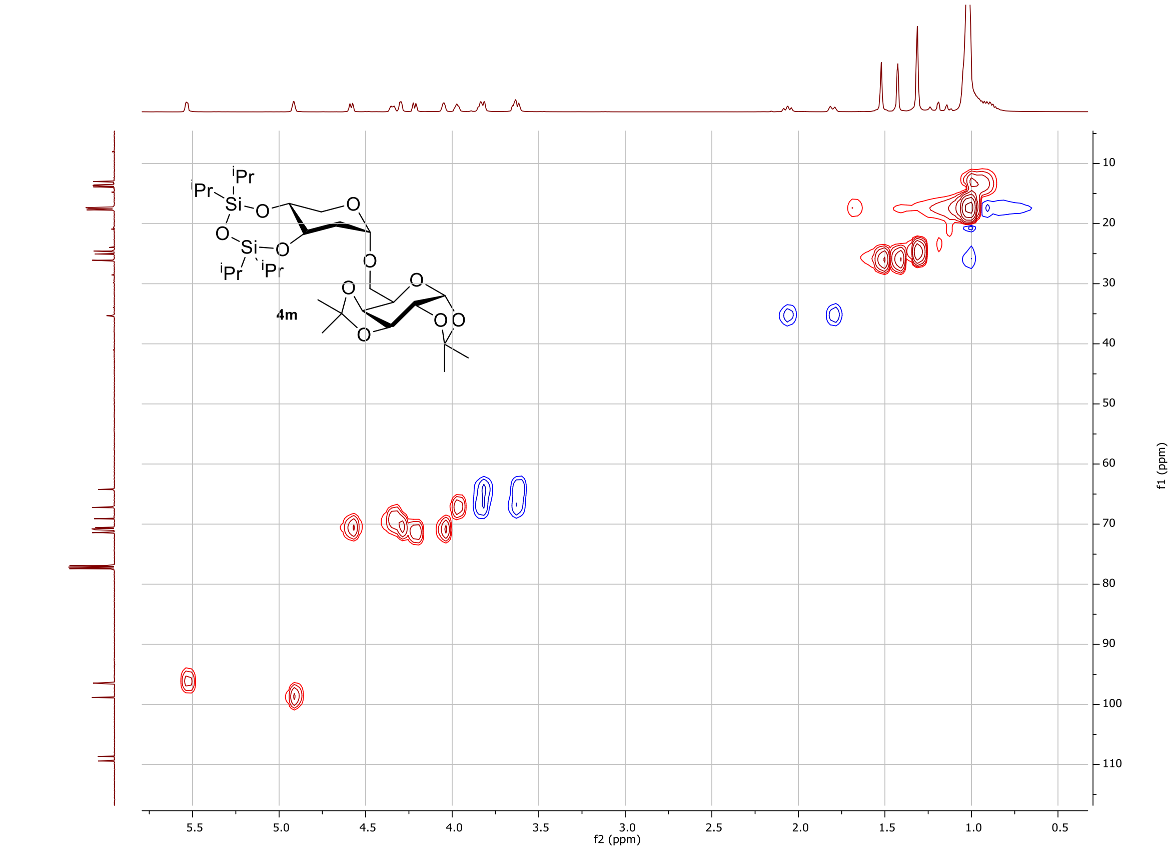

Supplementary figure S504: HSQC spectra for **4m**

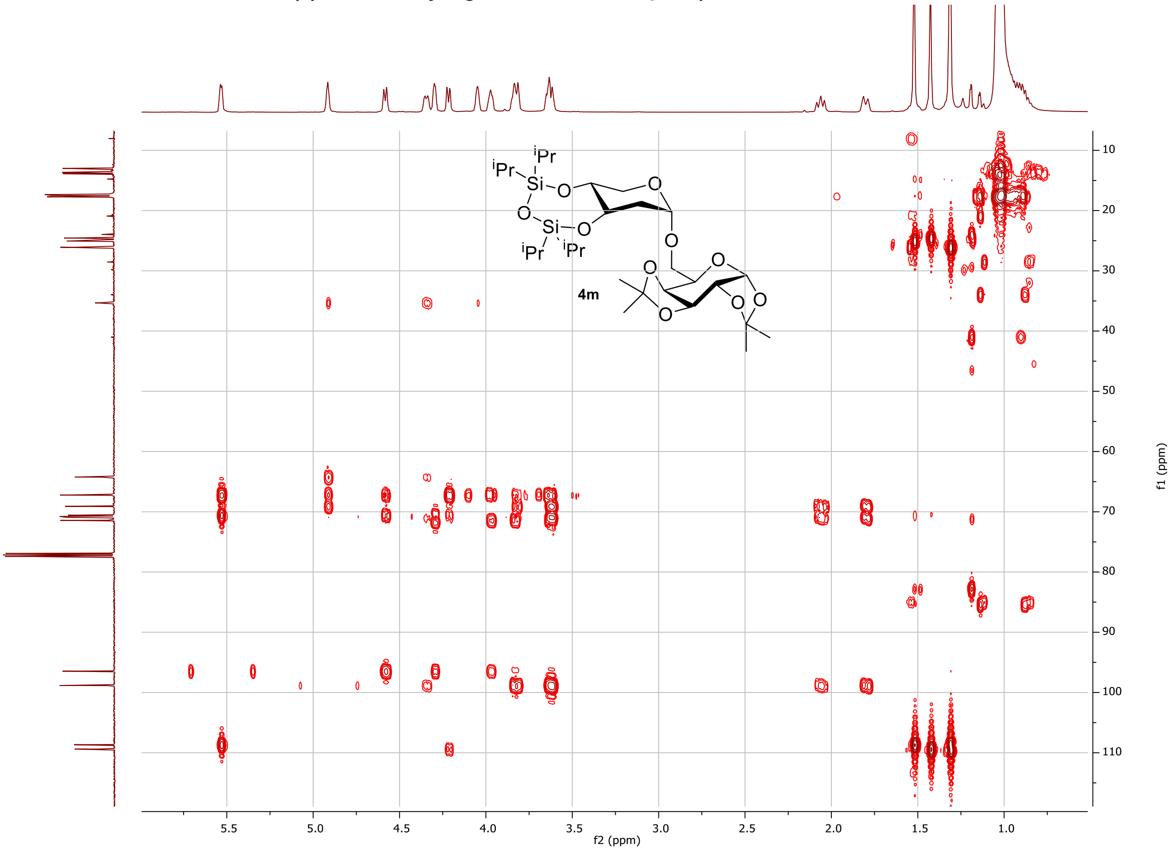

Supplementary figure S505: HMBC spectra for **4m**

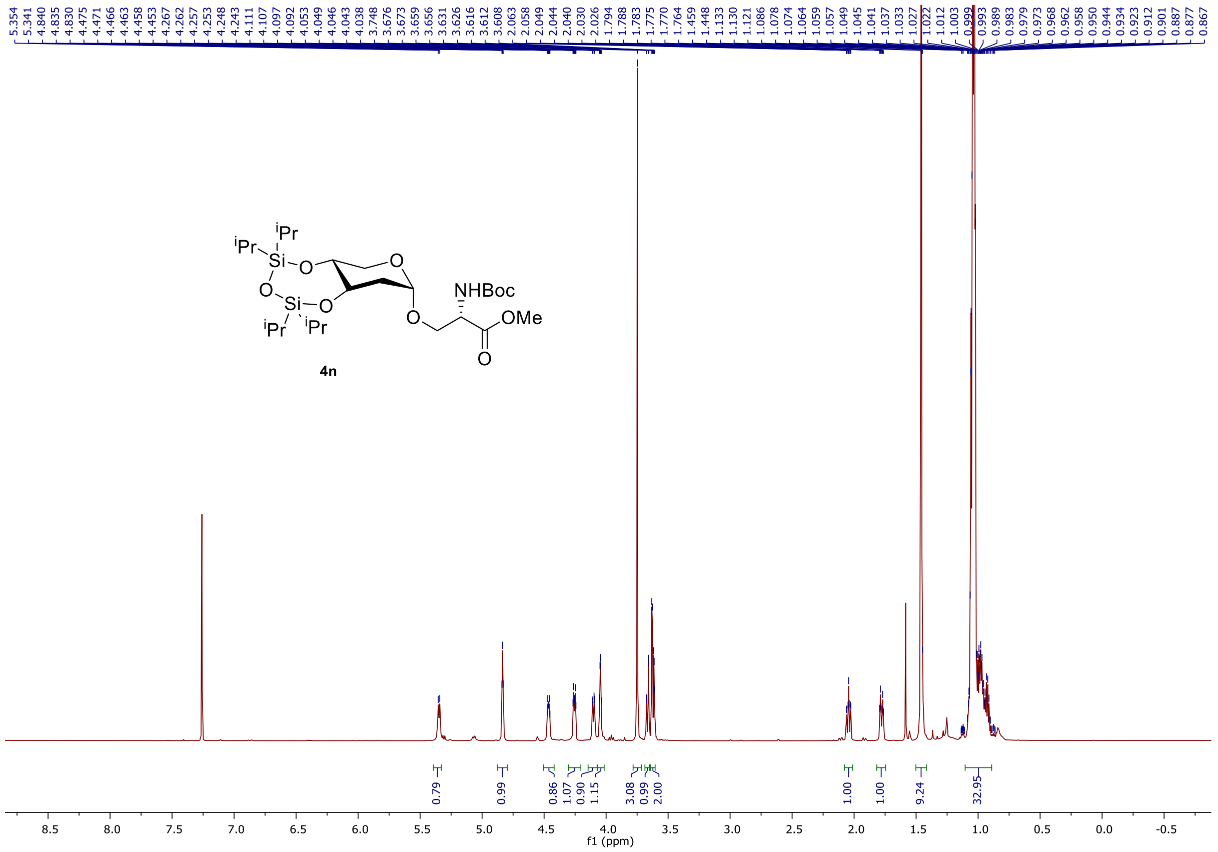

Supplementary figure S506: ^{1}H spectra for **4n**

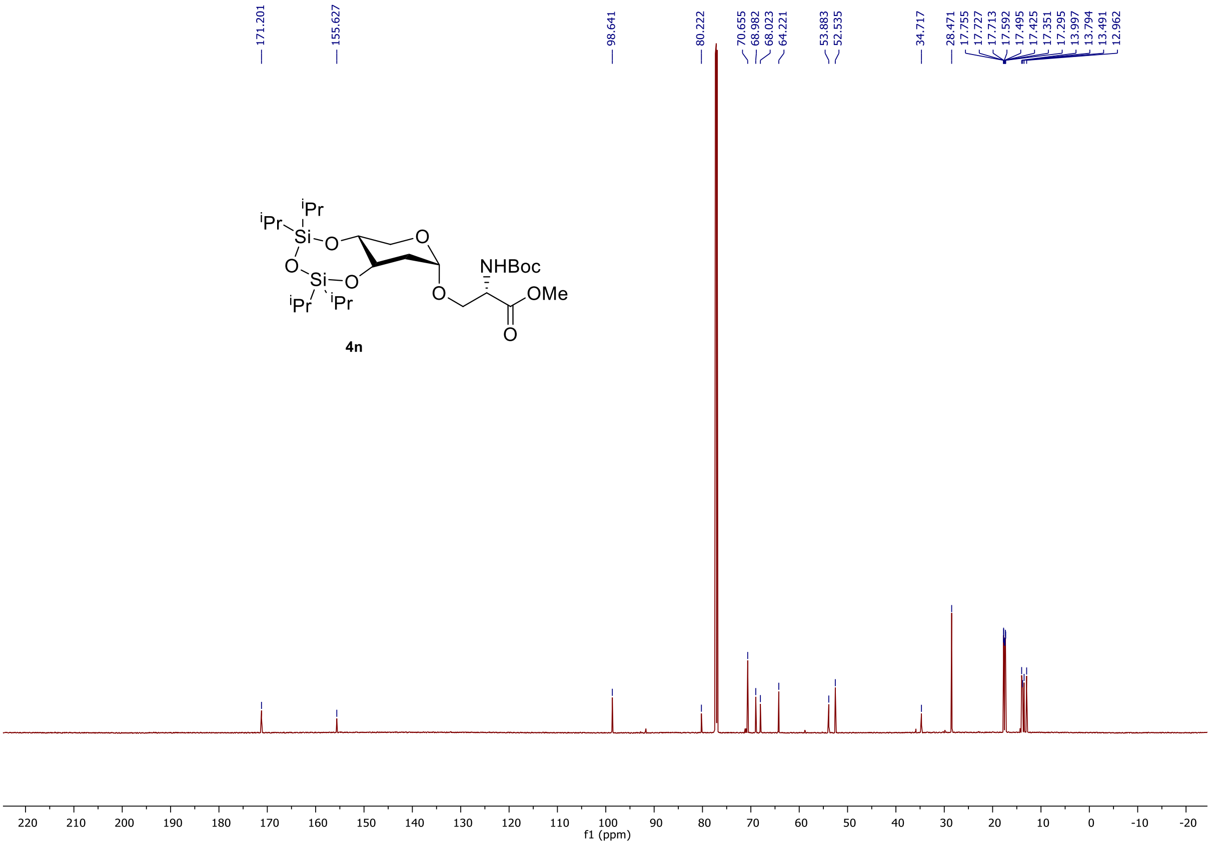

Supplementary figure S507: ^{13}C spectra for **4n**

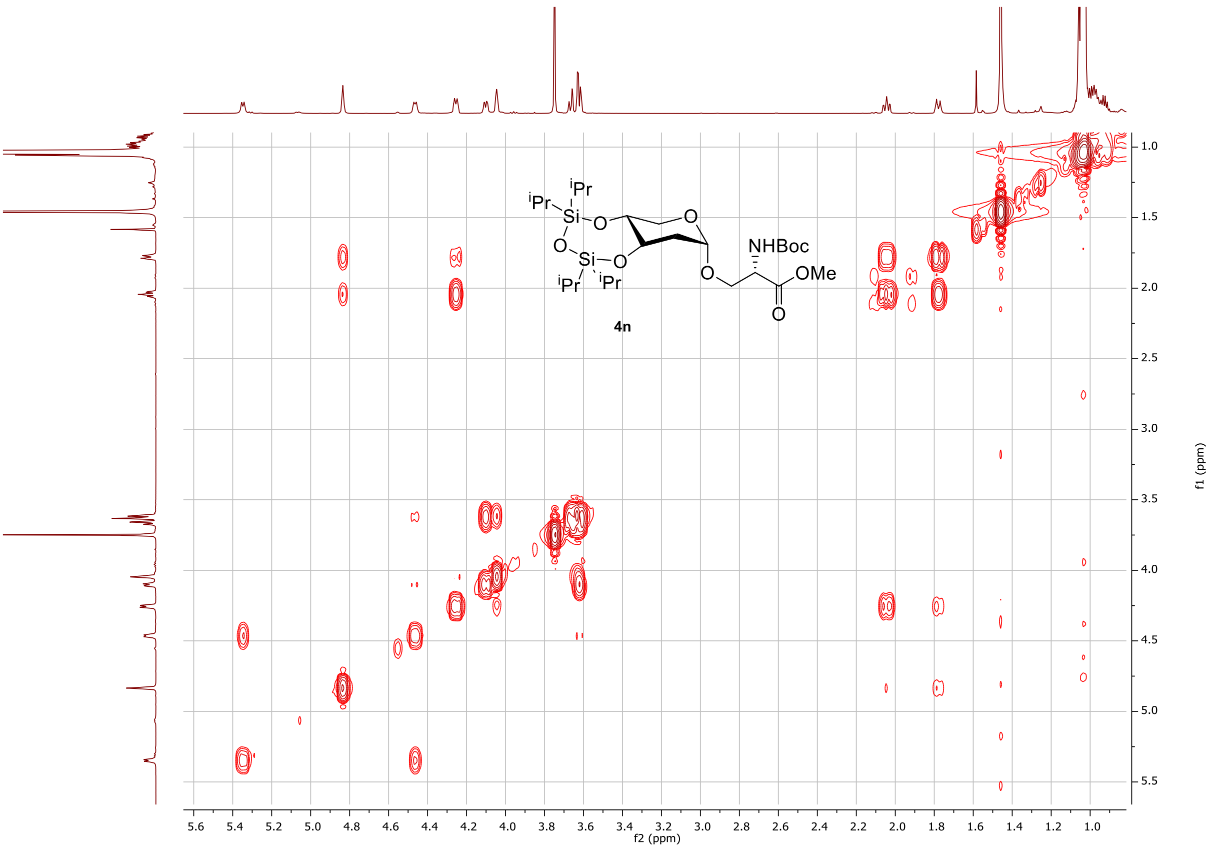

Supplementary figure S508: COSY spectra for **4n**

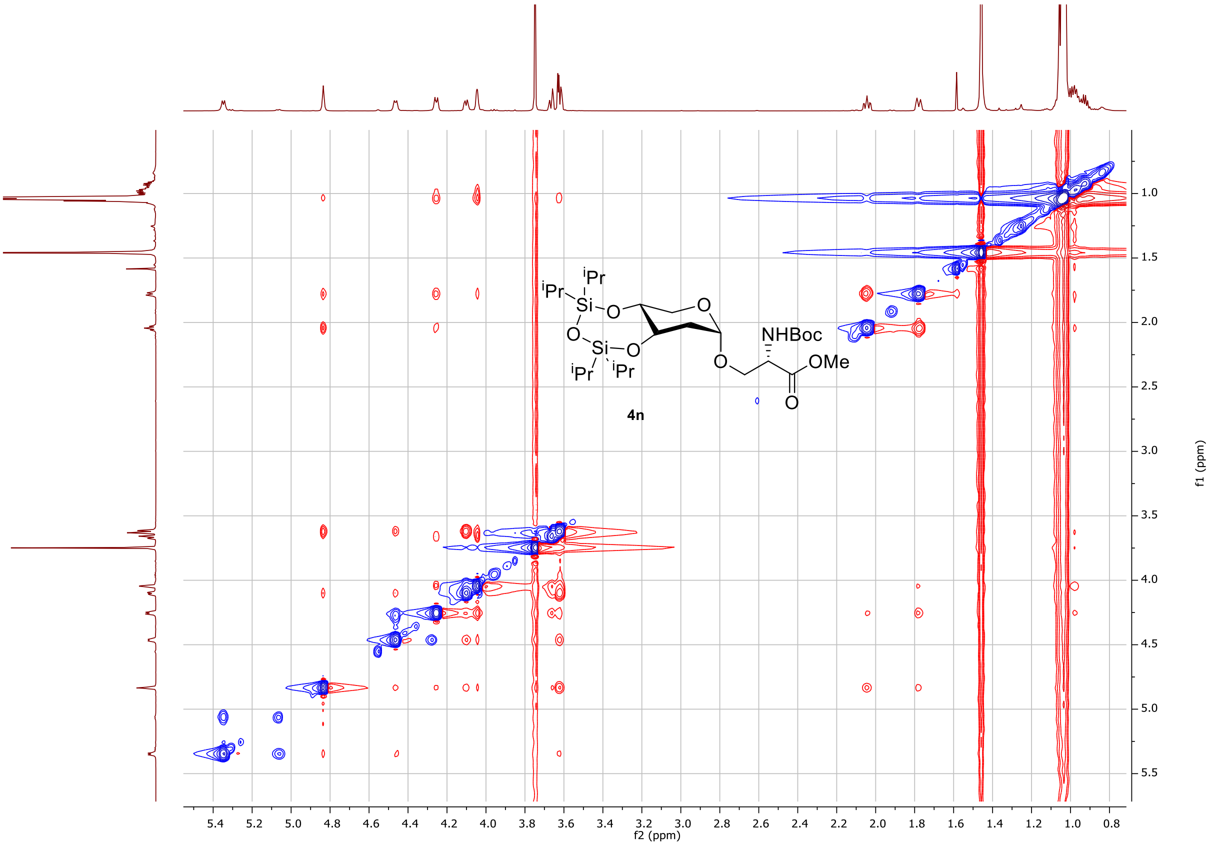

Supplementary figure S509: NOESY spectra for **4n**

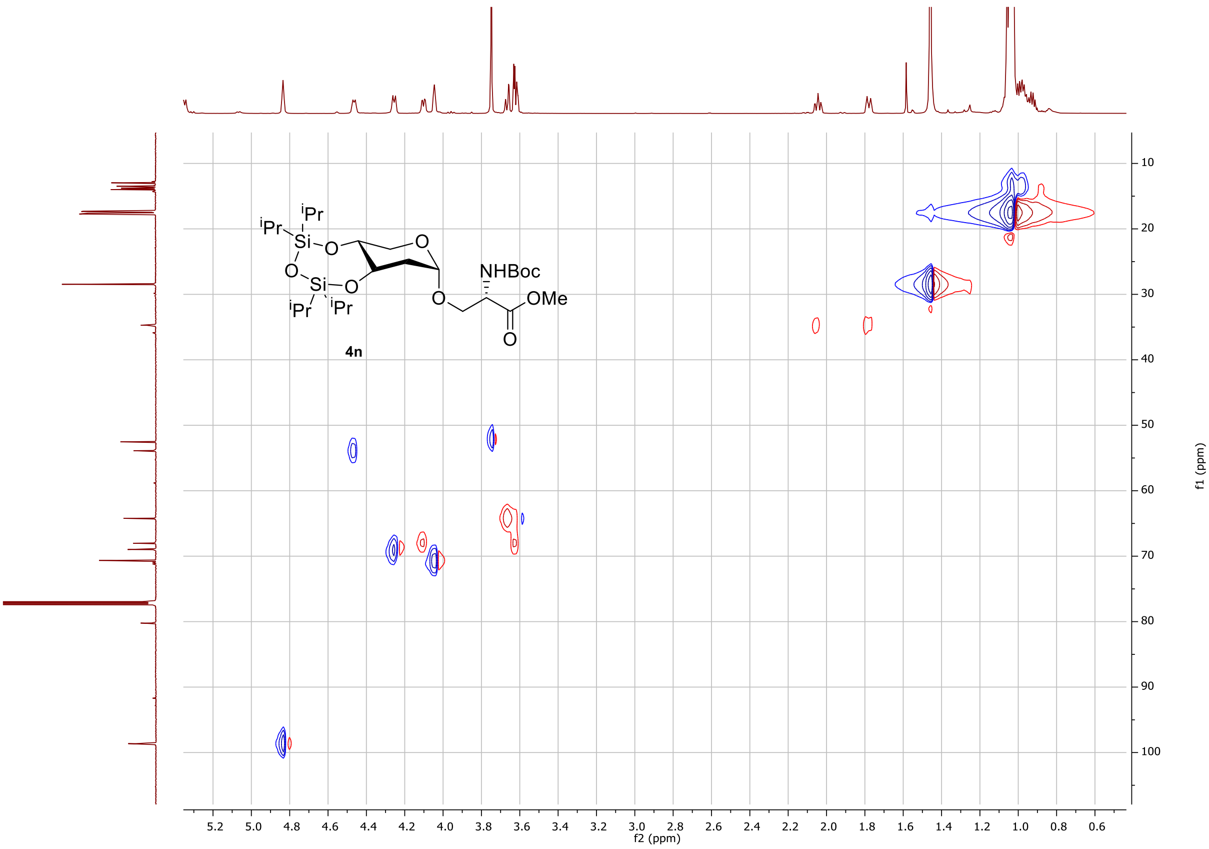

Supplementary figure S510: HSQC spectra for **4n**

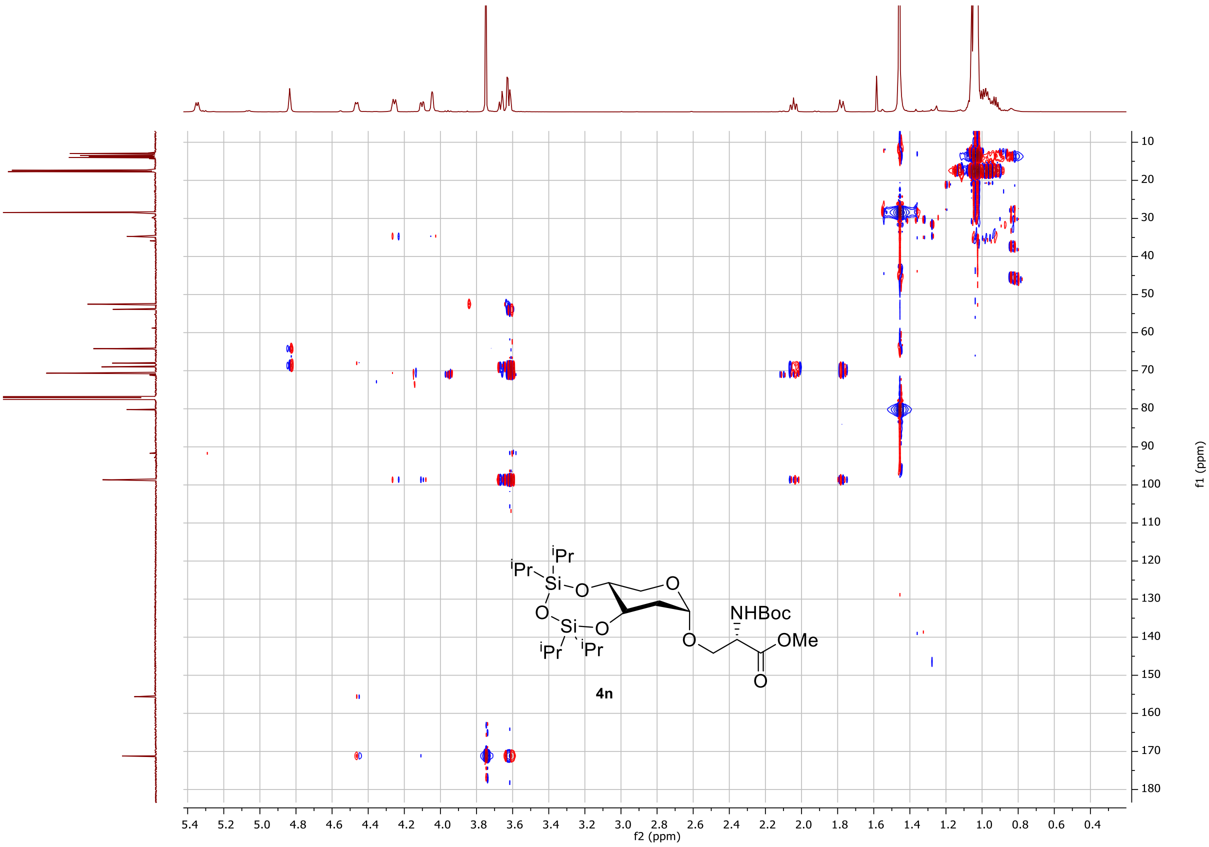

Supplementary figure S511: HMBC spectra for **4n**

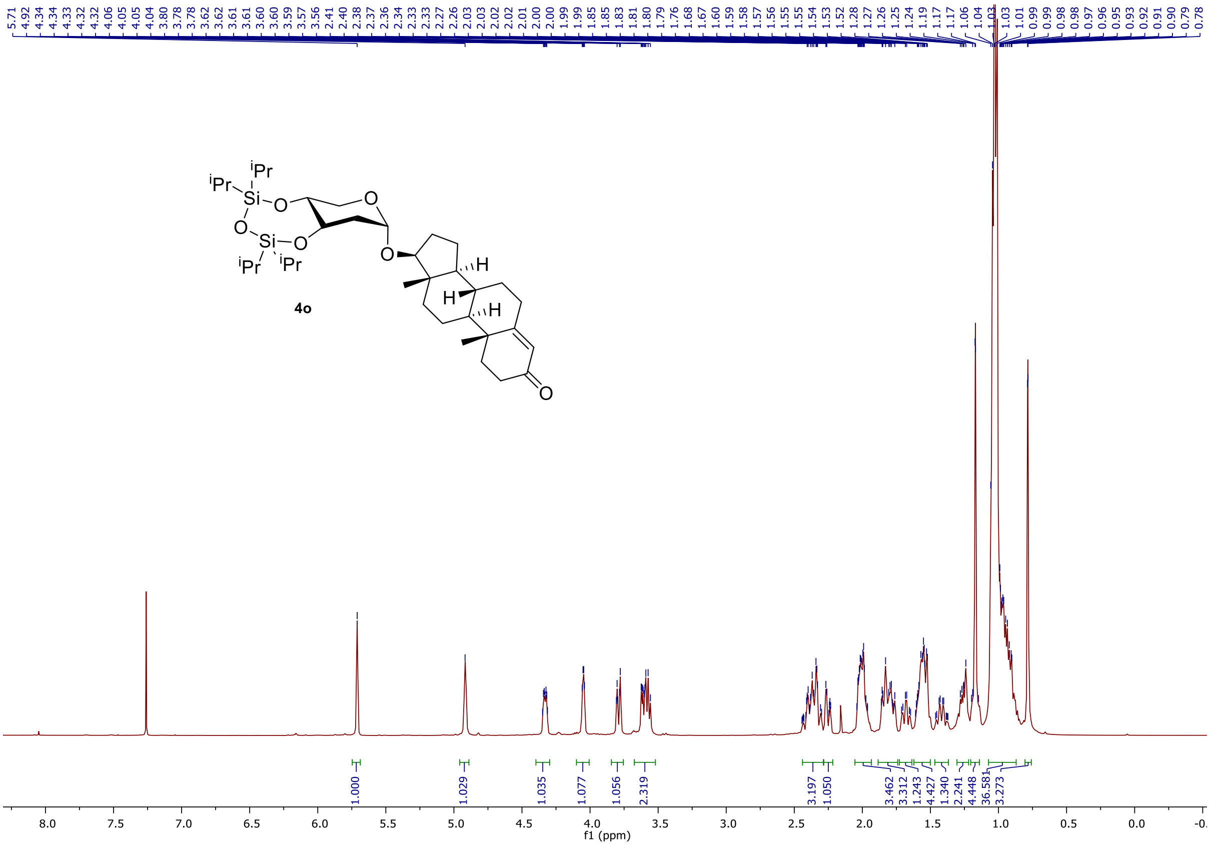

Supplementary figure S512: ^{1}H spectra for **4o**

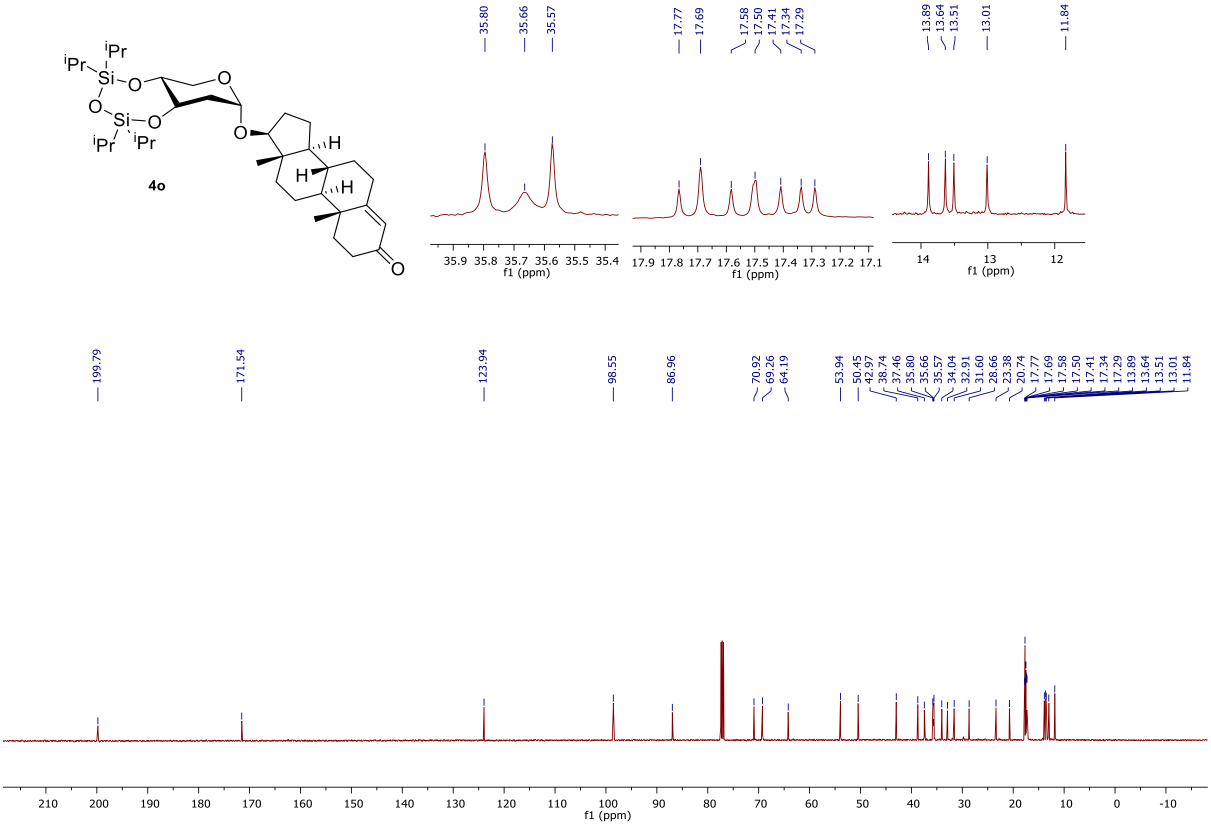

Supplementary figure S513: ^{13}C spectra for **4o**

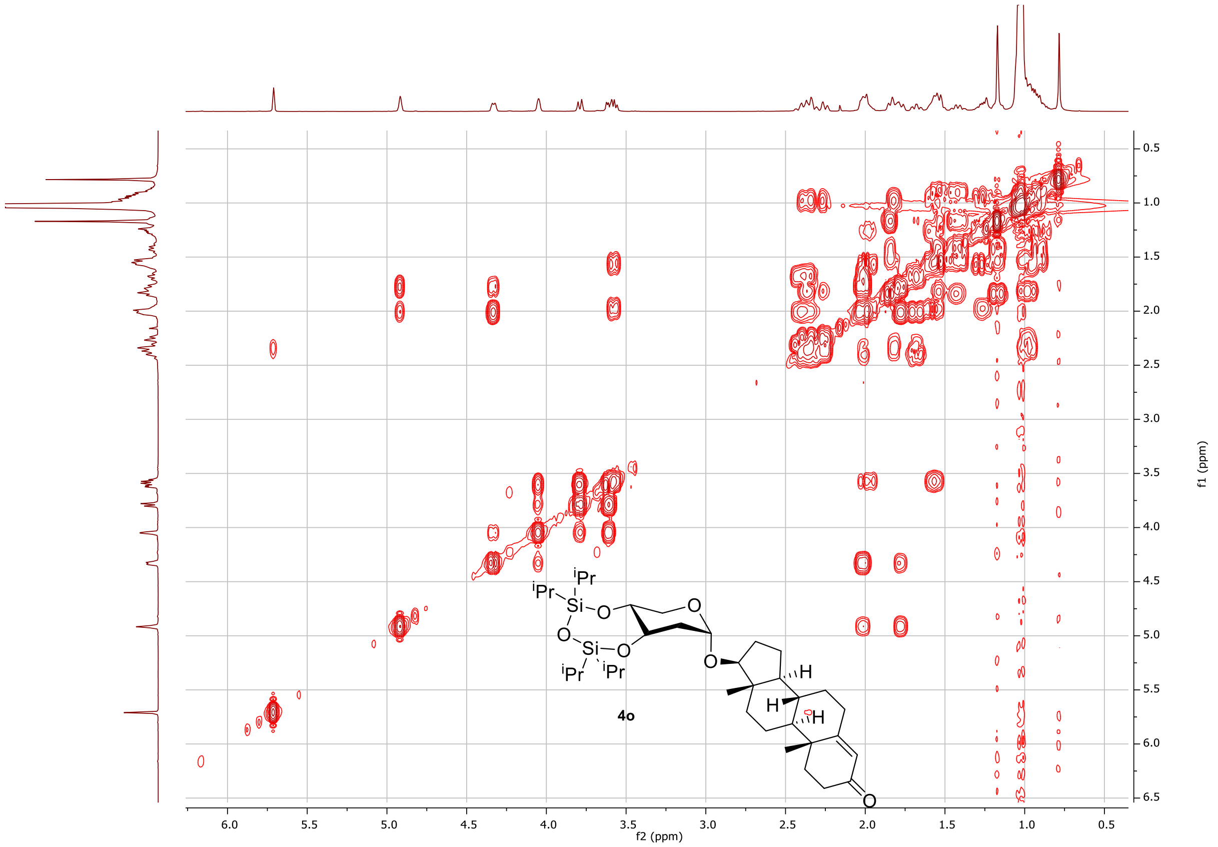

Supplementary figure S514: COSY spectra for **4o**

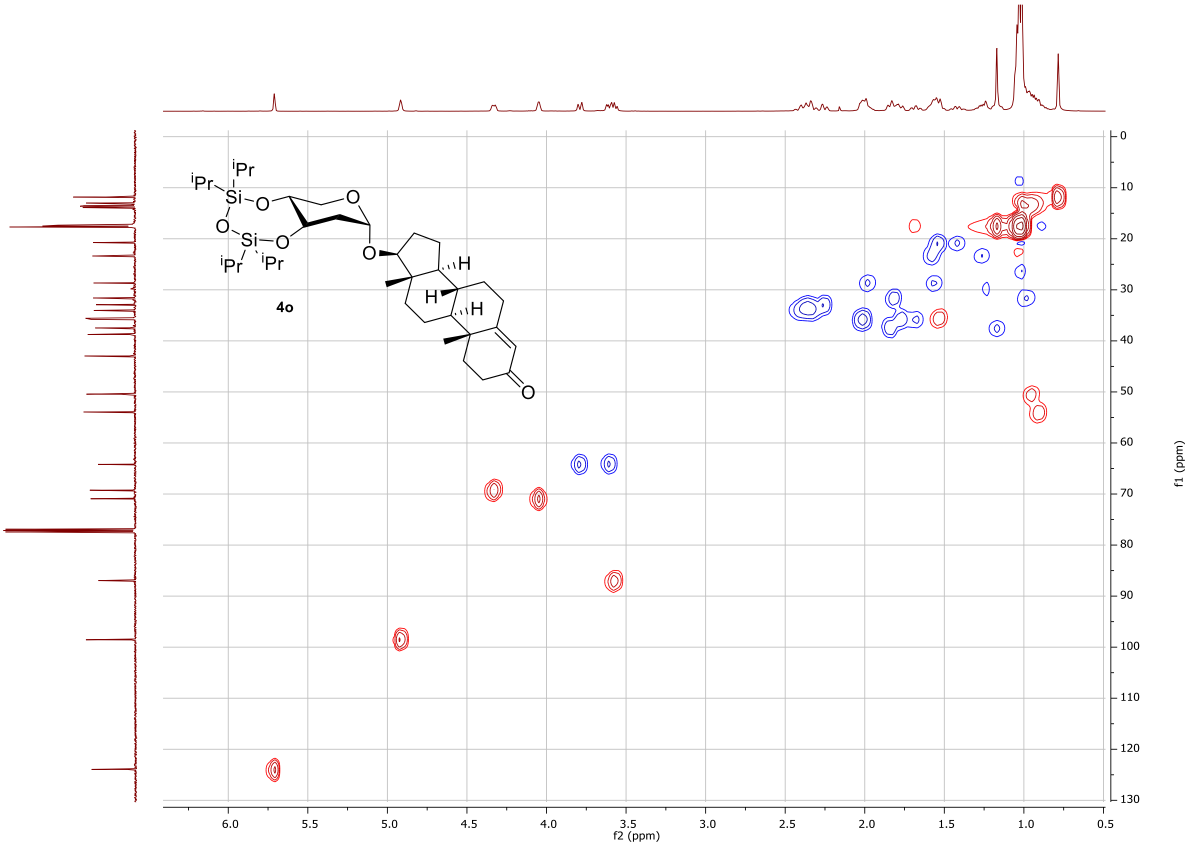

Supplementary figure S515: HSQC spectra for **4o**

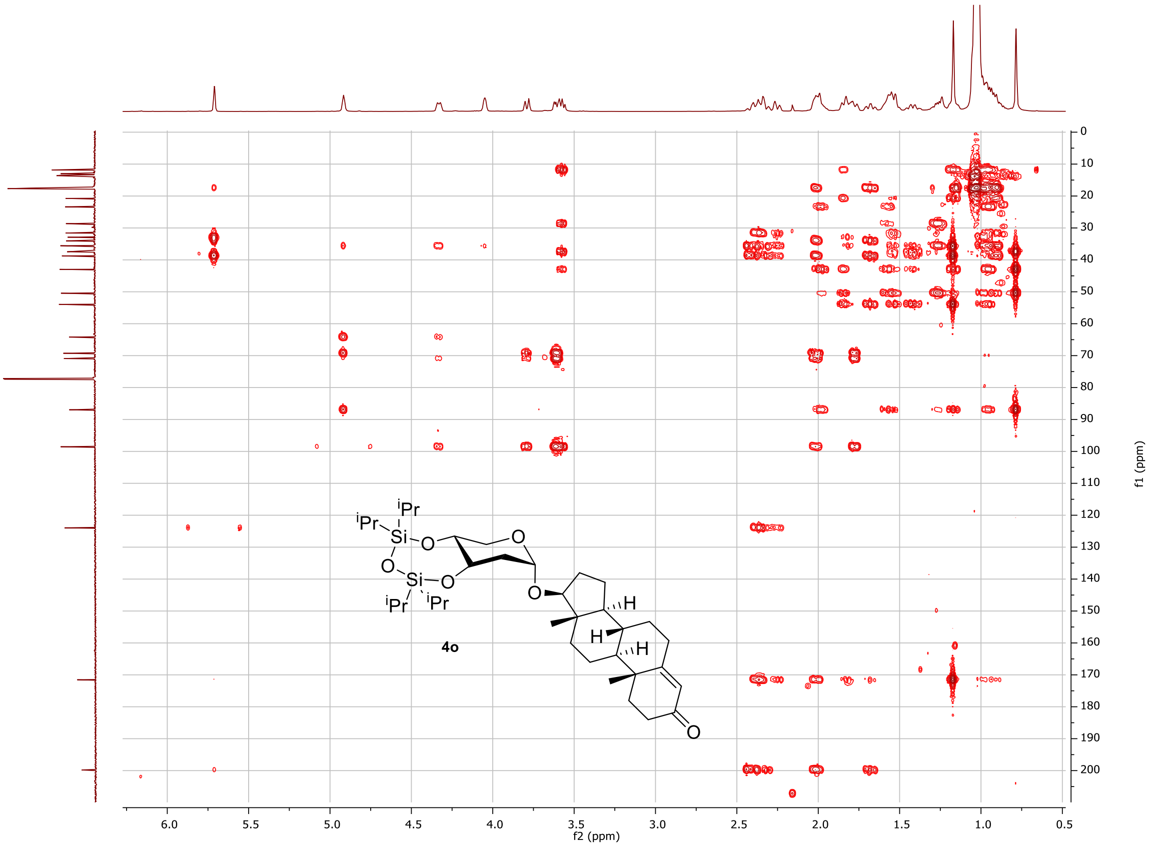

Supplementary figure S516: HMBC spectra for **4o**

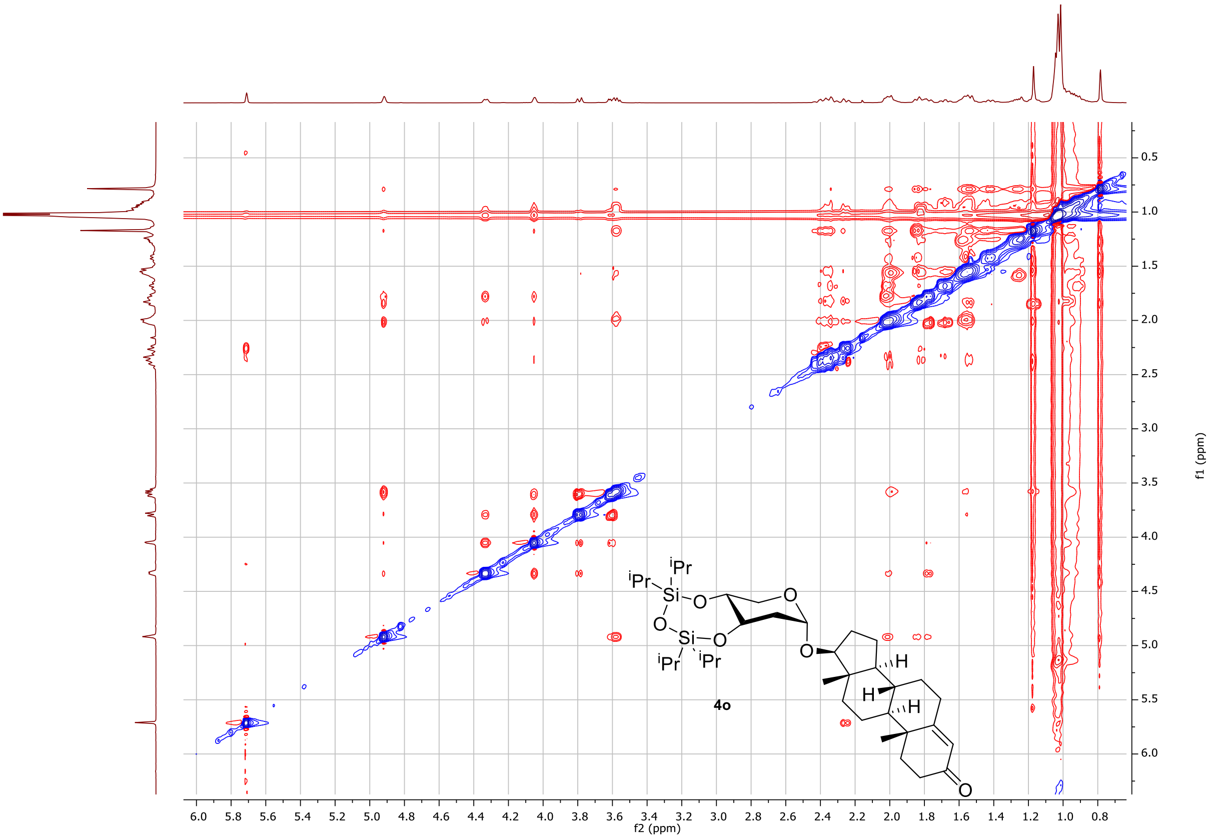

Supplementary figure S517: NOESY spectra for **4o**

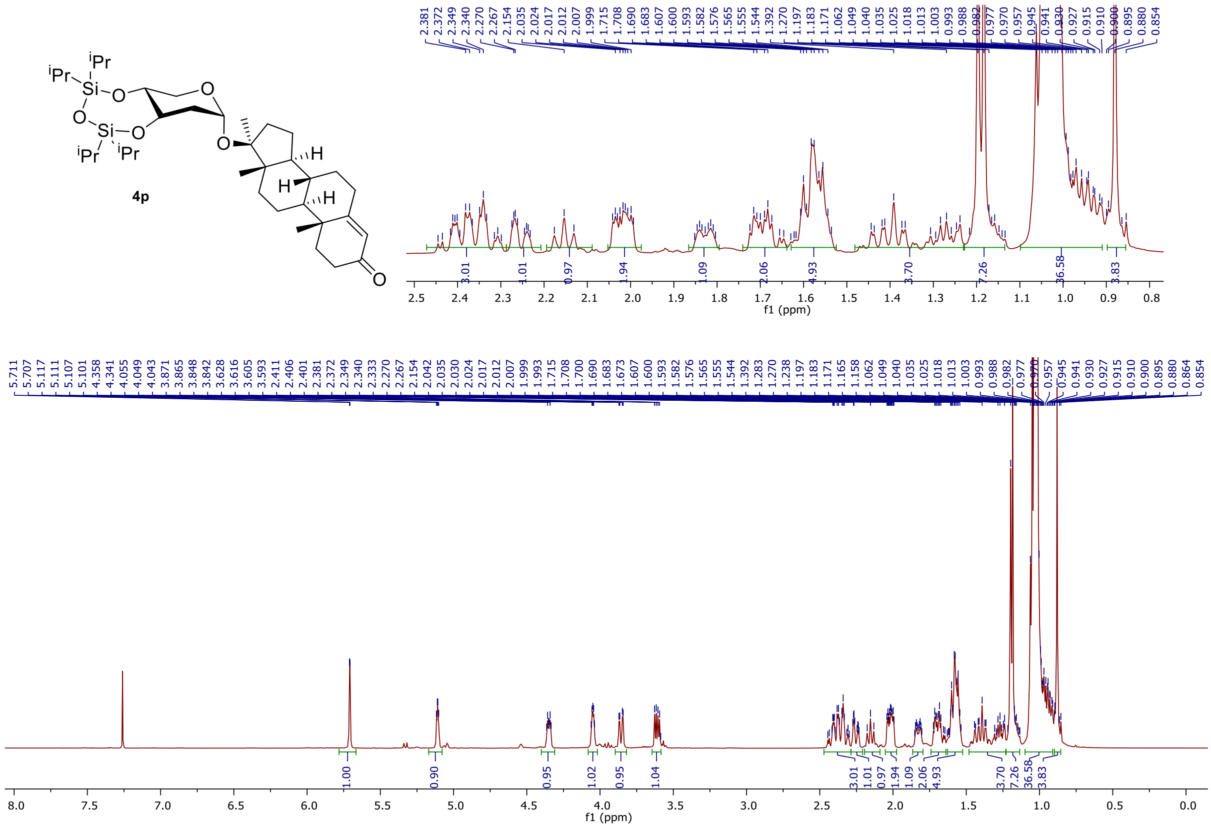

Supplementary figure S518: ^{1}H spectra for **4p**

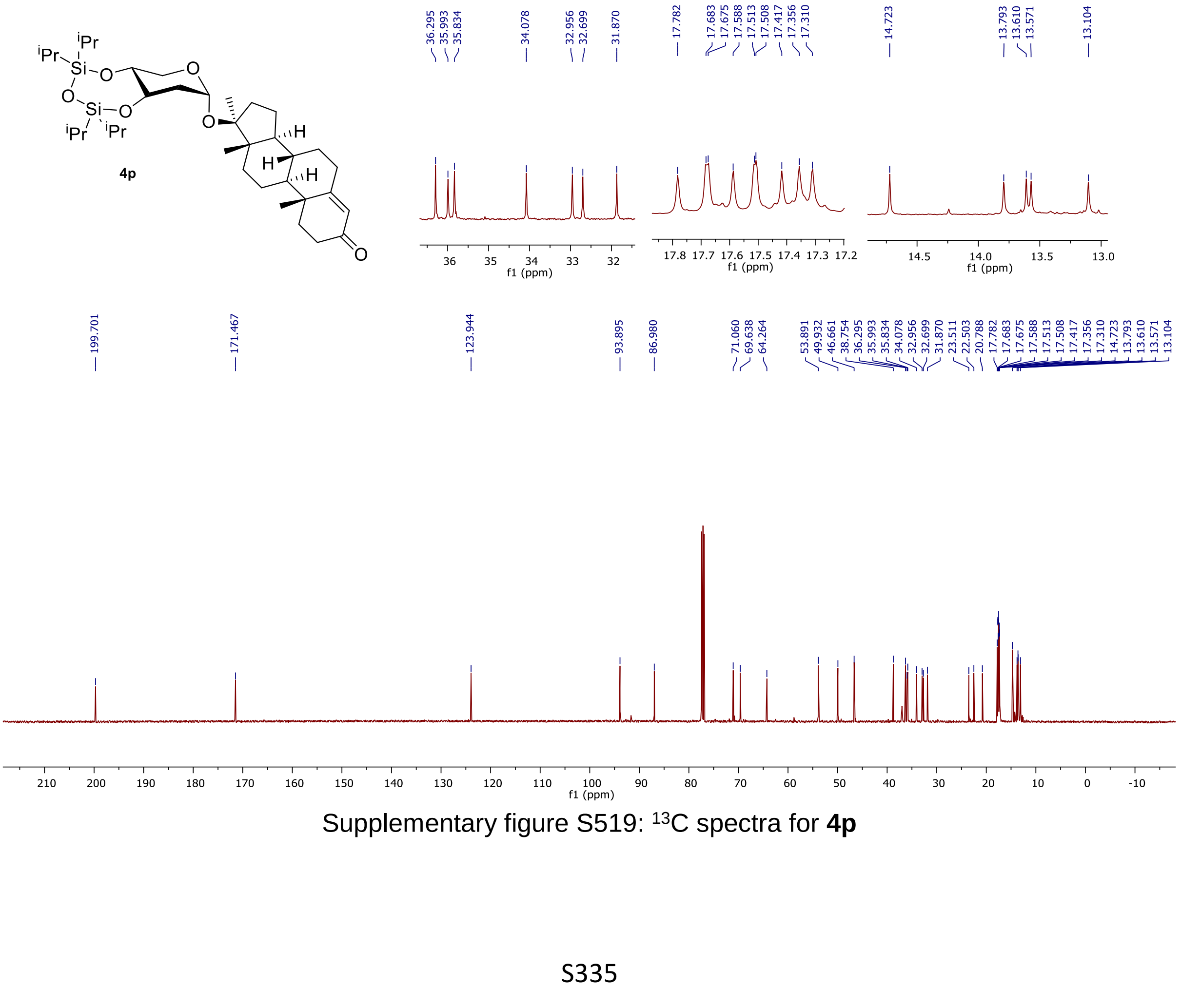

Supplementary figure S519: ^{13}C spectra for **4p**

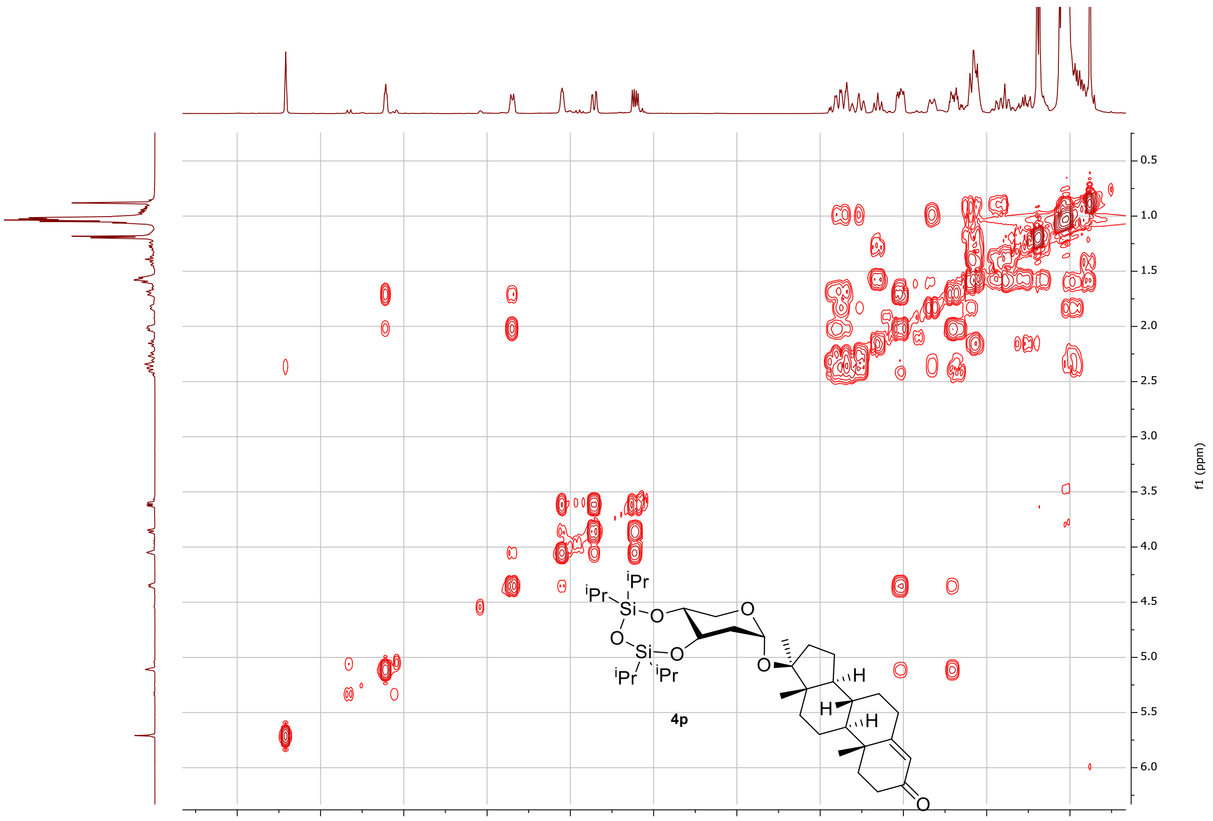

Supplementary figure S520: COSY spectra for **4p**

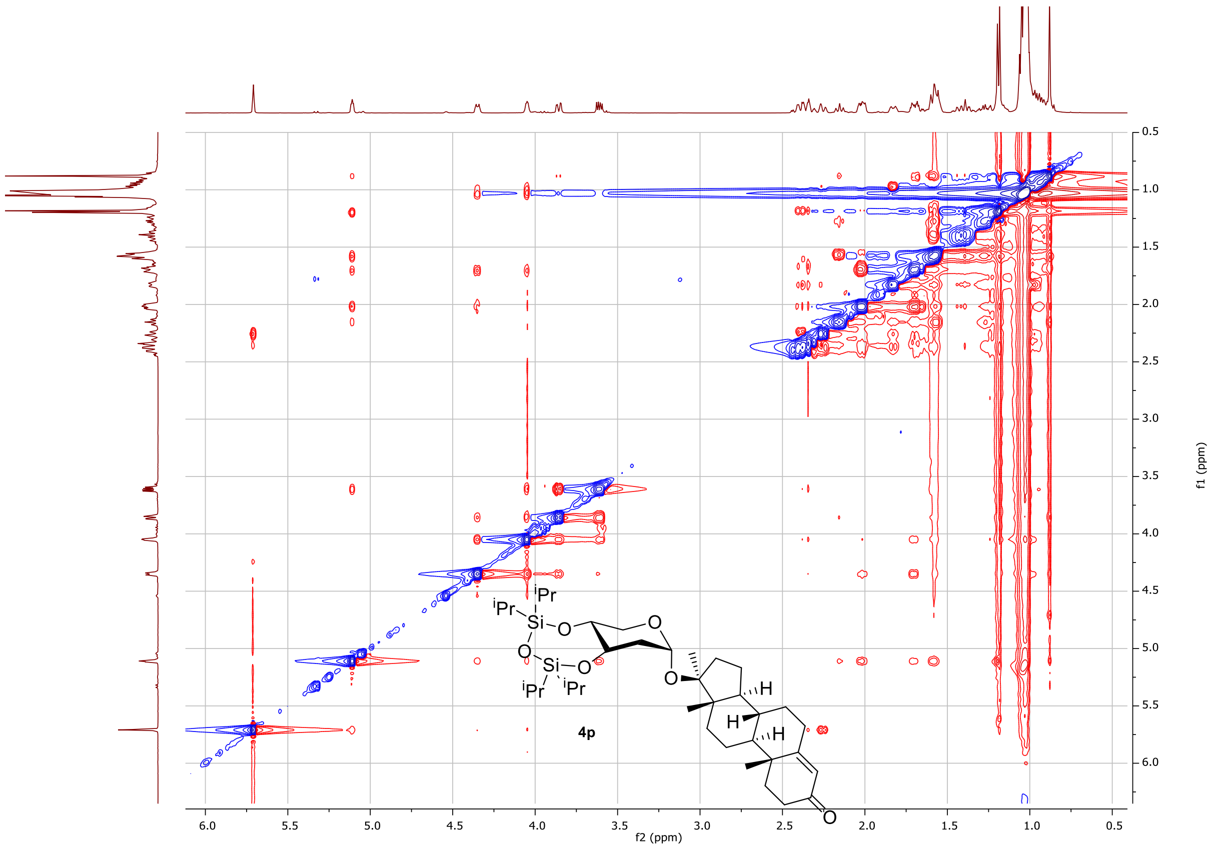

Supplementary figure S521: NOESY spectra for **4p**

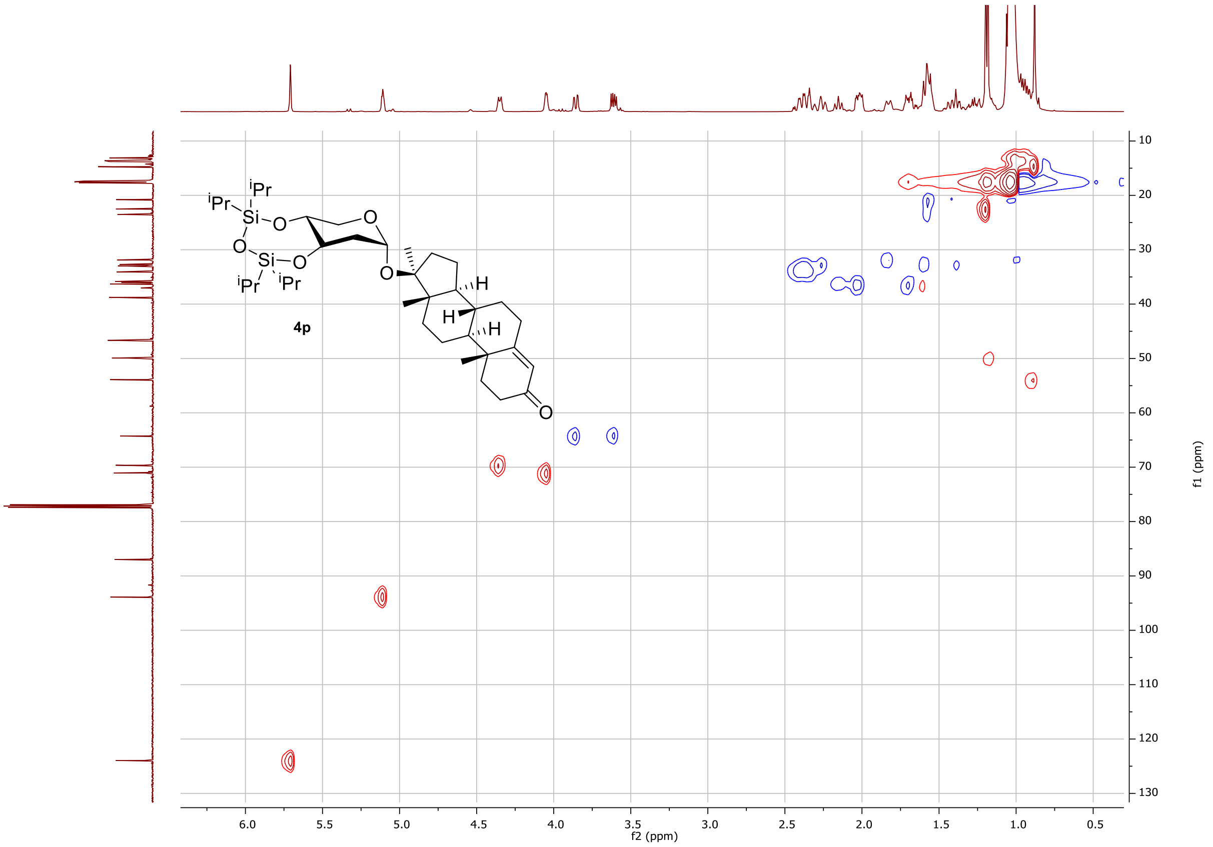

Supplementary figure S522: HSQC spectra for **4p**

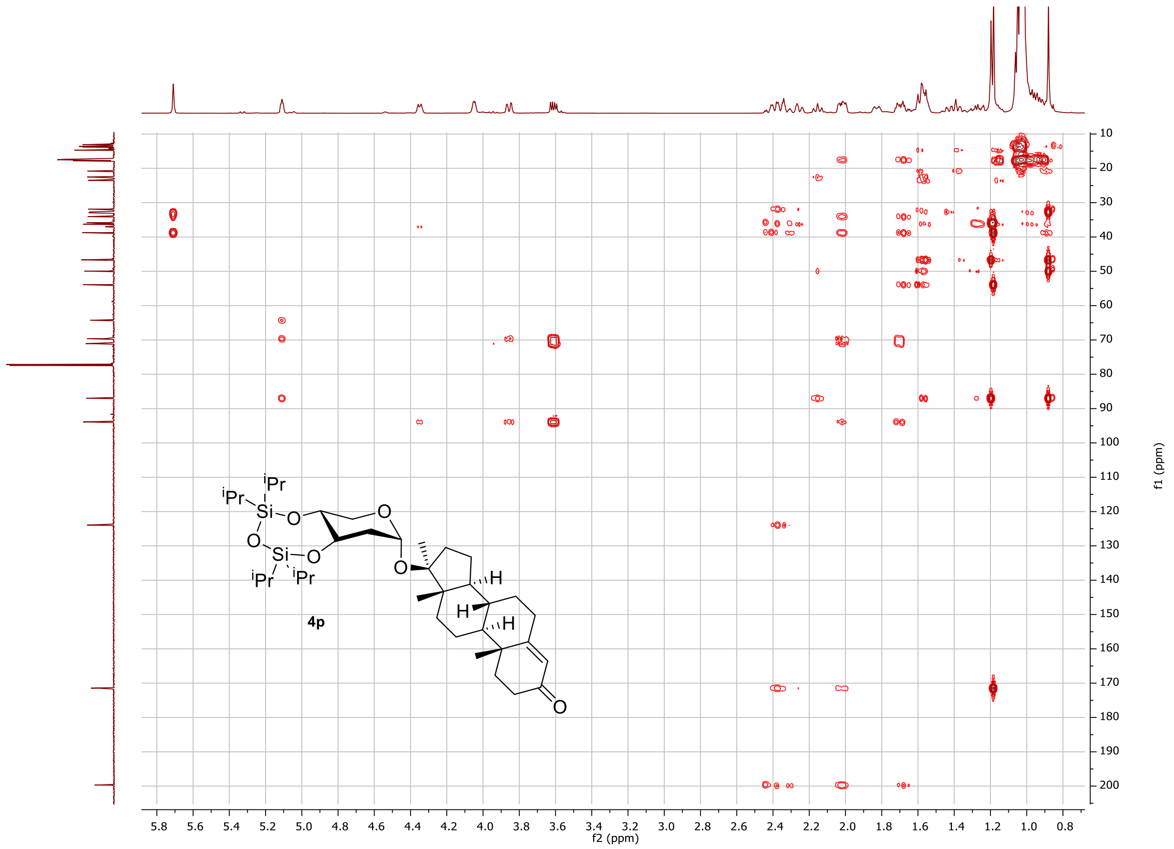

Supplementary figure S523: HMBC spectra for **4p**

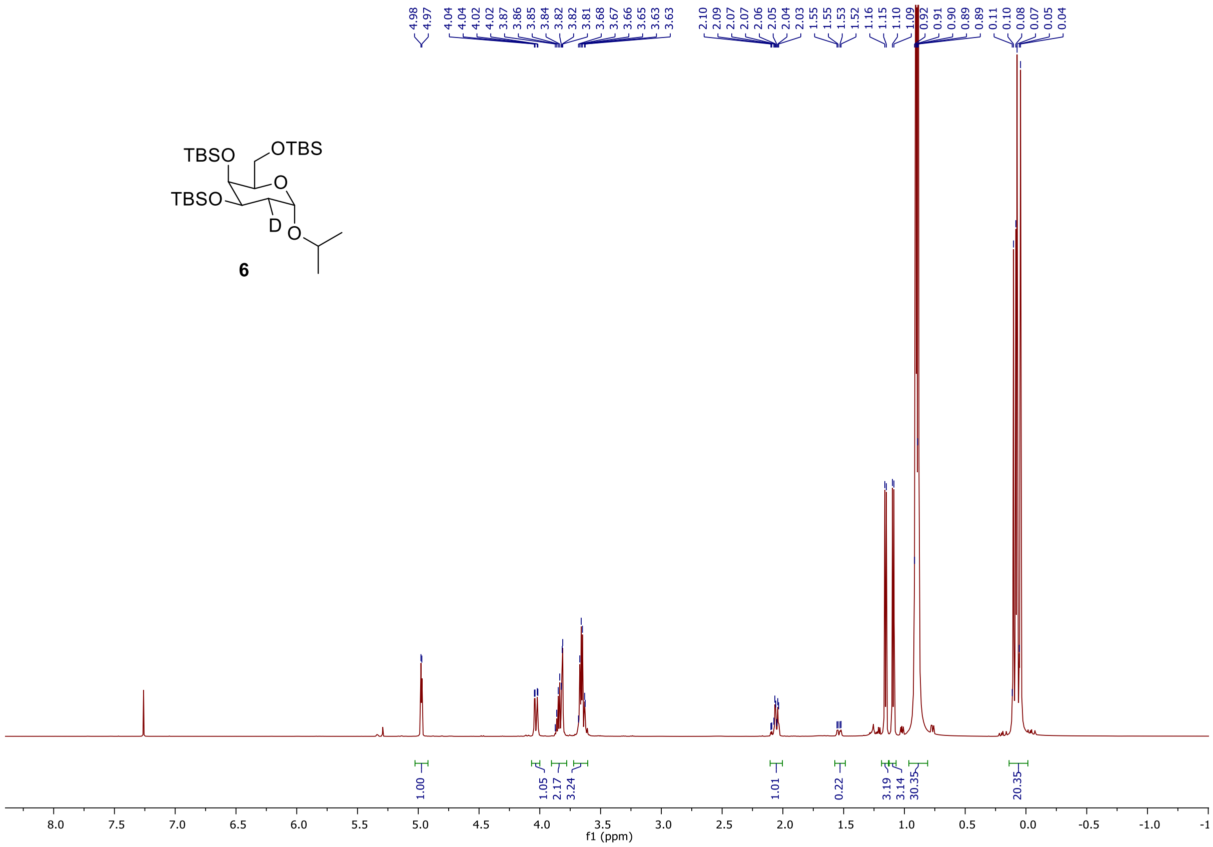

Supplementary figure S524: ^{1}H spectra for **6**

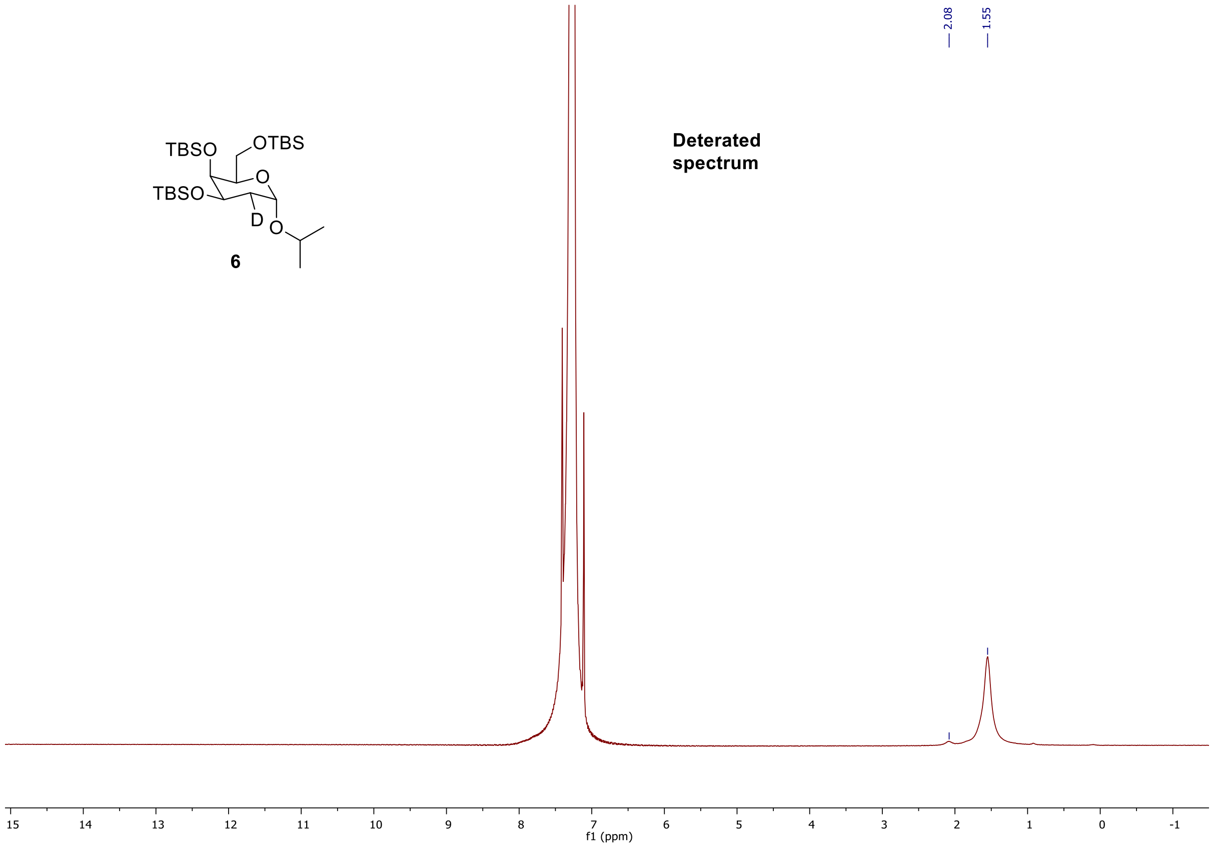

Supplementary figure S525: ^{2}H spectra for **6**

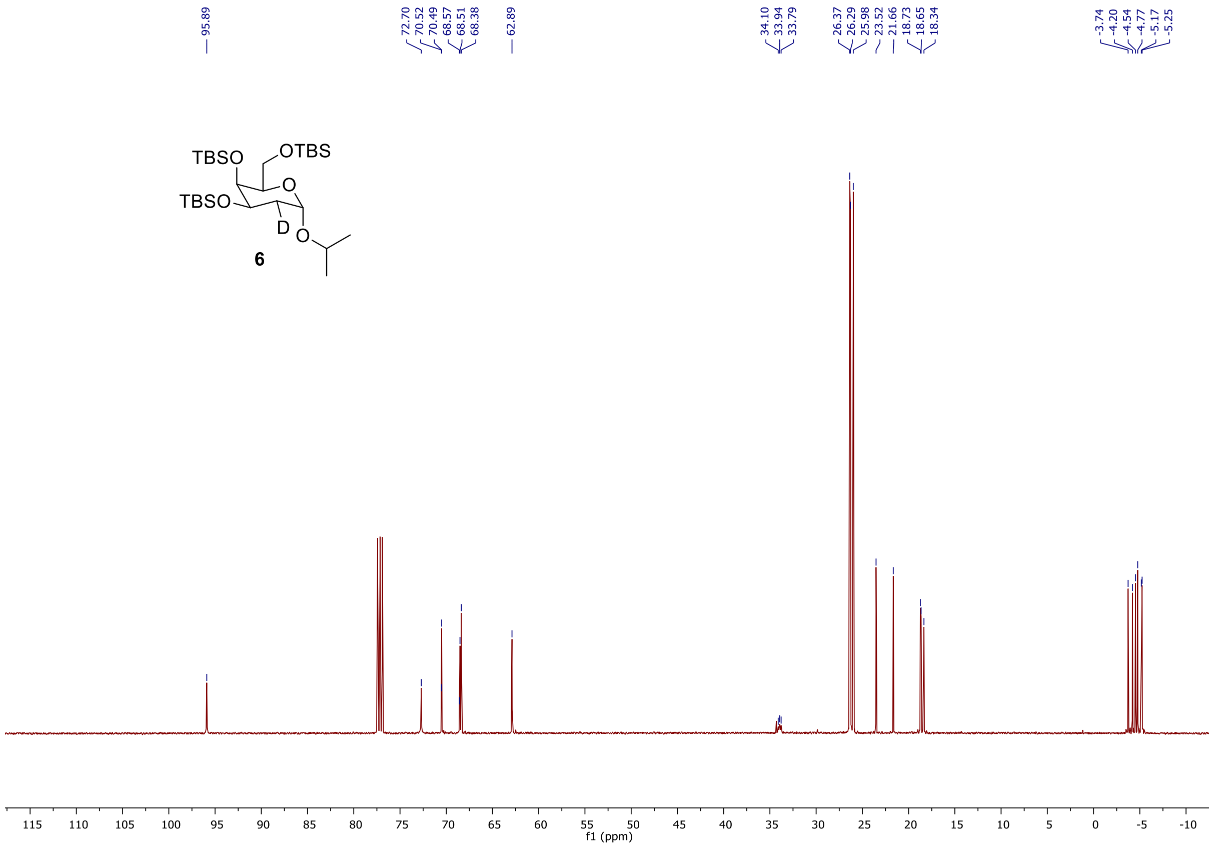

Supplementary figure S526: ^{13}C spectra for **6**

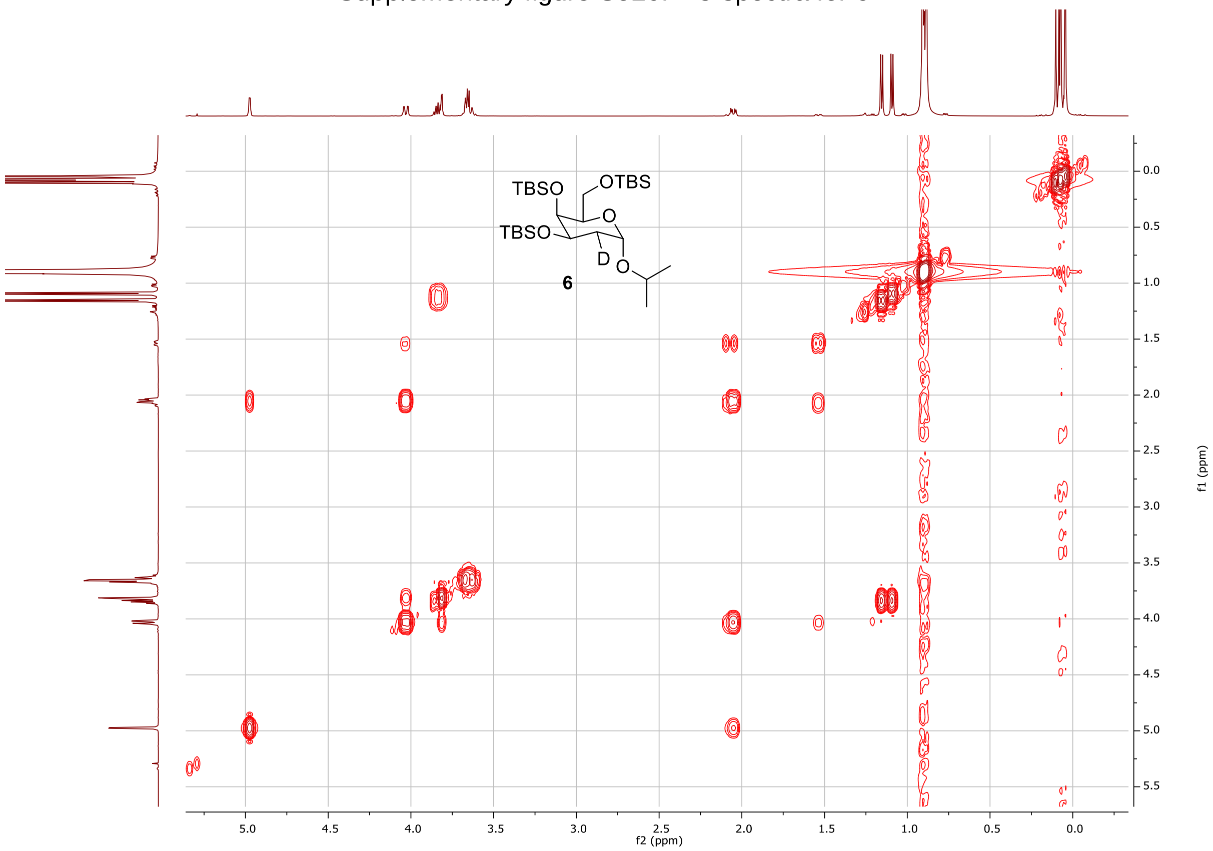

Supplementary figure S527: COSY spectra for **6**

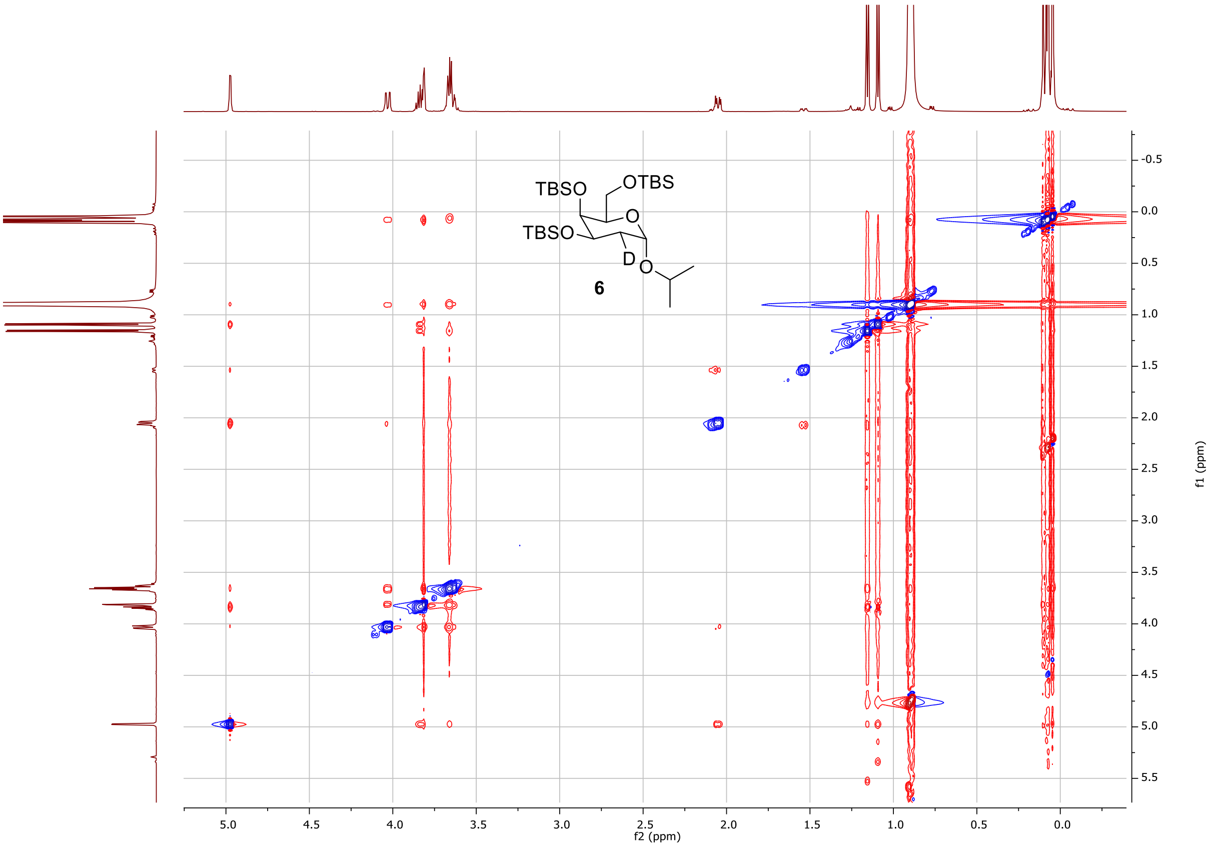

Supplementary figure S528: NOESY spectra for **6**

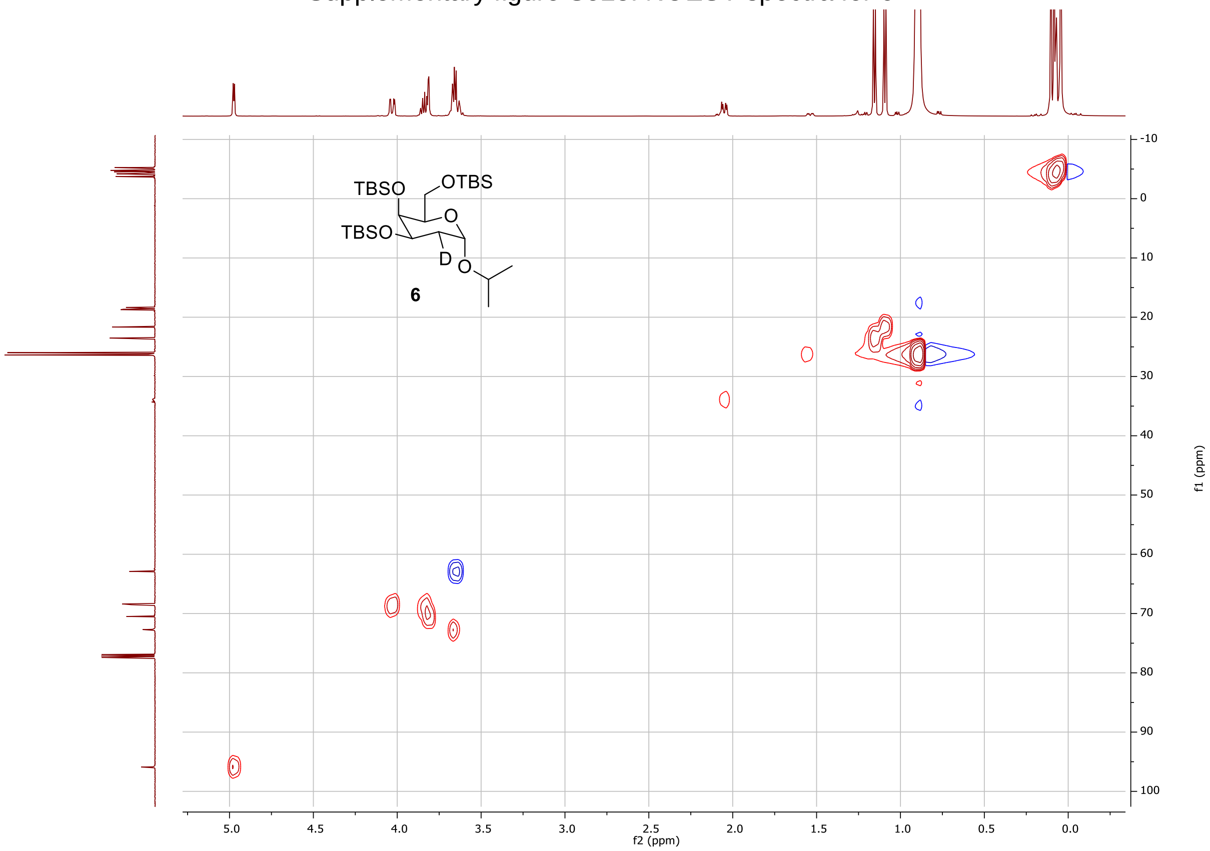

Supplementary figure S529: HSQC spectra for **6**

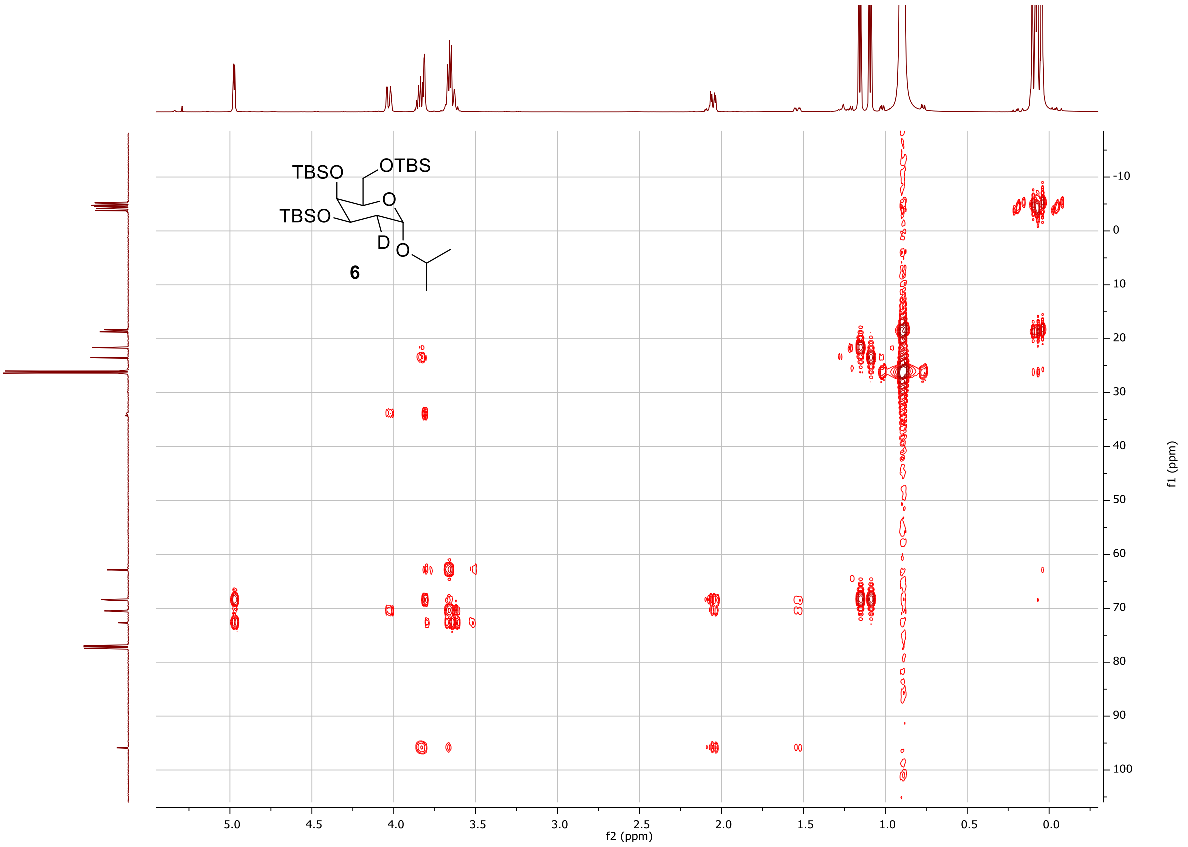

Supplementary figure S530: HMBC spectra for **6**

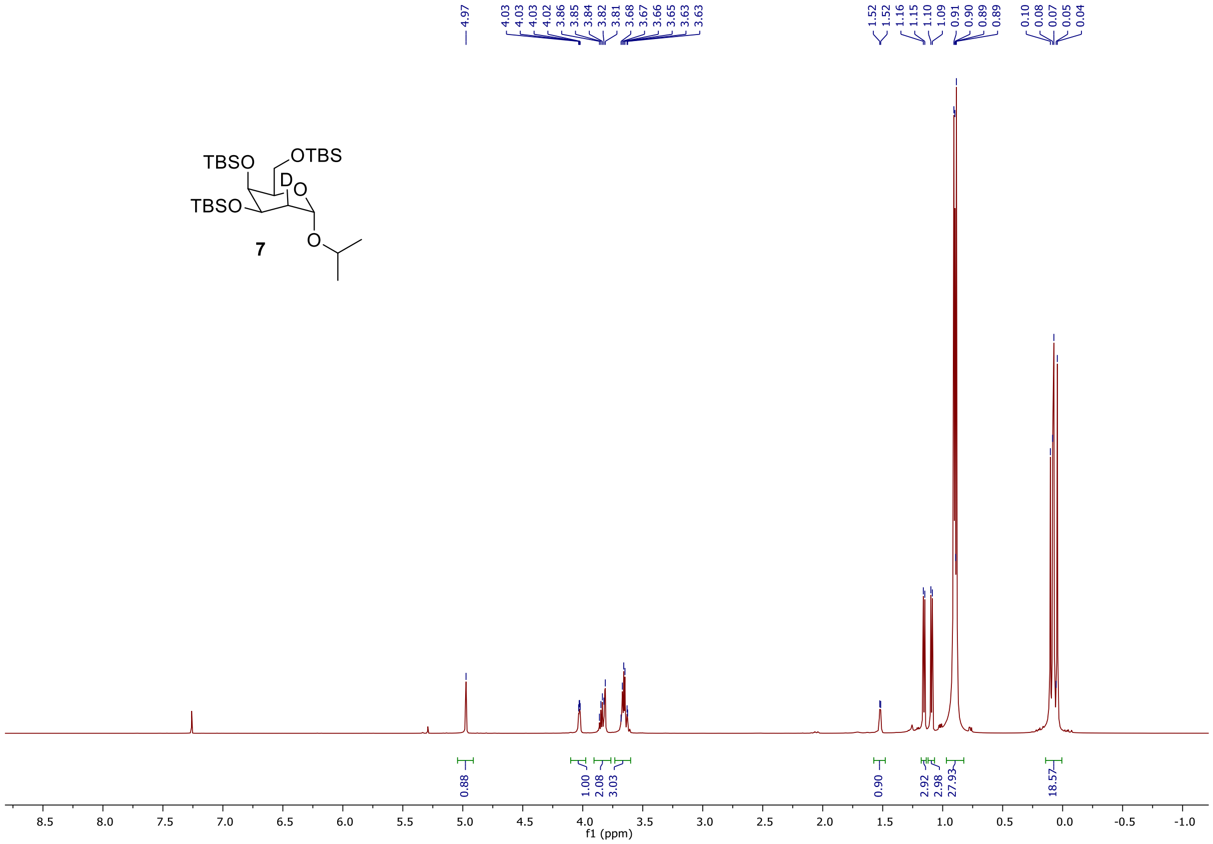

Supplementary figure S531: ^{1}H spectra for **7**

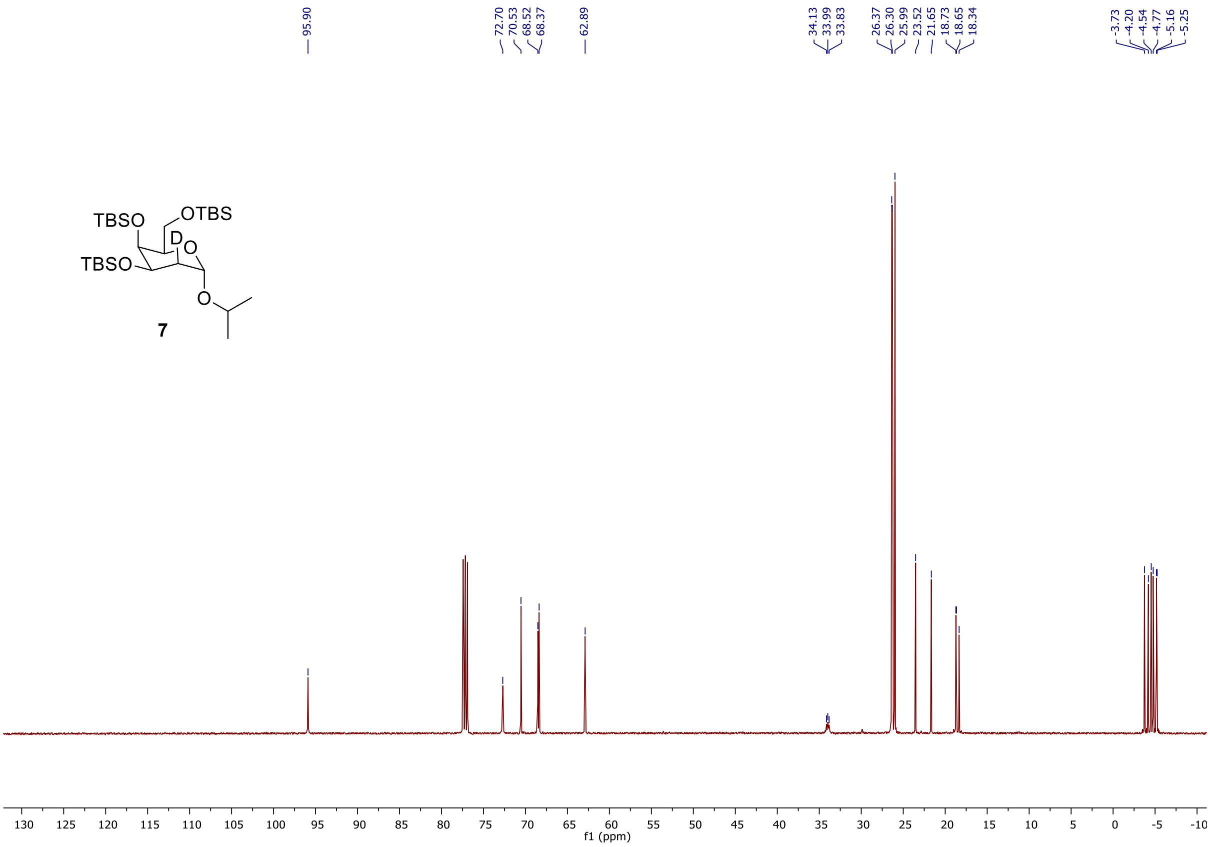

Supplementary figure S532: ^{13}C spectra for **7**

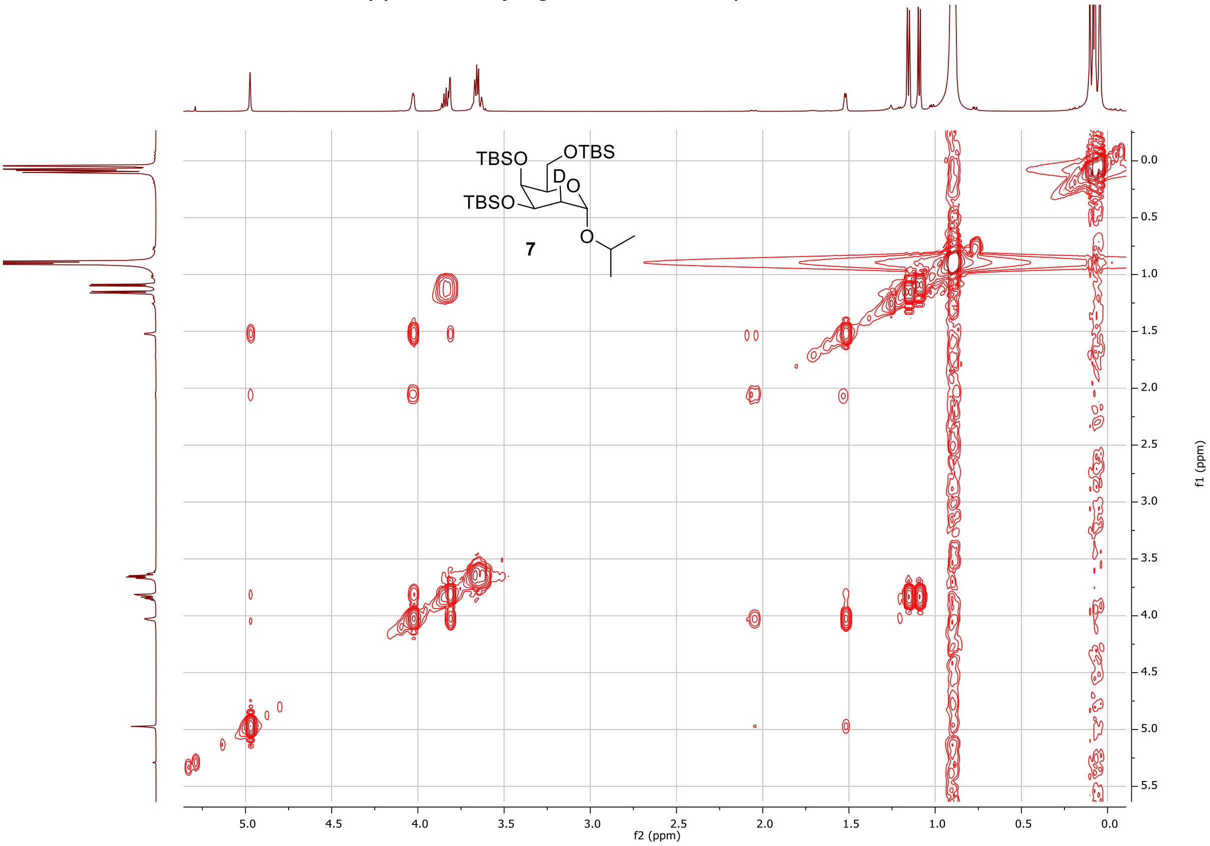

Supplementary figure S533: COSY spectra for **7**

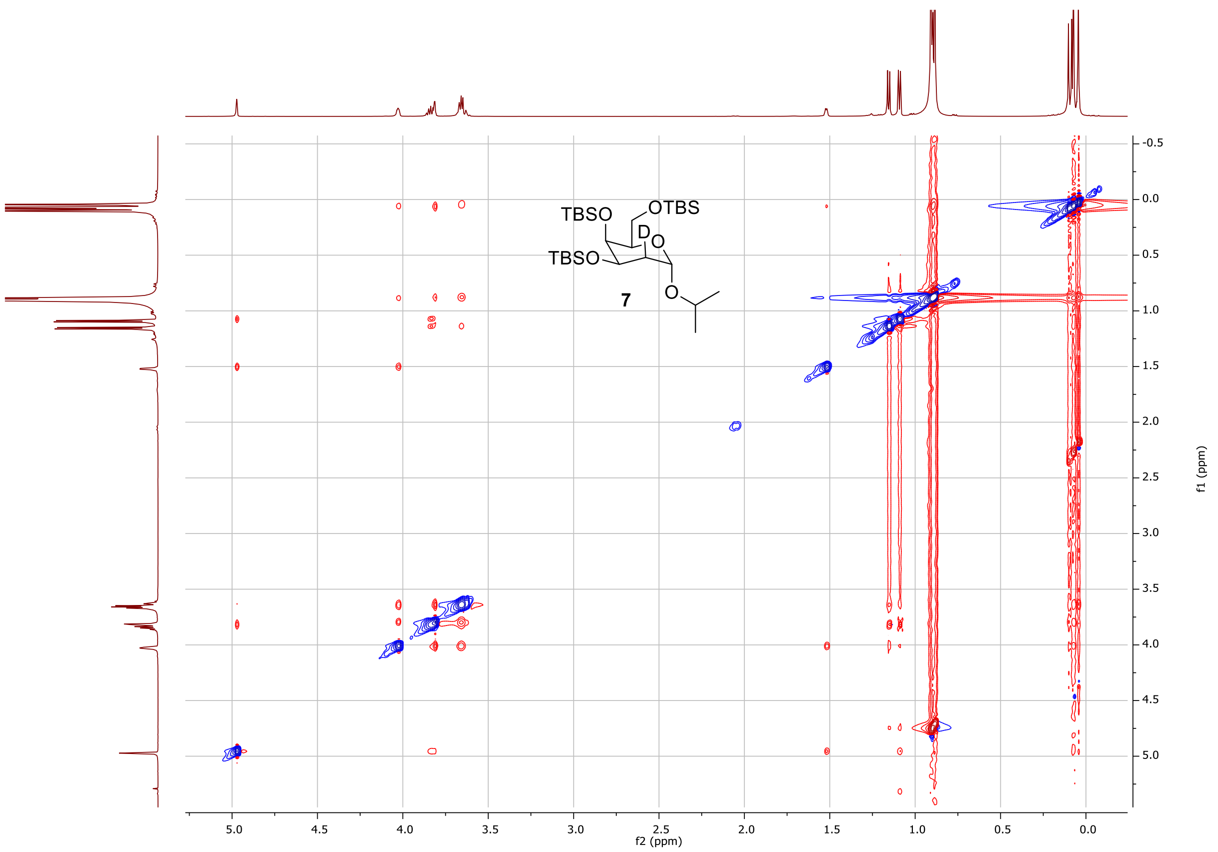

Supplementary figure S534: NOESY spectra for **7**

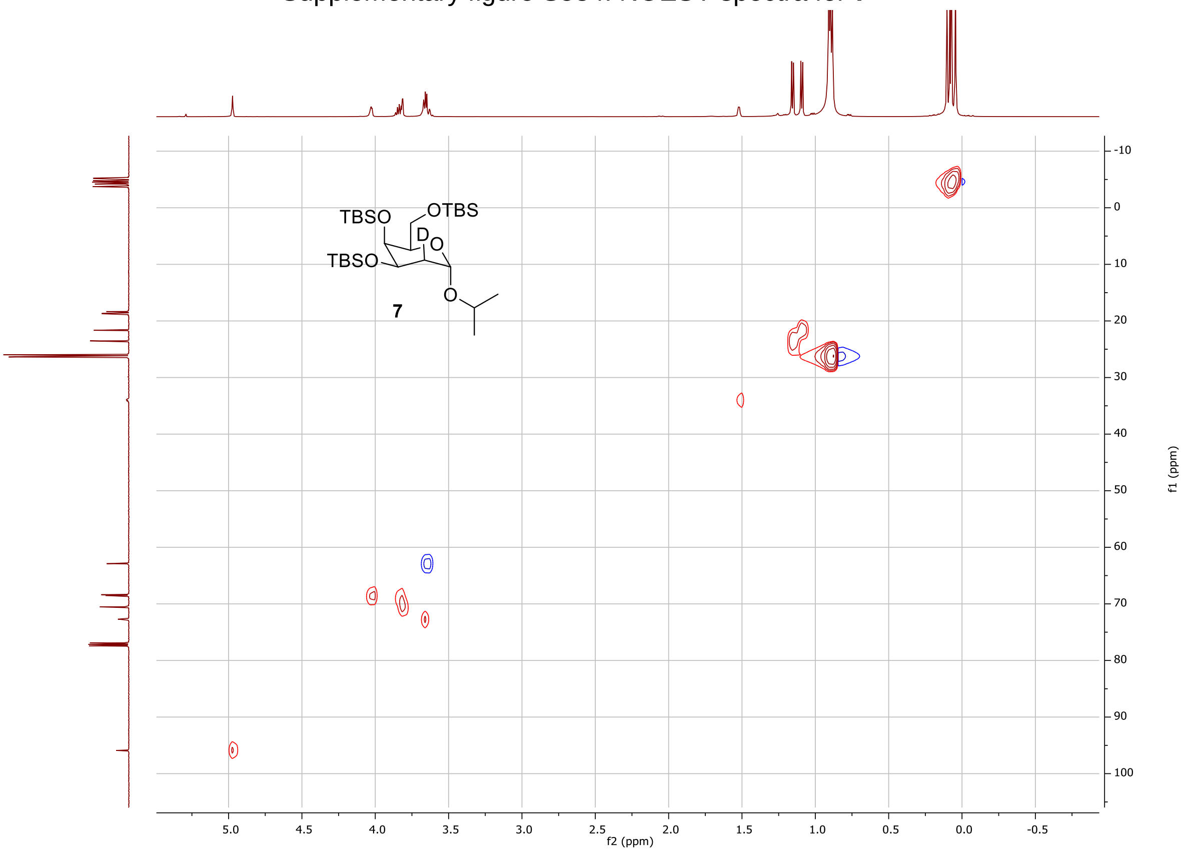

Supplementary figure S535: HSQC spectra for **7**

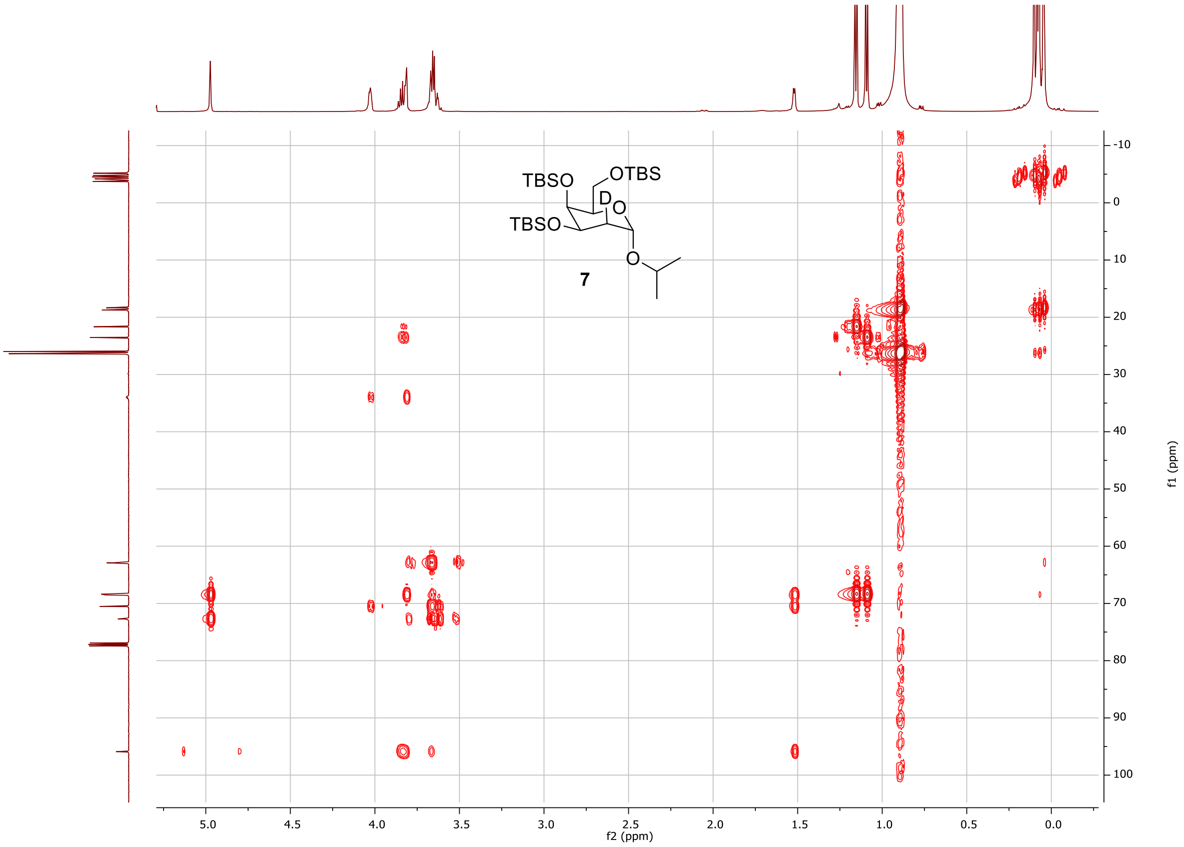

Supplementary figure S536: HMBC spectra for **7**
